# Co-opted and canonical glycerol channels play a major role during anhydrobiosis of an extremophile crustacean

**DOI:** 10.1186/s12915-025-02262-3

**Published:** 2025-06-03

**Authors:** Ángel Rey-Alfonso, José Luis Maestro, François Chauvigné, Jèssica Gómez-Garrido, Tyler Alioto, Peter Bossier, Roderick Nigel Finn, Joan Cerdà

**Affiliations:** 1 Institute of Marine Sciences, Spanish National Research Council (CSIC), Barcelona, 08003 Spain; 2 Institute of Biotechnology and Biomedicine (IBB), Universitat Autònoma de Barcelona, Cerdanyola del Vallès, Bellaterra 08193 Spain; 3 Institute of Evolutionary Biology (CSIC-Universitat Pompeu Fabra), Barcelona, 08003 Spain; 4 National Center of Genomic Analysis (CNAG), Barcelona, 08028 Spain; 5 University of Barcelona, Barcelona, 08028 Spain; 6 Laboratory of Aquaculture & Artemia Reference Center, Faculty of Bioscience Engineering, Ghent University, Ghent, 9000 Belgium; 7 Department of Biological Sciences, University of Bergen, Bergen, 5006 Norway

**Keywords:** Artemia, Diapause, Entomoglyceroporin, Aquaglyceroporin, Desiccation tolerance, Freeze tolerance

## Abstract

**Background:**

Extremophiles evolved capacities to survive extended exposure to harsh environmental conditions such as complete desiccation (anhydrobiosis) and freezing (cryobiosis). Accumulation of the three-carbon polyhydric alcohol glycerol is commonly observed in anhydrobiotic organisms, although it is considered to preferentially enhance cryobiosis rather than anhydrobiosis.

**Results:**

Here, using dormant stages of the halophilic extremophile crustacean *Artemia franciscana*, we show that this role is reversed. We find that *A. franciscana* and related branchiopods evolved co-opted entomoglyceroporin (Eglp)-like aquaporin-type channels previously only characterized in hexapods. Phylogenomic and site-directed mutagenesis analyses indicate that EglpL orthologs likely evolved during the early Cambrian in the common ancestor of the Pancrustacea. RNAi-mediated knockdown experiments show that the *A. franciscana* EglpL glycerol transporter is subfunctionally co-regulated with canonical aquaglyceroporins (Glps) to mediate glycerol accumulation in the diapause cysts. Termination of diapause using either desiccation or hydrogen peroxide and further exposure of the cysts to freezing suggest that the acquired glycerol plays a more critical role in anhydrobiosis rather than cryobiosis.

**Conclusions:**

These findings uncover the essential role of evolutionary divergent aquaporin-type glycerol channels in the accrual of glycerol in an anhydrobiotic organism and reveal a previously overlooked function of this polyol for desiccation tolerance.

**Supplementary Information:**

The online version contains supplementary material available at 10.1186/s12915-025-02262-3.

## Background

Anhydrobiosis (life without water) is an adaptive state permitting organisms to survive complete desiccation imposed by environmental perturbations. It evolved in selected organisms across all kingdoms of life and is most commonly activated through stage-specific genetically programmed, but environmentally induced alterations in the phenotypic trajectories that promote a cryptobiotic state of dormancy [1–5]. The major effectors of anhydrobiosis have been associated with the accumulation of non-reducing sugars such as trehalose, or intrinsically disordered proteins such as late embryogenesis abundant proteins (LEAs), as well as of molecular chaperones including small heat shock proteins p26, Hsp21, -22, and -70, and artemin [6–12]. Glycerol is also known to be accumulated in anhydrobiotic organisms, however, due to its colligative properties it is considered a more important effector for freeze tolerance (cryobiosis) [13–18]. Recent in vitro studies have nevertheless shown that addition of small quantities of glycerol promotes higher glass-transition temperatures and lower fragilities of trehalose containing vitrified matrices [19], which are necessary for desiccation-tolerant stages [20]. However, an in vivo anhydrobiotic role for glycerol and the pathways facilitating its accumulation remain unknown.

The brine shrimp *Artemia franciscana* is a halophilic crustacean (Branchiopoda, Anostraca), in which dormant encysted embryos arrested in gastrulation are considered the most resistant of all animal life history stages to extremes of environmental stress [21]. The extended ability of *A. franciscana* to tolerate stressors such as complete desiccation and freezing is associated with an environmentally induced change from the ovoviviparous release of live nauplii, to the oviparous production and release of encysted diapause embryos (cysts) [8, 22]. The programmed diapause may be broken by desiccation or signaling molecules such as hydrogen peroxide (H_2_O_2_), whereupon the anhydrobiotic, metabolically arrested cysts can also tolerate freezing and may remain quiescent for decades until favorable conditions for normothermal hydrobiotic life return [11, 22]. *A. franciscana* thus offers an excellent model organism to investigate the molecular pathways involved in glycerol accrual in diapause embryos, as well as the role of the polyol during anhydrobiosis and cryobiosis.

Here, using transcriptomics, in silico genomic screens, molecular phylogenetics, and functional analyses employing site-directed mutagenesis and RNA interference (RNAi), we show that co-opted entomoglyceroporin-like (EglpL) aquaporin-type channels [23] co-evolved with canonical aquaglyceroporins (Glps) to regulate glycerol accumulation in diapause cysts, with the acquired glycerol unexpectedly playing a critical role in anhydrobiosis.

## Results

### The aquaporin superfamily in Branchiopoda

To identify the repertoire of aquaporins expressed in *A. franciscana*, we initially conducted stage-specific transcriptomic analyses of adults (males and females), nauplii, and desiccated cysts by RNA sequencing (RNA-seq), using a combination of short Illumina sequencing reads and long cDNA reads from Oxford Nanopore Technologies (ONT). The transcriptomes of each stage were combined into a single final assembled transcriptome that consisted of 187,758 transcripts that cluster into 28,929 genes (Additional file 1: Table S1). The N50 length of these transcripts was of 3600 bp, and BUSCO [24] assessment of the assembled transcriptome reported single gene completeness of 94.1% (arthropoda_odb10 database). A subset of 107,448 of the assembled transcripts (14,475 genes) were annotated as protein coding, and functional annotation labels could be assigned to 67% of the protein-coding transcripts.

**Fig. 1:**
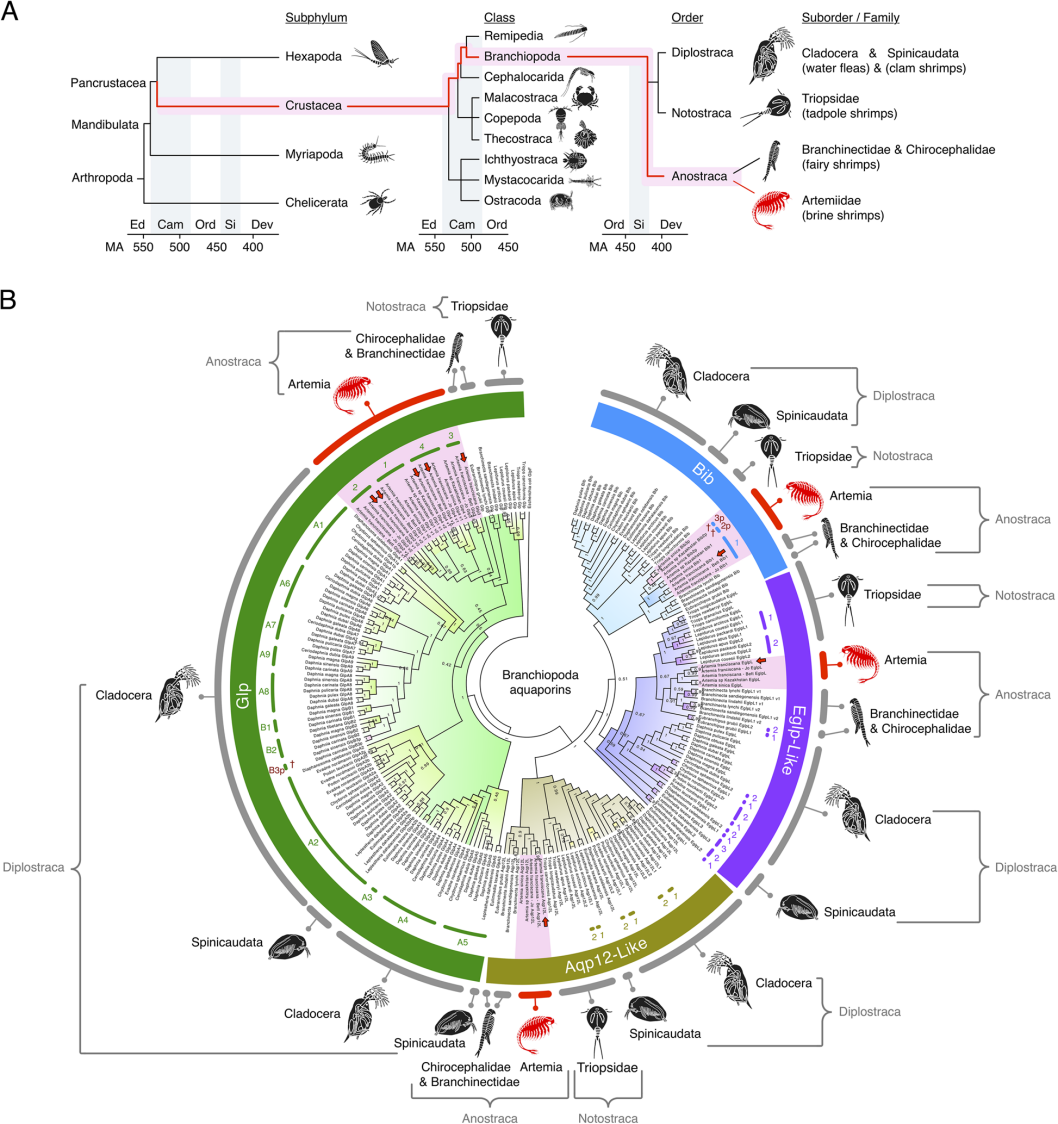
Aquaporin superfamily in Branchiopoda. **A** Systematic classification of Artemiidae, small extremophile arthropod crustaceans within the class Branchiopoda and order Anostraca. Divergence times for subphyla and classes are based on Giribet and Edgecombe [26] with orders based on median TimeTree values [29]. Ed, Ediacaran; Cam, Cambrian; Ord, Ordovician; Si, Silurian; Dev, Devonian; Car, Carboniferous; MA, millions of years ago. **B** Bayesian majority rule consensus tree of branchiopod aquaporins rooted with *Escherichia coli glpF*. The tree is inferred from five million MCMC generations (nucmodel = 4by4, nst = 2, rates = gamma) of 554,321 nucleotide sites aligned by codon (Additional file 4: Dataset S1; *n* = 232 taxa). Red arrows indicate *Artemia franciscana* channels analyzed here. Duplicates are shown with Arabic numerals or alphanumeric numerals for diplostracan *glps* after Catalán-García et al. [27]. † = pseudogene

The transcriptomic searches coupled with genomic screening (see below) using tblastn [25] identified eight aquaporin coding sequences (CDS) in *A. franciscana*. Phylogenetic analyses of the *A. franciscana* channel CDS were then conducted in relation to the genomic complements in congeners and other branchiopods (22 genomes) via Bayesian inference. Branchiopoda are estimated to have diverged from other classes of Crustacea during the Cambrian period (Fig. 1A) [26]. The Bayesian results identified four major clades of aquaporins in Branchiopoda, a clade including two AQP4-related orthologs, Big brain (Bib) and EglpL channels, a clade of unorthodox AQP12-like (Aqp12L) channels, and a clade of canonical Glps (Fig. 1B). No AQP8-type CDS were found; however, lineage-level duplications were identified in each of the other aquaporin clades with most duplicates found in diplostracan water fleas and clam shrimps, including a major expansion of the canonical Glps noted previously in water fleas [27]. A surprising observation was the apparent absence of Prip-like (PripL) orthologs found in other crustaceans and arthropods [27, 28] and the apparent presence of EglpL channels. To confirm these observations, we conducted a separate Bayesian analysis of 585 AQP4-related CDS in arthropods. These results show that all arthropods encode Bib, with chelicerate, myriapod, and pancrustacean lineages also encoding a novel Bib-like integral membrane protein (Blip) and a PripL channel, including a novel lepidopteran integral membrane protein (Leip) (Additional file 2: Fig. S1, Additional file 3: Fig. S2, Additional file 4: Dataset S1, Additional file 5: Dataset S2, and Additional file 6: Dataset S3). However, although crustacean ostracods retain the PripL channel, they also encode an EglpL channel that forms a sister clade to the insect Eglps (posterior probability [pp] = 1). Conversely, the branchiopod EglpL sequences cluster next to these clades, but outside of the arthropod PripL clusters (pp = 1).

### Genomic mapping of the *Artemia* aquaporin repertoires

In order to further validate the *A. franciscana* aquaporin CDS identified in our transcriptome, we mapped each transcript to three chromosomal-level assemblies, including two for *A. franciscana* from South Korea [30] and Utah (USA) [31] and one for *A. sinica* from Tanggu salterns (China) [32]. A fourth genome for *Artemia* sp. Kazakhstan is publicly available (GenBank BioProject PRJNA866468; original source unknown), but this genome is only assembled at the scaffold level and was not used for mapping purposes. The genomic data for the two *A. franciscana* chromosomal assemblies reveal that each of the identified aquaporins is assigned to different chromosomes (Additional file 7: Fig. S3A). For *bib*, *eglpL*, and *aqp12L* paralogs, the gene structures are similar, consisting of eight, six, and five exons, respectively. However, we noted several pseudoexons in the *eglpL* genes that differentially localized within the functional *eglpL* gene and a separate downstream pseudogene fragment in the Bett et al. [31] assembly (Additional file 7: Fig. S3B). The same gene structures are observed in the *A. sinica* assembly, which also retains the downstream *eglpL* pseudogene as well as two differentially localized *bib* pseudogenes. Conversely, the canonical *glp* genes, which are all localized in clusters, showed complex repertoires within each genome. In the Bett et al. [31] assembly, four *glps* (*glp1-4*) were identified, while only three (*glp2-4*) exist in the Jo et al. [30] genome and two (*glp1* and *glp4*) in the *A. sinica* genome. Bayesian inference of the deduced transcripts, assembled genes, and clones (see below) confirmed these observations (Additional file 7: Fig. S3C). This allowed us to map transcriptomic isoforms to the specific genes. Two N-terminal isoforms were thus mapped to *glp1* in the Bett et al. [31] genome, but to *glp2* in the Jo et al. [30] genome, and are consequently named *glp1_v1*, *glp1_v2* and *glp2_v1*, *glp_v2*, respectively. In addition, two C-terminal splice variants were mapped as *glp4_v1* and *glp4_v2* in both genomes; however, in the *A. sinica* genome only the *glp1_v1* and *glp1_v2* variants were found. The structural basis for the N-terminal isoforms arises through the existence of a 5′ exon 1 (M1) that is spliced to an upstream extended region (UER) of the M45 start codon, with the M1 exon 1 being identical for *glp1_v2* and *glp2_v2* in the Bett et al. [31] and Jo et al. [30] assemblies, respectively (Additional file 7: Fig. S3D). The UER exists for each of the *glp1-4* genes, and consequently the M1 exon 1 could potentially be spliced to *glp3* and *glp4*, but this was not observed in the transcriptome. Conversely, the C-terminal isoforms arise through the existence of an additional exon 7, which when spliced disrupts the exon 6 stop codon and extends the translated protein by five amino acids. Our transcriptomic data thus showed highest congruence with the Jo et al. [30] genome.

### Branchiopods encode functional EglpL channels

**Fig. 2:**
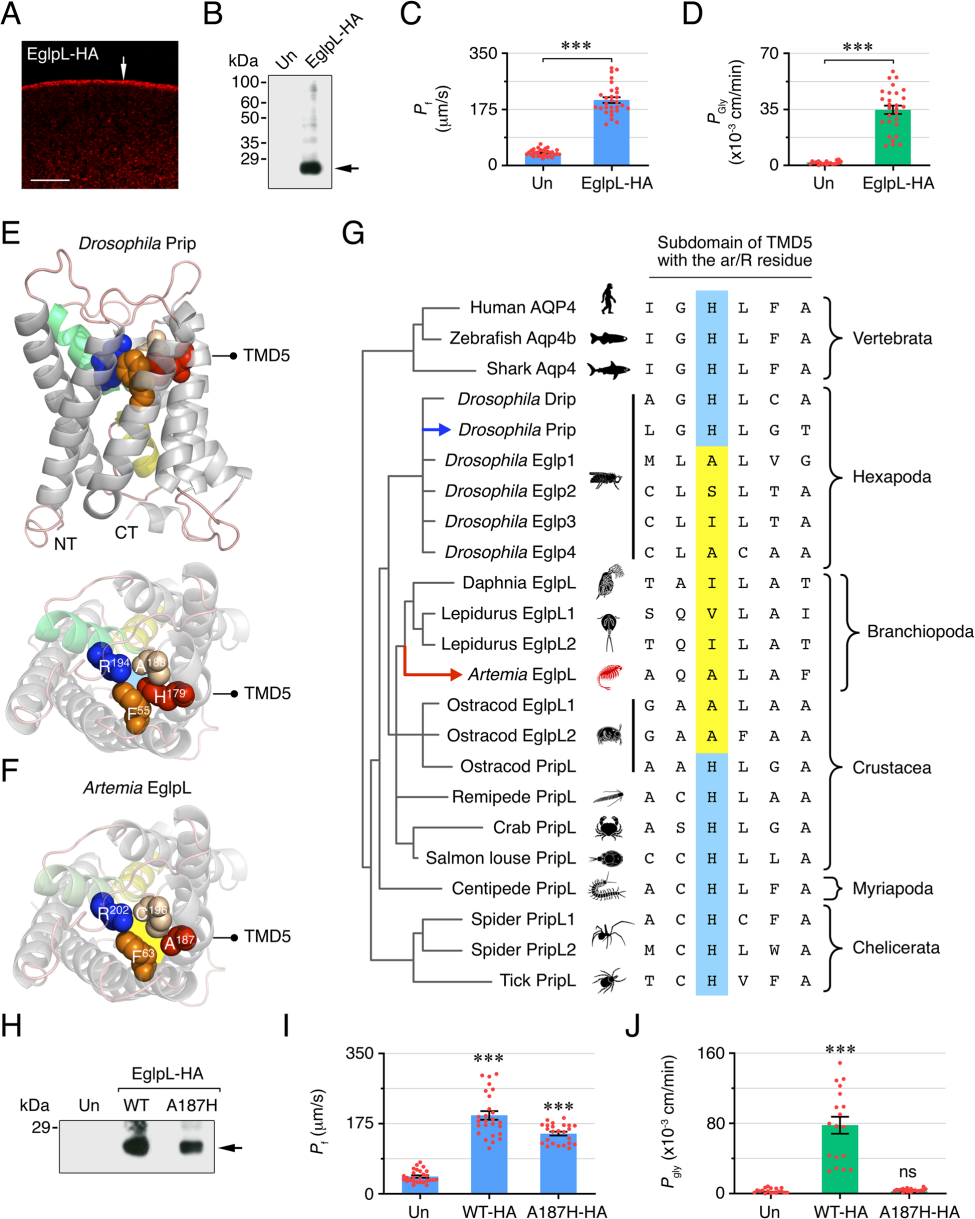
Co-opted EglpL channels in crustaceans. **A** Representative immunostaining of *X. laevis* oocytes expressing *A. franciscana* HA-tagged EglpL (EglpL-HA) using an HA antibody. The arrow points to the plasma membrane. Scale bar, 10 µm. **B** Representative immunoblot of total membranes from non-injected control oocytes (Un) and oocytes expressing the EglpL-HA, with molecular markers shown to the left. The arrow points to a band that matches the predicted molecular mass of the channel monomer. Osmotic water (**C**) and glycerol (**D**) permeabilities (*P*_f_ and *P*_gly_, respectively) of control oocytes and oocytes expressing the EglpL-HA. Data (mean ± SEM; *n* = 26–35 oocytes/treatment, red dots) from two independent experiments were combined and statistically analyzed by the unpaired Student’s *t*-test (***, *p* < 0.001). **E**, **F** Three-dimensional cartoon renders of *Drosophila melanogaster* Prip (FBpp0087236) and *Artemia franciscana* EglpL channels. Models are generated via the Swiss-Prot data pipeline and show intramembrane and extracellular views, with the ar/R selectivity residues rendered as spacefill. TMD, transmembrane domain. **G** Alignment of vertebrate and arthropod AQP4-related TMD5 subdomains showing a conserved His in AQP4 and PripL orthologs, but a substituted residue in the EglpL channels. **H** Immunoblot of total membranes from control oocytes and oocytes expressing wild-type (WT) EglpL-HA or the EglpL-A187H-HA mutant. The arrow indicates the monomer of each channel, and the molecular markers are shown to the left. **I**, **J** *P*_f_ and *P*_gly_ of the corresponding oocytes. Data (mean ± SEM; *n* = 18–27 oocytes per treatment, red dots) from two independent experiments were combined and statistically analyzed by the Kruskal–Wallis test, followed by Dunn’s multiple comparisons test (***, *p* < 0.001, with respect to the controls). ns, not statistically significant. Uncropped immunoblots are available in Additional file 13: Fig. S6

To evaluate whether the Branchiopoda EglpL channels are capable of permeating glycerol, we used the *Xenopus laevis* oocyte expression system. In these experiments, human influenza hemagglutinin (HA)-tagged *A. franciscana* EglpL cRNA was expressed in oocytes, its expression and subcellular localization assessed by immunoblotting and immunofluorescence microscopy, and the osmotic water and glycerol permeabilities (*P*_f_ and *P*_gly_, respectively) of oocytes determined by measuring the rates of cell swelling. Immunofluorescence and western blot analyses confirmed that EglpL-HA was expressed and constitutively targeted to the oocyte plasma membrane (Fig. 2A and B). The functional assays showed that EglpL-HA-injected oocytes elicited an ~ 5- and ~ 40-fold increase *P*_f_ and *P*_gly_, respectively, with respect to the uninjected control oocytes (Fig. 2C and D), thus confirming that *A. franciscana* EglpL is a water and glycerol transporter.

Further evidence that branchiopods and ostracods evolved EglpL channels arose from observations that, in contrast to insect Prips, which harbor a His residue in the aromatic-arginine (ar/R) selectivity filter on the fifth transmembrane domain (TMD5) (Fig. 2E), the *Artemia* EglpL orthologs substituted this residue for a smaller uncharged Ala (Fig. 2F), as reported for insect Eglps [23]. Comparing this site across all arthropod AQP4-related channels shows that only ostracod and branchiopod EglpL orthologs have undergone His-(Ala, Val, Ile) substitutions in this site (Fig. 2G). To verify that the TMD5 Ala^187^ residue in *A. franciscana* EglpL is critical for glycerol flux, Ala^187^ was mutated into His, and both wild-type (WT) EglpL-HA and EglpL-HA-A187H were expressed in *X. laevis* oocytes. The data show that both channels were expressed approximately equally in oocytes and function as water channels. However, the EglpL-HA-A187H completely abolished oocyte glycerol permeability with respect to oocytes expressing EglpL-WT-HA (Fig. 2H–J), thus confirming that it is a hexapod-type, co-opted water channel [23].

### Permeability properties of *A. franciscana* Glps

**Fig. 3:**
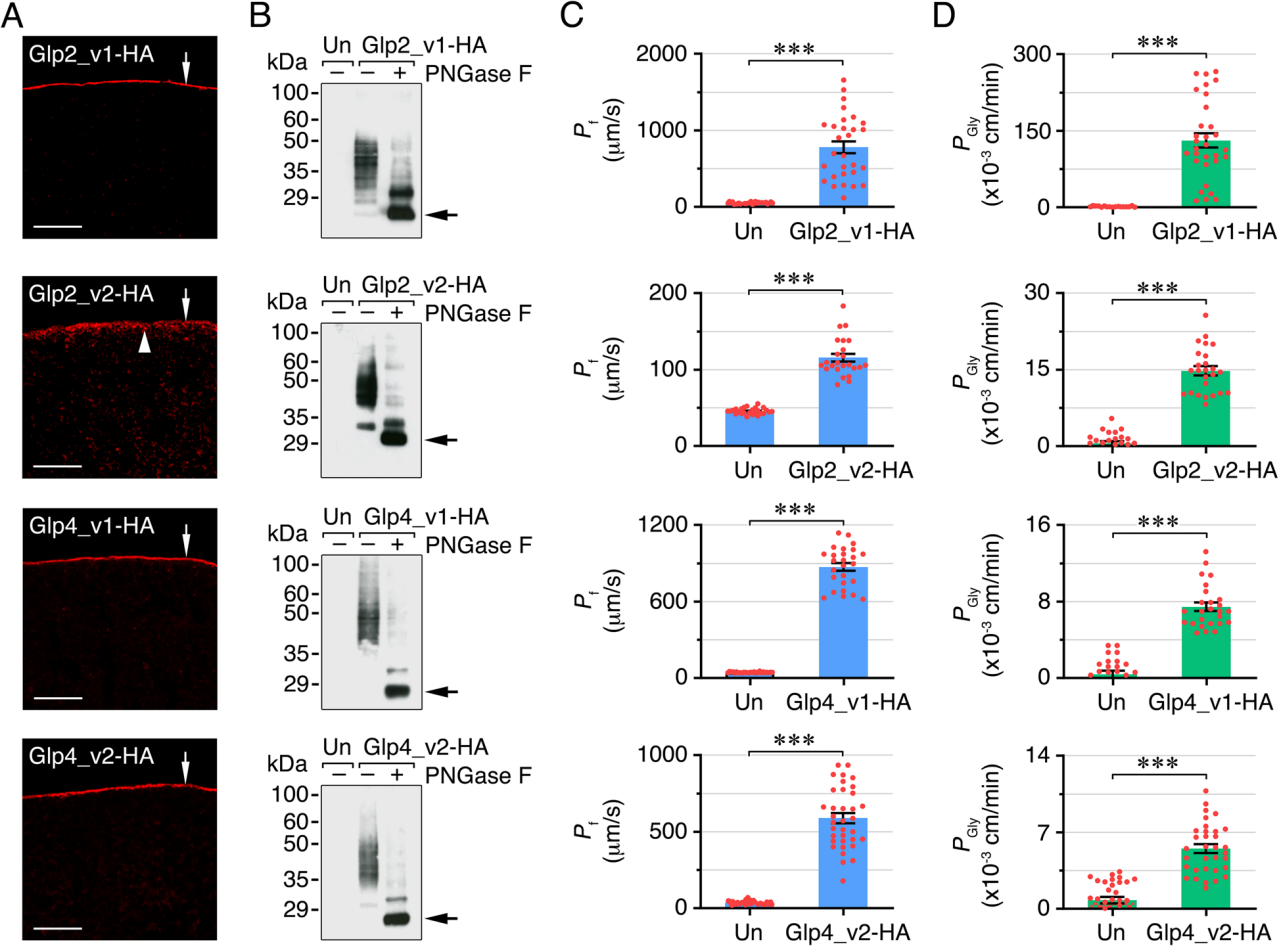
Functional characterization of *A. franciscana* Glps in *X. laevis* oocytes. **A** Representative immunostaining of oocytes expressing HA-tagged Glp2_v1, Glp2_v2, Glp4_v1, and Glp4_v2 using an HA antibody. Arrows point to the plasma membrane, while the arrowhead indicates the cortical ooplasm. Scale bars, 10 µm. **B** Representative immunoblots of total membranes from non-injected oocytes (Un) and oocytes expressing Glp2_v1-HA, Glp2_v2-HA, Glp4_v1-HA, or Glp4_v2-HA, with molecular markers shown to the left. Plus and minus signs indicate the protein extracts respectively treated with or without PNGase F prior to SDS-PAGE. Arrows in each blot point to bands that match the predicted monomeric molecular mass of the Glp paralogs and isoforms. For all Glps, the reactions appeared as faint smear of ~ 30–50 kDa which were sensitive to PNGase F treatment, indicating that the channels were glycosylated. Osmotic water (**C**) and glycerol (**D**) permeabilities (*P*_f_ and *P*_gly_, respectively) of oocytes expressing Glp2_v1-HA, Glp2_v2-HA, Glp4_v1-HA, or Glp4_v2-HA. Data (mean ± SEM; *n* = 26–35 oocytes/treatment, red dots) from two independent experiments were combined and statistically analyzed by the unpaired Student’s *t*-test (***, *p* < 0.001). Uncropped immunoblots are available in Additional file 13: Fig. S6

To investigate the functional properties of the *A. franciscana* Glps, we isolated and cloned the full-length cDNAs encoding the two isoforms of Glp2 (Glp2_v1 and Glp2_v2) and Glp4 (Glp4_v1 and Glp4_v2), and their corresponding HA-tagged cRNAs were expressed in *X. laevis* oocytes as above. The cDNAs for Glp1 and Glp3 were not isolated because *glp1* was only identified in one of the *A. franciscana* genomes and is not present in our transcriptome or the Jo et al. genome [30], while only a fragment of *glp3* could be found in this transcriptome. The results of the experiments showed that the four Glps were constitutively targeted to the oocyte plasma membrane, although the Glp2_v2-HA was partially retained in the cortical ooplasm (Fig. 3A). Immunoblotting using undigested and N-glycosidase F-digested protein extracts from oocytes expressing the different Glp constructs revealed that the four channels were post-translationally modified by N-linked glycans (Fig. 3B). The functional assays showed that oocytes expressing each of the Glps tested were able to conduct water and glycerol albeit with different efficiency. Thus, oocytes injected with Glp2_v1-HA exhibit a higher increase in *P*_f_ and *P*_gly_ (of ~ 15- and ~ 450-fold, respectively) than those expressing the Glp2_v2-HA isoform (~ 3- and ~ 25-fold increase in *P*_f_ and *P*_gly_, respectively), possibly as a result of the partial retention in the ooplasm of the channel with the longer N-terminus (Fig. 3C and D). In contrast, oocytes injected with each of the Glp4 isoforms showed a similar increment in *P*_f_ (of ~ 19- and ~ 15-fold for Glp4_v1-HA and Glp4_v2-HA, respectively), while glycerol transport was more efficient in oocytes expressing Glp4_v1-HA (~ 19-fold increase in *P*_gly_) than in those injected with Glp4_v2 (~ sevenfold increase in *P*_gly_) (Fig. 3C and D). These data confirm that *A. franciscana* Glps can transport water and glycerol, and suggest that the two Glp2 isoforms may be under different intracellular trafficking regulation.

### Expression regulation of glycerol channels during ovoviviparous and oviparous development

**Fig. 4:**
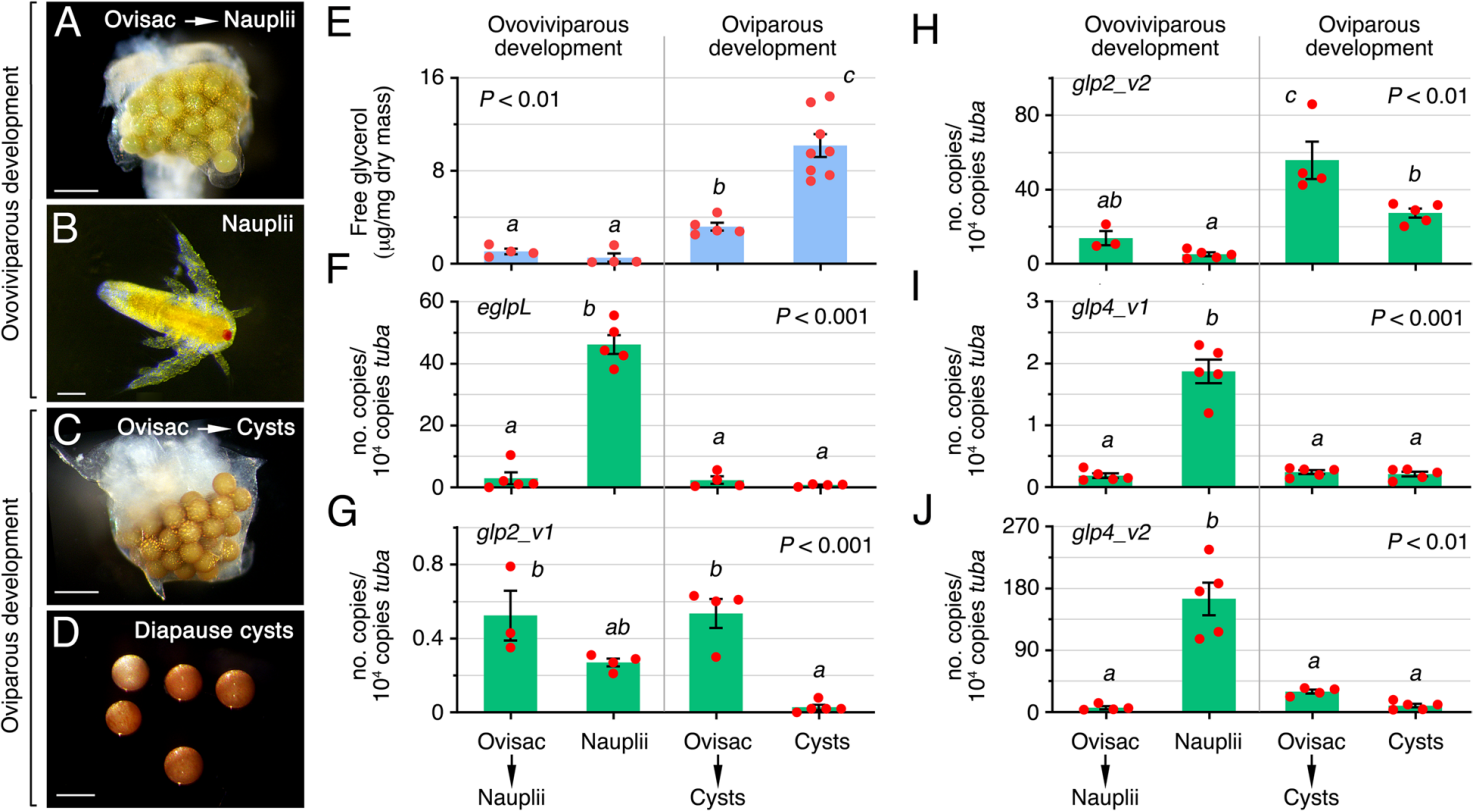
Glycerol accumulation and aquaporin expression during ovoviviparous and oviparous development in *A. franciscana*. Photomicrographs of ovisacs containing oocytes programmed to develop into nauplii (**A**) or encysted embryos (**C**) prior to fertilization, nauplii (**B**), and released diapause cysts (**D**). Scale bars, 500 µm (**A** and **C**), 100 µm (**B**), and 250 µm (**D**). **E** Free glycerol content in the developmental stages shown in (**A**–**D**). **F**–**J** *eglpL* and *glps* expression levels in the same stages as determined by RT-qPCR. Transcript levels of α-tubulin (*tuba*) were used as reference, and relative transcript levels were calculated as copy numbers normalized against those of *tuba*. Data (mean ± SEM; *n* = 4–5 independent experiments, red dots) were statistically analyzed by one-way ANOVA or Kruskal–Wallis test, followed by the Tukey’s multiple comparisons test or Dunn’s multiple comparisons test, respectively. Bars with different superscripts are significantly different (*p* values indicated in each panel)

In order to determine which of the aquaporin paralogs may be involved in glycerol accumulation in *A. franciscana* diapause cysts, we investigated the amount of free glycerol and the mRNA expression levels of the glycerol channels *eglpL*, *glp2_v1*, *glp2_v2*, *glp4_v1*, and *glp4_v2* in different developmental stages. The selected stages were ovisacs from females destined to produce either nauplii (ovoviviparous development) or diapause cysts (oviparous development), free nauplii, and released hydrated diapause cysts. The relative abundance of free glycerol remained low (~ 0.1% dry weight) in ovisacs with developing nauplii or in released nauplii, whereas ~ 3- and ~ tenfold increases of the glycerol levels were respectively observed in ovisacs carrying developing cysts and in diapause cysts, with the latter glycerol content reaching ~ 1.3% of the cyst dry weight (Fig. 4E). Real-time quantitative PCR (RT-qPCR) gene expression analysis indicated that the transcript levels of *eglpL*, *glp4_v1*, and *glp4_v2* were more accumulated in nauplii (Fig. 4F, I, and J), whereas those of *glp2_v2* increased specifically in ovisacs destined to produce encysted embryos, with a small decrease in the released diapause cysts (Fig. 4H). In contrast, the *glp2_v1* mRNA levels were much lower than those from the other genes in ovoviviparous or oviparous ovisacs and nauplii, and further decreased in released cysts (Fig. 4G).

### Knockdown of glycerol channels impairs oviparity and stress tolerance of *A. franciscana* cysts

**Fig. 5:**
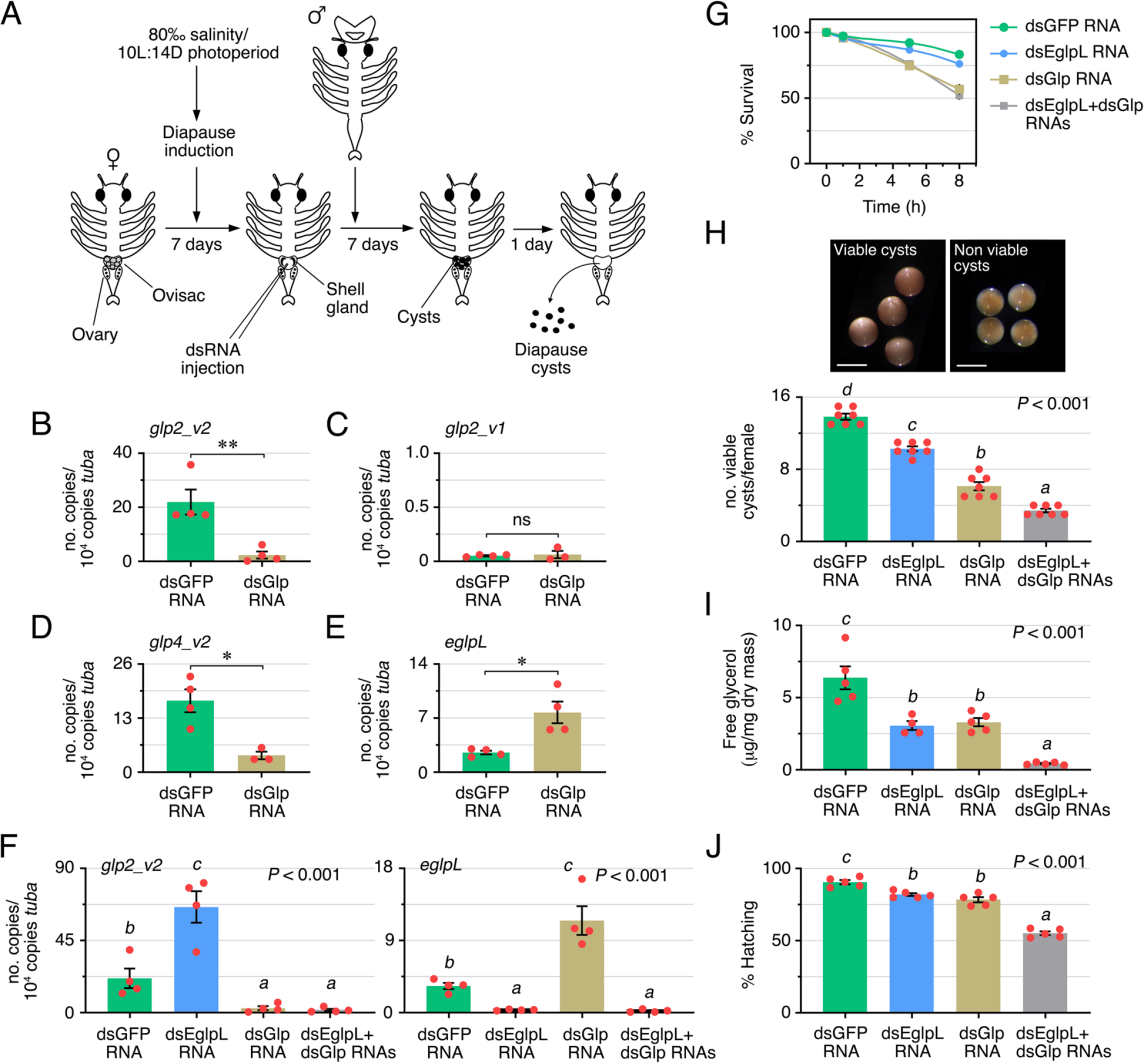
Knockdown of glycerol channels reduces oviparous reproduction success, glycerol accumulation, and stress tolerance of *A. franciscana* cysts. **A** Schematic diagram of the protocol employed for the dsRNA-mediated knockdown of *eglpL* and *glp* mRNAs in *A. franciscana* embryos. Transcript levels of *glp2_v2* (**B**), *glp2_v1* (**C**), *glp4_v2* (**D**), and *eglpL* (**E**) in cysts released from oviparous females injected with GFP (control) or Glp dsRNAs (dsGFP and dsGlp, respectively) determined by RT-qPCR using α-tubulin mRNA as reference. **F** Transcript levels of *glp2_v2* (left panel) and *eglpL* (right panel) in cysts produced by females injected with dsGlp RNA, dsEglpL RNA, or both, determined as above. **G** Survival of females injected with single or double dsRNAs as in (**F**). **H** Number of viable cysts upon release from females treated with the different dsRNAs as in (**F**). Above the panel, photomicrographs of viable (dark brown color) and non-viable (light brown color and partially transparent) diapause cysts are shown. Scale bars 250 µm. **I** Free glycerol content in cysts released by control females and females injected with dsEglpL and/or dsGlp RNAs. **J** Changes in the hatching rates at 16 h post-activation of cysts produced by females treated as in (**I**) after desiccation-mediated diapause termination. In (**B**–**E**), data (mean ± SEM; *n* = 3–4 independent experiments, red dots) were statistically analyzed by an unpaired Student’s *t*-test (**, *p* < 0.01; *, *p* < 0.05). ns, not statistically significant. In the rest of the panels, data (mean ± SEM; *n* = 4–7 independent experiments, red dots) was statistically analyzed by one-way ANOVA or the Kruskal–Wallis test, followed by the Tukey’s multiple comparisons test or Dunn’s multiple comparisons test, respectively. Bars with different superscripts are significantly different (*p* values indicated in each panel)

The previous data indicate that Glp2_v2 was the only paralog with increased mRNA expression concomitant with the highest accumulation of glycerol in diapause cysts, suggesting that this channel may be the major mediator of glycerol transport during the formation of *A. franciscana* encysted embryos. To examine this hypothesis, we used the double-stranded RNA (dsRNA) mediated RNAi method to silence the transcription of *glp2_v2* and determine the effects on oviparous reproduction and cysts stress tolerance. However, since the *glp2_v2* cDNA shows 90% identity with both *glp4* isoforms at the nucleotide level, we designed and synthesized in vitro a dsRNA that could potentially reduce the transcription of both *A. franciscana glp2* and *glp4* genes and their isoforms. This dsRNA (named dsGlp RNA) covered a target nucleotide sequence of the *glp2_v2* cDNA showing 100% identity to that of *glp2_v1* and 92% identity to those of *glp4_v1* and *glp4_v2*, but only 27% identity to the *eglpL* nucleotide sequence. As control, we used a dsRNA designed against the transcript of the green fluorescent protein (GFP) from the jellyfish *Aequorea victoria* (termed dsGFP RNA). The dsGlp and dsGFP RNAs, both containing phenol red, were injected into the ovisac of females maintained at high salinity and short photoperiod (10 h light:14 h dark) to induce the production of cysts, which show a brown shell gland (Fig. 5A). Females exhibiting phenol red staining throughout the animal for at least 2 h after injection and normal behavior were subsequently mixed with males for fertilization, and released cysts were collected after ~ 7 days (Fig. 5A).

Determination of transcript levels by RT-qPCR at the end of the experiment verified that dsGlp RNA injection of females reduced the *glp2_v2* and *glp4_v2* mRNAs in released diapause cysts by 90% and 76%, respectively, with respect to cysts produced by females receiving dsGFP RNA (Fig. 5B and D). However, the *glp2_v1* mRNA levels were not affected, possibly because the transcript levels in the control cysts were already very low (Fig. 5C), as previously observed (Fig. 4G). For the same reason, the cyst *glp4_v1* mRNA levels after dsGFP or dsGlp RNA injection were not determined. Nevertheless, we observed that dsGlp RNA injection of oviparous females increased *eglpL* transcript levels in the cysts by ~ threefold with respect to the controls (Fig. 5E). This indicated a potential genetic compensation mechanism between *glps* and *eglpL*. To investigate this possibility, we injected females with dsGlp and dsEglpL RNAs separately, or with a mixture (1:1) of both dsRNAs, and determined the transcript levels of *glp2_v2* and *eglpL* in diapause cysts. The results showed that the cyst *glp2_v2* mRNA levels were reduced by 96% and 92% in response to dsGlp and dsGlp + dsEglpL RNAs, respectively, compared to those produced by dsGFP RNA-treated females (Fig. 5F). However, treatment of females with dsEglpL RNA alone resulted in an ~ threefold mRNA increment of *glp2_v2* in cysts (Fig. 5F). For the *eglpL* mRNA, we found that injection of dsEglpL RNA alone or in combination with dsGlp RNA also reduced very efficiently the *eglpL* cyst transcript levels (90% and 93% inhibition, respectively), while we again observed that dsGlp RNA induced an ~ threefold increase in the *eglpL* mRNA (Fig. 5F). These findings therefore suggest that *glps* and *eglpL* can genetically compensate each other at the RNA level in *A. franciscana* cysts in response to dsRNA-mediated specific gene knockdown of either of the two types of channels.

The effects of single and double RNAi treatment on female survival, success of oviparous development, cyst glycerol accumulation, and stress tolerance, were subsequently examined. *A. franciscana* females injected with dsGFP or dsEglpL RNAs showed 8 days post-injection survival rates of 83 ± 1% and 76 ± 1% (mean ± SEM), respectively, while the survival rates of females treated with dsGlp RNA alone or together with dsEglpL RNA dropped to 57 ± 2% and 52 ± 1%, respectively (Fig. 5G). The females that survived in each of the treatment groups showed a progressive decrease in the production of viable cysts in diapause, i.e., showing a brown or dark brown color (14, 10, 6, and 3 viable cysts/female, in females treated with dsGFP, dsEglpL, dsGlp, and dsEglpL + dsGlp RNAs, respectively) (Fig. 5H). These data suggest that both EglpL and Glp2_v2 are involved in pathways that promote the survival of *A. franciscana* females under high salinity stress as well as successful oviparous development.

The glycerol accumulation in cysts produced by the dsRNA-injected females was measured in isolated viable cysts. Treatment of females with either dsEglpL or dsGlp RNAs reduced the free glycerol levels in cysts by 52% and 48%, respectively, with respect to the cysts produced by the dsGFP RNA-injected females, while the combination of both dsRNAs further decreased the cyst glycerol content by 93% with respect to the controls (Fig. 5I). To determine the longer-term developmental outcomes, the hatching rates of the viable cysts produced by each group were investigated by desiccation-mediated diapause termination, followed by activation in salt water at low salinity and constant light. This revealed that the hatching rate of cysts at 16 h post-activation paralleled their glycerol levels at diapause with 91 ± 1%, 82 ± 1%, 78 ± 2%, and 55 ± 1% in cysts produced by females treated with dsGFP, dsEglpL, dsGlp, or dsEglpL + dsGlp RNAs, respectively (Fig. 5J). These observations suggest that glycerol accumulation in *A. franciscana* diapause cysts requires both the EglpL and Glp channels, and that the observed genetic compensation at the mRNA level is not enough to reverse the reduction of glycerol levels.

### EglpL- and Glp2_v2-mediated glycerol accumulation in diapause cysts is necessary for anhydrobiosis but not developmental activation

**Fig. 6:**
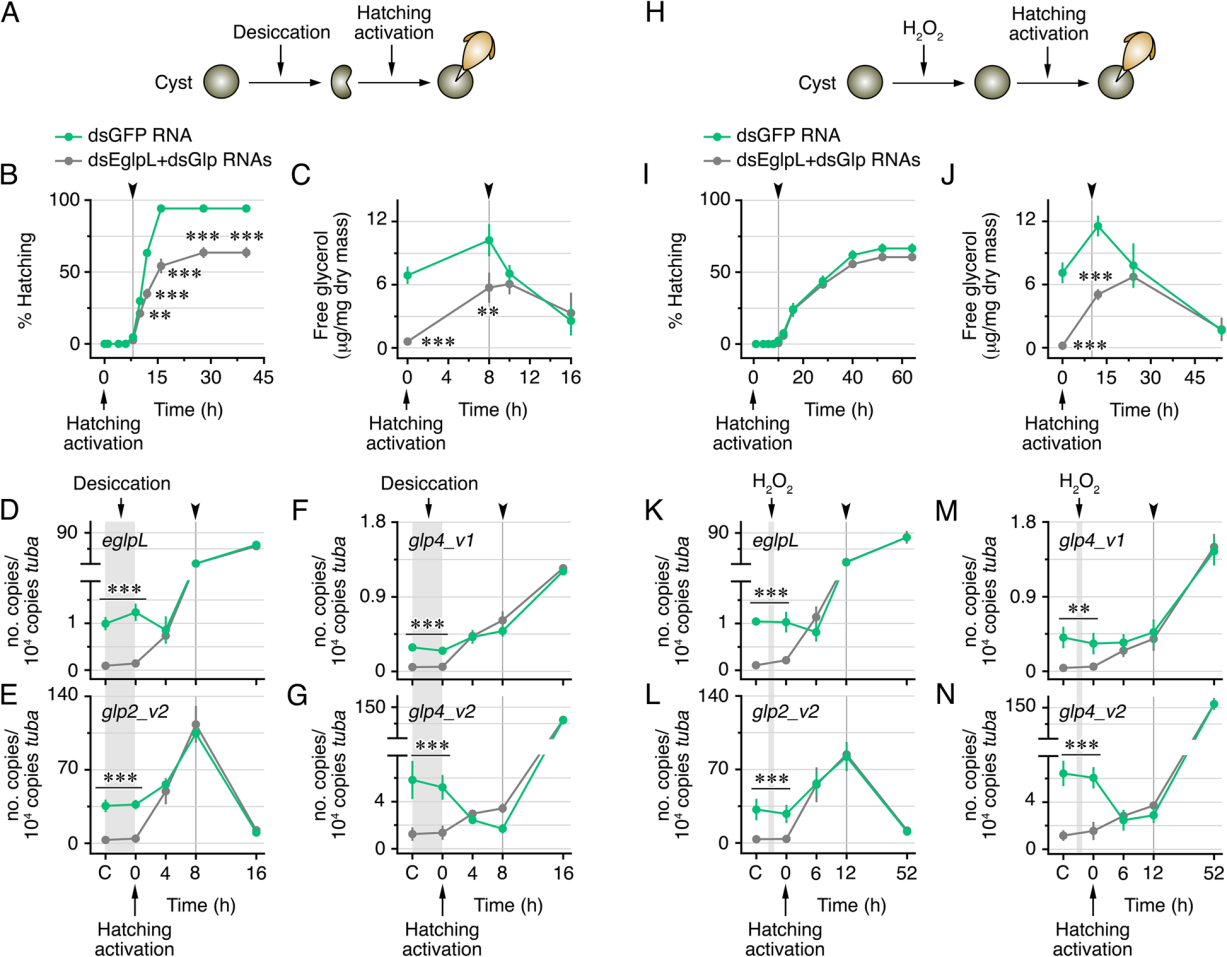
EglpL- and Glp knockdown-mediated glycerol depletion of *A. franciscana* cysts impairs desiccation tolerance but not diapause termination and hatching competence. Time-course of hatching rates and glycerol accumulation in cysts produced by females injected with dsGFP (control) or dsEglpL + dsGlp RNAs, in which diapause was terminated by 48 h of desiccation (**A**–**C**) or 1 h of H_2_O_2_ treatment (**H**–**J**) before hatching activation. Data (mean ± SEM) compiled from 2 to 3 independent experiments were statistically analyzed by an unpaired Student’s *t*-test (**, *p* < 0.01; ***, *p* < 0.001, with respect to the controls at each time point). ns, not statistically significant. Transcript levels of *eglpL*, *glp2_v2*, *glp4_v1*, and *glp4_v2* determined by RT-qPCR using α-tubulin mRNA as reference, in cysts from the two groups before and after diapause termination by desiccation (**D**–**G**) or H_2_O_2_ (**K**–**N**) and during hatching. In some panels, the data from the dsEglpL + dsGlp RNAs group are not visible because they completely overlap with those from the controls. The arrowhead in each panel indicates the time of hatching initiation. Data (mean ± SEM; *n* = 3–4 independent experiments) were statistically analyzed by an unpaired Student’s *t*-test (**, *p* < 0.01; ***, *p* < 0.001, with respect to the dsEglpL + dsGlp-treated group at each time point)

To examine in more detail whether EglpL- and Glp-mediated glycerol accumulation in diapause cysts is required for desiccation tolerance, the activation of embryo development, or both, we conducted additional experiments in which diapause of *eglpL* + *glp*-depleted and control cysts was terminated by either desiccation or a short treatment with H_2_O_2_ [11] (Fig. 6A and H). We subsequently determined the time-course of hatching, glycerol accumulation, and the expression of the *eglpL* and *glp* genes. The data confirmed that desiccation-mediated diapause termination reduced the hatching rates of *eglpL* + *glp*-depleted cysts by ~ 33% with respect to the controls from 28 h post-activation onwards (Fig. 6B). The glycerol levels in control cysts were elevated at 8 h post-activation, when hatching was initiated, whereas they rapidly decreased at 10 h, and further dropped at 16 h when maximum hatching was observed (Fig. 6C). Interestingly, although the glycerol levels in *eglpL* + *glp*-depleted desiccated cysts were ~ 10 times lower than in the dsGFP RNA-treated controls at activation, the concentration of glycerol was strongly increased by 8 h, and then paralleled the level and subsequent decrease in the controls between 10 and 16 h post-activation (Fig. 6C). The expression levels of *eglpL*, *glp2_v2*, *glp4_v1*, and *glp4_v2* in cysts produced by females treated with dsEglp + dsGlp RNAs remained lower than in the controls during the desiccation treatment (Fig. 6D–G). However, in both groups the *eglpL*, *glp4_v1*, and *glp4_v2* mRNAs rapidly increased at the onset of hatching and onwards (Fig. 6D, F, and G), while those of *glp2_v2* specifically accumulated upon the initiation of hatching and strongly decreased thereafter when hatching was completed (Fig. 6E). The expression of *glp2_v1* mRNA remained equally low in both *eglpL* + *glp*-depleted and control cysts, and only increased when hatching was completed (Additional file 8: Fig. S4A).

In contrast to the above observations using desiccation as diapause terminating signal, the hatching rates of cysts with depleted glycerol transporters in which diapause was broken with H_2_O_2_ did not differ from the controls, even though the glycerol content in these cysts was strongly reduced compared to the controls (Fig. 6I and J). The *eglpL*, *glp2_v1*, *glp2_v2*, *glp4_v1*, and *glp4_v2* mRNAs in cysts undergoing hatching after H_2_O_2_ treatment were nevertheless regulated in same manner as the desiccated treated cysts (Fig. 6K–N and Additional file 8: Fig. S4B). These observations suggest that EglpL + Glp-mediated glycerol accumulation in *A. franciscana* diapause cysts is therefore required for anhydrobiosis and not the activation of embryo development or hatching. In addition, our data indicate that diapause termination through either desiccation or H_2_O_2_ exposure breaks dsRNA-mediated depletion of the glycerol channels, since their expression levels are upregulated during subsequent embryogenesis and hatching.

### Glycerol has a minor role for freeze tolerance of *A. franciscana* diapause embryos

**Fig. 7:**
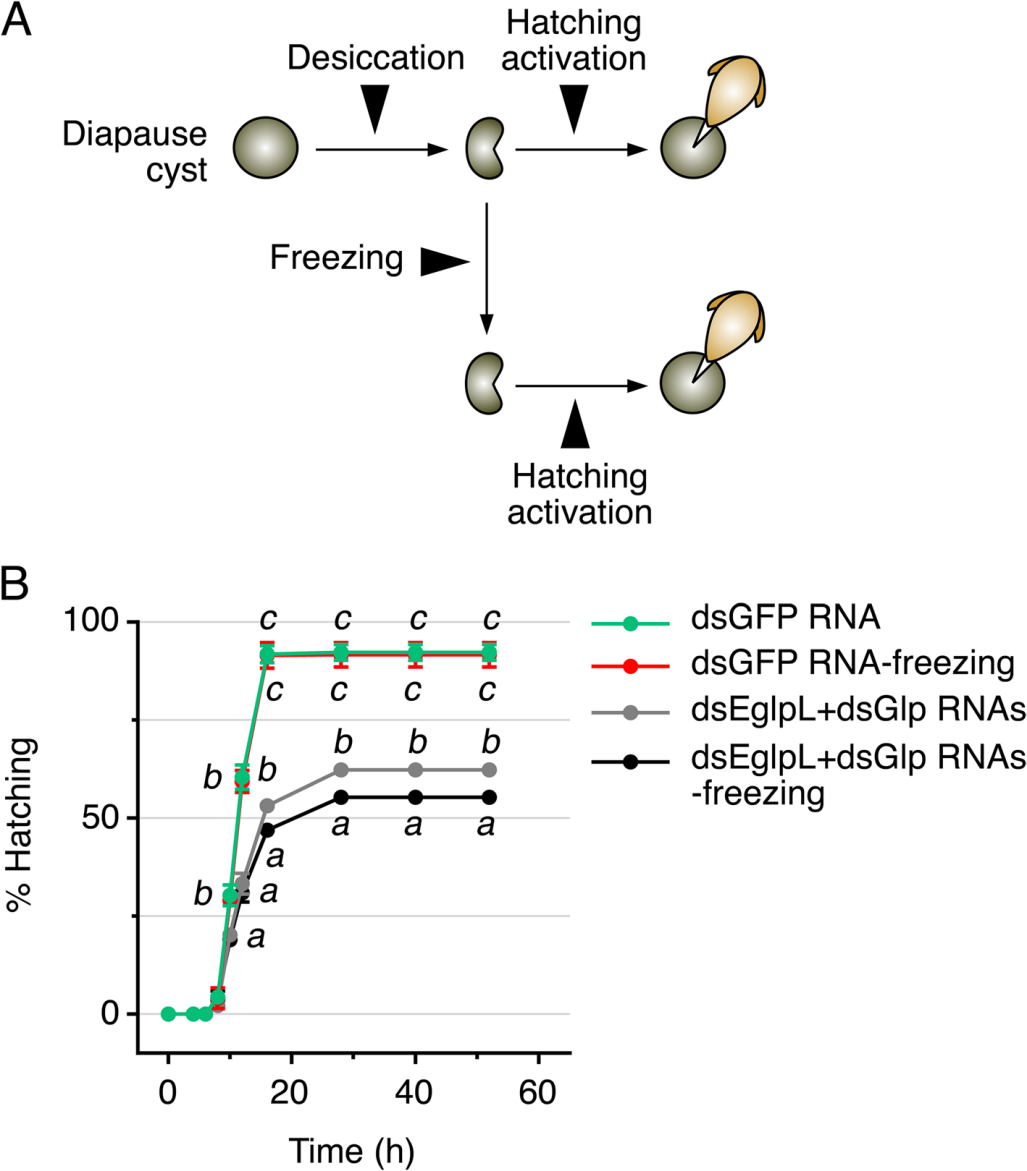
EglpL and Glp gene knockdown slightly reduces freeze tolerance of *A. franciscana* desiccated cysts. **A** Cysts produced by dsEglpL + dsGlp RNAs-treated broods were desiccated for 48 h, flash frozen or not in liquid nitrogen, and activated for hatching. **B** Time-course of hatching rates of cysts treated as above. Data (mean ± SEM, *n* = 4 separate experiments) were analyzed by one-way ANOVA followed by the Tukey’s multiple comparisons test, at each time point. Dots with different superscript are statistically different (*p* < 0.001)

We finally investigated whether the glycerol levels in *A. franciscana* diapause cysts could impair freeze tolerance in addition to anhydrobiosis. To independently test the effect of freezing, we initially conducted an experiment in which cysts produced by females injected with dsEglpL + dsGlp RNAs or by control females treated with dsGFP RNA were frozen. In both cases, survival at hatching was ~ 3% (Additional file 9: Table S2) demonstrating that hydrated cysts are not freeze-tolerant regardless of the presence or absence of glycerol. To test the effect of freezing, we therefore prepared desiccated cysts with and without depleted glycerol as above and evaluated whether or not freezing stress induces an additional effect on survival. In this experiment, desiccated, unfrozen dsGFP RNA control cysts showed an equal percentage of hatching (92 ± 1%) compared to desiccated + frozen cysts (92 ± 2%) (Fig. 7B). Conversely, desiccated and unfrozen *eglpL* + *glp*-depleted cysts showed a hatching rate of 62 ± 1%, which was a 32% reduction with respect to the controls, as previously observed (Fig. 7B). However, the hatching percentage of the desiccated + frozen *eglpL* + *glp*-depleted cysts was of 55 ± 1%, thus showing an increment of only 7% in the reduction of survival with respect to desiccated *eglpL* + *glp*-silenced cysts (Fig. 7B). Although we cannot rule out that the cysts that did not survive desiccation would also not have survived freezing, these data suggest that glycerol accumulation in *A. franciscana* diapause cysts plays a more critical role in anhydrobiosis rather than cryobiosis.

## Discussion

The identification of co-opted EglpL channels in crustaceans in the present work was unexpected. Previously, such channels have only been characterized in hexapods, in which substitution of the AQP4-related TMD5 His in the ar/R selectivity filter for a smaller uncharged residue resulted in more efficient glycerol permeation compared to the canonical Glps [23]. This latter biophysical property is considered to underly the selective basis for supplantation of the canonical Glps by Eglps in hemipteran and holometabolous insects [23, 33].

In the present context, the branchiopod AQP4-related EglpL channels co-exist with canonical Glps as in basal lineages of hexapods [23], but have been positively selected at the expense of PripL channels found in other arthropods. Beyond water transport, Eglp and Prip channels also permeate urea [23, 28, 34]; however, whether this function contributed to the loss of PripL channels in branchiopods remains to be established. Alternatively, the pseudoexon/gene fragments observed within and downstream of the *A. franciscana* and *A. sinica eglpL* channels could represent degraded vestiges of the *pripL* genes; however, the fragments are too short for phylogenetic investigation. Phylogenetic analyses of arthropod AQP4-related CDS nevertheless shows that ostracods, a basal class of extant crustaceans [26], and branchiopods encode EglpL channels, while none was found in other classes of Crustacea. These inferences were strengthened via site-directed mutagenesis and functional tests of glycerol permeation of the *A. franciscana* EglpL channel, which confirmed that substitution of the smaller uncharged TMD5 ar/R residue Ala to a PripL His abolished the function. The combined data sets therefore suggest that EglpL channels may have evolved in the common ancestor of the Pancrustacea during the early Cambrian period, and were differentially retained in Ostracoda, Branchiopoda, and Hexapoda, but seemingly lost in other classes of Crustacea.

Glycerol accumulation has been observed in anhydrobiotic organisms; however, it has primarily been considered as a compatible osmolyte or respiratory carbon source for resistance to water loss, cold hardiness, or cryobiosis [16, 18, 35–39]. The former anti-desiccation function is consistent with the role of glycerol in hydrobiotic organisms under high osmotic stress [40–44]. In this study, we show that glycerol accumulation is restricted to the oviparously released diapause cysts and that RNAi-mediated knockdown of both the co-opted EglpL and canonical Glps is necessary to prevent the accumulation. This shows that aquaporin-type glycerol transporters mediate accrual of the polyol in *A. franciscana* diapause cysts and uncovers a subfunctionalized physiological role of EglpL channels in arthropods. However, selective knockdown of either the EglpL or the Glp channels only reduced glycerol accumulation by ~ 50% in viable cysts, and respectively resulted in a genetic compensation of increased dosage of the *glp2_v2* isoform and *eglpL* transcript. Since genetic compensation did not result in recovery of glycerol accumulation beyond ~ 50%, our data suggest that each type of glycerol channel is necessary but not sufficient to reach maximum accrual of the polyol in diapause cysts.

Unlike trehalose, which is considered to be synthesized in the embryo [6], the source of glycerol in *A. franciscana* diapause embryos remains unknown. Although our data do not solve this question, they do indicate that ovisacs containing developing cysts show elevated levels of glycerol compared to ovisacs destined to produce nauplii. These data suggest that glycerol may originate from the female hemolymph and accumulate in the ovisac, to be further transferred to the embryo entering into diapause via the EglpL and Glp channels. Alternatively, glycerol might be produced by certain embryonic cells and further be distributed to other cells in the embryo via the glycerol channels. In any event, the observation that knockdown of *eglpL* and *glp* genes reduces the amount of glycerol in the cysts by approximately the same proportion as the expression levels of *eglpl* and *glp2_v2* (~ 93% in both cases) reinforces the notion that both channels are essential for glycerol accumulation in diapause embryos. Further investigation is, however, needed to clarify the source of glycerol synthesis during oviparous reproduction in *A. franciscana*.

In the present study, we confirm that hydrated cysts are not freeze-tolerant regardless of the presence or absence of glycerol, and that survival in the face of freezing stress requires the absence of water. The RNAi-mediated depletion of cyst glycerol and sequential analysis of desiccation and freezing stress suggest that the polyol has a more important role for desiccation tolerance. It is known that anhydrobiosis and cryobiosis require water replacement and the formation of vitrified glass-like states to immobilize macromolecular and cellular fluctuation [45–47]. Nevertheless, the type of glass formed depends on the effector composition, with in vitro investigations of the physical chemical properties of dehydrated trehalose-glycerol mixes showing that addition of small quantities of glycerol minimizes the glass-transition temperature suppression and fragility of vitrified matrices required for high viscosity biopharmaceutical stabilization [19]. Higher glass-transition temperatures and lower glass former fragilities are found in the desiccation-tolerant stages compared to the desiccation-intolerant stages [20]. However, an in vivo role of glycerol in organism anhydrobiosis had not previously been reported. The present in vivo observations that some diapause cysts with almost no glycerol can survive desiccation and still show high freeze tolerance suggest that trehalose accumulation pathways contributing to glass transition [6] were unaffected by the RNAi-mediated inhibition. However, the evolutionary recruitment of glycerol-transporting pathways during oviparous cyst formation appears to selectively enhance the vitrified state to maximize viability during anhydrobiotic dormancy.

Reaffirmation of the role of glycerol in anhydrobiosis arose from the experiments using either desiccation or H_2_O_2_ as the diapause-termination signal. In this instance, knockdown of the glycerol transporters significantly impaired the hatching viability of desiccated cysts, but not those treated with H_2_O_2_. This occurred despite equivalent levels of glycerol depletion. These results not only imply that glycerol is necessary for anhydrobiosis, but that desiccation is an obligate process that enhances oviparous embryonic viability. These experiments further revealed, however, that embryos that activated development re-initiated transcriptional regulation of the glycerol transporters, in addition to the synthesis of glycerol from the metabolism of trehalose [35]. The former observation suggests that the dsRNA degradome, a natural defense mechanism in eukaryotes against viral infection [48, 49], may be activated in developing nauplii that are no longer afforded the protection of the cyst capsule. It is noteworthy, however, that there is a disconnect in the transcriptional increase in the steady state levels of the glycerol transporters and the glycerol content once hatching is completed. The depletion of glycerol in hatched nauplii is nevertheless consistent with diapause termination in insects [50] and with previous observations that encysted anostracan crustaceans initially utilize glycerol to generate osmotic pressure for hatching and subsequently as a metabolic substrate for growth [13, 35, 51]. Similarly, the knockdown of the *A. franciscana* Glps, but not the EglpL channels, had a deleterious effect on the survival of adult females transferred to high salinity indicating that the canonical glycerol-transporting pathways continue to play a more conventional role in the osmohomeostasis and metabolism of this stage of the life cycle.

## Conclusions

This study shows that EglpL channels likely evolved during the early Cambrian period in the common ancestor of the Pancrustacea and are subfunctionally regulated with canonical Glps to facilitate glycerol accumulation in the oviparous cysts of the extremophile crustacean *A. franciscana*. In contrast to the widely held notion that glycerol primarily contributes to cryobiosis during arthropod dormancy, our data reveal that the acquired glycerol confers a vital in vivo capacity for anhydrobiosis.

## Methods

### Experimental animals and sample collection

Commercially available *A. franciscana* dried cysts (INVE Aquaculture Thailand, EG Artemia, distributed by ACUAZUL, S.C., Spain, #81544) were hydrated and hatched overnight at 28°C in conic tanks filled with 30 g/l salt water (Reef Salt SPS grade, #7740020) under vigorous aeration and constant illumination. Free-swimming nauplii were collected by filtering through a150-μm net and grown to adult stage in 30 g/l salt water at 28°C with gentle aeration. Animals were fed daily with a mixture of microalgae and probiotic microorganisms commercially available (RotiBomb #ROB0050G, Algova, Spain). To induce the oviparous development, adults were maintained under high salinity (80 g/l) and short photoperiod (10 h white light:14 h dark) for 7 days [52]. Females with oocytes programmed to develop oviparously were identified by a brown shell gland appearing in the ovisac [53]. Samples of released cysts and free-swimming nauplii for molecular analysis were collected from the bottom of the aquarium and by filtering the aquarium water, respectively. These samples were transferred into a Petri dish, and viable cysts and nauplii were sorted under a stereo microscope using a 200-μl micropipette. The ovisacs from females developing ovoviviparously or oviparously were dissected from animals anaesthetized with 60% ethanol using watchmaker forceps (Dumont No. 5, Merck #F6521) under a stereo microscope. All samples were washed twice with sterile water to remove the salts, frozen in liquid nitrogen, and stored at − 80°C.

Adult *X. laevis* were purchased from the Centre de Ressources Biologiques Xénopes (University of Rennes, France) and maintained at the AQUAB facilities of the Universitat Autònoma de Barcelona (UAB, Spain) as previously described [54]. Oocytes were collected by surgical laparotomy from anesthetized females following a procedure approved by the Ethics Committee for Animal and Human Experimentation (CEEAH) from UAB and the Catalan Government (Direcció General de Polítiques Ambientals i Medi Natural; Project no. 10985).

### Sequencing of *A. franciscana* transcriptome

Total RNA from a pool of adult males and females, nauplii, and dried cysts were extracted with the GenElute mammalian RNA extraction kit (Merck, #RTN70) and treated with DNAse I (Merck, #DNASE10) following the manufacturer’s instructions. PolyA^+^ RNA was further purified using the Dynabeads™ mRNA Purification Kit (Invitrogen, #61006) following the manufacturer’s instructions. The full-spectrum UV–Vis spectro-photometer NanoDropVC 2000 (Thermo Fisher Scientific) was used to determine the purity and concentration of the extracted RNA by measuring their 260/280 nm absorbance ratio. RNA size distribution profiles were analyzed using the Agilent 2100 Bioanalyzer (Agilent Technologies). The RIN values for the total RNA samples were 8.60 (cysts), 7.20 (nauplii), and 9.20 (adults).

For each of the three developmental stages collected, we constructed one stranded RNA library from total RNA for NovaSeq6000 S4 (Illumina) short read sequencing, and another library constructed from polyA^+^ RNA for long read sequencing using ONT. For Illumina sequencing, libraries were constructed with the KAPA stranded mRNA kit (Kapa Biosystems, #KR0960-v6.17) and sequenced in paired-end mode with a read length of 2 × 76 bp using TruSeq SBS Kit v4. We generated more than 50 million paired-end reads for each sample in a fraction of a sequencing v4 flow cell lane, following the manufacturer’s protocol. Image analysis, base calling, and quality scoring of the run were processed using the manufacturer’s software Real Time Analysis (RTA 1.18.66.3) and followed by generation of FASTQ sequence files by CASAVA 1.8. Transcriptome long read sequencing libraries were prepared using the Direct cDNA Sequencing Kit (ONT, #SQK-DCS109), following the manufacturer’s instructions. Sequencing runs were performed on GridION™ Mk1 (ONT) using a Flowcell R9.4.1 FLO-MIN106D (ONT). The quality parameters of the sequencing runs were monitored in real time using the MinKNOW™ platform (version 22.05.7), and the base calling was performed using Guppy (ONT, version 6.1.5; Oxford Nanopore Technologies, Oxford, UK).

The *A. franciscana* transcriptome assembly was obtained using a combination of short Illumina reads and long cDNA sequencing ONT reads. RNA-Bloom [55] with default parameters was used to assemble the reads into a combined transcriptome. To improve the base accuracy of the transcripts, Ratatosk [56] was run to correct the assembled transcripts using the Illumina data. Next, the obtained transcripts were clustered using RapClust [57], which realigns the Illumina data to determine which assembled sequences belong to the same biological transcript and/or gene. The whole assembly workflow is summarized in Additional file 10: Fig. S5. Finally, the open reading frames were annotated in the transcripts with Transdecoder [58], and the resulting peptides were functionally annotated using a Diamond Blastp [59] search against the nr database (last accessed in April 2022), followed by Interproscan [60] to detect protein domains in the annotated proteins. All these data were combined by Blast2GO [61], which produced the functional annotation results. To complement the annotations produced by Blast2GO, the *Daphnia magna* proteins were aligned to the *A. franciscana* proteins with BLAST [25] and functions were added to those assembled transcripts that were lacking a functional annotation after Blast2GO but that could be assigned to a *D. magna* protein.

### Aquaporin sequence assembly, phylogenetic analyses, and modeling

Aquaporin CDS were obtained from the transcriptome and clones or assembled from open-source whole genome shotgun, transcriptome shotgun, and nucleotide databases (blast.ncbi.nlm.nih.gov and ensembl.org). For CDS assembly, either full-length proteins or exon-deduced peptides were used as tblastn queries as described previously [27, 62]. Full-length proteins were aligned to generate multiple sequence alignments using the G-INS-I algorithm of MAFFT v7.453 [63]. Corresponding nucleotide sequences were then retrieved from the respective DNA contigs or linkage groups, trimmed to match each peptide, and subsequently converted to codon alignments using Pal2Nal [64]. Phylogenetic analyses were conducted via Mr Bayes v3.2.7a [65] of the full-length alignments following removal of gapped regions containing less than three sequences. Bayesian model parameters were nucmodel = 4by4, nst = 2, rates = gamma for codon alignments and aamodel = mixed for amino acid alignments. Between 0.5 and 75 million Markov chain Monte Carlo (MCMC) generations were run with three heated and one cold chain with resulting posterior distributions examined for convergence and an effective sample size > 1000 using Tracer version 1.7.1 [66] and majority rule consensus trees summarized with a burnin of 25%. Gene loci were obtained via tBlastn with the respective deduced protein, and gene structures mapped using the exons. Homology modeling of insect Prip and *Artemia* EglpL channels was conducted via the Swiss-Model data pipeline with default parameters using 7uze as a template. The resultant PDB files were rendered with PyMOL (pymol.org).

### Cloning of *A. franciscana* aquaporin cDNAs and site-directed mutagenesis

Full-length cDNAs encoding *A. franciscana* EglpL, Glp2_v1, Glp2_v2, Glp4_v1, and Glp4_v2 were isolated by RT-PCR using adult or cysts total RNA as previously described [54]. Oligonucleotide primers were designed based on the *A. franciscana* transcriptome sequenced in this study and genome sequence data [67]. The cDNAs were subcloned into the pT7Ts expression vector for further expression in frog oocytes [68] and fused with an HA epitope tag at the C-terminus of the encoded proteins by using PCR. The A187H mutation was introduced into the EglpL amino acid sequence by using the QuikChange lightning kit (Agilent Technologies, #210518) following the manufacturer’s instructions. Sanger sequencing was carried out to verify that the mutation was introduced.

### Functional characterization of *A. franciscana* aquaporins

Water and glycerol permeabilities of the *A. franciscana* aquaporins were tested using the *X. laevis* oocyte expression system as previously described [68]. Oocytes were injected with 15 ng of cRNA of each aquaporin or not injected (controls). The *P*_gly_ was determined volumetrically in isotonic Modified Barth’s Solution (MBS: 88 mM NaCl, 1 mM KCl, 2.4 mM, NaHCO_3_, 0.82 mM MgSO_4_, 0.33 mM Ca(NO_3_)_2_, 0.41 mM CaCl_2_, 10 mM HEPES, and 25 μg/ml gentamycin) at pH 7.5, where NaCl was replaced by 160 mM glycerol. The osmolarity of the solutions was measured with a vapor pressure osmometer (Vapro 5600, Wescor, USA) and adjusted to 200 mOsm with NaCl if necessary.

### Immunofluorescence microscopy and immunoblotting

*X. laevis* oocytes expressing HA-tagged aquaporin constructs were fixed for 6 h in 4% paraformaldehyde in PBS (137 mM NaCl, 2.7 mM KCl, 10 mM Na_2_HPO_4_, 1.8 mM KH2PO4, pH 7.5), dehydrated, and mounted in paraffin as described elsewhere [54]. Sections (8 μm) were blocked with PBST (PBS with 0.05% Tween-20) containing 5% normal goat serum and 0.1% BSA for 1 h at room temperature and incubated (1:400) with anti-HA antibodies (Invitrogen, #PA1-985) overnight at 4°C in a humidified chamber. Sections were subsequently washed three times with PBS and incubated (1:800) with sheep anti-rabbit IgG coupled with Cy3 (Merck, #C2306) for 1 h at room temperature. The sections were mounted with fluoromount aqueous anti-fading medium (Merck, #F4680), and images were acquired with a Zeiss Axio Imager Z1/ApoTome fluorescence microscope (CarlZeiss Corp., Oberkochen, Germany).

The isolation of the total membrane fraction of *X. laevis* oocytes (*n* = 10) and immunoblotting were carried out as described previously [53, 69]. Protein extracts were treated or not with 500 units of PNGase F (New England Biolabs Inc., #P0704) for 3 h at 37°C prior to SDS-PAGE. Membranes were incubated (1:1000) with HA antibodies overnight at 4°C, and horseradish peroxidase-conjugated goat anti-rabbit IgG secondary antibodies (Bio-rad, #172–1019) were used at 1:5000 dilution for 1 h at room temperature.

### Free glycerol determination

Samples of ovisacs destined to produce nauplii or cysts (50–100 mg), free-swimming nauplii and cysts (20–40 mg each) were homogenized in 350 μl of PBS in a glass tissue manual homogenizer, filtered in 200 mesh net, and centrifuged at 1600 × *g* for 10 min at 4°C to precipitate solid residues. Total glycerol levels from an aliquot of the supernatant (50 µl) were measured using the Amplite™ Fluorimetric Glycerol Assay Kit (ATT Bioquest) following the manufacturer’s instructions. Data were expressed as dry mass, previous gravimetric determination of the water content of each sample.

### RT-qPCR gene expression analysis

Total RNA was purified as described above, and the cDNA was synthetized from 1 μg of total RNA using the Superscript IV reverse transcriptase enzyme (Invitrogen, #18090010) following the manufacturer’s instructions. The qPCR reactions were performed in a final volume of 10 μl with 1 μl of freshly made cDNA, 5 μl of iTaq Universal SYBR Green Supermix (BioRad, #1725120), and 0.5 μM of oligonucleotide primers specific for EglpL, Glp2_v1, Glp2_v2, Glp4_v1, and Glp4_v2 (Additional file 11: Table S3). The amplification reactions were carried out with an initial denaturing step of 3 min at 95°C, followed by 40 cycles at 95°C for 10 s and 60°C for 30 s, ending with a melting curve from 65 to 95°C increasing 0.5°C every 5 s. Copy numbers were determined from a standard curve of the Ct values and normalized against *A. franciscana* α-tubulin.

### RNAi knockdown of *A. franciscana* EglpL and Glps

The MEGAscript® RNAi kit (Invitrogen, #AM1626) was used for dsRNA synthesis and purification following the single PCR protocol, in which a T7 promoter sequence is added to the 5′ end of each forward and reverse primer. The pGEM-T easy plasmid (Promega, #A1360) containing the *eglpL* or *glp2_v2* cDNAs were used as templates. The nucleotide sequence of the designed dsEglpL RNA showed 30% identity with that of *glp4_v1* and *glp4_v2* and 27% identity with *glp2_v1* and *glp2_v2*. The dsGlp RNA sequence showed 100% identity to *glp2_v2* and *glp2_v1* and 92% identity to *glp4_v1* and *glp4_v2*. Finally, the dsGFP RNA control nucleotide sequence showed 20% and 30% identity with *A. franciscana eglpL* and *glps*, respectively.

To synthesize each dsRNA, two PCR tubes were prepared containing 40 ng of plasmid, 0.2 μM of forward and reverse primer specific for each dsRNA (Additional file 12: Table S4), and 2.5 U of the Easy-A high-fidelity DNA polymerase (Agilent, #600400) in 50 μl. The PCR conditions were an initial denaturing step of 2 min at 95°C followed by 5 cycles at 95°C for 30 s, 49°C for 30 s, and 72°C for 1 min. This was followed by another 40 cycles at 95°C for 30 s, 65°C for 30 s, and 72°C for 1 min, finishing with 72°C for 1 min. The PCR products were run in 1% agarose (Invitrogen) and purified with High pure PCR product purification kit (Roche, #11732668001) following the manufacturer’s instructions. The PCR bands from the two tubes were pooled and used as template for in vitro dsRNA transcription and purification using the MEGAscript® RNAi kit manufacturer’s instructions. The purified dsRNAs were checked in 1% agarose gels and their quality and concentration measured on a Nanodrop 1000 (Thermo Fisher Scientific).

Prior to dsRNA injection, adult females were separated from males to prevent fertilization and transferred to an aquarium maintained at high salinity and restricted photoperiod for 1 week to induce cyst production [52]. Females showing a brown shell gland in the ovisac were selected for injection, and 40–50 females were used in each experiment. Females were placed in a 3% agarose cold plate for immobilization and the selected dsRNAs were injected into the ovisac [70] employing a digital microinjector (XenoWorks, Sutter Instrument Co.) and a micropipette (Narishige, #GD-1) prepared with a Pipette Puller (Sutter Instrument Co.). To reduce the transcription of a single gene, females were injected with 200 nl of water with 1% phenol red (Merck, #P3532) containing 100 ng of the respective dsRNA, whereas for double depletion females were injected with 50 ng of each dsRNA in the same 200-nl volume. Controls were injected with 100 ng of dsGFP. Females maintaining the dye for 4 h after injection and showing normal swimming behavior were mixed with males 1 day post-injection, and cysts were collected from the aquaria after 7 days. To ensure the specificity and efficiency of the RNAi, 5–10 mg of cyst collected from dsRNA-injected females was used to determine gene expression by RT-qPCR as described above.

### Viability and stress tolerance tests

The survival of females was scored at 1, 5, and 8 days post-injection, whereas the production of cysts per female and the number of viable cysts, i.e., showing brown or dark brown color [71], produced by each female were determined 8 days after injection. The stress tolerance of control and aquaporin-depleted viable cysts was assessed using different protocols. In some experiments, groups of cysts (*n* = 200 per treatment) were placed over a foil inside a Petri dish and desiccated at 28°C for 48 h using a temperature-controlled incubator (IPP30 Memmert GbmH). Subsequently, hatching was activated by transferring the cysts to a 100-ml glass beaker with 30 g/l salt water and under vigorous aeration and constant illumination (white light) at 28°C. The percentage of hatching was determined at different times by filtration of each beaker and counting the number of free-swimming nauplii under a stereo microscope. The hatching rates were calculated as the number of free-swimming nauplii with respect to the total number of cysts. In other experiments, diapause was terminated by exposure of hydrated diapause cysts to 3% H_2_O_2_ for 1 h [11], and subsequently hatched as above. Finally, to test the freezing tolerance of dried cysts, 72 h-desiccated encysted embryos were transferred to an Eppendorf tube and flash frozen in liquid nitrogen for 5 min. Subsequently, the cysts were thawed in a water bath at 37°C for 30 min and hatched.

### Statistical analysis

Data are expressed as mean ± SEM, and percentages were square root transformed prior to analyses. Data were tested for both normal distribution and homogeneity of variance by the Kolmogorov–Smirnov and Bartlett’s tests, respectively. Pairwise statistical comparisons were made by the two-tailed unpaired Student’s *t*-test or by the nonparametric Mann–Whitney’s *U* test. Statistical differences among multiple groups were analyzed by one-way ANOVA, or the nonparametric Kruskal–Wallis test, followed by the Tukey’s multiple comparison test or the Dunn’s test, respectively. Statistical analyses were performed using GraphPad Prism v9.4.1 software (GraphPad Software). In all cases, statistical significance was defined as *p* < 0.05 (*), *p* < 0.01 (**), or *p* < 0.001 (***).

## Supplementary Information

Additional file 1: Table S1. Summary of the *A. franciscana* transcriptome assembly process.

Additional file 2: Fig. S1. Summarized Bayesian majority rule consensus tree of arthropod AQP4-related channels.

Additional file 3: Fig. S2. Annotated Bayesian majority rule consensus tree including accession numbers of Additional file 23: Fig. S1. The data show that within each subcluster, gene duplication is lineage-specific. Amongst Chelicerata, horseshoe crabs (class Merostomata) encode six Bib, six PripL, and two Blip channels, while arachnid spiders and scorpions may have inherited duplicated *pripL* genes from a common ancestor. This also appears to be the case for myriapods as well as malacostracan amphipods and thecostracan barnacles. Conversely, sarcopteriform mites and lepidopteran butterflies and moths evolved divergent forms of PripL channels, including the novel lLepidopteran integral membrane protein (Leip). Some ostracod, branchiopod, and insect lineages also independently duplicated the *eglp*/*eglpL* genes.

Additional file 4: Dataset S1. Codon alignment for Fig. 1.

Additional file 5: Dataset S2. Codon alignment for Figs. S1 and S2.

Additional file 6: Dataset S3. Amino acid alignment for Fig. S3C.

Additional file 7: Fig. S3. Genomic organization of the aquaporin superfamily in *Artemia*. (A) Chromosomal loci. (B) Gene structures drawn to scale with exons depicted as blue bars, introns as linker lines, and pseudoexons and pseudogenes (†) as red bars. kKb, kilobases; nf, not found. (C) Bayesian majority rule consensus tree of *Artemia* Glps analyzed in the study. The tree is midpoint rooted and inferred from 500,000 MCMC generations of 10,071 aligned amino acids (*n* = 30 taxa) with model parameters set to aamodel=mixed. (D) Schematic representation of isoform generation. UER, upstream extended region; UTR, untranslated region.

Additional file 8: Fig. S4. Transcript levels of *glp2_v1* in cysts produced by females injected with dsGFP (control) or dsEglpL+dsGlp before and after diapause termination by desiccation (A) or H_2_O_2_ (B) and during hatching. Data (mean ± SEM; *n*= 3–-4 independent experiments) at each time point were not statistically different between the controls and dsEglpL+dsGlp groups when analyzed by an unpaired Student’s *t*-test.

Additional file 9: Table S2. Effect of freezing on *A. franciscana* hydrated diapause cysts.

Additional file 10: Fig. S5. Summary of the main steps followed to assemble the *A. franciscana* transcriptome generated in this study.

Additional file 11: Table S3. Oligonucleotide primers used for RT-qPCR.

Additional file 12: Table S4. Oligonucleotide primers used for dsRNA synthesis.

Additional file 13: Fig. S6. Uncropped immunoblots from Figs. 2 and 3.

## Data Availability

The A. franciscana transcriptome sequencing data produced in this study have been deposited in the ENA (European Nucleotide Archive; https://www.ebi.ac.uk/ena/browser/home) under the accession number PRJEB80313. The A. franciscana full-length cDNAs for EglpL, Glp4_v1, Glp4_v2, Glp2_v2, Glp2_v1, Aqp12L and Bib were submitted to GenBank (https://submit.ncbi.nlm.nih.gov/subs/genbank/) under accession numbers PQ469250, PQ469251, PQ469252, PQ469253, PQ469254, PQ469255 and PQ469256, respectively. All other relevant data can be found within the article and its supplementary information.

## Abbreviations

AQP: Aquaporin
Ar/R: Aromatic-arginine
Bib: Big brain
Blip: Bib-like integral membrane protein
CDS: Coding DNA sequence
dsRNA: Double-stranded RNA
Eglp: Entomoglyceroporin
EglpL: Entomoglyceroporin-like
Glp: Aquaglyceroporin
HA: Human influenza hemagglutinin
H_2_O_2_: Hydrogen peroxide
LEA: Late embryogenesis abundant
Leip: Lepidopteran integral membrane protein
ONT: Oxford Nanopore Technologies
*P*_f_: Osmotic water permeability
*P*_gly_: Osmotic glycerol permeability
RNAi: RNA interference
RNA-seq: RNA sequencing
RT-qPCR: Real-time quantitative PCR
TMD: Transmembrane domain
UER: Upstream extended region
UTR: Untranslated region
