# Supplementary material for: Co-opted and canonical glycerol channels play a major role during anhydrobiosis of an extremophile crustacean
Source: BMC Biol. 2025 Jun 3;23:151. doi: 10.1186/s12915-025-02262-3 (PMC12135271; doi:10.1186/s12915-025-02262-3)
Supplement: Supplementary file 1 — Additional file 1: Table S1. Summary of the A. franciscana transcriptome assembly process. [file 12915_2025_2262_MOESM1_ESM.pdf]

Table S1. Summary of the *A. franciscana* transcriptome assembly process[1]

Step	No. of transcripts	N. of clusters (genes)	N50	Busco v5 (metazoa_odb10), *n* = 954
Raw ONT reads	23,799,235	-	1,438	C: 93.5%
RNA-Bloom	251,259	-	3,308	C: 92.6%
RNA-Bloom + Ratatosk	251,259	-	3,314	C: 94.1%
RNA-Bloom + Ratatosk + RapClust	187,758	28,929	3,600	C: 94.1%

[1] See Materials and Methods.
