# Supplementary material for: Co-opted and canonical glycerol channels play a major role during anhydrobiosis of an extremophile crustacean
Source: BMC Biol. 2025 Jun 3;23:151. doi: 10.1186/s12915-025-02262-3 (PMC12135271; doi:10.1186/s12915-025-02262-3)
Supplement: Supplementary file 2 — Additional file 2: Fig. S1. Summarized Bayesian majority rule consensus tree of arthropod AQP4-related channels. [file 12915_2025_2262_MOESM2_ESM.pdf]

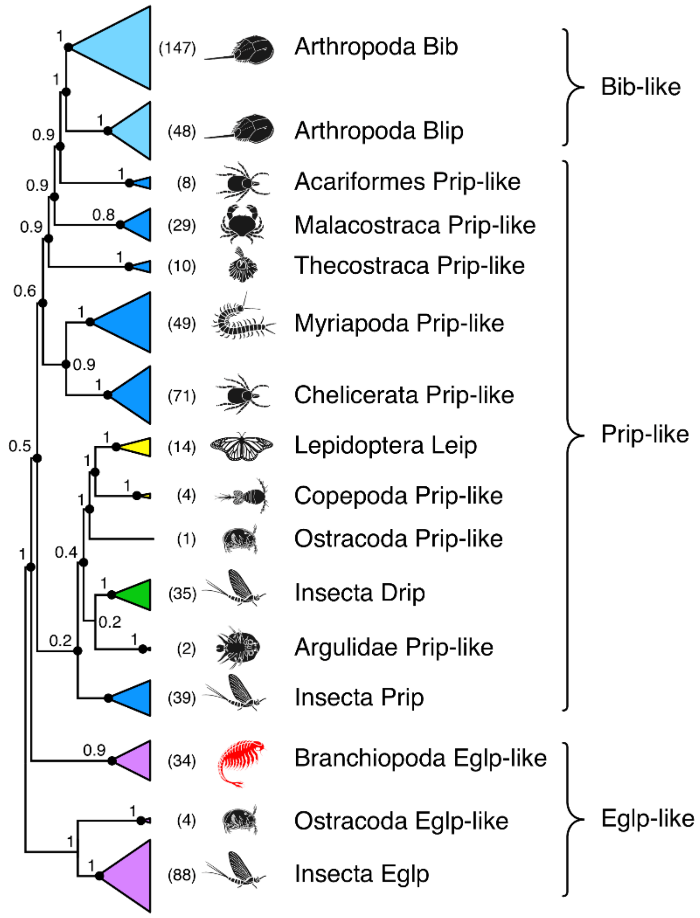

Figure S1. Summarized Bayesian majority rule consensus tree of arthropod AQP4-related channels. The tree is midpoint rooted and inferred from 75 million MCMC generations (nucmodel = 4by4, nst = 2, rates = gamma) of 2,225,342 nucleotide sites aligned by codon (N = 585 taxa). The number of taxa included in each collapsed cluster is indicated in brackets. The fully annotated tree is shown in Additional file 3: Fig. S2, and the nucleotide and amino acid alignments provided in Additional file 5: Dataset S2 and Additional file 6: Dataset S3. Blip, Bib-like integral membrane protein; Leip, Lepidopteran integral membrane protein.
