# Supplementary figures and images for: Co-opted and canonical glycerol channels play a major role during anhydrobiosis of an extremophile crustacean
Source: BMC Biol. 2025 Jun 3;23:151. doi: 10.1186/s12915-025-02262-3 (PMC12135271; doi:10.1186/s12915-025-02262-3)

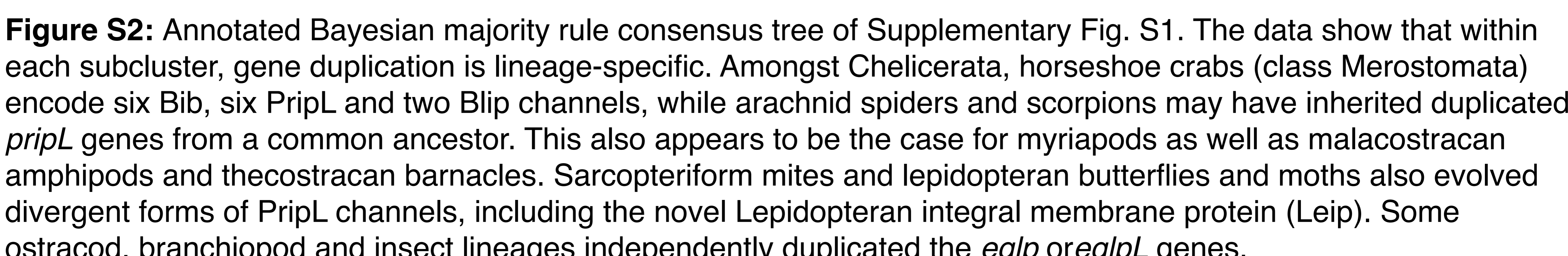

Supplement: Supplementary file 3 — Additional file 3: Fig. S2. Annotated Bayesian majority rule consensus tree including accession numbers of Additional file 23: Fig. S1. The data show that within each subcluster, gene duplication is lineage-specific. Amongst Chelicerata, horseshoe crabs (class Merostomata) encode six Bib, six PripL, and two Blip channels, while arachnid spiders and scorpions may have inherited duplicated pripL genes from a common ancestor. This also appears to be the case for myriapods as well as malacostracan amphipods and thecostracan barnacles. Conversely, sarcopteriform mites and lepidopteran butterflies and moths evolved divergent forms of PripL channels, including the novel lLepidopteran integral membrane protein (Leip). Some ostracod, branchiopod, and insect lineages also independently duplicated the eglp/eglpL genes. [file 12915_2025_2262_MOESM3_ESM.pdf]

Fig. 2B

EgIpL

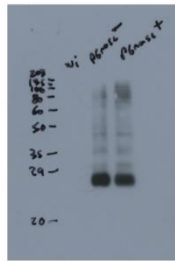

Fig. 2H

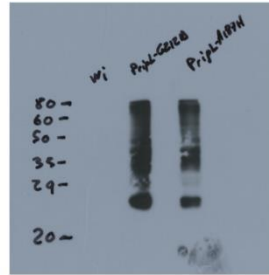

Fig. 3B

Glp2\_v1

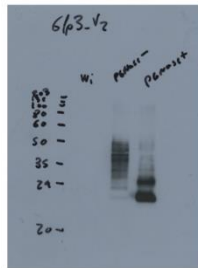

Glp2\_v2

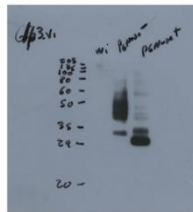

Glp4\_v1

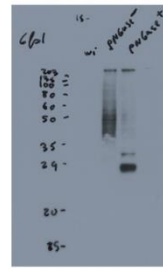

Glp4\_v2

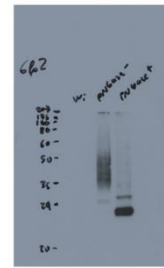

**Figure S6.** Uncropped immunoblots from Figures 2 and 3.

Supplement: Supplementary file 13 — Additional file 13: Fig. S6. Uncropped immunoblots from Figs. 2 and 3. [file 12915_2025_2262_MOESM13_ESM.pdf]
