# Supplementary material for: Co-opted and canonical glycerol channels play a major role during anhydrobiosis of an extremophile crustacean
Source: BMC Biol. 2025 Jun 3;23:151. doi: 10.1186/s12915-025-02262-3 (PMC12135271; doi:10.1186/s12915-025-02262-3)
Supplement: Supplementary file 4 — Additional file 4: Dataset S1. Codon alignment for Fig. 1. [file 12915_2025_2262_MOESM4_ESM.pdf]

>EFX82861_Daphnia_pulex_Bib
---------------------ATGGCGACAATGAGTGTGGCCACGACTCCACTG------
------ACGAGTGCCGGTCTCGAGAATCTGATGACGACATTGCTGGAACGTTTCGAGACG
GCCTGTTTGGAGCAGCAG---CAGGCGGGAAATGGCAACAAG---ATGCGGGTGCCAATG
CAGGCCGAAATCCGATCGCTGGATTTCTGGCGTTCCATTGTGGCCGAATGCCTGGCTTCC
TTTTTCCTCGTCTTCATCGTCTGCGGCTCGTTCATACCTTGGTCGGGA---------CAC
ACTCCGCCAGCCATCAGCATCGCCTTGGCTTCCGGCTTCGCTGTGGCAGCCCTCACACTC
TCCTTCGCCCAAATATCTGGAGCTCATTTGAATCCAGTCATGTCGCTGGCATTGCTCGTC
ACTCGCAAAATATCGCCCATGAAAGCTTTGCTCTACGTCACGTCTCAAGGAGGTGGAGCC
ATCGCTGGAGCTGCTCTTCTTTACGGATGCACCAGTCCTGGACATCAA---GGATACCTC
GGA---------------------------------------------------------
------------------GCTACAATCGTCTCTACCGGTTTAACCGATTGGCAAGCGCTG
GGCATTGAACTATGCCTGACGTTTGTCATCACGCTCGTCTATTTGAACCAAAAAGATGAT
CATCGACGTGGGTTATCG---GCTTCCATAGGACCTGGACCCATCACGCTCGGCCTCACC
TAC---ACGGCCTGCTCCTTTGTTGCTTTGCCATCAACTGGGGCCTCATTCAACCCTATG
AGATCACTTGGTCCGGCTTTCGTCATG---------------------------------
AAC---AAATGGGACGCTCACTGGGTTTATTGGCTGGGACCCATCGTGGGTGGCTGTCTG
GCGGCCATCCTCTAC---GAGTTCATTTTCAACCCGTATCGCAACTCG---------CAA
CGCCGTAAGGGATCCATCCCCGATGGTGACGCGTCGAGCATTCACAGCGACGAGGACAAC
TACGACGACGTGAAATCAACTGTGGCT------------AATTACCAG---------AGT
GCCCGCTCTCAGCACTACGATCAGTACCGGCCGGCAATTAGTCCAGGTGGTCCGTCTAAT
CCAATTTCTCCTAGCGGTGGTGGTGTCGGT---GGCGCTGTAGGAAACAACTGGCGGAAC
CAGCAGCGG---ATG---GAACGAGCCGAATCGGTTTATGGCGGCACCAAGTCGCTTTAC
CATTGCGGCAAATCACCTGGACCGCCCAGCCGTTCGAGCAATCTCAACCGCTCGCAGTCG
GTCTACGCCAAACCTCCGGCCGCTCCGGCTGTCCAACCTCCCAAC---GTTTACGCTTCC
CGCGGAGCTCAGCTCCAGTCGGCGCAGAGCACTTATCTGGCCGCCCGCAACGTCGACCTC
ATCCGCACCGAGAGCATTTACGCCACGCGACAGCCGCCGGCT------CCTCTG---GCG
------------------------------TCCGGAAACAGCAGCCGGGCCGAAAGCGTT
TACGGC------AGC---CGCCATCCGCGCCATATGGCCGATATGACGGAC---GTGGAT
CGACCC---GTGCGCGACAATTCGCTCCTGACGTCTTCGGTGGCTAGAGAACCCGTTTAC
GCCACCAGGGCTCCC------------------AGTCAC------GACCGTTCCAAACCG
GAATCGTACCAACTGGAACCCATTTACCAGACTCGACGTGAAGTTGTACCATCGGTGGCG
GCCGTCGAGCCG---GCCCAGCGCGGTGAAAGCCTCTACGATCGCAAAGTC---CCTACT
TATCGGCCGGAAGCGACTGTAGCCGCTTACAATCGTCGACCCAATCCGGAGCAG---ATG
GAAACATCGGCAACCAGCGCC---GGAGGAGCTCCAATTTCTCCTAGCGACACGTCAAAG
GATTCGGCCTATGGATCCGTCCACGGCACTTCGATTCAAAGTCCAGCGGAATGGTCAGGT
CACGGCCAGGAG---------------------AACAATCAGAGCAACTACAATCAACAA
CAGCAGCAGCAACAACAACAACAG---CATGTTCAACATCTCCAACATGCATCCGCCTTC
AGTCCTCATTTACCCAACCAGCCTCCACAAGGTTCTTACCAGATCCAGCAGCAGCAGCAG
CAAGCCGGTCATCATCATCACTACGCTCAGAAGTTCCGAGCGGATTTGATGTCACCCAAC
------AGCCAACAACATTCG------CCCGGATCA---------TTTCACTCACCGGTG
CAATAC
>XP_046637937_Daphnia_pulicaria_Bib
---------------------ATGGCGACAATGAGTGTGGCCACGACTCCACTG------
------ACGAGTGCCGGTCTCGAGAATCTGATGACGACATTGCTGGAACGTTTCGAGACG
GCCTGTTTGGAGCAGCAG---GAGGCGGGAAATGGCAACAAG---ATGCGGGTGCCAATG
CAGGCCGAAATCCGATCGCTAGATTTCTGGCGTTCCATTGTGGCCGAATGCCTGGCTTCC
TTTTTCCTCGTCTTCATCGTCTGCGGCTCGTTCATACCTTGGTCGGGA---------CAC

ACTCCGCCAGCCATCAGCATCGCATTGGCTTCCGGCTTCGCTGTGGCAGCCCTCACACTC
TCCTTCGCCCAAATATCTGGAGCTCATTTGAATCCAGTCATGTCGCTGGCATTGCTCGTC
ACTCGCAAAATATCGCCCATGAAAGCTTTGCTCTACGTCACGTCTCAAGGAGGTGGAGCC
ATCGCTGGAGCTGCTCTTCTTTACGGTGTAACCAGTCCTGGACATCAA---GGATACCTC
GGA---------------------------------------------------------
------------------GCTACAATCGTCTCTACCGGTTTAACCGATTGGCAAGCGCTG
GGCATTGAACTATGCCTGACGTTTGTCATCACGCTCGTCTATTTGACAACGATGGATGAT
CATCGACGTGGGTTATCG---GCTTCCATAGGACCTGGACCCATCACGCTCGGCCTTACC
TAC---ACGGCCTGCTCCTTTGTCGCTTTGCCATCAACTGGTGCCTCATTCAACCCTATG
AGATCACTTGGTCCGGCTTTCGTCATG---------------------------------
AAC---AAATGGGACGCTCACTGGGTTTATTGGCTGGGACCCATCGTGGGTGGCTGTCTG
GCGGCCATCCTCTAC---GAGTTCATTTTCAACCCGTATCGCAACTCG---------CAA
CGCCGCAAGGGATCCATCCCCGATGGTGACGCGTCGAGCATTCACAGCGACGAGGACAAC
TACGACGACGTGAAATCAACTGTGGCT------------AATTACCAG---------AGT
GCCCGCTCTCAGCACTACGATCAGTACCGGCCGGCAATTAGTCCAGGTGGTCCGTCTAAT
CCAATTTCACCTAGCGGTGCTGGTGTCGGT---GGCGCTGTAGGAAACAACTGGCGGAAC
CAGCAGCGG---ATG---GAACGAGCCGAATCGGTTTATGGCGGCACCAAGTCGCTTTAT
CATTGCGGCAAATCACCTGGACCGCCCAGCCGTTCGAGCAATCTCAACCGCTCCCAGTCG
GTCTACGCCAAACCTCCGGCCGCTCCGGCTGTCCAACCTCCCAAC---GTTTACGCTTCC
CGCGGAGCTCAGCTCCAGTCGGCGCAGAGCACTTATCTGGCCGCTCGCAACGTCGACCTC
ATTCGCACCGAGAGCATTTACGCCACGCGACAGCCGCCGGCT------CCTATG---GCG
------------------------------TCCGGAAACAGCAGCCGGGCCGAAAGCGTT
TACGGC------AGC---CGCCATCCGCGCCATATGGCCGATATGACGGAC---GTGGAT
CGACCC---GTGCGCGACAATTCGCTCCTGACGTCTTCGGTGGCTAGAGAACCCGTTTAC
GCCACCAGGGCTCCC------------------AGTCAC------GACCGTTCCAAACCG
GAATCGTACCAACTGGAACCCATTTACCAGACTCGACGTGAAGTTGTACCATCGGTGGCG
GCCGTCGAGCCG---GCCCAGCGCGGTGAAAGCCTCTACGATCGCAAAGTC---CCTACT
TATCGGCCGGAAGCGACTGTGGCCGCTTACAACCGTCGACCCAATCCGGAGCAG---ATG
GAAACATCGGCAACCAGCGCC---GGAGGAGCTCCCATTTCTCCTAGCGACACGTCAAAA
GATTCTGCCTATGGATCCGTCCACGGCACTTCGATTCAAAGTCCAGCGGAATGGGCAGGT
CACGGCCAGGAG---------------------AACAATCAGAGCAACTACAATCAACAA
CAGCAGCAACAACAACAACAG------CATGTTCAACATCTCCAACATGCATCCGCCTTT
AGTCCTCATTTACCCAACCAGCCTCCGCAAGGTTCTTACCAGATCCAGCAACAGCAGCAG
CAAGCCGGTCATCATCATCACTACGCTCAGAAGTTCCGAGCGGATTTGATGTCACCCAAC
------AGCCAACAACATTCG------CCCGGATCA---------TTTCACTCACCGGTG
CAATAC
>JAACYE010000001_Daphnia_obtusa_Bib
---------------------ATGGCGACAATGAGTGTGGCCACGACTCCACTG------
------ACGAGTGCCGGTCTCGAGAATCTGATGACGACATTGCTGGAACGTTTCGAGACG
GCCTGCTTGGAGCAGCAG---GAGGCGGGAAATGGCAACAAG---ATGCGGGTGCCAATG
CAGGCCGAAATCCGATCGCTGGATTTCTGGCGTTCCATTGTGGCCGAATGCCTGGCTTCC
TTTTTCCTCATCTTCATCATCTGCGGCTCGTTCATACCTTGGTCGGGA---------CAC
GCTCCGCCAGCCATCAGCATCGCCTTGGCTTCCGGCTTCGCCGTGGCAGCCCTCACACTC
TCCTTCGCCCAAATATCTGGAGCTCATTTGAATCCAGTCATGTCGCTGGCGTTGCTGGTC
ACTCGCAAAATCTCGCCCATGAAAGCTTTGCTCTACGTCACGTCTCAAGGAGGTGGAGCC
ATCGCTGGGGCTGCTCTTCTTTACGGGGTATCCAGTCCTGGACATCAA---GGATACCTC
GGA---------------------------------------------------------
------------------GCTACGATCGTCTCTACCGGTTTATCCGATTGGCAAGCGCTG

GGCATTGAACTATGCCTGACGTTTGTCATCACGCTCGTCTATTTGGTAACGATGGATGAT
CATCGACGTGGGTTATCG---GCTTCCATAGGACCTGGACCCATCACGCTCGGCCTCACC
TAC---ACGGCCTGTTCCTTTGTCGCTTTGCCATCAACTGGTGCCTCATTCAACCCTATG
AGATCACTTGGTCCGGCTTTCGTCATG---------------------------------
AAC---AAATGGGGCGCTCACTGGGTTTATTGGCTGGGGCCCATCGTGGGTGGCTGTCTG
GCGGCCATCCTGTAC---GAGTTCATTTTCAACCCGTATCGCAACTCG---------CAA
CGCCGTAAGGGATCCATCCCCGATGGTGACGCGTCGAGCATTCACAGCGACGAGGACAAC
TACGACGACGTGAAATCAACTGTGGCT------------AATTACCAG---------AGT
GCCCGCTCTCAGCACTACGATCAGTACCGGCCGGCAATTAGTCCAGGTGGTCCGTCTAAT
CCAATTTCTCCTAGCGGTGGTGGTGGCGGT---GGCGCTGTAGGAAACAACTGGCGGAAC
CAGCAGCGG---ATG---GAACGAGCCGAATCGGTTTATGGCGGCACCAAGTCGCTTTAC
CATTGCGGCAAATCACCTGGGCCGCCCAGCCGCTCGAGCAATCTCAACCGCTCTCAGTCG
GTCTACGCCAAACCTCCAGCCGCTCCGGCTGTCCAACCTCCGAAC---GTTTACGCTTCC
CGCGGAGCTCAGCTCCAGTCGGCGCAGAGCACTTATCTGGCCGCTCGCAACGTCGACCTC
ATTCGCACCGAGAGCATTTACGCCACGCGACAACCGCCGGCT------CCCCTG---GCG
------------------------------TCCGGAAACAGCAGCCGGGCCGAAAGCGTT
TACGGC------AGC---CGCCATCCGCGCCATATGGCCGATATGACGGAC---GTGGAT
CGACCC---GTGCGCGACAATTCGCTCCTGACGTCCTCGGTGGCTAGGGAACCCGTTTAC
GCCACCAGGGCTCCC------------------AGTCAC------GACCGTTCCAAACCG
GAATCGTACCAACTGGAGCCCATTTACCAGACTCGACGTGAAGTTGTACCGTCGGTGACG
GCCGTGGAGCCG---GCCCAGCGCGGTGAAAGCCTCTACGATCGCAAAGTC---CCTACT
TATCGGCCGGAAGCGACTGTAGCCGCTTACAACCGTCGACCCAATCCGGAGCAG---ATG
GAAACATCGGCAACCAGCGCT---GGAGGAGCTCCAGTTTCTCCTAGCGACACGTCAAAG
GATTCGGCCTATGGATCCGTCCACGGCACTTCGATTCAAAGTCCAGCGGAATGGACAGGT
CACGGCCAGGAG---------------------AACAATCAGAGCAACTACAATCAACAA
CAGCAGCAGCAACAACAACAACAG---CATGTTCAACATCTCCAACATGCTTCCGCCTTC
AGTCCTCATTTACCCAACCAGCCTCCACAAGGTTCTTACCAGGTCCAGCAGCAGCAGCAA
---GCCGGCCATCATCATCACTACGCTCAGAAGTTCCGAGCGGATTTGATGTCGCCCAAC
------AGCCAACAACATTCG------CCCGGATCA---------TTTCACTCACCGGTG
CAATAC
>JAAVJA010004589_Daphnia_dubai_Bib
---------------------ATGACGACGATGAGTGTAGCGACGACTCCACTA------
------ACGAGTGCTGGTCTCGAGAATTTGATGACGACATTACTGGAACGTTTCGAGACA
GCCTGCTTGGAGCAGCAG------GCGGCCAATGGCAACAAG---ATGCGGGTGCCGATG
CAGGCTGAAATCCGATCATTGGATTTCTGGCGTTCCATTGTGGCCGAATGCTTGGCTTCC
TTTTTTCTTGTCTTCATCATCTGCGGCTCGTTCATACCTTGGTCGGGA---------CAC
ACTCCGCCAGCCATCAGCATCGCCTTGGCTTCCGGCTTTTCCATTGCAGCTCTCACACTC
TCCTTCGCCCAAATATCTGGAGCTCATTTGAATCCAGTCATGTCATTGGCTTTGCTGGTC
ACTCGCAAAATATCGCCCATGAAAGCTCTGCTCTACGTCACGTCTCAAGGAGGTGGAGCC
ATCGCCGGCGCTGCTCTTCTTTACGGGGTAACCAGTCCTGGACATCAA---GGATACCTT
GGA---------------------------------------------------------
------------------GCTACAATCGTCTCTACCGGTTTATCCGATTGGCAAGCGCTG
GGCATTGAACTGTGCCTGACATTTGTCATCACGCTCGTCTATTTGGTA------------
----------------------------ATCGGACCGGGGCCCATCACGCTCGGCCTCACC
TAC---ACGGCCTGTTCCTTTGTCGCTTTGCCATCGACTGGTGCCTCATTCAATCCTATG
AGATCACTTGGACCNGCTTTCGTCATG---------------------------------
AAC---AAATGGGACGCTCACTGGGTTTATTGGCTCGGACCCATCGTGGGTGGCTGTCTG
GCGGCCATCCTCTAC---GAGTTCATTTTCAACCCGTATCGCAACTCT---------CAG

CGCCGTAAGGGATCCATCCCCGATGGAGACGCGTCCAGCATTCACAGCGACGAGGACAAC
TACGACGACGTGAAATCGACTGTGGCT------------AATTACCAG---------AGT
GCCCGCTCTCAGCATTACGATCAGTACCGACCGGCCATTAGTCCAGGTGCTCCGTCCAAT
CCCATTTCTCCTAGCGGTGCAGGTGGTGGC------GCTGTAGGGAACAACTGGCGGAAC
CAGCAGCGG---ATG---GAACGAGCCGAATCGGTTTATGGTGGCACGAAATCGCTTTAC
CATTGCGGCAAATCTCCTGGACCTCCTAGCCGCTCCAGTAATCTCAACCGCTCGCAATCG
GTTTACGCTAAACCTCCAACCACTCCGGCTGTCCAACCTCCCAAT---GTTTACGCTTCT
CGCGGAGCTCAACTCCAGTCTGCCCAGAGCACTTATCTCGCCGCTCGCAACGTCGACCTC
ATTCGCACCGAGAGCATTTACGCCACACGACAACCACCGGCT------CCTTTG---GCG
------------------------------TCCGGAAACAGTAGCAGAGCCGAGAGCGTT
TACGGC------AGC---CGCCATCCGCGCCATATGTCTGATATGACGGAT---GTGGAT
CGACCT---GTACGGGACAATTCGCTTCTAACGTCTTCGGTGGCTAGAGAACCCGTTTAC
GCCACCAGGGCTCCC------------------AGTCAC------GACCGTTCGAAACCC
GAGTCGTATCAATTGGAGCCAATTTACCAGACTCGACGTGAAGTTGTACCTTCGGTTGCG
GCCGTTGAGCCG---GCCCAGCGCGGTGAAAGCCTCTACGATCGTAAAGTA---CCGACT
TATCGGCCGGAAGCGACTGTAGCCGCTTAC------------------------------
---------------------------GGAGCTCCTGTTTCTCCTAGTGACACATCAAAG
GATTCGGCCTACGGATCCGTTCATGGCACTTCGATACAAAGTCCGGCTGAATGGCAAGTT
CACGGCCAGGAG---------------------AACAATCAGAGCAACTACAATCAACAG
CAACAACAACAGCAA------------------------------CATGCTTCCGCCTTC
AGTCCTCATCTACCCAATCAGCCTCCTCAAGGTTCTTACCCGATCCAGCAGCAACAA---
------GGTCATCACCATCATTACGCTCAGAAGTTCCGAGCGGATTTGATGTCGCCCAAT
------AGCCAACAACATTCG------CCCGGATCA---------TTTCACTCACCGGTG
CAATAC
>CAH0109652_Daphnia_galeata_Bib
---------------------ATGACGACAATGAGTGTAGCTACGACTCCATTA------
------ACGAGTGCCGGTCTTGAGAATTTGATGACGACATTATTGGAACGTTTCGAGACT
GCCTGCTTGGAGCAGCAA------GCGGGAAATGGCAACAAG---ATGCGGGTACCGATG
CAAGCTGAAATTCGATCATTGGATTTCTGGCGTTCCATTGTGGCCGAATGCCTGGCTTCG
TTTTTTCTCGTCTTCATCATCTGTGGCTCGTTCATACCTTGGTCGGGA---------CAC
ACTCCGCCAGCCATCAGCATCGCATTGGCTTCCGGCTTTTCTGTAGCAGCTCTCACACTC
TCGTTTGCCCAAATATCTGGAGCTCATTTGAATCCAGTCATGTCATTGGCTTTACTGGTC
ACTCGCAAAATATCGCCCATGAAAGCTTTGCTCTACGTCACGTCTCAAGGAGGTGGAGCC
ATTGCTGGCGCTGCTCTTCTTTACGGTGTAACCAGTCCTGGACATCAA---GGATATCTT
GGA---------------------------------------------------------
------------------GCTACAATAGTTTCTACCGGTTTATCCGATTGGCAAGCGCTG
GGCATTGAACTGTGTCTCACATTTGTCATCACGCTCGTCTATTTGACAACGATGGATGAT
CATCGACGCGGGTTATCG---GCTTCCATCGGACCGGGGCCCATCACGCTCGGCCTCACC
TAC---ACGGCCTGTTCCTTTGTCGCTTTGCCATCGACTGGTGCCTCATTTAATCCTATG
AGATCACTTGGACCGGCTTTCGTCATG---------------------------------
AAC---AAATGGGACGCTCACTGGGTTTATTGGCTCGGACCCATCGTGGGTGGCTGTCTG
GCGGCTATTCTCTAC---GAGTTCATTTTCAACCCGTATCGCAACTCG---------CAG
CGCCGCAAGGGATCTATCCCCGATGGAGACGCGTCCAGCATTCACAGCGACGAGGACAAC
TACGACGACGTGAAATCAACGGTAGCT------------AATTACCAG---------AGT
GCTCGCTCTCAGCATTACGATCAGTACCGACCGGCCATTAGTCCAGGTGCTCCGTCCAAT
CCAATTTCTCCTAGTGGTGGTGGTGGCGGCGGCGGCGCTGTAGGAAACAACTGGCGGAAC
CAGCAGCGG---ATG---GAACGAGCCGAATCGGTTTATGGCGGCACAAAATCACTTTAC
CATTGCGGCAAATCACCTGGACCTCCTAGCCGTTCCAGCAATCTCAATCGTTCACAATCC

GTCTACGCTAAACCTCCGGCTACTCCGGCTGTCCAACCGCCCAAT---GTTTATGCTTCT
CGTGGAGCTCAACTTCAATCTGCCCAGAGCACTTATCTGGCCGCTCGCAATGTTGACCTT
ATTCGTACTGAAAGCATTTACGCTACACGACAACCGCCGGTC------CCTTTG---GCG
------------------------------TCCGGAAACAGTAGCAGAGCCGAAAGCGTT
TACGGC------AGT---CGCCATCCGCGACATATGGCCGATATGACGGAT---GTCGAT
CGACCTCCTGTACGCGACAATTCTCTTCTAACGTCTTCGATCGCTAGAGAACCCGTTTAC
GCTACCAGGGCTCCC------------------AGTCAT------GATCGTTCCAAACCC
GAATCCTACCAATTGGAGCCGATTTATCAGACTCGACGTGAAGTTGCACCTTCTGTTGCG
GCCGTAGAGCCCGTGGCCCAACGTGGTGAAAGTCTCTACGATCGTAAAGTC---CCAACT
TATCGGCCGGAAGCGACTGTAGCCGCTTATAATCGTCGACCCAATCAGGAGCAG---ATG
GAAACATCGGCCACCAGTGCT---GGAGGAGCTCCCGTTTCTCCTAGCGACACTTCAAAG
GATTCAGCTTACGGATCTGTTCATGGCACTTCGATTCAAAGTCCAGCGGAATGGCAAGTT
CACGGCCAGGAG---------------------AACAATCAAAGCAATTACAATCAACAA
CAGCAGCAGCAACAACAACAACAACAGCATGTACAACATCTCCAACATGCTTCCGCCTTT
AGTCCCCATCTACCCAACCAACCTCCTCAAGGTTCTTACCCGATCCAGCAGCAACAGCAA
---GCTGGGCATCATCATCACTACGCTCAGAAATTCCGAGCGGATTTGATGTCGCCCAAC
------AGCCAACAACATTCG------CCCGGATCA---------TTTCACTCACCGGTG
CAATAC
>CM023192_Daphnia_carinata_Bib
---------------------ATGACT------AGTGTGGCGACGACACCGTTG------
------ACGAGTGCCGGTCTTGAGAATTTGATGACGACATTGTTGGAACGTTTCGAGACG
GCACGTTTGGAGGAGGAA------GCGGGAAATGGCAACAAG---ATGCGGGTACCTATG
CAGGCTGAAATTCGATCGCTAGATTTCTGGCGTTCCATTGTGGCCGAATGCCTGGCTTCC
TTTTTTCTCGTCTTCATCATCTGCGGCTCGTTCATTCCATGGTCGGGG---------CAC
ACGCCACCCGCCATCAGCATCGCCTTGGCTTCCGGCTTCTCTGTGGCAGCACTGACACTC
TCCTTCGCCCAAATATCTGGAGCTCATTTGAATCCAGTCATGTCATTGGCTTTGCTGGTC
ACTCGCAAAATATCACCCATGAAAGCTTTGCTCTACGTCACGTCTCAAGGCGGTGGAGCT
ATTGCAGGAGCCGCCCTCCTTTACGGGGTAACCAGTCCTGGACATCAA---GGATACCTT
GGA---------------------------------------------------------
------------------GCCACCATCGTCTCTACCGGTTTATCCGATTGGCAAGCCCTG
GGCATTGAACTGTGCTTGACATTTGTCATCACGCTCGTCTACTTGACAACGATGGATGAT
CATCGCCGCGGTTTATCG---GCCTCCATTGGACCCGGCCCACTCACGCTCGGACTCACT
TAC---ACGGCCTGCTCGTTTGTCGCTTTACCGTCAACTGGAGCCTCGTTCAACCCCATG
AGATCGCTTGGCCCTGCTTTTGTAATG---------------------------------
AAT---AAATGGGACGCGCATTGGGTTTATTGGCTGGGGCCCATCGTGGGCGGCTGTCTG
GCCGCCATCCTGTAC---GAGTTCATTTTCAACCCGTATCGCAACTCG---------CAA
CGCCGTAAGGGATCCATTCCGGACGGTGATGCGTCCAGCATTCACAGTGACGAGGACAAC
TACGACGATGTGAAATCAACCGTGGCC------------AATTACCAG---------AGT
GCCCGCTCTCAGCATTACGATCAGTACCGACCTGCAATTAGTCCAGGTGGCCCGTCCAAT
CCGATTTCACCTAGCGGTGGTGGTGGCGGTAGTGGCGCTGTGGGAAACAACTGGCGCAAT
CAGCAGCGC---ATG---GAACGAGCCGAATCGGTTTATGGCGGTACGAAATCGCTTTAC
CATTGCGGCAAGTCACCTGGCCCGCCCAGCCGTTCCAGCAATCTCAATCGCTCGCAATCC
GTCTACGCTAAACCCCCGGCCGGACCGGCCGTTCAGCCGCCCAAC---GTTTACGCTTCG
CGCGGAGCTCAACTGCAGTCGGCGCAGAGCACTTACCTGGCCGCCCGTAATGTCGACCTC
ATTCGCACGGAAAGCATTTACGCCACTCGACAACCTCCGCAACAACCTCCTCTCCCGACG
------------------------------TCCGGCAATAGCAGCAGAGCCGAAAGCGTT
TACGGC------AGC---CGCCATCCGCGCCACATGGCCGACATGACGGAC---GTGGAT
CGACCT---GTACGAGACAATTCTTTGCTGACGTCGTCCATGGCAAGGGAACCTGTCTAC

GCTACACGAACTAATCAGAGT---------TCGGGTCAT------GATCGTTCCAAACCG
GAATCGTACCAACTGGAGCCCATTTACCAGACTAGACGTGAAGTTGTGCCTTCCGTGGCC
ACTGTGGAGCCC---GCCCAGCGCGGTGAAAGCCTTTACGATCGTAAAGTG---CCTACT
TACCGGCCGGAAGCGACTGTGGCCGCGTATAATCGCCGACCCAATCAGGAGCAG---ATG
GAGACATCCGGGACCAGCACT------GGAGCCCCTATTTCTCCCAGTGACACGTCCAAG
GATTCGGCATATGGATCTGTTCATGGCAATTCAGCTCAAAGTCCATCCGAATGGCCG---
---GCTCAGGAG---------------------AATAATCAAAGCAATTACAACCAACAG
CAGCAGCCACAG---------------CACGTGCCACATCTGCAACATGCATCCGCTTTC
AGTCCTCATCTCCCTAATTCACCCAATCAAGGACCTTACCAGATGCAACAACAACAA---
---GCTGGACACCACCATCACTACGCTCAGAAATTCCGAGCGGATTTGATATCGCCCGGT
---GGTCAACAACAGCATTCG------CCCGGATCG---------TTTCACTCACCGGTG
CAATAT
>KAI9558856_Daphnia_sinensis_Bib
---------------------ATGACT------AGTGTGGCGACGACACCGTTG------
------ACGAGTGCCGGTCTTGAGAATTTGATGACGACATTGTTGGAACGTTTCGAGACG
GCACGTTTGGAGGAGGAA------GCGGGAAATGGCAACAAG---ATGCGGGTACCTATG
CAGGCTGAAATTCGATCGCTAGATTTCTGGCGTTCCATTGTGGCCGAATGCCTGGCTTCC
TTTTTTCTCGTCTTCATCATCTGCGGCTCGTTCATTCCATGGTCGGGG---------CAC
ACGCCACCCGCCATCAGCATCGCCTTGGCTTCCGGCTTCTCTGTGGCAGCACTGACACTC
TCCTTCGCCCAAATATCTGGAGCTCATTTGAATCCAGTCATGTCATTGGCTTTGCTGGTC
ACTCGCAAAATATCACCCATGAAAGCTTTGCTCTACGTCACGTCTCAAGGCGGTGGAGCT
ATTGCAGGAGCCGCCCTCCTTTACGGAGTAACCAGTCCTGGACATCAA---GGATACCTT
GGA---------------------------------------------------------
------------------GCCACCATCGTCTCTACCGGTTTATCCGATTGGCAAGCCCTG
GGCATTGAACTGTGCTTGACATTTGTCATCACGCTCGTCTACTTGACAACGATGGATGAT
CATCGCCGCGGTTTATCG---GCCTCCATTGGACCCGGCCCACTCACGCTCGGACTCACT
TAC---ACGGCCTGCTCGTTTGTCGCTTTACCGTCAACTGGAGCCTCGTTCAACCCCATG
AGATCGCTTGGCCCTGCTTTTGTAATG---------------------------------
AAT---AAATGGGACGCGCATTGGGTTTATTGGCTGGGGCCCATCGTGGGCGGCTGTCTG
GCCGCCATCCTGTAC---GAGTTCATTTTCAACCCGTATCGCAACTCG---------CAA
CGCCGTAAGGGATCCATTCCGGACGGTGATGCGTCCAGCATTCACAGTGACGAGGACAAC
TACGACGATGTGAAATCAACCGTGGCC------------AATTACCAG---------AGT
GCCCGCTCTCAGCATTACGATCAGTACCGACCTGCAATTAGTCCAGGTGGCCCGTCCAAT
CCGATTTCACCTAGCGGTGGTGGCGGT---AGTGGCGCTGTGGGAAACAACTGGCGCAAT
CAGCAGCGC---ATG---GAACGAGCCGAATCGGTTTATGGCGGTACGAAATCGCTTTAC
CATTGCGGCAAGTCACCTGGCCCGCCCAGCCGTTCCAGCAATCTCAATCGCTCGCAATCC
GTCTACGCTAAACCCCCGGCCGGACCGGCCGTTCAGCCGCCCAAC---GTTTACGCTTCG
CGCGGAGCTCAACTGCAGTCGGCGCAGAGCACTTACCTGGCCGCCCGTAATGTCGACCTC
ATTCGCACGGAAAGCATTTACGCCACTCGACAACCTCCGCAACAACCTCCTCTCCCGACG
------------------------------TCCGGCAATAGCAGCAGAGCCGAAAGCGTT
TACGGC------AGC---CGCCATCCGCGCCACATGGCCGACATGACGGAC---GTGGAT
CGACCT---GTACGAGACAATTCTTTGCTGACGTCGTCCATGGCAAGGGAACCTGTCTAC
GCTACACGAACTAATCAGAGT---------TCGGGTCAT------GATCGTTCCAAACCG
GAATCGTACCAACTGGAGCCCATTTACCAGACTAGACGTGAAGTTGTGCCTTCCGTGGCC
ACTGTGGAGCCC---GCCCAGCGCGGTGAAAGCCTTTACGATCGTAAAGTG---CCTACT
TACCGGCCGGAAGCGACTGTGGCCGCGTATAATCGCCGACCCAATCAGGAGCAG---ATG
GAGACATCCGGGACCAGCACT------GGAGCCCCTATTTCTCCCAGTGACACGTCCAAG
GATTCGGCATATGGATCTGTTCATGGCAATTCAGCTCAAAGTCCATCCGAATGGCCG---

---GCTCAGGAG---------------------AATAATCAAAGCAATTACAACCAACAG
CAGCAGCCACAG---------------CACGTGCCACATCTGCAACATGCATCCGCTTTC
AGTCCTCATCTCCCTAATTCACCCAATCAAGGACCTTACCAGATGCAACAACAACAA---
---GCTGGACACCACCATCACTACGCTCAGAAATTCCGAGCGGATTTGATATCGCCCGGT
---GGTCAACAACAGCATTCG------CCCGGATCG---------TTTCACTCACCGGTG
CAATAT
>XM_032927904_Daphnia_magna_Bib
---------------------ATGACT------AGTGTGGCGACGACACCGTTG------
------ACGAGTGCCGGTCTTGAGAATTTGATGACGACATTACTCGAACGTTTTGAGACG
GCCCGTTTGGAGGAGGAA------GCGGGAAATGGCAACAAG---ATGCGGGTACCTATG
CAGGCTGAAATTCGATCGCTAGATTTCTGGCGTTCCATTGTGGCCGAATGCCTGGCTTCC
TTTTTTCTCGTCTTCATCATCTGCGGCTCGTTCATTCCATGGTCGGGG---------CAC
ACGCCACCCGCCATCAGCATCGCCTTGGCTTCCGGCTTCTCTGTGGCAGCACTCACCCTC
TCCTTCGCCCAAATATCTGGAGCTCATTTGAATCCAGTTATGTCATTGGCTTTGCTGGTC
ACTCGCAAAATATCACCCATGAAAGCTTTGCTCTACGTCACGTCACAAGGCGGTGGAGCT
ATTGCAGGAGCCGCTCTTCTTTACGGAGTAACCAGTCCTGGACATCAA---GGATACCTT
GGA---------------------------------------------------------
------------------GCTACCATCGTCTCTACCGGTTTATCTGATTGGCAAGCCCTG
GGCATTGAACTGTGCTTGACATTTGTCATCACGCTCGTCTATTTGACAACGATGGATGAT
CATCGTCGCGGTTTGTCG---GCCTCCATTGGACCCGGCCCACTCACGCTCGGCCTCACT
TAC---ACGGCCTGTTCGTTTGTCGCTCTGCCATCGACCGGAGCCTCGTTCAACCCTATG
AGATCGCTTGGTCCGGCTTTCGTAATG---------------------------------
AAT---AAATGGGACGCGCATTGGGTTTATTGGCTGGGTCCCATCGTGGGCGGCTGTCTG
GCCGCCATCCTGTAC---GAGTTCATTTTCAACCCGTATCGCAACTCG---------CAA
CGCCGTAAGGGATCCATTCCGGACGGTGATGCGTCCAGCATTCACAGTGACGAGGACAAC
TACGACGATGTGAAATCAACCGTGGCC------------AATTACCAG---------AGT
GCCCGCTCTCAGCATTACGATCAGTACCGACCGGCAATTAGTCCAGGTGGCCCGTCCAAT
CCGATTTCTCCTAGCGGTGGTGGCGGTGGT---GGCGCTGTGGGAAACAACTGGCGCAAT
CAGCAGCGC---ATG---GAACGAGCCGAATCGGTTTATGGCGGTACGAAATCACTTTAC
CATTGCGGCAAGTCACCTGGCCCACCCAGCCGCTCGAGCAATCTCAATCGGTCGCAATCC
GTCTACGCTAAACCTCCAGCCGGACCGGCCGTTCAGCCGCCCAAC---GTTTACGCTTCG
CGTGGAGCTCAACTGCAGTCGGCGCAGAGCACTTACATTGCCGCTCGTAATGTCGACCTC
ATTCGCACGGAAAGCATTTACGCCACTCGACAACCTCCGCAACAACCTCCT---CCGGCG
------------------------------TCCGGTAATAGCAGCAGAGCCGAAAGCGTT
TACGGC------AGT---CGCCATCCGCGTCACATGGCTGACATGACGGAT---GTGGAT
CGACCT---GTACGAGACAATTCTTTGCTGACGTCGTCCATCGCAAGGGAACCTGTCTAC
GCCACACGAACT---CAAAGT---------TCGGGTCAC------GATCGTTCCAAACCG
GAATCGTACCAACTGGAACCTATTTACCAGACTAGACGTGAAGTTGTGCCTTCCGTGGCC
ACTGTCGAGCCC---GCCCAGCGCGGTGAAAGCCTTTACGATCGTAAAGTC---CCTACT
TACCGGCCGGAAGCGACTGTGGCTGCGTATAACCGTCGCCCCAATCAGGAGCAG---ATG
GAGACATCCGGGACCAGCACT------GGAGCGCCTATTTCGCCCAGTGACACGTCCAAG
GATTCGGCTTATGGATCTGTTCATGGCAATTCGATACAAAGTCCATCCGAATGGCCG---
---GCGCAGGAG---------------------AATAATCAAAGCAATTATAACCAACAG
CAGCAACAACAA---------------CACGTGCCACATCTGCAACATGCTTCCGCCTTC
AGTCCTCATCTCCCTAATCCACCTAATCAAGGACCTTACCAGATGCAACAACAACAA---
---GCTGGACACCACCATCACTACGCTCAGAAATTCCGAGCGGATTTGATATCGCCCAGT
---GGTCAACAACAACATTCG------CCCGGATCG---------TTTCACTCACCGGTG
CAATAT

>JAKUCG010000182_Ceriodaphnia_dubia_Bib
---------------------ATGACGACGATGAGTGTTGCCACGACACCGTTG------
------ACCAGTGCCGGTCTTGAGAACATGATGACGACATTGCTGGAGCGATTCGAGACG
GTGAGATTGGAGCAG---------GCGGGAAATAGCAACAGG---ATGCGGGTGCCGATG
CAAGCTGAAATTCGATCGCTCGATTTCTGGCGTTCGATCGTGGCTGAATGCTTGGCATCT
TTCTTCCTCGTCTTCATTATTTGCGGATCGTTTATCCCCTGGTCGGGG---------CAC
ACGCCACCAGCCATCAGCATCGCCCTGGCTTCCGGCTTTTCCATCGCAGCTCTCACCCTG
TCTTTCGCCCAAATATCAGGAGCTCATTTGAATCCAGTCATGTCTCTGGCTTTGTTGGTC
ACTCGCAAAATATCGCCGATGAAGGCTCTGCTCTACGTCACATCGCAAGGAGGTGGTGCC
ATCGCTGGAGCGGCTCTCCTTTATGGGGTGACCAGTCCTGGACATCAA---GGATACCTC
GGA---------------------------------------------------------
------------------GCTACGATCGTCTCCAACGGCCTTTCCGATTGGCAAGCCTTG
GGCATCGAACTGTGTCTGACATTTGTCATTACTCTTGTTTATTTGACAACGATGGATGAT
CACCGAAGAGGTTTATCG---GCTTCGATCGGGCCGGGTCCCCTTACGCTCGGACTAACT
TAC---ACGGCCTGTTCTTTTGTCGCTTTGCCGTCGACCGGAGGTTCTTTCAATCCAATG
CGGTCGCTCGGACCGGCTTTCGTCATG---------------------------------
AAC---AAATGGGACGCGCATTGGGTTTATTGGCTTGGACCCATCGTGGGCGGTTGTCTG
GCCGGCATCCTGTAC---GAGTTTATATTCAACCCGTATCGCAACTCG---------CAG
CGCCGCAAGGGATCCATCCCCGATGGTGATGCGTCGAGCATTCACAGCGACGAGGACAAC
TACGACGACGTCAAATCGACTGTGGCT------------AATTACCAG---------AGC
GCCCGCTCCCAGCATTACGATCAGTACCGTCCGGCTATTAGTCCAGGTGGTCCGTCCAAT
CCAATCTCGCCTAGTGCAGGTGGCGGCGGC---CCT---GTCGGTAACAACTGGAGGAAC
CAACAACGC---ATG---GAACGAGCCGAATCGGTTTACGGCGGTACAAAGTCGCTTTAT
CATTGCACCAAGTCACCTGGCCCGCCCAGTAGGTCAAGCAATCTCAACCGTTCTCAGTCG
GTGTACGCAAAACCCCCTGCCGGACCGGCTGTCCAGCCGCCCAAT---GTTTACGCCTCG
CGCGGAGCTCAAATGCAGTCGGCACAGAGCACTTATCTGGCT---CGTAATGTCGACCTC
ATTCGCACCGAAAGCATTTATGCCACTCGTCCGCCGCCC------------GTC------
------------------------------------------AACAGAGCCGAAAGCGTT
TACGGT------ACC---AGGCATCCGCGCCATATGACCGATATGACGGAC---GTAGAC
CGGCCC---GGTCGAGAAGTGTCATTGCTCACCTCA------CCTAGAGAACCGGTTTAC
TCTGCCAGAAACACT---------------GCCGGACACCAACAGGATCGTTCTAAACCC
GAGTCTTACCAGTTGGAGCCCATCTACCAGACGAGGCGCGAAGTTGTGGCCCCATCGGCC
GTTGTCGAGCCG---GCCCAGCGCGGCGAAAGCTTGTATGATCGCAAAGTC---CCGACT
TATCGTCCGGAAGCGACTGTAGCCGCGTACAACCGCCGACCCAATCAGGAACAG---ATG
GAAACATCTGCCAATAGCACT------GGAGCGCCAATTTCGCCCAGCGATACGTCCAAG
GATTCAGCTTATGGATCCGTCCATGGAAATTCGATCCAAAGCCCAGCCGAGTGGCCGCAC
AATCAGCAGGAA------------------ATGAGCAATCAGACGAATTACAGCCAGCAA
CAGCAGCAGCAGCAA------------CATATA------CAGCAGCATGCCTCAGCTTTC
GCTCCTCATCAACCCAAT---------CAGGGACCTTACCAGATCCAGCAACAGCCG---
---GGCGGA------CATCAGTACGCTCAGAAATTCCGAGCTGATCTGATATCGCCCAAT
---GGCCAACAGCAACATTCG------CCCGGATCA---------TTTCACTCACCGGTG
CAATAT
>CM057933_Chydorus_sphaericus_Bib
---------------------ATG------------------ACTTCT---CTA------
------ACCAGTGCTAGTATAGAAAATCTCTTAGCAACGTTATTAGAACGTTTCGAAACT
TTAAAATTGGACAGA---------GCTCTACCAATCAAAACA---AACATGCCGGCAGTT
CAGGCAGAAATCCGTTCTCTCGACTTTTGGCGCTCTATCGTCGCCGAATGTCTCGCATCT
TTTTTCTTGGTGTTTATCATCTGCGGCTCTTTCGTGCCTTGGTCGGGT---------CAC

ACCCCACCGGCCATTGGCATCGCCTTGGCCGCCGGATTCTCCATCTCTGCGCTCACACTT
TCCTTTGCTCACATTTCAGGTGCTCATTTGAATCCAGTCATCTCACTCGCCCTTTTAGTC
ACTCGAAAGATATCTCCGATGAAAGCGTTTTTGTACGTCACATCGCAAGGAGGTGGAGCT
ATTGCGGGTGCCGCCCTTCTCTACGGGGTGACTAGGACTGGAGATCAA---GGCAATTTA
GGA---------------------------------------------------------
------------------GCGACTATTATTTCCGCTGGATTGACCGAATGGCAAGGACTT
GGAATTGAACTCTGCCTGACATTTGTGATTGTTTTAGTCTACTTGACCACCATGGATGAT
CACCGAAGGATATTGCCG---GCCGCAATTGGACCTGGACCTCTGACCATCGGTCTTACT
TAT---ACTGCTTGCTCTTTCGTTGCTTTGCCGTCCACTGGAGCTTCTTTCAATCCTATG
AGATCCTTGGACCGGCGTTCGTCATG----------------------------------
AAT---AAATGGGATTCACACTGGGTGTACTGGCTCGGACCAATTGTAGGCGGTTGTCTA
GCCGGCATCCTCTAC---GAGTTCATTTTCAACCCGTACCGCAATTCCGCTCAA---CAG
CGCCGTAAAGAATCCGTGGCCGACGGAGACTCATCCAGTATTCATAGCGATGAGGATAAT
TACGATGACGTCAAATCCACCGTTGCCAGC---------AATTATCAA---------GCG
GCACGATCCCAACATTACGATCAGTACCGCCCGGCCATCAGTCCAGGTGGCGGCTCCAAT
CCCATCTCACCTGGTAAATTTCTC---------GGAGCAGTGGGTAACAACTGGCGTAAT
ACCCAAAGAGCTATGCTGGAACGTGCCGAATCCGTTTACGGCGGCACTAAATCCCTCTAC
CCTTGCCCAAAATCCCCTGGACCATCAAGCCGGACAGTCAACTTGAATCGATCTCAATCC
GTCTACGCTAAAGCGCCG---CCAAGCGGTCGGAATCAGCAACAA---CAAGCTCAACAG
CAGCCG---ATCCTTCAATCCGCTCAGAGTACTTACCTAACTGCACGTAATGTGGATCTC
ATCCGGACAGAGAGCATCTACGCCGCCCGTCCACCT---CAACAG---CCC---------
---------------------------------------------------------ATC
TATGGGGCT------------------AGACGGACAGCTGAG---ACGGAGATGAGGGAG
---CCA---CTGTACGGAAGGACTCTT---CAGGATCGTTCCCCG---GAATGT---TAC
CAATTG---GAACCG---------ATTTAT---GGA---ACT---AGGCGT---CATCCG
GAAACGATTATTACAGCATCTATCGAGCAGCAACAGCAGAGA------CCTCAAGCG---
---------------------------GAGAACCTTTACGGCCGG---------------
---------------------------------------------TCTGTCCAG---CAG
AATATGGAT---AATAGCTCA---------GTGCCCATCTCTCCGAGCGATACGTCCAAG
GATTCCGCTTATGGATCGGTCCACCATCATCATCCG---AATCCGCATGATTGGTCG---
------------------------------------GCCGGAAGCTCATAT---------
------------------------------------------------------------
---------------------------------------------------CAGCCG---
---GTTGGAAATGGTCACGTCTACGCCCAAAAGTTCAGGACAGATTTGATGTCGCCTACT
GGAGGGCAGCACTCTCCGTCATCTCAGCCCGGCTCTGCT------TTTCATTCTCCGGTT
CAGTAC
>GKAM01012522_Podon_leuckartii_Bib
---------------------ATGGCGACA------------GTGAGTGGCGTA------
------ACGAGCGCCAGTGTGGAAACTCTTTTGCAGACGCTGCTAGAGCGTTTCGAAACG
TTGCGGATGGACCAG---------------CCG---AAGGTT---ATGCGGGTGCCCATG
CAGGCGGAAATACGATCGCTGGATTTTTGGCGCTCGATCGTTGCAGAATGTCTTGCTTCG
TTCTTCCTAATCTTCATCATCTGCGGATCTTTCATTCCCTGGTCGGGT---------CAC
ACGCCCCCGTCCATTGGAATAGCCCTAGCAGCAGGATTTGCCGTCGCTTCGCTCACCCTC
ACTTTCGCACATGTTTCTGGGGCTCATTTGAACCCGGTGATATCATTGGCGCTGCTGGTC
ACACGCAAGATTTCGCCAATCAAAGCCTTTCTGTACGTGACGTCACAAGGAGGGGGCGCC
ATTGCCGGAGCAGCTCTTCTTTACGGTGTGACAACCAGAGGCCACCAG---GGCAATTTG
GGA---------------------------------------------------------
------------------GCGACGGTGATATCGGCCGGCCTATCGGAATTGCAGGCATTG

ACCATGGAAATGTGCCTTACCTTCGTGCTCGTTTTGGTTTACCTGACTACAATGGATGAT
AATCGACGGGCATTGCCC---GCCTCCATCGGACCCGGTCCGCTCACTTTCGGCCTCACC
TAC---GCCGCCTGCTCTTTTGTTGCTCTGCCATCGACTGGAGCGTCGTTTAACCCGATG
CGCTCGCTGGGACCGGCCTTCGTCATG---------------------------------
AAC---AAATGGGACGCTCATTGGGTCTACTGGTTGGGTCCAATCATTGGTGGCTGTTTG
GCTGGCATCTTGTAC---GAGTTCATTTTCAATCCGTATCGTAATAAC---------ATC
CACCGCAAGGGCTCCATC---GACGGAGATTCCTCGAGCATCCATAGTGACGAAGACAAT
TACGACGACGTGAAATCGACGGTAGCC------------AATTACCAC---------AGC
GCCCGTTCGCGACATTATGATCAGTACAGACCTGCCATCAGTCCCGGAGCACCGTCCAAC
CCTCTGGTGTCGCCAGCTGGAGCA---------------GCAGGAAATAATTGGCGCAAT
CAA---CGT---ATG---GAGCGTGCGGAATCGGTTTATGGTGGAACCCGATCACTTTAC
---TGC---AAATCACCCGGA---GTGACAAGA---GCCAGCCTCAACCGCTCGCAATCA
GTCTACTCAAAAGCACCG---GGTACCTCGGTAACGAACAACCCT---CTTCCGGCCACA
CGT---------TTACAATCCGCCCAGAGCACCTACCTGGCC---CGCAACGTCGACCTC
ATCCGAACGGAAAGCATTTACGCCTCGCGCCCTTCGCCG---------CCCATGCCCGCT
------------------------------------------ACAAGAGCTGACAGCGTA
TACGGCAAC---TCGGTCAAACCGTTCCGTAATCCAGGCGAAACAACCGATGCGAGCGTA
CAC---------AGCGCCGGCTCCGCCGCAAACCCGCGTTCCCCTCGTGATCCTATCTAC
GGCGCTTCT---TCC---------GACTATTCGGCTCATTCAGGCCGGTCGGCCAAACCG
GAAGCATTCCAGTTGGAACCTCTTTATGCAACGCGGCCAGATGCTGCCGCTCAACTCAAC
GCAGCT---------AACCAACGCAACGACAGTCTCTACAGCCGTAAAATGACGCCGACG
TACAGACCGGATGGACCCAAC------TACGCCATGGGTCCTCCAACAGCCCTG---CCT
CGTACAGAATTGGACAGCTCT---------GCCCCTCTCTCTCCCAGTGACACGTCCAAG
GACTCTGCTTACGGATCCGTTCATGGCACAGCAGCGCACAGTCCGTCGGAATGGACA---
---------------------------------TCGGCGCAAACCAACTACAGCAGCAAC
CACAACAACCACAGTTCTTACAATATGGCGCCCTACCACCACCACCAC------------
------CATCAACAACAACAGCAGCAGCAACAGCAGCAGCAGCAGCAATCTCAA------
------------------ATGTACGCGCAGAAATTCCGGGCGGATCTTCTATCGCCAGCT
---AATCGCCAATCGCCCACT------GCCGGATCC---------TTCCACTCGCCCGTA
CAATAT
>GKAG01003178_Evadne_nordmanni_Bib
---------------------ATGGCGACTATGAGT---GCGGTGTCGCCGCTG------
------ACGAGCGCCAGTGTTGAGACGCTGCTGTCGACGTTGCTGGAGCGTTTCGAGACG
CTGCAGATGGAGCAG---------------CCG---AAGAGG---ATGCGGGTGCCGGTG
CAGGCGGAAATTCGATCGCTGGACTTTTGGCGGTCCATCGTTGCCGAGTGTCTTGCGTCC
TTCTTCCTGACGTTCATCATCTGCGGATCGTTCATCCCCTGGTCGGGC---------CAC
ACTCCGCCTTCCATCGGCATCGCCCTGGCGGCCGGATTCGCCGTGGCTTCACTCACGCTC
ACTTTCGCCCACATTTCCGGTGCTCATTTGAATCCGGTGGTGACGCTGGCGCTGCTGGTG
ACGCGTAAGGTGTCGCCGATGAAGGCGTTTCTCTTCATCACGTCGCAGGGCGGCGGTAGC
ATCGCTGGAGCGGCTCTCCTTTACGGGGTAACGGCCAGGGGTTTCCAG---GGCAATTTG
GGA---------------------------------------------------------
------------------GCGACGGTGATATCCGGCGGTCTGTCCGAGTGGCAGGCGCTG
GCCATTGAGCTGTGCCTTACGTTCGTTCTCGTCCTGGTGTACCTGACAACGATGGATGAC
CACCGCCGAGTTCTACCC---GCCTCCATCGGACCCGGACCGCTCACGCTGGGCCTCTCC
TAC---GCCGCCTGCTCTTTTGTCGCGTTGCCTTCAACCGGAGCCTCGTTCAATCCGATG
AGATCGCTGGGACCGGCCTTCGTCATG---------------------------------
AAC---AAATGGGACGGTCATTGGGTTTATTGGTTGGGTCCCATCGTCGGCGGATGTCTG
GCCGGAATTTTATAC---GAATTCATTTTCAGCCCGTACCGCAATTGC---------CAC

CTGCGTAAAGGTTCCATC---GATGGAGATTCGTCGAGCATCCACAGCGATGAGGACAAC
TACGACGATGTGAAATCGACGGTGGCC------------AACTACCAC---------AGC
GCCCGCTCGCAGCACTACGACCAGTACCGTCCGGCCATCAGTCCGGGAGGACCATCCAAT
CCAGCTGGATCCCCCGCTGGATTGGGCGGAAGCGGAGTGGCGGGTAACAACTGGCGCAAC
CAG---CGG---ATG---GAGCGTGCGGAATCGGTGTACGGCGGCACCAAATCGCTGTAC
---TGC---AAATCGCCCGGA---GTGACGCCGCGGGCCAGTCTCAACCGCTCGCAGTCG
GTTTACTCGAAACCGCCG------TCCGTCGTCGGCAACGGCCCC---GTTCCGGCGGCG
CGG---------ATGCAATCGGCCCAGAGCACCTACCTGGCC---CGCAACGTCGATCTC
ATCCGCACGGAGAGCATTTACGCGTCCAGGCCGTCGCCG---------CCCATGCCCGCC
------------------------------------------ATTAGAACGGAAGGTGTT
TACGGAAGC---TCG---AAACCCTTCCGGTACCCGTGCGAAACCACCGACGTCAGCGTC
CAC---------AGCGCCCACAGCGCCCACAACCCGCGCTCTCCCCGCGAGCCCATCTAC
GGCGCCAGCTGCTCG---------GAATATTCCGGCCACTCGGGGCGATCCGCCAAACCG
GAATGCTTCCAGCTGGAACCCGTCTACACGGCCCGGCCGGAATCGAACGGCCCCACCAAC
CCACCACCACCACCCGCAACGACTCAGCGCGGCGGCTACGGCCGTAGGATGACACCCACC
TACCGG---------------------------ATGGGACCCCCATCCGCACTGCCTCCG
CGTCCGGAATTGGAGACGTCGAATTCGGGA---CCCATTTCACCCAGTGACACGTCAAAG
GACTCGGCTTACGGATCCGTACACGGCACCAACCCTCACAACACATCCGAATGGACATCC
GCCGGAACCCAC---------------------TCCGCT---TTCAATATCTTCCAGCAA
CGACAGCAGCAACAACAA---CCGCTCATTCAACAACATCAACAACAT------------
------CAACAACAGCAACAACAGCAACAACAGCAACAACAGCAACAACAGCAACAACAA
CAG------------CCGTTATACACCCAGAAATTCCGGGGCGATCTCTTATCGCCGGCT
---GCACGTCACTCGCCATCC------GCCGGATCG---------TTCCATTCACCCGTT
CAATAT
>GGQP01079573_Diaphanosoma_celebensis_Bib
---------------------ATGACGACCATCAGC---GTCTCCGCTCCTCTA------
------ACGACGGCCAGTGTCGAGGGTTTGATGCAAACGTTGCTCGAGAGATTCGAAACG
CTTCGAATGGATCAA---------------CCG---AAACCG---ATGCGGGTGCCGATG
CAGGCCGAAATTCGATCCCTGGACTTCTGGCGCTCCATCGTGGCTGAATGTTTAGCCACT
TTCTTCTTGATCTTCATCATCTGTGGGGCCTTCATTCCTTGGGCAGGA---------CAC
ACGCCGCCGTCCATTGGCATCGCTTTAGCCGCCGGTTTCGCCACTGCGTCGCTAACGTTG
TGCTTCTCGCACGTTTCCGGTGCTCATTTCAATCCGGTGGTAACGTTAGCCCTTTTGGTA
ACGAGAAAGGTCTCGCCGATCAAAGCTTTCCTCTTCACAACGGCTCAAGGAGGCGGCGCC
ATTGCAGGAGCTGCTCTCCTCTACGGAGTGACGGCGTCTGGCTACCAA---GGATCTCTG
GGA---------------------------------------------------------
------------------GCCACGGTGATCTCGGCCGGTTTATCCGAATGGCAGGGACTG
GGCATCGAGCTATGCCTGTCGTTCATTCTGACGTTGGTCTACTTGACCACCATGGACGAC
CACCGTCGAACGCTGCCC---GCCTCTCTCGGACCTGGACCTCTGACCTACGGGCTGGCG
TAC---GCTGCTTGTTCCTTAATGGCTCTGCCCTGCACCGGAGCTTCTTTCAATCCGATG
CGTTCATTGGGACCGGCGTTCGTTATG---------------------------------
AAC---CGATGGGACGCTCACTGGATTTACTGGTTGGGTCCCATCGTCGGTGGATGTCTA
GCAGCGGTACTTTAC---GAATTTATTTTCAATCCTTACCGCAACGCC---------CAA
CGCCGTAAGGGATCCATC---GACGGAGATTCTTCCAGTATCCACAGCGACGAAGATAAT
TACGACGATATCAAGTCGACAGTGCAC------------AACTACCAG---------AGC
GCTCGCTCGCAGCATTACGACCAGTACCGACCGGCCATTACTCCCGGAGGACCGTCCAAT
CCCGTCTCACCGAATGGAATGCCAGGAAGTAAT------------------TGGCGCAAC
------CGC---ATG---GAACGCGCTGAATCGGTTTACGGTGGCACGAAATCCCTCTAC
---TGC---AAATCACCTGGG---GTCACTCGA---GCCAGTTTGAACCGTTCGCAATCG

GTTTACGCCAAATCGCCC---GGC---GCCACGATGCCGGCCAAC---TCGTACGCTTCG
CGGCCG---AACCTACAGGCTGCACAAAGCACTTACTTGGCC---CGCAACGTCGACCTC
ATCCGCACTGAAAGTATTTATGCTTCGCGTCCGACAACGACA------CCCGTC------
------------------------------------------AATCGGAACGAGAGTCTG
TACGGCCAG---ACC---AAGAGTCAGCCTCCGATGCAACAGCATCGTCAG---AACGTT
CATCCGACGGAAATGACCAGCTCGACTCGCGAGCCGCGTTCACCGCGCGAGCCTATTTAC
GGCACCAGTGCAACGACCTCCCTGGAGTAC---GGT---AACCAACGTGGCAAAACTGCC
GAATCCTACGCCATGGAGCCCGTCTACGGAACACGTCGAGATGTCGATCCCCCCAGC---
---------------------CGCACTGAGAGTCTTTACGGGCGCAAGACG---AGCGTG
TACCGTCAAGAATCAACT---------TACGGCATGGTACCA---TCAGTCACGCATCAA
GGCCAAGAGGCAGAAAGGTCAAACAGCGGCGTCGCCATCTCACCAAGTGACACGTCCAAG
GACTCGGCTTACGGATCTGTTCATGGAAATTCCATGCAGAGCCAAAGTGACTGGACGCAG
AACGGTCAGGAGGAACAAACCAACTTCCAGACCGGCAGCAGTGCGGCTTACAATCCACCG
GCCGGTCAGCCGCCAATTCCAGGTGCACACAACCACCACCAGAGTCACAATGTTGCAGGT
CAAGGCCCTCTGTCATCCTCGTCTTCGTCGTCAGCTGGTCAAGGATCATCCACTTCGTAT
CATGCGCAGAATGCGCACATTTACGCACAGAAATTCCGGTCGGATTTGGTGTCGCCCAAA
---GGGCATCATTCGCCGTCGTACACACCGGGGACA---------TACCACTCACCCGTG
CAGTAC
>NKDA01000002_Eulimnadia_texana_Bib
---------------------ATGACAACAGCCAGT---GTGACATCGCCATTA------
------ACGACAGCCAGTCTGGAATCGCTTCTTCAGACACTTTTGGAGCGTTTGGAGAGT
TCCAAAAACGATCTG---------------GCC---AAGCCG---TACAGTGTTCCTATG
CAAACAGAGATCCGTTCACTGGATTTTTGGCGAGCCATTGTTGCAGAATGTCTTGCTACT
TTCTTCCTGGTTTTTGTCATTTGCGGATCCTTCGTCCCTTGGAGTGGC---------CAT
AGCCCGCCGTATATCGGGATAGCCCTGGCTGCGGGATTCGTCGTGGCTGGCCTAACCCTG
GCTTTTAGCCACACTTCAGGTGCTAATTTTAACCCTGTCGTCTCGTTGGCGCTTCTTGTG
ACCCGCAGAATTTCGCCAATTAAGGCCTTCCTGTTTGTGACGGCGCAAGGCGGAGGCGCC
ATTGCTGGTGCTGCTTTATTATACGGGGTAACGATTCCGGGTAATCAG---GGTAACCTG
GGA---------------------------------------------------------
------------------GCAACGGTGGTATCGTCGGGACTGACGGAATGGCAGGCGCTG
GGTATCGAGTTGTGCATGACCTTCATCATCGTTCTAGTTTACCTGACTACCATGGATTCG
CAACGGCGGGCTCTGTCT---GCTTCCATCGGACCTGGACCTCTCACTTATGGCCTCATC
TAT---ATGGCCTGCTCCCTTGTGGCTCTGCCATCTACCGGCGCTTCGTTCAACCCGATG
CGGTCGCTGGGACCTGCCTTCGTTATG---------------------------------
AAC---AAGTGGGACGCCCACTGGGTATATTGGCTGGGTCCTATTGTTGGTGGCTGCATG
GCTGGATTGTTGTAC---GAGTTCATATTCAATCCCTATCGCAGTGGA---------CGC
AAACGTAAGGGATCCGTA---GATGGAGATTCTACCGGAGCCCAGAGTGACGAAGAA---
TACGACGAAGTCAAGTCTACGGTGCAG------------AACTACCAT---------TCC
ATGCGGATGCAGCACTACGATCAGTACCGTCCACCGTACAGCCCGGGAGGTCCTTTGAAC
CCCACCACATCCCACGGTAAATTA---TCAGCA---------------------------
------AGG---GTC---GATAGGGCGGAATCCGTTTACGGTGGGACCAAATCGCTGTAC
---TGC---AAATCACCTCCC---ATGACCAGG---GCCAATCTGAACCGTTCCCAGTCG
GTTTACGCCAAACCCCCG---GGTCTGAATCAGGGTCCAGCCGGTCCGGCTTACCCCATG
AAG------ACCCTACCTTCTGCCCAGAGCACCTACTTGGCC---CGAAATGTGGACCTA
ATCCGGACCGAAAGCATTTATGCTACCAGACCTGCTAACATA------CCCATC------
------------------------------------------AATCGG------------
TAC---------------------------CCAAAATCGAAAAACAGACAG---AAC---
------------------------------------------------------------

------------------------------------------------------------
------------------------------------------------------------
------------------------------------------------------------
------------------------------------------------------------
------------------------------------------------------------
------------------------------------------------------------
------------------------------------------------------------
---------------------------------------------------------GGC
CAGGGACCAATCAGAAACCAAGGAAAT---------TAC---------------CCGCCT
---------------CACGTCTATGGTCAGAAATTCAGAGCTGACTTGATGTCACCCGGA
AGCTCCGCCCACTCGCCCGGAAACACGCCCACAGCG---------TTTCATTCGCCCGTG
CAGTAT
>JAIQCU010005059_Leptestheria_dahalacensis_Bib
---------------------ATGACGACCATCAGT---GTAACCTCGCCACTG------
------ACAACGGCCAGTCTAGAATCGCTTCTTCAGACCCTGCTGGAACGTTTTGAGAGT
TGCAAAAGTGACTTA---------------CCC---AAAGCC---GGCAGTATCCCCATG
CAAGTGGAGATCCGCTCCTTGGACTTCTGGCGCGCTATTGTAGCCGAGTGCCTGGCCACT
TTTTTCCTCGTCTTTGTCATCTGCGGATCCTTCGTGCCTTGGAATGGA---------CAC
AGTCCGCCTTACATTGGAATCGCCTTGGCCACTGGGTTCGTGGTCGCTGGACTGACTCTC
TGTTTCACTCATACTTCCGGTGCTAATTTCAACCCCGTCGTCTCGCTGGCATTGTTGGTG
ACTCGCAGAATTTCGCCCATCAAGGCCTTCCTGTTTGTTACGGCGCAGGGAGGAGGGGCT
ATCGCTGGCGCCGCACTACTTTATGGGGTGACCGTGTCTGGACAACAA---GGCAACCTG
GGC---------------------------------------------------------
------------------GTCACTTCTGTTTCCCTGGGTCTGTCGGAATGGCAAGCGCTG
GGCATCGAACTGTGCATGACGTTCATCATCGTCCTCGTCTACCTGACCACCATGGACTCG
CACCGGCGGGCCCTGTCC---GCCTCCATCGGACCCGGACCCTTGACTTACGGACTCATC
TAC---ATGGCCTGTTCTCTTGTCGCTTTGCCATCTACCGGCGCTTCATTCAACCCTATG
CGATCGCTGGGACCTGCCTTCGTGATG---------------------------------
AAC---AAATGGGATGCTCATTGGGTGTATTGGTTGGGCCCCATCGTAGGCGGATGCATG
GCCGGACTCCTCTAC---GAGTTCATCTTTAACCCGTACCGCAGTGGA---------AGG
AGACGCAAGGGTTCTGTT---GACGGAGATTCGACGGGGGCTCACAGCGACGAAGAG---
TACGACGAAGTGAAATCCAGCACCCAG------------AACTATCAC---------TCG
GTGCGGATGCAGCACTACGATCAGTACCGTCCGCCCTACAGTGCCGGCGGACCCATGAAT
CCCACCTCTCCAACTGGT------------------------------------------
------CGC---CTG---GATCGTGCGGAATCAGTCTACGGTGGCACCAAATCGCTCTAC
---TGC---AAATCCCCGCCC---ATGAGCCGC---GTCAATCTGAACCGGTCCCAGTCC
GTCTACGCTAAACCGCCC---CCGCCGGCATCCGGCCCCGTGGGCGTGGCCTATCCAACC
AAG------ACACTTCCGGCGGCGCAGAGTACCTACCTGTCG---CGCAATGTGGATCTC
ATCCGCACCGAGAGTATTTACGCCACCCGCCCTGCCAATATT------CCCGTT------
------------------------------------------AACAGG------------
------------------------------------------------------------
------------------------------------------------------------
------------------------------------------------------------
------------------------------------------------------------
------------------------------------------------------------
------------------------------------------------------------
------------------------------------------------------------
------------------------------------------------------------

------------------------------------------------------------
------------------------------------------------------------
------------------------------------------------------------
------------------------------------------------------------
------------------------------------------------------------
------
>RJJA01000024_Lepidurus_arcticus_Bib
---------------------ATGGCTAATGTGAGC---TTG---GCGCCG---------
------AATTCCGCGTCCATGGAAACGTTGTTGCAAACTTTAATGGACAGGCTCGAATCG
CAGCGAACAGAGCGT---------------CCT---AAATTG---GCGCGAGTGCCTATG
CAAGCCGAAATTCGGAGCCTGGATTTTGGCGTGCCGTCGTCGCCGAGTGCCTCGCTACT
TTTTTCTACGTCTTCCTTATCTGCGGCTCTTTCGTGCCATGGAACGGG---------CAC
AGTCCGCCTTATGTCGGTATTGCCTTGGCAGCTGGCTTTGCTGTAGCTAGTCTTACCCTT
TCTTTTGCACACATTTCAGGCGCTCATCTAAACCCTTGCGTCACGTTAGCGCTTTTAATG
TCCAGGAAAATTTCTCCGATCAAAGGCCTTTTGTATGTCACTGCACAAGGGGGAGGAGCC
ATTGCGGGTTCTGCCTTATTATATGGGGTGACGTCCACGGGTTTACAA---GGATGGCTT
GGA---------------------------------------------------------
------------------GCCACAGTCCCCGCACCTGGGTTATCCGAATGGCAGGCATTA
GCCATCGAATTGAGTATGACCTTTGTGGTGGTTTTGACCCATCTAACTGCCTTGGACCCC
GTCCGGGGTTATTTGGTGAACACAAACATGGGACCCGGACCTTTGACTGTAGGCTTGGCC
TAT---GTGACATGCTCCCTAGTGGCGTTGCCCTCGACTGGAGCCTCTTTTAACCCAATG
CGGTCCTTAGGACCTGCCTTTGTTATG---------------------------------
AAC---AAATGGGATAGCCATTGGATTTACTGGATAGGACCCATAATTGGAGGATGTTTG
GCAGGATTGCTTTAC---GAATTCGTGTTCAATCCGTATCGCGGTACG---------AAG
AAACGCAAGGATTCCATC---GACGGCGACTCGCTGAGCATGCACAGTGATGAAGAC---
TTCGATGAAGAAAAAAACGGACGTCAATTAGCGATAGCTAATTTCCAG---------AAT
GCCCGGACTCAGCACTATGATCAATATCGCCCTGCTTTGCGC------------TCCAAT
CCCGTTTCTCCTACAGGTCACATGGCCTCTAAC------GCTGCACTGGCGTCAAGAAGC
AGC---AGA---CCT---GATAGAGGAGATCCAATTTATGATGGCTCTAAATCGCTCTAT
---TGC---AAAACGCCTCAA---GTTGCACCTAAAGCAATTTTGAATCGCTCCCAATCT
GTCTACACTAAACCACCGTTACCGCCCGCGAATCCCTCTCCT------CCTCTGCAGTCA
---------GGGCTGGCACCCGCCCAGAGTATGTATTTGGGCAAAGTTAGCAGTCAGATG
---AGCCAACAGCACCATCCGCTATCAGTGCCTTCCAGGACTGGAAGAATTTATTGTTTT
AAGGGGTCCATTTTTCATAATTTACGATGTCTTGTTCCTAAATACAGGAATGAAAGCGTT
TACGGCATGCACCGCGAGCGTGAGTCACCGTATGGTGCTAAGATCACCGAC---AATTAC
GGCCCAATTAGTCCGCCCAGTAATTACGCAGACCAAAAATCCAGCGAAGAAAATTATCTT
AACAGTTTCCCGGCCTCAAAGAGCGGCAAT---CCG---GCCAGCCATATGTATAAACCG
CCCATGGACAGGGAGTCCTATGAACGCAGTGAACCCATGTACACTCATAAGCCCAGAGAA
CAAATGCCCTACGGCGATACGAATGGAAAT------GGGAGCCGAATGGAA---TCTGTG
TATGGCATGCAAGGAGCCGGGCATGGAATTACCCAAGAATCCATCTACGCCACT---AAG
AAGTTGGAACCGAATGCCAATAATCCTCCTCAAATGGGACATTCT---GATCCTAACAAT
TATGCTATGTACCCCCGCAGC------GCTTCGCTAATC---CGTAACGAT---TCGGTT
TACGGAGTTGTCCCACCGATTCGGCGCCCGACTCCACCCATGCAGACG---CAAATG---
ATGAACAGTGGCAGCAACATGCCCGTCAACTCGCCTTCACCAGCTGATACTTCCAAAGAT
TCAGCCTACGGGTCTCAGTACGGTGGTCCGATGGATCATGAATTACTGAATAGACCGCCA
TCTCACGGCAGTTATACCACCAATATGAACAAAATGCGAGGTGACTTGATGTCGCCTAAC
TCTCAATCAAGGTCAAATCCACAAACACCTACGTCTGCGACGCCGTATCATTCTCCT---
CAGTAC

>JAGJRP010000055_Lepidurus_couesii_Bib
---------------------ATGGCTAATGTGAGC---TTG---GCGCCG---------
------AATTCCGCGTCCATGGAAACGTTGTTACAAACTTTAATGGATAGGCTCGAATCG
CAGCGAACAGAGCGT---------------CCT---AAATTG---GCGCGAGTGCCTATG
CAAGCCGAAATTCGGAGCCTGGATTTTTGGCGTGCCGTCGTCGCCGAGTGTCTCGCTACT
TTTTTCTACGTCTTCCTCATCTGCGGCTCTTTCGTGCCATGGAACGGG---------CAC
AGTCCGCCTTATGTCGGTATCGCCTTGGCAGCTGGCTTTGCTGTAGCTAGTCTTACCCTT
TCTTTTGCACACATTTCAGGCGCTCATCTGAACCCTTGCGTCACGTTAGCGCTTTTAATG
TCCAGGAAAATTTCCCCGATCAAAGGCCTTTTGTATGTCACCGCACAAGGGGGAGGAGCC
ATTGCGGGTTCTGCCTTATTATATGGGGTGACGTCCACGGGGTTGCAA---GGATGGCTT
GGA---------------------------------------------------------
------------------GCCACAGTCCCCGCACCTGGGTTATCCGAATGGCAGGCATTA
GCCATCGAATTGAGTATGACCTTTGTGGTCGTTTTGACCCATCTAACTGCCTTGGACCCC
GTCCGGGGTTATTTGGTGAACACAAACATGGGACCCGGACCTTTGACTGTAGGCTTGGCC
TAT---GTGACATGCTCCCTAGTGGCGTTGCCCTCGACTGGAGCCTCTTTTAACCCAATG
CGGTCCTTAGGACCTGCCTTTGTTATG---------------------------------
AAC---AAATGGGATAGCCATTGGATTTACTGGATAGGACCCATCATTGGAGGATGTTTG
GCAGGATTGCTCTAC---GAATTCGTGTTCAATCCGTATCGCGGTACG---------AAG
AAACGCAAGGATTCCATC---GACGGCGACTCGCTGAGCATGCACAGTGATGAAGAC---
TTCGATGAAGAGAAAAACGGACGTCAATTAGCGATAGCTAATTTCCAG---------AAT
GCCCGGACTCAGCACTATGATCAATATCGCCCTGCTCTGCGC------------TCCAAT
CCAGTTTCTCCTACAGGTCACATGGCCTCTAAC------GCTGCACTGGCGTCAAGAAGC
AGC---AGA---CCT---GATAGAGGAGATCCAATTTATGATGGCTCTAAATCGCTCTAT
---TGC---AAAACGCCTCAA---GTTGCACCTAAAGCAATTTTGAATCGTTCCCAATCT
GTCTACTCTAAACCACCGTTACCGCCCGCGAATCCCTCCCCT------CCTCTGCAGTCA
---------GGGCTGGCACCCGCCCAGAGTATGTATTTGGGCAAAGTTAGCAGTCAGATG
---AGCCAACAGCACCATCCGCTATCAGTGCCTTCCAGGACTGGGAGAATTTATTGTTTT
AAGGGGTCCATTTTTCATGATTTACGACGTCTTGTTCCTAAATACAGGAATGAAAGCGTT
TACGGCATGCACCGCGAGCGTGAGTCACCGTATGGTGCTAAGATCACCGAC---AATTAC
GGCCCAATTAGTCCGCCCAGTAATTACGCAGACCAAAAATCCAGCGAAGAAAATTATCTC
AACAGTTTCCCGGCCTCGAAGAACGGCAAC---CCG---GCCAGCCATATGTATAAACCG
CACATGGACAGGGAGTCCTATGAACGCAGTGAACCCATGTACACTCATAAGCCCAGAGAA
CCAATGCCCTACGGCGATACGAATGGAAAT------GGGAGCCGAATGGAA---TCTGTG
TATGGCATGCAAGGAGCCGGGCATGGAATTACCCAAGAATCCATCTACGCCACT---AAG
AAGTTGGAACCGAATGCCAATAATCCTCCTCAAATGGGACATTCT---GATCCTAGCAAT
TACGCTATGTACCCCCGCAGC------GCTTCGCTAATC---CGTAACGAT---TCGGTT
TACGGAGTTGTCCCACCGATTCGGCGCCCGACTCCACCCATGCAGACG---CAAATG---
ATGAACAGTGGCAGCAACATGCCCGTCAACTCGCCCTCACCAGCTGATACTTCCAAAGAT
TCAGCCTACGGGTCTCAGTACGGTGGTCCGATGGATCATGAATTACTGAATAGACCGCCA
TCTCACGGCAGTTATACCACCAATATGAACAAAATGCGAGGTGACTTAATGTCGCCCAAC
TCTCAATCAAGGTCAAATCCACAAACACCGACGTCTGCGACGCCGTATCACTCTCCT---
CAGTAC
>JAKZAN010000006_Lepidurus_packardi_Bib
---------------------ATGGCTAATATGAGC---TTG---GCGCCA---------
------AACTCCGCGTCGATGGAAACGTTGTTGCAAACTTTGATGGACAGGCTCGAATCG
CAGCGAACAGAGCGT---------------CCT---AAATTG---GCGCGAGTGCCTATG
CAAGCCGAAATTCGGGGCCTGGATTTTTGGCGTGCCGTCGTCGCCGAATGTCTCGCTACT
TTTTTCTACGTCTTCCTTATCTGCGGCTCTTTCGTACCATGGAACGGG---------CAC

AGTCCGCCTTATGTCGGTATTGCCTTGGCAGCTGGCTTTGCTGTAGCTAGTCTTACCCTT
TCTTTTGCACACATCTCAGGCGCTCATCTGAACCCTTGTGTCACGTTAGCGCTTTTAATG
TCCAGGAAAATTTCTCCGATCAAAGGCCTTTTGTATGTCACTGCACAAGGGGGAGGAGCC
ATTGCGGGTTCTGCCTTATTATATGGGGTGACGTCCACGGGCTTGCAA---GGATGGCTT
GGA---------------------------------------------------------
------------------GCCACAGTCCCCGCACCTGGATTATCCGAATGGCAGGCATTA
GCCATCGAATTGAGTATGACCTTTGTGGTGGTTTTGACCCATCTAACTGCCTTGGACCCC
GTCCGGGGTTATTTGGTGAACACAAACATGGGACCCGGACCTTTGACTGTAGGCTTGGCC
TAT---GTGACTTGCTCTCTAGTGGCGTTGCCCTCGACTGGAGCCTCCTTTAACCCAATG
CGGTCCTTAGGACCTGCCTTTGTGATG---------------------------------
AAC---AAATGGGATAGCCATTGGATTTACTGGATTGGACCCATCATTGGAGGATGTTTG
GCAGGATTGCTCTAC---GAATTCGTGTTCAATCCGTATCGCGGTACG---------AAG
AAACGCAAGGATTCCATC---GACGGCGACTCGCTGAGCATGCACAGTGATGAAGAC---
TTCGATGAAGAGAAAAACGGACGTCAATTAGCGATAGCTAATTTCCAG---------AAT
GCCCGGACTCAGCACTATGATCAATATCGCCCTGCTCTGCGC------------TCCAAT
CCTGTTTCTCCTACA---------------------------------------------
----------------------------------ATTTATGATGGCTCTAAATCGCTCTAT
---TGC---AAAACGCCTCAA---GTTGCACCTAAAGCAATTTTGAATCGCTCCCAATCT
GTCTACACTAAACCACCGTTACCGCCCGCGAATCCCTCCCCT------TCTCTGCAATCA
---------GGATTGGCACCCGCCCAGAGTATGTATTTGGGAAAAGTTAGCAGTCAG---
---AGCCAACAGCACCATCCGCTATCAATGCCTTCGAGGACTGGGAGAATTTATTGTTTA
AATGGGTCCAGTTTTCATGATTTACGACGTCTTGTTCCTGAATACAGGAATGAAAGCGTT
TATGGCATGCACCGCGAGCGTGAGTCACCGTATGGTGCTAAGATCACCGAT---AATTAC
GGCCCAATTAGTCCGCCCAGTAATTACGCAGACCAAAAATCCAACGAAGAAAATTATCTC
AACAGTTTCCCGGCCTCGAAGAGCGGCATT---CCG---GCCAGCCATATGTACAAACCC
CCCACGGACAGGGAGTCCTATGAACGCAGTGAACCCATGTACACTCATAAGCCCAGAGAA
CAAGCGCCCTACGGCGATACGAATGGAAAT------GGGAGCCGAATGGAA---TCTGTG
TATGGCATGCAAGGAGCCGGGCATGGAATTACCCAAGAATCCATCTATGCCACA---AAG
AAGTTGGAACCGAATGCCAATAGTCCTCCTCAAATGGGACATTCT---GATCCTAGCAAT
TATGCCATGTACCCCCGCAGC------GCTTCGCTAATC---CGTAACGAT---TCGGTT
TACGGAGTTGTCCCACCGATTCGGCGCCCGACTCCACCCATGCAGACA---CAAATG---
ATGAACAATGGCAGCAACATGCCCGTCAACTCGCCTTCACCAGCTGATACTTCCAAAGAT
TCAGCCTACGGGTCTCAGTACGGTGGTCCAATGGATCATGAATTACTGAATAGACCGCCA
TCTCATGGCAGTTATACCACCAATATGAACAAAATGCGAGGTGACTTAATGTCGCCCAAC
TCTCAATCAAGGTCAAATCCACAAACACCGACGTCTGCGACGCCGTATCATTCTCCA---
CAGTAC
>RJJB01000010_Lepidurus_apus_Bib
----------------------ATGGCTAATGTGAGC---TTG---GCGCCG---------
------AATTCCGCGTCGATTGAAGCGTTGTTGCAAACTTTGATGGACAGGCTCGAATCG
CAGCGAACAGAGCGT---------------CCT---AAATTG---GCGCGAGTGCCTATG
CAAGCCGAGATTCGGGGCCTGGATTTTTGGCGTGCCGTCGTCGCCGAATGTCTCGCTACT
TTTTTCTACGTCTTCCTTATCTGCGGCTCTTTCGTGCCATGGAACGGG---------CAC
AGTCCGCCTTATGTCGGTATTGCCTTGGCAGCTGGCTTCGCTGTAGCTAGTCTTACCCTT
TCTTTTGCACACATCTCAGGCGCTCATCTCAACCCGTGCGTCACTTTAGCGCTTTTAATG
TCCAGGAAAATTTCTCCGATCAAAGCCCTTTTGTATGTCACAGCACAGGGGGGAGGAGCC
ATTGCGGGTTCTGCCTTATTATATGGGGTGACGTCCTCGGGTTTGCAA---GGATGGCTT
GGA---------------------------------------------------------
------------------GCCACGATACCAGCACCTGGCTTATCCGAATGGCAGGCATTA

GCCATCGAATTGAGTATGACATTTGTGGTAGTTTTAACCCACCTAACTGCCTTGGACCCC
GTCCGGGGTTATTTGGTGAACACAAACATGGGACCTGGACCTTTGACTGTGGGGCTGGCC
TAT---GTGACTTGCTCCTTAGTGGCGTTGCCCTCGACTGGAGCCTCCTTTAACCCAATG
CGATCCTTAGGACCTGCCTTTGTTATG---------------------------------
AAC---AAATGGGATAGTCATTGGATTTATTGGATTGGACCCATCATAGGAGGATGTTTG
GCAGGATTGCTCTAC---GAGTTCGTGTTCAATCCTTATCGCGGTACG---------AAG
AAACGCAAGGATTCCATC---GATGGAGACTCGCTGAGTATGCACAGTGATGAAGAC---
TTCGATGAAGAGAAAAACGGACGTCAATTAGCGATAGCTAATTTCCAG---------AAT
GCCCGGACTCAGCACTATGATCAGTATCGCCCTGCGTTGCGC------------TCCAAT
CCAGTTTCTCCTACAGGTCACATGGCCTCTAAC------GCTGCACTGGCGTCAAGAGGC
AGC---AGA---CCT---GATAGAGGGGATCCAATTTATGATGGCTCTAAATCGCTCTAT
---TGC---AAAACGCCTCAA---GTTGCACCTAAAGCAATTTTGAATCGCTCCCAATCT
GTCTACACTAAACCACCGTTACCACCCGCGAATCCCTCCCCT------TCTTTGCAATCA
---------GGGCTAGCACCTGCCCAGAGTATGTATTTGGGCAAAGTTAGCAGTCAG---
---AGCCAACAGCACCATCCGCTATCAGTGCCTTCCAGGACTGGGAGAATTTATTGTTTT
AATGTGCCCATTTTTCATGATTTACGACGTCTTGTTTCTAAATACAGGAATGAAAGCGTT
TACGGCATGCACCGCGAGCGTGAGTCACCGTATGGTGCAAAGATCACCGAC---AACTAC
GGTCCAATTAGTCCGCCCAGTAATTACGCAGACCAAAAATCCAGCGAAGAAAATTATCTC
AACAGTTTTCCAGCCTCGAAGAGTGGCATT---CCG---GCCAGCCATATGTATAAACCG
CCCACGGACAGGGAGTCCTATGAACGCAGTGAACCCATGTACACTCATAAGCCCAGAGAA
CAAATGCCCTACGGCGACACGAATGGAAAT------GGGAGCCGAATGGAA---TCAGTC
TATGGTATGCAAGGAGCCGGACATGGAATTACCCAAGAATCCATCTACGCCACT---AAG
AAGTTGGAACTGAATGCCAATAATCCTCCTCAAATGGGACATTCT---GATCCTGGCAAT
TATGCCATGTACCCCCGCAGC------GCTTCGCTAATC---CGTAACGAT---TCGGTC
TACGGAGTTGTCCCACCGATTCGGCGCCCGACTCCACCCATGCAAACG---CAAATG---
ATGAACAGTGGCACCAACATGCCCGTCAACTCGCCCTCACCAGCTGATACTTCTAAAGAT
TCTGCCTATGGGTCTCAGTACGGTGGTCCGATGGATCATGAGTTACTGAATAGACCGCCT
TCTCATGGCAGTTATACCACCAATATGAACAAAATGCGAGGTGATTTAATGTCGCCCAAC
TCTCAATCACGGTCAAATCCACAAACACCGACGTCTGCGACGCCGTATCATTCTCCT---
CAGTAC
>GEHY01010105_Triops_newberryi_Bib
---------------------ATGGCTGGTGTGAGT---TTAACCACG------------
------AATGTGACTTCGATGGAATCCTTATTACAGACCTTAATGGACCGCTTGGAATCG
CAACGGACAGAGCGG---------------CCG---AAACTC---TCGCGTGTGCCAATG
CAAGCCGAAATACGCGGTCTGGATTTCTGGCGCGCAGTTGTCGCAGAATGTCTGGCGACA
TTTTTTTACGTCTTTCTCATTTGCGGATCCTTTGTACCGTGGAATGGA---------CAT
AGTCCCCCATACGTCGGTATTGCCTTAGCTGCTGGCTTTGCTGTCGCTAGTCTTTCCCTG
TCCTTCGCACACATATCAGGTGCTCATTTGAATCCATGCGTCACATTAGCGCTGTTAATG
TCCAGGAAAATTTCGCCTATCAAAGGACTTTTGTATGTCACAGCCCAAGGCGGAGGAGCA
ATTGCCGGAGCTGCCTTATTATATGGGGTGACGTCTTCAGGGCTGCAA---GGATGGCTC
GGA---------------------------------------------------------
------------------GCGACGGTCCCGGCTCCTGGTTTATCCGAATGGCAAGCGTTG
GCCATTGAATTGAGTTTGACCTTTGTGGTGGTTTTAACCCATTTGACGGCGTTGGATCCT
ATCCGAGGTTATTTGGTGAACACAAACTTAGGACCGGGACCTTTGACCGTGGGCTTAGCA
TAT---GCGACCTGCTCTTTAGTGGCTTTGCCTTCGACTGGCGCCTCTTTCAATCCTGTA
AGGTCCCTAGGACCCGCATTTGTTATG---------------------------------
AAC---AAATGGGATAGTCACTGGGTTTACTGGATTGGACCGATCATTGGCGGATGTTTG
GCAGGACTGCTTTAC---GAGTTTGTGTTTAATCCTTACCGGGGGACG---------AAG

AAGCGCAAAGATTCCATC---GACGGAGACTCGTTAAGTATGCACAGCGATGAAGAT---
TACGACGAAGAGAAGAACGGACGCCAGATGGCCATAACGAATTTTCAG---------AAT
GCGCGGACTCAGCATTACGACCAGTATCGTCCCACGTTGCGT------------TCGAAT
CCGGTTTCTCCTACAGGTCACATGGCTTCAAAT------GCTGCATTAGCATCAAGAGGC
AGT---CGA---CCT---GATAGAGCAGATCCCATTTACGATGGCTCAAAATCCTTATAC
---TGC---AAAACGCCTCAA---GTTGTGCCCAAAGCTCCGTTGCATCGCTCTCAATCT
GTCTACGCTAAACCACCGTTGCCACCCGCCAATCCTTCGCCG------CCTCTGCAGTCA
---------GGTCTGGCACCCGCCCAGAGTCTCTATTTAGGCAAAATTAGTAGCAGCCAG
AACCATCAACCCCATCATCCTCTGTCAGTGCCTTCCAGGAACGGC---ATT---------
-----------------------------------------CATCGCAATGAAAGCGTG
TATGGAATGCACCGTGAGCGTGAGTCACCGTACGGAGCCAAGATTACCGAC---AATTAC
GGCCCAATTAGTCCGCCCAGCAGTTACGCTGACCAGAAATCCGCCGAAGAAAACTATTTG
AACAGTTTCCCTGCT---AAGAACGGGAAT---CCA---AGTAGCAACATGTATAAACCG
ACACCGGAGCGAGAGTCATTCGAACGTAATGATTCTGTCTATACGCACAAACCTCGCGAA
GCAATGCAGTACAATGATAATAACGGCAATAGCAATGCCCCACGGTTAGAG---TCCGTA
TATGGCATGCAAGGAACTGGACACGCAATTACTCAAGAATCTATTTATGCTACG---AAA
AAATTGGAGCAAAATTCGAATAATCATCCCAACATGAGTAATTCGGGTGATAACGGTAAT
TATGGCATGTATCCCCGTAGTAATGGTGTTCCGGTGGTC---CGTAATGAC---TCGGTT
TACGGGGTTGTACCACCGATTCGACGCCCAACTCCGCCAGTGCAGTCC---CAAATG---
ATGAACAGT---AGCAATTTGCCCGTGAATTCGCCGTCACCTGCTGATACGTCAAAAGAT
TCTGCCTACGGATCCCAGTACGGTGGTCCAATGGATCACGAGTTGTTAAATCGACCTCCT
TCCCATGGCAGTTATTCTATCAATATGAACAAAATGAGGGGAGATCTAATTTCGCCCAAT
TCTCAATCAAGGTCCAACCCGCATACGCCGACGTCTACGACGCCGTATCATTCTCCT---
CAATAT
>JAGQDJ010000608_Triops_longicaudatus_Bib
---------------------ATGGCTGGTGTGAGT---TTAACTACG------------
------AATGTGACTTCGATGGAATCTTTATTGCAGACCTTAATGGACCGCTTGGAATCG
CAACGGACAGAGCGG---------------CCG---AAACTC---TCGCGTGTGCCAATG
CAAGCCGAAATACGCGGTCTGGATTTCTGGCGCGCAGTTGTCGCAGAATGTCTGGCGACG
TTTTTTTACGTCTTTCTTATTTGCGGATCCTTTGTACCGTGGAATGGA---------CAT
AGTCCCCCATACGTCGGTATTGCCTTAGCTGCTGGCTTTGCTGTCGCTAGTCTTTCCCTG
TCCTTCGCACACATATCAGGTGCTCATTTGAATCCATGCGTCACATTAGCGCTCTTAATG
TCCAGGAAAATTTCGCCTATCAAAGGACTTTTGTATGTCACAGCCCAAAGCGGAGGGGCA
ATTGCCGGAGCTGCCTTATTATATGGGGTGACGTCTTCAGGGCTGCAA---GGATGGCTC
GGA---------------------------------------------------------
------------------GCGACGGTCCCGGCTCCCGGTTTGTCCGAATGGCAAGCATTG
GCCATTGAATTGAGTTTGACCTTTGTGGTGGTTTTAACCCATTTGACGGCGTTGGATCCC
ATCCGGGGTTATTTGGTGAACACAAACTTAGGACCGGGACCTTTGACCGTGGGCTTAGCA
TAT---GCGACCTGCTCTTTAGTGGCTTTGCCTTCGACTGGCGCCTCTTTCAATCCTGTA
AGGTCCCTAGGACCCGCATTTGTTATG---------------------------------
AAC---AAATGGGATAGTCACTGGGTTTACTGGATTGGACCGATCATTGGCGGATGTTTG
GCAGGACTGCTGTAC---GAGTTTGTGTTTAATCCCTACCGGGGTACG---------AAG
AAGCGCAAAGATTCCATC---GACGGAGACTCGTTAAGTATGCACAGCGATGAAGAT---
TACGATGAAGAGAAGAACGGACGCCACATGGCCATAACGAATTTTCAG---------AAT
GCGCGGACTCAGCATTATGACCAGTATCGCCCCGCGTTGCGT------------TCGAAT
CCGGTTTCTCCTACAGGTCACATGGCTTCAAAT------GCTGCATTAGCATCAAGAGGC
AGT---CGA---CCT---GATAGAGCAGATCCCATTTACGATGGCTCAAAATCCTTGTAC
---TGC---AAAACACCTCAA---GTTGTGCCCAAAGCTCCGTTGAATCGCTCTCAATCT

GTCTACGCGAAACCACCGTTGCCACCCGCCAATCCTTCGCCG------CCTCTGCAGTCA
---------GGTCTAGCACCCGCCCAAAGTCTCTATTTAGGCAAAATTAGTAGCAGCCAG
AACCATCAACCCCACCATCCTCTGTCAGTGCCTTCCAGGAACGGC---ATT---------
------------------------------------------CATCGGAATGAAAGCATG
TATGGAATGCACCGTGAGCGTGAGTCACCGTATGGAGCCAAGATTACCGAC---AATTAC
GGCCCAATTAGTCCGCCCAGCAGTTACGCTGACCAGAAATCCGCTGAAGAAAACTACTTG
AACAGTTTCCCTGCC---AAGAACGGCAAT---CCA---AGTAGCAACATGTATAAACCG
ACACCGGAGAGAGAGTCATTCGAACGCAAAGATTCTGTCTATACGCACAAACCTCGCGAA
GCAATGCAGTACAATGATAATAACGGCAATAGCAATGCCCCGCGGTTAGAG---TCCGTG
TATGGTATGCAAGGAACTGGACACGCAATTACTCAAGAATCTATTTATGCTACG---AAA
AAATTGGAGCAAAATTCCAATAATCATCCCAACATAAGTAACTCGGGTGATAACGGTAAT
TATGGCATGTATCCCCGTAGTAATGGTGTCCCGGTGGTC---CGTAATGAC---TCGGTT
TACGGGGTTGTACCACCGATTCGACGCCCAACTCCGCCAGTGCAGTCC---CAAATG---
ATGAACAGT---AGCAATTTGCCCGTGAATTCGCCGTCACCTGCTGATACGTCAAAAGAT
TCTGCCTACGGGTCGCAGTATGGTGGTCCAATGGATCACGAGTTGTTAAATCGGCCTCCT
TCCCATGGCAGCTATTCTATCAATATGAACAAAATGAGGGGAGATCTAATTTCGCCCAAT
TCTCAATCAAGGTCCAACCCGCATACGCCGACGTCCACGACGCCGTATCATTCTCCT---
CAATAT
>GCAT01010270_Triops_cancriformis_Bib
---------------------ATGGCTGGTGTGAGT---TTAACC---CCG---------
------AATGTGGCTTCGATGGAATCCTTATTACAGACCTTAATGGACCGCTTGGAATCG
CAACGCACAGAGCGC---------------CCG---AAACTG---TCGCGTGTGCCAATG
CAAGCCGAAATACGCGGTCTGGACTTCTGGCGTGCAGTTGTCGCGGAATGTCTGGCGACA
TTTTTTTACGTCTTTCTTATTTGCGGATCCTTTGTACCGTGGAATGGA---------CAC
AGTCCTCCATACGTCGGCATTGCTTTGGCCGCTGGCTTTGCCGTCGCTAGTCTTTCCCTG
TCCTTCGCACACATATCAGGTGCC------------------------------------
------AAAATTTCGCCCATCAAAGGACTTTTGTATGTCACAGCCCAAGGCGGAGGAGCA
ATTGCCGGAGCTGCCTTATTATATGGGGTGACGTCATCAGGGCTGCAA---GGATGGCTC
GGA---------------------------------------------------------
------------------GCGACGGTCCCGGCACCGGGCTTATCAGAATGGCAAGCATTG
GCCATTGAATTGAGTTTGACCTTTGTGGTGGTTTTAACCCATTTGACGGCTTTGGATCCT
ATCCGGGGCTATTTGGTGAATACCAACTTAGGACCGGGACCTTTGACCGTGGGTTTAGCG
TAT---GCGACCTGCTCTTTAGTGGCTTTGCCTTCTACTGGTGCCTCTTTCAATCCTGTA
AGATCCCTAGGTCCCGCGTTTGTTATG---------------------------------
AAC---AAATGGGACAGTCATTGGGTGTATTGGATTGGACCGATCATTGGTGGATGTTTG
GCTGGATTACTGTAC---GAGTTTGTGTTTAATCCGTACCGCGGGACG---------AAG
AAACGCAAAGATTCCATT---GATGGAGATTCGCTAAGTATGCACAGCGATGAAGAT---
TACGACGAAGAGAAAAACGGACGCCAGATGGCCATAACGAATTTCCAG---------AAT
GCGCGGACGCAGCATTACGACCAGTATCGCCCTGCGTTGCGC------------TCAAAT
CCGGTTTCTCCTGCAGGTCACATGGCATCGAAT------GCTGCACTAGCATCAAGAGGC
AGT---CGA---CCT---GACAGAGCAGATCCAATTTATGATGGCTCGAAATCCTTGTAC
---AGC---AAAACGCCACAA---GTTGTACCCAAAGCTCCGTTGAATCGTTCTCAATCC
GTCTACGCTAAACCACCGTTACCACCCGCCAATCCTTCGCCG------CCTCTGCAGTCA
---------GGCCTGGCACCCGCCCAGAGTCTCTATTTGGCAAAATTAGTAGCAGCCAG
AACCACCAGCCCCATCATCCTTTGTCAGTGCCTTCCAGGAACGGT---ATT---------
------------------------------------------CATCGCAATGAAAGCGTG
TATGGAATGCACCGCGAGCGTGAGTCACCGTATGGAGCCAAGATTACCGAC---AATTAC
GGACCAATTAGTCCGCCCAGCACTTACGCTGACCAGAAATCCGCCGAAGAAAACTACTTG

AACAGTTTTCCTGCC---AAGAACGGCAAC---CCG---GGTAGCAACATGTACAAACCG
CCTCCGGAGCGAGAGTCATTTGAACGCAGTGATTCTGTCTACACGCACAAACCTCGCGAC
ACAATGCAATACAACGATAGTAACGGCAATAACAGTGGCCCTCGGCTGGAA---TCCGTG
TACGGTATGCAAGGACCTGGACATGCAATTACTCAAGAATCTATTTATGCCACA---AAA
AAGTTGGAACAGCATTCCAACCACCATCCTAACATGAGCAACTCGGGGGATAACGGTAAT
TATGCCATGTATCCTCGTAGTAGTGGTGCCTCGGTGATC---CGTAATGAC---TCGGTT
TACGGAGTTGTACCACCGATTCGACGCCCAACTCCGCCAGTGCAGTCC---CAAATG---
ATGAACAGT---AGCAATTTGCCCGTCAATTCGCCATCGCCTGCAGATACGTCGAAAGAT
TCTGCCTACGGGTCGCAATACGGGGCCCCAATGGATCACGAGTTGTTAAATCGACCTCCG
TCCCATGGCAGTTATTCTATCAATATGAACAAAATGAGAGGAGATCTAATTTCGCCCAAT
TCTCAATCAAGATCCAACCCGCATACACCGACATCTACAACGCCGTATCATTCTCCT---
CAATAC
>CM051258_Artemia_sinica_Bib3p
------------------------------------------------------------
------------------------------------------------------------
------------------------------------------------------------
------------------------------------------------------------
------------------------------------------------------------
------------------------------------------------------------
------------------------------------------------------------
------------------------------------------------------------
------------------------------------------------------------
------------------------------------------------------------
------------------------------------------------------------
------------------------------------------------------------
------------------------------------------------------------
------------------------------------------------------------
------------------------------------------------------------
------------------------------------------------------------
------------------------------------------------------------
------------------------------------------------------------
------------------------------------------------------------
------------------------------------------------------------
------------------------------------------------------------
------------------------------------------------------------
------------------------------------------------------------
------------------------------------------------------------
------------------------------------------------------------
---------------------------------------TCAGAAAGTATTTACGTCCCA
CGA---------------------------------AGCCAATTTCAAAATGAAGCAGTT
TATTAC---AAGAAG---AACGAAAACGTTTATTCAACAAAGCCTCCTCTTGCCTCCGTC
CCA---------CCA---------------------TGGAGT------GATTCTGAC---
TATTCCCAGAAGGCA---------GATCAG---CTC---TACCAGCCTCCTCTTCCTCCT
ACTCCTATGAGTGAAGACCAAAAGCTGGAGGAGGAAAATATAAGGAATTTCGACTGCACC
AGAAATGAGTACACCAGAAGCTCGAATGATCCC---TATAACCGAAATAAA---AATGAA
TACGGA---AGACGTCAGTTACCTCAT---AGGCACGAATCCGTTTGTGGG------GTA
GCTCCCCCAATAAGGAGG------AGTTGCAAACAATTTTTCCCAAATAATTCTCATAGT
GATTCCGCATATGAGTCTGTAAATACGCCCTCTAAACCT---AGCGGAAAT---AAAGGG

TTTAACTACTCCCTGGCTGAAAATCAAAATAAGAACTATCAGCAGACTGAC---------
---------------------------AATGAGCTTTTAATTGTTGACGCCACGCGGGAA
TCGCTCTATGGC------------------------------------------------
------------TATTCT------------AAA------CAAGAGATTCTG---------
------AATCGACCAAAT------------TTTACTGATAATAATAACGTGGAAGTTGTC
CGATAT
>JAQQPU010007720_Artemia_sp_Kazakhstan_Bib2p
------------------------------------------------------------
------------------------------------------------------------
------------------------------------------------------------
------------------------------------------------------------
------------------------------------------------------------
------------------------------------------------------------
------------------------------------------------------------
------------------------------------------------------------
------------------------------------------------------------
------------------------------------------------------------
------------------------------------------------------------
------------------------------------------------------------
------------------------------------------------------------
------------------------------------------------------------
------------------------------------------------------------
------------------------------------------------------------
------------------------------------------------------------
------------------------------------------------------------
------------------------------------------------------------
------------------------------------------------------------
------------------------------------------------------------
------------------------------------------------------------
------------------------------------------------------------
------------------------------------------------------------
------------------------------------AGGTCAGAAAGTATTTACGTCCCA
CGA---------------------------------AGCCAATTTCAAAATGAAGCAGTT
TATTAC---AAGAAG---AACGAAAACGTTTATTCAACAAAGCCTCCTCTTGCCTCCGAC
CCA---------CCA---------------------CGGAGT------GATTCTGGC---
TATTCCCAGAAGGCA---------GATCAG---CTC---TACCAGCCTCCTCTTCCTCCT
ACTCCTATGAGTGAAGACCAAAAGCTGGAGGAGGAAAATATAAGGAATTTTGACTGCACC
GGAAATGAGTACACCAGAAGCTCGAATGATCCC---TATAACCGAAATAAA---AATGAA
TACGGA---AGACATCAGTTACCTCAA---AGGCACGAATCCGTTTGTGGG------GTA
GCTCCCCCAATAAGGAGG---GAAAGTTGCAAACAATTTTTCCCAAATAATTCTCATAGT
GATTCCGCATATGAGTCTGTAAATACGCCCTCTACACCT---AGCGGAAAT---AAAGGG
TTTAACTACTCCCTGACTAAAAATCAAAATAAGAACTATCAGCAGACTGAACAATTAAGA
GGTACT---GAGTATAAT---AGTAGTCAGGGAGCTTTGATTGTTGACGCCACGTGGGAA
TCGCTCTATGGC------------------------------------------------
------------TATTCT------------AAG------CAAGAGATTCTG---------
---CAGAATCGACCAAAT------------TTGACTGATAATAATAACGTGGAAGTTGTC
CGATAT

>CM051265_Artemia_sinica_Bib2p
------------------------------------------------------------
------------------------------------------------------------
------------------------------------------------------------
------------------------------------------------------------
------------------------------------------------------------
------------------------------------------------------------
------------------------------------------------------------
------------------------------------------------------------
------------------------------------------------------------
------------------------------------------------------------
------------------------------------------------------------
------------------------------------------------------------
------------------------------------------------------------
------------------------------------------------------------
------------------------------------------------------------
------------------------------------------------------------
------------------------------------------------------------
------------------------------------------------------------
------------------------------------------------------------
------------------------------------------------------------
------------------------------------------------------------
------------------------------------------------------------
------------------------------------------------------------
------------------------------------------------------------
------------------------------------------------------------
------------------------------------AGGTCAGAAAGTATTTACGTCCCA
CGA---------------------------------AGCCAATTTCAAAATGAAGCAGTT
TATTAC---AAGAAG---AACGAAAACGTTTATTCAACAAAGCCTCCTCTTGCCTCCGAC
CCA---------CCA---------------------CGGAGT------GATTCTGGC---
TATTCCCAGAAGGCA---------GATCAG---CTC---TACCAGCCTCCTCTTCCTCCT
ACTCCTATGAGTGAAGATCAAAAGCTGGAGGAGGAAAATATAAGGAATTTCGAGTGCACC
AGAAATGAGTACACCAGAAGCTCGAATGATCCC---TATAACCGAAATAAA---AATGAA
TACGGA---AGACGTCAGTTACCTCAA---AGGCACGAATCCGTTTGTGGG------GTA
GCTCCC---------------GAAAGTTGCAAACAATTT---CCAAATAATTCTCATAGT
GATTCCGCATATGAGTCTGTAAATACGCCCTCTACACCT---AGCGAAAAT---AAAGGG
TTTAACTACTCCCTGACTAAAAATCAAAATAAGAACTATCAGCAGACTGAACAATTAAGA
GGTACT---GAGTATAAT---AGCAGTCAGGGAGCTTTAATTGTTGACGCCACGCGGGAA
TCGCTCTATGGC------------------------------------------------
------------TATTCT------------AAG------CAAGAGATTCTG---------
---CAGAATCGACCAAAT------------TTGACTGATAATAATAACGTGGAAGTTGTC
CGATAT
>JAQQPU010007788_Artemia_sp_Kazakhstan_Bib1
---------------------ATGGCGAAGAAAAGT---GTAACTGTTCCTTTAAATACA
AATATATCTTCAGCAACAGTAGACACATTACTAGTGACGCTATTAGAAAAACTCGATAAA
AGAAATGATGATGAT------------------------AAT---CTGGTTATACCATTT
CAAGCGGAAATTAGGACTTTGGAATTTTGGAGATCGGTGGTGGCTGAATGTGTTGGAACT
TTTTTTTTGATATTCCTGACATGCGGTGCGTTTATACCTTGGTCAGGA---------CAC

AGCCCTCCATATATTGGAATTGGTTTAACTGTTGGATTCACTGTAGCTATGTTAACACAT
TGTTTCGCTCTAATTTCCGGTTCAAATTTCAACCCTTCGATAACGTTGGCTTTGGTTGTC
AAAAGATCTATGTCTCCATTTCGTGGTGTTTTGTACTTTGTGGCACAAATTGGAGGAGCA
ATAGCCGGATCAGCTTTATTATACGGGGTGACTACTTCCGGCTCGCAG---GGTAGCCTT
GGT---------------------------------------------------------
------------------GTAACAGTCATCGCATCAGGACTTCGGGATTGGCAAGGGTTC
GCAATAGAATTTTCCTTCACATTCATTATTGTGCTGTCTTATCTGGGCTCGTTGCATCCT
GAGCAAAAAAGAACTGTTTCAACTTCAATGGGAAGTGGTTCAATCATGGTTGGATTTAGT
TAC---CTAGCCTGTTCCCTTGTTGCTTTACCGTCCACTGGCGCGTCGTTCAACCCTGTG
AGGTCGCTGGGACCGGCGTTTGTGATG---------------------------------
AAC---AAATGGGATAGTCACTGGATATACTGGATTGGACCATTACTTGGTGGCGCGTTT
GGAGGACTTATACAC---GAGTTTGTTTTTAATACGTATCAAGATGGC---------TGC
CAAAGAAAGTCAACAGGT---GAAGGTGATTCTTCAAGTGTACATAGCGACGAAGAATGC
TATGAAGAGGCAGACCATGGTAGCACTCCATTGAGAGGTCCAGTTCCGGGAGCTCTGACT
GCTAGAAATCAACATTACGAAGCTTATAAGCCAATGACCGTT------------------
---------------------GCACAAGACACC------TTGTGCAAATCTTCAAGGAAT
------------TTA---GACCAATCAGAATCAATATATGCTGGAACGCGTTCACTTTAT
---TGT---CGTTCACCGATT---GCCGCTCAGCGCACCATGCTCAATCGCTCTCAGTCG
GTCTACTCCAAGTCCCAAAATCTGGTCAATCAA------AGGATTCATCCAGTGGCTAGC
GTC---GTGGCGCCAAAAAATGGTGGCTTTGAAGCACTTTCAAGACAAGAGAATATTTAC
GTT---GGTAGATCCGTTGCACCATCTATG------AGGTCAGAAAGTATTTACGTCCCA
CGA---------------------------------AGCCAATTTCAAAATGAAGCAGTT
TATTACTCAAAAAAACAGAACGAAAACGTTTATTCAACAAAGCCTCCTCTTGCCTCCGAT
CCA------TACCCA---------------------CGGAGT------GATTCTGGC---
TATTCTCAGAAGGCA---------GATCAG---CTC---TACCAACCTCCTCTTCCCCCT
ACTCCTATGAGTGAAGATCAAAAGCTGGAGGAGGAAAATATAAGGAATGCCGAGTGCACC
AGAAATGAGTACACCAGAAGCTCGAATGATCCC---TATAACCGAAATGAA---AATGAA
TACGGA---AGACGTCAGTTACCTCAA---AGGCATGAATCCGTTTGTGGG------GTA
GTTCCTCCAATAAGGAGGGATGAAAGTTACAAACAATTCTCCCCAAACAATTCTCATAGT
GATTCCGCATATGGGTCTGTAAATACGCCCTCTACACCT---AGCGGAAAT---AAAGGG
TTTAACTACTCCCTGACTGAAAATCAAAATAAGAACTATCAGCAGACTGAACAATTAAGA
GGTAAT---GAGTATAAT---AGCAATCAGGGAGCTTTAATCGTTGACGCCACGCGGGAA
TCGCTCTATGGC------------------------------------------------
------------TATTCT------------AAA------CAAGAGATGCTG---------
---CAGAATCGACAGAAT------------TCAACGGATAATCATAATGTGCAAGCTGTC
AGATAT
>CM051265_Artemia_sinica_Bib1
---------------------ATGGCGAAGAAAAGT---GTAACTGTTCCTTTGAATACA
AATATATCTTCAGCAACAGTAGACACATTACTAGTGACGCTATTAGAAAAACTCGATAAA
AGAAATGATGATGAT------------------------AAT---CTGGTTATACCATTT
CAAGCGGAAATCAGGACTTTGGAATTTTGGAGATCAGTGGTGGCTGAATGTGTTGGGACC
TTTTTTTTTATATTCTTGACATGCGGTGCGTTTATACCTTGGTCAGGA---------CAC
AGCCCTCCATATATTGGAATTGGTTTAACTGTTGGATTCACTGTAGCCATGTTAACACAT
TGTTTCGCTCTAATTTCCGGTGCAAATTTCAACCCTTCGATAACGTTGGCTTTGGTTGTC
AAAAGATCTATGTCTCCATTTCGTGGTGCTTTGTACTTTGTGGCACAAATTGGAGGAGCA
ATAGCCGGATCAGCTTTATTATACGGGGTGACTACTTCCGGCTCGCAG---GGTAGCCTT
GGT---------------------------------------------------------
------------------GTAACAGTCATCGCATCAGGTCTTCGGGATTGGCAAGGGTTC

GCAATAGAATTTTCCTTCACATTCATTATTGTGCTGTCTTATCTGGGCTCGTTGCATCCT
GAGCAAAAAAGAACTGTTTCAACTTCAATGGGAAGTGGTTCAATCATGGTTGGATTTAGT
TAC---CTAGCCTGTTCCCTTGTTGCTTTACCGTCCACTGGCGCGTCGTTCAACCCTGTG
AGGTCGCTGGGACCGGCGTTTGTGATG---------------------------------
AAC---AAATGGGATAGTCACTGGATATACTGGATTGGACCATTACTTGGTGGCGCGTTT
GGAGGACTTATACAC---GAGTTTGTTTTAATACGTATCAAGATGGC---------TGC
CAAAGAAAGTCAACAGGT---GAAGGTGATTCTTCAAGTGTACATAGCGACGAAGAATGC
TATGAAGAGGCAGACCATGGTAGCACTCCATTGAGAGGTCCAGTTCCGGGAGCTCTGACT
GCTAGAAATCAACATTATGAAGCTTATAAGCCAATGACCGTT------------------
---------------------GCACAAGACACC------TTGTGCAAATCTTCAAGGAAT
------------TTA---GACCAATCAGAATCAATATATGCTGGAACGCGTTCACTTTAT
---TGT---CGTTCACCGATT---GCCGCTCAGCGCACCATGCTCAATCGCTCTCAGTCA
GTCTACTCCAAGTCCCAAAATCTGGTCAGTCAA------AGGATTCATCCAGTGGCTAGC
GTC---GTGGCGCCAAAAAATGGTGGCTTTGAAGCACTTTCAAGACAAGAGAATATTTAC
GTT---GGTAGATCCGTTGCACCATCTATG------AGGTCAGAAAGTATTTACGTCCCA
CGA---------------------------------AGCCAATTTCAAAATGAAGCAGTT
TATTACTCAAAAAAGCAGAACGAAAACGTTTATTCAACAAAGCCTCCTCTTGCCTCCGAT
CCA------TACCCA---------------------CGGAGT------GATTCTGGC---
TATTCTCAGAAGGCA---------GATCAG---CTC---TACCAACCTCCTCTTCCCCCT
ACTCCTATGAGTGAAGATCAAAAGCTGGAGGAGGAAAATATAAGGAATGCCGAGTGCACC
AGAAATGAGTACACCAGAAGCTCGAATGATCCC---TATAACCGAAATGAA---AATGAA
TACGGA---AGACGTCAGTTACCTCAA---AGGCATGAATCCGTTTGTGGG------GTA
GTTCCTCCAATAAGGAGGGATGAAAGTTACAAACAATTCTCCCCAAACAATTCTCATAGT
GATTCCGCATATGGGTCTGTAAATACGCCCTCTACACCT---AGCGGAAAT---AAAGGG
TTTAACTACTCCCTGACTGAAAATCAAAATAAGAACTATCAGCAGACTGAACAATTAAAA
GGTAAT---GAGTATAAT---AGCAATCAGGGAGCTTTAATCGTTGACGCCACGCGGGAA
TCGCTCTATGGC------------------------------------------------
------------TATTCT------------AAA------CAAGAGATGCTG---------
---CAGAATCGACAGAAT------------TCAACGGATAATCATAATGTGCAAGCTGTC
AGATAT
>JAVRJZ010000002_Artemia_franciscana_Jo_Bib
---------------------ATGGCGAAGAAAAGT---GTAACTGTTCCTTTAAATACA
AATATATCTTCAGCAACAGTAGACACATTACTAGTGACGCTATTAGAAAAGCTTGATAAA
AGACATGATGATGAT------------------------AAT---GTGGCTGTACCATTT
CAAGCAGAAGTTAGGACTTTGGAATTTTGGAGATCAGTTGTGGCTGAATGTGTTGGAACT
TTTTTTTTGATATTCCTGACATGTGGTGCGTTTATACCTTGGTCAGGA---------CAC
AGCCCTCCATATATTGGAATCGGTTTAACTGTTGGATTCACTGTAGCCATGTTAACACAT
TGTTTCGCTCTAATTTCCGGTGCAAATTTCAACCCTTCGATAACGTTGGCTTTGGTTGTC
AAAAGATCTATGTCTCCATTTCGTGGAGCTTTGTACTTTGTGGCACAAATTGGAGGAGCA
ATTGCGGGATCAGCCTTATTATACGGGGTGACTACTTCCGGCTCGCAG---GGTAGCCTT
GGT---------------------------------------------------------
------------------GTAACAGTCATCGCATCAGGTCTTCGGGATTGGCAAGGGTTC
GCAATAGAATTTTCCTTCACATTCATTATTGTACTGTCCTATCTGGGCTCCTTGCATCCT
GAGCAGAAAAGAACCGTTTCAACTTCAATGGGAAGTGGTTCAATCATGGTTGGATTTAGT
TAC---CTAGCATGTTCCCTTGTTGCTTTACCGTCCACTGGCGCGTCGTTCAACCCTGTG
AGGTCGCTGGGACCGGCGTTTGTGATG---------------------------------
AAC---AAATGGGATAGTCACTGGATATACTGGATTGGACCATTACTTGGTGGCGCATTT
GGAGGACTTATACAC---GAGTTTGTTTTAATACATATCAAGATGGC---------TGC

CAAAGAAAGTCAACAGGT---GAAGGCGATTCTTCAAGTGTACATAGCGACGAAGAATGC
TATGAAGAGGCAGACCATGGTAGCACTCCACTGAGAGGTCCAGTTCCGGGAGCTCTGACT
GCTAGAAATCAACACTATGAAGCTTATAAGCCAATGACCGTT------------------
---------------------GCGCAAGACAAC------TTGTGCAAATCTTCAAGGAAT
------------TTG---GACCAATCAGAATCAATATATGCTGGAACGCGTTCACTTTAT
---TGT---CGCTCACCGATA---GCTGCTCAGCGCACCATGCTCAATCGCTCTCAGTCA
GTCTACTCCAAGTCCCAAAATCTGGTTAATCAA------AGGATTCATCCAGTGGCTAGC
GCT---GTGGCACCAAAAAATGGTGGCTTTGAAGCACTTTCAAGACAAGAGAATATTTAC
GTT---GGTAGACTTGGGGCACCATCTATG------AGGTCAGAAAGTATTTATGTCCCA
CGA---------------------------------AGCCAATTTCAAAATGAAGCAGTT
TATTACTCAAAAAAGCAGAACGAAAACGTTTATTCAACAAAGCCTCCTCTTGCTTCCGAT
CCA------TACCCA---------------------CGGAGT------GATTCAGGC---
TATTCCCAGAAAGCA---------GATCAG---CTC---TACCAACCTCCTCTTCCTCCT
ACTCCTATGAGTGAAGACCAAAAGCTGGAGGAGGAAAATATAAGGAATGCTGAGTGCACC
AGAGCTGAGTACACCAGAAGCTCGAATGATCCC---TATAACCGAAATGAA---AATGAA
TACGGA---AGACGTCAGTTACCTCAA---AGGCATGAATCCGTTTGTGGG------GTA
GTTCCCCCAATAAGGAGAGATGAAAGTTACAAACAATTTTCCCCAAACAATTCTCATAGT
GATTCCGCATATGGTTCTGTAAATACGCCCTCTACACCT---AGCGGAAAT---AAAGGG
TTTAACTACTCCCTCTCTGAAAATCAAAATAAGAACTATCAGCAGACTGAACAGTTAAGA
GGTAAT---GAGTATAAT---TGCAATCAGGGAGCTTTAATTGTTGACGCCACGCGGGAA
TCCCTCTATGGC------------------------------------------------
------------TATTCT------------AAG------CAAGAGATGCTG---------
---CAGAATCGACAAAAT------------TCAACTGATAATCATAACGTGCAAGCTGTC
CGATAT
>PQ469256_Artemia_franciscana_Bib
---------------------ATGGCGAAGAAAAGT---GTAACTGTTCCTCTAAATACA
AACATATCTTCAGCAACAGTAGACACATTACTAGTGACGCTATTAGAAAAGCTCGATAAA
AGACACGATGATGAT------------------------AAT---GTGGCTATACCATTT
CAAGCAGAAGTTAGGACTTTGGAATTTTGGAGATCAGTTGTGGCTGAATGTGTTGGAACT
TTTTTTTTGATATTCCTGACATGTGGTGCGTTTATACCTTGGTCAGGA---------CAC
AGCCCTCCATATATTGGAATCGGTTTAACCGTTGGATTCACTCTAGCCATGTTAACACAT
TGTTTCGCTCTAATTTCCGGTGCAAATTTCAACCCTTCGATAACGTTGGCTTTGGTTGTC
AAAAGATCTATGTCTCCATTTCGTGGAGCTTTGTACTTTGTGGCACAAATTGGAGGAGCA
ATTGCGGGATCAGCCTTATTATACGGAGTGACTACTTCCGGCTCACAG---GGTAGCCTT
GGT---------------------------------------------------------
------------------GTAACAGTCATCGCATCAGGTCTTCGGGATCGGCAAGGGTTC
GCAATAGAATTTTCCTTCACATTCATTATTGTACTGTCCTATCTGGGCTCCTTGCATCCT
GAGCAGAAAAAAACCGTTTCAACTTCAATGGGAAGTGGTTCAATCATGGTTGGATTTAGT
TAC---CTAGCATGTTCCCTTGTTGCTTTACCGTCCACTGGCGCGTCGTTCAACCCTGTG
AGGTCGCTGGGACCGGCGTTTGTGATG---------------------------------
AAC---AAATGGGATAGTCACTGGATATACTGGATTGGACCATTACTTGGTGGCGCATTT
GGAGGACTTATACAC---GAGTTTGTTTTTAATACATATCAAGATGGC---------TGC
CAAAGAAAGTCAACAGGT---GAAGGCGATTCTTCAAGTGTACATAGCGACGAAGAATGC
TATGAAGAGGCAGACCATGGTAGCACTCCACTGAGAGGTCCAGTTCCGGGAGCTCTGACT
CCTAGAAATCAACACTATGAAGCTTATAAGCCAATGACCGTT------------------
---------------------GCCCAAGACAAC------TTGTGCAAATCTTCAAGGAAT
------------TTG---GACCAATCAGAATCAATATATGCTGGAACGCGTTCACTTCAT
---TGT---CGCTCACCGATA---GCTGCTCAGCGCACCATGCTCAATCGCTCTCAGTCA

GTCTACTCGAAGTCCCAAAATCTGGTTAATCAA------AGGATTCATCCAGTGGCTAGC
GCT---GTGGCACCAAAAAATGGTGGCTTTGGAGCACTTTCAAGACAAGAGAATATTTAC
GTT---GGTAGACTTGGGGCACCATCTATG------AGGTCAGAAAGTATTTACGTCCCA
CGA---------------------------------AGCCAATTTCAAAATGAAGCAGTT
TATTACTCAAAAAAGCAGAACGAAAACGTTTATTCAACAAAGCCTCCTCTTGCTTCCGAT
CCA------TACCCA---------------------CGGAGT------GATTCAGGC---
TATTCCCAGAAAGCA---------GATCAG---CTC---TACCAACCTCCTCTTCCTCCT
ACTCCTATGAGTGAAGACCAAAAGCTGGAGGAGGAAAATATAAGGAATGCTGAGTGCACC
AGAGCTGAGTACACCAGAAGCTCGAATGATCCC---TATAACCGAAATGAA---AATGAA
TACGGA---AGACGTCAGTTACCTCAA---AGGCATGAATCCGTTTGTGGG------GTA
GTTCCCCCAATAAGGAGAGATGAAAGTTACAAACAATTTTCCCCAAACAATTCTCATAGT
GATTCCGCATATGGTTCTGTAAATACGCCCTCTACACCT---AGCGGAAAT---AAAGGG
TTTAACTACTCCCTGTCTGAAAATCAAAATAAGAACTATCAGCAGACTGAACAGTTAAGA
GGTAAT---GAGTATAAT---TGCAATCAGGGAGCTTTAATTGTTGACGCCACGCGGGAA
TCCCTCTATGGC------------------------------------------------
------------TATTCT------------AAG------CAAGAGATGCTG---------
---CAGAATCGACAAAAT------------TCAACTGATAATCATAACGTGCAAGCTGTC
CGATAT
>JAYKOS010000004_Artemia_franciscana_Bett_Bib
---------------------ATGGCGAAGAAAAGT---GTAACTGTTCCTTTAAACACA
AATATATCTTCAGCAACAGTAGACACATTACTAGTGACGCTATTAGAAAAGCTCGATAAA
AGACATGATGATGAT------------------------AAT---GTGGCTGTACCATTT
CAAGCAGAAGTTAGGACTTTGGAATTTTGGAGATCAGTTGTGGCTGAATGTGTTGGAACT
TTTTTTTTGATATTCCTGACATGTGGTGCGTTTATACCTTGGTCAGGA---------CAC
AGCCCTCCATATATTGGAATCGGTTTAACTGTTGGATTCACTGTAGCCATGTTAACACAT
TGTTTCGCTCTAATTTCCGGTGCAAATTTCAACCCTTCGATAACGTTGGCTTTGGTTGTC
AAAAGATCTATGTCTCCATTTCGTGGAGCTTTGTACTTTGTGGCACAAATTGGAGGAGCA
ATTGCGGGATCAGCCTTATTATACGGGGTGACTACTTCCGGCTCGCAG---GGTAGCCTT
GGT---------------------------------------------------------
------------------GTAACAGTCATCGCATCAGGTCTTCGGGATTGGCAAGGGTTC
GCAATAGAATTTTCCTTCACATTCATTATTGTACTGTCCTATCTGGGCTCCTTGCATCCT
GAGCAGAAAAAAACCGTTTCAACTTCAATGGGAAGTGGTTCAATCATGGTTGGATTTAGT
TAC---CTAGCATGTTCCCTTGTTGCTTTACCGTCCACTGGCGCGTCGTTCAACCCTGTG
AGGTCGCTGGGACCGGCGTTTGTGATG---------------------------------
AAC---AAATGGGATAGTCACTGGATATACTGGATTGGACCATTACTTGGTGGCGCATTT
GGAGGACTTATACAC---GAGTTTGTTTTTAATACATATCAAGATGGC---------TGC
CAAAGAAAGTCAACAGGT---GAAGGCGATTCTTCAAGTGTACATAGCGACGAAGAATGC
TATGAAGAGGCAGACCATGGTAGCACTCCACTGAGAGGTCCAGTTCCGGGAGCTCTGACT
CCTAGAAATCAACACTATGAAGCTTATAAGCCAATGACCGTT------------------
---------------------GCCCAAGACAAC------TTGTGCAAATCTTCAAGGAAT
------------TTG---GACCAATCAGAATCAATATATGCTGGAACGCGTTCACTTTAT
---TGT---CGCTCACCGATA---GCTGCTCAGCGCACCATGCTCAATCGCTCTCAGTCA
GTCTACTCGAAGTCCCAAAATCTGGTTAATCAA------AGGATTCATCCAGTGGCTAGC
GCT---GTGGCACCAAAAAATGGTGGCTTTGAAGCACTTTCAAGACAAGAGAATATTTAC
GTT---GGTAGACTTGGGGCACCATCTATG------AGGTCAGAAAGTATTTACGTCCCA
CGA---------------------------------AGCCAATTTCAAAATGAAGCAGTT
TATTACTCAAAAAAGCAGAACGAAAACGTTTATTCAACAAAGCCTCCTCTTGCTTCCGAT
CCA------TACCCA---------------------CGGAGT------GATTCAGGC---

TATTCCCAGAAAGCA---------GATCAG---CTC---TACCAACCTCCTCTTCCTCCT
ACTCCTATGAGTGAAGACCAAAAGCTGGAGGAGGAAAATATAAGGAATGCTGAGTGCACC
AGAGCTGAGTACACCAGAAGCTCGAATGATCCC---TATAACCGAAATGAA---AATGAA
TACGGA---AGACGTCAGTTACCTCAA---AGGCATGAATCCGTTTGTGGG------GTA
GTTCCCCCAATAAGGAGAGATGAAAGTTACAAACAATTTTCCCCAAACAATTCTCATAGT
GATTCCGCATATGGTTCTGTAAATACGCCCTCTACACCT---AGCGGAAAT---AAAGGG
TTTAACTACTCCCTGTCTGAAAATCAAAATAAGAACTATCATCAGACTGAACAGTTAAGA
GGTAAT---GAGTATAAT---TGCAATCAGGGAGCTTTAATTGTTGACGCCACGCGGGAA
TCCCTCTATGGC------------------------------------------------
------------TATTCT------------AAG------CAAGAGATGCTG---------
---CAGAATCGACAAAAT------------TCAACTGATAATCATAACGTGCAAGCTGTC
CGATAT
>JAKZAO010000014_Branchinecta_lindahli_Bib
---------------------ATGGGTCGCTCAAGT---GCAGCAGTTCCCTTAAACCAA
AACCTGTCATCAGCAACAGTTGACACCCTGCTTTTGACTTTGTTGAGGAAACTTGACAAA
GAAAAGGAGGAA------------------CCT------TGG---GCGAAAGTTCAATTC
CAAGCCGAAGTGAGAACTTTGGAATTTTGGAGATCTGTAGTTGCCGAATGTGTTGGAACA
TTTTTTCTTGTGTTTCTAACCTGTGGAGCATTTGTGCCGTGGGCTGGG---------CAT
ACTCCCCCGCCTATTGGAATTGCCCTGACTGTTGGATTTGGGGTTGTGATGTTAACACAC
TGCTTTGCGCTCATTTCAGGTGCCAATTTTAATCCTGTGGTGACGCTTGCGCTTGTGGTC
AAAAGAAGCATGTCACCGTTTCGCGGCGCTCTCTATTTCACTGCCCAGGTCGGAGGATCC
ATCGCCGGATCAGCTTTGCTCTATGGGGTGACGTCATCCGGATCTCAA---GGGAATCTG
GGA---------------------------------------------------------
------------------GTAACTACACTTGCTGCCGGCTTGAAGGACTGGCAAGGCTTT
GCCATCGAACTCTGCTTCAGTTTCATCATCGTGCTGACTTACCTTTGTACCCTCCATCCA
GAGCAAAAGCGGACAGTATCTACATCTATGGGAACAGGTCCTATCACTGTGGGTTTTGCC
TAT---TTGGCTTGCTCGCTCGTGGGGCTGCCGTCTACTGGAGCTTCATTCAACCCCGTT
CGGTCGTTGGGTCCAGCTTTTGTCATG---------------------------------
AAC---AAATGGGATAGCCACTGGATTTACTGGGTGGACCCCTAATTGGAGGAGCCTTC
GGAGGTCTTATCCAC---GAGTTTGTCTTCAACGCCTATCGGGAAGGA---------AGC
AGCAGGAAGTGCAACGGC---GACGGAGATTCTTCGAGCATCCACAGCGATGAAGAATGC
TACGAGGAGGGGCCAGCTGGG---CCCCCTGTCCGAGGTCCAGTTCCGGGAGCTCTTGCT
GTCCGAAGCCAGCATTACGACACGTATAAGCCGATCGCCATA------------------
---------------------GGTATGGAAGGC------ATGTGCAAATCAACAAGGAAC
------------CTT---GACCAGACCGAAGGGATCTACTCTGGGACCCGCTCCCTCTAC
---TGC---CGATCGCCCGGT---CCTGCTCAGCGAAACATCCTCAACAGGTCCCAGTCC
GTCTACGCCAAGAATCCGGCGATCGGCGGGCAAAACTCCCGCATCCATCCAGCTGCCAGC
ATA---ACGACCACAAAACCCGAAGGATTTGAAGCAATCAGCCGCCAAGAGAGCATTTAC
TCGAGCGGCAGATGTCAGCCGCAG------CCCATAAGGTCAGGGAGTATCTACGCTCCT
CGA---------------------------------AGCCAAATTCAAAAAGAGACCGTC
TTCTACTCCAAGCAGCAGAATGAAAATGTTTACGCAACAAGGCCACCCCTTCCTTCAGAC
CCCGGCGTGTACCCCCCT------------------CGCCAT------GAGACACCC---
AACTCCTTC---GCT---------CCCCAG---GGA---TACCAGGCCCCCCTTCCCCCC
ACGCCAGTCAACGAGGACCAGAAGCTTGAAGAAGAAAACATGCGAAATGCAGAAAGCACA
AAG---------------GGCGACTTTGAC------TACGGAAGAAATGAA---GATGAC
TACGCCATGCGGAGAAAATTTCAACCT---CGACACGAGTCCGTGTGTGGA------GTG
GTGCCCCCCATAAGGCGGGAAGACAGTGGAAAGGGGTTCTCCCCCAACAATTCACATAGT
GACTCCGCCTATGGCTCAGTTAACACGCCATGCACGCCG---AATGTTAAC---CAGACC

TTTAACTACTCCCAAGGTGATCAGCAGAATCAAAATTACTCCCAAAATTACGAGCAATTT
AGGAGCAGTGAACAAGGGTTCGGCAATCAAGGCACCCTGGTTGTTGACGCTACGAGAGAG
TCGCTCTATGGT------------------------------------------------
------------TACTCG------------AAG------TCTAACCCGGGA---------
---CCGAAGAGATATCCG------------GCAGACAAGTCTCAGAATGTGCAGCCAGTT
CGGTAT
>JAKZAP010000001_Branchinecta_lynchi_Bib
---------------------ATGGGTCGCTCAAGT---GCAGCAGTTCCCTTAAACCAA
AACTTGTCATCAGCAACAGTTGACACGCTGCTTTTGACATTATTGAGGAAACTTGATAAA
GAAAAGGAGGAA------------------CCT------TGG---GCGAAAATTCCGTTC
CAAGCAGAAGTGAGAACTTTGGAATTTTGGAGATCTGTAGTTGCAGAATGTGTTGGAACA
TTTTTTCTTGTGTTTCTAACATGTGGAGCGTTTGTACCGTGGGCTGGG---------CAT
ACGCCCCCGCCTATTGGAATTGCCCTGACTGTTGGATTCGGCGTTGTGATGTTAACGCAC
TGCTTTGCACTCATTTCAGGTGCCAATTTTAATCCTGTGGTGACCCTTGCGCTTGTGGTC
AAAAGAAGCATGTCGCCATTTCGCGGGGCCCTCTATTTCACTGCCCAAGTCGGAGGATCC
ATCGCCGGATCAGCCTTGCTCTATGGGGTGACGTCATCCGGATCTCAA---GGGAATCTG
GGA---------------------------------------------------------
------------------GTAACCACACTTGCTGCCGGTTTGAAGGACTGGCAAGGATTT
GCCATTGAACTCTGCTTCAGTTTCATCATCGTGCTGACTTACCTTTGTACCCTCCACCCA
GAGCAAAAGCGGACTGTGTCTACATCAATGGGAACAGGTCCTATCACTGTGGGTTTCGCC
TAT---TTGGCTTGCTCGCTCGTGGGGCTACCATCTACTGGAGCTTCATTCAACCCCGTT
CGGTCGTTGGGTCCAGCTTTTGTCATG---------------------------------
AAC---AAATGGGATAGCCACTGGATTTACTGGGTGGGACCCCTAATCGGGGGAGCCTTC
GGAGGTCTTATCCAC---GAGTTTGTCTTCAACGCCTATCGAGAAGGA---------AGC
AGCAGGAAGTGCAATGGC---GACGGAGATGCTTCGAGCATCCACAGCGATGAAGAATGC
TACGAGGAGGGGCCAGCTGGG---CCCCCTGTCCGAGGGCCAGTTCCGGGAGCTCTCGCT
GTCCGAAGCCAGCATTATGATACGTATAAGCCGATCGCCATA------------------
---------------------GGTATGGAAGGC------ATGTGCAAATCAACACGAAAT
------------CTC---GACCAGACCGAAGGGATCTACTCCGGGACCCGCTCCCTCTAC
---TGC---CGATCCCCCGGT---CCTGCTCAGCGAAACATCCTCAACAGGTCCCAGTCC
GTCTACGCGAAGAATCCGGCGATCGGTGGGCAAAACTCCCGCATCCACCCAGCTGCCAGC
ATA---ACGACAACGAAACCCGGAGGATTTGAAGCAATCAGCCGCCAAGAGAGCATTTAC
TCGAGCGGCAGATGTCAGCCGCAG------CCCATAAGG---------------------
------------------------------------------------AAAGAGACCGTC
TTTTACTCGAAGCAGCAGAGCGAACACATCTACGCAACAAGGCCACCCCTTCCATCAGAC
CCGGGCGTGTACCCACCT------------------CGCCAT------GAGACACCC---
AACTCCTTC---GCT---------TCCCAG---GGA---TACCAGGCCCCCCTTCCCCCC
ACGCCAGTCAACGAGGACCAGAAGCTTGAAGAAGAAAACATGCGAAATGCAGAAAGCACA
AAA----------------GGAGACTTTGAC------TACGGAAAAAATGAA---GATGAT
TACGCCATGCGGAGGAAATTCCAACCT---CGACACGAGTCCGTGTGCGGA------GTG
GTGCCGCCCGTTCGGCGGGAAGACAGTGGAAAGGGGTTCTCCCCCAACAATTCTCATAGT
GACTCCGCCTATGGCTCCGTTAACACGCCGTGCACGCCG---AATGGTAAC---CAGACA
TTTAACTACTCCCAAGGGGATCAGCAGAATCAAAATTACTCCCAAAATTACGAGCAATTT
AGGAGCAGTGAACAAGGGTTCGGCAATCACGGCACCCTGGTTGTTGACGCTACGAGGGAG
TCACTTTATGGT------------------------------------------------
------------TACTCT------------AAG------TCGAAACCGGGA---------
---CCGAAGAGATATCCG------------GTGGAGAAGTCTCAAAATGTGCAGCCAGTT
CGGTAT

>JAUPMV010000002_Branchinecta_lindahli_Bib
---------------------ATGGGTCGCTCAAGT---GCAGCAGTTCCCTTAAACCAA
AACCTGTCATCAGCAACAGTTGACACGCTACTTTTGACATTGTTGAGGAAACTTGACAAA
GAAAAGGAGGAA------------------CCT------TGG---GCGAAAATTCCGTTC
CAAGCTGAAGTGAGAACTTTGGAATTTTGGAGATCTGTAGTTGCAGAATGTGTTGGAACA
TTTTTTCTTGTGTTTCTAACCTGTGGAGCGTTTGTACCATGGGCTGGG---------CAT
ACTCCCCCGCCTATTGGAATTGCCCTGACTGTTGGATTCGGGGTTGTGATGTTAACACAC
TGCTTTGCGCTCATTTCAGGTGCCAATTTTAATCCTGTGGTGACCCTTGCGCTTGTGGTC
AAAAGAAGTATGTCACCGTTTCGTGGGGCCCTTTATTTCACTGCCCAAGTCGGAGGATCC
ATCGCCGGATCAGCCTTGCTCTATGGGGTGACGTCATCCGGATCTCAA---GGGAATCTG
GGA---------------------------------------------------------
------------------GTAACTACACTTGCTGCCGGTTTGAAGGACTGGCAAGGCTTT
GCCATCGAACTCTGCTTCAGTTTCATCATCGTGCTGACTTACCTTTGTACCCTCCACCCA
GAGCAAAAGCGGACTGTGTCTACATCTATGGGAACAGGTCCTATCACTGTGGGTTTCGCC
TAT---TTGGCTTGCTCGCTCGTGGGGCTGCCATCTACTGGAGCTTCATTCAACCCCGTT
CGGTCGTTGGGTCCAGCTTTTGTCATG---------------------------------
AAC---AAATGGGATAGCCACTGGGTTTACTGGGTGGGACCCCTAATTGGAGGAGCCTTC
GGAGGTCTTATCCAC---GAGTTTGTCTTTAATGCCTATCGGGAAGGA---------AGC
AGCAGGAAGTGCAATGGC---GACGGAGGTAGGTCTTATCTTGTTAGCGATGAAGAATGC
TACGAGGAGGGGCCAGCTGGG---CCCCCTGTCCGAGGGCCAGTTCCGGGAGCTCTCGCT
GTCCGTAGCCAGCATTACGATACGTATAAGCCGATCGCCATA------------------
---------------------GGTATGGAAGGC------ATGTGCAAATCAACACGAAAT
------------CTC---GACCAGACCGAAGGATTTACTCCGGACCCGCTCGCTCTAC
---TGC---CGATCGCCCGGT---CCTGCTCAGCGAAACATCCTCAACAGGTCCCAGTCC
GTCTACGCCAAGAATCCGGCGGTCGGTGGGCAAAACTCCCGCATCCATCCAGCTGCCAGC
ATA---ACGACAACGAAACCCGGAGGATTTGAAGCAATCAGCCGCCAAGAGAGCATTTAC
TCGAGCGGCAGATGTCAGCCGCAG------CCCATAAGGTCAGAGAGTATCTACGCTCCT
CGA---------------------------------AGCCAAATCCAGAAAGAGACCGTC
TTCTACTCCAAGCAGCAGAGCGAACACATCTACGCAACAAGGCCACCCCTTCCGTCTGAC
CCCGGCGTGTACCCACCT------------------CGCCAT------GAGACACCC---
AACTCCTTC---GCT---------CCCCAG---GGA---TACCAGGCCCCCCTTCCCCCC
ACGCCAGTCAACGAGGACCAGAAGCTTGAAGAAGAAAACATGCGAAATGCAGAAAGCACA
AAA---------------GGTGACTTTGAC------TACGGAAGAAATGAA---GATGAT
TACGCCATGCGGAGGAAATTCCAACCT---CGACATGAGTCCGTGTGCGGA------GTG
GTGCCGCCCGTCCGGCGGGACGACAGTGGAAAGGGGTTCTCCCCCAACAATTCTCATAGT
GACTCCGCCTATGGCTCCGTTAATACGCCATGCACGCCG---AATGGTAAC---CAAACC
TTTAACTACTCCCAAGGTGATCAGCAGAATCAAAATTACTCCCAAAATTACGAGCAATTT
AGGAGCAGTGAACAAGGGTTCGGCAATCAAGGCACCCTGGTTGTTGACGCTACGAGGGAG
TCGCTTTATGGT------------------------------------------------
------------TACTCG------------AAG------TCGAACCCGGGA---------
---CCGAAGAGATATCCG------------GTAGACAAGTCGCAAAATGTGCAGCCAGTT
CGGTAT
>GCBP01020129_Eubranchipus_grubii_Bib
------------------------------------------------------------
------------------------------------------------------------
------------------------------------------------------------
------------------------------------------------------------
------------------------------------------------------------

------------------------------------------------------------
------------------------------------------------------------
------------------------------------------------------------
------------------------------------------------------------
------------------------------------------------------------
------------------------------------------------------------
------------------------------------------------------------
------------------------------------------------------------
------------------------------------------------------------
------------------------------------------------------------
------------------------------------------------------------
------------------------------------------------------------
------------------------------------------------------------
------------------------------------------------------------
------------------------------------------------------------
------------------------------------------------------------
------------------------------------------------------------
------------------------------------------------------------
------------------------------------------------------------
------------------------------------------------------------
------------------------------------AGGTCGGAGGCCATTTACGTCCCA
CGT---------------------------------ATGCATCTACAGAAGGAGGCCGTC
TATTTCTCAAAGACACAAACGGAAAATATTTACTCGACAAGGCCTCCACTCCCGTCAGAC
CCGGGT---TATCCCTCT------------------CGCAGT------GAAACTAAC---
TACCCGTGTCGCCAA---------GATCAA---GGA---TACCAGGTCCCACTACCTCCA
ACCCCCATCAACGAAGACCAGAAACTCGAAGAAGAAAATATGCGAAACGCTGAGTGTACA
AAA---------ACGCAGGGTGAGTTTGATCCA---TACGGAAGAAATGAG---GACGAC
TATGCTGCGCGGAGAAAATTTCAACCA---CGACAAGAGTCGGTG---------------
------------------------------------------------------------
------------------------------------------------------------
------------------------------------------------------------
------------------------------------------------------------
------------------------------------------------------------
------------------------------------------------------------
------------------------------------------------------------
------
>JAGQDJ010000046_Triops_longicaudatus_EglpL
------------------------------------------------------------
------------------------------------------ATGGGA------------
---------------------GGCTGGGACAGCATGAAGAAC---GCGTTCGGTTGCGGC
GAATTCAAC------AACGGTGAATTGTGGAAGTCCCTTTTGGCGGAGTTTATCGGCACC
TTATTTCTCGTGCTCGTCGGCTGTGCATCTTGTTCACAAGGTTGGAATGAA---AGCTAC
TCTCCAACTATGGTTCAAGTCGCCCTTTCCTTCGGTGTCACCATCGCTACTATGGCGCAG
GCGATTGGCCATGTCAGTGGTTGTCATATTAATCCCGCAGTCACTACGTCTTTGATGATA
GTGGGCAAGATTTCTCTGCTGAAAGCAATTTTCTATATCGCAGCCCAGTGTGTTGGAGCC
ATTTCAGGGGCTGCAATCTTACAGGCTCTGACACCTGCGGAGTTCCAC---AGTACATTA
GGT---------------------------------------------------------
------------------GTTACTGAACTACACGCCAAGTTGTCAGCCACTCAAGGCTTT

GGAGTCGAGTTTTTCGCCACTTTTACTCTTGTTCTGATCGTCTGTGGCGTTTGTGACGAA
AATCGCAATGATATTAAA------------GGTTCTGCTCCCTTGGCAATTGGCCTCACC
GTT---ACAACGCAGATTTTGGCTATTGGAATGTATACAGGAGGAAGCTTGAATCCAGCA
AGATCTTTAGGTCCGGCCGTGATCCTG---------------------------------
AAC---AAGTGGGACAGCCATTGGGTGTACTGGGTGGGTCCCATGGTGGGAGGTGCTGTA
GCTTCCCTGTTATAT---CAGAGAGCCTTCACAGCGCCGAGTAACAAGCGGGAG---CCT
GACGAGGAG------------GAACCGGACTACCCTTATCGGTACCGGGCGACG------
AACGATAAAGAGAAG------------------------------------GAAATAATC
GCAGATAGGACAACATCAATC---------------------------------------
------------------------------------------------------------
------------------------------------------------------------
------------------------------------------------------------
------------------------------------------------------------
------------------------------------------------------------
------------------------------------------------------------
------------------------------------------------------------
------------------------------------------------------------
------------------------------------------------------------
------------------------------------------------------------
------------------------------------------------------------
------------------------------------------------------------
------------------------------------------------------------
------------------------------------------------------------
------------------------------------------------------------
------------------------------------------------------------
------------------------------------------------------------
------------------------------------------------------------
------------------------------------------------------------
------------------------------------------------------------
------
>GEHY01012481_Triops_newberryi_EglpL
------------------------------------------------------------
------------------------------------------ATGGGA------------
---------------------GGCTGGGACAGCATGAAGAAC---GCGTTCGGTTGCGGC
GAATTCAAC------AACGGTGAATTGTGGAAGTCCCTTTTGGCGGAGTTTATCGGCACC
TTATTTCTCGTGCTCGTCGGCTGTGCATCTTGTTCACAAGGTTGGAATGAA---AGCTAT
TCTCCAACTATGGTTCAAGTCGCCCTTTCCTTCGGTGTCACCATCGCTACTATGGCGCAG
GCGATTGGCCATGTCAGTGGTTGTCATATTAATCCCGCAGTCACTACGTCCATGATGATA
GTGGGCAAGATTTCTCTGCTGAAAGCAATTTTCTATATTGCAGCCCAATGTGTTGGAGCC
ATTTCAGGGGCTGCAATCTTACAGGCTCTGACACCTGCAGAGTTCCAC---AGTACATTA
GGT---------------------------------------------------------
------------------GTTACTGAACTACACCCCAAGTTGTCAGCCACTCAAGGCTTT
GGAGTCGAGTTTTTCGCCACTTTTACTCTTGTTCTGATCGTCTGTGGCGTTTGTGACGAA
AATCGCAATGATATTAAA------------GGTTCTGCTCCCTTGGCAATTGGCCTCACC
GTT---ACAACGCAGATTTTGGCTATTGGAATGTATACAGGAGGAAGTTTGAACCCAGCA
AGATCTTTAGGTCCGGCCGTAATCTTA---------------------------------
AAC---AAGTGGGACAGCCATTGGGTGTACTGGGTGGGTCCCATGGTGGGAGGTGCTGTA
GCTTCCCTGTTATAC---CAGAGAGCCTTCACAGCGCCGAGTAACAAGCGGGAG---CCT

```
GACGAGGAG------------GAACCGGACTACCCTTATCGGTACCGGGCGACG------
AACGATAAAGAGAAG------------------------------------GAAATAATT
GCAGATAGGACAACATCAATC---------------------------------------
------------------------------------------------------------
------------------------------------------------------------
------------------------------------------------------------
------------------------------------------------------------
------------------------------------------------------------
------------------------------------------------------------
------------------------------------------------------------
------------------------------------------------------------
------------------------------------------------------------
------------------------------------------------------------
------------------------------------------------------------
------------------------------------------------------------
------------------------------------------------------------
------------------------------------------------------------
------------------------------------------------------------
------------------------------------------------------------
------------------------------------------------------------
------------------------------------------------------------
------------------------------------------------------------
------------------------------------------------------------
------
>AB293442_Triops_granarius_EglpL
------------------------------------------------------------
------------------------------------------------------------
------------------------------------------------------------
------------------------------------------------------------
------------------------------------------------------------
------------------------------------------------------------
------------------------------------------------------------
------------------------------------------------------------
---------------ATCTTACAGGCTCTGACACCTCCGGAGTTCCAC---AGTACATTA
GGT---------------------------------------------------------
------------------GTTACTGAGCTACACCCCAAGTTGTCGGCTACTCAAGGCTTC
GGAGTCGAGTTTTTCGCCACTTTTACTCTTGTTCTGATCGTCTGTGGCGTTTGTGACGAA
AATCGCAATGACATCAAA------------GGTTCTGCTCCCTTGGCAATTGGCCTCACC
GTT---ACAACGCAGATTTTGGCTATTGGAATGTATACAGGAGGAAGTTTGAATCCAGCA
AGAACTTTAGGCCCAGCCGTAATCCTG---------------------------------
AAC---AAGTGGGACAGCCATTGGGTTTACTGGGTGGGTCCAATGGTGGGAGGTGCTGTA
GCTTCCCTGTTATAC---CAGAGAGCCTTCACAGCGCCGAGTAACAAGCGGGAG---CCT
GACGAGGAG------------GAGCGAGACTACCCTTATCGGTACCGGGCGGCG------
AACGATAAAGAAAAG------------------------------------GAAATAATT
GCAGATAGGACCACATCAATC---------------------------------------
------------------------------------------------------------
------------------------------------------------------------
------------------------------------------------------------
```

------------------------------------------------------------
------------------------------------------------------------
------------------------------------------------------------
------------------------------------------------------------
------------------------------------------------------------
------------------------------------------------------------
------------------------------------------------------------
------------------------------------------------------------
------------------------------------------------------------
------------------------------------------------------------
------------------------------------------------------------
------------------------------------------------------------
------------------------------------------------------------
------------------------------------------------------------
------------------------------------------------------------
------------------------------------------------------------
------------------------------------------------------------
------
>GCAT01014384_Triops_cancriformis_EglpL
------------------------------------------------------------
------------------------------------------ATGGGA------------
----------------------GGCTGGACAGCATGAAGAAC---GCGTTCGGTTGCGGC
GAATTCAAC------AACGGGGAATTATGGAAGTCCCTTTTGGCGGAGTTTATCGGCACT
TTGTTTCTCGTGCTAGTCGGCTGTGCGTCGTGTTCACAAGGTTGGAATGAA---AGCTAT
TCCCCAACAATGGTTCAAGTCGCCCTTTCTTTCGGTGTCACTATTGCTACTATGGCGCAG
GCGATTGGCCATGTCAGTGGTTGCCATATTAATCCCGCCGTCACTACATCCTTGATGATA
GTGGGCAAGATTTCTCTACTGAAAGCACTTTTTTATATCGCAGCTCAATGTGTGGGGGCC
ATTTCGGGGGCTGCAATCTTACAGGCTCTGACACCTGCGGAGTTTCAC---AGTACATTA
GGT---------------------------------------------------------
------------------GTTACTGAACTACACCCCAAGTTGTCAGCCACTCAAGGCTTC
GGAGTCGAGTTTTTTGCCACGTTTACTCTTGTTTTGATCGTCTGTGGCGTTTGTGACGGA
AATCGCAATGATATCAAA------------GGTTCTGCTCCTTTGGCAATTGGCCTCACC
GTT---ACAACGCAAATTTTGGCCATTGGAATGTATACAGGAGGAAGTCTAAATCCAGCA
AGGTCTCTAGGTCCAGCCGTAATCCTG---------------------------------
AAC---AAGTGGGACAGCCATTGGGTGTACTGGGTGGGCCCAATGGTGGGAGGTGCTGTG
GCTTCCCTGTTGTAC---CAGAGAGCTTTCACAGCGCCGAGCAACAAGCGGGTG---CCC
GACGAGGAG------------GAACCAGACTACCCTTATCGGTACCGGGCGGCG------
AACGATAAAGAGAAG------------------------------------GAAATAATT
GCAGATAGGACTACATCAATC---------------------------------------
------------------------------------------------------------
------------------------------------------------------------
------------------------------------------------------------
------------------------------------------------------------
------------------------------------------------------------
------------------------------------------------------------
------------------------------------------------------------
------------------------------------------------------------
------------------------------------------------------------

```
------------------------------------------------------------
------------------------------------------------------------
------------------------------------------------------------
------------------------------------------------------------
------------------------------------------------------------
------------------------------------------------------------
------------------------------------------------------------
------------------------------------------------------------
------------------------------------------------------------
------------------------------------------------------------
------------------------------------------------------------
------
>RJJA01000114_Lepidurus_arcticus_EglpL1
------------------------------------------------------------
------------------------------------------ATGATTTGCTTGTTCAGG
GAAACGACAACGCAT------GCCTGGGCTGGTTTCAAAAGT---TCACTGCAATGTTGC
GACTTCAAC------AGCCATGAGCTATGGAAGTGCTTGCTAGCGGAATTCATTGGCACT
TTGTTTCTCGTGTTTTCTGGCTGTGCTGCATGTTCTGAAGGTTGGAACGAA---TCCTAC
TCGCCAACAATGGTTCAAGTGGCTCTTTGTTTTGGTATCACCGTCGCTACCATGGTGCAG
GCGATCGGTCATATAAGTAAATGCCATATCAACCCGGCGGTAACTACGTCGATGATGCTG
GTAGGCAAGATTACGGTGCTGAAAGGACTAGCTTATGTCATTGCACAATGCACAGGCGGT
ATCGCAGGAGTTGCTGTTTTGCAGATTTTGACACCTCCGGAATTTCGC---AGCACTTTA
GGC---------------------------------------------------------
------------------GTTACGGAACTGCACAACAAAGTGACACCATCGCAAGGGTTT
GGTATCGAATTTTTCGCTACTTTTGTGCTTGTGCTTATCATCTGCAGTGTATGTGACGAA
AATCGCACTGATGTTGGA------------GTATTAGCACCCTTAGCCATCGGCCTTACT
GTG---TCGTCACAAGTTTTAGCTATTGGAATGTATACTGGAGGTAGTATGAATCCAGCG
CGATCGTTAGGTCCTGCAGTGCTTCTT---------------------------------
AAC---AAATGGAGTTGCCATTGGGTGTATTGGGTTGGTCCCGTCATTGGCGGCGGAGTA
GCTGCATTAGTTTAC---CAACGGGCTTTTGCAGCTCCTAGC---AAA------------
------------------------------------------------------------
------------------------------------------------------------
------------------------------------------------------------
------------------------------------------------------------
------------------------------------------------------------
------------------------------------------------------------
------------------------------------------------------------
------------------------------------------------------------
------------------------------------------------------------
------------------------------------------------------------
------------------------------------------------------------
------------------------------------------------------------
------------------------------------------------------------
------------------------------------------------------------
------------------------------------------------------------
------------------------------------------------------------
------------------------------------------------------------
------------------------------------------------------------
```

------------------------------------------------------------
------------------------------------------------------------
------------------------------------------------------------
------------------------------------------------------------
------------------------------------------------------------
------
>JAGJRP010000017_Lepidurus_couesii_EglpL1
------------------------------------------------------------
------------------------ATGATCCAGAGGTCTAACATGATTTGCTTGTTCAGG
GAAACGACAACGTAT------GCCTGGGCTGGTTTCAAAAGT---TCACTGCAATGTTGC
GACTTCAAC------AGCCATGAGCTATGGAAGTGCTTGCTAGCGGAATTCATTGGCACT
TTGTTTCTCGTGTTTTCTGGCTGTGCTGCATGTTCTGAAGGTTGGAACGAA---TCCTAC
TCGCCAACAATGGTTCAAGTGGCTCTTTGTTTTGGTATCACCGTCGCTACCATGGTGCAG
GCGATCGGGCATATAAGTAAATGCCACATCAACCCGGCGGTAACTACGTCGATGATGCTG
GTAGGCAAGATTACGGTGGTGAAAGGACTAGCTTATGTCATTGCACAATGCACAGGCGGT
ATCGCAGGAGTTGCTGTTTTGCAGATTCTGACACCTCCGGAATTTCGC---AGCACTTTA
GGC---------------------------------------------------------
------------------GTTACGGAACTGCACAACAAAGTGACACCATCGCAAGGGTTT
GGTATCGAATTTTTCGCTACTTTTGTGCTTGTGCTTATCATCTGCAGTGTATGTGACGAA
AATCGCACTGATGTTGGA------------GTATTAGCACCCTTAGCCATCGGCCTTACT
GTG---TCGTCACAAATTTTGGCTATTGGAATGTATACTGGAGGTAGCATGAATCCAGCG
CGGTCGTTAGGTCCTGCAGTGATTCTT---------------------------------
AAC---GAATGGAGTTGCCATTGGGTGTATTGGGTTGGTCCCATCATTGGCGGCGGAGTA
GCTGCATTAGTTTAC---CAATGGGCTTTTGCAGCTCCTAGC---AAA------------
------------------------------------------------------------
------------------------------------------------------------
------------------------------------------------------------
------------------------------------------------------------
------------------------------------------------------------
------------------------------------------------------------
------------------------------------------------------------
------------------------------------------------------------
------------------------------------------------------------
------------------------------------------------------------
------------------------------------------------------------
------------------------------------------------------------
------------------------------------------------------------
------------------------------------------------------------
------------------------------------------------------------
------------------------------------------------------------
------------------------------------------------------------
------------------------------------------------------------
------------------------------------------------------------
------------------------------------------------------------
------------------------------------------------------------
------------------------------------------------------------
------------------------------------------------------------
------

>JAKZAN010000001_Lepidurus_packardi_EglpL1
------------------------------------------------------------
------------------------ATGATCCAA---TCTAACATGATTTGCTTGTTCAGA
GAAACGACAACGTAT------TCCTGGGCTGGTTTCAAAAAT---TCACTACGATGTTGC
GACTTCAAC------AGCCATGAGCTATGGAAGTGCTTGCTAGCAGAATTCATTGGCACT
TTGTTTCTCGTGTTTTCTGGCTGTGCTGCATGTTCTGAAGGTTGGAACGAA---TCCTAC
TCGCCAACAATGGTTCAAGTGGCTCTTTGTTTTGGTATCACCGTCGCTACCATGGCGCAG
GCGATTGGTCATATAAGTGGATGCCATATCAACCCGGCGGTAACTACGTCGATGATGCTG
GTAGGCAAGATTACGGTGCTGAAAGGACTAGCTTATGTTATTGCACAATGCACAGGTGGT
ATCGCAGGAGTTGCTGTTTTGCAGATTTTGACACCTCCGGAATTTCGC---AGCACTTTA
GGC---------------------------------------------------------
------------------TTTACGGAGCTGCACAACAAAGTGACACCATCGCAGGGGTTT
GGTATCGAATTTTTCGCTACGTTTATGCTTGTGCTAATCATCTGCAGTGTATGTGACGAA
AATCGCACGGATATTGGA------------GGATTAGCCCCCTTAGCCATAGGCTTTACT
GTG---TCGTCACAGGTTTTGGCTATTGGAATGTATACTGGAGGTAGCATGAATCCAGCG
CGATCGTTAGGTCCTGCAGTGATTCTT---------------------------------
AAC---AAGTGGAGTTGCCATTGGGTGTATTGGGTTGGTCCCGTCATTGGCGGTGGTGCA
TCTGCATTAGTTTAC---CAACGGCTTTTGCAGCTCCTAGC---AAA-------------
------------------------------------------------------------
------------------------------------------------------------
------------------------------------------------------------
------------------------------------------------------------
------------------------------------------------------------
------------------------------------------------------------
------------------------------------------------------------
------------------------------------------------------------
------------------------------------------------------------
------------------------------------------------------------
------------------------------------------------------------
------------------------------------------------------------
------------------------------------------------------------
------------------------------------------------------------
------------------------------------------------------------
------------------------------------------------------------
------------------------------------------------------------
------------------------------------------------------------
------------------------------------------------------------
------------------------------------------------------------
------------------------------------------------------------
------------------------------------------------------------
------------------------------------------------------------
------
>RJJB01000085_Lepidurus_apus_EglpL1
------------------------------------------------------------
------------------------ATGCTCCAG---TCTACCATGATTTGCTTGTTCAGA
GAAACGACAACGTAC------TCCTGGGCTGGTTTCAAAAGT---TCACTGCGATGTTGC
GACTTCAAC------AGCCATGAGCTATGGAAGTGCCTGCTAGCAGAATTCATCGGCACT
TTGTTTCTCGTGTTTTGTGGCTGTGCCGCATGTTCTGAAGGTTGGAACGAA---TCCTAT

TCGCCAACAATGGTTCAAGTGGCTCTTTGTTTTGGTATCACCATCGCTACCATGGCGCAG
GCGATTGGTCATATTAGTGGATGCCATATCAACCCGGCGGTAACTACGTCGATGATGGTG
GCAGGCAAGATTGCGGTACTGAAAGGACTAGCTTATATCATTGCCCAATGCACAGGTGCT
ATCGCAGGAGCTGCTGTTTTGCAGATTTTGACACCTCCGGAATTCCGC---AGCACTTTA
GGC---------------------------------------------------------
------------------GTTACGGAGCTGCACAGCAAAGTGACACCATCGCAAGGGTTT
GGTATCGAATTTTTCGCTACTTTTGTGCTTGTGCTTATCATCTGCAGTGTATGTGACGAA
AATCGCACTGATATTGGA------------GGATTAGCACCCTTAGCAATCGGACTTACT
GTG---TCGTCACAGGTTCTGGCTATTGGAATGTATACTGGAGGAAGCATGAATCCAGCG
CGATCGTTAGGTCCTGCAGTGATTCTT---------------------------------
AAC---AATTGGAGTTGCCATTGGGTGTACTGGGTTGGTCCCATCATTGGTGGTGGCGTA
GCTGCCCTAGTGTAC---CAACGGGCTTTTACTGCCCCTAGT---AAA------------
------------------------------------------------------------
------------------------------------------------------------
------------------------------------------------------------
------------------------------------------------------------
------------------------------------------------------------
------------------------------------------------------------
------------------------------------------------------------
------------------------------------------------------------
------------------------------------------------------------
------------------------------------------------------------
------------------------------------------------------------
------------------------------------------------------------
------------------------------------------------------------
------------------------------------------------------------
------------------------------------------------------------
------------------------------------------------------------
------------------------------------------------------------
------------------------------------------------------------
------------------------------------------------------------
------------------------------------------------------------
------------------------------------------------------------
------------------------------------------------------------
------------------------------------------------------------
------
>RJJB01000085_Lepidurus_apus_EglpL2
------------------------------------------------------------
------------------------------------------ATGGGA------------
----------------------GGCTGGATAGTATGAAGAAC---GTGTTTGGCTGCGGC
GAGTTCAAC------AACGGTGAAGTGTGGAAATCCCTATTGGCGGAATTCATTGGCACT
TTGTTTCTCGTACTCGTTGGTTGTGCGTCGTGTTCACAAGGCTGGAATGAA---TCTTAT
TCGCCAACAATGGTTCAAGTGGCCCTTTCCTTCGGTGTCACCATTGCCACAATGGCACAG
TCGATTGGTCACGTCAGCGGATGTCATATCAATCCCGCAGTCACAACATCGATGATGATG
GTTGGCAAGATTTCTGTGCTGAAGGCTCTTTTCTACATCGCAGCCCAATGCGTTGGAGCT
ATTTCTGGAGCTGCTATTTTGCAGGCCCTGACACCTGTGGAGTTCCAC---AGTACATTA
GGC---------------------------------------------------------
------------------GTTACAGAGTTACATCCCAAGTTATCAGCCACCCAGGGTTTT

GGTGTCGAGTTTTTTGCCACTTTCACGCTGGTCCTCATCGTCAGTGGCGTTTGTGATGAA
AATCGCAATGATATCAAA------------GGATCCGCTCCCTTGGCCATCGGTCTCACC
GTT---ACCACGCAAATTCTAGCTATTGGAATGTACACAGGAGGAAGCCTGAATCCCGCA
CGGTCCTTGGGTCCCGCCGTAATACTC---------------------------------
AAC---AAATGGGACAACCATTGGGTGTACTGGGTGGGTCCCATCGTGGGCGGAGCTGTA
GCTGCCTTGTTATAC---CAACGGGCTTTCACCGCACCGAGCCATAAGCGAGAG---CCC
GACGAGGAA------------GAACCAGATTATCCTTATCGTTATCGGGCTGCA------
AACGAAAAAGAGAAG------------------------------------GAAGTAATT
GCGGACAGGACCACGTCAATC---------------------------------------
------------------------------------------------------------
------------------------------------------------------------
------------------------------------------------------------
------------------------------------------------------------
------------------------------------------------------------
------------------------------------------------------------
------------------------------------------------------------
------------------------------------------------------------
------------------------------------------------------------
------------------------------------------------------------
------------------------------------------------------------
------------------------------------------------------------
------------------------------------------------------------
------------------------------------------------------------
------------------------------------------------------------
------------------------------------------------------------
------------------------------------------------------------
------------------------------------------------------------
------------------------------------------------------------
------------------------------------------------------------
------
>JAKZAN010000001_Lepidurus_packardi_EglpL2
------------------------------------------------------------
------------------------------------------ATGGGT------------
---------------------GGCTGGGATAGTATGAAGAAC---GTGTTTGGCTGCGGC
GAGTTCAAT------AATGGTGAAGTGTGGAAATCCCTATTGGCGGAATTCATTGGCACG
TTGTTTCTCGTACTCATTGGTTGTGCGTCATGTTCACAAGGTTGGAATGAA---TCTTAT
TCGCCAACAATGGTTCAAGTGGCCCTTTCCTTCGGTGTCACCATTGCCACAATGGCACAG
TCGATTGGCCACGTCAGCGGATGTCATATCAATCCCGCAGTTACAACATCGATGATGATG
GTTGGCAAGATCTCTGTGTTGAAGGCTCTTTTCTACATCGCAGCCCAATGTGTTGGAGCT
ATTGCCGGAGCTGCTATTTTGCAGGCCCTGACACCTGTGGAGTTCCAC---AGTACATTA
GGC---------------------------------------------------------
------------------GTTACAGAATTACATCCCAAGTTATCAGCCACCCAAGGTTTT
GGTGTCGAGTTTTTTGCCACTTTCACGCTGGTCCTCATCGTCAATGGCGTTTGCGATGAA
AATCGCAATGATATCAAA------------GGTTCCGCTCCCTTGGCCATCGGTCTCACC
GTT---ACCACGCAAATTCTGGCTATTGGAATGTACACAGGAGGAAGCCTGAATCCTGCA
CGGTCCTTGGGTCCAGCCGTAATACTC---------------------------------
AAC---AAATGGGATAATCATTGGGTGTACTGGGTGGGTCCCATCGTGGGCGGTGCTGTA
GCTGCCCTGTTATAC---CAACGGGCTTTCACCGCGCCGAGCCATAAGCGAGAG---CCT

GACGAGGAA------------GAACCAGATTATCCTTATCGTTATCGGGCTGCA------
AACGAAAAAGAGAAG------------------------------------GAAGTAATA
GCGGACAGGACCACGTCAATC---------------------------------------
------------------------------------------------------------
------------------------------------------------------------
------------------------------------------------------------
------------------------------------------------------------
------------------------------------------------------------
------------------------------------------------------------
------------------------------------------------------------
------------------------------------------------------------
------------------------------------------------------------
------------------------------------------------------------
------------------------------------------------------------
------------------------------------------------------------
------------------------------------------------------------
------------------------------------------------------------
------------------------------------------------------------
------------------------------------------------------------
------------------------------------------------------------
------------------------------------------------------------
------------------------------------------------------------
------------------------------------------------------------
------
>RJJA01000114_Lepidurus_arcticus_EglpL2
------------------------------------------------------------
------------------------------------------ATGGGA------------
---------------------GGCTGGGATAGTATGAAGAAC---GTGTTTGGCTGCGGC
GAGTTCAAC------AACGGTGAAGTGTGGAAATCCCTATTGGCGGAATTCATTGGCACT
TTGTTCCTCGTACTCGTTGGTTGTGCGTCGTGTTCACAAGGTTGGAATGAA---TCTTAT
TCGCCAACAATGGTTCAAGTGGCCCTTTCCTTCGGTGTCACCATTGCCACAATGGCGCAG
TCGATTGGCCACGTCAGCGGATGTCATATCAATCCCGCGGTCACAACGTCGATGATGATG
GTTGGCAAGATCTCTGTGCTGAAGGCTCTTTTCTACATCGCAGCTCAATGTGTTGGAGCT
ATTGCCGGAGCTGCTATTTTGCAGGCCCTGACACCTGTGGAGTTCCAC---AGTACATTA
GGC---------------------------------------------------------
------------------GTTACAGAATTACATCCCAAGTTATCAGCCACCCAAGGTTTT
GGTGTCGAGTTTTTTGCCACTTTCACGCTGGTCCTCATCGTCAGTGGCGTTTGTGATGAA
AATCGCAATGATATCAAA------------GGCTCCGCTCCCTTGGCCATCGGTCTCACC
GTT---ACCACGCAAATTCTGGCTATTGGAATGTACACAGGAGGAAGCCTGAATCCTGCA
CGGTCCTTGGGTCCCGCCGTAATACTC---------------------------------
AAC---AAATGGGACAACCATTGGGTGTACTGGGTGGGTCCCATCGTGGGCGGTGCTGTA
GCTGCCCTGTTATAT---CAGCGGGCTTTCACGGCGCCGAGCCATAAGAGAGAG---CCT
AACGAGGAA------------GAACCAGATTATCCTTATCGTTATCGGGCCGCA------
AACGAAAAGGAGAAG------------------------------------GAAGTAATT
GCGGACAGGACCACGTCAATC---------------------------------------
------------------------------------------------------------
------------------------------------------------------------
------------------------------------------------------------

------------------------------------------------------------
------------------------------------------------------------
------------------------------------------------------------
------------------------------------------------------------
------------------------------------------------------------
------------------------------------------------------------
------------------------------------------------------------
------------------------------------------------------------
------------------------------------------------------------
------------------------------------------------------------
------------------------------------------------------------
------------------------------------------------------------
------------------------------------------------------------
------------------------------------------------------------
------------------------------------------------------------
------------------------------------------------------------
------------------------------------------------------------
------
>JAGJRP010000017_Lepidurus_couesii_EglpL2
------------------------------------------------------------
------------------------------------------ATGGGA------------
----------------------GGCTGGATAGTATGAAGAAC---GTGTTTGGCTGCGGC
GAGTTCAAC------AACGGTGAAGTGTGGAAATCCCTATTGGCGGAATTCATTGGCACT
TTGTTTCTCGTACTCGTTGGCTGTGCGTCGTGTTCACAAGGTTGGAATGAA---TCTTAT
TCGCCAACAATGGTTCAAGTGGCCCTTTCTTTCGGTGTCACCATTGCCACAATGGCGCAG
TCGATTGGCCACGTCAGCGGATGTCATATCAATCCCGCAGTCACAACGTCGATGATGATG
GTTGGCAAGATCTCTGTGCTGAAGGCTCTTTTCTACATCGCAGCTCAATGTGTTGGAGCT
ATTGCCGGAGCTGCTATTTTGCAGGCCCTGACACCTGTGGAGTTCCAC---AGTACATTA
GGC---------------------------------------------------------
------------------GTTACAGAATTACATCCCAAGTTATCAGCCACCCAAGGTTTT
GGTGTCGAGTTTTTTGCCACTTTCACGCTGGTCCTCATCGTCAGTGGCGTTTGCGATGAA
AATCGCAATGATATCAAA------------GGCTCCGCTCCCTTGGCCATCGGTCTCACC
GTT---ACCACGCAAATTCTGGCTATTGGAATGTACACAGGAGGAAGCCTAAATCCTGCA
CGGTCCTTGGGTCCGGCCGTAATACTC---------------------------------
AAC---AAATGGGACAACCATTGGGTGTACTGGGTGGGTCCCATCGTGGGCGGTGCTGTA
GCTGCCCTGTTATAC---CAACGGGCTTTCACGGCGCCGAGCCATAAGCGAGAA---CCT
GACGAGGAA------------GAACCAGATTATCCTTATCGTTATCGGGCCGCA------
AACGAAAAAGAGAAG------------------------------------GAAGTAATT
GCGGACAGGACCACGTCAATC---------------------------------------
------------------------------------------------------------
------------------------------------------------------------
------------------------------------------------------------
------------------------------------------------------------
------------------------------------------------------------
------------------------------------------------------------
------------------------------------------------------------
------------------------------------------------------------
------------------------------------------------------------

------------------------------------------------------------
------------------------------------------------------------
------------------------------------------------------------
------------------------------------------------------------
------------------------------------------------------------
------------------------------------------------------------
------------------------------------------------------------
------------------------------------------------------------
------------------------------------------------------------
------------------------------------------------------------
------------------------------------------------------------
------
>JAQQPU010002184_010005704_010037015_Artemia_sp_Kazakhstan_EglpL
------------------------------------------------------------
------------------------------------------ATGGGCTGCTTCAGT---
------------GGT------TTGTGGGAGCGGGTGAAAAAA---ATGGCCAAGTTCTCA
AAAATGATGGGA---AGT---AACATTTGGCAAGCTCTTTTTGCTGAAGCAATGGGTACC
ATGCTTTTAGTTTATGTAGGATGTGGCTCTGCATTGCCAATACATGGACCA---------
---CCGAACCCAGTGGCAATCTCTTTAGCTTTTGGGCTGACTGTGGCAACACTAGTTCAG
GGTATTGGACATGTGAGCGGTTGTCACATAAATCCTGCTGTAACTGCAGCTATGGCAGTT
ACTGGAAAAATACCATTTCTCCGTGCAGCTCTCTATGCTGTAGCACAATGCATCGGTGCA
ATTGCTGGATCAGCTTGCCTTGAGTGGCTAACACCAGAGCAGTTCCGT---GGCACTTTG
GGT---------------------------------------------------------
------------------GCAACTACCGTCAGTGAACATGTAACACCATCTCAAGCACTT
GGCATTGAGTTCTTTTCAACTTTTATTTTATTGTTTTTCATTTTCGCAGTCACTGATGAA
CGAAAAACGGATATTCTC------------GGATCTCCTGCATTGGCTATTGGTTTTGTA
GTC---ACAGCTCAAGCCCTGGCTTTTGGTTCCTATGCTGGATGTAGTATGAATCCTGCA
AGAAGTCTTGGTCCTGCAGTTCTGATT---------------------------------
GGA---GACTTCAGGCTGCACTGGGTATATTGGGTTGGACCCATTTTTGGAGCTATAGTA
GCAGGTCTTGTTTAT---GAGAAGTTCTTCAGAGCAGAATTATATTCTGAACCA---GCA
AATGACGAGATA---------TTTGAAACTAAGAAGTATCATTTTCAGGTAATA------
---TCACCAAGCAGTCTTGTTAATGATATAGAA---------AAA------GAATTAATT
GTAGAAACAGATAAA---------------------------------------------
------------------------------------------------------------
------------------------------------------------------------
------------------------------------------------------------
------------------------------------------------------------
------------------------------------------------------------
------------------------------------------------------------
------------------------------------------------------------
------------------------------------------------------------
------------------------------------------------------------
------------------------------------------------------------
------------------------------------------------------------
------------------------------------------------------------
------------------------------------------------------------
------------------------------------------------------------
------------------------------------------------------------

------------------------------------------------------------
------------------------------------------------------------
------------------------------------------------------------
------------------------------------------------------------
------------------------------------------------------------
------
>CM051267_Artemia_sinica_EglpL
------------------------------------------------------------
------------------------------------------ATGGGCTGCTTCAGT---
------------GGT------TTGTGGGAGCGGGTGAAAAAA---ATGGCCAAGTTCTCA
AAAATGATGGGA---AGT---AACATTTGGCAAGCTCTTTTTGCTGAAGCAATGGGTACC
ATGCTTTTAGTTTATGTAGGATGTGGCTCTGCATTGCCAATACATGGACCA---------
---CCGAACCCAGTGGCAATCTCTTTAGTTTTTGGGCTGACTGTGGCAACACTTGTTCAG
GGTATTGGACATGTGAGCGGTTGTCACATAAATCCTGCTGTAACTGCAGCTATGGCAGTT
ACTGGAAAAATACCATTTCTCCGTGCAGCTCTCTATGCTGTAGCGCAATGCATCGGTGCA
ATTGCTGGATCAGCTTGCCTTGAGTGGCTAACACCAGAGCAGTTCCGT---GGCACTTTG
GGT---------------------------------------------------------
------------------GCAACTACCGTCAGTGAACATGTAACACCATCTCAAGCACTT
GGCATTGAGTTCTTTTCAACTTTTATTTTATTGTTTTTCATTTTCGCAGTCACTGATGAA
CGAAAAACGGATATCCTC------------GGATCTCCTGCATTGGCTATTGGTTTTGTA
GTC---ACAGCTCAAGCCCTGGCTTTTGGTTCCTATGCTGGATGTAGTATGAATCCTGCA
AGAAGTCTTGGTCCTGCAGTTCTGATT---------------------------------
GGA---GACTTCAGGCTGCACTGGGTATATTGGGTTGGACCGATTTTTGGAGCTATAGTA
GCAGGTCTTGTCTAT---GAGAAGTTCTTCAGAGCAGAATTATATGCTGAACCA---GCA
AATGACGAAATA---------TTTGAAACTAAGAAGTATCATTTTCAG------------
---TCACCAAGCAGTCTTGTTAATGATATAGAA---------AAA------GAATTAATT
GTAGAAACAGATAAA---------------------------------------------
------------------------------------------------------------
------------------------------------------------------------
------------------------------------------------------------
------------------------------------------------------------
------------------------------------------------------------
------------------------------------------------------------
------------------------------------------------------------
------------------------------------------------------------
------------------------------------------------------------
------------------------------------------------------------
------------------------------------------------------------
------------------------------------------------------------
------------------------------------------------------------
------------------------------------------------------------
------------------------------------------------------------
------------------------------------------------------------
------------------------------------------------------------
------------------------------------------------------------
------------------------------------------------------------
------------------------------------------------------------
------

>JAVRJZ010000004_Artemia_franciscana_Jo_EglpL
------------------------------------------------------------
------------------------------------------ATGGGCTGCTTCAGT---
------------GGT------TTGTGGGAGCGGGTGAAAAAA---ATGGCCAAGTTCTCA
AAAATGATGGGA---AGT---AACATTTGGCAAGCTCTTTTCGCTGAAGCAATGGGTACC
ATGCTTTTAGTTTATGTAGGATGTGGCTCTGCATTGCCAATACATGGACCA---------
---CCTAACCCAGTGGCAATCTCTTTAGTTTTTGGGTTGACTGTGGCAACACTAGTTCAG
GGTATTGGACATGTGAGCGGTTGTCACATAAATCCTGCTGTAACTGCAGCCATGGCAGTA
ACTGGAAAAATGCCATTTCTCCGTGCAGCTCTCTATGTGGTAGCACAGTGCATCGGTGCA
ATTGCTGGATCAGCTTGCCTTGAGTGGCTAACACCAGAGCAGTTCCGT---GGAACTTTG
GGC---------------------------------------------------------
------------------GCAACTACTGTCAGTGAACATGTAACGCCATCTCAAGCACTT
GGCATTGAGTTTTTTTCAACTTTTATTTTATTGTTTTTCATTTTCGCAGTCACGGATGAA
CGAAAAACGGATATCCTC------------GGATCCCCTGCATTGGCTATTGGTTTTGTA
GTC---ACAGCTCAAGCCCTGGCTTTTGGTTCTTATGCTGGTTGTAGTATGAATCCTGCG
AGAAGTCTTGGTCCTGCAGTTCTGATT---------------------------------
GGA---GACTTCAGGCTGCACTGGGTATATTGGGTTGGGCCGATTTTTGGAGCTATAGTA
GCAGGTTTCGTTTAT---GAGAAGTTCTTCAGAGCAGAATTATATGCTGAACGA---GCA
AATGACGAGATA---------TTTGAAACTAAGAAGTATCATTTTCAG------------
AAATCTCCAAGCAGTCTCGTTAATGATATAGAA---------AAA------GAACTAATT
GTGGAAACAGATAAA---------------------------------------------
------------------------------------------------------------
------------------------------------------------------------
------------------------------------------------------------
------------------------------------------------------------
------------------------------------------------------------
------------------------------------------------------------
------------------------------------------------------------
------------------------------------------------------------
------------------------------------------------------------
------------------------------------------------------------
------------------------------------------------------------
------------------------------------------------------------
------------------------------------------------------------
------------------------------------------------------------
------------------------------------------------------------
------------------------------------------------------------
------------------------------------------------------------
------------------------------------------------------------
------------------------------------------------------------
------------------------------------------------------------
------
>PQ469250_Artemia_franciscana_EglpL
------------------------------------------------------------
------------------------------------------ATGGGCTGCTTCAGT---
------------GGT------TTGTGGGAGCGGGTGAAAAAA---ATGGCCAAGTTCTCA
AAAATGATGGGA---AGT---AACATTTGGCAAGCTCTTTTCGCTGAAGCAATGGGTACC
ATGCTTCTAGTTTATGTAGGATGTGGCTCTGCATTGCCAATACATGGACCA---------

---CCTAACCCAGTGGCAATCTCTTTAGTTTTTGGGTTGACTGTGGCAACACTAGTTCAG
GGTATTGGACATGTGAGTGGTTGTCACATAAATCCTGCTGTAACTGCAGCCATGGCAGTA
ACTGGAAAAATGCCATTTCTCCGTGCAGCTCTCTATGTGGTAGCACAGTGCATCGGTGCA
ATTGCTGGATCAGCTTGCCTTGAGTGGCTAACACCAGAGCAGTTCCGT---GGAACTTTG
GGC---------------------------------------------------------
------------------GCAACTACTGTCAGTGAGCATGTAACGCCATCTCAAGCACTT
GGCATTGAGTTTTTTTCAACTTTTATTTTATTGTTTTTCATTTTCGCAGTCACGGATGAA
CGAAAAACGGATATCCTC------------GGATCCCCTGCATTGGCTATTGGTTTTGTA
GTC---ACAGCTCAAGCCCTGGCTTTTGGTTCTTATGCTGGTTGTAGTATGAATCCTGCG
AGAAGTCTTGGTCCTGCAGTTCTGATT---------------------------------
GGA---GACTTCAGGCTGCACTGGGTATATTGGGTTGGGCCGATTTTTGGAGCTATAGTA
GCAGGTTTCGTTTAT---GAGAAGTTCTTCAGAGCAGAATTATATGCTGAACGA---GCA
AATGACGAGATA---------TTTGAAACTAAGAAGTATCATTTTCAG------------
AAATCTCCAAGCAGTCTCGTTAATGATATAGAA---------AAA------GAACTAATT
GTGGAAACAGATAAA---------------------------------------------
------------------------------------------------------------
------------------------------------------------------------
------------------------------------------------------------
------------------------------------------------------------
------------------------------------------------------------
------------------------------------------------------------
------------------------------------------------------------
------------------------------------------------------------
------------------------------------------------------------
------------------------------------------------------------
------------------------------------------------------------
------------------------------------------------------------
------------------------------------------------------------
------------------------------------------------------------
------------------------------------------------------------
------------------------------------------------------------
------------------------------------------------------------
------------------------------------------------------------
------------------------------------------------------------
------------------------------------------------------------
------
>JAYKOS010000014_Artemia_franciscana_Bett_EglpL
------------------------------------------------------------
------------------------------------------ATGGGCTGCTTCAGT---
------------GGT------TTGTGGGAGCGGGTGAAAAAA---ATGGCCAAGTTCTCA
AAAATGATGGGA---AGT---AACATTTGGCAAGCTCTTTTCGCTGAAGCAATGGGTACC
ATGCTTTTAGTTTATGTAGGATGTGGCTCTGCATTGCCAATACATGGACCA---------
---CCTAACCCAGTGGCAATCTCTTTAGTTTTTGGGTTGACTGTGGCAACACTAGTTCAG
GGTATTGGACATGTGAGTGGTTGTCACATAAATCCTGCTGTAACTGCAGCCATGGCAGTA
ACTGGAAAAATACCATTTCTCCGTGCAGCTCTCTATGTGGCAGCACAGTGCCTTGGTGCA
ATTGCTGGATCAGCTTGCCTTGAGTGGCTAACACCAGAGCAGTTCCGT---GGCACTTTG
GGC---------------------------------------------------------
------------------GCAACAACTGTCAGTGAACATGTAACGCCATCTCAAGCACTT

GGCATTGAGTTTTTTTCAACTTTTATTTTATTGTTTTTCATTTTCGCAGTCACGGATGAA
CGAAAAACGGATATCCTC------------GGATCCCCTGCATTGGCTATTGGTTTTGTA
GTC---ACAACTCAAGCCCTGGCTTTTGGTTCTTATGCTGGTTGTAGTATGAATCCTGCG
AGAAGTCTTGGTCCTGCAGTTCTGATT---------------------------------
GGA---GACTTCAGGCTGCACTGGGTATATTGGGTTGGGCCGATTTTTGGAGCTATAGTA
GCAGGTTTCGTTTAT---GAGAAGTTCTTCAGAGCAGAATTATATGCTGAACGA---GCA
AATGACGAGATA---------TTTGAAACTAAGAAGTATCATTTTCAG------------
AAATCTCCAGGCAGTCTTGATAATGATATAGAA---------AAA------GAATTAATT
GTGGAAACAGATAAA---------------------------------------------
------------------------------------------------------------
------------------------------------------------------------
------------------------------------------------------------
------------------------------------------------------------
------------------------------------------------------------
------------------------------------------------------------
------------------------------------------------------------
------------------------------------------------------------
------------------------------------------------------------
------------------------------------------------------------
------------------------------------------------------------
------------------------------------------------------------
------------------------------------------------------------
------------------------------------------------------------
------------------------------------------------------------
------------------------------------------------------------
------------------------------------------------------------
------------------------------------------------------------
------------------------------------------------------------
------------------------------------------------------------
------
>JAKZAO010000012_Branchinecta_lindahli_EglpL1_v1
------------------------------------------------------------
------------------------------------------ATGGGTTGTACTTGTTGT
AGTGCTTGGTGGGGAAGA---CAGTGTGACAGAATGAAGGAT---ACACTTGGTTGTTCC
GAGATTGCTGGGGAAAATAGGCACATTTGGAGGTGTGCCCTAGCGGAGGCGATTGGAACT
TTGTTTCTTGTTCTTGTCGGCTGCGGGTCGTGTTTGTCGATTCATGGTGAA---------
---GCCAACTCCGTTGCTGTTGCGGTGTCCTTTGGACTTGCACTGGCTACAATGGCTCAG
TCAATTGGCCACGTCAGCGGGTGTCACATCAACCCAGCCGTAACGGCCGGATTTTTGATC
ACCGGAAAAATCACGATTGTCCGGGCAATTCTTTATGTTGCCGCGCAGTGCATCGGAGCT
ACTGCCGGATCAGCAGCACTTGAGTGCCTCACGCCTCCAAAGTTCCAC---GGATCACTG
GGA---------------------------------------------------------
------------------GTCACCGCAATCAACGCTGATGTCTCTCCATCGCAAGCGTTT
GGAGTGGAATTTTTTGCGACCTTCGTCCTCATCTTCGTTATTTCGCCGTCTGCGATGAA
AACAGAACTGACGTCAAG------------GGATCGGCCCCGATGGCCATTGGCCTTACC
GTC---ACCATGATGGCTCTTGCTTTTGGCTCCTACACTGGCTGCAGTATGAACCCCGCC
AGAAGTCTTGGACCTGCCATCATCACA---------------------------------
GGA---AGATTTGAACATCACTGGGTTTACTGGGTTGGACCCATCCTAGGTGGAATCGTT
GCAGCCCTTATCTAC---CAATCCACTTTCAGAGTCCGGCCAGAACCCCTGCCA---CTC

GAAGACGAAGAA---------ACCGGTGGCAAGATTTACCACTTTAGGGTAAGGTCCTTA
AACAAGAAAAAGTTTCCGTTCCGTAAAAAGTCGTTTTTTTCTAAAACTATCGATTTGATG
AGCCAGTTGGCAAATACTCCCCCCCCCCCCCCCCAAAAAAAAAAAAAAAAACAGTTTGGC
------------------------------------------------------------
------------------------------------------------------------
------------------------------------------------------------
------------------------------------------------------------
------------------------------------------------------------
------------------------------------------------------------
------------------------------------------------------------
------------------------------------------------------------
------------------------------------------------------------
------------------------------------------------------------
------------------------------------------------------------
------------------------------------------------------------
------------------------------------------------------------
------------------------------------------------------------
------------------------------------------------------------
------------------------------------------------------------
------------------------------------------------------------
------------------------------------------------------------
------------------------------------------------------------
------------------------------------------------------------
------
>JAKZAP010000012_Branchinecta_lynchi_EglpL1_v1
------------------------------------------------------------
------------------------------------------ATGGGTTGTACTTGCTGT
AGTGGTTGGTGGGGAAGA---CAGTGTGCAAGAATGAAGGAT---ACACTTGGTTGTTCC
GAGATTGCTGGGGAAAATAGGCACATTTGGAGATGTGCTCTTGCGGAGGCGATTGGAACT
TTGTTCCTTGTGCTTGTGGGAAGCGGGTCGTGTTCGTCCATTCATGGTGAA---------
---CCCAACTCCCTTGCTGTTGCGCTGTCCTTCGGACTTGCGCTGGCTGCAATGGTTCAG
TCAATTGGACACGTCAGCGGGTGTCACATCAACCCAGCCGTAACGGGCGCATTTTTGATC
ACCGGAAAAATCACGATTGTCCGGGCAGTTCTTTACGTCGCCGCACAGTGCATCGGAGCT
ACTGCCGGATCAGCAGCCCTTCAGTGTCTGACGCCCCCGAAGTTTCAC---GGGTCACTG
GGA---------------------------------------------------------
------------------GTCACGGCTATCCACGCTGATGTCTCTCCGTCCCAAGCGTTT
GGGGTGGAATTTTTCGCCACCTTCGTCCTTATCCTCGTTATTTTTGCTGTCTGCGATGAA
AACAGAACTGACGTCAAA------------GGATCGGCACCGATGGCCATTGGCCTCACC
GTC---ACCATGATGGCTCTTGCTTTTGGCTCCTACACTGGCTGCAGTATGAACCCCGCC
AGAAGTCTTGGACCTGCCATCATCACA---------------------------------
GGA---AGATTCGAACATCACTGGGTTTACTGGGTTGGACCCATCCTAGGGGGAGTCGTT
GCAGCCCTTATCTAC---CAGTCCACTTTCAGAGTCCGGCCAGAACCCATGCCA---CAT
GAAGACGAAGAA---------ACCGGTGGCAATATATACCACTTTAAGGTAAGGTTCTCT
AACGAGAAAAAAGCTTCCGTGTCG------------------------------------
------------------------------------------------------------
------------------------------------------------------------
------------------------------------------------------------
------------------------------------------------------------

------------------------------------------------------------
------------------------------------------------------------
------------------------------------------------------------
------------------------------------------------------------
------------------------------------------------------------
------------------------------------------------------------
------------------------------------------------------------
------------------------------------------------------------
------------------------------------------------------------
------------------------------------------------------------
------------------------------------------------------------
------------------------------------------------------------
------------------------------------------------------------
------------------------------------------------------------
------------------------------------------------------------
------------------------------------------------------------
------------------------------------------------------------
------
>JAUPMV010000014_Branchinecta_sandiegonensis_EglpL1_v1
------------------------------------------------------------
------------------------------------------ATGGGTTGTACTTGCTGT
AGTGCTTGGTGGGGAAGA---CAGTGTGCAAGAATGAAGGAT---ACACTTGGTTGTTCC
GAGATTGCCGGGGAAAATAGGCACATTTGGAGATGTGCTCTTGCGGAGGCGATTGGAACT
TTGTTCCTTGTGCTTGTCGGCTGCGGGTCGTGTTTGTCGATTCATGGTGAA---------
---GCCAACCCCGTTGCTGTTGCGTTGTCCTTCGGACTAGCACTGGCTGCAATGGCTCAG
TCAATTGGCCACGTCAGCGGATGTCACATCAACCCAGCCGTAACGGCCGGATTTTTGATC
ACCGGAAAAATCACGATTGTCCGGGCAATTCTTTACGTCGCCGCACAGTGCATCGGAGCT
ACTGCCGGATCAGCAGCACTTGAGTGTCTGACACCCCCGAAGTTCCAC---GGGTCACTG
GGA---------------------------------------------------------
------------------GTCACCGCTATCAACGCTGATGTCTCTCCGTCGCAAGCGTTT
GGAGTGGAATTTTTCGCGACCTTCGTCCTCGTTCTGGTTATTTTCGCCGTCTGCGATGAG
AACAGAACTGACGTCAAA------------GGATCGGCCCCGATCGCCATTGGCCTCACC
GTC---ACCATGATGGCTCTTGCTTTTGGCTCCTACACTGGTTGCAGTATGAATCCCGCC
AGAAGTCTTGGACCTGCCATCATCACA---------------------------------
GGA---AGATTCGAACATCACTGGGTTTACTGGGTTGGACCTATCCTAGGTGGAATCGTT
GCAGCCCTTATCTAC---CAGTCCACTTTCCGAGTCCGACCAGAACCCCTGCCA---CAC
GAAGACGAAGAA----------ACCGGTGGCAAGATTTACCACTTTAGGTAAGGTTC---
------------------------------------------------------------
------------------------------------------------------------
------------------------------------------------------------
------------------------------------------------------------
------------------------------------------------------------
------------------------------------------------------------
------------------------------------------------------------
------------------------------------------------------------
------------------------------------------------------------
------------------------------------------------------------
------------------------------------------------------------

------------------------------------------------------------
------------------------------------------------------------
------------------------------------------------------------
------------------------------------------------------------
------------------------------------------------------------
------------------------------------------------------------
------------------------------------------------------------
------------------------------------------------------------
------------------------------------------------------------
------------------------------------------------------------
------------------------------------------------------------
------
>JAKZAO010000012_Branchinecta_lindahli_EglpL1_v2
------------------------------------------------------------
------------------------------------------ATGGGTGTTCCTTGC---
---------TTTGGCAAC---CTTTTTGAGAGAATGAAGGAC---GCCATGAGTTGTTCG
GAAATGTCTGGA---AAT---CAGATTTGGAAGATGTTGCTGTCCGAGGCCCTGGGTACT
ATGCTGCTTGTCTTTATAGGCTGTGCTTCGTGTTCCCCAATCCATGGAGAG---------
---CCAAATGCAGTGGGGATTTCCATTACGTTTGGATTGGCAGTGGCTGCACTTGCTCAG
TCAATTGGCCACGTCAGCGGGTGTCACATCAACCCAGCCGTAACGGCCGGATTTTTGATC
ACCGGAAAAATCACGATTGTCCGGGCAATTCTTTATGTTGCCGCGCAGTGCATCGGAGCT
ACTGCCGGATCAGCAGCACTTGAGTGCCTCACGCCTCCAAAGTTCCAC---GGATCACTG
GGA---------------------------------------------------------
------------------GTCACCGCAATCAACGCTGATGTCTCTCCATCGCAAGCGTTT
GGAGTGGAATTTTTTGCGACCTTCGTCCTCATCTTCGTTATTTTCGCCGTCTGCGATGAA
AACAGAACTGACGTCAAG------------GGATCGGCCCCGATGGCCATTGGCCTTACC
GTC---ACCATGATGGCTCTTGCTTTTGGCTCCTACACTGGCTGCAGTATGAACCCCGCC
AGAAGTCTTGGACCTGCCATCATCACA---------------------------------
GGA---AGATTTGAACATCACTGGGTTTACTGGGTTGGACCCATCCTAGGTGGAATCGTT
GCAGCCCTTATCTAC---CAATCCACTTTCAGAGTCCGGCCAGAACCCCTGCCA---CTC
GAAGACGAAGAA----------ACCGGTGGCAAGATTTACCACTTTAGGTAAGGTCCTTA
AACAAGAAAAAGTTTCCGTTCCGTAAAAAGTCGTTTTTTTCTAAAACTATCGATTTGATG
AGCCAGTTGGCAAATACTCCCCCCCCCCCCCCCCAAAAAAAAAAAAAAAAACAGTTTGGC
------------------------------------------------------------
------------------------------------------------------------
------------------------------------------------------------
------------------------------------------------------------
------------------------------------------------------------
------------------------------------------------------------
------------------------------------------------------------
------------------------------------------------------------
------------------------------------------------------------
------------------------------------------------------------
------------------------------------------------------------
------------------------------------------------------------
------------------------------------------------------------
------------------------------------------------------------
------------------------------------------------------------

------------------------------------------------------------
------------------------------------------------------------
------------------------------------------------------------
------------------------------------------------------------
------------------------------------------------------------
------
>JAKZAP010000012_Branchinecta_lynchi_EglpL1_v2
------------------------------------------------------------
------------------------------------------ATGGGTGTTCTCTGC---
---------TTTGGCAAC---CTTGTCGAGAGAATGAAGGGC---GCCATGAGTTGTTCG
GAAATGTCTGGA---AAT---CAGATTTGGAAGATGTTGCTATCTGAGGCTCTGGGTACA
ATGCTGCTTGTATTTGTAGGCTGTGCTTCTTGCTCACCCATCCATGGTGAA---------
---CCAAATGCGGTGGGAATTTCTTTGACGTTTGGATTGGCAGTGGCCGCACTTGCTCAG
TCAATTGGACACGTCAGCGGGTGTCACATCAACCCAGCCGTAACGGGCGCATTTTTGATC
ACCGGAAAAATCACGATTGTCCGGGCAGTTCTTTACGTCGCCGCACAGTGCATCGGAGCT
ACTGCCGGATCAGCAGCCCTTCAGTGTCTGACGCCCCCGAAGTTTCAC---GGGTCACTG
GGA---------------------------------------------------------
------------------GTCACGGCTATCCACGCTGATGTCTCTCCGTCCCAAGCGTTT
GGGGTGGAATTTTTCGCCACCTTCGTCCTTATCCTCGTTATTTTTGCTGTCTGCGATGAA
AACAGAACTGACGTCAAA------------GGATCGGCACCGATGGCCATTGGCCTCACC
GTC---ACCATGATGGCTCTTGCTTTTGGCTCCTACACTGGCTGCAGTATGAACCCCGCC
AGAAGTCTTGGACCTGCCATCATCACA---------------------------------
GGA---AGATTCGAACATCACTGGGTTTACTGGGTTGGACCCATCCTAGGGGGAGTCGTT
GCAGCCCTTATCTAC---CAGTCCACTTTCAGAGTCCGGCCAGAACCCATGCCA---CAT
GAAGACGAAGAA---------ACCGGTGGCAATATATACCACTTTAAGGTAAGGTTCTCT
AACGAGAAAAAAGCTTCCGTGTCG------------------------------------
------------------------------------------------------------
------------------------------------------------------------
------------------------------------------------------------
------------------------------------------------------------
------------------------------------------------------------
------------------------------------------------------------
------------------------------------------------------------
------------------------------------------------------------
------------------------------------------------------------
------------------------------------------------------------
------------------------------------------------------------
------------------------------------------------------------
------------------------------------------------------------
------------------------------------------------------------
------------------------------------------------------------
------------------------------------------------------------
------------------------------------------------------------
------------------------------------------------------------
------------------------------------------------------------
------------------------------------------------------------
------------------------------------------------------------
------

```
>JAUPMV010000014_Branchinecta_sandiegonensis_EglpL1_v2
------------------------------------------------------------
------------------------------------------ATGGGTGTTCCTTGC---
---------TTTGGCAAC---CTTGTCGAGAGAATGAAGGGC---GCCATGAGTTGTTCG
GAAATGTCTGGA---AAT---CAGATTTGGAAGATGTTGCTGTCTGAAGCTTTTGGTACT
ATGCTGCTTGTCTTTGTAGGCTGTGCTTCGTGCTCCCCCATCCATGGAGAA---------
---CCAAATGCGGTGGGAATTTCTTTGACGTTTGGATTGACAGTGGCTGCACTTGCTCAG
TCAATTGGCCACGTCAGCGGATGTCACATCAACCCAGCCGTAACGGCCGGATTTTTGATC
ACCGGAAAAATCACGATTGTCCGGGCAATTCTTTACGTCGCCGCACAGTGCATCGGAGCT
ACTGCCGGATCAGCAGCACTTGAGTGTCTGACACCCCCGAAGTTCCAC---GGGTCACTG
GGA---------------------------------------------------------
------------------GTCACCGCTATCAACGCTGATGTCTCTCCGTCGCAAGCGTTT
GGAGTGGAATTTTTCGCGACCTTCGTCCTCGTTCTGGTTATTTTCGCCGTCTGCGATGAG
AACAGAACTGACGTCAAA------------GGATCGGCCCCGATCGCCATTGGCCTCACC
GTC---ACCATGATGGCTCTTGCTTTTGGCTCCTACACTGGTTGCAGTATGAATCCCGCC
AGAAGTCTTGGACCTGCCATCATCACA---------------------------------
GGA---AGATTCGAACATCACTGGGTTTACTGGGTTGGACCTATCCTAGGTGGAATCGTT
GCAGCCCTTATCTAC---CAGTCCACTTTCCGAGTCCGACCAGAACCCCTGCCA---CAC
GAAGACGAAGAA---------ACCGGTGGCAAGATTTACCACTTTAGGGTAAGGTTC---
------------------------------------------------------------
------------------------------------------------------------
------------------------------------------------------------
------------------------------------------------------------
------------------------------------------------------------
------------------------------------------------------------
------------------------------------------------------------
------------------------------------------------------------
------------------------------------------------------------
------------------------------------------------------------
------------------------------------------------------------
------------------------------------------------------------
------------------------------------------------------------
------------------------------------------------------------
------------------------------------------------------------
------------------------------------------------------------
------------------------------------------------------------
------------------------------------------------------------
------------------------------------------------------------
------------------------------------------------------------
------------------------------------------------------------
------------------------------------------------------------
------
>GCBP01028516_Eubranchipus_grubii_EglpL2
------------------------------------------------------------
------------------------------------------ATGGGCTGCTGTGACCAT
---------------------TTGTTCAGAAAAGTGAGGAAT---ATGGCCAACTGCGAA
GAAATGTCCGGG---AACAAG---ATCTGGCTATCTCTTCTGTCAGAGGCTCTGGGAACC
ATGCTCCTGGTTTTCATTGGCTGTGCGTCATGCTTGCCTATCCACGGAGAG---------
```

---CCAAATTCAGTGGGAATTTCATTGGCTTTCGGCCTATTAGTCGCATCTTTAGCTCAG
GCACTTGGTCACGTCAGCGGTTGCCATATTAATCCAGCAGTGACAGCGGGATTTCTAATT
ACCGGCAAGATCACGATTGTTCGCGCCATTCTTTATATTGCGGCACAATGTATCGGCGCC
ACGGCAGGAGCGGCATGCCTTGAGTGTCTGACCCCAGAAGAGTTCCAC---GGGGCCCTT
GGC---------------------------------------------------------
------------------GTTACCTCTATTGCGGCGAAAGTGTCGCCATCTCAAGGATTC
GGCGTCGAATTTTTCGCTACGTTCACACTCGTTCTCGTAATTTTTGCCGTATGCGATGAG
AACCGAACTGATGTTAAG------------GGATCCGCCCCTCTAGCCATCGGACTTGTG
GTC---ACCATGCAGGCTCTTGCTTTCGGCTCATACACGGGTTGCAGTATGAATCCAGCA
AGGAGTCTTGGACCGGCAATTATAAAA---------------------------------
GGA---AAATTTGAACATCACTGGGTGTATTGGGTTGGACCGATCCTTGGAGGGATCGTC
GCTGCACTACTTTAC---GAAAACGCATTTAAAGCACAATCCGAAGTTGAATGTGAAGAT
GATGAAGAGCAA---------ATTAAGCCACGAGTTTATCATTTCAAGGAAAAC------
AATCGAAAAGGAAGTTTTATTGCCGAGAAGGAA---------AAA---TTGGAATTAATT
GTAGATCGTCAATCGTCACTC---------------------------------------
------------------------------------------------------------
------------------------------------------------------------
------------------------------------------------------------
------------------------------------------------------------
------------------------------------------------------------
------------------------------------------------------------
------------------------------------------------------------
------------------------------------------------------------
------------------------------------------------------------
------------------------------------------------------------
------------------------------------------------------------
------------------------------------------------------------
------------------------------------------------------------
------------------------------------------------------------
------------------------------------------------------------
------------------------------------------------------------
------------------------------------------------------------
------------------------------------------------------------
------------------------------------------------------------
------------------------------------------------------------
------
>GCBP01028494_Eubranchipus_grubii_EglpL1
------------------------------------------------------------
------------------------------------------ATGGGTTGCTGTAGTCAA
TCT------TGTGCTCGG---TTGATGAATAAGATGAAGAAT---ATATTGGGATGTTCT
GAAATTGCTGGTGAAAATAGGCAAATATGGAAAGCACTCCTTGCGGAGGCACTGGGAACT
ATGTTCCTTGTGCTAGTGGGTTGTGCATCGTGTGTGGAATGGAACGGAGTA---------
GCGCCAAGCCCGGTGGCAATTTCGTTGGCTTTTGGTGTAACGTTGGCGGCAATGGCACAG
GCACTTGGTCACGTCAGCGGTTGCCATATTAATCCAGCAGTGACAGCGGGATTTCTAATT
ACCGGCAAGATCACGATTGTTCGCGCCATTCTTTATATTGCGGCACAATGTATCGGCGCC
ACGGCAGGAGCGGCATGCCTTGAGTGTCTGACCCCAGAAGAGTTCCAC---GGGGCCCTT
GGC---------------------------------------------------------
------------------GTTACCTCTATTGCGGCGAAAGTGTCGCCATCTCAAGGATTC

GGCGTCGAATTTTTCGCTACGTTCACACTCGTTCTCGTAATTTTTGCCGTATGCGATGAG
AACCGAACTGATGTTAAG------------GGATCCGCCCCTCTAGCCATCGGACTTGTG
GTC---ACCATGCAGGCTCTTGCTTTCGGCTCATACACGGGTTGCAGTATGAATCCAGCA
AGGAGTCTTGGACCGGCAATTATAAAA---------------------------------
GGA---AAATTTGAACATCACTGGGTGTATTGGGTTGGACCGATCCTTGGAGGGATCGTC
GCTGCACTACTTTAC---GAAAACGCATTTAAAGCACAATCCGAAGTTGAATGTGAAGAT
GATGAAGAGCAA---------ATTAAGCCACGAGTTTATCATTTCAAGGAAAAC------
AATCGAAAAGGAAGTTTATTGCCGAGAAGGAA----------AAA---TTGGAATTAATT
GTAGATCGTCAATCGTCACTC---------------------------------------
------------------------------------------------------------
------------------------------------------------------------
------------------------------------------------------------
------------------------------------------------------------
------------------------------------------------------------
------------------------------------------------------------
------------------------------------------------------------
------------------------------------------------------------
------------------------------------------------------------
------------------------------------------------------------
------------------------------------------------------------
------------------------------------------------------------
------------------------------------------------------------
------------------------------------------------------------
------------------------------------------------------------
------------------------------------------------------------
------------------------------------------------------------
------------------------------------------------------------
------------------------------------------------------------
------------------------------------------------------------
------
>EFX74648_Daphnia_pulex_EglpL
------------------------------------------------------------
------------------------------------------------------------
---------------------------------ATGCGCAAC---GTATTCGGCTGCGAT
GAGCTCAAC---AAGAACCGCGACATCTGGCGCATGCTCATGGCCGAATTCGTCGGCCCG
CTCTTTCTCGTCCTCATCGGCTGCGCTTCCTGCGTCGAGGGATGGAACGAC---CAGTAC
TCGCCGCACATTGTCCAGGTCGCCCTCTCCTTCGGCGTCACCATCGCAACAATGGCCCAG
GCCTTGGGACATGTGAGCGGAGGCCATTTCAACCCGGCCGTGACGGTGGCCTGTCTCGTG
ACCGGCAAAATCTCCATCGTCAAGTCCATCTTCTACATCGTTGCCCAGTGCCTGGGCGCC
ATATGTGGAGCTGCTCTTCTCCAGGCTCTGACGCCGACTGATTTCCAC---AATACGTTG
GGC---------------------------------------------------------
------------------GTGACGGAGATCCACAAAGCCCTGACCCCGACGCAGGGCTTC
GGCGTCGAGTTCTTCTCGACGTTCACGCTGGTCCTGGTCGTCTTTGGCGTCTGCGACGAT
AACCGCAAAGACGTCAAA------------GGATCGGCCCCGCTGGCCATCGGCCTCTGC
ATC---GCCACCGCCATTTTGGCCACCGGTAATTACACTGGCGGCAGCTTGAATCCGGCC
CGCTCCCTAGGACCGGCAGTCATCAGC---------------------------------
AAC---AAGTGGGCTTATCACTGGGTCTACTGGGCGGGACCGATCGTCGGTGGAGTAGTG
GCAGCACTCACCTAC---CAGAAGGCATTCAAGGCCCGTTCG---------------CCC

GAAGAGGAAGTC---------GAGCTGGAATCC---TACCAGTACCGAGTGGCC------
AACTCGAAAGAGAGC------------------------------------GAGATTATC
GCCGACCGAACGACCACCATC---------------------------------------
------------------------------------------------------------
------------------------------------------------------------
------------------------------------------------------------
------------------------------------------------------------
------------------------------------------------------------
------------------------------------------------------------
------------------------------------------------------------
------------------------------------------------------------
------------------------------------------------------------
------------------------------------------------------------
------------------------------------------------------------
------------------------------------------------------------
------------------------------------------------------------
------------------------------------------------------------
------------------------------------------------------------
------------------------------------------------------------
------------------------------------------------------------
------------------------------------------------------------
------------------------------------------------------------
------------------------------------------------------------
------
>XM_046802164_Daphnia_pulicaria_EglpL
------------------------------------------------------------
------------------------------------------------------------
------------ATGGAA---TTTTGGGCGAGAATGCGCAAC---GTATTCGGCTGCGAT
GAGCTCAAC---AAGAACCGCGACATCTGGCGCATGCTCATGGCCGAATTCGTCGGCCCG
CTCTTTCTCGTCCTCATCGGCTGCGCTTCCTGCGTCGAGGGATGGAACGAC---CAGTAC
TCGCCGCACATTGTCCAGGTCGCCCTCTCCTTCGGCGTCACCATCGCAACAATGGCCCAG
GCCTTGGGACATGTGAGCGGAGGCCATTTCAACCCGGCCGTGACGGTGGCCTGTCTCGTG
ACCGGCAAAATCTCCATCGTCAAGTCCATCTTCTACATCGTTGCCCAGTGCCTGGGCGCC
ATATGTGGAGCTGCTCTTCTCCAGGCTCTGACGCCGAGCGATTTCCAC---AATACGTTG
GGC---------------------------------------------------------
------------------GTGACGGAGATCCACAAAGCCCTGACCCCGACGCAGGGCTTT
GGCGTCGAGTTCTTCTCGACGTTCACGCTGGTCCTGGTCGTCTTTGGCGTCTGCGACGAG
AACCGCAAAGACGTCAAA------------GGATCGGCCCCGTTGGCCATCGGCCTCTGC
ATC---GCCACCGCCATTTTAGCCACTGGTAATTACACTGGCGGCAGCTTGAATCCGGCC
CGCTCCCTAGGACCGGCAGTCATCAGC---------------------------------
AAC---AAGTGGGCTTATCACTGGGTCTACTGGGCGGGACCGATCGTCGGTGGCGTAGTG
GCAGCACTCACCTAC---CAGAAGGCCTTCAAGGCCCGTTCG---------------CCC
GAAGAGGAAGTC---------GAGCTGGAATCC---TACCAGTACCGAGTCGCC------
AACTCGAAAGAGAGT------------------------------------GAGATTATC
GCCGACCGAACGACCACCATC---------------------------------------
------------------------------------------------------------
------------------------------------------------------------
------------------------------------------------------------

------------------------------------------------------------
------------------------------------------------------------
------------------------------------------------------------
------------------------------------------------------------
------------------------------------------------------------
------------------------------------------------------------
------------------------------------------------------------
------------------------------------------------------------
------------------------------------------------------------
------------------------------------------------------------
------------------------------------------------------------
------------------------------------------------------------
------------------------------------------------------------
------------------------------------------------------------
------------------------------------------------------------
------------------------------------------------------------
------------------------------------------------------------
------
>JAACYE010000007_Daphnia_obtusa_EglpL
------------------------------------------------------------
------------------------------------------------------------
------------ATGGAA---TTTTGGGCGAGAATGCGCAAC---GTATTCGGCTGCGAT
GAGCTCAAC---AAGAACCGCGACATCTGGCGCATGCTCATGGCCGAATTCGTCGGCCCG
CTCTTTCTCGTCCTCATCGGCTGCGCTTCCTGCGTCGAGGGATGGAACGAC---CAGTAC
TCGCCGCACATCGTCCAGGTCGCCCTCTCCTTCGGCGTCACCATCGCAACAATGGCCCAG
GCCTTGGGGCATGTGAGCGGAGGCCATTTCAACCCGGCCGTGACGGTGGCCTGTCTCGTG
ACCGGCAAAATCTCCATCGTCAAGTCCATCTTCTACATCATTGCCCAGTGCCTGGGCGCC
ATATGTGGAGCTGCTCTTCTGCAGGCTCTGACGCCGACCGATTTCCAC---AATACGTTG
GGC---------------------------------------------------------
------------------GTGACGGAGATACACAAAGCCCTGACCCCGACCCAGGGCTTC
GGCGTCGAGTTCTTCTCGACGTTCACACTGGTCCTAGTCGTCTTCGGCGTCTGCGATGAT
AACCGCAAAGACGTCAAA------------GGATCGGCCCCGTTGGCCATCGGTCTCTGC
ATC---GCCACCGCCATTTTGGCCACCGGTAATTACACTGGCGGCAGCTTGAATCCGGCC
CGTTCCCTCGGACCGGCAGTCATCAGC---------------------------------
AAC---AAGTGGACTTATCACTGGGTGTACTGGGCGGGACCGATCGTCGGTGGCGTAGTG
GCGGCACTCACCTAC---CAGAAGGCATTCAAGGCCCGCTCG---------------CCC
GAAGAGGAAGTC---------GAGCTGGAATCC---TACCAGTACCGAGTCGCC------
AACTCGAAAGAGAGC------------------------------------GAGATTATC
GCCGACCGAACGACCACCATC---------------------------------------
------------------------------------------------------------
------------------------------------------------------------
------------------------------------------------------------
------------------------------------------------------------
------------------------------------------------------------
------------------------------------------------------------
------------------------------------------------------------
------------------------------------------------------------
------------------------------------------------------------

------------------------------------------------------------
------------------------------------------------------------
------------------------------------------------------------
------------------------------------------------------------
------------------------------------------------------------
------------------------------------------------------------
------------------------------------------------------------
------------------------------------------------------------
------------------------------------------------------------
------------------------------------------------------------
------------------------------------------------------------
------
>HAFN01000200_Daphnia_galeata_EglpL
------------------------------------------------------------
------------------------------------------------------------
------------ATGGCA---TTTTGGGCGAGAATGCGCAAC---GTATTCGGCTGCGAT
GAGCTCAGC---AAGAACCGCGACATCTGGCGTATGCTCATGGCCGAGTTCGTCGGGCCG
CTCTTTCTCGTCCTCGTCGGCTGCGCTTCCTGTGTCGAAGGATGGAACGAT---CAGTAC
TCACCGCACATCGTGCAGGTCGCCCTCTCCTTCGGCATCACCATCGCAACAATGGCCCAG
GCCTTGGGACATGTGAGCGGAGGGCATTTCAACCCGGCCGTGACGGTGGCCTGTCTCGTG
ACCGGCAAAATTTCCATCGTCAAGTCCATCTTCTACATCATTGCCCAGTGTTTGGGCGCC
ATATGTGGAGCTGCTCTTCTACAGGCTCTGACGCCGACCGAGTTCCAC---AACACGTTG
GGC---------------------------------------------------------
------------------GTGACGGAGGTACACAAAGCCCTGACGCCGACACAGGGTTTC
GGTGTTGAGTTTTTCTCTACATTCACACTTGTCCTAGTCGTCTTTGGTGTATGCGACGAT
AACCGCAAAGATGTCAAA------------GGATCGGGCCCACTGGCCATCGGCCTCTGT
ATT---GCCACCGCCATTTTGGCCACTGGTAATTACACTGGCGGCAGCTTGAATCCGGCC
CGCTCTCTCGGACCGGCAGTCATCAGC---------------------------------
AAC---AAGTGGAATTATCACTGGGTGTACTGGGCAGGACCAATCGTCGGTGGCGTAGTT
GCAGCTCTTACCTAC---CAGAAGGCATTCAAGGCCCGTTCT---------------CCC
GAAGAAGAAGTG---------GAATTGGAATCCTACCACCAATACCGCATAGCC------
AATTCGAAAGAGAGT------------------------------------GAGATCATC
GCCGACCGAACGACTACCATC---------------------------------------
------------------------------------------------------------
------------------------------------------------------------
------------------------------------------------------------
------------------------------------------------------------
------------------------------------------------------------
------------------------------------------------------------
------------------------------------------------------------
------------------------------------------------------------
------------------------------------------------------------
------------------------------------------------------------
------------------------------------------------------------
------------------------------------------------------------
------------------------------------------------------------
------------------------------------------------------------
------------------------------------------------------------

------------------------------------------------------------
------------------------------------------------------------
------------------------------------------------------------
------------------------------------------------------------
------------------------------------------------------------
------
>JAAVJA010000733_010012108_Daphnia_dubia_EglpL
------------------------------------------------------------
------------------------------------------------------------
------------ATGGCA---TTTTGGGCGAGAATGCGCAAC---GTATTCGGCTGCGAT
GAGCTCAGC---AAGAACCGCGACATCTGGCGTATGCTCATGGCCGAGTTCGTTGGGCCG
CTCTTTCTCGTCCTCGTCGGCTGCGCTTCCTGTGTTGAAGGATGGAACGAT---CAGTAC
TCACCGCACATCGTCCAGGTCGCCCTCTCCTTCGGCGTCACCATCGCAACAATGGCCCAG
GCCTTGGGGCATGTGAGCGGAGGCCATTTCAACCCGGCCGTGACGGTGGCTTGTCTCGTG
ACCGGCAAAATCTCCATCGTCAAGTCCATCTTCTACATCATTGCCCAGTGCTTGGGCGCC
ATATGTGGAGCTGCTCTTCTG---GCTCTGACGCCGACCGAGTTTCAC---AATACCTTG
GGC---------------------------------------------------------
------------------GTGACGGAGGTACACAAAGCCCTGACTCCGACCCAGGGTTTC
GGCGTCGAGTTCTTCTCGACATTCACACTCGTCCTGGTCGTCTTTGGTGTCTGCGATGAG
AACCGAAAAGACGTCAAA------------GGATCGGCTCCATTGGCCATCGGCCTGTGT
ATT---GCCACCGCCATTTTGGCCACTGGCAATTACACTGGTGGCAGCTTGAATCCGGCC
CGTTCTCTCGGCCCGGCAGTCATCAGC---------------------------------
AAC---AAGTGGAATTACCACTGGGTATACTGGGCAGGGCCGATCGTCGGTGGAGTAGTG
GCAGCTTTGACCTAC---CAAAAGGCTTTCAAGGCTCGTTCA---------------CCC
GAGGAGGAAGTC---------GAGTTGGAATCCTACCACCAGTACCGAGTCGCC------
AATTCGAAAGAGAGC------------------------------------GAGATCATC
GCCGACCGAACGACTACCATC---------------------------------------
------------------------------------------------------------
------------------------------------------------------------
------------------------------------------------------------
------------------------------------------------------------
------------------------------------------------------------
------------------------------------------------------------
------------------------------------------------------------
------------------------------------------------------------
------------------------------------------------------------
------------------------------------------------------------
------------------------------------------------------------
------------------------------------------------------------
------------------------------------------------------------
------------------------------------------------------------
------------------------------------------------------------
------------------------------------------------------------
------------------------------------------------------------
------------------------------------------------------------
------------------------------------------------------------
------------------------------------------------------------
------

```
>CM023197_Daphnia_carinata_EglpL
------------------------------------------------------------
------------------------------------------------------------
------------ATGGCA---ATCTGGCAGAGAATGCGCAAC---GTATTCGGCTGCGAT
GAGCTCAGC---AAGAACCGCGACATCTGGCGGATGCTCATGGCCGAGTTCGTCGGCCCA
CTCTTTCTCGTCCTCATCGGCTGTGCTTCCTGCGTCGAAGGATGGAACGAC---CAGTAC
TCACCGCACATCGTCCAGGTCGCTCTCTCCTTCGGCGTCACCATCGCCACAATGGCGCAG
GCCTTGGGTCATGTGAGTGGAGGCCATTTCAACCCGGCCGTGACGGTCGCTTGTCTTGTG
ACGGGCAAGATTTCCATCGTCAAGTCCATCTTCTACATCGTGGCCCAGTGTCTGGGCGCC
ATATGTGGAGCTGCTCTTTTGCAGGCATTGACTCCGACTGATTTCCAC---AACACGTTG
GGC---------------------------------------------------------
------------------GTGACAGAGATCCACAAGGCCCTGACTCCGACGCAGGGTTTC
GGCGTGGAATTCTTCTCCACGTTCACGCTCGTCCTCGTCGTCTTCGGTGTCTGCGATAGC
AATCGCAAAGACGTCAAA------------GGATCGGCACCATTGGCTATCGGCCTCTGC
ATC---GCCACCGCCATCTTGGCCACCGGAAACTACACGGGTGGCAGTTTGAATCCGGCT
CGATCCCTCGGCCCAGCAGTCATCAGC---------------------------------
AAC---AAGTGGAGTTATCACTGGGTTTACTGGGCAGGACCAATCATCGGTGGTGTGGTG
GCCGCCCTTCTCTAT---CAGAAGGTTTTCAAAGCGCGTTCG---------------GCC
GAAGAAGAAAGC---------GAACTGGAATCT---TACCAGTACCAGGCGGCC------
AATTCAAAGGAAAGC------------------------------------GAAATTATC
GCTGATCGAACCACGACCATC---------------------------------------
------------------------------------------------------------
------------------------------------------------------------
------------------------------------------------------------
------------------------------------------------------------
------------------------------------------------------------
------------------------------------------------------------
------------------------------------------------------------
------------------------------------------------------------
------------------------------------------------------------
------------------------------------------------------------
------------------------------------------------------------
------------------------------------------------------------
------------------------------------------------------------
------------------------------------------------------------
------------------------------------------------------------
------------------------------------------------------------
------------------------------------------------------------
------------------------------------------------------------
------------------------------------------------------------
------------------------------------------------------------
------
>KAI9552347_Daphnia_sinensis_EglpL
------------------------------------------------------------
------------------------------------------------------------
------------ATGGCA---ATCTGGCAGAGAATGCGCAAC---GTATTCGGCTGCGAT
GAGCTCAGC---AAGAACCGCGACATCTGGCGGATGCTCATGGCCGAGTTCGTCGGCCCA
CTCTTTCTCGTCCTCATCGGCTGTGCTTCCTGCGTCGAAGGATGGAACGAC---CAGTAC
```

TCACCGCACATCGTCCAGGTCGCTCTCTCCTTCGGCGTCACCATCGCCACAATGGCGCAG
GCCTTGGGTCATGTGAGTGGAGGCCATTTCAACCCGGCCGTGACGGTCGCTTGTCTTGTG
ACGGGCAAGATTTCCATCGTCAAGTCCATCTTCTACATCGTGGCCCAGTGTCTGGGCGCC
ATATGTGGAGCTGCTCTTTTGCAGGCATTGACTCCGACTGATTTCCAC---AACACGTTG
GGC---------------------------------------------------------
------------------GTGACAGAGATCCACAAGGCCCTGACTCCGACGCAGGGTTTC
GGCGTGGAATTCTTCTCCACGTTCACGCTCGTCCTCGTCGTCTTCGGTGTCTGCGATAGC
AATCGCAAAGACGTCAAA------------GGATCGGCACCATTGGCTATCGGCCTCTGC
ATC---GCCACCGCCATCTTGGCCACCGGAAACTACACGGGTGGCAGTTTGAATCCGGCT
CGATCCCTCGGCCCAGCAGTCATCAGC---------------------------------
AAC---AAGTGGAGTTATCACTGGGTTTACTGGGCAGGACCAATCATCGGTGGTGTGGTG
GCCGCCCTTCTCTAT---CAGAAGGTTTTCAAAGCGCGTTCG---------------GCC
GAAGAAGAAAGC---------GAACTGGAATCT---TACCAGTACCAGGCGGCC------
AATTCAAAGGAAAGC------------------------------------GAAATTATC
GCTGATCGAACCACGACCATC---------------------------------------
------------------------------------------------------------
------------------------------------------------------------
------------------------------------------------------------
------------------------------------------------------------
------------------------------------------------------------
------------------------------------------------------------
------------------------------------------------------------
------------------------------------------------------------
------------------------------------------------------------
------------------------------------------------------------
------------------------------------------------------------
------------------------------------------------------------
------------------------------------------------------------
------------------------------------------------------------
------------------------------------------------------------
------------------------------------------------------------
------------------------------------------------------------
------------------------------------------------------------
------------------------------------------------------------
------------------------------------------------------------
------
>XM_032941243_Daphnia_magna_EglpL
------------------------------------------------------------
------------------------------------------------------------
------------ATGGCA---ATCTGGCAGAGAATGCGCAAC---GTATTCGGCTGCGAT
GAGCTTAGC---AAGAACCGCGACATCTGGCGGATGCTCATGGCCGAGTTCGTCGGCCCA
CTCTTTCTCGTCCTCATCGGATGTGCTTCCTGCGTCGAAGGATGGAACGAC---CAGTAC
TCACCGCACATCGTCCAGGTCGCTCTCTCCTTTGGCGTCACCATCGCCACAATGGCGCAG
GCCTTGGGTCATGTGAGTGGAGGCCATTTCAACCCAGCCGTGACGGTGGCCTGTCTTGTG
ACGGGAAAGATTTCTATCGTCAAGTCCATCTTCTACATCTTGGCACAGTGCTTAGGCGCC
ATATGTGGAGCTGCTCTTTTGCAGGCATTGACTCCGACCGAGTTCCAC---AACACACTG
GGC---------------------------------------------------------
------------------GTGACGGAGATTCACAAAGCCCTGACTCCGACGCAGGGTTTC

GGTGTGGAATTCTTCTCTACGTTCACGCTCGTCCTCGTCGTCTTCGGTGTCTGCGATAGC
AATCGCAAAGACGTCAAA------------GGATCGGCACCATTGGCCATCGGCCTCTGC
ATC---GCCACCGCCATTTTGGCCACCGGAAACTACACGGGTGGTAGTTTGAATCCGGCT
CGATCCCTCGGTCCGGCAGTCATCAGC---------------------------------
AAC---AAGTGGAGTTATCATTGGGTTTACTGGCAGGACCAATCATCGGTGGTGTGGTG
GCCGCCCTTCTCTAC---CAGAAGGTTTTAAAGCCCGTTCG----------------GTC
GAAGAAGAAATC---------GAATTGGAATCT---TACCAGTACCATGTGGCC------
AATTCAAAGGAGAGC------------------------------------GAAATTATC
GCTGATCGAACCACGACCATC---------------------------------------
------------------------------------------------------------
------------------------------------------------------------
------------------------------------------------------------
------------------------------------------------------------
------------------------------------------------------------
------------------------------------------------------------
------------------------------------------------------------
------------------------------------------------------------
------------------------------------------------------------
------------------------------------------------------------
------------------------------------------------------------
------------------------------------------------------------
------------------------------------------------------------
------------------------------------------------------------
------------------------------------------------------------
------------------------------------------------------------
------------------------------------------------------------
------------------------------------------------------------
------------------------------------------------------------
------------------------------------------------------------
------
>JAKUCG010000213_Ceriodaphnia_dubia_EglpL
------------------------------------------------------------
------------------------------------------------------------
------------ATGGCA---TTTTGGGCGAGAATGAAGAGC---GTATTCGGTTGCGAT
GAGCTCAGC---AAGAACCGCGACATCTGGCGAATGCTCATGGCCGAATTCGTCGGCCCA
CTCTTCCTCGTCCTCATCGGCTGCGCTTCTTGCGTCGAGGGATGGAACGAG---CAATAT
TCACCACACATCGTTCAGGTCGCCCTATCCTTCGGCGTCACCATAGCCACAATGGCACAG
GCCTTGGGACATGTTAGCGGTGGCCACTTCAACCCTGCCGTGACGGTGGCCTGCCTCGTG
ACCGGCAAAATCACCATCGTCAAGTCCATCTTCTACATAATAGCCCAATGTTTGGGCGCC
ATATGTGGAGCTGCTCTTCTCCAGGCTTTGACGCCGAGCGAGTTCCAC---AACACACTG
GGC---------------------------------------------------------
------------------GTGACGGAAGTGCACAAAGCCCTGACGCCGACGCAGGATTC
GGCGTCGAGTTCTTCTCCACATTCACCCTGGTGCTGGTCGTTTTCGGCGTTTGCGACGAG
AACCGCAAAGATGTCAAA------------GGATCGGCCCCATTAGCCATCGGCCTCTGC
ATC---GCTACCGCCATTTTGGCCACCGGTAACTACACAGGCGGCAGCTTGAATCCGGCC
CGTTCCCTGGGACCAGCAGTCATCAGC---------------------------------
AAC---AAATGGGCTTACCACTGGGTGTATTGGTCTGGACCGATCGTCGGTGGTATTGTT
GCTGCTTTGCTCTAC---CAAAAAGCCTTCAAGGCTCGTTCG----------------GCA

GATGAAGAACAG---------GAGCTTGAATCC---TATCAGTATCGCGCTGCC------
AACTCGAAAGAGAGT------------------------------------GAAATTATC
GCCGACCGAACCACTACTATT---------------------------------------
------------------------------------------------------------
------------------------------------------------------------
------------------------------------------------------------
------------------------------------------------------------
------------------------------------------------------------
------------------------------------------------------------
------------------------------------------------------------
------------------------------------------------------------
------------------------------------------------------------
------------------------------------------------------------
------------------------------------------------------------
------------------------------------------------------------
------------------------------------------------------------
------------------------------------------------------------
------------------------------------------------------------
------------------------------------------------------------
------------------------------------------------------------
------------------------------------------------------------
------------------------------------------------------------
------------------------------------------------------------
------
>CM057932_Chydorus_sphaericus_EglpL2
------------------------------------------------------------
------------------------------------------------------------
------------ATGGCA---TTTTGGGCCAGAATGAAGAAC---GTCTTTAGCAGCGAG
GAATTCTCC---AACAACCGAGGCATTTGGCGCATGTTGTTGGCTGAATTTGTTGGGCCC
CTCTTTCTTGTTCTCATCGGCTGCGCCAGCTGTGTTGAAGGATGGAATGAG---GCTTAC
TCACCTCACATTGTTCAAGTGGCTCTCTCTTTCGGCGTCACCATTGCCACCATGGCCCAG
GCCATGGGACATGTCAGCGGAGGTCATTTCAATCCTGCAGTCACAACGGCTTGTCTGGTG
ACTGGAAAAATTAGCTTCGCCAAAGCCTTGTCTTACATTGTTTCTCAGTGCTTGGGAGCC
ATTTCAGGAGCGGCTCTCCTTCAGACCTTGACTCCATCCGAGTTCCAC---AACACACTT
GGA---------------------------------------------------------
------------------GTAACGCAGATGCATCCTAAATTGTCACCCACTCAGGGCTTT
GGCGTTGAATTCTTCTCAACTTTCACCCTTGTTCTCGTCGTCTTCAGCGTTTGTGACTCG
AACCGCAAAGACATCAGG------------GGCTCCGCTCCGTTGGCCATTGGGCTCGCC
ATC---TCGACCGCTATTCTAGCCACGGGCGTGTACACAGGAGGAAGTCTAAATCCTGCT
CGCTCTTTGGGCCCGGCGTTGGTCAGC---------------------------------
AAT---AATTGGACGTACCACTGGGTTTACTGGGCCGGCCCGATCGTTGGAGGTATTGTG
GCTGCTCTTCTCTAC---GAAAAAGTCTTCAGGGCGCGAACG---------------GTG
GAGGAAGAGAGG---------GAACTGGAG---GAGTACCAGTACAGGGCTGCT------
AATGCCGAGGAGAGT------------------------------------GAAATCATT
GTTGATCGCACCACTACCATC---------------------------------------
------------------------------------------------------------
------------------------------------------------------------
------------------------------------------------------------

------------------------------------------------------------
------------------------------------------------------------
------------------------------------------------------------
------------------------------------------------------------
------------------------------------------------------------
------------------------------------------------------------
------------------------------------------------------------
------------------------------------------------------------
------------------------------------------------------------
------------------------------------------------------------
------------------------------------------------------------
------------------------------------------------------------
------------------------------------------------------------
------------------------------------------------------------
------------------------------------------------------------
------------------------------------------------------------
------------------------------------------------------------
------
>CM057932_Chydorus_sphaericus_EglpL1
------------------------------------------------------------
------------------------------------------------------------
------------ATGGCA---TTTTGGGCCAGAATGAAGAAC---GTCTTCGGCTGCGAG
GAGTTCTCC---AAGAACCGAGACATTTGGCGCATGTTGATGGCCGAATTTGTTGGGCCC
CTCTTTCTTGTTCTCATCGGCTGCGCCAGCTGTGTTGAAGGATGGAACGAA---GCTTAC
TCACCTCACATTGTTCAAGTGGCTCTTTCCTTCGGCGTCACCATTGCCACCATGGCCCAG
GCCATGGGACATGTGAGCGGAGGTCATTTCAATCCTGCAGTCACAACGGCCTGTCTGGTG
ACTGGAAAAATTAGCATTGCTAAAGCCTTGGCTTACATTGTTTCTCAGTGCTTGGGAGCC
ATTTCAGGAGCGGCTCTCCTTCAGGCCTTGACTCCATCAGAGTTCCAC---AACACGCTG
GGA---------------------------------------------------------
------------------GTAACGCAGATTCACCCGAAATTGTCGCCCACTCAGGGCTTT
GGCGTTGAATTCTTCTCAACTTTCACGCTTGTTCTCGTCGTCTTCGGCGTTTGCGACTCG
AACCGCAAAGACATCAGG------------GGCTCCGCTCCGCTGGCCATTGGGCTCGCC
ATC---TCCACCGCTATTCTAGCCACGGGCGTGTACACTGGAGGAAGTCTAAATCCTGCT
CGCTCCTTGGGACCGGCGTTGGTCAGC---------------------------------
AAT---AATTGGACGTACCACTGGGTTTACTGGGCCGGCCCGATCGTTGGAGGTATTGTG
GCTGCTCTTCTCTAC---GAAAAAGTCTTCAGGGCGCGAACG---------------GTG
GAGGAAGAGAGG---------GAACTGGAG---GAGTACCAGTACAGGGCTGCT------
AACGCCAAGGAGAGT------------------------------------GAAATCATT
GCTGATCGCACCACTACCATC---------------------------------------
------------------------------------------------------------
------------------------------------------------------------
------------------------------------------------------------
------------------------------------------------------------
------------------------------------------------------------
------------------------------------------------------------
------------------------------------------------------------
------------------------------------------------------------
------------------------------------------------------------

------------------------------------------------------------
------------------------------------------------------------
------------------------------------------------------------
------------------------------------------------------------
------------------------------------------------------------
------------------------------------------------------------
------------------------------------------------------------
------------------------------------------------------------
------------------------------------------------------------
------------------------------------------------------------
------------------------------------------------------------
------
>GKAG01008350_Enord_PripL2
------------------------------------------------------------
------------------------------------------ATGAAG---GGCAACTGC
---------TTCATGGAT---TTCGTCGAGAAAATGAAGAAA---GTATTCGGAAGCGAT
GACCTGGGCGCCAGGAAAGGCGAGATCTGGTGCATGCTGACGGCCGAGTTTGTTGGAACA
CTCTTCCTCGTCCTCTTCGGCTGCGCTTCCTGCATTTCGGGATTCAACACT---------
CCCGCCGACATCGTTCAAATCGCCCTGACGTTCGGACTCACCATCGCCACCATGGCTCAG
GCATTCGGTCACGTGAGTGGGTGTCACCTGAACCCGGCCGTCACCATCGGCTTGCTGGTC
GCTGGTAAAATCAGCTTCGTTAAGGCCGGTTGCTACATCGTATCCCAGTGCCTCGGCGCC
ATCTGTGGGGCTGCTCTACTCGAGTCTTTGACCCCGGCCGACTACCAC---GGTTCAATG
GGA---------------------------------------------------------
------------------GTGACATCGCTGCATGCCATGCTGACCCCGGCACAAGGTTTC
GGCCTCGAGTTCTTCACCACCTTCAGTCTGGTCATCGTCGTGCTGGGCGTTTGTGATCCT
CACCGGACTGACGTTAAG------------GGATCGGCACCGCTAGCCATCGGACTTACC
GTA---GCCACTGGCATCTTCGTCACCGGTAATTCCACGGGTGGCAGCATGAACCCGGCC
CGTTCCTTTGGACCAGCTGTAGTCAAC---------------------------------
AAC---CAATGGGCCGCGCACTGGGTTTATTGGGCTGGTCCTATCGTGGGTGGTATCGTC
GCTGCACTTGTTTAC---CAGAAAGCTTTCCGAGCTCGTTCG---------------CCC
GAGGAAGAACAA---------GAAATTCAAGT---TACAGCTACCAGCCGGCA------
GTTGATAAGGAAACT------------------------------------GAAGTTATC
ACCGACAGGACGACCACCATT---------------------------------------
------------------------------------------------------------
------------------------------------------------------------
------------------------------------------------------------
------------------------------------------------------------
------------------------------------------------------------
------------------------------------------------------------
------------------------------------------------------------
------------------------------------------------------------
------------------------------------------------------------
------------------------------------------------------------
------------------------------------------------------------
------------------------------------------------------------
------------------------------------------------------------
------------------------------------------------------------
------------------------------------------------------------

------------------------------------------------------------
------------------------------------------------------------
------------------------------------------------------------
------------------------------------------------------------
------------------------------------------------------------
------
>GKAG01008350_Evadne_nordmanni_EglpL2r
---------------------------------------------ATGTTGAGTCAGTCA
AGTCAAGTCGTCGGAGCAGAGCGGTCGGTCTTGGTCACTTGGATGAAG---GGCAACTGC
---------TTCATGGAT---TTCGTCGAGAAAATGAAGAAA---GTATTCGGAAGCGAT
GACCTGGGCGCCAGGAAAGGCGAGATCTGGTGCATGCTGACGGCCGAGTTTGTTGGAACA
CTCTTCCTCGTCCTCTTCGGCTGCGCTTCCTGCATTTCGGGATTCAACACT---------
CCCGCCGACATCGTTCAAATCGCCCTGACGTTCGGACTCACCATCGCCACCATGGCTCAG
GCATTCGGTCACGTGAGTGGGTGTCACCTGAACCCGGCCGTCACCATCGGCTTGCTGGTC
GCTGGTAAAATCAGCTTCGTTAAGGCCGGTTGCTACATCGTATCCCAGTGCCTCGGCGCC
ATCTGTGGGGCTGCTCTACTCGAGTCTTTGACCCCGGCCGACTACCAC---GGTTCAATG
GGA---------------------------------------------------------
------------------GTGACATCGCTGCATGCCATGCTGACCCCGGCACAAGGTTTC
GGCCTCGAGTTCTTCACCACCTTCAGTCTGGTCATCGTCGTGCTGGGCGTTTGTGATCCT
CACCGGACTGACGTTAAG------------GGATCGGCACCGCTAGCCATCGGACTTACC
GTA---GCCACTGGCATCTTCGTCACCGGTAATTCCACGGGTGGCAGCATGAACCCGGCC
CGTTCCTTTGGACCAGCTGTAGTCAAC---------------------------------
AAC---CAATGGCCGCGCACTGGGTTTATTGGCTGGTCCTATCGTGGGTGGTATCGTC
GCTGCACTTGTTTAC---CAGAAAGCTTTCCGAGCTCGTTCG---------------CCC
GAGGAAGAACAA---------GAAATTCAAGT---TACAGCTACCAGCCGGCA------
GTTGATAAGGAAACT------------------------------------GAAGTTATC
ACCGACAGGACGACCACCATT---------------------------------------
------------------------------------------------------------
------------------------------------------------------------
------------------------------------------------------------
------------------------------------------------------------
------------------------------------------------------------
------------------------------------------------------------
------------------------------------------------------------
------------------------------------------------------------
------------------------------------------------------------
------------------------------------------------------------
------------------------------------------------------------
------------------------------------------------------------
------------------------------------------------------------
------------------------------------------------------------
------------------------------------------------------------
------------------------------------------------------------
------------------------------------------------------------
------------------------------------------------------------
------------------------------------------------------------
------------------------------------------------------------
------

>GKAM01021750_Podon_leuckartii_PripL2
------------------------------------------------------------
------------------------------------------ATGAAG---GGCAACTGC
---------TTCATGGAT---TTCGTCGAGAAAATGAAGAAA---GTATTCGGAAGCGAT
GACCTGGGCGCCAGGAAAGGCGAGATCTGGTGCATGCTGACGGCCGAGTTTGTTGGAACA
CTCTTCCTCGTCCTCTTCGGCTGCGCTTCCTGCATTTCGGGATTCAACACT---------
CCCGCCGACATCGTTCAAATCGCCCTGACGTTCGGACTCACCATCGCCACCATGGCTCAG
GCATTCGGTCACGTGAGTGGGTGTCACCTGAACCCGGCCGTCACCATCGGCTTGCTGGTC
GCTGGTAAAATCAGCTTCGTTAAGGCCGGTTGCTACATCGTATCCCAGTGCCTCGGCGCC
ATCTGTGGGGCTGCTCTACTCGAGTCTTTGACCCCGGCCGACTACCAC---GGTTCAATG
GGA---------------------------------------------------------
------------------GTGACATCGCTGCATGCCATGCTGACCCCGGCACAAGGTTTC
GGCCTCGAGTTCTTCACCACCTTCAGTCTGGTCATCGTCGTGCTGGGCGTTTGTGATCCT
CACCGGACTGACGTTAAG------------GGATCGGCACCGCTAGCCATCGGACTTACC
GTA---GCCACTGGCATCTTCGTCACCGGTAATTCCACGGGTGGCAGCATGAACCCGGCC
CGTTCCTTTGGACCAGCTGTAGTCAAC---------------------------------
AAC---CAATGGGCCGCGCACTGGGTTTATTGGGCTGGTCCTATCGTGGGTGGTATCGTC
GCTGCACTTGTTTAC---CAGAAAGCTTTCCGAGCTCGTTCG---------------CCC
GAGGAAGAACAA---------GAAATTCAAAGT---TACAGCTACCAGCCGGCA------
GTTGATAAGGAAACT------------------------------------GAAGTTATC
ACCGACAGGACGACCACCATT---------------------------------------
------------------------------------------------------------
------------------------------------------------------------
------------------------------------------------------------
------------------------------------------------------------
------------------------------------------------------------
------------------------------------------------------------
------------------------------------------------------------
------------------------------------------------------------
------------------------------------------------------------
------------------------------------------------------------
------------------------------------------------------------
------------------------------------------------------------
------------------------------------------------------------
------------------------------------------------------------
------------------------------------------------------------
------------------------------------------------------------
------------------------------------------------------------
------------------------------------------------------------
------------------------------------------------------------
------------------------------------------------------------
------
>GKAM01000593_Podon_leuckartii_EglpL1
------------------------------------------------------------
------------------------------------------ATGAATGTTGCCTCGCTC
---------TTGATGGGT---TTTCTGGAAAAAATGCGCAAA---CTATTCGGCTGCGAT
GAGCTGGGC---AATAAGAACGACGTTTGGTGCATGCTGACTGGCGAATTTGTGGGAACT
TTATTCCTCGTCCTGTTCGGCTGTGCCTCCTGTGTCACCGGTTGGGATAAG---GATCAT

TCGCCGCATATTGTCCAAGTGGCCCTCGCCTTCGGAATCACCATTGCCACCATGGCCCAG
GCATTCGGACATGTCAGCGGTTGTCATCTAAATCCAGCCGTGACCATCGGTATGTTTGCT
GCCGGTAAAATCAGTTTGGTTAAAGCCGCTTGTTACGTCGTCACCCAGTTGATCGGCGCC
ATCTGCGGAGCTGGTCTGCTCGAGGCTTTAACGCCGACGCAATTTCAT---AATTCGATG
GGT---------------------------------------------------------
------------------GTGACGTCACTGCACCCGTCGCTGACACCGGCCCAAGGATTC
GGTATCGAATTCTTCACCACCTTCAGCCTGGTCATCGTCGTGTTCGGTGTGTGTGACCCG
AACCGCACCGACATCAAA------------GGATCGGCTCCCCTGGCCATCGGACTCACC
GTC---TCCACCGGCATCTTGGCTACCGGGGTTTACACCGGGGGAAGTATGAACCCAGCC
CGCTCATTTGGACCGGCCGTCGTTAGC---------------------------------
AAC---CAATGGGCTGCCCACTGGGTGTACTGGGCTGGTCCTATTGTCGGTGGTGTTGTT
GCTGCTCTACTTTAC---CAAAAGGCATTCAGAGCTCGATCG---------------CCC
CATGAGGAGCAA---------------------AGATGCAGCTACGAAGCGGCC------
GTTGACAAAGAA---------------------------------------GAGATTATC
GCTGACAGAATGACGACCATT---------------------------------------
------------------------------------------------------------
------------------------------------------------------------
------------------------------------------------------------
------------------------------------------------------------
------------------------------------------------------------
------------------------------------------------------------
------------------------------------------------------------
------------------------------------------------------------
------------------------------------------------------------
------------------------------------------------------------
------------------------------------------------------------
------------------------------------------------------------
------------------------------------------------------------
------------------------------------------------------------
------------------------------------------------------------
------------------------------------------------------------
------------------------------------------------------------
------------------------------------------------------------
------------------------------------------------------------
------------------------------------------------------------
------
>GGQP01006117_Diaphanosoma_celebensis_EglpL1
------------------------------------------------------------
------------------------------------------------------------
------------ATGGCC---ATTTGGGCGAAGATGCGCGCA---ATCTTCGGCTGCGAT
GAACTGTCC---AAGAACCGCGACATCTGGCGAATGCTCATCGCCGAATTCGTCGGCACC
ATGATGCTCGTCCTCATCGGATGCGCCTCCTGCGTCGAAGGATGGAACGAG---CAATAT
TCCGTCTCGCTGGTCGCCGTCGCCCTCACGTTCGGCATCACCATCGCCACCTTGGCTCAG
GTGATCGGACATGTTAGCGGTTGTCACATCAATCCGGCCGTCTCCATCAGCTTGGCCACG
ACGGGCAAAATGCCCATCGTCCGGGCCATTTTCTACATCGCTTCGCAGTGCGTCGGCGCC
ATTTGCGGCTCTGCTCTCCTGCAGTCTCTGACTCCGGAGAAATTCCAC---AACACCATG
GGC---------------------------------------------------------
------------------GTCACGTCGGTTCACGAGTCCGTGACACCGACGCAGGCCTTC

GGAGTCGAGTTCTTCGGCACGTTCATCCTGGTGCTGATCGTCTTCGCGCTCACCGACGAG
AACCGTAAAGATGTTCAT------------GGAACGGCGCCGATGGCCATCGGCTTGACC
GTC---ACTGCCGTAGTCTGCGCCATCGGAATGTACAGTGGCGCCAGTTTGAACCCGGCG
CGTTCATTAGGACCATCCGTCGTCACC---------------------------------
GGC---AAATGGGATCTTCACTGGGTCTACTGGGCAGGGCCGATCGTCGGCGCAATCGTG
GCGGCACTGCTCTAC---CAGAAGGTCTTCCGGAGCCGCTCG---------------CCC
GAAGAGGAGCAG---------CAGTTCGAA---GACTACAAATACCGCGCCGCC------
GACGTCAAGGAGAAC------------------------------------GAAGTGATC
GCCGATCGAACCACCACCATC---------------------------------------
------------------------------------------------------------
------------------------------------------------------------
------------------------------------------------------------
------------------------------------------------------------
------------------------------------------------------------
------------------------------------------------------------
------------------------------------------------------------
------------------------------------------------------------
------------------------------------------------------------
------------------------------------------------------------
------------------------------------------------------------
------------------------------------------------------------
------------------------------------------------------------
------------------------------------------------------------
------------------------------------------------------------
------------------------------------------------------------
------------------------------------------------------------
------------------------------------------------------------
------------------------------------------------------------
------------------------------------------------------------
------
>GGQP01085796_Diaphanosoma_celebensis_EglpL2
------------------------------------------------------------
------------------------------------------------------------
------------ATGGCC---ATTTGGGAAAAAATGCGATCG---GTCCTCGGCTGCAAG
GAATTCTCC---AAGAACCGCGACATTTGGCAAATGCTGATCGCCGAATTCGTTGGTACC
ATGTTCCTTGTGCTCATTGGCTGCGCTGCTTGTGTCGAAGGCTGGAGCGAG---CAATAT
TCGTCCAGCATCGTCCAAGTCGCCTTATCTTTCGGTGTCACCATCGCCACAATGGCTCAG
ACCATCGGCCATGTCAGCGGCTGTCACATCAACCCTGCGGTGACAACGGCGATGATGATC
ACAGGCAAAATCAGCGTCATGAGGGCCATTGCGTACGTCGTTTCGCAATGCATCGGCGCC
ATTTGTGGAGCAGCTCTTCTCCAGTCCCTGACACCGGAGAAGTTCCAC---AACACGCTG
GGT---------------------------------------------------------
------------------GTCACCACGGTCCACGAATTCCTCACCCCGACCCAGGGCTTC
GGCGTGGAGTTTTTCACCACTTTCACCTTGGTGCTGATCGTTTTCGGCGTCAGCGATGAA
AACCGCACCGATGTCAAG------------GGCTCGGCCCCTTTGGCCATTGGGCTGACT
GTC---ACCACGGCCATTTTGGCCACGGGAATGTATACCGGCGGCAGCTTGAATCCGGCG
CGCTCCTTGGGTCCGGCAGTCGTCACT---------------------------------
GGC---AAATTTGCCCTCCACTGGGTCTACTGGGCGGGTCCGATCGTCGGTGGCATGGTG
GCCGCCCTGCTCTAC---GAGAAAGTCTTCCGTGCCCGTTCC---------------GCC

GAGGAGGAGCAAGAATGGCGCGAAATGGAG---GACTATCAGTACCGCATTGCC------
AACGCCAAGGAGACC------------------------------------GAAATCACC
GGCGAACGAGTCGTCACCGTCGATGTTCAGAAGCAC------------------------
------------------------------------------------------------
------------------------------------------------------------
------------------------------------------------------------
------------------------------------------------------------
------------------------------------------------------------
------------------------------------------------------------
------------------------------------------------------------
------------------------------------------------------------
------------------------------------------------------------
------------------------------------------------------------
------------------------------------------------------------
------------------------------------------------------------
------------------------------------------------------------
------------------------------------------------------------
------------------------------------------------------------
------------------------------------------------------------
------------------------------------------------------------
------------------------------------------------------------
------------------------------------------------------------
------------------------------------------------------------
------
>NKDA01000001_Eulimnadia_texana_EglpL3
------------------------------------------------------------
------------------------------------------------------------
------------ATGGCCAGGTGC---AATAGAATGCGCAAC---ATCATTGGCTGTCAC
GAGTTCACC---GAGAACCGGGATGTCTGGAGAGCGATCCTGGCGGAATTTGTAGGAACT
ATGTTCCTGGTACTCATCGGATGCGCTTCCTGCGTTAACGGCTGGAACGAT---TCCTAC
AATCCGCATATTGTCCAAGTCGCACTGTCCTTCGGTATCACTATTGCCACTATGGCACAG
GTAATTGGTCACGTGAGCGGTTGTCACATCAACCCTGCAGTGACGGTGGCCATGTTGCTG
AGCGGCAAGATGAGCATCCTTCGCGCCATTTTCTATGTCCTTGCCCAATGCATAGGATCC
ATTTCCGGTGCTGCAATCCTGCAGGCACTGACACCTCCCGAATTGCAC---TCCAGTCTG
GGC---------------------------------------------------------
------------------GTCACAGTTTTACACCCAAAACTGAGTCCAACCCAGGGTTTC
GGCGTCGAATTTTTCGCCACGTTCACGCTGATCATCATCGTCTTCGGAGTCTGCGACGAG
AACCGCACCGACGTCAAG------------GGATCCGCGCCCTTGGCCATCGGACTCACC
GTC---TCCACGGCCATCTTGGCTATTGGCCAGTACACGGGAGGTAGTCTGAATCCGGCT
CGTTCGTTGGGACCCGCAGTTATCTGC---------------------------------
AAT---ACGTGGACGAATCATTGGGTATACTGGGCTGGACCTATTGTTGGTGGAATCGTG
GCTTCGATGTTGTAT---GACAGAGCATTCAGTGCACGAAAA---------GCTAATCAG
GACGAATCC------------GATAAATATATCGATTACCGATACCAGCCGGCC------
GACGGCAAGGCAAGGAACTG----------------------AAG------GAAATCATC
GCCGACCGAACTACGACCATT---------------------------------------
------------------------------------------------------------
------------------------------------------------------------
------------------------------------------------------------

------------------------------------------------------------
------------------------------------------------------------
------------------------------------------------------------
------------------------------------------------------------
------------------------------------------------------------
------------------------------------------------------------
------------------------------------------------------------
------------------------------------------------------------
------------------------------------------------------------
------------------------------------------------------------
------------------------------------------------------------
------------------------------------------------------------
------------------------------------------------------------
------------------------------------------------------------
------------------------------------------------------------
------------------------------------------------------------
------------------------------------------------------------
------
>JAIQCU010000047_Leptestheria_dahalacensis_EglpL3
------------------------------------------------------------
------------------------------------------------------------
------------ATGGCCAGGTGT---CAGAGAATGCGCAAT---ATCGTCGGCTGCCAC
GAATTCACC---GAGAATCGCGATGTGTGGCGGGCGATTCTGGCCGAATTCGTCGGTACC
ATGTTTCTGGTGCTGATCGGATGCGCGTCGTGCGTCAACGGATGGAACGAC---TCGTAC
AGTCCGCACATTGTCCAGGTGGCCATCTCCTTTGGAGTCACCATTGCTACCATGGCGCAG
GCAATCGGACACGTGAGCGGTTGCCACATCAATCCCGCAGTTACAGTAGCCATGCTGCTC
AGCGGGAAGATCAGCATTCTGCGGGGAATCTTCTACGTTGTTGCTCAATGTGTAGGCTCC
ATTTCCGGTGCTGCAATCCTACAGGCCTTGACACCTCCCGAGTTCCAC---TCGACACTG
GGC---------------------------------------------------------
------------------GTGACGTCTTTGCATCCCAAGCTGAGCCCGACTCAAGGTTTC
GGAGTCGAATTTTTCGCCACCTTCACCCTGGTGCTCATCGTCTTCGGCGTCTCTGATGAA
AACCGCAATGACGTCAAA------------GGATCAGCGCCTCTGGCCATCGGTCTTACC
GTC---ACAACATCCATTCTGGCCATTGGAATGTACACAGGCGGAAGTTTGAATCCGGCG
CGATCCTTGGGGCCTGCCATCATCAGT---------------------------------
AAC---TCCTGGGAGCATCACTGGGTGTACTGGGCCGGTCCTATCATCGGAGGTATCGTC
GCTTCCATGATGTAC---GACAAGGCGTTCCGGGCGCGGAAG---------GCCAACAAC
GACGACGAC------------GACGTCTACATCGATTACCGCTACAGACCCGCT------
GACGTCAAAGAGAAGGAACTG---------------------AAG------GAAATAATC
GCCGATAGAACGACCACAATT---------------------------------------
------------------------------------------------------------
------------------------------------------------------------
------------------------------------------------------------
------------------------------------------------------------
------------------------------------------------------------
------------------------------------------------------------
------------------------------------------------------------
------------------------------------------------------------
------------------------------------------------------------

------------------------------------------------------------
------------------------------------------------------------
------------------------------------------------------------
------------------------------------------------------------
------------------------------------------------------------
------------------------------------------------------------
------------------------------------------------------------
------------------------------------------------------------
------------------------------------------------------------
------------------------------------------------------------
------------------------------------------------------------
------
>NKDA01000003_Eulimnadia_texana_EglpL2
------------------------------------------------------------
------------------------------------------------------------
------------ATGGCGAAGTGCGGCACCAGAATGCGCAAC---ATCATTGGCTGCGGC
GAATTCTCATCGGCCAATCGGGACATTTGGCGCGCCATTTTGGCTGAATTTATCGGCACC
TTCTTCTTGGTGCTGATCGGTTGCGCCTCTTGCGTCAGTGGCTGGAACGAA---GATCAC
TCGCCCCACATCGTACAAGTGGCCCTGGCCTTCGGTGTCACTATTGCCACTATGGCACAG
GCAATTGGTCACGTGTCTGGCTGTCACATCAACCCTGCGGTTTCCATTGCGCTGCTGATC
ACGGGAAAGATCTCCATTCTTCGCACCATCTTCTACATCATCGCCCAATGCGTTGGATCC
ATCGCCGGAACCGCCGTCCTTCAGGCTCTGACTCCGGCCGACTTCCAC---TCCAACTTA
GGT---------------------------------------------------------
------------------ATCACTTCACTTCACCCCAAAGTGACTCCCAGCCAGGGTTTC
GGCGTCGAGTTCTTCGCTACTTTCGTCCTCGTCCTCGTCGTCTTCGCCGTTTGCGACGAC
AGGCGCGACGACATCAAG------------GGATCAGCTCCTCTGGCCATCGGTTTGACA
GTC---TCAGCTGCTGTCCTGTCTATCGCAAGCTACACTGGAGCTAGTTTGAACCCGGCC
CGTTCCTTCGGACCTGCTGTCATTAGC---------------------------------
AAC---TCTTGGGAAAACCACTGGGTATACTGGATCGGACCTATTATTGGTGGATCAGTA
GCGGCCATGATGTAC---CAGAAGGCCTTCCGCGCTCGCAAG------------TCCCCG
GAAGCGGAG------------GATAACCGCAGCGATTACCGCTATGAGCTAGCT------
GACGGCAAAAACAGC---------------------------AAA------CAA------
GCC------ACCAGTTCCGCT---------------------------------------
------------------------------------------------------------
------------------------------------------------------------
------------------------------------------------------------
------------------------------------------------------------
------------------------------------------------------------
------------------------------------------------------------
------------------------------------------------------------
------------------------------------------------------------
------------------------------------------------------------
------------------------------------------------------------
------------------------------------------------------------
------------------------------------------------------------
------------------------------------------------------------
------------------------------------------------------------
------------------------------------------------------------

------------------------------------------------------------
------------------------------------------------------------
------------------------------------------------------------
------------------------------------------------------------
------------------------------------------------------------
------
>JAIQCU010000075_Leptestheria_dahalacensis_EglpL2
------------------------------------------------------------
------------------------------------------------------------
------------ATGGCC---TTTTGGAGCAAGATGCGAGAG---ATTTTTGGCTGTGCG
GAATTCTCG---GCCAATCGCGATGTCTGGCGCGCCATTTTGGCGGAATTCGTCGGAACC
TTGTTCCTGGTGCTGATCGGATGCGCCTCGTGCGTCTCCGGATGGAATGAG---GATCAC
TCGCCGCATGTGGTTCAAGTGGCACTGGCTTTCGGTGTCACCATTGCTACTATTGCACAG
GCTATCGGGCACGTCTCTGGTTGCCATCTGAACCCGGCCGTGTCCGTCGCCATGTTGATC
ACCGGTAAAATCAGTATCCTCCGGACCATCACCTACAGTATCGCCCAGTGCTTGGGAGCC
ATTTCTGGCGCTGCGATCCTTCAGGCCCTGACTCCACCAGAATTCCAC---GCAACTCTG
GGT---------------------------------------------------------
------------------GTGACCTCCCTCCATCCGAAAGTGACAGTAGCGCAGGGCTTC
GGCATTGAATTCTTCGCCACGTTCATGTTGCTGCTCATCATCTTCGGCGTGTGCGACTCC
AAACGCGATGATATCAGA------------GGATCGGCGCCCTTGGCCATCGGTTTGACC
GTA---TCGGCAGCTGCACTGTCCATCGGTAGCTACACAGGCGCTAGTTTGAACCCAGCC
CGCTCTCTCGGCCCTGCAGTCATCGGC---------------------------------
AAC---TCCTGGGAACACCATTGGGTCTACTGGGTGGGTCCGATCGTGGGAGGCTCAGTG
GCCGGCATGGTGTAC---GAGAAGGCGTTCCGGGCACGCAAA---------------CCG
GAGGAAGAG------------GATAACCACAGCAACTACCGTTATGAATTAACC------
GATGTCAAGGATGTG---------------------------AAG------GAAGTCATC
GCTGACCGGACCACTTCAATC---------------------------------------
------------------------------------------------------------
------------------------------------------------------------
------------------------------------------------------------
------------------------------------------------------------
------------------------------------------------------------
------------------------------------------------------------
------------------------------------------------------------
------------------------------------------------------------
------------------------------------------------------------
------------------------------------------------------------
------------------------------------------------------------
------------------------------------------------------------
------------------------------------------------------------
------------------------------------------------------------
------------------------------------------------------------
------------------------------------------------------------
------------------------------------------------------------
------------------------------------------------------------
------------------------------------------------------------
------------------------------------------------------------
------

```
>NKDA01000001_Eulimnadia_texana_EglpL1
------------------------------------------------------------
------------------------------------------------------------
------------ATG------ATT---ACTAGATTTGGGAAT---ATTATCGGATTGGGA
GAGCTGTCG---AGCAGTACTAATCTGTGGCGTGCTGTTATCGCCGAATTCATCAGTACG
TTGCTGCTGGTCTTCTTCGGTTGCGCTTCCTGCGTATCGGAATGGAGAGAG---AACTAC
TCGCCCAACATTATCCAGATTGCGCTTACCTTTGGCATCGTTATTGCTACCATGGTTCAG
GTAATTGGTCATATCTCGGGAGGACACATCAACCCAGCAATAAGTGTAGCGATGTTACTG
GCCGGTAAAATGAGCGTTCTGCGGACGTTACTCTACATAGTCGCCCAAACGGTGGGAGCT
ATTGCGGGAGCTGGAATTCTGCAGGGACTGATTCCATCAGACATGCAG---TCCAACATC
GGA---------------------------------------------------------
------------------CTTACAAACATTCACCACAGACTGACTCCAGCACAGGGTTTC
GGAGCAGAATTTTTCGCGACTTTTTTGCTGGCGATTGTTGTTTTCGGGGTGACGGACGAC
AGCCGCAGGGACATCCAA------------GGATCTCGACCGCTCGCTATCGGCCTTGCT
GTA---ACGACTGCAATTCTGGCTATTGGTGACTTTACAGGGGGCGGTTTGAATCCAGCT
CGTTCTTTCGGCCCGGCTGTGGTCAAG---------------------------------
AAC---TTCTGGAACAATCACTGGGTATATTGGGTGGGACCAATGGCAGGGGCAGTGGCT
GCAGCGTTTATCTAT---CAGGGTATTTTTCGCTCGGGAACA------TCGGTGAGCGTG
GTGGAACCA------------GCAGGCGTAACTCTTCAGCAAGCAGTGTTAGCC------
AACATG---GACGCGGAT------------------------AAA---GAACCAACAACA
GTGGATCCTGCTACATCCATGATA------------------------------------
------------------------------------------------------------
------------------------------------------------------------
------------------------------------------------------------
------------------------------------------------------------
------------------------------------------------------------
------------------------------------------------------------
------------------------------------------------------------
------------------------------------------------------------
------------------------------------------------------------
------------------------------------------------------------
------------------------------------------------------------
------------------------------------------------------------
------------------------------------------------------------
------------------------------------------------------------
------------------------------------------------------------
------------------------------------------------------------
------------------------------------------------------------
------------------------------------------------------------
------------------------------------------------------------
------------------------------------------------------------
------
>EFX67799_Daphnia_pulex_Aqp12L
------------------------------------------------------------
------------------------ATG---GCTGCAGCTTTGATAGCCTCTTGTCTGTTG
ATTGCAGCTTCCTGCCTA---TCATCCGAACTGTTACGCATC---ATCATT---AATAAA
AATGTCGAG---------ACTCCCTTATCCAAACTTCTATGTCTGGAATTTATTGCTTCA
GCCGAGCTTTGTGCCGTTTGTTTTGAGTTGATTGTGGTTGCG------------------
```

------GAGGAGTACGGAATTTGGATGTATGCCCTGGTTCTGTTTCTTCTTACTCTGTGG
TGGGCAGCTCATTGGGGTGATGCGTCCGCTTGTCCTTATATTCATCTTGAAGCGTATCTC
GATGGTTCTATCCATTTCAGTCAAGCTTTCCTTGCTTCTTTGGCAGAAATAGCCGGAGGA
TTAGCTATTTACAAATACGTACAATATACATGGATTTAGAATTATCT---GGCCACATA
AAT---------------------------------------------------------
------AGATCAGTCTGGGACTGCGACGCAGACCTACACGTTCCAGCATTTCAGGGAGCT
TTGATCGAAGGTGTGGCAACTTTTCTG---------------TGTCGAATTGTGTCAAAG
CTTATCGCTGAGATTGAA------------CCGCGGTTTGGAGGTGTTCTCGATGCATTT
ATTGGAACTTCTTTAGTTGTAGCAGCTTGTGACTATTCTGGTGGATATTTCAACCCAGTG
TTGGCCACATCATTAAAGCTTAACTGTCGC---GGT------------------------
AATACTGTTGCAGAACACTTTGTCGTCTACTGGATTGGTTCGATTGCTGGATCAGTCGTT
TCATGGCGGGTTTAT------CCTTTCATTAAAGAAAATCTATTTCCTAAACCATCGACA
AAT------------AAAAAAGACCAA---------------------------------
------------------------------------------------------------
------------------------------------------------------------
------------------------------------------------------------
------------------------------------------------------------
------------------------------------------------------------
------------------------------------------------------------
------------------------------------------------------------
------------------------------------------------------------
------------------------------------------------------------
------------------------------------------------------------
------------------------------------------------------------
------------------------------------------------------------
------------------------------------------------------------
------------------------------------------------------------
------------------------------------------------------------
------------------------------------------------------------
------------------------------------------------------------
------------------------------------------------------------
------------------------------------------------------------
------------------------------------------------------------
------------------------------------------------------------
------------------------------------------------------------
------
>XM_046796963_Daphnia_pulicaria_Aqp12L
------------------------------------------------------------
------------------------ATG---GCTGCAGCTTTGATAGCCTCTTGTCTGTTG
ATTGCAGCTTCCTGCCTA---TCATCCGAACTGTTACGCATC---ATCATT---AATAAA
AATGTCGAG---------ACTCCCTTATCCAAACTTCTATGTCTGGAATTTATTGCTTCA
GCCGAGCTTTGTGCCGTTTGTTTTGAGTTGATTGTGGTTGCG------------------
------GAGGAGTACGGAATTTGGATGTATGCCCTGGTTCTGTTTCTTCTTACTCTGTGG
TGGGCAGCTCATTGGGGTGATGCGTCCGCTTGTCCTTATATTCATCTTGAAGCGTATCTC
GATGGTTCTATCCATTTCAGTCAAGCTTTCCTTGCTTCTTTGGCAGAAATAGCCGGAGGA
TTAGCTATTTACAAATACGTACAATATACATGGATTTAGAATTATCT---GGCCACATA
AAT---------------------------------------------------------
------AGATCAGTCTGGGACTGCGACGCAGACCTACACGTTCCAGCATTTCAGGGAGCT

TTGATCGAAGGTGTGGCAACTTTTCTG---------------TGTCGAATTGTGTCAAAG
CTTATCGCTGAGATTGAA------------CCGCGGTTTGGAGGTGTTCTCGATGCATTT
ATTGGAACTTCTTTAGTTGTAGCAGCTTGTGACTATTCTGGTGGATATTTCAACCCAGTG
TTGGCCACATCATTAAAGCTTAACTGTCGC---GGT------------------------
AATACTGTTGCAGAACACTTTGTCGTCTACTGGATTGGTTCGATTGCTGGATCAGTCGTT
TCATGGCGGGTTTAT------CCTTTCATTAAAGAAAATCTATTTCCTAAACCATCGACA
AAT------------AAAAAAGACCAA---------------------------------
------------------------------------------------------------
------------------------------------------------------------
------------------------------------------------------------
------------------------------------------------------------
------------------------------------------------------------
------------------------------------------------------------
------------------------------------------------------------
------------------------------------------------------------
------------------------------------------------------------
------------------------------------------------------------
------------------------------------------------------------
------------------------------------------------------------
------------------------------------------------------------
------------------------------------------------------------
------------------------------------------------------------
------------------------------------------------------------
------------------------------------------------------------
------------------------------------------------------------
------------------------------------------------------------
------------------------------------------------------------
------------------------------------------------------------
------------------------------------------------------------
------
>JAACYE010000006_Daphnia_obtusa_Aqp12L
------------------------------------------------------------
------------------------ATG---GCTGCAACTTTGATAGCCTCTTGCCTGTTG
ATTGCAGCTTCCTGCCTA---TCATCAGAACTGTTACGCCTC---ATCATT---AATAAA
AATGTCGAG---------ACTCCTTTATCCAAACTTCTATGTCTGGAATTTATTGCTTCA
GCCGAGCTTTGTGCCGTTTGTTTTGAGTTGATTGTGGTTGCG------------------
------GAGGAGTACGGAATTTGGATGTATGCCCTGGTTCTGTTTCTTCTTACTCTGTGG
TGGGCAGCCCATTGGGGTGATGCGTCCGCTTGTCCTTATATTCATCTTGAAGCGTATCTC
GATGGTTCTATCCATTTCAGTCAAGCTTTTCTTGTTTCGTTGGCAGAAATAGCCGGAGGA
TTAGCTATTTACAAGTACGTACAATATACATGGGATTTAGAATTATCT---GGCCACATA
AAT---------------------------------------------------------
------AGATCAGTCTGGGACTGCGACGCAGACCTACACGTACCAGCATTTCAGGGAGCT
TTGATCGAAGGTGTGGCAACTTTTCTG---------------TGTCGAATTGTGTCAAAG
CTTATCGCTGAGATTGAA------------CCCCGGTTTGGAGGTGTTCTCGATGCATTT
ATTGGAACTTCTTTAGTTGTAGCAGCTTGTGACTATTCTGGTGGATATTTCAACCCAGTG
TTGGCCACATCATTAAAGCTGAACTGTCGC---GGT------------------------
AATACTGTTGCAGAACACTTTGTCGTCTACTGGATTGGTTCGATTGCTGGATCAGTCGTT
TCATGGCGGGTTTAT------CCTTTTATTAAAGAAAATCTTTTTCCTAAACCATTGACA

AAT------------AAAAAAGACCAA---------------------------------
------------------------------------------------------------
------------------------------------------------------------
------------------------------------------------------------
------------------------------------------------------------
------------------------------------------------------------
------------------------------------------------------------
------------------------------------------------------------
------------------------------------------------------------
------------------------------------------------------------
------------------------------------------------------------
------------------------------------------------------------
------------------------------------------------------------
------------------------------------------------------------
------------------------------------------------------------
------------------------------------------------------------
------------------------------------------------------------
------------------------------------------------------------
------------------------------------------------------------
------------------------------------------------------------
------------------------------------------------------------
------------------------------------------------------------
------------------------------------------------------------
------
>HAFN01005048_Daphnia_galeata_Aqp12L
------------------------------------------------------------
------------------------ATG---GCTTCTGCTTTGATAGCTTCCTGTCTGTTA
ATTGCAGCATCCTGTCTA---TCGTCGGAATTGATACGCATC---ATTATT---AATAAA
AATGTCGAA---------ACTCCGTTATCCAAACTTCTATGTCTGGAATTTATTGCTTCG
GCTGAACTTTGTGCTGTTTGTTTTGAGTTGATTGTGGTTGCG------------------
------GAAGAGTACGGAATTTGGATGTATGCTCTGATTTTGTTTCTTCTTACTCTGTGG
TGGGCAGCTCATTGGGCTGATGCATCCGCTTGTCCTTATATCCATCTTGAAGCTTACCTT
GATGGTTCTGTCCACTTCAGTCAAGCTTTTCTGGCTACGTTGGCAGAAATAGCCGGAGGT
TTAGCTATTTACAAATACGTGCAATATACATGGGATTTACAATTATCT---GGCCACATA
AAT---------------------------------------------------------
------AGGTCAGTCTGGGATTGCGACGCAGACCTACACGTACCAGCATTTCAGGGGGCA
TTGATTGAAGGTGTAGCGACTTTTCTG---------------TGTCGAATTATGTCAAAG
ATTATTGCTGAGTTTGAG------------CCAAGATTTGGAGGTGTTCTGGATGCCTTT
ATAGGAACTTCTTTAGTTGTCGCCGCTTGCGATTATTCTGGTGGATACTTTAACCCAGTG
TTGGCCACATCACTTAAGTTAAACTGTCGC---GGA------------------------
AATACTGTTGCAGAACACTTTGTCGTCTACTGGGTTGGCTCGATTGGTGGATCAGTCGTG
TCTTGGTGGGTTTAT------CCTTTCGTTAAAGAAAATCTTTTTTCTAAAACATCGACA
GAT------------AAAAAACAGAAA---------------------------------
------------------------------------------------------------
------------------------------------------------------------
------------------------------------------------------------
------------------------------------------------------------
------------------------------------------------------------

------------------------------------------------------------
------------------------------------------------------------
------------------------------------------------------------
------------------------------------------------------------
------------------------------------------------------------
------------------------------------------------------------
------------------------------------------------------------
------------------------------------------------------------
------------------------------------------------------------
------------------------------------------------------------
------------------------------------------------------------
------------------------------------------------------------
------------------------------------------------------------
------------------------------------------------------------
------------------------------------------------------------
------------------------------------------------------------
------------------------------------------------------------
------
>JAAVJA010010846_Daphnia_dubai_Aqp12L
------------------------------------------------------------
------------------------ATG---GCTGCTGCTTTGATAGCTTCTTGTCTGTTG
ATTGCAGCTTCCTGTCTA---TCATCAGAATTGTTACGCATC---ATTGTA---AACAAA
AATGTCGAG---------ACTCCCTTATCTAAACTTCTGTGTCTGGAATTTATTGCTTCA
GCTGAGCTTTGTGCCGTATGTTTTGAGTTGATTGTGGTTGCG------------------
------GAGGAGTACGGAATATGGATGTATGCTCTGATTCTGTTTCTTCTTACTTTGTGG
TGGGCAGCTCATTGGGGTGATGCATCCGCTTGTCCTTATATTCATCTTGAAGCATACCTT
GATGGATCAATCCACTTCAGTCAGGCTTTTCTGGCTTCATTGGCAGAAATAGCTGGAGGT
TTAGCTATTTATAGGTACGTGCAATATACATGGGATTTACAATTATCT---GGCCACATA
AAC---------------------------------------------------------
------AGATCCGTTTGGGACTGCGACGCAGACTTACATGTACCGGCATTTCAGGGGGCA
ATGATCGAAGGTGTGGCGACTTTTCTG---------------TGTCGAATTATGTCAAAG
ATCATCGCTGAGATT---------------CCCCGTTTTGGAGGTGTTCTCGATGCCTTT
ATTGGAACTTCTTTAGTTGTCGCCGCTTGCGACTATTCTGGTGGATACTTCAACCCAGTG
TTGGCCACATCACTAAAGCTGAACTGCCGC---GGC------------------------
AATACTGTTACAGAACACTTT---GTCTATTGGATTGGTTCGATCGCTGGATCAATCGTG
TCATGGTGGGTTTAT------CCTTTCGTNGATGAC---ATAAATTGTAGGTACCGGCAT
---------------TTCAGGGGGCAA---------------------------------
------------------------------------------------------------
------------------------------------------------------------
------------------------------------------------------------
------------------------------------------------------------
------------------------------------------------------------
------------------------------------------------------------
------------------------------------------------------------
------------------------------------------------------------
------------------------------------------------------------
------------------------------------------------------------
------------------------------------------------------------

------------------------------------------------------------
------------------------------------------------------------
------------------------------------------------------------
------------------------------------------------------------
------------------------------------------------------------
------------------------------------------------------------
------------------------------------------------------------
------------------------------------------------------------
------------------------------------------------------------
------------------------------------------------------------
------------------------------------------------------------
------
>CM023191_Daphnia_carinata_Aqp12L1
------------------------------------------------------------
------------------------ATG---GCTGGTTCTTTAATAGCGTCTTGTTTGTTG
ATTGCAGCTTGCTGTTTA---TTTTCTCAGCTCATCCGTGTG---ATTGTT---GACAAA
AATGTTTCG---------ACTCCCTTGTCCAAGCTTCTATGCCTGGAATTTGTTGCTTCA
GCTGAGCTTTGTGCCGCTTGCTTCGAGTTGATTGTGGTTGCG------------------
------GAAGAATATGGCATTTGGATGTACGCTTTGATTTTATTTCTTCTTACTCTGTGG
TGGGCCGCCCATTGGGGTGATGCATCTGCTTGTCCTTACATTCATCTTGAAGCTTATCTT
GATGGTTCCATCAGTTTTAGCCAAGCTTTTCTCGTTTCCTTGGCAGAAATAGCCGGGGGC
CTGGCTATATACAGGTACGTTCAGTACACTTGGGATTTGCAATTATCG---GCACAC---
AAG---------------------------------------------------------
------AGATCGGTCTGGGATTGCGACGCCGACCTACATGTATCTGCATTTTACGGAGCA
TTGGTCGAAGGTGTCGCGACTTTTCTG---------------TGTAGGATCGTATCAAGA
ACTATTGCTGAGCTCGAA------------CCTCGTTTTGGAAGTGCCCTTGATGCCTTC
ATTGGTACTTCCTTAGTTGTTGCAGCCTGCGACTATTCTGGCGGATATTTTAACCCAGTA
TTGGCAACATCTCTCAAGCTGAACTGTCGC---GGA-------------------------
AATACTTTTGCGGAGCACTTCGTCGTCTATTGGATTGGTTCGATCGCCGGATCTTTTCTT
TCGTGGTGGACCTAT------CCTATTGTTAAAGAAAGTCTATTTCCCATACAAACGAAG
GAT------------AAAAAATATAAA---------------------------------
------------------------------------------------------------
------------------------------------------------------------
------------------------------------------------------------
------------------------------------------------------------
------------------------------------------------------------
------------------------------------------------------------
------------------------------------------------------------
------------------------------------------------------------
------------------------------------------------------------
------------------------------------------------------------
------------------------------------------------------------
------------------------------------------------------------
------------------------------------------------------------
------------------------------------------------------------
------------------------------------------------------------
------------------------------------------------------------
------------------------------------------------------------

------------------------------------------------------------
------------------------------------------------------------
------------------------------------------------------------
------------------------------------------------------------
------------------------------------------------------------
------
>KAI9550673_Daphnia_sinensis_Aqp12L1
------------------------------------------------------------
------------------------ATG---GCTGGTTCTTTAATAGCGTCTTGTTTGTTG
ATTGCAGCTTGCTGTTTA---TTTTCTCAGCTCATCCGTGTG---ATTGTT---GACAAA
AATGTTTCG---------ACTCCCTTGTCCAAGCTTCTATGCCTGGAATTTGTTGCTTCA
GCTGAGCTTTGTGCCGCTTGCTTCGAGTTGATTGTGGTTGCG------------------
------GAAGAATATGGCATTTGGATGTACGCTTTGATTTTATTTCTTCTTACTCTGTGG
TGGGCCGCCCATTGGGGTGATGCATCTGCTTGTCCTTACATTCATCTTGAAGCTTATCTT
GATGGTTCCATCAGTTTTAGCCAAGCTTTTCTCGTTTCCTTGGCAGAAATAGCCGGGGGC
CTGGCTATATACAGGTACGTTCAGTACACTTGGGATTTGCAATTATCG---GCACAC---
AAG---------------------------------------------------------
------AGATCGGTCTGGGATTGCGACGCCGACCTACATGTATCTGCATTTTACGGAGCA
TTGGTCGAAGGTGTCGCGACTTTTCTG---------------TGTAGGATCGTATCAAGA
ACTATTGCTGAGCTCGAA------------CCTCGTTTTGGAAGTGCCCTTGATGCCTTC
ATTGGTACTTCCTTAGTTGTTGCAGCCTGCGACTATTCTGGCGGATATTTTAACCCAGTA
TTGGCAACATCTCTCAAGCTGAACTGTCGC---GGA------------------------
AATACTTTTGCGGAGCACTTCGTCGTCTATTGGATTGGTTCGATCGCCGGATCTTTTCTT
TCGTGGTGGACCTAT------CCTATTGTTAAAGAAAGTCTATTTCCCATACAAACGAAG
GAT------------AAAAAATATAAA---------------------------------
------------------------------------------------------------
------------------------------------------------------------
------------------------------------------------------------
------------------------------------------------------------
------------------------------------------------------------
------------------------------------------------------------
------------------------------------------------------------
------------------------------------------------------------
------------------------------------------------------------
------------------------------------------------------------
------------------------------------------------------------
------------------------------------------------------------
------------------------------------------------------------
------------------------------------------------------------
------------------------------------------------------------
------------------------------------------------------------
------------------------------------------------------------
------------------------------------------------------------
------------------------------------------------------------
------------------------------------------------------------
------------------------------------------------------------
------------------------------------------------------------
------

>CM023191_Daphnia_carinata_Aqp12L2
------------------------------------------------------------
------------------------ATG---GCTGGTTCTTTAATAGCGTCTTGTTTGTTG
ATTGCAGCTTGCTGTTTA---TTTTCTCAGCTCATCCGTGTG---ATTGTT---GACAAA
AATGTTTCG---------ACTCCCTTGTCCAAGCTTCTATGCCTGGAATTTGTTGCTTCA
GCTGAGCTTTGTGCCGCTTGCTTCGAGTTGATTGTGGTTGCG------------------
------GAAGAATATGGCATTTGGATGTACGCTTTGATTTTATTTCTTCTTACTCTGTGG
TGGGCCGCCCATTGGGGTGATGCATCTGCTTGTCCTTACATTCATCTTGAAGCTTATCTT
GATGGTTCCATCAGTTTTAGCCAAGCTTTTCTCGTTTCCTTGGCAGAAATAGCCGGGGGC
CTGGCTATATACAGGTACGTTCAGTACACTTGGGATTTGCAATTATCG---GCACAC---
AAG---------------------------------------------------------
------AGATCGGTCTGGGATTGCGACGCCGACCTACATGTATCTGCATTTTACGGAGCA
TTGGTCGAAGGTGTCGCGACTTTTCTG---------------TGTAGGATCGTATCAAGA
ACTATTGCTGAGCTCGAA------------CCTCGTTTTGGAAGTGCCCTTGATGCCTTC
ATTGGTACTTCCTTAGTTGTTGCAGCCTGCGACTATTCTGGCGGATATTTTAACCCAGTA
TTGGCAACATCTCTCAAGCTGAACTGTCGC---GGA------------------------
AATACTTTTGCGGAGCACTTCGTCGTCTATTGGATTGGTTCGATCGCCGGATCTTTTCTT
TCGTGGTGGACCTAT------CCTATTGTTAAAGAAAGTCTATTTCCCATACAAACGAAG
GAT------------AAAAAAAATAAA---------------------------------
------------------------------------------------------------
------------------------------------------------------------
------------------------------------------------------------
------------------------------------------------------------
------------------------------------------------------------
------------------------------------------------------------
------------------------------------------------------------
------------------------------------------------------------
------------------------------------------------------------
------------------------------------------------------------
------------------------------------------------------------
------------------------------------------------------------
------------------------------------------------------------
------------------------------------------------------------
------------------------------------------------------------
------------------------------------------------------------
------------------------------------------------------------
------------------------------------------------------------
------------------------------------------------------------
------------------------------------------------------------
------------------------------------------------------------
------------------------------------------------------------
------
>KAI9559478_Daphnia_sinensis_Aqp12L2
------------------------------------------------------------
------------------------ATG---GCTGGTTCTTTAATAGCGTCTTGTTTGTTG
ATTGCAGCTTGCTGTTTA---TTTTCTCAGCTCATCCGTGTG---ATTGTT---GACAAA
AATGTTTCG---------ACTCCCTTGTCCAAGCTTCTATGCCTGGAATTTGTTGCTTCA
GCTGAGCTTTGTGCCGCTTGCTTCGAGTTGATTGTGGTTGCG------------------

------GAAGAATATGGCATTTGGATGTACGCTTTGATTTTATTTCTTCTTACTCTGTGG
TGGGCCGCCCATTGGGGTGATGCATCTGCTTGTCCTTACATTCATCTTGAAGCTTATCTT
GATGGTTCCATCAGTTTTAGCCAAGCTTTTCTCGTTTCCTTGGCAGAAATAGCCGGGGGC
CTGGCTATATACAGGTACGTTCAGTACACTTGGGATTTGCAATTATCG---GCACAC---
AAG---------------------------------------------------------
------AGATCGGTCTGGGATTGCGACGCCGACCTACATGTATCTGCATTTTACGGAGCA
TTGGTCGAAGGTGTCGCGACTTTTCTG---------------TGTAGGATCGTATCAAGA
ACTATTGCTGAGCTCGAA------------CCTCGTTTTGGAAGTGCCCTTGATGCCTTC
ATTGGTACTTCCTTAGTTGTTGCAGCCTGCGACTATTCTGGCGGATATTTTAACCCAGTA
TTGGCAACATCTCTCAAGCTGAACTGTCGC---GGA------------------------
AATACTTTTGCGGAGCACTTCGTCGTCTATTGGATTGGTTCGATCGCCGGATCTTTTCTT
TCGTGGTGGACCTAT------CCTATTGTTAAAGAAAGTCTATTTCCCATACAAACGAAG
GAT------------AAAAAAAATAAA---------------------------------
------------------------------------------------------------
------------------------------------------------------------
------------------------------------------------------------
------------------------------------------------------------
------------------------------------------------------------
------------------------------------------------------------
------------------------------------------------------------
------------------------------------------------------------
------------------------------------------------------------
------------------------------------------------------------
------------------------------------------------------------
------------------------------------------------------------
------------------------------------------------------------
------------------------------------------------------------
------------------------------------------------------------
------------------------------------------------------------
------------------------------------------------------------
------------------------------------------------------------
------------------------------------------------------------
------------------------------------------------------------
------------------------------------------------------------
------------------------------------------------------------
------
>XM_032928782_Daphnia_magna_Aqp12L
------------------------------------------------------------
------------------------ATG---GCTGGTTCTTTGATAGCGTCTTGTCTGCTA
ATTGCAGCTTGCTGTTTA---TTTTCTCAGCTCGTCCGTGTG---ATTATT---AACAAA
AAAGTTTCG---------ACTCCCTTGTCCAAGCTTCTATGTCTGGAATTTGTTGCTTCA
GCTGAGCTTTGTGCTGCCTGCTTCGAGTTGATTGTGGTTGCG------------------
------GACGACTATGGAATTTGGATGTACGCTGTGATATTATTTCTTCTTACTCTGTGG
TGGGCCACCCATTGGGGTGATGCATCTGCTTGTCCTTACATTCATCTTGAAGCATATCTC
GATGGTTCTATCAGTTTTAGCCAAGCTTTTCTTGTCTCCTTGGCAGAAATAGCCGGGGGC
CTGGCTATCTACAGGTACGTTCAGTACACTTGGGATTTACAATTATCG---GTACAC---
AAT---------------------------------------------------------
------AGATCGGTCTGGGATTGCGACGCCGATCTACACGTATCTGCATTTCACGGGGCA

```
TTGGTCGAAGGTGTCGCGACCTTTCTG---------------TGTAGGATCGTATCAAGG
ACTATTGCTGAGCTCGAA------------CCTCGTTTTGGAACTGCTCTCGATGCCTTC
ATTGGTACTTCCTTAGTTGTTGCAGCCTGCGACTATTCTGGTGGATATTTCAACCCAGTA
TTGGCAACATCTCTCAAGCTGAACTGTCGT---GGA------------------------
CATACTTTTGCAGAGCACTTCATTGTCTATTGGATTGGTTCGATCGCTGGATCCTTCCTT
TCGTGGTGGACCTAT------CCTGTCGTTCAAGGAAGTCTATTTCCCATACAATCAATG
GAT------------AAAAAACATAAA---------------------------------
------------------------------------------------------------
------------------------------------------------------------
------------------------------------------------------------
------------------------------------------------------------
------------------------------------------------------------
------------------------------------------------------------
------------------------------------------------------------
------------------------------------------------------------
------------------------------------------------------------
------------------------------------------------------------
------------------------------------------------------------
------------------------------------------------------------
------------------------------------------------------------
------------------------------------------------------------
------------------------------------------------------------
------------------------------------------------------------
------------------------------------------------------------
------------------------------------------------------------
------------------------------------------------------------
------------------------------------------------------------
------------------------------------------------------------
------------------------------------------------------------
------
>JAKUCG010000177_Ceriodaphnia_dubia_Aqp12L
------------------------------------------------------------
------------------------ATG---GTTGCTGCACTGATAGCATCCTGTCTGTTG
ATTGCAGTTTCCTGCTTA---GTATCGGAATTGCTGCGCATC---ATCATT---AATAAA
AACGTCGTG---------ACTCCCTTATCCAAACTTCTGTGTCTGGAGTTCATAGCATCT
GCGGAGCTTTGCGCCGTCTGTTTCGAGTTGATTGTGGTGGCG------------------
------GAGGAATACGGGATATGGATGTATGCACTTGCCCTGTTCTTACTTACAATATGG
TGGGCTGGTCATTGGGGAGAAGCAGCCGCTTGTCCTTACATTCATATTGAAGCATATCTC
GATGGCTCCACCGAATTTAGTCAAGCCTTTCTTGCCTCCCTAGCAGAAATAGCCGGTGGT
TTGGCTATCTATAGGTATGTTCAATATACATGGGATTTGCAATTATCGGGGAGTCACTTG
ACT---------------------------------------------------------
------AGATCAGTCTGGGAATGCGAAGCCGATCTCCACGTACCTGCTCTTCACGGAGCT
TTGGTTGAAGGAGTGGCTACTTTCCTT---------------TGCCGATTGGTGTCGAAG
ACCATTTCTGAGCTGGAA------------CCTCGCTTTGGGGGCGCTTTAGATGCTTTT
ATCGGCACTTCTTTAGTCGTTGCCGCTTGCGACTATTCCGGTGGGTATTTCAACCCTGTC
TTGGCCACATCACTGAAGCTTAACTGCCCA---GGT------------------------
AATACTCTGATGGAACACTTTGTGGTCTATTGGGTTGGCTCAATTGTTGGTTCCGTCGCT
TCTTGGTGGGTATAT------CCTTTGATTCAAAAACCCTTATTGTTTCAACTGATAAAA
```

AAGATAATT------AGAAATAGACAT---------------------------------
------------------------------------------------------------
------------------------------------------------------------
------------------------------------------------------------
------------------------------------------------------------
------------------------------------------------------------
------------------------------------------------------------
------------------------------------------------------------
------------------------------------------------------------
------------------------------------------------------------
------------------------------------------------------------
------------------------------------------------------------
------------------------------------------------------------
------------------------------------------------------------
------------------------------------------------------------
------------------------------------------------------------
------------------------------------------------------------
------------------------------------------------------------
------------------------------------------------------------
------------------------------------------------------------
------------------------------------------------------------
------------------------------------------------------------
------------------------------------------------------------
------
>CM057928_Chydorus_sphaericus_Aqp12L
------------------------------------------------------------
------------------------ATG---GTAGCTCCTTTGATTGCTTCCTGCCTGTTG
ATGGCCGTGTCCTGTTTG---GTCGCAGAACTTGCCCGCATT---CTGGTC---AACAAA
CCATCTGGA------ATACCACCGTTGACCAAAATGCTTTTCCTGGAGTTCATAGCCTCC
GCAGAGCTCTGCGCCGTCTGTTTTGAGCTTATCATCGTCGCT------------------
------GAGGTGTACGGCGTGTGGATGTATGCCGTTCTGCTCTTCCTGCTGACACTCTGG
TGGGCAGCTCATTGGGGCGACGCATCCGCCTGCCCTTACATCCATCTCGAGGCCTATCTG
GATGGATCCAGTACTTTTAGCGACAGCCTGTTGGCTTCCTTGGCGGAATTGGCGGGCGGT
TTGGCCATTTACAGGTATGTGCAGTATACGTGGGATTTGCAACTATCCGGAAGCCATGTA
GCC---------------------------------------------------------
------AAATCTGTATGGGATTGCGAAGCGGATTTACACGTACCTGCTCTTCAAGGAATG
TTGATTGAAGGTGTAGCCACTTTTCTC---------------TGCCGGATCGTATCCAAA
AGCATTGCTGAGCTGGAA------------CCTCGTTTTGGCGGAACGATGGACGCATTT
ATAGGAACAGCTTTGGTCGTTGCTGCTTGCGACTATTCTGGCGGATACTTCAATCCCGTG
CTGGCTACTTCTCTCAAACTCAACTGCCAA---GGC------------------------
AACACGCTAACCGAGCATTTGGTTGTCTATTGGATCGGCTCCATTGCCGGATCTACTGCC
GCCTTTTATCTATTCAAGAAGCCTATCATCCACGATAAATTAGTTGGGAGCACACCTTCT
CCGATCAAGGAA---AAAGTCAAT------------------------------------
------------------------------------------------------------
------------------------------------------------------------
------------------------------------------------------------
------------------------------------------------------------
------------------------------------------------------------

------------------------------------------------------------
------------------------------------------------------------
------------------------------------------------------------
------------------------------------------------------------
------------------------------------------------------------
------------------------------------------------------------
------------------------------------------------------------
------------------------------------------------------------
------------------------------------------------------------
------------------------------------------------------------
------------------------------------------------------------
------------------------------------------------------------
------------------------------------------------------------
------------------------------------------------------------
------------------------------------------------------------
------------------------------------------------------------
------------------------------------------------------------
------
>GKAM01008610_Podon_leuckartii_Aqp12L1
------------------------------------------------------------
------------------------ATG---GCAGTCCCTTTAATTGTTTCCTGCTTATTC
ATAGCATTGTCTTGTTTG---ATTGCTGAATTAGTTCGAATT---GTGATC---AACAAA
GTCCTTGCG------GATAAGCCTCTGCCTCGCGTGCTGTGTCACGAGTTCGTAGCGACG
GCCGAACTATGCGCCGTTTGCTTCGAAATGGCATCGTTGCC-------------------
------GATGTATACGGTGTGTGGACGTACGCCGTGCTGCTGTTCGTTTTAACTTTGTGG
TGGGCTGCACACTGGCAAGATGCGGCGGCATGTCCTTACGTTCACGTAGAAAATTATTTG
GAAGGAACGAATGACCTCAGTTACTCAACATTATGCTGTTTGGCCGAAATGGCTGGTGGA
TTTGCCATCTATAGGTACGTGCAGTACACATGGAGTCTGCAGTTATCAACAAGTCATATA
GCG---------------------------------------------------------
------AAATCGGTGTGGGATTGTGAGGCAGATCTCCATGTACCCGCGCTTCAAGGTGCT
ATTGTCGAATGCGTTGCCACGATGCTG---------------TGTCGATTAACTTCAAAA
ATGATTGCAGCAGTGGAG------------CCCAAGTTCGGTGGAAGTATTGATGCTTTC
ATCGGCACTTCTATGGTCGTCGCCGCTTTGGATTATTCCGGCGGTTATTTCAATCCTGCA
CTAGCGACATCGCTCAAATTGAATTGCCGG---GGT------------------------
AACACCGTCATGGAACATGTCGTCGTGTATTGGATCGGATCTTTGGCTGGATCGGCCCTA
GCTTGGTGGCTGTTCCAAAAATCTATCTTCCAGGATACGTTGATCGTCTCACTCGGTGGC
AAACAGGAGGAGCTGAAACAACCGCCGAAACTGGTCGACAATAAAACGCCCAAGCCTCAA
AAGAAAGATTCCAAGCAAAAAACAAAAACGAAT---------------------------
------------------------------------------------------------
------------------------------------------------------------
------------------------------------------------------------
------------------------------------------------------------
------------------------------------------------------------
------------------------------------------------------------
------------------------------------------------------------
------------------------------------------------------------
------------------------------------------------------------
------------------------------------------------------------

------------------------------------------------------------
------------------------------------------------------------
------------------------------------------------------------
------------------------------------------------------------
------------------------------------------------------------
------------------------------------------------------------
------------------------------------------------------------
------------------------------------------------------------
------------------------------------------------------------
------------------------------------------------------------
------------------------------------------------------------
------
>GKAG01009778_Evadne_nordmanni_Aqp12L1
------------------------------------------------------------
------------------------ATG---GCAGTCCCTTTAATTGTTTCCTGCTTATTC
ATAGCATTGTCTTGTTTG---ATTGCTGAATTAGTTCGAATT---GTGATC---AACAAA
GTCCTTGCG------GATAAGCCTCTGCCTCGCGTGCTGTGTCACGAGTTCGTAGCGACG
GCCGAACTATGCGCCGTTTGCTTCGAAATGGGCATCGTTGCC------------------
------GATGTATACGGTGTGTGGACGTACGCCGTGCTGCTGTTCGTTTTAACTTTGTGG
TGGGCTGCACACTGGCAAGATGCGGCGGCATGTCCTTACGTTCACGTAGAAAATTATTTG
GAAGGAACGAATGACCTCAGTTACTCAACATTATGCTGTTTGGCCGAAATGGCTGGTGGA
TTTGCCATCTATAGGTACGTGCAGTACACATGGAGTCTGCAGTTATCAACAAGTCATATA
GCG---------------------------------------------------------
------AAATCGGTGTGGGATTGTGAGGCAGATCTCCATGTACCCGCGCTTCAAGGTGCT
ATTGTCGAATGCGTTGCCACGATGCTG---------------TGTCGATTAACTTCAAAA
ATGATTGCAGCAGTGGAG------------CCCAAGTTCGGTGGAAGTATTGATGCTTTC
ATCGGCACTTCTATGGTCGTCGCCGCTTTGGATTATTCCGGCGGTTATTTCAATCCTGCA
CTAGCGACATCGCTCAAATTGAATTGCCGG---GGT------------------------
AACACCGTCATGGAACATGTCGTCGTGTATTGGATCGGATCTTTGGCTGGATCGGCCCTA
GCTTGGTGGCTGTTCCAAAAATCTATCTTCCAGGATACGTTGATCGTCTCACTCGGTGGC
AAACAGGAGGAGCTGAAACAACCGCCGAAACTGGTCGACAATAAAACGCCCAAGCCTCAA
AAGAAAGATTCCAAGCAAAAAACAAAAACGAAT---------------------------
------------------------------------------------------------
------------------------------------------------------------
------------------------------------------------------------
------------------------------------------------------------
------------------------------------------------------------
------------------------------------------------------------
------------------------------------------------------------
------------------------------------------------------------
------------------------------------------------------------
------------------------------------------------------------
------------------------------------------------------------
------------------------------------------------------------
------------------------------------------------------------
------------------------------------------------------------
------------------------------------------------------------
------------------------------------------------------------

------------------------------------------------------------
------------------------------------------------------------
------------------------------------------------------------
------------------------------------------------------------
------------------------------------------------------------
------
>GKAM01008344_Podon_leuckartii_Aqp12L2
------------------------------------------------------------
------------------------ATG---GCAGTTAGCATACTTGTTTCGTGTTTGCTT
ATAGCAATGTCTAGTCTG---ATTGCTGAATTATTTCGAATT---GTGATT---AACAAA
ACTGTGCAC---------AACCCTTTACACCGTGTGCTATTTATGGAATTTGTTGCAACC
GCAGAATTATGCGCTGTATGTTTTGAGCTGATTATTGTTGCG------------------
------GAGGTGTACGGCATTTGGGTTTACGCCGGCCTTCTATTCTTATTAACTCTGTGG
TGGGCTGCGCATTGGCAAGATGCTGCTGCATGTCCCTACATCCACGTCGAAGATTACTTG
CAAGGAACGAGTACATTCAATCACACTGTTCTATGCTGCCTAGCTGAATTGGCTGGGGGT
TTAGCAATTTATAGATATGTCCAATATACCTGGAGTTTGCAATTATCAACCAGTCACATA
GCT---------------------------------------------------------
------AAATCCGTTTGGGATTGCGACGCAGATCTTCAAGTACCAGCTCTTCAAGGAGCC
ATTGTTGAAGGTATAGCAACAATGCTG---------------TGTCGCTTAACTACCAAA
ATGATTGCGACAATGGAG------------CCAAAATACGCTGGGAGCATTGAAGCATTC
ATCGGAACTTCTTTGGTCGTAGCAGCTGTGGATTATTCCGGCGGCTATTTCAATCCTGTA
CTGGCAACATCGCTGAAGTTCAACTGTCGT---GGA------------------------
AACACCTTTATTGAACACTTGATCGTTTACTGGATCGGATCTCTTGCCGGATCTGTTCTA
TCTTGGTGGTTATTCCAAAAACCTTGGTTCCAGCAAACTATATTGGATCCATTAGCTTCC
AAACCAAAAGAAACGCCCACAAAACCCGCACAATCCCCACCCAAA---CCAAAAAGGAAA
CCTTCCAATCCAAAAGAAAAAGTCAAAAGTAAAAAGGAA---------------------
------------------------------------------------------------
------------------------------------------------------------
------------------------------------------------------------
------------------------------------------------------------
------------------------------------------------------------
------------------------------------------------------------
------------------------------------------------------------
------------------------------------------------------------
------------------------------------------------------------
------------------------------------------------------------
------------------------------------------------------------
------------------------------------------------------------
------------------------------------------------------------
------------------------------------------------------------
------------------------------------------------------------
------------------------------------------------------------
------------------------------------------------------------
------------------------------------------------------------
------------------------------------------------------------
------------------------------------------------------------
------------------------------------------------------------
------

>GGQP01019073_Diaphanosoma_celebensis_Aqp12L
------------------------------------------------------------
------------------------ATG---GCCATCCCGCTGGTCGTGTCCATCGGCTTC
ATCGTCGTCACCTGCTTA---GTGGCCGAGCTTTTGCGTTCG---GCCGTC---AACTCG
GCGGTGACG---------ACGCCGCTCAGTCGGCTCCTCTTTCTGGAATTCATCGCCGCC
GCCGAGCTGTGCAGCATCTGCTTCGAGCTGATCATCGTGACC------------------
------GAGTCGTACGGAATCTGGATGTACGCCGCTTTCCTGTTCCTGTTGACGATCTGG
TGGGCGAATCACTGGCAGGACGCCGCCGGATGCCCGTACATCCACGTCGAGAACTACTTG
GAAGGGGTCAGCTCCTTCTCGCAGTCGTTCCTCTGCTGTCTGGCCGAAATCGCCGGCGGA
CTGGCCATTTACAAGTACGTCCAGCTGCTGTGGGACCTGAAGCTGTCGGACAGCTATGAA
GCG---------------------------------------------------------
------AAGAACGTGTGGGACTGCGACGCCGACCTGCAGGTGCCGATGCTGTACGGAGCG
CTGATCGAAGGTGCCGCCACCTTCCTG---------------TGCCGCATCGTGTCGCGG
ATGATCTCGGAAGTGGAG------------CCGCGTTTCGGCGGCGCGCTCGACGCCTTC
ATCGGCACCTCCCTCGTCGTGGCCGCCTTCAACCATTCTGGCGGCTACTTCAACCCGGTG
CTGGCCACTTCGCTCAAGTTGCACTGTCGC---GGA------------------------
CACACGGACATGGAACACTTTGTCGTGTACTGGATCGGAGCCACGGCCGGATCTGTGGCC
TCGTGGCTCCTCTAC---AAGCCGGTCATCAGCGGCAGCCTCTTGGGC------------
AAGGCGGACAAG---GAGAAATCGCAG---------------------------------
------------------------------------------------------------
------------------------------------------------------------
------------------------------------------------------------
------------------------------------------------------------
------------------------------------------------------------
------------------------------------------------------------
------------------------------------------------------------
------------------------------------------------------------
------------------------------------------------------------
------------------------------------------------------------
------------------------------------------------------------
------------------------------------------------------------
------------------------------------------------------------
------------------------------------------------------------
------------------------------------------------------------
------------------------------------------------------------
------------------------------------------------------------
------------------------------------------------------------
------------------------------------------------------------
------------------------------------------------------------
------------------------------------------------------------
------------------------------------------------------------
------
>NKDA01000007_Eulimnadia_texana_Aqp12L
------------------------------------------------------------
------------------------ATGGCGTTTTCTGCTTTATTAGTGTCTTGTATTTTT
ATGGCAGTTTCTTGCTTA---CTTGCGGAAATAGCCAGAAAA---TTCATT---AACTCT
CTCATCAAA---------ACCGAACTATCCAGACTACTTTTCACGAATTCATTGCAGCG
GCGGAACTGTGTGCTGTCTGTTTCGAACTGGTTATAGTGGCT------------------

------GAAGTCTACGGAATCTCTACGTATGCCATTTTGCTGTTTGTTTTAACCATCTGG
TGGGCAATTCGATGGCATGAAGCTGCTGGATGTCCTTACATCCACCTGGAAGCTTACCTG
GAAGGATCCTCCACATTTACTGAAAGCTTTCTGTGCTGTCTAGCAGAACTAGCAGGCGGA
CTAGCCATTTACAAGTATGTTCAGTTTCTATGGATCTGAAACTAGCAGACATTCACGAA
GAG---------------------------------------------------------
------AAAAGCGTCTGGGAATGCGAAGCAGAAATTAAGGTTTCTGCTTTGTATGGCGCC
TTTGTTGAGGGAATTGCAACTTGTTTA---------------TGTCGACTGGCTTCGAGA
TTGATTGCAGCTACTGAA------------CCGCGGTTCGCTACTGCCATTGATTCATTC
ATTGGGACTTCACTCGTTGTAGCAGCTTGCAATTATTCTGGTGGTTATTTCAACCCTGTT
CTGGCCACTTCGTTGAAATTCAACTGTCCC---GGC------------------------
AACACGGCGACTGAGCATTTCGTCGTTTACTGGGTCGGAGCTGCAGCGGGATCTATTCTG
TCGTGGTGGATTCATCAGAAAAAT---TACCTGGACAATATCATAGGTCTCACTAAACCC
---GCCGAAAAC---AAGAAAATTAAACAGAAT---------------------------
------------------------------------------------------------
------------------------------------------------------------
------------------------------------------------------------
------------------------------------------------------------
------------------------------------------------------------
------------------------------------------------------------
------------------------------------------------------------
------------------------------------------------------------
------------------------------------------------------------
------------------------------------------------------------
------------------------------------------------------------
------------------------------------------------------------
------------------------------------------------------------
------------------------------------------------------------
------------------------------------------------------------
------------------------------------------------------------
------------------------------------------------------------
------------------------------------------------------------
------------------------------------------------------------
------------------------------------------------------------
------------------------------------------------------------
------------------------------------------------------------
------
>JAIQCU010002348_Leptestheria_dahalacensis_Aqp12L
------------------------------------------------------------
------------------------ATGCCGGTTTCAGGTTTGATCGTATCATGCCTTTTC
ATGGCAGTTTCTTGCCTC---ATTGCAGAGTTATCCAGGAAA---CTGATC---GACAAA
TTCATTCAC---------GCGGAACTGTCGCGCCTCTTGTTCCATGAATTTGTTGCCTCG
GCGGAACTGTGCGCCGTTTGTTTCGAGCTCCTCATTGTTGCT------------------
------GATGTGTACGGAATCTCCACTTATGCCATCTTACTCTTCGTCTTGACTATTTGG
TGGTCAATGAAATGGCACGATGCTGCCGGATGTCCTTACATCCACCTGGAGTCCTACCTG
GAAGGGTCGTCCAGTTTCAGTCAAAGCTTCCTTTGCTGCCTTGCTGAAATGGCTGGTGGA
CTTGCTATATACAAGTATGTGCAGTACTTATGGATCTGAAATTCGACGACATCTACGAA
GAC---------------------------------------------------------
------CAGAGCATTTGGGAGTGCGAAGCAGACCTTCATGTACCCGCACTGCAAGGTGCG

TTGATTGAAGGCGTTGCGACATGCCTC---------------TGTCGCCTGGCGTCGCGC
GTTATCGCCACTACTGAA------------CCGCGTTTCGCTACTGCGATCGACTCTTTC
ATTGGAACGTCGCTCGTCGTTGCCGCATGCAATTATTCGGGCGGTTATTTCAACCCTGTC
TTGGCTACGTCGCTGAAGTTCAACTGTCCG---GGA------------------------
AACACCGCCATGGAGCATTTCGTCGTCTACTGGGTCGGCGCTGCCGCAGGGTCGGTAGCC
TCATGGTGGATCTGT---AAATATGGATACCTGGATAACATGCTTTCCTTGACGCGTCCT
---TCTGACGAG---AAAAAATCCAAA---------------------------------
------------------------------------------------------------
------------------------------------------------------------
------------------------------------------------------------
------------------------------------------------------------
------------------------------------------------------------
------------------------------------------------------------
------------------------------------------------------------
------------------------------------------------------------
------------------------------------------------------------
------------------------------------------------------------
------------------------------------------------------------
------------------------------------------------------------
------------------------------------------------------------
------------------------------------------------------------
------------------------------------------------------------
------------------------------------------------------------
------------------------------------------------------------
------------------------------------------------------------
------------------------------------------------------------
------------------------------------------------------------
------------------------------------------------------------
------------------------------------------------------------
------
>RJJA01001228_Lepidurus_arcticus_Aqp12L1
------------------------------------------------------------
------------------------ATG---TGGGCTCCTTTAATCGCCTCGTTATTGTTC
ATAGCCTTATCTTGTGGT---ATTGCGGAAGTATTGCGCTTT---GGAATC---CGCGCT
GGAATCAAG---------GATGAATTGTCGCGATTACTCTTTTTAGAGTTCGTAGCTTCT
GCCGAGCTCTGTGCTGCGTGTTTTGAGCTCGTTATTGTGGCC------------------
------GACAATTACGGAATTTGGATTTATGCTGCGGCGCTCTTCCTTTTATCGATATGG
TGGTGTAACAAGTGGCATGAGGAGTCTGGATGTCCGTATATACATATGGAGTCTTATCTA
GCTGCCCAATCGCCGTTTAATCAAGCCTTTTTGTGCTCCTTGGCTCAATTAGCAGGCGGA
TTAGCTATTTACAGGTATGTGCAGTACTTGTGGAGCTTGGAAATCACGGATCATCACGTT
GGG---------------------------------------------------------
------AAATCGTTATGGGACTGCGAAGCTGATTTACAAGTCACCGTATTTCAAGGTGCC
ATTATCGAAGGTCTAGCGACTTGCTTG---------------TGTCGTTTAGCTTCAAGA
GCATTAGCAGATTTGGAA------------CCTCAATTTGCTTCTGCTATCGACGCGTTT
GTGAGTACTGGACTCGTCGTAACGGCTTTTAACTACTCTGGAGGTTACTTTAACCCAGTA
TTGGCTACATCATTGAAGCTAAATTGTAGA---GGA------------------------
CACACGAATTTAGAACATTTCGTAGTATATTGGATAGGCGCCTTTGGCGGCTCGGCTTTA
TCGTTTTACGTGTAC---AAAATCCCGGCCATTAGAGCGTTGCTTACTGGGGGAACATTC

---GAGGAAGAG---AAGAAAACCAAA---------------------------------
------------------------------------------------------------
------------------------------------------------------------
------------------------------------------------------------
------------------------------------------------------------
------------------------------------------------------------
------------------------------------------------------------
------------------------------------------------------------
------------------------------------------------------------
------------------------------------------------------------
------------------------------------------------------------
------------------------------------------------------------
------------------------------------------------------------
------------------------------------------------------------
------------------------------------------------------------
------------------------------------------------------------
------------------------------------------------------------
------------------------------------------------------------
------------------------------------------------------------
------------------------------------------------------------
------------------------------------------------------------
------------------------------------------------------------
------------------------------------------------------------
------
>RJJA01001228_Lepidurus_arcticus_Aqp12L2
------------------------------------------------------------
------------------------ATG---TGGACTCCTTTAATCGCTTCGTTATTGTTC
ATAGCCTTAACTTGTGGT---ATTGCGGAAGTATTGCGCTTT---GGAATC---CGTGCT
GAAATCAAG---------GATGAATTGTCGCGATTACTCTTTTTAGAGTTCGTAGCTTCT
GCCGAGCTCTGTGCTGCGTGTTTTGAGCTCGTTATTGTGGCC------------------
------GACAATTACGGAATTTGGATTTATGCTGCGGCGCTCTTCCTTTTATCGATATGG
TGGTGTAACAAGTGGCATGAGGAGTCTGGATGTCCGTATATACATATGGAGTCTTATCTA
GCTGCCCAATCGCCGTTTAATCAAGCCTTTTTGTGCTCCTTGGCTCAATTAGCAGGCGGA
TTAGCTATTTACAGGTATGTGCAGTACTTGTGGAGCTTGGAAATCACGGATCATCACGTT
GGG---------------------------------------------------------
------AAATCGTTATGGGACTGCGAAGCTGATTTACAAGTCACCGTATTTCAAGGTGCC
ATTATCGAAGGTCTAGCGACTTGCTTG---------------TGTCGTTTAGCTTCAAGA
GCATTAGCAGATTTGGAA------------CCTCAATTTGCTTCTGCTATCGACGCGTTT
GTGAGTACTGGACTCGTCGTAACGGCTTTTAACTACTCTGGAGGTTACTTTAACCCAGTA
TTGGCTACATCATTGAAGCTAAATTGTAGA---GGA------------------------
CACACGAATTTAGAACATTTCGTAGTATATTGGATAGGCGCCTTTGGCGGCTCGGCTTTA
TCGTTTTACGTGTAC---AAAATCCCGGCCATTAGAGCGTTGCTTACTGGGGCAACATTC
---GAGGAAGAG---AAGAAAATCAAA---------------------------------
------------------------------------------------------------
------------------------------------------------------------
------------------------------------------------------------
------------------------------------------------------------
------------------------------------------------------------

------------------------------------------------------------
------------------------------------------------------------
------------------------------------------------------------
------------------------------------------------------------
------------------------------------------------------------
------------------------------------------------------------
------------------------------------------------------------
------------------------------------------------------------
------------------------------------------------------------
------------------------------------------------------------
------------------------------------------------------------
------------------------------------------------------------
------------------------------------------------------------
------------------------------------------------------------
------------------------------------------------------------
------------------------------------------------------------
------------------------------------------------------------
------
>JAGJRP010000075_Lepidurus_couesii_Aqp12L
------------------------------------------------------------
------------------------ATG---TGGGCTCCTTTAATCGCTTCGTTATTGTTC
ATAGCCTTAACTTGTGGT---ATTGCGGAAGTATTGCGCTTT---GGAATC---CGCGCT
GGAATCAAG---------GATGAATTGTCGCGATTACTCTTTTTAGAGTTCGTAGCTTCT
GCCGAGCTCTGTGCTGCGTGTTTTGAGCTCGTTATTGTGGCT------------------
------GACAATTACGGAATTTGGATTTATGCTGCGGCGCTCTTCCTTTTATCGATATGG
TGGTGTAACAAGTGGCATGAGGAGTCTGGATGTCCGTATATACATATGGAGTCTTATCTA
GCTGCCCAAACGCCGTTTAATCAAGCATTTTTGTGCTCCTTGGCTCAATTAGCAGGCGGA
TTAGCTATTTACAGGTATGTGCAGTACTTGTGGAGCTTAGAAATCACGGATCATCACGTT
GGG---------------------------------------------------------
------AAATCGTTATGGGACTGCGAAGCTGATTTACAAGTCACCGTATTTCAAGGTGCC
ATTATCGAAGGTCTAGCGACTTGCTTG---------------TGTCGTTTAGCTTCAAGA
GCATTAGCAGATTTGGAA------------CCTCAATTTGCTTCTGCTATCGACGCGTTT
GTGAGCACTGGACTCGTCGTAACGGCTTTTAACTACTCTGGAGGTTACTTTAACCCAGTA
TTGGCTACTTCATTGAAGCTAAATTGTAGA---GGA------------------------
CACACGAATTTAGAACATTTCGTAGTATATTGGATAGGCGCCTTTGGCGGCTCGGCTTTA
TCGTTTTACGTGTAC---AAAATCCCGGCCATTAGAGCGTTGCTTACGGGGGGAACATTC
---GAGGAAGAG---AAGAAAACCAAA---------------------------------
------------------------------------------------------------
------------------------------------------------------------
------------------------------------------------------------
------------------------------------------------------------
------------------------------------------------------------
------------------------------------------------------------
------------------------------------------------------------
------------------------------------------------------------
------------------------------------------------------------
------------------------------------------------------------
------------------------------------------------------------

------------------------------------------------------------
------------------------------------------------------------
------------------------------------------------------------
------------------------------------------------------------
------------------------------------------------------------
------------------------------------------------------------
------------------------------------------------------------
------------------------------------------------------------
------------------------------------------------------------
------------------------------------------------------------
------------------------------------------------------------
------
>JAKZAN010000007_Lepidurus_packardi_Aqp12L
------------------------------------------------------------
------------------------ATG---TGGGCTCCTTTAATCGCCTCGTTATTGTTC
ATAGCGTTAACTTGTGGT---ATTGCGGAAGTATTGCGTTTT---GGAATC---CGCGCT
GGCATCAAG---------GATGAATTGTCGCGATTACTTTTTTTAGAGTTCGTAGCTTCT
GCCGAGCTCTGTGCTGCGTGTTTTGAGCTCGTTATTGTGGCT------------------
------GACAATTATGGAATTTGGATTTATGCTGCGGCGCTCTTCCTTTTATCGATATGG
TGGTGCAATAAGTGGCAGGAGGAGTCTGGATGTCCGTATATACACATGGAATCTTATCTA
GCTGCCCAAACGCCGTTTAATCAAGCCTTTTTGTGCTCCTTGGCTCAATTAGCAGGCGGA
TTAGCTATTTACAGGTATGTGCAGTACTTGTGGAGTTTGGAAATCACGGACCATCACGTT
GGG---------------------------------------------------------
------AAATCGTTATGGACTGCGAAGCTGATTTACAAGTCACCGTATTTCAAGGTGCC
ATTATCGAAGGTCTAGCGACTTGCTTG---------------TGTCGTCTAGCTTCAAGA
GCATTAGCAGACTTGGAA------------CCTCAATTTGCTTCTGCTATCGACGCGTTT
GTGAGTACTGGACTCGTCGTAACGGCTTTTAACTACTCGGGAGGTTATTTTAACCCAGTC
TTGGCTACCTCATTGAAGCTAAATTGTAGA---GGA------------------------
CACACGAATTTAGAACATTTTGTGGTATATTGGGTTGGGGCCTTTGGCGGCTCGGCTCTA
TCATATTACGTGTAC---AAAATCCCCTCCATTAGAGCGTTGCTCACTGGGGAACCTTT
---GAGGAAGAG---AAGAAAACCAAA---------------------------------
------------------------------------------------------------
------------------------------------------------------------
------------------------------------------------------------
------------------------------------------------------------
------------------------------------------------------------
------------------------------------------------------------
------------------------------------------------------------
------------------------------------------------------------
------------------------------------------------------------
------------------------------------------------------------
------------------------------------------------------------
------------------------------------------------------------
------------------------------------------------------------
------------------------------------------------------------
------------------------------------------------------------
------------------------------------------------------------
------------------------------------------------------------

------------------------------------------------------------
------------------------------------------------------------
------------------------------------------------------------
------------------------------------------------------------
------------------------------------------------------------
------
>RJJB01001427_Lepidurus_apus_Aqp12L
------------------------------------------------------------
------------------------ATG---TGGGCTCCTTTAATTGCCTCGTTATTGTTC
ATAGCCTTAACTTGTGGT---ATTGCGGAAGTCTTGCGTTTT---GGAATC---CGGGCT
GGAATCAAG---------GATGAACTGTCGCGATTACTCTTTTTAGAATTTGTAGCTTCT
GCGGAGCTCTGTGCTGCGTGTTTTGAGCTCGTTATTGTGGCT------------------
------GAAAATTACGGAATTTGGATTTATGCTGCGGCGCTCTTCCTTTTATCCATATGG
TGGTGTAATAAGTGGCACGAGGAGTCTGGATGTCCGTATATACATATGGAGTCCTATTTA
GCTGCCCGAACGCCGTTTAATCAAGCGTTTTTGTGCTCCTTAGCTCAATTAGCAGGCGGA
TTAGCGATTTACAGGTACGTGCAGTACTTATGGAGTCTGGAAATCACGGATCATCACGTT
GGA---------------------------------------------------------
------AAATCGTTATGGGATTGCGAAGCTGATTTACAAGTCACCGTATTTCAAGGTGCC
ATTATTGAGGGTCTAGCGACTTGCTTG---------------TGTCGTTTAGCTTCAAGA
GCATTAGCAGACTTGGAA------------CCTCAATTTGCTTCTGCTATCGACGCGTTT
GTGAGCACTGGACTTGTCGTAACGGCTTTTAACTACTCTGGAGGCTACTTTAACCCAGTC
TTGGCTACTTCGTTGAAGCTAAATTGTAGG---GGA------------------------
CACACGAATTTAGAACATTTTATGGTATATTGGGTTGGCGCTTTTGGCGGCTCGGCTTTG
TCGTATTACGTGTAC---AAAATCCCAGCCATTAGAGCGTTGCTCACTGGAGGAACATTC
---GAGGAAGAG---AAGAAAACCAAA---------------------------------
------------------------------------------------------------
------------------------------------------------------------
------------------------------------------------------------
------------------------------------------------------------
------------------------------------------------------------
------------------------------------------------------------
------------------------------------------------------------
------------------------------------------------------------
------------------------------------------------------------
------------------------------------------------------------
------------------------------------------------------------
------------------------------------------------------------
------------------------------------------------------------
------------------------------------------------------------
------------------------------------------------------------
------------------------------------------------------------
------------------------------------------------------------
------------------------------------------------------------
------------------------------------------------------------
------------------------------------------------------------
------------------------------------------------------------
------------------------------------------------------------
------

```
>GEHY01001443_Triops_newberryi_Aqp12L
------------------------------------------------------------
------------------------ATG---TGGGCTCCTTTAATTGCTTCGTTAATTTTT
GTTGTGATAACGTGTACC---ATAGCCGAAGTATTGCGAATC---GGCATT---CGCGCG
GTCGTCAAG---------GATGAATTATCCCGGTTATTGTTCCTAGAGTTTGTAGCTTCC
GCAGAGTTGTGTGCTGCTTGCTTCGAACTCATTATCGTGGCA------------------
------GAGCACTACGGAGTATGGGTATACGCGGCGGCGTTGTTCCTTTTAACGATTTGG
TGGTGTAACAAATGGCATGATGAATCTGGATGTCCATACATCAATATGGAATCGTACCTC
GCAGGTGAAACACCCTTCAATCAAGCATTCTTATGCTCCCTGGCTCAGTTAGCTGGAGGT
TTAGCTATTTACAAGTATGTGCAATACTTATGGAGCCTAGAAATTACAGACAATCATGTG
GGA---------------------------------------------------------
------AAGTCCGTATGGGACTGCGAAACGGATTTGCAAGTTACCGTATTTGAGGGAGCA
ATTGTTGAAGGAATAGCCACTTGTTTG---------------TGCCGTTTGGCTTCGCGC
GCTTTGTCAGACTTGGAA------------CCGCAATTCGCTTCCGTAATTGACTCGTTC
GTGGGTACTGGACTGGTCGTGGCGGCTTGCAATTATTCCGGAGGATACTTTAACCCCGTT
TTGGCCACGTCGTTGAAGATGAACTGTCGG---GGT------------------------
CATACAAATTTGGAACATTTCGTGGTGTACTGGGTTGGCGCTTTTGCCGGATCGGCTTTC
TCTTATTATTTGTAC---AAGCTTCCTGCTGTTAAAGCGTTGCTAACTGGTGGAATGTTT
---GAAGAAGAG---AAGAAAACCAAA---------------------------------
------------------------------------------------------------
------------------------------------------------------------
------------------------------------------------------------
------------------------------------------------------------
------------------------------------------------------------
------------------------------------------------------------
------------------------------------------------------------
------------------------------------------------------------
------------------------------------------------------------
------------------------------------------------------------
------------------------------------------------------------
------------------------------------------------------------
------------------------------------------------------------
------------------------------------------------------------
------------------------------------------------------------
------------------------------------------------------------
------------------------------------------------------------
------------------------------------------------------------
------------------------------------------------------------
------------------------------------------------------------
------------------------------------------------------------
------------------------------------------------------------
------
>JAGQDJ010000158_Triops_longicaudatus_Aqp12L
------------------------------------------------------------
------------------------ATG---TGGGCTCCTCTAATTGCTTCGTTAATTTTT
GTTGTGATAACGTGTACC---ATCGCCGAAGTATTGCGAATC---GGCATC---CGCGCA
GTCGTCAAG---------GATGAATTATCCCGGTTATTGTTCCTAGAGTTTGTAGCTTCC
GCAGAATTGTGTGCTGCTTGCTTCGAACTCATTATCGTGGCA------------------
```

------GAGCACTATGGAGTATGGGTATACGCGGCGGCATTGTTCCTTTTAACTATTTGG
TGGTGTAACAAATGGCATGATGAATCTGGATGTCCATACATCAATATGGAATCGTACCTC
GCAGGTGAAACACCCTTCAATCAAGCGTTCTTATGTTCACTGGCTCAGTTAGCTGGAGGT
TTAGCTATTTACAAGTATGTGCAATACTTATGGAGCCTAGAAATTACAGACAATCATGTG
GGA---------------------------------------------------------
------AAGTCCGTATGGGACTGCGAAACGGATTTGCAAGTTACCGTATTTGAGGGAGCA
ATTGTTGAAGGAATAGCCACTTGTTTG---------------TGCCGTTTGGCTTCGCGC
GCTTTGTCAGACTTGGAA------------CCGCAATTCGCGTCCGTAATTGACTCGTTC
GTGGGTACTGGACTGGTCGTGGCGGCTTGCAATTATTCCGGAGGATATTTTAACCCAGTT
TTGGCCACGTCGTTGAAGATGAACTGTCGG---GGT------------------------
CATACGAATTTGGAACATTTCGTGGTGTACTGGGTTGGCGCTTTTGCCGGATCGGCTTTA
TCTTATTATTTGTAC---AAACTTCCTGCTGTTAAAGCATTGTTAACTGGTGGAATGTTT
---GAAGAAGAG---AAGAAAACCAAG---------------------------------
------------------------------------------------------------
------------------------------------------------------------
------------------------------------------------------------
------------------------------------------------------------
------------------------------------------------------------
------------------------------------------------------------
------------------------------------------------------------
------------------------------------------------------------
------------------------------------------------------------
------------------------------------------------------------
------------------------------------------------------------
------------------------------------------------------------
------------------------------------------------------------
------------------------------------------------------------
------------------------------------------------------------
------------------------------------------------------------
------------------------------------------------------------
------------------------------------------------------------
------------------------------------------------------------
------------------------------------------------------------
------------------------------------------------------------
------------------------------------------------------------
------
>GCAT01006816_Triops_cancriformis_Aqp12L
------------------------------------------------------------
------------------------ATGATGTGGGCTCCTTTAATTGCTTCTTTAATGTTT
GTGGCGTTGACGTGTACA---ATCGCCGAAGTTTTGCGTATC---GGCATT---CGCGCG
GCTGTCAAG---------GATGAGTTATCCCGGTTATTGTTCCTAGAATTCGTTGCTTCG
GCCGAATTGTGTGCTGCTTGCTTTGAGCTCGTTGTCGTGGCA------------------
------GAAAACTACGGAATATGGGTGTACGCGGCGGCATTGTTCGTGCTGACTGTTTGG
TGGTGTAACAAATGGCACGATGAGTCTGGATGTCCGTACATCAATATGGAATCTTACCTC
GCGGGTGAAACACCCTTTAACCAAGCGTTTTTGTGCTCATTGGCTCAGTTAGCGGGAGGT
TTGGCCATTTACAAATATGTGCAATACTTGTGGAGCTTGGAAATAACAGACAATCACGTG
GGG---------------------------------------------------------
------AAATCTGTGTGGGACTGCGAGACGGATTTGCAGGTTACTGTATTCGAGGGAGCG

ATTGTCGAAGCAATAGCGACATGTTTC---------------TGTCGTTTGGCTTCGCGC
GCTTTAGCAGACATGGAA------------CCTCAGTTTGCGTCTGTTGTTGACTCGTTT
GTGGGCACTGGCCTGGTCGTAGCAGCGTGCAATTACTCCGGAGGATATTTTAACCCAGTT
TTGGCTACCTCGTTGAAGATGAATTGTCGC---GGT------------------------
CATACCAATTTGGAGCATTTTGTGGTGTATTGGATTGGTGCTTTCGCTGGATCCGCCATG
TCTTATTATTTATAC---AAACTACCTGCTGTGAAAGCATTGTTAACTGGTGGTATGTTC
---GAAGAACAA---AAGAAAACCAAA---------------------------------
------------------------------------------------------------
------------------------------------------------------------
------------------------------------------------------------
------------------------------------------------------------
------------------------------------------------------------
------------------------------------------------------------
------------------------------------------------------------
------------------------------------------------------------
------------------------------------------------------------
------------------------------------------------------------
------------------------------------------------------------
------------------------------------------------------------
------------------------------------------------------------
------------------------------------------------------------
------------------------------------------------------------
------------------------------------------------------------
------------------------------------------------------------
------------------------------------------------------------
------------------------------------------------------------
------------------------------------------------------------
------------------------------------------------------------
------
>JAQQPU010004360_37717_74753_18163_Artemia_sp_Kazakhstan_Aqp12L
------------------------------------------------------------
------------------ATGGCCATGGACATTGAAGCATTGGCGATTTCTGTTAGCTAC
ATCGCTCTTACATCTGTT---GTTTGCTTCTATGTTCGCAAA---GCAGCT---GGAAGC
TATGTCACT---------GCAGAACCGTCTCGATTCATGTGTCTAGAATTCTTGGCCACT
GCAGAGATGTGTGCCACTGGATTTGAACTGCTCGTCGTTGCC------------------
------GAGGCTCACGGTCTGTGGATCTATGCCATTTGCCTGTTCTTTTATACAATCTAC
TTTTCGAAGTGTTGTGGTGAAGCTTGCGCCTGTCCTTATATCTACATCGAAAATTACCTA
AGCGGCAGCATTGATTCTACAAGGGCAGTTTTAGCTATTGCGGCCGAGATTGCTGGTGGT
TTAGCCGTCTTCCAGTATGTAAACGGACTCTGGAGTCTTGGCTTGGAGTTACATCACGTT
GGT---------------------------------------------------------
------CGTTCTGTCTGGATATGTGAAGCGGCCTTACAGGTTCCTGTTTTACAAGGTGCT
ATAGTTGAAGGCATTGGAACTTGTATA---------------TGTCGGCTTGCTTCTAAG
GCGCTTTCGGACGTGGAT------------CTATCTGTCAGCTTTGCCTTAGATTCACTT
ATTGGAACTTCTATCGTCTTGGCAGCCTGGAACTTCTCTGGAGGCTATTTCAATCCAGCC
TTAGCCACCTCTATTAAGTATGGATGCCTT---GGC------------------------
CACACTGCAGCTGAACACTTCGTGGTATATTGGATTGGTGCCACTGCAGGTGCTGTCGTT
GCACAATATTTATAC---TATCAACCAAATGTGAAGAGCCTTTTAACTGGTGGCATAGGT

```
---CCT---CAG---AAA---GCGGAC---------------------------------
------------------------------------------------------------
------------------------------------------------------------
------------------------------------------------------------
------------------------------------------------------------
------------------------------------------------------------
------------------------------------------------------------
------------------------------------------------------------
------------------------------------------------------------
------------------------------------------------------------
------------------------------------------------------------
------------------------------------------------------------
------------------------------------------------------------
------------------------------------------------------------
------------------------------------------------------------
------------------------------------------------------------
------------------------------------------------------------
------------------------------------------------------------
------------------------------------------------------------
------------------------------------------------------------
------------------------------------------------------------
------------------------------------------------------------
------------------------------------------------------------
------
>CM051260_Artemia_sinica_Aqp12L
------------------------------------------------------------
------------------ATGGCCATGGACATTGAAGCATTGGCGATTTCTGTTAGCTAC
ATCGTTCTTACATCTGTT---GTTTGCTTCTATGTTCGCAAA---GCAGCT---GGAAGC
TATGTCACT---------GCAGAACCGTCTCGATTCATGTGTCTAGAATTCTTGGCCACT
GCAGAGATGTGTGCCACTGGGTTTGAACTGCTCGTCGTTGCC------------------
------GAGGCTCAGGGTCTGTGGATCTATGCCATTTGCCTGTTCTTTTTTACAATCTAC
TTTTCGAAGTGTTGTGGTGAAGCTTGTGCCTGTCCTTATATCTACATCGAAAATTACCTA
AGCGGCAGCATTGATTCTACAAGGGCAGTTTTAGCTATTGCGGCCGAGATTGCTGGTGGT
TTAGCCGTCTTCCAGTATGTAAACGGACTCTGGAGTCTTGGCTTGGAGTTACATCACGTT
GGT---------------------------------------------------------
------CGTTCTGTCTGGATATGTGAAGCGGCCTTACAGGTCCCTGTTTTACAAGGTGCT
ATAGTTGAAGGCATTGAACTTGTACA----------------TGTCGGCTTGCTTCTAAG
GCGCTTTCGGACGTGGAT------------CTATCTGTCAGCTTTGCCTTAGATTCACTT
ATTGGAACTTCTATCGTCTTGGCA------------------------------------
------------------------------------------------------------
------------------------------------------------------------
------------------------------------------------------------
------------------------------------------------------------
------------------------------------------------------------
------------------------------------------------------------
------------------------------------------------------------
------------------------------------------------------------
------------------------------------------------------------
```

------------------------------------------------------------
------------------------------------------------------------
------------------------------------------------------------
------------------------------------------------------------
------------------------------------------------------------
------------------------------------------------------------
------------------------------------------------------------
------------------------------------------------------------
------------------------------------------------------------
------------------------------------------------------------
------------------------------------------------------------
------------------------------------------------------------
------------------------------------------------------------
------------------------------------------------------------
------------------------------------------------------------
------------------------------------------------------------
------------------------------------------------------------
------
>JAVRJZ010000017_Artemia_franciscana_Jo_Aqp12L
------------------------------------------------------------
------------------ATGGCCATGGACATTGAAGCATTGGTGATTTCTGTTAGCTAC
ATCGTTCTTACATCGGCT---TTTTGCTTCTATGTTCGCAAA---GCGGTT---GAAAGC
TATGTTACT---------GCAGAACCGTCCCGATTCATATGTCTAGAATTCTTGGCCACT
GCAGAGATGTGTGCCACTGGATTTGAACTACTCGTAGTTGCC------------------
------GAGGCTCACGGTCTCTGGATCTATGCTGTTTGCCTGTTCTTGTTTACAATCTAC
TTTTCGAAGTGTTGTGGTGAAGCCTGTGCCTGTCCTTATATCTACATCGAAAATTACTTA
AGTGGCAGCATTGATTCTACAAGGGCAGTTTTAGCTATTGCTGCCGAGATTGCTGGTGGC
TTAGCCGTATTTCAGTATGTTAACGCACTCTGGAGTCTTGGTTTGGAGTTACATCACGTT
GGT---------------------------------------------------------
------CGTTCTGTCTGGATATGTGAAGCGGCTTTGCAGGTTCCTGTTTTACTTGGTGCT
TTAGCCGAGGGCATTGGAACTTGTCTG---------------TGTCGGCTTTCTTCTAAG
GCGCTTTCGGACACGGAT------------GTATCTGTCAGCTTTGCATTAGATTCATTT
ATTGGAACTTCTATCGTCTTGGCAGCCTGGAACTTCTCTGGAGGTTACTTCAATCCAGCC
TTGGCCACCTCTATGAAGTATGGATGCCTT---GGC------------------------
CACACTGCAGCCGAACACTTCATGGTATATTGGATTGGTGCCACTGCAGGCGCTGTTGCT
GCACAATATTTATAC---AATCAACCAATTGTGAAGAGTCTTTTAACTGGTGGTATAGGT
---CCT---CAG---AAA---GCGGAC---------------------------------
------------------------------------------------------------
------------------------------------------------------------
------------------------------------------------------------
------------------------------------------------------------
------------------------------------------------------------
------------------------------------------------------------
------------------------------------------------------------
------------------------------------------------------------
------------------------------------------------------------
------------------------------------------------------------
------------------------------------------------------------

------------------------------------------------------------
------------------------------------------------------------
------------------------------------------------------------
------------------------------------------------------------
------------------------------------------------------------
------------------------------------------------------------
------------------------------------------------------------
------------------------------------------------------------
------------------------------------------------------------
------------------------------------------------------------
------------------------------------------------------------
------
>PQ469255_Artemia_franciscana_Aqp12L
------------------------------------------------------------
------------------ATGGCCATGGACATTGAAGCATTGGTGATTTCTGTTAGCTAC
ATCGTTCTTACATCGGCT---TTTTGCTTCTATGTTCGCAAA---GCGGTT---GAAAGC
TATGTTACT---------GCAGAACCGTCCCGATTCATATGTCTAGAATTTTTGGCCACT
GCAGAGATGTGTGCCACTGGATTTGAACTACTCGTCATTGCC------------------
------GAGGCTCACGGTCTCTGGATCTATGCTGTTTGCCTGTTCTTGTTTACAATCTAC
TTTTCGAAGTGTTGTGGTGAAGCCTGTGCCTGTCCTTATATCTACATCGAAAATTACTTA
AGTGGCAGCATTGATTCTACAAGGGCAGTTTTAGCTATTGCTGCCGAGATTGCTGGTGGC
TTAGCCGTATTTCAGTATGTTAACGCACTCTGGAGTCTTGGTTTGGAGTTACATCACGTT
GGT---------------------------------------------------------
------CGTTCTGTCTGGATATGTGAAGCGGCTTTGCAGGTTCCTGTTTTACTTGGTGCT
TTAGCCGAGGGCATTGGAACTTGTCTG---------------TGTCGGCTTTCTTCTAAG
GCGCTTTCGGACACGGAT------------GTATCTGTCAGCTTTGCATTAGATTCATTT
ATTGGAACTTCTATCGTCTTGGCAGCCTGGAACTTCTCTGGAGGTTACTTCAATCCAGCC
TTGGCCACCTCTATGAAGTATGGATGCCTT---GGC------------------------
CACACTGCAGCCGAACACTTCATCGTATATTGGATTGGTGCCACTGCAGGCGCTGTTGCT
GCACAATATTTATAC---AATCAACCAATTGTGAAGAGTCTTTTAACTGGTGGTATAGGT
---CCT---CAG---AAA---GCGGAC---------------------------------
------------------------------------------------------------
------------------------------------------------------------
------------------------------------------------------------
------------------------------------------------------------
------------------------------------------------------------
------------------------------------------------------------
------------------------------------------------------------
------------------------------------------------------------
------------------------------------------------------------
------------------------------------------------------------
------------------------------------------------------------
------------------------------------------------------------
------------------------------------------------------------
------------------------------------------------------------
------------------------------------------------------------
------------------------------------------------------------
------------------------------------------------------------

------------------------------------------------------------
------------------------------------------------------------
------------------------------------------------------------
------------------------------------------------------------
------------------------------------------------------------
------
>JAYKOS010000019_Artemia_franciscana_Bett_Aqp12L
------------------------------------------------------------
------------------ATGGCCATGGACATTGAAGCATTGGTGATTTCTGTTAGCTAC
ATCGTTCTTACATCGGCT---TTTTGCTTCTATGTTCGCAAA---GCGGTT---GAAAGC
TATGTTACT---------GCAGAACCGTCCCGATTCATATGTCTAGAATTCTTGGCCACT
GCAGAGATGTGTGCCACTGGATTTGAACTACTCGTAGTTGCC------------------
------GAGGCTCACGGTCTCTGGATCTATGCTGTTTGCCTGTTCTTGTTTACAATCTAC
TTTTCGAAGTGTTGTGGTGAAGCCTGTGCCTGTCCTTATATCTACATCGAAAATTACTTA
AGTGGCAGCATTGATTCTACAAGGGCAGTTTTAGCTATTGCTGCCGAGATTGCTGGTGGC
TTAGCCGTATTTCAGTATGTTAACGCACTCTGGAGTCTTGGTTTGGAGTTACATCACGTT
GGT---------------------------------------------------------
------CGTTCTGTCTGGATATGTGAAGCGGCTTTGCAGGTTCCTGTTTTACTTGGTGCT
TTAGCCGAGGCATTGGAACTTGTCTG----------------TGTCGGCTTTCTTCTAAG
GCGCTTTCGGACACGGAT------------GTATCTGTCAGCTTTGCATTAGATTCATTT
ATTGGAACTTCTATCGTCTTGGCAGCCTGGAACTTCTCTGGAGGTTACTTCAATCCAGCC
TTGGCCACCTCTATGAAGTATGGATGCCTT---GGC------------------------
CACACTGCAGCCGAACACTTCATCGTATATTGGATTGGTGCCACTGCAGGCGCTGTTGCT
GCACAATATTTATAC---AATCAACCAATTGTGAAGAGTCTTTTAACTGGTGGTATAGGT
---CCT---CAG---AAA---GCGGAC------------------------------------
------------------------------------------------------------
------------------------------------------------------------
------------------------------------------------------------
------------------------------------------------------------
------------------------------------------------------------
------------------------------------------------------------
------------------------------------------------------------
------------------------------------------------------------
------------------------------------------------------------
------------------------------------------------------------
------------------------------------------------------------
------------------------------------------------------------
------------------------------------------------------------
------------------------------------------------------------
------------------------------------------------------------
------------------------------------------------------------
------------------------------------------------------------
------------------------------------------------------------
------------------------------------------------------------
------------------------------------------------------------
------------------------------------------------------------
------------------------------------------------------------
------

```
>JAKZAP010000010_Branchinecta_lynchi_Aqp12L
------------------------------------------------------------
------------------------ATGGACTTCGAAGCATTGGGCGTGTCGACTGTGTAC
GTCTTGCTCACCGTCTAT---GCAGGACACTTCATCCGGAAG---ACAGTA---GAGGCA
AAAGTGGGC---------ATCGAACTGACTAGGCTACTGTGCTTGGAGTTCGTGGCTTCT
GCGGAAATGTGCGCCGTTGGCTTTGAGCTTCTCGTTGTTGCT------------------
------GAAACTCATGGATTATCTGTGTACGCAGTCTTCCTCTTTTTGTTCACGATTTAC
GCATCTAAGTCATGGGGTGAAGCCTGCGCTTGCCCATACGTTTATGTTGAAAATTTTATT
AGCGGAGATGTATATCTTTCGAAGGCGATCTTGGCTATTGCTGCTCAGCTTGCTGGAGGG
TTCGCCGTCTATCAGTATGTTATGGGCTATTGGGGTCTCGGTTACGAACTCCATCACGTC
GGC---------------------------------------------------------
------AGATCTGTTTGGAACTGCGATGCTGACCTCCAGGTTTCCGTTCTGCAAGGGGCT
TTGATTGAGGGGATAGCTACTTGCTTG---------------TGCCGACTGGCCTCAAGA
GCCTTGTCAGACGTTGAC------------TTCTCGCTTGGCACTGCCTTCGACTCTTTC
ATTGGCACATCTCTTGTCCTGGCCGCGTGGAACTACTCCGGTGGCTATTTCAACCCAGTT
CTTGCCACCTCCTTGAAATACGGGTGTCGC---GGC------------------------
CACACGGGGATTGAACATTTCATAGTCTACTGGATTGGCTCTAGCGCTGGAGCCGTTTTG
GCTCAGAAACTGTAC---AACGTCCCAACCGTCAAAAGCATCTTGACTGGCGGGCAAGGA
---CCAGAAGAA---AAGAAGGCCAAG---------------------------------
------------------------------------------------------------
------------------------------------------------------------
------------------------------------------------------------
------------------------------------------------------------
------------------------------------------------------------
------------------------------------------------------------
------------------------------------------------------------
------------------------------------------------------------
------------------------------------------------------------
------------------------------------------------------------
------------------------------------------------------------
------------------------------------------------------------
------------------------------------------------------------
------------------------------------------------------------
------------------------------------------------------------
------------------------------------------------------------
------------------------------------------------------------
------------------------------------------------------------
------------------------------------------------------------
------------------------------------------------------------
------------------------------------------------------------
------------------------------------------------------------
------
>JAKZAO010000009_Branchinecta_lindahli_Aqp12L
---------------------------------ATGGCTCTCCTTTATGATGAATATTCC
TGTAGGTCCTTCAGCTGGCTTGTAATGGACTTCGAAGCATTGGCCGTGTCGACTGTCTAC
GTCTTGCTCACCGTCTAT---GCAGGACACTTCATCCGAAAG---ACCATA---GAGACA
AAAGTGAAC---------GTCGAGCCGACTAGACTACTGTGCTTGGAGTTCGTAGCTTCT
GCGGAAATGTGCGCCATTGGCTTTGAGCTTCTTGTTGTTGCT------------------
```

------GAAGCTCATGGATTATCTGTCTACGCAGTTTTCCTCTTTTTGTTCACGATTTAT
GCATCTAAGTCATGGGGTGAAGCCTGTGCTTGCCCATATGTTTACGTTGAGAATCTTATT
AGCGGAGATGTATATCTTTCAAAGGCGATCTTAGCAATCGCCGCTCAGCTTGCTGGAGGG
TTTGCCGTCTATCAGTATGTCATGGGCTATTGGGGTCTCGGTTACGAACTCCATCACGTC
GGA---------------------------------------------------------
------AGATCCGTTTGGAACTGCGATGCCGACCTCCAGGTTTCCGTTCTGCAAGGGGCT
TTGATTGAGGGGATAGCTACTTGCTTA---------------TGCCGACTGGCTTCCAGA
GCCTTGTCGGACGTTGAC------------TTCTCACTTGCCACTGCCTTCGACTCTTTC
ATTGGCACATCTCTTGTCATCGCTGCGTGGAACTACTCTGGTGGCTATTTCAACCCAGTT
CTTGCCACCTCCTTGAAATACGGGTGCCGC---GGC------------------------
CATACGGGGATTGAACATTTCATAGTCTACTGGATTGGCTCCAGCGCTGGAGCCGTCATG
GCTCAGAAACTGTAC---AACCTCCCTACCGTCAAAAGCATCTTGACTGGCGGGCAAGGA
---CCAGAAGAA---AAGAAGGCCAAG---------------------------------
------------------------------------------------------------
------------------------------------------------------------
------------------------------------------------------------
------------------------------------------------------------
------------------------------------------------------------
------------------------------------------------------------
------------------------------------------------------------
------------------------------------------------------------
------------------------------------------------------------
------------------------------------------------------------
------------------------------------------------------------
------------------------------------------------------------
------------------------------------------------------------
------------------------------------------------------------
------------------------------------------------------------
------------------------------------------------------------
------------------------------------------------------------
------------------------------------------------------------
------------------------------------------------------------
------------------------------------------------------------
------------------------------------------------------------
------------------------------------------------------------
------
>JAUPMV010000011_Branchinecta_sandiegonensis_Aqp12L
------------------------------------------------------------
------------------------ATGGACTTCGAAGCATTGGCAGTATCGACTGTGTAC
GTCTTGCTCACCGTCTAT---GCAGGACACTTTATCCGGAAG---ACCATA---GAGGCA
AAAGTTGAC---------ATCGAACTGACGAGATTACTGTTCTTGGAGTTCGTAGCTTCT
GCGGAAATGTGCGCCGTTGGTTTTGAGCTTCTCGTTGTTGCT------------------
------GAAGCTCATGGATTATCTGTGTACGCAGTCTTCCTCTTTTTGTTCACGATTTAC
GCATCTAAGTCGTGGGGTGAAGCCTGCGCTTGCCCATACGTTTATGTTGAAAATTTTCTT
AGCAGAGATGTATATCTTTCGAAGGCTATCTTGGCAATCGCTGCTCAGCTTGCCGGAGGG
TTCGCCGTCTATCAGTACGTCATGGGCTATTGGGGTCTCGGTTACGAACTCCATCACGTC
GGG---------------------------------------------------------
------AGATCTGTTTGGAACTGCGATGCTGACCTCCAGGTTTCCGTTCTGCAAGGGGCT

TTGATTGAGGGGATAGCTACTTGCTTG---------------TGCAGACTGGCCTCAAGA
GCTTTGTCGGACGTTGAC------------TTCTCACTTGGCACTGCCTTCGACTCTCTC
ATTGGCACATCTCTTGTCCTCGCTGCGTGGAACTACTCCGGCGGCTATTTCAACCCAGTT
CTCGCCACCTCCTTGAAATACGGGTGTCGC---GGC------------------------
CACACGGGGATTGAACATTTCATAGTCTACTGGATTGGCTCCAGTGCTGGAGCCGTTTTG
GCTCAGAAATTGTAC---AACGTCCCAACCGTCAAAAGCATCTTGACTGGCGGCAAGGA
---CCAGAAGAA---AAGAAGACCAAG---------------------------------
------------------------------------------------------------
------------------------------------------------------------
------------------------------------------------------------
------------------------------------------------------------
------------------------------------------------------------
------------------------------------------------------------
------------------------------------------------------------
------------------------------------------------------------
------------------------------------------------------------
------------------------------------------------------------
------------------------------------------------------------
------------------------------------------------------------
------------------------------------------------------------
------------------------------------------------------------
------------------------------------------------------------
------------------------------------------------------------
------------------------------------------------------------
------------------------------------------------------------
------------------------------------------------------------
------------------------------------------------------------
------------------------------------------------------------
------------------------------------------------------------
------
>GCBP01006171_Eubranchipus_grubii_Aqp12L
------------------------------------------------------------
------------------------ATGGACTACGAAGCTCTTCTCGTATCAACGGTATAC
ATAGTCCTTACTCTCTAC---TTGGGACGTTTCATCAGAAGA---TGGATA---GAAGCT
GCTATAACT---------ACAGACCTAACTCGTCTGATGTGCCTAGAGTTCGTTGCATCA
GCTGAGATGTGTGCCACTGGCTTCGAGATGCTTGTCGTTGCT------------------
------GAGACGCACGGACTTGGCATGTATGCGGTTTTTCTGTTTGTAATAACCATCTAC
CTGTCAAAATCATGGAATGAAGCCTGCGCATGTCCATATATCTACATTGAAAACTATATA
AGTGGTGACGTCTACTTTTCGAAGGCCATTCTGGCAATAGCGGCCCAGTTGGCAGGAGGC
TTTGCCGTTTATCAGTACGTCATGGGATACTGGGGACTCGGTTATGAGCTACATCATGTC
GGC---------------------------------------------------------
------CGGTCGGTTTGGAACTGCGACGCTGATCTTCAGGTTACCGTTTTGCAAGGCGCT
TTGATTGAGGGTATAGCTACTTGCTTA---------------TGTCGTCTTGGTTCGAGA
GCTTTGTCCGACATTGAT------------TTCTCTTTGGGAGCTGCTCTGGATTCTCTA
GTTGGGACTTCCCTTGTTTGTGCTGCTTGGAACTATTCCGGTGGATACTTCAACCCCGTC
CTTGCGACGTCTTTGAAATATGGGTGTCGT---GGT------------------------
CACACCGGACTAGAACACTTTGCAGTCTACTGGATTGGTGCCAGCGTTGGAGCAGTGGTT
GCCCAAAAATTGTAT---GCTTTACCAACAACGAAGAGCATTTTAACTGGTGGCATAGGT

---CCGGAAGAA---AAGAAGAAGTTA--------------------------------
------------------------------------------------------------
------------------------------------------------------------
------------------------------------------------------------
------------------------------------------------------------
------------------------------------------------------------
------------------------------------------------------------
------------------------------------------------------------
------------------------------------------------------------
------------------------------------------------------------
------------------------------------------------------------
------------------------------------------------------------
------------------------------------------------------------
------------------------------------------------------------
------------------------------------------------------------
------------------------------------------------------------
------------------------------------------------------------
------------------------------------------------------------
------------------------------------------------------------
------------------------------------------------------------
------------------------------------------------------------
------------------------------------------------------------
------------------------------------------------------------
------
>CM023192_Daphnia_carinata_GlpB3P
------------------------------------------------------------
------------------------------------------------------------
------------------------------------------------------CCTACG
AATTGCAGT------TGTCCTTGCCTAACTCGCGCCGTTTTGGCCGAGTTCATCGGTACG
TTCATCCTTGTTGTTATTGGC------AGTGTAGCTCAATCGCAACCAACCAATGGCCAA
AAAGGCGATTACTCTACCATCAACTGGGGATGGGCTCTAGGATGTTCGCTGGGAATCCTC
GTTTCG------------GGTGGCCATTTGAATACGGCATTGACGCTGGCCCTGGCCGTG
GCCAAAGATTTTCCCTGGAAGAGGCTGCTTGCGTATTGGTTAGGCCAGTACCTGGGATCT
TTGGCCGCTTCCGGG---------------------------------------AAAATG
AAC---------GGTGAATTT---ATTAACTCC------AATTTATCTGAACCGGAGTTG
GACTCGGCAGCCATTTTCGCCAATTATACGACGCCCTATTCTCACGTCGTTACTGGTTTA
GTAGAAGAGATTCTATCAACAATGATCTTCATACTGATTATTTGTGTGGTGACCAATTCC
AAATATTCAAAAGTGCCG------------AGTTTTTTGCAGCCGCTCTACACCGGTTTC
ACGCTGTTAGCCCTTGGCGCGGCGTACGGATCCAACGGAGGTTACGCTCTA------GCG
CGTGATTTGGCTCCCCTGTTAGTCACGCATTTCGGAGGA---------------------
------------------------------------------------------------
------------------------------------------------------------
------------------------------------------------------------
------------------------------------------------------------
------------------------------------------------------------
------------------------------------------------------------
------------------------------------------------------------
------------------------------------------------------------

------------------------------------------------------------
------------------------------------------------------------
------------------------------------------------------------
------------------------------------------------------------
------------------------------------------------------------
------------------------------------------------------------
------------------------------------------------------------
------------------------------------------------------------
------------------------------------------------------------
------------------------------------------------------------
------------------------------------------------------------
------------------------------------------------------------
------------------------------------------------------------
------------------------------------------------------------
------------------------------------------------------------
------------------------------------------------------------
------------------------------------------------------------
------
>WJBH02000005_Daphnia_sinensis_GlpB3p
------------------------------------------------------------
------------------------------------------------------------
------------------------------------------------------CCTACG
AATTGCAGT------TGTCCTTGCCTAACTCGCGCCGTTTTGGCCGAGTTCATCGGTACG
TTCATCCTTGTTGTTATTGGC------AGTGTAGCTCAATCGCAACCAACCAATGGCCAA
AAAGGCGATTACTCTACCATCAACTGGGGATGGGCTCTAGGATGTTCGCTGGGAATCCTC
GTTTCGTTCAAAGCCTCAGGTGGCCATTTGAATACGGCATTGACGCTGGCCCTGGCCGTG
GCCAAAGATTTTCCCTGGAAGAGGCTGCTTGCGTATTGGTTAGGCCAGTACCTGGGATCT
TTGGCCGCTTCCGGG------------------------------------AAAAAAATG
AAC---------GGTGAATTT---ATTAACTCC------AATTTATCTGAACCGGAGTTG
GACTCGGCAGCCATTTTCGCCAATTATACGACGCCCTATTCTCACGTCGTTACTGGTTTA
GTAGAAGAGATTCTATCAACAATGATCTTCATACTGATTATTTGTGTGGTGACCAATTCC
AAATATTCAAAAGTGCCG------------AGTTTTTTGCAGCCGCTCTACACCGGTTTC
ACGCTGTTAGCCCTTGGCGCGGCGTACGGATCCAACGGAGGTTACGCTCTA------GCG
CGTGATTTGGCTCCCCTGTTAGTCACGCATTTCGGAGGA---GGTCCTCGCGTATTT---
------------------------------------------------------------
------------------------------------------------------------
------------------------------------------------------------
------------------------------------------------------------
------------------------------------------------------------
------------------------------------------------------------
------------------------------------------------------------
------------------------------------------------------------
------------------------------------------------------------
------------------------------------------------------------
------------------------------------------------------------
------------------------------------------------------------
------------------------------------------------------------
------------------------------------------------------------

------------------------------------------------------------
------------------------------------------------------------
------------------------------------------------------------
------------------------------------------------------------
------------------------------------------------------------
------------------------------------------------------------
------------------------------------------------------------
------------------------------------------------------------
------------------------------------------------------------
------------------------------------------------------------
------------------------------------------------------------
------
>CM023192_Daphnia_carinata_GlpB2
------------------------------------------------------------
------------------------------------------------------------
---------ATGGCCAAC---GACCAGCAAAGAGGCGACAAA---CTCACT---CCG---
AATTGCAAC------TTCCCCAGTCTGGCTCGAGCCGTGTTGGCGGAGTTCATCGGCACC
TTCATCCTTGTCACTATCGGAAGTGGCAGCGTAGCTCAATCGCAGCTAACCAATGGCAAA
AAAGGCGATTACTTCACCATCAACTGGGGATGGGCTCTAGGATGTTCGCTGGGCATCCTC
GTTGCGTCCAAAGCCTCAGGCGGCCACTTGAATCCGGCCGTGACGATGGCCCTGGCCGTG
GCCAAAGATTTCTCCTGGAAGAGGCTGCCCGCCTATTGGTTAGCGCAGTACTCTGGAGCT
CTGGTCGCTTCCGGCACCGTCCTCGGCATCTACTACGAAGCCATAGCTGACAAGAAAGTG
GAC---------GGTGAGTTTCGAATCAATGCC------AATTCGTCGGATCCGGAGCCC
GGCTCGGCGGCCATTTTCGCCAACTATCCGACGCCTTATTCGTCTGTTGTTACCGGCTTA
ATGGAAGAGATTTTGTCGACCATGATCTTCATGCTGATCATTTGCGTCGTGACCAATTCG
AAGTATTCGAAAGTGCCG------------AATTTCTTGCAGCCGCTCTACATTGGCTTC
ACGTTGCTGGCCATCGGCGTGGCCTACGGATCCAACGGAGGCTACGCTCTGAACCCGGCT
CGGGATTTGGCCCCCCGATTAGTCTCTTACTTCGGAGGATGGGGACCGCGCGTATTCAGC
TTTCGCGGCTACAACTGGTTCTGGATTTTCCTGGTCGGGCCACACGTGGGAGCTCTGCTC
GGCGTCGGCATTTTC---CACTCCGTCTTCTTGCGATTGGAGCAGTCAGCGGTC------
---GGAACAGAC---GGAACTACGGATTCGCAGCTGCCGATGGTCGACTACGAATATGCA
ATGACCAACGTGCCCGATAACAAATCGAGCGTC------ATCAAAGTAACG---AGAAGG
---------------------------------CAG---AGCCACGAAGAGTATATTGGA
TGCAGCATCGTCACGCCGATG---------------------------------------
------------------------------------------------------------
------------------------------------------------------------
------------------------------------------------------------
------------------------------------------------------------
------------------------------------------------------------
------------------------------------------------------------
------------------------------------------------------------
------------------------------------------------------------
------------------------------------------------------------
------------------------------------------------------------
------------------------------------------------------------
------------------------------------------------------------
------------------------------------------------------------
------------------------------------------------------------

------------------------------------------------------------
------------------------------------------------------------
------------------------------------------------------------
------------------------------------------------------------
------------------------------------------------------------
------
>KAI9557769_Daphnia_sinensis_GlpB2
------------------------------------------------------------
------------------------------------------------------------
---------ATGGCCAAC---GACCAGCAAAGAGGCGACAAA---CTCACT---CCG---
AATTGCAAC------TTCCCCAGTCTGGCTCGAGCCGTGTTGGCGGAGTTCATCGGCACC
TTCATCCTTGTCACTATCGGAAGTGGCAGCGTAGCTCAATCGCAGCTAACCAATGGCAAA
AAAGGCGATTACTTCACCATCAACTGGGGATGGGCTCTAGGATGTTCGCTGGGCATCCTC
GTTGCGTCCAAAGCCTCAGGCGGCCACTTGAATCCGGCCGTGACGATGGCCCTGGCCGTG
GCCAAAGATTTCTCCTGGAAGAGGCTGCCCGCCTATTGGTTAGCGCAGTACTCTGGAGCT
CTGGTCGCTTCCGGCACCGTCCTCGGCATCTACTACGAAGCCATAGCTGACAAGAAAGTG
GAC---------GGTGAGTTTCGAATCAATGCC------AATTCGTCGGATCCGGAGCCC
GGCTCGGCGGCCATTTTCGCCAACTATCCGACGCCTTATTCGTCTGTTGTTACCGGCTTA
ATGGAAGAGATTTTGTCGACCATGATCTTCATGCTGATCATTTGCGTCGTGACCAATTCG
AAGTATTCGAAAGTGCCG------------AATTTCTTGCAGCCGCTCTACATTGGCTTC
ACGTTGCTGGCCATCGGCGTGGCCTACGGATCCAACGGAGGCTACGCTCTGAACCCGGCT
CGGGATTTGGCCCCCCGATTAGTCTCTTACTTCGGAGGATGGGGACCGCGCGTATTCAGC
TTTCGCGGCTACAACTGGTTCTGGATTTTCCTGGTCGGGCCACACGTGGGAGCTCTGCTC
GGCGTCGGCATTTTC---CACTCCGTCTTCTTGCGATTGGAGCAGTCAGCGGTC------
---GGAACAGAC---GGAACTACGGATTCGCAGCTGCCGATGGTCGACTACGAATATGCA
ATGACCAACGTGCCCGATAACAAATCGAGCGTC------ATCAAAGTAACG---AGAAGG
---------------------------------CAG---AGCCACGAAGAGTATATTGGA
TGCAGCATCGTCACGCCGATG---------------------------------------
------------------------------------------------------------
------------------------------------------------------------
------------------------------------------------------------
------------------------------------------------------------
------------------------------------------------------------
------------------------------------------------------------
------------------------------------------------------------
------------------------------------------------------------
------------------------------------------------------------
------------------------------------------------------------
------------------------------------------------------------
------------------------------------------------------------
------------------------------------------------------------
------------------------------------------------------------
------------------------------------------------------------
------------------------------------------------------------
------------------------------------------------------------
------------------------------------------------------------
------------------------------------------------------------
------

>NW_022655294_Daphnia_magna_GlpB2
------------------------------------------------------------
------------------------------------------------------------
---------ATGGCGAAC------AACCAACGAGACGACAAA---TTG------CCTTCT
AATTGCAAT------ATGCCCAGTCTGGCTAAGGCGGTTTTGGCGGAGTTTATTGGCACC
TTTATTCTTGTCGCTATCGGCAGTGGCAGCGTAGCTCAATCGCAATTGACGAACGGCAAA
AAAGGCGACTACTTCACCATCAATTGGGGGTGGGCTTTGGGATGCTCGTTGGGAATCCTC
GTTTCGGGCAAAGCATCAGGTGGTCACTTGAATCCGGCCGTGACGATGGCCCTGGCCGTA
GCCAAGGATTTCTCCTGGAAGAGGCTACCTGCTTATTGGTTAGCGCAGTACTCTGGAGCT
CTGGTCGCCTCTGGCGCTGTTCTAGGCATCTACTACGAAGCCATAGCTGTTAGAAAGGTT
GAC---------GGCGAGTTTCGAATTAATTCC------AATTCATCTGAACCGGAGCCC
GGCTCGGCAGCCATTTTCGCCAATTATCCGACCCCTTATTCGTCCGTTCTTACCGGCTTA
ATGGAAGAGATTTTATCGACCATGATCTTCATGCTGATCATTTGCGTCGTGACCAATTCG
AGATATTCCAAAGTGCCG------------AATTTCTTGCAACCGCTCTACATTGGATTC
ACGTTGCTGGCCATCGGCGTGGCCTACGGATCCAACGGAGGCTACGCTCTTAACCCGGCT
CGTGATTTGGCTCCCAGGTTAGTCACGTATTTCGGAGGATGGGGACCGCGCGTATTTAGC
TTCCGCAGCTACAATTGGTTCTGGATTTTCCTGGTCGGGCCACACGTGGGAGCTCTTCTC
GGCGTCGGCATTTTC---CATGCCGTTTTCTTGCGATTGGAGCAAACAGCGGTC------
---GCAGCAGAT---GGA---ACGGATTCGCAGCTACAAATGGTGGACTACGAATATGCA
ATGGCCAACGTACCCGATAACAAATCAAGCGTC------ATCAAGGTGACG---AGAAGG
---------------------------------CAA---AGCCACGAAGAATATGTTGGA
TGCAGCATTGTCACGCCGATG---------------------------------------
------------------------------------------------------------
------------------------------------------------------------
------------------------------------------------------------
------------------------------------------------------------
------------------------------------------------------------
------------------------------------------------------------
------------------------------------------------------------
------------------------------------------------------------
------------------------------------------------------------
------------------------------------------------------------
------------------------------------------------------------
------------------------------------------------------------
------------------------------------------------------------
------------------------------------------------------------
------------------------------------------------------------
------------------------------------------------------------
------------------------------------------------------------
------------------------------------------------------------
------------------------------------------------------------
------
>MG254756_Daphnia_iaTibetana_GlpB2
------------------------------------------------------------
------------------------------------------------------------
---------ATG---ACG---CACAATCACCGCGATGAGAGA---CTGGCGTTGCCTTCC
AACCGCAAT------TTGCCCAGTCTGACTCGAGCAGTTCTGGCTGAGTTTGTTGGCACC
TTTATCCTTGTCGCTGTTGGCAGTGGTAGTGTGGCTCAATCGCAATCGACCAATGGCCAA

AAAGGCGACTACTTTACCATCAACTGGGGATGGTTCTTAGGATGCACCATGGGCATCCTC
GTTTCAGCCAAAGCGTCAGGCGGTCATCTGAATCCGGCGGTGACGATGGCCCTGACCGTG
GCCAAGGATTTCCCGTGGAAAAGGCTTCCTGTTTATTGGTTTGCCCAGTATCTGGGGGCT
CTGGTGGCATCGGGCACGGTGCTCGGCATCTACTTTGAAGCCATCATTGAGAAAAAAGAT
GGA---------GGTGAGTTTCGGATCAATTTC------AATTCGTCGGAACCGGAGCCG
GGCTCAGCTGCCATTTTCGCCAATTATCCGGCACCTTATTCGTCAGTTCTCACCGGTTTG
ATGGAGGAGGTTTTATCGACGATGATATTCATGCTGGTCATATGCGTCGTGACCAATTCG
AAATATTCAAAAGTGCCG------------ACTTTCTTGCAGCCGTTGTACATTGGTCTC
TCCTTGCTGGCCATGGGCGTGGCTTACGGATCCAATGGAGGCTACGCCCTTAACCCGGCC
CGCGATTTGGCTCCCCGATTGGTGACGTATTTTGGTGGATGGGGACCTCGCGTATTCAGC
TTCCGCGATTACAATTGGTTCTGGATTTTCTTGGTCGGACCACACGTTGGAGCTCTTCTT
GGTGTCGCTATTTTT---TATTCCGTTTTATTG------GATGGAGCGCCGATT------
---GGATCCGAC---AGC---GACGATTCGCAGCTGCCAGTGGTCGATTACCAGTATGCA
TTGGATCATCTACCCGACGAAAAATCTAATGTC------TTCAAAGTAACG---AGAAGA
---------------------------------CAG---AGCCATGAAGAATACGTTCGA
TGTAACATCGTCGCTCCAATG---------------------------------------
------------------------------------------------------------
------------------------------------------------------------
------------------------------------------------------------
------------------------------------------------------------
------------------------------------------------------------
------------------------------------------------------------
------------------------------------------------------------
------------------------------------------------------------
------------------------------------------------------------
------------------------------------------------------------
------------------------------------------------------------
------------------------------------------------------------
------------------------------------------------------------
------------------------------------------------------------
------------------------------------------------------------
------------------------------------------------------------
------------------------------------------------------------
------------------------------------------------------------
------------------------------------------------------------
------
>CM023192_Daphnia_carinata_GlpB1
------------------------------------------------------------
------------------------------------------------------------
---------ATG---ACG---AGC------AAAGGAGACAAT------CGTAATTGTCGT
TTGTCCAGC------------GCACTCGTTCGCGCCGCCTTAGCCGAGTTTATTGGCACG
TACATCCTTGTTGTTATTGGCAATGGTAGTGTGGCCCAGTCGCAACTGACGAATGGCAAA
CAAGGCGACTACTTCACCATCAACTGGGGATGGGCTTTGGGATGCTCGTTGGGAATCCTC
ATTTCGGCCGAAGCATCAGGTGGTCACTTGAACCCGGCCGTGACGCTGGCCCTGGCCGTG
GCGAAAGATTTCCCGTGGAAAAGGCTACCCGTGTATTGGTTAGCGCAGTATTTTGGAGCG
CTGGTGGCGTCTGGCAGCGTCCTCGGCGTTTATTACGAAGCCATCCTCGTTGGAAAACGA
GAC---------GGAAAGTTTCGAATTAACGCG------AATTCGTCCGAACCGGAGCCA
GGCTCTGCGGCCATATTTGCTAATTATCCGACTCCTTATTCGTCAGTGATTACTGGTTTA

GTAGAAGAGACTTTATGTACAATGATCTTTATGCTGATTATCTGCGTGGTGACCAATGCG
AAATACTCAAAAGTGCCG------------AGCTTCTTGCAGCCGCTCTACATTGGTTTC
ACGTTGTTGGCCATGGGCGTGGCCTACGGATCGAATGGAGGCTACGCGCTAAATCCCGCC
CGCGATTTAGCTCCCCGATTAGTTACGTATTTCGGAGGATGGGACCTCGTGTATTTAGC
TTCCGCGGCTACAATTGGTTCTGGATTTTCTTAGTCGGGCCACATGTGGGGGCCCTTCTT
GGCGTCGGTATTTTT---CATACCGTTTTCTTTCTACCAGAACAGACGGTGGTT------
---GCGTTCGAC---AGA---ACCGATTCACAGCTACCAATTGTCAATTACGAATATGCA
ATGGCCAACTTACCTGTTAACAAATCGAGTGCT------ATCAAAATTACG---AGA---
---------------------------------CAAGAGAGCCACGAAGAATATGTTGGG
TGCCGGGTGGTTACAGCAATG---------------------------------------
------------------------------------------------------------
------------------------------------------------------------
------------------------------------------------------------
------------------------------------------------------------
------------------------------------------------------------
------------------------------------------------------------
------------------------------------------------------------
------------------------------------------------------------
------------------------------------------------------------
------------------------------------------------------------
------------------------------------------------------------
------------------------------------------------------------
------------------------------------------------------------
------------------------------------------------------------
------------------------------------------------------------
------------------------------------------------------------
------------------------------------------------------------
------------------------------------------------------------
------------------------------------------------------------
------
>KAI9557849_Daphnia_sinensis_GlpB1
------------------------------------------------------------
------------------------------------------------------------
---------ATG---ACG---AGC------AAAGGAGACAAT------CGTAATTGTCGT
TTGTCCAGC------------GCACTCGTTCGCGCCGCCTTAGCCGAGTTTATTGGCACG
TACATCCTTGTTGTTATTGGCAATGGTAGTGTGGCCCAGTCGCAACTGACGAATGGCAAA
CAAGGCGACTACTTCACCATCAACTGGGGATGGGCTTTGGGATGCTCGTTGGGAATCCTC
ATTTCGGCCGAAGCATCAGGTGGTCACTTGAACCCGGCCGTGACGCTGGCCCTGGCCGTG
GCGAAAGATTTCCCGTGGAAAAGGCTACCCGTGTATTGGTTAGCGCAGTATTTTGGAGCG
CTGGTGGCGTCTGGCAGCGTCCTCGGCGTTTATTACGAAGCCATCCTCGTTGGAAAACGA
GAC---------GGAAAGTTTCGAATTAACGCG------AATTCGTCCGAACCGGAGCCA
GGCTCTGCGGCCATATTTGCTAATTATCCGACTCCTTATTCGTCAGTGATTACTGGTTTA
GTAGAAGAGACTTTATGTACAATGATCTTTATGCTGATTATCTGCGTGGTGACCAATGCG
AAATACTCAAAAGTGCCG------------AGCTTCTTGCAGCCGCTCTACATTGGTTTC
ACGTTGTTGGCCATGGGCGTGGCCTACGGATCGAATGGAGGCTACGCGCTAAATCCCGCC
CGCGATTTAGCTCCCCGATTAGTTACGTATTTCGGAGGATGGGACCTCGTGTATTTAGC
TTCCGCGGCTACAATTGGTTCTGGATTTTCTTAGTCGGGCCACATGTGGGGGCCCTTCTT
GGCGTCGGTATTTTT---CATACCGTTTTCTTTCTACCAGAACAGACGGTGGTT------

---GCGTTCGAC---AGA---ACCGATTCACAGCTACCAATTGTCAATTACGAATATGCA
ATGGCCAACTTACCTGTTAACAAATCGAGTGCT------ATCAAAATTACG---AGA---
---------------------------------CAAGAGAGCCACGAAGAATATGTTGGG
TGCCGGGTGGTTACAGCAATG---------------------------------------
------------------------------------------------------------
------------------------------------------------------------
------------------------------------------------------------
------------------------------------------------------------
------------------------------------------------------------
------------------------------------------------------------
------------------------------------------------------------
------------------------------------------------------------
------------------------------------------------------------
------------------------------------------------------------
------------------------------------------------------------
------------------------------------------------------------
------------------------------------------------------------
------------------------------------------------------------
------------------------------------------------------------
------------------------------------------------------------
------------------------------------------------------------
------------------------------------------------------------
------------------------------------------------------------
------
>NC_046177_Daphnia_magna_GlpB1
------------------------------------------------------------
------------------------------------------------------------
---------ATGGCG------AGC------AAAGGAGAC---------AAGAATTGTCGT
TTGTCCAGC------------ACACTTGTTCGCGCCGCCTTAGCCGAGTTTATTGGCACC
TACATTCTTGTTGTCATTGGCAACGGTAGTGTTGCCCAGTCGCAACTGACGAATGGCAAA
CAAGGCGATTATTTCACCATCAACTGGGGATGGGCTTTGGGATGCTCGTTGGGAATCCTC
ATTTCGGCCGAGGCATCAGGTGGTCACTTGAACCCGGCCGTGACGCTGGCCCTGGCCGTG
GCTAAAGATTTCCCATGGAAACGGCTACCCGTGTATTGGTTAGCCCAGTATTTTGGAGCG
CTGGTTGCGTCTGGCAGCGTCCTCGGTGTTTATTACGAAGCCATTCTCCTTGGAAAACGA
GAT---------GGAAAGTTTCGAATTAACGCC------AATTCGTCCGAACCGGAGCCT
GGCTCAGCGGCCATTTTTGCAAACTATCCGACTCCTTATTCGTCAGTGGTTACCGGTCTA
GTAGAAGAGACTTTATGTACAATGATCTTCATGCTGATTATATGCGTGGTGACGAATGCC
AAATATTCAAAAGTGCCG------------AGCTTCTTGCAGCCGCTCTACATTGGTTTC
ACGTTGTTGGCCATGGGCGTGGCTTACGGATCTAACGGAGGCTACGCGCTAAATCCGGCT
CGCGATTTGGCTCCCCGATTAGTTACTTATTTCGGAGGATGGGGACCTCGTGTATTTAGC
TTCCGCGGCTACAATTGGTTCTGGATTTTCCTAGTCGGGCCACATGTGGGTGCCCTTCTT
GGCGTCGGCATTTTT---CATGCCGTTTTCTTTCTACCAGAACAGAGAGTGGTT------
---GCGTTCGAC---AGA---ACCGATTCACAGCTACCAATTGTGAATTACGAATATGCA
ATGGCCAATGTACCCGATAACAAATCAAATGCT------TTCAAAATTACG---AAA---
---------------------------------CAAGAGAGCCACGAAGAATATGTTGGA
TGCCGAGTAGTTACAGCAATG---------------------------------------
------------------------------------------------------------
------------------------------------------------------------

------------------------------------------------------------
------------------------------------------------------------
------------------------------------------------------------
------------------------------------------------------------
------------------------------------------------------------
------------------------------------------------------------
------------------------------------------------------------
------------------------------------------------------------
------------------------------------------------------------
------------------------------------------------------------
------------------------------------------------------------
------------------------------------------------------------
------------------------------------------------------------
------------------------------------------------------------
------------------------------------------------------------
------------------------------------------------------------
------------------------------------------------------------
------
>HAFN01005095_Daphnia_galea_GlpA8
------------------------------------------------------------
------------------------------------------------------------
------------------------------------ATGAAA---ATGCGCGTGTGTTGT
GCCAATGGT------GTTCATCCTCTGGTTCGAGCCGCTTTGGCCGAATTTATTGGCACC
TTCATTCTTGTTGCCATCGGCAATGGCAGCGTAGCCCAATCGCAATTGACCCAAGGTGAA
AAAGGCAATTATTTCACTATCAATTGGGGATGGTGTTTAGGATGTTGCCTCGGAATTCTC
GTTTCAGCTAAAGCATCAGGTGGCCATTTGAATCCGGCTGTAACAATGGCTTTCGCAGTG
GCTAAAGATTTTCCATGGAAAAGGCTTCCAGTGTATTGGTTTGCCCAGTACCTAGGAGCT
CTAGCCGCGTCAGCCACTGTCCTTGGAGTTTACTACGAAGCTATTATCATTAGAAAAGTT
GAT---------GGCCATTTCAGAATTCATGAT------AATGCTTCTGAACCC------
GGGTCGGCGGCCATTTTTGCCAATTATCCGGCTCCTTATTCGTCGGTTTGTACTGGTTTA
GTCGAGGAGATTTTATGTACAACAATATTTATGATTGTTATCTGTTCGGTTACGAATCCC
AAGTATTCAAAAGTCCCT------------AGCTCCTTGCAGCCGCTCTACATTGGTTTC
ACTCTTTTGGCTGTCGGAGTAGCTTACGGTTCCAACGGAGGATACGCTCTTAACCCGGCT
CGTGATTTGGCTCCTCGACTAGTTTCTTACTTCGGTGGATTTGGATCACGCGTATTTAGT
TTCCGTGGTTACAATTGGTTTTGGATCTTTTTGGTTGGACCGCACGTGGGAGCCATTTTA
GGTGTTTTTATCTTT---CACTTTGTTCTTCTGGAAACGAATCAACCGGCGAAA------
---GTAGAAGGA---AAAATGAATAATTCGCAGTTGCCAATCGTGGATTATGAATATGAG
GTGGCGAATGAGATAGACAGCAAACCTCAAGCCAATACCAGTAAAACATCTGAGCGACGT
CTA------------------------------CAATATCATTCTGAAGAGTATGTTGAC
------ATCGCTTCACCCATT---------------------------------------
------------------------------------------------------------
------------------------------------------------------------
------------------------------------------------------------
------------------------------------------------------------
------------------------------------------------------------
------------------------------------------------------------
------------------------------------------------------------
------------------------------------------------------------

------------------------------------------------------------
------------------------------------------------------------
------------------------------------------------------------
------------------------------------------------------------
------------------------------------------------------------
------------------------------------------------------------
------------------------------------------------------------
------------------------------------------------------------
------------------------------------------------------------
------------------------------------------------------------
------------------------------------------------------------
------
>JAAVJA010000055_Daphnia_dubai_GlpA8
------------------------------------------------------------
------------------------------------------------------------
------------------------------------------------------------
------------------------------------------GCCGAATTTATTGGCACC
TTCATCCTTGTCGTCATAGGTAATGGCAGCGTAGCCCAATCGCAATTGACTCAAGGTGAA
AAAGGCAATTATTTCACGATTAACTGGGGGTGGTGTTTAGGTTGTTGTCTCGGAATTCTC
GTTTCAGCTAAAGCATCAGGTGGCCATTTAAATCCTGCTGTTACAATGGCTCTCGCAGTG
GCAAAAGATTTTCCGTGGAAAAGGCTTCCAGTGTATTGGTTGGCGCAGTATCTAGGAGCC
CTAGCTGCGTCTGGTACTGTTCTTGGCGTTTATTACGAAGGAATTATCGTCAGGAAAGTT
GAT---------GGTCATTTCAGGATTCATGCC------AATGCTTCTGATCCC------
GGATCGGCGGCCATTTTTGCCAATTATCCGGCTCCTTATTCGTCAGTTTGTACTGGTTTA
ATAGAAGAGATTTTATGTACAACAATATTTATGCTTGTTATATGCGCAGTAACGAATCCT
AAATATTCAAAAGTCCCT------------AGTTCCTTGCAGCCAATCTACATTGGATTC
ACGCTTTTGGCCGTCGGAGTAGCTTACGGATCTAACGGAGGATACGCTCTTAACCCGGCT
CGTGATTTGGCTCCTCGTCTAGTTTCTTATATCGGTGGATTTGGTCCACGCGTCTTCAGT
TTCCGTGGCTACAATTGGTTTTGGATCTTTTTGGTTGGACCGCACGTGGGAGCCATTTTA
GGTGTTTTCATCTTT---CACTTCATACTCTTGGAAACGGACCAACCAGCAAAG---GAC
---GTAGCAGAA---AAAATGAATAATTCCCAATTGCCAATCGTGGAGTATGAATATGAA
ATGGCGAATGAACTGGGCAACAAAACTCAGGCCAATACCAGCAAAACCTCTGAGCAGCGT
CTG------------------------------CAACATCATTGCGAAGAATATGTTGAC
------ATCGCATCGCCAATT---------------------------------------
------------------------------------------------------------
------------------------------------------------------------
------------------------------------------------------------
------------------------------------------------------------
------------------------------------------------------------
------------------------------------------------------------
------------------------------------------------------------
------------------------------------------------------------
------------------------------------------------------------
------------------------------------------------------------
------------------------------------------------------------
------------------------------------------------------------
------------------------------------------------------------
------------------------------------------------------------

------------------------------------------------------------
------------------------------------------------------------
------------------------------------------------------------
------------------------------------------------------------
------------------------------------------------------------
------
>EFX66203_Daphnia_pulex_GlpA8
------------------------------------------------------------
------------------------------------------------------------
---------------------------------------------ATGCGT---CTGTGC
---TGTCGT------GTTCATCCCCTGGTTCGAGCCGCTTTTGCCGAATTTATTGGCACC
TTCATTCTTGTTGCCATCGGAAATGGAAGCGTAGCCCAATCACAATTGACCCAAGGCGAA
AAGGGCAATTATTTCACTATCAACTGGGGATGGTGTATTGGATGTTGCCTTGGAATTCTC
GTTTCAGCTAAAGCATCAGGTGGCCATCTGAATCCAGCAGTAACAATGGCTCTCGCAGTG
GCAAAAGATTTCCCGTGGAAAAGGCTTCCAGTGTACTGGTTTGCACAGTACTTGGGAGCT
CTGGCTGCGTCTGGTACTGTCCTTGGAGTTTATTACGAAGCAATTATCGTTAGAAAAATT
GAT---------GGCAAATTCAGGATTCATGCC------AATGCTTCTGAGCCC------
GGATCGGCTGCCATTTTTGCCAATTATCCGGCCCCTTATTCGTCGGTTGTTACCGGTTTA
GTCGAGGAGATTTTATGTACAACGATATTTATGCTGGTTATATGCGTGGTAACAAATCCA
AAGTATTCAAAAGTCCCC------------AGTTTTTTGCAGCCGTTCTACATTGGTTTC
ACCCTTTTGGCCGTCGGTGTGGCTTACGGATCTAATGGAGGATACGCTCTTAACCCGGCT
CGTGATTTAGCTCCTCGACTTGTTTCTTATTTCGGTGGATTTGGATCACGCGTTTTCAGT
TTCCGTGGATACAATTGGTTTTGGATCTTTTTGGTTGGGCCGCATGTTGGAGCCATTCTG
GGTGTTTTTATCTTT---CATTTTATTCTCATGGAAACGGACAAACCAGCGAGC------
---GTAGAAGAA---AAAATGAATAGTTCGCAGTTGCCAATCGTGGATTACGAATATGAA
ATGGCGAATGAGGTGGACGTCAAGCCCCAAGCCAATAACAACAAAGCTCCTGAGCAGCGT
CTG------------------------------CAATATCAATGTGAAGAATACGTTGAC
------ATCGCATCGCCAATT---------------------------------------
------------------------------------------------------------
------------------------------------------------------------
------------------------------------------------------------
------------------------------------------------------------
------------------------------------------------------------
------------------------------------------------------------
------------------------------------------------------------
------------------------------------------------------------
------------------------------------------------------------
------------------------------------------------------------
------------------------------------------------------------
------------------------------------------------------------
------------------------------------------------------------
------------------------------------------------------------
------------------------------------------------------------
------------------------------------------------------------
------------------------------------------------------------
------------------------------------------------------------
------------------------------------------------------------
------

```
>XM_046779672_Daphnia_pulicaria_GlpA8
------------------------------------------------------------
------------------------------------------------------------
---------------------------------------------ATGCGT---CTGTGC
---TGTCGT------GTTCATCCCCTGGTTCGAGCCGCTTTTGCCGAATTTATTGGCACC
TTCATTCTTGTTGCCATCGGAAATGGAAGCGTAGCCCAATCACAATTGACCCAAGGCGAA
AAGGGCAATTATTTCACTATCAACTGGGGATGGTGTATTGGATGTTGCCTTGGAATTCTC
GTTTCAGCTAAAGCATCAGGTGGCCATCTGAATCCAGCAGTTACAATGGCTCTTGCTGTG
GCAAAAGATTTCCCGTGGAAAAGGCTTCCAGTGTACTGGTTTGCACAGTACTTGGGAGCT
CTGGCTGCGTCTGGTACTGTCCTTGGCGTTTATTACGAAGCAATTATCGTTAGAAAAATT
GAT---------GGCAAATTCAGGATTCATGCC------AATGCTTCTGAGCCC------
GGATCGGCTGCCATTTTTGCCAATTATCCGGCCCCTTATTCGTCAGTTGTTACTGGTTTA
GTAGAGGAGATTTTATGTACAACGATATTTATGCTGGTTATATGCGTGGTAACAAATCCA
AAGTATTCAAAAGTCCCC------------AGTTTTTTGCAGCCGTTCTACATTGGTTTC
ACTCTTTTGGCCGTCGGAGTGGCTTACGGATCTAATGGAGGATACGCTCTTAACCCGGCT
CGTGATTTGGCTCCTCGACTTGTTTCTTATTTCGGTGGATTTGGATCACGCGTATTCAGT
TTCCGTGGATACAATTGGTTTTGGATCTTTTTGGTTGGGCCGCATGTTGGAGCCATTCTG
GGTGTATTTATCTTT---CATTTTATTCTCATGGAAACGGACCAACCAGCGAGC------
---GTAGAAGAA---AAAATGAATAGTTCGCAGTTGCCAATCGTGGATTACGAATATGAA
ATGGCGAATGAGCTGGACGTCAAACCTCAAGCCAATAACAGCAAAGCTTCTGAGCCGCGT
CTG------------------------------CAATATCAATGTGAAGAATACGTTGAC
------ATCGCATCGCCAATT---------------------------------------
------------------------------------------------------------
------------------------------------------------------------
------------------------------------------------------------
------------------------------------------------------------
------------------------------------------------------------
------------------------------------------------------------
------------------------------------------------------------
------------------------------------------------------------
------------------------------------------------------------
------------------------------------------------------------
------------------------------------------------------------
------------------------------------------------------------
------------------------------------------------------------
------------------------------------------------------------
------------------------------------------------------------
------------------------------------------------------------
------------------------------------------------------------
------------------------------------------------------------
------------------------------------------------------------
------
>CM023188_Daphnia_carinata_GlpA8
------------------------------------------------------------
------------------------------------------------------------
---------------------------------------------ATGCGA---TCGAGG
---TGTACC------ATTCCTCCTCTGGCTCGAGCCGCCGTTGCCGAATTTATTGGCACC
TTCGTTCTCGTTACGATTGGCAGTGGTAGTGTAGCTCAATCGCAATTGACCGACGGTGCA
```

AAGGGTAATTACTTCACCATCAACTGGGGATGGGCCATAGGATGTTCCTTAGGAATCCTC
GTATCGGCTAAAGCATCAGGTGGCCACTTAAATCCGGCTGTGACAATGGCTCTTGCAGTG
GGCAAAGATTTCCCGTGGAAAAGATTACCTGTGTATTGGTGTGCCCAATACTTGGGATCT
CTGGCCGCATCCGGGACAGTCCTAGGAGTTTATTACGAGGCCATAGCCATGTACAAAATC
GAC---------GGCCAATTCCGCATTAATGCA------AATGCTTCCGAGCCA------
GGCTCAGCCGCCATTTTCGCCAACTATCCAGCTGCTTATTCCTCCGTTATCACCGGCCTC
ATCGAAGAGATTTTGTGTACCACGATATTCATGATTGTTATCTGCGTCGTGACTAATCCG
AAACACCTGAAAGTACCC------------AGTTACTTGCAGCCACTTTACATCGGTTTC
ACCTTGTTGGCAGTTGGCGTTGCTTATGGATCTAATGGAGGCTACGCTTTGAACCCGGCT
CGTGATTTGGCTCCACGGTTGGTTTCGTACCTTGGTGGATGGGGAACACGAGTGTTTAGC
TTCCGCAACTACAATTGGTTTTGGATTTTTCTTGTCGGACCACATGTGGGAGCCATTCTT
GGTGTTGGTATTTTT---CATTTCCTCATCTTGGAAAAAGACCAGATCCCGGAAACA---
---ACG---AGC---AAAATGAACAGTTCACAAGCGCCAATTCTCGAATACGAATATCAA
ATGAACAATGGGATGGAAAACAGG---CAG---AATTTCAGTAAACTGCTTGAGCAACGA
TTA------------------------------CCTTATCGCAGTGACGAAAATGCGGGG
GGTAACATCATCACACAAATT---------------------------------------
------------------------------------------------------------
------------------------------------------------------------
------------------------------------------------------------
------------------------------------------------------------
------------------------------------------------------------
------------------------------------------------------------
------------------------------------------------------------
------------------------------------------------------------
------------------------------------------------------------
------------------------------------------------------------
------------------------------------------------------------
------------------------------------------------------------
------------------------------------------------------------
------------------------------------------------------------
------------------------------------------------------------
------------------------------------------------------------
------------------------------------------------------------
------------------------------------------------------------
------------------------------------------------------------
------
>KAI9565893_Daphnia_sinensis_GlpA8
------------------------------------------------------------
------------------------------------------------------------
---------------------------------------------ATGCGA---TCGAGG
---TGTACC------ATTCCTCCTCTGGCTCGAGCCGCCGTTGCCGAATTTATTGGCACC
TTCGTTCTCGTTACGATTGGCAGTGGTAGTGTAGCTCAATCGCAATTGACCGACGGTGCA
AAGGGTAATTACTTCACCATCAACTGGGGATGGGCCATAGGATGTTCCTTAGGAATCCTC
GTATCGGCTAAAGCATCAGGTGGCCACTTAAATCCGGCTGTGACAATGGCTCTTGCAGTG
GGCAAAGATTTCCCGTGGAAAAGATTACCTGTGTATTGGTGTGCCCAATACTTGGGATCT
CTGGCCGCATCCGGGACAGTCCTAGGAGTTTATTACGAGGCCATAGCCATGTACAAAATC
GAC---------GGCCAATTCCGCATTAATGCA------AATGCTTCCGAGCCA------
GGCTCAGCCGCCATTTTCGCCAACTATCCAGCTGCTTATTCCTCCGTTATCACCGGCCTC

```
ATCGAAGAGATTTTGTGTACCACGATATTCATGATTGTTATCTGCGTCGTGACTAATCCG
AAACACCTGAAAGTACCC------------AGTTACTTGCAGCCACTTTACATCGGTTTC
ACCTTGTTGGCAGTTGGCGTTGCTTATGGATCTAATGGAGGCTACGCTTTGAACCCGGCT
CGTGATTTGGCTCCACGGTTGGTTTCGTACCTTGGTGGATGGGGAACACGAGTGTTCAGC
TTCCGCAACTACAATTGGTTTTGGATTTTTCTTGTCGGACCACATGTGGGAGCCATTCTT
GGTGTTGGTATTTTT---CATTTCCTCATCTTGGAAAAAGACCAGATCCCGGAAACA---
---ACG---AGC---AAAATGAACAGTTCACAAGCGCCAATTCTCGAATACGAATATCAA
ATGAACAATGGGATGGAAAACAGG---CAG---AATTTCAGTAAACTGCTTGAGCAACGA
TTA------------------------------CCTTATCGCAGTGACGAAAATGCGGGG
GGTAACATCATCACACAAATT---------------------------------------
------------------------------------------------------------
------------------------------------------------------------
------------------------------------------------------------
------------------------------------------------------------
------------------------------------------------------------
------------------------------------------------------------
------------------------------------------------------------
------------------------------------------------------------
------------------------------------------------------------
------------------------------------------------------------
------------------------------------------------------------
------------------------------------------------------------
------------------------------------------------------------
------------------------------------------------------------
------------------------------------------------------------
------------------------------------------------------------
------------------------------------------------------------
------------------------------------------------------------
------------------------------------------------------------
------
>NC_046175_Daphnia_magna_GlpA8
------------------------------------------------------------
------------------------------------------------------------
---------------------------------------------ATGCGA---TCTAGG
---TGTACC------ATTCCTCCCTTGGCACGAGCCGCCGTTGCCGAATTTATTGGCACC
TTCGTTCTCGTTACTATTGGCAGCGGTAGTGTAGCTCAATCGCAATTGACCGACGGTGCA
AAGGGCAACTACTTCACCATCAACTGGGGATGGGCTATAGGATGTTCTTTAGGAATCCTC
GTATCGGCTAAAGCATCAGGTGGCCACTTAAATCCGGCTGTGACAATGGCTCTTGCAGTG
GGCAAAGATTTCCCGTGGAAAAGACTACCCGTGTATTGGTGTGCCCAATACTTGGGGGCT
CTGGCCGCGTCCGGGACAGTCCTAGGCGTTTATTACGAAGCCATAGCCATGCACAAGGTG
GAT---------GGTCAATTCCGCATAAACGCA------AATGCTTCCGAGCCA------
GGCTCAGCCGCCATTTTCGCCAACTATCCAGCTGCTTATTCCTCGGTTATCACTGGCCTT
ATCGAAGAGGTTTTTTGTACGACGATATTCATGCTTGTCATTTGCGTCGTGACCAATCCG
AAACACTTGAAAGTACCA------------AGTTATTTGCAGCCAATTTACATTGGTTTC
ACCTTGTTGGCAGTTGGCGTAGCTTATGGATCTAATGGAGGCTACGCTTTGAACCCGGCT
CGTGATTTGGCTCCACGATTGGTTTCTTATCTTGGTGGATGGGGAAAACGAGTGTTCAGC
TTCCGTGGCTACAATTGGTTTTGGATTTTTCTTGTTGGACCCCATGTCGGAGCCATTATT
GGTGTCGGTATTTTC---CACTTTCTGATCTTGGAAGAAGACCAGACTCCGGAAACA---
```

```
---GTG---AAT---GCAATGGAAAATTCACAGGCGCCAATCATCGAATATGAATACCAA
ATGACCAATGGTCTGGAATACCAG---CAG---AATTTCAGTAAACTTTTTGAGCAACGA
TTA------------------------------CCTTACCGCAGTGACGGAAATATTGGG
TGTAACATC---ACACAAATT---------------------------------------
------------------------------------------------------------
------------------------------------------------------------
------------------------------------------------------------
------------------------------------------------------------
------------------------------------------------------------
------------------------------------------------------------
------------------------------------------------------------
------------------------------------------------------------
------------------------------------------------------------
------------------------------------------------------------
------------------------------------------------------------
------------------------------------------------------------
------------------------------------------------------------
------------------------------------------------------------
------------------------------------------------------------
------------------------------------------------------------
------------------------------------------------------------
------------------------------------------------------------
------------------------------------------------------------
------
>CM023188_Daphnia_carinata_GlpA9
------------------------------------------------------------
---------------------------------ATGCCTGGTCTTTTTGAGTTTTGGAAC
CGGAGAAAGAGAAGGGAT---TCGACATTGTCGGCCGAG------ATGCTG---TCTTGC
AGTCGGAAT------GATCCTTCCCTGACAAGGGCGGCTTTTGCGGAATTCATTGGAACC
TTCGTTCTCGTCGCTATCGGCAATGGAAGTGTTGCCCAGTCACTATTAACTAATGGCGAA
AAGGGCAACTATTTCACCATCAACTGGGGATGGGGTTTAGGATGTTTCTTAGGCATTCTC
GTATCCGCTAAAACATCAGGGGGTCACATAAATCCGGCCGTGACGATGGCTCTGGCAGTG
ACGAAAGAGTTCCCATGGAACCGGCTACCGGCATATTGGTGTGCACAATATCTGGGAGCT
CTTGCAGCATCTAGCACAGTTCTGTCTGTGTATTACGAGGCCATCATGAATGGTAAAGTG
AAC---------GGTGAATTCAAAATCCACAAG------GATCCTTCCGATCCG------
GGCTCAGCGGCTATTTTTGCTAATTATCCAGCTCCATATTCATCAGCTAGTGCTGGCCTG
ATGGAAGAGATTTTGTCCACTGTCATCTTCATGTTGGTTATCCGTGTGGTAACTGATAGG
CAA---TCAAAAGTACCC------------AGTTTTCAGCAACCGCTTTACATCGGTTTC
ACTTTATTGGCAATCGGCTTGTCTTACGGATCAAACGGCGGCTATGCACTGAATCCGGCT
AGAGATATTGCTCCTCGAGTTGTTACACTTTTTGCTGGATGGGGAATCCATGTTTTCAGC
ATCCGTGGATACAATTGGTTCTGGATTTATCTGGTGGGTCCGCACGTTGGAGCCATCCTA
GGCGTTGTCATCTTC---CATCTCTTCGTAAGACAAGATGGGACAATGGACGACACAAGA
---TCAGAATCT---CCTTCAATCGATTTGGAAAATGGA---------TATGGCAAAGAG
CTTGATGTGATATGGCGGTTGAACGAAAATCGGGACATCGAATCCGATATGAGCAAGCTG
GACAAAGAAAATCCTCCGCGTTACAGCGTAGCCTACGGTAGCAGCAGACCGCAATTAGGA
CGTGGCCTATCGTGTCCAGTCGTGACCACGAGTAGG------------------------
------------------------------------------------------------
------------------------------------------------------------
```

------------------------------------------------------------
------------------------------------------------------------
------------------------------------------------------------
------------------------------------------------------------
------------------------------------------------------------
------------------------------------------------------------
------------------------------------------------------------
------------------------------------------------------------
------------------------------------------------------------
------------------------------------------------------------
------------------------------------------------------------
------------------------------------------------------------
------------------------------------------------------------
------------------------------------------------------------
------------------------------------------------------------
------------------------------------------------------------
------------------------------------------------------------
------
>KAI9565894_Daphnia_sinensis_GlpA9
------------------------------------------------------------
---------------------------------ATGCCTGGTCTTTTTGAGTTTTGGAAC
CGGAGAAAGAGAAGGGAT---TCGACATTGTCGGCCGAG------ATGCTG---TCTTGC
AGTCGGAAT------GATCCTTCCCTGACAAGGGCGGCTTTTGCGGAATTCATTGGAACC
TTCGTTCTCGTCGCTATCGGCAATGGAAGTGTTGCCCAGTCACTATTAACTAATGGCGAA
AAGGGCAACTATTTCACCATCAACTGGGGATGGGGTTTAGGATGTTTCTTAGGCATTCTC
GTATCCGCTAAAACATCAGGGGGTCACATAAATCCGGCCGTGACGATGGCTCTGGCAGTG
ACGAAAGAGTTCCCATGGAACCGGCTACCGGCATATTGGTGTGCACAATATCTGGGAGCT
CTTGCAGCATCTAGCACAGTTCTGTCTGTGTATTACGAGGCCATCATGAATGGTAAAGTG
AAC---------GGTGAATTCAAAATCCACAAG------GATCCTTCCGATCCG------
GGCTCAGCGGCTATTTTTGCTAATTATCCAGCTCCATATTCATCAGCTAGTGCTGGCCTG
ATGGAAGAGATTTTGTCCACTGTCATCTTCATGTTGGTTATCCGTGTGGTAACTGATAGG
CAA---TCAAAAGTACCC------------AGTTTTCAGCAACCGCTTTACATCGGTTTC
ACTTTATTGGCAATCGGCTTGTCTTACGGATCAAACGGCGGCTATGCACTGAATCCGGCT
AGAGATATTGCTCCTCGAGTTGTTACACTTTTTGCTGGATGGGGAATCCATGTTTTCAGC
ATCCGTGGATACAATTGGTTCTGGATTTATCTGGTGGGTCCGCACGTTGGAGCCATCCTA
GGCGTTGTCATCTTC---CATCTCTTCGTAAGACAAGATGGGACAATGGACGACACAAGA
---TCAGAATCT---CCTTCAATCGATTTGGAAAATGGA---------TATGGCAAAGAG
CTTGATGTGATATGGCGGTTGAACGAAAATCGGGACATCGAATCCGATATGAGCAAGCTG
GACAAAGAAAATCCTCCGCGTTACAGCGTAGCCTACGGTAGCAGCAGACCGCAATTAGGA
CGTGGCCTATCGTGTCCAGTCGTGACCACGAGTAGG------------------------
------------------------------------------------------------
------------------------------------------------------------
------------------------------------------------------------
------------------------------------------------------------
------------------------------------------------------------
------------------------------------------------------------
------------------------------------------------------------
------------------------------------------------------------

------------------------------------------------------------
------------------------------------------------------------
------------------------------------------------------------
------------------------------------------------------------
------------------------------------------------------------
------------------------------------------------------------
------------------------------------------------------------
------------------------------------------------------------
------------------------------------------------------------
------------------------------------------------------------
------------------------------------------------------------
------
>NC_046175_Daphnia_magna_GlpA9
------------------------------------------------------------
---------------------------------ATGCCTGGTCTTTTTGAGTTTTGGAAT
AGGAAAAAGAGGAGAGAT---TCGACATTGTCGTCCCAGACT---ATGTTA---TCCTGT
AGTGGGACT------GATCCCTCCCTCACTCGGGCGGCGTTTGCAGAATTCATAGGGACC
TTCGTTCTCGTTGCTATAGGCAATGGCAGTGTGGCCCAGTCGCAGTTAACGAATGGCGAA
AAGGGCAACTACTTCACCATCAACTGGGGATGGGCTTTAGGATGTTCCTTGGGCATTCTC
ATATCCGCTAAAACATCAGGGGGTCACATAAATCCGGCCGTGACGATGGCTCTGGCGGTA
GCGAAAGAATTTCCATGGAAACGGCTACCTGCATATTGGTGTGCACAGTATTTGGGAGCT
CTCGCAGCGTCTAGCACGGTTCTAGGAGTCTATTACGAGGCCATCATGAACGGCAAAGTC
AAC---------GGGGAGTTCAAAATCCAAAAG------GATTCTTCCGATCCG------
GGCTCAGCGGCTATTTTTGCTAATTATCCAGCTCCATATTCATCAGCTAGTACTGGCCTA
ATGGAAGAGATTTTGTCCACTGTGGTCTTCATGTTGGTTATCCGTGTGGTAACAGATAGG
CAA---TCAAAAGTACCC------------AGTTTTCTGCAACCGCTTTACATTGGGTTC
ACTTTACTGGCAATCGGCGTGTCCTATGGATCGAACGGCGGCTATGCTCTGAATCCGGCT
AGAGATTTAGCTCCTCGAGTTGTTACACTTTTCGCTGGATGGGGAAGCCATGTTTTCAGC
TTCCGTGGATACAATTGGTTCTGGATTTATCTGCTGGGTCCTCACGTTGGAGCCATTCTA
GGCATAGGCATCTTC---AATCTCTTCCTGAAGTCGGATGGAACAATGGACGACACAAGA
---TCAGAATCT---CCTTGTATCGATTTAGAAAATGGA---------TATGGTAAAGAG
CTTGATGTGATATGGCGGTTGAACGAAAATCGTGACATTGAACCCGATATGAACAAGCTG
GATAAAGAAAATCCTCCGCGGTATAGCGTAGCCTACGCTAGCAGCAGACCGCAGTTAGGC
CGTGGCCTATCGTGTCCAGTCGTCACCTCGAATAGG------------------------
------------------------------------------------------------
------------------------------------------------------------
------------------------------------------------------------
------------------------------------------------------------
------------------------------------------------------------
------------------------------------------------------------
------------------------------------------------------------
------------------------------------------------------------
------------------------------------------------------------
------------------------------------------------------------
------------------------------------------------------------
------------------------------------------------------------
------------------------------------------------------------
------------------------------------------------------------

------------------------------------------------------------
------------------------------------------------------------
------------------------------------------------------------
------------------------------------------------------------
------------------------------------------------------------
------
>JAKUCG010000092_Ceriodaphnia_dubia_GlpA9
------------------------------------------------------------
---------------------------------ATGCGCGCCATTTTTGAACGCTGGTAC
AGGAGAGGAAGCAGAGAA---ACGCCTTTGCGGGAAGAT---------------------
---------------GGTCCGTCACTGACTCGAGCTGCCATTGCAGAATTCATCGGCACC
TTTATCCTCGTTGCCATCGGCAACGGTAGCGTCGCTCAGTCGCAATTGACCAGAGGTGAA
AAAGGCGATTACTTTACCATCAACTGGGGATGGGCTTTAGGTTGCTCGTTAGGAATCTTC
GTTTCGTCGAGCGTCTCGGGGGGACATTTGAACCCGGCGGTAACGATGGCTCTGGCTTTC
ACTAAAGAATTCCCTTGGAAGAAGGTGCCCGCTTATTTCCTTGCCCAGTACTTGGGGGCT
CTGGCCGCGTCCGGCATGGTCCTCGGTGTCTATTACGAGGCTATCATGCAACTAAGAGGC
AAT---------GGCGAATTCCGCATCCGCAAC------GATCCAACCGATCCG------
GGCTCGGCGTCTATTTTTGCCAACTATCCAGCTCCCTACTTGTCGGCCAGTACCGGATTA
GTGGAAGAAATTTTGTCGACGATGATCTTTATGCTCGTCATCTGCGTCACGACGGATAAA
AATTATTCAAAGGTGCCC------------AGCTATTTGCAGCCGCTCTTTATCGGGTTC
ACCTTGTTGGCCATCGGAGTGGCCTACGGTTCCAACGGTGGATACGCGCTCAATCCGGCC
CGTGACTTTGCACCTCGTCTCGTTTCCTTATTTGGCGGATGGGGAATCAACGTATTTAGG
TTTCGTGGTTACAATTGGTTCTGGATTTATCTGGTGGGACCGCACATCGGAGCTATTCTG
GGCGTCTGCATTTTC---CAACTTTTACTCAAAGACCAAAAAAGCGTCGTCGACGGATGC
---GACGGTGCC---AGTCTCTCCGAGTCCGAATCGCCCGTCGTTGATTTTGGAAAAGTG
CTGGACGCTCAGTGGCAACTCTAC---------GACATTGAGCCGGACATGAACAAATGC
GAACGAGATAAGCCTCCGAGTTAC---------CACCGCACGTCGTGGCCGCAAGTTTTC
AAATCGCCGGCCAGCTCCAGCATTTTT---------------------------------
------------------------------------------------------------
------------------------------------------------------------
------------------------------------------------------------
------------------------------------------------------------
------------------------------------------------------------
------------------------------------------------------------
------------------------------------------------------------
------------------------------------------------------------
------------------------------------------------------------
------------------------------------------------------------
------------------------------------------------------------
------------------------------------------------------------
------------------------------------------------------------
------------------------------------------------------------
------------------------------------------------------------
------------------------------------------------------------
------------------------------------------------------------
------------------------------------------------------------
------------------------------------------------------------
------

```
>GGXW01231656_HAFN01017572_Daphnia_galeata_GlpA6
------------------------------------------------------------
------------------------------------------------------------
---------------------------------------------ATGTGT---AAAAGG
TTTCAC---------GTTCCATCATTGTTTCGAGCAGCATTTGCTGAATTTATGGGTACC
TACATCTTGGTTGTCATTGGAAACGGGAGCATAGCACAATCACAGTTAACCAATGGCGAA
AAAGGTGATTACTTCACGATAAACTGGGGATGGCTTTAGGTTGTTCGTTGGGAATTCTC
ATTTCAGCTAATATATCAGGTGGTCATTTAAACCCAGCAGTTACACTGGCTCTGGCATTA
GTTCGTCATTTTCCGTGGAAAAAATTACCTGTATATTGGTGTGCTCAGTATTTGGGAGCG
ATGGCCGCTTCTGGAACTGTTCTTGGAGTTTATTATGAGGGAATCTTGGAAGGAAAAGTC
GGC---------GGTGAATTTCGAATCAGGAAG------AACATTACCGAACCG------
GGTATGGCATCTATTTTTGCCAACTATCCAGCCCCATATTCGTCGGCTAGCATTGGACTT
GTTGAGGAGATTTTATCGACTATGATATTCATGCTCGTTATATGCGTTGTGACTGACAAA
AGGTATTCATGCGTGCCG------------AATTTCTTGCAACCACTCTACATCGGCTTT
GCCCTGTTGGCCATCGGCGTTGCGTACGGATCGAATGGAGGTTACGCTCTCAACCCGGCT
CGTGATTTGGCTCCTCGGTTGGTCTCATTTCTTGCCGGATGGGGACCAGGTGTATTTAGT
TTTCGGGATTACAATTGGTTCTGGATATTTTTGGTCGGCCCGCATGTTGGAGCCATTTTG
GGCGTTTGCATTTTT---CACTTGATTCTTAAAAAAGGTTCAGCAGAAACCAGC---GAG
---TTGGCTTCA---CCTACGACAAGCTCATCTATGGATTCACGCCAATGGCAGCATTTT
ACCACG------------------------------------------------------
------------------------------------------------------------
------------------------------------------------------------
------------------------------------------------------------
------------------------------------------------------------
------------------------------------------------------------
------------------------------------------------------------
------------------------------------------------------------
------------------------------------------------------------
------------------------------------------------------------
------------------------------------------------------------
------------------------------------------------------------
------------------------------------------------------------
------------------------------------------------------------
------------------------------------------------------------
------------------------------------------------------------
------------------------------------------------------------
------------------------------------------------------------
------------------------------------------------------------
------------------------------------------------------------
------------------------------------------------------------
------------------------------------------------------------
------
>JAAVJA010002980_Daphnia_dubai_GlpA6
------------------------------------------------------------
------------------------------------------------------------
---------------------------------------------ATGTGT---AAAAAG
TTTCAA---------GTTCCATCGTTGTTTAGAGCAGCATTCGCTGAATTTATTGGTACC
TACATCTTGGTTGTCATCGGGAACGGGAGCATAGCGCAGTCACAGTTAACCAATGGCGAA
```

AAAGGNGATTACTTCACGATTAACTGGGGATGGGCTTTAGGATGTTCGTTGGGAATTCTC
ATTTCTGCTAATATATCAGGAGGTCATTTGAATCCAGCAGTGACACTGGCGCTGGCACTA
GTTCGTCATTTCCCGTGGAAAAAGTTACCTGTTTATTGGTGTGCCCAATATCTCGGAGCG
ATGGCAGCTTCTGGAACCGTCCTTGGAGTCTATTACGAGGGAATACTGGAAGGAAAAGTC
GAA---------GGTGAATTTCGGATCAGGAAG------AACATTACCGAACCG------
GGAATGGCATCTATTTTTGCAAACTATCCAGCTCCATATTCATCAGCTAGTATTGGACTG
GTTGAGGAAATTTTATCGACTATGATCTTCATGCTGGTTATATGCGTTGTGACTGACAAA
AGGTATTCAAACGTN---------------------------------TACATAGGCTTC
GCCCTGTTGGCCATTGGTGTTGCATACGGA---------------------------GCT
CGTGATTTAGCTCCTAGGTTGGTCTCTTTTCTGGGTGGATGGGGACCAGGTGTATTTAGT
TTTCGGGATTACAATTGGTTCTGGATATTTTTGGTCGGTCCTCACGTTGGAGCCGTTCTG
GGCGTTTCCATTTTT---CATCTGGTTTTGAAAAAGGGCTCAGCAGATAGCAGC---GAG
---TTGGCTTCA---TCTGTGACAGCCTCTTCAATTGATTCACGTCAATGGCGACATTTC
ACCACATATCCGCCCAATGAA---------GATCATCAAGAGCATTAT---------AAA
GAA------------------------------AGCCACAATAACAGAGAT---TATAAC
ATGAACACACTA------------------------------------------------
------------------------------------------------------------
------------------------------------------------------------
------------------------------------------------------------
------------------------------------------------------------
------------------------------------------------------------
------------------------------------------------------------
------------------------------------------------------------
------------------------------------------------------------
------------------------------------------------------------
------------------------------------------------------------
------------------------------------------------------------
------------------------------------------------------------
------------------------------------------------------------
------------------------------------------------------------
------------------------------------------------------------
------------------------------------------------------------
------------------------------------------------------------
------------------------------------------------------------
------------------------------------------------------------
------
>FLTH02000034_Daphnia_pulex_GlpA6
------------------------------------------------------------
------------------------------------------------------------
---------------------------------------------ATGTGT---AAAAGA
TTCCGT---------GTCCCGTCTTTGTTTCGAGCAGCATTCGCTGAATTCATTGGCACC
TACATCTTGGTTGTCATCGGGAACGGGAGCATAGCGCAATCACAATTAACCAATGGCGAA
AAAGGTGATTACTTCACCATTAACTGGGGATGGGCTTTAGGTTGTTCGTTGGGAATTCTT
ATTTCATCCAATATATCAGGGGGCCATTTGAATCCAGCCGTGACACTGGCCCTTGCACTA
GTTCGTCATTTCCCGTGGAAAAAGTTGCCGGTGTATTGGTGTGCCCAGTATCTAGGAGCG
ATGGCCGCATCTGGAACAGTCCTTGGAGTCTATCATGAGGCAATCATGGAAGGAAAAGTC
GAC---------GGGGAATTTCGGATCAGAAAG------AACATTACCGAACCC------
GGCATGGCTTCCATTTTCGCCAATTACCCAGCCCCATATTCATCGGCTAGCATTGGACTG

```
GTCGAAGAGATTTTATCGACCATGATCTTCATGCTCGTGATATGTGTGGTGACGGACAAA
AGGTATTCAAGCGTGCCG------------AATTTCTTGCAGCCACTCTACATCGGCTTC
ACCTTGTTGGCCATCGGGGTCGCGTACGGATCGAATGGAGGTTACGCTCTCAACCCGGCT
CGTGATTTGGCTCCCCGTTTGGTCACCTTTCTTGGTGGATGGGGGCTAGGTGTATTTAGT
TTTCGGGATTACAATTGGTTCTGGATATTTTTGGTCGGTCCGCACGTTGGAGCCATTCTG
GGCGTTTGCATATTT---CACTTCATTTTGAAAAAAGGCCAAGCAGATCCCGCT---GAA
---ATGGTTTCA---TCTATAATAACCTCGTCAATTGATTCGCGCCATTGGCAGCACTTC
AACACGCATCTGCCAAATGCA---------GAGCAACAAGCGCACTATCCATTCCCAGAA
GAA------------------------------AACCACCAAAGCAGAGACTCCTATAAC
ACGAAAACACTG------------------------------------------------
------------------------------------------------------------
------------------------------------------------------------
------------------------------------------------------------
------------------------------------------------------------
------------------------------------------------------------
------------------------------------------------------------
------------------------------------------------------------
------------------------------------------------------------
------------------------------------------------------------
------------------------------------------------------------
------------------------------------------------------------
------------------------------------------------------------
------------------------------------------------------------
------------------------------------------------------------
------------------------------------------------------------
------------------------------------------------------------
------------------------------------------------------------
------------------------------------------------------------
------------------------------------------------------------
------
>XM_046779320_Daphnia_pulicaria_GlpA6
------------------------------------------------------------
------------------------------------------------------------
---------------------------------------------ATGTGT---AAAAGA
TTCCGT---------GTCCCGTCTTTGTTTCGAGCAGCATTCGCTGAATTCATTGGCACC
TACATCTTGGTTGTCATCGGGAACGGGAGCATAGCGCAATCGCAGTTAACCAATGGCGAA
AAAGGTGATTACTTCACCATTAACTGGGGATGGGCTTTAGGGTGTTCGCTGGGAATTCTT
ATTTCATCCAATATATCAGGGGGCCATTTGAATCCAGCCGTGACACTGGCACTTGCACTA
GTTCGTAATTTCCCGTGGAAAAAGTTGCCGGTGTATTGGTGTGCCCAGTATCTAGGAGCT
ATGGCCGCTTCTGGAACCGTCCTGGGAGTCTATCACGAGGCAATCTTGGAAGGAAAAGTC
GAC---------GGGGAATTTCGGATCAGAAAG------AACATTACCGAACCC------
GGTATGGCTTCTATTTTCGCCAATTACCCAGCCCCATATTCATCGGCTAGCATTGGACTG
GTCGAAGAGATATTATCGACCATGATCTTCATGCTCGTGATATGTGTGGTGACAGACAAA
AGGTATTCAAACGTGCCG------------AATTTCTTGCAGCCACTCTACATCGGCTTC
ACCTTGTTGGCCATCGGGGTCGCGTACGGATCGAATGGAGGTTACGCTCTCAACCCGGCT
CGTGATTTGGCTCCCCGTTTAGTCACCTTTCTTGGTGGGTGGGGGCTAGGTGTATTTAGT
TTTCGGGATTACAATTGGTTCTGGATATTTTTGGTCGGTCCGCACGTTGGAGCCATTCTG
GGCGTTTGCATATTT---CACTTCATTTTGAAAAAAGGCCAAGCAGATCCCGCT---GAA
```

---ATGGTTTCA---TGCATAACAACTTCGTCAATTGATTCGCGCCACTGGCAGCACTTC
AACGCGCATCCGCCAGATGCA---------GAGCAACAAGCGCACTATCAATTCCCAGAA
GAA------------------------------AGCCACAAAAGCAGAGACTCTTATAAC
ACGAAAACACTA------------------------------------------------
------------------------------------------------------------
------------------------------------------------------------
------------------------------------------------------------
------------------------------------------------------------
------------------------------------------------------------
------------------------------------------------------------
------------------------------------------------------------
------------------------------------------------------------
------------------------------------------------------------
------------------------------------------------------------
------------------------------------------------------------
------------------------------------------------------------
------------------------------------------------------------
------------------------------------------------------------
------------------------------------------------------------
------------------------------------------------------------
------------------------------------------------------------
------------------------------------------------------------
------------------------------------------------------------
------
>CM023188_Daphnia_carinata_GlpA6
------------------------------------------------------------
------------------------------------------------------------
---------------------------------------------ATGTGT---AAGAGA
TTCCAC---------GTTCCTTCTGTATTTCGAGCAGCATTCGCCGAATTTTTAGGCACC
TATATTTTGGTAGTCATTGGGAACGGAAGTATAGCTCAGTCGCAGTTGACCAACGGCGAA
AAAGGGGACTATTTTACTATCAACTGGGGATGGGCTTTGGGTTGTTCTTTAGGAATGCTC
GTGTCAGCCAATGTATCAGGAGGTCATCTGAATCCCGCGGTGACAATGGCGCTAGCATTA
GTTCGTCGTTTTCCATGGAAAAGGCTGCCAGTGTATTGGTGTGCCCAATACATGGGAGCT
CTTGCCGCTTCTGGAACGGTTCTCGGAGTATATCACGAAGGCATTTTGGAAGGAAAAGTC
AAT---------GGCAAATTCCGCATTGAGAAG------AACATTACAGAACCT------
GGTATGGCCTCCATTTTTGCTAACTATCCAGCACATTACTCGTCGGCTAGTATTGGTTTA
ATCGAAGAAATTTTATCGACCATGATCTTCACGCTCGTCATATGCGTGGTAACCGACAAA
CGGTATTCAAACGTGCCA------------AACTTCTTGCAGCCACTCTATGTCGGATTC
ACGTTGTTGGCCATCGGTGTCGCCTACGGATCCAATGGAGGTTACGCCCTCAATCCGGCG
CGGGATTTGGCTCCCCGGTTGGTTTCCTTCCTTGGTGGATGGGGACTAGGTGTATTCAGT
TTTCGAGACTACAACTGGTTCTGGATATTCTTGGTCGGCCCGCATGTTGGAGCCATTCTT
GGTGTCTGTATCTTC---CACCTGATCTTGAAGAATGGCTCGAAAGATATCAGT---GAG
---CGGGTTTCA---TCCATAGACGTTTCCATGATCAATCCACGTCTATGGCACCACTTT
CCCAACAGAGGACTTGACGTTGAACCATCAGATGGACGGGAATCTTACAACAGTCACGAA
GAG------------------------------AATCATAAAAGCACAGACTACAATATT
GCGAGGACACTC------------------------------------------------
------------------------------------------------------------
------------------------------------------------------------

------------------------------------------------------------
------------------------------------------------------------
------------------------------------------------------------
------------------------------------------------------------
------------------------------------------------------------
------------------------------------------------------------
------------------------------------------------------------
------------------------------------------------------------
------------------------------------------------------------
------------------------------------------------------------
------------------------------------------------------------
------------------------------------------------------------
------------------------------------------------------------
------------------------------------------------------------
------------------------------------------------------------
------------------------------------------------------------
------------------------------------------------------------
------
>WJBH02000001_Daphnia_sinensis_GlpA6
------------------------------------------------------------
------------------------------------------------------------
---------------------------------------------ATGTGT---AAGAGA
TTCCAC---------GTTCCTTCTGTATTTCGAGCAGCATTCGCCGAATTTTTAGGCACC
TATATTTTGGTAGTAATTGGGAACGGAAGTATAGCTCAGTCGCAGTTGACCAACGGCGAA
AAAGGGGACTATTTTACTATCAACTGGGGATGGGCTTTGGGTTGTTCTTTAGGAATGCTC
GTGTCAGCCAATGTATCAGGAGGTCATCTGAATCCCGCGGTGACAATGGCGCTAGCATTA
GTTCGTCGTTTTCCATGGAAAAGGCTGCCAGTGTATTGGTGTGCCCAATACATGGGAGCT
CTTGCCGCTTCTGGAACGGTTCTCGGAGTATATCACGAAGGCATTTTGGAAGGAAAAGTC
AAT---------GGCAAATTCCGCATTGAGAAG------AACATTACAGAACCT------
GGTATGGCCTCCATTTTTGCTAACTATCCAGCACATTACTCGTCGGCTAGTATTGGTTTA
ATCGAAGAAATTTTATCGACCATGATCTTCACGCTCGTCATATGCGTGGTAACCGACAAA
CGGTATTCAAACGTGCCA------------AACTTCTTGCAGCCACTCTATGTCGGATTC
ACGTTGTTGGCCATCGGTGTCGCCTACGGATCCAATGGAGGTTACGCCCTCAATCCGGCG
CGGGATTTGGCTCCCCGGTTGGTTTCCTTCCTTGGTGGATGGGGACTAGGTGTATTCAGT
TTTCGAGACTACAACTGGTTCTGGATATTCTTGGTCGGCCCGCATGTTGGAGCCATTCTT
GGTGTCTGTATCTTC---CACCTGATCTTGAAGAATGGCTCGAAAGATATCAGT---GAG
---CGGGTTTCA---TCCATAGACGTTTCCATGATCAATCCACGTCTATGGCACCACTTT
CCCAACAGAGGACTTGACGTTGAACCATCAGATGGACGGGAATCTTACAACAGTCACGAA
GAG------------------------------AATCATAAAAGCACAGACTACAATATT
GCGAGGACACTC------------------------------------------------
------------------------------------------------------------
------------------------------------------------------------
------------------------------------------------------------
------------------------------------------------------------
------------------------------------------------------------
------------------------------------------------------------
------------------------------------------------------------
------------------------------------------------------------

------------------------------------------------------------
------------------------------------------------------------
------------------------------------------------------------
------------------------------------------------------------
------------------------------------------------------------
------------------------------------------------------------
------------------------------------------------------------
------------------------------------------------------------
------------------------------------------------------------
------------------------------------------------------------
------------------------------------------------------------
------
>NC_046175_Daphnia_magna_GlpA6
------------------------------------------------------------
------------------------------------------------------------
---------------------------------------------ATGTGC---AAGCGG
TTCCAT---------GTTCCTTCTGTATTTCGAGCAGCATTCGCAGAATTTTTAGGCACC
TATATTTTGGTAGTCATTGGGAACGGGAGTATAGCTCAGTCGCAGTTAACCAAAGGTGAA
AAGGGTGACTATTTTACTATCAACTGGGGATGGGCTTTGGGTTGTTCTTTAGGGATGCTG
GTGTCAGCCAATATATCAGGAGCTCATCTGAATCCCGCGGTGACAATGGCGCTATCATTA
GTTCGTCGTTTTCCTTGGAAAAGGCTGCCAGTGTATTGGTGTGCCCAATACATGGGAGCT
CTTGCCGCTTCTGGAACAGTTCTCGGAATATATCACGAAGGAATTTTGGAAGGAAAAGTC
AAC---------GGCAAATTCCGGATTGAGAAG------AACATTACAGAACCT------
GGGATGGCCTCCATTTTTGCCAACTATCCAGCCCATTACTCGTCAGCTAGTATTGGTTTG
ATCGAAGAAATCTTATCGACCATGATTTTCACGCTCGTCATATGCGTGGTAACCGACAAA
CGATATTCAAACGTGCCA------------AACTTCTTGCAGCCACTCTACGTTGGTTTC
ACGTTGTTGGCCATCGGTGTCGCCTACGGATCCAATGGAGGTTACGCCCTCAATCCGGCG
CGGGATTTGGCTCCCCGGTTGGTTTCCTTCCTTGGCGGATGGGGACTAGGTGTATTCAGT
TTTAGAGACTACAACTGGTTCTGGATATTCTTGGTCGGCCCGCATGTTGGAGCCATTCTT
GGTGTATGCATCTTT---CACCTGATTTTGAAGAATGGCTCAACAGATATCAGT---GAG
---CGGGTTTCA---TCCATAGACGTTTCGATGATCAATCCACGTCTATGGCACCACTTT
CCCAACAGAGGACTTGAAGTTGAACCATCAGAAGAACGGGAATCTCACAACAGTCACGAA
GAG------------------------------AATCATAAAAGCACAGACTACAATATT
GGGATGACACTC------------------------------------------------
------------------------------------------------------------
------------------------------------------------------------
------------------------------------------------------------
------------------------------------------------------------
------------------------------------------------------------
------------------------------------------------------------
------------------------------------------------------------
------------------------------------------------------------
------------------------------------------------------------
------------------------------------------------------------
------------------------------------------------------------
------------------------------------------------------------
------------------------------------------------------------
------------------------------------------------------------

------------------------------------------------------------
------------------------------------------------------------
------------------------------------------------------------
------------------------------------------------------------
------------------------------------------------------------
------
>JAKUCG010000092_Ceriodaphnia_dubia_GlpA6
------------------------------------------------------------
------------------------------------------------------------
---------------------------------------------ATGTTCTTTCCCCGT
TTCCGG---------TTGCCAACTGTGGTTCGAGCTGCTTTCGCCGAATTCATTGGCACC
TACGTCCTCGTTGTGGTTGGTAATGGAAGCATAGCCCAGTCTCAATTGACCAATGGTGAA
AAGGGTGATTATTTCACCATTAACTGGGGCTGGGCGTTGGGTTGTTCGTTGGGAATCCTC
GTCTCGGCCAATATATCAGGTGGTCATTTGAACCCGGCCGTGACGTTGGCGCTGGCGCTC
GTCCGTGATTTCCCTTGGAGAAGACTTCCTGCCTATTGGTGCGCTCAATATTTAGGGGCC
TTGGCCGCTTCTGGGACAGTCCTCGGAGTCTATCACGAAGGAATTTTAGAAGGGAAAGTC
AAC---------GGCGAGTTCCGCATTCACCAG------AATGCAGAAGATCCG------
GGATCGGCCGCTATTTTCGCCAATTATCCAGCTCCGTATTCGTCAGCCAGTATTGGCTTA
GTAGAAGAGATTTTGTCGACGATGATCTTTATGCTCGTCATTTGTGTGACTACCGACAAA
AGATATTCCAAAGTGCCC------------GGACACTTGCAGCCGCTTTACATTGGCTTC
ACTTTATTGGCCATCGGAGTGGCTTACGGCTCCAATGGCGGCTACGCTCTCAATCCCGCT
CGCGATTTGGCCCCTCGACTGGTTTCATTTTTTGGTGGATGGGGAACAGGCGTTTTCAGG
TTCCGAGACTACAATTGGTTTTGGATTTTCCTGGTGGGACCTCACGTCGGAGCAATTCTA
GGTGTCTGCATTTTC---CACTTTTTATTAAAGAACGCCAGCCAAGATGAAATC---GAC
---------AGT---GGCGAGCATCATCACGATTCTCCCCTTGTCCTTGTCTCGACAATC
GAAACTCGGCCGTGG---ATGCAAGACGATCGTCCTTGTGACGTGGATCATAATAAA---
---------------------------------ACTAAAAGCGAGTACAACAGTTACGCC
TCGATGACTCTT------------------------------------------------
------------------------------------------------------------
------------------------------------------------------------
------------------------------------------------------------
------------------------------------------------------------
------------------------------------------------------------
------------------------------------------------------------
------------------------------------------------------------
------------------------------------------------------------
------------------------------------------------------------
------------------------------------------------------------
------------------------------------------------------------
------------------------------------------------------------
------------------------------------------------------------
------------------------------------------------------------
------------------------------------------------------------
------------------------------------------------------------
------------------------------------------------------------
------------------------------------------------------------
------------------------------------------------------------
------

>FLTH02000034_Daphnia_pulex_GlpA7
------------------------------------------------------------
------------------------------------------------------------
---------------------------------------------ATGCGT---CGGTGC
CTTTGCAGC------GTCCCCCAACTAGTTCGTGCAGCCTTCGCCGAATTTATTGGCACC
TTCATCTTCATTGTCATCGGGAACGGGAGCATTGCTCAGTTCCATTTGAGCAAAGGCAGA
AAATCCGAGTACCTCACTGTTAACTGGGGATGGTCTTTAGGATGTTGTCTTGGAACTCTA
ATTTCGGTTAAAATATCAGGCGGCCATTTGAACCCGGCGGTGACGATGGCTCTCACACTT
GCCCGCGATTTCCCGTGGAAAAGGCTGCCCGTCTATTGGATCGCCCAATATCTCGGGGCT
CTGGCCGCATCTGGAACTACCCTCGGTATCTATTACGAATCGCTCGTTCAAAGTAAACTC
ATC---------GGCGAGTTCCGTCCATGCAAG------AACACAACG------------
------------------------------------------------------------
---------ATTTTATCCACGATGATTTTCATGTTAGTCATTTGCGTGACAACGAATGCA
AATTATTCGAATGTGCCG------------TCATTCCTGCAGCCGGTCTACATTGGTTTC
GCTTTAATGGCGTGTGGCATATCTTACGGCTCCAATGGCGGTTACGCTCTCAACCCGGCA
CGCGATTTATCTCTTCGTCTTGTTTTTATCTCAGTGGTTGGGAGGCAAGCGCTTTCAGT
TTCCGGGGCTACAATTGGTTCTGGATCTTTCTGGTGGGACCGCACGTCGGAGCACTCTTA
GGAGTCGGAGTCTTT---CACATCTTTCTGAAG---------ACTGAGCTTACG---GAC
------TGGGCA---CCTGAATACTTT---TCCAGCCTGAAACGCTCAGCAAATAAG---
CCAGTATTAGTTGCTGAGCGATCTGAC---GAATTGGGTTTGAGATCGCAGTGGCGTCTG
CAGCAA------------------------------CGCTCCAACGTCAACCTTGTAGAC
TCGATGGTCTCCACAAAGTTTATCGAGCTGGAAAGTCGCAGCCCAGGTGCCAGAAGATCA
TCAAGGGCATCTCAAATCAGCACGAAATGGATA---------------------------
------------------------------------------------------------
------------------------------------------------------------
------------------------------------------------------------
------------------------------------------------------------
------------------------------------------------------------
------------------------------------------------------------
------------------------------------------------------------
------------------------------------------------------------
------------------------------------------------------------
------------------------------------------------------------
------------------------------------------------------------
------------------------------------------------------------
------------------------------------------------------------
------------------------------------------------------------
------------------------------------------------------------
------------------------------------------------------------
------------------------------------------------------------
------------------------------------------------------------
------
>XM_046779298_Daphnia_pulicaria_GlpA7
------------------------------------------------------------
------------------------------------------------------------
---------------------------------------------ATGCAC---CGGTGC
CTTTGCAGC------GTCCCACAACTAGTTCGTGCAGCCTTCGCCGAATTTATTGGCACC
TTCATCTTCGTTGTCATCGGGAACGGGAGCATTGCTCAGTTCCATTTGAGCAAAGGCAGA

AAATCCGAGTACCTCACTGTTAACTGGGGATGGGCTTTAGGATGTTGTCTTGGAACTCTA
ATTTCGGTTAAAATATCAGGCGGCCATTTGAACCCGGCAGTGACGATGGCTCTCACACTC
GCCCGCGATTTCCCGTGGAAAAGGCTGCCCGTCTATTGGATCGCCCAATATCTCGGGGCT
CTGGCCGCATCTGGAACTGCCCTCGGTATCTATTACGAATCGCTCGTTCAAAGTAAACTT
ATC---------GGCGAGTTCCGTCCATGCAAG------AACACGACGGAAACG------
---GCGGCCGTATTATTAGCCAACCATTCCTCCCACGAG---TCAGTTTATACTGGATTA
GTAGAACAGATTTTATCCACGATGATTTTCATGTTAGTCATTTGCGTGACAACGAATGCA
AATTATTCGAATGTGCCG------------TCATTCTTGCAGCCGGTCTACATTGGTTTC
GCTTTAATGGCGTGTGGCATATCTTACGGCTCCAATGGCGGTTACGCTCTCAACCCGGCA
CGCGATTTATCTCTTCGTCTTGTTTTTTATTTCAGTGGTTGGGAGGCAAACGCTTTCAGT
TTCCGGGGCTACAATTGGTTCTGGATCTTTCTGGTGGGACCGCACGTCGGAGCACTCTTA
GGCGTGGGAGTTTTT---CACATCTTTTTGAAG---------ACTGAGCTCACG---GAC
------TGGGCA---ACTGAATACTACGTTTCCAGTCTGAAACGCTCAGCAAATAAG---
CCAGTATTAGTTGCCGAGCGATCTGAC---GAATTAGGTTTGAGATCGCAGTGGCGTCTG
CAA---------------------------------CGCTCCAACGTCAACCTTGTAGAC
TCGATGGTCTCCACAAAGTTTATCGAGCTGGAAAGTCGCAGCCCAGGTGTCAGAAGATCA
TCAAGGTCATCTCAAATCAGCACGAAATGGATA---------------------------
------------------------------------------------------------
------------------------------------------------------------
------------------------------------------------------------
------------------------------------------------------------
------------------------------------------------------------
------------------------------------------------------------
------------------------------------------------------------
------------------------------------------------------------
------------------------------------------------------------
------------------------------------------------------------
------------------------------------------------------------
------------------------------------------------------------
------------------------------------------------------------
------------------------------------------------------------
------------------------------------------------------------
------------------------------------------------------------
------------------------------------------------------------
------------------------------------------------------------
------
>GGXW01360757_Daphnia_galeata_GlpA7m
------------------------------------------------------------
------------------------------------------------------------
---------------------------------------------ATGCGT---CGGTGT
TTTTGCAGC------GTCCCACAACTAGTTCGTGCAGCGTTTGCCGAATTTTTGGGCACT
TTCGTCTTTGTTGTCATCGGAAACGGGAGCATTGCCCAGTCCCATTTGAACAAAGGCAGA
AAATTGGATTATTTCACTATTAACTGGGCATGGGCTCTTGGATGTTGTCTTGGAACGTTA
ATTTCGGTTAAAGTATCGGGAGGACACTTAAATCCGGCGGTGACGATGGCTCTCTCTTTC
GTACGCGATTTTCCTTGGAAAAGGTTAGCGGTGTATTGGTTCGCGCAGTATCTAGGTGCT
CTGGCCGCGACTGGAACAGTCGTCGGCATCTATTACGAAGCGCTCGTTCGAGGCAAACTC
AATACT------GGTGAGTTCCAT---CACAAG------AACACCACG------------
------------------------------------------------------------

------GAAATTTTATCCTCAATGATTTTTCTAGTAGTCATTTGCGTGACAACGAATGCA
AAGTATTCAAATGTGCCG------------TCTTTCTTGCAGCCGATCTACATCGGTTTC
ACTTTACTGGCATGTGGCATATCTTACGGCTCCAATGGCGGTTACGCTTTAAACCCGGCA
CGCGATTTATCTCTTCGTCTTGTTTTTTATCTCGGTGGATGGGAGGCAGCCGCGTTCAGT
TTTCGGGGCTACAATTGGTTCTGGATTTTTTTGGTGGGACCTCACATTGGAGCACTCCTG
GGAGTATTCATTTTT---CACATATTTCTGAAG---ACTGAGACTGAACTGAAAGCAGAC
------TGGGCA---GCTGAATACGTTTCCAGGCTTAAACTTCCAACAGCAGCAGCAGCA
GCAACAACAATGGCCCAGCATTTTGAATTGGAAACGAATTTGGGGTCACAGTGGCATCTG
CAACAC------------------------------------------------------
------------------------------------------------------------
------------------------------------------------------------
------------------------------------------------------------
------------------------------------------------------------
------------------------------------------------------------
------------------------------------------------------------
------------------------------------------------------------
------------------------------------------------------------
------------------------------------------------------------
------------------------------------------------------------
------------------------------------------------------------
------------------------------------------------------------
------------------------------------------------------------
------------------------------------------------------------
------------------------------------------------------------
------------------------------------------------------------
------------------------------------------------------------
------------------------------------------------------------
------------------------------------------------------------
------------------------------------------------------------
------
>JAAVJA010002980_Daphnia_dubai_GlpA7
------------------------------------------------------------
------------------------------------------------------------
---------------------------------------------ATGCGT---TGGTGT
CTCTGCAGC------GTCCCACAAATAGTTCGTGCAGCA---GCCGAATTTATTGGCACC
TTCGTCTTCGTCGTCATCGGAAATGGAAGCATTGCTCAGTCCCATTTGAGCAAAGGGAAA
AAATTNGATTACTTCACGATTAACTGGGGTTGGGCTTTAGGATGTTGTCTTGGAACCCTA
ATTTCAGCTAAAATATCAGGCGGCCACTTGAACCCGGCGGTGACGATGGCTCTCTCTCTC
GTCCGCGATTTTCCTTGGAAAAGGTTGCCGGTTTATTGGTTC------------------
---------TCA------------------------GAAGCGCTAGTTCAAGGCAAACTT
AAC---------GGTGAGTTC---------------------------------------
------------------------------------------------------------
---------ATTTTATCTACGATGATTTTCATGATAGTCATTTGCGTGACCACGAATGCA
AAATATTCGAATGTTCCG------------TCTTTCTTGCAGCCGATCTACATTGGTTTC
ACCTTACTGGCCTGTGGCATATCTTACGGCNNNNNNNGAGGTTACGCTCTCAACCCGGCA
CGCGATTTATCTCTTCGTCTTGTTTTTTATCTCGCTGGATGGGGCGAAGGCGCCTTC---
------------------------------------GGACCGCACATTGGAGCTTTTTTG
GGAGTATATATCTTT---CACATCTTTTTGAGG---ACTGAG---CTGAATACG---GAT

```
------TGGACA---ACTGAATACGTTTCCAGACTGAAA---CCTTCACCGGCAGCA---
CCA---ACAGTCGCCCAGTTTTCTGAATCTGAACAACTTCTTAGACTCCAT---GATCTC
CACAAA------------------------------------------------------
------------------------------GACGATCAGTCTGAGAAATCGCCCCGATGG
GAGAAGAACGTCAAGAGCATCTCAAATTACCAC---------------------------
------------------------------------------------------------
------------------------------------------------------------
------------------------------------------------------------
------------------------------------------------------------
------------------------------------------------------------
------------------------------------------------------------
------------------------------------------------------------
------------------------------------------------------------
------------------------------------------------------------
------------------------------------------------------------
------------------------------------------------------------
------------------------------------------------------------
------------------------------------------------------------
------------------------------------------------------------
------------------------------------------------------------
------------------------------------------------------------
------------------------------------------------------------
------------------------------------------------------------
------
>JAKUCG010000092_Ceriodaphnia_dubia_GlpA7
------------------------------------------------------------
------------------------------------------------------------
---------------------------------------------ATGAGG---TTGTGC
TGCAAA---------TTTCCGCAAATTATTCGAGCTGCCTTGGCTGAATTCATCGGCACC
TACGTCCTTGTTGTGATTGGAAACGGAAGCATAGCCCAATCGCACCTGTCCAAAGGTGAA
AAGGGCGATTACTTTACAATCAATTGGGGCTGGGCGTTGGGGTGTTCGCTGGGCATTCTC
ATTTCCGCCAACGCATCAGGCGGCCACTTGAACCCGGCAGTGACAATGGCTTTAGCCTTT
GCGCGGGATTTCCCTTGGAAAAAATTGCCCATCTATTGGCTGGCTCAATACCTGGGCTCT
TTAGCTGCTTCCGCGACGGTCCTCGGCATTTATTACGATAAAATATTACAAGGGAAAATC
AAA---------GGTGAATTCCGCATCCGCACA------AATGAATCCGAACCT------
GGAATGGCCGTCATTTTTTCCAATTATCCGTCTCATGAGACGTCGGTCATCACGGGATTA
GTAGAAGAAATTTTATCGACGGCGATTTTTATGCTCGTCATTTGCGTGACGACGGACAAG
AAATTTTCCAAAGTCCCC------------AATCACTTGCAGCCGTTTTACATTGGCTTC
ACTCTGTTGGCCATGGGTGTGTCTTACGGCTCTATTGGAGGCTACGCTCTTAATCCGGCA
CGCGATTTGGCGCCGCGACTTGTTTCTTATCTCGGCGGATGGGGACTCGGCGTATTCAGG
TTCCGTGAGTACAACTGGTTCTGGATTTTCCTTGTCGGTCCTCACGTCGGCGCTATTTTG
GGCGTCGGAGTCTTT---CATCTGGTCCTTCTAAAGACGGAC---GAAACATCT---GAC
---AGATCTAAT---AACGAGTGGGGACTAGTCGTCCCACCCGTCGCGATGAGTTACGTA
CCT------GAATTG------AACAGAAAGGCCCTCGGATTGGATTTTAGACTGCCGGCA
ACA---------------------------------AGAACGAAG------------GAC
GAGACTCGTTCCTGGAAA---TCAAGACCCCGAAAAAGGACTCTGGCAGCTCCAGCAGTA
TCGTTTAGCTGTAGCATTTGCGCCGTAGTCACG---------------------------
------------------------------------------------------------
```

------------------------------------------------------------
------------------------------------------------------------
------------------------------------------------------------
------------------------------------------------------------
------------------------------------------------------------
------------------------------------------------------------
------------------------------------------------------------
------------------------------------------------------------
------------------------------------------------------------
------------------------------------------------------------
------------------------------------------------------------
------------------------------------------------------------
------------------------------------------------------------
------------------------------------------------------------
------------------------------------------------------------
------------------------------------------------------------
------------------------------------------------------------
------
>HAFN01012924_Daphnia_galeata_GlpA1
------------------------------------------------------------
------------------------------------------------------------
---------------------------------------------ATGCGT---TCGAGG
TTTTCT---------ATTCCACTTATAGTGCGCATAGCTTTCGCCGAGTTCGTCGGTACT
TACGTTTTGGTTGTCATTGGCAACGGAAGTGTAGCGCAGGCCCAACTGACTCATGGTCAG
AAAGGTGACTATTTCGCAATCAATTGGGGTTGGGCACTCGGTTGTTTTCTTGGCGTGTTG
ATTTCGGCTAACGTATCAGGCGGCCACTTAAATCCAGCAGTCACCCTGGCCCTGGCCATC
ACTCGCAGGTTCGAATGGAAAAAACTCCCCGTTTACTGGTTTGCGCAATACCTGGGAGCT
CTGACTGCTTCTGGAACGGTTCTAGGGGTTTATTATGAGGCAATTATGCAAGGCAAAGTA
AAT---------GGCGAATTCCGTATTCATAAG------GATTCTTCTGATCCA------
GGTTCGGCTTCAATTTTTGCAAATTATCCAGCTCCTTACTCGTCGGCTGGCCTTTGTTTG
GTCGAAGAGATATTAGCCACGATGGTATTTATGTTGGTCATATGCGTTACAACGGATAAA
AGATATTCGAAAATACCT------------CCGTTCTTGCAGCCCTTATTTATTGGCTTT
GCCTTACTGGCTCTTGGCATTTCCTATGGAGCTAACAGTGGCTACGCTTTGAATCCGGCT
CGCGATCTTGGTCCTCGCATAACATCTTACTTTGGAGGATGGGGAACTGGAGTTTTCAGC
TTTAGGGACTATAACTGGTTTTGGATCTTTATTTTGGGGCCACACATCGGTGCCATTTTG
GGAGTTTGTATCTTC---CAACTTCTCTTGAAGCTGCATCAATCCAATGATCTA---GAT
---CTCATTACA---AAGGCTGAACATGATCAGCAACTGCAACGGAAGGAACAAGAGCAG
CATAACATTGAACTT---CACAACATCAAGGCCATACATGACGATCATCATGTTCCA---
---------------------------------GCGATAGTCGAATTACCCAGAATAGGC
CTACGCTGG---------------------------------------------------
------------------------------------------------------------
------------------------------------------------------------
------------------------------------------------------------
------------------------------------------------------------
------------------------------------------------------------
------------------------------------------------------------
------------------------------------------------------------
------------------------------------------------------------

------------------------------------------------------------
------------------------------------------------------------
------------------------------------------------------------
------------------------------------------------------------
------------------------------------------------------------
------------------------------------------------------------
------------------------------------------------------------
------------------------------------------------------------
------------------------------------------------------------
------------------------------------------------------------
------------------------------------------------------------
------
>JAAVJA010001231_010009739_Daphnia_dubai_GlpA1
------------------------------------------------------------
------------------------------------------------------------
---------------------------------------------ATGCGT---CCGAGG
TTTTCT---------ATTCCGCTTATGGTACGCACAGCTTTCGCTGAGTTCGTCGGCACA
TATGTTTTGGTTGTTATTGGCAACGGAAGTGTAGCGCAAGCCCAATTGACTCACGGTCAG
AAAGGTGACTAT------------------------------------------------
------------------GGTGGCCACTTAAATCCAGCAGTTACCCTGGCCCTGGCCATA
ACCCGTAGGTTCGAATGGAAAAAACTACCCGTTTACTGGCTCGCGCAATACCTAGGAGCT
CTGGCGGCGTCTGGAACGGTTTTAGGGGTTTATTACGAGGCAATCATG---GGCAAAGTA
AAT---------GGCGAATTCCGTATTCATAAG------GATTCTTCTGACCCA------
GGATCGGCATCAATTTTTGCAAATTATCCGGCTCCTTACTCGTCGGCTGGTCTCTGTTTG
GTCGAAGAGATTTTGGCTACCATGATTTTCATGTTAGTCATTTGCGCAACAACGGATAAA
AGATATTCGAAAATACCT------------GTTTTCTTGCAGCCCTTATTTATTGGATTT
GCTTTACTGGCTATTGGAATTTCCTATGGAGCTAACAGCGGCTACGCTTTGAATCCAGCT
CGCGATCTTGGTCCTCGTATAACATCTTACTTTGGAGGATGGGGAACTGGAGTTTTTAGC
TTCAGGGACTATAACTGGTTTTGGATCTTTATTGTGGGGCCACATATCGGTGCCATTTTG
GGAGTTTGTGTCTTT---CAACTTCTCTTGAAGTTGCATAACTCCAATGATCTA---GAT
---CTT---ACA---AAGGCTGAACACGATCATCAAATGCAACGGAAGGAACAAGAGCAG
CATGACATTGAACTC---AACAACATCAAGGCCATATATGATGAACACCATGCCCCA---
---------------------------------GCGATAGTCGATTTACCCAGGATAGGC
CTACGCTGG---------------------------------------------------
------------------------------------------------------------
------------------------------------------------------------
------------------------------------------------------------
------------------------------------------------------------
------------------------------------------------------------
------------------------------------------------------------
------------------------------------------------------------
------------------------------------------------------------
------------------------------------------------------------
------------------------------------------------------------
------------------------------------------------------------
------------------------------------------------------------
------------------------------------------------------------
------------------------------------------------------------

------------------------------------------------------------
------------------------------------------------------------
------------------------------------------------------------
------------------------------------------------------------
------------------------------------------------------------
------
>EFX88619_Daphnia_pulex_GlpA1
------------------------------------------------------------
------------------------------------------------------------
---------------------------------------------ATGCGT---CCGAGG
TTTTCT---------ATTCCACTCATGGTGCGCACAGCTTTCGCTGAGTTCGTCGGAACA
TACGTTTTGGTTGTTATTGGCAACGGAAGTGTAGCGCAAGCCCAATTGACTCACGGCCAG
AAAGGTGACTATTTCGCGATCAATTGGGGTTGGGCTCTCGGCTGTGTTCTCGGCATTTTA
ATTTCGGCCAATGTATCAGGTGGCCACTTAAATCCAGCAGTTACACTGGCCCTGGCCATT
ACTCGTAGATTCGAATGGAAAAAACTACCCGTTTACTGGCTCGCGCAATATCTAGGAGCT
CTGACTGCGTCTGGAACGGTTTTAGGGGTTTATTACGAGGCAATCATGCAAGGGAAAGTG
AAT---------GGCGAATTCCGCATTCATAAA------GACTCGTCTGACCCA------
GGATCGGCTTCAATTTTTGCAAATTATCCGGCTCCTTACTCGTCGGCTGGTCTCTGTTTG
GTCGAAGAGATATTAGCTACCATGATTTTCATGCTGGTCATTTGCGTAACAACGGATAAA
AGATATTCGAAAATACCT------------GCCTTCTTGCAGCCCCTATTTATTGGTTTC
GCGTTACTTGCTATTGGAATTTCATATGGAGCAAACAGCGGCTACGCTTTGAATCCGGCT
CGAGATCTGGGTCCTCGCATAACATCGTACTTAGGTGGATGGGGGACGGGAGTTTTCAGC
TTCAGGGACTACAACTGGTTTTGGATCTTTATTGTGGGGCCACACATCGGTGCCATTTTG
GGAGTTTGCATCTTT---CAACTTCTCTTGAAGCTCCATCAATCCAATGCTCTA---GAT
---CTT---ACA---AAGGTTGAGCATGATCAGCAAATTCAACGTAAGGAACAGGAGCAG
CATGACATTGAACTG---AATAACATCAAGGCGATTCATGATGACCATCATGCCCAC---
---------------------------------GCGGTAGTCGAATTACCAAGGATAGGC
CTACGCTGG---------------------------------------------------
------------------------------------------------------------
------------------------------------------------------------
------------------------------------------------------------
------------------------------------------------------------
------------------------------------------------------------
------------------------------------------------------------
------------------------------------------------------------
------------------------------------------------------------
------------------------------------------------------------
------------------------------------------------------------
------------------------------------------------------------
------------------------------------------------------------
------------------------------------------------------------
------------------------------------------------------------
------------------------------------------------------------
------------------------------------------------------------
------------------------------------------------------------
------------------------------------------------------------
------------------------------------------------------------
------

```
>XM_046794230_Daphnia_pulicaria_GlpA1
------------------------------------------------------------
------------------------------------------------------------
---------------------------------------------ATGCGT---CCGAGG
TTTTCC---------ATTCCACTCATGGTGCGCACAGCTTTCGCTGAGTTCGTCGGAACA
TACGTTTTGGTTGTTATTGGCAACGGAAGTGTAGCGCAAGCCCAATTGACTCACGGCCAG
AAAGGTGACTATTTCGCGATCAATTGGGGTTGGGCTCTCGGCTGTGTTCTCGGCGTTTTA
ATTTCGGCCAATGTATCAGGTGGCCACTTAAATCCTGCAGTTACACTGGCCCTGGCCATT
ACTCGTAGATTCGAATGGAAAAAACTACCCGTTTACTGGCTCGCGCAATATCTAGGAGCT
CTGACTGCGTCTGGAACGGTTTTAGGGGTTTATTACGAGGCAATCATGCAAGGGAAAGTG
AAT---------GGCGAATTCCGCATTCATAAA------GACTCGTCTGACCCA------
GGATCGGCTTCAATTTTTGCAAATTATCCGGCTCCTTACTCGTCGGCTGGTCTCTGTTTG
GTCGAAGAGATATTAGCTACCATGATTTTCATGCTGGTCATTTGCGTAACAACGGATAAA
AGATATACGAAAATACCT------------GCCTTCTTGCAGCCCCTATTTATTGGTTTC
GCGTTACTGGCTATTGGAATTTCATATGGAGCAAACAGCGGCTACGCTTTGAATCCGGCT
CGAGATCTGGGTCCTCGCATAACATCGTACTTAGGTGGATGGGGAACGGGAGTTTTCAGC
TTCAGGGACTACAACTGGTTTTGGATCTTTATCGTGGGGCCACACATCGGTGCCATTTTG
GGAGTTTGCATCTTT---CAACTTCTCTTGAAGCTCCATCAATCCAATGCTCTA---GAT
---CTT---ACA---AAGGTTGAGCATGATCAGCAAATTCAACGTAAGGAACAGGAGCAG
CATGACATTGAACTG---AATAACATCAAGGCGATTCATGATGACCATCATGCCCAC---
---------------------------------GCGGTAGTCGAATTACCAAGGATAGGC
CTACGCTGG---------------------------------------------------
------------------------------------------------------------
------------------------------------------------------------
------------------------------------------------------------
------------------------------------------------------------
------------------------------------------------------------
------------------------------------------------------------
------------------------------------------------------------
------------------------------------------------------------
------------------------------------------------------------
------------------------------------------------------------
------------------------------------------------------------
------------------------------------------------------------
------------------------------------------------------------
------------------------------------------------------------
------------------------------------------------------------
------------------------------------------------------------
------------------------------------------------------------
------------------------------------------------------------
------------------------------------------------------------
------
>CM023188_Daphnia_carinata_GlpA1
------------------------------------------------------------
------------------------------------------------------------
---------------------------------------------ATGCGT---CCGAGG
TTTTGC---------ATTCCACTTATGGTACGCGCTGCTTTTGCAGAGTTTGTTGGGACC
TACGTTTTAGTTGTTATCGGCAACGGAAGTGTAGCGCAAGCTCAGCTTACTCATGGTCAG
```

AAAGGAGACTATTTCGCCGTCAACTGGGGCTGGGCTTTAGGCTGTATGTTAGGCATCCTG
GTATCGGCCAACGTATCAGGCGGCCACTTAAATCCGGCAGTAACGCTGGCCCTGGCAATC
ACACGTAAGTTTCAATGGAAGAAACTTCCCATCTACTGGTTAGCGCAATATCTAGGAGCT
CTGGCCGCATCTGGAACAGTCCTCGGCGTATATTACGAGGCAATAATGGAAGGCAGAGTG
AAT---------GGAGAATTTCGCATTCAGAAA------AATGCGGCAGACCCA------
GGTACGGCGGCAATTTTCGCTAATTATCCAGCTCCTTATTCGTCGTCGGGTCTCTGTTTG
GTTGAAGAGATTTTAGCTACGATGATTTTCATGTTAGTCATTTGTGCGACAACCGACAAA
CGATACTCTAAGGTTCCC------------ACTTTCCTGCAACCTTTCTATATCGGCTTC
GCCTTGTTGTCCATGGGATTGTCCTATGGGGCCAACAGTGGGTACGCTTTGAATCCGGCT
CGCGATTTGGGTCCTCGCATTACATCGTACTTTGGCGGATGGGGCTCTGGAGTCTTCAGT
TTCAGAGACTATAACGGGTTTTGGATTTTCATCGCCGGACCCCATATCGGTGCTATTTTA
GGAGTTTGCATCTTT---GAACTTCTTTTAAAGCTCCGCCCATCCGACAAAGCA---GAT
---CTA---ATG---AAGGTCGAGCATGAACAACAAATGCAGCGGAAGGAACAAGAACAA
CTCGAGATCGAATTA---AGTAATGTCAAGGCTATACGTGAGGAATACCATGCTTCA---
---------------------------------CCAATGGTCGAATTGCCAAGGATAGGC
CTACGTTGG---------------------------------------------------
------------------------------------------------------------
------------------------------------------------------------
------------------------------------------------------------
------------------------------------------------------------
------------------------------------------------------------
------------------------------------------------------------
------------------------------------------------------------
------------------------------------------------------------
------------------------------------------------------------
------------------------------------------------------------
------------------------------------------------------------
------------------------------------------------------------
------------------------------------------------------------
------------------------------------------------------------
------------------------------------------------------------
------------------------------------------------------------
------------------------------------------------------------
------------------------------------------------------------
------------------------------------------------------------
------
>KAI9564117_Daphnia_sinensis_GlpA1
------------------------------------------------------------
------------------------------------------------------------
---------------------------------------------ATGCGT---CCGAGG
TTTTGC---------ATTCCACTTATGGTACGCGCTGCTTTTGCAGAGTTTGTTGGGACC
TACGTTTTAGTTGTAATCGGCAACGGAAGTGTAGCGCAAGCTCAGCTTACTCATGGTCAG
AAAGGAGACTATTTCGCCGTCAACTGGGGCTGGGCTTTAGGCTGTATGTTAGGCATCCTG
GTATCGGCCAACGTATCAGGCGGCCACTTAAATCCGGCAGTAACGCTGGCCCTGGCAATC
ACACGTAAGTTTCAATGGAAGAAACTTCCCATCTACTGGTTAGCGCAATATCTAGGAGCT
CTGGCCGCATCTGGAACAGTCCTCGGCGTATATTACGAGGCAATAATGGAAGGCAGAGTG
AAT---------GGAGAATTTCGCATTCAGAAA------AATGCGGCAGACCCA------
GGAACGGCGGCAATTTTCGCTAATTATCCAGCTCCTTATTCGTCGTCGGGTCTCTGTTTG

GTTGAAGAGATTTTAGCTACGATGATTTTCATGTTAGTCATTTGTGCGACAACCGACAAA
CGATACTCTAAGGTTCCC------------ACTTTCCTGCAACCTTTCTATATCGGCTTC
GCCTTGTTGTCCATGGGATTGTCCTATGGGGCCAACAGTGGGTACGCTTTGAATCCGGCT
CGCGATTTGGGTCCTCGCATTACATCGTACTTTGGCGGATGGGGCTCTGGAGTCTTTAGT
TTCAGAGACTATAACGGGTTTTGGATTTTCATCGCCGGACCCCATATCGGTGCTATTTTA
GGAGTTTGCATCTTT---GAACTTCTTTTAAAGCTCCGCCCATCCGACAAAGCA---GAT
---CTA---ATG---AAGGTCGAGCATGAACAACAAATGCAGCGGAAGGAACAAGAACAA
CTCGAGATCGAATTA---AGTAATGTCAAGGCTATACGTGAGGAATACCATGCTTCA---
---------------------------------CCAATG---------------------
------------------------------------------------------------
------------------------------------------------------------
------------------------------------------------------------
------------------------------------------------------------
------------------------------------------------------------
------------------------------------------------------------
------------------------------------------------------------
------------------------------------------------------------
------------------------------------------------------------
------------------------------------------------------------
------------------------------------------------------------
------------------------------------------------------------
------------------------------------------------------------
------------------------------------------------------------
------------------------------------------------------------
------------------------------------------------------------
------------------------------------------------------------
------------------------------------------------------------
------------------------------------------------------------
------------------------------------------------------------
------
>NC_046175_Daphnia_magna_GlpA1
------------------------------------------------------------
------------------------------------------------------------
---------------------------------------------ATGCGT---CCGAGG
TTTTGC---------ATTCCACTTATGGTACGCGCTGCTTTGGCAGAGTTTGTTGGGACC
TACGTTTTAGTTGTTATTGGCAACGGAAGTGTAGCGCAAGCTCAGTTAACTCATGGCCAG
AAAGGAGACTATTTTGCCATCAATTGGGGCTGGGCTTTAGGCTGTATGTTAGGTATCCTG
GTATCGGCCAACGTATCAGGTGGTCACTTAAATCCGGCAGTAACGCTGGCCCTGGCCATC
ACTCGTAAGTTTCAATGGAAGAAACTTCCCATCTACTGGTTTGCGCAATATCTGGGAGCT
CTAGCTGCGTCTGGAACAGTTCTAGGCGTTTATTACGAGGCAATAATGGAAGGCAGAGTA
AAT---------GGAGAATTTCGCATTCAGAAA------AATGCGGCTGACCCA------
GGTACGGCGGCAATATTTGCTAATTATCCAGCTCCTTATTCGTCATCGGGTCTCTGTTTA
GTTGAAGAGATTTTGGCTACCATGATTTTCATGTTAGTCATTTGTGCAACAACAGACAAA
CGATACTCTAAGGTTCCT------------GCTTTCCTGCAACCTTTCTATATCGGCTTC
GCCTTGTTGGCCATGGGATTATCCTATGGGGCTAACAGTGGTTACGCTTTGAACCCGGCC
CGCGATTTGGGTCCTCGCATAACATCGTACTTTGGCGGATGGGGCTCTGGAGTCTTCAGT
TTCAGAGATTATAACGGGTTTTGGATTTTCATCGCCGGACCCCATATCGGAGCCATTTTA
GGAGTCTGCATCTTT---GAGCTTCTCTTAAAGCTCCGCCCTTCCGACAAAACA---GAT

---CTA---ATG---AAGGTCGAGCATGAACAACAAATACAGCGAAAGGAACAGGAACAA
CTCGAGATCGAATTA---AGCAATGTCAAGGCCATACGTGAGGAATACCATGCTTCA---
---------------------------------CCAATGGTCGAATTGCCAAGGGTAGGC
CTACGTTGG---------------------------------------------------
------------------------------------------------------------
------------------------------------------------------------
------------------------------------------------------------
------------------------------------------------------------
------------------------------------------------------------
------------------------------------------------------------
------------------------------------------------------------
------------------------------------------------------------
------------------------------------------------------------
------------------------------------------------------------
------------------------------------------------------------
------------------------------------------------------------
------------------------------------------------------------
------------------------------------------------------------
------------------------------------------------------------
------------------------------------------------------------
------------------------------------------------------------
------------------------------------------------------------
------------------------------------------------------------
------
>JAKUCG010000141_Ceriodaphnia_dubia_GlpA1
------------------------------------------------------------
------------------------------------------------------------
---------------------------------------------ATGCGC---TCGAGG
TTTTTC---------ATTCCACTTTTGGTGCGAACAGCTTTTGCGGAATTTGTTGGAACG
TACGTCTTAATCGTGATTGGCAACGGAAGTGTGGCCCAAGCCCAATTAACACACGGAGAA
AAAGGTGATTATTTTGCCATCAACTGGGGTTGGGCTTTAGGCTGCACACTGGGTATCCTG
ATCTCGGCCAATATATCAGGAGGTCATTTGAATCCAGCAGTGACTTTGGCTTTTGCCGTT
ACTCGTTCTTTCTCGTGGAAAAAAGTTCCAATTTATTGGTTGGCGCAATATTTGGGTTCT
CTAGCCGCTTCTGGTACCGTTTTAGGGATATATTATGAAGCGATAATGCAAGGCAGAGTA
AAC---------GGCGAATTCCGCATCCACCAA------AACCCTGCTGATCCA------
GGAAGTGCAGCTATATTTGCTAACTACCCAGCTCCCTATTCGTCAGCTGGACTTGGATTA
GTAGAAGAGATTTTAGCAACAATGATTTTCATGTTGGTAATTTGCGTAGCGACGGACAAG
CAATTTTCAAAAATTCCT------------AGCTTCTTGCAGCCCCTCTATATAGGGTTC
ACCTTGCTCGCGATTGGCATTGCCTACGGCGCAAACAGTGGCTATGCTTTAAATCCAGCA
CGAGATTTGGGTCCGCGGATCGTTTCGTATTTGGGGGGATGGGGTCCAGGAGTTTTTAGG
TTCAGGGGCTACAACTGGTTCTGGATTTTTATCGTCGGTCCGCACATTGGAGCTATATTG
GGAGTTAGTATCTTT---CAACTACTTTTAAAAATGCGCCGGACGGACGATCTA---GAT
---CTC---TTG---AAGACAGATCATGCTCGAGTACAAATGCAACTTAATGAAAAACAG
CACGAAATCGAGTTG---TGCCATATCAAGACCATCAATGAAGAACACCATGCATCCCAT
---------------------------------GCGATAGTAGAAATACCAAGGATAGGT
CTACGCTGG---------------------------------------------------
------------------------------------------------------------
------------------------------------------------------------

------------------------------------------------------------
------------------------------------------------------------
------------------------------------------------------------
------------------------------------------------------------
------------------------------------------------------------
------------------------------------------------------------
------------------------------------------------------------
------------------------------------------------------------
------------------------------------------------------------
------------------------------------------------------------
------------------------------------------------------------
------------------------------------------------------------
------------------------------------------------------------
------------------------------------------------------------
------------------------------------------------------------
------------------------------------------------------------
------------------------------------------------------------
------
>CM057928_Chydorus_sphaericus_GlpA1b
------------------------------------------------------------
------------------------------------------------------------
---------------------------------------------ATGCTC---CAACGA
TTCAGG---------ATTCCGATCCTTTTAAGAGTCGCTTTCGCAGAGTTTATCGGCACC
TACATCTTAGTGGTAATTGGGAACGGGTGCATAGCTCAAGCTACACTTAGTCAGGGTAAA
CTGGGCAATTTCCTCACCATTAACTGGGGATGGGCTCTTGGAGTTATGTTCGGAATCTTA
GTATCTAACAACATCTCAGGGGGCCACTTGAACCCAGCAGTGACTTTAGCCCTGTGCTTA
ACGAGGGTTTTTCCTTGGAAAAGGCTACCAATCTATTGGTTGGCCCAGTACCTTGGAGCC
ATGGCTGCTTCTGGCACTATTCTAGGAATCTACAGAGAGGCAATCGTTCTAATGATGAAA
ACAGAA------GGTCTCTTTAATGACGATAACGAGATGAACCCGGTCGATCCC------
GGCTCGGCTGCCATATTTGCAAACTATCCATCACCTTACCTACCCACTGGAGTTGGACTC
GCCCAAGAAATATTGGCTACTGCAATATTTATGCTAATCATATGTGTGACTACGGACCAA
AAATTTTCGAAAATTCCA------------GTATTTCTCCAACCATTCTACATAGGATTC
GCCTTGCTCGCCATAGGTTCATCTATGGGAGGCGACTACGGATATGCCCTCAATCCCGCA
CGAGATTTATCACCACGACTTGTATCTTTTTTTAGCGGATGG------------TTTAGT
TTTAGGGATTACAACTGGTTCTGGATTTTCATTGTCGGACCACATATCGGTGCCATTTTG
GGCGTATGCATTTTC---CAACTTTTTATGAAGACCCATCAGCAGCAGGGTATTCTCGGA
GGCGAATCAATT---AAGACCGTCACCACAGAGATTGGACAAACGCGACCACAGCTTCAT
CACGACTATGAATTC---GATAACATTGATAACACTAGGTTC------------------
------------------------------------------------------------
------------------------------------------------------------
------------------------------------------------------------
------------------------------------------------------------
------------------------------------------------------------
------------------------------------------------------------
------------------------------------------------------------
------------------------------------------------------------
------------------------------------------------------------
------------------------------------------------------------

------------------------------------------------------------
------------------------------------------------------------
------------------------------------------------------------
------------------------------------------------------------
------------------------------------------------------------
------------------------------------------------------------
------------------------------------------------------------
------------------------------------------------------------
------------------------------------------------------------
------------------------------------------------------------
------------------------------------------------------------
------
>CM057928_Chydorus_sphaericus_GlpA1a
------------------------------------------------------------
------------------------------------------------------------
---------------------------------------------ATGCTG---GGAAGA
TTCAGG---------ATTCCAATCGTTGTCAGAGCAGCTTTCGCTGAGTTCATCGGTACA
TTCATTCTCGTGGCGATCGGGAACGGGAGCATTGCTCAGGCTAGACTAACTAATGGAGAG
AAAGGAAATTTCTTCTCCATCAACTGGGGATGGGCTCTCGGAGTGATGCTCGGAATCTTA
GCATCTGGCAGTATTTCAGGAGGCCACTTGAATCCAGCAGTGACATTAGCGCTTGCTTTA
ACCAGAAACTTTTCCTGGAAAAAGTTACCTGTCTACTGGTTGGCTCAATATCTCGGAGCA
ATGGCTGCATCCGGCACTGTTCTAGGAATCTACAAAAACGCGATCATTTTAACCAAGTCG
AAG---------GGTATTTTCAAGATT------GAAATGAACCCTGAAGATCCG------
GGCTCAGCCGCTATATTTGCAAATTATCCAGCTGCTTACTTGTCCACCGGAATGGGCCTC
GTCGAAGAGATATTGGCTACCACAGTTTTTATGCTGATCATCTGCGTAACAACGGATCAA
AAGTATTCGAAAGTTCCA------------GCGTTCCTCCAGCCGTTTTACATCGGATTT
GCTCTTCTTGCTATTGGTACCGCTATGGGAGGCAATTCTGGATATGGTCTCAATCCCGCA
CGAGATTTAGCGCCTCGACTTGTCTCTTACTTCGGTGGATGG------------TTTAGC
TTCCGGGGCTACAACTGGTTCTGGATCTTCATCGTCGGTCCGCACATCGGTGCTATTTTG
GGCGTATCCATTTTT---CACCTTCTTCTAAGGACTCAGCAGGCCATCCAGGCAAGTGAG
---GTG---------GATTTCATCAAGGCAAAAGAACTTCGACGACAGCGGCAACTC---
CATGACATTGAACTG---TGCGACATTAAAGTCATCTATGACGAGCACTACCATCAC---
---------------------------------AAT---GACCCCCTTCCTACAGATGCT
AAACTATAC---------------------------------------------------
------------------------------------------------------------
------------------------------------------------------------
------------------------------------------------------------
------------------------------------------------------------
------------------------------------------------------------
------------------------------------------------------------
------------------------------------------------------------
------------------------------------------------------------
------------------------------------------------------------
------------------------------------------------------------
------------------------------------------------------------
------------------------------------------------------------
------------------------------------------------------------
------------------------------------------------------------

```
------------------------------------------------------------
------------------------------------------------------------
------------------------------------------------------------
------------------------------------------------------------
------------------------------------------------------------
------
>GGQP01091236_Diaphanosoma_celebensis_GlpA1
------------------------------------------------------------
------------------------------------------------------------
------------ATG------------CACTGT---------------CGC---CCGAAC
TGGTCTCTA---ACTCCGCCCCTTCTCCTGAAGCAGGCCTTTGCTGAATTCGTTGGCACT
TTCATCCTAGTGCTGATTGGCAATGGCAGCATCGCCCAGTCTCTGCTGTCCAACGAAGAG
AAAGGCGACTTCTTCTCCATCAATTGGGGCTGGGCCATGGGAGTCACTCTCGGTGTCCTG
ATATCCGCCAACATCTCAGGTGGCCATTTAAATCCAGCCATCACGTTGGCATTAAGTTTG
AAGCGTCAATTTCCCTGGAAGAGACTGCTCGTCTACTGGGCGGCACAATATCTGGGCGCT
CTGGTCGCTTCTGGAACAGTCTTTGGACTCTATCGAGAGGCAATATATGGAGGAAAACCC
CAT---------CACAAATTTCGCATC---AAA---GTGAATCCCAACGACCCG------
GGAACGGCGGTCATTTTTGCCAACTATCCGGAACCTTACACGTCCACAGGAGAAGCTCTA
GCCGAAGAGATTGTGGCGACGATGGTGCTCATGCTGGCCATTTGCGTCGTGACCGATCCT
CGATATTCCAAAGTTCCT------------GCTTACCTGCAGCCATTTTATGCCGGATTC
GCCATGCTGTCCATCGGACTCGCTTACGGAGCCAACTGCGGCTACGCACTGAATCCGGCC
CGTGATTTAGCTCCGCGACTCGTCTCTCTCATCGGCGGATATGGCGTCGGTGTTTTCAGT
TTCCGAGGCTACAACTGGTTCTGGGTGTTCATCGTCGGTCCGCACATTGGCGCCGTTTTG
GGCTACATAATCTTC---CGGCTGCTGTTAAAGTCCGTCCCTTCCGATCCGGCTCAGTCA
---ACCCAG------AAACCAAATAACCCGCCCGAAGTGTTCTACATGAAACCCACCTAC
AAAAGCACTTCCTTC---AATGACAACGAAGTTCAACTTGAATGCGTTAAATGT------
------------------------------------------------------------
------------------------------------------------------------
------------------------------------------------------------
------------------------------------------------------------
------------------------------------------------------------
------------------------------------------------------------
------------------------------------------------------------
------------------------------------------------------------
------------------------------------------------------------
------------------------------------------------------------
------------------------------------------------------------
------------------------------------------------------------
------------------------------------------------------------
------------------------------------------------------------
------------------------------------------------------------
------------------------------------------------------------
------------------------------------------------------------
------------------------------------------------------------
------------------------------------------------------------
------------------------------------------------------------
------------------------------------------------------------
------
```

>CM023188_Daphnia_carinata_GlpA3
------------------------------------------------------------
------------------------------------------------------------
------------ATGGTC---GATAAACGCTGC------------GTGCGG---AAGGCC
ATCAGCCTG---TCCGCCTACCCCCTTATCCGCGAATTCCTGGGAGAGCTTTCCGGAACC
TTCGTTCTAGTGTTATGCACCATAGGAACGCAGGCTCAGGCTGTTCTGACCAAAAGGAAA
CTGGCAGAGTTCTTGGCTAACAGGATGGGCGATGGAATAGGTGTTATGGCAGGAATTTGG
GTTGCAGGCGGCGTGTCAGGTGCCCATATGAACCCAGCAATGACGTTTGCTTTGGCCTGC
ACTGGAAAGGTTCCATGGCGTAAAGTACCGGCTTATTGGGCAGCTCAGTATCTTGGCGCT
TTTGCAGCTACATCTTGTGTTTTCGGAGTTTATTATGATGCAATTTACGCTCAAACTGGG
------------GGCCAGTTTATTATGACCGAT------------GAAGATCCG------
GGATTCGCTGCCATTTTTGCGCCCTACCCATCTGCCTATCTTTCAACGGCGGGGGCCGTG
GTCGATTCAATAATTGGATCAACATTATTGCTGATATGTGCCTCCGCCATAGTTGACCAA
AGGAATTGCAAAGTTCCA------------GTTCCTTTTATTGCCTTCTACATGGGATTT
ATTGTTATAGGGTTAGGCATAAGCCATAGCATGAACTGCGGCCTACCAATCAATCCTGCT
CGAGATTTTTCAGCAAGGCTCTTCACGTATCTAGTCGGATGGGGCCCTCAAGTATTCAGT
TACGACAACTGGATGTGGTTTTGGATACCGCTTGTGATCCCTCATTTCGGAGCATTCCTT
GGAGCTTTCATATAC---ATAATTTTTGTTAGTTCCCACTGGCCTGTAGTA---------
------------------------------------------------------------
------------------------------------------------------------
------------------------------------------------------------
------------------------------------------------------------
------------------------------------------------------------
------------------------------------------------------------
------------------------------------------------------------
------------------------------------------------------------
------------------------------------------------------------
------------------------------------------------------------
------------------------------------------------------------
------------------------------------------------------------
------------------------------------------------------------
------------------------------------------------------------
------------------------------------------------------------
------------------------------------------------------------
------------------------------------------------------------
------------------------------------------------------------
------------------------------------------------------------
------------------------------------------------------------
------------------------------------------------------------
------------------------------------------------------------
------------------------------------------------------------
------
>KAI9564579_Daphnia_sinensis_GlpA3
------------------------------------------------------------
------------------------------------------------------------
------------ATGGTC---GATAAACGCTGC------------GTGCGG---AAGGCC
ATCAGCCTG---TCCGCCTACCCCCTTATCCGCGAATTCCTGGGAGAGCTTTCCGGAACC
TTCGTTCTAGTGTTATGCACCATAGGAACGCAGGCTCAGGCTGTTCTGACCAAAAGGAAA

CTGGCAGAGTTCTTGGCTAACAGGATGGGCGATGGAATAGGTGTTATGGCAGGAATTTGG
GTTGCAGGCGGCGTGTCAGGTGCCCATATGAACCCAGCAATGACGTTTGCTTTGGCCTGC
ACTGGAAAGGTTCCATGGCGTAAAGTACCGGCTTATTGGGCAGCTCAGTATCTTGGCGCT
TTTGCAGCTACATCTTGTGTTTTCGGAGTTTATTATGATGCAATTTACGCTCAAACTGGG
------------GGCCAGTTTATTATGACCGAT------------GAAGATCCG------
GGATTCGCTGCCATTTTTGCGCCCTACCCATCTGCCTATCTTTCAACGGCGGGGGCCGTG
GTCGATTCAATAATTGGATCAACATTATTGCTGATATGTGCCTCCGCCATAGTTGACCAA
AGGAATTGCAAAGTTCCA-------------GTTCCTTTTATTGCCTTCTACATGGATTT
ATTGTTATAGGGTTAGGCATAAGCCATAGCATGAACTGCGGCCTACCAATCAATCCTGCT
CGAGATTTTTCAGCAAGGCTCTTCACGTATCTAGTCGGATGGGGCCCTCAAGTATTCAGT
TACGACAACTGGATGTGGTTTTGGATACCGCTTGTGATCCCTCATTTCGGAGCATTCCTT
GGAGCTTTCATATAC---ATAATTTTTGTTAGTTCCCACTGGCCTGTAGTA---------
------------------------------------------------------------
------------------------------------------------------------
------------------------------------------------------------
------------------------------------------------------------
------------------------------------------------------------
------------------------------------------------------------
------------------------------------------------------------
------------------------------------------------------------
------------------------------------------------------------
------------------------------------------------------------
------------------------------------------------------------
------------------------------------------------------------
------------------------------------------------------------
------------------------------------------------------------
------------------------------------------------------------
------------------------------------------------------------
------------------------------------------------------------
------------------------------------------------------------
------------------------------------------------------------
------------------------------------------------------------
------------------------------------------------------------
------------------------------------------------------------
------------------------------------------------------------
------
>CM023188_Daphnia_carinata_GlpA4
------------------------------------------------------------
------------------------------------------------------------
------------ATGGTC---GATAAACGCCGC------------GTGCGG---AAGGCC
ATTAGCCTG---TCGGCCTACCCTCTTATTCGCGAATTCTTAGGAGAGCTTGCCGGGACC
TTCGTTCTAGTGTTATGCATCATCGGAACGCAGGCTCAAGCCGTTTTGACCAACGGGAGA
CTAGCAGAGTTCTTGGCTAACCGGATGGGCGAAGGATTTGGTGTCATGGCCGGAGTCTGG
ATTGCTGGCGGAGTATCAGGTGCCCATTTGAACCCAGCCCTGTCATTTGCCTTGGCCTGC
ACCGGAAAATGTCAATGGCGCAAATTACCGGTTTATTGGATAGCCCAGTATCTCGGGGCT
TTCGCAGCTGCAGCTTGTGTCTTTGCAGTTTATCTCGAGGCAATTTTCTATCGCACCGGA
------------GGCCAGTTTGTCATGACCGAC------------GAAGACCCA------
GGATTCGCTGCCATTTTCGCTCCTTACCCGTCTGACTATCTTTCAATGACGGGCGCTATT

```
GTCGATTCGATAGTTGGATCAGCATTGTTGCTGATATGTGCCTCCGCCATAGTTGACCAA
AGGAATTGCAAAGTCCCT------------GTTTCTCTTCTTGCCTTTTACATGGATTC
GCCGTTATGGGATTGGGCATAAGCTTTAGCATGAACTGTGGCGTACCAATCAATCCTGCT
AGAGATTTTTCACCAAGGCTTTTCACTTACCTGGTTGGGTGGGGCCAACAAGTATTCAGT
TACAACAACTGGATGTGGTTTTGGATCCCTCTTGTTATCCCTCACTTTGGTGCTTTGCTT
GGAGCTTTCATCTAT---ATCGTTTTTATTGATGCCCACTGGCCCGCA------------
------------------------------------------------------------
------------------------------------------------------------
------------------------------------------------------------
------------------------------------------------------------
------------------------------------------------------------
------------------------------------------------------------
------------------------------------------------------------
------------------------------------------------------------
------------------------------------------------------------
------------------------------------------------------------
------------------------------------------------------------
------------------------------------------------------------
------------------------------------------------------------
------------------------------------------------------------
------------------------------------------------------------
------------------------------------------------------------
------------------------------------------------------------
------------------------------------------------------------
------------------------------------------------------------
------------------------------------------------------------
------------------------------------------------------------
------------------------------------------------------------
------------------------------------------------------------
------
>KAI9564580_Daphnia_sinensis_GlpA4
------------------------------------------------------------
------------------------------------------------------------
------------ATGGTC---GATAAACGCCGC------------GTGCGG---AAGGCC
ATTAGCCTG---TCGGCCTACCCTCTTATTCGCGAATTCTTAGGAGAGCTTGCCGGGACC
TTCGTTCTAGTGTTATGCATCATCGGAACGCAGGCTCAAGCCGTTTTGACCAACGGGAGA
CTAGCAGAGTTCTTGGCTAACCGGATGGGCGAAGGATTTGGTGTCATGGCCGGAGTCTGG
ATTGCTGGCGGAGTATCAGGTGCCCATTTGAACCCAGCCCTGTCATTTGCCTTGGCCTGC
ACCGGAAAATTGCAATGGCGCAAATTACCGGTTTATTGGATAGCCCAGTATCTCGGGGCT
TTCGCAGCTGCAGCTTGTGTCTTTGCAGTTTATCTCGAGGCAATTTTCTATCGCACCGGA
------------GGCCAGTTTGTCATGACCGAC------------GAAGACCCA------
GGATTCGCTGCCATTTTCGCTCCTTACCCGTCTGACTATCTTTCAATGACGGGCGCTATT
GTCGATTCGATAGTTGGATCAGCATTGTTGCTGATATGTGCCTCCGCCATAGTTGACCAA
AGGAATTGCAAAGTCCCT------------GTTTCTCTTCTTGCCTTTTACATGGATTC
GCCGTTATGGGATTGGGCATAAGCTTTAGCATGAACTGTGGCGTACCAATCAATCCTGCT
AGAGATTTTTCACCAAGGCTTTTCACTTACCTGGTTGGGTGGGGCCAACAAGTATTCAGT
TACAACAACTGGATGTGGTTTTGGATCCCTCTTGTTATCCCTCACTTTGGTGCTTTGCTT
GGAGCTTTCATCTAT---ATCGTTTTTATTGATGCCCACTGGCCCGCA------------
```

------------------------------------------------------------
------------------------------------------------------------
------------------------------------------------------------
------------------------------------------------------------
------------------------------------------------------------
------------------------------------------------------------
------------------------------------------------------------
------------------------------------------------------------
------------------------------------------------------------
------------------------------------------------------------
------------------------------------------------------------
------------------------------------------------------------
------------------------------------------------------------
------------------------------------------------------------
------------------------------------------------------------
------------------------------------------------------------
------------------------------------------------------------
------------------------------------------------------------
------------------------------------------------------------
------------------------------------------------------------
------------------------------------------------------------
------------------------------------------------------------
------------------------------------------------------------
------
>NC_046175_Daphnia_magna_GlpA4
------------------------------------------------------------
------------------------------------------------------------
------------ATGGTC---GATAAGCGCCGC------------GTGCGG---AAGGCC
ATCAGCCTG---TCGGCCTACCCTCTTATCCGCGAATTCTTAGGAGAGCTTGCCGGGACC
TTCGTTCTAGTGTTATGCATTATCGGAACGCAGGCTCAAGCCGTTTTGACCAACGGGAGA
CTAGCAGAGTTCTTGGCTAACCGGATGGGTGAAGGATTTGGTGTCATGGCCGGAGTTTGG
ATTGCTGGCGGCGTGTCAGGTGCCCATTTGAACCCAGCCCTGTCGTTTGCCTTGGCCTGC
ACTGGAAAATGTCAATGGCGCAAATTACCGGTTTATTGGATAGCCCAATATCTCGGGGCT
TTCGCAGCTGCGGCTTGTGTCTTTGCAGTTTATCTCGAGGCAATATTTTATCGCACCGGA
------------GGCCAGTTTGTCATGACCGAC------------GAAGACCCG------
GGATTCGCTGCCATTTTCGCTCCTTACCCGTCTGCCTATCTTTCAATGACGGGCGCTATT
GTTGATTCAATAGTTGGATCAGCATTGTTGCTGATATGTGCCTCCGCCATAGTTGACCAA
AGGAATTGCAAAGTCCCT------------GTTTCTCTTGTTGCCTTTTACATGGATTC
GCCGTTATGGGATTGGGCATAAGCTTTAGCATGAACTGTGGGGTACCAATCAATCCTGCT
AGAGATTTATCACCAAGGCTTTTCACTTACCTGGTTGGTTGGGGTCAACAAGTATTCAGT
TACAACAACTGGATGTGGTTTTGGATCCCTCTTGTGATCCCTCACTTCGGTGCTGTGCTT
GGAGCTTTCATCTAT---ATCGTTTTTGTTGATGCCCACTGGCCCGCA------------
------------------------------------------------------------
------------------------------------------------------------
------------------------------------------------------------
------------------------------------------------------------
------------------------------------------------------------
------------------------------------------------------------

------------------------------------------------------------
------------------------------------------------------------
------------------------------------------------------------
------------------------------------------------------------
------------------------------------------------------------
------------------------------------------------------------
------------------------------------------------------------
------------------------------------------------------------
------------------------------------------------------------
------------------------------------------------------------
------------------------------------------------------------
------------------------------------------------------------
------------------------------------------------------------
------------------------------------------------------------
------------------------------------------------------------
------------------------------------------------------------
------------------------------------------------------------
------
>JAKUCG010000105_Ceriodaphnia_dubia_GlpA4
------------------------------------------------------------
------------------------------------------------------------
------------ATGGTC---GATAAGCACCGAGGCGGAGGCGCCGTGCGG---AGGGCC
ATCAGCCTT---TCGGCCTACCCTCTTATCCGCGAATTCCTCAGCGAGCTGGCAGGAACT
TTTGTTTTAGTGTTGTGCATCATTGGAACAAAGGCACAAACCGTTCTCACCAACGGGAAC
CTGGCAGAATTCATGGCGAACCGGTTTGGCGAAGGATTCGGTGTCATGGCCGGTGTTTGG
ATTGGTGGCGGTGTGACAGGTGGTCATTTGAATCCCGCAATGACGTTCGCTTTGGCCTGT
ATGGGGAGAGTTCCTTGGCGTAAAGTCCCCGTCTACTGGATTGCTCAGTACATTGGCGCT
TTTGGAGCAGCTGCTTGTGTCTTCGGAGTCTATCTCGAGGCCATGGTTTATACAACCGGT
GGC---------CAACTATTCGTCATGACGGAT------------GACGATCCA------
GGATACGCAGCCATTTTCGCTCCCTATCCGGCAGCCTATCTTTCAATGATAGGGGCTATA
ATCGATTCGATTGTCGGATCAGCCTTGTTGTTAATATGCGCCTGCGCTATAGTCGACCAG
AAAAACTGCAACGTGCCT------------CTCTCGCTAGTTGCCTTTTATATGGGATGT
GTTATTATAGGACTAGGTATCAGTTTTAGCATGAACTGTGGTGTACCCGTAAATCCTGCA
AGGGATTTATCTCCAAGGTTGTTCACCTACCTAGTCGGCTGGGGCCCCAAAGTTTTCAGG
TATAACAACTGGATGTGGTTTTGGATCCCCCTCGCTATCCCGCACATCGGTGCTGTCCTT
GGAGGTTATATCTAT---ATAATTTTCATTGAAGCTCACTGGCCCAAA------------
------------------------------------------------------------
------------------------------------------------------------
------------------------------------------------------------
------------------------------------------------------------
------------------------------------------------------------
------------------------------------------------------------
------------------------------------------------------------
------------------------------------------------------------
------------------------------------------------------------
------------------------------------------------------------
------------------------------------------------------------
------------------------------------------------------------

------------------------------------------------------------
------------------------------------------------------------
------------------------------------------------------------
------------------------------------------------------------
------------------------------------------------------------
------------------------------------------------------------
------------------------------------------------------------
------------------------------------------------------------
------------------------------------------------------------
------------------------------------------------------------
------------------------------------------------------------
------
>HAFN01024804_Daphnia_galeata_GlpA4
------------------------------------------------------------
------------------------------------------------------------
------------ATGGTC---GATAAGCGTCGC------------GTGAGA---AGGGCC
ATCAGCCTG---TCTTCCTACCCCCTTATCCGCGAATTCCTTGGAGAACTGGCCGGGACC
TTCGTTCTAGTGTTATGCATTATTGGAACAAAGGCTCAATCAGTTCTGACCAACGGGAAA
CTTGCAGAGTTCTTGGCTGGCAGATTTGGCGATGGATTCGGTGTAATGGCCGGAGTTTGG
GTTGCTGGCGGTGTGTCAGGTGCTCATCTCAACCCTGCAATGTCGTTCGCTCTGGCCTGT
ATTGGAAGATTTCAATGGCGGAAATTACCGGTTTATTGGACTGCGCAGTATATTGGCGCT
TTTGCAGCTTCATCTTGCGTTTTCGCAGTTTACCTCGAGGCGATTTATTACTTTACTGGG
------------GGGCAATTTGTGATGACTGAA------------GATGATCCT------
GGATTCGCTGCCGTTTTCGCTCCATATCCTTCTGCGTACCTTTCAACGACGGGAGCCATA
ATCGATTCAGTAGTAGGATCAGCGTTGCTATTACTATGTGCCTGCGCTATAGTCGACCAA
CAAAACTGCAGAGTCCCA------------CTCTCTCTCTTTGCCTTATATATGGGATTC
CTCATAATGGCATTGGGCATAAGTTTCAGTATGAACTGCGGTGTACCCGTAAACCCGGCT
CGAGATTTATCCCCAAGGTTGTTCACTTACCTAGTCGGATGGGGTCCTCAAGTATTTAGC
TACGACAACTGGATGTGGTTCTGGATCCCTCTTGTGATTCCGCACTTCGGCGCAACTCTT
GGGGCTTTCATCTAC---ATTATTTTCATCGAAGCTCACTGGCCGAAA------------
------------------------------------------------------------
------------------------------------------------------------
------------------------------------------------------------
------------------------------------------------------------
------------------------------------------------------------
------------------------------------------------------------
------------------------------------------------------------
------------------------------------------------------------
------------------------------------------------------------
------------------------------------------------------------
------------------------------------------------------------
------------------------------------------------------------
------------------------------------------------------------
------------------------------------------------------------
------------------------------------------------------------
------------------------------------------------------------
------------------------------------------------------------
------------------------------------------------------------

------------------------------------------------------------
------------------------------------------------------------
------------------------------------------------------------
------------------------------------------------------------
------------------------------------------------------------
------
>JAAVJA010004895_Daphnia_dubai_GlpA4
------------------------------------------------------------
------------------------------------------------------------
------------ATGGTC---GATAAGCGTCGC------------GTGAGA---AGGGCC
ATCAGCCTG---TCTTCCTACCCCCTTATCCGCGAATTCCTTGGAGAACTGGCCGGGACC
TTCGTTCTAGTGTTATGCATTATTGGAACAAAGGCTCAAGCTGTTCTGACCAACGGGAAA
CTGGCAGAGTTCCTGGCTAGCAGATTTGGTGATGGATTCGGTGTCATGGCCGGAGTTTGG
GTTGCAGGCGGTGTGTCAGGTGCTCATTTAAACCCTGCAATGACGTTCGCTCTGGCCTGT
GTTGGAAAATGTCAATGGCGTAAATTACCCATCTACTGGACTGCCCAGTATATTGGCGCT
TTTGCAGCTTCTTCTTGCGTCTTCGCTGTTTACCTCGAGGCGATTTACTACTTCACTGGG
------------GGCCAATTTGACATGACTGAA------------GATGATCCT------
GGATTCGCTGCCGTTTTCGCTCCCTATCCTTCCGCCTACCTCTCAACGGCAGGAGCCATT
GTCGATTCAATAGTAGGATCAGCGTTGTTATTGCTATGTGCCTGCGCTATAGTCGATCAA
CAAAACTGTAAAGTCCCA------------CTCTCTCTCTTTGCCTTGTATATGGGATTC
CTCATAATGGGATTGGGCATAAGTTTCAGTATGAACTGCGGTGTACCCGTAAACCCGGCT
CGAGATTTATCCCCAAGACTGTTCACTTACTTAGTCGGATGGGGTCCTCAAGTATTTTCC
TACGACAATTGGATGTGGTTCTGGATCCCTCTTGTGATTCCACACTTTGGTGCAACCCTT
GGTGCCTTCATTTAC---ATCATTTTCATCGAAGCTCACTGGCCGAAA------------
------------------------------------------------------------
------------------------------------------------------------
------------------------------------------------------------
------------------------------------------------------------
------------------------------------------------------------
------------------------------------------------------------
------------------------------------------------------------
------------------------------------------------------------
------------------------------------------------------------
------------------------------------------------------------
------------------------------------------------------------
------------------------------------------------------------
------------------------------------------------------------
------------------------------------------------------------
------------------------------------------------------------
------------------------------------------------------------
------------------------------------------------------------
------------------------------------------------------------
------------------------------------------------------------
------------------------------------------------------------
------------------------------------------------------------
------------------------------------------------------------
------------------------------------------------------------
------

```
>EFX88758_Daphnia_pulex_GlpA4
------------------------------------------------------------
------------------------------------------------------------
------------ATGGTC---GATAAGCGTCGC------------GTGAGA---AGGGCC
ATCAGCCTG---GCTTCCTACCCCCTTATCCGGGAATTTCTTGGAGAGCTGGCCGGGACC
TTCGTTCTAGTGTTATGCATTATCGGAACAAAGGCACAAACGGTGTTGACCAACGGGAAA
CTGGCAGAATTCATGGCCGGCAGATTCGGCGATGGATTCGGTGTCATGGCCGGTGTTTGG
GTTGCAGGCGGTGTGTCAGGTGCCCATCTGAACCCAGCAATGTCGTTCGGTCTGGCCTGT
GTTGGAAGACTTGAATGGCGAAAGTTACCAGTCTATTGGGCTGCTCAGTATATCGGCGCT
TTTGCAGCTGCATCTTGCGTCTTTGCAGTTTACCTCGAGGCGATTTATTATCAAACTGGG
------------GGCCAATTTGTGATGACAGAA------------GATGATCCA------
GGATTCGCTGCCATTTTCGCTCCTTATCCTTCTGCCTACCTCTCAACAGCAGGAGCCATT
GTCGATTCGATAGTAGGATCGGCCCTGTTGTTGCTGTGTGCCTGCGCTATAGTCGATCGA
CTAAATTGCAAAGTTCCA------------CTTTCTGTCTTTGCCTTGTACATGGGATTT
CTCATCATGGGATTGGGCATCAGTTTCAGTATGAACTGCGGTGTGCCCGTCAATCCGGCT
CGAGATTTATCTCCAAGACTGTTCACTTACTTAGTCGGATGGGGTCCACAAGTATTTAGC
TACGACAATTGGATGTGGTTCTGGATCCCTCTTGTGATTCCGCACTTTGGTGCAACCCTT
GGAGCCTTCATTTAC---ATCATTTTTATCGAAGCTCACTGGCCGGAA------------
------------------------------------------------------------
------------------------------------------------------------
------------------------------------------------------------
------------------------------------------------------------
------------------------------------------------------------
------------------------------------------------------------
------------------------------------------------------------
------------------------------------------------------------
------------------------------------------------------------
------------------------------------------------------------
------------------------------------------------------------
------------------------------------------------------------
------------------------------------------------------------
------------------------------------------------------------
------------------------------------------------------------
------------------------------------------------------------
------------------------------------------------------------
------------------------------------------------------------
------------------------------------------------------------
------------------------------------------------------------
------------------------------------------------------------
------------------------------------------------------------
------------------------------------------------------------
------
>XM_046788235_Daphnia_pulicaria_GlpA4
------------------------------------------------------------
------------------------------------------------------------
------------ATGGTC---GATAAGCGTCGC------------GTGAGA---AGGGCC
ATCAGCCTG---GCTTCCTACCCCCTTATCCGGGAATTTCTTGGAGAGCTGGCCGGGACC
TTCGTTCTAGTGTTATGCATTATCGGAACGAAGGCACAAACGGTGTTGACCAACGGGAAA
```

CTGGCAGAATTCATGGCCGGCAGATTTGGCGATGGATTCGGTGTCATGGCCGGTGTTTGG
GTTGCAGGCGGTGTTTCAGGTGCCCATCTGAACCCAGCAATGTCGTTCGGTCTGGCCTGT
GTTGGAAGACTTGAATGGCGAAAGTTACCAGTCTATTGGGCTGCTCAGTATATCGGCGCT
TTTGCAGCTGCTTCTTGCGTCTTCGCAGTTTACCTCGAGGCGATTTATTATCAAACTGGG
------------GGCCAATTTGTGATGACAGAA------------GATGATCCA------
GGATTCGCTGCCATTTTCGCTCCTTATCCTTCTGCCTACCTCTCAACAGCAGGAGCCATT
GTCGATTCGATAGTAGGATCGGCCCTGTTGTTGCTGTGTGCCTGCGCTATAGTCGATCGA
CTAAATTGCAAAGTTCCA------------CTTTCTGTCTTTGCCTTATACATGGATTT
CTCATCATGGATTGGGCATCAGTTTCAGTATGAACTGCGGTGTGCCCGTCAATCCGGCT
CGAGATTTATCTCCAAGACTGTTCACTTACTTAGTCGGATGGGGTCCACAAGTATTTAGC
TACGACAATTGGATGTGGTTCTGGATCCCTCTTGTGATTCCGCACTTCGGTGCAACCCTT
GGAGCCTTCATTTAC---ATCATTTTTATCGAAGCTCACTGGCCGGAA------------
------------------------------------------------------------
------------------------------------------------------------
------------------------------------------------------------
------------------------------------------------------------
------------------------------------------------------------
------------------------------------------------------------
------------------------------------------------------------
------------------------------------------------------------
------------------------------------------------------------
------------------------------------------------------------
------------------------------------------------------------
------------------------------------------------------------
------------------------------------------------------------
------------------------------------------------------------
------------------------------------------------------------
------------------------------------------------------------
------------------------------------------------------------
------------------------------------------------------------
------------------------------------------------------------
------------------------------------------------------------
------------------------------------------------------------
------------------------------------------------------------
------------------------------------------------------------
------
>CM057928_Chydorus_sphaericus_GlpA4
------------------------------------------------------------
------------------------------------------------------------
------------ATGGTG---GGTAAGCGTGTT---------ACTTCTAAG---AGATCC
TGCAACTTC---GGTTCCTACCCGCTTCTACGCGAGTTCTTGGCTGAATTCACAGGGACA
CTCATCCTTGTGTTATGCATCAATGGATCCACAGCTCAAAGCATGCTGACTGGAGGTCGG
TTGGCTGGCTTTTTAGCTGGTAACTTTGCGGGTGGTGTAGGAGTGATGGCCGGTGTTTGG
ATTGCTGGCGGAATAACAGGAGGACACCTAAATCCAGCAATGTCATTTGGCCTTGCCTGT
GCTGGAAAGGTTTCATGGCGTAAACTACCTATTTACTGGTTAGCGCAATACCTTGGAGCA
TTTGCAGCAGCTTCTTGCGTCTTTGGCGTCTATATGGAGGCCATTTATAACAAGACTGGA
------------GGAGAACTTGTCATGACAGAC------------CAAGATCCT------
GGTTTGGCTGCCATATTTAGCACGTACCCCTCTCCGTACCTCTCAGTATTTGGATCGATC

CTTGACCAAACAGTAGAAGCAAGTTTGTTGCTGATTTCTGCCAGCGCGATCAACGATTCG
AGGAATTGTAACGTTCCT------------CTTTACTGGGTTGCATTCTACATGGGAATG
CTTGTTACCGGTTTGGGAGTAGGATTCTCAGCTAATTGTGGATCCCCACTTAATCCTGCC
AGAGATCTATCTCCTAGATTATTCAGCTATCTCGTGGATGGGGACGCCCGTTTTCAGT
TACAATGACTGGATGTGGTTTTGGGTTCCTGTTGTCTTTCCACATCTAGGAGCAGCCATT
GGAGCTATTATTTAT---CTCGTATTCATTGAGGCTCACTGGTCTCCG------------
------------------------------------------------------------
------------------------------------------------------------
------------------------------------------------------------
------------------------------------------------------------
------------------------------------------------------------
------------------------------------------------------------
------------------------------------------------------------
------------------------------------------------------------
------------------------------------------------------------
------------------------------------------------------------
------------------------------------------------------------
------------------------------------------------------------
------------------------------------------------------------
------------------------------------------------------------
------------------------------------------------------------
------------------------------------------------------------
------------------------------------------------------------
------------------------------------------------------------
------------------------------------------------------------
------------------------------------------------------------
------------------------------------------------------------
------------------------------------------------------------
------------------------------------------------------------
------
>GKAG01002845_Evadne_nordmanni_GlpA4
------------------------------------------------------------
------------------------------------------------------------
------------ATGGGG---GATAACCGCCGA---------------AAA---TTAGCT
ATACGT------TCCACA------TTCATTCGCGATTTGCTGTCAGAGTTCCTCAGTACC
TTCGTTTTCGTGCTATGCGTCAACGGACTCACAGCTCAATCCTTGGTAACTTCCGGCGAG
AAAGCCACCGCTTTAGCCTGTCAATTCGGTCATGGGATCGGCGCTATGGCTGGGATGTTC
ATCGGCGGAGGAGTTACAGGTGCTCATATGAACCCGGCCTTAACTCTCGGAATGGCTACC
GTAGGAAAACTGCCATGGCGGAAATTACCAGCGTACTGGATCGGCCAATATCTTGGGGCT
TTCACAGCTGCTACGGTGGTTTTCGTAGTCTACACTGAAGCCGTAATAGCA---ACC---
---------------GAT---------ACTAAT---------------------------
---TTGGCCACTATATTTGCAACGTATCCAGCGTCTTTCTTGTCCGTCGGAGGAGCCATT
TTTGATCAATTCGTTGGAACCGCTTTGCTAGTTGGCTGTGCAAGCGGCATCACAGATTTT
CGAAACAGTCGTGTTTCT------------CTAGCGGAAACAGCAATTTACATAGGCTTC
CTTATCATGGCCATACAAATCTCTTTCGCCGGTAACTGCGGAAGTTCCATGAATCCGGCT
CGAGATCTTTCACCTCGACTCTTGAGTTATTTGGTCGGATCAGGAATCGAAGTTTTCAGT
TTCCAAAATTGGTCGTGGTTTTGGATCCCAGTACTAATCCCGCACATTGGTGGCATAGTT
GGCGCTCTTCTTTAC---ACTATTTTCATCGAAGTTCACTGGCCTCAT------------

------------------------------------------------------------
------------------------------------------------------------
------------------------------------------------------------
------------------------------------------------------------
------------------------------------------------------------
------------------------------------------------------------
------------------------------------------------------------
------------------------------------------------------------
------------------------------------------------------------
------------------------------------------------------------
------------------------------------------------------------
------------------------------------------------------------
------------------------------------------------------------
------------------------------------------------------------
------------------------------------------------------------
------------------------------------------------------------
------------------------------------------------------------
------------------------------------------------------------
------------------------------------------------------------
------------------------------------------------------------
------------------------------------------------------------
------------------------------------------------------------
------------------------------------------------------------
------
>EFX88760_Daphnia_pulex_GlpA5
------------------------------------------------------------
------------------------------------------------------------
------------ATGGCT---TTCACATCAGCTCTGAAGTTATTGAAAAGT---TCCGTG
TCCGCTATT---TATCATCGGCTTTATGTTCGAGCTTTTTTAGCCGAATTTATAAGCACA
TTTGCTATTTTGGCGTTTGTGAATGGCTTAATCGCCCAGGCTGTCCTTACGGACGGAGAG
AAGGGAAGCGGAGTCGCCCCGAAATGGGGTGCGTTCGTTGCCGCCATGTCTGGTATCTTA
ATCGCTTGCGGTGCTTCAGGCGCTCACTCTAATCCGGCATTCTCTCTTCTATTCGCTCTG
AGAGGTATTTTACCCTGGGCTAAATTGCCGGTTTACTGGATAGCACAATATCTTGGTGCT
TTTTGCGGAGCAGCAGCTGTTCTTGGAGTTTATTGGGACGCCATAATGTTTAAAGGCAAT
------------GGCGAATTTTACATCACAAAC------------GAAGATCCG------
GGATTGGCTGCCATTTTTGCTACATATCCTATGCCATACGTCTCCATCGCAGGATGTATT
ATAGATCAGCTTTTTGGAAGTGCCTTACTTGCACTCTGTGTGGCCGCTTTGACCGATCCC
AAGAACATGCGTATTCCG------------CTTCCGCTCGTGGCCCTGTATATTGGATTT
TTGGTTTTGGAATTGGCTATTGGATTCTCACTTAATACAGGTGGCTCTCCGAATCCTGCT
CGCGATTTATCTCCTAGGATTTTGACTTACTTTGCTGGATGGGGAATCCAGGTCTTT---
---AGAGACTGGACCTGGTTTTGGATTCCCGTAGTAATACCTCACATTGGTGTCGTAATC
GGTTATTTAATATAT---TTTATTCTCATTGAAGCGCATTGGCCAACACAA---------
------------------------------------------------------------
------------------------------------------------------------
------------------------------------------------------------
------------------------------------------------------------
------------------------------------------------------------
------------------------------------------------------------

------------------------------------------------------------
------------------------------------------------------------
------------------------------------------------------------
------------------------------------------------------------
------------------------------------------------------------
------------------------------------------------------------
------------------------------------------------------------
------------------------------------------------------------
------------------------------------------------------------
------------------------------------------------------------
------------------------------------------------------------
------------------------------------------------------------
------------------------------------------------------------
------------------------------------------------------------
------------------------------------------------------------
------------------------------------------------------------
------------------------------------------------------------
------
>XM_046788233_Daphnia_pulicaria_GlpA5
------------------------------------------------------------
------------------------------------------------------------
------------ATGGCT---TTCACATCAGCTCTGAAGTTATTGAAAAGT---TCCGCG
TCCGCTATT---TATCATCGGCTTTATGTTCGAGCTTTTTTAGCCGAATTTATTAGCACA
TTTGCTATTTTGGCGTTTGTGAATGGCTTAATTGCCCAGGCTGTCCTTACGGACGGAGAG
AAGGGAAGCGGAGTCGCCCCGAAATGGGGTGCGTTCGTTGCCGCAATGTCCGGTATCTTA
ATCGCTTGCGGTGCTTCAGGCGCTCACTCTAATCCGGCATTCTCTCTTCTATTCGCTCTG
AGAGGTATTTTACCCTGGGCTAAATTGCCGGTTTACTGGATAGCACAATATCTTGGTGCT
TTTTGCGGAGCAGCAGCTGTTCTTGGAGTTTATTGGGACGCCATAATGTTTAAAGGCAAT
------------GGCGAATTTTACATCACAAAC------------GAAGATCCG------
GGATTGGCTGCCATTTTTGCTACATACCCTTTGCCATACGTCTCCATCGCAGGATGTATT
ATAGATCAGCTTTTTGGAAGTGCCTTACTTGCACTCTGTGTGGCCGCTTTGACCGATCCC
CAGAACATGCGTATTCCG------------CTTCCGCTCGTTGCCCTGTATATTGGATTT
TTGGTTTTGGAATTGGCTATTGGATTCTCACTTAATACAGGTGGCTCTCCGAATCCTGCT
CGCGATTTATCTCCTAGGATTTTGACTTACTTTGCTGGATGGGGAATCCAGGTCTTTAGT
TTCAGAGACTGGACCTGGTTTTGGATTCCCGTAGTAATACCTCACATTGGTGTCGTAATC
GGTTATTTAATATAT---TTTATTCTCATTGAAGCGCATTGGCCAGCACAA---------
------------------------------------------------------------
------------------------------------------------------------
------------------------------------------------------------
------------------------------------------------------------
------------------------------------------------------------
------------------------------------------------------------
------------------------------------------------------------
------------------------------------------------------------
------------------------------------------------------------
------------------------------------------------------------
------------------------------------------------------------
------------------------------------------------------------

------------------------------------------------------------
------------------------------------------------------------
------------------------------------------------------------
------------------------------------------------------------
------------------------------------------------------------
------------------------------------------------------------
------------------------------------------------------------
------------------------------------------------------------
------------------------------------------------------------
------------------------------------------------------------
------------------------------------------------------------
------
>GGXW01231853_Daphnia_galeata_GlpA5
------------------------------------------------------------
------------------------------------------------------------
------------ATGGCT---TTCACATCAGCGCTGAAGTTACTGAAAAGA---TCCGGG
TCTGCTATG---TATCATCGTCTTTATGTTCGAGCTTTTTTAGCCGAATTTATTAGCACT
TTTGCTATTTTGGCGTTTGTGAATGGCTTAATCGCCCAGGCTGTTCTTACGGACGGAGAA
AAAGGAAACGGAGTCGCCCCGAAATGGGGTGCTTTCGTTGCCGCTATGTCTGGTATCTTA
ATTGCTTGTGGTGCATCAGGTGCACATTCTAATCCGGCGTTCTCTCTCCTCTTCGCTCTG
AGAGGTATTTTGCCATGGGCTAAATTGCCGGTTTACTGGTTAGCACAATATCTTGGTGCT
TTTTGCGGAGCAGTAGCTGTCCTTGGAGTCTATTGGGACGCTATAATGTTTAAAGGGAAT
------------GGCGAATTTTACATCACAAAT------------GATGATCCG------
GGATTGGCTGCCATTTTTGCTACATATCCTATGCCTTACGTTTCCATCTCAGGCTGCATT
ATAGATCAGCTTTTTGGTAGCGCGTTACTTGCTCTCTGCGTGGCTGCTTTAACCGATCCC
AAAAACATGCGAATTCCG------------CTGCCTTTGGTGGCTTTTTATATTGGATTT
TTGGTTCTAGAATTGGCAATCGGATTCTCACTTAATACTGGTGGCTCACCGAATCCTGCT
CGTGACTTATCTCCGAGGATTTTAACTTATTTTGCTGGATGGGGAATCAAGGTCTTTAGT
TTCCGTGATTGGACCTGGTTTTGGATTCCAATTGTGATACCTCATATTGGTGTTGTGATC
GGTTATTTAATATAT---TTTCTTCTCATTGAAGCGCATTGGCCAACTCAA---------
------------------------------------------------------------
------------------------------------------------------------
------------------------------------------------------------
------------------------------------------------------------
------------------------------------------------------------
------------------------------------------------------------
------------------------------------------------------------
------------------------------------------------------------
------------------------------------------------------------
------------------------------------------------------------
------------------------------------------------------------
------------------------------------------------------------
------------------------------------------------------------
------------------------------------------------------------
------------------------------------------------------------
------------------------------------------------------------
------------------------------------------------------------
------------------------------------------------------------

------------------------------------------------------------
------------------------------------------------------------
------------------------------------------------------------
------------------------------------------------------------
------------------------------------------------------------
------
>JAAVJA010004895_Daphnia_dubai_GlpA5
------------------------------------------------------------
------------------------------------------------------------
------------ATGACT---TTCACATCAGGTCTGAAGTTATTGAAACGA---TCCGGC
TCCACTATA---TATCATCGACTTTATGTTCGAGCTTTTTAGCCGAATTTATTAGTACG
TTTGCTGTTTTGGCTTTTGTGAATGGTTTGATCGCTCAGGCCGTCCTTACGGACGGAGAG
AAGGGAAGTGGAGTCGCTCCGAAGTGGGGTGCGTTTGTTGCCGCCATGTCAGGTATCTTA
ATCGCTTGTGGAGCATCAGGTGCGCATTCTAATCCGGCGTTCTCTCTCCTTTTTGCCCTG
AGAGGTATTCTACCGTGGGCTAAATTGCCGGTTTACTGGTTAGCACAATACCTTGGGGCT
TTTTGCGGAGCAGCAGCCGTTCTTGGAGTCTATTGGGACGCGATAATGTTCAAAGGGAAT
------------GGCGAATTTTACATCACTAAC------------GAAGATCCA------
GGGTTAGCTGCCATTTTCGCTACATATCCCATGCCTTACGTTTCGATCACAGGCTGTATT
ATAGATCAG---TTTGGTAGTGCCTTACTTGCTCTCTGTGTGGCCGCTTTAACCGATCCC
AAAAACATGCGCATTCCA------------CTTCCGCTAGTGGCATTGTATATTGGATTT
CTGGTGCTAGAATTGGCGATTGGATTTTCACTTAATACCGGTGGATCACCGAATCCTGCT
CGAGATTTATCTCCGAGGATTTTAACTTATTTTGCTGGATGGGGAATAAGGGTCTTTAGC
TTCCGAGACTGGACCTGGTTTTGGATTCCAGTCGTGATCCCGCATTTTGGTGTGGTGTTT
GGTTATTTAATTTAT---TTTATTCTTATTGAAGCGCATTGGCCAACACAA---------
------------------------------------------------------------
------------------------------------------------------------
------------------------------------------------------------
------------------------------------------------------------
------------------------------------------------------------
------------------------------------------------------------
------------------------------------------------------------
------------------------------------------------------------
------------------------------------------------------------
------------------------------------------------------------
------------------------------------------------------------
------------------------------------------------------------
------------------------------------------------------------
------------------------------------------------------------
------------------------------------------------------------
------------------------------------------------------------
------------------------------------------------------------
------------------------------------------------------------
------------------------------------------------------------
------------------------------------------------------------
------------------------------------------------------------
------------------------------------------------------------
------------------------------------------------------------
------

```
>JAKUCG010000105_Ceriodaphnia_dubia_GlpA5
------------------------------------------------------------
------------------------------------------------------------
------------ATGACT---TACACA---GGTCTGAAGGCTTTAAGCACA---TTCGGG
TCCGTCATT---TACCACCGACTGTACGTTCGAGCTTTTTTGGCCGAATTCATCAGCACA
TTTGCCATCATAGCTTTTGTCAATGGCTTAATCGCGCAGTCGATACTCACCGATGGAGAA
AAAGGAAGCGGGGTCGGGCCGCTTTTCGGAGCTCTAGTAGCCGCAATGTCTGGTATCTTG
ATCGCGTGTGGGGCTTCGGGTGCTCATTCCAACCCCGCATTCTCTTTGCTTTTTGCGCTA
AGAGGTATTTTGCCGTGGGCTAAATTGCCGGTTTATTGGATAGCGCAATATCTTGGCGCT
TTTTTTGGAGCTGCGGCTGTCCTTGGAGTGTACTGGGATGCATTGTCGATCAGGTATTTT
------------CGAACTGAATATATTAGCAAT---------------------------
------------------------------------------------------------
---------TTGGTTGGAAGCGCATTGTTAGCCCTGTGTGTCGCCGCCATAACTGATCCA
AAAAACATGAGAATCCCA------------CTCCCATGGGTGGCGTTCTATTTGGGACAT
CTAGTCCTAGGTCTAGCGATTGCATTATCCAGCAACACCGGTGGTTCTCCAAATCCCGCT
CGAGATTTGTCTCCTAGGATTCTAAGTTGTATTGCCGGATGGGGAATTAAAGTTTTTAGC
TTCCGAGACTGGACCTGGTTTTGGATACCTATTGTGATGCCTCATTTTGGTGTAATGATT
GGATACTCGACATAC---TTTTTGCTCATTGAAGCTCATTGGCCGATC------------
------------------------------------------------------------
------------------------------------------------------------
------------------------------------------------------------
------------------------------------------------------------
------------------------------------------------------------
------------------------------------------------------------
------------------------------------------------------------
------------------------------------------------------------
------------------------------------------------------------
------------------------------------------------------------
------------------------------------------------------------
------------------------------------------------------------
------------------------------------------------------------
------------------------------------------------------------
------------------------------------------------------------
------------------------------------------------------------
------------------------------------------------------------
------------------------------------------------------------
------------------------------------------------------------
------------------------------------------------------------
------------------------------------------------------------
------------------------------------------------------------
------------------------------------------------------------
------
>CM057928_Chydorus_sphaericus_GlpA5
------------------------------------------------------------
------------------------------------------------------------
------------ATGATT---CAGTGGCCATGGCAGACCTGGAAAAGGCGG---TTCCGA
GCGTTCTTT---CAAGATCGTCTATATGTGCGCGCATTCTTGGGTGAATTCATCAGCACC
TACGCCATTATTGCGTTTATAAATGGAATGATTGCGCAAGCCGTCCTTACAGACGGAGAA
```

AAATCGCCCGGTGTAGGTCCTTCATGGGGAGCAGCAGTTGCCTCAATGACCGGCATCTGG
ATAGCAATCGGAGTGTCGGGAGCGCACTCAAATCCAGCATTCTCTCTTTTATTCACTCTT
CGAGGCTTTATTACTTGGAGGCAATTACCCATCTACTGGCTAGGACAATACCTGGGCGCT
ATAGCGGGAGCCGCTTCCGCACTTGGTGTCTATTGGGAGGCGCTGATGGATAAGACTGGA
------------GGCCAATTGAGTATTACGAAT------------ACGGATCCC------
GGTTTAGCTATGATCTTTGCCACCTATCCGGCACCTTACGTCTCAGTTTTAGGAGCTATT
CCTGAACAGATAGCAATTAGCTCTTTAATGGCGCTTTGCGTAACCGCTATGACGGATCAG
AAGAACATGAAGGTTCCC------------TTTACAATGCTATCAATCTACCTTGGGTTT
ATGATTCTTGCCCTATCCCTTGGCTTCTCTATGAATTCCGGAGGAAGTCCCAATCCAGCC
AGGGATCTGGGCCCACGCATCGTGAGCTATTTTGCCGGTTGGGGTCTTCCTGTTTTCAGT
TTTCGGAACTGGACTTGGTTTTGGGTTCCCGTTCTCATGCCTCACATAGGCGTCATTATC
GGTTATGCTATTTAT---TTTGTGTTTATTGAAGCCCATTGGCCAATAGAAAATGGAAAT
---CCCAAT---------------------------------------------------
------------------------------------------------------------
------------------------------------------------------------
------------------------------------------------------------
------------------------------------------------------------
------------------------------------------------------------
------------------------------------------------------------
------------------------------------------------------------
------------------------------------------------------------
------------------------------------------------------------
------------------------------------------------------------
------------------------------------------------------------
------------------------------------------------------------
------------------------------------------------------------
------------------------------------------------------------
------------------------------------------------------------
------------------------------------------------------------
------------------------------------------------------------
------------------------------------------------------------
------------------------------------------------------------
------------------------------------------------------------
------------------------------------------------------------
------------------------------------------------------------
------
>NKDA01000001_Eulimnadia_texana_GlpA5
------------------------------------------------------------
------------------------------------------------------------
------------ATG------------------------------AATCTT---TGGAGG
ATCATGAGC---------AGGAAAGTAATTCGCGAATTTTCGGCAGAATTTCTTGGAACT
TTTGTTCTGATTCTTTTCGGTAATGCAAGTGTAGCGCAGTCACAAGTCACCAATGAATCT
AAAGGAGATTTTTTCGCCATTAACTGGGGCTGGGCCCTTGGTGTCATGATGGGTATCCTC
ATATCGCAGAAGATATCTGGCGCCCATCTGAATCCAGCTGTGACGGGAGCACTAGCCATT
TTCGGACGATTTTCGTGGCGAAAGATTTGGTTTTACTGGTTAGCACAGTACCTGGGAGCA
TTTCTAGCAGCTGCTTGCGTCTACGGTGCCTACTACGAGGGTATTGTGGCGCGGTCTGGT
------------GGCAGCTTTCGCATGAGTGAA------------AGCAATCCA------
GGCTTAGCTGTGATCTTCTCGACTTATCCGTCGTCGTACCTGTCGGCAGCTGGAGGTTTA

GGAGACCAGATACTAGGAACAGCTTTACTGCTCATCTGCATTTGCGCCATTACCGATCCT
GAAAAT---CGTATCCCG------------GATTATTTCGTTGCTTTTCTCGTGGGTTTA
GTGATATTAGCCATTGGCATTTGCTTTGGGGCCAATTGTGGATACGCACTGAACCCAGCA
AGGGATCTAGCTCCTCGTCTTGTCAGTCTCATAGGGGGTTGGGGTCCTGAAGTGTTCAGG
TTCCGACATTACAACTGGTTCTGGATCTTAGTAGTTGGACCACACATTGGCGCGGTCCTG
GGTATTTTGATTTAC---GTGATCCTCATAGAAATGCCACGGACACAGAACAGTGAGGAC
---CGGCAAGAATCCGGTTCGTCCCATTGCGAACTTATGGCGAAACCAGTGCGCATC---
------ACTGAAGACGTTAAAATCCATATGTCGCGTAAG---------------------
------------------------------------------------------------
------------------------------------------------------------
------------------------------------------------------------
------------------------------------------------------------
------------------------------------------------------------
------------------------------------------------------------
------------------------------------------------------------
------------------------------------------------------------
------------------------------------------------------------
------------------------------------------------------------
------------------------------------------------------------
------------------------------------------------------------
------------------------------------------------------------
------------------------------------------------------------
------------------------------------------------------------
------------------------------------------------------------
------------------------------------------------------------
------------------------------------------------------------
------------------------------------------------------------
------------------------------------------------------------
------------------------------------------------------------
------
>JAIQCU010000268_Leptestheria_dahalacensis_GlpA5
------------------------------------------------------------
------------------------------------------------------------
------------ATG---------------------------------CTG---CGAAGG
ATACTAAGG---------CGAGACGGAATCCGCGAGTTCTTTGCAGAATTCCTGGGAACT
TTTGTGTTAATTCTCTTCGGCAATGCAAGTGTAGCTCAGTCTCAGGTTTCTAACGAATCC
AAAGGAGATTTCTTTGCCATCAACTGGGGTTGGGCCCTAGGAGTCATGATGGGCATCTTG
GTATCTCAGAAAACTTCAGGAGCCCATTTAAATCCAGCCGTAACAGGAGCCCTGGCGGTT
TTCAAAAGATTCCCCTGGCAGAAGATCTGGATCTATTGGTCTGCGCAGTATCTTGGAGCT
CTCTTAGCAGCTGTATGTGTATACGGAGCCTACAGAGAGGGTATCATGGCTCAAACTGGC
------------GGACACTATTTGATGACCGAG------------ACAAACCCA------
GGCCTTGCCGGAATATTCGCTACGTATCCAGCGTCTTATCTTTCAGCTTTTGGTGGACTT
GGAGATCAGATATTAGGAACCGCTTTACTTCTGATTTGCATCTGCGCCATAACCGATCCT
GAAAAT---CAAATCCCA------------AGTTATGCAGTTGCATTCTTGGTCGGTTTG
GTTATTCTGGCCATTGGAATTTGTTTCGGAGCAAACTGCGGTTACGCATTGAACCCAGCT
AGGGATCTAGCTCCGCGACTAGTGAGTTTACTTGCAGGATGGGGTCCACAAGTTTTCAGG
TATAGGAATTTCAACTGGTTCTGGATTCTCGTAATCGGTCCGCATGTCGGAGCGATCCTG
GGGATGTTGATCTAC---ATCGGCCTTATCGAGGTACCTCGTAAGGCGAAACAAGAATAT

------ATCGAACAAAATCACTATACCGGTGATACCGCTCTGAAGTCCCAGGTTACA---
------CTTGACCAAATCACAATAAGCATAACCCGTAAGTGTTGCATTTCAATA------
------------------------------------------------------------
------------------------------------------------------------
------------------------------------------------------------
------------------------------------------------------------
------------------------------------------------------------
------------------------------------------------------------
------------------------------------------------------------
------------------------------------------------------------
------------------------------------------------------------
------------------------------------------------------------
------------------------------------------------------------
------------------------------------------------------------
------------------------------------------------------------
------------------------------------------------------------
------------------------------------------------------------
------------------------------------------------------------
------------------------------------------------------------
------------------------------------------------------------
------------------------------------------------------------
------------------------------------------------------------
------------------------------------------------------------
------
>RJJA01000048_Lepidurus_arcticus_Glp
------------------------------------------------------------
------------------------------------------------------------
------------ATG------GAG------------------------CTG---TTCCAG
TCTTACCAA---ATTAAAACGCCGCTGATACGCGAAATGTTGGCGGAATTTTTGGGAACC
TTCTTGCTCGTGTTGTTTGGAGATGCCAGCGTGGCGCAGTCTAAGCTCACCAACAATGTC
AATGGAGATTTCTTTTCGATTAATTGGGGTTGGGGGTTAGCAGTCATGATGGGAGTTCTA
GTAGCCGGAAAAGTATCAGGTGCACACTTGAATCCTGCCGTCTCAGTCGCTTTTGCGATA
TTTGGCAAATTCCCTTGGCTGAAGCTACTGCCCTATGTATTAGCGCAATACCTTGGAGCC
TTGGCTGCTGCAGCATCCGTTTTTGGGGTCTATTCAGAAGCTATATGGGATTTTTCGGGC
------------GGAAATCTTACTGTT---GAT------------GGTATAACC------
GGCACCGCAGGTATATTTGCAACGTACCCAGCGGAATACGTCTCAGCTTCAGGTGGATTT
GGGGATCAAGTCTTGGGAACCATGCTTCTCTTGCTCTGTATTTGCGCCATTGTTGACAAG
AAAAATATGTCGGTTCCG------------AGCGGTTTGGTTCCGTTATATATTGGTTTC
GTTATTTTGGCCATTGGAGTATGTTTCGGCTCCAACTGCGGTTATGCTATTAACCCTGCT
AGGGATCTAGCGCCTCGCTTCCTTACCTTCATTGCCGGATGGGGTACTGATGTGTTTAGG
TATCGAGGCTACAACTGGTTCTGGGTACCTATCGTTGGCCCGCACATTGGCGCCATCTTG
GGAGTACTCATATAC---ACTCTGTGCATTGAACTGCATTGGCCGGAAACTCCAGAAATT
---CCTGTTGGC---AAT---------------------------------GCTGGA---
------AGGGACGATAAGAATGTTCGTGTTGTTGAATTGGAAAGACTC------------
------------------------------------------------------------
------------------------------------------------------------
------------------------------------------------------------
------------------------------------------------------------

------------------------------------------------------------
------------------------------------------------------------
------------------------------------------------------------
------------------------------------------------------------
------------------------------------------------------------
------------------------------------------------------------
------------------------------------------------------------
------------------------------------------------------------
------------------------------------------------------------
------------------------------------------------------------
------------------------------------------------------------
------------------------------------------------------------
------------------------------------------------------------
------------------------------------------------------------
------------------------------------------------------------
------------------------------------------------------------
------------------------------------------------------------
------
>JAGJRP010001432_Lepidurus_couesii_Glp
------------------------------------------------------------
------------------------------------------------------------
------------ATG------GAG------------------------CTG---TTCCAG
TCTTACCAC---ATTAAAACGCCGTTGATACGCGAAATGTTGGCGGAATTTTTGGGAACC
TTCTTGCTCGTGTTGTTTGGAGATGCCAGCGTGGCGCAGTCTAAACTCACCAACAATGTT
AATGGGGATTTCTTTTCGATTAATTGGGGTTGGGGGTTGGGAGTCATGATGGGAGTTCTA
GTGGCCGGAAAAGTATCAGGTGCACACTTGAATCCTGCCGTCTCAGTCGCTTTTGCGATA
TTTGGTAAATTCCCTTGGCTGAAGCTACTACCCTATGTATTAGCGCAATACCTCGGAGCC
TTGGCTGCTGCAGCATCCGTTTTTGGAGTCTATTCAGAAGCTATATGGGATTTTTCGGGC
------------GGAAATCTTACTGTT---GAT------------GGTAATACC------
GGCACCGCAGGTATATTTGCAACGTACCCAGCGGAATACGTCTCAGCTTCAGGCGGATTT
GGGGATCAAGTCTTGGGAACCATGCTCCTCCTGCTCTGTATTTGCGCCATTGTTGACAAG
AAAAATATGTCGGTTCCG------------AGCGGGTTGGTTCCTTTATACATTGGTTTC
GTTATTTTGGCCATTGGAGTATGTTTCGGCTCCAATTGCGGTTATGCTATTAACCCTGCT
AGGGATCTAGCGCCTCGCTTCCTTACCTTCATTGCCGGATGGGGTACTGATGTGTTTAGG
TATCGAGGCTACAACTGGTTCTGGGTACCTATCGTTGGCCCGCACATTGGCGCCATCTTG
GGAGTACTCATATAC---ACTCTGTGCATCGAACTGCATTGGCCGGAAACCCCAGAAATT
---CCTGTTGGC---AAT---------------------------------GCTGGG---
------AGGGACGATAAGAATGTTCGTGTTGTTGAATTGGATAGACTT------------
------------------------------------------------------------
------------------------------------------------------------
------------------------------------------------------------
------------------------------------------------------------
------------------------------------------------------------
------------------------------------------------------------
------------------------------------------------------------
------------------------------------------------------------
------------------------------------------------------------
------------------------------------------------------------

------------------------------------------------------------
------------------------------------------------------------
------------------------------------------------------------
------------------------------------------------------------
------------------------------------------------------------
------------------------------------------------------------
------------------------------------------------------------
------------------------------------------------------------
------------------------------------------------------------
------------------------------------------------------------
------------------------------------------------------------
------
>JAKZAN010000001_Lepidurus_packardi_Glp
------------------------------------------------------------
------------------------------------------------------------
------------ATG------GAG------------------------CTG---TTCCAG
TCTTACCAA---ATTAAAACGCCGTTGATACGCGAAATGTTGGCGGAATTTTTGGGAACC
TTCTTGCTTGTGTTATTTGGTGATGCCAGCGTGGCGCAGTCTAAACTCACCAAGTATGTC
AATGGGGATTTCTTTTCGATTAATTGGGGTTGGGGGCTAGGAGTCATGATGGGAGTTCTA
GTGGCCGGAAAAGTATCAGGTGCACACTTGAATCCTGCCGTGTCAGTCGCTTTTGCGATA
TTTGGTAAATTCCCTTGGCTAAAGCTACTGCCCTATGTATTAGCGCAATACCTTGGAGCC
TTGGCGGCTGCAGCATCCGTTTTTGGAGTCTATTCAGAAGCTATATGGGATTTTTCGGGC
------------GGAAATCTTACTATT---GAT------------GGTAATACC------
GGCACCGCAGGTATATTTGCCACGTACCCTGCGGAATACGTCTCAGCTTCAGGCGGATTT
GGGGATCAAGTCTTGGGAACCATGCTTCTCCTACTCTGTATTTGCGCCATTGTTGACAAG
AAAAATATGTCGATTCCG------------AGCGGGTTAGTTCCGTTATATATTGGTTTC
GTTATATTGGCCATTGGAGTGTGCTTCGGCTCCAACTGCGGTTATGCTATTAATCCTGCT
AGGGATCTAGCACCTCGCTTCCTTACCTTCATTGCCGGATGGGGTACTGATGTGTTTAGG
TATCGAGGCTATAACTGGTTCTGGGTGCCTATCGTTGGACCGCACATTGGCGCCATCTTG
GGAGTGCTCATATAC---ACACTGTGCATCGAACTGCATTGGCCGGAAACTCCAGAAATT
---GCTGCTGTC---CAT---------------------------------GCTGGA---
------AAGGACGATAAGAACGTTCGTGTTGTTGAATTGGAAAGACTC------------
------------------------------------------------------------
------------------------------------------------------------
------------------------------------------------------------
------------------------------------------------------------
------------------------------------------------------------
------------------------------------------------------------
------------------------------------------------------------
------------------------------------------------------------
------------------------------------------------------------
------------------------------------------------------------
------------------------------------------------------------
------------------------------------------------------------
------------------------------------------------------------
------------------------------------------------------------
------------------------------------------------------------
------------------------------------------------------------

------------------------------------------------------------
------------------------------------------------------------
------------------------------------------------------------
------------------------------------------------------------
------------------------------------------------------------
------
>RJJB01000217_Lepidurus_apus_Glp
------------------------------------------------------------
------------------------------------------------------------
------------ATG------GAG------------------------CTG---TTCCAG
TCTTACCAA---ATTAAAACGCCGTTGATCCGCGAAATGTTGGCGGAATTTTTGGGAACC
TTCATGCTTGTGTTATTTGGAGATGCCAGCGTTGCGCAGTCTAAACTTACCAACACAGAC
AATGGTGATTTCTTTTCGATTAATTGGGGCTGGGGATTAGGAGTCATGATGGGAGTTCTA
GTGGCCGGAAAAGTATCAGGTGCACACTTGAATCCTGCCGTCTCTGTCGCTTTTGCGATA
TTTGGTCGATTCCCTTGGCTGAAGCTACTCCCCTATGTATTAGCGCAATACCTTGGAGCC
TTGGCTGCTGCAGCATCCGTTTTCGGAGTCTACTCAGAAGCTATATGGAATTTTTCGGGC
------------GGGAACCTTACTGTT---GAT------------GGTAGTTCC------
GGCACCGCAGGTATATTTGCAACGTACCCTGCGGAATACGTCTCGGCTTCAGGTGGATTT
GGGGATCAAGTTATGGGAACCATGCTTCTCCTGCTCTGTATTTGCGCCATTGTTGACAAG
AAAAACATGTCGGTTCCG------------AGCGGATTGGTTCCGTTATACATTGGTTTC
GTTATTTTGGCCATCGGAGTGTGTTTTGGCTCCAACTGTGGTTATGCTATTAATCCAGCC
AGGGATCTAGCGCCCCGTTTCCTTACCTTCATTGCCGGATGGGGCACTGAAGTGTTCAGG
TATCGGGGCTACAACTGGTTTTGGGTGCCTATCGTTGGCCCGCATATTGGCGCCATCTTG
GGAGTGCTCATATAC---ACTCTGTGCATCGAACTGCATTGGCCGGAAACACCTGAAATT
---CCTGCTGGC---AAT---------------------------------GTCGGA---
------AAGGACGATAAGAATGTTCGTGTTGTTGAATTAGAAAGACTC------------
------------------------------------------------------------
------------------------------------------------------------
------------------------------------------------------------
------------------------------------------------------------
------------------------------------------------------------
------------------------------------------------------------
------------------------------------------------------------
------------------------------------------------------------
------------------------------------------------------------
------------------------------------------------------------
------------------------------------------------------------
------------------------------------------------------------
------------------------------------------------------------
------------------------------------------------------------
------------------------------------------------------------
------------------------------------------------------------
------------------------------------------------------------
------------------------------------------------------------
------------------------------------------------------------
------------------------------------------------------------
------------------------------------------------------------
------

>GEHY01004014_Triops_newberryi_Glp
------------------------------------------------------------
------------------------------------------------------------
------------ATG------GAG------------------------CTT---TTCCAG
CGTTACCAT---ATAAAACACCCCTTAATCCGGGAAATGTTAGCGGAATTCCTGGGTACC
TTTTTACTTGTGCTATTCGGTGATGCCAGTGTAGCTCAGTCGAAGCTCACTAACAACGCG
AACGGTGATTTCTTTTCGATTAACTGGGGATGGGGTTTGGGAGTAATGATGGGAATTCTG
GTAGCCGGTAACATCTCAGGTGCACATTTGAATCCTGCCGTTTCACTTGCACTTGCCATG
TTCAACAAGTTCTCCTGGATGAAATTACTGCCGTATATTTTCGCTCAGTACTTGGGGGCA
TTAGCGGCTGCTGCTTGCGTTTTGGGAGTCTATTCAGAAGCCATATGGGACTTCTCGAAT
------------GGTAACCTAACGGTT---GAC------------GGTAGTACT------
GGCACAGCGGGCATCTTCTCCACGTACCCTGCCGAATACGTTTCAGCAGCAGGAGGTTTC
GGCGATCAGGTTATGGGAACGATGTTGCTCCTGCTTTGCATTTGTGCCATTGTTGATAAG
AAGAACGCGAACATCCCG------------AGCTCGCTGGTGCCATTGTACATCGGTTTC
GTGATATTAGCTATCGGAGTTTGCTTCGGCTCCAACTGTGGTTACGCTATTAACCCTGCC
AGAGATTTGGCCCCGCGGTTCTTGACGTATATCGGTGGATGGGGTACTGGCGTTTTCAGT
TACCGGAATTACAACTGGTTCTGGGTTCCTATTATTGGCCCACATATTGGCGCCATTTTG
GGGGTACTCATCTAC---ACCGTATGCATCGAATTACACTGGCCGGAAACTTTCGAACTT
---GGT---GGT---GTG---------------------------------CAAGCA---
------GTTGACGATAAACGCACCCGAGTTGTTGAGCTGGATAGGCTT------------
------------------------------------------------------------
------------------------------------------------------------
------------------------------------------------------------
------------------------------------------------------------
------------------------------------------------------------
------------------------------------------------------------
------------------------------------------------------------
------------------------------------------------------------
------------------------------------------------------------
------------------------------------------------------------
------------------------------------------------------------
------------------------------------------------------------
------------------------------------------------------------
------------------------------------------------------------
------------------------------------------------------------
------------------------------------------------------------
------------------------------------------------------------
------------------------------------------------------------
------------------------------------------------------------
------------------------------------------------------------
------------------------------------------------------------
------
>BAYF01005257_Triops_cancriformis_Glp
------------------------------------------------------------
------------------------------------------------------------
------------ATG------GAA------------------------CTC---TTCCAG
CGTTACCAT---ATAAGAAATGCCTTGATCCGGGAAATGTTAGCGGAATTCCTGGGTACA
TTTTTACTTGTGCTATTCGGAGATGCCAGTGTGGCGCAGTCGAAGCTCACCAACAACGTG

AACGGCGATTTCTTTTCAATTAATTGGGGATGGGGTTTGGGAGTAATGATGGGGATTTTG
GTAGCTGGTAACATCTCAGGTGCACATTTGAATCCTGCCGTTTCCCTCGCACTTGCAATG
TTCAACAAGTTCTCTTGGATGAAATTACTACCGTATATTTTTGCTCAATACTTGGGGGCG
TTGGCAGCTGCCGCATGCGTTTTAGGAGTTTATTCAGAAGCCATATGGGAGTTTTCGAAC
------------GGTAACCTAACGGTT---GAT------------GGTAGTACT------
GGCACAGCGGGCATATTCGCTACGTACCCTGCCGAGTACGTTTCAGCAGCGGGTGGTTTT
GGAGATCAAGTAATGGGAACGATGTTGCTTCTGCTTTGCATTTGTGCCATTGTTGATAAG
AAGAACGCGAACATCCCA------------AGTTCTCTGGTCCCCTTGTACATCGGTTTC
GTGATCTTGGCTATTGGAGTTTGTTTCGGCGCAAACTGTGGTTACGCCATTAACCCTGCC
CGAGATTTGGCCCCGCGGTTCTTGACCTTTATCGGTGGATGGGGTACTGACGTTTTCAGG
TACCGGAATTACAACTGGTTTTGGGTACCTATTATTGGGCCACATATTGGTGCCATTTTG
GGAGTTCTGATCTAC---ACCGTTTGCATCGAATTGCACTGGCCAGAAACTTACGAACTT
---GGT---GGT---GTA---------------------------------GCAACT---
------GTTGACGATAAACGGAACCGTGTGGTTGAGCTGGATAGAATT------------
------------------------------------------------------------
------------------------------------------------------------
------------------------------------------------------------
------------------------------------------------------------
------------------------------------------------------------
------------------------------------------------------------
------------------------------------------------------------
------------------------------------------------------------
------------------------------------------------------------
------------------------------------------------------------
------------------------------------------------------------
------------------------------------------------------------
------------------------------------------------------------
------------------------------------------------------------
------------------------------------------------------------
------------------------------------------------------------
------------------------------------------------------------
------------------------------------------------------------
------------------------------------------------------------
------------------------------------------------------------
------------------------------------------------------------
------
>JAIQCU010000658_Leptestheria_dahalacensis_Glp3Rp
------------------------------------------------------------
------------------------------------------------------------
------------------------------------------------------------
------------------------------------------------------------
------------------------------------------------------------
------------------------------------------------------------
------------------------------------------------------------
------------------------------------------------------------
------------------------------------------------------------
------------------------------------------------------------
------------------------------------------------------------

---------ATTATTGTTATGCTGATGCCCCTGCTTTGCATCTGCGCCATCACGGACAAG
AAAAACAGCAAGGTCCCA------------TCATCGCTGGTCACGCTGTACATTGGCTTT
CTCACCCTGGTCATTGGTATCTGTTTCGGAACCAACTGCGGTTACGCAATCAACCCGGCC
AGAGATTTGGCGCCAAGACTCATCACCCTGTTTGCCGGTTGGGGTGAGGGTGTGTTCAGG
TTCCGCAACTATAATTGGTTCTGGGTACCCATTATTGGACCTCATATCAGAGCCCTTTTA
GGAGTTCTCGTCTAC---ATCCTGTTCATTGAAGCCCACTGGCCCGAAGAGGAGGGCGAC
---GATGCCGTT---CGTAATGGCGAT---GAATCCAGTTCT----------GCTTTA---
------GGGAAGGCCGTGAAGGAAAGTACCATTGATACGGAA---GGT------------
------------------------------------------------------------
------------------------------------------------------------
------------------------------------------------------------
------------------------------------------------------------
------------------------------------------------------------
------------------------------------------------------------
------------------------------------------------------------
------------------------------------------------------------
------------------------------------------------------------
------------------------------------------------------------
------------------------------------------------------------
------------------------------------------------------------
------------------------------------------------------------
------------------------------------------------------------
------------------------------------------------------------
------------------------------------------------------------
------------------------------------------------------------
------------------------------------------------------------
------------------------------------------------------------
------------------------------------------------------------
------------------------------------------------------------
------
>JAIQCU010000658_Leptestheria_dahalacensis_GlpA2b
------------------------------------------------------------
------------------------------------------------------------
------------ATGGCG---ACCCGG---------------------TTG---ATTTCT
AAACTACGT---GTCGATGTTCCTATTCTGAGGGAGATTATGGCCGAGTTTCTGGGAACC
TACGTTTTGGTGCTATTGGGTGATGCCAGTATCGCCCAATCGAGTCTAACCGACTCGAAA
AATGGCGACTTTTTCTCCATCAACTGGGGCTGGGGTATAGGAGTCATGATGGGAGCCCTC
ATCGCCGGTAGAGTATCAGGGGGCCATTTGAACCCGGCAGTGAGCCTGGCAATGGCTAGC
GTTGGCAAGTTCCCTTTCGTCAAGGTCCCAGCTTACTGGTTAGCGCAGTACCTCGGTGCC
TTGGCTGCAGCTGCATCGGTACTCGGAGTCTACAGCGAGGCGATTCAGTACAAAACCAAC
------------GGTGACCTGACTATCACTAAT------------GACAACCCG------
GGCACTGCCGGTATCTTTGCCACGTATCCCGCTGTTTACCTATCGGCTCAGGGTGGATTA
GGAGATCAGATACTTGGTACCATGATGCTCCTGCTCTGCATCTGCGCCATCACGGACAAG
AAAAACAGCAATGTCCCA------------TCATCGCTGGTCCCCATGTACATCGGCTTC
GTCATCCTGGTCATTGGTATCTGTTTCGGAGCCAACTGCGGTTACGCAATCAACCCGGCC
AGAGATTTGGCGCCAAGACTCATCACCCTGTTTGCCGGTTGGGGTGAGGGTGTGTTCAGG
TTCCGCAACTATAATTGGTTCTGGGTACCCATTATTGGACCTCATATCGGAGCCCTTTTG
GGAGTTCTCATCTAC---ATCCTGTTCATTGAAGCCCACTGGCCCGAAGAGGAGGACGAC

---GATGCCGGT---CGTAATGGCGAT---GGATCTAGTTCT---------GCTGTA---
------AGAAAGTCCGAGAAGGAAAGTACCATTGAAATGGAA---GGT------------
------------------------------------------------------------
------------------------------------------------------------
------------------------------------------------------------
------------------------------------------------------------
------------------------------------------------------------
------------------------------------------------------------
------------------------------------------------------------
------------------------------------------------------------
------------------------------------------------------------
------------------------------------------------------------
------------------------------------------------------------
------------------------------------------------------------
------------------------------------------------------------
------------------------------------------------------------
------------------------------------------------------------
------------------------------------------------------------
------------------------------------------------------------
------------------------------------------------------------
------------------------------------------------------------
------------------------------------------------------------
------------------------------------------------------------
------
>NKDA01000002_Eulimnadia_texana_GlpA2b
------------------------------------------------------------
------------------------------------------------------------
------------ATGGCG---CCAAAA---------------------CTG---ATGTCC
AAACTACGG---ATTGACGTACCCATTCTCCGAGAAATTTTGGCCGAATTTCTGGGAACT
TACATTCTTGTGCTATTCGGAGATGCCAGTGTTGCTCAATCGAACTTAACGGACTCGAAG
AATGGCGACTTCTTCTCCATCAACTGGGGCTGGGGCCTGGGTGTCATGTTCGGAGTCATG
GTCGCTGGCGGAGTCTCCGGTGGTCACTTGAATCCTGCAGTGAGTCTGGCAATGGCCTCT
GTAGGAAAGTTTCCGTTTATCAAAGTTCCTGCTTATTGGTTAGCTCAGTACCTTGGCGCT
TTAGCAGCTGCTGCTTCAGTCCTGGGAGTCTACAGCGAGGCAATTCAGCACAAGACTGGC
------------GGGAACTTGTCTCTAACTAAC------------GAAGATCCT------
GGAACTGCTGGAATTTTTGCCACTTATCCTGCGGTGTACCTGTCAGCACAAGGAGGCCTT
GGAGATCAAATCCTGGGTACCATGATGCTACTCCTCTGTATCTGCGCCATAACCGACAAG
CGTAACAACAAGGTACCT------------TCACATCTAGCACCTCTCTACGTGGGCTTC
ACCATCTTAGCCATCGGTGCTACCCTGGGCGCAAACTGCGGCTACGCCATCAACCCGGCC
CGCGACTTAGCGCCTAGACTCATCACCCTCTTTGCTGGCTGGGGAACTGAAGTCTTCAGG
TTTCGGGATTATAACTGGTTCTGGGTGCCGATTATTGGTCCCCATATTGGAGCCCTGTTA
GGGGTTGTTGTCTAC---ATCCTGTTCATTGAAGCTCACTGGCCTGAGGAGGAAGAGGAA
---GAA------------------GAG---CAGACGACGCCG---------CGCGCC---
------TCTGTCAACTACAAGGAAAACACCATTGAGATGCAG---GGTAATTTAAATTTC
CAATTGACT---------------------------------------------------
------------------------------------------------------------
------------------------------------------------------------
------------------------------------------------------------

------------------------------------------------------------
------------------------------------------------------------
------------------------------------------------------------
------------------------------------------------------------
------------------------------------------------------------
------------------------------------------------------------
------------------------------------------------------------
------------------------------------------------------------
------------------------------------------------------------
------------------------------------------------------------
------------------------------------------------------------
------------------------------------------------------------
------------------------------------------------------------
------------------------------------------------------------
------------------------------------------------------------
------------------------------------------------------------
------------------------------------------------------------
------
>NKDA01000001_Eulimnadia_texana_GlpA2a
------------------------------------------------------------
------------------------------------------------------------
------------ATGGCG---TCGACT---------------------CTG---GTCTCC
AAGATTCGC---GTGGATTTGCCGATCCTCAGAGAAATTATGGCGGAATTCCTGGGAACT
TTCATCCTAGTGCTCTTCGGAATCGGCAGTGTAGCACAGGCTGTGCTAACTAGTTCGGAG
AAAGGCAATTTCTTCGCCATCAACTGGGGCTGGGGCTTCGGCGTCATGATGGGCGTCCTA
GTCGCAGGCGGAGTTTCAGGAGCTCATTTGAACCCGGCAGTGACGCTTGCTATGGCCGCC
ATCGGAAAGTTTCCGTTTATTAAAGTGCTACCTTACTGGCTAGCACAATATTTGGGATCC
TTAGCTGCTGCTGCGTCCGTGCTAGGAGTATATAGTGAGGCTCTTCAGCACAAGACTGGC
------------GGTGAACAGACGATAACAAAC------------CAAGATCCC------
GGCACTGCAGGAATTTTCGCTACTTACCCTGCGCCATATTTGTCAATCCTAGGCGGTCTA
GGAGACCAGATTTTAGGGACTATGATTCTTCTCATTTGCATTTGCGCCATCACCGACAAG
CGGAACAGCAAAGTCCCC------------ACATCCCTGGTGCCGATGTACGTGGGATTC
GTCGTCGTAGTCATCGGTATCTGCTTCGGAGCAAACTGCGGATACGCACTGAATCCAGCG
CGCGATTTAGCGCCCAGGATCCTTACTTGGGTCTCTGGATGGGGCGGTGAAGTCTTCAGG
TTCCGCAATTATAACTGGTTCTGGGTCCCGGTTCTGGGTCCGCATATCGGAGCTCTGCTC
GGAGTTTTCGTCTAC---GCCATTTTCATCGAAGCTCACTGGCCGGAAAACGACGACCAT
---ATCATCCCA---CTATCGATT------CAGAGCAGCGAT---------AATCGA---
------TACGAATCGAAATCGGAAAAACATATCGAAATGGAGTGTAAGATTCTTAATAAA
TTATTTGATTTTTTCCACTGTTTAATTGAAAAATTTCTGCAATTTTCAGATTTG------
------------------------------------------------------------
------------------------------------------------------------
------------------------------------------------------------
------------------------------------------------------------
------------------------------------------------------------
------------------------------------------------------------
------------------------------------------------------------
------------------------------------------------------------
------------------------------------------------------------

------------------------------------------------------------
------------------------------------------------------------
------------------------------------------------------------
------------------------------------------------------------
------------------------------------------------------------
------------------------------------------------------------
------------------------------------------------------------
------------------------------------------------------------
------------------------------------------------------------
------------------------------------------------------------
------------------------------------------------------------
------
>JAIQCU010001241_Leptestheria_dahalacensis_Glp2a
------------------------------------------------------------
------------------------------------------------------------
------------ATGGCG---CCATCG---------------------CTG---AGCAAA
AAAATCCGA---ATCAGGATGCCCATTTTGAGAGAAATCATGGCCGAGTTTCTGGGCACT
TTCATTCTCGTGTTGCTAGGCAACGCGTGTGGCACAATCCGTTTTGTCTAAATCCGTC
AAAGGTGATTTTTTTGCCATCAATTGGGGCTGGGGCTTCGGCGCCATGATGGGGGTCATC
ATAGCAGGAGGAGTTTCAGGCGCCCATTTAAATCCAGCTGTCACTCTGGCTCTGGCTTCC
GTGAGGCGATTCCCGTTCCTCAAGATCTTCGCTTACTGGGCGGCGCAGTACCTCGGGGCT
TTGGCTGCAGCTGCGTCTGTCCTTGGGGTCTATAGTGAGGCCCTACAGCACTCGACTGGT
------------GGGAATTTGACTGTCTCGGAC------------CAAGACCCG------
GGTACCGCGGGAATCTTTGCGACATACCCCGAGTCGTACCTTTCTGCTGAAGGCGGGCTT
GGAGATCAAATTCTGGGTTCGATGTTCCTGCTATTATGCATTTGTGCCATCACCGACAAG
AAGAACACCCACGTTCCT------------GCCCCGCTGGTCCCACTGTATATCGGTTTC
GCCATCGTTGCTATCGGTGTTTGCTTCGGTGCTAATTGTGGTTACGCAGTGAATCCAGCT
CGAGATCTGGCACCCAGGATTCTTACTTTGCTAGCGGGATGGGGATCCGGAGTGTTCAGG
TTCCGCAACTACAACTGGTTCTGGGTCCCTATAGTCGGTCCTCACATTGGAGCAATCCTG
GGAGTCCTTGTTTAC---ATCGTTTTCATCGAAGCTCACTGGCCGGAAGGCGAGCAGCCC
ACCATTTTCCCG---CTGTCCATC---CGG---------------------CATCGT---
------AAAGACGACAGGCATAAGGAAGCCATTGATATGGAGAGTACG------------
------------------------------------------------------------
------------------------------------------------------------
------------------------------------------------------------
------------------------------------------------------------
------------------------------------------------------------
------------------------------------------------------------
------------------------------------------------------------
------------------------------------------------------------
------------------------------------------------------------
------------------------------------------------------------
------------------------------------------------------------
------------------------------------------------------------
------------------------------------------------------------
------------------------------------------------------------
------------------------------------------------------------
------------------------------------------------------------

------------------------------------------------------------
------------------------------------------------------------
------------------------------------------------------------
------------------------------------------------------------
------------------------------------------------------------
------
>HAFN01014029_Daphnia_galeata_GlpA2
------------------------------------------------------------
------------------------------------------------------------
------------ATGGCT---CCCAAA---------------------CTT---ATGAAA
ATGATACGT---GTCGAGGCGCCACTTCTCCGCGAGATTATGGCTGAATTCCTGGGCACT
TTCATTCTTGTGCTTTTTGGTGACGCCAGTGTAGCCCAGTCTAAATTATCCAACGAGGCC
AATGGAGACTTCTTCTCCATCAACTGGGGCTGGGCCGTCGGTGTTATGATGGGGGTCTTG
GTTGCTGGGGGCGTATCGGGTGCCCATTTAAATCCGGCTGTCACGCTTGCAATGGCTTGT
GCTGGTCGTATTGCACTCATCAAAGTTTTTTTCTACTGGCTCGCCCAATATTTGGGCGCA
TTCGCCGCTGCTGCCTCCGTCCTCGGAGTCTATAGCGATGCAATCCAATATGCCAGCAAC
------------GGGACTTTGACTATGAACAAT------GAATTGAACGATCCG------
GGCTTGGCTGGCATCTTTGCTACTTATCCGGCCCATTGGCTCTCCGTTGCTGGAGGGCTC
GGCGACCAGATTTTGGGAACCATGCTGTTACTCCTCTGCATTTGCGCCATCACGGACAAG
AAAAACACGCAAATTCCG------------TCGGCTCTAGTGCCAATGTACGTCGGATTC
ACCATTCTGGGTATCGGCGTTTGCTTTGGGGCCAACTGTGGCTACGCACTTAACCCAGCA
AGAGATCTTTCACCTCGTCTCATCAGTCTCATCGCTGGATGGGAC---CAATCTTTCAGC
TGGAACAATTACAACTGGTTCTGGATACCTATCGTTGGTCCTCATATTGGAGCTATCCTC
GGGGTCTTTATTTAC---ATCGCATTTGTCGAAGCCCACTGGCCGGAGGATGGCGACGAA
---GTGGTGCCA---ATGACCGTTCCACGTGCGAATTTCAAC------------------
---AAAGAGCGAGGAGAAAAGATTCAGAACATGGACATGGAGAGTTTTACCACGTCAAAT
------------------------------------------------------------
------------------------------------------------------------
------------------------------------------------------------
------------------------------------------------------------
------------------------------------------------------------
------------------------------------------------------------
------------------------------------------------------------
------------------------------------------------------------
------------------------------------------------------------
------------------------------------------------------------
------------------------------------------------------------
------------------------------------------------------------
------------------------------------------------------------
------------------------------------------------------------
------------------------------------------------------------
------------------------------------------------------------
------------------------------------------------------------
------------------------------------------------------------
------------------------------------------------------------
------------------------------------------------------------
------------------------------------------------------------
------

```
>JAAVJA010004895_Daphnia_dubai_GlpA2
------------------------------------------------------------
------------------------------------------------------------
------------ATGGCC---CCCAAA---------------------CTT---ATGAAG
ATGATACGT---GTCGAGGCGCCACTTCTCCGCGAGATTATGGCAGAATTCCTGGGCACT
TTCATTCTTGTGCTTTTTGGTGACGCCAGTGTGGCCCAGTCTAAACTATCCAACGAAGCC
AATGGAGATTTCTTCTCCATCAACTGGGGTTGGGCTGTTGGTGTCATGATGGGGGTCTTG
GTTGCTGGGGGCGTATCGGGTGCCCATTTAAATCCGGCTGTCACGTTGGCCATGGCTTGT
GCTGGTCGTATTGCACTCATCAAAGTTTTCTTCTACTGGCTCGCACAGTATTTGGGAGCA
TTCGCCGCTGCTGCCTCCGTCCTCGGAGTTTACAGCGATGCAATCCAATATGTTAGCAAC
------------GGGACTTTGACTATGAACAAT------GATTTGAACGATCCG------
GGCTTGGCTGGTATCTTTGCTACTTATCCGGCCCCTTGGCTCACCATTGCTGGAGGGCTT
GGCGACCAGGTG---------ATGCTATTACTCCTCTGCATTTGCGCCATCACGGACAAG
AAAAACACGCAAATTCCG------------TCCGCTCTAGTGCCAATGTACGTCGGATTC
ACCATTCTGGGCATCGGCGTTTGCTTTGGAGCCAACTGCGGCTACGCA------------
---------------------------------------------------------AGT
TGGAATAATTTCAACTGGTTCTGGATACCCATCGTTGGTCCTCACATCGGAGCTATCCTC
GGGGTCTTTATTTAC---ATCGCCTTTGTCGAAGCCCATTGGCCAGAGGATGGCGACGAA
---GTGGTGCCA---ATGACCGTTCCAAGAGCAAATTTCAAC------------------
------------------------------------------------------------
------------------------------------------------------------
------------------------------------------------------------
------------------------------------------------------------
------------------------------------------------------------
------------------------------------------------------------
------------------------------------------------------------
------------------------------------------------------------
------------------------------------------------------------
------------------------------------------------------------
------------------------------------------------------------
------------------------------------------------------------
------------------------------------------------------------
------------------------------------------------------------
------------------------------------------------------------
------------------------------------------------------------
------------------------------------------------------------
------------------------------------------------------------
------------------------------------------------------------
------------------------------------------------------------
------------------------------------------------------------
------------------------------------------------------------
------
>EFX88757_Daphnia_pulex_GlpA2
------------------------------------------------------------
------------------------------------------------------------
------------ATGGCC---CCAAAA---------------------CTT---ATGAAA
ATGATACGA---GTCGAGGCGCCACTTCTCCGTGAGATTATGGCGGAATTCCTGGGCACT
TTCATTCTTGTGCTTTTTGGTGACGCCAGCGTCGCCCAGTCTAAACTTTCAAACGAAGCC
```

AATGGAGACTTTTTCTCCATCAACTGGGGCTGGGCTGTCGGTGTCATGATGGGGGTCTTG
GTTGCTGGGGGCGTATCGGGTGCTCATTTAAATCCGGCCGTCACGTTGGCAATGGCCTGC
GCTGGTCGTATTGCCATCATCAAAGTTTTCTTCTACATGCTGGCCCAGTATCTGGGCGCC
TTCGCCGCAGCCGCCTCCGTCCTCGGAGTCTACAGCGATGCCATCGAATATTACAGCAAC
ACCAACCACAACGGGACTTTGTACATGAACAAT------GAAGCCAACGATCCG------
GGCTTGGCCGGCATTTTTTCCACCTATCCGGCCCCTTGGCTCTCCATTGCTGGAGGGATT
GGCGACCAGATTTTGGGAACCATGCTGCTACTCCTCTGCATCTGCGCCATCACGGACAAG
AAAAACACGCAAATTCCG------------TCGGCCCTAGTGCCAATGTACGTGGGATTC
ACCATTCTGGGCATCGGCGTTTGCTTCGGGGCCAACTGCGGCTACGCGCTCAATCCGGCC
AGAGATCTTTCGCCCCGTCTCATCACTCTCATCGCCGGATGGGAC---CAATCTTTCAGC
TGGAACAATTACAACTGGTTCTGGATACCCATCGTCGGCCCACATATCGGAGCTATCCTC
GGAGTCTTCATTTAC---ATCGCCTTTGTCGAAGCCCATTGGCCGGAGGAAGGCGACGAA
---GTGGTGCCC---ATCACCGTTCCACGAGCGAATTTCAAC------------------
---AAAGAGCGAGGAGAAAAGATCCAGAACATGGACATGGAGAGTTTC------------
------------------------------------------------------------
------------------------------------------------------------
------------------------------------------------------------
------------------------------------------------------------
------------------------------------------------------------
------------------------------------------------------------
------------------------------------------------------------
------------------------------------------------------------
------------------------------------------------------------
------------------------------------------------------------
------------------------------------------------------------
------------------------------------------------------------
------------------------------------------------------------
------------------------------------------------------------
------------------------------------------------------------
------------------------------------------------------------
------------------------------------------------------------
------------------------------------------------------------
------------------------------------------------------------
------------------------------------------------------------
------------------------------------------------------------
------
>XM_046788163_Daphnia_pulicaria_GlpA2
------------------------------------------------------------
------------------------------------------------------------
------------ATGGCC---CCAAAA---------------------CTT---ATGAAA
ATGATACGT---GTCGAGGCGCCACTTCTCCGCGAGATTATGGCGGAATTCCTGGGCACT
TTCATTCTTGTGCTTTTTGGTGACGCCAGCGTCGCCCAGTCTAAACTTTCAAACGAAGCC
AATGGAGACTTTTTCTCCATCAACTGGGGCTGGGCTGTTGGTGTCATGATGGGGGTCTTG
GTTGCTGGGGGCGTATCGGGTGCTCATTTAAATCCGGCCGTCACCTTGGCAATGGCCTGC
GCTGGTCGTATTGCCATCATCAAAGTTTTCTTCTACATGCTGGCCCAGTATCTGGGCGCC
TTCGCTGCAGCCGCCTCCGTCCTCGGAGTCTACAGCGATGCCATCGAATATTACAGCAAC
GCCAACCACAACGGGACTTTGTACATGAACAAT------GAAGCCAACGATCCG------
GGCTTGGCCGGCATCTTTGCCACCTATCCGGCCCCTTGGCTCTCCATTGCTGGAGGGATT

GGCGACCAGATTTTGGGAACCATGCTGCTACTCCTCTGCATCTGCGCCATCACGGACAAG
AAAAACACGCAAATTCCG------------TCAGCCCTAGTGCCAATGTACGTGGGATTC
ACCATTCTGGGCATCGGCGTTTGCTTCGGGGCCAACTGCGGCTACGCGCTCAATCCGGCC
AGAGATCTTTCGCCCCGTCTCATCACTCTCATCGCCGGATGGGAT---CAATCTTTCAGC
TGGAACAATTACAACTGGTTCTGGATACCCATCGTCGGCCCTCACATCGGAGCTATCCTC
GGGGTCTTCATTTAC---ATCGCCTTTGTCGAAGCCCATTGGCCGGAGGAAGGCGACGAA
---GTGGTGCCC---ATCACCGTTCCACGAGCGAATTTCAAC------------------
---AAAGAGCGAGGAGAAAAGATCCAGAACATGGACATGGAGAGTTTTACCACGTCAAAT
------------------------------------------------------------
------------------------------------------------------------
------------------------------------------------------------
------------------------------------------------------------
------------------------------------------------------------
------------------------------------------------------------
------------------------------------------------------------
------------------------------------------------------------
------------------------------------------------------------
------------------------------------------------------------
------------------------------------------------------------
------------------------------------------------------------
------------------------------------------------------------
------------------------------------------------------------
------------------------------------------------------------
------------------------------------------------------------
------------------------------------------------------------
------------------------------------------------------------
------------------------------------------------------------
------------------------------------------------------------
------------------------------------------------------------
------
>CM023188_Daphnia_carinata_GlpA2
------------------------------------------------------------
------------------------------------------------------------
------------ATGGCC---CCAAAG---------------------CTT---ATGAAG
ATGATACGT---GTCGAGGCTCCACTCCTTCGCGAGATCATGGCTGAATTCCTGGGCACT
TTTATTCTAGTGCTGTTTGGCGACGCCAGTGTAGCCCAATCGAAACTATCAAATGAAGCC
AATGGTGACTTCTTCTCCATAAACTGGGGCTGGGCAATTGGTGTCATGATGGGAGTCTTG
GTTGCTGGGGGCGTCTCGGGTGCCCATTTGAATCCTGCTGTCACGTTGGCAATGGCTTGT
GCTGGTCGCCTAGCAATCATCAAAGTTTTCTTTTACATGCTGGCCCAGTATTTGGGAGCG
TTCGCTGCCGCCGCCTCCGTCCTTGGCGTCTACAGCGATGCGATCCAGTATGCAAGCAAC
------------GGGACTTTGACTATGGACGAT------GAGGCAAACGATCCA------
GGACTGGCTGGCATTTTTGCCACTTATCCAGCCCCATGGCTCTCAGTTGCTGGAGGGCTT
GGCGACCAGATATTGGGCACCATGTTGTTATTGCTCTGCATCTGCGCCATTACGGACAAA
AAGAACACGCAAATCCCG------------TCCGCTTTAGTGCCCATGTACGTAGGATTC
ACCATTCTGGGCATCGGTGTTTGCTTTGGTGCTAATTGCGGCTATGCGCTCAATCCGGCC
AGAGATCTTTCTCCCCGACTCATCAGCCTCATCGCCGGATGGGAA---CAATCTTTCAGC
TGGGACAACTACAACTGGTTCTGGATTCCTATTGTTGGCCCTCATATCGGAGCTATCCTT
GGAGTCTTTATTTAC---ATCGCATTCGTTGAAGCTCACTGGCCTGAGGATGGAGATGAA

---CTGGTACCG---GTGAACGTGCCACGAGGAAATTTCAAC------------------
---AAAGACAGGGGTGAGAAGATCCAGAACATGGATATGGAGAGTAAGAGTCCTAGATTT
TATATTGAAATGAGTATCCTGCCCACTTTGAGC---------------------------
------------------------------------------------------------
------------------------------------------------------------
------------------------------------------------------------
------------------------------------------------------------
------------------------------------------------------------
------------------------------------------------------------
------------------------------------------------------------
------------------------------------------------------------
------------------------------------------------------------
------------------------------------------------------------
------------------------------------------------------------
------------------------------------------------------------
------------------------------------------------------------
------------------------------------------------------------
------------------------------------------------------------
------------------------------------------------------------
------------------------------------------------------------
------------------------------------------------------------
------------------------------------------------------------
------------------------------------------------------------
------
>KAI9564578_Daphnia_sinensis_GlpA2
------------------------------------------------------------
------------------------------------------------------------
------------ATGGCC---CCAAAG---------------------CTT---ATGAAG
ATGATACGT---GTCGAGGCTCCACTCCTTCGCGAGATCATGGCTGAATTCCTGGGCACT
TTTATTCTAGTGCTGTTTGGCGACGCCAGTGTAGCCCAATCGAAACTATCAAATGAAGCC
AATGGTGACTTCTTCTCCATAAACTGGGGCTGGGCAATTGGTGTCATGATGGGAGTCTTG
GTTGCTGGGGGCGTCTCGGGTGCCCATTTGAATCCTGCTGTCACGTTGGCAATGGCTTGT
GCTGGTCGCCTAGCAATCATCAAAGTTTCTTTTACATGCTGGCCCAGTATTTGGGAGCG
TTCGCTGCCGCCGCCTCCGTCCTTGGCGTCTACAGCGATGCGATCCAGTATGCAAGCAAC
------------GGGACTTTGACTATGGACGAT------GAGGCAAACGATCCA------
GGACTGGCTGGCATTTTTGCCACTTATCCAGCCCCATGGCTCTCAGTTGCTGGAGGGCTT
GGCGACCAGATATTGGGCACCATGTTGTTATTGCTCTGCATCTGCGCCATTACGGACAAA
AAGAACACGCAAATCCCG------------TCCGCTTTAGTGCCCATGTACGTAGGATTC
ACCATTCTGGGCATCGGTGTTTGCTTTGGTGCTAATTGCGGCTATGCGCTCAATCCGGCC
AGAGATCTTTCTCCCCGACTCATCAGCCTCATCGCCGGATGGGAA---CAATCTTTCAGC
TGGGACAACTACAACTGGTTCTGGATTCCTATTGTTGGCCCTCATATCGGAGCTATCCTT
GGAGTCTTTATTTAC---ATCGCATTCGTTGAAGCTCACTGGCCTGAGGATGGAGATGAA
---CTGGTACCG---GTGAACGTGCCACGAGGAAATTTCAAC------------------
---AAAGACAGGGGTGAGAAGATCCAGAACATGGATATGGAGAGTTTCACCGCGTCGACA
------------------------------------------------------------
------------------------------------------------------------
------------------------------------------------------------
------------------------------------------------------------

------------------------------------------------------------
------------------------------------------------------------
------------------------------------------------------------
------------------------------------------------------------
------------------------------------------------------------
------------------------------------------------------------
------------------------------------------------------------
------------------------------------------------------------
------------------------------------------------------------
------------------------------------------------------------
------------------------------------------------------------
------------------------------------------------------------
------------------------------------------------------------
------------------------------------------------------------
------------------------------------------------------------
------------------------------------------------------------
------------------------------------------------------------
------
>NC_046175_Daphnia_magna_GlpA2
------------------------------------------------------------
------------------------------------------------------------
------------ATGGCC---CCAAAG---------------------CTT---ATGAAG
ATGATACGT---GTCGAGGCTCCACTCCTTCGCGAGATCATGGCTGAATTCCTGGGCACT
TTTATTCTAGTGCTGTTTGGCGACGCCAGTGTAGCCCAATCGAAACTATCAAACGAAGCC
AATGGTGACTTCTTCTCCATAAACTGGGGTTGGGCAATTGGTGTCATGATGGGAGTCTTG
GTTGCTGGGGGCGTCTCGGGTGCCCATTTGAATCCTGCTGTCACGTTGGCAATGGCTTGT
GCTGGTCGTCTAGCAATCATCAAAGTCTTCTTTTACATGCTGGCGCAATATTTGGGCGCG
TTCGCTGCAGCCGCGTCCGTCCTTGGCGTCTACAGCGATGCGATCCAGTATGCAAGCAAC
------------GGGACTTTGACTATGAACGAT------GAAGCAAACGATCCG------
GGACTGGCTGGCATTTTCGCTACTTATCCAGCCCCATGGCTCTCGGTTGCTGGAGGGCTT
GGCGACCAGATATTGGGCACCATGTTGTTATTGCTCTGCATCTGCGCCATTACGGACAAG
AAGAACACGCAAATCCCG------------TCCGCTTTAGTGCCCATGTACGTGGGATTC
ACCATTCTAGGCATCGGTGTTTGCTTTGGTGCTAATTGCGGCTATGCGCTCAATCCGGCC
AGAGATCTTTCCCCACGACTCATCAGTCTCATCGCCGGATGGGAA---CAGTCTTTCAGC
TGGGACAACTACAACTGGTTCTGGATTCCTATTGTTGGCCCTCATATCGGAGCTATTCTC
GGAGTCTTTATTTAC---ATCGCATTCGTTGAAGCGCACTGGCCAGAGGATGGAGATGAA
---CTGGTACCC---GTGGGCGTGCCACGAGCAAATTTCAAC------------------
---AAAGACAGGGGTGAGAAGATCCAGAACATGGATATGGAGAGTAAGAATCATAGATTT
ATGTTGCAATCGTTATCCTGCCCACTT---------------------------------
------------------------------------------------------------
------------------------------------------------------------
------------------------------------------------------------
------------------------------------------------------------
------------------------------------------------------------
------------------------------------------------------------
------------------------------------------------------------
------------------------------------------------------------
------------------------------------------------------------

------------------------------------------------------------
------------------------------------------------------------
------------------------------------------------------------
------------------------------------------------------------
------------------------------------------------------------
------------------------------------------------------------
------------------------------------------------------------
------------------------------------------------------------
------------------------------------------------------------
------------------------------------------------------------
------------------------------------------------------------
------
>JAKUCG010000105_Ceriodaphnia_dubia_GlpA2
------------------------------------------------------------
------------------------------------------------------------
------------ATGGCT---CCCAAG---------------------CTT---ATGAAG
ATGATACGC---GTCGAGGCCCCACTCCTTCGCGAGATTATGGCTGAATTCCTGGGCACC
TTCATTCTAGTGCTGTTTGGTGACGCATCTGTGGCCCAGTCGAAACTCTCAAACGGAGCT
AATGGCGAGTTCCTCTCTATCAACTGGGGATGGGCCGTTGGTGTCATGATGGGTGTCCTG
GTTGCCGGGGGCGTATCGGGTGCCCATTTGAATCCGGCTGTCACACTAGCCATGGCTTGC
GCCGGCCGGCTCGCCATCATCAAAGTCTTCTTTTACATGCTCGCCCAGTATTTGGGTGCC
TTCGCCGCGGCTGCCTCCGTTCTTGGAGTCTACAGCGATGCCATTCAGTATGTAAGCAAC
------------GGAACGTTGACAATGAATGAC------GCGGCCGACGATCCG------
GGATTGGCCGGTATCTTTGCCACGTATCCGGCACCTTGGCTTTCGGTAGCCGGAGCCCTT
GGCGACCAGATCTTGGGAACAATGTTGCTCCTTCTCTGCATCTGCGCTATTACGGACAAG
AGGAACACCCAAGTACCT------------TCCGCCTTGGTGCCCATGTACGTTGGATTC
GTCATTTTGGGCATCGGTGTTTGTTTCGGAGCCAACTGCGGCTACGCTCTCAATCCCGCA
AGAGATCTTTCACCTCGTATCATCAGTCTCATCGCTGGATGGGAT---CAATCCTTTAGG
TACGACAATTACAACTGGTTTTGGATTCCTATTGTTGGGCCTCACATTGGAGCCATTTTG
GGAGTCTACATCTAC---ATCTTATTTGTGGAGGCACACTGGCCGGAGGAAGGCGACGAA
---GTGGTGCCA---ATAACTGTGCCGCGAGCCAACTTCAAC------------------
---AAAGATCGGGGAGAAAAGATCCAAAATATGGACATGGAGAGTAAGAACGTAGATTCA
TATCGGAAACAA------------------------------------------------
------------------------------------------------------------
------------------------------------------------------------
------------------------------------------------------------
------------------------------------------------------------
------------------------------------------------------------
------------------------------------------------------------
------------------------------------------------------------
------------------------------------------------------------
------------------------------------------------------------
------------------------------------------------------------
------------------------------------------------------------
------------------------------------------------------------
------------------------------------------------------------
------------------------------------------------------------
------------------------------------------------------------

------------------------------------------------------------
------------------------------------------------------------
------------------------------------------------------------
------------------------------------------------------------
------------------------------------------------------------
------
>CM057928_Chydorus_sphaericus_GlpA2
------------------------------------------------------------
------------------------------------------------------------
------------ATGGCG---ACCAAA---------------------GTG---ATAAGG
GCTATCCGA---GTGGAGGCTCCTCTCCTCCGTGAAATCATGGCCGAATTCCTCGGCACA
TTCATCTTGGTGATTTTCGGTGATGGTTGCGTCGCCCAATCCAAACTGACTGGTGGCGCC
AATGGAGATTTCTTGTCCATCAACTGGGGCTGGGCTGTTGGAGTCATGATGGGCATCTTA
GTGGCCGGTGGAGTCTCAGGTGCTCACTTGAACCCCGCCGTTTCTTTGGCAATGGCCTGT
GTTGGAAGGCTCCCGTTTATCAAAGTGGCATTTTACTGGTTGGCGCAGTATCTGGGCGCA
TTTGCAGCTGCTGCCTGCGTCCTCGGTGTCTATAGCGAGGCTATTAACGAGGTGAGCAAC
------------GGTACTTTGACGATGAGCGAC------------GGTGATCCT------
GGATTGGCCGGCATCTTCGCCACTTATCCGGCTAACTATCTCTCAATCGGTGGGGGTCTG
GGCGACCAGATTTTGGGTACTATGCTCCTCCTGCTGTGTGTATGTGCAATTACAGACAAG
AAGAACACACAAGTACCC------------TCATCTCTTGTCCCTCTGTACGTGGGTTTC
ACCATTCTGGGTATCGGTGTCTGTTTCGGAGCCAACTGCGGATACGCCATCAATCCTGCC
CGAGATTTGGCGCCCAGGCTTCTCAGTCTCATCGCCGGATGGGAT---CAATCTTTCAGC
TACAACAATTTCGAATGGTTTTGGATTCCAATTGTCGGACCCCATATCGGTGCAATCCTT
GGAGTTATTATTTAC---ATCGCTTTCGTTGAAGCTCACTGGCCGGAAGACATGGATGAA
---GCGGTGCCC---ATGACCGTACCCAGAGTTAATTACAATAGC---------AAC---
---AAAGTTCAGAGCGAGAAAGGAAATAATATGGACATGGAGAGTATGACC---------
------------------------------------------------------------
------------------------------------------------------------
------------------------------------------------------------
------------------------------------------------------------
------------------------------------------------------------
------------------------------------------------------------
------------------------------------------------------------
------------------------------------------------------------
------------------------------------------------------------
------------------------------------------------------------
------------------------------------------------------------
------------------------------------------------------------
------------------------------------------------------------
------------------------------------------------------------
------------------------------------------------------------
------------------------------------------------------------
------------------------------------------------------------
------------------------------------------------------------
------------------------------------------------------------
------------------------------------------------------------
------------------------------------------------------------
------

>GKAM01014060_Podon_leuckartii_GlpA2a
------------------------------------------------------------
------------------------------------------------------------
------------ATGATA---TCGAAA---------------------ATC---ACGGCG
CTCGTCCGA---ATCGAGGCGCCTCTCCTCCGCGAGGTATTCGCCGAGTTTTTGGGTACC
TTCATTCTGGTGATTTTCGGAGATGCTGCAGTGGCTCAATCGAAACTCAGCGACTCGAAG
AATGGCGATTTTCTCTCCATCAACTGGGGCTGGGGAATTGGTCTCATAATGGGAGTCTTG
GTAGCTGGTGGAGTTTCAGGAGCTCATTTGAATCCGGCAGTTTCGCTGGCTTTTGCCGTC
GTCGGCAAATTCCCATTCAAGAAAGTTCTATTCTACTGGCTGGCCCAATATCTGGGAGCC
TTCGCAGCGGCCGCTTCTGTCTTGGGAGTTTACAGCGACGCTATTCAGCACTACGCGGCA
ACGCACTCTGACGGACAGTTGGTAATGAATGAC------ACAATGAACGATCCT------
GGACTGGCTGGAATCTTCGCTACTTATCCTGCTGCTTGGTTATCCATTCAAGGAGGAATG
GGCGACCAGATTTTAGCTACGATGCTTCTCTTGCTCTGTATCTGCGCCATCACCGATAAA
AGAAATACTCAAGTCCCT------------TCTTCGCTGGTACCGATGTACTTGGGATTC
TTACTCCTCGCCATCGGTGTCTGTTTCGGAGCCAATTGCGGCTATGCTTGCAATCCTGCT
CGTGATCTCGCACCCAGATTCCTCACTCTTATCGCCGGATGGGGAACACAAGTCTTCAGC
TACAAAGACTACGCCTGGTTTTGGGTACCGATCATCGGACCTCATATTGGAGCCATTTTC
GGTGTCATTATCTAC---ATGCTATTCATCGAAGTTCACTGGCCTGAACAAGCTGACGGA
---ATGCTTCCC---ATCACT------------TCTTCCCAGCAA---------------
---GATGACAAACGTTCCAAATCCGCCAGCGCGGATTTGACTAATCTC------------
------------------------------------------------------------
------------------------------------------------------------
------------------------------------------------------------
------------------------------------------------------------
------------------------------------------------------------
------------------------------------------------------------
------------------------------------------------------------
------------------------------------------------------------
------------------------------------------------------------
------------------------------------------------------------
------------------------------------------------------------
------------------------------------------------------------
------------------------------------------------------------
------------------------------------------------------------
------------------------------------------------------------
------------------------------------------------------------
------------------------------------------------------------
------------------------------------------------------------
------------------------------------------------------------
------------------------------------------------------------
------------------------------------------------------------
------
>GKAG01002845_Evadne_nordmanni_GlpA2a
------------------------------------------------------------
------------------------------------------------------------
------------ATGATA---TCGAAA---------------------ATC---ACGGCG
CTCGTCCGA---ATCGAGGCGCCTCTCCTCCGCGAGGTATTCGCCGAGTTTTTGGGTACC
TTCATTCTGGTGATTTTCGGAGATGCTGCAGTGGCTCAATCGAAACTCAGCGACTCGAAG

AATGGCGATTTTCTCTCCATCAACTGGGGCTGGGGAATTGGTCTCATAATGGGAGTCTTG
GTAGCTGGTGGAGTTTCAGGAGCTCATTTGAATCCGGCAGTTTCGCTGGCTTTTGCCGTC
GTCGGCAAATTCCCATTCAAGAAAGTTCTATTCTACTGGCTGGCCCAATATCTGGGAGCC
TTCGCAGCGGCCGCTTCTGTCTTGGGAGTTTACAGCGACGCTATTCAGCACTACGCGGCA
ACGCACTCTGACGGACAGTTGGTAATGAATGAC------ACAATGAACGATCCT------
GGACTGGCTGGAATCTTCGCTACTTATCCTGCTGCTTGGTTATCCATTCAAGGAGGAATG
GGCGACCAGATTTTAGCTACGATGCTTCTCTTGCTCTGTATCTGCGCCATCACCGATAAA
AGAAATACTCAAGTCCCT------------TCTTCGCTGGTACCGATGTACTTGGGATTC
TTACTCCTCGCCATCGGTGTCTGTTTCGGAGCCAATTGCGGCTATGCTTGCAATCCTGCT
CGTGATCTCGCACCCAGATTCCTCACTCTTATCGCCGGATGGGGAACACAAGTCTTCAGC
TACAAAGACTACGCCTGGTTTTGGGTACCGATCATCGGACCTCATATTGGAGCCATTTTC
GGTGTCATTATCTAC---ATGCTATTCATCGAAGTTCACTGGCCTGAACAAGCTGACGGA
---ATGCTTCCC---ATCACT------------TCTTCCCAGCAA---------------
---GATGACAAACGTTCCAAATCCGCCAGCGCGGATTTGACTAATCTC------------
------------------------------------------------------------
------------------------------------------------------------
------------------------------------------------------------
------------------------------------------------------------
------------------------------------------------------------
------------------------------------------------------------
------------------------------------------------------------
------------------------------------------------------------
------------------------------------------------------------
------------------------------------------------------------
------------------------------------------------------------
------------------------------------------------------------
------------------------------------------------------------
------------------------------------------------------------
------------------------------------------------------------
------------------------------------------------------------
------------------------------------------------------------
------------------------------------------------------------
------------------------------------------------------------
------------------------------------------------------------
------------------------------------------------------------
------
>GKAM01014061_Podon_leuckartii_GlpA2b
------------------------------------------------------------
------------------------------------------------------------
------------ATGACA---CCGAAG---------------------ATA---CTGTCC
AAATTACGA---ATCGAAACCCCAATTCTTCGGGAAATTCTCGCTGAATTCTTGGGAACG
TTCATTATAGTGATATTTGGAGATGGTTGTGTAGCTCAAGTGGTATTGAGTGAATCAAAG
AATGGAGACTTCTTCTCCATCAACTGGGGCTGGGGTATTGGTGTTATGATGGGAGTCTTG
GTAGCTGGTGGAGTTTCAGGGGCCCACTTGAATCCAGCAGTTTCGCTGGCGATGGCTGTC
GTTGGCAAATTTCCGTTCCAAAAGGTATTATTCTACTGGCTCGCCCAATATCTGGGAGCT
TTCGCTGCCGCTGCTTCTGTTTTGGGAGTATACAGTGATGCTATTCAGAGCTACGCGGCA
ACACACTCAGACGGACAATTGGTAATGAATGAC------ACAATGAATGATCCT------
GGACTTGCTGGAATCTTCGCTACTTATCCTGCTGCTTGGTTATCCATTCACGGAGGAATG

GGCGATCAGATTTTAGGCACTATGCTCCTATTGTTGTGCATCTGTGCAATCACTGATAAA
AGAAATTCTCAAGTCCCC------------TCTTCAATGGTGCCCATGTATGTAGGATTG
TCAATCATAGCCATCGGTGTCTGTTTTGGAGTCAATGGTGGTTATGCGCTCAATCCTGCT
CGTGATCTCGCTCCCAGATTTCTGACGCTTATTTCCGGTTGGGGAACCCAAGTATTCAGC
TATAAAAACTATTCCTGGTTCTGGCAACCAATCATTGGACCTCACATTGGAGCAATTTTT
GGTGTCATTATCTAC---ATGCTCTTTATCGAAATTCACTGGCCTAATGAAGCTGATGAA
---GTTCTCGCA---ACTGACGTTCTC---GTGACCACTCCTGAA---------------
---CAACGAAAGGAACAATATTCAAGCCAAGTTGATATCAAGGAA---------------
------------------------------------------------------------
------------------------------------------------------------
------------------------------------------------------------
------------------------------------------------------------
------------------------------------------------------------
------------------------------------------------------------
------------------------------------------------------------
------------------------------------------------------------
------------------------------------------------------------
------------------------------------------------------------
------------------------------------------------------------
------------------------------------------------------------
------------------------------------------------------------
------------------------------------------------------------
------------------------------------------------------------
------------------------------------------------------------
------------------------------------------------------------
------------------------------------------------------------
------------------------------------------------------------
------------------------------------------------------------
------------------------------------------------------------
------
>GKAG01002845_Evadne_nordmanni_GlpA2b
------------------------------------------------------------
------------------------------------------------------------
------------ATGACA---CCGAAG---------------------ATA---CTGTCC
AAATTACGA---ATCGAAACCCCAATTCTTCGGGAAATTCTCGCTGAATTCTTGGGAACG
TTCATTATAGTGATATTTGGAGATGGTTGTGTAGCTCAAGTGGTATTGAGTGAATCAAAG
AATGGAGACTTCTTCTCCATCAACTGGGGCTGGGGTATTGGTGTTATGATGGGAGTCTTG
GTAGCTGGTGGAGTTTCAGGGGCCCACTTGAATCCAGCAGTTTCGCTGGCGATGGCTGTC
GTTGGCAAATTTCCGTTCCAAAAGGTATTATTCTACTGGCTCGCCCAATATCTGGGAGCT
TTCGCTGCCGCTGCTTCTGTTTTGGGAGTATACAGTGATGCTATTCAGAGCTACGCGGCA
ACACACTCAGACGGACAATTGGTAATGAATGAC------ACAATGAATGATCCT------
GGACTTGCTGGAATCTTCGCTACTTATCCTGCTGCTTGGTTATCCATTCACGGAGGAATG
GGCGATCAGATTTTAGGCACTATGCTCCTGTTGTTGTGCATCTGTGCAATCACTGATAAA
AGAAATTCTCAAGTCCCC------------TCTTCAATGGTGCCCATGTATGTAGGATTG
TCAATCATAGCCATCGGTGTCTGTTTTGGAGTCAATGGTGGTTATGCGCTCAATCCTGCT
CGTGATCTCGCTCCCAGATTTCTGACGCTTATTTCCGGTTGGGGAACCCAAGTATTCAGC
TATAAAAACTATTCCTGGTTCTGGCAACCAATCATTGGACCTCACATTGGAGCAATTTTT
GGTGTCATTATCTAC---ATGCTCTTTATCGAAATTCACTGGCCTAATGAAGCTGATGAA

---GTTCTCGCA---ACTGACGTTCTC---GTGACCACTCCTGAA---------------
---CAACGAAAGGAACAATATTCAAGCCAAGTTGATATCAAGGAA---------------
------------------------------------------------------------
------------------------------------------------------------
------------------------------------------------------------
------------------------------------------------------------
------------------------------------------------------------
------------------------------------------------------------
------------------------------------------------------------
------------------------------------------------------------
------------------------------------------------------------
------------------------------------------------------------
------------------------------------------------------------
------------------------------------------------------------
------------------------------------------------------------
------------------------------------------------------------
------------------------------------------------------------
------------------------------------------------------------
------------------------------------------------------------
------------------------------------------------------------
------------------------------------------------------------
------------------------------------------------------------
------------------------------------------------------------
------
>GKAM01003464_Podon_leuckartii_GlpA2c
------------------------------------------------------------
------------------------------------------------------------
------------ATGAAC---GGTATGGCCTCC---------------TTT---ATATCA
AACGCACGA---ATTGAAACACCACTTCTTCGTGAAGTTCTTGCTGAGTTCTTGGGTACC
TTCATTCTAGTGATTTTTGGAATTGGCTCTGGGGCTCAATCGAAACTCACCGACTCGGCT
AATGGAGATTTTTTTCCATCAACTGGGGTTCGGCGGCTGGCCTTATGATGGGAGTTTTA
GTTGCCGGTGGAGTTTCTGGAGCTCATCTGAATCCAGCAGTTTCACTAGCCATGGCTGTT
GGAGGAAAATTTCCATTTAAAAAGTACTTGTTTACTGGCTTGCCCAATACCTTGGAGCA
TTAGCAGCAGCTGCTTCTGTTTTGGGAGTTTACAGTGATGCAATCCAGGACTATACCTCA
AGACATTCAAATGGTACTTTGATCATAGATGAT------ACTACTAATGATCCC------
GGCCTTGCTGGAATCTTTGCAACATATCCTGCTGCTTGGTTAACTATTCAAGGAGGAATG
GGTGATCAGATACTTGGGACTATGCTTCTGTTGGTGTGCATTTGTGCAATTACTGACAAA
AAAAATACTCAAGTTCCT------------AGTTCATTGGTACCCATCTATGTTGGTTTT
ACAATCCTTGCCATTGGTATTAGTTTTGGAGCCAATTGCGGCTATGCTCTCAATCCAGCT
CGTGATCTTGCTCCCAGATTATTGACTCTCATTGCAGGATGGGGAACCCAAGTGTTCAGC
TACAAAAACTATACCTGGTTTTGGATACCAATAGTTGGACCTCATATTGGAGCCATTTTT
GGTGTGATTATCTAT---ATACTGTTCATTGAGGCACATTGGCCAGAAGATGCAGATCAA
---GCTCTTCCG------------------GTCACAACACCAACG------ATTTCC---
---TCTGGACAACGC---AAAAGTTCGATTATAGAT------------------------
------------------------------------------------------------
------------------------------------------------------------
------------------------------------------------------------
------------------------------------------------------------

------------------------------------------------------------
------------------------------------------------------------
------------------------------------------------------------
------------------------------------------------------------
------------------------------------------------------------
------------------------------------------------------------
------------------------------------------------------------
------------------------------------------------------------
------------------------------------------------------------
------------------------------------------------------------
------------------------------------------------------------
------------------------------------------------------------
------------------------------------------------------------
------------------------------------------------------------
------------------------------------------------------------
------------------------------------------------------------
------------------------------------------------------------
------
>GKAG01009425_Evadne_nordmanni_GlpA2c
------------------------------------------------------------
------------------------------------------------------------
------------------------------------------------------------
ATGATACGT---ATTGAATCGCCCCTGCTTCGCGAATCTATCGCCGAGTTCATAGGTACC
TTCGTATTAGTGATTTTCGGAGATAGCAGCGTTGCTCAGTCAACACTTTCAAACTCTAAA
AACGGGGATTTCTTTTCCATCAACTGGGGCTGGGGCCTCGGTGTTATGATCGGAGTTCTC
GTAGCTGGCGGAGTTTCAGGTGGTCATTTGAATCCGGCCGTATCGCTAGCCATGGCAATA
GTTGGGAAATTGCCAGTAAAAAAAGTATTGGTTTATTGGTTGGCTCAATACCTTGGAGCA
TTTGTTGCGGCTGCTTCTATCTATGGAGTTTACAATGATGCAATCTTGAACTATGCTGCG
ATCAATTCAAATAATATGTTCATAATGAACGAT------ACCGCCAACGACCCT------
GGTCTCGCAGGACTTTTCGGCACATATCCTGCTGCTTGGTTATCCATTCAAGGTGGTATC
GGGGATCAGATATTGGGTACTTTCCTGCTGTTGCTTGGCATCTGCGCGATTACTGACAAG
AAGAATAACAAAGTACCG------------GATGGATTGGTGCCCATGTATGTAGGATTT
TTAATCTTAGCCATCGGAGTTTGTTTCGGAGCGAATTGCGGCTTTGCGATCAATCCAGCT
CGTGATCTTGCTCCCAGGTTGTTATCTCTCATTGCAGGATGG---AACGAAGCTTTCAGT
TACAAGGGTTACACTTGGTTTTGGATTCCAATCGTCGGACCTCATATTGGAGCCATTCTT
GGTGTCCTAGTCTAT---ACGGTATGCATTGAAGCGCATTGGCCAGAAAAC---AGACTA
---GAAAACCTT---TATCAAGTT------------------------------------
---TCTGCTAGTAAA---------------------------------------------
------------------------------------------------------------
------------------------------------------------------------
------------------------------------------------------------
------------------------------------------------------------
------------------------------------------------------------
------------------------------------------------------------
------------------------------------------------------------
------------------------------------------------------------
------------------------------------------------------------
------------------------------------------------------------

------------------------------------------------------------
------------------------------------------------------------
------------------------------------------------------------
------------------------------------------------------------
------------------------------------------------------------
------------------------------------------------------------
------------------------------------------------------------
------------------------------------------------------------
------------------------------------------------------------
------------------------------------------------------------
------------------------------------------------------------
------
>GGQP01066089_Diaphanosoma_celebensis_GlpA2
------------------------------------------------------------
------------------------------------------------------------
------------ATGTCG---CCCAAG---------------------TTC---ATGAGC
AAGATCCGC---ATCGAGGCGCCGCTGCTCCGCGAGATCCTCGCCGAGTTCCTCGCCACT
TTCATCCTCGTCCTGTTTGGAGATGCCAGCGTTGCTCAGTCGGTGCTCTCCGGCGAGAAG
AACGGCGACTTCTTCTCCATCAACTGGGGCTGGGGTCTGGGCGTCATGATGGGCGTCCTG
ATCGCCGGCCGCGTGTCCGGTGCTCACCTGAACCCAGCCGTGACGCTGGCCATGGCCATT
TTCGGCCGCTTTCCCTGGATCAAGGTCCTCTTCTACTGGGCGGCTCAATACCTGGGCGCG
TTGGCCGCCGCTGCCTCCGTACTCGGCGTCTACAGCGAGGCCATTCACAACAAGACGGGC
------------GGATCGTTCACGATGCTGCCG------------GGTGATCCG------
GGCACGGCCGGAATTTTCGCCACCTACCCGGCTCCATATCTGTCGACCCTCGGCGGCTTG
GGCGATCAGATCCTGGGAACGTTCCTGCTGCTCCTGTGCGTGTGCGCCATTACCGACAAG
AAGAACGGCAACGTGTCG------------CCGGCCCAGGCGCCGATCTACGTCGGCATG
ACCATCATCGCCATCGGCGCCTGTTTCGGCGTCAACTGCGGCTACGCCATCAACCCGGCG
CGCGATCTAGCGCCGCGTGTCTTGACCTGGTTTGCCGGCTGGGGCACCGACGTCTTCACC
TACGGAAACTAC---TGGTTCTGGGTACCGATCGTTGGTCCGCACATCGGAGCCGTGCTC
GGCGTTGCCGTTTAC---ATTCTGTTCATCGAAGCCCACTGGCCCGACGATGGCGAACCG
---GACGCTTACGACGCTTCCAGAATCCCGCGCGTGTCCAACAGCGGC---GCCGTCGGC
GGCCGGCCGGAGAAGTCTCCGTCCTTCACCATGGACATGGAAAGTGGCCTATCTACCGTC
AGACCAGCCAAC------------------------------------------------
------------------------------------------------------------
------------------------------------------------------------
------------------------------------------------------------
------------------------------------------------------------
------------------------------------------------------------
------------------------------------------------------------
------------------------------------------------------------
------------------------------------------------------------
------------------------------------------------------------
------------------------------------------------------------
------------------------------------------------------------
------------------------------------------------------------
------------------------------------------------------------
------------------------------------------------------------
------------------------------------------------------------

------------------------------------------------------------
------------------------------------------------------------
------------------------------------------------------------
------------------------------------------------------------
------------------------------------------------------------
------
>PQ469251_Artemia_franciscana_Glp4_v1
------------------------------------------------------------
------------------------------------------------------------
------------ATGGAC---AAAAAA---------------------ATT---AAGAGC
AAATTGAGG---GTATCTAGTCCCATTTTTAGAGAGTTCGTTGCAGAATGTCTTGGAACA
TTTTTTCTAGTGATGATTGGAGATGCATCTGTGGCACAGTCAGTCCTGTCGAAAGAAGAA
AAGGGAGATTTTTTCTCCATAAATTGGGGCTTCGGGATGGGTGCTATGCTTGCTGTACTT
ATCTGTGGTGGAGTATCAGGGGCTCATTTGAACCCAGCTGTTACTCTTGCCATGGCCATA
GTTGGTAAACATCCATGGAAAAAAGTGTTGCATTATATGGCGGGTCAATATCTGGGAGGA
TTTTTAGCTGCTGCAGTTGTTTTAGGCGTATACAGTGAGGGAATATATTATTATGAAGAT
CAAGTTGGTAATGGTACCCTAAACATTGGCAAT---------------------------
---ACGGCGGGTATTTTTGCGACGTATCCTTATTTGTGGACAACCACATTAGGAGGCTTT
GTTGACCAGATTGTTGGAACCATGGTTCTTCTTATTGCTATTTGTGCTATAACAGATGAA
AAGAACATGCAAATCTCC------------AAGCCCCTGATACCTCTATATGTTGGCTTT
ACCTTTGTGGCAATTGGTGTTGCCTTTGGAGCGAATTGCGGTTATGCCATCAACCCAGCA
AGAGATTTGTCACCGCGTATCATTACTCTGATTGCAGGATGGGGCTCAGCCACATTCACA
GTGAACGACTAC---TGGTTCTGGGTGCCTATTGTTGGTCCTCACGTTGGCGCTATTCTT
GGTGTTTTTATCTAC---ATCCTTTGTATAGAAATGCATTGGCCAGAAGATGACAAAAGT
---GAGTCATTA---GCG------------------------------------------
------AAATCCAAAGTGGAAAAACAAACTATCGACATTGATAGTAAGTCAATTAATTTC
------------------------------------------------------------
------------------------------------------------------------
------------------------------------------------------------
------------------------------------------------------------
------------------------------------------------------------
------------------------------------------------------------
------------------------------------------------------------
------------------------------------------------------------
------------------------------------------------------------
------------------------------------------------------------
------------------------------------------------------------
------------------------------------------------------------
------------------------------------------------------------
------------------------------------------------------------
------------------------------------------------------------
------------------------------------------------------------
------------------------------------------------------------
------------------------------------------------------------
------------------------------------------------------------
------------------------------------------------------------
------------------------------------------------------------
------

>JAVRJZ010000021_Artemia_franciscana_Jo_Glp4_v1
------------------------------------------------------------
------------------------------------------------------------
------------ATGGAC---AAAAAA---------------------ATT---AAGAGC
AAATTGAGG---GTATCTAGTCCCATTTTTAGAGAGTTCGTTGCAGAATGTCTTGGAACA
TTTTTTCTAGTGATGATTGGAGATGCATCTGTGGCACAGTCAGTCCTGTCGAAAGAAGAA
AAGGGAGATTTTTTCTCCATAAATTGGGGCTTCGGGATGGGTGCTATGCTTGCTGTACTT
ATCTGTGGTGGAGTATCAGGGGCTCATTTGAACCCAGCTGTTACTCTTGCCATGGCCATA
GTTGGTAAACATCCATGGAAAAAAGTGTTGCATTATATGGCGGGTCAATATCTGGGAGGA
TTTTTAGCTGCTGCAGTTGTTTTAGGCGTATACAGTGAGGGAATATATTATTATGAAGAT
CAAGTTGGTAATGGTACCCTAAACATTGGCAAT---------------------------
---ACGGCGGGTATTTTTGCGACGTATCCTTATTTGTGGACAACCACATTAGGAGGCTTT
GTTGACCAGATTGTTGGAACCATGGTTCTTCTTATTGCTATTTGTGCTATAACAGATGAA
AAGAACATGCAAATCTCC------------AAGCCCCTGATACCTCTATATGTTGGCTTT
ACCTTTGTGGCAATTGGTGTTGCCTTTGGAGCGAATTGCGGCTATGCCATCAACCCAGCA
AGAGATTTGTCACCGCGTATCATTACTCTGATTGCAGGATGGGGCTCAGCCACATTCACG
GTGAACGACTAC---TGGTTCTGGGTGCCTATTGTTGGTCCTCACGTTGGCGCTATTCTT
GGTGTTTTTATCTAC---ATCCTTTGTATAGAAATGCATTGGCCAGAAGATGACAAAAGT
---GAGTCATTA---GCG------------------------------------------
------AAATCCAAAGTGGAAGAACAAACTATCGACATTGATAGTAAGTCAATTAATTTC
------------------------------------------------------------
------------------------------------------------------------
------------------------------------------------------------
------------------------------------------------------------
------------------------------------------------------------
------------------------------------------------------------
------------------------------------------------------------
------------------------------------------------------------
------------------------------------------------------------
------------------------------------------------------------
------------------------------------------------------------
------------------------------------------------------------
------------------------------------------------------------
------------------------------------------------------------
------------------------------------------------------------
------------------------------------------------------------
------------------------------------------------------------
------------------------------------------------------------
------------------------------------------------------------
------------------------------------------------------------
------------------------------------------------------------
------
>JAYKOS010000005_Artemia_franciscana_Bett_Glp4_v1
------------------------------------------------------------
------------------------------------------------------------
------------ATGGAC---AAAAAA---------------------ATT---AAGAGC
AAATTGAGG---GTATCTAGTCCCATTTTTAGAGAGTTCGTTGCAGAATGTCTTGGAACA
TTTTTTCTAGTGATGATTGGAGATGCATCTGTGGCACAGTCAGTCCTGTCGAAAGAAGAA

AAGGGAGATTTTTTCTCCATAAATTGGGGCTTCGGGATGGGTGCTATGCTTGCTGTACTT
ATCTGTGGTGGAGTATCAGGGGCTCATTTGAACCCAGCTGTTACTCTTGCCATGGCCATA
GTTGGTAAACATCCATGGAAAAAAGTGTTGCATTATATGGCGGGTCAATATCTGGGAGGA
TTTTTAGCTGCTGCAGTTGTTTAGGCGTATACAGTGAGGGAATATATTATTATGAAGAT
CAAGTTGGTAATGGTACCCTAAACATTGGCAAT---------------------------
---ACGGCGGGTATTTTTGCGACGTATCCTTATTTGTGGACAACCACATTAGGAGGCTTT
GTTGACCAGATTGTTGGAACCATGGTTCTTCTTATTGCTATTTGTGCTATAACAGATGAA
AAGAACATGCAAATCTCC------------AAGCCCCTGATACCTCTATATGTTGGCTTT
ACCTTTGTGGCAATTGGTGTTGCCTTTGGAGCGAATTGCGGTTATGCCATCAACCCAGCA
AGAGATTTGTCACCGCGTATCATTACTCTGATTGCAGGATGGGGCTCAGCCACATTCACG
GTGAACGACTAC---TGGTTCTGGGTGCCTATTGTTGGTCCTCACGTTGGCGCTATTCTT
GGTGTTTTTATCTAC---ATCCTTTGTATAGAAATGCATTGGCCAGAAGATGACAAAAGT
---GAGTCATTA---GCG------------------------------------------
------AAATCCAAAGTGGAAGAACAAACTATCGACATTGATAGTAAGTCAATTAATTTC
------------------------------------------------------------
------------------------------------------------------------
------------------------------------------------------------
------------------------------------------------------------
------------------------------------------------------------
------------------------------------------------------------
------------------------------------------------------------
------------------------------------------------------------
------------------------------------------------------------
------------------------------------------------------------
------------------------------------------------------------
------------------------------------------------------------
------------------------------------------------------------
------------------------------------------------------------
------------------------------------------------------------
------------------------------------------------------------
------------------------------------------------------------
------------------------------------------------------------
------------------------------------------------------------
------------------------------------------------------------
------------------------------------------------------------
------
>PQ469252_Artemia_franciscana_Glp4_v2
------------------------------------------------------------
------------------------------------------------------------
------------ATGGAC---AAAAAA---------------------ATT---AAGAGC
AAATTGAGG---GTATCTAGTCCCATTTTTAGAGAGTTCGTTGCAGAATGTCTTGGAACA
TTTTTTCTAGTGATGATTGGAGATGCATCTGTGGCACAGTCAGTCCTGTCGAAAGAAGAA
AAGGGAGATTTTTTCTCCATAAATTGGGGCTTCGGGATGGGTGCTATGCTTGCTGTACTT
ATCTGTGGTGGAGTATCAGGGGCTCATTTGAACCCAGCTGTTACTCTTGCCATGGCCATA
GTTGGTAAACATCCATGGAAAAAAGTGTTGCATTATATGGCGGGTCAATATCTGGGAGGA
TTTTTAGCTGCTGCAGTTGTTTAGGCGTATACAGTGAGGGAATATATTATTATGAAGAT
CAAGTTGGTAATGGTACCCTAAACATTGGCAAT---------------------------
---ACGGCGGGTATTTTTGCGACGTATCCTTATTTGTGGACAACCACATTAGGAGGCTTT

GTTGACCAGATTGTTGGAACCATGGTTCTTCTTATTGCTATTTGTGCTATAACAGATGAA
AAGAACATGCAAATCTCC------------AAGCCCCTGATACCTCTATATGTTGGCTTT
ACCTTTGTGGCAATTGGTGTTGCCTTTGGAGCGAATTGCGGCTATGCCATCAACCCAGCA
AGAGATTTGTCACCGCGTATCATTACTCTGATTGCAGGATGGGGCTCAGCCACATTCACA
GTGAACGACTAC---TGGTTCTGGGTGCCTATTGTTGGTCCTCACGTTGGCGCTATTCTT
GGTGTTTTTATCTAC---ATCCTTTGTATAGAAATGCATTGGCCAGAAGATGACAAAAGT
---GAGTCATTA---GCG------------------------------------------
------AAATCCAAAGTGGAAGAACAAACTATCGACATTGATAGC---------------
------------------------------------------------------------
------------------------------------------------------------
------------------------------------------------------------
------------------------------------------------------------
------------------------------------------------------------
------------------------------------------------------------
------------------------------------------------------------
------------------------------------------------------------
------------------------------------------------------------
------------------------------------------------------------
------------------------------------------------------------
------------------------------------------------------------
------------------------------------------------------------
------------------------------------------------------------
------------------------------------------------------------
------------------------------------------------------------
------------------------------------------------------------
------------------------------------------------------------
------------------------------------------------------------
------------------------------------------------------------
------------------------------------------------------------
------
>CM051264_Artemia_sinica_Glp4_v1
------------------------------------------------------------
------------------------------------------------------------
------------ATGGAC---AAACAA---------------------ATT---AGGAAC
AAATTGAGA---ATATCTAGTCCTATCTTCAGAGAGTTCGTTGCAGAATGTCTTGGAACC
TTTTTCCTGGTGACGTTTGGAGACGCATCTGTGGCACAATCAGTCCTGTCGAAAGAAGAA
AAGGGAGATTTTTTCTCCATAAATTGGGGCTTCGGGATGGGTGCTATGCTTGCTGTACTT
ATCTGTGGGGGAGTATCAGGGGCTCATTTGAACCCAGCTGTTACTCTTGCCATGGCCATA
GTTGGTAAACATCCATGGAAAAAAGTGTTGCATTATATGGCGGGCCAATACCTGGGAGGA
TTTCTAGCTGCAGCAGTTGTTTTAGGCGTATACAGTGAGGGAATATATTATTATGAAGAT
CAAGTTGGTAACGGTACCCTGAACATTGGCAAT---------------------------
---ACGGCGGGTATTTTTGCGACGTACCCTTATTTGTGGACAACCACATTAGGAGGCCTT
GTTGACCAGATTGTTGGAACCATGGTTCTTCTTATTGCTATTTGCGCTATAACAGATGAA
AAGAACATGCAGATATCC------------AAACCCCTAATCCCTCTATATGTTGGCTTC
ACCTTTGTGGCAATTGGCGTTGCCTTTGGAGCGAATTGCGGCTACGCTATCAACCCAGCA
AGAGATTTGTCAGCGTATATCATTACTCTGATTGCAGGATGGGGCACTACCACATTCACG
GTGAACGACTTT---TGGTTTTGGGTCCCTATTGTTGGCCCTCATGTTGGTGCCATTCTT
GGTGTTTTTATTTAC---ATCCTTTGTATAGAAGTACATTGGCCCGAAGATGACAAAAAT

```
---GAGTCATTA---ACG------------------------------------------
------AAATCCAAAGTGAAAGAACAAACTATTGACATTGATAGTAAGTCAATTAATTCT
------------------------------------------------------------
------------------------------------------------------------
------------------------------------------------------------
------------------------------------------------------------
------------------------------------------------------------
------------------------------------------------------------
------------------------------------------------------------
------------------------------------------------------------
------------------------------------------------------------
------------------------------------------------------------
------------------------------------------------------------
------------------------------------------------------------
------------------------------------------------------------
------------------------------------------------------------
------------------------------------------------------------
------------------------------------------------------------
------------------------------------------------------------
------------------------------------------------------------
------------------------------------------------------------
------------------------------------------------------------
------------------------------------------------------------
------
>JAQQPU010016311_47589_21864_Artemia_sp_Kazakhstan_Glp4_v1
------------------------------------------------------------
------------------------------------------------------------
------------ATGGAC---AAACAA---------------------ATT---AGGAAC
AAATTGAGA---ATATCTAGCCCTATCTTCAGAGAGTTTGTTGCAGAATGTCTTGGAACC
TTTTTCCTGGTGACGTTTGGAGACGCATCTGTGGCACAATCAGTCCTGTCGAAAGAAGAA
AAGGGAGATTTTTTCTCCATAAATTGGGGCTTCGGGATGGGTGCTATGCTTGCTGTACTT
ATCTGTGGGGGAGTATCAGGGGCTCACTTGAACCCAGCTGTTACTCTTGCCATGGCCATA
GTTGGTAAACATCCATGGAAAAAAGTGTTGCATTATATGGCAGGCCAATATCTGGGAGGA
TTTCTAGCTGCAGCAGTTGTTTTAGGCGTATACAGTGAGGGAATATATTATTATGAAGAT
CATGTTGGTAACGGTACCCTGAACATTGGCAAT---------------------------
---ACGGCGGGTATTTTTGCGACGTACCCTTATTTGTGGACAACCACATTAGGAGGCCTT
GTTGACCAGATTGTTGGAACCATGGTTCTTCTTATTGCTATTTGTGCTATAACAGATGAA
AAGAACATGCAGATATCC------------AAACCCCTGATCCCTCTATATGTTGGCTTC
ACCTTTGTGGCAATTGGCGTTGCCTTTGGAGCGAATTGCGGCTACGCTATCAACCCAGCA
AGAGATTTGTCACCGCGTATCATTACTCTGATCGCAGGATGGGGCACTACCACATTCACG
GTGAACGACTAT---TGGTTTTGGGTCCCTATTGTTGGCCCTCATGTTGGTGCCATTCTG
GGTGTTTTTATTTAC---ATCCTTTGTATAGAAGTACATTGGCCTGAAGATGACAAAAAT
---GAGTCATTA---GCG------------------------------------------
------AAATCCAAAGTGAAAGAACAAACTATTGACATTGATAGTAAGTCAATTAATTCT
------------------------------------------------------------
------------------------------------------------------------
------------------------------------------------------------
------------------------------------------------------------
```

------------------------------------------------------------
------------------------------------------------------------
------------------------------------------------------------
------------------------------------------------------------
------------------------------------------------------------
------------------------------------------------------------
------------------------------------------------------------
------------------------------------------------------------
------------------------------------------------------------
------------------------------------------------------------
------------------------------------------------------------
------------------------------------------------------------
------------------------------------------------------------
------------------------------------------------------------
------------------------------------------------------------
------------------------------------------------------------
------------------------------------------------------------
------
>JAVRJZ010000021_Artemia_franciscana_Jo_Glp3
------------------------------------------------------------
------------------------------------------------------------
------------ATGGAC---AAAGGA---------------------ATT---AAGAAC
AAATTAAGA---GTATCTAGTCCTATCTTCAGGGAGTTTGCTGCAGAATGTCTTGGAACG
CTTATCCTAGTGGTGTTTGGAGATGCAGGTGTTGCACAGTCAGTCCTGTCAAAAGGGGAA
AAGGGAGATTTCTTCTCCACAAATTGGGGCTGGGGCCTAGGTGGCATGCTTGCTGTACTT
ATCTGTGGTGGAGTATCAGGGGCTCATTTGAACCCAGCTCTTACTTTTGCCATGGCCATA
GTTGGTAAACATCCATGGAAAAAAGTGTTGCATTATATGGCAGGCCAATACCTGGGAGGA
CTTATAGCTGCTGCAGTTGTTTTAGGCGTATACAGTGAGGGAATATATTATTACGAAGAT
GAAGTTGGCAACGGTACCTTAAACATTGGCAAT---------------------------
---ACGGCGGGTATTTTTGCAACGTACCCTTATAAGTGGACAACAACATTAGGAGGCCTT
GCTGACGCAATTGTTGGAACCATGGTTCTTCTTATTGTTATTTGTGCTATAACAGATGAA
AAGAACATGCAGATCTCT------------AAACCTCTGATCCCTCTATATGTTGGCTTC
ACCTTTTTGGCAATTGGCGTTTGCTTTGGAGTAAATTCCGGCTGTGCCATCAACCCAGCA
AGAGATTTGTCCCCACGTATTATTACTCTGATCGCAGGATGGGGCACTGGCACATTCACG
GTGAACGACTAT---TGGTTCTGGGTGCCTATTGTTGGTCCTCACGTTGGCGCCATTCTT
GGTGTTTTTATCTAC---ATCCTTTGTATAGAAGTGCATTGGCCCGAAGATGACAAAAAT
---GAGTCATTA---GCC------------------------------------------
------AAATCCAAAGTGAAAGATAAAACTATTGACATTGATAGTAAGTTAAATAATTTC
------------------------------------------------------------
------------------------------------------------------------
------------------------------------------------------------
------------------------------------------------------------
------------------------------------------------------------
------------------------------------------------------------
------------------------------------------------------------
------------------------------------------------------------
------------------------------------------------------------
------------------------------------------------------------

------------------------------------------------------------
------------------------------------------------------------
------------------------------------------------------------
------------------------------------------------------------
------------------------------------------------------------
------------------------------------------------------------
------------------------------------------------------------
------------------------------------------------------------
------------------------------------------------------------
------------------------------------------------------------
------------------------------------------------------------
------
>Afra2B019209T1_Artemia_franciscana_Glp3
------------------------------------------------------------
------------------------------------------------------------
------------------------------------------------------------
------------------------------------------------------------
------------GTGTTTGGAGATGCAGGTGTTGCACAGTCAGTCCTGTCAAAAGGGGAA
AAGGGAGATTTCTTCTCCACAAATTGGGGCTGGGGCCTAGGTGGCATGCTTGCTGTACTT
ATCTGTGGTGGAGTATCAGGGGCTCATTTGAACCCAGCTCTTACTTTTGCCATGGCCATA
GTTGGTAAACATCCATGGAAAAAAGTGTTGCATTATATGGCAGGCCAATACCTGGGAGGA
CTTATAGCTGCTGCAGTTGTTTTAGGCGTATACAGTGAGGGAATATATTATTACGAAGAT
GAAGTTGGCAACGGTACCTTAAACATTGGCAAT---------------------------
---ACGGCGGGTATTTTTGCAACGTACCCTTATAAGTGGACAACAACATTAGGAGGCCTT
GCTGACGCAATTGTTGGAACCATGGTTCTTCTTATTGTTATTTGTGCTATAACAGATGAA
AAGAACATGCAGATCTCT------------AAACCTCTGATCCCTCTATATGTTGGCTTC
ACCTTTTTGGCAATTGGCGTTTGCTTTGGAGTAAATTCCGGCTGTGCCATCAACCCAGCA
AGAGATTTGTCCCCACGTATTATTACTCTGATCGCAGGATGGGGCACTGGCACATTCACG
GTGAACGACTAT---TGGTTCTGGGTGCCTATTGTTGGTCCTCACGTTGGCGCCATTCTT
GGTGTTTTTATCTAC---ATCCTTTGTATAGAAGTGCATTGGCCAGAAGATGACAAAAAT
---GAGTCATTA---GCC------------------------------------------
------AAATCCAAAGTGAAAGATAAAACTATTGACATTGATAGT---------------
------------------------------------------------------------
------------------------------------------------------------
------------------------------------------------------------
------------------------------------------------------------
------------------------------------------------------------
------------------------------------------------------------
------------------------------------------------------------
------------------------------------------------------------
------------------------------------------------------------
------------------------------------------------------------
------------------------------------------------------------
------------------------------------------------------------
------------------------------------------------------------
------------------------------------------------------------
------------------------------------------------------------
------------------------------------------------------------

------------------------------------------------------------
------------------------------------------------------------
------------------------------------------------------------
------------------------------------------------------------
------------------------------------------------------------
------
>JAYKOS010000005_Artemia_franciscana_Bett_Glp3
------------------------------------------------------------
------------------------------------------------------------
------------ATGGAC---AAAGGA---------------------ATT---AAGAAC
AAATTAAGA---GTATCTAGTCCTATCTTCAGGGAGTTTGCTGCAGAATGTCTTGGAACG
TTTATCCTAGTGGTGTTTGGAGATGCAGGTATTGCACAGTCAGTCCTGTCAAAAGGGGAA
AAGGGAGATTTCTTCTCCACAAATTGGGGCTGGGGCCTAGGTGGCATGCTTGCTGTACTT
ATCTGTGGTGGAGTATCAGGGGCTCATTTGAACCCAGCTCTTACTTTTGCCATGGCCATA
GTTGGTAAACATCCATGGAAAAAAGTGTTGCATTATATGGCAGGCCAATACCTGGGAGGA
CTTATAGCTGCTGCAGTTGTTTTAGGCGTATACAGTGAGGGAATATATTATTACGAAGAT
GAAGTTGGCAACGGTACCTTAAACATTGGCAAT---------------------------
---ACGGCGGGTATTTTTGCAACGTACCCTTATAAGTGGACAACAACATTAGGAGGCCTT
GCTGACGCAATTGTTGGAACCATGGTTCTTCTTATTGTTATTTGTGCTATAACAGATGAA
AAGAACATGCAGATCTCT------------AAACCTCTGATCCCTCTATATGTTGGCTTC
ACCTTTTTGGCAATTGGCGTTTGCTTTGGAGTAAATTCCGGCTGTGCCATCAACCCAGCA
AGAGATTTGTCCCCACGTATTATTACTCTGATCGCAGGATGGGGCACTGGCACATTCACG
GTGAACGACTAT---TGGTTCTGGGTGCCTATTGTTGGTCCTCACGTTGGCGCCATTCTT
GGTGTTTTTATCTAC---ATCCTTTGTATAGAAGTGCATTGGCCCGAAGATGACAAAAAT
---GAGTCATTA---GCC------------------------------------------
------AAATCCAAAGTGAAAGATAAAACTATTGACATTGATAGTAAGTTAAATAATTTC
------------------------------------------------------------
------------------------------------------------------------
------------------------------------------------------------
------------------------------------------------------------
------------------------------------------------------------
------------------------------------------------------------
------------------------------------------------------------
------------------------------------------------------------
------------------------------------------------------------
------------------------------------------------------------
------------------------------------------------------------
------------------------------------------------------------
------------------------------------------------------------
------------------------------------------------------------
------------------------------------------------------------
------------------------------------------------------------
------------------------------------------------------------
------------------------------------------------------------
------------------------------------------------------------
------------------------------------------------------------
------------------------------------------------------------
------

>PQ469254_Artemia_franciscana_Glp2_v1
------------------------------------------------------------
------------------------------------------------------------
------------ATGGAC---AGAGGA---------------------ATT---AAGAAC
AAATTGAAA---GTATCTAGTCCTATCTTCAGAGAGTTCGTTGCAGAGTGTCTTGGTACT
TTTATCCTAGTGGCGTTTGGAGATGCATGTGTTGCTCAGTCGGTCCTGTCGAAAGGGGAA
AAGGGAGATTTCTTCTCGATAAACTGGGGCTGGGGTTTAGGTGGCATGCTTGCTGTGCTT
ATCTGTGGTGGAGTATCAGGGGCTCATTTGAACCCAGCTGTTACTCTTGCCATGGCCGTA
GTTGGTAAACATCCATGGAAAAAAGTGTTGCATTATATGGCGGGTCAATACCTGGGAGGA
CTTATAGCTGCTGCAGTTGTATTAGGCGTATACTGTGAGGGAATATATTATTACGAAGAT
CAAGTTGGCAACGGTACCCTAAGCATTGGCAAT---------------------------
---ACGGCAGGTATTTTTGCAACGTACCCTTATATGTGGGCAACAACATTAGGAGGCTTA
GATGACCAGATCTTCGGAACCATGACTGTTCTTATAGCTGTTTGTGCTATAACAGATGAA
AAGAACAGGCAGATCTCC------------AAGCCTCTGATCCCTCTATATGTTGGCTTC
ACCATTTTGGCAATTGGCGTTTGCTTTGGAGCAAATTGCGGCTATGCCATCAACCCAGCA
AGAGATTTGTCCCCACGTATTATTACTCTGATCGCAGGATGGGGCACTGGCACATTCACG
GTGAACGACTAT---TGGTTCTGGGTGCCTATTGTTGGTCCTCACGTTGGCGCCATTCTT
GGTGTTTTTATCTAC---ATCCTTTGTATAGAAGTGCATTGGCCAGAAGATGACAAAAAT
---GAGTCATTA---ACG------------------------------------------
------AAATCCGAAGTGAAAGAACAAACCATTGACATTGATAGT---TTG---------
------------------------------------------------------------
------------------------------------------------------------
------------------------------------------------------------
------------------------------------------------------------
------------------------------------------------------------
------------------------------------------------------------
------------------------------------------------------------
------------------------------------------------------------
------------------------------------------------------------
------------------------------------------------------------
------------------------------------------------------------
------------------------------------------------------------
------------------------------------------------------------
------------------------------------------------------------
------------------------------------------------------------
------------------------------------------------------------
------------------------------------------------------------
------------------------------------------------------------
------------------------------------------------------------
------------------------------------------------------------
------------------------------------------------------------
------
>JAYKOS010000005_Artemia_franciscana_Bett_Glp2_v1
------------------------------------------------------------
------------------------------------------------------------
------------ATGGAC---AGAGGA---------------------ATT---AAGAAC
AAATTGAAA---GTATCTAGTCCTATCTTCAGAGAGTTCGTTGCAGAGTGTCTTGGTACT
TTTATCCTAGTGGCGTTTGGAGATGCATGTGTTGCTCAGTCGGTCCTGTCGAAAGGGGAA

AAGGGAGATTTCTTCTCGATAAACTGGGGCTGGGGTTTAGGTGGCATGCTTGCTGTACTT
ATCTGTGGTGGAGTATCAGGGGCTCATTTGAACCCAGCTGTTACTCTTGCCATGGCCATA
GTTGGTAAACATCCATGGAAAAAAGTGTTGCATTATATGGCGGGTCAATACCTGGGAGGA
CTTATAGCTGCTGCAGTTGTTTAGGCGTATACAGTGAGGGAATATATTATTACGAAGAT
CAAGTTGGCAACGGTACCCTAAACATTGGCAAT---------------------------
---ACGGCAGGTATTTTTGCAACGTACCCTTATATGTGGACAACAACACTAGGAGGCTTT
GCTGACCAGATCTTCGGAACCATGACTCTTCTTATAGCTGTTTGTGCTATAACAGATGAA
AAGAACATGCAGATCTCC------------AAGCCTCTGATCCCTCTATATGTTGGCTTC
ACCATTTTGGCAATTGGCGTTTGCTTTGGAGCAAATTGCGGCTATGCCATCAACCCAGCA
AGAGATTTGTCCCCACGTATTATTACTCTGATCGCAGGATGGGGCACTGGCACATTCACG
GTGAACGACTAT---TGGTTCTGGGTGCCTATTGTTGGTCCTCACGTTGGCGCCATTCTT
GGTGTTTTTATCTAC---ATCCTTTGTATAGAAGTGCATTGGCCAGAAGATGACAAAAAT
---GAGTCATTA---ACG------------------------------------------
------AAATCCAAAGTGAAAGAACAAACCATTGACATTGATAGTAAGTTAAATAGTTTC
------------------------------------------------------------
------------------------------------------------------------
------------------------------------------------------------
------------------------------------------------------------
------------------------------------------------------------
------------------------------------------------------------
------------------------------------------------------------
------------------------------------------------------------
------------------------------------------------------------
------------------------------------------------------------
------------------------------------------------------------
------------------------------------------------------------
------------------------------------------------------------
------------------------------------------------------------
------------------------------------------------------------
------------------------------------------------------------
------------------------------------------------------------
------------------------------------------------------------
------------------------------------------------------------
------------------------------------------------------------
------------------------------------------------------------
------
>JAVRJZ010000021_Artemia_franciscana_Jo_Glp2_v1
------------------------------------------------------------
------------------------------------------------------------
------------ATGGAC---AGAGGA---------------------ATT---AAGAAC
AAATTGAAA---GTATCTAGTCCTATCTTCAGAGAGTTCGTTGCAGAGTGTCTTGGTACT
TTTATCCTAGTGGCGTTTGGAGATGCATGTGTTGCTCAGTCGGTCCTGTCGAAAGGGGAA
AAGGGAGATTTCTTCTCGATAAACTGGGGCTGGGGTTTAGGTGGCATGCTTGCTGTACTT
ATCTGTGGTGGAGTATCAGGGGCTCATTTGAACCCAGCTGTTACTCTTGCCATGGCCATA
GTTGGTAAACATCCATGGAAAAAAGTGTTGCATTATATGGCGGGTCAATACCTGGGAGGA
CTTATAGCTGCTGCAGTTGTTTAGGCGTATACAGTGAGGGAATATATTATTACGAAGAT
CAAGTTGGCAACGGTACCCTAAACATTGGCAAT---------------------------
---ACGGCAGGTATTTTTGCAACGTACCCTTATATGTGGACAACAACATTAGGAGGCTTA

GCTGACCAGATCTTCGGAACCATGACTCTTCTTATAGCTGTTTGTGCTATAACAGATGAA
AAGAACATGCAGATCTCC------------AAGCCTCTGATCCCTCTATATGTTGGCTTC
ACCATTTTGGCAATTGGCGTTTGCTTTGGAGCAAATTGCGGCTATGCCATCAACCCAGCA
AGAGATTTGTCCCCACGTATTATTACTCTGATCGCAGGATGGGGCACTGGCACATTCACG
GTGAACGACTAT---TGGTTCTGGGTGCCTATTGTTGGTCCTCACGTTGGCGCCATTCTT
GGTGTTTTTATCTAC---ATCCTTTGTATAGAAGTGCATTGGCCAGAAGATGACAAAAAT
---GAGTCATTA---ACG------------------------------------------
------AAATCCAAAGTGAAAGAACAAACCATTGACATTGATAGTAAGTTAAATAGTTTC
------------------------------------------------------------
------------------------------------------------------------
------------------------------------------------------------
------------------------------------------------------------
------------------------------------------------------------
------------------------------------------------------------
------------------------------------------------------------
------------------------------------------------------------
------------------------------------------------------------
------------------------------------------------------------
------------------------------------------------------------
------------------------------------------------------------
------------------------------------------------------------
------------------------------------------------------------
------------------------------------------------------------
------------------------------------------------------------
------------------------------------------------------------
------------------------------------------------------------
------------------------------------------------------------
------------------------------------------------------------
------------------------------------------------------------
------
>JAVRJZ010000021_Artemia_franciscana_Jo_Glp2_v2
ATGGGGTTCAGTGAGGAAGTTGCTAAAGAGTACTATGATTACTACTATAAAAGCTCGACA
GACCCCGACGGCACAGCAAATAAGTATCCATCAAGCTTGAGTGCTTACAGCACAAACACA
GATATATTCCAAATGGAC---AGAGGA---------------------ATT---AAGAAC
AAATTGAAA---GTATCTAGTCCTATCTTCAGAGAGTTCGTTGCAGAGTGTCTTGGTACT
TTTATCCTAGTGGCGTTTGGAGATGCATGTGTTGCTCAGTCGGTCCTGTCGAAAGGGGAA
AAGGGAGATTTCTTCTCGATAAACTGGGGCTGGGGTTTAGGTGGCATGCTTGCTGTACTT
ATCTGTGGTGGAGTATCAGGGGCTCATTTGAACCCAGCTGTTACTCTTGCCATGGCCATA
GTTGGTAAACATCCATGGAAAAAAGTGTTGCATTATATGGCGGGTCAATACCTGGGAGGA
CTTATAGCTGCTGCAGTTGTTTTAGGCGTATACAGTGAGGGAATATATTATTACGAAGAT
CAAGTTGGCAACGGTACCCTAAACATTGGCAAT---------------------------
---ACGGCAGGTATTTTTGCAACGTACCCTTATATGTGGACAACAACATTAGGAGGCTTA
GCTGACCAGATCTTCGGAACCATGACTCTTCTTATAGCTGTTTGTGCTATAACAGATGAA
AAGAACATGCAGATCTCC------------AAGCCTCTGATCCCTCTATATGTTGGCTTC
ACCATTTTGGCAATTGGCGTTTGCTTTGGAGCAAATTGCGGCTATGCCATCAACCCAGCA
AGAGATTTGTCCCCACGTATTATTACTCTGATCGCAGGATGGGGCACTGGCACATTCACG
GTGAACGACTAT---TGGTTCTGGGTGCCTATTGTTGGTCCTCACGTTGGCGCCATTCTT
GGTGTTTTTATCTAC---ATCCTTTGTATAGAAGTGCATTGGCCAGAAGATGACAAAAAT

---GAGTCATTA---ACG------------------------------------------
------AAATCCAAAGTGAAAGAACAAACCATTGACATTGATAGTAAGTTAAATAGTTTC
------------------------------------------------------------
------------------------------------------------------------
------------------------------------------------------------
------------------------------------------------------------
------------------------------------------------------------
------------------------------------------------------------
------------------------------------------------------------
------------------------------------------------------------
------------------------------------------------------------
------------------------------------------------------------
------------------------------------------------------------
------------------------------------------------------------
------------------------------------------------------------
------------------------------------------------------------
------------------------------------------------------------
------------------------------------------------------------
------------------------------------------------------------
------------------------------------------------------------
------------------------------------------------------------
------------------------------------------------------------
------------------------------------------------------------
------
>PQ469253_Artemia_franciscana_Glp2_v2
ATGGGGTTCAGTGAGGAAGTTGCTAAAGAGTACTATGATTACTACTATAAAAGCTCGACA
GACCCCGACGGCACAGCAAATAAGTATCCATCAAGCTTGAGTGCTTACAGCACAAACACA
GATATATTCCAAATGGAC---AGAGGA---------------------ATT---AAGAAC
AAATTGAAA---GTATCTAGTCCTATCTTCAGAGAGTTCGTTGCAGAGTGTCTTGGTACT
TTTATCCTAGTGGCGTTTGGAGATGCATGTGTTGCTCAGTCGGTCCTGTCGAAAGGGGAA
AAGGGAGGTTTCTTCTCGATAAACTGGGGCTGGGGTTTAGGTGGCATGCTTGCTGTACTT
ATCTGTGGTGGAGTATCAGGGGCTCATTTGAACCCAGCTGTTACTCTTGCCATGGCCGTA
GTTCGTAAACATCCATGGAAAAAAGTGTTGCATTACATGGCAGGTCAATATCTGGGAGGA
TTTTTAGCTGCTGCAGTTGTTTTAGGCGTATACAGTGAGGGAATATATTATTACGAAGAT
CAAGTTGGCAACGGTACCCTAAACATTGGCAAT---------------------------
---ACGGCAGGTATTTTTGCAACGTACCCTTATATGTGGACAACAACATTAGGAGGCTTA
GCTGACCAGATCTTCGGAACCATGACTCTTCTTATAGCTGTTTGTGCTATAACAGATGAA
AAGAACATGCAGATCTCC------------AAGCCTCTGATCCCTCTATATGTTGGCTTC
ACCATTTTGGCAATTGGCGTTTGCTTTGGAGCAAATTGCGGCTATGCCATCAACCCAGCA
AGAGATTTGTCCCCACGTATTATTACTCTGATCGCAGGATGGGGCACTGGCACATTCACG
GTGAACGACTAT---TGGTTCTGGGTGCCTATTGTTGGTCCTCACGTTGGCGCCATTCTT
GGTGTTTTTATCTAC---ATCCTTTGTATAGAAGTGCATTGGCCAGAAGATGACAAAAAT
---GAGTCATTA---ACG------------------------------------------
------AAATCCAAAGTGAAAGAACAAACCATTGACATTGATAGT---TTG---------
------------------------------------------------------------
------------------------------------------------------------
------------------------------------------------------------
------------------------------------------------------------

------------------------------------------------------------
------------------------------------------------------------
------------------------------------------------------------
------------------------------------------------------------
------------------------------------------------------------
------------------------------------------------------------
------------------------------------------------------------
------------------------------------------------------------
------------------------------------------------------------
------------------------------------------------------------
------------------------------------------------------------
------------------------------------------------------------
------------------------------------------------------------
------------------------------------------------------------
------------------------------------------------------------
------------------------------------------------------------
------------------------------------------------------------
------
>CM051264_Artemia_sinica_Glp1_v2
ATGGGGTTCAGTGAGGAAGTTGCTAAAGAGTACTATGATTACTACTATAAAAGCTCGACA
GACCCCGACGGCACAGCAAATAAGTATCCATCAAGCTTGAGTGCTTACAGTACAAGCACA
GATATATTCCAAATGGAC---AGAGGA---------------------ATT---AAGAAC
AAATTGAGA---GTATCTAGTCCTATCTTCAGAGAGTTCGTTGCAGAATGTCTTGGAACT
TTTATCCTAGTGGCATTTGGAGATGCATGTGTTGCACAGTCGGTCCTGTCGGAAAGGGAA
AAGGGAGATTTCTTCTCTATAAATTGGGGCTGGGGGCTAGGTGGCATGCTTGCTGTACTT
ATCTGTGGTGGAGTATCAGGGGCTCATTTGAACCCAGCTGTTACTCTTGCCATGGCCATA
GTTGGTAAACATCCATGGAAAAAAGTGTTGCATTATATGGCGGGCCAATACCTGGGAGGA
TTTCTAGCTGCTGCAGTTGTTTTAGGCGTATACAGTGAGGGAATATATTATTACGAAGAT
CAAGTTGGCAATGGTACCCTAAACATCGGCAAC---------------------------
---ACGGCTGGTATTTTTGCGACATACCCTTATATGTGGACAACGACATTAGGAGGCTTT
GCTGACCAGATTTTCGGAACCATGACTCTTCTTATTGCTGTTTGTGCTATAACAGATGAA
AAGAACATGCAGATTTCC------------AAGCCTCTGATTCCTCTATATGTTGGCTTT
ACCATTTTGGCAATTGGTGTTTGCTTTGGAGCAAATTGTGGCTACGCCATCAACCCAGCA
AGAGATTTGTCACCACGTATTATTACTCTGATTGCAGGATGGGGCACTGGCACATTCACG
GTGAACAACTAT---TGGTTTTGGGTGCCCATTGTAGGTCCTCACGTTGGTGCCATTCTT
GGTGTTTTTATTTAC---ATCCTTTGTATAGAAGTACATTGGCCCGAAGATGACAAAAAT
---GAGTCATTA---GCG------------------------------------------
------AAATCCAAAGTGAAAGAACAAACTATTGACATTGATAGTAAGTCAATTAATTTT
------------------------------------------------------------
------------------------------------------------------------
------------------------------------------------------------
------------------------------------------------------------
------------------------------------------------------------
------------------------------------------------------------
------------------------------------------------------------
------------------------------------------------------------
------------------------------------------------------------
------------------------------------------------------------

------------------------------------------------------------
------------------------------------------------------------
------------------------------------------------------------
------------------------------------------------------------
------------------------------------------------------------
------------------------------------------------------------
------------------------------------------------------------
------------------------------------------------------------
------------------------------------------------------------
------------------------------------------------------------
------------------------------------------------------------
------
>CM051264_Artemia_sinica_Glp1_v1
------------------------------------------------------------
------------------------------------------------------------
------------ATGGAC---AGAGGA---------------------ATT---AAGAAC
AAATTGAGA---GTATCTAGTCCTATCTTCAGAGAGTTCGTTGCAGAATGTCTTGGAACT
TTTATCCTAGTGGCATTTGGAGATGCATGTGTTGCACAGTCGGTCCTGTCGGAAAGGGAA
AAGGGAGATTTCTTCTCTATAAATTGGGGCTGGGGGCTAGGTGGCATGCTTGCTGTACTT
ATCTGTGGTGGAGTATCAGGGGCTCATTTGAACCCAGCTGTTACTCTTGCCATGGCCATA
GTTGGTAAACATCCATGGAAAAAAGTGTTGCATTATATGGCGGGCCAATACCTGGGAGGA
TTTCTAGCTGCTGCAGTTGTTTTAGGCGTATACAGTGAGGGAATATATTATTACGAAGAT
CAAGTTGGCAATGGTACCCTAAACATCGGCAAC---------------------------
---ACGGCTGGTATTTTTGCGACATACCCTTATATGTGGACAACGACATTAGGAGGCTTT
GCTGACCAGATTTTCGGAACCATGACTCTTCTTATTGCTGTTTGTGCTATAACAGATGAA
AAGAACATGCAGATTTCC------------AAGCCTCTGATTCCTCTATATGTTGGCTTT
ACCATTTTGGCAATTGGTGTTTGCTTTGGAGCAAATTGTGGCTACGCCATCAACCCAGCA
AGAGATTTGTCACCACGTATTATTACTCTGATTGCAGGATGGGGCACTGGCACATTCACG
GTGAACAACTAT---TGGTTTTGGGTGCCCATTGTAGGTCCTCACGTTGGTGCCATTCTT
GGTGTTTTTATTTAC---ATCCTTTGTATAGAAGTACATTGGCCCGAAGATGACAAAAAT
---GAGTCATTA---GCG------------------------------------------
------AAATCCAAAGTGAAAGAACAAACTATTGACATTGATAGTAAGTCAATTAATTTT
------------------------------------------------------------
------------------------------------------------------------
------------------------------------------------------------
------------------------------------------------------------
------------------------------------------------------------
------------------------------------------------------------
------------------------------------------------------------
------------------------------------------------------------
------------------------------------------------------------
------------------------------------------------------------
------------------------------------------------------------
------------------------------------------------------------
------------------------------------------------------------
------------------------------------------------------------
------------------------------------------------------------
------------------------------------------------------------

```
------------------------------------------------------------
------------------------------------------------------------
------------------------------------------------------------
------------------------------------------------------------
------------------------------------------------------------
------
>JAQQPU010002487_42379_07693_Artemia_sp_Kazakhstan_Glp1_v2
ATGGGTTTCAGTGAGGAAGTTGCTAAAGAGTACTATGATTACTACTATAAAAGCTCGACA
GACCCCGACGGCACAGCAAATAAGTATCCATCAAGCTTGAGTGCTTACAGTACAAGCACA
GATATATTCCAAATGGAC---AGAGGA---------------------ATT---AAGAAC
AAATTGAGA---GTATCTAGTCCTATCTTCAGAGAGTTTGTTGCAGAATGTCTTGGAACT
TTTATCCTAGTGGCATTTGGAGATGCATGTGTTGCACAGTCGGTCCTGTCTAAAGGGGAA
AAGGGAGATTTCTTCTCCATAAATTGGGGCTGGGGGCTAGGTGGCATGCTTGCTGTACTT
ATCTGTGGTGGAGTATCAGGGGCTCACTTGAACCCAGCTGTTACTCTTGCCATGGCCATA
GTTGGTAAACATCCATGGAAAAAAGTGTTGCATTATATGGCAGGCCAATATCTGGGAGGA
TTTCTAGCTGCTGCAGTTGTCTTAGGCGTATACAGTGAGGGAATATATTATTACGAAGAT
CAAGTTGGCAATGGTACCCTAAACATCGGTAAC---------------------------
---ACGGCTGGTATTTTTGCGACATACCCTTATATGTGGACAACGACATTAGGAGGCTTT
GCTGACCAGATTTTCGGAACCATGACTCTTCTTATTGCTGTTTGTGCTATAACAGATGAA
AAGAACATGCAGATTTCC------------AAGCCTCTGATTCCTCTATATGTTGGCTTT
ACCATTTTGGCAATTGGTGTTTGCTTTGGAGCAAATTGCGGCTACGCCATCAACCCAGCA
AGAGATTTGTCACCACGTATTATTACTCTGATTGCAGGATGGGGCACTGGCACATTCACG
GTGAACAACTAT---TGGTTTTGGGTGCCCATTGTAGGTCCTCACGTTGGTGCCATTCTT
GGTGTTTTTATTTAC---ATCCTTTGTATAGAAGTACATTGGCCCGAAGATGACAAAAAT
---GAGTCATTA---GCG------------------------------------------
------AAATCCAAAGTGAAAGAACAAACTATTGACATTGATAGTAAGTCAATTAATTCT
------------------------------------------------------------
------------------------------------------------------------
------------------------------------------------------------
------------------------------------------------------------
------------------------------------------------------------
------------------------------------------------------------
------------------------------------------------------------
------------------------------------------------------------
------------------------------------------------------------
------------------------------------------------------------
------------------------------------------------------------
------------------------------------------------------------
------------------------------------------------------------
------------------------------------------------------------
------------------------------------------------------------
------------------------------------------------------------
------------------------------------------------------------
------------------------------------------------------------
------------------------------------------------------------
------------------------------------------------------------
------------------------------------------------------------
------
```

```
>JAQQPU010002487_42379_07693_Artemia_sp_Kazakhstan_Glp1_v1
------------------------------------------------------------
------------------------------------------------------------
------------ATGGAC---AGAGGA---------------------ATT---AAGAAC
AAATTGAGA---GTATCTAGTCCTATCTTCAGAGAGTTTGTTGCAGAATGTCTTGGAACT
TTTATCCTAGTGGCATTTGGAGATGCATGTGTTGCACAGTCGGTCCTGTCTAAAGGGGAA
AAGGGAGATTTCTTCTCCATAAATTGGGGCTGGGGGCTAGGTGGCATGCTTGCTGTACTT
ATCTGTGGTGGAGTATCAGGGGCTCACTTGAACCCAGCTGTTACTCTTGCCATGGCCATA
GTTGGTAAACATCCATGGAAAAAAGTGTTGCATTATATGGCAGGCCAATATCTGGGAGGA
TTTCTAGCTGCTGCAGTTGTCTTAGGCGTATACAGTGAGGGAATATATTATTACGAAGAT
CAAGTTGGCAATGGTACCCTAAACATCGGTAAC---------------------------
---ACGGCTGGTATTTTTGCGACATACCCTTATATGTGGACAACGACATTAGGAGGCTTT
GCTGACCAGATTTTCGGAACCATGACTCTTCTTATTGCTGTTTGTGCTATAACAGATGAA
AAGAACATGCAGATTTCC------------AAGCCTCTGATTCCTCTATATGTTGGCTTT
ACCATTTTGGCAATTGGTGTTTGCTTTGGAGCAAATTGCGGCTACGCCATCAACCCAGCA
AGAGATTTGTCACCACGTATTATTACTCTGATTGCAGGATGGGGCACTGGCACATTCACG
GTGAACAACTAT---TGGTTTTGGGTGCCCATTGTAGGTCCTCACGTTGGTGCCATTCTT
GGTGTTTTTATTTAC---ATCCTTTGTATAGAAGTACATTGGCCCGAAGATGACAAAAAT
---GAGTCATTA---GCG------------------------------------------
------AAATCCAAAGTGAAAGAACAAACTATTGACATTGATAGTAAGTCAATTAATTCT
------------------------------------------------------------
------------------------------------------------------------
------------------------------------------------------------
------------------------------------------------------------
------------------------------------------------------------
------------------------------------------------------------
------------------------------------------------------------
------------------------------------------------------------
------------------------------------------------------------
------------------------------------------------------------
------------------------------------------------------------
------------------------------------------------------------
------------------------------------------------------------
------------------------------------------------------------
------------------------------------------------------------
------------------------------------------------------------
------------------------------------------------------------
------------------------------------------------------------
------------------------------------------------------------
------------------------------------------------------------
------------------------------------------------------------
------
>JAYKOS010000005_Artemia_franciscana_Bett_Glp1_v2
ATGGGGTTCAGTGAGGAAGTTGCTAAAGAGTACTATGATTACTACTATAAAAGCTCGACA
GACCCCGACGGCACAGCAAATAAGTATCCATCAAGCTTGAGTGCTTACAGTACAAGCACA
GATATATTCCAAATGGAC---AAAGGA---------------------ATT---AAGAAC
AAATTGAGG---GTATCTAGTCCTATCTTCAGGGAGTTTGTTGCAGAGTGTCTCGGGACT
TTTATCCTAGTGGCGTTTGGAGATGCATGTGTTGCTCAGTCGGTCCTGTCGAAAGGGGAA
```

AAGGGAGATTTCTTCTCGATAAACTGGGGCTGGGGTTTAGGTGGCATGCTTGCTGTACTT
ATCTGTGGTGGAGTATCAGGGGCTCATTTGAACCCAGCTGTTACTCTTGCCATGGCCGTA
GTTGGTAAACATCCATGGAAAAAAGTGTTGCATTACATGGCAGGTCAATATCTGGGAGGA
TTTTTAGCTGCTGCAGTTGTTTTAGGCGTATACAGTGAGGGAATATATTATTACGAAGAT
CAAGTTGGCAACGGTACCCTAAACATTGGCAAT---------------------------
---ACGGCAGGTATTTTTGCAACGTACCCTTATATGTGGACAACAACATTAGGAGGCTTA
GCTGACCAGATCTTCGGAACCATGACTCTTCTTATAGCTGTTTGTGCTATAACAGATGAA
AAGAACATGCAGATCTCC------------AAGCCTCTGATCCCTCTATATGTTGGCTTC
ACCATTTTGGCAATTGGCGTTTGCTTTGGAGCAAATTGCGGCTATGCCATCAACCCAGCA
AGAGACTTGTCACCACGTATCATTACTCTGATTGCAGGATGGGGCTCAGCCACATTCACG
------------------------------------------------------------
------------------------------------------------------------
------------------------------------------------------------
------------------------------------------------------------
------------------------------------------------------------
------------------------------------------------------------
------------------------------------------------------------
------------------------------------------------------------
------------------------------------------------------------
------------------------------------------------------------
------------------------------------------------------------
------------------------------------------------------------
------------------------------------------------------------
------------------------------------------------------------
------------------------------------------------------------
------------------------------------------------------------
------------------------------------------------------------
------------------------------------------------------------
------------------------------------------------------------
------------------------------------------------------------
------------------------------------------------------------
------------------------------------------------------------
------------------------------------------------------------
------------------------------------------------------------
------------------------------------------------------------
------
>JAYKOS010000005_Artemia_franciscana_Bett_Glp1_v1
------------------------------------------------------------
------------------------------------------------------------
------------ATGGAC---AAAGGA---------------------ATT---AAGAAC
AAATTGAGG---GTATCTAGTCCTATCTTCAGGGAGTTTGTTGCAGAGTGTCTCGGGACT
TTTATCCTAGTGGCGTTTGGAGATGCATGTGTTGCTCAGTCGGTCCTGTCGAAAGGGGAA
AAGGGAGATTTCTTCTCGATAAACTGGGGCTGGGGTTTAGGTGGCATGCTTGCTGTACTT
ATCTGTGGTGGAGTATCAGGGGCTCATTTGAACCCAGCTGTTACTCTTGCCATGGCCGTA
GTTGGTAAACATCCATGGAAAAAAGTGTTGCATTACATGGCAGGTCAATATCTGGGAGGA
TTTTTAGCTGCTGCAGTTGTTTTAGGCGTATACAGTGAGGGAATATATTATTACGAAGAT
CAAGTTGGCAACGGTACCCTAAACATTGGCAAT---------------------------
---ACGGCAGGTATTTTTGCAACGTACCCTTATATGTGGACAACAACATTAGGAGGCTTA

GCTGACCAGATCTTCGGAACCATGACTCTTCTTATAGCTGTTTGTGCTATAACAGATGAA
AAGAACATGCAGATCTCC------------AAGCCTCTGATCCCTCTATATGTTGGCTTC
ACCATTTTGGCAATTGGCGTTTGCTTTGGAGCAAATTGCGGCTATGCCATCAACCCAGCA
AGAGACTTGTCACCACGTATCATTACTCTGATTGCAGGATGGGGCTCAGCCACATTCACG
------------------------------------------------------------
------------------------------------------------------------
------------------------------------------------------------
------------------------------------------------------------
------------------------------------------------------------
------------------------------------------------------------
------------------------------------------------------------
------------------------------------------------------------
------------------------------------------------------------
------------------------------------------------------------
------------------------------------------------------------
------------------------------------------------------------
------------------------------------------------------------
------------------------------------------------------------
------------------------------------------------------------
------------------------------------------------------------
------------------------------------------------------------
------------------------------------------------------------
------------------------------------------------------------
------------------------------------------------------------
------------------------------------------------------------
------------------------------------------------------------
------------------------------------------------------------
------------------------------------------------------------
------------------------------------------------------------
------
>JAKZAO010000003_Branchinecta_lindahli_Glp
------------------------------------------------------------
------------------------------------------------------------
------------ATGGAA---AAATCA---------------------ATC---CGAAAC
AAGTTGCGG---ATCACCAGTCCGGTCTTCCGAGAGTTTGTTGCGGAGTTCCTGGGAACT
TTCATTCTTGTGGTCTTTGGCGATGGGTCCGTGGCGCAGTCAGTCTTAACAGGGGCGGAG
AAAGGCGATTTCTTTTCCATTAACTGGGGATGGGGTGTCGCAGTCATGTTGGGAGTTCTT
GTCTCTGGAGGTGTTTCCGGTGGCCACTTGAACCCGGCCGTGACACTTGCAATGGCCATT
GTTGGAAAGCATCCGTGGAAGAAGGTACTGCATTACTGGTTTGCCCAGTACCTTGGGGGT
TTTGTTGCAGCCGCGTGTGTCCTTGGAATCTACAGCGAGGGAATCTACTTTTTCGAAGGA
------AGCGAT---CATCTCACAGTCAATAAG---------------------------
---ACAGCTGGAATTTTCGCTACATACCCTCATGTTTGGACGTCAACTGCTGGAGGATTG
GCTGATCAGATCCTTGGAACGATGCTACTGCTTCTGGTGATTTGCGCAATTACCGACCCA
CGAAACATGAATGTTGCA------------AAAAATCTCATCCCGCTGTATGTTGGATTC
ACAATTCTCGGGATCGGGGTCTGCTTTGGAGTCAACTGCGGCTATGCCCTAAACCCAGCA
AGGGACTTGGCTCCCCGATTGTTGACGCTGATTGGCGGATGGGGCCCTGAAGTATTTTCG
GCCAATAACTTC---TGGTTCTGGGTTCCAGTTGTCGGTCCGCACGTTGGTGCCATCCTC
GGTGTCTTTATTTAT---GTTTTGTGCATAGAAATCCATTGGCCTGAAGACGATGATTTG

---GACGCA---------------------------------------------------
------AAAGCAAAGGCGAAAGAGCAGACAATAGATATGGAAAGTAGG------------
------------------------------------------------------------
------------------------------------------------------------
------------------------------------------------------------
------------------------------------------------------------
------------------------------------------------------------
------------------------------------------------------------
------------------------------------------------------------
------------------------------------------------------------
------------------------------------------------------------
------------------------------------------------------------
------------------------------------------------------------
------------------------------------------------------------
------------------------------------------------------------
------------------------------------------------------------
------------------------------------------------------------
------------------------------------------------------------
------------------------------------------------------------
------------------------------------------------------------
------------------------------------------------------------
------------------------------------------------------------
------------------------------------------------------------
------
>JAKZAP010000014_Branchinecta_lynchi_Glp
------------------------------------------------------------
------------------------------------------------------------
------------ATGGAG---AAATCC---------------------ATC---CGAAAC
AAGTTGCGG---ATCACCAGTCCGGTCTTCCGGGAGTTTGTTGCGGAATTCCTGGGAACT
TTCATTCTTGTGGTCTTTGGTGATGGGTCCGTGGCGCAGTCAGTCTTGACAGGGGCGGAG
AAAGGCGATTTCTTTTCCATTAACTGGGGATGGGGCGTTGCAGTCATGTTGGGAGTTCTT
GTCTCTGGAGGCGTTTCCGGTGGCCACTTGAACCCGGCCGTCACGCTCGCAATGGCCATT
GTTGGAAAGCATCCATGGAAGAAGCTCCTGCACTACTGGTTGGCCCAGTACCTTGGAGCT
TTCGTTGCAGCCGCTTGTGTCCTTGGAATATATAGCGAGGGAATCTACTTTTTCGAAGGA
------GGCGAT---CATCTCACAGTCAATAAG---------------------------
---ACAGCTGGAATTTTCGCTACGTACCCTCATGTCTGGACGTCAACTGCTGGAGGGTTA
GCGGATCAGATCCTTGGAACGATGCTACTGCTTCTGGTAATATGCGCCATTACCGACCCG
CGAAATATGAATGTTGCA------------AAAAATCTCATCCCCCTCTATGTTGGATTC
ACAATTCTTGGAATCGGTGTCTGCTTTGGAGTCAACTGCGGCTATGCCCTAAACCCAGCA
AGGGACTTGGCTCCCCGACTGTTGACGCTGATAGGCGGATGGGGCCCTGAAGTCTTTTCG
GCCAATAACTTC---TGGTTCTGGGTTCCAGTTGTCGGTCCGCACGTCGGTGCCATTCTC
GGTGTCTTCATTTAT---GTTTTGTGCATAGAAATCCATTGGCCTGAAGACGATGATTTA
---GACGCA---------------------------------------------------
------AAAGCCAAGGCCAAAGAACAGACAATAGATATGGAAAGTAGG------------
------------------------------------------------------------
------------------------------------------------------------
------------------------------------------------------------
------------------------------------------------------------

------------------------------------------------------------
------------------------------------------------------------
------------------------------------------------------------
------------------------------------------------------------
------------------------------------------------------------
------------------------------------------------------------
------------------------------------------------------------
------------------------------------------------------------
------------------------------------------------------------
------------------------------------------------------------
------------------------------------------------------------
------------------------------------------------------------
------------------------------------------------------------
------------------------------------------------------------
------------------------------------------------------------
------------------------------------------------------------
------------------------------------------------------------
------
>JAUPMV010000020_Branchinecta_sandiegonensis_Glp
------------------------------------------------------------
------------------------------------------------------------
------------ATGGAG---AAATCA---------------------ATC---CGAAAC
AAGTTGCGG---ATCACCAGTCCGGTCTTCCGAGAGTTTGTTGCGGAATTCCTGGGAACT
TTCATTCTTGTGGTCTTTGGTGATGGGTCCGTGGCGCAGTCAGTCTTGACAGGGGCAGAG
AAAGGCGATTTCTTTTCCATTAACTGGGGATGGGGCGTTGCAGTCATGTTGGGAGTTCTT
GTCTCGGGAGGCGTTTCCGGTGGCCACTTGAACCCGGCCGTCACACTTGCAATGGCCATT
GTTGGAAAGCATCCATGGAAGAAAGTCCTGCACTACTGGTTGGCCCAGTACCTTGGGGCT
ATCGTTGCAGCCGCTTGTGTCCTTGGAATATACAGTGAGGGAATCTACTTTTTCGAAGGA
------GGCGAT---CATCTCACAGTCAATAAG---------------------------
---ACAGCTGGAATTTTCGCTACATACCCTCATGTTTGGACGTCAACTGCTGGAGGATTG
GCGGATCAGATCCTTGGAACGATGCTACTGCTTCTGGTAATATGCGCCATTACAGACCCG
CGAAACATGAATGTTGCA------------AAAAATCTCATCCCGCTCTATGTTGGATTC
ACAATCCTCGGAGTCGGTGTCTGCTTTGGAGTCAACTGCGGCTATGCCCTAAACCCAGCA
AGGGACTTGGCTCCCCGACTGTTGACGCTGATTGGTGGATGGGGCCCTGAAGTCTTTTCG
GCCAATAACTTC---TGGTTCTGGGTTCCAGTTGTTGGTCCGCATGTCGGTGCCATTCTC
GGTGTCTTCATTTAT---GTTTTGTGCATAGAAATCCATTGGCCTGAAGATGATGATTTG
---GACGCG---------------------------------------------------
------AAAGCCAAGGCGAAAGAACAGACAATAGATATGGAAAGTAGG------------
------------------------------------------------------------
------------------------------------------------------------
------------------------------------------------------------
------------------------------------------------------------
------------------------------------------------------------
------------------------------------------------------------
------------------------------------------------------------
------------------------------------------------------------
------------------------------------------------------------
------------------------------------------------------------

------------------------------------------------------------
------------------------------------------------------------
------------------------------------------------------------
------------------------------------------------------------
------------------------------------------------------------
------------------------------------------------------------
------------------------------------------------------------
------------------------------------------------------------
------------------------------------------------------------
------------------------------------------------------------
------------------------------------------------------------
------
>GCBP01024579_Eubranchipus_grubii_Glp
------------------------------------------------------------
------------------------------------------------------------
------------ATGGAG---AAATCA---------------------GTA---CGAAGC
AAGTTGAGG---ATCAGTAGTCCCATCTTCAGGGAGTTTGTGGCGGAGTTTCTAGGAACT
TTTATTCTTGTTGTTCTTGGCGACGGCAGTGTTGCTCAGTCCGTCCTTTCAGACAAAGAG
AAGGGAGACTTTTTCTCCATCAACTGGGGCTGGGGGATTGCCGTCATGCTGGCAGTCCTT
ATCTCGGGTGGCGTATCTGGTGGTCACTTAAACCCTGCGGTGACAGTAGCAATGGCAGTC
GTTGGAAAGCATCCTTGGAAGAAAGTTCTTCACTATTGCTTGGCGCAGTACCTCGGCGCC
TTCTTGGCCGCTGCATGCGTCCTTGGAATTTACAGTGAGGGAATTTACTACTTCGAACTA
GCCCACGGGGATGGAACATTGAATGTCACCAAC---------------------------
---ACAGCTGGCATTTTTGCAACATACCCTTACCTTTGGACGACTCTAACAGGTGGACTT
GCTGACCAGATTCTTGGTACCATGATCCTCTTGCTCGTGATATGTGCCATTTCAGACAAT
CGAAATATGCAAGTAGCG------------AAGCCTCTTATCCCGCTGTACGTCGGATTC
ACTATTCTTGGAATTGGGGTATGCTTTGGTGCCAACTGCGGCTATGCTGTGAACCCCGCG
AGAGATTTGGCACCGAGGTTACTGACCCTGATTGCCGGTTGGGGACCTGAAGTTATGACA
ATAAATGACTAC---TGGTTCTGGGTTCCGATCGTTGGCCCCCACATTGGTGCGATTCTC
GGGGTACTCATATAT---TCCCTTTGCATAGAAGTTCACTGGCCAGAAGATGATGAAACT
---GAAGCCGAA---GCA------------------------------------------
------AGAGCAAAAGCAAAGGAACAGACAATTGACATGGAAAGTTTA------------
------------------------------------------------------------
------------------------------------------------------------
------------------------------------------------------------
------------------------------------------------------------
------------------------------------------------------------
------------------------------------------------------------
------------------------------------------------------------
------------------------------------------------------------
------------------------------------------------------------
------------------------------------------------------------
------------------------------------------------------------
------------------------------------------------------------
------------------------------------------------------------
------------------------------------------------------------
------------------------------------------------------------
------------------------------------------------------------

------------------------------------------------------------
------------------------------------------------------------
------------------------------------------------------------
------------------------------------------------------------
------------------------------------------------------------
------
>WP_032330355_Escherichia_coli_GlpF
------------------------------------------------------------
------------------------------------------------------------
------------------------------------------------------ATGAGT
CAA------------ACATCAACCTTGAAAGGCCAGTGCATTGCTGAATTCCTCGGTACC
GGGTTGTTGATTTTCTTCGGTGTGGGTTGCGTTGCAGCACTAAAAGTCGCT---GGTGCG
TCTTTTGGTCAGTGGGAAATCAGTGTCATTTGGGGACTGGGGGTGGCAATGGCCATCTAC
CTGACCGCAGGGATTTCCGGCGCGCATCTTAATCCCGCTGTTACCATTGCATTGTGGCTG
TTTGCCTGTTTCGACAAGCGCAAAGTTATTCCTTTTATCGTTTCACAAGTTGCCGGCGCT
TTCTGTGCTGCGGCTTTAGTTTACGGGCTTTACTACAATTTATTTTTCGACTTCGAGCAG
ACTCATCACATTGTTCGCGGCAGCGTT---GAA------AGTGTT---------------
GATCTGGCTGGCACTTTCTCTACTTACCCTAATCCTCATATCAATTTTGTGCAGGCTTTC
GCAGTTGAGATGGTGATTACCGCTATTCTGATGGGGCTGATCCTGGCGTTAACGGACGAT
GGCAACGGTGTACCACGC------------GGCCCTTTGGCTCCCTTGCTGATTGGTCTA
CTGATTGCGGTCATTGGCGCATCTATGGGCCCATTGACAGGTTTTGCCATGAACCCAGCG
CGTGACTTCGGTCCGAAAGTCTTTGCCTGGCTGGCGGGCTGGGGCAATGTCGCCTTTACC
GGCAGAGACATTCCTTACTTCCTGGTGCCGCTTTTCGGCCCTATCGTTGGCGCGATTGTA
GGTGCATTTGCCTAC---CGCAAACTGATTGGTCGCCATTTGCCTTGCGATATCTGTGTT
---GTGGAAGAA---AAGGAAACCACAACTCCTTCAGAACAAAAA------GCTTCG---
------CTG---------------------------------------------------
------------------------------------------------------------
------------------------------------------------------------
------------------------------------------------------------
------------------------------------------------------------
------------------------------------------------------------
------------------------------------------------------------
------------------------------------------------------------
------------------------------------------------------------
------------------------------------------------------------
------------------------------------------------------------
------------------------------------------------------------
------------------------------------------------------------
------------------------------------------------------------
------------------------------------------------------------
------------------------------------------------------------
------------------------------------------------------------
------------------------------------------------------------
------------------------------------------------------------
------------------------------------------------------------
------------------------------------------------------------
------------------------------------------------------------
------
