# Supplementary material for: Co-opted and canonical glycerol channels play a major role during anhydrobiosis of an extremophile crustacean
Source: BMC Biol. 2025 Jun 3;23:151. doi: 10.1186/s12915-025-02262-3 (PMC12135271; doi:10.1186/s12915-025-02262-3)
Supplement: Supplementary file 6 — Additional file 6: Dataset S3. Amino acid alignment for Fig. S3C. [file 12915_2025_2262_MOESM6_ESM.pdf]

```
>PQ469251_Artemia_franciscana_Glp4_v1
--------------------------------------------MDKKIKSKLRVSSPIF
REFVAECLGTFFLVMIGDASVAQSVLSKEEKGDFFSINWGFGMGAMLAVLICGGVSGAHL
NPAVTLAMAIVGKHPWKKVLHYMAGQYLGGFLAAAVVLGVYSEGIYYYEDQVGNGTLNIG
NTAGIFATYPYLWTTTLGGFVDQIVGTMVLLIAICAITDEKNMQISKPLIPLYVGFTFVA
IGVAFGANCGYAINPARDLSPRIITLIAGWGSATFTVNDYWFWVPIVGPHVGAILGVFIY
ILCIEMHWPEDDKSESLAKSKVEKQTIDIDSKSINF
>JAVRJZ010000021_Artemia_franciscana_Jo_Glp4_v1
--------------------------------------------MDKKIKSKLRVSSPIF
REFVAECLGTFFLVMIGDASVAQSVLSKEEKGDFFSINWGFGMGAMLAVLICGGVSGAHL
NPAVTLAMAIVGKHPWKKVLHYMAGQYLGGFLAAAVVLGVYSEGIYYYEDQVGNGTLNIG
NTAGIFATYPYLWTTTLGGFVDQIVGTMVLLIAICAITDEKNMQISKPLIPLYVGFTFVA
IGVAFGANCGYAINPARDLSPRIITLIAGWGSATFTVNDYWFWVPIVGPHVGAILGVFIY
ILCIEMHWPEDDKSESLAKSKVEEQTIDIDSKSINF
>JAYKOS010000005_Artemia_franciscana_Bett_Glp4_v1
--------------------------------------------MDKKIKSKLRVSSPIF
REFVAECLGTFFLVMIGDASVAQSVLSKEEKGDFFSINWGFGMGAMLAVLICGGVSGAHL
NPAVTLAMAIVGKHPWKKVLHYMAGQYLGGFLAAAVVLGVYSEGIYYYEDQVGNGTLNIG
NTAGIFATYPYLWTTTLGGFVDQIVGTMVLLIAICAITDEKNMQISKPLIPLYVGFTFVA
IGVAFGANCGYAINPARDLSPRIITLIAGWGSATFTVNDYWFWVPIVGPHVGAILGVFIY
ILCIEMHWPEDDKSESLAKSKVEEQTIDIDSKSINF
>Afra2B006330T5_Artemia_franciscana_Glp4_v2
--------------------------------------------MDKKIKSKLRVSSPIF
REFVAECLGTFFLVMIGDASVAQSVLSKEEKGDFFSINWGFGMGAMLAVLICGGVSGAHL
NPAVTLAMAIVGKHPWKKVLHYMAGQYLGGFLAAAVVLGVYSEGIYYYEDQVGNGTLNIG
NTAGIFATYPYLWTTTLGGFVDQIVGTMVLLIAICAITDEKNMQISKPLIPLYVGFTFVA
IGVAFGANCGYAINPARDLSPRIITLIAGWGSATFTVNDYWFWVPIVGPHVGAILGVFIY
ILCIEMHWPEDDKSESLAKSKVEEQTIDIDS-----
>KAK2705112_Artemia_franciscana_Glp4_v2
--------------------------------------------MDKKIKSKLRVSSPIF
REFVAECLGTFFLVMIGDASVAQSVLSKEEKGDFFSINWGFGMGAMLAVLICGGVSGAHL
NPAVTLAMAIVGKHPWKKVLHYMAGQYLGGFLAAAVVLGVYSEGIYYYEDQVGNGTLNIG
NTAGIFATYPYLWTTTLGGFVDQIVGTMVLLIAICAITDEKNMQISKPLIPLYVGFTFVA
IGVAFGANCGYAINPARDLSPRIITLIAGWGSATFTVNDYWFWVPIVGPHVGAILGVFIY
ILCIEMHWPEDDKSESLAKSKVEEQTIDIDS-----
>PQ469252_Artemia_franciscana_Glp4_v2
--------------------------------------------MDKKIKSKLRVSSPIF
REFVAECLGTFFLVMIGDASVAQSVLSKEEKGDFFSINWGFGMGAMLAVLICGGVSGAHL
NPAVTLAMAIVGKHPWKKVLHYMAGQYLGGFLAAAVVLGVYSEGIYYYEDQVGNGTLNIG
NTAGIFATYPYLWTTTLGGFVDQIVGTMVLLIAICAITDEKNMQISKPLIPLYVGFTFVA
IGVAFGANCGYAINPARDLSPRIITLIAGWGSATFTVNDYWFWVPIVGPHVGAILGVFIY
ILCIEMHWPEDDKSESLAKSKVEEQTIDIDS-----
>GJHQ01003151_Artemia_franciscana_Glp4_v2
--------------------------------------------MDKKIKSKLRVSSPIF
REFVAECLGTFFLVMIGDASVAQSVLSKEEKGDFFSINWGFGMGAMLAVLICGGVSGAHL
NPAVTLAMAIVGKHPWKKVLHYMAGQYLGGFLAAAVVLGVYSEGIYYYEDQVGNGTLNIG
NTAGIFATYPYLWTTTLGGFVDQIVGTMVLLIAICAITDEKNMQISKPLIPLYVGFTFVA
IGVAFGANCGYAINPARDLSPRIITLIAGWGSATFTVNDYWFWVPIVGPHVGAILGVFIY
```

ILCIEVHWPEDDKSESLAKSKVEEQTIDIDS-----
>CM051264_Artemia_sinica_Glp4_v1
--MDKQIRNKLRISSPIF
REFVAECLGTFFLVTFGDASVAQSVLSKEEKGDFFSINWGFGMGAMLAVLICGGVSGAHL
NPAVTLAMAIVGKHPWKKVLHYMAGQYLGGFLAAAVVLGVYSEGIYYYEDQVGNGTLNIG
NTAGIFATYPYLWTTTLGGLVDQIVGTMVLLIAICAITDEKNMQISKPLIPLYVGFTFVA
IGVAFGANCGYAINPARDLSAYIITLIAGWGTTTFTVNDFWFWVPIVGPHVGAILGVFIY
ILCIEVHWPEDDKNESLTKSKVKEQTIDIDSKSINS
>JAQQPU010016311_47589_21864_Artemia_sp_Kazakhstan_Glp4_v1
--MDKQIRNKLRISSPIF
REFVAECLGTFFLVTFGDASVAQSVLSKEEKGDFFSINWGFGMGAMLAVLICGGVSGAHL
NPAVTLAMAIVGKHPWKKVLHYMAGQYLGGFLAAAVVLGVYSEGIYYYEDHVGNGTLNIG
NTAGIFATYPYLWTTTLGGLVDQIVGTMVLLIAICAITDEKNMQISKPLIPLYVGFTFVA
IGVAFGANCGYAINPARDLSPRIITLIAGWGTTTFTVNDYWFWVPIVGPHVGAILGVFIY
ILCIEVHWPEDDKNESLAKSKVKEQTIDIDSKSINS
>JAVRJZ010000021_Artemia_franciscana_Jo_Glp3
--MDKGIKNKLRVSSPIF
REFAAECLGTLILVVFGDAGVAQSVLSKGEKGDFFSTNWGWGLGGMLAVLICGGVSGAHL
NPALTFAMAIVGKHPWKKVLHYMAGQYLGGLIAAAVVLGVYSEGIYYYEDEVGNGTLNIG
NTAGIFATYPYKWTTTLGGLADAIVGTMVLLIVICAITDEKNMQISKPLIPLYVGFTFLA
IGVCFGVNSGCAINPARDLSPRIITLIAGWGTGTFTVNDYWFWVPIVGPHVGAILGVFIY
ILCIEVHWPEDDKNESLAKSKVKDKTIDIDSKLNNF
>XM_065710899_Artemia_franciscana_Glp3
--MDKGIKNKLRVSSPIF
REFAAECLGTLILVVFGDAGVAQSVLSKGEKGDFFSTNWGWGLGGMLAVLICGGVSGAHL
NPALTFAMAIVGKHPWKKVLHYMAGQYLGGLIAAAVVLGVYSEGIYYYEDEVGNGTLNIG
NTAGIFATYPYKWTTTLGGLADAIVGTMVLLIVICAITDEKNMQISKPLIPLYVGFTFLA
IGVCFGVNSGCAINPARDLSPRIITLIAGWGTGTFTVNDYWFWVPIVGPHVGAILGVFIY
ILCIEVHWPEDDKNESLAKSKVKDKTIDIDS-L---
>Afra2B019209T1_Artemia_franciscana_Glp3

--------------VFGDAGVAQSVLSKGEKGDFFSTNWGWGLGGMLAVLICGGVSGAHL
NPALTFAMAIVGKHPWKKVLHYMAGQYLGGLIAAAVVLGVYSEGIYYYEDEVGNGTLNIG
NTAGIFATYPYKWTTTLGGLADAIVGTMVLLIVICAITDEKNMQISKPLIPLYVGFTFLA
IGVCFGVNSGCAINPARDLSPRIITLIAGWGTGTFTVNDYWFWVPIVGPHVGAILGVFIY
ILCIEVHWPEDDKNESLAKSKVKDKTIDIDS-----
>JAYKOS010000005_Artemia_franciscana_Bett_Glp3
--MDKGIKNKLRVSSPIF
REFAAECLGTFILVVFGDAGIAQSVLSKGEKGDFFSTNWGWGLGGMLAVLICGGVSGAHL
NPALTFAMAIVGKHPWKKVLHYMAGQYLGGLIAAAVVLGVYSEGIYYYEDEVGNGTLNIG
NTAGIFATYPYKWTTTLGGLADAIVGTMVLLIVICAITDEKNMQISKPLIPLYVGFTFLA
IGVCFGVNSGCAINPARDLSPRIITLIAGWGTGTFTVNDYWFWVPIVGPHVGAILGVFIY
ILCIEVHWPEDDKNESLAKSKVKDKTIDIDSKLNNF
>PQ469254_Artemia_franciscana_Glp2_v1
--MDRGIKNKLKVSSPIF
REFVAECLGTFILVAFGDACVAQSVLSKGEKGDFFSINWGWGLGGMLAVLICGGVSGAHL
NPAVTLAMAVVGKHPWKKVLHYMAGQYLGGLIAAAVVLGVYCEGIYYYEDQVGNGTLSIG
NTAGIFATYPYMWATTLGGLDDQIFGTMTVLIAVCAITDEKNRQISKPLIPLYVGFTILA

```
IGVCFGANCGYAINPARDLSPRIITLIAGWGTGTFTVNDYWFWVPIVGPHVGAILGVFIY
ILCIEVHWPEDDKNESLTKSEVKEQTIDIDS-L---
>Afra2B004009T7_Artemia_franciscana_Glp2_v1
--------------------------------------------MDRGIKNKLKVSSPIF
REFVAECLGTFILVAFGDACVAQSVLSKGEKGDFFSINWGWGLGGMLAVLICGGVSGAHL
NPAVTLAMAVVGKHPWKKVLHYMAGQYLGGLIAAAVVLGVYSEGIYYYEDQVGNGTLNIG
NTAGIFATYPYMWTTTLGGLADQIFGTMTLLIAVCAITDEKNMQISKPLIPLYVGFTILA
IGVCFGANCGYAINPARDLSPRIITLIAGWGTGTFTVNDYWFWVPIVGPHVGAILGVFIY
ILCIEVHWPEDDKNESLTKSKVKEQTIDIDS-L---
>Afra2B004009T8_Artemia_franciscana_Glp2_v2
MGFSEEVAKEYYDYYYKSSTDPDGTANKYPSSLSAYSTNTDIFQMDRGIKNKLKVSSPIF
REFVAECLGTFILVAFGDACVAQSVLSKGEKGDFFSINWGWGLGGMLAVLICGGVSGAHL
NPAVTLAMAVVGKHPWKKVLHYMAGQYLGGLIAAAVVLGVYSEGIYYYEDQVGNGTLNIG
NTAGIFATYPYMWTTTLGGLADQIFGTMTLLIAVCAITDEKNMQISKPLIPLYVGFTILA
IGVCFGANCGYAINPARDLSPRIITLIAGWGSATFTVNDYWFWVPIVGPHVGAILGVFIY
ILCIEVHWPEDDKNESLTKSKVKEQTIDIDS-L---
>JAYKOS010000005_Artemia_franciscana_Bett_Glp2_v1
--------------------------------------------MDRGIKNKLKVSSPIF
REFVAECLGTFILVAFGDACVAQSVLSKGEKGDFFSINWGWGLGGMLAVLICGGVSGAHL
NPAVTLAMAIVGKHPWKKVLHYMAGQYLGGLIAAAVVLGVYSEGIYYYEDQVGNGTLNIG
NTAGIFATYPYMWTTTLGGFADQIFGTMTLLIAVCAITDEKNMQISKPLIPLYVGFTILA
IGVCFGANCGYAINPARDLSPRIITLIAGWGTGTFTVNDYWFWVPIVGPHVGAILGVFIY
ILCIEVHWPEDDKNESLTKSKVKEQTIDIDSKLNSF
>JAVRJZ010000021_Artemia_franciscana_Jo_Glp2_v1
--------------------------------------------MDRGIKNKLKVSSPIF
REFVAECLGTFILVAFGDACVAQSVLSKGEKGDFFSINWGWGLGGMLAVLICGGVSGAHL
NPAVTLAMAIVGKHPWKKVLHYMAGQYLGGLIAAAVVLGVYSEGIYYYEDQVGNGTLNIG
NTAGIFATYPYMWTTTLGGLADQIFGTMTLLIAVCAITDEKNMQISKPLIPLYVGFTILA
IGVCFGANCGYAINPARDLSPRIITLIAGWGTGTFTVNDYWFWVPIVGPHVGAILGVFIY
ILCIEVHWPEDDKNESLTKSKVKEQTIDIDSKLNSF
>XM_065710897_Artemia_franciscana_Glp2_v1
--------------------------------------------MDRGIKNKLKVSSPIF
REFVAECLGTFILVAFGDACVAQSVLSKGEKGDFFSINWGWGLGGMLAVLICGGVSGAHL
NPAVTLAMAIVGKHPWKKVLHYMAGQYLGGLIAAAVVLGVYSEGIYYYEDQVGNGTLNIG
NTAGIFATYPYMWTTTLGGLADQIFGTMTLLIAVCAITDEKNMQISKPLIPLYVGFTILA
IGVCFGANCGYAINPARDLSPRIITLIAGWGTGTFTVNDYWFWVPIVGPHVGAILGVFIY
ILCIEVHWPEDDKNESLTKSKVKEQTIDIDS-L---
>JAVRJZ010000021_Artemia_franciscana_Jo_Glp2_v2
MGFSEEVAKEYYDYYYKSSTDPDGTANKYPSSLSAYSTNTDIFQMDRGIKNKLKVSSPIF
REFVAECLGTFILVAFGDACVAQSVLSKGEKGDFFSINWGWGLGGMLAVLICGGVSGAHL
NPAVTLAMAIVGKHPWKKVLHYMAGQYLGGLIAAAVVLGVYSEGIYYYEDQVGNGTLNIG
NTAGIFATYPYMWTTTLGGLADQIFGTMTLLIAVCAITDEKNMQISKPLIPLYVGFTILA
IGVCFGANCGYAINPARDLSPRIITLIAGWGTGTFTVNDYWFWVPIVGPHVGAILGVFIY
ILCIEVHWPEDDKNESLTKSKVKEQTIDIDSKLNSF
>XM_065710896_Artemia_franciscana_Glp2_v2
MGFSEEVAKEYYDYYYKSSTDPDGTANKYPSSLSAYSTNTDIFQMDRGIKNKLKVSSPIF
REFVAECLGTFILVAFGDACVAQSVLSKGEKGDFFSINWGWGLGGMLAVLICGGVSGAHL
NPAVTLAMAIVGKHPWKKVLHYMAGQYLGGLIAAAVVLGVYSEGIYYYEDQVGNGTLNIG
```

NTAGIFATYPYMWTTTLGGLADQIFGTMTLLIAVCAITDEKNMQISKPLIPLYVGFTILA
IGVCFGANCGYAINPARDLSPRIITLIAGWGTGTFTVNDYWFWVPIVGPHVGAILGVFIY
ILCIEVHWPEDDKNESLTKSKVKEQTIDIDS-L---
>PQ469253_Artemia_franciscana_Glp2_v2
MGFSEEVAKEYYDYYYKSSTDPDGTANKYPSSLSAYSTNTDIFQMDRGIKNKLKVSSPIF
REFVAECLGTFILVAFGDACVAQSVLSKGEKGGFFSINWGWGLGGMLAVLICGGVSGAHL
NPAVTLAMAVVRKHPWKKVLHYMAGQYLGGFLAAAVVLGVYSEGIYYYEDQVGNGTLNIG
NTAGIFATYPYMWTTTLGGLADQIFGTMTLLIAVCAITDEKNMQISKPLIPLYVGFTILA
IGVCFGANCGYAINPARDLSPRIITLIAGWGTGTFTVNDYWFWVPIVGPHVGAILGVFIY
ILCIEVHWPEDDKNESLTKSKVKEQTIDIDS-L---
>GJHQ01003152_Artemia_franciscana_glp2_v2
MGFSEEVAKEYYDYYYKSSTDPDGTANKYPSSLSAYSTNTDIFQMDRGIKNKLKVSSPIF
REFVAECLGTFILVAFGDACVAQSVLSKGEKGDFFSINWGWGLGGMLAVLICGGVSGAHL
NPAVTLAMAIVGKHPWKKVLHYMAGQYLGGFLAAAVVLGVYSEGIYYYEDQVGNGTLNIG
NTAGIFATYPYMWTTTLGGLADQIFGTMTLLIAVCAITDEKNMQISKPLIPLYVGFTILA
IGVCFGANCGYAINPARDLSPRIITLIAGWGTGTFTVNDYWFWVPIVGPHVGAILGVFIY
ILCIEVHWPEDDKNESLTKSKVKEQTIDIDS-L---
>GJHQ01003152_Artemia_franciscana_glp2_v1
--MDRGIKNKLKVSSPIF
REFVAECLGTFILVAFGDACVAQSVLSKGEKGDFFSINWGWGLGGMLAVLICGGVSGAHL
NPAVTLAMAIVGKHPWKKVLHYMAGQYLGGFLAAAVVLGVYSEGIYYYEDQVGNGTLNIG
NTAGIFATYPYMWTTTLGGLADQIFGTMTLLIAVCAITDEKNMQISKPLIPLYVGFTILA
IGVCFGANCGYAINPARDLSPRIITLIAGWGTGTFTVNDYWFWVPIVGPHVGAILGVFIY
ILCIEVHWPEDDKNESLTKSKVKEQTIDIDS-L---
>CM051264_Artemia_sinica_Glp1_v2
MGFSEEVAKEYYDYYYKSSTDPDGTANKYPSSLSAYSTSTDIFQMDRGIKNKLRVSSPIF
REFVAECLGTFILVAFGDACVAQSVLSEREKGDFFSINWGWGLGGMLAVLICGGVSGAHL
NPAVTLAMAIVGKHPWKKVLHYMAGQYLGGFLAAAVVLGVYSEGIYYYEDQVGNGTLNIG
NTAGIFATYPYMWTTTLGGFADQIFGTMTLLIAVCAITDEKNMQISKPLIPLYVGFTILA
IGVCFGANCGYAINPARDLSPRIITLIAGWGTGTFTVNNYWFWVPIVGPHVGAILGVFIY
ILCIEVHWPEDDKNESLAKSKVKEQTIDIDSKSINF
>CM051264_Artemia_sinica_Glp1_v1
--MDRGIKNKLRVSSPIF
REFVAECLGTFILVAFGDACVAQSVLSEREKGDFFSINWGWGLGGMLAVLICGGVSGAHL
NPAVTLAMAIVGKHPWKKVLHYMAGQYLGGFLAAAVVLGVYSEGIYYYEDQVGNGTLNIG
NTAGIFATYPYMWTTTLGGFADQIFGTMTLLIAVCAITDEKNMQISKPLIPLYVGFTILA
IGVCFGANCGYAINPARDLSPRIITLIAGWGTGTFTVNNYWFWVPIVGPHVGAILGVFIY
ILCIEVHWPEDDKNESLAKSKVKEQTIDIDSKSINF
>JAQQPU010002487_42379_07693_Artemia_sp_Kazakhstan_Glp1_v2
MGFSEEVAKEYYDYYYKSSTDPDGTANKYPSSLSAYSTSTDIFQMDRGIKNKLRVSSPIF
REFVAECLGTFILVAFGDACVAQSVLSKGEKGDFFSINWGWGLGGMLAVLICGGVSGAHL
NPAVTLAMAIVGKHPWKKVLHYMAGQYLGGFLAAAVVLGVYSEGIYYYEDQVGNGTLNIG
NTAGIFATYPYMWTTTLGGFADQIFGTMTLLIAVCAITDEKNMQISKPLIPLYVGFTILA
IGVCFGANCGYAINPARDLSPRIITLIAGWGTGTFTVNNYWFWVPIVGPHVGAILGVFIY
ILCIEVHWPEDDKNESLAKSKVKEQTIDIDSKSINS
>JAQQPU010002487_42379_07693_Artemia_sp_Kazakhstan_Glp1_v1
--MDRGIKNKLRVSSPIF
REFVAECLGTFILVAFGDACVAQSVLSKGEKGDFFSINWGWGLGGMLAVLICGGVSGAHL

```
NPAVTLAMAIVGKHPWKKVLHYMAGQYLGGFLAAAVVLGVYSEGIYYYEDQVGNGTLNIG
NTAGIFATYPYMWTTTLGGFADQIFGTMTLLIAVCAITDEKNMQISKPLIPLYVGFTILA
IGVCFGANCGYAINPARDLSPRIITLIAGWGTGTFTVNNYWFWVPIVGPHVGAILGVFIY
ILCIEVHWPEDDKNESLAKSKVKEQTIDIDSKSINS
>JAYKOS010000005_Artemia_franciscana_Bett_Glp1_v2
MGFSEEVAKEYYDYYYKSSTDPDGTANKYPSSLSAYSTSTDIFQMDKGIKNKLRVSSPIF
REFVAECLGTFILVAFGDACVAQSVLSKGEKGDFFSINWGWGLGGMLAVLICGGVSGAHL
NPAVTLAMAVVGKHPWKKVLHYMAGQYLGGFLAAAVVLGVYSEGIYYYEDQVGNGTLNIG
NTAGIFATYPYMWTTTLGGLADQIFGTMTLLIAVCAITDEKNMQISKPLIPLYVGFTILA
IGVCFGANCGYAINPARDLSPRIITLIAGWGSATFT------------------------
------------------------------------
>JAYKOS010000005_Artemia_franciscana_Bett_Glp1_v1
--------------------------------------------MDKGIKNKLRVSSPIF
REFVAECLGTFILVAFGDACVAQSVLSKGEKGDFFSINWGWGLGGMLAVLICGGVSGAHL
NPAVTLAMAVVGKHPWKKVLHYMAGQYLGGFLAAAVVLGVYSEGIYYYEDQVGNGTLNIG
NTAGIFATYPYMWTTTLGGLADQIFGTMTLLIAVCAITDEKNMQISKPLIPLYVGFTILA
IGVCFGANCGYAINPARDLSPRIITLIAGWGSATFT------------------------
------------------------------------
```
