# Supplementary material for: Co-opted and canonical glycerol channels play a major role during anhydrobiosis of an extremophile crustacean
Source: BMC Biol. 2025 Jun 3;23:151. doi: 10.1186/s12915-025-02262-3 (PMC12135271; doi:10.1186/s12915-025-02262-3)
Supplement: Supplementary file 7 — Additional file 7: Fig. S3. Genomic organization of the aquaporin superfamily in Artemia. (A) Chromosomal loci. (B) Gene structures drawn to scale with exons depicted as blue bars, introns as linker lines, and pseudoexons and pseudogenes (†) as red bars. kKb, kilobases; nf, not found. (C) Bayesian majority rule consensus tree of Artemia Glps analyzed in the study. The tree is midpoint rooted and inferred from 500,000 MCMC generations of 10,071 aligned amino acids (n = 30 taxa) with model parameters set to aamodel=mixed. (D) Schematic representation of isoform generation. UER, upstream extended region; UTR, untranslated region. [file 12915_2025_2262_MOESM7_ESM.pdf]

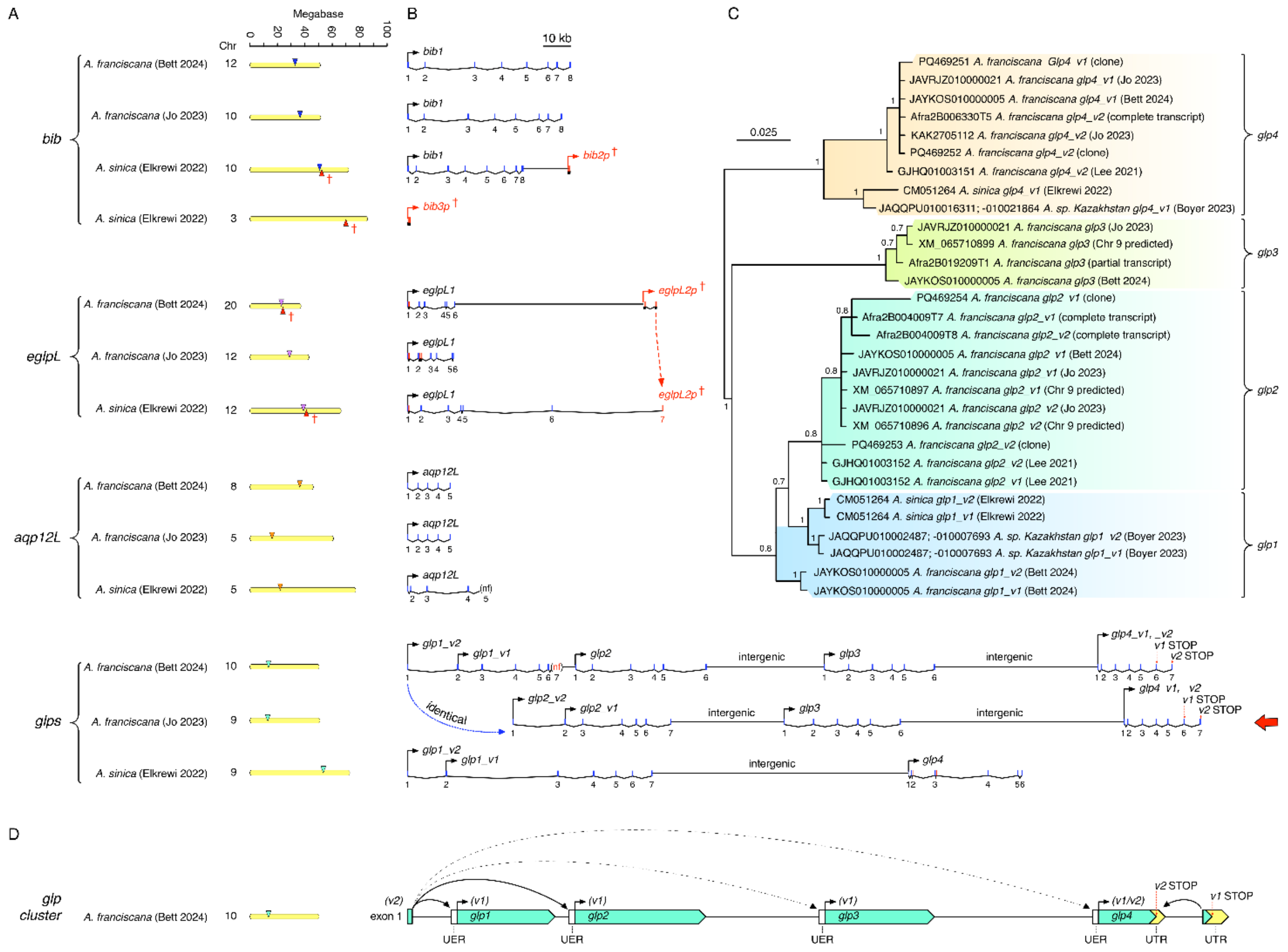

Figure S3. Genomic organization of the aquaporinsuperfamily in *Artemia*. (*A*) Chromosomal loci. (*B*) Gene structures drawn to scale with exons depicted as blue bars, introns as linker lines, pseudoexons and pseudogenes (†) as red bars. Kb, kilobases; nf, not found. (*C*) Bayesian majority rule consensus tree of Artemia Glps analyzed in the study. The tree is midpoint rooted and inferred from 500,000 MCMC generations of 10,071 aligned amino acids (*n* = 30 taxa) with model parameters set to aamodel=mixed. (*D*) Schematic representation of isoform generation. UER, upstream extended region; UTR, untranslated region.
