# Supplementary material for: Co-opted and canonical glycerol channels play a major role during anhydrobiosis of an extremophile crustacean
Source: BMC Biol. 2025 Jun 3;23:151. doi: 10.1186/s12915-025-02262-3 (PMC12135271; doi:10.1186/s12915-025-02262-3)
Supplement: Supplementary file 8 — Additional file 8: Fig. S4. Transcript levels of glp2_v1 in cysts produced by females injected with dsGFP (control) or dsEglpL+dsGlp before and after diapause termination by desiccation (A) or H2O2 (B) and during hatching. Data (mean ± SEM; n= 3–-4 independent experiments) at each time point were not statistically different between the controls and dsEglpL+dsGlp groups when analyzed by an unpaired Student’s t-test. [file 12915_2025_2262_MOESM8_ESM.pdf]

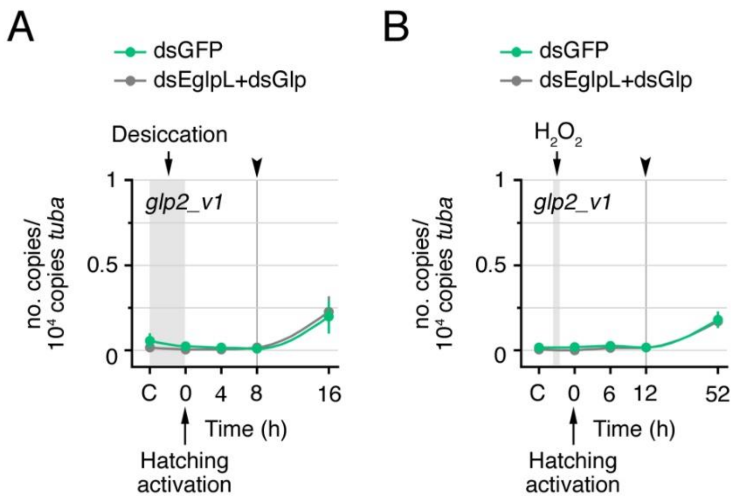

Figure S4. Transcript levels of *glp2_v1* in cysts produced by females injected with dsGFP RNA (control) or dsEglpL+dsGlp RNAs before and after diapause termination by desiccation (*A*) or H_2O_2 (*B*) and during hatching. Data (mean ± SEM; *n* = 3-4 independent experiments) at each time point were not statistically different between the controls and dsEglpL+dsGlp RNA groups when analyzed by an unpaired Student's *t*-test.
