# Supplementary material for: Co-opted and canonical glycerol channels play a major role during anhydrobiosis of an extremophile crustacean
Source: BMC Biol. 2025 Jun 3;23:151. doi: 10.1186/s12915-025-02262-3 (PMC12135271; doi:10.1186/s12915-025-02262-3)
Supplement: Supplementary file 9 — Additional file 9: Table S2. Effect of freezing on A. franciscana hydrated diapause cysts. [file 12915_2025_2262_MOESM9_ESM.pdf]

Table S2. Effect of freezing on *A. franciscana* hydrated diapause cysts

Treatment	Replicate	*N*	No. Hatched nauplii	Hatching rate (%)	Mean
dsGFP	1	200	6	3.00	1.50
	2	200	0	0.00	
dsEglpL+dsGlp	1	200	2	1.00	1.75
	2	200	5	2.50	
