# Supplementary material for: Co-opted and canonical glycerol channels play a major role during anhydrobiosis of an extremophile crustacean
Source: BMC Biol. 2025 Jun 3;23:151. doi: 10.1186/s12915-025-02262-3 (PMC12135271; doi:10.1186/s12915-025-02262-3)
Supplement: Supplementary file 10 — Additional file 10: Fig. S5. Summary of the main steps followed to assemble the A. franciscana transcriptome generated in this study. [file 12915_2025_2262_MOESM10_ESM.pdf]

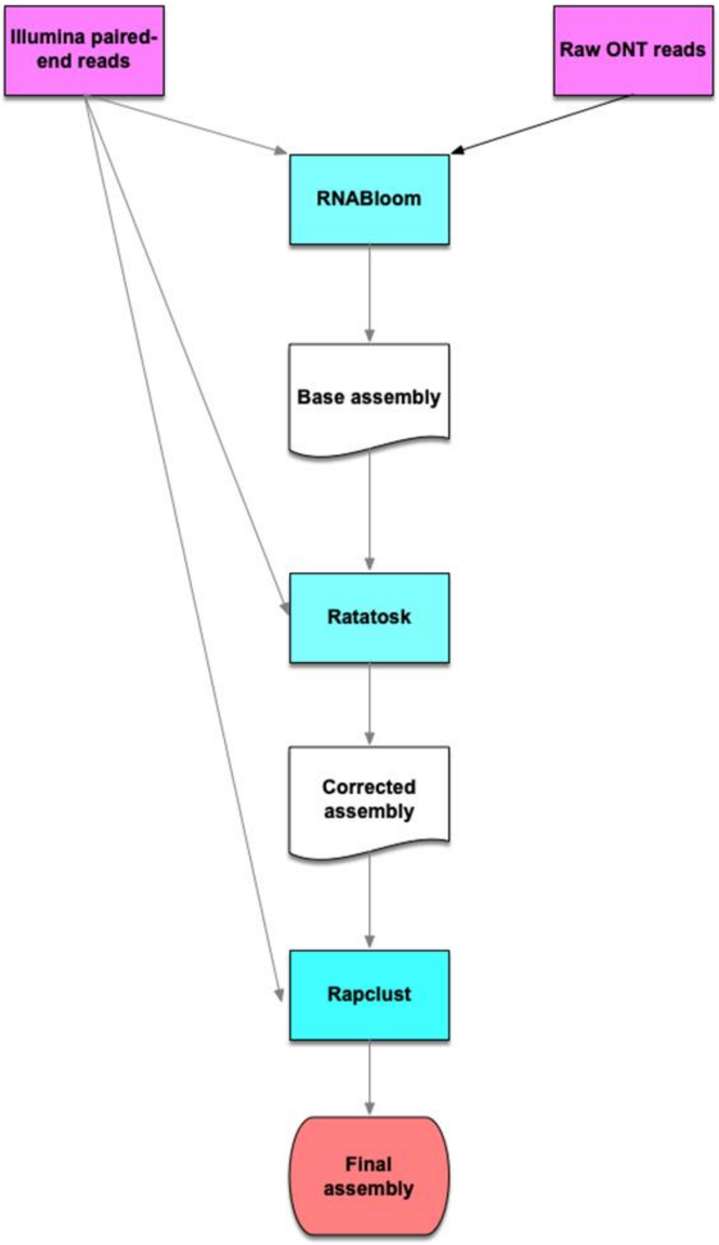

Figure S5. Summary of the main steps followed to assemble the *A. franciscana* transcriptome generated in this study.
