# Supplementary material for: Co-opted and canonical glycerol channels play a major role during anhydrobiosis of an extremophile crustacean
Source: BMC Biol. 2025 Jun 3;23:151. doi: 10.1186/s12915-025-02262-3 (PMC12135271; doi:10.1186/s12915-025-02262-3)
Supplement: Supplementary file 11 — Additional file 11: Table S3. Oligonucleotide primers used for RT-qPCR. [file 12915_2025_2262_MOESM11_ESM.pdf]

Table S3. Oligonucleotide primers used for qRT-PCR

Gene	GenBank accession no.	Primer sequence Forward-Reverse (5'-3')	Amplicon size (bp)
eglpL	PQ469250	F: CAGAGCAGAATTATATGCTGAACG<br>R: TCAACTCTATCACCTTGGCTGT	155
glp2_v1	PQ469254	F: CCTCACTGGGAAGCTTAAAAGA<br>R: CCAAGACACTCTGCAACGAA	176
glp2_v2	PQ469253	F: GAATGGGGTTCAGTGAGGAA<br>R: CCAAGACACTCTGCAACGAA	208
glp4_v1	PQ469251	F: GTTGGCGCTATTCTTGGTGT<br>R: TCACAGCGAATGACTCAAGC	170
glp4_v2	PQ469252	F: GAAATGCATTGGCCAGAAGA<br>R: TTTGGTCTAAAAACGGAAACG	151
atub	XM_065700301	F: CCCGTCTTGACCACAAGTTT<br>R: TAATACTCTTCGCCGGCTTC	187
