# Supplementary material for: Co-opted and canonical glycerol channels play a major role during anhydrobiosis of an extremophile crustacean
Source: BMC Biol. 2025 Jun 3;23:151. doi: 10.1186/s12915-025-02262-3 (PMC12135271; doi:10.1186/s12915-025-02262-3)
Supplement: Supplementary file 12 — Additional file 12: Table S4. Oligonucleotide primers used for dsRNA synthesis. [file 12915_2025_2262_MOESM12_ESM.pdf]

Table S4. Oligonucleotide primers used for dsRNA synthesis

**dsRNA**	**Primer sequence Forward-Reverse (5'-3')**[1]	**Amplicon size (bp)**
dsGlp	F: TAATACGACTCACTATAGGGAGATGGGGTTTAGGTGGC<br>R: TAATACGACTCACTATAGGGAGAAAAACACCAAGAATG	592
dsEglpL	F: TAATACGACTCACTATAGGGAGACTTTTAGTTTATGTA<br>R: TAATACGACTCACTATAGGGAGAGATCCGAGGATATCC	410
dsGFP[2]	F: TAATACGACTCACTATAGGGAGAGCCCTGGCCCACCCT<br>R: TAATACGACTCACTATAGGGAGACAGCACGGGGCCGTC	418

[1] The T7 promoter sequence added to each primer is underlined.

[2] Synthesized from the pEGFP1 plasmid (Addgene; https://www.addgene.org/vector-database/2486/), containing a modified *gfp* from the jellyfish *Aequorea victoria*.
