# Supplementary material for: Co-opted and canonical glycerol channels play a major role during anhydrobiosis of an extremophile crustacean
Source: BMC Biol. 2025 Jun 3;23:151. doi: 10.1186/s12915-025-02262-3 (PMC12135271; doi:10.1186/s12915-025-02262-3)
Supplement: Supplementary file 5 — Additional file 5: Dataset S2. Codon alignment for Figs. S1 and S2. [file 12915_2025_2262_MOESM5_ESM.pdf]

```
>FBpp0079519_Drosophila_melanogaster_Bib
ATGGCCGACGAAAGT---------------------------------------------
------------------------------CTGCACACCGTGCCCCTGGAGCAC---AAC
ATAGACTACCACATCGTGACGCTATTCGAGCGCCTGGAGGCGATGCGTAAGGACTCGCAC
GGCGGTGGCCACGGG---------------------------------------------
------------------------------------GTCAACAATCGCCTGTCCAGCACC
CTGCAGGCTCCCAAGCGGAGCATGCAGGCTGAGATTCGCACGCTG---GAGTTTTGGAGA
TCCATCATCAGCGAGTGTCTGGCCTCCTTCATGTACGTGTTCATCGTCTGCGGTGCCGCC
GCTGGCGTCGGAGTGGGG------------------------------------------
------------------------------------------GCCAGTGTGTCGTCCGTG
CTTTTGGCCACGGCTCTGGCCTCCGGCCTGGCGATGGCTACACTGACGCAGTGCTTCCTC
CACATCTCGGGCGCCCACATCAATCCCGCCGTAACCCTGGCTCTATGCGTAGTGCGATCC
ATATCTCCGATCCGGGCTGCCATGTACATAACCGCACAATGTGGCGGAGGAATCGCCGGA
GCTGCTCTGCTTTATGGCGTAACTGTGCCTGGTTACCAGGGG------------------
------------------------------AATCTGCAGGCCGCCATCTCACATAGCGCT
GCT------CTGGCCGCCTGGGAAAGATTCGGTGTGGAATTCATCCTCACCTTTCTGGTG
GTTCTGTGCTATTTCGTA---TCCACGGATCCCATGAAG---------------------
---AAGTTCATGGGC---------------AATTCGGCCGCCTCGATTGGATGTGCTTAC
AGCGCCTGCTGCTTTGTGTCGATGCCG------------TACCTGAATCCAGCCCGCTCC
CTGGGTCCTTCGTTTGTGCTTAAC---------AAATGGGACAGCCATTGGGTGTACTGG
TTCGGACCACTGGTGGGCGGCATGGCCTCTGGCCTGGTGTACGAGTACATCTTCAACTCG
---CGCAACCGCAACCTGCGCCAC------AACAAGGGCAGCATC---GACAAC------
------------------------GAT------------TCCAGTTCGATTCACTCGGAG
GAC------GAACTGAACTACGACATGGATATG---GAGAAGCCCAACAAGTATCAGCAG
TCG---------------------------------------------CAGGGCACCTAC
CCG---------------CGT---------------------------------------
------GGCCAGTCCAATGGCAAT------GGCGGAGGACAGGCGGCC---GGAAATGGT
CAGCACCAAGCTGCCAACATGGGCCAGATGCCGGGCGTAGTGGCCAATGCCGGTCAGGGC
---AATTACTGC------------------------------CAGAATCTGTACACTGCT
------CCGCCGCTCTCCTCGAAG---------------------------TACGATCAG
CAGCAGGAACCT---------------------------CTGTACGGTGGAACCCGCTCA
CTG---TAC---TGC------------------CGTTCGCCC---ACTCTGACCAGA---
------------------------------------------------------------
------------------------------AGCAACCTGAATCGCTCGCAATCTGTGTAC
GCCAAGAGCAACACGGCCATCAATCGA---------GACATT------GTGCCGCGTCCA
GGTCCTCTGGTGCCCGCCCAGAGT---CTTTATCCCATGCGCACCCAGCAG---------
---------------------------------------CAACAGCAACAGCAACAAGTG
GCACCCGCACCTCAATCTTCCCATTTACAGAACCAAAATGTCCAGAAT------------
------------------------CAGATGCAG---------------CAGCGCAGCGAG
AGCATCTACGGAATGAGGGGCTCCATGCGCGGACAGCAG---CAGCCAATCCAGCAGCAA
CAACAA---CAGCAGCAGCAGCAGCAGCTTCAGCAA------------------------
------------CAACAGCCCAACATGGGAGTGCAGCAG---CAACAGATGCAGCCTCCG
------------------------------CCACAGATGATGTCCGATCCTCAGCAGCAG
CCGCAGGGCTTCCAGCCAGTCTAC------GGCACACGCACAAATCCGACGCCGATGGAC
GGAAATCAC------------------------AAGTATGAC------------------
------------------------------------------------------------
------------------------------------------------------------
------------------------------------------------------------
```

```
------------------------------CGACGT------------------------
---CCGCAGCAAATGTACGGCGTGACGGGACCA---------------------------
------------AGGAATCGC---------------------------------------
---------------------GGACAGTCGGCG---------------------------
---CAGTCGGACGACAGCTCCTATGGCTCATATCAC------------------------
------------------------------------------------GGCTCG------
------------------------------------------------------------
------------GCTGTCACGCCG------------------------------------
--------CCA---------------------------GCTCGT---------------C
AT---CCCAGCGTGGAG------CCATCACCTCCG------------------CCACCGC
CC------------------------------------------------------ATGC
TGATGTATGCCCCGCCCCCACAGCCGAATGCAGCCCACCCGCAACCCATTCGCACGCAGT
CGGAGCGAAAAGTTAGTGCTCCAGTTGTGGTATCCCAGCCGGCAGCGTGCGCCGTG---A
CCTACACAACCTCGCAA------------------------GGATCAGCGGTGACAGCCC
AACAGCAGCAACAACAACAGCAG------------------------------------C
AGCAGCAGCAACAACAGCAGCAGCAGCAGCAACAGATGATG-------------------
-----ATGCAACAACAGCAGCAACATTATGGAATGCTGCCGCTGAGGCCCAAC-
>FBpp0144970_Drosophila_grimshawi_Bib
ATGGCCGACGAGAGT---------------------------------------------
------------------------------CTGCACACAATGCCACTGGAGCAC---AAT
ATAGACTATCACATTGTCACATTGTTCGAGCGCTTGGAGGCGATGCGCAAAGATTCACAC
GGCAATGCC---------------------------------------------------
------------------------------------ATCAACAATCGGTTCTCCAGCACC
CTGCAGGTGCCCAAGCGCAGCATGCAGGCCGAGATACGGACCCTG---GAGTTTTGGAGA
TCCATCATCAGCGAGTGCTTAGCCTCGTTCCTGTATGTGTTCATCGTTTGTGGCGCTGCA
GCGGGCGCCGGTGTGGGT------------------------------------------
------------------------------------------GCCAGTGTCTCCTCCGTG
CTGCTGGCCACATCTCTTGCCTCCGGCCTGGCAATGGCAACGCTAACGCAGTGCTTTCTG
CATATATCGGGCGCACACATCAATCCCGCCGTTACCTTGGCCTGTTGTGTGGTGCGTAGC
ATCTCCCCAATCCGGGCCGCCATGTACATGACCGCCCAGTGTGGCGGAGGCATCGCTGGC
GCCGCCCTACTCTACGGTGTCACCGTGCCGGGCTATCAGGGC------------------
------------------------------AATCTGCAAGCGGCAATCTCGCACAGCGCC
GCG------TTAGCCGCCTGGGAACGGTTCGGCGTGGAATTCATACTGACATTCGTTGTG
GTGCTCTGCTATTTCGTA---TCGACCGATGCCATTAAG---------------------
---AAGTTCATGGGC---------------AATTCGGCGTTCGCCATCGGTGCGGCATAC
AGCGCCTGTTGCTTTGTGTCGATGCCC------------TATCTGAATCCAGCTCGCTCT
TTGGGTCCATCGTTTGTGCTTAAC---------AAATGGGATAATCATTGGGTATATTGG
TTCGGACCGCTCGTTGGTGGCATGGCTTCTGGCCTGGTTTATGAGTACATATTCAGCTCG
---AGG---CGCAGCGTGCGACAC------GCAAAAAGTAGCATT---GACAAC------
------------------------GAC------------TCGAGCTCCATCCACTCCGAG
GAT------GAGCTCAACTACGACATGGACATG---GAGAAGCCAAACAAATATCAACAA
TCA---------------------------------------------CAGGGCACATAT
CCG---------------CGT---------------------------------------
------GGCCAATCGAATGGAGGC---------------GCTGCAACA---GCAGCTCAA
CATGCCAACGCCGCCAACATGGCCCAGATG---GGTGTTGTGGGTGCT---GGAGCGGGC
---AACTACTGC------------------------------CAGAATCTGTACACTGCA
------CCGCCACTCTCATCCAAG---------------------------TACGACCAG
CAGCAGGAACCG---------------------------CTCTATGGCGGCACTCGTTCA
```

```
CTT---TAC---TGC------------------CGTTCCCCA---ACTTTGACTCGG---
------------------------------------------------------------
------------------------------AGCAGTTTGAATCGCTCACAGTCTGTTTAC
GCCAAGAGCAACACGGCCATCAATCGT---------GACATT------GTGCCACGTCCG
GGTCCATTGGTGCCGGCACAGAGT---CTTTACCCTATGCGCACACAACAGCAA------
---------------------CACCAGCAGCAGCAGCAGCAACAACAACAGCAACAGGCA
GTTACAGCAGCCCAATCTTCGCATTTACAAAACCAAAATGTGCAAAAT------------
------------------------CAAATGCAG---------------CAGCGCTCTGAA
AGCATTTATGGCATGCGTGGCTCAATTCGCGGTCAGCAG---CAACCACTGCAGCAGCAG
CAGCAA---CAGCAGCAGCAACAGGCA---------------------------------
---------------------ACGGTAACAATGCAACAG---CAACAATTGCAGGCGGCA
------------------------------CCGCAGCTTTTGTCGGAGCCACAGTCGCAA
CCACAGGGCTTCCAGCCAGTCTAT------GGATCACGCAACAATCCCACACCACTCGAC
GGCAATCAC------------------------AAGTTCGAG------------------
------------------------------------------------------------
------------------------------------------------------------
------------------------------------------------------------
------------------------------CGACGC------------------------
------GATCAACTCTATGGGATGACTGGACCA---------------------------
---------ACAAGGAATCGC---------------------------------------
---------------------GGTCAGTCTGCC---------------------------
---CAGTCCGATGATAGTTCGTATGGGTCGTATCAT------------------------
------------------------------------------------GGCTCG------
------------------------------------------------------------
------------GCAGTCACGCCG------------------------------------
--------CCG---------------------------GCACGC---------------C
AT---CCCGGTGTGGAG------CCATCGCCGCCG------------------CCACCAC
CA------------------------------------------------------ATGC
TGATGTATGCACCACCACCGCAACCAAATGCGGCACATCCACAGCCCATACGGACGCAAT
CCGAGCGTAAGGTAAGTGCGCCCGTTGTTGTCTCACAGCCGGCTGCCTGTGCCGTC---A
CCTATACCACATCCCAGGTGCCCACAACG------------GTCACAACAGCAACACAGC
AACAGCAGCAGCAGCAGCAACAG------------------------------------C
AGCAGCAACAGCAGATGATGATGCAGCAACAGCAGCAGCAG-------------------
-----GCTCAGGCACATCAGCAACATTATGCCATGCTGCCGTTGCGACCCAAC-
>AFP49901_Glossina_morsitans_morsitans_Bib
ATGACGGACGATACG---------------------------------------------
------------------------------TTACAAACGTTACCGTTAGATACT---AAT
ATCGACGCCCATATTATAACTTTATTCGAGCGTCTTGAGACCATTCGTCGAGATCAACAA
ACTAACGCC---------------------------------------------------
------------------------------------TTTAATAGCCGTTTGTCTAATACA
CTACAG---CCCAAGAAAAGCATGCAAGCCGAAATCAGAACGTTG---GAATTTTGGAGA
TCTGTTATAAGCGAATGTCTAGCATCATTTTTTTATGTGTTTATTGTGTGCGGAGCAGCA
GCAGGAGCAGGGGTTGGT------------------------------------------
------------------------------------------GCCAGCGTATCTTCAGTT
CTGCTGGCTACTGCTTTAGCATCCGGCTTAGCTATGGGAACGCTAACCCAATGTTTTCTA
CATATATCTGGTGCTCACATAAACCCAGCCGTTACATTAGCATCCTGCGTGATACGGACT
ATATCACCTATGCGGGCAACAATGTATATTACCGCACAATGCGGTGGCGGTATTGCTGGG
GCAGCACTTCTTTATGGTGTAACTGTACCCGGTTACCAAGGC------------------
```

```
------------------------------AACTTGCAAGCAGCTATTTCACATAATGCC
TCA------TTGGCTGCTTGGGAACGTTTGGGTGTGGAATTCATTTTGACTTTTGTGGTA
GTGTTAACATACTTCATT---TCTACGGATTCGGTCAAG---------------------
---AAAGTTATGGT----------------AATTCAGCCCCTTCAATAGCAGCCTCTTAC
AGCGCTTGCAGTTTTGTATCGATGCCA------------TATCTAAATCCTGCAAGGTCG
TTGGGCCCATCGTTTGTATTGAAC---------AAATGGGAAAATCATTGGGTGTACTGG
TTTGGTCCACTTATAGGCGGTTTTGCATCTGGGCTTGTCTATGAATACATATTTAATACA
---CGC---CGCAACTCTCGCCGT------ATTAAAGTCAACATA---GAAAAT------
------------------------GAT------------TCATCATCGATCAACTCGGAA
GAC------GACTTGAATTATGATTTGGATATG---GAAAAACCGAATATGCATAATAAA
TTT---------------------------------------------CAGGGCACTTAT
CAA---------------AGA---------------------------------------
------GCGCAAGCTAATAGTGGT---------------CAT---GGA---CAATCG---
------AAT---ACCAATTCGGGTAAAAGTAACGGT---------------GGA------
---AACTATTGC--------------------------------CAGAATCTTTACCACT
------CCACCGCTTTTAAATAAA---------------------------TTTGAA---
CAAGCCGAGCCT---------------------------TTATATGGTGGTACTCGTTCA
ATG---TAT---TGC------------------CGTTCGCCT---ACTTTGACGCGT---
------------------------------------------------------------
------------------------------ACTAATCTAAACCGTTCGCAATCGGTGTAC
GCAAAGAGTAATACTGCACTCAATCGC---------GATATA------GTGCCACGTTCA
GGACCGTTAGTGCCGGCTCAAAGT---CTGTACCCTTTAAGATTGGGTTTTAGC------
------------------------------------------------------------
---------GCTCAATCATCACATTTACAAAATCAGAACGTCCAAAAT------------
------------------------CAATTGCAG---------------CAACGTTCAGAA
AGTATATATGCCATACGT---AGCCTAAGGCAAAATCAGCTTCAACAACAACAACAACAA
CAGCAACATAAACAACAACAACAGCAGCAGCGTCAT------------------------
------------CAACAGCCTCAACAAGCGACAGAACAT---ATGCAACAAAGCCAACCA
---------------GTAGCTGCCCAACGAGTACAAACTGCTCAAGAAACTCAA------
---GCATGTTTTCAAGCAGTTTAC------GGCACACGTCTCAATCCTAATCCTAACGAT
GGCCATCTC------------------------AAATTCGAT------------------
------------------------------------------------------------
------------------------------------------------------------
------------------------------------------------------------
------------------------------CGTCGT------------------------
---CCCGAAACTGTGTATGGCGTGACTATGCCG---------------------------
------------GGCAATCGT---------------------------------------
---------------------GGACAGTCGGCA---------------------------
---CAATCTGATGACAGTTCTTATGGTTCCTATCCT------------------------
------------------------------------------------AGTTCG------
------------------------------------------------------------
------------TCGGTTACACCA------------------------------------
--------CCT---------------------------GCACGC---------------A
CT---------------------------------------------GAA---CCCCCTC
CG------------------------------------------------------GTTC
TCTTATATGCCCCTCCACCACATCCAAACTCTGCAACAACACCACCTTTACGAACTCAAT
CCGAACGTAAGGTTTCTGCACCAGTAGTCGTCTCCCAAGCTTCTACGATATCAATGGGCC
AATATAGTGCAAATCAA---------------------------TCTTCTAACGCAAATC
```

AATCCGGTAGTATTCAAAACCAA------------------------------------C
CTCAGCAACAACAAATAATCATGCAACATGCTCAACAATCG-------------------
-----------CATCAGCATCAGCACTATGCTATAATGCCATTAAGATCGAAT-
>AAEL004741_Aedes_aegypti_Bib
ATGGCAGAGGAAAGT---
------------------------------CTTCATGTT---CCG---GATTCC---AAT
ATCGACTTCCATATCGTGCAACTGTTTGAGCGGTTGGAGAATCTTCGCCGGGAACCGGGC
GGTGCTAAG---
------------------------------------CTGTCCAGTTCTATTCAA------
GGCCGG------TCAACTAGTATGCATAATGAACTACGAAGTCTG---GAGTTTTGGAGA
TCGATAACCTCCGAGTGTTTGGCATCGTTCTTCTACGTGTTCATCGTGTGCGGCGCTGCG
GCCGGTGCCGGCGTCGGA--
--GCCAGCGTATCCTCGGTC
CTGCTGGCAACTGCTCTCTCGTCCGGATTCGCCGTCACCGCGTTGACCCAGTGCTTCCTT
CACGTGTCAGGAGCCCACATCAATCCGGCCGTAACGATTTCCTTGGCCATTACCCGCATG
ATATCTCCTCTGCGAGCTATTCTGTACATGATAGCGCAGTGTGGAGGTTCGATAGCCGGT
GCAGCTCTCCTGTATGGAGTGACGGTCCCCGGCTATCAGGGC------------------
------------------------------AACCTGCAGGCAGCCGTGTCGCACACCTCT
GCG------CTGGCCGCCTGGGAACGCTTCGGTGTCGAGTTCATCCTGACCTTCGTCGTG
GTCCTATCCTACCTGATC---TCGACCAATTCGTACAAA---------------------
---AAGTACTTTGGC---------------TCGTCGGCGATTGCCATCGGGGCGGCCTAC
AGTGCATGCAGTTTCGTCTCGATGCCT------------TACCTGAATCCTGCGCGGTCA
CTAGGGCCATCTTTCGTGCTGAAT---------AAATGGGACAACCACTGGGTCTACTGG
GTAGGTCCACTGATAGGAGGAATGGTGTCCGGATTGCTGCATGAGTTCATCTTCAGCACC
---AAG---AAGACGTCCAAAAAA------TCGAAGGATGACGTC---GAT---------
--TCCAGCGTAAACTCCGAC
GAG------GATATCAACTACGATATGGACATG---GACAAGCCTCAACAA---------

------TCGGGAAAGTACCACACT---------------TATCGGCCT---TCAAGTGGA
---------------ACCCTCGTTAGCCAACAG---------------------------
---CGCTTCTGC------------------------------CAAAGCATCTACACTGCC
------GAACCAACCTCG---AAG---------------------------ACTGAT---
CGCGTTGAATCG---------------------------ATCTACGGTGGCACCAAATCT
ATG---TAC---TGT------------------AAGTCTCCT---CCGTTGACCCGT---

------------------------------GCCAACCTGAACCGATCGCAGTCAGTCTAC
ACCAAAAGCAACACTGCCCTGAACCGT---------GACATG------AGCATCCGCCCT
GGACCCCTAGTTCCTGCCCAGAGC---CTATACCCGTTGAGGGTCACCCAG---------

---------CCACAGAGTTCACATCTACAGAACCAGAACGTTCAGAAC------------
------------------------CAACTTCAA---------------CAGCGATCAGAA
AGCATCTACGGCATTCGAAGCTCTATGCGT------------CAGCAAGGACCTCCGGAA
---CGAATGCCCCAACAACTACAGCCC------CAT---------------------------
------------------CCCCAAGCGGGT------------------------------
---GAACCACAA------
------GGCTTCCAACCAATCTAC------GGCTCCCGCAACAATCCCAACTGCTCATCA
GATGCCTTG------------------------AAATTTGACCGGGAACCT---------

```
------------------------------------------------------------
------------------------------------------------------------
---CGTGAAAATAGCCGTGACGACTCC---ATGTCCTCCAAGTTC---TCCCGCTCC---
------------------------------ACTCGG------------------------
---CCAGAGTCCATGTACGGC---------------------------------------
------------CAACGCCGG---------------------------------------
---------------------GTACAGTCAGCC---------------------------
---CAATCGGATGACAGTTCCTACGGGTCATACCAT------------------------
------------------------------------------------GGACCAGGCCCT
------------------------------------------------------------
------------GCCATAACTCCG------------------------------------
--------CCA---------------------------ACCCGCAACGCCAGCAATAGCA
GC---AGCAACTACAAC------GGCCAAATGATGCTGCCAAACTACGGA---------C
CA----------------------------------------------------------
-----------GGTCCA---------------------GTGGGGATGGCCCAAATGCAAT
CAGAACGCAAAGCATCGACTTCTTCGCAGCAGCAGCAGCAACAACAACAACAGCAA---C
AGACCATGGGCAACCGAACGCCATCT----------------------------------
--AGTATACAGAACACC---AAC------------------------------------C
TCCCACCGCCACCACCTCAGCTGCAAGCCCATCCAGGGGGC-------------------
-----------TACCCAATGGCCCAC------------GCCGTGCGGCAGAAC-
>CPIJ016447_Culex_quinquefasciatus_Bib
ATGGCCGAGGAAAGC---------------------------------------------
------------------------------CTTCACGTG---CCC---GATTCC---AAC
ATCGACTACCACATCGTGCAGCTGTTCGAGCGGCTGGAGAACCTGCGGCGGGAGCCGGGC
ACCAAG------------------------------------------------------
------------------------------------CTGTCCACCTCGATGCAG------
GGTGGT---GGCCGCGCCAGCATGCACAACGAGCTGCGGAGTCTC---GAGTTCTGGAGA
TCGATAACGGCCGAGTGTTTGGCGTCCTTCTTCTACGTGTTCATCGTGTGCGGTGCCGCG
GCCGGAGCCGGCGTCGGG------------------------------------------
------------------------------------------GCCAGCGTGTCCTCGGTC
CTGCTGGCCACGGCCCTCTCGTCCGGATTTGCCGTCACCGCGCTCACCCAGTGCTTCCTG
CACGTTTCAGGTGCCCACATCAATCCGGCCGTGACCATCGCCCTCGCAGTGACCCGCATG
ATATCGCCCCTGCGCTTGGTCCTGTACCTAATAGCCCAGTGTGGAGGCTCGATAGCTGGT
GCCGCCGTACTCTATGGCGTGACCGTCCCCGGCTATCAGGGC------------------
------------------------------AATCTGCAGGCGGCCGTGTCCCACACCTCG
ACC------CTCGCCGCCTGGGAGCGCTTCGGCGTCGAGTTCATCCTGACCTTCGTCGTC
GTCCTGTCCTACCTGATC---TCGACCAACTCGTACAAG---------------------
---AAGTACTTTGGC---------------TCGTCGGCGATCGCCATCGGGGCGGCCTAC
AGTGCCTGCAGCTTCGTCTCGATGCCC------------TACCTGAACCCGGCCCGCTCA
CTGGGACCGTCCTTCGTACTGAGC---------AAATGGGACAACCACTGGGTTTACTGG
GTGGGACCGCTGGTTGGTGGTATCATGTCCGGTCTGCTGCACCAGTTCATCTTCAGCACT
---AAG---AAAACCATGAAAAAG------TCCAAGGATGACGTC---GAT---------
------------------------------------------TCGAGCATCAACTCCGAT
GAG------GACATCAATTACGACGGGGATCTG---GACAAGCCCCAGCAG---------
------------------------------------------------------------
------------------------------------------------------------
------CCTGGCAAGTATCACACG---------------TACCGGCCC---CAGTCGGGA
---------------ACGCTTGCCAGCCAGCAA---------------------------
```

```
---CGGTACTGC------------------------------CAGAGCATCTACACTGCA
------GAACCCACCGCCAACAAG---------------------------ACTGAC---
CGCGTCGAATCA---------------------------ATCTACGGAGGAACCAAGTCT
ATG---TAC---TGC------------------AAGTCGCCT---CCGCTGACCCGT---
------------------------------------------------------------
------------------------------GCCAACCTGAGCCGATCCCAGTCGGTGTAC
ACCAAGAGCAATACCGCCCTGAACCGGGGCCCG---GACATG------AGCATCCGACAG
GGACCGCTCGTCCCCGCCCAGAGC---CTGTACCCGCTGAGGGTCAACCCA---------
------------------------------------------------------------
---------CCGCAGAGCTCCCACCTGCAGAACCAGAACGTCCAGAAC------------
------------------------CAGCTGCAG---------------CAGCGTTCGGAA
AGCATCTACGGCATCCGCAGCTCGTTGCGTCAA---CCC---CAGCAGGGTCCCAACGAC
GGCCGAATGACCCAGCAGATGGTCGCC------CAC------------------------
------------------CCGCAGCAAGGC------------------------------
---------------------------------------------GACATCCAG------
------GGCTTCCAGCCCATCTAC------GGTACCCGCAACAATCCAAACTGCTCGGCT
GACGGCATC------------------------AAGTTTGATCGCGAAGCG---------
------------------------------------------------------------
------------------------------------------------------------
---CGCGAAAATAGCCGCGACGACTCGATGATGTCGGCCAAGTTTGCCGCCCGCGGT---
------------------------------GCCCGG------------------------
---CCCGAGTCCATGTACGGAGTGTCC---GGT---------------------------
------------CAGCGGCGC---------------------------------------
---------------------GTCCAGTCAGCC---------------------------
---CAGTCCGATGACAGCTCGTACGGCTCGTACCAC------------------------
------------------------------------------------GGATCCGGTCCG
------------------------------------------------------------
------------GCCATCACCCCG------------------------------------
---------CCC--------------------------ACCCGGAACGCCAGCAACAGCA
GC---AGCAACTACAAC------GGTGGCCTGATGCTGCCCAACTACGGA---------C
CC----------------------------------------------------------
-----GCCGGAGGACCCGGAATGGTCGCCATGAACGCCCAGCAGATGAACCAGCAGCAGC
AAGAACGGAAAGCATCCTCTTCCTCGTCCAACCAGCAGCAGCAGCAGCAGCAACAG---C
AATCGAACCGCAACCAGCAGCAGCAGCAGGTC----------------------------
--AGCATACAGGGAACG---AAT------------------------------------C
TGCCACCGCCACCACCGGGAATGCAAAACCACCAGGCCGGC-------------------
-----------GGCTACCAGATGCAT------------CCGGTGCGGCAGAAC-
>XP_037047996_Bradysia_coprophila_Bib
ATGGCTGACGAAACG---------------------------------------------
------------------------------GTTCAATTA---CCATCGGATCAA---AGT
TTAGACTATCATGTGATTACGTTGTTTCAGCGACTGGAATCGCTTCGAAAGGATACATCG
AACAGATGT---------------------------------------------------
------------------------------------ATCGGATGGAATATGCCGCATTCA
ATGCAT---CCCAACGCGAATATGCAAACGGAAATTCGAACATTG---GAATTTTGGAGA
TCCATAATAGCTGAATGTTTGGCATCGTTTCTGTATGTGTTTATTGTATGTGGAGCGGCA
GCTGGCGCTGGAGTGGGT------------------------------------------
------------------------------------------GCCAGCGTATCGTCGGTA
CTATTGGCTACAGCATTGGCATCGGGATTTGCAGTAACAACCCTTACACAATGTTTTTCC
```

CACATCTCAGGTGCTCACATTAATCCCTCCATAACGTTGGGTATGGCTGTAATTAGAGCA
ATTTCTCCATTACGAGCAGCCATGTACATAACGGCCCAATGTGGAGGCGGCATTGCTGGT
GCTGCTCTATTATACGGTGTCACCGTACCCGGTTATCAGGGA------------------
------------------------------AACTTGCAAGCTGCTGTTTCACACAGTGCA
GCA------CTGGCTGCATGGGAACGTTTCGGTGTTGAGTTTATTTTAACATTCGTCGTT
GTGCTGGCCTATTTCATG---TCGACCCATTCATACAAG---------------------
---TCGTATTTCGGT---------------AGTTCGTCAGTGGCTATTGGTGCTGCGTAC
AGTGCGTGCAGCTTTGTATCGATGCCA------------TATTTAAATCCAGCTCGATCG
TTGGGTCCATCATTCGTTCTGAAC---------AAATGGGACAATCATTGGGTGTATTGG
TTTGGCCCATTAGTCGGTGGCGCCGTTTCAGCATTGGTGTACGAGTACATTTTCAATCCG
---AAGCGTCAC---TTTAAGGAC------AACAAAAACAGCCTG---GACAAC------
------------------------GAT------------TCGTCCAGTATGCAGTCAGAA
GAT------GATTTGAACTACGACATGGACTTG---GAAAAGCAGAACATGCAA------

------CAGCAGAAATTCCATTCAAACACT---------TATCGATCG---TCGGCAGGT
GGTGGA---------GTTTTGAGCCAACAAAGTGCG---------------GGC------
------TATTGT------------------------------CAGAGCTTGTATTCCACT
TCGATTGCGCCA---------AAG---------------------------TTTGAT---
CATAACGAGCCG---------------------------TTGTATGGAGGCACCAGATCG
CTG---TAC---TGT------------------AAATCACCA---CCGTTGACCAGA---

------------------------------GCTAATCTGAATCGGTCTCAATCGGTCTAT
GCTAAAAGTCAGACAGCAATCAATCGT---------GAC---------TTCCCACGGCCT
GGACCATTAGTGCCCGCTCAAAGC---CTGTACCCGATGAGAGTGTCCACA---------

------------CAGAACAGTCACTTGCAAAATCAAAATGTTCAAAAC------------
------------------------CAATTACAA---------------CAACGGTCGGAA
AGCATTTACGGCATTCGTGGATCA---------------------------ATGAAACAG
GAACGTCCGCCACAAGATAGCCAAGCA---------------------------------

---------TTTCAACCAGTTTAC------GGCACACGTACGGTGACAACAGCAAGTATT
GGTGACGGA------------------TTATGCAAATACGATCGTGATCCCAGAGAA---

------------ATGAGAGATGATGGA---ACGGCAGTCAAATTCTCGAATCGAGCC---
------------------------------CATCGA------------------------
---CCAGAATCCATGTATGGTGTTACCAGCGGC---------------------------
------------CAACGACGC---------------------------------------
---------------------GGCCAATCAACC---------------------------
---CAATCCGATGACAGTTCATACGGATCGTATCAT------------------------
--GGTCCACCACCA

------------TCAGTAACACCG------------------------------------
--------CCG---------------------------ACACGC---------------A
AT---CAAAACGGTCAT---
---G

CCATTAACGGTGATCCTATGAATCCCGGCTATCAGCAAAATAAAAGTGGATCAAACCAAC
CGGAAAGGAAAACATCGGCAAGTGGGTCGGAGAGACAGGGATACAATGGAAAGCCG----

-----CCTAGCAGTGGATCGTCAATGTCGCATCCGTATAGT-------------------
-----------ATGCAACAAATGCAA------------ATGGTGCGACAGACT-
>CAEZ01006773_Heliconius_melpomene_melpomene_Bib
ATGGGTGAACAAACC---
------------------------------TTTAACATC------GAAGACAAT---AAT
CTCGAACAGCATATAGTGACATTATTGAAAAATTGGAGTGTTTACGTCGGGATGCGAAT
TCTAAT--
---------------------------------------AGTGATAACGTG---------
TTGTCT---GGACGAGTGCCGGCGAGGTTGGAAGTGAGGACCCTT---TCGCTGTGGAAG
GCCGTGGTGGCGGAGTGTGCGGCTTCCTTCCTGTATGTGTTTATAGTATGCGGTGCGGCG
GGCGGAGCGGGCGTCGGG--
--GCCTCCGCATCAGCTGTG
CTCCTCTCCACTGCACTCGCTTCCGGCTGTGCCATAGCAACGTTGACACTATGCTTCGCT
CATATATCTGGTGCACATGTAAACCCGGCAGTGTCGGTGGCGCTGTGCGTGCGCAGACGG
GTGTCGCCGCTACGGACCGCTCTCTACGTAGCAGCTCAGTGCGGCGGCGCCATAGCAGGC
TCCGCGGCCATATATGGGGTAAGCATACCAGGGTACGCCGGG------------------
------------------------------GGCCTGTCAGCGTCGTTACCAGGTGGAGGG
AGTAGT---GTAGCAGCGTGGGAGAGACTTGGAGCAGAACTGTTACTGTCTGGTTTGGTA
TCAGCCGCATACTTCGCG---GCCATGGAG------AGA---------------------
---AGGTGGACTGGT---------------GCCGCCCCAGTGCTAGTTGGTGCCGCGTAT
TGCGCTGCTAGTTTTGTTTCTATGCCG------------TCGCTTAACCCTGCTCGATCT
CTAGGACCTTCCTTCGTACTATCT---------CGTTGGGATAGTCACTGGGTATGCTGG
GTGGGTGGTATCGGCGGTGGCATCGCTTGTGCCTTACTTCACGAGTGG---------GGA
---TCACGAAGA------------------CCACGAGCAAATTCT------AAC------
--TCCCGCGCATCATCACCT
CGG------GAC------ATGGAT---GATATG---GACAAACCAGCGTTT---------

------CCCTCTCATCAT---------------------TATAGACAT---GCCCCA---

---ACTTACTGT---------------------------------TCT------GCTGCC
------GCTCCT---------CGA---------------------------CCTGAT---
ACTACGGAACCG---------------------------CTGTATTCGGGGACTAAGTCC
TTG---TAC---TGC------------------AGGTCGCCT---CCGCCAGCTCGT---

------------------------------CATACACTCCACAGGTCTCAGTCTCTTCTC

---------------------------CTCCGCCCT------------------------

---------CCCACGCACACTGTCACTATGAACCAGAACGTGCATAAC------------
------------------------------GCT---------------CAGAGA---GAC
CCCGCTTATGGGACCACTACTGCGAAT---------------------------------

------------------------------GGCATTAGAACTGGACCTGCTGGT------
GGCGCAGCAGTC--

---------------------------CACGAACGC------------------------
---CGCGAGTCCGTATACGGCGGTAGTACAGCG---------------------------
---------AGCGCGTCGC---
---------------------GGCCCT---CTC---------------------------
---TCCTCAGAGGACAGCGCGTACGGGAGCTACGCG------------------------

--------CCG---------------------CGC---TACCGC----------------

---CCCGACCACTAC-
>XM_045593420_Melitaea_cinxia_Bib
ATGGGCGAGCAAACG---
------------------------------TTCAATATC------GACGATAAC---AAT
TTAGAACAGCACATAGTTACGTTATTCGAAAAGTTGGAGTGTTTACGGAAGGATGCAAAT
TCGAAC--
---------------------------------------AGTGATAACGTG---------
TTAGCA---GGTCGAGTGCCGGCGAGGCTGGAAGTGCGGACGCTG---TCGTTGTGGAAA
GCTGTTGTCGCGGAGTGTGTTGCGTCGTTCCTGTATGTTTTTATAGTGTGCGGAGCGGCG
GGCGGAGCGGGCGTCGGC--
--GCGTCCGCCTCAGCTGTG
CTACTTGCCACCGCGCTCGCTTCCGGCTGTGCCATAGCCACGTTAACTTTATGCTTTGCT
CACATATCTGGTGCTCACGTCAATCCAGCGGTCTCTGTTGCGTTATGTGTTCGACGCCGT
GTATCTCCGCTTCGTACGGCCTTGTACGTGGCCGCTCAGTGTGGAGGCGCCATAGCAGGC
TCAGCTGCTATATATGGAGTTAGTGTCCCTGGGTACGCCGGT------------------
------------------------------GGTCTCTCCGCTTCATTGCCTGGAGGTGGA
GTAGCGGCTGGCGCAGCATGGGAACGACTTGGAGCTGAACTACTGCTGACAGGACTGGTC
GCTGGTGCTTACTTTGCT---GCGATGGAA------CGA---------------------
---AGGTGGACTGGT----------------GCTGCACCGGCGTTGCTCGGTGCTGCATAT
TGTGCTGCTAGCTTCGTATCTATGCCG------------TCTCTGAATCCAGCACGGTCT
TTAGGACCGTCCTTTGTTCTGTCT---------CGCTGGGAAAGCCATTGGGTCTGCTGG
GTTGGTGGCATCGGAGGGGGCATTGCCTGTGCTTTGCTACATGAATGG---------GGC
---ACACGGAGA-------------------CCACGTGCTAACTCC------AAC------
--TCTCGCGCCTCTTCACCC
AGA------GAC------CTAGAC---GAACTG---GACAAACCGGCCTTT---------

------CCGTCTCATCATCAC------------------TATCGACAC---GCACCG---

---ACTTACTGC---------------------------------TCT------GCGGCT
------GCGCCT---------CGA---------------------------CCAGAT---
ACTACGGAGCCA---------------------------CTATACTCCGGAACTAAGTCA
CTC---TAC---TGC------------------AGATCTCCT---CCGCCTGCTCGC---

------------------------------CACACTCTCCACAGGTCGCAGTCAGTATAT
AGCAAGAGCAGCGGCGCGGTGGCG------------AGTGTG------GCGCAGGCGGGC
GCGGCGCTGGTGGGCGCTCAGACGCTACTGCGGCCG------------------------

---------CCG---CACTCCATCGCACTCAACCAGAACGTTCACAAT------------
------------------------------GCA---------------CAGCGA---GAC
CCCGCATATGGCAGCACG---GTTAAC---------------------------------

------------------------------GGCGTGCGACCTGGACCAGCTGGATGTGCA
GGCGCCGCAGCG--

---------------------------CAGGAACGG------------------------
---CGCGAGTCAGTGTACGGCGGCAGC---------------------------------
---------------CGGCGC---------------------------------------
---------------------GGGCCG---CTG---------------------------
---TCGTCCGAGGACAGCGCCTACGGCAGCTTCGCG------------------------

--------CCCGCG------------------CGCGCCTACCGC----------------

--------------------------------------CCGCCCGACCACTAC-
>XM_025977931_Agrilus_planipennis_Bib
ATGGCCGAGGACGTG---
------------------------------CTACACATTTCAAATCTTCCGCAA---AAT
TTAGATTTTCACATAGTGACTCTCTTTGAAAAGTTGGAAACTCTAAGACAAGAAAATGGT
TGT---
---------------------------------------AATAGAAAA------------
AGTTTT---TTGAAACTTCCGATGAAGGCGGAAATTCGTACATTA---GAATTTTGGCGG
TCAGTTATTTGTGAGTGCATTGCTTCTTTTTGCTGTGTCTTTATTGTATGTGGGGCGGCA

```
GCTGGTACAGGGGTTGGT------------------------------------------
------------------------------------------GCGTCGGCGGCAAGTATT
CTTCTTGCCACTGCTCTTGCAGCTGGTTTTGCAATGACTACACTTTCACAGTGTTTTTTA
CACATTTCCGGTGGTCACATCAATCCTTCGGTGACAGTAGCGATGGTAGTTACCAAAAGA
ATAACCATTATCAGGGCTGTTATGTATGTGATAGCTCAAAGTGGTGGAGCTGTAGCAGGG
GTAGCTTTTCTTTATGGAGTGACTGTTCCTGGCTATCAAGGA------------------
------------------------------AACTTAAAGGCAGCAGTTCAACATACAGCA
TCTGCA------GCAGCCTGGAGCGTTTTGGCGTAGAACTAATCCTTACTTTTTCTCATA
GTATTAACATATTTAATG---TCGATAGATTCTTATAAT---------------------
---AAATACATGGGA---------------AGTACATCTCTTACGATCGGGGCATCATAT
GCGGCGTGTTCATTTGTTTCCATGCCT------------TATTTAAATCCGGCACGAGCT
TTGGGACTTTCGTTTGTTTTAAGT---------AAATGGGAAGGTCACTGGGTCTACTGG
ATAGGTCCTCTACTTGGAGGTATTACCTCAGGTCTTCTCTACGAATACGTTTTCAATAAG
---CAGAGGACG---AGTCGT---------CCCAAGGATTCGGCC---GATGGG------
------------------------GAT------------TCGTCGAGCATAAACTCAGAC
GAA------GAT---ACTTACGAC---GATTTA---GACAAACCTGTTGGTACAAAATTC
ACT---------------------------------------------------------
------------------------------------------------------------
------GGTTCCACTTACAATAGT---------------TATAGACCA---GCAGGAAGT
------------------AGTAACGCGGGAGTT---------------------------
---AGCTACTGCGCAAGTCTTACA---------------TCCGCAAGTCTATATGCCGCT
------CCTCCAACA------AAA---------------------------TTGGAG---
AGGGTCGAATCA---------------------------CTTTATGGAGGAACAAAATCC
CTT---TAT---TGC------------------AAATCTCCA---CCTCTTACAAGA---
------------------------------------------------------------
------------------------------GCCAACCTAAATAGATCACAATCCGTCTAT
ACGAAATCTAACAGCGGTTCAAACAAA---------GATACC------CTTCCTCGATCT
GGACCATTAGTACCAGCTCAATCA---TTGTATCCGATCCGTTTAAATCAG---------
------------------------------------------------------------
------------CAGCAAAATCACGTACAGAACCAAAACGTCCAAAAT------------
------------------------CAATTGCAA---------------CAAAGATCAGAA
AGTATTTACGGAATTAGA---------------------------------GGAAATTCT
CAGCGT---CCAGAG---------------------------------------------
------------------GGTGCTTATGGAACAAGT------------------------
------------------------------------------------------------
AATGGTAATAATGGCCCTTTTCAG------GGAAGAAGTACTAATACTACCACCGCAACC
ACTGAT---------------------------AAGTAT---------------------
------------------------------------------------------------
------------------------------------------------------------
------------------------------GAAGATACGCAAAAACCGACCAGAAAT---
------------------------------AACAGG------------------------
---CCCGAATCTATGTATGGAATGAGTCAG------------------------------
------------AGTAGAAGA---------------------------------------
---------------------GGGCAATCAGCG---------------------------
---CAGTCTGATGATTCCAGTTACAGTTCATATCAT------------------------
------------------------------------------------GGATCAGTT---
------------------------------------------------------------
------------------------------------------------------------
```

```
--------------------------------------TCACGT---AGTAATACAGCTA
AC---AGTACTTACAAT------GGGCCAACTAGCAATTTTTCTAGTAAA---GGC----
-----AGTAATGGTGGAAGCAGCACTTATGGCGTGAGA------------------TCCG
ATATTAGGAAATCCCCG-------------------------------------------
------------------------------------------------------------
--------CCCACACAGTTAATCACTGCTACT----------------------------
------------------------------------------------------------
-----CCACATAGC---GCATCTTATCATCATAATAATTTG-------------------
--------------CCACAA---CAT---------TCCCCTTCATCTCAATAC-
>TC010832_Tribolium_castaneum_BIb
ATGGCAGAGGAACGC---------------------------------------------
------------------------------GTTAATTTATCTAGTGAGCCG------AGC
ATAGACTACCACATAGTGACACTTTTCGAAAAGTTGGAAGCTTTAAGAAATGATAAGCCG
AAC---------------------------------------------------------
---------------------------------------------TCG------------
CAGTTTACTTCAAAATTGTCAATTAAAGTCGAGGTCCGCACTTTG---GAATTTTGGAGA
TCGGTCATCTCCGAGTGCATAGCGTCTTTTGTTTACGTGTTTGTGGTGTGCGGTGCAAAG
GCAGGTGCTTTTGACGGAGGTTCG------------------------------------
------------------------------------------GCAACTACCTCTTTGGGA
GTTCTTTCAACAGCACTGGCAGCTGGCTTTGCAATGATATCGTTAACACAGTGCTTCGGA
CACATCTCTGGGGCGCACATAAACCCGGCCGTGAGTCTGGCCATGGGCATTACGAAGAAT
ATTTCGCTTCTGCGGACGACAATGTTCATTGTCGCACAATGCGGGGGTGCGATCGCCGGC
GCCGCTTTCCTCTACGGCGTATCCATTCCGCGAACTCAGGAG------------------
------------------------------GATCTCCCGATGTCCCTGCCG---ATAAAA
GATGGG------CCT---TGGGAGAGGTTCGGGAACGAGTTTATTCTGACTTTCGTGGTG
GTTTTGGCCTATTTCGTC---TCAATGGACGCCCAAAGG---------------------
---AGGTGGATGGGC---------------ATGTCGGCGATTACGATCGGAGCCACGTAC
TCCGCTTGCTCGTTCGTTTCGATGCCG------------CACTTGAACCCAGCGCGTTCG
CTGGGGCCGTCCTTTGTTTTGAAA---------AAATGGGACAACCACTGGGTTTATTGG
CTGGGGCCCATAATTGGCGGCGTGGCAGCTGGGTTAATCCACGAGTACATTTTCAACTCG
---AAGCGCTCG---AAACGC---------CAAAACAGCTCGAGT---GACGGC------
------------------------GAC------------TCGTCCAGCATCCAGTCGGAT
GAG------GAC---ACTTACGAC---GACCTC---GACAAACCCAATGCCCCAAAATAC
CAG---------------------------------------------------------
------------------------------------------------------------
------GAATCGACTTACAACTCC---------------TACAGAGCC------------
------------GGCGCTCCGAACGGCCCCACC------------------GGT------
------TATTGTCCGAGTTTGACA---------------TCGGCCAGTCTATATTCAACA
------CCTGCCACA------AAA---------------------------TTAGAA---
CGGGTTGAGTCT---------------------------CTCTACGGAGGGACAAGGTCG
CTG---TAT---TGT------------------AAATCGCCT---CCACTCACTAGA---
------------------------------------------------------------
------------------------------GCCAATTTAAACCGCTCGCAGTCGGTTTAC
ACGAAAAACAGCGGCGGCCCT---CGG---------GACATC------CTCCCGAAGCCC
GGTCCCCTGGTCCCCACGCAGTCG---CTACATTCGATAAAACTGAAC------------
------------------------------------------------------------
------------CAACAAACGCACGTTCATAACCAGAACTTGCAAAAT------------
------------------------CAGCTCCAA---------------CAGCGCTCCGAA
```

```
AGCGTGTATGGGGTGAGGGGAAACCCGCCG---------------------GGGAGCCTC
TCGCGG---CCCGAA---------------------------------------------
------------------------------------------------------------
------------------------------------------------------------
------ACCGAGAAAAGCGCCTAT------CCCAGTAAAAATAGCGCGCCGAATAGT---
------------------------------------------------------------
------------------------------------------------------------
------------------------------------------------------------
------------------------------GAAGAGAGCAAAAAGAACTCGAAGATA---
------------------------------AACAGG------------------------
---CCGGAGTCGATGTACGGGATTATGAGTTTG---------------------------
------------CAGCGTAGG---------------------------------------
---------------------GGACAGTCGGCC---------------------------
---CAGTCGGACGACTCGAGCTACGGGTCCTATCAT------------------------
------------------------------------------------GGACCAGCT---
------------------------------------------------------------
------------------------------------------------------------
--------------------------------------CCGAGG---------------A
AC---AACACGTATCAT------TCCAACACTTCGGGTTTCCAGAATAAGGCCGGAAGC-
-----------GTGGCGAGT------TATGTGAGCAGGAACGCCGGG---------TCC-
--GCGGAGGGGAGACCGCCCCCTTCG----------------------------------
------------------------------------------------------------
--------GCAAACCAGCTCCTCACCACCACA----------------------------
------------------------------------------------------------
-----GGAAACCCGACCAATTCGTACCATCACCAC-------------------------
-----------------CAA---CAC---------TCGCCCTCGTCTCAATAT-
>JU421678_Pogonus_chalceus_Bib
ATGGCTGAAGAAAGT---------------------------------------------
------------------------------TTAAATATACCAAGTGACACACAA---AAT
ATAGAATATCATATAGTGACATTATTCGAAAAATTAGATGCTTTAAGACGTGATAGTGCT
TGT---------------------------------------------------------
------------------------------------GTACCCTTAAGTACC---------
GTTGGTAATCAAAAACCACCAATGCAAGTTGAGATAAGAACATTA---GAATTTTGGCGG
AGCATTATTTCCGAATGTTTAGCATCGTTTTTCTATATTTTTATTGTTTGTGGTGCTACT
GTTGGT------------------------------------------------------
------------------------------------------GCTGAAATCACTTCAGCA
CTTCTTGCATCTTCTTTAGCTGCTGGTTTTGCCATGGCAACTTTGACTCAATGTTTTGGG
CATATTTCAGGGGCTCATGTCAATCCTGCAGTTACTGTTGCAATGGCAGTTACCAGAAAT
ATTTCTCCACTTCGTGCTGCCATGTTTATAACTGCACAATGTGGAGGAGGCATTGCTGGA
GCAGCTCTACTTTATGGAGTATCCGTTCAAAATTACCAAGGG------------------
------------------------------CAACGAAATTTATCGTCAATGTTAATGGCT
AGTGGAAGTATACCAGCTTGGGAACGATTTGGTGTTGAATTTATTTTAACATTTATGGTT
GTTTTTACGTATTTCGTA---TCCATGGATACTTATCGC---------------------
---AAATGGATGGGA---------------AGCTCTGCTCTGACAATTGGAGCTTCATAC
AGTGCATGCACTTTTGTTTCGACTCCG------------TATTTGAATCCTGCTCGTTCT
TTGGGGCCATCGTTCGTTATGAAC---------AAATGGGATAATCATTGGGTGTATTGG
ATTGGTCCATTAGTTGGAGGAATAATGTCTGGACTTATCTACGAATTTATTTTCAATCCA
---AGAAGACAT---GTACGCAAA------CAAAAAGATTCTATT---GATGGT------
```

------------------------GAT------------TCATCTAGTATCCATTCAGAT
GAA------GAT---ACTTATGAT---GATTTA---GATAAACCTGCTGGACCTAAATTC
CAT---

------GGATCGACTTATAATACG---------------TATAGATCC---TCTGGTGCA
AATGGT------CCAAATTCTGCATCAGGAGTA------------------GGC------
------TATTGCGCTAGCGTAGCT---------------TCAGCTAGTTTATACTCTGCT
------CCGCCAACA------AAA---------------------------TTAGAA---
CGCGTAGAATCT---------------------------TTATACGGTGGAACAAAATCA
TTA---TAC---TGC------------------AAATCTCCT---CCATTGACTAGA---

------------------------------GCAAACTTAAACCGGTCACAATCAGTTTAC
GCAAAATCAAATACCGCAATAAACCGC---------GATATT------ATTCCAAGACCA
GGTCCATTAGTACCAGCTCAATCT---TTATACCCTATGCGTTTAAATCAA---------

------------CAACAAACTCATTTACAAAACCAAAACGTTCAAAAT------------
------------------------CAAATGCAA---------------CAACGATCAGAA
AGTGTTTATGGAGTCAGACAAGTTGCTCCA---------------------GGGTTAGCT
ACAAGA---ACTGAAGGAAGC---------------------------------------
------------------GGAGCGTATGGAGGAGCACCCGCT------------------
---GGA
AATGGAAATAATGGAACAATTTAC------GGAACAAGAAATAACCCGTCTGGTTCATCA
GATAATCAA------------------------AAATAT---------------------

------------------------------GAAGAAAATCAAAAGTTCGGTCGTGGT---
------------------------------AATCGG------------------------
---CCTGAATCTATGTATGGTATGGTAAGTGGT---------------------------
---------CAACAACGAAGA---------------------------------------
------------------TGTGGACAATCTGCT---------------------------
---CAGTCGGATGACTCGAGTTATGGTTCATACCAT------------------------
--GGTTCAAGCACA
ACT---

--------------------------------------CCAAGAAATGTTTCTAATAACA
AT---GGTAACTATAAC------TCAGGAACTACA---TACAGCACCAAA---GGAACT-
--ACTAGCTCAGCAGCTCAGAGCGATTTTAATCGACGACAAGTAGGTGGT------AATG
AAGTTAGACAATCTCCA---

--------CCTACACAGCTGTTACCAAATGTA----------------------------

-----CCAAATTCG---AATAGTTTTCATCATGCAAGGCAA-------------------
--------------CCTCAA---CAC---------TCGCCTAGCTCTCAATAT-
>XM_022072663_Zootermopsis_nevadensis_Bib
ATGGCAATAGGAAGC---
------------------------------CTGTCTCTGACTCAAGACCACCAA---AAT
GTGGAATACCATTTGGTGACGCTGTTCGAAAAGCTTGACGCAGTTCGTCGTGACGCCACG
TGC---

------------------------------------GTTTCGCCGTCAGCA---------
TCGACGCCAACCCACCCATCAATGCAAGTCGAAGTGCGGACCCTC---GAGTTTTGGAGG
TCTATCATTTCGGAATGCCTAGCCGCGTTCTTCTACGTCTTCATCGTTTGTGGCGCCGCA
TCGGGGGCCGGGGGGACTCCCAGCTCGCTGCTCTCG------------------------
---------------------------------ACGACGACGACACCCACTTCGTCCGTG
ACACTCGCCACGGCCCTGGCCACGGGGTTCGCTGTCGCAACCCTCACGCAGTGCTTCGGA
CACATCTCCGGTGCCCACGTGAACCCTGCCGTCACAGTCGCCATGGTGGTGACTCGAAAC
ATCACTCCCCTCCGGGCGATCATGTTTGTGACGGCGCAGTGTGGCGGCGGCATCGCGGGG
GCGGCTTTGCTGTATGGGGTGGCTGTTCCCGGCTACATGGGT------------------
------------------------------AACCTGAGCGCCACCGTGTCCCATCTGCCG
GCGCAC---CTCAACTCCTGGGAGAGATTTGCTGTTGAGTTCATCCTCACCTTCATTGTC
GTCTTCACATACTTCGTC---TCCATGGACACCTACCGC---------------------
---AAGTGG---CCC---
------TGCACTCTGGTGTCGATGCCG------------TCTATGAATCCTTCAAGAACA
TTAGGCCCAGCATTTGTGATGAAC---------AAGTGGGACAGTCATTGGGTTTATTGG
TTTGGTCCAATGCTTGGAGGAATGGTGTCTGGTCTCATCTATGAATTTATTTTTAATCCA
---CGAAGACAC---GTTCGACAG------CCCAAGGAATCCATT---GACGGA------
------------------------GAT------------TCTTCAAGCATTCACTCTGAC
GAG------GAT---CCTTATGAT---GAGTTG---GACAAGCCTGCAGCACCAAAATTC
GAA---GGATCTACTTAC
AACACGCTG---------CGT---------------------------------------
------------GGTGCAGACATC---------------TACAGGCCA---GCTGGGAAT
CATGGAGGACCCCCAACACCAGCTTCACCCACA------------------GGTCCCAGT
AATGCATATTGCCCAAGCCTGACA---------------TCTGCAAGTCTGTACTCAGCA
------CCACCATGT------AAG---------------------------CTCGAT---
CGTGTCGAGTCC---------------------------TTATATGGTGGAACCAAGTCT
TTG---TAT---GCA------------------AAATCTCCT---CCACTGACAAGA---

------------------------------GCCAACCTCAATCGATCACAGTCAGTCTAC
AGCAAAGTTCCATCTGGGCCTGTGTGT---ACTCGTGATGGC------CTTCCTCGACCA
GGACCACTAGTACCTGCACAGTCT---CTTTACCCAATGAGATTTAGC------------

------------CAAAACCATAACACAGCCAATCAGAATGTTCAGAATCAGCAG------
---------------------AGCCAGCTGCAG---------------CAGCGGTCAGAG
AGTATATACGGCATACGTGGCATT---------------------------GCAAATGTG
AACCGT---GGAGAA---

---------AATGGTGGTGTTTAT------GGTGTTAGCCATGGAAATGCTGCTACTGTA
GGCAACACA---

------------------------------AGTGTGGGGAAATTTGGTGGTGGATCA---
------------------------------AATCGC------------------------
---CCTGAATCAATGTATGGAATGGTGGTG------------------------------
------------AATCGTCGC---------------------------------------
------------------------CAG---------------------------------
---GATTCTGCAGACTCATCATACAGCTCTTACCAC------------------------

--AGCAGTGGTAAT
GGT---
------GGTGGTGGTGTAAGGCCAGGAAATGGAAACTTTGTTGGTGGAAATGTGGGGGAC
CACCACCACCACCACAAGGATTCAGCTCA---------ACTAGG---------------A
AT--
---G
ACTTGCGCCAATCTCCTCAGACGCCC----------------------------------

--------CAGCATCAGCTGCTATCTCCTGCT------------------------TCCA
CCTCAATCATAAACACG---
-----GCTAATTCTGTAGCATCCTACCACCATAATCAA----------------------
-----------------------CAT------CTTTCACCTAATCCACAGTAT-
>GHMS01033837_Reticulitermes_aculabialis_Bib
ATGGCAATAGGAAGC---
------------------------------CTTTCGTTGGCTCAGGACCATCAG---AAT
ATGGAATATCACTTAGTAACGCTGTTCGAAAAACTTGACGCCGTGCGTCGTGACGCTACG
TGT---
------------------------------------GTTTCGTCGTCGACA---------
TCGGCACCTGTCCGCCCGTCTATGCACGTCGAAGTGCGGACGCTC---GAATTTTGGAGG
TCGATCATTTCGGAATGCTTGGCGGCTTTCTTCTACGTCTTCATCGTCTGTGGCGCTGCG
TCGGGGGCTGGGGGGGCTCCCAGCAGTTTGATGTCG------------------------
--ACACCTACTTCTTCAGTG
ACCCTTGCCACGGCACTGGCTACGGGATTCGCCATCGCCACTCTTACTCAGTGCTTCGGA
CACATATCAGGTGCCCATGTGAACCCAGCTGTGACGGTCGCCATGGCGGTGACGCGTAAC
GTAACTCCACTGAGGGCGATCATGTTCGTCACGGCGCAGTGTGGGGGTGGCATCGCGGGG
GCTGCTCTGCTTTATGGCGTGACAGTCCCCGGCTACATGGGT------------------
------------------------------AATCTGAGCGCTACCGTGTCGCATCTGCCG
GCACAC---CTCAACTCCTGGGAGAGGTTTGGTGTCGAGTTCAGCCTCACCTTCATCGTG
GTGTTCACGTACTTCGTG---TCCATGGACACGTACCGC---------------------
---AAGTGGCTAGGC---------------AGCTCACCACTCGTGATCGGCTCGGCATAC
CTGGCATGCACCCTTGTCTCGATGCCT------------TCTCTGAACCCATCTCGAACA
TTGGGTCCAGCATTTGTGATGAAC---------AAGTGGGACAGTCACTGGGTTTACTGG
TTTGGCCCAATACTTGGAGGGGTGGCAGCTGGCCTCATCTATGAATTCATTTTCAATCCA
---CGGAGACAT---ATGCGACAG------CCCAAGGAATCCATT---GATGGA------
------------------------GAT------------TCTTCAAGTATTCACTCTGAT
GAA------GAT---CCCTATGAT---GAACTG---GACAAGCCTGCAGCACCTAAATTT
GAA---GGGTCTGCTTTT
AACACAATG---------CGC---------------------------------------
------------AGTGCAGATGTA---------------TACAGGCCA---ACTGTAAAT
CATGGAGGACCACAAACACCAGCTTCTCCGACA------------------GGTCCTGGT
AATGCGTATTGCCCAAGCTTGACA---------------TCTGCAAGTCTCTACTCAGCA
------CCGCCATGT------AAG---------------------------CTTGAT---
CGAGTTGAGTCC---------------------------TTGTATGGTGGAACCAAGTCT
CTG---TAT---GCA------------------AAGTCTCCT---CCCCTGACACGA---

------------------------------GCCAACCTCAATCGATCCCAGTCCGTCTAC
AGCAAAGTTCCACCAGGACCTATGTGC---TCACGTGATGGT------CTGCCTCGACCA
GGACCACTAGTACCCGCACAGTCT---CTTTACCCAATGAGATTTAGC------------

------------CAGAACCATAACACAACCAATCAGAATGTCCAGAATCAGCAG------
---------------------AACCAACTGCAG---------------CAACGGTCAGAC
AGTATATACGGTGTGCGTGGGATT---------------------------GCAAGTGTG
AATCGT---GGAGAA---

---------ACTGGTGGTGTTTAT------GGTGTTAGCCATGGAAATGCTGCTACT---
---AACACA---

------------------------------GCTGTGGGAAAATTTGGTGGTGGAACA---
------------------------------AATCGT------------------------
---CCTGAGTCCATGTATGGAATGGTGGTG------------------------------
------------AATCGTCGC---------------------------------------
------------------------CAG---------------------------------
---GATTCCGCAGATTCATCCTACAGTTCTTACCAT------------------------
--AGTAGTGGTAAC

------AGTGGTGGTGTGAGGCCAGGAAATGGAAACTTTGTGGGTGGAAATGTGGAGGG-
-----CTACCACAACAAGGATTTAGCTCG---------ACTAGG---------------A
AT--
---G
ACTTGCGCCAGTCTCCTCAGACACCA----------------------------------

--------CAGCATCAGTTGCTCTCCCCTGGT------------------------CCCA
CCTCTGTTGTAAACACA---
-----ACTAATTCTGTAGCATCATATCACCACAACCAA----------------------
-----------------------CGC------CTTTCACCAAGCCCACAATAT-
>RZF36961_Laodelphax_striatellus_Bib
ATGACAACGGAG--
------------------------------------CTATCCCGT---------------
CTGGATTACCAGGTGATAACGCTACTTGAAAAATTGGAGACGGTCCGTCGCGATGCTCCT
CCT---

------CCAAGACTTCTGCCGGCTAATGCCGAAATGCGAACCCTC---GAATTTTGGAGG
TCAATTATTTCAGAATGTTTGGCCTCTTTTTTCTATGTGTTCATGGTCTGCGGTGCCGCT
GCGGCAGTCACGTCCGGA--
--GCCACGTCGGGCCAAACC
ATTCTAGCAGCTGCATTGGCATCAGGCTTCACGATCGCCGCACTCACACAGTCTTTCGGA
CATATTTCAGGCTGTCACGTGAACCCAGCAGTGAGCGCAGCGATGGCGATTACGCGCAAC
ATTTCTCTGCTGCGTGGACTGATGTTCGTCACAGCCCAGTGCGGAGGCAGCATTGCAGGT
GCAGCTCTACTCTACGGAGTGACAGTGCCTGGACGGCAAGGC------------------
------------------------------AGCCTGTCAGTGACGGTGTTGCAACTGCCG
CCGCAC---CTGACTCCGTGGGAGCGATTCGGACTGGAGCTGATCCTCACCTTTGTGGTT
GTCTTCACCTATTTCGTG---GCCATGGACTCGCAACGC---------------------
---CGCTGGACTGGC----------------ACTGCTGGCATCACTGTGGGCGCCGCCTAT
CTTGCCGCTTCTCTTGTCTCGATGCCA------------TCTCTGAATCCAGCTAGAGCA

TTGGGTCCTGCATTTGTCATGAAT---------AAATGGGATAACCACTGGGTATTTTGG
TTTGCACCTATCCTGGGAGGAGTATTGGCTGGATTCATATACGAATTCATTTTCAATCCA
---CGAAGACAC---ACCAGGAGA------TCGAAGGACTCAATT---GACGGA------
------------------------GAT------------TCATCAAGCATCCACTCTGAT
GAG------GAC---ACTTATGAT---GAATTG---GAGAAGCCAAGA------------
---TCGACTTAC
AACACTTTG---------AGA---------------------------------------
GCGGTTCCTCCTGGAAGTGATCTG---------------TACAGGGCA---GCTGGAGGA
------------CTGACACCCGCATCACCATCT------------------GGTGGAGGC
AACCCGTACTGCCCAAGCCTAGCT---------------TCAGCCAGTTTGTATTCAGCT
GCT---CCGCCCTGC------AAA---------------------------TTGGAC---
CGAGTGGAGTCG---------------------------CTGTACGGCGGCACCAAATCG
CTC---TAC---GCC------------------AAATCTCCT---CCACTGACAAGG---

------------------------------GCGAATCTGAATCGCTCACAGTCTGTCTAC
ACCAAGTCTTCAGGTTGCCCACCAATGGCAAGTAGAGACGGCCCT---TTGCCGCATCCT
GGTCCACTCGTGCCCGCCCAGTCA---CTGTATCCCATGCGACTGAACTCAGTA------
---------------AAT------TCGTCTAGC---------------------------
------AGTGCC---AACACCAACGCGGCCAATCAAAACACTCAGAAC------------
------------------------CAACAACGT---------------CTTGGAGGCGAG
AGTGTTTATGGAGTGAGGCCTGTCAATAAT---------------------GGA------
---------GGTGGC---
------------------GGAATCTATGGAAAGATTCCTGGA------------------
---CGA------
---------ACAGAATCTATCTAT------GGAACTAGAAGACAGAAT------TCT---

---GAGGATTCAGCTTATGGCAGCTCG---------------------------------
------------TCCCATCAC------------TCCAATAATGGAAATCAGATGACAATA
CAGCAGCAGCAGCAGCAGCAACAACAACAGCAG---------------------------
---CAGCAGAATCGTAGCAACTACGGACATCTTGGA------------------------
--GGC---------

------------AAACCATTCCCCAGTGGTTCAAATTAT---GGACATTT----------
--------------------------------CAAGGAAATCGC----------------
-----CAGCAGCATCAT---TCACCGCAACAACAT--------------------------
----
-----CAACAACCACCCCCTCCTCCT------------------------------CAAC
ATCAGCAACAG---
-----------AATCAACAGCAACAACAACAA------------------CAGCAGCAGC
AGCAGCAACCGCAAAATGTTGGA------------------------------------A
TTCCGTCCCCACCTCCTCCCTACATCCACCACACCCAA----------------------
-----------------------CGC------AACTCCCCAATCTGCCCTAC-
>XP_039294816_Nilaparvata_lugens_Bib
ATGACAACAGAG--

------------------------------------CTGTCGCGT---------------
CTGGATTATCAAGTGATAACGCTCCTTGAAAAGCTGGAGACAGTTCGTCGCGATGCTCCA
CCT---

------CCCCGACTTATGCCGGCAAACGCAGAAATGCGAACCCTC---GAGTTCTGGAGG
TCTATAATTTCGGAATGTCTGGCATCGTTTTTCTATGTGTTCACCGTCTGCGGCGCCGCG
GCGGCTGTCACGTCTGGG--
--GCGACGTCGGGTCAGACA
ATTCTGGCGGCTGCACTCGCTTCAGGATTCACCATCGCTGCTCTCACCCAGTCTTTTGGA
CATATCTCAGGCTGTCACGTGAACCCAGCAGTGAGTGCAGCAATGGCGATCACGCGTAAC
ATTTCGCTGTTGCGTGGTCTGATGTTCGTTGTGGCTCAGTGCGGCGGCAGCATCGCAGGT
GCAGCTCTGCTCTACGGAGTGACAGTGCCTGGACGCCAAGGC------------------
------------------------------AGTCTGTCGGTGACAGTGCTGCAACTGCCG
CCCCAC---CTGACGCCGTGGGAGCGCTTTGGACTGGAGCTGATCCTCACCTTCGTGGTC
GTCTTCACCTACTTCGTC---GCCATGGACTCGCAGCGC---------------------
---CGCTGGACGGGC---------------ACGGCAGGCCTCACTGTGGGCGCCGCTTAC
CTCGCTGCTTCTCTTGTCTCGATGCCT------------TTCCTGAATCCAGCTAGAGCT
TTGGGTCCAGCATTTGTCATGAAT---------AAATGGGATAATCATTGGGTATTTTGG
TTTGCACCTATACTGGGAGGAGTATTGGCTGGATTCATCTATGAATTCATTTTCAATCCA
---CGCAGACAT---ACCAGGAGG------TCCAAGGAGTCGATT---GATGGA------
------------------------GAT------------TCATCAAGCATCCACTCTGAT
GAG------GAC---ACTTACGAT---GAGTTG---GAGAAGCCAAGA------------
---TCGACATAC
AATACTCTG---------AGA---------------------------------------
GCAGTTCCTCCAGGAAGTGATCTG---------------TACAGAGCA---GCTGGAGGA
------------TTGACACCCGCATCACCATCT------------------GGTGGAGGT
AACCCGTACTGCCCGAGTCTAGCC---------------TCAGCCAGTCTGTATTCAGCT
GCT---CCACCGTGC------AAG---------------------------TTGGAC---
CGAGTGGAGTCT---------------------------CTGTACGGCGGCACCAAGTCG
CTC---TAC---GCC------------------AAGTCGCCA---CCACTGACAAGA---

------------------------------GCCAACCTGAATCGCTCTCAGTCAGTCTAC
ACCAAGTCCTCGGGTTGTCCACCAATGGCCAACAGAGACGGTCCT---CTCCCGCACCCA
GGCCCACTTGTGCCCGCTCAATCC---CTCTACCCCATGCGTCTCAACTCGGTG------
---------------AAC------TCGTCTAGC---------------------------
------AGTGCC---AACACCAATGCGGCCAATCAGAACACCCAGAAC------------
------------------------CAGCAGCGG---------------CTCGGAGGCGAG
AGTGTCTACGGAGTGCGGCCTGTCAATAAC---------------------GGA------
---------GGTGGC---
------------------GGCATCTATGGCAAAATCCCTGGA------------------
---CGA------
---------ACCGAGTCCATTTAC------GGAACTAGGCGACAGAAC------TCA---

---GAGGACTCAGCCTACGGC---AGC---------------------------------

```
------------TCCCATCAT------------TCCACTAATGGAAACCAGATGAATATG
------------CAACAACAACAGCAGCAGCAG---------------------------
---CAACAGACTCGCAGCAACTATGGACATGTTGGA------------------------
------------------------------------------------GGC---------
------------------------------------------------------------
------------AAATCCTTCCCCAGTGGCTCGAACTAT---GGACACCT----------
--------------------------------CAAGGCAACCGC----------------
-----CAGCAACATCAT---TCGCCT----------------------------------
------------------------------------------------------------
-----CAACAACCTCCTCCACCTCCT------------------------------CAAC
ACCAGCAGCAGCAGCAGCAG---------------------------------------C
AACATCAGCCACAACAACAACAACAGCAACAG------------------AGCCAACAAC
AACAGCAGCAGCAAAATGTTGGA------------------------------------A
TCCCTTCCCCGCCTCCACCCTACATCCACCACACACAG----------------------
-----------------------CGC------AATTCGCCCAATCTGCCCTAC-
>EGI69716_Acromyrmex_echinatior_Bib
ATGGCAAACGAG------------------------------------------------
---------------------------------ACGTTGGCGCGC---GAA---------
GTGGACGCGCACATTGTGACTATTCTATCGAGGCTA---TCGGCCTTCCGAGAGAACAAT
GCC---------------------------------------------------------
---------------------------------------------------GAT------
GTGACTGACAGAAAGGCACCCATGTCCGTCGAGGCACGCAGTTTA---GAGCTATGGCGA
GCGGTGGCCATCGAATGCTTAGCCACATTCCTCTTCAGCCTCATAGTGGCTGGTGCGGCC
GCCGCCTCCGCTGTA---------------------------------------------
------------------------------------------TCCGGAAGCGGCCTCTCT
GCGTTAGCCACCGCTGTAGCTTCCGGTTTCGCTATCGCTGCGATCTCCCTCATCTTCAGC
CACATAAGCGGTGGACACGTGAATCCAGCGGTTTCGGTATCGTTTGCTTTGTGCAGACGG
ATTTCCCCACTTCGTGCTGTACTGTACGTGGCGGCACAATGCGGTGGCGGCATAGCTGGG
GCGGCGATGCTTTATGGAGTAACTACACCTTCGAGCACGCAG------------------
------------------------------ACTCTGACG---TCCCCTGGTCGATTAGGA
GGT------------CCAGTCGAGAGGCTTCTAATCGAAGTAGCACTATCAGTATTTATT
GTGCTAGCGCATTTC------ACTGTAGACTCACCTAGA---------------------
---TCACTGCCACCT---ACGATTTCTTCGAAAGCTTCCGCAGTTTTGGCTGCAGCCTAT
ACAGCAGCTACTCTCGTTTTTAGTCCT------------TTCCTGAATCCAGCCCGCGCG
TTGGGTCCGGCGTTCGTGATGAAT---------CGCTGGGACAATCACTGGGTGTACTGG
GTTGGCCCGCTGTCGGGAGGTGCTATAGCAGCTCTGCTGCACGAATACGTCCTGAGTTCC
------AGGAAG---ACGAAGGAC------TCGCGCGATTTAGAT---GACGGC------
------------------------GAC------------AATTCGTCCATGAGATCCGAC
GAA------GAC---ACGTACGAC---GATCTG---GATAAACCAAACGGGCCCAAGTTC
------------------------------------------------------------
------------------------------------------------------------
------CCCAGCGCGTACGCAACT---------------TATCGTCCA---GTCGCCGGC
------------GCA---------------------------------------------
---------------------------------------TCCTCGTCGATCTACAGTGCA
------CCGCCATCC------GCG---------------------------TTGGAG---
CGTGTCGAGTCG---------------------------ATTTATGGTGGTACCAAGTCG
CTC---TAC---TGC------------------AAATCACCG---CCGTTGACACGC---
------------------------------------------------------------
```

------------------------------GCTAATCTGAACCGTTCACAAAGCGTTTAC
GCGAAATCGACGTCGGGGCCGCGG------------GATGGTTTGATGATCCCGAAACCG
GGCCCACTGGTGCCAGCACAGAGT---CTGTATCCAATCAGATTAGGTCAGAAT---AGC
TGTCAAAAC---GGTAATCGTGAGATCCAGTCCGGACAGCCACTCGGCGGTCCA---CCG
AGCCAATCGGCACAGAATCACAACAGCACCAATCAGAACATGCAGAAT------------
------------------------CAACTGCAA---------------CAATGCACGCAG
AGCATCTACGGCATCCGCGGGATCTCCACG---------------------GGTCTCAGC
ACTCGG---GAGAAC---
------------------GGTATTTATGGA------------------------------
--GTTTCTACC---
---------GGATCCACCATCTAC------GGTCGTGCACCCGCACCACCGCCTAGC---
---------------------------------AGCCAAAATTCGCAGCAGTCCGGGCAG
---AGTGGCCAGTCCTCGATACAAAATCATCCACCACCGCAGCCTCCATCG---------
---CAGCAGCAGCAGCAGCAGCAGCAGCAGCAGCAGCAG---------CAACTTCAGAAC
GGACCATTATCGAGCAATTCGGATAATGGG---GCCCCC---------------------
------------------------------AGGCGG------------------------
---CCCGAGAGCATGTATGGCCATGTG---------------------------------
------------TCGAGACGC---------------------------------------

------AGTGATGATTCCGCGTATGGTAGTTACCAGAGTTCCACGCGAGGAAATTACGGC
AAGGTACCTGGACCTCCG---------GGCCCACCCAAT---------GGACCTCCACAA
TACCGCGATCAGGGA---
------------AGACCGAGTACAGCCGGG------------------------------
-----CCTCTTCAGCAAAGTCAAAGCGTG---CAG---AGTAAC---------------T
TC------CAAAGAAAC---TCGCCGAACCCT----------------------------

---CAATAT-
>XP_012055670_Atta_cephalotes_Bib
ATGGCAAACGAG--
---------------------------------ACGTTGGCGCGC---GAG---------
GTGGACGCGCACATTGTGACTATTCTATCGAGGCTA---TCGGCCTTCCGAGAGAACAAT
GCC---
---GAT------
GTGACTGATAGAAAGGCACCCATGTCCGTCGAGGCACGCAGTTTA---GAGCTATGGCGA
GCGGTAGCTGTCGAATGTTTGGCCACATTTCTCTTCAGCCTCGTAGTGGCTGGTGCAGCC
GCCGCCTCCGCTGTA---
--TCCGGAAGCGGCCTCTCT
GCGTTAGCCACCGCTGTAGCTTCCGGTTTCGCTATCGCTGCGATCTCCCTCATCTTCAGC
CACATAAGCGGTGGACACGTGAATCCAGCGGTTTCGGTATCGTTTGCTTTGTGCAGACGG
ATTTCCCCACTTCGCGCTGTACTGTACGTGGCGGCACAATGCGGTGGCGGCATAGCTGGG
GCGGCGATGCTTTATGGGGTAACTACACCTTCGAGCACGCAG------------------
------------------------------ACTCTGACA---TCCCCTGGTCGATTAGGA
GGC------------CCAGTTGAGAGGCTTCTAATCGAGATAGCACTATCAGTATTTATT

GTGCTAGCGCATTTC------ACTGTAGACTCACCTAGA---------------------
---TCACTGCCACCT---ATGATTTCTTCGAAAGCTTCCGCAGTTTTAGCTGCAGCCTAT
ACAGCAGCTACGCTTGTTTTTAGTCCT------------TTCTTGAATCCAGCCCGCGCG
TTAGGTCCGGCGTTCGTGATGAAT---------CGCTGGGACAATCACTGGGTGTATTGG
GTTGGTCCGCTGTCGGGAGGTGCTATAGCAGCTCTGCTGCACGAATACGTCCTGAGTTCC
------AGGAAG---ACAAAGGAT------TCGCGCGATTTAGAT---GACGGC------
------------------------GAC------------AATTCGTCCATGAGATCCGAC
GAA------GAC---ACGTACGAT---GATCTG---GACAAACCGAACGGGCCCAAGTTC

------CCCAGCGCGTACGCGACT---------------TATCGTCCA---GTCGCCGGC
------------GCA---
---------------------------------------TCCTCGTCGATCTACAGTGCA
------CCGCCATCC------GCG---------------------------TTGGAG---
CGTGTCGAATCG---------------------------ATTTACGGTGGTACCAAGTCG
CTC---TAC---TGC------------------AAATCACCG---CCGTTGACACGC---

------------------------------GCCAATCTGAACCGTTCGCAAAGCGTTTAC
GCGAAATCGACGTCGGGACCGCGG------------GATGGTTTGATGATCCCGAAACCG
GGCCCACTGGTGCCAGCGCAGAGT---CTGTATCCAATCAGATTAGGT------------
---CAGAAT---GGCAATCGTGAGACCCAGTCCGGACAGCCACTCGGCGGTCCA---CCA
AGCCAATCGGCACAGAATCACAATAGCACCAATCAGAACATGCAGAAT------------
------------------------CAATTGCAA---------------CAATGTACGCAG
AGCATCTACGGTATCCGCGGAATCTCCACG---------------------GGTCTCAGC
ACTCGG---GAGAAC---
------------------GGTATTTACGGA------------------------------
--GTTTCTACT---
---------GGATCCACCATCTAC------GGTCGTGCACCCGCACCACCGCCTAGC---
---------------------------------AGCCAAAATTCGCAGCAATCCGGGCAG
---AGTGGCCAGTCCTCGATACAAAATCATCCGCCACCGCAGCCTCCATCGCAACAGCAG
CAACAACAGCAACAGCAGCAGCAGCAGCAGCAGCAGCAG---------CAACTTCAAAAC
GGACCATTATCGAGCAATTCGGATAACTGG---GGCCCCCAGGCGGCC------------

>AEAQ01016184_Solenopsis_invicta_Bib
ATGGCGAACGAG--
---------------------------------ACGTTGACGCGC---GAG---------
GTGGACGCGCACATTGTCACCATCCTATCGAGACTA---TCGGCTCTCCGGGAGAATAAT
GCC---
---GAC------
GTGACGGACAGAAAGGCGCCCATAGCCGTCGAGGCTCGCAGTTTG---GAGCTGTGGCGA
GCGGTGGCTGTCGAATGCCTCGCCACTTTCCTCTTCTGCCTCGTAGTGGCCGGCGCGGCC
GCCGCCTCCTCCGTA---
--TCCGGAAGCGGCCTTTCC
GCGTTAGCGACCGCTGTAGCTTCCGGTTTCGCCATCGCCGCGATCTCCCTCATCTTCAGC
CACATAAGCGGTGGACACGTGAATCCGGCAGTCTCGGTATCGTTCGCTTTGTGCAGACGG
ATTTCCCCACTTCGTGCGATGCTGTACGTGGCGGCACAATGCGGTGGCGGTATTGCTGGG
GCGGCGATGCTCTATGGGGTAACTACACCTTCGAGTACGCAG------------------
------------------------------ACTCTGACG---TCTCCTGGTCGGCTAGGA
GGC------------CCAATCGAAAGACTTTTAATCGAAATAGCACTATCGGTGCTTATC
GTGCTAGCCCATTTC------TCTGTGGACTCACCTAGA---------------------
---TCACTGCCA------ATGATTTCTTCAAAAGCTTCTGCAGTTTTGGCAGCAGCCTAT
ACAGCTGCTACTCTCGTTTTCAGTCCT------------TTCTTGAATCCAGCGCGCGCG
TTGGGTCCGGCCTTCGTGATGAAT---------CGTTGGGAAAATCACTGGGTGTACTGG
GTGGGTCCGCTGTCGGGAGGTGCCGTAGCGGCTCTGCTGCACGAATACGTCCTGAGTTCC
------AGACGC---ACAAAGGAC------TCACGCGACATGGAC---GACGGC------
------------------------GAT------------AACTCGTCCATGAGATCCGAC
GAG------GAC---ACGTACGAC---GATCTG---GACAAGCCGAATGGACCAAAGTTC

------CCCGGCGCGTACGCGACC---------------TATCGTCCG---GTTGCCGGC
------------GCG---
---------------------------------------TCCACGTCGATCTACAGCGCA
------CCACCGTCC------GCG---------------------------TTGGAG---
CGGGTCGAGTCG---------------------------ATCTACGGCGGTACCAAGTCG
CTC---TAT---TGC------------------AAATCACCG---CCATTGACGCGC---

------------------------------GCCAATCTGAATCGTTCTCAGAGCGTTTAC
GCGAAGTCGACGTCGGGACCACGG------------GATGGTTTAATGATCCCGAAGCCG
GGCCCGCTGGTGCCGGCGCAGAGT---CTGTATCCCATCAGACTAGGTCAGAAT---GGT
TGTCAGAAC---GGTACCCGTGAGATCCAGCCCGGA------CTTGGCGGGGTT---CCA
CCGAACCAGACGCAGAATCACAACAGCACCAACCAGAATATGCAGAAC------------
------------------------CAACTGCAG---------------CAGTGCACGCAG
AGTATCTACGGTATCCGCGGGATCTCCACG---------------------GGTCTCAGT
ACTCGG---GAAAAC---
------------------GGTATCTATGGG------------------------------
--GTGTCCACC---
---------GGATCTACCATTTAC------GGTCGCGCGCCGGCACCACCGCCGAGC---
---------------------------------AGTCAAAATTCGCAGCAATCCAGTCAG
---AGCGGACAATCCTCGATACAG------------------------------------

>XP_011268932_Camponotus_floridanus_Bib
ATGACGACCGAA--
---------------------------------TCGTTGTCGCGC---GAG---------
GTGGATGCACACATCGTCACCATCCTGACGAGGCTG---TCGGCCCTCCGGGAGAACAAT
GCC---
---GAC------
GTCGTCGAGAGGAAGGCACCGATGTCCATCGAAGCTCGCAGTCTG---GAGCTGTGGCGT
GCGGTAGCTGTTGAGTGCTTCGCTACTTTCCTCTTCAGCCTGGTGGTATCCGGCGCGGCC
GCGGCCTCCGCCGTA---
--TCCGGAAGCGGACTCTCC
GTGTTAGCTACCGCGGTGGCCTCTGGCTTTGCCATCGCCGCGATCTCTCTCATCTTCAGC
CATGTCAGCGGTGGACACGTGAATCCAGCGGTCTCCGTGTCGTTTGCTTTGTGCAGACGG
ATCTCTCCGCTTCGCGCGGTGCTCTATATAGCGGCGCAATGTGGCGGCGGCATAGCTGGG
GTGGCGATGCTTTATGGGGTAACTACACCCTCGAATACGCAG------------------
------------------------------ACCCTGACA---TCCCCCGGTCGACTAGGA
GGT------------CCGATCGAGAGGCTTTTAGTCGAAATAGCACTGTCTGTGCTTATC
GTGCTAGCTCATTTT------GCTGCGGATTCATCCAGA---------------------
---GCATTACCACCT---ACGATTTCTTCAAAAGCTTCCGCGGTTTTGGCCGCAGCTTAT
ACAGCGGCAACTCTCGTTTCCAGTCCC------------TTTCTAAATCCAGCCCGCGCG
TTGGGCCCGGCCTTCGTTATGAAT---------CGCTGGGACAATCATTGGGTATACTGG
GTGGGCCCGCTTTCGGGAGGCGCCGTGGCGGCTCTACTTCACGAGTACGTCCTAAGTCCC
------AGGCCC---GCGAAGGAT------TCGCGCGACATGGAC---GACGGC------
------------------------GAT------------AATTCGTCCATGAGATCCGAC
GAG------GAT---ACGTACGAC---GATCTG---GACAAACCGAACGGGCCCAAGTTT

------CCCAGTGCATACGCAACC---------------TATCGTCCA---GTGGCTGGC
------------GCG---
---------------------------------------TCGTCGTCGATCTACAGCGCA
------CCACCAACC------GCG---------------------------TTGGAA---

CGCGTCGAATCG---------------------------ATCTACGGCGGCACTAAATCG
CTC---TAC---TGC------------------AAATCGCCA---CCGTTGACGCGT---

------------------------------GCCAATCTGAATCGCTCCCAGAGCGTTTAC
GCGAAATCGACGTCGGGCCCGCGG------------GATGGCCTGATGATCCCGAAGCCG
GGTCCGCTGGTGCCGGCGCAGAGT---CTGTATCCTATCAGACTTGGCCAGAACGGTGGT
TGCCAGAGCGGTGGTAACCGCGAGAGCCAACCTATA------CCTGGTGGTCCG---CCG
AGCCAGTCGTCGCAGAATCACAACAGCACTAATCAGAACATGCAGAAC------------
------------------------CAATTGCAG---------------CAGTGCACGCAG
AGCATCTACGGCATCCGCGGGATCTCCACG---------------------AGTCTTAGT
ACACGG---GAAAAC---
------------------GGTATCTATGGT------------------------------
--GTATCTACT---
---------GGTTCCAGCGTCTAC------GGTCGTGCACCAGCACCACCGCCGAGC---
---------------------------------AGCCAGAATTCGCAGCAGTCCGGTCAA
AGTAGCGGTCAGTCCTCGATACAGAATCATCCGCCACCACCGCCGCCGTCGCAGCAGCAG
CAGCAGCAGCAACAGCAGCAGCAGCAGCAGCAGCAGCAA---------CAACATCAGAAC
GGACCGTTGTCGAGTAATTCGGATAACGGGCCAAGCACC---------------------
------------------------------AGACGG------------------------
---CCCGAAAGCATGTACGGCCACGTT---------------------------------
------------TCGAGACGC---------------------------------------

------AGTGACGATTCCGCGTACGGTAGCTATCAGAGCTGCACGCGAGGAAATTATGGC
AAAATGCCT---------------------------------------GGACCACCGCAA
TATCGCGATCAGGGT---
------------AGACCGAGTCCGGCCGGG------------------------------
-----CCCCTTCAGCAAAGTCAGAGCGCG---CAG---AATAAC---------------T
TC------CAAAGAAAC---TCGCCGAATCCT----------------------------

---CAGTAT-
>XP_003399074_Bombus_terrestris_BIb
ATGACCACGGAA--
---------------------------------ACGTTTTCAAGA---GAG---------
TTAGATGGTCACATCGTGACCTTGCTTACGAGGATT---TCTTCCCTGAAGGAAAATAAC
ATG---
---GAA------
CTAACAGAGAAGAAAGCACCTATGTCGATAGAGGCTCGCAGTTTG---GAACTCTGGAGA
GCAGTAGCTGTAGAGTGTTTCGCTACCTTTCTCTTCAGTCTCGTTGTATCCGGCGCAGCC
GCATCCTCCGCAGTT---
--TCCGGAAGCGGACTCTCT
GTCTTAGCTACCGCTGTAGCATCCGGTTTTGCGATTTCGGCGATCTGCTTCATCTTTGGT
CATATCAGTGGTGGCCACGTGAATCCAGCAGTCTCACTATCTTTCGCTTTGTGTCGACGA
ATCTCCTTCCTCCGAGCTGCTCTGTACATCGCCGCACAGTGTGGAGGCGGTATAGCCGGA

```
GCTGCGATGCTGTATGGAGTGACTACACCTTCCAATACGCAG------------------
------------------------------ACCTTAACT---TCCGTAGGTCGTCTCGGA
GGG------------CCTATCGAGAGGCTCTTGGTAGAATTAGCACTTTCTGTGCTGATT
GTCCTATCTCATTTT------ACCTCAGAATGCTCAAAA---------------------
---ATACTACCAATG---TCAGTTTCTTCGAAACCAGCTGGCGTATTAGCTGCAGCCTAC
ACGGCAGCCACTTTGGTTTCGAGTCCA------------TTCTTAAATCCCGCACGCGCA
CTTGGACCAGCATTCGTCACCAAT---------CGTTGGGACAACCATTGGGTGTACTGG
GTAGGTCCACTGTCGGGAAGCGCAGTGGCAGCCCTTCTTCACGAGTATGTTCTAAATCCC
------AAACGA---TCTAGAGAC------CCACGGGATATGGAT---GACGGT------
------------------------GAC------------AATTCATCAATGAGATCCGAC
GAG------GAC---ACCTACGAC---GATCTG---GACAAACCAACGGGTCCCAAATTT
------------------------------------------------------------
------------------------------------------------------------
------CCCAGCGCCTATGCAACC---------------TATCGCCCA---GTTGCTGGA
------------GCA---------------------------------------------
---------------------------------------TCTTCCTCGATCTACAGTACG
------CCTCCGACA------GCC---------------------------TTAGAA---
CGTGTCGAATCC---------------------------ATTTACGGGGGAACCAAATCG
CTC---TAC---TGT------------------AAATCCCCA---CCCTTAACCAGG---
------------------------------------------------------------
------------------------------GCAAATTTAAATCGTTCGCAAAGTGTATAC
GCTAAATCAACTTCCGGAGCCCGA------------GACGGCCTAATGATCCCCAAACCG
GGTCCTCTAGCCCCCGCACAAAGC---CTGTACCCGATTAGAATAGGTCAAACA---GGT
TCAAATTCA---GGA---AGAGATAGTCAACAGCAA------AATGGATCAAATGGTCAG
AATCAAAATTCTCAAAATCATAACAGTACCAACCAAAATATGCAGAAT------------
------------------------CAACTGCAA---------------CAGTGCACGCAG
AGCATTTATGGAATCAGAGGAATTTCCACG---------------------AGTCTTAGT
ACGAGG---GAGAAT---------------------------------------------
------------------GGTATCTATGGT------------------------------
------------------------------------------------GTATCTACG---
---------GGGTCCAGCATATAT------GGCCGAGCTCCAGCTCCTCCTCCGAGT---
---------------------------------AGTCAGAATTCTCAACAATCAGGTCCG
---AATGTCCAATCTTCCACTCAAAATCATCAT---------------------------
CAGCCACAACAACAACAGCAACAGCAGCAACAACAACAG---------CAGCATCAGAAT
GGAACGATATCCACCTGCTCTGATAACGCAGCAAACTCT---------------------
------------------------------AGAAGA------------------------
---CCGGAAAGCATGTACGGGCATATT---------------------------------
------------TCTAGACGC---------------------------------------
------------------------------------------------------------
------AGTGACGATTCTGCGTATGGCAGTTATCAAGGTTCGACGCGAGGCAATTACGGA
AAGGTACCTGGACCTCCTGGATCAACGGGTCCCCCTAACGGACCACCTGGTCCTCCTCAG
TACCGCGAACAAGGA---------------------------------------------
------------AGACCCAGTCCAGCTGGG------------------------------
-----CCATTGCAACAGAACCAG---------CAA---AATAAT---------------T
TT------CAAAGAAT---TCGCCAAATCCT-----------------------------
------------------------------------------------------------
------------------------------------------------------------
------------------------------------------------------------
```

```
------------------------------------------------------------
------------------------------------------------------------
------------------------------------------------------------
-----------------------------------------------CAATAT-
>XP_024222043_Bombus_impatiens_BIb
ATGACCACGGAA------------------------------------------------
---------------------------------ACGTTTTCAAGA---GAG---------
TTAGACGGTCACATCGTGACCTTGCTTACGAGGATT---TCTTCCCTGAAGGAAAATAAC
ATG---------------------------------------------------------
---------------------------------------------------GAA------
CTAACAGAAAAGAAAGCGCCTATGTCGATAGAGGCTCGCAGTTTG---GAACTCTGGAGA
GCAGTAGCTGTGGAGTGTTTCGCTACCTTTCTCTTCAGTCTCGTTGTATCCGGCGCAGCC
GCATCCTCCGCAGTT---------------------------------------------
------------------------------------------TCCGGAAGCGGACTCTCT
GTCTTAGCTACCGCTGTAGCATCCGGTTTTGCAATTTCGGCAATCTGCTTCATCTTTGGT
CATATCAGTGGTGGCCACGTGAATCCAGCAGTCTCGCTATCTTTCGCTTTGTGTCGACGA
ATCTCCTTCCTCCGAGCTGCTCTGTACATCGCCGCACAATGTGGAGGCGGTATAGCCGGA
GCTGCGATGCTGTATGGAGTGACTACACCTTCCAATACGCAG------------------
------------------------------ACCTTAACT---TCCGTAGGTCGTCTCGGA
GGA------------CCAATCGAGAGGCTCTTGGTAGAATTAGCACTTTCTGTGCTGATC
GTCCTATCTCATTTT------ACCTCAGAATGCTCAAAA---------------------
---ATACTACCAATG---TCAGTTTCTTCGAAACCAGCTGGCGTATTAGCTGCAGCTTAC
ACGGCAGCCACTTTGGTTTCGAGTCCA------------TTCTTAAATCCCGCACGCGCA
CTTGGACCAGCATTCGTCACCAAT---------CGTTGGGACAACCATTGGGTGTACTGG
GTAGGTCCACTGTCCGGAAGCGCAGTGGCAGCCCTTCTTCACGAGTACGTTCTAAATCCC
------AAACGA---TCTAGAGAT------CCACGGGATATGGAC---GACGGT------
------------------------GAC------------AATTCATCAATGAGATCCGAC
GAG------GAT---ACCTACGAC---GATCTG---GACAAACCAACGGGTCCCAAATTC
------------------------------------------------------------
------------------------------------------------------------
------CCCAACGCCTATGCAACC---------------TATCGTCCA---GTTGCTGGA
------------GCA---------------------------------------------
---------------------------------------TCTTCCTCGATCTATAGTACG
------CCTCCGACA------GCC---------------------------TTAGAA---
CGTGTTGAATCC---------------------------ATTTACGGGGGAACCAAATCG
CTC---TAC---TGT------------------AAGTCCCCA---CCCTTAACTAGG---
------------------------------------------------------------
------------------------------GCAAATTTAAATCGTTCGCAAAGTGTATAC
GCTAAGTCAACTTCCGGAACCCGA------------GACGGCCTAATGATCCCCAAACCG
GGTCCTCTAGCCCCCGCACAAAGT---CTATACCCGATTAGAATAGGTCAAATA---GGT
TCAAATTCA---GGA---AGAGACAGTCAACAGCAA------AATGGATCAAATGGTCAG
AATCAAAATTCTCAAAATCATAACAGTACCAACCAAAATATGCAGAAT------------
------------------------CAACTGCAA---------------CAGTGCACGCAG
AGCATTTATGGAATCAGAGGAATTTCCACA---------------------AGCCTCAGT
ACAAGG---GAGAAT---------------------------------------------
------------------GGTATCTATGGT------------------------------
------------------------------------------------GTATCTACG---
---------GGGTCCAGCATATAT------GGCCGGGCTCCAGCTCCTCCGAGT---
```

```
---------------------------------AGTCAGAATTCTCAACAGTCAGGTCCA
---AATGTCCAATCTTCCACTCAAAATCATCAT---------------------------
CAGCCACAGCAGCAGCAGCAGCAGCAGCAACAACAACAG---------CAACATCAGAAT
GGAACGATATCCACCTGCTCTGATAACGCAGCAAACTCT---------------------
------------------------------AGAAGA------------------------
---CCGGAAAGCATGTACGGGCATATT---------------------------------
------------TCTAGACGC---------------------------------------
------------------------------------------------------------
------AGTGACGATTCTGCGTATGGCAGTTATCAAGGTTCGACGCGAGGCAATTACGGA
AAGGTACCTGGACCTCCTGGAACAACGGGTCCTCCTAACGGACCACCTGGTCCTCCTCAG
TACCGCGAACAAGGA---------------------------------------------
------------AGACCCAGTCCAGCTGGG------------------------------
-----CCATTGCAACAGAACCAG---------CAA---AATAAT---------------T
TT------CAGAGGAAT---TCGCCAAATCCT----------------------------
------------------------------------------------------------
------------------------------------------------------------
------------------------------------------------------------
------------------------------------------------------------
------------------------------------------------------------
------------------------------------------------------------
-----------------------------------------------CAATAT-
>KAF7384315_Vespula_vulgaris_Bib
ATGACGACGGAA------------------------------------------------
---------------------------------TCATTGCCACGC---GAA---------
ATGGACGCGCACATCGTGAATCTTTTGACGAGACTT---TCCAATATCAGAGAGAACAAT
CCT---------------------------------------------------------
---------------------------------------------------GAT------
TTATCCGAAAGAAGGATACCAATGTCAACCGAGGCGCGAAGTTTA---GAACTTTGGAGG
GCGGTCGCGGTCGAGTGCCTCGCAACTTTCCTCTTCGGCTTGGTTGTATCCGGAGCAGCC
TCTTCTACCGCAGTC---------------------------------------------
------------------------------------------TCCGGAAGCGGACTCTCC
ATCCTGGCGACCGCAATTGCTTCCGGTTTCGCTGTCTCAGCGATCTATCTCATCTTTGGC
CATGTTAGCGGAGCACACGTAAATCCTGCCGTATCAATATCCTTTTCCTTATGTCGTCGT
ATCTCACCGTTACGCGCAGCTCTTTACATAGCAGCTCAATGTGGTGGAGGTATAGCAGGA
GCTGCGATGCTGTACGGCGTTACAACACCCGCGAATACGCAA------------------
------------------------------ACGTTAACA---TCACCCGGACGTATCAGC
GGA------------CCTATCGAAAGACTTTTAGTCGAATTGGCACTTTCGGTTCTTATC
GTTTTGGCCCACTTT------GCTGCGGAGACATCAAGG---------------------
---TCTTTATCCATT---GGAATCTCCTCGAAGTCAGCCGGCGTTTTGGGTGCTGCATAT
ACAGCTGCAACTCTCGTTTCGAGTCCG------------TTCGTGAATCCAGCACGTGCT
TTGGGGCCTGCTTTTGTAATGAAT---------CGTTGGGAGAATCATTGGGTTTATTGG
GTCGGTCCGATATCAGGTGGTGCCGTCGCGGCATTGCTTCACGAATACGTTCTCAGTCCG
------AGAAGA---TCGAGAGAT------TCTCGCGAGATGGAC---GACGGT------
------------------------GAT------------AATTCATCGATGAGATCGGAC
GAG------GAC---ACTTACGAT---GACTTG---GACAAACCAAGCGGGCCAAAGTTT
------------------------------------------------------------
------------------------------------------------------------
------CCAGGAGCTTATGCTACC---------------TATCGTCCA---GTCGCCGGT
```

```
------------GCC---------------------------------------------
---------------------------------------TCCTCTTCGATCTACAGTGCA
------CCACCGACG------GCT---------------------------TTGGAA---
CGCGTAGAATCG---------------------------ATCTACGGGGGCACCAAGTCT
CTC---TAT---TGC------------------AAATCTCCA---CCTCTAACAAGA---
------------------------------------------------------------
------------------------------GCGAATCTGAATCGTTCGCAAAGCGTTTAC
GCTAAATCGACGTCCGGTCCACGG------------GACGGTTTGATGATACCGAAACCA
GGTCCACTTGTACCAGCTCAAAGT---CTTTATCCTATGAGAATAGGACAATCC---TCA
ACTCAGAATGCAGGA---AGGGATGGGCAACAA------------CCGGTAACTGGTCCC
AATCAATCATCCCAAAATCACAACAGTACCAATCAAAACATGCAGAAT------------
------------------------CAATTGCAA---------------CAATGTACGCAG
AGTATATACGGCATTAGAGGAATATCGACG---------------------AGTCTGAGT
ACGCGG---GAGAAT---------------------------------------------
------------------GGAATTTATGGT------------------------------
------------------------------------------------ATGTCGACG---
---------GGATCTAGTATATAT------GCTAGAGCACCTGTACCACCACCGAGT---
---------------------------------AGTCAAAGCTCGCATCAATCTGGACAG
---AGT---CAATCCTCTACGCAAGGTCATCAA---CAGCAGCAACAACAACAACAACAA
CAACAACAACAACAACAATCTCAGCAACAGCAACAGCAA---------CAACATCCAAAT
GGCACACTCTCAACCAATTCTGACAACGCAGCAAATTCG---------------------
------------------------------AGGAGA------------------------
---CCCGAAAGCATGTACGGTCATGTA---------------------------------
------------TCAAGACGT---------------------------------------
------------------------------------------------------------
------AGCGATGATTCTGCTTATGGTAGCTATCAAGGTTCGACCAGAGGAAATTATGGT
AAAGTTCCTGGTCCA---------TCTGGACCACCTAATGGACCACCTGGACCACCTCAA
TATCGTGATCAAGGT---------------------------------------------
------------AGACCCAGTCCAGCAGGG------------------------------
-----CCTCTTCAACAAAACCAGAAT------CAA---CAA------------------A
TG------CTTCGAGAG---TCT-------------------------------------
------------------------------------------------------------
------------------------------------------------------------
------------------------------------------------------------
------------------------------------------------------------
------------------------------------------------------------
------------------------------------------------------------
-----------------------------------GGCATTGAACGA-------
>XP_016841019_Nasonia_vitripennis_Bib
ATGACGACGACCACGGCTACGGTC------------------------------------
------------------------ACGGCGGCGAGTCTCACCCGG---GAC---------
GTCGACTCGCACATCGTCACGCTTCTGAGTAAGCTC---TCGGCACTGCGGGAGCACAAC
CAGCAGCGC---------------------------------------------------
---------------------------------------------------GAGCAGCAG
CACGACGAGCACAAGCAGCCGATGTCAATCGAGGCCAGGAGTCTG---GAGCTCTGGAGG
TCCGTCGCCGTCGAGTGTTTCGCCACCTTCCTCTTCGCCTTCGTCGTCGCCGGGGCTGCT
ATAGCGTCGTCTGTA---------------------------------------------
------------------------------------------TCCGGAAGCGGACTGAAC
```

GTGCTGGCAACTGCTATAGCAAGTGGATTCGCCATCGCGGCTGTGCAGCTCATCTTCGGA
CCCGTCAGCGGTGGGCACGTGAACCCGGCAGTCTCGGTGTCGTTCGCCCTGTCGCGGAAG
GTGTCACCCCTCAGGGCTGGACTGTACGTCGCGGCCCAGTGCGGCGGTGGAATCGCCGGC
GCGGCTATGCTCTATGGAGTCGCC------AATTCGCCGCAG------------------
------------------------------ACCCTGATGATCTCCCCGGGACGTCTGAGC
GAA---------CAGGCGCTGCTGAAGCTGCTCGTCGAGCTGTCCCTCTCGGTCCTCGTG
GTTCTCGCTCACTTC------GCCAGCGAGTCGCCCAAG---------------------
---ACTCTGCCGTCG---ACCGTCTCGGCCAAACCCGCCTGCGTTCTCGCAGCCGCCTAC
ACCGCCGCTACTCTGGTTTCGAGCCCC------------TTCCTGAACCCGGCGCGAGCC
TTGGGCCCGGCCTTCGTGATGAAC---------AAGTGGGACAACCACTGGGTCTACTGG
ATCGGCCCGATCGCCGGTGGCGCCATCGCTGCGGTGCTTCACGAGTACGTGCTCAGCAAC
---AACCGTCGC---TCGCGGGAT------AACCGTGATCTCGAC---GACGGC------
------------------------GAG------------AGCTCGTCCATGCGGTCGGAC
GAC------GAG---ACCTACGAC---GATCTC---GACAAGGGC---GCCCCCAAGTTT

------CCCGGAGCCTACGCCACC---------------TACCGGCCG---GTCGCCGGT
------------GCT---
--GCCTCCATCTACGGGCCA
------CCTCCGAGC------GCC---------------------------CTGGAA---
CGCGTCGAGTCC---------------------------ATCTACGGCGGCACCAAGTCC
CTC---TAC---TGC------------------AAGAGTCCG---CCGCTGACCCGG---

------------------------------GCTAACCTCAACAGGTCTCAGAGCGTCTAC
GCCAAGTCCACGTCCGGTACGCGC------------GACGGTCTGATCCTTCCCAAACCC
GGACCTCTGGTGCCAGCTCAGAGC---CTGTACCCCATCAGACTCAACGCCGTC---AGC
TCTCAGGCCCCGGGA---CGCGAACTCATGCCCAGTGGTCAACCCCAGAGCAATGGCCAG
CCCTCGACTACTCAGAACCACAACAGCACCAACCAGAACATGCAGAAC------------
------------------------CAACTGCAG---------------CAGTGCACTCAG
AACATCTACGGTATTCGGGGTATCACCACG---------------------AGTCTGAGC
ACCCGC---GAC--
------------------GGTATCTACGGCCATCTG------------------------
---------------------------GGTAGTCGCGAGAACGGTACCATCACCACTGGC
GGCGGTGGCGCCTCCAACATCTAC------GGCAGGGCCCCGGCACCTCCACCATCC---

------------------------AACCACGGACCC------------------------
CCTGGCCAACAGCAGCAACAACAACAACAACAGCAACAG---------CAGGGCCAGATT
CAGAGTCCCCCCACCCGT---GAGAACGGTCAGGGACCTAGTATACCACCCCACCAG---
---------------------GACGAGGCCAGACGCTCTGAGCCACCAAGA---------
---CCCGAGAGCATGTACGGTCACGTG---------------------------------
---------------AGACGC---------------------------------------

------GGAGATGACTCAGCCTACAGTACCTACCAGAGCTCGTCACGGAGCACCTACGGC
AAGGCCCCGGGACCCCCA------------CCACCATCCGGCTGG---------------

------------CGTCCATCGCCCGTCAAC-----------------------------G
TA---CCCCCG---------------------CAG---GGCAAC---------------T
TC------ACCAGACAC---TCGCCCAACCCA----------------------------

--CCCCAGTAC-
>TRY73210_Tigriopus_californicus_Bib
ATGGCT--
---------------------------------------GCCAGACTCGATTCG------
CTCCATGCTCAATTGGGTTGGCTTCTGGAAAATGCTCAATCGTTACTAGCCGATTCT---
GCCCCGTCCTCG---------ACCACAGGGGGCGGCAACCTCTATTATGGCGGTGGGAGT
GGTCGA---GGAGGCCATGGTTCTACCATAGGC---------------CAAGCCCCACCC
CCGGTGTATTCATTCGTAGCTATGCAAAGCGAGATCCGAACCCTG---GAATTTTGGCGG
TCCATAATTGCTGAATGCATGGCCACTTTTGTGTATGTGATGATTGTTTTATCCGTGTCC
ACCACCTCGTCCGAACACACG---------------------------------------
---CAA
CAGACGAACTGTGCCCTGGCTTCTGGTCTCGTTATGGTCATGTTGGTGGCCGTCTTCGCC
CGTGTGTCTGGCAGTCACACCAACCCAGCCATAACCCTAGGGATGACCCTAACTCGATAC
ATATCGCCCTTGAGGTCTGTTCTCTACATGTGTGCGCAATGCGGGGGTGGCATTGCCGGC
GCCGCTTTGACTTTGGGCGTT------------CAC---GGA------------------
------------------------------CGAATTAAT------GACCCGGCTCTC---
---------AAATCGGAACGAGGCACGTTTGGAATGGAATACGTTCTGTCCTTCTTGGTG
GTGTACACATACTTCGCT---GCCAAAGAAACTCGAGTA---TCTCCCAGCCATCGCACA
CGAACTCTAGTACCT---GGCCACATTCGCATCAATCCAATCTTTGTTGGGGTCGCTCAT
---GCAGCCTGTCTAGTGGCTTGGAAG------GGA---TCTCTGAACCCTGCCAGAGCT
CTTGGGTCCGCCTTCGTCAGCCGTAGTCCTGATCGGTTCGATCAACATTGGGTATTTTGG
GTGGGTCCTCTGCTAGGGGCAATCACTGGTGCCTTTGCCTTCGAGTACATCTTTAACCCA
---AGGCGAAAAGCGTTTACGTTTTCCCGCTTAAGAGGCCCTCAC---GATCTT------
------------------------GAA------------AACGTCTCCATTCGCTCCGAG
GAC------GAC---ATGATTGAC---GATTTA---GAGCGGGCCAAGCAATACCGTGCG

---AACATC---
------ATGCAAGATTTC---CAA------------------GATCCC------------
------------ACACACCCTGCCTCTTCCCGA------------------GGGGGT---
---------CCT---------------------------------------TACAGT---
---------------AAA---AGACCGGGAGCTTCC---GGGATGAATCATATCAAC---
GGAGCTGACTCG---------------------------GTGTATGGGGGGTCCAAGTCC
GTG---TATAACCAA---------------------TCG---ATTTATGATTCCAAG---
------TCCATGTAC---GAT---GGAACCAAATCAATGTATGATGGATATGAT---GGG
------ATG---------GACCGAAATGCAGCCAATCTGAGACGTTCCCGATCAATTCAC
ACCAAGTTCCCGCCCCCTAACCAT------------------------------------

------------------------GCTGTGACT---------------CGACGAAACCCT
TACGACTAC---CTTCCGGACGAACCC---------------------------------
ACCAAG---GCGGACATGATGCAAACA---------------------------------

------------GAGAGT------------GGGCCT---CGACTCATGGGCAAGTCC---

------------------------GGTAGCCCTCGATATTTGGACGGCTACGGCAACGAG
AAA---ATGGATCTGTACGGAGAGACGGGT------TATGGT------------------
---------GCTTTGCGAGGCGGC---CCTATGGGCAAAGGAGGCTTG------------
---CTCGCCACGGCCAGTGACATGCGACCCATG---------------------------
---------GACGACTCATATGACTCCAATTATAGCAGTTCTGGCGTCAAGAGGTCATCT
GCCCTCCAGTCT--GGCTTG
AGTGCCTCCGACTATCATCATCGTCAGCAACAGGAAGTCAATCATTTGAGC---------
------CGAGATCGTGATTTCCCGCCTTCGCGCGGTGGAGTAGGAGTGGGGGGGCAGTG
TATCTGGCCCT---------------------GGGCACACGCGATTACGA------GGGA
CA---ACCGACTACTATCCCTCACCCAGTCAG---CAGTAC-------------------

--------------AATGCCACTTAT----------------------------------
--TCACAACAAAATGGGTGCTCA------------------------------------A
TTACCAACAACCCAGTGCCCAATTACCATCACTCTCCCAGTGGAGCAGTGACTGCCGTAG
GCGCG---------TCTGTCCCAGCGGCTCCTGTGGGCACGGGAGCAAATTAC-
>GCHA01024795_Tigriopus_japonicus_Bib
ATGGCT--
---------------------------------------GCCAGGTTCGATTCG------
CTACATGCCCAATTGGGGTGGCTTCTCGAAAATGCTCAATCGCTTCTGGCCGATTCA---
GCCCCGCCCTCA---------ACCACGGGGGGAGGTAACCCTTACTATGCAGGTGGGAGT
GGTCGA---GGAGCCCATGGTTCCACGATTGGC---------------CAAGCTCCTCCC
CCCGTGTACTCTTTCGTGGCCATGCAAAGTGAGATTCGCACCCTG---GAATTCTGGCGG
TCCATCATTGCGGAGTGCATGGCGACGTTTGTCTATGTCATGATTGTTCTGTCCGTGTCC
ACCACTTCTTCCGAGCATACT---------------------------------------
---CAA
CAAACGAATTGTGCCCTGGCTTCAGGCCTTGCCATGGCCATGTTGGTAGCCGTTTTTGCT
CGAGTGTCAGGAAGTCACACCAACCCGGCCATAACTCTGGGGATGACCCTAACTCGATAC
ATATCTCCCTTGAGGTCGGTCCTCTACATGTGTGCCCAATGCGGGGGTGGCATTGCAGGG
GCAGCCTTGACTTTGGGCGTT------------CAC---GGA------------------
------------------------------CGGATAAAT------GATCCAGCTCTC---
---------AAGTCGGAACGAGGCACGTTCGGCATGGAATTTGTTCTTTCGTTTTTGGTT
GTGTACACATATTTTGCT---GCCAAGGAAACTCGAGTG---TCTTCTTCGTATCGCACA
CGAACTCTCGTACCT---GGCCACATTCGCATCAACCCTATTTTTGTCGGAGTCGCTCAT
---GCTGCCTGTTTAGTGGCTTGGAAA------GGA---TCTCTGAACCCTGCCAGAGCC
CTTGGGTCCGCCTTTGTCAGCCGCAGTCCTGATCGGTTCGATCAGCATTGGGTATTTTGG
GTGGGTCCTCTCTTGGGAGCAATCACTGGTGCCTTTGCTTTCGAGTACATCTTCAACCCG
---AGGCGAAAAGTGTTCACATTTTCAAGTTTAAGAGGCCCTCAT---GACCTT------
------------------------GAA------------AACGTGTCAATTCGCTCCGAG
GAC------GAC---ATGATAGAC---GATTTA---GAGCGCGCCAAGCAATATCGCGCT

---AACATC---
------ATGCAAGACTTC---CAA------------------GATCCC------------
------------ACACATCCTTCCTCAGCTCGA------------------GGGGGT---
---------CCT---------------------------------------TACGGC---
---------------AAA---AGATTAGGGGCCGCAGGAGGGATGAATCATATCAAC---
GCTAGCGACTCG---------------------------GTGTATGGGGGGTCCAAGTCC
GTG---TACAACCAA---------------------TCG---ATCTACGATTCCAAG---
------TCAATGTAC---GAT---GGAACCAAATCCATGTATGATGGATATGAT---GGG
------ATG---------GATCGGAATGCAGCCAATCTGAGACGTTCCCGTTCAATTCAC
ACCAAGTTCCCGCCCCCCAACCAC------------------------------------

------------------------TCTGTGACT---------------CGACGAAACCCA
TATGACTAT---CTACCCGATGAGCCC---------------------------------
AGTAAG---GCGGACATGATGCAAACG---------------------------------

------------GAGAGT------------GGGCCC---CGCCTCATGGGAAAGTCT---

------------------------GGTAGTCCTCGCTATCTGGACAGTTACGGTAACGAG
AAA---ATGGATCTCTACGGAGAGACAGGGGGC---TATGGT------------------
---------GCTTTGCGTGGCGGA---CCAATGGGCAAAGGTGGTTTG------------
---CTCGCTACCACGAGTGACATGCGACCCATG---------------------------
---------GACGACTCCTATGACTCCAATTATAGTAGTTCTGGCGTCAAAAGGTCATCT
GCTCTCCAGTCT--GGCTTG
AGTGCCTCCGACTATCATCATCGTCAGCAACAGGAAGTCAATCATTTGAAC---------
------CGAGAACGTGATTTCCCGCCTTCTCGT-----------------GGTGGCAGCG
CCTCTGGCCCT---------------------GGGCACACGCGATTACGA------GGGA
CA---ACGGACTACTATCCCTCACCGAGTCAG---CAGTAC-------------------

--------------AATGCCACTTAT----------------------------------
--TCACAACAAAATGGGTGCTCA------------------------------------A
TTACCAATAACCCAGTGCCCAATTACCATCACTCTCCCAGTGGAGCGGTGACTGCCGTGG
GCACG---------TCCGTCCCACCGGCTCCTGTGGGCACGGGAGCAAATTAC-
>JABCAI010000001_Tigriopus_kingsejongensis_Bib
ATGGCC--
---------------------------------------TCCAGACTCGAGTCC------
TTGCACGCACAACTCGGTTGGTTGCTGGAGAATGCTCAGACATTGCTCGCAGATTCG---
GCACCTTCCTCC---------ACCACC---GGGGGCAATCCCTACTTT---GGCGGTTCC
ACGCGGGCGGGACATCATGGATCCGCCACGGGGGCC------GCTGTTCAGGCC---CCA
CCCGTCTACTCGTTCACGGCCATGCAGGCCGAGATCCGAACCCTG---GAGTTCTGGCGC

```
TCCATCATTGCCGAGTGCATGGCCACCTTCTTCTACGTGGTCATCGTCCTCTCCGTGACG
ACCTCGTCCTCGGAGCACACG---------------------------------------
---------------------------------------------------------CTT
CAGACCAATAGTGCATTTGCCGCAGGTCTGGCCATGGCCATGCTCACTGCGGTCTTCTCG
CGGGTGTCTGGTGCCCACATCAATCCGGCGGTCACCCTAGGCATGACTCTCACCCGCTAC
GTGTCCCCTCTGCGGGCCATCCTCTACATATGCGCCCAATGCGGCGGCGGCATTGCCGGA
GCCGCTCTCATCCTGGGGGTG------------CAC---GGG------------------
------------------------------CGAATCAAC------GACGTGGCCCTC---
---------ACCACGGAGCGAGGCACATTCGGCATGGAGTTTGTGCTCTCCTTCCTCGTG
GTGTACGCTTACTTTGCA---GCCAAGGAGACGCGGATC------AGCACCTACCGGCTG
AGACCCTTGGTCCCC---GGCAATATCCGCATCAACCCGGTGATCGTGGGCGTGTCTTAC
---GCCGCCTGCCTGGTGGGCTGGAAG------GCC---TCCCTCAATCCGGCCCGAGCA
CTCGGCTCGGCCTTCGTGAGTCGCGGGGCGGACCGCTTCAATCAACACTGGATCTTCTGG
GTGGGTCCCCTGCTCGGATCCATCACCGGCGCCTTCGCCTTTGAGTACATTTTCAATCCC
---CGTCGAAAGAGCTTCACGTTGGCCGGTCTGCGAGGTCAGCAC---GACCTT------
------------------------GAA------------AATGTGTCAGTTCGATCGGAG
GAC------GAC---ATGATTGAC---GATCTG---GAGCGTGCCAAGCAGTACCGGGCC
------------------------------------------------------------
---AACATA---------------------------------------------------
------ATGCAAGACTTC---CAA------------------GATCCC------------
------------ATGCACCCAAGTGGAACACGC------------------GGAGCC---
---------------------------------------------------TACGGG---
---------------AAA---CGAGGCGGGGCCATG---GGAATGAACCCAATGAGC---
GGCACCGAGTCG---------------------------GTCTACGGGGGCTCCAAGTCC
ATG---TACAATCAG---------------------TCC---ATGTACGACTCAAAG---
------TCCATGTAC---GAC---GGCTCCAAATCGGTCTACGACGGCTATGAG---GGT
------GCA---------GATCGCCAAGGCAACAACCTCCGCCGCAGTCGATCCATCCAC
ACCAAGTTCCCGCCGCCAAACCAC------------------------------------
------------------------------------------------------------
------------------------------------------------------------
------------------------------------------------------------
------------------------CCGATGAGT---------------CGGCGCAATCCC
TACGACTAC---TTGCCCGACGAGCCA---------------------------------
AGCAAA---GCCGACATGATGCAAACC---------------------------------
------------------------------------------------------------
------------------------------------------------------------
------------GAGGCG------------GGCCCC---CGGCTCATGGGCAAGTCA---
------------------------------------------------------------
------------------------------------------------------------
------------------------------------------------------------
------------------------------------------------------------
------------------------GCCAGTCCGCGGTACCTGGACAACTACCCACCCGAC
AAG---ATGGACATTTACGGCGAGTCGGCC------TATGGG------------------
---------CCCCTGCGGGGG------CCC------AAGGGCGGTCTC------------
---CTCTCCACCGCCAACGACCTCCGGCCCATG---------------------------
---------GACGACTCGTACGAGTCGAATTACAGCACTTCGGGCGCCAAGAGGGCGGCG
GCTCTCCAGTCG------------------------------GGCGTCGGCTCGGGCCTG
ACTGGCCCCGACTATCACCATCGG---CAACAGGAAGTCAATCATTTGAGC---------
```

------------CGAGATTTCCCG------CGT--------------------GGA---G
CATCTGGCCCTGGT------------------GGGCACACGCGTCTCCGTGGAGCGGGCA
CC---GCGGACTACTACCCTTCCCCCAGTCAG---CAGTAC-------------------

--------------AATGCCAGCTAT----------------------------------
--TCTCAACAGAATGGATGTTCA------------------------------------A
TTACAAGCAACACGGTGCCCAATTACCACCACTCGCCCAGTGGAGCCACCGGGGTCGTGG
GTAGC---------TCGTCCACTGCCGCGCCAGTGGAGCAGGAGCCAACTAC-
>GHXK01163663_Platychelipus_littoralis_Bib
ATGACCAGTTCAACT---
---------------------------------------TCTCGCCTTGATTCG------
TTGCATGCTCAACTGAGCACTCTTCTGGAAAATGCCCAGTACCTTCTGTCAGACTCA---
ACCCTG---TAC------------------GGTGGAAATGTTCCACTC---GGCAGTTCC
ACGAGGTCAAACAAACAGGGAAAT---CTTCCC---------------CTGGCC---CCG
CCTGTGTATTCCTTCTTACACATGCAAGCGGAAATAAGAACCTTG---GAATTCTGGAGA
TCAATCATTGCAGAGTGTATTGCCACATTCGTGTACGTCATGATTGTCGTGTCTGTGGCC
ACCTCATCAACTGAGCACACT---------------------------------------
---TTA
CAGACCAATTCAGCCTTATCAGCAGGTCTTGCCATGGGAATGCTTTCTGCTTGCTTTGGA
CAGGTGTCCGGAGCCCATGCCAACCCTGCCATAACCATTGGCATGATGATTGCAAAACAT
ATCTCGCCATTAAGAACCGTGTTATACATCTGTGCCCAATGTGGTGGTGGCATTGCTGGA
GCTGCCCTGGTCTTGGGAGTC------------CAG---GGT------------------
------------------------------CGCATAAAC------GACCCAGCCCTC---
---------ATATCAGCGCGGGGGACCTTTGGAATGGAGTTCGTCTTGACTTTTCTTGTC
ATGTACGCTTTCCTGGCA---TCAAGGCAATCACCCCCTCCCTCCAGCTCTTACAAATCA
CCGCGTCGAACACCT---GTTCCAATCAGGATAGATGTGTTTGCCGTGGGCATTGCTTAT
---GCTGCCTGTCTCATTTGCTGGAAG------GGC---TCCTTGAACCCAGCCAGGGCA
CTAGGGTCAGCATTTGTCAGCAAGAGCACGTCACGATTTCAGCAGCACTGGATGTACTGG
GTGGGACCGATCCTAGGAGCTGTAACTGGGGCTTTCACACATGAGTACATATTCCACACA
---AGAAGGAAACCA------------GGACAGAGGAATCCAAAT---GATCCATTG---
------------------------GAT------------CAGATGTCCATGCACAGTGAA
GAC------------ATGATTGAT---GATTTG---GAGAGAGCAAAACAGTACAAAGCA

---AACATA---
------ATACAGGATTTT---CAC------------------GATCCA------------
------------TCGCTACCACCAGGACAGACG------------------GCA------
---------GGT---------------------------GCTATTTCAACCTACAACAGG
TCATAC---AGCAATAAG---CGT---------------------AAC---ACCAGC---
CATCTGGAGCCA---------------------------GTCTATGGTGGGACAAAATCC
ATG---TACAACCAGTCA------------------ACTGGGATGTACGAT---AGG---
------GATAGGTACCCAGAT---GGAACCAAATCCATGTACAGTGGTTATGAC------
------------------------AATAATTCCAACTTGAGACGATCCCGATCAATTCAT
GCCAAATTCACCCCAGGTGGCAGT------------------------------------
---------------ACTACAACT------------------------------------

---ACTGGA---

------------------------GGCATGAGC---------------AGACGTAACCCC
TACGACTAC---TTGCCAGATGAACCA------------------------GGCTCTATT
ACCAAA---CCAGACATCATGCAATCAAGCATGGTC------------------------

---ACATCCATGAAC---------------GGAACGAGCAGGATCATGCCAGGTTCA---

------AAATCGGCCAATCCG------AGTCCCCGTTACTCTGACAACTATGCAAATGAG
AAGCTTTCTGATACCTACGGAGAA------------TATGGG------------------
---------ACTAACCGAATC---------------AAGGATCCCGATTATGGAACA---
---GCCCGTCTGAAGGAAGACTATGAAAATGCC---------------------------
------AACTATGGC------AAGAGAAATTACACTTCTTCAGGTGGGGTTTCTCAAAAC
TACAGACAAGAT------------------------------GTAATGGGTCGAAATGTC
GGTGGTTCACAACAA------------ACCCAAGATTTCCAACGTATGTCA---------

---CGCATGCGC---------A
GT------GAATTCGAT---TCGCCG---------CAGTAC-------------------

--------------AACGCCTCCTAC----------------------------------
--TCACAGCAGAACGGCTGTTCA------------------------------------A
TCGCTGCCAACACAAGTGTGAATCCGGGTGGCGTCACGAGTGGAGGCGTTGGTGGCGTCA
CGAGTCAAAATCCTAGTAATACATATGGAAATGCTGCGACAGCCTCAAATTAT-
>KR005658_Lepeophtheirus_salmonis_Bib
ATGGATACAGGGACTTATGGAACGGGGGGTGGCTATGGGGGAACAACGACGACGGGAACA
CATTCA---------ACCCAACCCTCACATGTTCATCATCAACATATGGAC---------
TTCCAAGGACAACTGGGTATGTTCCTTGAGCAA---GGGACTTCTCTTTACGAGGCCCTC
ACAGGTTCTTATAACTCCTATACGACTCAAGGAGGCTACTATGACGTCCAAGGAAGACCT
CGACCCTATTATAGCTCTTCCACTACGACGTTGTCCTCTACAACTAGAGCCAAAAGACCA
CCGATTTATGGGTTTGTAGCGATGCAAGCAGAGATCCGAACACTG---GAATTTTGGAGG
AGTGTCATCGCTGAGTCTGTTGCAACTTTTTTTTATACAGCTCTTATCTGTTCCGTCAAT
ACATCCTCCACATTACATACC---------------------------------------
---ATT
CAGACTAATGTTGCCATGGCTTCTGGTTTTAGCATTGCTGTTCTCACTGCGTGTTTTGCC
TCCATTTCTGACTGTCATGCCAACCCATCCATGACCATTGCTCGCACATTTACTCGCCAC
ATCTCTCCTCTAAGGTCCTTACTTTATATCTGTGCTCAGTGTGGGGGAGCTATTGCAGGT
GCTGCATTAGTTTATGGTGTG------------TAC---TCT------------------
------------------------------CGGATCCAA------GATCCTATTCTTGAA
---------ATGTCA---GGAGGTGCCTTTGGCATGGAGTTTGTTCTCTCCTTCTTTATT
GCTTATGCTTTTTATGCT---TCTCGTGCTGGACATAAG---------------------
------------------------------GCAGATCCTGTACTCATTGGCTTGGCATAT
---GCAGGTGCACTTGTGGCTTGGAAA------GGA---AGTCTCAATCCTGCTCGTGCC
CTGGGATCTGTATTTGTTTCTAAAGAGGAGGGCCGTTTCGCAAAGCATTGGATATTTTGG
ATTGGTCCTTTCCTGGGTGCACTCACAGGTGCCTTTACTTTCGAGTACATTTTTAATCCT

```
---CGTAGAAAGAAAACCACGACTACTGTCATAACCAACAAAATTGATGACCTTCAC---
------CCAACAACCACA---ACTCAT------------CATCCAGCAACTTTTACTGAG
GAA------GAC---ATGATTGAC---GATTTG---GAAAGAGCCAAGCAGTATAAGGCC
------------------------------------------------------------
---ACAAGT---------------------------------------------------
------ATGATTGACTAC---GGA------------------GAT---------------
------------------------------------------------------------
------------------------------------------TCATCTGCATATAATCGC
TCTGTC------TATAAA---CGC---------------------AATCCTCCT------
TATCCAGAGTCG---------------------------ATGTACAGTGGCTCAAAAAGC
TTA---TATAATCAAACA---------------GGGACTTCTGGCTATGATAGTAAA---
------GGAGTGTATGGAGAACTTGGGAGCAAATCATGCTATGAAGGCTACGAGAGTGGT
---GTCATA---------GATCGAGATATTTCTAGTTTAAGAAGAGGAACCAAT------
---CGAGGCATGGGCATGTCCTCC------------------------------------
ACAGCTGTCAATGGGAACAATAAC------------------------------------
------------------------------------------------------------
---------------------AAT------AATCGAAATTGTCAACA---ACAGGA---
------------------------GGAGTCTCAAGCGGAGCA------CGGAGGAATCCT
TATGACTAT---CTCCCGGAAGAGCCT------------------------CCAGGATCT
AATAAA---CCGGATATTATTTCGGGTAGGAAAATA------------------------
------------TCAAATCGCAATTATTCACCCACTAAATAC------------------
------------GAA---GATAAT---TCCTTGAGAATCAATGATAATTTTGATTTGGAC
ACTACTAATGCAAACCCCCCATATCGAAGTGGAACCACT---------------------
------------------------------------------------------------
------------------------------------------------------------
------------------------------------------------------------
------------------------------------------------------------
------------------CCT---GGAACTGGAAAA------------------------
---ACTAACGATATATAT------------------------------------------
------------------------------------------------------------
------------------------------------------------------------
------AATGAT---------TCAGCCAATTACAACGAT---CCCCTTAAACGCCAG---
------------------------------------------GCTCCTCCTGCA------
------------------------------GCA------CCAGTCATG------------
------------GTGGACAATTCC------------------------------------
--AAT---------------------------TATGGGACTCGA---------------A
CCGCAGGAGACTATCAAAGAATGGGGAGTAGTCGCCATCGAGGGGAC-------------
------------------------------------------------------------
-----TTTTACTCTCCTTCGTAT-------------------------------------
------------------------------------------------------------
--------------AATGCGTCATAC----------------------------------
--TCTCAACAAAACGGCTGCTCC------------------------------------A
TCTCTCAA---ACAGCTTATAAT-------------------------------------
-----------------------------------------------AACTAT-
>GAZX01030744_Caligus_rogercresseyi_Bib
ATGGATAGTGGTACGTATGGGACC----------TATGGGGTACCACGACCACGGGGACT
GCGTCCCACTTGCAGCAAAGCGCCTCTTCCGTGCATCACCAACAAATGGAC---------
TTTCAGGGGCATCTTGGGGTCTTCCTTGAGCAG---GGGACGTCCCTTTATGAAGCCCTT
```

AGTGGTTCTTTTAGCTCCTATACGACCACAGGAGGATACTATGACGTCCGGGGTCGTCCC
CGGCCATATTATTCTTCTACGACAACAACTGCAAGCTCC------------AAGCGACCC
CCCATTTACGGCTTTGTAGCCATGCAGGCCGAAATACGGACTTTG---GAGTTTTGGCGC
AGCGTAATAGCCGAGTGTGTGGCAACCTTTTTTTACACGGCGCTTGTTTGCTCTGTGAAC
ACTTCCTCCACGCTGCACACC---------------------------------------
---ATT
CAGACCAACGCGGCCATGGCATCTGGCTTCGGCATGGCGGTTCTCACTGCCTGTTTTGCC
TCTGTCTCCGATTGTCACGCCAACCCTTCCATGACCTTTGCTCGAACCTTCACGCGCCAC
ATCTCCCCTCTGCGGAGCCTCCTATATATTTGTGCCCAATGTGGTGGGTCCATTGCAGGG
GCGGCTCTTGTATACGGCGTA------------TAC---TCC------------------
------------------------------CGCATACAG------GACCCTGTGCTTGAA
---------ATGTCT---GGTGGGGCATTTGGCATGGAATTTGTTCTCTCCTTTTTCATT
GCATATGCCTTTTATGCC---TCACGCTCAGGCTACAAG---------------------
------------------------------GCAGACCCTGTACTCATTGGCCTAGCCTAT
---GCCGGTGCCTTGGTCGCATGGAAA------GGC---TCCCTCAATCCTGCACGCGCA
CTCGGCTCTGTTTTTGTCTCCAAGGAAGAAGGCCGCTTTGCCAAGCATTGGATATTTTGG
CTGGGACCCTTCCTGGGTGCACTTGCGGGCGGATTCACATTTGAATACATCTTTAGCCCA
---CGAAAACGACGCAATAATACTACTGTCATCAGCAATAAGATCGACGAACTTCAC---
------CCCACATCCTCTGGCACACAT------------CATCCC------TTCACAGAG
GAA------GAG---TTGATCGAT---GATCTC---GAGCGTGCAAAGTCTTATAAG---

---TCCAGC---
------ATGGTGGAATATGGAGGG------------------GACGGC------------

---TACAACCAA
CGC---------TATAAG---CGG---------------------AACCCTCCA------
TACCCAGAGTCC---------------------------ATGTACAGCGGCTCCAAGAGT
CTC---TATAACGCAACC---TAC---------GATGCCACCGGC------ACTAAGAGC
GGGGGAGGAATATATGGAGAACTGGGAAACAAGTCCTGCTATGAAGGATACGATAGCGGG
GCGGTCATT---------GATCGCGACATGTCTAGTCTACGAAGAGGTACTTCC------
TCACGCGTCCTT---ACCAATGGA------------------------------------
GGAAACACCAACGCCAATAATACG------------------------------------

------------------------------AATCGCAATATATCCAGTGCAAGCGGA---
------------------------GGAGGAACC---------------CGACGGAATCCC
TATGACTAT---CTACCTGAGGAACCG------------------------GGCAATAGC
AATAAA---CCTGATATTATCTCCGGGCGAAAAATA------------------------
------------TCCAATCGGAATTACTCCCCCACGAAATAC------------------
------------GGCGCAGGAGGA---GAGGATCGCCTCAATGATAAC------TTGGAC
TCAAATAACGCTAATACCGTGGTACGCAGTGGATCA------------------------

---------------------------GCCTCAAAG------------------------
---GCCAGTGACATTTAT--

------AATGATCCA------GCCGCCAACTACAACGAT---CCCCTTAAGCGCCCA---
---GGTCCTACTTCA---
---------------------------TCCGCATTGCAGCCC------------------
------------ATTGATAACTCC------------------------------------
--TCCGGAGGAGGC------------------TATGGGGTTCGT---------------A
AT---GGAGACTATGGACGGAGTCCCGGCACTGCAGCCCCTGGCGAC-------------

-----TTCTACTCCCCTGCCTAC-------------------------------------

--------------AACGCATCCTAC----------------------------------
--TCACAGCAAAACGGATGCTCC------------------------------------A
TCTCACAAAGCACGCCATATACC-------------------------------------
---AACTAC-
>GGQW01006062_Tracheliastes_polycolpus_Bib
ATG------GAGACC---
---------------------------------TCTGCTCGCACCCTTCTCACTCAAATC
AAAGAGCTC---CTTAACTCTGTTCCAGAGCTCTCAGGG------CTCAAAGTCTCCACA

---GCTCCTCTT
CCCATCTATGGCTTCGTTTCCATGCAAGCCGAAATTCGAACTCTG---GAATTTTGGCGT
TCCATTTTCGCAGAGTGCCTCTCGACTTTTTTCTACGTTCTCATCATTTGCTCTGTAAAT
ACCTCTTCATCCTCTAGTGTA---------------------------------------
---CTT
CAAACAAATGTTGGACTTGCTGCTGGACTCTCCATCTCCGTTCTTACCCTCTCTTTTTCC
TCTGTCTCTCACTGTCACAGCAATCCTGCCCTTAGTATTGCTTCCCTACTAACGAAAGAC
CTGAGTCCACTTCGAGCAATTCTTTACATTGCAGCTCAGTGTGGAGGTGGAATTGCTGGA
GCGGCACTCGTTCTTGCTGTC------------TAC---TCT------------------
------------------------------CGAATTTCG------GATCCACTCTTTGAG
ACGGGT---GGATCC---GGTGGAGCCTTTGCCATGGAATTCGTTTTGACGACCCTACTC
TCCTATGCCTTTTATGCC---TCCAAAAAAAACTCGAGT---------------------
---------------------------TTGAGTCATGGACTCTCAATGGGCCTCGTCAAC
---GGCTCATGTCTTCTCTCATGGAGA------GGA---AGTTTGAATCCAGCTCGAGCT
CTTGGCTCTGCCTTCGTCTCGAAAGAGTCTCGTCGTTTTGCTCGTCATTGGATCTACTGG
ATTGCTCCTCTCCTTGGAGCCATCACGGGAGCCTTTTCCTTTACTTATATTTTTAATCCG
---AGAAAAAAATCCGGTGGGTCCACTCATAGTCAAGTCAAAAATAATCCTTTG------
------GCCTCCTCTTCCTTCCTGAGT------------GTAGAGGACGTACATAATGAT
GAG------------------------GATGTA---GAGAGAGGTAACAAATAC------

------ATTCAGCAG---GGTCCC------------------------------------

---------------------------------------TCAGAGTCCTTTTCTGAGTCA
AGAGCTAAT---TATCGA---AAG---------------GGACGAAAT------GAC---
GGAGTCGAGTCT---------------------------CTCTAC------TCCAAAAGC
CTG---CATAATGCTCCATTGTAT------------GCCCATGAAAAGGGCTCTCGC---
---------TTCTACGGAGAC------TCAAAGAGCCTGTATTCAGGGATTGAGGAAGAG

AGTCGAGGCTCTAGTAATTCTCGG------------------------------------

---------------------------CCCACC---------------AATCGAAATCCC
TACGACTAC---CTTCCTGATGAGCCG---------------------------------
AATAAA---CCGGATATTATT------CGTAAA---------------------------

---GGTAATCCC---TAC--

---AGT
TCCTCAAACTATGAAACGTCATCAAAC------TTGGAG---AATCAA------------

---------------------------TCCTCTGAC---AATCTCAAGTCA---------
------------CGAGAATTTGGG------CGC--------------------GGA----
--AGTGAGTTTGGACGC---------CTT---GGACGCCTTCGC---------------T
CC------GAATTCTAT---TCTCCATCCTCC------TAC-------------------

--------------AATGCCTCCTAC----------------------------------
--TCACAAACAAACGGATGCTCC-------------------------------------A
TTAATTCCTCATCTCCCTACAACACC----------------------------------
--AATTAC-
>GINW01258431_Temora_longicornis_Bib
ATGGCC---CCCACC---
---------------------------------AAGCAAAGCGAGCAGATCATCGCTCTG
CTCGAGAGT---GCACAGAGCCTTCTCGAGGAT---------------AAGGAGGAGAAG

GGGAAGGCGGGAAAACCACCCATGCAAATTGAAATAAGGACACTG---GAATTTTGGCGA
GCAATTATCGGAGAGTGCATCGCCACCTTCTTTTACGTGATGTTGCTCTCTTCAGTACAC
CAGGCACTGAACCATCCGAAGGAC------------------------------------
---AGTATCGCGGTGGTC
CAGATGTTCAGTGCGCTCACGGCCGGCCTTGCAATGATCACCCTCACCCACGCTTTCTTT
CAGGTGTCCGGCTCTCATTTCAACCCCGCCATAACCATCGCCTCAGCGCTCATGAAGAAA
ATCTCAATTTTGCGCGCCGCTGCGTACATTTGTGCGCAGTGTGGCGGTGCCATTGCCGGC
GCGGCGCTCGTGTACGGCGTG------------TAC---GGC------------------
------------------------------CGCAACGGCGCCAAGGACCAGTTCGGGCCG
---------GCCTCA---ATCTCAAACTTTGGGATGGAGTTCATCTTGAGTTTCCTCGTC
TCCTATGTGTATTTCAGC---GCTGCTGCCTCCGCTAAA---------------------
---GCTAGAACG------TCGGATTCCCTATCGTCGAGCGTGAGCATCGGCTTGGCGTAC

```
---ATGACAGCGCTGTCGGCGTACCGG------GGC---AGCCTCAACCCGGCCCGGGCC
CTCGGCCCCGCCTTCGTGGCCGAC---------GCGTTCTCGTACCACTGGGTGTTCTGG
GTGGGGCCCATCCTGGGCGCAGCCTGTGGAGCCTTCTGCTACACATTCATATTCAACTTA
---AACAAACCCAGGTGGGTG------CGGGACAAGGAC---GTA---GAGAAC------
------------------------------------------GTGTCCATGAAGAGTGAA
GAT------GAT---ATGATCGAC---GACCTC---GAACGTGTCAGACAATACAAGTCC
------------------------------------------------------------
---TCCATG---------------------------------------------------
------ATGGCGACGTAT---AAT------------------GACAAC---CTGCAC---
------------ACC---------------------------------------------
---------------------------------------ACCACCTCCCTCTACAACACC
GCCGTCAACCCCTACAAG---------------------CCCCGC---CCC---------
GGCCCCGAGTCC---------------------------GTCTACGGCGGCACCAAGAGC
ATG---TACAACGGAGGTGGATACTCAGGCCGCCGGACCCCCGGGTTCGACAGCAAG---
---------------------------------TCCATGTACGGCGGGATGGACGAGGAC
GGGCGCGAGGCAGGCCGCCAGAGGTCCAGCTCCCAGCTCAAGCGTTCAATGTCCGTACAT
TCCAAGATGGCGCGTAGGAATCCG------CAG---GATATACTG---CCAGACGAACCG
GCCCGGTCAGCCCAGCTGTTA---------------------------------------
------------------------------------------------------------
---------------------------------AGACAGCAGCAGCAGCAGCAGCTGGCT
------------------------------------------------GAACATCAGCAG
ATGCCGCAGCATCCTAGACAGATTTCGCAG------------CTC---ACTGACAAGGTG
GAAAGG---GCTGAAGGG------------------------------------------
------------------------TATACGTCA---------------------------
------------------------------------------------------------
------------GAACAAATGTAC------------AGAGAA------------------
------------------------------------TATATCCGTCAGAAGAACGCAGCA
---GCTGACCAAGGTTCTCCGATCGATCAGCTGTCAGGCAACAGCTGTCAG---------
------------GAGCAGCTATCAGGCAGCAGCTCC------------------------
GGCCAGTGCTCCGGCAGCTCC---GGCTACTATTCCTCAGCCCGGGAG------------
---------------------------CAGCAG---------------------------
------GAGGAGCTGCGCCTGCGC------------------------------------
------GAAGAGCAGATGCGC---------------------------------------
---------GCAGAAGCGCAGCTTAAGTCG------GAGCAGCTCCACCGC------AGG
GACAGAAGCTTTGGGAGCAGCCGCGGGGAGCAGCTC---------AGA------------
------------------------------------------------------------
------------------------------------------------------------
------------------------------------------------------------
--------------------------------TTGGGAGCTAGG---------------C
CG---GCAAACTAT---------CCATCTAGCAAC---TCT-------------------
------------------------------------------------------------
------------------------------------------------------------
------------------------------------------------------------
--------------------TCTTAC----------------------------------
------------------------------------------------------------
------------------------------------------------------------
-----------------------------------------GGA---GGCTAT-
>GFWO01050173_Labidocera_madurae_Bib
```

ATGATG---GACCAG---
---------------------------------AGATCTACCGACCAAGTGATTGAACTG
CTGGAGAGT---GCACACAGTTTACTGGATGTG---GGG---------AAGAAGGGGACA

---AACAAGGGAAAACTTCCCATGCAGATAGAAATCAGGACACTG---GAATTTTGGCGC
GCAATTATTGCAGAATGCATCGCAACATTCTTTTATGTGATGCTACTGTCTTCCACCCAC
CAAGCACTAAACCACCCTAAGGAT------------------------------------
---AGTATCGCTACAGTA
CAGATGTTCTCGGCGATAACGGCGGGATTGGCGATGGTCACACTCACTCAGGCCTTTCTG
CAGGTGTCAGGATCGCACTTCAATCCATCAATCACTGTTGCTTGTGTTCTCATGAAGAAG
ATCTCTGTGTTGCGCGCAGCCATGTACATCTGCGCCCAGTGCGGCGGTGCTATTGCCGGC
GCTGCCCTCGTCTACGGCGTG------------TAC---GGT------------------
------------------------------CGCAACGGGGCCAAGGACCAGTTCGGTCAG
---------GCGTCC---ATCGCCAACTTCGGGATGGAGTTCATCCTGACGTTCCTGGTG
GCCTACGTGTTCTTCAGC---GTGTCGGCCAACCTCAAC---------------------
------AAGTTG------AACAACAATCTTCAGTCCAGCTTGAGCATCGGGCTGGCCTAC
---ATGGCGTCGCTGGCCGCCTACCGG------GGC---TCCCTCAACCCGGCGCGGGCA
CTCGGCCCTGCCTTCGTCGCCGAC---------TCGTTCGACTACCACTGGGTGTTCTGG
GTCGGTCCGATCCTCGGAGCTTCCTGCGGCGGGTTCTGTTACACCTTCATCTTCAACATG
---AAGAAGCCCCGTTGGCGC-------------CGCGAGAAA------GAGGGC------
------CCG---------------GAA------------ACGATGTCGGTCAAAAGCGAC
GAC------GAC---ATGCTGGAC---GACCTG---GAGCGAGTGAGGCAGTACAAGACC

---AACATG---
------ATGGCAACGTAC---AAC------------------GAGGGT---CTGAAC---
---GGCGGC---
---------GGTGTG---------------------------GGCTCTGTCTACAACACG
GCCATCAAGCCGTACAAG------------------------CGG---GGCGCG------
GGTCCAGAGTCT---------------------------GTGTACGGCGGAACCAAGAGC
TTG---TTTAACGTGCCGCCGTACGAGGGTCGACGCACGCCAGGATTCGACAGCAAG---
---------------------------------TCCATGTACGGGGGAATCGACGACGGT
------------------CGACAGTCTAGAGCGCAGTTGAAGAGATCCATGTCGGTTCAC
TCTCGGGTACAGAGGAGACATCCC------CAG---GACAACCTG---CCTGACGAACCA
GCGGCTTAC---

---------------------------------CGGCAA---------------------
------------------------CCGCCTAAC------------------CGACAGACT
GCCGTCGAACATTCGCGTCAGGTT---GCC---------------GACAGCGGAGCTAAT
GATAGA---CTGGAGTCC--
------------------ACGGCTTATACATCT---------------------------

------------GATGCAATGTAC------------AGGGAA------------------
------------------------------------TATATTCGACAGAAAAATGTAGCA
---GCTGATCAAACGGCTCCCTTGGAGCAATATTCG------------------------
------------------------GGAAGTAGCTCC------------------------
GGCCAGTGTTCCGGCAGCAGCTCCGGATACTATTCGACTGCTCGGGAG------------
---------------------------CAG------------------------------

------GAGGAG---CAC---CGGAGG------------------------GAG------
CAGCTCCGCGCCGACCACGAG---------------------------------------
---------CTTATCCGGGAGCAGAAGCAA------GAGCATTTCCATCGT------CGC
GAGCGCAGCGTTGCCAGCACGCGTAGCGAC---GCG---------CGC------------

---------------------------------------CGCATAGCA------------
------GACGGTCATCATCACCCGGCG---------------------------------
-----CCGGCT------------------------GGTCAGCGT---------------G
CA---CAGAACTACACG------CCCAGCACCACC---TCC-------------------

-----------------------------TCATAC------------GGATAC-
>XP_023326243_Eurytemora_carolleeae_Bib
ATGGCGATGGAA--
---------------------------------AAACAGAACGATGAAATTCTAGCATTG
TTGGAAAGT---GCCCAAGTTCTGCTGGAACAG---GGA---------AGAGATAATATG

---AAAAAAGGAAAACTACCAATGCAAATAGAAATAAGAACCCTT---GAGTTTTGGCGG
GCAATAATTGGCGAGTGTATTGCCACATTTTTCTATGTTATGCTAATAAGTTCAACACAT
CAGGCGTTGAATTTTCCAAATGAT------------------------------------
---AGTATTGCAACGGTT
CAATTCTTCTCTGCTATTACCGCTGGTTTAGCCATGCTAACCCTTACTCATGCCTTTTAC
AAGATCTCTGGTTCACACCTTAACCCTGCAATAAGTGTAGCCGCAGCGCTTCTGAAGCGT
ATAACTGTGCTCCGTGCGGCACTTTATATTTGTGCGCAGTGCGGCGGTGCCATTGCTGGT
GCCGCGCTCATTTACGGCGTG------------TAC---GGC------------------
------------------------------AAAAATGGCGCCAAGGACCAGTTTAGAGAT
---------ACATCA---ATCGCTAACTTTGGAATGGAGTTCATCATGACCTTCCTTGTG
GCCTTTGTTTACTTTGGT---GTGACCTCCAAGAAGAGG---------------------
------------------TCGGATACCTGGACCTCTAGCGTCAGTATAGGGATGGCGTAC
---ATGACCTCGTTGGTTGCGTACCGT------GGC---TCATTAAATCCTGCACGGGCT
CTTGGTCCCGCATTTGTTGCTGAC---------GCATTTGCATTCCACTGGGTTTACTGG
GTCGGACCCATTCTAGGGGCAGCTTGTGGAGGATTCTGCTATACATTCATATTCAATATT
---CATAAGACCAAGTGGAAG---------AAGAAGGAC---GTA---------------
------------------------GAT------------AACATATCAATGAAGAGCGAC
GAT------GAC---ATGCTCGAC---GATTTG---GAGCGTGTGCGTCAATTTAAAGCA

---AGTATG---
------ATGACCAACTAT---AAC------------------GATGGA---CTTGCC---

---------GGTGTA---------------------------CCTTCAGTGTACAATACT
GCAGTGAAACCCTACAAA------------------------------CAGGCA------
GTACAAGAGTCT---------------------------ATGTACAGTGGTACAAAAAGT
TTG---TACAATGCACCAATGACTGACGGACGACGAACCCCTGGATTTGATTGC------

---------------------------AGTAAGTCTATGTATGGTGGTATTGACGAA---
---GTA---------------AAATCCAGGGTTCAGCTAAAGCGATCTCTCTCTGTACAC
TCAAAGTTACAGAGAAGAAATCCG------CAA---GATAATCTA---CCCGACGAGCCA
GCAGCTCTCCGGGCAGCGCATACA------------------------------------

---------------------------------AGACAGCAGAGC------CATCTGGCA
------------------------GAGGGGCAG---------------CTACGCCAAACG
GTGGAAGGGCAGCTACGACAGGCG---GCA---------------GAG---GGGCAGATG
AGAGTT---GGAGATGGT--
------------------CCTGTTTTTGCCTCA---------------------------

------------GATGCTATGTAC------------AGAGAA------------------
------------------------------------TATATTCGACAGAAAAATATAGCA
---GCAGATCAGCACTCTCCGGTGGAT---------------------------------

---CAATGCTCTGGGAGCAGCTCCGGGTATTATAGCAGCTCCAGAGAG------------
---------------------------CAG------------------------------
------GAGGAGCTA------AGGAGA------------------------GAG------
CAG---GAGGAGCTACGAAGG---------------------------------------
------------GAGCAGGAACTTAAACAG------GAGTATTTCCACCGG------AGG
GAGCGGAGTGTAGGGAGCACACGAGGGGAG---TAT---------CGG------------

---------------------------AGGGAAGAATTGAGACAGGAGAGG---------
------GAGGGCAGATCTGCTCCA------------------------------------
-----CCTCTAGCAGAT------GATATA---AGAGGAGGGCGG---------------A
GT---CAAAACTACACG------CCTAGCACCACA---TCT-------------------

-----------------------------TCATTTCATTCAAAC---GGATAC-
>EFX82861_Daphnia_pulex_Bib
ATGGCGACAATGAGT---
------------------------------GTGGCCACGACTCCACTGACGAGTGCCGGT
CTCGAGAATCTGATGACGACATTGCTGGAACGTTTCGAGACGGCCTGTTTGGAGCAGCAG
CAG---
---GCGGGAAAT
GGCAACAAGATGCGGGTGCCAATGCAGGCCGAAATCCGATCGCTG---GATTTCTGGCGT
TCCATTGTGGCCGAATGCCTGGCTTCCTTTTTCCTCGTCTTCATCGTCTGCGGCTCGTTC
ATACCTTGGTCGGGACACACT---------------------------------------
--CCGCCA
GCCATCAGCATCGCCTTGGCTTCCGGCTTCGCTGTGGCAGCCCTCACACTCTCCTTCGCC
CAAATATCTGGAGCTCATTTGAATCCAGTCATGTCGCTGGCATTGCTCGTCACTCGCAAA
ATATCGCCCATGAAAGCTTTGCTCTACGTCACGTCTCAAGGAGGTGGAGCCATCGCTGGA
GCTGCTCTTCTTTACGGATGCACCAGTCCTGGACATCAAGGA------------------
------------------------------TACCTCGGAGCTACAATCGTCTCTACCGGT

---------TTAACCGATTGGCAAGCGCTGGGCATTGAACTATGCCTGACGTTTGTCATC
ACGCTCGTCTATTTGAAC---CAAAAAGATGATCATCGA---------------------
---CGTGGGTTATCG---GCTTCCATAGGACCTGGACCCATCACGCTCGGCCTCACCTAC
ACGGCCTGCTCCTTTGTTGCTTTGCCATCAACTGGGGCCTCATTCAACCCTATGAGATCA
CTTGGTCCGGCTTTCGTCATGAAC---------AAATGGGACGCTCACTGGGTTTATTGG
CTGGGACCCATCGTGGGTGGCTGTCTGGCGGCCATCCTCTACGAGTTCATTTTCAACCCG
---TATCGCAACTCGCAACGC---------CGTAAGGGATCCATCCCCGATGGT------
------------------------GAC------------GCGTCGAGCATTCACAGCGAC
GAG------GAC---AACTACGAC---GACGTG---AAATCAACTGTG------------
---GCTAATTAC
CAGAGTGCC---------CGC---------------------------------------
------TCTCAGCACTACGATCAG---------------TACCGGCCG---GCAATTAGT
CCAGGTGGTCCGTCTAATCCAATTTCTCCTAGC------GGTGGT------GGTGTCGGT
---------GGCGCT------------------------GTAGGAAACAACTGGCGGAAC
------CAGCAG---CGG---------------------------------ATGGAA---
CGAGCCGAATCG---------------------------GTTTATGGCGGCACCAAGTCG
CTT---TACCATTGCGGC---------------AAATCACCTGGACCGCCCAGCCGT---

---------------------------TCGAGCAATCTCAACCGCTCGCAGTCGGTCTAC
GCCAAA---CCTCCGGCCGCTCCGGCTGTCCAACCTCCCAACGTTTACGCTTCCCGCGGA
GCTCAGCTCCAGTCGGCGCAGAGC---ACTTATCTGGCC---------------------

------------------------------GCCCGCAACGTCGAC---------CTC---
--ATCCGCACCGAG
AGCATTTACGCCACGCGACAGCCGCCGGCT------CCT---CTGGCGTCCGGAAACAGC
AGCCGG---GCCGAA---
------------------AGCGTTTACGGCAGC---------CGCCATCCGCGCCATATG
GCCGATATGACGGACGTGGATCGA---CCCGTGCGCGACAATTCGCTCCTGACG---TCT
TCGGTGGCTAGAGAACCCGTTTAC------GCCACCAGGGCTCCCAGT------------
--CACGACCGTTCCAAA---

---CCGGAATCGTACCAACTGGAACCCATTTACCAGACTCGACGTGAAGTTGTACCATCG
GTGGCGGCCGTCGAGCCG------GCCCAGCGC---------------------------
---GGTGAAAGCCTCTACGATCGCAAAGTCCCTACTTATCGG------CCGGAAGCGACT
GTAGCCGCTTACAATCGTCGA------CCC------------AATCCGGAGCAG------
---------ATGGAAACATCGGCAACCAGCGCCGGAGGAGCTCCAATTTCTCCT---AGC
GACACGTCAAAGGATTCGGCCTATGGATCC---GTC---------CAC------------
--GGCACTTCGATT

---------------CAAAGTCCAGCGGAA------------------------------
-----TGGTCAGGTCACGGCCAGGAG------------AACAAT---------------C
AG---AGCAACTACAAT------CAACAACAGCAGCAGCAACAACAA-------------

---CAACAGC
ATGTTCAACATCTCCAACATGCATCCGCC------------TTCAGTCCTCATTTA---C
CCAACCAGCCTCCACAAGGTTCTTACCAGATC------------------------CAGC
AGCAGCAGCAGCAAGCCGGTCATCATCATCACTACGCTCAGAAGTTCCGAGCGGATTTGA

TGTCACCCAACAGCCAA---------CAACATTCGCCCGGA------------------
------------------------------TCATTTCACTCACCGGTGCAATAC-
>XP_046637937_Daphnia_pulicaria_Bib
ATGGCGACAATGAGT---
-------------------------------GTGGCCACGACTCCACTGACGAGTGCCGGT
CTCGAGAATCTGATGACGACATTGCTGGAACGTTTCGAGACGGCCTGTTTGGAGCAGCAG
GAG---
---GCGGGAAAT
GGCAACAAGATGCGGGTGCCAATGCAGGCCGAAATCCGATCGCTA---GATTTCTGGCGT
TCCATTGTGGCCGAATGCCTGGCTTCCTTTTTCCTCGTCTTCATCGTCTGCGGCTCGTTC
ATACCTTGGTCGGGACACACT---------------------------------------
--CCGCCA
GCCATCAGCATCGCATTGGCTTCCGGCTTCGCTGTGGCAGCCCTCACACTCTCCTTCGCC
CAAATATCTGGAGCTCATTTGAATCCAGTCATGTCGCTGGCATTGCTCGTCACTCGCAAA
ATATCGCCCATGAAAGCTTTGCTCTACGTCACGTCTCAAGGAGGTGGAGCCATCGCTGGA
GCTGCTCTTCTTTACGGTGTAACCAGTCCTGGACATCAAGGA------------------
-------------------------------TACCTCGGAGCTACAATCGTCTCTACCGGT
---------TTAACCGATTGGCAAGCGCTGGGCATTGAACTATGCCTGACGTTTGTCATC
ACGCTCGTCTATTTGACA---ACGATGGATGATCATCGA---------------------
---CGTGGGTTATCG---GCTTCCATAGGACCTGGACCCATCACGCTCGGCCTTACCTAC
ACGGCCTGCTCCTTTGTCGCTTTGCCATCAACTGGTGCCTCATTCAACCCTATGAGATCA
CTTGGTCCGGCTTTCGTCATGAAC---------AAATGGGACGCTCACTGGGTTTATTGG
CTGGGACCCATCGTGGGTGGCTGTCTGGCGGCCATCCTCTACGAGTTCATTTTCAACCCG
---TATCGCAACTCGCAACGC---------CGCAAGGGATCCATCCCCGATGGT------
------------------------GAC------------GCGTCGAGCATTCACAGCGAC
GAG------GAC---AACTACGAC---GACGTG---AAATCAACTGTG------------
---GCTAATTAC
CAGAGTGCC---------CGC---------------------------------------
------TCTCAGCACTACGATCAG---------------TACCGGCCG---GCAATTAGT
CCAGGTGGTCCGTCTAATCCAATTTCACCTAGC------GGTGCT------GGTGTCGGT
---------GGCGCT------------------------GTAGGAAACAACTGGCGGAAC
------CAGCAG---CGG---------------------------------ATGGAA---
CGAGCCGAATCG---------------------------GTTTATGGCGGCACCAAGTCG
CTT---TATCATTGCGGC---------------AAATCACCTGGACCGCCCAGCCGT---

---------------------------TCGAGCAATCTCAACCGCTCCCAGTCGGTCTAC
GCCAAA---CCTCCGGCCGCTCCGGCTGTCCAACCTCCCAACGTTTACGCTTCCCGCGGA
GCTCAGCTCCAGTCGGCGCAGAGC---ACTTATCTGGCC---------------------

------------------------------GCTCGCAACGTCGAC---------CTC---
--ATTCGCACCGAG
AGCATTTACGCCACGCGACAGCCGCCGGCT------CCT---ATGGCGTCCGGAAACAGC
AGCCGG---GCCGAA---
------------------AGCGTTTACGGCAGC---------CGCCATCCGCGCCATATG
GCCGATATGACGGACGTGGATCGA---CCCGTGCGCGACAATTCGCTCCTGACG---TCT
TCGGTGGCTAGAGAACCCGTTTAC------GCCACCAGGGCTCCCAGT------------
--CACGACCGTTCCAAA---

---CCGGAATCGTACCAACTGGAACCCATTTACCAGACTCGACGTGAAGTTGTACCATCG
GTGGCGGCCGTCGAGCCG------GCCCAGCGC---------------------------
---GGTGAAAGCCTCTACGATCGCAAAGTCCCTACTTATCGG------CCGGAAGCGACT
GTGGCCGCTTACAACCGTCGA------CCC------------AATCCGGAGCAG------
---------ATGGAAACATCGGCAACCAGCGCCGGAGGAGCTCCCATTTCTCCT---AGC
GACACGTCAAAAGATTCTGCCTATGGATCC---GTC---------CAC------------
--GGCACTTCGATT

---------------CAAAGTCCAGCGGAA------------------------------
-----TGGGCAGGTCACGGCCAGGAG------------AACAAT---------------C
AG---AGCAACTACAAT---------CAACAACAGCAGCAACAACAA-------------

---CAACAGC
ATGTTCAACATCTCCAACATGCATCCGCC------------TTTAGTCCTCATTTA---C
CCAACCAGCCTCCGCAAGGTTCTTACCAGATC------------------------CAGC
AACAGCAGCAGCAAGCCGGTCATCATCATCACTACGCTCAGAAGTTCCGAGCGGATTTGA
TGTCACCCAACAGCCAA---------CAACATTCGCCCGGA-------------------
-----------------------------TCATTTCACTCACCGGTGCAATAC-
>JAACYE010000001_Daphnia_obtusa_Bib
ATGGCGACAATGAGT---
------------------------------GTGGCCACGACTCCACTGACGAGTGCCGGT
CTCGAGAATCTGATGACGACATTGCTGGAACGTTTCGAGACGGCCTGCTTGGAGCAGCAG
GAG---
---GCGGGAAAT
GGCAACAAGATGCGGGTGCCAATGCAGGCCGAAATCCGATCGCTG---GATTTCTGGCGT
TCCATTGTGGCCGAATGCCTGGCTTCCTTTTTCCTCATCTTCATCATCTGCGGCTCGTTC
ATACCTTGGTCGGGACACGCT---------------------------------------
--CCGCCA
GCCATCAGCATCGCCTTGGCTTCCGGCTTCGCCGTGGCAGCCCTCACACTCTCCTTCGCC
CAAATATCTGGAGCTCATTTGAATCCAGTCATGTCGCTGGCGTTGCTGGTCACTCGCAAA
ATCTCGCCCATGAAAGCTTTGCTCTACGTCACGTCTCAAGGAGGTGGAGCCATCGCTGGG
GCTGCTCTTCTTTACGGGGTATCCAGTCCTGGACATCAAGGA------------------
------------------------------TACCTCGGAGCTACGATCGTCTCTACCGGT
---------TTATCCGATTGGCAAGCGCTGGGCATTGAACTATGCCTGACGTTTGTCATC
ACGCTCGTCTATTTGGTA---ACGATGGATGATCATCGA---------------------
---CGTGGGTTATCG---GCTTCCATAGGACCTGGACCCATCACGCTCGGCCTCACCTAC
ACGGCCTGTTCCTTTGTCGCTTTGCCATCAACTGGTGCCTCATTCAACCCTATGAGATCA
CTTGGTCCGGCTTTCGTCATGAAC---------AAATGGGGCGCTCACTGGGTTTATTGG
CTGGGGCCCATCGTGGGTGGCTGTCTGGCGGCCATCCTGTACGAGTTCATTTTCAACCCG
---TATCGCAACTCGCAACGC---------CGTAAGGGATCCATCCCCGATGGT------
------------------------GAC------------GCGTCGAGCATTCACAGCGAC
GAG------GAC---AACTACGAC---GACGTG---AAATCAACTGTG------------
---GCTAATTAC
CAGAGTGCC---------CGC---------------------------------------
------TCTCAGCACTACGATCAG---------------TACCGGCCG---GCAATTAGT
CCAGGTGGTCCGTCTAATCCAATTTCTCCTAGC------GGTGGT------GGTGGCGGT
---------GGCGCT------------------------GTAGGAAACAACTGGCGGAAC

------CAGCAG---CGG----------------------------------ATGGAA---
CGAGCCGAATCG---------------------------GTTTATGGCGGCACCAAGTCG
CTT---TACCATTGCGGC---------------AAATCACCTGGGCCGCCCAGCCGC---

---------------------------TCGAGCAATCTCAACCGCTCTCAGTCGGTCTAC
GCCAAA---CCTCCAGCCGCTCCGGCTGTCCAACCTCCGAACGTTTACGCTTCCCGCGGA
GCTCAGCTCCAGTCGGCGCAGAGC---ACTTATCTGGCC--------------------

------------------------------GCTCGCAACGTCGAC---------CTC---
--ATTCGCACCGAG
AGCATTTACGCCACGCGACAACCGCCGGCT------CCC---CTGGCGTCCGGAAACAGC
AGCCGG---GCCGAA---
------------------AGCGTTTACGGCAGC---------CGCCATCCGCGCCATATG
GCCGATATGACGGACGTGGATCGA---CCCGTGCGCGACAATTCGCTCCTGACG---TCC
TCGGTGGCTAGGGAACCCGTTTAC------GCCACCAGGGCTCCCAGT------------
--CACGACCGTTCCAAA---

---CCGGAATCGTACCAACTGGAGCCCATTTACCAGACTCGACGTGAAGTTGTACCGTCG
GTGACGGCCGTGGAGCCG------GCCCAGCGC---------------------------
---GGTGAAAGCCTCTACGATCGCAAAGTCCCTACTTATCGG------CCGGAAGCGACT
GTAGCCGCTTACAACCGTCGA------CCC------------AATCCGGAGCAG------
---------ATGGAAACATCGGCAACCAGCGCTGGAGGAGCTCCAGTTTCTCCT---AGC
GACACGTCAAAGGATTCGGCCTATGGATCC---GTC---------CAC------------
--GGCACTTCGATT

---------------CAAAGTCCAGCGGAA------------------------------
-----TGGACAGGTCACGGCCAGGAG------------AACAAT---------------C
AG---AGCAACTACAAT------CAACAACAGCAGCAGCAACAACAA-------------

---CAACAGC
ATGTTCAACATCTCCAACATGCTTCCGCC------------TTCAGTCCTCATTTA---C
CCAACCAGCCTCCACAAGGTTCTTACCAGGTC---------------------------C
AGCAGCAGCAGCAAGCCGGCCATCATCATCACTACGCTCAGAAGTTCCGAGCGGATTTGA
TGTCGCCCAACAGCCAA---------CAACATTCGCCCGGA------------------
-----------------------------TCATTTCACTCACCGGTGCAATAC-
>JAAVJA010004589_Daphnia_dubai_Bib
ATGACGACGATGAGT---
------------------------------GTAGCGACGACTCCACTAACGAGTGCTGGT
CTCGAGAATTTGATGACGACATTACTGGAACGTTTCGAGACAGCCTGCTTGGAGCAGCAG

---GCGGCCAAT
GGCAACAAGATGCGGGTGCCGATGCAGGCTGAAATCCGATCATTG---GATTTCTGGCGT
TCCATTGTGGCCGAATGCTTGGCTTCCTTTTTTCTTGTCTTCATCATCTGCGGCTCGTTC
ATACCTTGGTCGGGACACACT---------------------------------------
--CCGCCA
GCCATCAGCATCGCCTTGGCTTCCGGCTTTTCCATTGCAGCTCTCACACTCTCCTTCGCC
CAAATATCTGGAGCTCATTTGAATCCAGTCATGTCATTGGCTTTGCTGGTCACTCGCAAA

ATATCGCCCATGAAAGCTCTGCTCTACGTCACGTCTCAAGGAGGTGGAGCCATCGCCGGC
GCTGCTCTTCTTTACGGGGTAACCAGTCCTGGACATCAAGGA------------------
------------------------------TACCTTGGAGCTACAATCGTCTCTACCGGT
---------TTATCCGATTGGCAAGCGCTGGGCATTGAACTGTGCCTGACATTTGTCATC
ACGCTCGTCTATTTGGTA--
------------------------ATCGGACCGGGGCCCATCACGCTCGGCCTCACCTAC
ACGGCCTGTTCCTTTGTCGCTTTGCCATCGACTGGTGCCTCATTCAATCCTATGAGATCA
CTTGGACCNGCTTTCGTCATGAAC---------AAATGGACGCTCACTGGGTTTATTGG
CTCGGACCCATCGTGGGTGGCTGTCTGGCGGCCATCCTCTACGAGTTCATTTTCAACCCG
---TATCGCAACTCTCAGCGC---------CGTAAGGATCCATCCCCCGATGGA------
------------------------GAC------------GCGTCCAGCATTCACAGCGAC
GAG------GAC---AACTACGAC---GACGTG---AAATCGACTGTG------------
---GCTAATTAC
CAGAGTGCC---------CGC---------------------------------------
------TCTCAGCATTACGATCAG---------------TACCGACCG---GCCATTAGT
CCAGGTGCTCCGTCCAATCCCATTTCTCCTAGC---------GGT------GCAGGTGGT
---------GGCGCT------------------------GTAGGGAACAACTGGCGGAAC
------CAGCAG---CGG---------------------------------ATGGAA---
CGAGCCGAATCG---------------------------GTTTATGGTGGCACGAAATCG
CTT---TACCATTGCGGC---------------AAATCTCCTGGACCTCCTAGCCGC---

---------------------------TCCAGTAATCTCAACCGCTCGCAATCGGTTTAC
GCTAAA---CCTCCAACCACTCCGGCTGTCCAACCTCCCAATGTTTACGCTTCTCGCGGA
GCTCAACTCCAGTCTGCCCAGAGC---ACTTATCTCGCC---------------------

------------------------------GCTCGCAACGTCGAC---------CTC---
--ATTCGCACCGAG
AGCATTTACGCCACACGACAACCACCGGCT------CCT---TTGGCGTCCGGAAACAGT
AGCAGA---GCCGAG---
------------------AGCGTTTACGGCAGC---------CGCCATCCGCGCCATATG
TCTGATATGACGGATGTGGATCGA---CCTGTACGGGACAATTCGCTTCTAACG---TCT
TCGGTGGCTAGAGAACCCGTTTAC------GCCACCAGGGCTCCCAGT------------
--CACGACCGTTCGAAA---

---CCCGAGTCGTATCAATTGGAGCCAATTTACCAGACTCGACGTGAAGTTGTACCTTCG
GTTGCGGCCGTTGAGCCG------GCCCAGCGC---------------------------
---GGTGAAAGCCTCTACGATCGTAAAGTACCGACTTATCGG------CCGGAAGCGACT
GTAGCCGCTTAC--
------------------------------------GGAGCTCCTGTTTCTCCT---AGT
GACACATCAAAGGATTCGGCCTACGGATCC---GTT---------CAT------------
--GGCACTTCGATA

---------------CAAAGTCCGGCTGAA------------------------------
-----TGGCAAGTTCACGGCCAGGAG------------AACAAT---------------C
AG---AGCAACTACAAT------CAACAGCAACAACAACAG-------------------

--------------CAACATGCTTCCGCC------------TTCAGTCCTCATCTA---C
CCAATCAGCCTCCTCAAGGTTCTTACCCGATC---------------------------C
AGCAGCAACAA------GGTCATCACCATCATTACGCTCAGAAGTTCCGAGCGGATTTGA
TGTCGCCCAATAGCCAA---------CAACATTCGCCCGGA-------------------
-----------------------------TCATTTCACTCACCGGTGCAATAC-
>CAH0109652_Daphnia_galeata_Bib
ATGACGACAATGAGT---
------------------------------GTAGCTACGACTCCATTAACGAGTGCCGGT
CTTGAGAATTTGATGACGACATTATTGGAACGTTTCGAGACTGCCTGCTTGGAGCAGCAA

---GCGGGAAAT
GGCAACAAGATGCGGGTACCGATGCAAGCTGAAATTCGATCATTG---GATTTCTGGCGT
TCCATTGTGGCCGAATGCCTGGCTTCGTTTTTTCTCGTCTTCATCATCTGTGGCTCGTTC
ATACCTTGGTCGGGACACACT---------------------------------------
--CCGCCA
GCCATCAGCATCGCATTGGCTTCCGGCTTTTCTGTAGCAGCTCTCACACTCTCGTTTGCC
CAAATATCTGGAGCTCATTTGAATCCAGTCATGTCATTGGCTTTACTGGTCACTCGCAAA
ATATCGCCCATGAAAGCTTTGCTCTACGTCACGTCTCAAGGAGGTGGAGCCATTGCTGGC
GCTGCTCTTCTTTACGGTGTAACCAGTCCTGGACATCAAGGA------------------
------------------------------TATCTTGGAGCTACAATAGTTTCTACCGGT
---------TTATCCGATTGGCAAGCGCTGGGCATTGAACTGTGTCTCACATTTGTCATC
ACGCTCGTCTATTTGACA---ACGATGGATGATCATCGA---------------------
---CGCGGGTTATCG---GCTTCCATCGGACCGGGGCCCATCACGCTCGGCCTCACCTAC
ACGGCCTGTTCCTTTGTCGCTTTGCCATCGACTGGTGCCTCATTTAATCCTATGAGATCA
CTTGGACCGGCTTTCGTCATGAAC---------AAATGGGACGCTCACTGGGTTTATTGG
CTCGGACCCATCGTGGGTGGCTGTCTGGCGGCTATTCTCTACGAGTTCATTTTCAACCCG
---TATCGCAACTCGCAGCGC---------CGCAAGGGATCTATCCCCGATGGA------
------------------------GAC------------GCGTCCAGCATTCACAGCGAC
GAG------GAC---AACTACGAC---GACGTG---AAATCAACGGTA------------
---GCTAATTAC
CAGAGTGCT---------CGC---------------------------------------
------TCTCAGCATTACGATCAG---------------TACCGACCG---GCCATTAGT
CCAGGTGCTCCGTCCAATCCAATTTCTCCTAGT------GGTGGTGGT---GGCGGCGGC
---------GGCGCT------------------------GTAGGAAACAACTGGCGGAAC
------CAGCAG---CGG---------------------------------ATGGAA---
CGAGCCGAATCG---------------------------GTTTATGGCGGCACAAAATCA
CTT---TACCATTGCGGC---------------AAATCACCTGGACCTCCTAGCCGT---

---------------------------TCCAGCAATCTCAATCGTTCACAATCCGTCTAC
GCTAAA---CCTCCGGCTACTCCGGCTGTCCAACCGCCCAATGTTTATGCTTCTCGTGGA
GCTCAACTTCAATCTGCCCAGAGC---ACTTATCTGGCC---------------------

------------------------------GCTCGCAATGTTGAC---------CTT---
--ATTCGTACTGAA
AGCATTTACGCTACACGACAACCGCCGGTC------CCT---TTGGCGTCCGGAAACAGT
AGCAGA---GCCGAA---
------------------AGCGTTTACGGCAGT---------CGCCATCCGCGACATATG
GCCGATATGACGGATGTCGATCGACCTCCTGTACGCGACAATTCTCTTCTAACG---TCT

```
TCGATCGCTAGAGAACCCGTTTAC------GCTACCAGGGCTCCCAGT------------
------------------------------------------CATGATCGTTCCAAA---
------------------------------------------------------------
------------------------------------------------------------
---CCCGAATCCTACCAATTGGAGCCGATTTATCAGACTCGACGTGAAGTTGCACCTTCT
GTTGCGGCCGTAGAGCCC---GTGGCCCAACGT---------------------------
---GGTGAAAGTCTCTACGATCGTAAAGTCCCAACTTATCGG------CCGGAAGCGACT
GTAGCCGCTTATAATCGTCGA------CCC------------AATCAGGAGCAG------
---------ATGGAAACATCGGCCACCAGTGCTGGAGGAGCTCCCGTTTCTCCT---AGC
GACACTTCAAAGGATTCAGCTTACGGATCT---GTT---------CAT------------
------------------------------------------------GGCACTTCGATT
------------------------------------------------------------
---------------CAAAGTCCAGCGGAA------------------------------
-----TGGCAAGTTCACGGCCAGGAG------------AACAAT---------------C
AA---AGCAATTACAATCAA---CAACAGCAGCAGCAACAACAACAA-------------
------------------------------------------------------------
-----------------------------------------------------CAACAGC
ATGTACAACATCTCCAACATGCTTCCGCC------------TTTAGTCCCCATCTA---C
CCAACCAACCTCCTCAAGGTTCTTACCCGATC---------------------------C
AGCAGCAACAGCAAGCTGGGCATCATCATCACTACGCTCAGAAATTCCGAGCGGATTTGA
TGTCGCCCAACAGCCAA---------CAACATTCGCCCGGA-------------------
-----------------------------TCATTTCACTCACCGGTGCAATAC-
>XM_032927904_Daphnia_magna_Bib
ATGACT------AGT---------------------------------------------
------------------------------GTGGCGACGACACCGTTGACGAGTGCCGGT
CTTGAGAATTTGATGACGACATTACTCGAACGTTTTGAGACGGCCCGTTTGGAGGAGGAA
------------------------------------------------------------
---------------------------------------------------GCGGGAAAT
GGCAACAAGATGCGGGTACCTATGCAGGCTGAAATTCGATCGCTA---GATTTCTGGCGT
TCCATTGTGGCCGAATGCCTGGCTTCCTTTTTTCTCGTCTTCATCATCTGCGGCTCGTTC
ATTCCATGGTCGGGGCACACG---------------------------------------
------------------------------------------------------CCACCC
GCCATCAGCATCGCCTTGGCTTCCGGCTTCTCTGTGGCAGCACTCACCCTCTCCTTCGCC
CAAATATCTGGAGCTCATTTGAATCCAGTTATGTCATTGGCTTTGCTGGTCACTCGCAAA
ATATCACCCATGAAAGCTTTGCTCTACGTCACGTCACAAGGCGGTGGAGCTATTGCAGGA
GCCGCTCTTCTTTACGGAGTAACCAGTCCTGGACATCAAGGA------------------
------------------------------TACCTTGGAGCTACCATCGTCTCTACCGGT
---------TTATCTGATTGGCAAGCCCTGGGCATTGAACTGTGCTTGACATTTGTCATC
ACGCTCGTCTATTTGACA---ACGATGGATGATCATCGT---------------------
---CGCGGTTTGTCG---GCCTCCATTGGACCCGGCCCACTCACGCTCGGCCTCACTTAC
ACGGCCTGTTCGTTTGTCGCTCTGCCATCGACCGGAGCCTCGTTCAACCCTATGAGATCG
CTTGGTCCGGCTTTCGTAATGAAT---------AAATGGGACGCGCATTGGGTTTATTGG
CTGGGTCCCATCGTGGGCGGCTGTCTGGCCGCCATCCTGTACGAGTTCATTTTCAACCCG
---TATCGCAACTCGCAACGC---------CGTAAGGGATCCATTCCGGACGGT------
------------------------GAT------------GCGTCCAGCATTCACAGTGAC
GAG------GAC---AACTACGAC---GATGTG---AAATCAACCGTG------------
---------------------------------------------------GCCAATTAC
CAGAGTGCC---------CGC---------------------------------------
```

------TCTCAGCATTACGATCAG---------------TACCGACCG---GCAATTAGT
CCAGGTGGCCCGTCCAATCCGATTTCTCCTAGC------GGTGGT------GGCGGTGGT
---------GGCGCT------------------------GTGGGAAACAACTGGCGCAAT
------CAGCAG--
CGCATGGAACGA---------------GCCGAATCG---GTTTATGGCGGTACGAAATCA
CTT---TACCATTGCGGC---------------AAGTCACCTGGCCCACCCAGCCGC---

---------------------------TCGAGCAATCTCAATCGGTCGCAATCCGTCTAC
GCTAAA---CCTCCAGCCGGACCGGCCGTTCAGCCGCCCAACGTTTACGCTTCGCGTGGA
GCTCAACTGCAGTCGGCGCAGAGC---ACTTACATTGCC---------------------

------------------------------GCTCGTAATGTCGAC---------CTC---
--ATTCGCACGGAA
AGCATTTACGCCACTCGACAACCTCCGCAACAACCTCCT---CCGGCGTCCGGTAATAGC
AGCAGA---GCCGAA---
------------------AGCGTTTACGGCAGT---------CGCCATCCGCGTCACATG
GCTGACATGACGGATGTGGATCGA---CCTGTACGAGACAATTCTTTGCTGACG---TCG
TCCATCGCAAGGGAACCTGTCTAC------GCCACACGAACTCAAAGT------------
------------------------------------TCGGGTCACGATCGTTCCAAA---

---CCGGAATCGTACCAACTGGAACCTATTTACCAGACTAGACGTGAAGTTGTGCCTTCC
GTGGCCACTGTCGAGCCC------GCCCAGCGC---------------------------
---GGTGAAAGCCTTTACGATCGTAAAGTCCCTACTTACCGG------CCGGAAGCGACT
GTGGCTGCGTATAACCGTCGC------CCC------------AATCAGGAGCAG------
---------ATGGAGACATCCGGGACCAGCACT---GGAGCGCCTATTTCGCCC---AGT
GACACGTCCAAGGATTCGGCTTATGGATCT---GTT---------CAT------------
--GGCAATTCGATA

---------------CAAAGTCCATCCGAA------------------------------
-----TGGCCG------GCGCAGGAG------------AATAAT---------------C
AA---AGCAATTATAAC------CAACAGCAGCAA-------------------------

---CAACAAC
ACGTGCCACATCTGCAACATGCTTCCGCC------------TTCAGTCCTCATCTC---C
CTAATCCACCTAATCAAGGACCTTACCAGATG---------------------------C
AACAACAACAA---GCTGGACACCACCATCACTACGCTCAGAAATTCCGAGCGGATTTGA
TATCGCCCAGTGGTCAACAA------CAACATTCGCCCGGA-------------------
-----------------------------TCGTTTCACTCACCGGTGCAATAT-
>CM023192_Daphnia_carinata_Bib
ATGACT------AGT---
------------------------------GTGGCGACGACACCGTTGACGAGTGCCGGT
CTTGAGAATTTGATGACGACATTGTTGGAACGTTTCGAGACGGCACGTTTGGAGGAGGAA

---GCGGGAAAT
GGCAACAAGATGCGGGTACCTATGCAGGCTGAAATTCGATCGCTA---GATTTCTGGCGT
TCCATTGTGGCCGAATGCCTGGCTTCCTTTTTTCTCGTCTTCATCATCTGCGGCTCGTTC
ATTCCATGGTCGGGGCACACG---------------------------------------

--CCACCC
GCCATCAGCATCGCCTTGGCTTCCGGCTTCTCTGTGGCAGCACTGACACTCTCCTTCGCC
CAAATATCTGGAGCTCATTTGAATCCAGTCATGTCATTGGCTTTGCTGGTCACTCGCAAA
ATATCACCCATGAAAGCTTTGCTCTACGTCACGTCTCAAGGCGGTGGAGCTATTGCAGGA
GCCGCCCTCCTTTACGGGGTAACCAGTCCTGGACATCAAGGA------------------
------------------------------TACCTTGGAGCCACCATCGTCTCTACCGGT
---------TTATCCGATTGGCAAGCCCTGGGCATTGAACTGTGCTTGACATTTGTCATC
ACGCTCGTCTACTTGACA---ACGATGGATGATCATCGC---------------------
---CGCGGTTTATCG---GCCTCCATTGGACCCGGCCCACTCACGCTCGGACTCACTTAC
ACGGCCTGCTCGTTTGTCGCTTTACCGTCAACTGGAGCCTCGTTCAACCCCATGAGATCG
CTTGGCCCTGCTTTTGTAATGAAT---------AAATGGGACGCGCATTGGGTTTATTGG
CTGGGGCCCATCGTGGGCGGCTGTCTGGCCGCCATCCTGTACGAGTTCATTTTCAACCCG
---TATCGCAACTCGCAACGC---------CGTAAGGGATCCATTCCGGACGGT------
------------------------GAT------------GCGTCCAGCATTCACAGTGAC
GAG------GAC---AACTACGAC---GATGTG---AAATCAACCGTG------------
---GCCAATTAC
CAGAGTGCC---------CGC---------------------------------------
------TCTCAGCATTACGATCAG---------------TACCGACCT---GCAATTAGT
CCAGGTGGCCCGTCCAATCCGATTTCACCTAGC------GGTGGT------GGTGGCGGT
AGT------GGCGCT------------------------GTGGGAAACAACTGGCGCAAT
------CAGCAG--
CGCATGGAACGA---------------GCCGAATCG---GTTTATGGCGGTACGAAATCG
CTT---TACCATTGCGGC---------------AAGTCACCTGGCCCGCCCAGCCGT---

---------------------------TCCAGCAATCTCAATCGCTCGCAATCCGTCTAC
GCTAAA---CCCCCGGCCGGACCGGCCGTTCAGCCGCCCAACGTTTACGCTTCGCGCGGA
GCTCAACTGCAGTCGGCGCAGAGC---ACTTACCTGGCC---------------------

------------------------------GCCCGTAATGTCGAC---------CTC---
--ATTCGCACGGAA
AGCATTTACGCCACTCGACAACCTCCGCAACAACCTCCTCTCCCGACGTCCGGCAATAGC
AGCAGA---GCCGAA---
------------------AGCGTTTACGGCAGC---------CGCCATCCGCGCCACATG
GCCGACATGACGGACGTGGATCGA---CCTGTACGAGACAATTCTTTGCTGACG---TCG
TCCATGGCAAGGGAACCTGTCTAC------GCTACACGAACTAATCAG------------
---------------------------------AGTTCGGGTCATGATCGTTCCAAA---

---CCGGAATCGTACCAACTGGAGCCCATTTACCAGACTAGACGTGAAGTTGTGCCTTCC
GTGGCCACTGTGGAGCCC------GCCCAGCGC---------------------------
---GGTGAAAGCCTTTACGATCGTAAAGTGCCTACTTACCGG------CCGGAAGCGACT
GTGGCCGCGTATAATCGCCGA------CCC------------AATCAGGAGCAG------
---------ATGGAGACATCCGGGACCAGCACT---GGAGCCCCTATTTCTCCC---AGT
GACACGTCCAAGGATTCGGCATATGGATCT---GTT---------CAT------------
--GGCAATTCAGCT

---------------CAAAGTCCATCCGAA------------------------------
-----TGGCCG------GCTCAGGAG------------AATAAT---------------C

```
AA---AGCAATTACAAC------CAACAGCAGCAG-------------------------
------------------------------------------------------------
-----------------------------------------------------CCACAGC
ACGTGCCACATCTGCAACATGCATCCGCT------------TTCAGTCCTCATCTC---C
CTAATTCACCCAATCAAGGACCTTACCAGATG---------------------------C
AACAACAACAA---GCTGGACACCACCATCACTACGCTCAGAAATTCCGAGCGGATTTGA
TATCGCCCGGTGGTCAACAA------CAGCATTCGCCCGGA-------------------
-----------------------------TCGTTTCACTCACCGGTGCAATAT-
>JAKUCG010000182_Ceriodaphnia_dubia_Bib
ATGACGACGATGAGT---------------------------------------------
------------------------------GTTGCCACGACACCGTTGACCAGTGCCGGT
CTTGAGAACATGATGACGACATTGCTGGAGCGATTCGAGACGGTGAGATTGGAGCAG---
------------------------------------------------------------
---------------------------------------------------GCGGGAAAT
AGCAACAGGATGCGGGTGCCGATGCAAGCTGAAATTCGATCGCTC---GATTTCTGGCGT
TCGATCGTGGCTGAATGCTTGGCATCTTTCTTCCTCGTCTTCATTATTTGCGGATCGTTT
ATCCCCTGGTCGGGGCACACG---------------------------------------
------------------------------------------------------CCACCA
GCCATCAGCATCGCCCTGGCTTCCGGCTTTTCCATCGCAGCTCTCACCCTGTCTTTCGCC
CAAATATCAGGAGCTCATTTGAATCCAGTCATGTCTCTGGCTTTGTTGGTCACTCGCAAA
ATATCGCCGATGAAGGCTCTGCTCTACGTCACATCGCAAGGAGGTGGTGCCATCGCTGGA
GCGGCTCTCCTTTATGGGGTGACCAGTCCTGGACATCAAGGA------------------
------------------------------TACCTCGGAGCTACGATCGTCTCCAACGGC
---------CTTTCCGATTGGCAAGCCTTGGGCATCGAACTGTGTCTGACATTTGTCATT
ACTCTTGTTTATTTGACA---ACGATGGATGATCACCGA---------------------
---AGAGGTTTATCG---GCTTCGATCGGGCCGGGTCCCCTTACGCTCGGACTAACTTAC
ACGGCCTGTTCTTTTGTCGCTTTGCCGTCGACCGGAGGTTCTTTCAATCCAATGCGGTCG
CTCGGACCGGCTTTCGTCATGAAC---------AAATGGGACGCGCATTGGGTTTATTGG
CTTGGACCCATCGTGGGCGGTTGTCTGGCCGGCATCCTGTACGAGTTTATATTCAACCCG
---TATCGCAACTCGCAGCGC---------CGCAAGGGATCCATCCCCGATGGT------
------------------------GAT------------GCGTCGAGCATTCACAGCGAC
GAG------GAC---AACTACGAC---GACGTC---AAATCGACTGTG------------
---------------------------------------------------GCTAATTAC
CAGAGCGCC---------CGC---------------------------------------
------TCCCAGCATTACGATCAG---------------TACCGTCCG---GCTATTAGT
CCAGGTGGTCCGTCCAATCCAATCTCGCCTAGT---------GCA------GGTGGCGGC
---------GGCCCT------------------------GTCGGTAACAACTGGAGGAAC
------CAACAA------------------------------------------------
CGCATGGAACGA---------------GCCGAATCG---GTTTACGGCGGTACAAAGTCG
CTT---TATCATTGCACC---------------AAGTCACCTGGCCCGCCCAGTAGG---
------------------------------------------------------------
---------------------------TCAAGCAATCTCAACCGTTCTCAGTCGGTGTAC
GCAAAA---CCCCCTGCCGGACCGGCTGTCCAGCCGCCCAATGTTTACGCCTCGCGCGGA
GCTCAAATGCAGTCGGCACAGAGC---ACTTATCTG------------------------
------------------------------------------------------------
------------------------------GCTCGTAATGTCGAC---------CTC---
------------------------------------------------ATTCGCACCGAA
AGCATTTATGCCACTCGTCCGCCGCCCGTC------------------------------
```

AACAGA---GCCGAA---
------------------AGCGTTTACGGTACC---------AGGCATCCGCGCCATATG
ACCGATATGACGGACGTAGACCGG---CCCGGTCGAGAAGTGTCATTGCTCACC------
TCA---CCTAGAGAACCGGTTTAC------TCTGCCAGAAACACTGCC------------
---------------------------------GGACACCAACAGGATCGTTCTAAA---

---CCCGAGTCTTACCAGTTGGAGCCCATCTACCAGACGAGGCGCGAAGTTGTGGCCCCA
TCGGCCGTTGTCGAGCCG------GCCCAGCGC---------------------------
---GGCGAAAGCTTGTATGATCGCAAAGTCCCGACTTATCGT------CCGGAAGCGACT
GTAGCCGCGTACAACCGCCGA------CCC------------AATCAGGAACAG------
---------ATGGAAACATCTGCCAATAGCACT---GGAGCGCCAATTTCGCCC---AGC
GATACGTCCAAGGATTCAGCTTATGGATCC---GTC---------CAT------------
--GGAAATTCGATC

---------------CAAAGCCCAGCCGAG------------------------------
-----TGGCCGCACAATCAGCAGGAAATG---------AGCAAT---------------C
AG---ACGAATTACAGC------CAGCAACAGCAGCAG----------------------

---CAGCAAC
ATATA------CAGCAGCATGCCTCAGCT------------TTCGCTCCTCATCAA---C
CCAAT---------CAGGGACCTTACCAGATC---------------------------C
AGCAACAGCCG---GGCGGA------CATCAGTACGCTCAGAAATTCCGAGCTGATCTGA
TATCGCCCAATGGCCAACAG------CAACATTCGCCCGGA-------------------
-----------------------------TCATTTCACTCACCGGTGCAATAT-
>GGQP01079573_Diaphanosoma_celebensis_Bib
ATGACGACCATCAGC---
------------------------------GTC---TCCGCTCCTCTAACGACGGCCAGT
GTCGAGGGTTTGATGCAAACGTTGCTCGAGAGATTCGAAACGCTTCGAATGGATCAA---

CCGAAACCGATGCGGGTGCCGATGCAGGCCGAAATTCGATCCCTG---GACTTCTGGCGC
TCCATCGTGGCTGAATGTTTAGCCACTTTCTTCTTGATCTTCATCATCTGTGGGGCCTTC
ATTCCTTGGGCAGGACACACG---------------------------------------
--CCGCCG
TCCATTGGCATCGCTTTAGCCGCCGGTTTCGCCACTGCGTCGCTAACGTTGTGCTTCTCG
CACGTTTCCGGTGCTCATTTCAATCCGGTGGTAACGTTAGCCCTTTTGGTAACGAGAAAG
GTCTCGCCGATCAAAGCTTTCCTCTTCACAACGGCTCAAGGAGGCGGCGCCATTGCAGGA
GCTGCTCTCCTCTACGGAGTGACGGCGTCTGGCTACCAAGGA------------------
------------------------------TCTCTGGGAGCCACGGTGATCTCGGCCGGT
---------TTATCCGAATGGCAGGGACTGGGCATCGAGCTATGCCTGTCGTTCATTCTG
ACGTTGGTCTACTTGACC---ACCATGGACGACCACCGT---------------------
---CGAACGCTGCCC---GCCTCTCTCGGACCTGGACCTCTGACCTACGGCTGGCGTAC
GCTGCTTGTTCCTTAATGGCTCTGCCCTGCACCGGAGCTTCTTTCAATCCGATGCGTTCA
TTGGGACCGGCGTTCGTTATGAAC---------CGATGGACGCTCACTGGATTTACTGG
TTGGGTCCCATCGTCGGTGGATGTCTAGCAGCGGTACTTTACGAATTTATTTTCAATCCT
---TACCGCAACGCCCAACGC----------CGTAAGGATCCATC---GACGGA------
------------------------GAT-------------TCTTCCAGTATCCACAGCGAC

```
GAA------GAT---AATTACGAC---GATATC---AAGTCGACAGTG------------
---------------------------------------------------CACAACTAC
CAGAGCGCT---------CGC---------------------------------------
------TCGCAGCATTACGACCAG---------------TACCGACCG---GCCATTACT
CCCGGAGGACCGTCCAATCCCGTCTCACCGAAT---------------------------
---------GGAATG------------------------CCAGGAAGTAATTGGCGCAAC
------------------------------------------------------------
CGCATGGAACGC---------------GCTGAATCG---GTTTACGGTGGCACGAAATCC
CTC---TAC---TGC------------------AAATCACCTGGG---GTCACTCGA---
------------------------------------------------------------
------------------------------GCCAGTTTGAACCGTTCGCAATCGGTTTAC
GCCAAA---TCGCCCGGCGCCACGATG------CCGGCCAACTCGTACGCTTCGCGGCCG
---AACCTACAGGCTGCACAAAGC---ACTTACTTG------------------------
------------------------------------------------------------
------------------------------GCCCGCAACGTCGAC---------CTC---
------------------------------------------------ATCCGCACTGAA
AGTATTTATGCTTCGCGTCCGACAACGACA------CCC---GTC---------------
AATCGG---AACGAG---------------------------------------------
------------------AGTCTGTACGGCCAGACCAAGAGTCGTCAGAACGTTCATCCG
ACGGAAATGACCAGCTCGACTCGC---GAGCCGCGTTCA---------------------
------CCGCGCGAGCCTATTTAC------GGCACCAGTGCAACGACCTCC---------
------------------------------CTGGAGTACGGTAACCAACGTGGCAAA---
------------------------------------------------------------
------------------------------------------------------------
ACTGCCGAATCCTACGCCATGGAGCCCGTCTACGGAACACGTCGAGAT------------
---------GTCGATCCC------CCCAGCCGC---------------------------
---ACTGAGAGTCTTTACGGGCGCAAGACGAGCGTGTACCGT------CAAGAATCAACT
TACGGCATGGTACCATCAGTC------ACG------------CATCAAGGCCAA------
------GAGGCAGAAAGGTCAAACGGTTCCAGC---GGCGTCGCCATCTCACCA---AGT
GACACGTCCAAGGACTCGGCTTACGGATCT---GTT---------CAT------------
------------------------------------------------GGAAATTCCATG
------------------------------------------------------------
---------------CAGAGCCAAAGTGAC------------------------------
-----TGGACGCAGAACGGTCAGGAGAGCAGCAGCCAAGGTCGC------------GAAC
AA---ACCAACTTCCAG------ACCGGCAGCAGTGCG----------------------
-----------------------GCTTACAAT---------------CCA----------
--CCGGCCGGTCAGCCGCCAATTCCA---------------------------GGTGCAC
ACAACCACCACCAGAGTCACAATGTTGCAGGTCAAGGCCCTCTGTCATCCTCGTCT---T
CGTCGTCAGCTGGTCAAGGATCATCCACTTCG---------------------------T
ATCATGCGCAGAATGCG---------CACATTTACGCACAGAAATTCCGGTCGGATTTGG
TGTCGCCCAAAGGGCATCATTCGCCGTCGTACACACCGGGG-------------------
-----------------------------ACATACCACTCACCCGTGCAGTAC-
>NKDA01000002_Eulimnadia_texana_Bib
ATGACAACAGCCAGT---------------------------------------------
------------------------------GTG---ACATCGCCATTAACGACAGCCAGT
CTGGAATCGCTTCTTCAGACACTTTTGGAGCGTTTGGAGAGTTCCAAAAACGATCTG---
------------------------------------------------------------
------------------------------------------------------------
```

```
GCCAAGCCGTACAGTGTTCCTATGCAAACAGAGATCCGTTCACTG---GATTTTTGGCGA
GCCATTGTTGCAGAATGTCTTGCTACTTTCTTCCTGGTTTTTGTCATTTGCGGATCCTTC
GTCCCTTGGAGTGGCCATAGC---------------------------------------
------------------------------------------------------CCGCCG
TATATCGGGATAGCCCTGGCTGCGGGATTCGTCGTGGCTGGCCTAACCCTGGCTTTTAGC
CACACTTCAGGTGCTAATTTTAACCCTGTCGTCTCGTTGGCGCTTCTTGTGACCCGCAGA
ATTTCGCCAATTAAGGCCTTCCTGTTTGTGACGGCGCAAGGCGGAGGCGCCATTGCTGGT
GCTGCTTTATTATACGGGGTAACGATTCCGGGTAATCAGGGT------------------
------------------------------AACCTGGGAGCAACGGTGGTATCGTCGGGA
---------CTGACGGAATGGCAGGCGCTGGGTATCGAGTTGTGCATGACCTTCATCATC
GTTCTAGTTTACCTGACT---ACCATGGATTCGCAACGG---------------------
---CGGGCTCTGTCT---GCTTCCATCGGACCTGGACCTCTCACTTATGGCCTCATCTAT
ATGGCCTGCTCCCTTGTGGCTCTGCCATCTACCGGCGCTTCGTTCAACCCGATGCGGTCG
CTGGGACCTGCCTTCGTTATGAAC---------AAGTGGGACGCCCACTGGGTATATTGG
CTGGGTCCTATTGTTGGTGGCTGCATGGCTGGATTGTTGTACGAGTTCATATTCAATCCC
---TATCGCAGTGGACGCAAA---------CGTAAGGGATCCGTA---GATGGA------
------------------------GAT------------TCTACCGGAGCCCAGAGTGAC
GAA------GAA------TACGAC---GAAGTC---AAGTCTACGGTG------------
---------------------------------------------------CAGAACTAC
CATTCCATG---------CGG---------------------------------------
------ATGCAGCACTACGATCAG---------------TACCGTCCA---CCGTACAGC
CCGGGAGGTCCTTTGAACCCCACCACATCCCAC---------------------------
---------GGTAAA------------------------TTATCAGCA------------
------------------------------------------------------------
AGGGTCGATAGG---------------GCGGAATCC---GTTTACGGTGGGACCAAATCG
CTG---TAC---TGC------------------AAATCACCTCCC---ATGACCAGG---
------------------------------------------------------------
------------------------------GCCAATCTGAACCGTTCCCAGTCGGTTTAC
GCCAAA---CCCCCGGGTCTGAATCAGGGTCCAGCCGGTCCGGCTTACCCCATGAAG---
---ACCCTACCTTCTGCCCAGAGC---ACCTACTTG------------------------
------------------------------------------------------------
------------------------------GCCCGAAATGTGGAC---------CTA---
------------------------------------------------ATCCGGACCGAA
AGCATTTATGCTACCAGACCTGCTAACATA------CCC---ATC---------------
AATCGG---TACCCA---------------------------------------------
------------------AAATCGAAAAACAGACAGAACGGC---CAAGGAAATTACCCG
GCTGACTTGATGTCACCCGGAAGC---TCCGCCCACTCG---------------------
------CCCGGAAACACGCCCACA------GCGTTTCATTCGCCCAATCCC---------
------------------------------GTGCAGTAT---------------------
------------------------------------------------------------
------------------------------------------------------------
------------------------------------------------------------
------------------------------------------------------------
------------------------------------------------------------
------------------------------------------------------------
------------------------------------------------------------
------------------------------------------------------------
------------------------------------------------------------
```


>JAIQCU010005262_Leptestheria_dahalacensis_Bib
ATGACGACCATCAGT---
------------------------------GTA---ACCTCGCCACTGACAACGGCCAGT
CTAGAATCGCTTCTTCAGACCCTGCTGGAACGTTTTGAGAGTTGCAAAAGTGACTTA---

CCCAAAGCCGGCAGTATCCCCATGCAAGTGGAGATCCGCTCCTTG---GACTTCTGGCGC
GCTATTGTAGCCGAGTGCCTGGCCACTTTTTTCCTCGTCTTTGTCATCTGCGGATCCTTC
GTGCCTTGGAATGGACACAGT---------------------------------------
--CCGCCT
TACATTGGAATCGCCTTGGCCACTGGGTTCGTGGTCGCTGGACTGACTCTCTGTTTCACT
CATACTTCCGGTGCTAATTTCAACCCCGTCGTCTCGCTGGCATTGTTGGTGACTCGCAGA
ATTTCGCCCATCAAGGCCTTCCTGTTTGTTACGGCGCAGGGAGGAGGGGCTATCGCTGGC
GCCGCACTACTTTATGGGGTGACCGTGTCTGGACAACAAGGC------------------
------------------------------AACCTGGGCGTCACTTCTGTTTCCCTGGGT
---------CTGTCGGAATGGCAAGCGCTGGGCATCGAACTGTGCATGACGTTCATCATC
GTCCTCGTCTACCTGACC---ACCATGGACTCGCACCGG---------------------
---CGGGCCCTGTCC---GCCTCCATCGGACCCGGACCCTTGACTTACGGACTCATCTAC
ATGGCCTGTTCTCTTGTCGCTTTGCCATCTACCGGCGCTTCATTCAACCCTATGCGATCG
CTGGGACCTGCCTTCGTGATGAAC---------AAATGGGATGCTCATTGGGTGTATTGG
TTGGGCCCCATCGTAGGCGGATGCATGGCCGGACTCCTCTACGAGTTCATCTTTAACCCG
---TACCGCAGTGGAAGGAGA---------CGCAAGGGTTCTGTT---GACGGA------
------------------------GAT------------TCGACGGGGGCTCACAGCGAC
GAA------GAG------TACGAC---GAAGTG---AAATCCAGCACC------------
---CAGAACTAT
CACTCGGTG---------CGG---------------------------------------
------ATGCAGCACTACGATCAG---------------TACCGTCCG---CCCTACAGT
GCCGGCGGACCCATGAATCCCACCTCTCCAACT---------------------------
---------GGT--

CGCCTGGATCGT---------------GCGGAATCA---GTCTACGGTGGCACCAAATCG
CTC---TAC---TGC------------------AAATCCCCGCCC---ATGAGCCGC---

------------------------------GTCAATCTGAACCGGTCCCAGTCCGTCTAC
GCTAAA---CCGCCCCCGCCGGCATCCGGCCCCGTGGGCGTGGCCTATCCAACCAAG---
---ACACTTCCGGCGGCGCAGAGT---ACCTACCTG------------------------

------------------------------TCGCGCAATGTGGAT---------CTC---
--ATCCGCACCGAG
AGTATTTACGCCACCCGCCCTGCCAATATT------CCC---GTT---------------
AACAGG--

>RJJA01000024_Lepidurus_arcticus_Bib
ATGGCTAATGTGAGC---
------------------------------------TTGGCGCCG---AATTCCGCGTCC
ATGGAAACGTTGTTGCAAACTTTAATGGACAGGCTCGAATCGCAGCGAACAGAGCGT---

CCTAAATTGGCGCGAGTGCCTATGCAAGCCGAAATTCGGAGCCTG---GATTTTTGGCGT
GCCGTCGTCGCCGAGTGCCTCGCTACTTTTTTCTACGTCTTCCTTATCTGCGGCTCTTTC
GTGCCATGGAACGGGCACAGT---------------------------------------
--CCGCCT
TATGTCGGTATTGCCTTGGCAGCTGGCTTTGCTGTAGCTAGTCTTACCCTTTCTTTTGCA
CACATTTCAGGCGCTCATCTAAACCCTTGCGTCACGTTAGCGCTTTTAATGTCCAGGAAA
ATTTCTCCGATCAAAGGCCTTTTGTATGTCACTGCACAAGGGGGAGGAGCCATTGCGGGT
TCTGCCTTATTATATGGGGTGACGTCCACGGGTTTACAAGGA------------------
------------------------------TGGCTTGGAGCCACAGTCCCCGCACCTGGG
---------TTATCCGAATGGCAGGCATTAGCCATCGAATTGAGTATGACCTTTGTGGTG
GTTTTGACCCATCTAACT---GCCTTGGACCCCGTCCGG---------------------
---GGTTATTTGGTGAACACAAACATGGGACCCGGACCTTTGACTGTAGGCTTGGCCTAT
GTGACATGCTCCCTAGTGGCGTTGCCCTCGACTGGAGCCTCTTTTAACCCAATGCGGTCC
TTAGGACCTGCCTTTGTTATGAAC---------AAATGGGATAGCCATTGGATTTACTGG

ATAGGACCCATAATTGGAGGATGTTTGGCAGGATTGCTTTACGAATTCGTGTTCAATCCG
---TATCGCGGTACGAAGAAA---------CGCAAGGATTCCATC---GACGGC------
------------------------GAC------------TCGCTGAGCATGCACAGTGAT
GAA------GAC------TTCGAT---GAAGAA---AAAAACGGACGTCAATTAGCGATA
---GCTAATTTC
CAGAATGCC---------CGG---------------------------------------
------ACTCAGCACTATGATCAA---------------TATCGCCCT---GCTTTGCGC
------------TCCAATCCCGTTTCTCCTACA------------------GGT------
------CACATGGCC------------------------TCTAACGCTGCACTGGCGTCA
------AGAAGCAGC---
AGACCTGATAGA---------------GGAGATCCA---ATTTATGATGGCTCTAAATCG
CTC---TAT---TGC------------------AAAACGCCTCAAGTTGCACCTAAA---

------------------------------GCAATTTTGAATCGCTCCCAATCTGTCTAC
ACTAAA---CCACCGTTACCGCCCGCG------------AATCCCTCTCCTCCTCTGCAG
TCAGGGCTGGCACCCGCCCAGAGT---ATGTATTTGGGCAAAGTTAGCAGTCAG------

------ATGAGCCAACAGCACCATCCGCTATCAGTGCCTTCCAGGACTGGAAGAATTTAT
TGTTTTAAGGGGTCCATTTTTCATAATTTACGATGTCTTGTTCCTAAATACAGGAATGAA
AGCGTTTACGGC--ATGCAC
CGCGAG---CGTGAG---
------------------TCACCGTATGGT------------------------------
--GCTAAG------
---------ATCACCGACAATTAC------GGC------CCAATTAGTCCGCCCAGT---
---------------------------------AATTACGCAGACCAAAAATCCAGCGAA
GAAAATTATCTTAACAGTTTCCCGGCCTCAAAGAGCGGCAATCCGGCCAGCCATATGTAT
AAACCGCCCATGGACAGGGAGTCCTATGAACGCAGTGAACCCATGTACACTCATAAGCCC
AGAGAACAAATGCCCTACGGCGATACGAATGGAAATGGG---------------------
---------------------------AGCCGA---------------------------
---ATGGAATCTGTGTATGGCATGCAAGGAGCCGGGCATGGAATTACCCAAGAATCC---
------ATCTACGCCACTAAG---------------------------------AAGTTG
------------GAACCGAATGCCAATAATCCTCCTCAAATG---------------GGA
---CATTCTGATCCTAACAATTATGCT------ATG---TACCCCCGC------------
--AGCGCTTCGCTA
ATCCGTAACGAT--TCGGTT
TACGGAGTTGTCCCACCGATTCGGCGCCCGACTCCA------------------------
--------CCCATGCAGACGCAAATGATGAACAGTGGCAGCAAC---------------A
TG------CCCGTCAAC------TCGCCTTCACCAGCTGATACTTCC-------------
--------------AAAGATTCAGCCTACGGGTCTCAGTACGGTGGTCCGATGGATCATG
AATTACTGAATAGACCGCCATCTCAC----------------------------------
--------------GGCAGTTATACCACCAATATGAACAAAATGCGAGGTGACTTG---A
TGTCG---CCTAACTCTCAATCA-------------------------------------
-----AGGTCAAATCCACAAACA-------------------------------------
-----CCTACGTCTGCGACGCCGTAT---CATTCTCCT----------------------
---CAGTAC-
>JAGJRP010000055_Lepidurus_couesii_Bib
ATGGCTAATGTGAGC---
------------------------------------TTGGCGCCG---AATTCCGCGTCC

ATGGAAACGTTGTTACAAACTTTAATGGATAGGCTCGAATCGCAGCGAACAGAGCGT---

CCTAAATTGGCGCGAGTGCCTATGCAAGCCGAAATTCGGAGCCTG---GATTTTTGGCGT
GCCGTCGTCGCCGAGTGTCTCGCTACTTTTTTCTACGTCTTCCTCATCTGCGGCTCTTTC
GTGCCATGGAACGGGCACAGT---------------------------------------
--CCGCCT
TATGTCGGTATCGCCTTGGCAGCTGGCTTTGCTGTAGCTAGTCTTACCCTTTCTTTTGCA
CACATTTCAGGCGCTCATCTGAACCCTTGCGTCACGTTAGCGCTTTTAATGTCCAGGAAA
ATTTCCCCGATCAAAGGCCTTTTGTATGTCACCGCACAAGGGGGAGGAGCCATTGCGGGT
TCTGCCTTATTATATGGGGTGACGTCCACGGGGTTGCAAGGA------------------
------------------------------TGGCTTGGAGCCACAGTCCCCGCACCTGGG
---------TTATCCGAATGGCAGGCATTAGCCATCGAATTGAGTATGACCTTTGTGGTC
GTTTTGACCCATCTAACT---GCCTTGGACCCCGTCCGG---------------------
---GGTTATTTGGTGAACACAAACATGGGACCCGGACCTTTGACTGTAGGCTTGGCCTAT
GTGACATGCTCCCTAGTGGCGTTGCCCTCGACTGGAGCCTCTTTTAACCCAATGCGGTCC
TTAGGACCTGCCTTTGTTATGAAC---------AAATGGGATAGCCATTGGATTTACTGG
ATAGGACCCATCATTGGAGGATGTTTGGCAGGATTGCTCTACGAATTCGTGTTCAATCCG
---TATCGCGGTACGAAGAAA---------CGCAAGGATTCCATC---GACGGC------
------------------------GAC------------TCGCTGAGCATGCACAGTGAT
GAA------GAC------TTCGAT---GAAGAG---AAAAACGGACGTCAATTAGCGATA
---GCTAATTTC
CAGAATGCC---------CGG---------------------------------------
------ACTCAGCACTATGATCAA---------------TATCGCCCT---GCTCTGCGC
------------TCCAATCCAGTTTCTCCTACA------------------GGT------
------CACATGGCC------------------------TCTAACGCTGCACTGGCGTCA
------AGAAGCAGC---
AGACCTGATAGA---------------GGAGATCCA---ATTTATGATGGCTCTAAATCG
CTC---TAT---TGC------------------AAAACGCCTCAAGTTGCACCTAAA---

------------------------------GCAATTTTGAATCGTTCCCAATCTGTCTAC
TCTAAA---CCACCGTTACCGCCCGCG------------AATCCCTCCCCTCCTCTGCAG
TCAGGGCTGGCACCCGCCCAGAGT---ATGTATTTGGGCAAAGTTAGCAGTCAG------

------ATGAGCCAACAGCACCATCCGCTATCAGTGCCTTCCAGGACTGGGAGAATTTAT
TGTTTTAAGGGGTCCATTTTTCATGATTTACGACGTCTTGTTCCTAAATACAGGAATGAA
AGCGTTTACGGC--ATGCAC
CGCGAG---CGTGAG---
------------------TCACCGTATGGT------------------------------
--GCTAAG------
---------ATCACCGACAATTAC------GGC------CCAATTAGTCCGCCCAGT---
---------------------------------AATTACGCAGACCAAAAATCCAGCGAA
GAAAATTATCTCAACAGTTTCCCGGCCTCGAAGAACGGCAACCCGGCCAGCCATATGTAT
AAACCGCACATGGACAGGGAGTCCTATGAACGCAGTGAACCCATGTACACTCATAAGCCC
AGAGAACCAATGCCCTACGGCGATACGAATGGAAATGGG---------------------
---------------------------AGCCGA---------------------------
---ATGGAATCTGTGTATGGCATGCAAGGAGCCGGGCATGGAATTACCCAAGAATCC---
------ATCTACGCCACTAAG---------------------------------AAGTTG

------------GAACCGAATGCCAATAATCCTCCTCAAATG---------------GGA
---CATTCTGATCCTAGCAATTACGCT------ATG---TACCCCCGC------------
--AGCGCTTCGCTA
ATCCGTAACGAT--TCGGTT
TACGGAGTTGTCCCACCGATTCGGCGCCCGACTCCA------------------------
--------CCCATGCAGACGCAAATGATGAACAGTGGCAGCAAC---------------A
TG------CCCGTCAAC------TCGCCCTCACCAGCTGATACTTCC-------------
--------------AAAGATTCAGCCTACGGGTCTCAGTACGGTGGTCCGATGGATCATG
AATTACTGAATAGACCGCCATCTCAC----------------------------------
--------------GGCAGTTATACCACCAATATGAACAAAATGCGAGGTGACTTA---A
TGTCG---CCCAACTCTCAATCA-------------------------------------
-----AGGTCAAATCCACAAACA-------------------------------------
-----CCGACGTCTGCGACGCCGTAT---CACTCTCCT----------------------
---CAGTAC-
>JAKZAN010000006_Lepidurus_packardi_Bib
ATGGCTAATATGAGC---
------------------------------------TTGGCGCCA---AACTCCGCGTCG
ATGGAAACGTTGTTGCAAACTTTGATGGACAGGCTCGAATCGCAGCGAACAGAGCGT---

CCTAAATTGGCGCGAGTGCCTATGCAAGCCGAAATTCGGGGCCTG---GATTTTTGGCGT
GCCGTCGTCGCCGAATGTCTCGCTACTTTTTTCTACGTCTTCCTTATCTGCGGCTCTTTC
GTACCATGGAACGGGCACAGT---------------------------------------
--CCGCCT
TATGTCGGTATTGCCTTGGCAGCTGGCTTTGCTGTAGCTAGTCTTACCCTTTCTTTTGCA
CACATCTCAGGCGCTCATCTGAACCCTTGTGTCACGTTAGCGCTTTTAATGTCCAGGAAA
ATTTCTCCGATCAAAGGCCTTTTGTATGTCACTGCACAAGGGGGAGGAGCCATTGCGGGT
TCTGCCTTATTATATGGGGTGACGTCCACGGGCTTGCAAGGA------------------
------------------------------TGGCTTGGAGCCACAGTCCCCGCACCTGGA
---------TTATCCGAATGGCAGGCATTAGCCATCGAATTGAGTATGACCTTTGTGGTG
GTTTTGACCCATCTAACT---GCCTTGGACCCCGTCCGG---------------------
---GGTTATTTGGTGAACACAAACATGGGACCCGGACCTTTGACTGTAGGCTTGGCCTAT
GTGACTTGCTCTCTAGTGGCGTTGCCCTCGACTGGAGCCTCCTTTAACCCAATGCGGTCC
TTAGGACCTGCCTTTGTGATGAAC---------AAATGGGATAGCCATTGGATTTACTGG
ATTGGACCCATCATTGGAGGATGTTTGGCAGGATTGCTCTACGAATTCGTGTTCAATCCG
---TATCGCGGTACGAAGAAA---------CGCAAGGATTCCATC---GACGGC------
------------------------GAC------------TCGCTGAGCATGCACAGTGAT
GAA------GAC------TTCGAT---GAAGAG---AAAAACGGACGTCAATTAGCGATA
---GCTAATTTC
CAGAATGCC---------CGG---------------------------------------
------ACTCAGCACTATGATCAA---------------TATCGCCCT---GCTCTGCGC
------------TCCAATCCTGTTTCTCCTACA---------------------------

---------------------------------------ATTTATGATGGCTCTAAATCG
CTC---TAT---TGC------------------AAAACGCCTCAAGTTGCACCTAAA---

------------------------------GCAATTTTGAATCGCTCCCAATCTGTCTAC

ACTAAA---CCACCGTTACCGCCCGCG------------AATCCCTCCCCTTCTCTGCAA
TCAGGATTGGCACCCGCCCAGAGT---ATGTATTTGGGAAAAGTTAGCAGTCAG------

---------AGCCAACAGCACCATCCGCTATCAATGCCTTCGAGGACTGGGAGAATTTAT
TGTTTAAATGGGTCCAGTTTTCATGATTTACGACGTCTTGTTCCTGAATACAGGAATGAA
AGCGTTTATGGC--ATGCAC
CGCGAG---CGTGAG---
------------------TCACCGTATGGT------------------------------
--GCTAAG------
---------ATCACCGATAATTAC------GGC------CCAATTAGTCCGCCCAGT---
---------------------------------AATTACGCAGACCAAAAATCCAACGAA
GAAAATTATCTCAACAGTTTCCCGGCCTCGAAGAGCGGCATTCCGGCCAGCCATATGTAC
AAACCCCCCACGGACAGGGAGTCCTATGAACGCAGTGAACCCATGTACACTCATAAGCCC
AGAGAACAAGCGCCCTACGGCGATACGAATGGAAATGGG---------------------
---------------------------AGCCGA---------------------------
---ATGGAATCTGTGTATGGCATGCAAGGAGCCGGGCATGGAATTACCCAAGAATCC---
------ATCTATGCCACAAAG---------------------------------AAGTTG
------------GAACCGAATGCCAATAGTCCTCCTCAAATG---------------GGA
---CATTCTGATCCTAGCAATTATGCC------ATG---TACCCCGC-------------
--AGCGCTTCGCTA
ATCCGTAACGAT--TCGGTT
TACGGAGTTGTCCCACCGATTCGGCGCCCGACTCCA------------------------
--------CCCATGCAGACACAAATGATGAACAATGGCAGCAAC---------------A
TG------CCCGTCAAC------TCGCCTTCACCAGCTGATACTTCC-------------
--------------AAAGATTCAGCCTACGGGTCTCAGTACGGTGGTCCAATGGATCATG
AATTACTGAATAGACCGCCATCTCAT----------------------------------
--------------GGCAGTTATACCACCAATATGAACAAAATGCGAGGTGACTTA---A
TGTCG---CCCAACTCTCAATCA-------------------------------------
-----AGGTCAAATCCACAAACA-------------------------------------
-----CCGACGTCTGCGACGCCGTAT---CATTCTCCA----------------------
---CAGTAC-
>RJJB01000010_Lepidurus_apus_Bib
ATGGCTAATGTGAGC---
------------------------------------TTGGCGCCG---AATTCCGCGTCG
ATTGAAGCGTTGTTGCAAACTTTGATGGACAGGCTCGAATCGCAGCGAACAGAGCGT---

CCTAAATTGGCGCGAGTGCCTATGCAAGCCGAGATTCGGGGCCTG---GATTTTTGGCGT
GCCGTCGTCGCCGAATGTCTCGCTACTTTTTTCTACGTCTTCCTTATCTGCGGCTCTTTC
GTGCCATGGAACGGGCACAGT---------------------------------------
--CCGCCT
TATGTCGGTATTGCCTTGGCAGCTGGCTTCGCTGTAGCTAGTCTTACCCTTTCTTTTGCA
CACATCTCAGGCGCTCATCTCAACCCGTGCGTCACTTTAGCGCTTTTAATGTCCAGGAAA
ATTTCTCCGATCAAAGCCCTTTTGTATGTCACAGCACAGGGGGGAGGAGCCATTGCGGGT
TCTGCCTTATTATATGGGGTGACGTCCTCGGGTTTGCAAGGA------------------
------------------------------TGGCTTGGAGCCACGATACCAGCACCTGGC
---------TTATCCGAATGGCAGGCATTAGCCATCGAATTGAGTATGACATTTGTGGTA
GTTTTAACCCACCTAACT---GCCTTGGACCCCGTCCGG---------------------

```
---GGTTATTTGGTGAACACAAACATGGGACCTGGACCTTTGACTGTGGGGCTGGCCTAT
GTGACTTGCTCCTTAGTGGCGTTGCCCTCGACTGGAGCCTCCTTTAACCCAATGCGATCC
TTAGGACCTGCCTTTGTTATGAAC---------AAATGGATAGTCATTGGATTTATTGG
ATTGGACCCATCATAGGAGGATGTTTGGCAGGATTGCTCTACGAGTTCGTGTTCAATCCT
---TATCGCGGTACGAAGAAA---------CGCAAGGATTCCATC---GATGGA------
------------------------GAC------------TCGCTGAGTATGCACAGTGAT
GAA------GAC------TTCGAT---GAAGAG---AAAAACGGACGTCAATTAGCGATA
---------------------------------------------------GCTAATTTC
CAGAATGCC---------CGG---------------------------------------
------ACTCAGCACTATGATCAG---------------TATCGCCCT---GCGTTGCGC
------------TCCAATCCAGTTTCTCCTACA------------------GGT------
------CACATGGCC------------------------TCTAACGCTGCACTGGCGTCA
------AGAGGCAGC---------------------------------------------
AGACCTGATAGA---------------GGGGATCCA---ATTTATGATGGCTCTAAATCG
CTC---TAT---TGC------------------AAAACGCCTCAAGTTGCACCTAAA---
------------------------------------------------------------
------------------------------GCAATTTTGAATCGCTCCCAATCTGTCTAC
ACTAAA---CCACCGTTACCACCCGCG------------AATCCCTCCCCTTCTTTGCAA
TCAGGGCTAGCACCTGCCCAGAGT---ATGTATTTGGGCAAAGTTAGCAGTCAG------
------------------------------------------------------------
---------AGCCAACAGCACCATCCGCTATCAGTGCCTTCCAGGACTGGGAGAATTTAT
TGTTTTAATGTGCCCATTTTTCATGATTTACGACGTCTTGTTTCTAAATACAGGAATGAA
AGCGTTTACGGC------------------------------------------ATGCAC
CGCGAG---CGTGAG---------------------------------------------
------------------TCACCGTATGGT------------------------------
------------------------------------------------GCAAAG------
---------ATCACCGACAACTAC------GGT------CCAATTAGTCCGCCCAGT---
---------------------------------AATTACGCAGACCAAAAATCCAGCGAA
GAAAATTATCTCAACAGTTTTCCAGCCTCGAAGAGTGGCATTCCGGCCAGCCATATGTAT
AAACCGCCCACGGACAGGGAGTCCTATGAACGCAGTGAACCCATGTACACTCATAAGCCC
AGAGAACAAATGCCCTACGGCGACACGAATGGAAATGGG---------------------
---------------------------AGCCGA---------------------------
---ATGGAATCAGTCTATGGTATGCAAGGAGCCGGACATGGAATTACCCAAGAATCC---
------ATCTACGCCACTAAG---------------------------------AAGTTG
------------GAACTGAATGCCAATAATCCTCCTCAAATG---------------GGA
---CATTCTGATCCTGGCAATTATGCC------ATG---TACCCCCGC------------
------------------------------------------------AGCGCTTCGCTA
ATCCGTAACGAT------------------------------------------TCGGTC
TACGGAGTTGTCCCACCGATTCGGCGCCCGACTCCA------------------------
--------CCCATGCAAACGCAAATGATGAACAGTGGCACCAAC---------------A
TG------CCCGTCAAC------TCGCCCTCACCAGCTGATACTTCT-------------
--------------AAAGATTCTGCCTATGGGTCTCAGTACGGTGGTCCGATGGATCATG
AGTTACTGAATAGACCGCCTTCTCAT----------------------------------
--------------GGCAGTTATACCACCAATATGAACAAAATGCGAGGTGATTTA---A
TGTCG---CCCAACTCTCAATCA-------------------------------------
-----CGGTCAAATCCACAAACA-------------------------------------
-----CCGACGTCTGCGACGCCGTAT---CATTCTCCT----------------------
-----------------------------------------------CAGTAC-
```

>GEHY01010105_Triops_newberryi_Bib
ATGGCTGGTGTGAGT---
------------------------------------TTAACCACG---AATGTGACTTCG
ATGGAATCCTTATTACAGACCTTAATGGACCGCTTGGAATCGCAACGGACAGAGCGG---

CCGAAACTCTCGCGTGTGCCAATGCAAGCCGAAATACGCGGTCTG---GATTTCTGGCGC
GCAGTTGTCGCAGAATGTCTGGCGACATTTTTTTACGTCTTTCTCATTTGCGGATCCTTT
GTACCGTGGAATGGACATAGT---------------------------------------
--CCCCCA
TACGTCGGTATTGCCTTAGCTGCTGGCTTTGCTGTCGCTAGTCTTTCCCTGTCCTTCGCA
CACATATCAGGTGCTCATTTGAATCCATGCGTCACATTAGCGCTGTTAATGTCCAGGAAA
ATTTCGCCTATCAAAGGACTTTTGTATGTCACAGCCCAAGGCGGAGGAGCAATTGCCGGA
GCTGCCTTATTATATGGGGTGACGTCTTCAGGGCTGCAAGGA------------------
------------------------------TGGCTCGGAGCGACGGTCCCGGCTCCTGGT
---------TTATCCGAATGGCAAGCGTTGGCCATTGAATTGAGTTTGACCTTTGTGGTG
GTTTTAACCCATTTGACG---GCGTTGGATCCTATCCGA---------------------
---GGTTATTTGGTGAACACAAACTTAGGACCGGGACCTTTGACCGTGGGCTTAGCATAT
GCGACCTGCTCTTTAGTGGCTTTGCCTTCGACTGGCGCCTCTTTCAATCCTGTAAGGTCC
CTAGGACCCGCATTTGTTATGAAC---------AAATGGGATAGTCACTGGGTTTACTGG
ATTGGACCGATCATTGGCGGATGTTTGGCAGGACTGCTTTACGAGTTTGTGTTTAATCCT
---TACCGGGGGACGAAGAAG---------CGCAAAGATTCCATC---GACGGA------
------------------------GAC------------TCGTTAAGTATGCACAGCGAT
GAA------GAT------TACGAC---GAAGAG---AAGAACGGACGCCAGATGGCCATA
---ACGAATTTT
CAGAATGCG---------CGG---------------------------------------
------ACTCAGCATTACGACCAG---------------TATCGTCCC---ACGTTGCGT
------------TCGAATCCGGTTTCTCCTACA------------------GGT------
------CACATGGCT------------------------TCAAATGCTGCATTAGCATCA
------AGAGGCAGT---
CGACCTGATAGA---------------GCAGATCCC---ATTTACGATGGCTCAAAATCC
TTA---TAC---TGC------------------AAAACGCCTCAAGTTGTGCCCAAA---

------------------------------GCTCCGTTGCATCGCTCTCAATCTGTCTAC
GCTAAA---CCACCGTTGCCACCCGCC------------AATCCTTCGCCGCCTCTGCAG
TCAGGTCTGGCACCCGCCCAGAGT---CTCTATTTAGGCAAAATTAGTAGCAGC------

---CAGAACCATCAACCCCATCATCCTCTGTCAGTGCCTTCCAGGAACGGC---ATT---
--CATCGCAATGAA
AGCGTGTATGGA--ATGCAC
CGTGAG---CGTGAG---
------------------TCACCGTACGGA------------------------------
--GCCAAG------
---------ATTACCGACAATTAC------GGC------CCAATTAGTCCGCCCAGC---
---------------------------------AGTTACGCTGACCAGAAATCCGCCGAA
GAAAACTATTTGAACAGTTTCCCTGCT---AAGAACGGGAATCCAAGTAGCAACATGTAT
AAACCGACACCGGAGCGAGAGTCATTCGAACGTAATGATTCTGTCTATACGCACAAACCT
CGCGAAGCAATGCAGTACAATGATAATAACGGCAATAGCAAT------------------

------------------------GCCCCACGG---------------------------
---TTAGAGTCCGTATATGGCATGCAAGGAACTGGACACGCAATTACTCAAGAATCT---
------ATTTATGCTACGAAA---------------------------------AAATTG
------------GAGCAAAATTCGAATAATCATCCCAACATG---------------AGT
AATTCGGGTGATAACGGTAATTATGGC------ATG---TATCCCCGTAGTAAT------
--GGTGTTCCGGTG
GTCCGTAATGAC--TCGGTT
TACGGGGTTGTACCACCGATTCGACGCCCAACTCCG------------------------
--------CCAGTGCAGTCCCAAATGATGAACAGT---AGCAAT---------------T
TG------CCCGTGAAT------TCGCCGTCACCTGCTGATACGTCA-------------
--------------AAAGATTCTGCCTACGGATCCCAGTACGGTGGTCCAATGGATCACG
AGTTGTTAAATCGACCTCCTTCCCAT----------------------------------
--------------GGCAGTTATTCTATCAATATGAACAAAATGAGGGGAGATCTA---A
TTTCG---CCCAATTCTCAATCA-------------------------------------
-----AGGTCCAACCCGCATACG-------------------------------------
-----CCGACGTCTACGACGCCGTAT---CATTCTCCT----------------------
---CAATAT-
>JAGQDJ010000608_Triops_longicaudatus_Bib
ATGGCTGGTGTGAGT---
------------------------------------TTAACTACG---AATGTGACTTCG
ATGGAATCTTTATTGCAGACCTTAATGGACCGCTTGGAATCGCAACGGACAGAGCGG---

CCGAAACTCTCGCGTGTGCCAATGCAAGCCGAAATACGCGGTCTG---GATTTCTGGCGC
GCAGTTGTCGCAGAATGTCTGGCGACGTTTTTTTACGTCTTTCTTATTTGCGGATCCTTT
GTACCGTGGAATGGACATAGT---------------------------------------
--CCCCCA
TACGTCGGTATTGCCTTAGCTGCTGGCTTTGCTGTCGCTAGTCTTTCCCTGTCCTTCGCA
CACATATCAGGTGCTCATTTGAATCCATGCGTCACATTAGCGCTCTTAATGTCCAGGAAA
ATTTCGCCTATCAAAGGACTTTTGTATGTCACAGCCCAAAGCGGAGGGGCAATTGCCGGA
GCTGCCTTATTATATGGGGTGACGTCTTCAGGGCTGCAAGGA------------------
------------------------------TGGCTCGGAGCGACGGTCCCGGCTCCCGGT
---------TTGTCCGAATGGCAAGCATTGGCCATTGAATTGAGTTTGACCTTTGTGGTG
GTTTTAACCCATTTGACG---GCGTTGGATCCCATCCGG---------------------
---GGTTATTTGGTGAACACAAACTTAGGACCGGGACCTTTGACCGTGGGCTTAGCATAT
GCGACCTGCTCTTTAGTGGCTTTGCCTTCGACTGGCGCCTCTTTCAATCCTGTAAGGTCC
CTAGGACCCGCATTTGTTATGAAC---------AAATGGATAGTCACTGGGTTTACTGG
ATTGGACCGATCATTGGCGGATGTTTGGCAGGACTGCTGTACGAGTTTGTGTTTAATCCC
---TACCGGGGTACGAAGAAG---------CGCAAAGATTCCATC---GACGGA------
------------------------GAC------------TCGTTAAGTATGCACAGCGAT
GAA------GAT------TACGAT---GAAGAG---AAGAACGGACGCCACATGGCCATA
---ACGAATTTT
CAGAATGCG---------CGG---------------------------------------
------ACTCAGCATTATGACCAG---------------TATCGCCCC---GCGTTGCGT
------------TCGAATCCGGTTTCTCCTACA------------------GGT------
------CACATGGCT------------------------TCAAATGCTGCATTAGCATCA
------AGAGGCAGT---
CGACCTGATAGA---------------GCAGATCCC---ATTTACGATGGCTCAAAATCC

TTG---TAC---TGC------------------AAAACACCTCAAGTTGTGCCCAAA---

------------------------------GCTCCGTTGAATCGCTCTCAATCTGTCTAC
GCGAAA---CCACCGTTGCCACCCGCC------------AATCCTTCGCCGCCTCTGCAG
TCAGGTCTAGCACCCGCCCAAAGT---CTCTATTTAGGCAAAATTAGTAGCAGC------

---CAGAACCATCAACCCCACCATCCTCTGTCAGTGCCTTCCAGGAACGGC---ATT---
--CATCGGAATGAA
AGCATGTATGGA--ATGCAC
CGTGAG---CGTGAG---
------------------TCACCGTATGGA------------------------------
--GCCAAG------
---------ATTACCGACAATTAC------GGC------CCAATTAGTCCGCCCAGC---
---------------------------------AGTTACGCTGACCAGAAATCCGCTGAA
GAAAACTACTTGAACAGTTTCCCTGCC---AAGAACGGCAATCCAAGTAGCAACATGTAT
AAACCGACACCGGAGAGAGAGTCATTCGAACGCAAAGATTCTGTCTATACGCACAAACCT
CGCGAAGCAATGCAGTACAATGATAATAACGGCAATAGCAAT------------------
------------------------GCCCCGCGG---------------------------
---TTAGAGTCCGTGTATGGTATGCAAGGAACTGGACACGCAATTACTCAAGAATCT---
------ATTTATGCTACGAAA---------------------------------AAATTG
------------GAGCAAAATTCCAATAATCATCCCAACATA---------------AGT
AACTCGGGTGATAACGGTAATTATGGC------ATG---TATCCCCGTAGTAAT------
--GGTGTCCCGGTG
GTCCGTAATGAC--TCGGTT
TACGGGGTTGTACCACCGATTCGACGCCCAACTCCG------------------------
--------CCAGTGCAGTCCCAAATGATGAACAGT---AGCAAT---------------T
TG------CCCGTGAAT------TCGCCGTCACCTGCTGATACGTCA-------------
--------------AAAGATTCTGCCTACGGGTCGCAGTATGGTGGTCCAATGGATCACG
AGTTGTTAAATCGGCCTCCTTCCCAT----------------------------------
--------------GGCAGCTATTCTATCAATATGAACAAAATGAGGGGAGATCTA---A
TTTCG---CCCAATTCTCAATCA---------------------------------------
-----AGGTCCAACCCGCATACG-------------------------------------
-----CCGACGTCCACGACGCCGTAT---CATTCTCCT----------------------
---CAATAT-
>GCAT01002935_01010270_Triops_cancriformis_Bib
ATGGCTGGTGTGAGT---
------------------------------------TTAACCCCG---AATGTGGCTTCG
ATGGAATCCTTATTACAGACCTTAATGGACCGCTTGGAATCGCAACGCACAGAGCGC---

CCGAAACTGTCGCGTGTGCCAATGCAAGCCGAAATACGCGGTCTG---GACTTCTGGCGT
GCAGTTGTCGCGGAATGTCTGGCGACATTTTTTTACGTCTTTCTTATTTGCGGATCCTTT
GTACCGTGGAATGGACACAGT---------------------------------------
--CCTCCA
TACGTCGGCATTGCTTTGGCCGCTGGCTTTGCCGTCGCTAGTCTTTCCCTGTCCTTCGCA
CACATATCAGGTGCCAAAATTTCGCCC---------------------------------
---------ATCAAAGGACTTTTGTATGTCACAGCCCAAGGCGGAGGAGCAATTGCCGGA
GCTGCCTTATTATATGGGGTGACGTCATCAGGGCTGCAAGGA------------------

------------------------------TGGCTCGGAGCGACGGTCCCGGCACCGGGC
---------TTATCAGAATGGCAAGCATTGGCCATTGAATTGAGTTTGACCTTTGTGGTG
GTTTTAACCCATTTGACG---GCTTTGGATCCTATCCGG---------------------
---GGCTATTTGGTGAATACCAACTTAGGACCGGGACCTTTGACCGTGGGTTTAGCGTAT
GCGACCTGCTCTTTAGTGGCTTTGCCTTCTACTGGTGCCTCTTTCAATCCTGTAAGATCC
CTAGGTCCCGCGTTTGTTATGAAC---------AAATGGGACAGTCATTGGGTGTATTGG
ATTGGACCGATCATTGGTGGATGTTTGGCTGGATTACTGTACGAGTTTGTGTTTAATCCG
---TACCGCGGACGAAGAAA----------CGCAAAGATTCCATT---GATGGA------
------------------------GAT------------TCGCTAAGTATGCACAGCGAT
GAA------GAT------TACGAC---GAAGAG---AAAAACGGACGCCAGATGGCCATA
---ACGAATTTC
CAGAATGCG---------CGG---------------------------------------
------ACGCAGCATTACGACCAG---------------TATCGCCCT---GCGTTGCGC
------------TCAAATCCGGTTTCTCCTGCA------------------GGT------
------CACATGGCA------------------------TCGAATGCTGCACTAGCATCA
------AGAGGCAGT---
CGACCTGACAGA---------------GCAGATCCA---ATTTATGATGGCTCGAAATCC
TTG---TAC---AGC------------------AAAACGCCACAAGTTGTACCCAAA---

------------------------------GCTCCGTTGAATCGTTCTCAATCCGTCTAC
GCTAAA---CCACCGTTACCACCCGCC------------AATCCTTCGCCGCCTCTGCAG
TCAGGCCTGGCACCCGCCCAGAGT---CTCTATTTTGGCAAAATTAGTAGCAGC------

---CAGAACCACCAGCCCCATCATCCTTTGTCAGTGCCTTCCAGGAACGGT---ATT---
--CATCGCAATGAA
AGCGTGTATGGA--ATGCAC
CGCGAG---CGTGAG---
------------------TCACCGTATGGA------------------------------
--GCCAAG------
---------ATTACCGACAATTAC------GGA------CCAATTAGTCCGCCCAGC---
---------------------------------ACTTACGCTGACCAGAAATCCGCCGAA
GAAAACTACTTGAACAGTTTTCCTGCC---AAGAACGGCAACCCGGGTAGCAACATGTAC
AAACCGCCTCCGGAGCGAGAGTCATTTGAACGCAGTGATTCTGTCTACACGCACAAACCT
CGCGACACAATGCAATACAACGATAGTAACGGCAATAACAGT------------------
------------------------GGCCCTCGG---------------------------
---CTGGAATCCGTGTACGGTATGCAAGGACCTGGACATGCAATTACTCAAGAATCT---
------ATTTATGCCACAAAA---------------------------------AAGTTG
------------GAACAGCATTCCAACCACCATCCTAACATG---------------AGC
AACTCGGGGGATAACGGTAATTATGCC------ATG---TATCCTCGTAGTAGT------
--GGTGCCTCGGTG
ATCCGTAATGAC--TCGGTT
TACGGAGTTGTACCACCGATTCGACGCCCAACTCCG------------------------
--------CCAGTGCAGTCCCAAATGATGAACAGT---AGCAAT---------------T
TG------CCCGTCAAT------TCGCCATCGCCTGCAGATACGTCG-------------
--------------AAAGATTCTGCCTACGGGTCGCAATACGGGGCCCCAATGGATCACG
AGTTGTTAAATCGACCTCCGTCCCAT----------------------------------
--------------GGCAGTTATTCTATCAATATGAACAAAATGAGAGGAGATCTA---A
TTTCG---CCCAATTCTCAATCA-------------------------------------

-----AGATCCAACCCGCATACA-------------------------------------
-----CCGACATCTACAACGCCGTAT---CATTCTCCT----------------------
---CAATAC-
>JAKZAO010000014_Branchinecta_lindahli_Bib
ATGGGTCGCTCAAGTGCAGCAGTTCCC---------------------------------
------------------------------TTA---AACCAAAACCTGTCATCAGCAACA
GTTGACACCCTGCTTTTGACTTTGTTGAGGAAACTTGACAAAGAAAAGGAGGAA------

CCT---TGGGCGAAAGTTCAATTCCAAGCCGAAGTGAGAACTTTG---GAATTTTGGAGA
TCTGTAGTTGCCGAATGTGTTGGAACATTTTTTCTTGTGTTTCTAACCTGTGGAGCATTT
GTGCCGTGGGCTGGCATACT--
--CCCCCG
CCTATTGGAATTGCCCTGACTGTTGGATTTGGGGTTGTGATGTTAACACACTGCTTTGCG
CTCATTTCAGGTGCCAATTTTAATCCTGTGGTGACGCTTGCGCTTGTGGTCAAAAGAAGC
ATGTCACCGTTTCGCGGCGCTCTCTATTTCACTGCCCAGGTCGGAGGATCCATCGCCGGA
TCAGCTTTGCTCTATGGGGTGACGTCATCCGGATCTCAAGGG------------------
------------------------------AATCTGGGAGTAACTACACTTGCTGCCGGC
---------TTGAAGGACTGGCAAGGCTTTGCCATCGAACTCTGCTTCAGTTTCATCATC
GTGCTGACTTACCTTTGT---ACCCTCCATCCAGAGCAA---------------------
---AAGCGGACAGTATCTACATCTATGGGAACAGGTCCTATCACTGTGGGTTTTGCCTAT
TTGGCTTGCTCGCTCGTGGGGCTGCCGTCTACTGGAGCTTCATTCAACCCCGTTCGGTCG
TTGGGTCCAGCTTTTGTCATGAAC---------AAATGGGATAGCCACTGGATTTACTGG
GTGGGACCCCTAATTGGAGGAGCCTTCGGAGGTCTTATCCACGAGTTTGTCTTCAACGCC
---TATCGGGAAGGAAGCAGC---------AGGAAGTGCAACGGC---GACGGA------
------------------------GAT------------TCTTCGAGCATCCACAGCGAT
GAA------GAA---TGCTACGAG---GAGGGG---CCAGCTGGG---CCCCCTGTCCGA
--GGTCCAGTTCCG
GGAGCTCTTGCTGTC---CGA---------------------------------------
------AGCCAGCATTACGACACG---------------TATAAGCCG---ATCGCCATA

---------------------------------------GGTATGGAAGGCATGTGCAAA
------TCAACAAGG---
AACCTTGACCAG---------------ACCGAAGGG---ATCTACTCTGGGACCCGCTCC
CTC---TAC---TGC------------------CGATCGCCCGGTCCTGCTCAGCGA---

------------------------------AACATCCTCAACAGGTCCCAGTCCGTCTAC
GCCAAG---AATCCGGCGATCGGCGGG------------CAAAACTCC------------
CGC---ATCCATCCAGCTGCCAGC---ATAACGACCACAAAACCCGAAGGATTT------
---------------------GAAGCAATC------------------------------

--AGCCGCCAAGAG
AGCATTTACTCG---AGCGGCAGATGTCAG---------------------CCGCAGCCC
ATAAGG---TCAGGG---
------------------AGTATCTACGCT------------------------------
------------------------------CCTCGAAGCCAA------ATTCAA------
---------AAAGAGACCGTCTTC------------------------------------
------------------------------------TACTCCAAGCAGCAG------AAT

GAAAATGTTTACGCAACAAGGCCACCCCTTCCTTCAGAC---CCCGGC------GTGTAC
---CCCCCT---CGCCATGAG---------------------------------------
------------------ACACCCAACTCCTTC---------------------------

---GCTCCCCAGGGATACCAGGCCCCCCTTCCCCCCACGCCAGTCAACGAGGACCAG---
--AAGCTT
------------GAAGAAGAAAACATGCGA------AATGCA------------------
---GAAAGCACAAAGGGCGACTTT---------GAC---TACGGA---------------

---AGAAATGAAGATGACTACGCCATGCGGAGAAAATTTCAACCTCGACACGAGTCCGTG
TGTGGAGTGGTGCCCCCCATAAGGCGGGAA------------------------------
---G
AC------AGTGGAAAG------GGGTTCTCCCCCAACAATTCACAT-------------
--------------AGTGACTCCGCCTATGGCTCA-------------------------
-----GTTAACACGCCATGCACGCCGAATGTTAAC---CAGACCTTTAACTACTCCCAAG
GTGATCAGCAGAATCAAAATTACTCCCAAAATTACGAGCAATTTAGGAGCAGTGAA---C
AAGGGTTCGGCAATCAAGGCACCCTGGTTGTTGACGCTACGAGAGAGTCGCTCTATGGTT
ACTCGAAGTCTAACCCGGGACCG---AAGAGATAT-------------------------
-----CCG---GCAGACAAGTCTCAG---AATGTGCAG----------------------
---CCAGTTCGGTAT-
>JAKZAP010000001_Branchinecta_lynchi_Bib
ATGGGTCGCTCAAGTGCAGCAGTTCCC---------------------------------
------------------------------TTA---AACCAAAACTTGTCATCAGCAACA
GTTGACACGCTGCTTTTGACATTATTGAGGAAACTTGATAAAGAAAAGGAGGAA------

CCT---TGGGCGAAAATTCCGTTCCAAGCAGAAGTGAGAACTTTG---GAATTTTGGAGA
TCTGTAGTTGCAGAATGTGTTGGAACATTTTTTCTTGTGTTTCTAACATGTGGAGCGTTT
GTACCGTGGGCTGGGCATACG---------------------------------------
--CCCCCG
CCTATTGGAATTGCCCTGACTGTTGGATTCGGCGTTGTGATGTTAACGCACTGCTTTGCA
CTCATTTCAGGTGCCAATTTTAATCCTGTGGTGACCCTTGCGCTTGTGGTCAAAAGAAGC
ATGTCGCCATTTCGCGGGGCCCTCTATTTCACTGCCCAAGTCGGAGGATCCATCGCCGGA
TCAGCCTTGCTCTATGGGGTGACGTCATCCGGATCTCAAGGG------------------
------------------------------AATCTGGGAGTAACCACACTTGCTGCCGGT
---------TTGAAGGACTGGCAAGGATTTGCCATTGAACTCTGCTTCAGTTTCATCATC
GTGCTGACTTACCTTTGT---ACCCTCCACCCAGAGCAA---------------------
---AAGCGGACTGTGTCTACATCAATGGGAACAGGTCCTATCACTGTGGGTTTCGCCTAT
TTGGCTTGCTCGCTCGTGGGGCTACCATCTACTGGAGCTTCATTCAACCCCGTTCGGTCG
TTGGGTCCAGCTTTTGTCATGAAC---------AAATGGGATAGCCACTGGATTTACTGG
GTGGGACCCCTAATCGGGGGAGCCTTCGGAGGTCTTATCCACGAGTTTGTCTTCAACGCC
---TATCGAGAAGGAAGCAGC---------AGGAAGTGCAATGGC---GACGGA------
------------------------GAT------------GCTTCGAGCATCCACAGCGAT
GAA------GAA---TGCTACGAG---GAGGGG---CCAGCTGGG---CCCCCTGTCCGA
--GGGCCAGTTCCG
GGAGCTCTCGCTGTC---CGA---------------------------------------
------AGCCAGCATTATGATACG---------------TATAAGCCG---ATCGCCATA

```
---------------------------------------GGTATGGAAGGCATGTGCAAA
------TCAACACGA---------------------------------------------
AATCTCGACCAG---------------ACCGAAGGG---ATCTACTCCGGGACCCGCTCC
CTC---TAC---TGC------------------CGATCCCCCGGTCCTGCTCAGCGA---
------------------------------------------------------------
------------------------------AACATCCTCAACAGGTCCCAGTCCGTCTAC
GCGAAG---AATCCGGCGATCGGTGGG------------CAAAACTCC------------
CGC---ATCCACCCAGCTGCCAGC---ATAACGACAACGAAACCCGGAGGATTT------
---------------------GAAGCAATC------------------------------
------------------------------------------------------------
------------------------------------------------AGCCGCCAAGAG
AGCATTTACTCG---AGCGGCAGATGTCAG---------------------CCGCAGCCC
ATAAGG------------------------------------------------------
------------------------------------------------------------
------------------------------------------------------------
---------AAAGAGACCGTCTTT------------------------------------
------------------------------------TACTCGAAGCAGCAG------AGC
GAACACATCTACGCAACAAGGCCACCCCTTCCATCAGAC---CCGGGC------GTGTAC
---CCACCT---CGCCATGAG---------------------------------------
------------------ACACCCAACTCCTTC---------------------------
------------------------------------------------------------
---GCTTCCCAGGGATACCAGGCCCCCCTTCCCCCCACGCCAGTCAACGAGGACCAG---
------------------------------------------------------AAGCTT
------------GAAGAAGAAAACATGCGA------AATGCA------------------
---GAAAGCACAAAAGGAGACTTT---------GAC---TACGGA---------------
------------------------------------------------------------
---AAAAATGAAGATGATTACGCCATGCGGAGGAAATTCCAACCTCGACACGAGTCCGTG
TGCGGAGTGGTGCCGCCCGTTCGGCGGGAA------------------------------
-----------------------------------------------------------G
AC------AGTGGAAAG------GGGTTCTCCCCCAACAATTCTCAT-------------
--------------AGTGACTCCGCCTATGGCTCC-------------------------
-----GTTAACACGCCGTGCACGCCGAATGGTAAC---CAGACATTTAACTACTCCCAAG
GGGATCAGCAGAATCAAAATTACTCCCAAAATTACGAGCAATTTAGGAGCAGTGAA---C
AAGGGTTCGGCAATCACGGCACCCTGGTTGTTGACGCTACGAGGGAGTCACTTTATGGTT
ACTCTAAGTCGAAACCGGGACCG---AAGAGATAT-------------------------
-----CCG---GTGGAGAAGTCTCAA---AATGTGCAG----------------------
-----------------------------------------CCAGTTCGGTAT-
>PQ469256_Artemia_franciscana_Bib
ATGGCGAAGAAAAGTGTAACTGTTCCT---------------------------------
------------------------------CTA---AATACAAACATATCTTCAGCAACA
GTAGACACATTACTAGTGACGCTATTAGAAAAGCTCGATAAAAGACACGATGATGAT---
------------------------------------------------------------
------------------------------------------------------------
------AATGTGGCTATACCATTTCAAGCAGAAGTTAGGACTTTG---GAATTTTGGAGA
TCAGTTGTGGCTGAATGTGTTGGAACTTTTTTTTTGATATTCCTGACATGTGGTGCGTTT
ATACCTTGGTCAGGACACAGC---------------------------------------
------------------------------------------------------CCTCCA
TATATTGGAATCGGTTTAACCGTTGGATTCACTCTAGCCATGTTAACACATTGTTTCGCT
```

CTAATTTCCGGTGCAAATTTCAACCCTTCGATAACGTTGGCTTTGGTTGTCAAAAGATCT
ATGTCTCCATTTCGTGGAGCTTTGTACTTTGTGGCACAAATTGGAGGAGCAATTGCGGGA
TCAGCCTTATTATACGGAGTGACTACTTCCGGCTCACAGGGT------------------
------------------------------AGCCTTGGTGTAACAGTCATCGCATCAGGT
---------CTTCGGGATCGGCAAGGGTTCGCAATAGAATTTTCCTTCACATTCATTATT
GTACTGTCCTATCTGGGC---TCCTTGCATCCTGAGCAG---------------------
---AAAAAAACCGTTTCAACTTCAATGGGAAGTGGTTCAATCATGGTTGGATTTAGTTAC
CTAGCATGTTCCCTTGTTGCTTTACCGTCCACTGGCGCGTCGTTCAACCCTGTGAGGTCG
CTGGGACCGGCGTTTGTGATGAAC---------AAATGGGATAGTCACTGGATATACTGG
ATTGGACCATTACTTGGTGGCGCATTTGGAGGACTTATACACGAGTTTGTTTTTAATACA
---TATCAAGATGGCTGCCAA---------AGAAAGTCAACAGGT---GAAGGC------
------------------------GAT------------TCTTCAAGTGTACATAGCGAC
GAA------GAA---TGCTATGAA---GAGGCA---GACCATGGTAGCACTCCACTGAGA
--GGTCCAGTTCCG
GGAGCTCTGACTCCT---AGA---------------------------------------
------AATCAACACTATGAAGCT---------------TATAAGCCA---ATGACCGTT

---------------------------------------GCCCAAGACAACTTGTGCAAA
------TCTTCAAGG---
AATTTGGACCAA---------------TCAGAATCA---ATATATGCTGGAACGCGTTCA
CTT---CAT---TGT------------------CGCTCACCGATAGCTGCTCAGCGC---

------------------------------ACCATGCTCAATCGCTCTCAGTCAGTCTAC
TCGAAG---TCCCAAAATCTGGTTAAT------------CAA------------------
AGG---ATTCATCCAGTGGCTAGC---GCTGTGGCACCAAAAAATGGTGGCTTT------
---------------------GGAGCACTT------------------------------

--TCAAGACAAGAG
AATATTTACGTT------GGTAGACTTGGG---------------------GCACCATCT
ATGAGG---TCAGAA---
------------------AGTATTTACGTC------------------------------
------------------------------CCACGAAGCCAA------TTTCAA------
---------AATGAAGCAGTTTAT------------------------------------
------------------------------------TACTCAAAAAAGCAG------AAC
GAAAACGTTTATTCAACAAAGCCTCCTCTTGCTTCCGAT---CCA------------TAC
---CCACGG---
------------------AGTGATTCAGGCTATTCCCAG---------------------
------------------------------AAA---------------------------
---GCAGATCAGCTCTACCAACCTCCTCTTCCTCCTACTCCTATGAGTGAAGACCAA---
--AAGCTG
------------GAGGAGGAAAATATAAGG------AATGCT------------------
---GAGTGCACCAGAGCTGAGTACACC------AGA---AGCTCG---------------
--AATGATCCCTAT
AACCGAAATGAAAATGAATACGGA---AGACGTCAGTTACCTCAAAGGCATGAATCCGTT
TGTGGGGTAGTTCCCCCAATAAGGAGAGAT------------------------------
---G
AA------AGTTACAAA------CAATTTTCCCCAAACAATTCTCAT-------------
--------------AGTGATTCCGCATATGGTTCT-------------------------

```
-----GTAAATACGCCCTCTACACCTAGCGGAAAT---AAAGGGTTTAACTACTCCCTGT
CTGAAAATCAAAATAAGAACTATCAGCAGACT---GAACAGTTAAGAGGTAATGAG---T
ATAAT---TGCAATCAGGGAGCTTTAATTGTTGACGCCACGCGGGAATCCCTCTATGGCT
ATTCTAAGCAAGAGATGCTGCAG---AATCGACAA-------------------------
--------AATTCAACTGATAATCAT---AACGTGCAA----------------------
-----------------------------------------GCTGTCCGATAT-
>HACK01010029_Cherax_quadricarinatus_Bib
ATGGCTAGCACTGGTATGGGC---------------------------------------
------------------------------------ACTACT------GGGGGTGCCAGC
GTGGATGCCCAGCTAGTGGCACTCCTTGACAAGTTGGAGGGTCTTCGTCAGGAG------
------------------------------------------------------------
---------------------------------------------------------GGC
AGGCCACCCCCTAGGCCGCCCATGATTGCTGAGGTGCGCACGTTA---GAGTTGTGGAGG
GCAGTGATAGCAGAGTGTCTGGCCACTGTCTTCTACGTGTTCCTGGTGTGTGGTGCCTAC
AGTCCCTGGACGGGCAAGACACTCACA---------------------------------
---------------------------------------------------CAGGAAGGT
CAACTTACTATATCCCTGGTGGCTGGCCTCGCCATGGCTGTACTAGTACATAGCTTCGGC
CAGGTGTCAGGAGGGCACGTGAACCCGGCCGTGACGGTGTCACTAGCGGTGACAAGGAGG
GTCTCCCCCCTTAGGGCCGCCATGTTCGTCTTGGCTCAGCTGGGCGGCGGCATTGCTGGT
GCTGCTCTTCTCTACGGGGCGACGTCGTCAGGGTACACCGGT------------------
------------------------------GACCTGGGAGCTACTGTGGTGTCTCAGGTG
---------ATCTCCCCGTGGCAGGGTCTGGGTCTGGAGTTCCTGCTCACCTTCGTGGTG
GTGTTCGTGGTTTTCGCT---TCCTTCAACCCCTACCGC---------------------
---AGG---------------AACTTCGGCAACCCCGCCGTTGCCATCGGCCTGGCCTAC
CTCGCCTGTACGCTTGTTGGGCTGCCCCTGACAGGCGCCTCTATGAACCCGGCTCGCTCC
CTGGGACCTGCTTTCGTTAAGAAC---------AAGTGGGACGCTCACTGGGTGTACTGG
GTGGCTCCACTGTTGGGAGGGGTGACTGCTGGCCTCATCTATGAGTATATCTTCAATCCT
---CACAGACTCCCTCGTTCA---------CGCAAGGATTCCATA---GATGGA------
------------------------GAT------------TCATCAAGTGTTGGGAGTGAT
GAA------GAT---CCATATGAT---GAG---------TGTTCCAAGCCTCCACCT---
------------------------------------------------CGTTATAATTAC
AATGCCCTC---------CGA---------------------------------------
------GCCCAACACTACGACCAA---------------TACCGGCCT---GCCAATACC
------------TCCAACACCACCTCCCATTA-------------------GGT------
------TACCCAGCAAGTATGTATGGACATACA---GGAACTCCATCCGTTTACAATGCT
------TCTTCATAC---------------------------------------------
AAATATGACACTAGTTATACTCCTAACAAAGATGAA---ATTTATTCTGGTTCAAAGAGT
TTA---TAT---GCC------------------AAATCTCCT---CCATTGCCACGT---
------------------------------------------------------------
---------------------------TCTACATCCCTCAACCGTTCCCAATCTGTTTAC
ACTAAG---CCGCCACCACCTCGCATG------------GACAACTAT---------TCC
AGGAATCTTGCTCAGTCACAGAGT---CTGTGTTCCAAACCTGCTGCTAGCTTT------
---------------------TCTGCTCTT------------------------------
------------------------------------------------------------
------------------------------------------------TCTCACACGGAG
AGTATATAT------------------------------------------AAGAACAAT
CCTCGC---CAGGAC---------------------------------------------
------------------ACACTTTATGTT------------------------------
```

------------------------ACA---AGTAAATCTGGA---ATTGTTAAG------
---------CCAGAGCCAGTTTAT------GGTACAGCA---------------------
---------------------------------ACATACAACAAGCAAAAT---------

------------GACTCGGGGGATTCAAACTATTCAACT---------------------
---------------------------TACCGC---------------------------
---GGCTCTGCCAGTTAC--

---AGCTCCTCGCGCTCGTCC---------------------------------------
--AACCCCTGC---

------------CCACCGATGCCCATG---------------------------------
--------------------------------------AGTCGC---------------A
CT------CGCACTGAC------AATCTTACTCCC-------------------------

-----------AACTCAGTTGCTTCA----------------------------------
--GCCCATTCTGGTCATGCTACA-------------------------------------
-----CCAAACTCTGTAGCATTATGC---TATTCTCCT----------------------
---AATCCACAGTAT-
>GJEB01066093_Astacus_astacus_Bib
ATGGCGAGCACTGCCATGGGCGGT------------------------------------
--GGGAGCACCAGC
GTGGATGCCCAGCTGGTGGCACTCCTCGACAAGCTGGAGGGCCTTCGTCAGGAG------

---TCC
AGCCCGCCCCCCAGGCCCCCCATGATTGCCGAGGTGCGCACCTTG---GAGCTGTGGCGG
GCGGTGATAGCAGAGTGCCTGGCGACTGTGTTCTACGTGTTCCTGGTGTGCGGCGCATAC
AGCCCCTGGACGGGCAAGACCCTCACG---------------------------------
---CAGGAGGGT
CAGCTCACCATCTCCCTGGTGGCCGGCTTCGCCATGGCTGTCCTCGTCCATAGCTTCGGC
CAGGTGTCAGGAGGGCACGTGAACCCGGCGGTGACGATATCGCTGGCTGTGACCCGGCGT
GTCTCTCCCCTGAGAGCAGCCATGTTCGTCCTGGCTCAACTGGGTGGAGGCATTGCTGGT
GCCGCGCTTCTCTACGGGGCGACGTCTTCAGGGTACACGGGC------------------
------------------------------GACCTGGGGGCGACGGTGGTGTCGCAGGTC
---------ATCTCGCCGTGGCAGGGGCTGGGTATGGAGTTCCTGCTCACCTTCGTCGTC
GTCTTCGTCTTCTTCGCC---TCTGTCAACCCCTACCGC---------------------
---AGG---------------AGCTTCGGCAACCCCGCCATCGCCGTGGGCCTCGCCTAC
CTCGCCTGCACGCTCGTCGGGCTGCCGCTGACCGGGGCGTCGATGAACCCCGCACGCTCT
CTCGGCCCCGCCTTCGTCAAGAAC---------AAGTGGGACGCACACTGGGTGTACTGG
GTAGCTCCTCTGTTGGGAGGCGTGACAGCTGGCCTCATCTACGAGTACATCTTCAACCCA
---CACAGACTACCACGCTCC---------CGTAAGGACTCCATT---GATGGA------
------------------------GAT------------TCATCCAGTGTTGGGAGCGAT
GAA------GAT---CCATATGAT---GAG---------TGTTCGAAGCCTCCACCA---
--CGCTACAATTAC

AATGCTCTT---------CGA---------------------------------------
------GCCCAACACTATGGCCAA---------------TACCAGCCT---GCCAACACC
------------TCCAACACCACCTCCCCACTA------------------GGT------
------TACCCAGCAAGCATGTATGGACATACA---GGAACGCCTTCTGTTTACAATGCC
------TCTTCATAC---
AAATACGACACTAGTTATACCTCTAACAAAGATGAT---ATTTATTCTGGATCCAAGAGT
TTG---TAT---GCC------------------AAATCTCCA---CCAATGCCACGC---

---------------------------TCTACGTCTCTCAACCGTTCCCAGTCTGTTTAT
ACCAAA---CCACCCCCACCTCGCATG------------GACAACTAT---------TCC
AGGAATCTTGCTCAATCACAGAGT---CTGTGTTCCAAACCTGCTGCTAGCTTC------
---------------------TCTGCTCTT------------------------------

--TCTCACACTGAG
AGCATATAC--AAGAATAAT
CCTCGA---CAAGAC---
------------------ACACTTTATGTT------------------------------
------------------------ACA---AGTAAGTCTGGA---ATTGTTAAA------
---------CCAGAGCCAGTCTAT------GGTACAGCA---------------------
---------------------------------ACATAT---AAGCAAAAT---------

------------GACTCTGGGGATTCAAACTATTCAACT---------------------
---------------------------TACCGA---------------------------
---GGCTCTGCCAGCTAC--

---AGCTCGTCACGCTCATCC---------------------------------------
--AACCCCTGC---

------------CCACCAATACCTATG---------------------------------
--------------------------------------AGCCGT---------------A
CT------CGCACTGAC------AATCTCACTCCT-------------------------

-----------AATTCAGTTGCCTCA----------------------------------
--GCCCACTCTGGTCATGCCACA-------------------------------------
-----CCAAACTCTGCAACTTTGTGC---TATTCTCCT----------------------
---AATCCTCAGTAT-
>GFDA01108900_Homarus_americanus_Bib
ATGGCCAGCACTGTAATGAGTGGTGGTGGTGGTGGT------------------------
--GGTGGCACCAGC
GTGGATGCTCAGCTGGTGGCACTACTGGACAAGCTGGAGGGCCTTCGTCAGGAG------

---TCG
AGTCCTCCTCCCAGACCTCCAATGATCGCCGAGGTCCGCACCCTG---GAGTTGTGGAGG
GCGGTAATAGCCGAGTGTTTGGCTACGGTGTTCTACGTCTTCCTGGTTTGTGGCGCCTAC

AGCCCCTGGACGGGCAAGACCCTCACG---------------------------------
---CAGGAGGGG
CAGCTCACCATCTCTCTGGTGGCCGGGTTTGCCATGGCCATCCTCGTCCACAGTTTCGGC
CAGGTATCAGGAGGACACGTGAACCCGGCGGTGACAGTGTCGTTGGCTGTGACGCGACGA
GTGTCTCCCTTGAGGGCCGCCATGTTCGTGTTGGCCCAACTGGGAGGAGGCATCGCTGGG
GCTGCACTGCTCTACGGAGCCACGTCGTCTGGGTACATTGGT------------------
------------------------------GACCTAGGAGCCACGGGGGTGTCTCAGGTC
---------ATCTCCCCCTGGCAGGGTCTGGGGCTGGAGTTCCTGTTGACCTTCGTGGTG
GTGTTTGTCTTCTTCGCC---TCCGTCAACCCCTACAGG---------------------
---AGG---------------AACTTCGGTAACCCTGCTGTGGCCATCGGCCTCGCCTAC
CTCGCCTGCACACTTGTCGGACTGCCTCTGACGGGCGCTTCAATGAACCCAGCTCGCTCC
CTGGGTCCTGCCTTCGTCAAAAAC---------AAATGGGACGCACACTGGGTGTACTGG
GTGGCTCCTCTGCTAGGAGGGGTGACTGCTGGCCTCATCTACGAGTACATCTTCAACCCT
---CATAGACTCCCACGCTCA---------CGCAAGGACTCCATC---GATGGA------
------------------------GAT------------TCATCTAGTGTTGGGAGTGAT
GAA------GAT---CCATATGAT---GAG---------TGTTCCAAACCTCCACCG---
--CGCTACAACTAC
AATGCCCTC---------CGA---------------------------------------
------GCCCAACACTATGACCAG---------------TACCGACCC---GCCAATACC
------------TCCAACACTACCTCCCCCTTA------------------GGC------
------TATCCAGTAAGCATGTATGGGCATGCA---GGGACTCCTTCTGTGTACAATGCC
------TCTACATAC---
AAATATGAAAATGGCTACACCACCAACAAAGATGAT---ATTTATTCTGGTTCCAAGAGT
CTG---TAT---GCC------------------AAGTCTCCG---CCGATGCCACGT---

---------------------------TCTACATCTCTCAACCGTTCCCAGTCTGTCTAT
ACCAAG---CCACCACCACCTCGCATG------------GACAACTAT---------TCC
AGGAATCTTGCTCAGTCGCAGAGT---CTGTGCCCCAAGCCTTCTGCCGGATTC------
---------------------ACTGCTCTG------------------------------

--TCCCACTCAGAC
AGTATATAC--AAGAACAAC
CCTCGC---CAGGAC---
------------------ACACTCTATGTC------------------------------
------------------------ACA---AGTAAATCTGGG---ATTGTTAAG------
---------CCAGAGCCGGTCTAT------GGTACAGCA---------------------
---------------------------------ACCTACACCAAGCAGAAT---------

------------GACTCGGGTGACTCAAACTATTCAACG---------------------
---------------------------TACCGA---------------------------
---GGCTCTGCCAGTTAC--

---AGCTCGTCACGCTCTTCT---------------------------------------
--AACCCAGGC---

------------CCACCAATACCCATG---------------------------------

```
---------------------------------------AGTCGT--------------A
CT------CGCATTGAC------AATCTCACTCCC-------------------------
------------------------------------------------------------
------------------------------------------------------------
------------------------------------------------------------
-----------AATTCAGTTGCTTCA----------------------------------
--GCCCACTCTGGTCATGCAACA-------------------------------------
-----CCAAGCTCTACAACTTTGTGC---TACTCTCCT----------------------
------------------------------------------AATCCCCAGTAT-
>GHUJ01004462_Panulirus_argus_Bib
ATGGCCAGCACAGTGATGGGAGGT------------------------------------
-------------------------------------------------GGTGGCATCAGC
GTGGACGCCCAGCTGGTGGCTCTACTGGACAAGCTTGAGGGCCTTCGTCAGGAG------
------------------------------------------------------------
---------------------------------------------------------GCC
AGTCCACCCCCAAGGCCTCCTATGATCGCTGAAGTCCGTACCCTG---GAGCTGTGGAGG
GCCGTGATAGCGGAGTGCCTGGCCACCGTCTTCTACGTGTTCCTGGTGTGTGGTGCTTAC
AGCCCTTGGACTGGCAAGTCCCTCACG---------------------------------
---------------------------------------------------CAGGAGGGA
CAGCTCACCATCTCCCTGGTGGCTGGCTTTGCCATGGCCATCCTCATCCACAGCTTCGGC
CAGGTGTCAGGAGGGCACGTGAACCCCGCGGTGACCGTCTCCCTGGCGGTGACGCGGCGC
GTGTCTCCGCTCCGGGCGGCTATGTTCGTCCTGGCTCAGCTGGGAGGAGGGATCGCCGGC
GCTGCTCTACTATACGGGGCGACGTCGTCAGGATTCTCGGGC------------------
------------------------------GACCTGGGGGCCACTGGCGTGCACAAGGTC
---------ATCACGCCCTGGCAGGGGCTGGGCATCGAGTTCCTGCTGACCTTCGTGGTG
GTGTTCGTCTTCTTCGCC---TCCGTCAACCCCTACCGC---------------------
---AGG---------------AGCTTCGGCAACCCCGCCGTCGCCGTGGGTCTGGCCTAC
CTGGCCTGCACGCTGGTCGGGCTGCCCCTGACCGGCGCCTCCATGAACCCCGCTCGGTCC
CTGGGCCCTGCCTTTGTCAAGAAC---------AAGTGGGACGCACACTGGGTGTACTGG
GTTGCTCCACTGCTTGGTGGTGTCACTGCTGGCCTCATCTATGAATACATCTTCAACCCT
---CACAGACTTCCACGCTCA---------CGGAAGGATTCAATT---GATGGA------
------------------------GAT------------TCATCTAGTGTTGGGAGTGAA
GAA------GAT---CCATATGAT---GAA---------TGTTCTAAGCCACCACCT---
------------------------------------------------CGCTACAATTAT
AATGCCCTT---------CGA---------------------------------------
------GCCCAGCACTATGAACAG---------------TACCGACCT---GCCAACAGT
------------TCCAACACTACCTCTCCTTTA------------------GGT------
------TATCCAGCAAGCATGTATGGACATACA---GGGACTCCATCTGTTTACAATGCC
------TCTGCTTAT---------------------------------------------
AAGTATGAGAGTGGC------------AAAGATGAA---ATTTATTGTGGTTCCAAGAGT
ATG---TAT---GCT------------------AGATCTCCA---CCACTACCTCGC---
------------------------------------------------------------
---------------------------TCTACATCTCTCAATCGCTCTCAGTCTGTTTAT
ACCAAA---CCACCACCACCACGCATG------------GACAACTAT---------TCC
AGGAACCTTGCTCAGTCCCAAAGT---TTGTGTCCTAAGTCCACAGCTGGCTTT------
---------------------GCAGCTCTC------------------------------
------------------------------------------------------------
------------------------------------------------TCTCATACAGAC
```

AGCATGTAC--AAGAACAAC
CCTCGT---CAGGAC---
------------------ACTCTCTATGCC------------------------------
------------------------ACA---AGTAAGTCTGGG---ATTGTTAAG------
---------CCAGAGCCAGTCTAT------GGCACAGCA---------------------
---------------------------------CCCTATAACAAGCAAAAT---------

------------GACTCCGGAGATTCAAACTATTCAACT---------------------
---------------------------TACCGA---------------------------
---GGATCAGCCAGCTAC--

---AGTTCTTCACGCTCTTCC---------------------------------------
--AATCCTGGC---

------------CCACCAGTACCCATG---------------------------------
--------------------------------------AGTCGC---------------A
CA------CGCACAGAC------AACCTCACTCCT-------------------------

-----------AATTCAGTTGTTTCA----------------------------------
--GCCCATTCTGGTCATATCACT-------------------------------------
-----CCAAACTCTGCAGCTTTATGC---TATTCCCCT----------------------
---AATCCGCAATAT-
>GEFB01001794_Cancer_borealis_Bib
ATGGCCAGCACTGCG---GGTGGCGGCGGT------------------------------
--GGCGGGTCCACT
GTAGACGCCCAGCTCGTAGCGCTGCTGGACAAGTTGGAAGGCCTTCGGCAGGAG------

---GTT
ACTCCACCTCCAAGGCCGTCCATGATCGCAGAGGTGCGCACCCTG---GAGCTGTGGCGC
GCGGTGATCGCCGAGTGTCTGGCGACGGTGTTCTATGTGTTCCTCGTGTGCGGTGCCTAC
AGCCCTTGGACCGGCAAGGTTTTGACG---------------------------------
---CAGGACGGC
TATCTGACGGTGGCCCTGGTGGCGGGATTCACTATGGCCATTCTCGTCCATAGCTTCGGC
CAGGTATCCGGCGGCCACATCAACCCAGCGGTGACGATGTCGTTGGCAGTCACGCGGCAT
GTCTCCCCCCTGCGCGCCGCCATGTTCATATTGGCTCAGCTGGGTGGTGGAATAGCCGGT
GCTGCACTACTTTACGGGGCGACGTCATCTGGTTATACGGGC------------------
------------------------------GATCTGGGAGCCACCACGGTGTCGCAAGTT
---------ATCACCCAGTGGCAGGGACTCGGCCTGGAGTTTCTTCTGACCTTCGTGGTG
GTCTTCGTTTTCTTCTCT---TCCGTCAATCCCTACCGC---------------------
---AGG---------------AGCTTCGGAAATCCTTCCATCGTAATCGGCTTCGCCTAC
ATGGCCTGCACACTCGTAGGGCTGCCCTTGACTGGTGCTTCCATGAACCCTGCACGTTCC
CTCGGCCCCGCTTTTGTCAAGAAC---------AAGTGGGATGCTCACTGGGTGTACTGG
GTGGCTCCTCTGCTGGGCGGCATGACAGGAGGACTCATCTATGAATACATCTTCAACCCA
---CATCGGATGCCACGTTCA---------CGCAAGGACTCGATT---GATGGA------

------------------------GAT------------TCCTCAAGTGTAGGTAGTGAT
GAA------GAT---CCGTATGAC---GAA---------TGCTCCAAGCCTCCACCC---
--CGGTATAACTAC
AATGCTCTT---------CAT---------------------------------------
------GCCCAAAACTATGACCAG---------------TACCGACCT---GCCAACTCC
------------TCCAACACCACCTCTCCCTTA------------------GGA------
------TACCCAGCAAGTATGTACGGACATAAC---GGAGCATCTTCAGTTTGCAATGCA
------TCAGCATAC---
AAGTATGATAATGGTTATGTT------AAAGATGAT---ATTTACTCAGGATCAAAGAGT
TTA---TAC---ACC------------------AAATCTCCA---CCTTTGCCAAGA---

---------------------------TCCTCTTCTTTGAACCGCTCCCAGTCCGTGTAC
TCCAAA---CCTCCACCACCTCGCATG------------GATAACTAC---------AAC
AGGAACCTCACTCACTCTCAAAGC---CTGTGTCCGAAAGCAACTTCTGATTTT------
---------------------TCCACTCTC------------------------------

--CCTCACACAGAT
AACATGTAC--AAAAGCAAT
CCACGG---CGTGAC---
------------------AGTCTCTATGTC------------------------------
------------------------ACA---AGTAAGTCTGGG---ATTGTGAAA------
---------CCTGAGCCAGTATAT------GGAACA------------------------
---------------------------------TCATATGGTAAACAAAAT---------

------------GATTCAGGAGATTCAAACTACTCAACT---------------------
---------------------------TACCGA---------------------------
---GGCACAGCTAGCTAC--

---AGTTCATCTCGTTCCTCA---------------------------------------
--AACCCTGGG---

------------CCACCAGTTCCCATG---------------------------------
--------------------------------------AGTCGA---------------A
CA------CGTACTGAC------AATCTCACTCCT-------------------------

-----------AATTCTGTTGTTTCA----------------------------------
--GCTCATTCAAGTCTTGTTACT-------------------------------------
-----CCAAACTCAACAGGTTTGTGT---TACTCTCCT----------------------
---AATCCTCAGTAT-
>GHOI01026330_Metacarcinus_magister_Bib
ATGGCCAGCACTGCG---GGTGGCGGCGGT------------------------------
--GGCGGGTCCACT
GTAGACGCCCAGCTCGTAGCGCTGTTGGACAAGTTGGAGGGCCTTCGGCAGGAG------

---GTT
ACTCCGCCTCCAAGGCCGTCCATGATCGCAGAGGTGCGCACCCTG---GAGCTGTGGCGC
GCGGTGATCGCCGAGTGTCTGGCGACGGTGTTCTATGTGTTCCTCGTGTGCGGTGCCTAC
AGCCCTTGGACAGGCAAGGTTTTGACG---------------------------------
---CAGGACGGC
TACCTGACAGTGGCCCTGGTGGCGGGGTTCAGCATGGCCATTCTCGTCCATAGCTTCGGC
CAGGTATCCGGGGGCCACATCAACCCAGCGGTGACGATGTCGCTGGCAGTCACGCGGCAC
GTCTCCCCCCTGCGCGCCGCCATGTTCATATTGGCTCAGCTGGGTGGTGGAATAGCCGGT
GCTGCACTACTTTACGGGGCGACGTCATCTGGTTATACGGGC------------------
------------------------------GATCTGGGAGCCACTACGGTGTCGCAAGTT
---------ATCACCCATTGGCAAGGACTCGGCCTGGAGTTTCTTCTGACCTTCGTGGTG
GTCTTCGTTTTCTTCTCC---TCCGTCAATCCCTACCGC---------------------
---AGG---------------AGCTTTGGAAATCCTTCCATCGTAATCGGCTTCGCCTAC
ATGGCCTGCACACTCGTAGGGCTGCCCTTGACTGGTGCTTCCATGAACCCTGCACGTTCC
CTCGGCCCCGCTTTCGTCAAGAAC---------AAGTGGGATGCTCACTGGGTGTACTGG
GTGGCTCCTCTGCTGGGCGGCATGACAGGAGGACTCATCTATGAATACATCTTCAACCCA
---CACCGGATGCCACGTTCA---------CGCAAGGACTCGATT---GATGGA------
------------------------GAT------------TCCTCAAGTGTAGGTAGTGAT
GAA------GAT---CCGTATGAC---GAA---------TGCTCCAAGCCTCCACCC---
--CGGTATAACTAC
AATGCTCTT---------CAT---------------------------------------
------GCCCAAAACTATGACCAG---------------TACCGCCCT---GCCAACTCC
------------TCCAACACCACCTCTCCCTTA------------------GGA------
------TACCCAGCAAGTATGTATGGACACAAC---GGAGCATCTTCAGTTTGCAATGCA
------TCAGCATAC---
AAGTATGATAATGGTTATGTT------AAAGATGAT---ATTTACTCAGGATCAAAGAGT
TTA---TAC---ACC------------------AAATCTCCA---CCTTTGCCAAGA---

---------------------------TCCTCCTCTTTGAACCGCTCCCAGTCTGTGTAC
TCCAAA---CCTCCACCACCTCGCATG------------GATAACTAC---------AAC
AGGAACCTCACTCACTCTCAAAGC---CTGTGTCCGAAAGCAACTTCTGATTTC------
---------------------TCTACTCTC------------------------------

--CCTCACACAGAT
AACATGTAC--AAAAGCAAT
CCACGG---CGTGAC---
------------------AGTCTCTATGTC------------------------------
------------------------ACA---AGTAAGTCTGGG---ATTGTGAAA------
---------CCTGAGCCAGTATAT------GGAACA------------------------
---------------------------------TCATATGGTAAACAAAAT---------

------------GATTCAGGAGATTCAAACTACTCAACT---------------------
---------------------------TACCGA---------------------------
---GGCACAGCTAGCTAC--

---AGTTCATCTCGTTCCTCA---------------------------------------

--AACCCTGGG---

------------CCACCAGTTCCCATG---------------------------------
--------------------------------------AGTCGA---------------A
CA------CGTACTGAC------AATCTCACTCCA-------------------------

-----------AATTCTGTTGTTTCA----------------------------------
--GCTCATTCAAGTCTTGTTACT-------------------------------------
-----CCAAACTCAACAGGTTTGTGT---TACTCTCCT----------------------
---AATCCTCAGTAT-
>GFBL01068750_Eriocheir_sinensis_Bib
ATGGCCAGCACGGTG---GGAGGCGGAGGC------------------------------
--GGCGGCAGC---
GTGGACGCCCAGCTGGTGGCGCTCTTGGACAAGCTGGAGGGCCTTCGGCAGGAG------

---GTC
ACCCCGCTTCCAAGGCCCTCCATGATCGCCGAGGTGCGCACGCTG---GAGCTGTGGCGG
GCCGTGATCGCCGAGTGTCTGGCCACGGTGTTCTATGTGTTCCTGGTGTGCGGAGCCTAC
AGCCCCTGGACCGGCAAGGTGCTGACG---------------------------------
---CAGGACGGA
CAGCTCACCGTGGCCCTGGTGGCGGGTTTCTCCATGGCCATCCTCGTCCACAGCTTCGGA
CAGGTGTCCGGCGGCCACGTGAACCCCGCCGTGACGGTGTCCCTCGCCGTCACCCGGCAC
GTGTCTCCCCTCCGCGCAGCCATGTTCGTCTTGGCGCAGCTCGGGGGAGGGATAGCAGGC
GCTGCGCTGCTCTACGGAGCGACGTCTTCCGGCTACACGGGG------------------
------------------------------GACCTGGGCGCCACCTCCGTCTCGCAGGTC
---------ATCACACAGTGGCAAGGTCTCGGGCTGGAGTTCCTGCTCACTTTCGTCGTG
GTCTTCGTCTTCTTCTCT---TCCGTTAACCCCTACCGG---------------------
---AGG---------------AGCTTCGGGAACCCCTCCGTCGTGATTGGGTTCGCCTAC
ATGGCTTGCACTCTCGTGGGGCTGCCCCTGACCGGCGCCTCCATGAATCCCGCGCGCTCC
CTGGGTCCTGCTTTCGTCAAGAAC---------AAATGGGACGCGCACTGGGTGTACTGG
GTGGCCCCGCTGGTGGGCGGCGTGGCTGGCGGCCTCATCTACGAGTACATCTTCAACCCG
---CACCGCCTGCCACGCTCC---------CGCAAGGATTCCATC---GATGGA------
------------------------GAT------------TCGTCTAGTGTAGGGAGTGAG
GAA------GAT---CCGTATGAT---GAA---------TGCTCCAAGCCTCCACCT---
--CGGTATAATTAC
AATGCTCTT---------CAC---------------------------------------
------GCCCAAAACTATGACCAG---------------TACCGGCCT---GCCAACACC
------------TCCAACACCACCTCTCCCTTA------------------GGA------
------TACCCAGCAAGTATGTATGAACATAAT---GGAGCATCTTCGGTTTGCAATGCA
------GCAGCATAC---
AAGTATGATAATGGCTATGTT------AAAGATGAA---ATTTACTCAGCCTCAAAGAGT
TTG---TAC---ACC------------------AAATCTCCA---CCTTTGCCAAGA---

---------------------------TCCTCCTCTCTGAATCGCTCTCAATCAGTGTAC
ACTAAA---CCCCCACCACCTCGAATG------------GACAACTAC---------AAC
AGAAACCTCACCCACTCTCAAAGT---TTGTGTCCTAAAACAAGCTCTGACTTC------

---------------------ACCACCCTC------------------------------

--CCCCACACAGAC
AACATGTAC--AAAAACAAC
CCTCGA---CGTGAC---
------------------AGCCTCTATGTT------------------------------
------------------------ACA---AGCAAGTCAGGG---ATTGTGAAA------
---------CCTGAGCCAGTGTAT------GGAACA------------------------
---------------------------------TCATACAGTAAACAAAT----------

------------GATTCTGGAGACTCAAACTACTCCACA---------------------
---------------------------TACAGA---------------------------
---GGCACAGCCAGCTAT--

---AGTTCCTCACGCTCTTCA---------------------------------------
--AACCCAGGA---

------------CCACCAATACCTATG---------------------------------
--------------------------------------AGTCGA---------------A
CA------CGCACAGAC------AATCTCACCCCA-------------------------

-----------AATTCTGTTGCCTCA----------------------------------
--GCCCACTCAAGTCTTGTCACT-------------------------------------
-----ACAAACTCAGCAGGTCTGTGT---TATTCACCC----------------------
---AATCCTCAGTAT-
>GGXN01003003_Neocaridina_denticulata_Bib
ATGGCTAGCTCTGTCGTA--
--GGAGGA---ACA
CTAGAGATTCAGTTACTCACTCTACTTGACAAATTAGAGAGTTTGAGACCAGAA------

---CCT
AATCCCCCAACAAGACCTCCAATGATAGCAGAGGTCCGAACCCTA---GAATTATGGAGA
GCCGTTATTGCCGAATGCCTTGCCACCGTATTCTATGTATTCTTAGTCTGTGGGGCATAC
AGCCCTTGGACAGGGAAAACTCTCACC---------------------------------
---CCTGAGGGA
CAGCTCACCATATCCTTGGTGGCTGGACTAGCAATGGCTATACTAGTCCATAGCTTTGGA
CAGGTATCTGGTGGCCATATCAATCCAGCAGTTACTGTTTCTTTAGCAGTAACGCGACGA
GTGTCTCCTTTGAGAGCCGCGATGTTCATCTTGGCACAACTGGGAGGAGGCATTGCTGGA
GCTGCGCTTCTCTATGGAGCGACCTCTTCCGGCTACACGGGA------------------
------------------------------GACTTGGGAGCAACGACGGTATCCCAAGTG
---------ATAACACCATGGCAGGGTCTGGGCCTCGAGTTTCTGCTGACTTTCGTCGTG
GTGTTCGTTGTGTTTGCC---TCTGTCAACCCTTACAAG---------------------
---AGGAACTTG---------------GGTAATCCTGCCATGGCCATTGGCATTGCTTAC
CTCGCATGTACTCTCGTTGGGCTTCCATTGACTGGCGCCAGCATGAATCCTGCCCGTTCC

TTGGGTCCAGCATTCGTCAAGAAT---------AAATGGGACGCACATTGGGTTTATTGG
GTAGCACCACTCCTCGGAGGAATCACAGCTGGACTCATTTATGAATACATCTTCAATCCA
---CACCGACTTCCAAGATCA---------CGAAAGGATTCAATA---GATGGA------
------------------------GAC------------TCTTCAAGTGTTGGGAGTGAT
GAG------GAT---GCTTATGAT---GAA---------TGTTCCAAACCTCCTCCA---
--CGTTATAATTAC
AATGCTTTG---------CGT---------------------------------------
------GCTCAACACTACGATCAG---------------TACCGCCCT---GCCAACACT
------------TCCAACACTACTTCACCACTT------------------GGT------
------TATCCTGCCAGCATGTATGGACACACA---GTTGCCCCATCTGTTTACAATACT
------TCTACATAT---
AAATACGATAATGGTTGTGTGACCAACAAAGACGAC---ATTTATTCAGGCTCCAAGAGC
ATG---TAT---GCC------------------AGATCACCT---CCTTTGCAAAGG---

---------------------------GCAACTTCCCTCTCTCGCTCACAGTCAGTGTAC
ACCAAG---CCACCTCCTCCTCGAATA------------GATAACTAT---------TCT
AAAAATCTTACACAGTCTCACAGT---ATGTGTAATAAGACAGCTGCAGGTTTC------
---------------------ACGGCTCTC------------------------------

--TCGCACACAGAC
AGCATCTAT--AAGAATAAC
CCCCGC---CAGGAC---
------------------ACTATGTACATG------------------------------
------------------------ACC---AACAAGTCTGTC---ATTGTGAAA------
---------CCAGAACCTGTGTAT------GGAACAGCA---------------------
---------------------------------ACGTCTAATAGGCAACAC---------

------------GACTCCGGAGACTCCAATTATTCCACA---------------------
---------------------------TACAGA---------------------------
---GGCTCTGCAAGTTAC--

---AGCTCATCAAGGTCATCT---------------------------------------
--AACCCTGGC---

------------CCACCGTTGCCAATG---------------------------------
--------------------------------------AGTCGT---------------T
CA------CGGGCCGAA------AACATCACTCCC-------------------------

-----------AAC---------TCT----------------------------------
--GCTCATTCTGGTCTTATCACG-------------------------------------
-----CCAAACTCTGCTGCCTCATGT---TATTCTCCC----------------------
---AATCCTCAATAT-
>XP_027209493_Penaeus_vannamei_Bib
ATGGCCAGTGCAGTGATGGGCGGCGGTGGAGGCGGCGTGGGCGGCGGCGGCGGA------

```
------------------------------------------------GGGGCGGCCAAC
GTGGAGGCCCAGCTGGTGGCCCTCCTCGACAAGCTGGAGGGCCTCCGGCAGGAC------
------------------------------------------------------------
---------------------------------------------------------TCG
GCGCCCCCGTCCCGCCCCCCGATGATCGCCGAGGTGCGAACCCTG---GAGCTGTGGCGC
GCCGTGATCGCCGAGTGCCTGGCCACCGTGTTCTACGTGTTCCTGGTGTGCGGCGCCTAC
AGCCCCTGGACAGGCAAGACGCTCACG---------------------------------
---------------------------------------------------CCGGAGGGC
CAGCTCACCATCTCGCTGGTGGCCGGCATGGCCATGGCCGTCCTCGTGCACAGCTTCGGA
CAGGTCTCCGGAGGTCACGTGAATCCTGCAGTGACCGTGGCTCTGGCAGTGACCCGCCGC
GTGTCCCCCCTGCGCGCCGCCATGTTCGTTTTGGCGCAGCTGGGAGGTGGCATCGCCGGT
GCCGCGCTTCTTTTCGGCGCCACCTCCTCCGGTTACACGGGT------------------
------------------------------GACCTCGGCGCCACGGTGGTGTCCCAGGTC
---------ATCACCCCCTGGCAGGGTCTGGGCCTCGAGTTCCTCCTGACCTTCGTGGTG
GTGTTCGTGGTGTTCGCC---TCCGTCAACCCCTACCGC---------------------
---AGGAGCCTC---------------GGGAACCCCGCTATGGCGATCGGGTTGGCCTAC
CTCGCTTGCACGCTCGTTGGGCTCCCCCTGACGGGCGCCTCCATGAACCCCGCCCGCTCC
CTCGGCCCCGCCTTCGTCAAGAAC---------AAGTGGGACGCGCACTGGGTATACTGG
GTGGCCCCTCTGCTGGGCGGGGCAACGGCCGGCCTCATCTACGAGTACATCTTCAACCCC
---CACAGACTCCCTCGCTCG---------CGAAAGGACTCTATT---GATGGA------
------------------------GAC------------TCGTCAAGTGTTGGCAGTGAT
GAA------GAC---GCGTACGAC---GAG---------TGTTCCAAGCCGCCACCA---
------------------------------------------------AGGTACAATTAC
AATGCGCTC---------AGA---------------------------------------
------GCCCAGCACTATGACCAG---------------TACAGGCCC---GCCAACACT
------------TCAAACACTACCTCTCCACTA------------------GGC------
------TATCCAGTCAGCATGTACGGACACTCT---GGAACACCATCTGTTTACAACGCA
------TCAACTTAC---------------------------------------------
AAGTATGATAGTGGTTTTGTCACCAACAAGGAAGAG---ATCTACTCTGGCTCCAAGAGC
ATG---TAT---GCC------------------AAGTCTCCT---CCTCTGCCACGC---
------------------------------------------------------------
---------------------------TCCTCCTCCCTTAATCGCTCCCAGTCTGTCTAC
ACCAAG---CCGCCACCTCCACGCATG------------GATAACTAC---------TCG
AGGAACCTTGCTCAGTCTCAGAAC---CTGTGCCCGAAGTCTCCTGCAACTTTC------
---------------------ACAGCTCTT------------------------------
------------------------------------------------------------
------------------------------------------------TCCCACAGCGAG
AGTATGTAC------------------------------------------AAGAATAAC
CCACGC---CAGGAC---------------------------------------------
------------------TCCCTTTACATC------------------------------
------------------------ACT---AGCAAGTCTGGC---ATTGTGAAA------
---------CCCGAGCCTGTGTAC------GGGACAGCC---------------------
---------------------------------GCATACAACAAGCAGCAT---------
------------------------------------------------------------
------------------------------------------------------------
------------GACTCAGGTGATTCCAACTACTCAACC---------------------
---------------------------TACCGT---------------------------
---GGCTCTACAGGCTAC------------------------------------------
```


---AGCTCATCGCGCTCCTCT---------------------------------------
--AACCCTGGT---

------------CCTCCCATTCCCATG---------------------------------
--------------------------------------AGCCGC---------------A
CC------CGTCAAGAC------AACCTCACACCT-------------------------

-----------AACTCGAATGTCTCA----------------------------------
--GCCCATTCTGGACTTGTGACA-------------------------------------
-----CCCAACTCCGTGGCTTCATGT---TACTCTCCA----------------------
---AACCCACAGTAC-
>GEUA01058786_Farfantepenaeus_aztecus_Bib
ATGGCCAGTGCAGTGATGGGCGGCGGCGGGGGCGGCGTGGGCGGCGGCGGCGGA------
--GGGGCGGCCAAC
GTGGAGGCCCAGCTGGTGGCCCTCCTCGACAAGCTGGAGGGCCTCCGGCAGGAC------

---TCG
ACGCCCCCCTCCCGCCCCCCGATGATCGCCGAGGTGCGAACCCTG---GAGCTGTGGCGC
GCCGTGATCGCCGAGTGCCTGGCCACCGTGTTCTACGTGTTCCTGGTGTGCGGCGCCTAC
AGCCCCTGGACAGGCAAGACGCTCACG---------------------------------
---CCGGAGGGC
CAGCTCACCATCTCGCTGGTGGCCGGCATGGCCATGGCCGTCCTCGTGCACAGCTTCGGA
CAGGTCTCCGGAGGTCACGTGAACCCCGCAGTGACCGTGGCTCTAGCAGTGACCCGCCGC
GTGTCTCCCCTTCGCGCCGCCATGTTCGTTTTGGCACAGCTGGGAGGTGGCATCGCCGGT
GCCGCGCTTCTGTTCGGCGCCACCTCCTCCGGTTACACGGGT------------------
------------------------------GACCTCGGCGCCACGGTGGTGTCCCAGGTC
---------ATCACCCCCTGGCAGGGTCTGGGCCTCGAGTTCCTCCTGACCTTCGTGGTG
GTGTTCGTGGTGTTCGCC---TCCGTCAACCCCTACCGC---------------------
---AGGAGCCTC---------------GGCAACCCCGCCATGGCCATCGGGTTGGCCTAC
CTCGCCTGCACGCTCGTCGGGCTCCCCCTGACGGGCGCCTCCATGAACCCGGCCCGCTCC
CTCGGCCCCGCCTTCGTCAAGAAC---------AAGTGGGACGCGCACTGGGTATACTGG
GTAGCCCCTCTGCTTGGCGGGACGACTGCCGGCCTCATCTATGAGTACATCTTCAACCCC
---CACAGACTCCCTCGCTCC---------CGAAAGGACTCTATT---GACGGA------
------------------------GAC------------TCGTCAAGTGTTGGCAGTGAT
GAA------GAC---GCCTACGAT---GAG---------TGTTCCAAGCCGCCACCA---
--AGGTACAATTAC
AATGCGCTC---------AGA---------------------------------------
------GCCCAGCACTATGACCAG---------------TACAGGCCC---GCCAACACT
------------TCAAACACTACCTCTCCACTA------------------GGC------
------TACCCAGTCAGCATGTATGGACACACT---GGAACACCGTCTGTTTACAACGCA
------TCAACTTAC---
AAGTATGATAGTGGCTTTGTCACCAACAAGGAAGAG---ATCTACTCTGGCTCCAAGAGC
ATG---TAT---GCC------------------AAGTCTCCT---CCTCTGCCACGC---

---------------------------TCCTCCTCCCTTAATCGCTCCCAGTCTGTCTAC
ACCAAG---CCACCACCTCCACGCATG------------GATAACTAC---------TCA
AGGAACCTTGCTCAGTCTCAGAAC---CTGTGCCCCAAGTCTCCTGCAACTTTC------
---------------------ACAGCTCTT------------------------------

--TCCCACAGCGAG
AGCATGTAC--AAGAACAAC
CCACGC---CAGGAC---
------------------TCCCTTTACATC------------------------------
------------------------ACT---AGCAAGTCTGGC---ATTGTGAAA------
---------CCCGAGCCTGTGTAC------GGGACAGCC---------------------
---------------------------------GCATACAACAAGCAACAT---------

------------GACTCAGGTGATTCCAACTACTCAACC---------------------
---------------------------TACCGT---------------------------
---GGCTCAACAGGCTAC--

---AGCTCATCACGCTCCTCC---------------------------------------
--AACCCTGGT---

------------CCTCCCATTCCCATG---------------------------------
--------------------------------------AGCCGC---------------A
CC------CGTCAAGAC------AACCTTACACCT-------------------------

-----------AACTCGAATGTCTCA----------------------------------
--GCCCATTCTGGACTTGTGACA-------------------------------------
-----CCCAACTCTGTGGCTTCATGT---TACTCTCCA----------------------
---AACCCGCAGTAC-
>GETT01112222_Meganyctiphanes_norvegica_Bib
ATGTCGGGCGGTGGTGTACCAGGGGCGGGAGGAGGC------GGCGTGGGTGGT------
--AGTGATCCGGGC
GTGGAGGCCCAACTCGCCCTCCTGCTGGAGAAGCTGGAGGCAGGGCGTCGGGAC------

--CATGCC
GCTGCCATAGGGCGTCCTGCCATGATTGCTGAGGTCCGTACACTG---GAGCTTTGGCGT
GCAGTTATAGCAGAGTGTTTGGCTACCGTGTTTTATGTGTTCCTTGTGTGCGGGGCCTAC
AGCCCCTGGACAGCAAAGACGCCTACG---------------------------------
---CCTGAAAACGGCGTA
CAGCTCAACATTGCTATTGTTGCTGGGTTCGCTATGGCAGTACTCATACACAGCTTTGGA
CAGGTGTCTGGTGGGCATATAAATCCAGCTGTGACAGTGGCTTTGGCATGTGCACGTCGA
GTGTCACCACTCCGTGCAGCCATGTATATACTGGCCCAGCTTGGTGGCGGTATTGCTGGT
GCTGCGCTTTTATATGGGGCGACGTCTTCCGGGTACAACACT------------------
------------------------------GACCTGGGTGCGACGGTGCCTCGGCTGGGC
---------GTGACGCCCCTGCAGGCGTTCGGGCTTGAGTTCCTACTGACGTTTGTGGTG

```
GTGTTCGTTGTGTTCTCC---TCAGTGAACCCCTACAAA---------------------
---CGCAGCTTC---------------GGTAGTCCGGCCACCTCCATAGGTCTGGCGTAC
CTCTCCTGTACACTGGTTGGGTTCCCCCTGACTGGTGCATCAATGAACCCGGCACGATCG
CTGGGTCCTGCCTTCGTCATGAAC---------AAATGGGACGTGCATTGGGTATACTGG
TGCGCCCCTCTGCTGGGAGGTATTCTGGCTGGTCTGATATATGAGTACATCTTCAACCCA
---CATCGCACGCCAAGGTCC---------AGGAAGGATTCACTA---GACGGA------
------------------------GAT------------TCATCAAGTGTCGGTAGTGAT
GAG------GAT---GCCTATGAT---GAG---------TGCTCCAAGCCACCACCA---
------------------------------------------------CGCTACAACTAC
AATGCACTC---------AGA---------------------------------------
------GCACAGCATTATGATCAG---------------TACCGCCCG---GCCAACACA
---------------CACACAACATCTCCACTT------------------GGG------
------TTCCCATCCAGCGTATATGGGCAATCC---ACAGCCACTTCAGTTTACAATGGC
------GCCAGCTAC---------------------------------------------
AAATTTGATGGAGGCTTTGGGGGAAATAAGGAAGAG---ATTTACTCTGGCTCCAAAAGT
CTT---TAT---ACA------------------AAATCACCT---CCACTCCCACGA---
------------------------------------------------------------
---------------------------TCAACTTCTCTTAATCGTTCCCAGTCTGTGTAC
ACAAAA---CCTGCACCACCAAGAACT------------GATAATTAT---------TCA
AGGAATTTAGTGAGTGCACAGAGT---ATGAGTGCAAAATCTGCTGGCGACTTC------
---------------------GCAGTTCTA------------------------------
------------------------------------------------------------
------------------------------------------------TCTCATACAGGC
AGTATGTAC------------------------------------------AAAAATAAC
ACACGA---AACGAT---------------------------------------------
------------------TCTTTGTATGTA------------------------------
------------------------ACA---AGTAAATCTGGG---ATTGTAAAG------
---------CCTGAACCTGTGTAT------GGAGCAAGT---------------------
---------------------------------CCATATGGAAATCAGAAT---------
------GTAGAATCATCCTATGTTAAC---AAG---GGC---------------------
------------------------------------------------------------
------------GACTCAGGTGATTCCAGCTACTCCACA---------------------
---------------------------TACCGA---------------------------
---GGTTCCACAAGTTAT------------------------------------------
------------------------------------------------------------
------------------------------------------------------------
---AGTTCCTCACGCTCCTCT---------------------------------------
------------------------------------------------GGTCAGAAT---
------------------------------------------------------------
------------GGTCCGGTACCCATG---------------------------------
--------------------------------------AGCCGC---------------A
GC------CGTAATGAT------GCCATCACTCCC-------------------------
------------------------------------------------------------
------------------------------------------------------------
------------------------------------------------------------
-----------AACTCCATTATTTCG----------------------------------
--GCCCATTCTGGTTTAGTCACA-------------------------------------
-----CCCAGCTCCACCGCCACTTGC---TACTCGCCA----------------------
```

---AACCCTCAATAT-
>GFCS01141713_01407319_Euphausia_superba_Bib

---------------------------------------TTCCTGGTATGTGGTGCCTAC
AGCCCCTGGACAGCCAAGACACCGACT---------------------------------
---CCTGAAAACGGTGTT
CAGCTCAACATTGCTCTTGTGGCTGGTTTTGCCATGACTATCCTCGTCCATAGCTTCGGC
CAGGTGTCAGGTGGTCACGTAAACCCAGCTGTGACAGTAGCCCTGGCATGTGCTCGTAGA
GTGTCACCACTCAGAGCGGCTATGTACATCCTGGCCCAGCTTGGAGGCGGCATTGCTGGA
GCTGCTCTGCTATATGGGGCGACGTCCTCCGGGTACAACACG------------------
------------------------------GACCTTGGAGCAACAGTGCCTCGCCTCGGT
---------GTGAGCCCACTACAGGCCTTCGGTCTGGAGTTCCTCTTGACGTTCGTGGTG
GTGTTTGTGGTGTTCGCC---TCTGTCAACCCGTACAAA---------------------
---CGCAGCTTC---------------GGTAATCCGGCCACAGCAATAGGCCTGTCTTAC
CTGTCATGCACACTAGTAGGGTTCCCACTGACGGGTGCTTCAATGAATCCTGCCCGATCG
CTGGGCCCTGCCTTCGTAATGAAC---------AAATGGGATGTACATTGGGTATACTGG
TGCGCCCCTCTCCTGGGAGGTATTTTGGCCGGTTTGATCTACGAGTACATCTTTAATCCA
---CATCGCACACCAAGGTCC----------AGAAAAGATTCACTA---GACGGA------
------------------------GAT------------TCATCAAGCGTTGGTAGTGAT
GAG------GAT---GCTTATGAC---GAA---------TGTTCTAAGCCACCGCCA---
--CGCTACAACTAT
AATGCACTA---------AGA---------------------------------------
------GCCCAGCATTATGACCAG---------------TACCGCCCC---GCCAACACA
---------------AATACAACATCCCAATT-------------------GGT------
------TTCCCATCTAGCGTGTATGGACAATCA---ACAGCTAATTCAGTTTACAATGGC
------CACAGCTAT---
AAGCTTGATGGAGGGTTCGTAGGAAATAAGGAAGAT---ATATACTCTGGATCAAAAAGC
CTT---TAC---ACT------------------AAATCACCT---CCACTTCCAAGG---

---------------------------TCAACTTCTCTTAATCGTTCACAGTCTGTGTAC
ACTAAG---CCGGCCCCACCACGGACC------------GATAACTAT---------TCA
AGGAATTTAGTAACTGTACAGCAGGGCATGACCGCTAAATCGGCCAGTGACTTT------
---------------------ACAGTTCTC------------------------------

--TCTCATACAGCC
---AAAAACAAC
ACTCGA---AATGAT---
------------------TCATTGTATGTG------------------------------
------------------------ACA---AGCAAGTCAGGG---ATTGTAAAA------
---------CCTGAACCTGTTTAT------GGAGCAAAT---------------------
---------------------------------CCTTATGGAAATCAAAAA---------
------ATAGAATCGGCATATGTAAAC---AAA---GGC---------------------

------------GACTCGGGTGATTCAAGCTACTCCACA---------------------
---------------------------TACCGG---------------------------
---GGCTCCACAAGCTAT--

---AGTTCATCCCGCTCCTCT---------------------------------------
--GGGCAGAAT---

------------GGTCCGGTCCCGATG---------------------------------
--------------------------------------AGCCGC---------------A
GC------CGTAACGAT------GCCATCACTCCC-------------------------

-----------AACTCCATTGCCTCC----------------------------------
--GCCCATTCAGGATTGGTAACG-------------------------------------
-----CCAAGCTCAAACGCCACATGT---TACTCGCCA----------------------
---AACCCTCAGTAT-
>GFFH01012460_Oratosquilla_oratoria_Bib
ATGGCAAGTACG--
--AGCACC------
ATCGAATCGCAACTGCTGATATTACTCGAAAAGTTGGATGAAGCCTCGCGTAGCAGG---

--AATGGCGAAGGAAGCTCA
CTCATTCCTCCAAGGCCTCCGATGATAGCGGAGGTTAGAACTCTG---GAGTTGTGGAGG
GCTGTCATTGCTGAGTGTTTAGCCACCGTCTTCTTCGTGTTCATGGTGTGCGGGGCCTAT
TCTCCGTGGACAGGGAAGACTCCATCA---------------------------------
--GAT---GATGTA
CAACTGACCATCGCCTTGGTGGCTGGGTTTTCGATGGCTGTACTCGCCCACAGCTTTGGA
CAGGTGTCAGGCGGACACTTGAACCCGGCTGTGACCTTGGCACTGGCCGTGACACGTCGC
GTGTCACCACTCCGGGCCGCCATGTATGTTCTGGCACAACTTGGCGGCGGCATCGCTGGA
GCTGCGCTTCTCTACGGGGCCACGTCGTCGGGCTACGCGGGC------------------
------------------------------GAGTTGGGCGCCACGGTCCTCAGCCGCGTC
---------ATCACGCCTTGGCAGGGCTTCGGCATCGAGTTCCTCCTTACCTTTGTCGTC
GTCTTCGTCGTCTTCGCC---TCCGTCAACCCCTACAGG---------------------
---AGGACGCTC---------------GGGTCGCCGGCCGTGGCCGTGGGTCTGGCCTAC
CTGGCCTGCACCTTGGTCGGGCTGCCTCTCACAGGGGCTTCTATGAATCCGGCTCGCTCC
CTGGGCCCTGCCTTCGTCATGAAC---------AAGTGGGATGCACATTGGGTATACTGG
GTGGCTCCTCTCTTAGGTGGAACCACAGCAGGCCTGATCTACGAATACATATTCAACCCT
---CATCGTGCTCCTAGGTCA---------AGAAGGGACTCTACT---GACGGA------
------------------------GAC------------TCGTCGAGTGTGGGCAGTGAG
GAA------GAT---GCTTATGAT---GAG---------TGCTCCAAGCCACCCCCG---
--CGCTATAATTAC
AATGCCCTC---------AGA---------------------------------------
---GCCGCCCATCACTACGACCAA---------------TACCGTCCT---GCCAACACC
------------TCCACAACTGCATCCCATTA-------------------GGT------
------TACACAACAAGTGTGTATGGCCCAGCG---GGTGCTGCCTCAGTGTACAGCACT
------CCCCCTTAC---

AAATGTGATGGATCCTATGGTGTCAACAAAGATGAA---ATCTACTCAGGCTCTAAGAGT
CTT---TAT---GCC------------------AAGTCTCCC---CCTATGCCTCGT---

---------------------------TCTGCCTCTTTGACACGTTCCCAGTCTGTATAT
ACCAAG---CCACCACCACCTCGAACT------------GACAACTAC---------ATG
CGCAACATTGGACAGACACAAAGC---ATTTACAACAAATCTCCTGGAGGATTC------
---------------------ACTGCCTTG------------------------------

--TCTCGAACAGAC
AGCATGTATGCA------------------------------------ACCAAGAATAAC
CCTCGT---CAGGAG------ATA---GGATCT---------------------------
------------------ACCCTTTATGTT------------------------------
------------------------GCA---AACAAACCCATG---ATTGTTAAG------
---------CCTGAACCTGTGTAT------GGTATATCTCAACCCCAGCCCCAGCATAAC
---------------------------------ACCCTTCCCAAGCAGCAGCAGCAGCAG

------------GACTCTGGTGACTCCTCTTATTCAACT---------------------
---------------------------TATCGT---------------------------
---------GGCAGCTAC--

---AGCTCTTCCCGTTCATCT---------------------------------------

------------GGTCCAATGCCTATG---------------------------------
--------------------------------------AGCCGT---------------A
AT------CGTGCAGAT------GCCCTTACACCA-------------------------

-----------AACTCTGGCTTGTCA---------------------------GGA----
--GGACATTCGGAAATGCACACA-------------------------------------
-----CCTAATTCCATTGCATCATAC---CATTCCCCA----------------------
---AATCCACAGTAT-
>HAEU01040919_Proasellus_coiffaiti_Bib
ATGTCGACTGGGGGCATG--
--AATGGTCCAGGG
GTTGAAGCCCATTTGATATCTCTGTTAGAGCGTCTGGAAGAACGAGAAGGACCA------

--TCTGAA
AATGTTAAGAGAAAAATTAATATGATGGCGGAGGTTCGAACTCTT---GAACTTTGGAGG
GCTGTGATTGCCGAATGTCTGGCATCTGTTTTTTTCGTGTTCTTAGTCTGTGGTTCCTAC
AGTCCCTGGATGGGAAAAATTATTTCT---------------------------------
---CACGAACCAGAAGCC
AGAATTGTGGTTGCTCTGGTGTGTGGATTCTCAATTGCAATTCTTGTACATAGCTTCGGA
CAGGTATCTGGAGGACACTTAAATCCGGCTGTGAGCATTGGATTCGCTATAACGAGAAGG
ATATCGCCTTTGAGAGCATCTATGTTTATTTTAGCACAGTTAGGAGGTGGCATTGCTGGC

```
GCTGCTTTACTCTATGGAGCAACCTCTTCTGGGTATAGTGGT------------------
------------------------------AACTTGGGTGCCACAGAAATCTCAGGGGCA
---------ATTTCTGTTTGGCAGGGATTTGGTGTTGAGTTTTTGCTCACTTTAATAGTT
GTGTTCGTCGTATTCTCC---TCCTACAACCCCTACCGG---------------------
---AAGTCTTCCTTC------AGTGCTGGTTCTCCTGCAGTGATTACTGGCCTCGCTTAT
CTTGCAACTACATTAGTTGGGTTCCCACTAACAGGTGCGTCCATGAATCCCGCTCGTTCT
TTAGGACCCGCCTTCGTTATGAAC---------AAATGGAGCGCCCACTGGGTGTACTGG
GTCGCCCCCCTGTTTGGTGGAAGTACCGCCGCTCTCATTTACGAGTACATCTTCAATCCT
---CATCGAGCTGCCAGAATC---------AGGAAGGAATCTGTA---GATGGT------
------------------------GAT------------TCATCGAGTGTGGGAAGTGAG
GAG------GAT---GCGTACGAT---GAG---------TGCTCGAAACCACCTCCG---
------------------------------------------------AGATACAATTAC
AACGCCTTG---------AGG---------------------------------------
------ACCCAACACTATGACCAG---------------TATCGTGCC---GCTAGCACC
---------------AATACTCATTCTCCTTCA------------------GGC------
------TATGCTTCTAGTGTCTATGGTCCTACT---AGCAACGCATCTGTCTACAATGCC
------CCATCCTAT---------------------------------------------
AAACTAGTATCAGGCTACGCC------AAGGACGAG---ATCTATTGTGGTACCAAAAGT
TTG---TAT---GCG------------------AAATCTCCT---CCGTTGCCTCGC---
------------------------------------------------------------
---------------------------TCTACGAGTTTGAACCGATCTCAGTCTGTATAT
ACTCGC---CCTCCGCCACCTAGAACA------------GACAACTAC---------TCC
AGGAACTTAGCTCAGTCTCAGAAT---ATAAGTGCCAAATCTCCCGGAAGCTTC------
---------------------ACTGCTCTTCTG---------------------------
---------------------CAACAGCACCAACAGCAA---CAACAA------------
------------------------CAACAGCAACAAACT---------CTTGAAAACAAT
ACCCCATACTCT---------------------------------------AAAAATAAC
ATGCGA---CAAGAT---------------------------------------------
------------------TCTCTCTATGTT------------------------------
------------------------ACT---AGCAAGCCTGGA---CTGGTCAAA------
---------CCTGAACCCGTTTAT------GGTGTTGGTTTG------------------
---------------------------------AGTTATCCGAAGCAAACA---------
------------------------------------------------------------
------------------------------------------------------------
------------GATTCTGGAGACTCCACTTATTCAACT---------------------
---------------------------TACCAC---------------------------
---GGTTCTGGCAGCTAT------------------------------------------
------------------------------------------------------------
------------------------------------------------------------
---AGTTCTTCTCGCTCATCA---------------------------------------
------------------------------------------------GCCGGTCCA---
------------------------------------------------------------
------------CCCCCATTGCCTGCT---------------------------------
--------------------------------------AGCAGA---------------A
CC---CACAGGAACGAC------TTGCATACTCCT-------------------------
------------------------------------------------------------
------------------------------------------------------------
------------------------------------------------------------
```

-----------AATTCTATCGTCTCG----------------------------------
--GGCCACTCTGGGCTTATTACT-------------------------------------
-----CCTAGCTCGATGGCATCTTAT---CACTCTCCA----------------------
---AATCCTCAATAT-
>HAFF01021611_Proasellus_ortizi_Bib
ATGTCGAGTGGTGGCATG--
--AATGGTCCAGGG
GTGGAAGCCCATTTGATATCTCTGTTGGAACGTCTGGAAGAGAGAGAAGGACCTTCA---

---GTTTCAGAA
AATGTTAGAAGAAAAATGAATATGATGGCGGAGGTTCGAACCCTT---GAACTTTGGAGG
GCCGTGATTGCCGAATGTCTGGCCTCAGTATTTTTCGTGTTCTTAGTTTGTGGCTCTTAC
AGTCCGTGGATGGGAAAAATTATTTCT---------------------------------
---CACGAACCGGAAGCC
AGAATAGTGGTTGCCCTGGTGTGTGGATTCTCCATTTCAATTCTTGTACATAGCTTTGGA
CAGGTATCTGGTGGTCACTTAAATCCTGCTGTTACCATTGGATTTGCTATAACAAGAAGG
ATATCACCTTTGAGGGCTTCGATGTTTATTTTAGCACAATTAGGTGGTGGCATTGCTGGC
GCTGCTTTACTTTATGGAGCGACATCTTCAGGGTACAGTGGT------------------
------------------------------AACTTGGGTGCCACAGAAATCTCGGGGGCA
---------ATCTCAGTTTGGCAGGGATTTGGTGTTGAGTTCTTACTTACTCTGGTAGTT
GTTTTCGTAGTCTTTTCC---TCCTACAACCCCTACAGA---------------------
---AGGTCGTCTTTT------AGTGCTGGTTCACCTGCAGTGATTACAGGCCTCGCTTAT
CTTGCTACTACACTAGTTGGGTTCCCACTTACGGGTGCCTCAATGAATCCAGCCCGTTCC
TTAGGACCCGCCTTCGTTATGAAC---------AAATGGAACTTCCACTGGGTGTACTGG
GTAGCCCCTCTGTTGGGCGGAATTACTGCTGCCCTTATTTACGAGTATATTTTTAACCCT
---CATCGCGCTGCTAGAATC---------AGGAAAGAATCTGTT---GATGGT------
------------------------GAT------------TCATCGAGTGTGGGAAGTGAG
GAA------GAT---GCGTATGAT---GAG---------TGCTCGAAACCACCTCCG---
--AGATACAATTAC
AATGCCCTA---------AGG---------------------------------------
------ACACAACACTATGACCAA---------------TATCGTGCT---ACCAGTGCC
---------------AACACTCATTCGCCTTCT------------------GGC------
------TATGCTTCTAGTGTGTATGGTCCTACT---AGCAACGCATCTGTCTACAATGCC
------CCGTCCTAT---
AAATTAGTCTCAGGTTATGCA------AAGGACGAG---ATCTATTGTGGAACCAAAAGT
TTG---TAC---GCA------------------AAATCTCCT---CCTTTACCGCGC---

---------------------------ACGACGAGTTTAAACCGATCTCAATCTGTATAT
ACTCGC---CCTCCGCCGCCAAGGACA------------GATCACTAT---------TCG
AGGAATTTAGCTCAATCTCAGAAT---CTTAGTGCCAAATCTCCCGGAAGCTTC------
---------------------ACAGCTCTTTTG---------------------------
------CAGCATCATCAACAACAGCAGCATCAACAACAG---CAACAA------------
------------------------CAGCAACAGCAAACT---------CTTGATAACAAT
ACTCCATACTCT---------------------------------------AAAAATAAT
ATGCGA---CAAGAC---
------------------TCTCTGTATGTT------------------------------
------------------------ACT---AGCAAGCCTGGA---CTGGTCAAA------
---------CCAGAACCCGTTTAT------GGTGTTGGATTA------------------

```
---------------------------------AGTTATCCAAAACAAACC---------
------------------------------------------------------------
------------------------------------------------------------
------------GATTCTGGAGACTCTACTTATTCAACA---------------------
---------------------------TATCAT---------------------------
---GGTTCTGGCAGCTAT------------------------------------------
------------------------------------------------------------
------------------------------------------------------------
------AGTTCTCGCTCGTCG---------------------------------------
------------------------------------------------GCTGGTCCA---
------------------------------------------------------------
------------CCTCCATTGCCTGCT---------------------------------
--------------------------------------AGTAGA---------------A
CA---CATAGGAACGAT------TTGCATACTCCA-------------------------
------------------------------------------------------------
------------------------------------------------------------
------------------------------------------------------------
-----------AATTCTATTATCTCA----------------------------------
--GGCCACTCGGGTCTTATTACT-------------------------------------
-----CCCAGTTCGGTGGCATCATAT---CACTCTCCA----------------------
-----------------------------------------AATCCTCAGTAC-
>HAEN01049747_Bragasellus_peltatus_Bib
ATGTCCAGTGGTGCCGTA------------------------------------------
------------------------------------------------AATCTGGGTGGG
GTGGAAGCCCAACTTATGTCCCTTTTAGAGCGCCTTGAAGAAAGGGATTCCGCC------
------------------------------------------------------------
------------------------------------------------------CCCGAA
AACTCTCGCCGAAAAATTTCCATGATGGCTGAGGTTCGAACCCTG---GAACTCTGGAGG
GCCGTAATTGCGGAATGTCTGGCGACCGTCTTTTTCGTGTTCCTGGTTTGCGGCTCCTAC
AGTCCGTGGATGGAAAAATC---ACG----------------------------------
---------------------------------------------CATGAACCGGAGGCG
AGGATCGTTGTGGCTTTGGTGTGTGGATTCTCCTTAGCCATCCTTATCCATAGTTTCGGA
CAGATATCTGGCGGACACTTGAATCCTGCTGTGAGCATTGGATTCGCCATCACGAGAAGA
GTGTCCCCATTGCGAGCGTCCATGTTTGTTTTAGCTCAGCTGGGAGGAGGTATTGCTGGT
GCTGCCTTGCTGTATGGAGCGACCTCTTCTGGGTACAGCGGA------------------
------------------------------AACTTGGGGGCCACGGAAATATCCGGAGCT
---------ATCTCAGTTTGGCAGGGCTTCGGGGTCGAATTTCTGCTTACTTTGGTCGTG
GTTTTCGTGGTTTTCTCC---TCGTACAACCCTTATCGG---------------------
---AGGTCCTCGTTT------AGCGCTGGATCGCCCGCCGTAATCACCGGGTTGGCTTAC
CTTGCCGCTACGCTAGTTGGATTCCCCTTGACCGGTGCGTCCATGAATCCAGCTCGATCA
CTGGGACCGGCATTCGTTATGAAT---------AAATGGACAGCTCACTGGGTGTACTGG
GTAGCCCCCCTTTCCGGTGGCGTTACTGCCGCTCTGATATACGAATATATATTCAATCCC
---CACCGGGCAGCCAGAACC---------AGGAAAGAGTCGGTG---GATGGC------
------------------------GAT------------TCTTCGAGTGTGGGCAGTGAG
GAG------GAC---GCTTACGAC---GAG---------TGTTCGAAGCCGCCTCCA---
------------------------------------------------AGATACAGTTAC
AACGCTCTG---------CGG---------------------------------------
------CCCCAACAGTATGACCAA---------------TATCGCCCC---ACCAGCACC
```

---------------AACACCCACTCACCTTCA------------------GGT------
------TTCGCATCCAGCATGTACGGCCCGGCT---GGCGGTGCGTCCGTCTACAACGCC
------TCTTCGTAC---
AAACACGCAGCAGGATATACC------AAAGACGAG---ATCTATTGTGGGTCGAAAAGT
TTG---TAC---GCG------------------AAATCTCCT---CCGCTGCCTCGC---

---------------------------TCGACAAGCTTGAATCGCTCCCAGTCTGTTTAT
ACGCGA---CCTCCCCCACCTAGAACA---------GAGGGAAACTAC---------TCT
AGAAATTTAGCTCAGTCTCAGAAT---CTTAGCACGAAATCGCCTGGTAGCTTC------
---------------------ACTGCTTTATTG---------------------------
---------------------CAACAGCAACAACAGCAG---CAGCAG------------
------------------------CAGCAACAGACGCTG---------CTGGAGAACAGT
ACACCGTACTCG---------------------------------------AAGAACAAC
ATGAGG---CAAGAC---
------------------TCCTTATACGTC------------------------------
------------------------ACT---AGTAAGCCTGGA---TTAATCAAG------
---------CCCGAACCAGTTTAT------GGAGTGGGATTA------------------
---------------------------------ACATATCCCAAACAGCCA---------

------------GATTCTGGAGACTCCACTTACTCAACT---------------------
---------------------------TACCAT---------------------------
---GGTTCTGGTAGCTAT--

---AGTTCTTCACGCTCATCA---------------------------------------
--GCAGGTCCT---

------------CCTCCATTACCCGCT---------------------------------
--------------------------------------AGTAGA---------------A
GC---CACAGAAACGAA------ATGCATACTCCA-------------------------

-----------AACTCTGTTGTGTCT----------------------------------
--GGCCACTCTGGGCTCATCACT-------------------------------------
-----CCTAGCTCAATGGCATCATAC---CATTCGCCA----------------------
---AATCCTCAGTAC-
>GJCF01039890_Asellus_hilgendorfii_Bib
ATGTCAGGAGGGGGCATG--
--AGC---CACAGCGGGGGC
GTTGAGGCCCAACTGGTTTCCCTGTTGGAACGCCTGGAAGAAAGGGAAGTGGCC------

--CCCGAA
ACCTCCCGAAGGAAGATGACCATGATGGCCGAAGTGCGAACCCTG---GAGCTCTGGCGG
GCCGTGATCGCGGAGTGCCTGGCCACGGTCTTCTTCGTGTTCCTGGTCTGCGGTTCGTAC
AGCCCGTGGATGGGCAAAATCGTCTCC---------------------------------
---CACGAACCGGAAGCG

```
AGAATCGTCGTGGCTCTGGTGTGCGGATTCTCATTGGCTATTCTCGTTCACAGCTTTGGA
CAGGTTTCCGGCGGACATCTCAATCCCGCTGTGAGCATCGGGTTCGCCATCACGAGGAAA
GTTTCCCCCCTTCGTGCCTCCATGTTCATCTTGGCTCAACTCGGAGGAGGCATCGCGGGA
GCTGCTCTGCTTTATGGGCAACGTCATCAGGCTACAGTGGT-------------------
------------------------------AACTTAGGAGCTACAGAAATCTCAGGGGCG
---------ATCTCCGTGTGGCAGGGCTTCGGGGTAGAATACCTCCTGACCTTGGTAGTG
GTCTTTGTCGTCTTCTCC---TCCTACAACCCCTACAGG---------------------
---AGATCTTTATTT------AGCACCGGTTCTCCTGCAGTAATCACTGGCTTAGCTTAT
CTGGCAGCAACTTTAGTTGGGTTTCCTCTCACAGGAGCGTCGATGAACCCTGCTCGATCT
CTCGGTCCAGCCTTCGTCATGAAC---------AAATGGAACGGCCACTGGGTGTACTGG
GTGGCTCCGCTGTCTGGGGGGATCACTGCAGCCCTCATTTACGAATATATCTTTAATCCC
---CACCGAGCTGCCAGAACC----------AGGAAGGAATCCGTT---GACGGA------
------------------------GAT------------TCTTCGAGTGTGGGCAGTGAG
GAG------GAT---GCGTATGAT---GAG---------TGTTCGAAGCCGCCCCCG---
------------------------------------------------AGATATAATTAC
AACGCCCTT---------CGA---------------------------------------
------ACTCAACATTATGACCAA---------------TACCGCCCA---ACGAGTACC
---------------AACGCCCATTCGCCCTCA------------------GGT------
------TACGCATCTAGTGTGTATGGCCCTACGACTGGAACTGCATCAGTGTACAACGCT
------CCTTCTTAC---------------------------------------------
AAACTCGTGTCTGGCTACGGC------AAGGACGAA---ATCTACTGCGGAACCAAGAGC
TTG---TAC---GCC------------------AAGTCTCCT---CCACTTCCTCGA---
------------------------------------------------------------
---------------------------TCTACGAGTTTGAATCGATCACAGTCTGTTTAC
ACCCGT---CCTCCGCCCCCCAGGACT------------GATAACTAC---------TCC
CGGAATTTAGCCCAGTCACAGAAT---CTTGGCGGAAAATCGCCTGGCAGCTTC------
---------------------ACAGCACTCCTA---------------------------
------CAACAACAGCAACAGCAGCAGCAACAACAGCAACATCAACAA------------
------------------------CAGCAACAGCAAGCT---------CAAGAGAATAAC
ACTCCGTACTCC---------------------------------------AAAAATAAC
ATGCGC---CAGGAT---------------------------------------------
------------------TCGCTCTACGTC------------------------------
------------------------ACG---AGCAAGCCAGGG---CTCGTTAAA------
---------CCAGAACCTGTTTAT------GGAGTTGGTCTAAACATGAGT---------
---------------------------------AACTACCCAAAACAACCT---------
------------------------------------------------------------
------------------------------------------------------------
------------GATTCTGGAGACTCAACTTATTCGACC---------------------
---------------------------TACCAC---------------------------
---GGCTCTGGTAGCTAC------------------------------------------
------------------------------------------------------------
------------------------------------------------------------
---AGTTCCTCACGATCGTCT---------------------------------------
------------------------------------------------GGTGGCCCC---
------------------------------------------------------------
------------CCTCCATTACCAACG---------------------------------
--------------------------------------AGCAGA---------------A
AC---CACAGGAACGAA------TCGCATACCCCA-------------------------
```


-----------AATTCCATTATCTCC---------------------------------
--GGTCACTCTGGTCTCATCACT-------------------------------------
-----CCCAGCTCAATGGCGTCGTAT---CATTCCCCA----------------------
---AATCCACAGTAT-
>GEQS01046189_Ommatogammarus_flavus_Bib
ATGGGC--
--TCCTATAACTCAGAG---
TTGGAGAACCAGTTGTGGGCATTGTTATCTAAACTGGAAAATGGTGCCCCTGGTTGTGAC
TTGGAC--
--GGTAGAGGGGGCAGGCCC
AACCTGCGTTGGGGATTCGCCATGCTCGCCGAACTGAGGACCCTA---GACCTGTGGCGC
GCTGTAATAGGCGAGTGTTTGGCTGTGGTGTTCTACGTGCTGCTGGTGTGTGGTGCCTAC
AGTCCGTGGGCAGGGCAGCCTACTCCT---------------------------------
---CCAGAGAGA
CATCTAGTCATCTCATTGGTGGCAGGGTTCGCGATGACGGTGCTTGTGCACTGTTTCTGT
CAAGTGTCCGGGGGTCACGTCAACCCCGCCGTCACCATAGCCTTGGCCGCCATGCGTCGC
GTGTCGCCCCTCAGGGCCGCCCTGCTGGTGCTGGCTCAGCTGGGTGGAGGTATCGCGGGG
GCAGCGCTCCTCTTCGGGGCGACCTCCTCCGGCTACAGCGGG------------------
------------------------------GACCTGGGGGCCACTGTAATCTCCCGCGTC
---------ATCACCGTCGGCCAAGGCTTCGGGGTAGAGTTCCTGCTCTCCTTCGTCGTG
GTGCTGGCCTACTTCTCC---GCCATGTGCCCTCACAGG---------------------
---AGGCCTACGGGA------------GGCCATCCAGCCCTTATCGTGGGGCTTGCCTAC
CTCGCCACTACGCTTGTGGGGCTTCCTCTGACGGGGGCCTCCATGAATCCCGCGCGCTCC
CTGGGCCCTGCCTTCGTCATGAAC---------ACCTGGCACGCGCACTGGGTATATTGG
GCGGGCCCCATCTTGGGCGGCCTAACTGCCGGGTTCATCCACGAGTTCATCTTTAACCCC
---CGCAGATCCCCCCTCTCC---------CGCAACCACTCCACA---GATGGA------
---GGTGAGGGTGGCGACTGCAGCGAC------------GGCGTCGGTGTGGATGAGGAG
GAA------GGC---GCTCTGCGG---CAGGCC---ACAGTGACGTCATCGTCGCAGTCC
GGT-----------------------------------GGCAGCGCGCATGCGCAGTAC
AACCCACTGCGC------AGA---------------------------------------
------CATGCGGCGAGAAGCCAC---------------GCCCACCCT---CGTCAT---
------------AACTCCGCCTCTAGCCCTCCG---------------ACTGGGGAT---
--CAG---
---------ATATAC---
TCTGCTGAGAGCGTGTACGGC------TACGACGACTACATCTACAGCGGCAGTAAAAGC
CTC---TAC---------------------------ACC------CCCGCCTCCAGG---

---------------------------CCCACCAACCTTCACCGCTCACAGTCCGTCTAC
ACGCGC---CCTCTACCGGTGCCCAAC------------GCCGCCCCCACCGCAGCCAAC
CCTTCCGTGGGACCGGTCCAGGAC---CAGAAGCCAACCGTTACTGGTTTGCTGCAT---
---------------------AACAACCTCATG---------------------------
---------------GTCTACAATGCT---CAGCAACAGCAACAAAAC------------
--CAGAGACATCAA
CAGCAGTATCAGCAACAGCTAATG---CAGCAACAGCAT---ATGCCA---AATAACATT
AACAAG---CCTTCG---TCAGTATCGGGATCCGTTTCGCATTTCTCGAAGAACATCAGA

```
AATGTGGCGCCTTCCGAATCGGAATACTCTGTACAATCCGCA------------------
------------------AGAAGAACACCGTTGAAATCCAAC---CTCATCAAA------
---------CCTGAGCCCATCTACAAAACGGGGATCAGCACAAACGGCTCAACCACA---
---------------------------------AACCACAACAATCAAAACCATCTGCAC
CAC---------------------------------------------------------
---CAAATCCAGCACCTGCAACAGCATCTACAGCAACCGGTGGCGTACCAAGTTGACCCG
---AACCTGCAACAGCAGCCTCATCAGACCTACCAGAACACGACGACCCACAGTTCACAG
AGC---TCCAACAACGGG------ACTGAGCGG---------------------------
---TGCTCGAGTGTCCTCAGCGGCATCGGTGTGGCCAAC---------CCTCAC------
------------------------------------------------------------
---------GCAGCGGGAAAGTACAACTCTGCGGCAGAG---------------------
---GAGGACGACGCGAGCCAGTATGGCAACACTGCGAACTACCCACGC---AATTTC---
------------------------------------------------GGTCGTAATAGA
------------------------------------------------------------
------------CCTGAGCTGCCCACTCCT------------------------------
--------------------------------------GGGGGC---------------T
CC---GTGCACTCCGAC------ATAGCT-------------------------------
------------------------------------------------------------
------------------------------------------------------------
------------------------------------------------------------
------------------------------------------------------------
-----------GGGGTT-------------------------------------------
-----CCTAACTCGATGTTGTCCTACTCGCATTACCCTGGA-------------------
--------------------------------ACGCATGGCGACAAGCTATAC-
>GEQM01047324_Macropereiopus_parvus_Bib
ATGGGC------------------------------------------------------
------------------------------------------TCCTATAAAACAGAG---
TTGGAGAACCAGTTGTGGGCATTGTTATCTAAACTGGAAAATGGTGCCCCTGGTTGTGAC
ATGGAC------------------------------------------------------
------------------------------------------GGTAGAGGGGGCAGACCC
AACCTGCGTTGGGGATTCGCCATGCTCGCCGAACTGAGGACCCTA---GACCTGTGGCGC
GCTGTAATTGGCGAGTGTTTGGCTGTGGTGTTCTACGTGCTGCTGGTGTGTGGTGCCTAC
AGTCCGTGGCAGGGCAGCCTACTCCT----------------------------------
---------------------------------------------------CCAGAGAGA
CATTTAGTCATCTCATTGGTGGCAGGGTTCGCGATGACTGTGCTTGTGCACTGTTTCTGT
CAAGTGTCCGGTGGTCACGTCAACCCCGCCGTCACCATAGCCTTGGCCGCCATGCGTCGA
GTGTCGCCCCTCAGGGCCGCCCTGCTGGTGCTGGCTCAGCTGGGTGGAGGTATCGCGGGG
GCAGCGCTCCTCTTCGGGGCGACCTCCTCCGGCTACAGCGGG------------------
------------------------------GACCTGGGGGCCACTGTAATCTCCCGCGTC
---------ATCACCGTCGGCCAGGGCTTCGGGGTAGAGTTCCTGCTATCCTTCGTCGTG
GTGCTGGCCTACTTCTCC---GCCATGTGCCCTCACAGG---------------------
---AGGCCTATGGGA------------GGCCATCCCGCCCTCATCGTGGGGCTTGCCTAC
CTCGCCACTACGCTTGTAGGGCTTCCTCTGACGGGGGCCTCCATGAACCCCGCGCGCTCC
CTGGGCCCTGCCTTCGTCATGAAC---------ACCTGGCACGCGCACTGGGTATATTGG
GCGGGCCCCATCTTGGGCGGCCTAACTGCCGGGTTCATCCACGAGTTCATCTTTAACCCC
---CGTAGATCCCCCCTCTCC---------CGCAACCAATCCACA---GATGGA------
---GGTGAGGGCGGCGATTGCAGCGAC------------GGCGTCGGTGTGGATGAGGAG
GAA------GGC---GCTCTGCGG---CAGGCC---ACAGTGACGTCATCGTCGCAGTCC
```

GGT------------------------------------GGCAGCGCGCATGCGCAGTAC
AACCCACTGCGC------AGA---------------------------------------
------CATGCGGCGAGAAGCCAC---------------GCCCACCCT---CGTCAT---
------------AACTCCGCCTCCAGCCCTCCG---------------CCTGGGGAT---
--CAG---
---------ATATAC---
TCTGCTGAGAGCGTGTACGGC------TACGACGACTACATCTACAGCGGCAGTAAAAGC
CTC---TAC---------------------------ACC------CCCGCCTCCAGG---

---------------------------CCCACCAACCTTCACCGCTCACAGTCCGTCTAC
ACGCGC---CCTCTACCGGTGCCTAAC------------GCCGCCCCCACCGCAGCCAAC
CCTTCCGTGGGACCGGTCCAGGAC---CAGAAGCCAACCGTTACTGGTTTGCTGCAT---
---------------------AACAACCTCATG---------------------------
---------------GCCTACAATGCTCAGCAGCAGCAACAGCAACAA------------

>GEQC01070659_Eulimnogammarus_verrucosus_Bib
ATGGGC--
--TCCTATAACACAGAG---
TTGGAGAACCAGTTGTGGGCATTGTTATCTAAACTGGAAAATGGTGCTCCTGGTTGTGAC
ATGGAC--
--GGTAGAGGGGGCAGACCC
AACCTGCGTTGGGGATTCGCCATGCTCGCCGAACTGAGGACCCTA---GACCTGTGGCGC

GCTGTAATAGGCGAGTGTTTGGCTGTGGTGTTCTACGTGCTGCTGGTGTGTGGTGCCTAC
AGTCCGTGGGCAGGGCAGCCTACTCCT---------------------------------
---CCAGAGAGA
CATCTAGTGATCTCATTGGTGGCAGGGTTCGCGATGACGGTGCTTGTGCACTGTTTCTGT
CAAGTGTCCGGGGGTCACGTTAACCCCGCCGTCACCATAGCCCTGGCCGCCATGCGTCGC
GTGTCGCCCCTCAGGGCCGCCCTGCTGGTGCTGGCTCAGCTGGGGGGAGGTATCGCGGGG
GCAGCGCTCCTCTTCGGGGCGACCTCCTCCGGCTACAGCGGG------------------
------------------------------GACCTGGGGGCCACTGTAATCTCCCGCGTC
---------ATCACCGTCGGACAGGGCTTCGGGGTAGAGTTCCTGCTCTCCTTCGTCGTG
GTGCTGGCCTACTTCTCC---GCCATGTGCCCTCACAGG---------------------
---AGGCCTACGGGA------------GGCCATCCCGCCCTTATCGTGGGGCTTGCCTAC
CTCGCCACTACGCTTGTAGGGCTTCCTCTGACGGGGGCCTCCATGAATCCCGCGCGCTCC
CTGGGCCCTGCCTTCGTCATGAAC---------ACCTGGCACGCGCACTGGGTATATTGG
GCGGGGCCCATCTTGGGCGGCCTAACTGCCGGGTTCATCCACGAGTTCATATTTAACCCC
---CGTAGATCCCCCCTCTCC---------CGCAACCACTCCACA---GATGGA------
---GGTGAGGGCGGCGACTGCAGCGAC------------GGCGTCGGTGTGGATGAGGAG
GAA------GGC---GCTCTGCGG---CAGGCC---CCAGTGACGTCATCGTCGCAGTCC
GGT------------------------------------GGCAGCGCGCATGCGCAGTAC
AACCCACTGCGC------AGA---------------------------------------
------CATGCGGCGAGAAGCCAC---------------GCCCACCCT---CGTCAT---
------------AACTCCGCCTCCAGCCCTCCG---------------CCTGGGGAT---
--CAG---
---------ATATAC---
TCTGCTGAGAGCGTGTACGGC------TACGACGACTACATCTACAGCGGCAGTAAAAGC
CTA---TAC---------------------------ACC------CCCGCCTCCAGG---

---------------------------CCCACCAACCTTCACCGCTCACAGTCCGTCTAC
ACGCGC---CCTCTACCGGTGCCCAAC------------GCCGCCCCCACCGCAGCCAAC
CCTTCCGTGGGACCGGTCCAGGAC---CAGAAGCCAACCGTTACTGGTTTGCTGCAT---
---------------------AACAACCTCATG---------------------------
---------------GTCTACAATGCTCAGCAGCAACAGCAACAAAAC------------
--CAGAGACATCAA
CAGCAGTATCAGCAACAGCTAATG---CAGCAACAGCAT---ATGCCA---AATAACATT
AACAAG---CCTTCG---TCAGTATCGGGATCCGTTTCGCATTTCTCGAAGAACATCAGA
AATGTTGCGCCTTCCGAATCGGAATACTCGGTACAATCCGCG------------------
------------------AGAAGAACACCGTTGAAATCCAAC---CTCATCAAA------
---------CCTGAGCCCATCTACAAAACGGGGATTAGCACAAACGGCTCAACCGCA---
---------------------------------AACCACAACAATCAAAACCATCTGCAC
CAC---
---CAAACCCAGCACCTGCAACAGCATCTACAGCAACCGGTGGCGTACCAAGTTGCCCCG
---AACCTGCAACAGCAGCCTCATCAGACCTACCAGAACACGACGACCCACAGTTCAGAG
AGC---TCCAACAACGGG------ACTGAGCGA---------------------------
---TGCTCGAGTGTCCTCAGCGGCATCGGTGTGGCCAAC---------CCTCAC------

---------GCAGCGGGAAAGTACAACTCTGCGGCAGAG---------------------
---GAAGACGACGCGAGCCAGTATGGCAACACTGCGAACTACCCACGA---AATTTC---
--GGTCGTAATAGA

------------CCTGAGCTGCCCACTCCT------------------------------
--------------------------------------GGGGGC---------------T
CC---GTGCACTCCGAC------ATGGCT-------------------------------

-----------GTGGTT---
-----CCTAACTCGATGTTGTCCTACTCGCATTACCCTGGA-------------------
--------------------------------ACGCATGGCGACAAGCTATAC-
>GEQT01064983_Pachyschesis_branchialis_Bib
ATGGGC--
--TCCTATAACACAGAG---
TTGGAGAACCAGTTATGGGCATTGTTATCTAAACTGGAAACTGGTGCCCCTGGTTGTGAC
ATGGAC--
--GGTAGAGAGGGCAGACCC
AACCTGCGTTGGGGATTCGCCATGCTCGCCGAACTGAGGACCCTA---GACCTGTGGCGC
GCTGTAATAGGCGAGTGTTTGGCTGTGGTGTTCTACGTGCTTCTGGTGTGTGGGGCCTAC
AGTCCGTGGGCAGGGCAACCTACTCCT---------------------------------
---CCAGAGAGA
CATCTAGTCATCTCATTGGTGGCAGGGTTCGCGATGACGGTGCTTGTGCACTGTTTCTGT
CAAGTGTCCGGGGGTCACGTCAACCCCGCCGTCACCATAGCCCTGGCCGCCATGCGTCGC
GTGTCCCCCCTTAGGGCCGCCCTGCTGGTGCTGGCTCAGCTGGGGGGAGGGATCGCGGGG
GCAGCGCTCCTCTTCGGGGCGACCTCCTCCGGCTACAGCGGG------------------
------------------------------GACCTGGGGGCCACTGTGATCTCCCGCGTC
---------ATCACCGTCGGCCAGGGCTTCGGGGTAGAGTTCCTGCTCTCCTTCGTCGTG
GTGCTGGCCTACTTCTCC---GCCATGTGCCCTCACAGG---------------------
---AGGCCCACGGA-------------GGCCATCCCGCCCTTATCGTGGGGCTTGCCTAC
CTCGCCACTACGCTTGTAGGGCTTCCTCTGACGGGGGCCTCCATGAATCCCGCGCGCTCC
CTGGGCCCTGCCTTCGTCATGAAC---------ACCTGGCAAGCGCACTGGGTGTATTGG
GCGGGCCCCATCTTGGGCGGCCTAACTGCCGGGTTCATCCACGAGTTCATCTTTAACCCC
---CGTAGATCCCCCCTCTCC---------CGCAACCACTCCACA---GATGGA------
---GGTGAGGGTGGCGACTGTAGCGAC------------GGTGTCGGTGTGGAGGAGGAG
GAA------GGT---GCTTTGCGG---CAGGCC---CAAGTGACGTCATCGTCGCAGTCC
GGT------------------------------------GGCAACGCGCATGCGCAGTAC
AACCCACTGCGC------AGA---------------------------------------
------CATGCGGCGAGAAGCCAC---------------GCCCACCCT---CGTCAT---
------------AACTCCGCCTCCAGCCCTCCA---------------CCTGGGGAT---
--CAG---
---------ATATAC---
TCTGCTGAGAGCGTGTACGGC------CACGACGACTACATCTACAGCGGCAGTAAAAGC
CTC---TAC---------------------------ACC------CCCGCCTCCAGG---

---------------------------CCCACCAACCTTCACCGCTCACAGTCCGTCTAC
ACGCGC---CCTCTGCCGGTGCCCAAC------------GCCGCCCCCACCGCAGCCAAC
CCTTCCGTGGGACCGGTCCAGGAC---CAGAAGCCAACCGTTACTGGTTTGCTGCAT---
---------------------AACAACCTTATG---------------------------
---------------GTCTACAATGCTCAGCAGCAACAGCAACAAAAC------------

--CAGAGACATCAA
CAGCAGTATCAGCAACAGCTAATG---CAGCAACAGCAT---ATGCCA---AATAACATT
AACAAG---CCTTCG---TCGGTATCGGGATCCGTTTCGCATTTCTCGAAGAACATCAGA
AATGTGGCGCCTTCCGAATCGGAATACTCGGTACAATCCGCG------------------
------------------AGAAGAACCCCGTTGAAATCCAAC---CTCATCAAA------
---------CCTGAGCCAATCTACAAAACGGGGATCAGCACAAACGGCTCAACCGCA---
---------------------------------AACCACAACAATCAAAACCATCTGCAC
CAC---
---CAAACCCAGCACCTGCAACAGCATCTACAGCAACCGGTGGCGTACCAAGTTGACCCG
---AACCTGCAACAGCAGCCTCATCAGACCTACCAGAACACGACGACCCACAGTTCACAG
AGC---TCCAATAACGGG------ACTGAGCGC---------------------------
---TGCTCGAGTGTCCTCAGCGGCATTGGTGTGGCCAAC---------CCTCAC------

---------GCAGCGGGAAAGTACAACTCTGCGGCAGAG---------------------
---GAGGATGACGCGAGCCAGTATGGCAACACTGCCAACTACCCACGC---AATTTC---
--GGTCGTAATAGA

------------CCTGAGCTGCCCACTCCT------------------------------
--------------------------------------GGGGGC---------------T
CC---GTGCACTCCGAC------ATGGCT-------------------------------

-----------GTGGTT---
-----CCTACCTCGATGTTGTCCTACTCTCATTACCCTGGA-------------------
--------------------------------ACGCACGGCGACAAGCTATAC-
>GHVD01629989_Gammarus_fossarum_Bib
ATGGGC--
--TCCTATAACACGGAG---
TTGGAGAACCAGTTGTGGGCATTGTTATCTAAACTGGAAAATGGTGCCCCTGGTTGTGAC
CTGGAC--
--GTGAGGGGGGGCAGGCCT
AACCTGCGCTGGGGCTTCGCCATGCTCGCCGAACTGAGGACCCTC---GACCTGTGGCGT
GCTGTAATAGGGGAGTGTTTAGCGGTGGTGTTCTACGTGCTGCTGGTGTGTGGTGCCTAC
AGTCCGTGGCAGGGCAGCCAACTCCT----------------------------------
---CCTGAGAGA
CATCTAGTCATCTCATTGGTGGCAGGCTTCGCGATGACGGTGCTTGTGCACTGTTTCTGT
CAAGTGTCCGGGGGCCACGTTAACCCTGCCGTCACCATTGCTCTGGCCGCCATGCGTCGC
GTGTCGCCCCTCAGGGCCGTCCTGCTGGTGCTGGCCCAGCTGGGGGGAGGGATCGCGGGG
GCAGCGCTCCTCTTCGGGGCCACCTCCTCCGGCTACAGCGGG------------------
------------------------------GACCTCGGGGCCACCGTCATCTCCCGTGTG
---------ATCACCGTCGGCCAGGGCTTCGGGGTTGAGTTCCTGCTCTCCTTCGTCGTG
GTACTGGCCTACTTCTCC---GCCATGTGCCCCCACAGG---------------------
---AGGCCTACGGGA------------GGTCACCCCGCCCTTATCGTGGGGCTGGCCTAC
CTCGCCACTACGCTTGTGGGGCTTCCCCTGACGGGGGCCTCCATGAATCCTGCGCGCTCC
CTTGGCCCCGCCTTCGTCATGAAC---------ACCTGGCACGCGCACTGGGTATACTGG
GCGGGCCCCATCTTGGGTGGCCTTACTGCCGGGTTCATCCACGAGTTCATCTTCAACCCC

---CGAAGATCCCCCCTCTCT---------CGCAACCACTCAACA---GATGGA------
---GGTGAGGGCGGTGACTGCAGCGAT------------GGTGTTGGTGTGGACGAGGAG
GAG------GGC---GCCCTGAGG---CAAGCA---CCAGTGACGTCATCGTCACAGTCC
GGT------------------------------------GGCAGCGCACATGCACAGTAC
AACCCTCTGCGC------AGA---------------------------------------
------CACGCGGCGAGAAGCCAC---------------GCCCACCCT---CGTCAT---
------------AACTCCGCCTCCAGCCCGCCG---------------CCTGGGGAT---
--CAG---
---------ATATAC---
TCTGCTGAGAGCGTGTGCGGC------TACGATGACTACATTTACAGCGGCAGCAAGAGC
CTC---TAC---------------------------ACA------CCCGCCTCTAGG---

---------------------------CCCACCAACCTCCACCGCTCACAGTCCGTCTAC
ACGCGC---CCTCTGCCGGTGCCCAAC------------GCCGACCCCAACGCAGCCAAC
CCTTCCGTAACCCCGGCCCAGGAA---CAGAAGCCAACAGTTACTGGGTTGCTGCAC---
---------------------AACAACCTCATG---------------------------
---------------GTCTACAATGCTCAGCAACAGCAGCAACAAAAC------------
--CAGAGACATCAA
CAGCAGTATCAGCAACAACTAATGCAGCAGCAACAGCAT---ATGCCA---AACAACATT
AACAAG---CCTTCG---TCAGTATCGGGATCCGTTTCACATTTTTCAAAGAACATCAGA
AATGTGACTCCTTCTGAATCGGAATACTCTGTACAGTCCGCG------------------
------------------AGAAGAACACCATTGAAATCCAAC---CTCATCAAA------
---------CCTGAGCCCATCTACAAAACAGGGATCAGCACAAACGGCTCGACCGCA---
---------------------------------AACCACAACAACCAAAACCATCTGCAC
CAC---
---CAAACCCAGCACCTGCAACAGCACCAACAGCAACCGGTGGCGTACCAAGTTGACCCG
---AACCTG---CAGCAGCCTCATCAGACCTACCAAAACACGACGACCCATAGTTCCCAG
AGC---TCCAACAACGGG------ACTGAGCGG---------------------------
---TGCTCGAGCGTCCTCAGCGGCATCGGTGTTGCCAAC---------CCTCAC------

---------GTAGCAGGAAAGTACAACTCAGCGGCAGAA---------------------
---GAGGACGACGCGAGCCAGTATGGCAACACCGCAAACTACCCTCGT---AATTTC---
--GGTCGTAATAGA

------------CCTGAGCTGCCCACCCCC------------------------------
--------------------------------------GGGGGC---------------T
CC---GTGCACTCCGAC------ATGGCT-------------------------------

-----------GTGGTT---
-----CCTAACTCGATGCTGTCCTACTCGCACTACCCTGGA-------------------
--------------------------------ACGCATGGCGACAAGCTCTAC-
>GEPI01043883_Baikalogammarus_pullus_Bib
ATGGGC--
--TCTTATAACACAGAG---
TTGGAGAACCAGTTGTGGGCATTGTTATCTAAACTAGAAAATGGTGCCCCTGGTTGTGAC

ATGGAC--
--GGCAGGAGCGTCAGACCC
AACCTGCGGTGGGGCTTCGCCATGCTCGCCGAACTGAGAACCCTA---GACCTGTGGCGC
GCTGTGATCGGCGAGTGTTTGGCTGTGGTGTTCTACGTGCTGCTGGTGTGTGGTGCCTAC
AGTCCGTGGGCCGGGCAGCCAACTCCT---------------------------------
---CCAGAGAGG
CATCTAGTCATCTCGCTGGTGGCAGGCTTTGCCATGACTGTGCTTGTGCACTGTTTTTGT
CAAGTGTCCGGGGGCCACGTCAACCCCGCCGTCACCATCGCCCTCGCCGCCATGCGCCGC
GTGTCCCCCCTCAGGGCTGTCCTCTTGGTGCTGGCCCAGCTGGGGGGAGGCATCGCGGGT
GCAGCGCTCCTCTTCGGGGCGACCTCCTCCGGCTACAGTGGG------------------
------------------------------GACCTCGGCGCCACCGTCATCTCCCGCGTC
---------ATCACCGTCGGCCAGGGCTTCGGGGTGGAGTTCCTGCTCTCCTTCGTCGTG
GTGCTGGCCTACTTCTCC---GCCATGTGCCCCCATAGG---------------------
---AGGCCTACGGGG------------GGCCACCCCGCCCTTATCGTCGGACTGGCCTAC
CTCGCCACTACACTTGTGGGGCTTCCTCTGACTGGGGCCTCCATGAACCCCGCGCGCTCC
CTGGGTCCCGCCTTCGTCATGAAC---------ACCTGGCACGCGCACTGGGTATACTGG
GCGGGTCCCATCCTGGGTGGCCTTACTGCCGGGTTCATCCACGAGTTCATCTTCAACCCC
---CGCAGATCTCCTCTCTCC---------CGCAACCACTCCACA---GACGGA------
---GGTGAGGGCGGCGACTGCAGTGAT------------GGTGTCGGTGTAGAAGAAGAG
GAA------GGC---TCTCTCAGG---CAGGCT---CCAGTGACGTCATCATCGCAGTCT
GGT------------------------------------GGCAGCGCGCACGCGCAGTAC
AACCCTCTGCGC------AGA---------------------------------------
------CACGCGGCGAGAAGCCAC---------------GCCCACCCT---CGTCAG---
------------AACTCCGCCGCCAGCCCTCCA---------------CCTGGGGAT---
--CAG---
---------ATATAC---
TCTGCTGAGAGCGTGTACGGC------TACGACGATTACATCTACAGCGGCAGTAAGAGC
CTC---TAC---------------------------ACC------CCCGCCTCCAGG---

---------------------------GCCACCAACCTCCACCGCTCACAGTCCGTCTGC
ACACGC---CCGCTACCGGTGCCCAAC------------GCTGCCCCCACCGCAGCCAAC
CCTTCCGTGGGACCGGTCCAGGAC---CAGAAGCCAACCGTTACTGGTTTGCTGCAC---
---------------------AACAATCTCATG---------------------------
---------------GTGTACAATGCT------CAGCAGCAACAAAAC------------
--CAAAGACATCAA
CAGCAGTATCAGCAACAACTAATG------CAGCAACAT---ATGCCA---AATAACATC
AACAAG---CCTTCG---TCAGTGTCGGGATCCGCTTCACATTTCTCAAAGAACATAAGA
AATGCGGCGCCCTCTGAATCTGAATACTCTGTACAATCCGCG------------------
------------------AGAAGAACACCATTGAAATCCAAC---CTTATCAAA------
---------CCTGAGCCCATCTATAAAACAGGGATCAGCACAACCAGCTCAACCGCC---
---------------------------------AACCATAACAACCAAAACCACCTGCAC
CAC---
---CAAACCCAGCACCTGCAGCAGCACCAACAGCAACCGGTGGCTTACCAAGTGGACCCG
---AACCTG---CAGCAGCCTCATCAGCCCTACCAGAACACCACCACCCACAGTTCACAG
AGC---TCCAACAACGGG------ACGGAGCGG---------------------------
---TGCTCGAGCGTCCTCAGCAGCATGGGCGTGACCAAC---------CCTCAC------

---------GCGGTGGGGAAATACAACTCTGCTGCAGAGGAG------------------

GAGGACGACGAGGCAAGCCAGTATGGAAACACCGCCAACTACCCGCGT---AATTTC---
--GGTCGTAATAGA

------------CCTGAGCTGCCCACTCCA------------------------------
--------------------------------------GCGGGC---------------T
CC---GTCCACTCCGAC------ATGGCT-------------------------------

-----------ATGGTG---
-----CCGAACCCCATGTTGTCCTATGCACACTACCCCGGG-------------------
--------------------------------ACGCAGGGCGACAAGCTCTAC-
>GGJM01171084_Lepas_anatifera_Bib
ATGGCGATC---
---------------------------------------ACTTCGCTTTCTGCTGAAACC
CTGGACAGTCACATATTGACCCTAATCGACAAATTGGACCACGTGCAGGGCGAT------

--CTGAGT
GTCCCGCCAGTGCGGCTGCCCATGCACGTGGAAGTGCGGCGCCTC---GAATTTTGGCGG
GCTATCATTGCGGAATGCATGGCCACATTTTTCTTCGTATTTTTGATCTGTGCTGCAAAC
GTTCCGTGGAGCACTCACACAGCTTGG---------------------------------
--ACGTCCCAGAGT
TTGTACGCCGGAGCATTCACGGCCGGATTCGGGGCCGCGGCACTCACACAGTGTTTCTGG
CGTGTGTCCGGCGCCCACATGAACCCTGCCGTTTCACTAGCCCTGGCAGCGACGCGGAAG
ATATCGGCGCTGCGCTGCCTGCTGTACATGACGGCTCAATGTGGCGGGTCTATTGCCGGC
GCTGCTCTACTATACGGGTCGTCCACCACCAGCCTGCAGGGT------------------
------------------------------TCGCTGGGGGTGACGGTGGTGACGCCGCCG
---------CTGACCGCCTGGCAGGGCTTCGGCGTCGAGTTCGTGCTCACCTTCGTGCTC
ATCTTCGTCATATTCTCG---GTGACGGAGCCTTCGCGG---------------------
---CGGCCGCTG---------------GCCAGCAGCTCACTCATCATTGGCTTCACCTAC
CTCACAGTCTCCTTGGCCGGGATCCGGTGCACGGGCGCCTCAGTGAATCCTGCACGCTCT
CTGGGACCCGCCTTCGTCATGAAC---------ATCTGGAAGGATCACTGGGTGTACTGG
TTCTCTCCGCTGACCGCCGCCGTTATCGCCGCCTACGTACACGAGTACATCTTCAACCCC
---AGCCGGCGGTACCGC------------ATGAAAAATTCAATG---GACACA------
------------------------GAG------------TCGATCGCCGCCCACAGTGAC
GAC------GAG---GCATTCGAC---GAC------GAGCGTCCCAAGCTCTCGTCCCCG
GAC---GACTAC
AACACGCTG---------CGC---------------------------------------
------GCGCACAACTACGCCACC---------------TACGGGCCC------------
------------GAGGGCAAACCGAGCCCGGCC------------------GGCGGT---
---------------------------------------GCGCGCTCCGTGTTTTCTGTA
------CCCGCCTAC------CGG------GTACAC------------------------
CGCACCGAGTCTGTGTACGGC---------------------------GGTACCAAGTCG
CTG---TACCACTAC---------------------TCA------CCGCGTCCGAGC---

---------------------------CGCGCCAACCTCGCCAGGTCGCAGTCGGTGTAC
GCCAAG---GAGTCGCGCGGCGACGTC------------TCG---------TGCGGCGGC

GGCGGGATGACGGCCGCGCAGAGC---GTCTACCCTCGCATCTCCGGCGGC---------
---------------------GCCGGTCTC------------------------------

--GGCCACGCCGAC
TCCATGTACTCGCAGCGG---------------------------CCG------GTCTGC
TCTCGG---CCCGAG---
------------------CCGCTGTACCCGCTGCGCCGC---------------------
------------------------GAGCCGGCC---GAC------------CAG------
---------CGCAGGCCGCTCTAC------GCCGCCAAGGAAGACCCC------------
------------------------TACTTCAGCAAGGACGAGGCCGGCGGTGGACACGGC

TCCATTGCGGCAGCACGTACCCAGGAC---TACGTCACAGCCGTGACCTACACAGCC---
------------------------ACCTCCCGCCCG------GACGACTACGCCTACGGC
AAAAAACAGTCGGACTACGCCAAACAG---------------------CCGGAA------
------CTGTACTCGAAGCCGAGCTACCCT---TCCTACGGGGCGGCGGCGGCGGAG---
---CGGGAGAACGCCCACAACCAGCGGAGCGCTGCGGGCCGCCTGGTCGGC---------
ACCGGCCCCACCAGCCCGCCCTACGGCAGT---GGG---TAC------------------
--GGCCGGGCGGGG
CCG---
---------CCGCCGCCGCCGCCCCCGCCG------------------------------
--------------------------------------CCGCGC---------------A
AC---GGCACCATGATGTCG---CCGAGGTCGACGCTG----------------------

--------------TCGGAC--
--CCTCCCTCCGCCCTCACCACG-------------------------------------
-----CCCAACTCGATCGCGTCCTACCGC-------------------------------

>GHFC01035557_Neolepas_marisindica_Bib
ATGCCGATC---
---------------------------------------ACGTCTCTTTCGGCCGAGACC
CTGGACAGTCACATCCTGACCCTGATTGATAAACTGGACCACGTCCAAGGCGAA------

--CTGAGC
CCGCCGCCGGCCCGTCTGCCCATGCACGTGGAGGTGCGCCGGCTG---GAGTTCTGGCGC
GCCATCATCGGCGAGTGCGTCGCCACGTTCTTCTTCGTGTTCCTGATCTGCGCCGCCAAC
GTGCCGTGGAGCACTCACACGGCCTGG---------------------------------
--ACGTCGCAGAGC
CTGTACGCGGGAGCGTTCACGGCCGGCTCCGCGGCCGCCGCGCTGACCCAGTGCTTCTGG
CGCGTGTCGGGCGCCCACATGAACCCGGCCGTGTCGCTGGCGCTCGCCTCCACCAGGAAG
ATATCGCCGCTGCGCTGCCTCCTCTACATGACCGCCCAGTGCGGCGGGTCTATCGCCGGC
GCCGCCCTACTCTACGGGTCATCGACGTCCAGCCTGCAGGGG------------------
------------------------------TCTCTGGGCGTGACGGTGGTGACGCCGCCG
---------CTGACCGCCTGGCAGGGCTTCGGCGTCGAGTTCGTCCTGACGTTCGTGCTC
ATCTTCGTGTTCTTCTCG---GTGACTGAGCCGTCACGT---------------------
---CGACCGCTG---------------GCCAGCAGCTCGCTCATCATCGGCTTCACCTAC

CTCACCGTCTCGCTGGCTGGGATCCGTTGCACGGGAGCCTCCGTGAACCCAGCGCGTTCG
CTGGGACCCTCCTTCGTCATGAAC---------ATCTGGAAGGATCACTGGGTGTACTGG
TTCTCCCCGCTGACTGCCGCCGTATTCTCTGCGTACGTGCACGAGTACATCTTCAACCCC
---AGCCGCCGCTACAGG------------GTCAAGGACACGATG---GACGCC------
------------------------GAG------------TCGATCGCCGGTCAGAGCGAC
GAC------GAG---GCGTACGAC---GAC------GACCGGCCCAAGCTGTCGGCGCCC
GAC---AACTAC
AACACGCTG---------CGT---------------------------------------
------GCCCAGAACTACGTCACG---------------TACGGGCCG------------
------------GACGCCAAGCTGAGTCCGGTC------------------GGGCCG---
---------------------------------------GCCCGCTCCGTGTACTCGGTG
------CCCGCGTAC------CGG------GTACAC------------------------
CGCGCCGAGTCTGTGTACGGC---------------------------GGCACCAAGTCG
CTG---TACGCCTGC---------------------TCG------CCGCGGCCCATC---

---------------------------CGCGCCAACCTCGCCAGGTCTCAGTCGGTGTAC
ACCAAG---GAGACGCGCGTGGGGCAC------------GAG------------TCGCAC
GGTGGCATGGTAGCAGCTCAGAGC---GTGTACCCGCGCATC---ACCGGC---------
---------------------TCCGGACTG------------------------------

--GACCGCGTGGAG
ACCATGTACGCGCCGCGC---------------------------GCC------------
GCCCGG---CCGGAG---
------------------CCCATCTACCCGACCCGCAGC---------------------
------------------------GAGGCGACC---GAG------------CTG------
---------TGCAAGGTCGTCTACCACCTCGCCGGCAAGGAGGACAAC------------
------------------------------TGCCAGTAC------ATGGGCGGCCGC---

GTGGCGGTCGGGCCGCGCTCCGACGAA---TACGTCACGGCCGTGACGTACACGGCC---
------------------------ATGTCGCGGCCC------GACGACTACGCC------
---------------TACGGCAAG------------------------CCGGAG------
------CTGTACGCCAAGCCCAGCTACCCG---TCGTACCAGTCGGCGACCGCGGTCGCC
TGCACGGAGAACGCGCACAACCAGCGCAGCGCGGCCGGCCGCGGGTGC------------
---GTCGGCGGCGCCCGGCTCTACGGC------GGC---TAC---------AAC------
--GGCTCCGGCCCG
CCG---
------------CCGCCACCACCGCCGCCG------------------------------
--------------------------------------CCCAGA---------------A
AC---GGCAACATGGTGGTG---CCGCGCTCAGGCGCC----------------------

--------------TCCGAG--
--ACG---

>GGCF01055553_Pollicipes_pollicipes_Bib

ATGGCCATC---
---------------------------------------ACGTCGCTGTCCGCGGAGACG
CTCGACAGCCACATCCTGTCGCTGATCGACAAGCTGGACCACGTGCAAGGGGAC------

--CTGAGC
CCGCCGCCGGCCCGATTGCCCATGCACCTGGAGGTGCGGCGGCTC---GAGTTCTGGCGC
GCCGTCATCGCCGAATGCATGGCCACGTTCTTCTTCGTGTTCCTCATCTGCTCCGCCAAC
GTGCCATGGAGCACGCACACCGCCTGG---------------------------------
--ACGTCGCAGAGC
CTGTACGCGGGCGCATTCACGGCCGGGTTCGGGGCGGCGGCGCTGACCCAGTGCTTCTGG
CGGGTATCCGGCGCGCACATGAACCCGGCCGTCTCGCTGGCGCTGGCCGCCACCCGCAAG
ATCTCACCGCTGCGCTGCCTGCTCTATGTGACAGCCCAGTGCGGTGGCTCCATCGCAGGC
GCGGCGCTCGTGTACGGCTCGTCCACCTCGAGTCTGCAGGGC------------------
------------------------------TCGCTGGGCGTGACGGTGGTGACGCCGCCG
---------CTGACCGCCTGGCAGGGCTTCGGGGTGGAGTTCGTCCTGACGTTCGTGCTG
GTGTTCGTCGTGTTCTCG---GTGACGGAGCCGTCGCGC---------------------
---CGCCCACTG---------------GCCAACAGCTCGCTCATCATCGGCTTCACCTAC
CTCGCCGTCTCGCTGGCCGGGATCCGCTGCACTGGCGCCTCGGTCAACCCGGCCCGTTCG
CTCGGCCCCGCTTTCGTCATGAAC---------ATCTGGAAGGACCACTGGGTGTATTGG
TTCTCGCCTCTGACGGCGGCCGTGGTGGCCGCCTACGTGCACGAGTACATCTTCAATCCA
---AACCGGCGCTATAGA-------------ATGAAGGACTCTATG---GATACC------
------------------------GAG------------TCGATCGGAGGCCAGAGCGAC
GAT------GAG---GCCTACGAT---GAC------GAGCGACCGAAGCTCTCCTCG---
---TCCGACTAC
AACACGCTG---------CGC---------------------------------------
------GCCAAAACCTACGCCTCG---------------TACGGCCCG------------
------------AAGGCGGACCTGAGCCCGGCC------------------GGCGGC---
---------------------------------------GCGCGCTCGGTGTTCTCGGTG
------CCGGCGTAC------CGT------GTGCAC------------------------
CGCGCCGAGTCCGTGTACGGC---------------------------GGCACCAAGTCG
CTG---TACGCCTGC---------------------TCG------CCGAGGCCGAGC---

---------------------------CGGGCCAACCTGGCCAGGTCCCAGTCTGTCTAC
ACGAAG---GAGCCGCGCGGCGCGCAG------------GAG------------GGCCGC
GGCGGCATCTCGGCCGCGCAGAGC---CTGTACCCGCGCATC---GACGGC---------
---------------------GCCGGCCTG------------------------------

--GCGCAGACGGAG
AGCCTGTACGGGCCGCGC---------------------------GCC------GGCTGC
GGTCGC---CCAGAG---
------------------CCGGCCTACCCCACGCGCAGC---------------------
------------------------GAGGCGGCC---GAG------------GCG------
---------CGCAAGGCGCTGTAC------GCCGCCAAGGAGGAGGCG------------
------------------------------TGCGCATACGGCGCGGCCGGCGCGCAC---

TACTCGACCGGCGCGCGCCCCGACGAC---TACGTCTCGGCCGTGACATACACGGCT---
------------------------GCGGCGCGCGCC------GACGACTACGCC------

------------GTGTACGGCAAG------------------------CCGGAG------
------CTGTACGCCAAACCGCAGTACCCG---TCGTACCAGTCGGCGGCCGTGGCG
TGCTCTGAGAACGCGCACAACCAGCGCAGCGTGGCGTCGCGGCCGTAC------------
------GGCGGCGGGCCGCTGTACGGG------GCG---TAC---------TCG------
--GGCCGCGCTGGC
CCA---
------------CCGCCGCCGCCGCCCCCG------------------------------
-----------------------------------CCCCGC---------------A
AC---GACAACATGATGTCG---CCGCGCTCGGTGACC----------------------

-----------GGCTCTGAG--
--ACGGCATCGGGCCTCACCACG-------------------------------------
-----CCCAACTCGGTCAGCTCGTACCGC-------------------------------

>XP_043201541_Amphibalanus_amphitrite_Bib
ATGGCCATC---
---------------------------------------ACGTCGCTGTCAGCGGAGACG
CTGGACAGCCACATCCTGACGCTGATAGACAAGCTGGACCACGTGCAGGGCGAG------

--CTGAGC
CCGCCGCCGGCGCGGCTGCCCATGCACGTGGAGGTGCGCCGGCTC---GAGTTCTGGCGC
GCCATCATCGCCGAGTGCATGGCCACGTTCTTCTTCGTGTTCCTGATCTGCGCGGCCAAC
GTGCCGTGGAGCACGCACACGGTCTGG---------------------------------
--GCGGCGCAGAGT
CTGTTCGCCGGCGCGTTCACGGCCGGTCTGGCGGCCGCAGCGCTTACCCAGTGTTTCTGG
CGCGTCTCCGGCGCGCACATGAACCCGGCCGTGACACTGGCGCACGCCGCCACGCGCAAA
GTGTCGCCGCTCAGATGTCTGCTCTACATCACTGCCCAGTGCGGCGGCGCCATCGCCGGA
GCTGCGCTCCTCTACGGGTCGTCGACGACCAGCCTGCAGGGC------------------
------------------------------TCGCTGGGCGTGACGGTGGTGACGCCGCCG
---------CTCACCGCCTGGCAGGGCTTCGGCGTCGAGTTCGTCCTCACCTTCATCCTG
GTGTTCGTCGTGTTCTCG---GTCTCGGAGCCGTCGCGC---------------------
---CGGCCGCTG---------------GGCAACAGCGCGGTCGTCATCGGCTTCACCTAC
CTGGCCGTCAGCCTCGCCGGGATCCGCTGCACAGGAGCCTCCGTGAACCCGGCCCGCTCC
CTGGGACCGGCCTTCGTCATGAAC---------ATCTGGAAGGACCACTGGGTGTACTGG
TTCTCGCCCCTTACCGCCGCTGTGGTGGCCTCCTACGTGCACGAGTACATCTTCAACCCC
---AACCGACGCTACCGG------------CTCAAGGACACCATG---GATACA------
------------------------GAA------------TCTCTGGGCGGTCAGAGCGAT
GAC------GAA---GTGTTCGAG---GAG------GACCGGCCCAAGCTCTCCTCG---
---GCCGACTAC
AACACGCTG---------CGC---------------------------------------
------TCGCAGTCGTACGCCATC---------------TACGGCGCC------------
------------GGCTCCAAGCCGAGCCCGGCC------------------GGCGGC---
---------------------------------------GCGCGCTCCGTGTTCTCTGTA
------CCGGCGTAC------CGC---GGCGTACAC------------------------
CGGGCCGAGTCGGTGTACGGC---------------------------GGTACCAAGTCG
CTG---TACGCGTGC---------------------TCG------CCGCCGCCCAGC---

---------------------------CGCGCGCACCTCGCCAGGTCCCAGTCGGTGTAC
ACCAAG---GACCGCCGGTGTGCCCAG------------GAG---------GGGGGCCGC
GCCGGCATCACGGCCGCCCAGAGC---GTGTACCCGCGGATCGGCGGCGGC---------
---------------------GGCGGGCTG------------------------------

--GCGCAGACAGAG
TCCATGTACGCCACCCGC---------------------------AGC---------TGC
GCGCGC---CAGGAG---
------------------CCCACCTACGCCGACCGCCGC---------------------
------------------------GCCGCCGCCGCCGAGCAG---GCCGAGGCG------
---------CACAAGCCGCTGTAT------TCTACCAAGGAGGACGCG------------
------------------------------CTGTCCTACAGCACTGCCACCACCCAG---

TTCACGGCCAGCTCCCGGGCCGACGAC---TACGGCCAT---------TACCCAGCG---
------------------------GTGTCACGGCAC------GACGACTACGCC------
------------GTCTACGGCAAACAG---------------------CCAGAG------
------CTCTACTCCAAGCCGCAGTACCCG---AGCTACCATTCGGCGGCTGCC------
------GAGAACGCGCACAACCAGCGCAGTGCCGCCGGCCGGTCGCACGGCTCCGCGCCC
GGCGCCGGCGGCCTGCCCGCCTACGGC------GCC---TAC---------TCG------
--GGCCGGTCCGGA
CCG---
------------CCGCCGCCGCCGCCCCA-------------------------------
--------------------------------------CCGAGG---------------A
AC---GGAGACATGATGTCT---CCGCGGTCCGTGGCC----------------------

-----------GGATCGGAG--
--ACGACCTCCGGACTCACCACG-------------------------------------
-----CCCAACTCGGTGAGCTCCTACCGC-------------------------------

>KY508290_Amphibalanus_improvisus_Bib
ATGGCCATC---
---------------------------------------ACGTCCCTCTCCGCGGAGACT
CTGGACAGCCACATACTGGCGCTGCTGGATAAACTGGACCACGTGCAGGGCGAG------

--CTCAGT
CCGCCGCCCGCTCGCCTGCCGATGCACGTGGAGGTGCGCCGGCTC---GAGTTCTGGCGG
GCCATCATCGCGGAGTGCATGGCCACGTTTTTCTTCGTGTTCCTCATATGCGCGGCTAAC
GTGCCGTGGAGCACACAC------TGG---------------------------------
--GCGTCGCAGAGT
CTGATCGCGGGCGCGTTCACGGCGGGTTTCGCAGCGGCCGCGCTCACCCAGTGCTTCTGG
CGTGTGTCCGGTGCTCACATGAACCCGGCGGTCACGCTGGCGCACGCGTCCACACGTAAG
ATCTCGCCGCTCCGATGTCTGCTCTACGTGACGGCCCAGTGCGGAGGAGCCATCGCAGGA
GCCGCTCTACTATACGGGTCATCCACCACCAGCCTGCAGGGC------------------
------------------------------TCGCTGGGCGTGACGGTGGTGACGCCGCCG

---------CTGACCGCCTGGCAGGGCTTCGGCGTCGAGTTCGTGCTCACATTCATCCTC
GTATTCGTCGTGTTCAGC---GTCTCCGAGCCGTCCCGC---------------------
---CGGCCGCTG---------------GGCAACAGCTCGGTCGTCATCGGCTTCACCTAC
CTGGCCGTCAGCCTCGCCGGGATCCGCTGCACGGGAGCCTCGATGAACCCGGCCCGCTCG
CTCGGACCTGCCTTCGTCATGAAC---------ATCTGGAAGGACCACTGGGTGTACTGG
TTCTCCCCTCTGACGGCTGCCGTGATCGCCGCCTATGTGCACGAGTACATTTTTAACCCG
---AACCGGCGCTACCGT------------CTCAAGGACACCATG---GACAAC------
------------------------GAA------------TCACTCGGGGGACAGAGTGAC
GAT------GAA---GTATTCGAC---GAG------GAGCGTCCGAAGCTCTCTCCG---
---GCCGACTAC
AATACGCTG---------CGC---------------------------------------
------TCTCAGACGTACGCCGCC---------------TACGCGCCG------------
------------GCGTCCAAGGCGAGCCCGGCC------------------GGCGGC---
---------------------------------------GCGCGATCAGTGTTCTCTGTA
------CCGGCGTAC------CGGGGAGGTGTCAGT------------------------
CGTGCGGAGTCGGTGTACGGC---------------------------GGTACCAAGTCA
CTG---TATGCCTGC---------------------TCG------CCGCCGCCCAGC---

---------------------------CGCGCCAACCTCGCCAGGTCGCAGTCTGTATAC
ACCAAG---GAGCGCCGGTGTGGGATG------------GAG------------CCTCGT
GCGGGCATCACGGCTGCCCAGAGC---GTGTACCCTCGGATC---GGAGGC---------
---------------------GGCGGCGGC------------------------------

--GGCCTGGCTGAG
TCCATGTACGCCACACGT---------------------------GCC------------
CCGCGA---CAGGAG---
------------------ACGGTCTATCGCCCCGCCGAG---------------------
------------------------CCGGTC------GACTCG---------CAG------
---------AAACAGCAGCTGTAC------TCGGCTAAAGAGGACGCG------------
------------------------------CTCTCCTACAGCACGGCAACGACTCAC---

TTCACCGCCAGCTCTCGGCCTGACGAC---TACGCTGCG---------TAC---------
------------------------TCCCCGCGACAT------GACGACTATGGAGTC---
---CAGCAGGACGTCTATGGAAAGCAG---------------------CCGGAG------
------CTCTATGCCAAGCCGCAGTACCCGGGGAGCTACCAGTCAGCAGCAGCG------
------GAGAACGCTCACAACCAGCGCAACGCCTCTGGCCGGCCACACACCAGT------
GGGGGAGGTGGTCTGCCGGCATATGGA------GCC---TAC---------TCT------
--GGCCGAGCGGGA

------------CCGCCGCCGCCGCCCCCG------------------------------
--------------------------------------CCGCGG---------------A
AT---GGCGATATGATGTCT---CCACGCTCCATGGCG----------------------

-----------GGGTCTGAG--
--ACGACCTCCGGCCTCACCACG-------------------------------------

```
-----CCAAATTCTGTCAGTTCGTACCGC-------------------------------
------------------------------------------------------
>VOPJ01015035_GHBL01006535_Semibalanus_balanoides_Bib
ATGGCCATC---------------------------------------------------
---------------------------------------ACCTCGCTCTCCGCCGAGACG
CTCGACAGCCACATCCTGACGCTGATCGACAAGCTGGACCACGTGCAGGGCGAT------
------------------------------------------------------------
------------------------------------------------------CTGACC
CCGCCGCCCGTCCGCCTGCCGATGCATGTGGAGGTGCGCCGGCTC---GAGTTTTGGCGC
GCCATCATCGCCGAGTGCATGGCCACGTTCCTCTTCGTGTTCCTCATCTGCGCGGCCAAC
GTGCCCTGGAGCACCCACACGGTGTGG---------------------------------
------------------------------------------------GCGTCGCAGAGC
CTGTTCGCCGGTGCGTTCACGGCTGGACTGACGGCGGCGGCGCTGACGCAGTGTTTCTGG
CGCGTCTCGGGCGCGCACATGAACCCGGCCATCACGCTGGCGCACGCCGCCACCAGGAAG
GTGTCCCCGCTCAGATGTCTTCTCTACATCACGGCCCAGTGCGGCGGCGCCATCGCTGGG
GCCGCTCTCCTGTACGGGTCCTCCACGTCCAGCCTGCAGGGC------------------
------------------------------TCGCTGGGCGTGACGGTGGTGACGCCGCCG
---------CTGACCGCCTGGCAGGGCTTCGGCGTCGAGTTCGTGCTGACGTTCGTCCTC
GTCTTCGTCGTGTTCTCC---GTGTCGGAACCGTCGCGC---------------------
---CGGCCGCTC---------------GGCAACAGCTCGGTCATCATCGGCTTCACCTAC
CTGGCCATCAGCCTCGCCGGGATTCGCTGCACGGGAGCCTCGATGAACCCAGCTCGTTCT
CTGGGACCTGCGTTCGTGATGAAC---------ATCTGGAAAGACCACTGGGTGTACTGG
TTCTCCCCTCTGACTGCAGCTGTGGTCGCCTCCTATGTACACGAGTACATCTTCAACCCG
---AACAGACGGTACCGG-------------CTCAGGGACTCGGTG---GATACA------
------------------------GAG------------TCTCTCGGCGGTCAGAGTGAT
GAC------GAG---GCTTACGAA---GAG------GACCGTCCGAAGCTCTCCGCA---
---------------------------------------------------GCCGACTAC
AACACACTG---------CGC---------------------------------------
------TCGCAGAACTACGCCACG---------------TACGGACCA------------
------------GAGTCGAAGCTGAGTCCGGCC------------------GGCGGC---
---------------------------------------GCGCGCTCCGTCTTCTCAGTA
------CCGGCGTAC------CGC---GGCATGCAC------------------------
CGCGCCGAGTCCGTGTACGGC---------------------------GGTACCAAGTCG
CTG---TACGCCTGC---------------------TCG------CCGCCGCCCGGC---
------------------------------------------------------------
---------------------------CGCGCCAACCTGGCCAGGTCACAGTCGGTGTAC
ACGAAG---GAGCGCCGTGCCGCCCAG------------GAG------------AGCCGC
GGCGGCATCACAGCCGCCCAGAGC---GTATACCCTCGCATC---GGCGGC---------
---------------------GTCGGTCTG------------------------------
------------------------------------------------------------
------------------------------------------------GCGCAGACCGAG
TCCATGTACGCGACGCGG---------------------------AGC---------TGC
GCGCGA---CCGGAG---------------------------------------------
------------------CCTTCCTACGCGGACCACCGC---------------------
---------------------GCCACCGCGGCCGCTGAGCCC---------CCT------
---------CATAAGGCGATGTAT------GCCGCCAAGGAGGAGGCC------------
------------------------------CTCGCCTACAGCGGGGCCGGAGCCCAC---
------------------------------------------------------------
```

TTCACGGCCAGCCCGCGCGCGGAGGAT---TACGCCGCGGCCGTCACTTACACCGCC---
------------------------GCGTCCCGGCAC------GACGACTACGGC------
------------GTCTACGCGAAGCAG---------------------CCGGAG------
------CTGTACGCGAAGCCGCAGTACCCG---TCCTACCAGTCGGCGGCGGCC------
------GAGAACGCGCACAACCAGCGCAGCGCGGCCGGACGGCCGCAC------------
GGCGGCGGCGGCGGCCCGGTGTATGGA------GCC---TAC---------TCG------
--GGCCGGGCGGGT

---------------CCGCCGCCGCCGCCG------------------------------
--------------------------------------CCGCGG---------------A
AC---GGAGACATGATGTCT---CCGCGGTCGGCGGCC----------------------

-----------GGCTCGGAG--
--ACGGCGTCAGGCCTGACGACG-------------------------------------
-----CCCAACTCTGTCGGCTCGTACCGC-------------------------------

>JALBCS010002156_Sacculina_carcini_Bib
ATGACTGTG---
------------------------------TTA------TCTCCAATTACAATCGAGACT
TTCGACTCTCACGTCGTTTCTCTTCTAGATAAGCTGGACCATGTTCGTGGGGAG------

--CTGCGA
CATCACCCCACGCGGGCCCCCATGGATCTCGAAGTTCGTCGTCTG---GAATTTTGGCGC
GCAGTTCTGTGCGAATGCGTAGGAACGTTGCTTTTCGTCTTTCTTGTGTGTTCGGCCAGT
CTTCCATGGAGTCCTCTGGATACGTGG---------------------------------
--GCTCCACAGCGT
TTATACGCAGCCGCCACTACGGCCGGCGTATCTATGGCAGTGCTTACCCAGTGTTTGTGG
AGGTGTTCGGGAGCTCATTTCAACCCGGCGATAACAGTGGCCGCGGCGGTCGTGGGCCAT
ATCTCACCGTTGCGCTGTCTCATATATGTCGCGGCCCAGTGCGGCGGTTCTGTGGCTGGA
GCAACTCTACTGTACGGGTCTTCAACTCCGAGTGTCCAGGGA------------------
------------------------------TCGTTGGGCGTCACTCTGGTCACTCCTCCG
---------TTGTCGGCTTGGCAAGGATTCGGCAACGAGTTCGTGATGAGCTTCATCGTG
GCTCTGGTGTTCTTCAAC---GTCACGGATCCGTGTCGT---------------------
---CGGCCGCTG---------------GGCATGAGCTCTCTCATCATTGGACTCAGCTAC
ATGGCGGTGTCTTTGGCTGGTGTTCACTCAACAGGCGCGTCTATGAACCCAGCCAGATCT
CTGGGTCCTGCTTTTGTTATGGAC---------ATTTGGAATGATCATTGGGTG------


```
------------------------------------------------------------
------------------------------------------------------------
------------------------------------------------------------
------------------------------------------------------------
------------------------------------------------------------
------------------------------------------------------------
------------------------------------------------------------
------------------------------------------------------------
------------------------------------------------------------
------------------------------------------------------------
------------------------------------------------------------
------------------------------------------------------------
------------------------------------------------------------
------------------------------------------------------------
------------------------------------------------------------
------------------------------------------------------------
------------------------------------------------------------
------------------------------------------------------------
------------------------------------------------------------
------------------------------------------------------------
------------------------------------------------------------
------------------------------------------------------------
------------------------------------------------------------
------------------------------------------------------------
------------------------------------------------------------
------------------------------------------------------------
------------------------------------------------------------
------------------------------------------------------------
------------------------------------------------------------
------------------------------------------------------------
------------------------------------------------------------
------------------------------------------------------------
------------------------------------------------------------
------------------------------------------------------------
------------------------------------------------------------
------------------------------------------------------
>JW965253_Argulus_siamensis_Bib
ATGGCACCC---------------------------------------------------
---------------------------AAATTGAACTCTAGTCTTTCCAATCCCCGTAGT
CTGGAATCGCATATTGTAACATTATTCGAAAAATTGGATTCCTTG---------------
------------------------------------------------------------
------------------------------------------------------CCTCAC
ACATTGCCAGTCAAGCTTACGTTCAGGGAAGAAATCAGAACCCTT---GAGTTTTGGCGT
GCAATCATTGGTGAATGTTTAGCAACTTTTTTCCTTGTATTCCTTGTATGTGGCTCCCAT
ATTCCATGGACTGCTATGAATGGTGGA---------------------------------
------------------------------------------------------------
CCTGTCACCATATCATTAGCTACTGGAGTGTCTGTGGCAACAATTATACAGAGTTTTAGC
CACATTTCGGGTGCTCACATTAATCCCTCTGTCACAGCCGCATTTATGGCCACTAAAAAA
```

ATTTCTCTTGTTCGAGGAGGACTTTATATCACTGCCCAATGTGGTGGAGCAATAGCTGGA
GCTGCCTTGCTTTATGGGGTAACAGCCACCGGTCATCAAGGA------------------
------------------------------ACATTAGGGGTGACAATGATTGCCAGGGAG
---------ATGTCTTCGTGGCAGGCGTTTGGAATGGAATACGTCTTGACTTTCATTCTG
ATGTTTGCTATCTGTCAG---ATTACTGATCCTCACAGG---------------------
---AAAGCCATA---------------TGTACTCCATCTATTTGTATCGGAATGGCCTAC
ATGGCATGTACTTTTGTATCGGTTTCTGGGTCGGGGGGATCGTTAAACCCAGCGAGGTCG
TTGGGTCCAGCCTTTGTCATGTAC---------AAATGGGATTCCCACTGGGTGTTCTGG
CTTGGTCCTATCTTTGGAGCTTTAAGTGCAGCACTTATTTATGAATTCATCTTCAACAGC
---AACAGACAAAGGAAATTC---------ACCAAAGGTTCGATT---GATGGA------
------------------------GAT------------TCTTCAAGTCTAAAAAGCGAT
---------GAC---CCTTACGAT---GACATT---GATAAAACTACCACAGCCACTAAA
--TTCTCTACCTTT
AATACTCTG---------AGG---------------------------------------
------TCTCAACACTATGATCAA---------------TACCGACCC---CCAGCTTCG
GCAGTT------GCTAACAGAATCAATCAACAA------------------ACTCAG---
---------------------------CACAATTTTTTCATCAGTGCCCATTACAATTCT
------CCTAATGACGAT------------TCAAAA------------------------
AAGAGCGAACCTATTTACGAG---------------------------GGTTCTAAATCT
TTA---TAT---TGT------------------CGATCT------CCTGCATCCCCCACA
TTA---
---------------CTG---------AGAGCCAACCTTAATAGATCGGAAAGTGTCTAC
TCCAGG---GTACCCCTCAGCAATGATAATACATAT---GGCGTTAGGTCTTTAAGAGGA
CCACACGTCAACAGGTCTATGACT---ATATCCAATGGAAAACCCCAC------------

--AGCCGGCAAGAA
AGTACTTATTCAACACGCCCAGTTCCTCCC------------ATACCA------TCAATG
CCCAAA---AAAGAT---------AGC---------------------------------
---------------------ATTTACGGT------------AGACTCAACAGA------

---------GGCGATGGGATATAT------GGAGGGCTAGCTTCAACCGGAGACTCC---
------------------------------ATGCGACTCAATAGTCCAGCG---------

------------TCACGTATGGATGCAGTCTTTGGAGGCAATCCCAATACAATACATGAA
CCA---ATATAT------------CAGACAAGG---------------------------
---AATGAGGCCTTGTATAGTTTGGGGAGACCAGATTGC---------CCTAAC------
---------------------------CCA------------------------------
------------TATGAAACTTTGAGGTCTCCAGCTAGT------------------AAA
GATGAATCTCAGGACTCTTGCTATGGT------ACC---TAT---------GGC------

---------ACCAAATACCCCAAAAGC---------------------------------

--------ACCATGGCC------AATGGTACACTCACA----------------------

--------------TCAAGG--
--AAAGATACTCCACCAACT--
-----CCCCATTCAACTACCTCACCACATTTTTCCCCTGTG-------------------
---GAAAGTCATTAT-
>HBDT01003485_Argulus_foliaceus_Bib
ATGGTAGCG---
---------------------------AAACTGAGCTCCAGCCTTTCTAATAGTCGGAGT
TTGGAGTCACACATTGTGACTTTGTTCGAAAAACTGGACTCTTTA---------------

--CCTCAT
ACGTCTCAGGCTAAACTGTCTTTCCGGGAAGAAATCAGGACTCTG---GAGTTTTGGCGT
GCCATTATCGGCGAGTGCCTAGCGACTTTCTTTTTGGTTTTCCTCGTATGTGGGGCCCAC
ATTCCGTGGACTACATTAAATGGTGGT---------------------------------

CCCGTCACAATATCCATAGCCACTGGAGTGTCTGTGGCTACCATAATTCAGAGTTTTAGC
CAGATATCGGGATCTCACATAAACCCTGCCGTGACTGTAGCGTTTATGGTAATTAAAAAG
ATTTCTCCCATACGAGGAGCTCTGTACGTCACTGCTCAGTGTGGAGGGGCGATAGCGGGG
GCTGCACTTCTTTATGGTGTGACAGCTACCGGTCATCAAGGT------------------
------------------------------CTGCTAGGAGTGACGGTTATGTCGAAGGAA
---------ATGTCCTCAGTGCAAGCCTTCGGAATGGAATATGTTTTGACGTTCATTCTA
ATGTTTGCTATTTGTCAA---ATCAACGACCCTCATCGA---------------------
---AAAGCAATA---------------TGTACCCCATCGATATGCATTGGACTGGCGTAC
ATGGCGTGCACATTCGTGTCGGTTTCTGGATCGGGAGGTTCATTAAACCCGGCGCGATCC
TTGGGGCCGGCCTTTGTGATGTAC---------AAATGGGATTCCCACTGGGTGTACTGG
ATAGGTCCTATCCTGGGATCTCTAAGTGCAGCCCTTATTTATGAATTCATCTTTAACAGC
---AATAGGCACGGAAGACTT------GGA---AAAGGTTCGATA---GACGGA------
------------------------GAT------------TCTTCCAGTCTTCGAAGCGAC
---------GAT---CCTTATGAC---GATATC---GACAAGACGACGTCCACAACCAAA
--TTCTCCACATTC
AACACTCTG---------CGG---------------------------------------
------TCACAGCATTACGACCAA---------------TACAGACCA---ACACAGCCA
ACTACG------GCCAATAGATTAAATCAGCAA------------------GCTCAG---
---------------------------CACAACTTCTTCATCAGTGCCCACTACAACTCC
------CAGAACGAAGAG------------GCTAAGAGG---------------------
GCAGCGGACCCTATATACGAA---------------------------GGATCCAAGTCT
CTA---TAT---TGC------------------CGTTCC------CCGGCCTCCCCGACT
TTA---
---------------CTT---------AGGGCCAACCTCAACAGATCGGAAAGCGTGTAC
TGCAGA---ATTCCGCTGACGAACGACAACACTTAT---GGAATCAGACCTGTGAGGTCC
TCGGCGGCAAATCGGTCGCAGACT---TTGTACAACACCAAACAGAAC------------

--AGTCGGCAGGAA
AGCGTCTACTCGACCAGACCAGTGCCACCT------------ATCCCG------TCGATG
CCCAAG---AAAGAT---------AGT---------------------------------
---------------------ATTTATGGA------------AGGTTGGCTCGA------

```
---------GCCGATGGCATTTAC------GGAGGCCTGGTCACGCCAGGAGACTCG---
------------------------------GCCCGACTTAACAGCCCAAGA---------
------------------------------------------------------------
------------------------------------------------------------
------------------AATGACTCAGTCTTCGGAATCAACTCCACTAGCCTTCAGGAA
CCG---ATCTAC------------CAGACCCGA---------------------------
---AATGAGGCGCTATACGGAATTAACCGCCAGGAATGT---------CCCAAC------
---------------------------CCC------------------------------
------------TATGAAACGCTCAGAACTACTCCGAAC------------------AAA
GACGGATCTCAAGATTCTGGCTATGGG------AAC---TAC---------GGC------
------------------------------------------------------------
------------------------------------------------------------
---------ACCAAATACCCCAAAAGT---------------------------------
------------------------------------------------------------
-----GTCAGTATGGGT------AATGGCACCCTGCTC----------------------
------------------------------------------------------------
------------------------------------------------------------
------------------------------------------------------------
--------------AGCAGA----------------------------------------
--AAAAGTAGCTCGCCGACG----------------------------------------
-----CCCAACTCAACAGTTTCTCCACATTTCTCTCCAGTT-------------------
-----------------------------------------GAAAGTCATTAT-
>ICLE01113201_Heterocypris_incongruens_Bib
ATGGATCCC---------------------------------------------------
---------------------------------AATTCGGCGCTCCTCCTCAACAAATCC
ATCGATACCCATGTCCTGTCCTTGATCGAAAAACTCGACGCCCTTCGCGGCTCG---GTG
CCTGGTGGAGTGATAAACGCCTCCAAT---------------------------------
------------------------------------------GCCAACAGCCGAAATAAA
TCGCTGGCCAAACGGCGCCTTGCTCGCCAAGAAATTCGCACTCTC---GAACTATGGCGA
AGCATCATGTGCGAGTGCTTGTCCACGCTGTTTTTCGTCTTCCTGGTTTGCGGCAGTTAC
GTCCCATGGCAAGGATGGTCGCCG------------------------------------
---------------------------------------------------------ACG
TACGTCGGTGTGTCACTTGTGACGGGATTCGCCGCCATGACCATGACCTTGTGCTTTGGA
CAGGGAACTGGAATGCACATGAACCCTGCCATCACCTTGGCCATGTTTGCAGCTGACCAG
ATAACCGCCCTGAGAGCCATTCTGTACGCCATTGCACAATCAGGAGGTGCAATTGCCGGT
GCTGCTTTGATTTATGGGATGACA---CCGGGGAGAAGC---------------------
------------------------------CATCTTGGAGTTACTGTGTTGAGCGATGGA
---------TTGTCGACTTGGGCTGGTTTTGGACTCGAGTTGGTGTTGAGCTTCATTCTG
GCGTCCTCTGTCTTGACA---GCATTT---CATCACCTG---------------------
---AAGGTGTCCAGTGCATTGCAGTCAGCTAACGCTGGTTTCATGATGTGTGCTGCTTAC
GTCACTTGCTCCTTGGTTGCTGTCCCAGCAACCAGAGGAGGCTTGAACCCCGCAAGATCC
CTTGGGCCAGCTTTTGTGATGAAC---------CGCTGGGAGGACCATTGGATTTACTGG
CTTGGACCAATGGCTGGTGGAGGACTTGCTGGCCTTCTGAGAAAATACGTCTTCGATCCT
GAAAGAAGAAACTTCAAAGGCTCT---GGCAGCAGTGGATCTTTG---CCAAAC------
------------------------GAT------------GGCTCCAGTGTCAACTCGGGC
GAAGAAGCAGACTGCGACGACGAC---GACATC---GAG---TCCAAAACCACAGCCGGC
CACCCT------------------------------------------AGTGGAGGCCAC
GGTGACGTC---------ACC---------------------------------ATCACC
```

TCCTCCTTCTCGCCCTTGGACGAG---------CTCGTCAGTCGA------CAGCAACCA
------------GCCCAGCGTCGCTCAGTCAGA---------------------GGT---
---------------------------------------GCTCAATCAGTTTACAGCGCC
------CCAGCCTACGATCGCCACCAGCACAGACGTCGCAGCCAGCACCAGGTCTCTGTC
GAAGTCGAGGAAATTTACAAT---------------------------GGTTCCAAGTCG
TTG---TACGGTGTG------------------AGAGTGGCT---GGTGCT---CCCCAA
GTG---ACTTCAGGCCCC---------AGTAGGAGCACTTGTGCGAAT------------
------GCGACAGCAATG---------ACTTCGAACATTTCCAGATCTGAGAGCGTTTGG
TGCAGG---CTG--

>CAJPEX010002027_Notodromas_monacha_Bib
ATGGATCAC---
---------------------------------AGCTCGGCGCTTCTC---AACAAGTCC
CTGGACTGTCACGTCGTGACGTTGATCGAAAAGCTGAATGCAGTGCAGAATTCT------
------------TTGAAC--
--GCGTCGCGGAAAGGCGCC
AGTGTCGCGAAGCGTCGACCGTTTCGTCAGGAGATCCGCACTCTG---GAATTGTACAAG
AGCACTATGGTCGAGTGCATTTCTACATTGTTCTTCGTGTTCCTTGTATGTGGCAGTTAT
GTGCCATGGGAACGTCGTTCGCCGACT---------------------------------

---TACGAGACG
TATGTCGGCGTTTCCCTGGGCACTGGCTTTGCTGCCATGACAATGATGCTCAGCTTTGGC
CAGGGAATCGGGGTGCATTTGAATCCCGCTGTCACGTTGGCCATGCTTATATGCGAGCAG
ATAACGGCCCTGCGTGCCTTGTTCTACCTCACGGCTCAATCCGGCGGTGCTATCGCCGGC
GCTGCTTTGCTGTATGGATTACG---TCAGGGAAGAGC----------------------
------------------------------CATTTGGGAGTGACGATTTTGAGCGAAGGA
---------CTCACAACATGGGCCGGTTTCGGACTGGAACTGGTTCTCACCTTCGTGCTT
GCTTCTTCGGTGCTCATG---GCATTT---CATGAACTG---------------------
---AAAGCCGATAACCGA---CAACAGACGAGCGCGGCTCTCACAATGTGCGCTGCTTAC
ATCACGTGCTCCTTAGTCGCAGTGCCTGCGACTGGAGGAGGACTGAATCCAGCAAGGTCT
CTTGGGCCAGCTTTTGTGCTGAAG---------CGGTGGGACGACCATTGGGTT------

---------TCTTTC------------------GCCGCCACAGCT---GAA---------
------------------------GAA------------GCGTCGAGCGTGAATTCCCGC
GAAGACGTGGACGACGACGAAGAG---GATGTG---GAG---TCCAAAACGACGGCCGGG
CATCCG--AGTGGCGGCCAC
GGGGACGTG---------ACG---------------------------------CTGACG
------GTGGAGGCCGTCGAC---------------------AGG---------------
------------GCAGCAAGGCGGTCCATGCGA------------------CTTGGG---
---------------------------------------GCCCAGTCCGTCTTCAGTGCG
------CCTGCCTATGACCGGGGCGGACGCCAGCAGGGACGC---CACCAGCGCAGCCCC
GAGACATCCTTATTTTATTCA---------------------------------------
ATT---TTTGGCGTG------------------TGTGTC---------------------

>AFFK01019694_Strigamia_maritima_Bib1
ATGGCA--
---TCGCCGACC
GCTGATGCACATTGGGTACAGTTTTTTCAGAAATTGGACAGCTTACGACACGTG------

---CAC
ATGGAAGAAAAACGCAAGACGATGAAAGAGGAGATCAGATCGGTC---GAGTTCTGGAGA
GCGGTGATTTCCGAGTGTCTAGCAACTTTCTTCTGCGTTTTTCTTATATGTGGAGCTCAT
GTGCCCTGGCCAAGATATGAACCA------------------------------------
---ACT
CCGGGAAGTGTGAGTCTCACATGCGGCTTTGCCATGGCCACGCTGACGCATTGTTTCGGC
CAGATCTCCGGCAGTCATGTAAACCCAGCAGTAACTGTCGCTATGCTCATCACCCGCAAA
GTGTCGCCACTTGGAGCGTTATTATACATAACTGCGCAGTGTGGAGGAGCTATTGCGGGT
TGTGCGCTACTTTTTGGGGTTACTATTCCTGGCCACCAGGGA------------------
------------------------------TCTCTGGGGTCTACAACGGTGCATCCTCTG
---------TTGACGCCATGGCAGGGATTTGGTGTGGAGCTTGTGCTCACCTTCATCGTC
GTCTTCACAGTTTTCGCC---AGTCTGGATTCCCATCGA---------------------
---AAGATTCCC---------------GGTGGCTCCTCACTTGCCATTGGTGCTGCCTAC
CTCACATGTACTCTTGCAGGGTTACCATCAACCGGTGCTGCAGTCAACCCTGCTCGAGCA
CTAGGCCCAGCCTTCGTTATGAAC---------CGATGGACGTATCATTGGGTATATTGG
TTTGCACCTTTATGCGGAGGGTTGTTAGCCGGACTTTTATATGAATACGTCTTTGATCCT
---CGCAAGACTATCAAGTAC---------CTAAAAGAGACTCTA---GAAGAAGTG---
------------------------GATAAA---------GGT------------------

>GCAH01015177_Clinopodes_flavidus_Bib
ATGGCA--
---TCGCCGACC
GCTGATGCTCATTGGGTGCAGTTTTTTCAGAAGTTGGACAGCTTACGACATGTG------

---CAC
ATGGAAGAAAAACGCAAGACGATGAAAGAAGAAATCAGATCGGTC---GAGTTCTGGAGG
GCGGTTATTTCCGAGTGTCTAGCAACTTTCTTCTGCGTTTTTCTTATATGTGGAGCTCAC
GTACCCTGGCCAAGGTATGAACCA------------------------------------
---ACA
CCCGGAAGTGTTAGTCTCACGTGCGGCTTTGCCATGGCGACTCTGACGCACTGTTTCGGC
CAGATCTCCGGCAGTCATGTAAACCCAGCTGTAACTGTCGCTATGCTCATCGCCCGCAAG
GTGTCGCCACTTGGAGCGTTGTTATACATAACTGCTCAGTGTGGAGGAGCTATTGCGGGT
TGTGCGCTACTTTTTGGTGTTACTATTCCTGGCCACCAGGGA------------------
------------------------------TCTCTGGGGTCTACAACGGTGCATCCTCTG
---------TTGACGCCATGGCAGGGATTTGGTGTGGAGCTTGTCCTCACCTTCATCGTC
GTCTTCACAGTTTTCGCC---AGTCTGGATTCCCATCGA---------------------
---AAGATTCCC---------------GGTGGCTCCTCACTTGCCATTGGTGCTGCCTAC
CTCACATGTACTCTTGCAGGGTTACCATCAACCGGTGCTGCAGTCAACCCTGCTCGAGCA
CTAGGCCCAGCCTTCGTTATGAAC---------CGATGGACGTATCATTGGGTGTATTGG
TTTGCACCGTTATGCGGAGGGTTATTAGCCGGTCTTTTATATGAATACGTCTTTGATCCT
---CGCAAGACCATCAAGTAC---------CTAAAAGAGACTTTA---GAAGAA------
---------------------GTGGATAAA---------GATTCTAGCATGCAGAGCAAC

GAC------GAT---GACTACGAT---GACCCC---GAA---TCGAAAACTTCTAAGCCT
CCG---GCATCT------------------------------------CAATACAATACG
GGAACATAC---------CGT---------------------------------------
------AGTCAACATTACGATAAT---------------TATCGTCCT---GCCATTTCT
GCCTCG------GCTAACGCTAACGGTCCTACT------------------TACGGA---
---------CCGTCTACT---------GTGAATGCAGGG---AGTTCGAATTACGCT---
------CCCAGTATT---
ACCGGAGGATCAACTTACGGCGCAAAT------------------CCAGGCAACGGGTCC
ATC---TAC------------------------GAAGTGACT---CCATATCGTCCA---

>GCAQ01032452_Scutigera_coleoptrata_Bib
ATGGCG--
------------------------------------TCGGCAGCT------GCTCAGGCG
GCGGACGCCTATCTGCTGCAACTGGTTCAAAAGTTGGAGAGTGCCCGA---CTG------

---CAGCAGCAA

CAAGAAGAACAGCATCAACCCATGAGCCAAGAAATCCGTACTCTG---GAGTTCTGGCGA
GCCATCATCGCCGAATGCTTGGCGGCCTTCTTCTTTGTGTTTCTTTTGTGCGGCAGTCGG
ATCCCTTGGCCGGGCTATGAACCC------------------------------------
---ACG
CATGTTCATTCCGCTCTCGCCTGTGGATTTGCAATGGCTACTTTAGCTCAGTGTTTTGGA
CACATATCGGGGGGGCATGTTAACCCCGCCGTAACATTTGCATTGCTTGTCACGCAGAAA
GTGACCCCTCTGCGGGCCGCCCTCTACATGACTGCCCAATGCGGGGGAGCCATCGCCGGA
GCAGCTCTACTTTACGGTGTTACTGTTCCTGGCTATCAGGGA------------------
------------------------------GATCTGGGAGCAACAGTGCTTCATAATTCT
---------TTATCCCCCTGGCAGGGATTTGGAATAGAATTTGTTCTCACATTTATCATT
GTTTTTACTGTGTTTGCA---ACAATTGATCCCCACAGA---------------------
---CGGATGTTT---------------GGAAGCCCATCGTTAGCTGTTGGAATTGCATAC
CTTGTCTGTTCACTTGCAGGATTTCCGTCAACTGGTGCAGCCGTTAACCCTGCCAGAGCT
TTAGGACCAGCATTTGTAATGAAC---------AAATGGACTTATCATTGGGTGTACTGG
TCTGCCCCAATGTCTGGTGGGCTCCTAGCTGGTCTTATTTATGAATATATCTTTGATCCA
---AGAAGAAGTGTTCGAGGA---------CTGAAAGAAGCATTG---GAAGAATTA---
------------------------GATAGA---------GATTCCAGCATGCAGAGTCAG
GAC------GAG---GATTACGAT---GAACCA---GAG---TCTAAAATGTCCAAATTT
CCT---GCATCC------------------------------------CAGTAT------
GGCTCATAT---------CGTCCTCCTACGGTCACT------------------------
---GGC---CCTCTGTCT
TCT---------GGTGAAGATATCCCAGCACC-------------------TATGGA---
---------ATCCAAAAT---------ACAGATGTTCCC---AGCGCTGGATTT------
------GGATCCATCACT---GCCAACCCTGTTTCT------------------------
AACTACACCACTTCGTATGGCCCAGTC------------------CAAGGCAATGGGTCC
ATT---TAT------------------------GAAGCAGCT---CCATACCGATCT---

--CATTCCTCAGGG
AGTACCTACGCCAGT---------------------------------------ATTCAT
GGAGGG--

---------------------------AGTCGA---------------------------
---ATGGGATCTCTGTCC--


```
------------------------------------------------------------
------------AGAGGAGCTTCC------------------------------------
--------------------------------------CATCGT----------------
-----CTTGATTATGGG------CCAAACTCAACT-------------------------
------------------------------------------------------------
------------------------------------------------------------
------------------------------------------------------------
------------------------------------------------------------
------------------------------------------------------------
------------------------------------------------------------
-----------------------------------------------AAGTTT-
>JAFIDM010000003_Thereuonema_tuberculata_Bib
ATGGCG------------------------------------------------------
------------------------------------TCGGCAGCT------GCTCAGGCA
GCGGACGCCTATCTCCTGCAACTGGTTCAAAAGTTGGAGAGTGCCCGA---CTG------
------------------------------------------------------------
---------------------------------------------------CAGCAACTA
CAAGAGGAACAGCATCGACCCATGAGACAAGAAATTCGTACTTTG---GAGTTCTGGCGA
GCGATCATCGCCGAGTGTTTGGCGGCCTTCTTCTTTGTGTTCCTTTTGTGCAGCAGTCAG
ATCTCTTGGCCAAGTTACGAACCC------------------------------------
---------------------------------------------------------ACG
CACGTTCATTCCGCCCTCACCTGCGGATTTGCAATGGCCACTTTAGCTCAATGTTTCGGA
CACATATCGGGGGGACATGTTAATCCCGCCGTGACATTTGCATTGCTTGTCACGCAGAAA
GTGACCCCTCTACGGGCCGCCCTCTACATGACTGCCCAATGCGGAGGAGCCATCGCGGGA
GCAGCTCTACTCTATGGGGTTACTATCCCTGGCTATCAAGGA------------------
------------------------------GCTTTGGGAGCTTCAATGCTCCATATTTCT
---------CTGTCACCGTGGCAGGGATTTGGAATAGAGTTTGTTCTTACGTTCATCATT
GTTTTCACTGTGTTTGCC---ACGATTGATCCTCACAGA---------------------
---CGAATGTTT---------------GGAAGCTCATCGTTAGCTGTTGGAATAGCATAC
CTTGTGTGTTCACTTGCAGGACTTCCCTCAACTGGTGCAGCTTTGAACCCTGCTCGAGCG
TTAGGACCAGCATTTGTGATGAAC---------AAATGGACTTTTCATTGGGTGTATTGG
TCTGCTCCTATGTCTGGTGGACTCTTAGCAGGTCTTATTTATGAATATATCTTTGATCCA
---AGAAGAAGTGTCCATGGA---------CTGAAAGAAGCCCTG---GAAGAATTA---
------------------------GATAGA---------GATTCCAGCATGCAGAGTCAA
GAT------GAG---GATTATGAC---GAACCA---GAA---TCTAAGATGTCCAAATAC
CCA---ACATCC------------------------------------CAGTACAGCTCC
------TAT---------CGGCCACCTGCAGTCACA------------------------
---------------------------------------------GGT---CCTATGTCA
TCT---------GGAGAAGATATCCCA---------------------------------
---------------------------------------------TCCACTTAT------
------GGA---ATCCAG---AACACAGAT------------------------------
GTTCCTAGCACTGGGTTTGGATCCATC------------------ACTGCCAACCCCGTG
TCTAACTAT------------------------TCTGCTGCC------TACGGCCCAGTC
CAA---------------------------------------------------------
------------------------------------------------------------
------------------------------------------------------------
------------------------------------------------------------
------------------------------------------------------------
```

--GGCAAT---GGG
TCAATTTATGAGGCCGCTCCG---------------------------------TATCGA
TCTCAT---TCCTCA---------GGA---------------------------------
------------------AGTACCTATGCC------------------------------

------------AGC---ATTCAT------GGTGGG------------------------

---------------------------AGCCGA---------------------------
---ATGGGATCTCTGTCC--

------------AGAGGAGCATCA------------------------------------
--------------------------------------CACCGT----------------
-----CTCGATTATGGG------CCAAACTCGACT-------------------------

---AAGTTT-
>GCAP01022361_Scolopendra_cingulata_Bib
ATGGCA---GCCCCG---
---------------------------------------GCG---------GGTCAAGGG
TTCGACCCGCACGTTTTACAATTCTTCCAAAAACTCGACAGTTTGCGTCACGTG------

---CAC
ATCGAAGAAAAGAGAAAGTCGATGCGAGAAGAGCTCCGATCGGTG---GAGTTTTGGCGG
GCCGTCATATCCGAGTGCCTGGCCACTTTCTTCTGCGTCTTCCTCGTGTGTGGGGCCCAC
GTGCCTTGGCCCAGCTACGAACCG------------------------------------
---TCG
CACATCAACATCGCCCTGACCACCGGTTTCGCCATGGCCACGCTCACTCAGTGCTTCGGC
CAAATTTCTGGCGGCCACATTAATCCTGCGGTCACTTTTGCGATGCTATTGACGAGGAAA
GTGACCCCGCTTTGTGCCGTACTTTACGTCATAGCCCAGTGTGGCGGCGCCATCGCCGGT
TGTGCCCTACTCTTTGGGGCGACTATTCCTGGCCACCAGGGA------------------
------------------------------TCTCTGGGATCCACAGTGGTTCACGTCGAT
---------CTCACTCCCTGGCAAGGGTTTGGGGTCGAACTCGTGTTGACTTTCGTAATC
GTTTTCACGGTTTTTGCC---AGCCTGGATTCCCACCGC---------------------
---AAGATCCCC----------------GGTGGTTCATCTCTGGCCATCGGTGCTGCCTAC
CTCACGTGCTCTTTGGCAGGGTTACCTTCAACAGGGGCTGCAGTTAATCCTGCTAGAGCT
TTAGGTCCAGCGTTTGTTATGAAT---------AGATGGAGATATCATTGGGTCTATTGG

```
CTAGCTCCGTTTTGTGGTGGCCTTTTAGCTGGATTACTTTACGAGTACGTGTTTGATCCT
---CGGAAGACCGTCAAAATT---------TTGAAGGAAACCTTA---GAAGATTTG---
------------------------GACCGA------GCAGAATCAAGCATGCACAGCCAG
GAC------GAA---GATTACGAT---GAGACG---GAG---GCGAAATCATGTAAACCT
CAA---GCGACC------------------------------------CAATACTCTTCA
GGTTCTTAT---------CGA---------------------------------------
------AGTCAGCATTACGACTTG---------------TATAGGCCG---ACCATATCT
TCATCG------GCGAACACTAACGGCCTTCA----------------------GCT---
---------ATC---AAC---------ACG------GGA---GGGTCCACGTACGCT---
------CCCAGTATA---------------------------------------------
GCTGGAGGATCCACTTATGCCACCGTC------------------CATGGAAATGGTTCT
ATA---TAC------------------------GAAGCAGCC---CCTTATAGGCCT---
------------------------------------------------------------
------------------------------------------------------------
------------------------------------------------------------
------------------------------------------------------------
------------------------------------------------------------
------------------------------------------------------------
------------------------------------------------CCTTCAACAAAC
AGCACTTATGCCAGC---------------------------------------ATCCAC
GGA---------------------------------------------------------
------------------------------------------------------------
------------------------------------------------------------
------------------------------------------------------------
------------------------------------------------------------
------------------------------------------------------------
------------------------------------------------------------
------------------------------------------------------------
---------------------------AGTCGA---------------------------
---ATGGGTTCAATGGCG------------------------------------------
------------------------------------------------------------
------------------------------------------------------------
------------------------------------------------------------
------------------------------------------------------------
------------------------------------------------------------
------------AGAGGAACTCCC------------------------------------
--------------------------------------CAAAGA----------------
-----ATGGAATACGGT------CCAGGATCAACA-------------------------
------------------------------------------------------------
------------------------------------------------------------
------------------------------------------------------------
------------------------------------------------------------
------------------------------------------------------------
------------------------------------------------------------
-----------------------------------------------AAATTC-
>JAHWFO010084713_Rhysida_immarginata_Bib
ATGGCA---GCCCCG---------------------------------------------
---------------------------------------GCC---------GGTCAAGGG
```

TTCGACCCGCACGTTCTGCAGTTCTTCCAAAAACTCGACAGTTTGCGACATGTA------

---CAT
ATCGAGGAGAAGCGGAAGACGATGCGCGAAGAGTTACGTTCCGTC---GAGTTTTGGCGA
GCCGTCATTTCCGAGTGTTTGGCCACCTTCTTCTGCGTCTTCCTCGTGTGCAGTGCCCAC
GTGTCCTGGCCGGGTTACGAGCCC------------------------------------
---ACG
CACATCAACATAGCGCTCACAACCGGTTTCGCCATGGCCACTTTGACCCAGTGCTTTGGT
CAAATATCTGGCGGCCATATTAACCCTGCTGTCACTTTCGCTATGCTCTTAACACGCAAG
GTGACCCCCCTCTGTGCCGTGCTCTACGTCATTGCCCAGTGCGGCGGTGCCATCGCCGGA
TGTGCTCTCCTTTTCGGGGCAACTATCCCTGGCCACCAGGGA------------------
------------------------------TCTCTTGGGTCCACAAATGTGCATCCCTCT
---------CTAACACCCTGGCAAGGGTTTGGGGTCGAACTCGTGCTAACCTTTGTGGTC
GTCTTCACGGTTTTTGCC---AGCTTGGACTCCCATCGA---------------------
---AAGATCCCC---------------GGCGGTTCATCACTGGCCATCGGCGCCGCCTAT
CTAACGTGCTCCTTGGCAGGGTTACCTTCAACTGGAGCAGCCTTCAATCCTGCTCGAGCT
CTAGGTCCAGCGTTTGTCATGAAC---------AGATGGAGATACCATTGGGTCTATTGG
TTGGCCCCGTTCTGCGGTGGTCTATTAGCCGGACTGCTTTACGAGTACGTGTTTGATCCT
---CGGAAGACAGTGAAGATA---------TTAAAAGAAACGCTG---GAAGATTTA---
------------------------GATCGA------GCAGAATCAAGCATGCACAGTCAA
GAC------GAA---GATTACGAT---GAAACA---GAG---GCCAAGTCCTGTAAACCC
CAG---GCCACC------------------------------------CAATATTCGTCG
GGCTCTTAT---------CGG---------------------------------------
------AGCCAACACTACGATTTA---------------TATAGGCCG---ACCTTATCT
TCCTCC------GCAAACACGAACGGACCTTCG---------------------GCC---
---------ATT---AAC---------ACC------GGA---GGCTCAACCTATGCT---
------CCCAGCATC---
GCTGGAGGCTCGACATACGCCACCGTC------------------CACGGCAATGGTTCC
ATA---TAC------------------------GAGGCGGCT---CCTTACAGACCT---

--CCTTCAACAAAC
AGCACTTACGCCAGC---------------------------------------ATTCAC
GGC---

---------------------------TCCCGG---------------------------
---ATGGGGTCTATGGCC--

------------CGGGGCACGCCG------------------------------------
--------------------------------------CAGAGA----------------
-----ATGGAATACGGA------CCCGGGTCCACT-------------------------

---AAGTTC-
>GESI01022724_Polydesmus_complanatus_Bib
ATGAAAATGGCTCCTATGTCGTACATATCTCACGGAGGGGGAGGTGGTGTCAGT------
---------------------------AAA------GATAAAGAT------GTTAATCTT
CATCTCCTTCGAAACGGCGTTTTATTAAACAGATTAGACTTGTTGGTGCACATG------

---AATCGAAGC
GGTTGGGCTCATAGAAAAACATTTCGAGAAGAAATTCGAAGTCTA---GAGTTTTGGCGG
GCCGTGATAGCCGAATGTTTAGCTTCGTTTTTTTACATCTTTTTCACTTGCGGTTGTTAC
GCTTTG---GAGGCTGAAACTCCG------------------------------------
---TCC
GCTTTTCACTTGGCTTTAGCCAGTGGAAGCGCGATGGCCACGCTCATCTTGTGTTTCGGA
CCAATATCGGGCGGCCACGTTAATCCCGCCGTCACAGTTGCTTTGACGGTTACGCGCAAA
ATTACGTGGCTGCGAATGGTATTTTATGTGACGGCGCAGTGCGGTGGAGCCATTGCGGGA
ACAGCGCTTTTATACGGGATTACTGTTTCTGGTTCTAATGGT------------------
------------------------------AACTTAGGAAACCCTTCCGTTACTTTAAAA
CAA------CTTAGCCCGTTACAAGGATTTGGAGTGGAATTTGTTCTTACGTTCATTATT
GTTATGACCGTTTTCGCC---ACTTTAGATCCATATCAATCT------------------
---CCTATTTTC---------------AATTATCCATCACCTTTGGTGGGAATTGCTTAT
TTAAGTTGTTCTTTAACAGGGATGGGGTCAACTGGCGCTGCACTAAATCCGGCGAGAGCG
CTCGGTCCCGCTTTTGTGATGAAC---------AAATGGACTAATCATTGGATTTATTGG
GGAGGACCTTTAGTTGGAGCGATATTAGCCGGTTTAATATTTGAATATGTATTTGATTCG
---AGAAGAAATCTCAAAAGT---------CTGCACGATCTTATG---GAAGATTTG---
------------------------AATCAG---------GAATCGAGTCTTCAAAGTAAC
GAA------GAA---GACTGTGAT---CATACG---GAG------AATACAAATCATCTT
TCTAGTCCTCCTCCTCAT------------------------------ACCAGCCAAAAT
AACGTTTAC---------CAT------TTC------------------------------
------CATCCTCGGTACGACGAT---------TACAACTGTAGACAA---CGAGGATTA
TTAACT------GCTCCTCATACTAACAGCGGT------------------CATCAG---
------------CAGCAG---------CCT------CCCACGCATTCCAATTATGCACAA
TTTCAGCCCCCACCA---------CCTCCTCCT---------------------------
TTACCGCCTCTCCCACTCCCC------------------------AATGATTACAGAGCT
ATT---CAT------------------------AACGCGGCT------TACAGCGAGAGT
ACTTCG--

--GGAAACGCCGAT
TCGATTTATGAGAGCGCCAGCGCC------------------------------TTTAGA
CCGCAT---TCCAGC------GCGTCG---------------------------------
------------------AGAAATTATTCC------------------------------

------------TCT---ATTTAT------GGA---------------------------

---------------------------ACTAGA---------------------------
---TCTGGC---GTG---

------------AAACCA---CCG------------------------------------
--------------------------------------CAACGA----------------
-----TTAGAGTGCGGTGGA---CCGGGATCTACC-------------------------

---AAGTTT-
>GESA01038252_Haploglomeris_multistriata_Bib
ATGCCGAGCAACAACGCGGATCGCAGATTGGCGGCGATGGTACGAGCGGCGATG------
------------------------------------TCCGCCGCCGCA---GCCGCCAAT
TCTGCATCTTCGCCTCCAGAACTGCTGCAGGGATACGGTGTTGCCGGTAGT---------

------AACCAGAAGAGCAGCGTGCGAGACGAAGTGCGAAGCTTG---CAGTTTTGGCGG
GCGATTATCGCCGAATGTTTGTCCGCGTTTTTCTACGCGTTTCTATTGTGCGGCGCTTGC
GTTCCGTGGCCCGCACCGGGAGCGGTG---------------------------------
---ACGGCGACG
GGAGCGACGGTCGCGTTGACGAGCGGATTCGCGATGTGCACTCTAGTGCAGTGTTTCGGA
CATATTTCAGGAGCGCACGTCAACCCTGCACTCACGTTTGCGTTCGCCATCACTCGTCTT
ATTTCGCCGTTGAGAGCCCTACTTTATGTTACAGCCCAATGTGGAGGAGCAATAGCAGGA
GCAGCGTTACTTTATGGAGTTACCGTACCAGGGTCTCAGGGT------------------
------------------------------AACTTGGGCACCACGACGGTGCATCCGGCC
---------CTAAGCCCATGGCAAGCATTCGGCATAGAGTTCATCCTATCATTCATCGTA
GTGTTCACAGCCATGTCT---GCAGTTGATCCACACAGA---------------------

```
---AGAAGTGTC---------------GCGGGACCCGCGCTGACCATCGGAGCAGCGTAC
CTTGTATGTTCCCTGGCAGGGCTGCCATCGACCGGTGCCGCGATCAACCCAACGCGATCC
CTAGGTCCAGCTTTCGTGATGAAC---------AAGTGGTCTCATCATTGGGTCTATTGG
TTTGGACCTATGGGTGGTGGTGTTCTGGCTGGGATGATCTACGAGTACATCTTCGACCAG
---AAGAAATCGCCAAAGGGA---------CTGCGGGAAGTCCTG---GAAGAACTC---
------------------------GATCGC------GCCGAGTCAAGCGCGAACAGCCAA
GAC------GAA---GACTACGAG---GACCCC---GAT---GCCAAGGTGCCCAAGTAC
GGTATGACAACATCGGCAGGTGCA------------------TCGGGAACGTGGCC----
AGCCAGCCA---------AACGGCCCTTTCCTCCACTCTTCTACGCCAAATCATACGTTC
CAGCGACACCAGCACTATGACAAT---------------TACCGCGCC---GCGCTGCCC
CTT---------------------------------------------------------
------------------------------------------------------------
------CCTCCG------------------------------------------------
CTGCCACCG------------------------------------TCA---AACGGT---
---AACTAC------------------------GGTCCTCTGCCGGGAATGCAG---CAG
GTAACT------------------------------------------------------
------------------------------------------------------------
------------------------------------------------------------
------------------------------------------------------------
------------------------------------------------------------
------------------------------------------------------------
------------------------------------------------CCTTCCGGAGGG
AGCCTGTGCGACTCGGTGCAC---------------------------------TACAGG
CCACCA---AGCAGCAGCGTGGCGACG---------------------------------
------------------GGCAACTACGCC------------------------------
------------------------------------------------------------
------------ACC---ATACAC------GGT---------------------------
------------------------------------------------------------
------------------------------------------------------------
------------------------------------------------------------
------------------------------------------------------------
---------------------------AACAGACCG------------GGCGCG------
---GCGGTCACCATGGCC------------------------------------------
------------------------------------------------------------
------------------------------------------------------------
------------------------------------------------------------
------------------------------------------------------------
------------------------------------------------------------
------------CGA---GTGCCT------------------------------------
--------------------------------------CAAAGG----------------
-----ATGGAATACGGA------CCGGGTGCGACC-------------------------
------------------------------------------------------------
------------------------------------------------------------
------------------------------------------------------------
------------------------------------------------------------
------------------------------------------------------------
------------------------------------------------------------
-----------------------------------------------AAGTTC-
```

```
>AYEL01087177_Mesobuthus_martensii_Bib
ATGGCA------------------------------------------------------
---------------------------------------------------GTGGTGACT
TTGGAATCTTTTTTCCTACACCTGTTAGACAAGGTCGAACACGTTAGGAACCAG------
------------------------------------------------------------
------------------------------------------------------------
GTA---AATCGCAAGAAATCTATGAGAGATGAAGTGAGGACATTA---GAATTCTGGCGG
GGCATCATTGCCGAATGCCTTGCATCGTTTTTCTACGTTTTTATCCTTTGTGGCTCACAC
GTGGTTTGGCAGTCGGAACACACACCG---------------------------------
---------------------------------------------------------GGA
GTTTTAGAGAAAGCTATAACTAGTGGTATGACGATGGCCACACTATCACAGTGTTTCGGA
CATATATCTGGTGCACACATTAACCCTGCTGTAACCTTCGCAGCATTCGTTACGCAGAAA
GTAACACCACTCAGGGCGTTATTATATATAACAGCGCAGTGTGGTGGAGCCATAGCCGGA
GCCGCGTTACTATATGGGGTAACAATTCCTGGACATCTGGGA------------------
------------------------------GCACTGGGAATCTGTGAAGTTTATTCGTCG
---------TTGGGTCCATGGCAGGCGTTTGGGGTCGAATTCGTTCTGACGTTCGTCGTC
GTCTTCACAGTATTCGCC---ACTCTAGACCCCAATAGG---------------------
---AAATCTCTA---------------GGCAGTGATTCTCTGGCTATTGGTATAGCCTAT
CTCGCTTGTTCCATCACAGGGATGCCAGCAAGTGGAGCATCCATGAATCCTGCTCGTTCG
CTAGGACCGGCATTCGTGATGAAT---------CAGTGGGAACATCACTGGGTATATTGG
TTTGGACCTCTAACTGGCGGGCCATTAGCCGGACTCATTTACGAATATATCTTCGATACA
---AAGAAAACTGCCAAAACC---------CTGAAAGAAGCTCTG---GATGAGATT---
------------------------GACAGA---------GAATCC------AATACAGAC
---------GAC---GATTACGAA---GATCCC---GAA---GCGAAAATCAACAAGTAC
AGTAATGCCAGT------------------------------CAAGCACAAAGTGCC---
AGTAATTAC---------AGC---------------------------------------
------CGTCAACATTACGATAAC---------------TATCGACCA---GCTGTCAAC
------------------------------------------------------------
------------------------------------------------------------
------------------------------------------------------------
------------ACTTAC---------------------------TCACCTACTCCATCA
TCAAATTAT------------------------------------CCAGCATCC------
------------------------------------------------------------
------------------------------------------------------------
------------------------------------------------------------
------------------------------------------------------------
------------------------------------------------------------
------------------------------------------------------------
------------------------------------------------------TCCGAA
TCCGTTTACGGA------------------------------------------TCCCAC
GGTGTT---TCTTCT---------------------------------------------
------------------TCAAACTACGCA------------------------------
------------------------------------------------------------
------------AGTGGAGTATAC------GGC---------------------------
------------------------------------------------------------
------------------------------------------------------------
------------------------------------------------------------
------------------------------------------------------------
```

```
---------------------------AGCAGA---------------------------
---TACGCCACAACCATG------------------------------------------
------------------------------------------------------------
------------------------------------------------------------
------------------------------------------------------------
------------------------------------------------------------
------------------------------------------------------------
------------AGAACAGTTCCG------------------------------------
--------------------------------------AGCAGA----------------
-----ATGGAATACGGA------CCGGGTTCTACA-------------------------
------------------------------------------------------------
------------------------------------------------------------
------------------------------------------------------------
------------------------------------------------------------
------------------------------------------------------------
------------------------------------------------------------
-----------------------------------------------AAATTC-
>WUQG01000719_Androctonus_mauritanicus_Bib
ATGGCA------------------------------------------------------
---------------------------------------------------GTGGTGACT
TTGGAATCGTTTTTCCTACACCTGTTAGACAAGGTCGAACACGTTAGGAACCAG------
------------------------------------------------------------
------------------------------------------------------------
GTA---AATCGCAAGAAATCTATGAGAGATGAAGTGAGGACATTA---GAATTCTGGCGG
GGCATCATTGCCGAGTGCCTTGCATCGTTTTTCTACGTTTTTATCCTTTGTGGCTCACAC
GTGGTTTGGCAGTCGGAACACACACCG---------------------------------
---------------------------------------------------------GGA
GTTTTAGAGAAAGCTATAACTAGTGGTATGACGATGGCCACATTATCACAGTGTTTCGGA
CATATATCTGGTGCACACATTAACCCTGCTGTAACCTTCGCAGCATTCGTTACGCAGAAA
GTAACACCACTCAGGGCGTTATTATATATAACAGCGCAGTGTGGTGGAGCCATAGCCGGA
GCCGCGTTACTATATGGGGTAACAATTCCTGGACATCTGGGA------------------
------------------------------GCACTGGGAATCTGTGTAGTTTATTCGTCA
---------TTGGGTCCATGGCAGGCGTTTGGAGTCGAATTCGTTCTGACGTTCGTCGTC
GTCTTCACAGTATTCGCC---ACTCTAGACCCCAATAGG---------------------
---AAATCTCTA---------------GGCAGTGATTCTCTGGCTATTGGTATAGCCTAT
CTCGCTTGTTCCATCACAGGGATGCCAGCAAGTGGAGCATCCATGAATCCTGCACGTTCA
CTGGGACCGGCATTCGTGATGAAT---------CAGTGGGAACATCACTGGGTATATTGG
TTTGGACCTCTAACTGGCGGGCCATTAGCCGGACTCATTTACGAATACATCTTCGATACA
---AAGAAAACTGCCAAAACC---------CTGAAAGAAGCTCTG---GATGAGATT---
------------------------GACAGA---------GAATCC------AATACAGAC
---------GAC---GATTACGAA---GATCCC---GAA---GCGAAAATCAACAAGTAC
AGCAATGCCAGT------------------------------CAAGCACAAAGTGCC---
AGTAATTAC---------AGC---------------------------------------
------CGTCAACATTACGATAAC---------------TATCGACCA---GCCGTCAAC
------------------------------------------------------------
------------------------------------------------------------
------------------------------------------------------------
------------ACTTAC---------------------------TCACCTACTCCATCA
```

TCAAATTAT------------------------------------CCAGCATCC------

--TCCGAA
TCCGTTTACGGA--TCCCAT
GGTGTT---TCTTCT---
------------------TCAAACTACGCA------------------------------

------------AGTGGAGTGTAC------GGC---------------------------

---------------------------AGTAGA---------------------------
---TACGCCACAACCATG--

------------AGAACAGTTCCG------------------------------------
--------------------------------------AGCAGA----------------
-----ATGGAATACGGA------CCGGGCTCTACA-------------------------

---AAATTC-
>GBZU01018641_Tityus_serrulatus_Bib
ATGGCT--
---GTTGTGACT
TTGGAATCGTTTTTCCTACACCTGTTAGACAAGGTCGAGCATGTTCGAAATCAG------

GTA---AATCGTAAGAAATCTATGAAAGATGAGGTACGAACATTA---GAATTCTGGCGA
GGCATTATTGCCGAGTGCCTTGCGTCGTTTTTCTACGTTTTTATTTTGTGTGGCTCGCAC
GTGGGATGGTCCGGACGGGAGCCGGGC---------------------------------

GTGTTAGAAAAAGCTATTAGCAGTGGTGTGACTATGGCCACCCTATCGCAGTGTTTCGGA
CATATATCTGGTGCACACGTGAATCCGGCGGTCACCTTTGCCGCATTCGTAACGCAGAAA
ATAACGCCTCTCAGGGCGTTATTATATATCACAGCGCAATGTGGAGGAGCCATCGCCGGA
GCTGCGTTACTATACGGTGTAACAGTTCCAGGACATCAGGCA------------------

```
------------------------------GCACTGGGGAGCAGTGTAGTTTATTCATCA
---------TTGGGTCCATGGCAAGCATTTGGGGTCGAGTTCGTTCTGACATTCGTTGTT
GTCTTCACAGTATTCGCC---ACTCTAGACCCCAATAGG---------------------
---AAATCTCTA---------------GGCAGCGATTCTCTAGCTATTGGAATAGCCTAT
CTGGCTTGTTCCATCACAGGGATGCCTGCAAGTGGAGCATCCATGAATCCCGCACGTTCG
TTAGGACCAGCATTCGTGATGAAC---------CAATGGAATCATCACTGGGTGTATTGG
TTTGGACCTCTAACCGGTGGACCATTAGCCGGTCTCATCTACGAGTATATCTTCGACACA
---AAGAAAACTGCCAAAACC---------TTGAAAGAAGCTCTG---GATGAGATT---
------------------------GACAGA---------GAATCG------AACACAGAC
---------GAC---GACTACGAG---GATCCC---GAA---GCTAAAATCAACAAGTAC
AGCAACGCGAGT------------------------------CAAGCACAAAGTGCC---
AGCAATTAT---------AGC---------------------------------------
------CGACAACATTACGATAAC---------------TATCGACCA---GCAGTCAAC
------------------------------------------------------------
------------------------------------------------------------
------------------------------------------------------------
------------ACTTAC---------------------------TCACCTACACCATCA
TCTAATTAT------------------------------------CCAGCATCT------
------------------------------------------------------------
------------------------------------------------------------
------------------------------------------------------------
------------------------------------------------------------
------------------------------------------------------------
------------------------------------------------------------
------------------------------------------------------TCAGAA
TCCGTCTATGGA------------------------------------------TCCCAC
GGTGTT---TCTTCT---------------------------------------------
------------------TCCAACTACGCC------------------------------
------------------------------------------------------------
------------AGCGGCGTTTAC------GGT---------------------------
------------------------------------------------------------
------------------------------------------------------------
------------------------------------------------------------
------------------------------------------------------------
---------------------------AGCAGA---------------------------
---TACGCCACCACTATG------------------------------------------
------------------------------------------------------------
------------------------------------------------------------
------------------------------------------------------------
------------------------------------------------------------
------------------------------------------------------------
------------AGAACAGTTCCA------------------------------------
--------------------------------------AGTAGA----------------
-----ATGGAATACGGA------CCTGGGTCTACC-------------------------
------------------------------------------------------------
------------------------------------------------------------
------------------------------------------------------------
------------------------------------------------------------
```


---AAATTC-
>XP_023230709_Centruroides_sculpturatus_Bib
ATGGCT--
---GTGGTGACT
TTGGAATCGTTTTTCCTACACCTGTTAGACAAGGTCGAACACGTTCGAAATCAG------

GTA---AATCGTAAGAAATCCATGAAAGATGAAGTACGAACATTA---GAATTCTGGCGA
GGCATCATTGCCGAGTGCCTTGCGTCGTTTTTCTACGTTTTTATTTTGTGTGGCTCGCAC
GTGGTCTGGTCCGAACGGAAACCGGGC---------------------------------

GTTTTAGAAAAAGCTATAAGCAGTGGTATGACTATGGCCACCCTATCTCAGTGTTTCGGA
CACATATCTGGTGCACACGTGAACCCGGCGGTCACCTTTGCAACATTCGTAACACAGAAA
ATAACGCCTCTCAGGGCGTTATTATATATAACAGCACAATGTGGTGGAGCCATCGCTGGA
GCTGCTTTACTATACGGTGTAACAGTTCCAGGACATCAGGGA------------------
------------------------------GCACTGGGGAGCAGCGTAGTTTATTCGTCA
---------TTGGGTCCATGGCAAGCGTTTGGGGTCGAGTTCGTTCTGACGTTCGTTGTC
GTCTTCACAGTATTCGCC---ACTCTAGACCCCAATAGG---------------------
---AAATCTCTA---------------GGCAGCGATTCTCTAGCTATTGGTATAGCCTAT
CTGGCTTGTTCCATCACAGGGATGCCAGCGAGTGGCGCATCCATGAATCCTGCACGTTCG
TTAGGGCCTGCATTTGTGATGAAC---------CATTGGAATCACCACTGGGTCTATTGG
TTTGGACCTCTAACCGGAGGACCATTAGCTGGCCTCATCTACGAGTACATCTTCGACACA
---AAGAAAACTGCCAAAACT---------CTGAAAGAAGCTCTG---GATGAGATT---
------------------------GACAGA---------GAATCC------AACACAGAC
---------GAC---GATTACGAA---GATCCC---GAA---GCGAAAATCAACAAGTAC
AGCAACGCAAGT------------------------------CAAGCACAAAGTGCC---
AGCAATTAT---------AGC---------------------------------------
------CGCCAACATTACGATAAC---------------TATCGACCA---GCAGTCAAC

------------ACTTAC---------------------------TCCCCTACACCATCA
TCTAATTAT------------------------------------CCAGCATCT------

--TCAGAA
TCCGTCTATGGA--TCCCAT
GGTGTT---TCTTCT---
------------------TCCAACTATGCC------------------------------

------------AGCGGCGTCTAT------GGT---------------------------

---------------------------AGTAGA---------------------------
---TACGCCACTACCATG--

------------AGAACAGTTCCG------------------------------------
--------------------------------------AGCAGA----------------
-----ATGGAATATGGA------CCAGGATCTACC-------------------------

---AAATTC-
>GCAR01005754_Euscorpius_sicanus_Bib
ATGGCT--
---GTTGTGACA
CTAGAATCGTTTTTTCTCCACCTTATGGACAAAGTGGAATGTGTGAGGAATCAG------

GTG---AACCGTAAGAAATCCATGAAGCAGGAAATTCGGACATTA---GAATTTTGGCGC
GGTATTATAGCTGAGTGCCTTGCGACATTTTTTTATGTTTTCATACTATGCGGCTCGTAC
GTTGTGTGGCAGAATCACGAGTCCACC---------------------------------

GTTTCAAATAAAGCGCTAGCCAGTGGAATGACGATGGCAACTTTAGTCCAGTGCTTCGGA
CATATATCTGGCGCACACGTCAATCCAGCTGTGACTTTCGCCATGTTCATAACACAGAAA
GTAACGCCCCTACGAGCGTTACTTTACCTGACTGCCCAGTGCGGAGGTGCAATAGCAGGT
GCTGCGTTATTATACGGTGTCACCGTACCAGGACATCTGGGA------------------
------------------------------GCGCTGGGAAGCAGTGTCGTTCATAGAGAC
---------ATCAGTTCGTGGCAAGCATTTGGGGTCGAATTCGTTCTGACCTTCGTAGTG
GTCTTCACAGTATTTGCT---ACGTTAGATCCAAACAGA---------------------
---AAGTCACTT---------------GGAAGCGATTCTCTTGCTATTGGTATAGCGTAC
CTAGCTTGTTCTATCACAGGGATTCCGGCCAGCGGTGCATCTATGAATCCAGCGCGTTCT
CTGGGACCAGCTTTCGTTATGAAT---------CAGTGGAATCACCATTGGGTGTATTGG
CTAGGTCCTTTGACGGGTGGTCCTTTAGCTGGACTGATATACGAATATATATTTGACACC
---AAGAAAACTGCCAAGAAT---------TTAAAAGATGCTTTG---GATGAAATC---
------------------------GACAGA---------GAATCC------AACACAGAC
---------GAT---GATTACGAG---GACCCA---GAT---TCAAAAATAAATAAATAT
AACAACACCAGT------------------------------CAAGCACAAAGCGCCAGT
AGCAATTAC---------AGA---------------------------------------
------GCGCAGCACTACGATAAC---------------TACAGATCT---ACAGTCAAT

```
------------------------------------------------------------
------------------------------------------------------------
------------ACTTAT---------------------------TCGCCTACTCCATCC
TCCAACTAT------------------------------------CCTGCATCC------
------------------------------------------------------------
------------------------------------------------------------
------------------------------------------------------------
------------------------------------------------------------
------------------------------------------------------------
------------------------------------------------------------
------------------------------------------------------ACTGAA
TCAGTGTATGGC------------------------------------------ACACAT
GGGGTC---TCGACA---------------------------------------------
------------------TCCAACTACGCT------------------------------
------------------------------------------------------------
------------ACCGGTGTCTAT------GGT---------------------------
------------------------------------------------------------
------------------------------------------------------------
------------------------------------------------------------
------------------------------------------------------------
---------------------------GGTAGA---------------------------
---TACGCCACAGCAATG------------------------------------------
------------------------------------------------------------
------------------------------------------------------------
------------------------------------------------------------
------------------------------------------------------------
------------------------------------------------------------
------------AGGACTGTACCG------------------------------------
--------------------------------------AGTAGA----------------
-----ATGGACTACGGA------CCTGGATCAACC-------------------------
------------------------------------------------------------
------------------------------------------------------------
------------------------------------------------------------
------------------------------------------------------------
------------------------------------------------------------
------------------------------------------------------------
-----------------------------------------------AAATTC-
>JAEQBA010002507_4866_3454_0989_Phalangium_opilio_Bib
ATGGCT------------------------------------------------------
---------------------------------------------------CCCATCTCC
TTGGAAGCGTTCTTCCTCCACCTTATCGACAAGGTGGAATCACTGAGAACGCAA------
------------------------------------------------------------
------------------------------------------------------------
ATC---AACAGGAAGCGCTCTATGCGCGAGGAGATCCGAACTTTG---GAATTTTGGCGC
GCAATAATCGCGGAGTGCCTTGCCACCTTCTTCTTCGTGTTTTTATTGTGCGGGGCTCAT
ATCACTTGGCCTGGCTTCTACTCGCCA---------------------------------
---------------------------------------------------------ACG
GTCAGCACCGTGGCCATGACTTGCGGACTGTCGATGGCCACGCTCTCTCAGTGTTTCGGG
```

```
CACATATCAGGGGCGCACGTCAACCCCGCCGTCACCTTCACCATGTTCGTGACGAGAAAA
GTGACTTTGCTCAGAGCCCTCCTCTATATCATTGCCCAGTGCGGGGGTGCCATCGCCGGA
GCCGCCCTTCTCTACGGGGTAACGATTCCTGGCATCAAGGG-------------------
------------------------------ACGTTGGGGAGCACGTTAACCAACGAGCAA
---------CTCAACCCTTGGCAGTCCTTTGGAGTGGAATTTGTGCTAACCTTCGTCGTG
GTTTTCACAGTTTTCGCC---ACGATGGATTCCAGTCGT---------------------
---AAAGCCTTG---------------GGAAGCGACGCCTTGGCCATTGGAATCGCCTAC
CTAGCTTGCTCATTTACCGGGGTACCATCTAGCGGAGCTTCAATGAACCCAGCTAGGTCC
CTGGGACCCGCTTTCGTCATGAAC---------AAATGGACCAATCATTGGGTTTATTGG
CTAGGACCTAGTGCGGGAGGCCTTCTAGCGGGTCTTATCTACGAGTATATCTTCGACACG
---AAAAAGGTGGCTAAAACG---------TTACGAGACGCCCTG---GACGAGTTG---
------------------------GATAGA---------GATTCA------AACACGGAG
---------GAC---GACTACGAA---GATCCA---GAC---AGTAAAATAACCAAATAC
GAGTCAGTAACG------------------------------TTGGCTTCGGCCGCAGTC
TCAAACTAC---------AGG---------------------------------------
------AGTCATAACTACGAC------------------TACCGACCA---GCGGTCAAC
------------------------------------------------------------
------------------------------------------------------------
------------------------------------------------------------
------------ACTTAC---------------------------TCTCCCACCCCTTCG
TCAACCTAC------------------------------------CCGGCTTCA------
------------------------------------------------------------
------------------------------------------------------------
------------------------------------------------------------
------------------------------------------------------------
------------------------------------------------------------
------------------------------------------------------------
------------------------------------------------------ACCGAA
TCAACCTACGGC------------------------------------------GCCCAC
GCTGCC---CTAACCCAGGGTGGAGGT---------------------------------
---------------AACCCCAACTATTCC------------------------------
------------------------------------------------------------
------------GCGGGTGTCTAT------GGC---------------------------
------------------------------------------------------------
------------------------------------------------------------
------------------------------------------------------------
------------------------------------------------------------
---------------------------ACCAGA---------------------------
---TACAACACAGCCATG------------------------------------------
------------------------------------------------------------
------------------------------------------------------------
------------------------------------------------------------
------------------------------------------------------------
------------------------------------------------------------
------------AGAGGTGCGCCA------------------------------------
--------------------------------------AGTAGA----------------
-----ATGGAATACGGA------CCCAGTTCGACC-------------------------
------------------------------------------------------------
```

```
------------------------------------------------------------
------------------------------------------------------------
------------------------------------------------------------
------------------------------------------------------------
------------------------------------------------------------
-----------------------------------------------AAATTT-
>JAALXS010000005_Tachypleus_gigas_Bib6
ATGGCA------------------------------------------------------
---------------------------------------------------ACTATATCC
TTGGAAACGTTTTTCTTGCATCTGATTGACAGGGTGGAGAAACTCCACTCGCAA------
------------------------------------------------------------
------------------------------------------------------------
ATG---ATGAAGCAGCAGACCATTCGAGAGGAAATTAGAACTCTA---GAATTTTGGAGA
TCCATCATCGCAGAGTGCTTAGCAAGTTTCTTTTATGTCTTTCTCCTGTGTGGAGCTCAC
GTGACCTGGCCAGGTCACGATCCAAGT---------------------------------
---------------------------------------------------------AAC
TTGATGATCAAGGCCTTGACCAGCGGTCTAACAATGGCGACCCTGGCTCAGTGCTTTAAT
CACATCTCTGGTGCGCACGTGAACCCTGCCGTGACCTTCACCATGTTTTTGACGAAAAAA
ATATCGCCTCTCAGGGCTAGTCTATACGTTATAGCTCAGTGTGGTGGGGCCATTGCTGGA
GCGGCTCTCCTGTACGGGGTAACAATTCCTGGACACCAGGGT------------------
------------------------------ACGCTGGGGAGCACCCTGGTACACCCTGAT
---------CTGGGACCTTGGCAGGCTTTTGGGATTGAGTTTGTCTTAACGTTTGTGGTT
GTCTTCACGGTGTTTGCT---ACCCTCGATCCCAACAGA---------------------
---AGATCTCTA---------------GGAAGTGATGCACTGGCTGTGGGCATTGGCTAC
TTGGCCACCTCATTGCCCGGGATACCAGCCACTGGAGCAGCGCTAAACCCGGCTCGATCG
TTAGGCCCAGCTTTCGTGATGAAC---------AAATGGCCGCATTACTGGGTGTATTGG
TTCGGCCCCCTGGCGGGCGCTATGGTAGCTGGTTTAATTTATGAATATATTTTTGACACC
---AAGAAAGCTGCCAAAACA---------TTGAAAGAAGCCTTG---GACGAAGTC---
------------------------GACAAA---------GAATCC------AACCTGGAT
GAA------GAT---GATTACGAA---GATATT---GAAAGATCCAAAATGACAAAAGAT
GGA------------------------------------------GGATCCCAGGCAGTT
GGGAGATTT---------AGA---------------------------------------
------GCTCATCATTATGACCAC------------AACTTGAGGCCT---ACCCCTAAC
------------------------------------------------------------
------------------------------------------------------------
------------------------------------------------------------
------------ACTTAT---------------------------TCCCCTACCCCTTCT
ACT---TAT------------------------------------CCAGCACCC------
------------------------------------------------------------
------------------------------------------------------------
------------------------------------------------------------
------------------------------------------------------------
------------------------------------------------------------
------------------------------------------------------------
------------------------------------------------------ACAGAA
TCCACCTATGGA------------------------------------------ACGCAC
AGCGCT---CCTACA---------------------------------------------
------------------TCCAGTTGTAGT------------------------------
```

---------------GGGATCTAT------GGA---------------------------

---------------------------TCTCGG---------------------------
---TTTGGCGGTGGTTTT--

------------AGAGCTGTACCA------------------------------------
--------------------------------------ACCCGT----------------
-----ATGGAATACGAT------CCTGGAAGCACT-------------------------

---AAATTC-
>GGKL01025756_Tachypleus_tridentatus_Bib6
ATGGCA--
---ACCGTGCCG
CTGGAAACGTTTTTCCTTCACTTGCTTAGTAAGGTGGAAAATATTCAGTCGCAG------

ATG---AACAAGCACCAGAGTATCCAGGAAGAAATTAGAACACTG---GAGTTTTGGAGA
TCAATTATTGCAGAATGTCTGGCTAGTTTCTTTTATGTTTTCCTTCTGTGTGGCGCTCAC
GTAACCTGGCCTGGTCATGAACCCAGT---------------------------------
---AAC
TTAATGATTAAGGCCTTGACCAGTGGTCTAACCATGGCTACTCTTGCTCAGAGTTTCAGT
CACATTTCTGGTGCTCACGTGAACCCCGTGGTGACCTTTAGCATGTTCTTGACCAGAAAA
GTATCCTCACTCAGGGCTAGTATGTACGTAATCGCTCAGTGCGGTGGATCAATTGCTGGA
GCGGCCCTCCTGTACGGAGTAACAATTCCTGGACACCAGGGA------------------
------------------------------ACGCTGGGGAGCACCCTGGTGCACCCTGAT
---------CTGGGACCTTGGCAGGCTTTTGGGATTGAGTTTGTCTTAACGTTTGTGGTT
GTCTTCACGGTGTTTGCT---ACCCTCGATCCCAACAGA---------------------
---AGATCTCTA---------------GGAAGTGATGCACTGGCTGTGGGCATTGGCTAC
TTGGCCACATCATTGCCCGGGATACCAGCCACTGGAGCAGCGCTAAACCCGGCTCGATCG
TTAGGCCCAGCTTTCGTGATGAAC---------AAATGGCCGCATTACTGGGTGTACTGG
TTCGGCCCCCTGGCGGGAGCTATGGTAGCTGGTTTAATTTATGAATATATTTTTGACACC
---AAGAAAGCTGCCAAAACA---------TTGAAAGAAGCCTTG---GACGAAGTC---
------------------------GACAAA---------GAATCC------AACCTGGAT
GAA------GAT---GATTACGAA---GATCCT---GAAAGATCCAAGATGACAAAAGAT
GGA--GGATCACAGGCAGTT

GGGAGATTT---------AGA---------------------------------------
------GCTCACTATTATGACCAC------------AACTTGAGGCCT---ACCCCTAAC

------------ACTTAT---------------------------TCCCCTACCCCTTCT
ACT---TAT------------------------------------CCAGCACCC------

--ACAGAA
TCCACCTATGGA--ACGCAC
AGCGCT---CCTACA---
------------------TCCAGTTGTAGT------------------------------

---------------GGGATCTAT------GGA---------------------------

---------------------------TCTCGG---------------------------
---TTTGGCGGTGGTTTT--

------------AGAGCTGTACCA------------------------------------
--------------------------------------ACCCGT----------------
-----ATGGAATACGAT------CCTGGAAGCACT-------------------------

---AAATTC-
>WCHO01030093_Carcinoscorpius_rotundicauda_Bib6
ATGGCA--
---ACTATATCC
TTGGAAACGTTTTTCTTGCATCTGATTGACAGGGTGGAGAAACTCCACTCGCAA------

ATG---ATGAAGCAGCAGACCATTCGAGAGGAAATTAGAACTCTA---GAGTTTTGGAGA
TCCATCATAGCAGAGTGCTTAGCAAGTTTCTTTTATGTCTTTCTCCTGTGTGGAGCTCAC

GTGACCTGGCCAGGTCACGATCCCAGT---------------------------------
---AAC
TTGATGATCAAGGCCTTGACCAGCGGTCTAACAATGGCGACCCTGGCTCAGTGCTTTAAC
CACATCTCTGGTGCGCACGTGAACCCTGCCGTGACCTTCACCATGTTTTTGACGAAAAAA
ATATCGCCTCTCAGGGCTAGTCTGTACGTTATAGCTCAGTGTGGTGGGGCCATTGCTGGA
GCGGCTCTCCTGTACGGGGTAACAATTCCTGGACACCAGGGT------------------
------------------------------ACACTGGGGAGCACCCTGGTACACCCTGAT
---------CTGGACCTTGGCAGGCTTTTGGATTGAGTTTGTCTTAACGTTTGTAGTT
GTCTTCACGGTGTTTGCT---ACCCTCGATCCCAACAGA---------------------
---AGATCTCTA---------------GGAAGTGATGCACTGGCTGTGGCATTGGCTAC
TTGGCCACCTCATTGCCAGGGATACCAGCCACTGGAGCAGCGCTAAACCCTGCTCGATCG
TTAGGCCCAGCTTTCGTGATGAAC---------AAATGGCCGCATTACTGGGTGTATTGG
TTCGGCCCCCTGGCGGGAGCTATGGTAGCTGGTTTAATTTATGAATATATTTTTGACACC
---AAAAAAGCTGCCAAAACA---------TTGAAAGAAGCCTTG---GACGAAGTC---
------------------------GACAAA---------GAATCC------AACCTGGAT
GAA------GAT---GATTACGAA---GATCCT---GAAAGATCCAAGATGACAAAAGAT
GGA--GGATCTCAGGCAGTT
GGGAGATTT---------AGA---------------------------------------
------ACTCAGTATTATGACCAC------------AACTTGAGGCCT---ACCCCTAAC

------------ACTTAT---------------------------TCCCCTACCCCTTCT
ACT---TAT------------------------------------CCAGCACCC------

--ACAGAA
TCCACCTATGGA--ACGCAC
AGCGCT---CCTACA---
------------------TCCAGTTGTAGT------------------------------

---------------GGGATCTAT------GGA---------------------------

---------------------------TCTCGG---------------------------
---TTTGGCGGTGGTTTT--

------------AGAGCTGTACCA------------------------------------

--------------------------------------ACTCGT----------------
-----ATGGAATACGAT------CCTGGAAGCACT-------------------------

---AAATTC-
>AZTN01176538_XM_013927469_Limulus_polyphemus_Bib6
ATGGCA--
---ACCATATCC
TTGGAAACGTTTTTCCTGCATTTGATTGATAGAGTGGAGAAACTCCACTCTCAA------

ATG---AACAAGCACAAAACCATTCGAGAAGAAATCAGGACGCTC---GAATTTTGGAGA
TCCATAATTGCAGAATGTTTAGCAAGTTTCTTCTACGTATTTCTCCTGTGTGGAGCTCAC
GTGACCTGGCCAGGTCACGACCCCAGT---------------------------------
---AAC
TTGATGATCAAGGCCTTGACCAGTGGTCTAACAATGGCTACCTTGGCTCAGTGCTTTAAT
CACATCTCTGGTGCACACGTGAACCCCGCCGTGACCTTCACCATGCTTCTGACAAAAAAA
ATATCGTTTCTCAGGGCTAGTCTGTATGTTATAGCTCAATGTGGCGGGGCCATCGCTGGA
GCGGCTCTCTTGTATGGGGTAACAATTCCTGGACACCAGGGT------------------
------------------------------ACATTGGGGAGCACCCTGGTACATCCTGAC
---------CTGGGACCGTGGCAGGCTTTTGGAATTGAATTCGTCTTGACGTTTGTAGTG
GTCTTCACAGTGTTTGCT---ACACTTGACCCCAACAGA---------------------
---AGGTCTCTA---------------GGAAGTGATGCACTTGCCGTGGGTATTGGCTAC
TTGGCCACGTCACTGCCAGGGATACCAGCCACTGGAGCGTCGTTAAACCCAGCTCGATCC
CTGGGCCCCGCTTTTGTAATGAAC---------AAATGGCCGCATTACTGGGTGTACTGG
TTCGGCCCCCTGGCGGGAGCCATGGTAGCAGGTCTGATTTATGAATATATTTTTGACACC
---AAGAAAGCTGCCAAAACA---------TTGAAAGAAGCCTTA---GACGAAGTC---
------------------------GAAAAA---------GAATCC------AACCTGGAT
GAA------GAT---GATTACGAA---GATCCT---GAAAGATGCAAGATGACAAAAGAC
GGA--GGCTCACAGACGACC
GGGAGATTC---------AGA---------------------------------------
------GCTCAGTATTATGACCAC------------AACTTGAGGCCC---GCCCCCAAC

------------ACTTAT---------------------------TCCCCTACCCCTTCT
ACC---TAT------------------------------------CCAGCATCC------

--ACAGAA

TCCACCTACGGA--ACGCAC
AGCGCC---CCTACA---
------------------TCCAGTTATAGC------------------------------

---------------GGGATCTAT------GGA---------------------------

---------------------------TCTCGG---------------------------
---TTTGGAGGTGGTTTT--

------------AGAGCTGTACCA------------------------------------
--------------------------------------ACCTGT----------------
-----ATGGAATATGAT------CCTGGAACTACT-------------------------

---AAATTC-
>GILM01011930_Tachypleus_gigas_Bib5
ATGGCA--
---TCCATATCG
TTGGAATCGTTTTTTTTACATCTAATTGATAGGGTGGAGAAAATGCACTTGGAA------

ATG---AACAAACAAAAAAGCATCAAAGAGGAAATTAAGACGCTT---GAATTTTGGAGG
TCCATAATAGCAGAGTGCTTAGCAAGCTTCTTCTATGTTTTTCTTTTGTGTGGAGCTCAT
GTCACTTGGCCAGGTCACGACCCCAGC---------------------------------
---AAC
TTGACCACTAAGGCCTTGACCAGTGGTCTAACAATGGCTACTCTGACTCAGTGTTTCAGT
CATATTTCTGGTGCACACGTGAATCCTGCTGTGACTTTTACCATGTTTGTGACCAGGAAA
ATTTCACCCCTCAGGGCTAGTCTGTATGCCCTAGCTCAGTGTGGTGGGGCCATTGCTGGA
GCGGCTCTCCTTTACGGGGTTACAATTCCTGGACACCAAGGC------------------
------------------------------ACTCTGGGTAGTACTTTGGTACATGATGAC
---------CTAGGACCATGGCAGGCTTTTGGAATCGAATTCGTTTTGACGTTTGTTGTG
GTGTTTACAGTATTTGCT---ACGCAAGACCCCAACAGA---------------------
---AGATCTCTA---------------GGAAGTGATGCCCTGGCTGTAGGTATTGGCTAT
CTAGCCACATCTTTTCCTGGGATACCAGCCACAGGAGCGTCGTTAAATCCGGCTCGCTCG
CTGGGCCCAGCTTTTGTAATGAAC---------AAATGGCCTCATCACTGGGTATATTGG
TTTGGCCCTCTGGCAGGAGCTGTGATTGCAGGACTTATCTATGAGTATATTTTTGACACC
---AAGAAAACTGCCAAAACA---------TTGAAAGAAGCATTA---GACGAAGTT---

------------------------GATAAA---------GAATCC------AACCTAGAT
GAA------GAT---GACTATGAA---GATCCC---GAGAGGTGCAAGATGACCAAAGAT
GGA--GGTCCTCAAACATCT
GGAGGTTTC---------CGA---------------------------------------
------TCTCAATATTACGATCAG------------AACTTGAGGCCT---GCCCCTAAC

------------ACCTAC---------------------------TCTCCAACCCCATCT
ACC---TAT------------------------------------CCAGCGTCT------

--ACTGAG
TCCAATTATGGC--ATGCAC
AGTGCT---CCTGCA---
------------------TCCAATTATAGT------------------------------

---------------GGGATTTAC------GGG---------------------------

---------------------------TCTCGA---------------------------
---TACGGAGGTGCTTTC--

------------AGGGCTGTGCCA------------------------------------
--------------------------------------ACTCGA----------------
-----ATGGAGTACGAT------CCAGGGACAACT-------------------------

---AAATTC-
>QXHF01003839_Tachypleus_tridentatus_Bib5
ATGGCA--
---TCCATATCG
TTGGAATCGTTTTTTTTACATATAATTGATAGGGTGGAGAAAATGCACTTGGAA------

ATG---AACAAACAAAAGAGCATGAGAGAGGAGATTAAGACGCTT---GAATTTTGGAGG
TCCATAATAGCAGAGTGCTTAGCAAGCTTCTTTTATGTTTTTCTTTTGTGTGGAGCTCAT
GTCACTTGGCCAGGTCACGACCCCAGC---------------------------------
---AAC
TTGACCACTAAGGCCTTGACCAGTGGTCTAACAATGGCTACTCTGACTCAGTGTTTCAGT
CATATTTCTGGTGCACACGTGAACCCTGCTGTGACTTTTACCATGTTCGTGACCAGGAAG
ATTTCACCCCTCAGGGCTGGTCTGTATGCCCTAGCTCAGTGTGGTGGAGCCATTGCTGGA
GCGGCTCTCCTTTACGGGGTTACAATTCCTGGACACCAAGGT------------------
------------------------------ACTCTGGGCAGTACTTTGGTACATGATGAC
---------CTAGGACCATGGCAGGCTTTTGGAATCGAATTCGTCTTGACGTTTGTTGTG
GTGTTTACAGTATTTGCT---ACGCGAGACCCCAACAGA---------------------
---AGATCTCTA---------------GGAAGTGATGCCCTGGCTGTAGGTATTGGCTAT
CTAGCCACATCTTTTCCTGGGATACCAGCCACAGGAGCGTCGTTAAATCCGGCTCGCTCG
CTGGGCCCAGCTTTTGTAATGAAC---------AAATGGCCTCATCACTGGGTATATTGG
TTTGGCCCTCTGGCAGGAGCTGTGATTGCAGGACTTATCTATGAGTATATTTTTGACACC
---AAGAAAACTGCCAAAACA---------TTGAAGGAAGCATTA---GACGAAGTT---
------------------------GATAAA---------GAATCC------AACCTAGAT
GAA------GAT---GACTATGAA---GATCCC---GAGAGGTGCAAGATGACCAAAGAT
GGA--GGTCCTCAAACATCT
GGGGGTTTC---------CGA---------------------------------------
------TCTCAATATTACGATCAG------------AACTTGAGGCCT---GCCCCTAAC

------------ACCTAC---------------------------TCCCCATCTCCATCT
ACC---TAT------------------------------------CCAGCGTCT------

--ACTGAG
TCCAATTATGGG--ATGCAA
AGTGCT---CCTGGA---
------------------TCCAATTATAGT------------------------------

---------------GGGATTTAC------GGG---------------------------

---------------------------TCTCGA---------------------------
---TACGGAGGTGCTTTC--

------------AGGGCTGTGCCA------------------------------------
--------------------------------------ACTCGA----------------
-----ATGGAGTACGAT------CCAGGGACAACT-------------------------

---AAATTC-
>CM022393_Carcinoscorpius_rotundicauda_Bib5
ATGGCA--
---TCCATATCT
TTGGAATCGTTTTTTTTACATCTAATTGACAGGGTGGAGAAAATGCACTTGGAA------

ATG---AACAAACAAAAAAGCATGAGAGAGGAGATTAAGACGCTT---GAATTTTGGAGG
TCCATAATAGCAGAGTGCTTAGCGAGTTTCTTCTATGTTTTTCTTTTGTGTGGAGCTCAT
GTCACTTGGCCAGGTCACGACCCCAGC---------------------------------
---AAC
TTGACCACTAAGGCCTTGACCAGTGGTCTCACAATGGCTACTCTGACTCAGTGTTTCAGT
CATATTTCTGGTGCACACGTGAACCCTGCTGTGACTTTCACCATGTTCGTGACCAGGAAA
ATTTCACCCCTCAGGGCTAGTCTGTATGCCCTAGCTCAGTGTGGTGGAGCTATTGCTGGA
GCGGCTCTCCTGTACGGGGTTACAATTCCTGGACACCAAGGT------------------
------------------------------ACTCTGGGCAGTACTTTAGTACATGATGAC
---------CTGGGACCATGGCAGGCTTTCGGAATCGAATTCGTCTTGACGTTTGTTGTG
GTGTTTACAGTATTTGCC---ACGCAAGACCCCAACAGA---------------------
---AGATTTCTA---------------GGAAGTGACGCTCTGGCTGTAGGTATTGGCTAT
CTAGCCACATCTTTTCCTGGGATACCAGCCACAGGAGCGTCATTAAATCCGGCTCGCTCG
CTAGGCCCAGCTTTTGTAATGAAC---------AAATGGCCTCATCACTGGGTATATTGG
TTTGGCCCTCTGGCAGGAGCTGTGATTGCAGGACTTATCTATGAGTATATTTTTGACACC
---AAGAAAACTGCCAAGACA---------CTGAAAGAAGCATTA---GACGAAGTT---
------------------------GATAAA---------GAATCC------AACCTAGAT
GAA------GAT---GACTATGAA---GATCCC---GAGAGGTGCAAGATGACCAAAGAT
GGA--GGTCCTCAAACATCT
GGGGATTTC---------CGA---------------------------------------
------TCTCAATATTACGATCAG------------AACTTGAGGCCT---GCCCCTAAC

------------ACCTAC---------------------------TCCCCAACCCCATCT
ACC---TAT------------------------------------CCAGCGTCT------

--ACTGAG
TCCAATTATGGC--ATGCAC
AGTGCT---CCTGCA---
------------------TCCAATTATAGT------------------------------

---------------GGGATTTAC------GGG---------------------------

---------------------------TCTCGA---------------------------
---TACGGAGGTGCTTTC--

------------AGGGCTGTGCCA------------------------------------
--------------------------------------ACTCGA----------------
-----ATGGAGTATGAT------CCAGGGACAACT-------------------------

---AAATTC-
>LOC106470722_Limulus_polyphemus_Bib5
ATGGCA--
---ACCATATCG
TTGGAATCGTTTTTTTTACACCTAATTGATAGGGTGGAGAAAATGCACTTGCAA------

ATG---AACAAGCAAAAAAGCATCAGAGAGGAGATTAAGACGCTT---GAATTTTGGAGA
TCCATAATAGCAGAATGTTTAGCAAGCTTCTTCTATGTCTTTCTTTTGTGTGGAGCTCAC
ATTACTTGGCCAGGTCACGATCCCAGC---------------------------------
---AAC
TTGACCATCAAGGCCTTGACCAGTGGTCTAACAATGGCTACTCTGACTCAGTGTTTCAGT
CATATCTCTGGTGCACATGTGAACCCTGCTGTGACTTTTACCATGTTTGTGACCAGGAAA
ATATCATCTCTCAGGGCTAGTCTGTATGCGGTAGCTCAGTGTGGTGGAGCCATTGCTGGA
GCAGCTCTCCTTTACGGGGTTACAATTCCTGGACACCAAGGT------------------
------------------------------ACTCTGGGCAGTACTTTGGTCCATGATGAC
---------CTAGGACCGTGGCAAGCTTTTGGAATCGAATTCGTCTTGACGTTTGTAGTG
GTATTTACAGTATTTGCT---ACACAGGATCCCAACAGA---------------------
---AGGTCTTTA---------------GGAAGTGATGCACTGGCTGTAGGTATTGGCTAC
CTGGCCACATCTTTTACTGGGATACCAGCCACAGGAGCGTCGTTAAATCCGGCTCGTTCG

CTAGGCCCAGCTTTTGTAATGAAC---------AAATGGCCTCATCACTGGGTA------

>JAALXS010000003_Tachypleus_gigas_Bib4
ATGGCC--

---ACCATACCT
TTCGAAACGTTCATTTTACACCTGATTGACAGGGTGGAAAAACTTCAGGTGCAG------

GTA---AACAAGAACAGAAGTATTCGTGAAGAGGTGAGGACGCTG---GAGTTCTGGAGG
TCCATTATTGCAGAGTGCTTAGCAAGTTTCTTCTACGTGTTTCTTCTCTGTGGAGCTCAT
GTCTCCTGGCCCGGTCACGACCCCAGT---------------------------------
---AAT
TTAATGATCAAGGCCTTAACCAGTGGTTTGACAATGGCCACTTTGGCTCAGTGCTTTAAT
CATATCTCTGGTGCTCATGTTAACCCCGCCGTGACATTTACTATGTTTCTGACCAGAAAA
ATATCCCCCCTCAGAGCTAGTTTGTATGGAATAGCTCAGTGTGGAGGCTCAATCGCTGGG
GCTGCTCTGCTGTACGGGGTAACAATTCCTGGACACCAGGGA------------------
------------------------------ATGCTTGGAAATACTTTGGTACATCCTGAT
---------CTGGGGCCGTGGCAGGCTTTTGGAATTGAGTTTGTCTTGACTTTTGTGGTG
GTATTCACGGTGTTTGCC---ACACAGGATCCCGACAGA---------------------
---AAATTTTTC---------------GGTAGCGATGCACTGGCTGTGGGTATCGGCTAT
CTGGCAACGTCTTTACCTGGGATACCAGCAACGGGAGCTTCTTTAAATCCAGCACGCTCA
CTGGGACCTGCGTTTGTGATGAAC---------AAATGGCCTCACCATTGGGTATATTGG
TTCGGCCCTCTGGCGGGAGCACTTGTAGCAGGACTGATTTACGAATATATTTTTGACACC
---AAGAAAACTATCAAAACG---------CTTAGAGAAGCCATC---AATGAAGTC---
------------------------GACAAA---------GAATCT------AACTTGGAT
GAA------GAT---GGC---GAA---AATCCT---GAAAGATGTAACATGACCAAAGAT
GGC--AGCCCGCAGAACTAC
GAGAGTTAT---------GGA---------------------------------------
------GCTCAGTGCTATGATCAA------------ACCATAAGGCCT---GCCCTCAAT

------------ACCTAC---------------------------TCTCCAACTCATTCT
ACC---TAT------------------------------------CCAGTGTTC------

--ACAGAG
TCCTCTTACGGA--ACTCAC
AGTGGT---CCCTCC---
------------------TCCAACTATAGC------------------------------

---------------GGAATATAC------GGA---------------------------

---------------------------TCTCGT---------------------------
---GGTAGGAGTGGTTTC--

------------AGAACTGTGCCA------------------------------------
--------------------------------------ACGCGA----------------
-----ATGGAATATGAC------CGTGGGACTACT-------------------------

---AAATTC-
>QXHF01019598_Tachypleus_tridentatus_Bib4
ATGGCC--
---ACCATACCT
TTCGAAACGTTTATTTTACACCTGATTGACAGGGTGGAAAAACTTCAGGTGCAG------

GTA---AACAAGAACAGAAGTATTCGTGAAGAGGTGAGGACGCTG---GAGTTCTGGAGG
TCCATTATTGCAGAGTGCTTAGCAAGTTTCTTCTACGTGTTTCTTCTCTGTGGGGCTCAT
GTCTCCTGGCCCGGTCACGACCCCAGT---------------------------------
---AAC
TTAATGATCAAGGCTTTAACCAGTGGTTTGACAATGGCCACTTTGGCTCAGTGCTTTAAT
CATATCTCTGGTGCTCATGTTAACCCCGCCGTGACATTTACCATGTTTCTGACCAGAAAA
ATATCCCCCCTCAGAGCTAGTTTGTATGGAATAGCTCAGTGTGGAGGCTCAATCGCTGGG
GCTGCTCTGCTGTACGGGGTAACAATTCCTGGACACCAAGGA------------------
------------------------------ATGCTTGGAAATACTTTGGTACATCCTGAT
---------CTGGGGCCGTGGCAGGCTTTTGGAATTGAGTTTGTCTTGACTTTTGTGGTG
GTATTCACGGTGTTTGCC---ACACAGGATCCCGACAGA---------------------
---AGATCTTTC---------------GGTAGCGATGCACTGGCTGTGGGTATCGGCTAT
TTGGCAACATCTTTACCTGGGATACCAGCAACGGGAGCTTCTTTAAATCCAGCGCGCTCA
CTGGGACCTGCTTTTGTGATGAAC---------AAATGGCCACATCATTGGGTATATTGG
TTCGGCCCTCTGGCGGGAGCACTTGTAGCAGGACTGATTTACGAATATATTTTTGACACC
---AAGAAAACTATCAAAACA---------CTTAGAGAAGCCATC---AATGAAGTC---
------------------------GACAAA---------GAATCT------AACTTGGAT
GAA------GAT---GGC---GAA---AATCCT---GAAAGATGTAACATGACCAAAGAT
GGC--AGCCCGCAGAACTCC
GAGAGTTAT---------GGA---------------------------------------
------GCTCAGTGCTACGATCAA------------ACCATAAGGCCT---GCCATAAAT

------------ACCTAC---------------------------TCTCCAACTCATTCT
ACC---TAT------------------------------------CCAGTGTTC------

--ACAGAG
TCCTCTTACGGA--ATTCAC
AGTGGT---CCCTCC---
------------------TCCAACTATAGC------------------------------

---------------GAAATATAC------GGA---------------------------

---------------------------TCTCGT---------------------------
---GGTAGAAGTGGTTTT--

------------AGAACTGTGCCA------------------------------------
--------------------------------------ACGCGA----------------
-----ATGGAATATGAC------CGTGGGACTACA-------------------------

---AAATTC-
>CM022393_Carcinoscorpius_rotundicauda_Bib4
ATGGCC--
---ACCATACCT
TTCGAAACGTTTATTTTGCACTTGATTGACAGAGTGGAAAAACTTCAGGTGCAG------

GTA---AACAAAAACAGAAGCATTCGTGAAGAGGTGAGGACGCTG---GAGTTCTGGAGG
TCCATTATTGCAGAGTGCTTAGCAAGTTTCTTCTACGTGTTTCTTCTCTGTGGGGCTCAT
GTCTCCTGGCCCGGTCACGACCCCAGT---------------------------------
---AAC
TTAATGATCAAGGCCTTAACAAGTGGTTTGACAATGGCCACTTTGGCTCAGTGCTTTAAT
CATATTTCTGGTGCTCATGTTAACCCCGCCGTGACATTTACTATGTTTCTGACCAGAAAA
ATATCCCCTCTCAGAGCTAGTTTGTATGGAATAGCTCAGTGTGGAGGCTCAATCGCTGGG
GCTGCTCTGTTGTACGGGGTAACAATTCCTGGACACCAGGGA------------------
------------------------------ATGCTTGGAAATACTTTGGTACATCCCGAT
---------CTGGGGCCATGGCAGGCTTTTGGAATTGAGTTTGTCTTGACTTTTGTGGTG

```
GTATTCACGGTGTTTGCC---ACACAGGATCCCGACAGA---------------------
---AGATCTTTC---------------GGTAGCGATGCACTGGCTGTGGGTATCGGCTAT
CTGGCAACATCTTTACCTGGGATACCAGCAACGGGAGCTTCTTTAAATCCAGCGCGCTCA
CTGGGACCTGCTTTTGTGATGAAC---------AAATGGCCGCACCATTGGGTATATTGG
TTCGGCCCTCTGGCAGGAGCACTTGTAGCAGGACTGATTTACGAATATATTTTTGACACC
---AAGAAAACTATCAAAACA---------CTTAGAGAAGCCATC---AATGAAGTC---
------------------------GACAAA---------GAGTCT------AACTTGGAT
GAA------GAT---GGC---GAA---AATCCT---GAAAGATGTAACATGACCAAAGAT
GGC------------------------------------------AGTCCGCAGAACTCC
GAGAGTTAT---------GGA---------------------------------------
------GCTCAGTGCTACGATCAA------------ACCATAAGGCCT---GCCCTCAAT
------------------------------------------------------------
------------------------------------------------------------
------------------------------------------------------------
------------ACCTAC---------------------------TCTCCAACTCATTCT
ACC---TAT------------------------------------CCAGTGTTC------
------------------------------------------------------------
------------------------------------------------------------
------------------------------------------------------------
------------------------------------------------------------
------------------------------------------------------------
------------------------------------------------------------
------------------------------------------------------ACAGAG
TCCTCTTACGGA------------------------------------------ATTCAC
AGTGGT---CCCTCC---------------------------------------------
------------------TCCAACTATAGA------------------------------
------------------------------------------------------------
---------------GGAATATAC------GGA---------------------------
------------------------------------------------------------
------------------------------------------------------------
------------------------------------------------------------
------------------------------------------------------------
---------------------------TCTCGT---------------------------
---GGTAGGAGTGGTTTC------------------------------------------
------------------------------------------------------------
------------------------------------------------------------
------------------------------------------------------------
------------------------------------------------------------
------------------------------------------------------------
------------AGAACTGTGCCA------------------------------------
--------------------------------------ACGCGA----------------
-----ATGGAATATGAC------CGTGGGACTACT-------------------------
------------------------------------------------------------
------------------------------------------------------------
------------------------------------------------------------
------------------------------------------------------------
------------------------------------------------------------
------------------------------------------------------------
```

---AAATTC-
>XM_013918331_Limulus_polyphemus_Bib4
ATGGCC--
---ACCATATCC
TTCGAAACGTTTTTTTTACACCTGATTGATAGGGTGGAAAAACTTCAGGTGCAG------

GTG---AACAAGAACAGAAGTATTCGTGAGGAGATAAGGACGCTA---GAGTTCTGGAGG
TCGATTATTGCAGAGTGCTTAGCAAGTTTCTTCTACGTCTTTCTTCTCTGTGGGGCTCAT
GTCTCCTGGCCCAATCATGACCCCAGT---------------------------------
---AAC
TTAATGATCAAGGCCTTGACCAGTGGTTTAACAATGGCCACTTTGGCTCAGTGCTTTAAT
CATATCTCTGGTGCTCATGTTAACCCCGCCGTGACCTTTACAATGTTTGTGACCAGAAGA
ATATCCCCTCTCAGGGCTAGTTTGTATGTAATAGCTCAGTGTGGAGGCTCAATCGCTGGG
GCTGCTCTGCTGTACGGTGTAACAATTCCTGGACACCAGGGA------------------
------------------------------ACGCTTGGAAATACTTTGATACATCCTGAT
---------CTGGGGCCGTGGCAGGCTTTTGGGGTTGAATTTGTCTTGACGTTTTTGGTG
GTATTCACGGTGTTTGCC---ACACAGGATCCCAACAGA---------------------
---AGATCCTTA---------------GGAAGCGATGCACTGACTGTGGGTATTGGCTAT
CTGGCCACCTCTCTGCCTGGGATACCAGCAACAGGAGCTTCTTTAAATCCAGCTCGCTCT
CTGGGACCTGCCTTTGTGATGAAC---------AAATGGCCTCACCATTGGGTATATTGG
TTCGGCCCTCTGGCGGGAGCAGTTGTAGGAGGACTGATTTATGAGTATATTTTTGATACC
---AAGAAAGCTATCAAAACA---------CTCAGAGAAGCCATC---GATGAAGTT---
------------------------GATAAA---------GAATCT------AACCTGGAT
GAA------GAT---GACTACGAA---AATCCT---GAAAGATGTAACGTGACCAAAGAT
GGA--GGCCCGCAGACTTCC
GGGACTTAT---------GGA---------------------------------------
------GCTCAGTACTACGATCAA------------ACCATAAGGCCC---GCTCTCAAT

------------ACATAC---------------------------TCTCCAACTCATTCT
ACC---TAT------------------------------------CCAGTTTTC------

--ACAGAG
TCCAGCTACGGA--ACTCAC
AGTGGC---CCCTCC---
------------------TCCAACTATAGC------------------------------

---------------GGAATATAT------GGA---------------------------


```
------------------------------------------------------------
---------------------------TCTCGT---------------------------
---GGTGGGGGTGGTTTC------------------------------------------
------------------------------------------------------------
------------------------------------------------------------
------------------------------------------------------------
------------------------------------------------------------
------------------------------------------------------------
------------AGAACTGTGCCA------------------------------------
--------------------------------------GCGCGA----------------
-----ATGGAATATGAC------CCTGGGACTACT-------------------------
------------------------------------------------------------
------------------------------------------------------------
------------------------------------------------------------
------------------------------------------------------------
------------------------------------------------------------
------------------------------------------------------------
-----------------------------------------------AAATTC-
>JAALXS010000002_Tachypleus_gigas_Bib3
ATGGCA------------------------------------------------------
---------------------------------------------------ACCGTGCCG
CTGGAAACGTTTTTCCTTCACTTGCTTAGTAAGGTGGAAAATATTCAGTCGCAG------
------------------------------------------------------------
------------------------------------------------------------
ATA---AACAAGCACCAGAGTATCCAGGAAGAAATTAGGACACTG---GAGTTTTGGAGA
TCAATTATTGCTGAATGTCTGGCTAGTTTCTTTTATGTTTTCCTACTGTGTGGCGCTCAC
GTGACCTGGCCTGGTCATGAACCCAGT---------------------------------
---------------------------------------------------------AAC
TTAATGATTAAGGCCTTGACCAGTGGTCTAACCATGGCTACTCTTTCTCAGAGTTTCAGT
CACATTTCTGGTGCTCACGTGAACCCCGTGGTGACTTTTAGCATGTTCTTGACCAGAAAA
GTATCATCACTCAGGGCTAGTATGTACGTAATAGCTCAGTGTGGTGGATCAATTGCTGGA
GCGGCCCTCTTATACGGGGTAACAATTCCTGGACATCAGGGG------------------
------------------------------ATATTGGGCGCCACCTTGGTTCATCCTGAT
---------CTGGGACCATGGCAAGCTTTTGGAATTGAATTCATCCTGACCTTTCTAGTT
GTCTTCACAGTGTTTGCT---ACGCTGGATCCTAGCAGA---------------------
---AGATCTTTG---------------GGAAGTGATGCACTGGCTGTGGGTATTGGCTAT
CTTGTTACTTCACTGTCTGGTATACCAGCCACTGGAGCATCATTCAATCCAGCACGGTCG
CTTGGCCCGGCTTTCGTAATGAAC---------AAGTGGCTCCACCACTGGGTGTACTGG
TTCGGTCCTCTGGCGGGATCCGTTGTTGCAGGCCTAATCTATGAATACATCTTCGATACT
---AAAAAGACTGCCAAAACG---------TTGAAAAAAGCACTA---GACGAAATC---
------------------------GACAGA---------GAATCG------AATCTAGAT
GAA------GAC---GACTACGAC---GATCCA---GAGAGATTGCAGATGACCAAAGCC
AGA------------------------------------------AGTCCACAGACGTCT
GAAGAGTTC---------CGA---------------------------------------
------ACTCAGTGTTATGACCAA------------AACTTGAGGCTT---CCCCTTAAT
------------------------------------------------------------
------------------------------------------------------------
------------------------------------------------------------
```

------------ACCTAC---------------------------TCTACTACCCCTTCT
ACT---TAC------------------------------------CCAGCTTCC------

--ACAGAA
TCCACCTTTAGA--ACGCAC
AGCGCC---CCCACG---
------------------TCCAGCTATGGA------------------------------

---------------GAAATCAGT------GGG---------------------------

---------------------------CAACGG---------------------------
---TTTGGAGGTGGTTTT--

------------AAGGGTGTACCA------------------------------------
--------------------------------------ACCTCT----------------
-----ATGGAATATGAC------ACTGGGTCTGCA-------------------------

---AAGTTC-
>RXZF01087924_Tachypleus_tridentatus_Bib3
ATGGCA--
---ACCGTGCCG
CTGGAAACGTTTTTCCTTCACTTGCTTAGTAAGGTGGAAAATATTCAGTCGCAG------

ATG---AACAAGCACCAGAGTATCCAGGAAGAAATTAGAACACTG---GAGTTTTGGAGA
TCAATTATTGCAGAATGTCTGGCTAGTTTCTTTTATGTTTTCCTTCTGTGTGGCGCTCAC
GTAACCTGGCCTGGTCATGAACCCAGT---------------------------------
---AAC
TTAATGATTAAGGCCTTGACCAGTGGTCTAACCATGGCTACTCTTGCTCAGAGTTTCAGT
CACATTTCTGGTGCTCACGTGAACCCCGTGGTGACCTTTAGCATGTTCTTGACCAGAAAA
GTATCCTCACTCAGGGCTAGTATGTACGTAATCGCTCAGTGCGGTGGATCAATTGCTGGA

```
GCGGCCCTCCTGTACGGGGTAACAATTCCTGGACATCAGGGG------------------
------------------------------ATATTGGGCGCCACTTTGGTTCATCCTGAT
---------CTGGGACCATGGCAAGCTTTTGGAATTGAATTCATCCTGACCTTTCTAGTG
GTCTTCACAGTGTTCGCT---ACGCTGGATCCTAGCAGA---------------------
---AGATCTTTG---------------GGAAGTGATGCACTGGCTGTGGGTATTGGCTAT
CTGGTTACTTCACTGTCTGGAATACCAGCCACTGGAGCATCGTTCAATCCAGCACGGTCG
CTTGGCCCGGCTTTCGTAATGAAC---------AAGTGGCTCCACCACTGGGTATACTGG
TTCGGTCCTCTGGCGGGATCCGTTGTTGCAGGCCTAATCTATGAATACATCTTCGATACT
---AAAAAGACTGCCAAAACG---------TTGAAAAAAGCACTA---GACGAAATC---
------------------------GACAGA---------GAATCG------AATCTAGAT
GAA------GAC---GACTACGAC---GATCTA---GAAAGATTGCAGATGACCAAAGCC
AGA------------------------------------------AGTCCACAGACGTCT
GAAGAGTTC---------CGA---------------------------------------
------GCTCAGTGTTATGACCAA------------AACTTGAGGCTT---CCCCCTAAT
------------------------------------------------------------
------------------------------------------------------------
------------------------------------------------------------
------------ACCTAC---------------------------TCTACCACCCCTTCT
ACT---TAC------------------------------------CCAGCTTCC------
------------------------------------------------------------
------------------------------------------------------------
------------------------------------------------------------
------------------------------------------------------------
------------------------------------------------------------
------------------------------------------------------------
------------------------------------------------------ACAGAA
TCCACCTTTAGA------------------------------------------ACGCAC
AGCGCT---CCCACG---------------------------------------------
------------------TCCAGCTATGGA------------------------------
------------------------------------------------------------
---------------GAAATCAGT------GGG---------------------------
------------------------------------------------------------
------------------------------------------------------------
------------------------------------------------------------
------------------------------------------------------------
---------------------------CCACGG---------------------------
---TTTGGAGGTGGTTTT------------------------------------------
------------------------------------------------------------
------------------------------------------------------------
------------------------------------------------------------
------------------------------------------------------------
------------------------------------------------------------
------------AAGGGTGTGCCA------------------------------------
--------------------------------------ACCTGT----------------
-----ATGGAATATGAC------ACTGGGTCCGCA-------------------------
------------------------------------------------------------
------------------------------------------------------------
------------------------------------------------------------
```


---AAGTTC-
>WCHO01030093_Carcinoscorpius_rotundicauda_Bib3
ATGGCA--
---ACCGTGCCG
CTGGAAACGTTTTTCCTTCACTTGCTTAGTAAGGTGGAAAATATTCAGTCGCAG------

ATG---AACAAGCACCAGAGTATCCAGGAAGAAATTAGGACACTG---GAGTTTTGGAGA
TCAATTATTGCGGAATGTCTGGCTAGTTTCTTTTATGTTTTCCTTCTGTGTGGCGCTCAC
GTGACATGGCCTGGTCATGAACCCAGT---------------------------------
---AAT
TTAATGATTAAGGCCTTGACCAGTGGTCTAACCATGGCTACTCTTGTTCAAAGTTTCAGT
CACATTTCTGGTGCTCACGTGAACCCCGTGGTGACCTTTAGCATGTTCTTGACCAGAAAA
GTATCGTCACTCAGGGCTAGTATGTACGTGATAGCTCAGTGCGGTGGATCAATTGCTGGA
GCGGCCCTCCTGTACGGGGTAACAATTCCTGGACATCAGGGG------------------
------------------------------ATATTGGGCGCCACCTTGGTTCATCCTGAT
---------CTGGGACCATGGCAAGCTTTTGGAATTGAATTCATCCTGACCTTTCTAGTG
GTCTTCACAGTGTTCGCT---ACGCTGGATCCTCGCAGA---------------------
---AGATCTTTG---------------GGAAGTGATGCACTAGCTGTGGGTATTGGCTAT
CTGGTTACTTCACTGTCTGGGATACCAGCTACTGGAGGATCGTTCAATCCAGCACGGTCG
CTTGGCCCGGCTTTCGTAATGAAC---------AAGTGGCTCCACCACTGGGTATACTGG
TTCGGCCCTCTGGCAGGTTCCGTTGTTGCAGGCCTAATCTATGAATACATCTTCGATACT
---AAAAAGACTGCCAGAACG---------TTGAAAAAAGCACTA---GACGAAATC---
------------------------GACAGA---------GAATCA------AATCTAGAT
GAA------GAC---GACTACGAC---GATCTA---GAAAGATTGCAGATGACCAAAGCC
AGA--AGTCCACAGACGTCT
GAAGAGTTC---------CGA---------------------------------------
------GCTCAGTGTTATGACCCA------------AACTTGAGGCTT---CCCCCTAAT

------------ACCTAC---------------------------TCTACCACCCCTTCT
ACT---TAC------------------------------------CCAGCTTCC------

--ACAGAA
TCCACCTTTAGA--ACGCAC
AGCGCC---CCCACG---
------------------TCCAGCTATGGA------------------------------

---------------GAAATCAGT------GGG---------------------------

```
------------------------------------------------------------
------------------------------------------------------------
------------------------------------------------------------
------------------------------------------------------------
---------------------------TCACGG---------------------------
---TTTGGAGGTGGTTTT------------------------------------------
------------------------------------------------------------
------------------------------------------------------------
------------------------------------------------------------
------------------------------------------------------------
------------------------------------------------------------
------------AAGGGTGTGCCA------------------------------------
--------------------------------------GCCTGT----------------
-----ATGGAACATGAC------ACAGGATCCGCA-------------------------
------------------------------------------------------------
------------------------------------------------------------
------------------------------------------------------------
------------------------------------------------------------
------------------------------------------------------------
------------------------------------------------------------
-----------------------------------------------AAGTTC-
>AZTN01150950_XR_002613989_Limulus_polyphemus_Bib3
ATGGCA------------------------------------------------------
---------------------------------------------------ACCGTTTCG
CTGGAAACGTTTTTTCTGCACTTGCTTAACAGGGTGGAAACTATTCAGTCACAG------
------------------------------------------------------------
------------------------------------------------------------
ATG---AACAAGCACCATAGTATCCGGGAAGAAATTAGGACACTT---GAATTTTGGAGG
TCAATTATTGCAGAATGTTTAGCTAGTTTCTTTTATGTTTTTCTTCTGTGTGGCGCTCAC
GTGACCTGGCCTGGTCACGATCCCAGT---------------------------------
---------------------------------------------------------AAC
TTGATGATTAAGGCCTTGACCAGTGGTCTAACCATGGCTACTCTTGCTCAGAGTTTTAGT
CACATTTCCGGTGCTCACGTGAACCCCGTGGTGACCTTTAGCATGTTCTTGACCAGAAGA
GTATCATCACTCAGGGCTAGTATGTACGTAATAGCTCAGTGCGGCGGCTCAATCGCTGGG
GCGGCCCTCCTATATGGG------------------------------------------
------------------------------------------------------------
------------------------------------------------------------
------------------------------------------------------------
------------------------------------------------------------
------------------------------------------------------------
------------------------------------------------------TACTGG
TTCGGCCCTCTGGCGGGAGCCGTTGTGGCAGGCCTAATCTACGAGTATATCTTCGATACT
---AAGAAGACTGCCAAAACG---------TTGAAAAAAGCGCTA---AACGAAATA---
------------------------GACAGA---------GAGTCG------AACCTCGAC
GAA------GAC---GATTATGAA---GATCCT---GAAAGGTGCCAGATGACCAAAGCC
AGA------------------------------------------GGTCCACAGGCGTCC
GAAGAGTTT---------CAG---------------------------------------
------GCTCAGTGTTACGACCAA------------AACTTGAGGCCC------------
```


--AGCACCCCTTCT
ACT---TAC------------------------------------CCAGCTTCC------

--ACAGAA
TCCACCTACAGA--GCGCAC
AGCGCC---CCCACG---
------------------TCCAGCTACGGA------------------------------

---------------GAAATCAGT------GGG---------------------------

---------------------------CCACGG---------------------------
---TTTGGAGGTGGCTTT--

------------AGGGGTGTGCCA------------------------------------
--------------------------------------ACCCGT----------------
-----ATGGAATATGAC------GCTGGGTCCGCG-------------------------

---AAGTTT-
>GILM01003793_Tachypleus_gigas_Bib2
ATGGCA--
---AACGTGTCT
TTGGAGTCGTTCTTTTTACAGCTAGTGAACAAAGTGGAAAATATCCGCTCAAAT------

TTC---AACAAGAAAAAAACTATGAAAGAAGAGGTTCGAACCCTG---GAGTTTTGGCGA
TCCATTATAGCGGAGTGTTTGGCAACTTTTTTTTACGTGTTCTTACTATGTGGAGCCCAC
ATATCATGGCCAAACGTTGAACGCACC---------------------------------
---AAC

```
ATACTGGTTAAATCATTCACCAGTGGCCTGGCTATCGCCACACTCGTCCAGTGCTTCGGC
CACATTTCAGGAGCTCATGTTAACCCAGCAGTGACACTTGCGATGGTCTTAACCCAGAAG
ATTACCCCACTCAGGGCAGTTCTTTACGTAACCTCGCAATGTGGTGGAGCTATAGCTGGC
GCAGCCCTCCTGTACGGTGTAACGATTCCCGGTCACCAGGAG------------------
------------------------------ACATTGGGATGCACCTTGGTTCATGATTAT
---------CTTGGAGCCTGGCAAGCGTTTGGAATCGAATTCGTCCTAACGTTTGTAATT
GTCTTCACTGTGTTTGCA---ACAGTAGACCCAAACAGG---------------------
---AGAATGCTG---------------GGAAGTGATGCTGTTGCAATTGGAATTGCTTAC
CTAACTGTTTCATTAATAGGGCTGCCAGCAAGCGGTGCATCTCTAAACCCAGCGCGTTCT
CTTGGTCCTGCATTTGTGATGAAC---------AAGTGGCACCAACACTGGGTCTACTGG
TTTGGCCCTTTAGCTGGAGGTGTCACTGCTGGATTAATTTATGAGTATATATTCGATTCC
---AAGAAATTAGCCAAAACG---------TTAAAAGAGGCGTTG---GAAGACGTA---
------------------------GACAGA---------GAGTCC------AACACTGAT
---------GAC---GATTACGAT---GATGCA---GAGAGATGCAAAATTGCAAAAGAA
GAT---------------------------------------TGTGGCCCACAAGCCTCG
GGGGACTAC---------AGA---------------------------------------
------CCCCAGTACCAAGATAAC---------------TATAGGTTA---AATCCAAAC
------------------------------------------------------------
------------------------------------------------------------
------------------------------------------------------------
------------ACCTAC---------------------------TCCCCAACCCCCTCA
TCC---TAT------------------------------------CCAGTGTCC------
------------------------------------------------------------
------------------------------------------------------------
------------------------------------------------------------
------------------------------------------------------------
------------------------------------------------------------
------------------------------------------------------------
------------------------------------------------------ACCGAC
TCACCTTACGGA---------------------------------------TACAGTGGA
CCACGC---AGTGCTCCACCGCAA------------------------------------
------------------TCGTGCTCCTCA------------------------------
------------------------------------------------------------
---------------AACCTATAC------GGG---------------------------
------------------------------------------------------------
------------------------------------------------------------
------------------------------------------------------------
------------------------------------------------------------
---------------------------TCTCGC---------------------------
---TATGGAAACGGATAC------------------------------------------
------------------------------------------------------------
------------------------------------------------------------
------------------------------------------------------------
------------------------------------------------------------
------------------------------------------------------------
------------AGGACAATGCCC------------------------------------
--------------------------------------AGTCGC----------------
-----ATGGAATATGGT------CCTGGTTCTGTA-------------------------
```


---AAGTTC-
>GGKL01029363_Tachypleus_tridentatus_Bib2
ATGGCA--
---AACGTGTCT
TTGGAGTCGTTCTTTTTACACCTCGTGAACAAAGTGGAAAATATCCGCTCAAAC------

TTC---AACAAGAAAAAAACTATGAAAGAAGAGGTTCGAACCCTG---GAGTTTTGGCGA
TCCATTATAGCCGAGTGTTTGGCAACTTTCTTTTACGTGTTCTTACTATGTGGAGCCCAC
ATATCATGGCCAAACGTTGAACGCACC---------------------------------
---AAC
ATACTGGTTAAATCATTCACCAGTGGCCTGGCTATCGCCACACTCGTCCAGTGCTTCGGC
CACATTTCAGGAGCTCATGTTAACCCAGCAGTGACACTTGCGATGGTCTTAACCCAGAAG
ATTACCCCACTCAGGGCAGTTCTTTACGTAACCTCGCAATGTGGTGGAGCCATAGCTGGC
GCAGCCCTCCTGTACGGCGTAACGATTCCTGGTCACCAGGAG------------------
------------------------------ACATTGGGATGCACCTTGGTTCATGATTAT
---------CTTGGAGCCTGGCAAGCGTTTGGAATCGAATTCGTCCTAACGTTTGTAATT
GTCTTCACTGTGTTTGCA---ACAGTAGACCCAAACAGG---------------------
---AGAATGCTG---------------GGAAGTGATGCTGTTGCAATTGGAATTGCTTAC
CTAACTGTTTCATTAATAGGGCTGCCAGCAAGCGGTGCATCTCTAAATCCAGCACGTTCT
CTTGGTCCTGCATTTGTGATGAAC---------AAGTGGCACCAACACTGGGTCTACTGG
TTCGGCCCTTTAGCTGGAGGTGTCACTGCCGGATTAATTTATGAGTATATATTCGATTCC
---AAGAAATTAGCCAAAACG---------TTAAAAGAGGCGTTG---GAAGACGTA---
------------------------GACAGA---------GAGTCC------AACACTGAT
---------GAC---GATTACGAT---GATGCA---GAGAGATGCAAAATTGCAAAAGAA
GAT---------------------------------------GGTGGCCCACAAGCCTCG
GGGGACTTC---------AGA---------------------------------------
------CCCCAGTACCAAGATAAC---------------TATAGGTTA---AATCCAAAC

------------ACCTAT---------------------------TCCCCAACCCCCTCA
TCC---TAT------------------------------------CCAGTGTCC------

--ACCGAC
TCACCTTACGGA---------------------------------------TACAGTGGA
CCACGC---AGTGCTCCACCGCAA------------------------------------

```
------------------TCGTGCTCCTCA------------------------------
------------------------------------------------------------
---------------AACCTATAC------GGG---------------------------
------------------------------------------------------------
------------------------------------------------------------
------------------------------------------------------------
------------------------------------------------------------
---------------------------TCTCGC---------------------------
---TATGGAAACGGATAC------------------------------------------
------------------------------------------------------------
------------------------------------------------------------
------------------------------------------------------------
------------------------------------------------------------
------------------------------------------------------------
------------AGGACAATGCCC------------------------------------
--------------------------------------AGTCGC----------------
-----ATGGAATATGGT------CCTGGTTCTGTA-------------------------
------------------------------------------------------------
------------------------------------------------------------
------------------------------------------------------------
------------------------------------------------------------
------------------------------------------------------------
------------------------------------------------------------
-----------------------------------------------AAGTTC-
>GILQ01011214_Carcinoscorpius_rotundicauda_Bib2
ATGGCA------------------------------------------------------
---------------------------------------------------AACGTGTCT
TTGGAGTCGTTCTTTTTACAGCTCGTGAACAAAGTGGAAAATATCCGCTCGAAT------
------------------------------------------------------------
------------------------------------------------------------
TTC---AACAAGAAAAAAACTATGAAAGAAGAGGTTCGAACCCTG---GAGTTTTGGCGA
TCCATTATAGCCGAGTGTTTGGCAACTTTTTTCTACGTGTTCTTACTATGTGGAGCCCAT
ATATCCTGGCCAAACGTTGAACGCACC---------------------------------
---------------------------------------------------------AAC
ATACTGGTTAAATCATTCACCAGTGGCCTGGCTATCGCCACACTCGTCCAGTGCTTCGGC
CACATTTCAGGAGCTCACGTTAACCCAGCAGTGACACTTGCGATGGTCTTAACCCAGAAG
ATTACCCCACTTAGGGCAGTTCTTTACGTAACCTCGCAATGTGGTGGAGCCATAGCTGGC
GCAGCCCTCCTGTACGGTGTAACAATTCCTGGTCACCAGGAG------------------
-------------------------------ACATTGGATGTACCTTGGTTCATGATTAT
---------CTTGGAGCCTGGCAAGCTTTTGGAATCGAATTTGTCCTAACGTTTGTAATT
GTCTTCACTGTGTTTGCA---ACGGTAGACCCAAACAGG---------------------
---AGAATGCTG---------------GGAAGTGATGCTGTTGCAATTGGAATTGCTTAC
CTAACTGTTTCATTAATAGGGCTGCCAGCAAGCGGTGCATCACTAAATCCAGCACGTTCT
CTTGGTCCTGCATTTGTGATGAAC---------AAGTGGCACCAACACTGGGTCTACTGG
TTCGGCCCTTTAGCTGGAGGTGTCACTGCCGGATTAATTTATGAGTATATATTCGATTCC
---AAGAAATTAGCCAAAACG---------TTAAAAGAGGCGTTG---GAAGACGTA---
------------------------GACAGA---------GAGTCC------AACACTGAT
---------GAC---GATTACGAT---GATGCG---GAGAGATGCAAAATTGCAAAAGAA
```

GAT---------------------------------------GGTGGCCCACAAGCCTCG
GGGGACTTC---------GGA---------------------------------------
------CCCCAGTACCAAGATAAC---------------TATAGGTTA---AATCCAAAC

------------ACCTAC---------------------------TCCGCAACCCCCTCA
TCC---TAT------------------------------------CCAGTGTCT------

--ACCGAC
TCACCTTACGCA---------------------------------------TACAGTGGA
CCACGC---AGTGCTCCACCGCAA------------------------------------
------------------TCGTGCTCCTCG------------------------------

---------------AACCTATAC------GGG---------------------------

---------------------------TCTCGC---------------------------
---TATGGAAACGGATAC--

------------AGGACAATGCCT------------------------------------
--------------------------------------AGTCGC----------------
-----ATGGAATATGGT------CCTGGTTCTGTG-------------------------

---AAGTTC-
>XM_022390941_AZTN01035843_01080233_Limulus_polyphemus_Bib2
ATGGCA--
---AACGTGTCG
TTAGAGTCGTTCTTTTTACAGCTCGTGAACAAAGTGGAAAATATCCGCTCTCAT------

TTA---AATAGGAAAAAAACCATGAAAGAAGAGGTTCGAACCCTG---GAGTTTTGGCGA

```
TCCATTATAGCCGAGTGTTTGGCAACATTTTTTTACGTGTTCTTACTCTGTGGAGCCCAC
ATATCATGGCCAAATGTTGAACGCACC---------------------------------
---------------------------------------------------------AAC
ATTCTGGTCAAATCATTCACCAGTGGCTTGACTATCGCCACACTGGTCCAGTGCTTCGGC
CACATATCAGGCGCTCATGTTAACCCAGCAGTGACACTTGCGATGCTCTTAACCCAGAAG
ATCACCCCACTTAGAGCAGTTCTTTACGTAACCTCGCAATGTGGAGGAGCCATAGCTGGC
GCAGCTCTTCTGTACGGTGTAACGATCCCTGGTTACCAGGAG------------------
------------------------------ACATTGGGGTGCACCCTGGTTCACAGTGAT
---------CTTGGAGCCTGGCAAGCATTTGGAATTGAATTCGTTCTAACGTTTGTCATT
GTCTTCACTGTGTTTGCA---ACAATGGACCCAAACAGA---------------------
---AGAACGCTG---------------GGAAGTAATGCTGTTGCCATTGGAATTGCTTAC
CTAACTGCCTCATTAATCGGGCTGCCGGCAAGCGGAGCATCACTAAATCCAGCCCGTTCT
CTTGGTCCTGCATTTGTGATGAAC---------AAGTGGCACCAGCATTGGGTCTACTGG
TTTGGCCCCTTAGCTGGAGGTGTCACTGCTGGATTAATTTATGAATATATATTCGACTCG
---AAGAAATCAGCCAAGACG---------TTAAAAGAGGCGTTG---GAAGATGTA---
------------------------GACAGA---------GAGTCC------AACACTGAT
---------GAC---GATTACGAA---GATCCA---GAGAGATGCAAAATGGCAAAAGAA
GAT---------------------------------------AGTGGCCCCCAAGCGTCG
GGGGACTTC---------AGG---------------------------------------
------CCCCAATACCATGATAAC---------------TATAGGTTA---AATCCAAAC
------------------------------------------------------------
------------------------------------------------------------
------------------------------------------------------------
------------ACCTAC---------------------------TCCTCAACCCCCTCA
TCC---TAT------------------------------------GCAGTATCC------
------------------------------------------------------------
------------------------------------------------------------
------------------------------------------------------------
------------------------------------------------------------
------------------------------------------------------------
------------------------------------------------------------
------------------------------------------------------GCAGAA
TCACCGTACGGA---------------------------------------TACAGCGGG
CCACGA---AGTGCTCCACCGCCA------------------------------------
------------------TCCTGCTCCTCC------------------------------
------------------------------------------------------------
---------------AGCCTATAC------GGA---------------------------
------------------------------------------------------------
------------------------------------------------------------
------------------------------------------------------------
------------------------------------------------------------
---------------------------TCTCGG---------------------------
---TATGGAAACGGATAC------------------------------------------
------------------------------------------------------------
------------------------------------------------------------
------------------------------------------------------------
------------------------------------------------------------
------------------------------------------------------------
```

```
------------AGAACAATGCCT------------------------------------
--------------------------------------AGTCGC----------------
-----ATGGAATATGGT------CCTGGCTCTGTA-------------------------
------------------------------------------------------------
------------------------------------------------------------
------------------------------------------------------------
------------------------------------------------------------
------------------------------------------------------------
------------------------------------------------------------
-----------------------------------------------AAGTTC-
>CM024347_Tachypleus_gigas_Bib1
ATGGCA------------------------------------------------------
---------------------------------------------------AGCGTGACT
CTGGAGTCGTTTTTTCTACAACTTATCAACAAAGTGGAAAATATTCGCGACAAT------
------------------------------------------------------------
------------------------------------------------------------
TTA---AACAGGAAGAAAACGATGAACGAAGAGATTCGAACTCTG---GAATTTTGGCGA
TCTATAATAGCCGAATGTTTGGCTACGTTTTTTTACGTGTTTTTGCTCTGCGGAGCTCAT
GTATCGTGGCCAAATGTTGAACATACC---------------------------------
---------------------------------------------------------AAC
GTTCTGGTCAAGGCATTCACCAGTGGTCTGACTATCGCCACACTGGTCCAGTGTTTCGGC
CACATATCCGGGGCTCACGTTAACCCAGCAGTGACGTTTTCCATGTTCTTAACTCAGAAG
ATCACCCCACTTAGGGCTATACTTTATATAACCTCTCAGTGTGGTGGGGCTATAGCTGGG
GCAGCACTTCTGTATGGGGTAACGATTCCCGGACACCAGGAA------------------
------------------------------ACGTTAGGAAGTACCGTGGTTCACCCTGAT
---------CTTGGAGCCTGGCAAGCATTTGGAATTGAATTTATTTTAACGTTTGTCGTT
GTTTTCACCGTGTTTGCC---ACGGTTGATTCGAGTCGC---------------------
---AGGTCTCTG---------------GGAAGTGATGCTGTGGCAATCGGAATTGCGTAT
CTGGCTGTCTCTTTAATAGGGTTACCAGCAAGTGGAGCGTCATTGAATCCAGCCCGTTCT
CTCGGTCCTGCATTTGTGATGAAC---------AAGTGGCACCGACACTGGGTATATTGG
TTCGGTCCCTTGGCTGGAGGCGTTACTGCTGGATTAATCTACGAATACATATTTGACTCG
---AAGAAACCC---AAATCG---------TTGAAAGAAGTTTTG---GATGAATTG---
------------------------GATAGG---------GTGTCC------AACAAAGAT
---------GAT---GACTATGAT---GACCAG---GAGAGAGACAAAATGACAAAAGAG
GAT---------------------------------------GGTGAACTACAGGCCACA
GGGGGTTTC---------AGA---------------------------------------
------CCCCGGTATCAAGATAAT---------------TATCGGCTA---AATTCAAGC
------------------------------------------------------------
------------------------------------------------------------
------------------------------------------------------------
------------ACGTAT---------------------------TCCCCTACCGTTTCG
ACC---TAC------------------------------------CCAGCATCT------
------------------------------------------------------------
------------------------------------------------------------
------------------------------------------------------------
------------------------------------------------------------
------------------------------------------------------------
------------------------------------------------------------
```

--TCAGAA
ACACCTTACGGA---------------------------------------TACAGCGGC
GCTCGA---AGTACTCCATTACCA------------------------------------
------------------TCCTGTTCTTCT------------------------------

---------------GGTATATAC------GAC---------------------------

---------------------------TCTCGG---------------------------
---TATGGAAACGGTTAT--

------------AGGACAATGCCC------------------------------------
--------------------------------------AGTCGT----------------
-----TTGGAGTATGGC------CCCGGATCTCTT-------------------------

---AAATTT-
>RQPD01000008_Tachypleus_tridentatus_Bib1
ATGGCG--
---AGTGTGACT
CTGGAGTCGTTTTTTCTACAACTTATCAACAAAGTGGAAAATATTCACGACAAT------

TTA---AATAGGAAGAAAACGATGAACGAAGAGATTCGAACTCTG---GAATTTTGGCGA
TCTATAATAGCCGAATGTTTGGCTACGTTTTTTTACGTGTTTTTGCTCTGCGGAGCCCAT
GTATCGTGGCCAAATGTTGAACATACC---------------------------------
---AAC
GTTCTGGTCAAGGCATTCACCAGTGGTCTGACTATCGCCACACTGGTCCAGTGCTTCGGC
CACATATCCGGGGCTCACGTTAACCCAGCAGTGACGTTTTCCATGTTCTTAACTCAGAAG
ATCACCCCGCTTAGGGCTATACTTTATATAACCTCTCAGTGTGGTGGGGCTATAGCTGGG
GCAGCACTTCTGTATGGGGTAACGATTCCCGGCCACCAGGAA------------------
------------------------------ACGTTAGGAAGTACCGTGGTTCACCCTGAT
---------CTTGGAGCCTGGCAAGCATTTGGAATTGAATTCATTTTAACGTTTGTAGTT
GTTTTCACCGTGTTTGCC---ACGGTTGATTCGAGTCGC---------------------
---AGGTCTCTG---------------GGAAGTGATGCTGTAGCAATCGGAATTGCGTAT
CTGGCTGTCTCTTTAATAGGGTTGCCAGCAAGTGGAGCGTCGTTAAATCCAGCCCGTTCT
CTCGGTCCTGCATTTGTGATGAAC---------AAGTGGCACCGACACTGGGTATACTGG
TTCGGTCCCTTGGCTGGAGGCGTCACAGCTGGATTAATCTACGAATACATATTTGACTCG

```
---AAGAAAACC---AAATCG---------TTGAAAGAAGCTTTG---GATGAATTG---
------------------------GATAGG---------GTGTCC------AACAAAGAT
---------GAT---GACTATGAT---GACCAG---GAGAGAGACAAAATGGCAAAAGAG
GAT---------------------------------------GGTGAACTACAGGCCACA
GGGGGTTTC---------AGA---------------------------------------
------CCCCGGTATCAAGATAAT---------------TATCGGCTA---AATTCAAGC
------------------------------------------------------------
------------------------------------------------------------
------------------------------------------------------------
------------ACGTAT---------------------------TCCCCCACCGTTTCG
ACC---TAC------------------------------------CCAGCATCT------
------------------------------------------------------------
------------------------------------------------------------
------------------------------------------------------------
------------------------------------------------------------
------------------------------------------------------------
------------------------------------------------------------
------------------------------------------------------TCAGAA
ACACATTACGGA---------------------------------------TACAGCGGC
GCTCGA---AGTACTCCATTACCA------------------------------------
------------------TCCTGTTCTTCT------------------------------
------------------------------------------------------------
---------------GGTATATAC------GAC---------------------------
------------------------------------------------------------
------------------------------------------------------------
------------------------------------------------------------
------------------------------------------------------------
---------------------------TCTCGG---------------------------
---TATGGAAACGGTTAT------------------------------------------
------------------------------------------------------------
------------------------------------------------------------
------------------------------------------------------------
------------------------------------------------------------
------------------------------------------------------------
------------AGGACAATGCCC------------------------------------
--------------------------------------AGTCGT----------------
-----TTGGAGTATGGC------CCCGGATCTCTT-------------------------
------------------------------------------------------------
------------------------------------------------------------
------------------------------------------------------------
------------------------------------------------------------
------------------------------------------------------------
------------------------------------------------------------
-----------------------------------------------AAATTT-
>CM022398_Carcinoscorpius_rotundicauda_Bib1
ATGGCG------------------------------------------------------
---------------------------------------------------AGCGTGACA
CTGGAGTCGTTTTTTCTACAACTTATGAACAAAGTGGAAAATATTCACGACAAT------
```


TTA---AACAGGAAGAAAACGATGAACGAAGAGATTCGAACTCTG---GAATTTTGGCGA
TCTATAATAGCCGAATGTTTAGCTACATTTTTTTACGTGTTTTTGCTCTGCGGAGTCCAT
GTATCGTGGCCAAATGTTGAACATACC---------------------------------
---AAC
GTTCTGGTCAAGGCATTCACCAGTGGTCTGACTATCGCCACACTGGTCCAGTGCTTCGGC
CACATATCCGGGGCTCACGTTAACCCAGCAGTGACGTTTTCCATGTTCTTAACTCAGAAG
ATCACCCCACTTAGAGCTATACTTTATATAACCTCTCAGTGTGGTGGGGCTATAGCTGGG
GCAGCACTTCTGTATGGGGTAACGATTCCCGGCCACCAGGAA------------------
------------------------------ACGTTAGGAAGTACCATGGTTCACCCGGAT
---------CTTGGAGCCTGGCAAGCATTTGGAATTGAATTCATTTTAACGTTTGTCGTT
GTTTTCACCGTGTTTGCC---ACGGTTGATTCGAGTCGC---------------------
---AGGTCTCTG---------------GGAAGTGATGCTGTGGCAATCGGAATTGCGTAT
CTGGCTGTCTCTCTAATAGGGTTGCCAGCAAGTGGAGCGTCGTTAAATCCAGCCCGTTCT
CTGGGTCCTGCATTTGTGATGAAC---------AAGTGGCACCGACACTGGGTGTACTGG
TTCGGTCCCTTGGCTGGAGGCGTCACAGCTGGATTAATCTACGAATATATATTTGACTCG
---AAGAAAACC---AAATCG----------TTGAAAGAAGCTTTG---GATGAATTG---
------------------------GATAGG---------GTGTCC------AACAAAGAT
---------GAT---GACTATGAT---GACCAG---GAGAGAGACAAAATGGCAAAAGAG
GAT---------------------------------------GGTGAACTGCAGGCCACA
GGGGGTTTC---------AGA---------------------------------------
------CCCCGATATCAAGATAAT---------------TATCGGCTA---AATTCAAGC

------------ACGTAT---------------------------TCCCCTACCGTTTCG
ACT---TAC------------------------------------CCAGCATCT------

--TCAGAA
ACACCTTACGGA---------------------------------------TACAGCGGC
GCTCGA---AGTACTCCATTACCA------------------------------------
------------------TCCTGTTCTTCT------------------------------

---------------GGTATATAC------GAC---------------------------

---------------------------TCTCGG---------------------------
---TATGGAAACAGTTAT--


```
------------------------------------------------------------
------------------------------------------------------------
------------------------------------------------------------
------------AGGACAATGCCC------------------------------------
--------------------------------------AGTCGT----------------
-----TTGGAGTATGGC------CCCGGATCTCTT-------------------------
------------------------------------------------------------
------------------------------------------------------------
------------------------------------------------------------
------------------------------------------------------------
------------------------------------------------------------
------------------------------------------------------------
-----------------------------------------------AAATTT-
>XP_013784973_Limulus_polyphemus_Bib1
ATGGCA------------------------------------------------------
---------------------------------------------------AGCGTGACT
CTAGAGTCATTTTTTCTACAACTTGTCAACAAAGTGGAAAATATTCGCGATAAT------
------------------------------------------------------------
------------------------------------------------------------
TTA---AACAGGAAGAAAACGATGAAAGAAGAGATTCGAACCCTG---GAATTTTGGCGA
TCTATAATAGCTGAATGTTTGGCTACGTTTTTTTACGTGTTTTTGCTCTGCGGAGCCCAC
ATATCATGGCCAAACGTTGAACATACC---------------------------------
---------------------------------------------------------AAC
ATTCTGGTCAAGGCATTCACCAATGGTCTGACTATCGCCACACTGGTCCAGTGCTTCGGT
CACATATCAGGCGCTCACATTAACCCAGCAGTGACATTTGCGATGTTCTTAACCCAGAAG
ATCACCCCACTTCGGGCTATACTTTATGTAACCTCTCAGTGTGGTGGGGCTATAGCTGGG
GCAGCACTTCTGTATGGTGTAACGATTCCTGGCCACCAGGAA------------------
------------------------------ACGCTAGGAATCACCGTGATTCACCCTGAT
---------CTTGGAGCCTGGCAAGCATTCGGAATTGAATTCGTTTTAACGTTCGTCATT
GTTTTCACCGTGTTTGCC---ACAGTTGATTCGAGCCGT---------------------
---AGATCTTTG---------------GGAAGTGATGCTGTTGCCATAGGAATGGCGTAT
CTAGCTGTCTCACTAATAGGGTTGCCAGCAAGTGGAGCATCATTAAATCCAGCCCGTTCT
CTCGGTCCTGCGTTTGTGATGAAC---------AAGTGGCAACGACACTGGGTGTACTGG
TTCGGTCCCTTAGCTGGAGGTGTCACCGCTGGATTAATCTACGAATATATATTTGACTCG
---AAGAAAGCC---AGGTCG---------TTGAAAGAGGCTTTG---GATGACTTG---
------------------------GATAGG---------GTGTCC------AACACTGAT
---------GAT---GATTACGAG---GATCAA---GATAGATGCAAAATGGCAAAAGAT
GAT---------------------------------------GATGAACTACAGGCTACG
GGGGGTTTC---------AGG---------------------------------------
------CCCCAGTATCACGATAAT---------------TATCGGTTA---AATTCAAAC
------------------------------------------------------------
------------------------------------------------------------
------------------------------------------------------------
------------GCGTAC---------------------------TCTCCCACCGTTTCG
ACC---TAT------------------------------------CCAGCACCT------
------------------------------------------------------------
------------------------------------------------------------
------------------------------------------------------------
```


--TCAGAA
CCACCTTACGGA---------------------------------------TACGGCGGT
CCTCGA---AGTACTCCATTACCA------------------------------------
------------------TCCTGCTCCTCT------------------------------

---------------GGAGTATAC------GAC---------------------------

---------------------------TCTCGA---------------------------
---TATGGAAACGGTTTT--

------------AGGACAATACCC------------------------------------
--------------------------------------AGTCGT----------------
-----ATGGAGTATGGT------CCCGGATCTGTG-------------------------

---AAATTC-
>KX655543_Blomia_tropicalis_Bib
ATGATGGCAACA--
------------CCAAAATTG------------------------GGTCCGACCTCATCC
ACTGAATCACTTCTATTACATCTTTTATGTCGAGTCGATGCGGCTCGGCATCGTACATTA
GTT---

CGTCAACGAAAACGTCATTCGATGAGTACAGAATTTCGGACATTA---GACTTTTGGCGT
GCCATCATTGCTGAATGTTTAGCATCGTTTTTGTACGTTTTCCTATTGTGTGCCACCCAT
CTTACCTGGAATGGTTCGATGTTYATC---------------------------------
--TTTGGTCCGACGCCCAGT
TGGCTAGTGGTGGCATTGACCAGTGGTTTTGCCATGGCCACCTTAGTACAATGTTTCGGA
CACATTAGCGGTGGACACGTGAATCCAGCGCTCACGTGCGCCTTGTTAGTGACCAAGAAG
ATCAGTCCACTTCGAGCACTATTTATTTGATCGCCCATTGTAGCGGTGCAATCGCCGGA
GCAGCATTACTCTATGGAGTGACTGTTACCGGGAGTCAAAGT------------------
------------------------ATGGGACCATTGGGAATTACCATTGGACACGATAGT
---------CTCAACTCGTGGCAAGTGTTGACCGTTGAATTTATGCTCACATTCGTCGTC
ATCTTTACCACATTTGCA---ACCTATGATTCGAATCGA---------------------
---CGATCATTC---------------GGCAGCGATTCACTTTCAATTGGAGCAGCCTAT

TTGGTCGCATCGTTGACGGGGTTACCAGCGAGTGGTGCCTCGATGAACCCAGCGCGATCG
CTTGGGCCAGCGTTTGTGATGAAT---------CGATGGCAATATCATTGGGTTTATTGG
ATCGGACCATTGACTGGCGCCATCGTTGCCGCCCTAATCTATGAATTTATATTCGATACG
---AAAAAGGTTGGAACATTC---------GTTATGGACACATTC---GACGACATC---

>XP_015789281_Tetranychus_urticae_Bib

```
ATGGTTCCT---------------------------------------------------
------------CAACGAGAA------------------------TTTGGAACCTTATCT
AGTGAATCATTTTTAATCTCGTTAGTTGAAAGGATTGATAAACTTTTACTCCATGAT---
------------------------------------------------------------
------------------------------------------------------------
GGC---TTTCGAACCAAGAAAATGTCTCAAGAAGTTAAATCATTG---GAATTTTGGAGA
AGCTTAATTGCAGAATGTTTATCAACCTTTTTCTATGTGTTTCTTGTTTGTGCAGTCAAC
ATTTCATGGACAGGCTCATTGATTGGC---------------------------------
------------------------------------------------CATACACCCAAT
TGGATTGTAATGTCACTCACAAGTGGCCTAGCCATGGCCATACTTACCCAGTGTTTTGGT
CACATTTCGGGTTCTCACGTTAACCCAGCAGTCACTTGTGCTTTCCTTGTAACCCGTAAT
ATAACACCATTGAGGTCAATACTTTACATTATCGCTCAGTCAGGAGGAGCCATTGCTGGT
GCAGCTCTGCTTTATGGTGTTTCGATTCCTGGACATCAAGGA------------------
------------------------------GCACTTGGCATTGCACTGGCCCATGAAGCT
---------CTAGGAGCTGGTCAAATATTTGGAGTCGAATTTGTTCTCACCTTTCTCATT
GTGTTTACCATTTTCGCC---ACTTTGGACATTAACCGT---------------------
---AAATCAATG---------------GGCAACGATTCAATTACCATTGGCTCAGCTTAC
ATGGTTTGCTCATTGACTGGGTTGCCTGCAAGTGGAGCCTCTTTCAATCCTGCTAGAGCT
CTGGGACCAGCATTTGTTATGAAC---------AAGTGGAAACATCATTGGGTCTACTGG
ATTGCACCGATAACAGGCTCAATGCTGGCTGGATTAATATATGAATTTATATTTGACTCA
---AGAAAGAGC---AAAACT---------CTGCGAGATGTCATC---GATGACATT---
------------------------GACAAA---------GATTGC---------AATGAC
---------GAC---CATTATGAT---GAGCCC---GAAGTCAAGATGACCAATAAAACC
ATCATGGGTTTC------------------------------------------------
------------------------------------------------------------
---------------------------------------------CCTTTGAGTGCAAGT
------------------TCCCATTCAAATTCC------------------GGAAGCAGT
------------------------------------------------------------
------------------------------------------------------------
---TCAAGTTCAACTCATCAATCAAATACA---------------ACTGGT---TACTCA
AGC---ATAAAT---------------------------AGCAATCCTCAT---------
------------------------------------------------------------
------------------------------------------CACCATACTTTGACCCAT
CATCAACACCATCCAGGACTACCTCAATACCCA---------------------------
------------------------------------------------------------
------------------------------------------------------------
------------------------------------------------------------
------------------------------------------------------------
---------------------------------------------------ACCTCGGGT
CTAAAC---AGTTCACCAGGAAACAGC---------------------------------
------------------TTTCAACACACCGTGTTGACT---------------------
------------------------------------------------------------
---------CATGACGGTCTCTAC------GCCAATCAAACTGTAACCCTTCATCCTCAC
AGCAACCTAACGAGTGTCATCACAGGCACAAACAATATCAATAATAATAATCAT------
------------------------------------------------------------
------------------------------------------------------------
---AATAGCAATAGTAATAATAATAAT---AATAATAGTAATATAAATACCTCAACC---
---------------AAT---TCAAATAATCAT---------------------------
```

---AGCACTAATAACCATGTGACT------------------------CCATTG------

---TTGTCATCTTCA------TCTTCCTCTTCT---------------------------
TCACCATCAGCAGCCTCTGCGCCGTCCAATTAT---------------------------

------------CATCAATTCAACGCATCTTATGCTGGAATT------------------
--------------TATGGAACTCGAAATTCACATTTATCAAAC----------------
--AGAATGGATTACAATAATGTACCAAATAATATCAGTCAACCT----------------

--GGAAAATAT-
>GCAC01004291_Panonychus_ulmi_Bib
ATGATGGTTTTA--
------------CCGCGAACC---------------AACAACAATCAAATCAATTTATCA
ACTGAATCATTTTTGATTGGATTAGTTGAGAGAATTGATAAATTGTTACTCAATGAT---

GGT---TATCGAAATCAATCAATGTCCCAGGAAGTTAAATCACTT---GAATTTTGGCGA
AGTTTAATCGCCGAGTGTTTATCAACCTTTTTCTATGTGTTTCTTGTATGTGCGGTCAAC
ATTTCCTGGACTGGGTCACTTATCGGT---------------------------------
--CATACACCCAAT
TGGATTGTAATGTCGCTTACAAGTGGCCTGGCAATGGTCATTTTAACCCAATGTTTTAGT
CACATCTCAGGTGCTCATGTTAACCCGGCGGTCACTTGTGCTTATCTGGTCACTCGTAAC
ATCACACCCTTAAGGTCAATATTATATATTATTGCTCAATCGGGTGGAGCCATTGCTGGA
GCTGCTTTACTTTATGGAGTCTCAATTCCTGGTCATCAAGGT------------------
------------------------------GCTCTTGGCATTTCTCTAGCTCATGAATCA
---------CTGAGCGCAGGTCAAATTTTCGGCGTTGAATTTGTCCTTACCTTTCTGATT
GTGTTCACAATTTTCGCA---ACTTTGGACATTAACAGG---------------------
---AAATCCATG---------------GGAAATGATTCAATTACCATAGGATCGGCTTAC
ATGGTCTGTTCATTAGCAGGGTTACCTGCCAGTGGAGCTTCATTCAATCCCGCTCGAGCA
CTTGGACCTGCTTTCGTTATGAAT---------AAATGGCGACATCATTGGGTTTACTGG
ATTGCACCGATAACTGGATCAATGTTGGCTGGTTTAATATATGAATTTATATTTGATTCA
---AGGAAGAGT---AAAAAT---------TTAAGAGATACAATT---GATGATATT---
------------------------GATAAA---------GTTTGC---AATACGAACGAG
---------GAT---CATTATGAT---GAACCT---GAGGTTAAGATGAGCAGTAAAACT
ATTATGGGTTTC--

---CCTTTAAGCGGAAGT
------------------TCACATTCAAATTCC------------------GGAAGT---

AGTTCAAGTTCAACCCATCAATCAAATACA---------------ACAGGC---TATTCA
AGT---ATAAAT---------------------------AGTGCCCATCATCCAGTT---

---------------AAT---------ATTATCAACAACACCAATTCACAATCAGCACAT
CTTCAACATCATCCACCACCACCACCACCACCA---CAA---TTACCACCT---------

---------------------CATCTCCATCTTCAACAACAACAACAA------------
--CAACAACACCAA
AACTCATATAACACCATCAACACCAACACCAACAATAATAAT---------AACCTTGGC
TTAGGT---CATTCACTGGCCAATACT---------------------------------
------------------TTTCAACACACAGTGATAACT---------------------

---------CATGATGGCCTTTAC------GGCAATGGG---------------------

------------AATAATAATAATAAT---AATAATAATCATATAAATACCTCAAGT---
------------------------AATAGTCAT---------------------------
------------------GTGACA------------------------CCATTG------

---TTATCATCATCTTCCGGATCCTCTTCATCT---------------------------
TCATCTTCAACATCAACCTCATCGTCCAATTAT---------------------------

------------CAT------CCA------------------------------------
-----------------CAGTTCAAA------------TCCAAT----------------
--------------AATAAT---CATAACAACAAC-------------------------

>GEYB01107453_Hermannia_gibba_Bib
ATGGCCCCA---
---------------AAGTTG---------------------------GGCTCAGTTTCG
AACGAATCACTTTTCCTCCATTTGATGGGTCGGATCGATGCCACCCGATTACGA------

GCT---AATCGGAAGCGATCCATGTCTGAAGAGATCAGGACTTTG---GAGTTCTGGCGT
GCGATCATAGCCGAGTGTTTGTCATCATTTTTCTATGTTTTTCTATTGTGTGCCACACAT
ATATCTTGGACTGGGTCTCAATTAGGA---------------------------------
--CATCAGCCCAAT
TGGCTGGTCATGTCTCTGGCCAGCGGCTGTGCAATGGCCACTTTGTGCCAATGCTTCGGA
CATATCAGTGGCGCTCATGTCAACCCTGCGGTCACGTGTGCTCTATTCATCACTTCTAAG
ATAACACCACTTCGAGCCATACTTTATGTGATCGCACAGTGTGGCGGATCAATTGCTGGC
GCAGCACTTCTTTATGGTGTATCAGTGAGTGGTCATCAAGGT------------------
------------------------ATGGGCCCTCTGGGCATAACTGTAGCACATGATCAG

```
---------CTCAGTTCATGGCAGATATTCGGAGTGGAGTTCGTATTGACATTCCTTGTG
GTCTTCACTATATTCGCA---ACTCTGGACCCCAATCGT---------------------
---AAATCATTA---------------GGAAGTGATTCTCTATCTATTGGTATCGCATAT
CTTGTTTGTTCACTTACTGGCTACCTGCCAGTGGTGCATCTATGAACCCTGCTCGAACA
TTGGGTCCCTCGTTTGTCATGAAT---------CGATGGAAGCATCATTGGGTTTATTGG
TTGGGCCCACTCAGTGGTGGAGTATTAGCCGGATTAATATACGAATTTATATTTGACACT
---AAAAAAGTGGGTAAAACG---------ATACGCGACGCGTTC---GATGAGGTA---
------------------------GAAAGA---------GAATCA------AATATTGAT
---------GAC---GACTACGAA---GATCCT---GAACTTAAAATCAGTAATAAGGCT
GTGGTCGCATACAGC------------------------ACAACATCAGGTACTTCATCA
GGCAGTAGTAGTGGTCATCGGTCTAATCAAACCCAACACAGCAATGTCAGTGTTCACGCC
AATAACGCTGGACATTACGAT------------------TACAGAGTGGCTACGGTTAAC
------------------GCATACACTTCGTCA------------------CCGGCCATG
------------------------------------------------------------
------------------------------------------------------------
GATGGGGTCGGAGTGTATGGAACCAGT------------------GGTGGAACTCCACAT
CAC---GTCAAT------------------------------CTCCCGTCCAATAACTTC
------------------------------------------------------------
------------------------------------------------------------
------------------------------------------------------------
------------------------------------------------------------
------------------------------------------------------------
------------------------------------------------------------
------------------------------------------------CATCACAATAAC
TCTAATTATTCC------------------------------------------------
------------------------------------------------------------
------------------------------------------------------------
------------------------------------------------------------
---------------GGGGTTTAT------GGT---------------------------
------------------------------------------------------------
------------------------------------------------------------
------------------------------------------------------------
------------------------------------------------------------
---------------------------TCACGC---------------------------
---TATGGACACAACACTCTC---------------------------------------
------------------------------------------------------------
------------------------------------------------------------
------------------------------------------------------------
------------------------------------------------------------
------------------------------------------------------------
------------AGACAATTGCCA------------------------------------
--------------------------------------TCGAGA----------------
-----ATGGATTACAACGCT---CCGGGAAATACC-------------------------
------------------------------------------------------------
------------------------------------------------------------
------------------------------------------------------------
------------------------------------------------------------
------------------------------------------------------------
```

---AAATTT-
>GEYJ01078472_Nothrus_palustris_Bib
ATGACCCCA---
---------------AAGCTG--------------------------GGCTTAGTCTCG
AACGAGTCATTCTTCCTGCATCTGATGGGTCGCATCGATGCCACCCGATTAAGG------

GCT---AATCGCAAGCGATCAATGGCTGTGGAGATTAGGACTTTG---GAGTTTTGGAGA
GCGATCATAGCCGAGTGTTTGTCTTCATTTTTTATGTATTTCTATTATGTGCGACACAT
ATATCGTGGTCGGGATCACAGTTAGCA---------------------------------
--CATCAGCCCAAT
TGGCTGGTCATGTCTTTGACGAGTGGCTGTGCAATGGCCACACTTTCTCAATGCTTCGGA
CATATCAGTGGCGCTCACGTGAATCCAGCGGTTACGTGCGCTCTCTTTATTACATCTAAG
ATAACACCGCTTCGAGCACTGCTTTATGTGATCGCACAGTGTGGCGGATCAATTGCTGGC
GCAGCACTTCTTTATGGAGTATCAGTGACTGGCCATCAAGGT------------------
------------------------ATGGGTCCTCTGGGCATCTCTGTAGCACATGACCAG
---------CTCAGCTCATGGCAGATATTCGGCGTCGAATTTGTCTTGACTTTCCTGGTC
GTCTTCACCGTATTTGCA---ACTCTCGACCCAAACCGT---------------------
---AAATCATTG---------------GGTAGTGATTCGCTCTCCATTGGAATCGCATAT
CTTGTTTGCTCTCTAACTGGGTTACCTGCAAGTGGTGCAAGTTTCAATCCGGCGCGTTCT
TTAGGTCCCAGTTTTGTAATGAAT---------CGATGGAAGAATCATTGGGTTTATTGG
TTGGGCCCTTTGAGTGGTGGAGTGTTGGCCGGATTAATATACGAGTTTATATTTGACACC
---AAGAAAACTGGTAAAACC---------ATACGCGACGCCTTT---GATGAGTTA---
------------------------GAAAGA---------GAATCA------AATATAGAT
---------GAC---GACTACGAA---GATCCT---GATATCAAAATCAGTAATAAAGCA
AAGGTAGCTTATAGT------------------------ACAACATCGGGCACCTCTACT
GGCAGTAGTAGTGGTCATCGATCCAATCACACTCAACACAGCAATGTCAGCGTCCAC---
------GGCGGCCATTATGACAAC---------------TATAGGGTGGCCACAATCAAC
------------------GCATACACTTCAACA------------------CCTGCTATG

GATGGGGTGGGTTTGGTGGGG------------------------ACGGGAACACCACAT
CAT---GTAAAC------------------------------ATACCGCCCAATAACTAT

--CATCACAATAAC
ACCAATTATGCT--

---------------GGAGTTTAT------GGC---------------------------

---------------------------TCCAGA---------------------------
---TATGGACAAAACACGTTA---------------------------------------

------------AGACAATTGCCG------------------------------------
--------------------------------------TCGCGA----------------
-----ATGGATTACAGC------CCAGTAAATACC-------------------------

---AAATTC-
>GJDQ01005150_Ornithodoros_brasiliensis_Bib
ATGGCAGTG---
--GCAACT
TTGGAGACGCTGTTCCTGCAGCTTCTCACGCAGCTGGAACGCACATCGCGA---------

GCG------GAGCGACGTTCGATGCGCGATGAGATTCGAACCCTG---GAGCTGTGGCGT
GCAGTGATCGGCGAGTGTTTGGCGACGTTCTTTTACGTGTTTCTAGTGTGTGGTGCCCAC
GTGTCGTGGCCTGGCTATGCCGAG------------------------------------
--CCCTCG
GAGCTGGTGATTGCGCTGACCGCGGGCACGGCAGTGGCCACCCTGGTGCAGTGTTACGGC
CACATCTCGGGCTCCCACCTGAATCCCGCTGTTACTCTGGCTATGTTGGCGACGAGGAAA
GTGAGTCCATTGAGGGCGCTCCTCTACATCACTGCCCAATGTGGAGGTGCCATCGCAGGA
GCGGCCTTACTCTACGGCGTGACGGTACCAGGTCACCAGAGC------------------
------------------------------TCTCTGGGCAGCAGTCGACCCCACGAAGCT
---------TTGGGCGCCTGGCAGGCATTCGGCGTGGAGTTTGTGCTCACCTTCCTTGTC
GTCTCGACGGTCTTTGCC---TCCAGGGATCCCAACAGG---------------------
---AATCACCTG---------------GGAGGAGAAGGATTACCCATAGGCTTTGCCTAT
CTTTCATGTACGCTCACTGGGCTTCCTGCCAGTGGAGCGTCCATGAACCCAGCCCGTTCT
TTGGGTCCTGCTTTTGTCATGAAC---------AAATGGACGAATCATTGGGTCTACTGG
TTAGGACCTGTGGCGGGAGGTCTACTTGCAGGCCTCATCTACGAGTACATCTTCGACACC
---AAAAAGAGCACCAAGAGT---------CGGAAACAGGGCCTC---GAAGACACC---
------------------------GATAGA---------GAGTCG------GCGGTGGAC
---------GAC---GACTACGAG---GATCCT---GAGTCAAAGATCACGAAGTACGCG
AGCGTTCCTCAGGGTCCCAACAACGGC---------------ACGTACCGGTCGCAGCAG
CCACTGTACAGC---------------GACACAAGT------------------------
---ACCGGTGGT---TAC---------------------CATCGACCG---AGTGGGAAC
------------------ACCTATTCGCCCACC------------------CCGTCTACG

```
------------------------------------------------------------
------------GCATTTCCT------------------------GTCTCAACGGAGTCT
ACC---TACAAC---------------------------------GGAGCCAACGGGGCT
------------------------------------------------------------
------------------------------------------------------------
------------------------------------------------------------
------------------------------------------------------------
------------------------------------------------------------
------------------------------------------------------------
------------------------------------------------------------
GCGAGTTACTCC------------------------------------------------
------------------------------------------------------------
------------------------------------------------------------
------------------------------------------------------------
------------ACTCCCATTTAC------GATGTGGAAATTCCGTCA---GAACCTTGC
---------CAATGCGCTTGGACTATGGACCGGGACCTGGGTCGACGA------------
------------------------------------------------------------
------------------------------------------------------------
------------------------------------------------------------
------------------------AATTCTAAGCTC------------------------
---TACATC---------------------------------------------------
------------------------------------------------------------
------------------------------------------------------------
------------------------------------------------------------
------------------------------------------------------------
------------------------------------------------------------
------------------------------------------------------------
------------------------------------------------------------
------------------------------------------------------------
------------------------------------------------------------
------------------------------------------------------------
------------------------------------------------------------
------------------------------------------------------------
------------------------------------------------------------
------------------------------------------------------------
------------------------------------------------------
>GGLE01006456_Ornithodoros_turicata_Bib
---GCAGTG---------------------------------------------------
------------------------------------------------------GCAACT
TTGGAAACATTATTTCTGCAGCTTCTAACGCAGCTGGAACGTTCGTCGCGA---------
------------------------------------------------------------
------------------------------------------------------------
GCG------GAACGTCGTTCGATGCGAGATGAGATTCGAACCTTG---GAGCTGTGGCGT
GCGGTGATCGGCGAGTGTTTGGCGACGTTTTTCTACGTGTTTCTGGTGTGTGGTGCCCAC
GTGTCGTGGCCTGGCTATGCCGAG------------------------------------
------------------------------------------------------CCCTCG
GAGCTGGTGATTGCCCTGACCGCGGGCACGGCAGTGGCCACGCTGGTGCAGTGTTACGGT
CACATCTCGGGGGCCCACCTCAATCCTGCTGTCACTCTGGCCATGGTGGCGACGAGGAAA
```

```
GTGAGTCCATTGAGGGCGCTCCTTTACATCACTGCGCAATGTGGCGGTGCCATCGCTGGA
GCGGCCTTACTCTACGGCGTGACGGTGCCAGGTCACCAGAGC------------------
------------------------------TCTCTGGGCAGCAGCCGACCTCACGAAGCT
---------TTGGGCGCCTGGCAAGCGTTCGGCGTCGAGTTCGTGCTCACTTTCCTCCTC
GTCTCGACCGTCTTTGCC---TCCAGGATCCCAACAGG----------------------
---AATCATCTT---------------GGAGGAGAAGGGCTTCCTATAGGATTCGCCTAT
CTTTCCTGCACACTCACTGGTCTTCCAGCCAGTGGTGCCTCGATGAATCCGGCCCGTTCT
TTGGGGCCTGCCTTTGTCATGAAC---------AAATGGATGAACCATTGGGTCTACTGG
TTCGGACCTGTGGCTGGAGGTCTTCTTGCCGGTCTTATTTACGAGTTCATCTTCGATACC
---AAAAAAAGCACGAAGATT---------CGTAAACGAGGTCTC---GAAGACACC---
------------------------GATAGA---------GAATCG------GCAGTGGAC
---------GAT---GACTACGAG---GACCCC---GAGTCAAAGATCACGAAGTACGCG
AGCGTTCCTCAGGGTCCCAATAAC---------------AGCACGTACCGGTCGCAGCAG
CCACAGTAC------------------GACAGCACC------------------------
---AGT---GGC---TAC---------------------CATCGACCAGCTGGAGGCAAC
------------------ACGTATTCGCCCACC------------------CCGTCGACG
------------------------------------------------------------
------------------------------------------------------------
------------GCCTTTCCA------------------------GCCTCTACGGAATCC
ACG---TACGGA---------------------------------GGAGCCAATGCTAGC
------------------------------------------------------------
------------------------------------------------------------
------------------------------------------------------------
------------------------------------------------------------
------------------------------------------------------------
------------------------------------------------------------
------------------------------------------------------------
------TACTCC------------------------------------------------
------------------------------------------------------------
------------------------------------------------------------
------------------------------------------------------------
------------ACTCCCATTTAC------GATGTGGAAATTCCGTCA---GAACCTTGC
---------CGATGCGCTTGGACTATGGACCGGGACCTGGGTCGACGA------------
------------------------------------------------------------
------------------------------------------------------------
------------------------------------------------------------
------------------------AATTCTAAACTG------------------------
---TACATC---------------------------------------------------
------------------------------------------------------------
------------------------------------------------------------
------------------------------------------------------------
------------------------------------------------------------
------------------------------------------------------------
------------------------------------------------------------
------------------------------------------------------------
------------------------------------------------------------
------------------------------------------------------------
------------------------------------------------------------
```


>GFWV01008108_Ornithodoros_erraticus_Bib
ATGGCAGTG---
--GCGACG
TTGGAAACATTGTTTCTGCAGCTCCTGACGCAGCTGGAACGTTCGTCGCGA---------

GCG------GAACGTCGTTCGATGCGAGATGAGATTCGAACCCTA---GAGCTGTGGCGT
GCGGTGATCGGCGAGTGTCTGGCGACGTTTTTCTACGTGTTTCTGGTGTGTGGTGCCCAC
GTGTCGTGGCCTGGCTATGCCGAG------------------------------------
--CCCTCG
GAGCTGGTGATTGCCCTGACCGCGGGCACGGCAGTGGCCACCCTGGTGCAGTGTTACGGC
CACATCTCGGGGGCCCACCTCAACCCTGCCGTTACTCTGGCGACATTAGCGACGAGGAAA
GTGAGCCCTTTGAGGGCGCTCCTTTACATCACGGCGCAATCTGGAGGTGCGATTGCTGGA
GCGGCCTTACTCTATGGCGTGACGGTACCAGGTCACCAGAGC------------------
------------------------------TCTCTGGGCAGCAGCCGGCCTCACGAAGCT
---------TTGGGCGCCTGGCAAGCATTCGGCGTGGAGTTTGTGCTCACCTTCCTTCTC
GTCTCCACCGTCTTTGCC---TCCAGGGATCCCAACAGG---------------------
---AATCACCTC---------------GGAGGGGAAGGACTTCCCATAGGATTTGCCTAC
CTCTCATGTACACTCACTGGTCTTCCAGCCAGTGGTGCCTCCATGAACCCGGCCCGTTCA
CTGGGGCCTGCCTTTGTCATGAAC---------AAATGGATGAACCATTGGGTATACTGG
TTCGGACCTGTGGCTGGAGGTCTGCTTGCCGGTCTAATCTACGAGTACATCTTCGACACC
---AAAAAGAGCACAAAGATT---------CGCAAACGAGGTCTC---GAGGACACC---
------------------------GACAGA---------GAGTCT------GCGGTGGAC
---------GAT---GATTACGAA---GACCCC---GAGGCAAAGATCACGAAGTACGCG
AGCGTTCCTCAGGGTCCAAACAAC------------------ACCTACCGATCGCAGCAG
CCACTCTAC------------------GACACAACA------------------------
---ACCGGTGGC---TAC---------------------CACCGACCGGCG---GCCAAC
------------------ACGTACTCC---------------------------------

>XP_050026463_Dermacentor_andersoni_Bib2
ATGCTG--
---ACC---GCGGCCAGC
CTGGAGTCCCTGCTCCTGCAGATGCTGGACCGGCTGGACTCGCCGTCCCCGGCCGGCGGA
CGCCACCGA---
---GCACGCCAG
CGG------GGCCGCTGCTCGCTGCGCGCCGAGATCCGGACGCTG---GACCTGTGGCGC
GCCGTGGTGGGCGAGTGCCTGGCCACGTTCTTCTACGTGTTCCTCGTGTGCGGCGCGCAC
GTGTCGTGGCCCGGATACGCCGAG------------------------------------
--CCCTCG
GTGCTGGCCATCTCGCTCGCGACCGGAGCCGCCGCGGCGGCGGTGGTGCAGTGCTACGCG
CGCGTCTCCGGCGCCCACATGAACCCGGCCGTCACGCTCGCCGCGTTCGCGACGCGCAAA
GTGAGCCCGCTGCGCGCCCTGCTCTACGTGACGGCCCAGTGCGGCGGGGGCATCGCCGGA
GCGGCGCTCCTCTACGGCGTCACACTTCCGGGCCACCGGAGT------------------
------------------------------TCCCTGGGAAGCAGCCGTCCCCACGAGGCA
---------CTGGGAGCCTGGCAGGCGTTTGGCGTCGAGTTCGTGCTCAGCTTCCTCCTC
GCGTCCACGGTGTTCGCT---ACGCGGACCCTCACCGA----------------------
---TGCCATCTG------TCCACGGGAGGCGCGGACGCCCTTGTCGTCGGGTTCGCCTAC
CTCACCTGCACCCTGGCCGGGCTACCGGCTTCAGGAGCTTCACTGAACCCAGCTCGTTCT
CTCGGTCCTGCCTTTGTCATGAAC---------AAGTGGAAAAATCACTGGGTGTACTGG
TTCGGGCCCGTGGCCGGAGGTCTGTTGGCCGGCCTCATCTACGAGTACATCTTCGACACC
---AAGAGATCAAGCCGGGAC------GTGGGGAGGCGAAGCGTC---GAGGACGCC---
------------------------GACAAA---------GAGTAC------CGGCTGGAG
---------GAC---GACTACGAG---GACCCATCCAGTGCAAAGAACGCAAAGTACGCG
ACCGTCTCGCAGAGCCTAAACGGAGGGGTGGCCTCTGGGAGCACAGCGCGGCACGCTCGC
---GCGGCGAAGGCCGTCCGC---CAGCAGCAGCAACAGCAGCAGCAGCAGTCGCAG---

---CAGCAGCAGCAGTACGAGGCC------GGCTGCTTCGGCCGGCCCGCT---GCGAAC
------------------ACCTACTCGCCCACG------------------CCGTCCCTG

------------GCCTTCCCG------------------------TCATGCACCGAGGCG
GCC---TACGCG--

--AGCACT
CTTGCAGCCGCA--

---------------------AACTGCACT------------------------------

------------GCGCAGATCTAC------GACCTGGAGATGCCTTCC---GGTCACTTT
---------CGGCGCGCCTGGAGTACGGACAAGGACCTCGACCTTCAG------------

------------------------GACTCCAAGTTG------------------------
---TATATA---

>XP_037577743_Dermacentor_silvarum_Bib2
ATGCTG--
---ACC---GCCGCCAGC
CTGGAGTCCCTGCTTCTGCAGATGCTGGACCGGCTGGACTCGCCGTCCCAGCCGGCGGT
CGCCACCGA---
---GGACGCCAA
CGCGGCGGCGGGCGCTGCTCGCTTCGCGCCGAGATCCGGACGCTT---GACCTGTGGCGC
GCCGTGGTGGGCGAGTGCCTGGCCACGTTCTTCTACGTGTTCCTCGTGTGCGGCGCGCAC
GTGTCGTGGCCCGGATACGCCGAG------------------------------------

--CCCTCG
GTGCTGGCCATCTCGCTCGCGACTGGAGCCGCGGCGGCGGTGGTGCAGTGCTACGCG
CGCGTCTCCGGCGCCCACATGAACCCGGCCGTGACGCTCGCGGCGTTCGCGACGCGCAAA
GTGAGCCCGCTGCGCGCCCTGCTCTACGTGACGGCCCAGTGCGGCGGAGGCATCGCGGGA
GCGGCGCTCCTCTATGGCGTCACACTTCCGGACACCGGAGT-------------------
------------------------------TCTCTGGGAAGCAGTCGTCCCCACGAGGCA
---------CTGGGAGCCTGGCAGGCGTTCGGCGTCGAGTTCGTGCTCAGCTTCCTCCTC
GCGTCCACGGTGTTCGCG---ACAAGGACCCCCACCGA----------------------
---TGCCACCTG------TCCACGGGAGGCGCGGACGCCCTGGTCGTCGGGTTCGCCTAC
CTCACCTGCACCCTGGCCGGGCTACCGGCTTCAGGAGCTTCACTGAACCCTGCTCGTTCT
CTCGGTCCTGCATTTGTCATGAAC---------AAATGGAAAAACCATTGGGTGTACTGG
TTCGGGCCCGTGGCCGGAGGCCTGTTGGCCGGTCTCATCTACGAGTATATCTTCGACACG
---AAGAGATCAAGCCGGGAT------GTGGGAAGGCGAAGCGTC---GAAGACGCC---
------------------------GACAAA---------GAGTAC------CGGCTGGAG
---------GAC---GACTACGAG---GACCCATCCAGTGCAAAGAGCGCAAAGTACGCG
ACCGTCTCGCAGAGCCTAAACGGAGGAGTGGCCTCTGGGAGCACAGCACGGCATGCTCGC
---GCAGCGAAGGCCGTCCGC---CAGCAACAACAACAGCAGCAG---------------
---CAGCAGCAGCAGTACGAGGCC------GGCTGCTTCGGCCGGCCCGCC---GCGAAC
------------------ACCTACTCGCCCACG-------------------CCGTCCTG

------------GCCTTCCCG------------------------TCTTCCACCGAGGCG
GCC---TACGCG--

--AGCACG
CTCGCAGCCGCA--

---------------------AACTGCACT------------------------------

------------GCACAGATCTAC------GACCTGGAGATGCCTTCC---GGTCACTTT
---------CGGCGCGCCTGGAGTACAGACAAGGACCTCGACCTTCAG------------

------------------------GACTCCAAGTTG------------------------
---TATATA---

>XP_037578657_Dermacentor_silvarum_Bib1
ATGGCGGCC---
--AACACCGCGGCCGTCAGC
CTGGAGTCGCTGTTCCTCCAGCTGCTGGGCCGGCTGGACACGACGGGCCGGTGCCCGTCT
TCGTCG--
---ACGTCGTCG
TGC------CGTCGTCGCTCGCTCCGCGCCGAGATCCGCAGCCTG---GAGCTGTGGCGC
GCCGTGATCGGCGAGTGCCTGGCCACCTTCTTCTACGTGTTCCTCGTCTGCGGCGCGCAC
GTCTCCTGGCCGGGCTACGCCGAG------------------------------------
--CCCTCG
GTGCTGGGCATCGCCCTGACGGCCGGCGCGGCGGCCGCGACGCTGGTGCAGTGCTACGGT
CACATATCGGGCGCGCACATGAACCCCGCCGTGACGCTGGCCACGTTCGCCACGCGAAAA
GTGAGCCCGCTCCGGACGCTCCTGTACGTGACGGCCCAGTGCGGAGGAGCTATCGCGGGA
GCTGCGCTTCTGTACGGCGTGTCGGTGCCAGGCCACCAGAGC------------------
------------------------------TCTCTGGGCAGCAACCGCCCCCACGAGGCC
---------CTAGGCGCCTGGCAGGCGTTCGGCGTCGAGTTCGTGCTCAGCTTCCTGCTG
GCCTCCACAGTGTTCGCC---ACCAGGGACCCGCACCGC---------------------
---AGCCACCTG---------GGCGCCGGCTCGGACGCCATTGTCATCGGTTTCGCCTAC
TTGGCCTGCACTCTCGCTGGGCTGCCCGCGACGGGAGCTTCAATGAACCCGGCGAGGTCC
TTGGGACCGGCTTTTGTGATGAAC---------AAGTGGGTCGACCACTGGGTGTACTGG
TTCGGACCCATATCCGGAGGCCTGCTGGCTGGTCTCATCTACGAGTACATCTTCGACACC
---AAGAAGCAGGCGCGGGCG---------CTGCGGAAAACACTC---GAGGAAGCT---
------------------------GACAAA---------GAGTCC------AACACGGAC
---------GAC---GACTACGAG---GACCCT---GACGCGAAGATCGCGAAGTACGCG
ACCGTGGCCCAGAGTCCTCCCAACGGGGCG------CCCTCTAGCGCCCACCAGGCGGCG
CCGTTGCAGCAGCTGGTGCAG------CACCAGCAACAAGGCCGGCCACAC---CAC---
---CAGCACCAGCAGTACGAGGCA------GCGGGCTTCGGCCGGCCCGCCGGCGGGAAC
------------------ACGTACTCGCCCACT------------------CCGTCCTCG

------------GCCTTCCCG------------------------GCACCC---------

--GCCGAG
GCGGCGTACGCG--GGCTCG

CTGCGT---CCCACC---
---------------------AATTACGCG------------------------------

------------TCGTCCGTGTAC------GGC---------------------------

------------------------ACCAGCAGA---------------------------
---ATGGGAAATGCCCTT--

------------CGAACACTTCCA------------------------------------
--------------------------------------GCCCGA----------------
-----TTGGAGTACGGACAA---CCTGGATCTCCA-------------------------

---AAATTT-
>XP_050027417_Dermacentor_andersoni_Bib1
ATGGCGGCC---
--AACACCGCGGCCGTCAGC
CTGGAGTCGCTGTTCCTCCAGCTGTTGGGCCGTCTGGACTCGGCGGGCCGCTGCCAGACT
TCCTCG--
---ACGTCGACG
TGC------CGGCGTCGCTCGCTTCGCGCCGAGATTCGCAGCCTG---GAGCTGTGGCGC
GCCGTGATCGGCGAGTGCCTGGCCACCTTCTTCTACGTGTTCCTCGTGTGCGGCGCGCAC
GTGTCCTGGCCGGGCTACGCCGAG------------------------------------
--CCCTCG
GTGCTAGGCATCGCCCTGACGGCCGGCGCGGCGGCCGCGACGCTGGTGCAGTGCTACGGC
CACATATCGGGCGCGCACATGAACCCCGCGGTGACGCTGGCCACGTTCGCCACGCGAAAA
GTGAGCCCGCTCCGGACGCTCCTGTACGTGACGGCCCAGTGCGGAGGAGCTATCGCGGGA
GCTGCGCTTCTGTACGGCGTGTCGGTGCCAGGCCACCAGAGC------------------
------------------------------TCTCTGGGCAGCACTCGCCCCCACGAGGCC
---------CTAGGCGCCTGGCAAGCGTTCGGCGTCGAGTTCGTGCTCAGCTTCCTGCTG
GCCTCCACAGTCTTCGCA---ACCCGCGACCCGCACCGA---------------------
---AGCCACTTG---------GGCGCCGGCTCGGACGCCATTGTCATCGGGTTCGCCTAC
TTGGCCTGCACTCTCGCTGGGCTGCCCGCGACGGGAGCATCAATGAACCCAGCGAGGTCC
TTGGGACCGGCTTTTGTGATGAAC---------AAGTGGGTAGACCACTGGGTGTACTGG
TTCGGGCCCATATCCGGAGGCCTGCTGGCCGGTCTCATCTACGAGTACATCTTCGACACC
---AAGAAGCAGGCGCGGGCG---------CTGCGGAAAACACTC---GAGGAAGCT---
------------------------GACAAA---------GAGTCC------AACACGGAC

---------GAC---GACTACGAG---GACCCT---GACGCGAAGATCGCGAAGTACGCG
ACCGTGGCCCAGAGTCCTCCCAACGGGGCG------CCCTCCAGCGCCCACCAGGCTGCG
CCGTTGCAGCAGTTGGTGCAG------CACCAGCAACAGGGCCGGCCGCAC---------
---CAGCACCAGCAGTACGAGGCG------GTGGGCTTCGGCCGGCCCGCCGGCGGGAAC
------------------ACCTACTCGCCCACT------------------CCGTCCTCG

------------GCCTTCCCG------------------------GCGTCC---------

--GCCGAG
GCGGTGTACGCG--GGCTCG
CTGCGC---CCTACG---
---------------------AACTACGCG------------------------------

------------TCGTCCGTGTAC------GGC---------------------------

------------------------ACCGGCAGA---------------------------
---ATTGGAAATGCCCTT--

------------CGAACACTTCCA------------------------------------
--------------------------------------GCCCGA----------------
-----CTGGAGTATGGACAA---CCTGGATCTCCA-------------------------

---AAATTT-
>XP_037281147_Rhipicephalus_microplus_Bib
ATGGCGGCG---
--AACACCTCGGCCGTCAGC
CTGGAGTCGCTGTTCCTCCAGCTGCTGGGCCGGCTGGACTCGGCGGGACGCTGCACGTCG
TCGTCGACGACG--
---ACGTCTTCC

TGC------CGGCGTCGTTCGCTGCGTTCCGAGATCCGCAGCCTG---GAGCTCTGGCGC
GCGGTGATCGGCGAGTGCCTGGCCACCTTCTTCTACGTGTTCCTGGTGTGCGGCGCGCAC
GTGTCCTGGCCCGGTTACGCCGAG------------------------------------
--CCCTCG
GTGCTGGCCATCGCGCTCACGTCCGGAGCGGCGGCCGCGACGCTGGTGCAGTGCTACGGC
CACATATCGGGCGCCCACATGAACCCCGCCGTGACGCTGGCGACGTTCGCGACGCGAAAA
GTGAGCCCCATACGGACGATCCTCTACGTGTCGGCGCAGTGTGGAGGTGCTATCGCGGGA
GCTGCGCTTCTGTACGGCGTGTCGGTGCCAGGCCACCAGAGC------------------
------------------------------TCTCTGGGCAGCAACCGGCCCCACGAGGCT
---------TTAGGCGCCTGGCAGGCGTTCGGCGTCGAATTCGTGCTCAGCTTCCTGCTG
TCCTCTACAGTGTTCGCC---ACCAGGGACCCTCACCGT---------------------
---AGCCACCTG---------AGTGCCGGCTCGGACGCCGTGGTCATCGGGTTCGCATAC
CTCGCCTGCACTCTGGCCGGGCTGCCAGCGACTGGAGCGTCGATGAACCCGGCGAGGTCG
TTGGGACCGGCTTTTGTGATGAAC---------AAGTGGGTCGACCATTGGGTGTACTGG
TTCGGGCCGATATCAGGAGGCCTGCTGGCTGGTCTCATCTACGAGTACATCTTCGAAACC
---AAGAAGCAAGCGCGGGCG---------CTGAGGAAAACTCTC---GAGGACGCT---
------------------------GACAAA---------GAGTCC------AACACAGAC
---------GAC---GACTATGAA---GACCCT---GACGCGAAGATCGCGAAGTACGCG
ACCGTGGCCCAGAGTCCTCCCAATGGGGCGGCG---CCCTCCAGCGCCCACCAGGCTGCG
CCATTGCAG---CTGGTGCAG------CACCAGCAGCAAGGCCGGCCGCAC---------
---CAGCACCAGCAGTACGAGGCG------GCGGGCTTCGGCCGGCCTGTTGGAGGGAAC

------------ACCTAC---------------------------TCGCCCACTCCGTCG
TCCGCCTTCCAG------------------------------GTGCCC------------

--ACAGAG
GCGGCGTACGCA--GGATCG
CTGCGC---TCCGCC---
---------------------AACTACGCG------------------------------

------------TCGTCCGTGTAT------GGCGGC------------------------

---------------------------ACCAGA---------------------------
---CTTGGAACTGCACTT--

------------CGGACACTTCCG------------------------------------
--------------------------------------GCCCGA----------------
-----TTGGAGTATGGACAA---CCCGGATCTCCA-------------------------

---AAATTT-
>XP_002408745_XP_002408744_Ixodes_scapularis_Bib
ATGGCAGCG---
---ACGGTGAGC
CTCGAGACGCTCTTCCTCCAGCTGCTGGGCCGGCTGGAGTCGACCGGGCGCTGCCCCGTG
GCCTCCGGGACGGTC---
--TTGTCCCCGTCG
GCTGCCAAGCAGCGTCGCTCGCTGCGGGTCGAGATCCGGACGCTG---GAGCTGTGGCGC
GCCGTGATCGGCGAGTGCCTGGCCACCTTCTTCTACGTGTTTCTGGTGTGCGGCGCTCAC
GTGTCGTGGCCGGGATACGCGGAA------------------------------------
--CCGTCG
GTGCTGGGCATCGCGCTCACGGCGGGCGCGGCGGCGGCCACGCTGTGCCAGTGCTACGGC
CACATCTCGGGCTGCCACATGAACCCGGCCGTGACGCTGGCCACATTCGCGACGCGCAAA
GTGAGCCCGCTCAGGGCGCTCCTCTACGTGACGGCGCAGTGTGGAGGCGCCATCGCGGGA
GCTGCGCTTCTCTACGGG---ACGGTGCCAGGCCACCAGAGC------------------
------------------------------TCTCTGGGCAGCAGCCGGCCCCACGAGGCC
---------CTAGGCGCCTGGCAGGCGTTCGGCGTGGAGTTCGTGCTCAGCTTCCTGCTC
GCCTCCACCGTGTTCGCC---AGCCGAGATCCCAGCAGG---------------------
---AGCCACCTG---------------GGAGGAGACGCCGTCGTCATCGGCTTCGCCTAC
CTCGCCTGCACGCTGGCCGGGTTGCCGGCGACGGGAGCTTCCATGAACCCGGCCAGGTCT
CTGGGACCTGCCTTTGTCATGAAC---------AAGTGGATCGACCACTGGGTGTACTGG
TTCGGACCTGTGGCTGGAGGTCTGCTGGCCGGTCTCATCTACGAGTACATCTTCGATACG
---AAGAAGGGATCGAGGGCG---------CTCAAGCAGAGTCTC---GAAGACGCC---
-------------------------GACAAA---------GAGTCG------AACACGGAC
---------GAC---GACTACGAG---GACCCT---GACGCGAAGATCACGAAGTACGCG
ACCGTGGCGCAGAGTCCCAAGGGCGGCGTG---------TCGGGCCACCAGCAGTGCCGG
GGCGCGCAG---
---CAGCAGCAGCAGTACGAGGCT------GCCGCGGGATTCCGGCCCGCC---GCCAAC

------------ACCTAC---------------------------TCGCCCACTCCATCG
TCGGCCTTC---------------------------------CCGGCCGCC------

--ACGGAC
ACCTACGGGGGA--TCCCAC
GCAGCG---GCGGGC---
---------------------ACCTACGCT------------------------------

------------GCGCCGATCTAT------GGT---------------------------

---------------------------ACCAGG---------------------------

>GEWZ01021936_Nephilengys_cruentata_Bib
ATGGCG--
---ACGGTGTCA
TTAGAGGCATTTCTTGTGCACTTATTGCACAAAGCAGAACAGACTCGCACTGAA------

TTG---AACAGAAAGAAGACAATGATTGTGGAACTTCGCACACTT---GAGTTTTGGAGA
GCAATCATTGCTGAATGCCTGGCCACGTTTATTTACGTGTTTCTAGTCTGTGGATCCCAT
GTGATGTGGCCATTGTATTCTATT------------------------------------
---AAC
ACCTTAACCAAATCTTTTGCGAATGGTCTGGCAATGGCCACAGCAGCTCAATGCTTCGGA
CACATTTCTGGAGCTCATGTGAATCCTGCTTTTACTTTCGCAATGCTAGTCATCCAGAAA
GTTACACCTCTACGAGCTTTCTTGTACATCACCGCACAATGTGGAGGAGCTATAGCGGGT
TCTGCACTTTTGTACGGGGTTACCGTACCTGGAATTGGTTTC------------------
-------------------------CGAGAGAACATGGATGTACTTTGGTCAGTGACTCC
---------CTCTCTTCTTGGCAGGGCATGGGCCTGGAGCTAGTCCTCACCTTTGTGGTC
ACATTTACTATGTGTTCT---ACAGCAGACCCGAATAGG---------------------
---AAGTCTCTC---------------GGCAGCGATTCTTTGGCCGTAGGATTGGCCTAC
CTTGTGTGCACTTTAATTGCTCTTCCAGCTACTGGTGCATCAATGAATCCTGCTCGATCT
CTTGGGCCCGCTTTTGTCTCCAAC---------ACGTGGACCAATCATTGGATCTACTGG

```
GCCGGCCCTGTGGTGGGAGGATTACTAGCTGCTCTCATTTACGAGTACATCTTTGATTCA
---CCAAAG------AAAGTT---------TTGCCT---ACGGTT---AAAGACAGA---
------------------------GAT------------GATTCG------GAGAAAGAT
GTATCT---GAG---ATCAAAGAT---GACAGC---GACAGTAAAACATCATCCGTAATA
------------TCGCCAAGA---------------------------GCTCAAATGGCG
CAAACT------------------------------------------------------
---CAAGTACAGCCAACAGTTGTA---GGTGGACCTTACTATAGACCGCTGACACCACAC
------------------GATGCCAGT------------------------TCTACAATG
------------------------------------------------------------
---------------------CGA---CCT------------------------------
CATTCTCGAAGCACATAT---------------------------ACG---------ACT
GCAAAGTATATTGCT---------------------------AGTCCAAGTGGCAGT---
------------------------------------------------------------
------------------------------------------------------------
------------------------------------------------------------
------------------------------------------------------------
------------------------------------------------------------
---------------------ATGGGCAACCATCCACCGATGCCGAATACA---------
------------------------------------------------------------
ACTTACTATAGT------------------------------------------ACACTA
CCGAGA------------------------------------------------------
------------------------------------------------------------
------------------------------------------------------------
------------------------------------------------------------
------------------------------------------------------------
------------------------------------------------------------
------------------------------------------------------------
------------------------------------------------------------
------------------------------------------------------------
------GGCAGTAGCTAC------------------------------------------
------------------------------------------------------------
------------------------------------------------------------
------------------------------------------------------------
------------------------------------------------------------
------------------------------------------------------------
------------------------------------------------------------
--------------------------------------AGGTCT---------------T
GG---AACGATTATGGT------TCTACGCAGTTC-------------------------
------------------------------------------------------------
------------------------------------------------------------
------------------------------------------------------------
------------------------------------------------------------
------------------------------------------------------------
------------------------------------------------------------
-----------------------------------------------AAGTTT-
>GFKT014357330_Nephila_clavipes_Bib
ATGGCG------------------------------------------------------
---------------------------------------------------ACGGTGTCA
```

```
TTAGAGGCATTTCTTGTGCACTTACTGCACAAAGCGGAACAGACTCGTACCGAA------
------------------------------------------------------------
------------------------------------------------------------
TTG---AACAGAAAAAAGACAATGATTGTAGAACTTCGCACGCTG---GAATTTTGGCGA
GCAATCATTGCTGAATGTTTGGCTACATTTATTTACGTATTTCTAGTCTGTGGATCCCAT
GTGATGTGGCCATTGTATTCCATC------------------------------------
---------------------------------------------------------AAC
ACTTTGACCAAATCTTTTGCGAATGGCCTGGCTATGGCCACAGCAGCTCAGTGCTTCGGA
CACATCTCTGGAGCTCATGTGAATCCTGCATTTACTTTTGCAATGCTAGTCATCCAGAAA
GTTACACCTCTACGAGCTTTCTTGTACATCACTGCACAATGTGGAGGGGCTATAGCGGGT
TCTGCTCTTTTGTACGGCGTAACCGTACCTGGAATAGGGTTT------------------
------------------------CGAGAAAACATGGGATGTACTTTGGTCAGTGATTCC
---------CTCTCTTCTTGGCAGGGCATGGGCCTGGAGCTAGTCCTCACCTTCGTGGTC
ACTTTCACCATGTGTTCT---ACAGCAGATCCGAACAGG---------------------
---AAGTCTCTC---------------GGAAGTGATTCTTTAGCTGTAGGATTGGCTTAT
CTTGTATGCACTTTGATAGCTCTTCCAGCTACTGGTGCATCGATGAATCCAGCCAGATCT
CTTGGACCCGCCTTTGTCTCTAAC---------ACGTGGACCAATCATTGGATCTACTGG
GCTGGCCCTGTGGTGGGAGGATTACTGGCTGCCCTCATTTATGAGTATATCTTTGATTCA
---CCCAAG------AAAGTC---------TTACCT---ACGGTC---AAAGATAGA---
------------------------GAT------------GATTCG------GAGAAAGAT
GTATCT---GAG---ATAAAAGAC---GACAGC---GACAGCAAAACATCATCTGTAATA
------------TCGCCACGA---------------------------ACTCAACTGGCT
CAAACC------------------------------------------------------
---CAAGTGCAGCCGACAGTTGTA---GGTGGACCTTACTATAGACCACTGACACCTCAC
------------------GATGGCAGT------------------------TCTACGATG
------------------------------------------------------------
---------------------AGA---CCC------------------------------
CATTCTCGAAGCACATAT---------------------------ACG---------TCT
GCAAAGTACATTGCT---------------------------AGTCCTAGTGGCAGC---
------------------------------------------------------------
------------------------------------------------------------
------------------------------------------------------------
------------------------------------------------------------
------------------------------------------------------------
---------------------ATGGGCAACCACCAACCGATGCCGAATACA---------
------------------------------------------------------------
ACTTACTACAGC------------------------------------------ACCTTG
CCCAGA------------------------------------------------------
------------------------------------------------------------
------------------------------------------------------------
------------------------------------------------------------
------------------------------------------------------------
------------------------------------------------------------
------------------------------------------------------------
------------------------------------------------------------
------------------------------------------------------------
------GGCAGTAGTTAC------------------------------------------
------------------------------------------------------------
```


--------------------------------------AGATCT---------------T
GG---AACGATTATGGC------TCTACGCAGTTC-------------------------

---AAGTTT-
>GIWY01069267_Argiope_argentata_Bib
ATGGCA--
---ACCGTGTCG
TTGGAAGCATTTCTTGTGCACCTGATCCACAAGGCAGAACAAACCAGGGGCGAG------

CTG---AACAGAAAGAAGACAATGATTGTGGAACTTCGCACCCTT---GAATTTTGGAGG
GCTATTATAGCTGAATGTCTGGCCACTTCTATATACGTGTTTTTGGTCTGTGGATCCCAC
GTTATGTGGCCTCTGTACTCTATT------------------------------------
---AAC
ACTTTAACCAAATCGTTTGCAAATGGCCTGGCCATGGCCACTGCTGCCCAATGCTTCGGA
CACATATCTGGGGCTCACGTTAACCCTGCGTTTACGTTTGCGATGCTAGTCATTCAAAAG
GTGACACCTCTAAGAGCTTTCTTGTACATTACAGCCCAGTGTGGAGGGGCCATTGCCGGG
GCTGCGCTTCTGTATGGTGTGACCGTACCTGGCATTGGCTTC------------------
------------------------AGAGAGAACATGGGTTGCACTTTGGTCAGCGATTCG
---------CTGTCCTCCTGGCAGGGCATGGGTCTGGAGCTGGTTCTCACCTTTGTGATC
GCCTTCACCATGTGCTCC---ACTGCCGATCCGAACCGG---------------------
---AAATCTCTG---------------GGCAGTGACTCTTTAGCAGTGGGCCTGGCATAT
CTTGTATGCACCCTCATAGCTCTACCAGCTACTGGGGCATCTATGAATCCAGCTAGATCT
CTTGGACCTGCTTTCGTTTCAAAT---------ACTTGGACTAACCATTGGGTTTACTGG
GCCGGACCTGTTCTCGGAGGATTGCTGGCCGGTCTTATCTACGAGTACATCTTTGATTCC
---CCAAAG------AAGGTC---------TTACCT---GCTGTC---AAAGATAGG---
------------------------GAC------------GATTCC------GATAAAGAT
ATGTCT---GAA---GTCAAAGAT---GACAGC---GACAGCAAGACCTCCTCCGTTATA
------------TCCCCAAGA---------------------------GCTCAAATTCCA
CAAACG--
---CCTGTGCAACAACCTTCAGCT---GGCGGGCCATACTACAGACCTCTGACTCCCCAT
------------------GATGGCGTC------------------------TCCACCATG

---------------------AGA---CCC------------------------------
CATTCCCGAGGCACCTAC---------------------------ACT---------TCA
GCCAAATACATTGCT---------------------------AGTCCGGGTGCCATG---

---------------------GTGAGTCATCAACAGGTGCAACCAGCGACT---------

ACTTACTACAGT--ACCCTT
CCTAGA---GGG--

---GGCGGAGGTACCTAC--

--------------------------------------AGGTCA---------------T
GG---AATGATTATGGG------TCAACGCAGTTC-------------------------

---AAATTT-
>GITM01031683_Tetragnatha_brevignatha_Bib
ATGGCG--
---ACAGTATCC
CTGGAGGCCTACATGGTCCACCTCATACACAAGGCAGACCAGACCCGGTGCGAG------

TTG---AACCGCAAGAAGACAATGATCGCCGAGCTTCGCACCCTG---GAGTTCTGGCGG
GCGATCATCGCAGAGTGCCTGGCCACCTTCGTGTACGTTTTCCTGGTGTGCGGATCGCAC
GTCATGTGGCCTATGTACTCCATC------------------------------------
---AAC
ACCTTGACCAAGTCCTTCGCCAATGGCCTGGCCATGGCGACTGCTGCGCAGTGTTTTGGA
CATATATCGGGGGCGCACGTCAACCCTGCGTTCACCTTTGCTATGCTGGTCACGCAAAAA
GTCACCCCATTGAGGGCTTTCTTGTATATCACGGCGCAGTGTGGAGGGGCCATCGCTGGA
GCTGCCTTGCTCTACGGAGTGACGGTGCCTGGAGCGGGGTTC------------------
------------------------AGGGACAACCTGGGCTGCACGCTGCTGAGCGAGTCG
---------CTGTCGGCCTGGCAAGGGCTAGGGCTGGAGCTGGTGCTAAGCTTCGTGGTC
GCCTTCACCATGTGCGCC---TCAGCCGACCCGAACCGC---------------------

---ACCGCACTG---------------GGCAGCGATGCCCTCGCCGTCGGCCTCGCCTAC
CTCGTCTGCACGCTCGTCGCGTTACCGGCTACTGGAGCATCAATGAATCCTGCGAGATCA
TTGGACCTGCTTTTGTTTCAAAT----------ACATGGTCTAATCATTGGATATATTGG
GCCGGTCCCGTGATTGGAGGACTCTTGGCTGGACTGATCTACGAGTACATATTTGAATCT
---CCCAAG------AAACTT---------CTGCCC---ATCATC---AAGGACAGA---
------------------------GAA------------GATTCC------GAAAGAGAC
ATCTCG---GAC---GCTAAAGAC---GACAGC---GACAGTAAGTCCTCTTCCGTGCTG
------------TCTCCTCGA---------------------------ACC------CCC
AACAGC--
---CAGATCCAGAACCAGACCCCTCCCACCAGACCCTACTACCGCCCCTTGACCCCTCAC
------------------GACACC---------------------------GGGAGCGTC

---------------------CGG---TCC------------------------------
CAGTCTCGAGGCACCTAC---------------------------GCC------CCCGCT
GCCAAGTACATGTCT---------------------------AGTCCCGTCAACAAC---

------------------------------CACCAGCAGTTCCCCAATGCC---------

ACCTACTACAGT--ACCCTC
CCTAGG---GGT--

---GGAGGAGGAGGGTAC--

--------------------------------------AGGAAC---------------T
GG---AACGAGTACAGC------CAGACCCAATTC-------------------------

---AAGTTC-

>GBCS01010810_Latrodectus_hesperus_Bib
ATGGCT--
---ACTGTATCC
TTGGAAGGATATCTTGTACATTTGATCCACAAAGTGGAACAATCGCGTTATGAG------

CTC---AACAAGAAGAAAACAATGATTGCTGAATTGAGAACTCTA---GAATTTTGGCGA
GCAATCATAGCAGAGTGCCTGGCTACATTCATATATGTGTTTCTAGTGTGTGGCTCACAT
GTCATGTGGCCACTGTACTCCATC------------------------------------
---AAC
ACCCTCACGAAGTCATTTACCAGCGGACTAGCCATGGCGACCGCCGCTCAATGTTTTGGC
CACATATCTGGAGCTCATGTCAATCCAGCATTTACATTTGCAATGCTCGTCATTCAGAAA
ATAACTCCACTGAGAGCTTTTCTGTATATAACAGCCCAGTGCGGTGGAGCTATAGCAGGT
GCAGCTCTGCTCTACGGAGTGACAGTTTCTGGCATTGGACAT------------------
------------------------CGAGAGAACTTGGGATGCACAGTGATAAGCGACTCC
---------TTATCGTCGTGGCAAGGCATGGGCCTAGAGTTGGTCCTGAGCTTCATTGTT
TCTTTCACAATGTGTTCC---ACAGCTGATTCTAATCGG---------------------
---AAGACATTG---------------GGAAGTGACGCCTTAGCAATTGGATTCGCTTAT
TTGGTGTGCACATTGATTGCTCTTCCCGCTACAGGCGCCTCCATGAATCCAGCGAGATCA
TTGGGTCCAGCTTTTGTCATGAAT---------ACGTGGACAAATCATTGGGTATACTGG
GCAGGTCCTGTAATTGGAGGCTTATTAGCTGGTCTAATATATGAATACATATTCGAATCG
---CCGAAG------AAAATT---------TTAGCT---ACTTAT---AAGGAACGT---
------------------------GAA------------GATTCA------GAAAGAGAT
ACATCA---GAA---GCGAAGGAT---GACAGC---GACAGTAAATCTTCAGCAGTGCTC
------------TCACCACGA---------------------------TCACATATTCCT
CCAATA--
---CCATTACAGCATCAACCTTCATCAGGAGGTCCTTACTATAGACCACTCACTCCCCAT
------------------GAGCAG---------------------------TCAAGTCTG

---------------------AGG---CCA------------------------------
CATTCCCGCAACGCCTAC---------------------------ACATCCACTTCTACC
AAG---TACACCACA---------------------------AGTCCAAGTGCTTCA---

------------------------------CACCAGCATCTTCAAAATCCA---------

GGATATTACAGC--TCTTTG
CCGAGA---GGTGGC---

---GGGGGTGGTACTTAT--

--------------------------------------AGATCA---------------T
GG---AATGAATATGGG------AGTTCGCAATTC-------------------------

---AAGTTT-
>IAAA01028386_Parasteatoda_tepidariorum_Bib
ATGGCG--
---ACTGTTTCT
TTAGAAGCATTTCTTGTGCATTTGATTCACAAAGTTGAACAATCGCGATGCGAA------

ATC---AACAAAAAGAAAACTATGATAGCCGAATTAAGAACTTTG---GAATTTTGGAGA
GCCATTATTGCTGAATGTTTGGCGACTTTTATGTATGTTTTCTTAGTTTGTGGCTCACAC
GTAATGTGGCCTCTATATTCCATT------------------------------------
---AAC
ACTCTCACAAAATCATTCACCACTGGACTAGCAATGGCAACTGCGGCTCAGTGCTTTGGT
CATATATCTGGAGCTCATGTGAATCCAGCTTTTACTTTCGCTATGGTGGTAATACAAAAA
ATCACACCTTTGAGAGCTTTTCTTTATATAACTGCTCAATGTGGTGGAGCTATTGCTGGA
GCTGCTTTGCTTTATGGCGTAACAGTGCCTGGTATGGGACAC------------------
------------------------AGAGAAAATCTAGGCTGTACAGTAATAAGCGAATCC
---------TTAACATCATGGCAGGGCATGGGATTGGAATTAGTCTTAACATTTATAGTA
GCTTTCACAATGTGTTCT---ACGGCTGATCCTAATCGA---------------------
---AAAACTCTT---------------GGAAGTGATGCCTTGGCAGTAGGATTCGCTTAT
TTAGTGTGCACACTAATTGCGTTACCAGCCACTGGTGCTTCTATGAATCCTGCACGTTCT
TTGGGACCAGCATTTGTAATGAAT---------ACATGGACAAACCACTGGGTATATTGG
CTAGGGCCAGGTATAGGAGGACTATTGGCTGGCTTAATATACGAATACATATTCGAATCT
---CCTAAG------AAGATA---------ATGCCA---ATTTAT---AAAGAAAGA---
------------------------GTT------------GATTCT------GAAAGAGAT
ATTTCC---GAA---GCTAAAGAT---GACAGC---GACAGCAAATCTTCTACAGCTTTG
------------TCTACACGA---------------------------ACACAGATACCT
CCGATA--
---CCTTTACAGCATCCGCCTTCTGCAAAGGGTCCGTACTACAGGCCTTTAACACCACAC
------------------GAGGCAACC------------------------ACAACGCTT

---------------------AGA---CCA------------------------------
CATACTCGGAATGCTTAC---------------------------ACATCAACAGCAACA

TCGAAATATGTGCCT---------------------------AGTCCAAGTACTTCT---

------------------------------CATCAACAGCTTCACAATTCT---------

GGGTACTACAAT--TCGCTG
CCTAGG--

---GGTGCCGGTACTTAT--

--------------------------------------AGGTCA---------------T
GG---AATGAATACGGC------TCTTCTCAATTC-------------------------

---AAATTT-
>IAQA01014588_Octonoba_yesoensis_Bib
ATGGCT--
---ACTGTTTCT
TCGGACGCTTACCTTCTGCAATTATTTTACAATGCTGAAAGATCTCGTCATGCA------

CTT---AACAAGAAAAAAACCATGTTGGTGGAATTGCGCACTCTT---GAATTCTGGCGG
GCTATTATAGCCGAATGCTTGGCGACGTTCTTGTACGTGTTTCTGGTTTGCGGTTCTCAT
GTGATGTGGTCTATACATTCTACG------------------------------------
---AAC
ACATTGACAAAGTCTTTTACGAACGGTTTTGCGATGGCCACTGCTGCCCAGTGCTTTGGC
CACATTTCTGGAGCCCACATCAACCCCGCTTTCACCTTTGCGATGCTGGTGACACAGAAA
GTTACGCCGTTGCGAGCGTTTTTGTACATCACTGCCCAGTGTGGGGGTGCAATAGCCGGT
GCAGCTTTGTTGTACGGGATTACAGCTACCGGTCTAGCAGCTCCC---------------

------------------------CGAGAGAGTCTTGGCTGCACAGTAGTAAACGAATCT
---------CTTTCCCCGTGGCAAGGCGTGGGTCTAGAACTCGTGCTTACTTTCGTGGTG
GCTTTCACGATGTGTGCC---ACTCAAGATCCGAACAGA---------------------
---CGATCTCTG---------------GGAAGCGACGCTTTGGCAGTAGGCGTTGCTTAT
CTGGTCTGCACACTTGTAGCACTTCCAGCAACCGGCGCTTCCATGAATCCCGCACGGTCT
CTGGGTCCAGCTTTTGTCATGAAT---------ACATGGACTAATCACTGGGTGTACTGG
CTCGGACCAGTTGTAGGCGGGCTGCTTGCTGGATTGATTTATGAGTACATATTCGATTCA
CCGCCGAAG------AAAATT---------ATAGCC---AGTAAT---AAAGATAGA---
------------------------GAT------------GATTCG------GAGCGAGAT
ATTTCG---GAA---ATTAGAGAC---GATAGC---GACAGCAAGTCTTCATCGGCATTG
------------TCCCCGCGG---------------------------ACAGCAGTTCCT
CAACTG--
---CAGTTGCATCCTCAGCAACCCACTGGAGGACCCTACTACAGACCTCTCACCCCTCAC
------------------GAAAACCAC---------------------------------

---------------------AGA---CCA------------------------------
CAATCCCGC---

>GFT15823_Nephila_pilipes_Blip
ATGGCCAGCCCG--AACGAT
CTCTATTCCATTCCCGAATCAAAC---------------------------AAATTAACC
AAC---------CCGCCTAACGCTTATTCTCCCGTTGAAACCATGCGACGTTACATAGAT

---AGC
CTTCCGCAAGATCTG---TCACTCCGGGAAGAACTCACAGACCAG---CATTTCTGGAAA
GCTGTAAGGACAGAATTTTTGGCTACTTTACTCCTCACCGTGTTGGGCTGTGGATCTTGT
TTACGATGGAAGGCTCCTCCAACC------------------------------------
------------------------------GAT------CAGATAACAGCATCGGCCGTA
GAGTTGAGGGTAGCCTTAGCCTTCGGACTCGGCGTGGCTACTCTGGTTCAGTGCGTGGGT
CAAGTAAGCGGTGGCCACATGAATCCTGCTGTAACAGTAGCCTTGTTGGTAACTAGACAT
ATCAGTGTAGCCAGAACTGCTGCGTATGTACTGGCTCAATGTCTTGGCGGACTCGCAGGA
GCAGGCATTCTGTATGGTCTTACTCCTCAGGGTCAGAGGTTAAGT---------------
---------------------------GCTGGACTTGGAGCTACAGAACCAGCAGTGGGA
---------GTGGCGGCTTCGGAAGCGTTTGGCATAGAGTTCATGGCTTCTGTGGTGGTG
GTTATTACGGTGCTATCT---AATATGGACCCACAGAGA---------------------
---ACTGAGATG---------------GGATGTAAATCGCTATCCATAGGGTTTTCGTAC
GTCGTGGGTCATCTTTTGGCGTTCCCTTATACAGGGGCCAGTTTCAATCCAGCAAGATCA
TTTGGTCCAGCGATTGTTACAAAT---------ACATGGAGACTACATTGGATTTATTGG
GTGGCTCCCGTTCTGGGAGGTATCATCGGCGGTGTCACCTACGATTATACACAAGAATCC
---CATTCG---CAGCAAACC---------CTACGGCGTTCGTTT---CGAAAAAAG---
------------------------TCAAGC---------AGGACTGGCATGCAACGAGAT
ATGAGC---GCT---TTATCCAATAACGAGACA---GAACTCAGCTTGGATCACTGT---
------------CGTCTC--

>GFKT014317704_Nephila_clavipes_Blip
ATGGCCACCCCG--AACGAT
CTCTATTCTATTCCCGAATCTAAC---------------------------AAACTAACC
AAC---------CCGTCAAACGTTTACTCTCCTGTTGAAACAATGCGACGCTACATCGAT

---AGC
CTTCCGCAAGATCTG---ACTCTCAGAGAAGAACTCACCGACCAG---CATTTCTGGAAA
GCTGTAAGGACAGAATTTTTGGCTACTTTACTCCTTACCGTGCTGGGCTGTGGTTCTTGT
TTACGATGGAAGGCACCCCCAACT------------------------------------
------------------------------GAT------CAAATGACAGCATCAGCTGTA
GAGTTGAGGGTAGCCTTAGCCTTCGGACTCGGCGTGGCTACTCTTGTCCAGTGCGTGGGT
CAAGTAAGTGGTGGCCACATGAATCCGGCTGTAACAATAGCCTTGTTGGTAACCAGACAT
ATCAGTGTGGCCAGAACTGCTGCGTATGTGCTGGCTCAATGTCTTGGCGGACTCGCAGGG
GCAGGTATTCTGTACGGTCTCACCCCTCAGGGTCAAAGGTTGAGT---------------
---------------------------GCAGGACTTGGAGCCACAGAACCAGCAGTAGGA
---------GTGGCTGCTTCTGAGGCATTTGGCATAGAATTCATGGCTTCTGTGGTGGTA
GTTATCACGGTGCTATCT---AATATGGATCCACAAAGG---------------------
---ACTGAAATG---------------GGATGCAAATCGCTATCCATTGGGTTTTCGTAT
GTTGTGGGGCATCTGTTGGCGTTCCCTTATACTGGTGCCAGTTTCAATCCAGCAAGATCG
TTTGGTCCAGCGATTGTAACAAAT---------ACTTGGAGACTACATTGGATTTACTGG
GTGGCTCCTGTGCTGGGAGGTATCATCGGTGGTGTCACGTACGATTATACGCAGGAATCC
---CATTCG---CAGCAAACT---------TTACGGCGTTCTTTT---CGGAAAAAG---
------------------------ACAAGC---------AGGACTGGGATTCAACGGGAT
TTGAGC---GCC---TTGTCCAATAACGAGACA---GAACTTAGCTTAGATCACTGT---
------------CGTCTT--


```
------------------------------------------------------------
------------------------------------------------------------
------------------------------------------------------------
------------------------------------------------------------
------------------------------------------------------------
------------------------------------------------------------
------------------------------------------------------------
------------------------------------------------------------
------------------------------------------------------------
------------------------------------------------------------
------------------------------------------------------------
------------------------------------------------------------
------------------------------------------------------------
------------------------------------------------------------
------------------------------------------------------------
------------------------------------------------------------
------------------------------------------------------------
------------------------------------------------------------
------------------------------------------------------------
------------------------------------------------------------
------------------------------------------------------------
------------------------------------------------------------
------------------------------------------------------------
------------------------------------------------------------
------------------------------------------------------------
------------------------------------------------------------
------------------------------------------------------------
------------------------------------------------------------
------------------------------------------------------------
------------------------------------------------------------
------------------------------------------------------------
------------------------------------------------------------
------------------------------------------------------------
------------------------------------------------------------
------------------------------------------------------------
------------------------------------------------------------
------------------------------------------------------
>KAF8768105_Argiope_bruennichi_Blip
ATGGCCAGCTCC------------------------------------------ACTGAT
CTCTACTCCATCCCCGAGACAGGC---------------------------AAACTGACC
AAC---------CCAAACAACGTCTACTCTCCGGTCGAGACCATGCGGCGTTATATGGAA
------------------------------------------------------------
---------------------------------------------------------AGT
CTCCCGCAGGACCTC---ACCCTCAAAGAAGAACTTACAGACCAG---CATTTCTGGAAG
GCTGTACGGACAGAATTCTTGGCTACTCTGTTACTCACAGTGTTGGGTTGTGGGTCCTGC
TTGCGATGGAAGGCACCTCCTTCT------------------------------------
------------------------------GAC------CAGATGACTGCCTCGGCCGTC
GAACTGAGAGTGGCCCTGGCTTTTGGACTCGGTGTGGCCACTTTGGTCCAGTGTGTAGGC
```

CAGGTGAGCGGTGGTCACATGAACCCGGCTGTGACAGTGGCCCTGCTTGTGACCAGGCAC
ATCAGTTTGGCCAGGAGTGCTGCGTACGTGCTGGCCCAGTGTCTGGGTGGTCTTGCTGGG
GCTGGAATTCTGTATGGTCTGACACCTCAGGGTCAAAGACTGAGC---------------
---------------------------GCTGGCCTAGGGGCCACCGAGCCGGCGGATGGC
---------GTGGCAGCTTCTGAAGCGTTCGGCATAGAGTTCATGGCTTCTGTTGTAGTG
GTTATCACGGTGCTATCT---AACATGGATCCTCAGAGA---------------------
---ACTGAGATG---------------GGCTGCAAGTCGTTATCAATTGGGTTTTCGTAT
GTGGTAGGACATCTGCTGGCGTTTCCTTATACCGGTGCCAGTTTCAATCCAGCAAGGTCA
TTCGGTCCAGCAATCGTCACGAAC---------ACGTGGAGATTACATTGGATCTACTGG
GTGGCACCAGTGCTCGGTGGCATAATTGGAGGCGTGACCTACGACTACACGCAGGAGTCG
---CATTCT---CATCAATCT---------CTACGGCGGTCCTTC---CGCAAGAAA---
------------------------CCTGCC------------------------AGGGAT
CTCAGC---GCG---CTCTCGAACAACGAGACT---GAGCTCAGCCTGGATCACTGC---
------------AGACTC--

>GIY42265_Caerostris_extrusa_Blip
ATGGCCAGTTCG--AACGAT
CTCTATTCCATCCCCGAATCCAGA---------------------------AAGCTCTCT
GCT---------CCCAGCAACGTCTATTCACCAGTCGAGACCATGCGTCGGTACATGGAC

---AGC
CTCCCCCAGGATTTA---TCTCTCAAGGAAGAACTGACAGATCAA---CATTTCTGGAAG
GCCGTACGGACAGAATTCCTGGCCACTTTGTTGCTCACAGTGCTGGGATGTGGGTCGTGC
CTTAGATGGAAGGCACCCCCCACA------------------------------------
------------------------------GACCATGACCAGATTACCATGGCAATTGTA
GAACTGAGGGTAGCCTTAGCTTTTGGACTTGCAGTGGCTACTCTTGTCCAGTGCGTTGGA
CAGGTGAGCGGTGGACACATGAATCCAGCCGTGACAGTGGCCCTTTTGGTGACAAGACAC
ATCAGTGTCGCCAGGAGTGCAGCATATGTTCTGGCCCAATGTCTTGGTGGTCTTGCAGGA
GCAGGAATATTGTACGGCTTGACCCCCCAGGGCCAGCGGTTGAAT---------------
---------------------------GCTGGACTTGGAGCAACCGAGCCAGCGGTTGGA
---------GTGGCGGCGTCAGAGGCTTTTGGAATGGAGTTCATGGCTTCAGTGGTGGTG
GTGATTACAGTGCTATCT---AATATGGATCCTCAGAGG---------------------
---ACTGAGATG---------------GGATGCAAATCATTGTCCATTGGGTTTTCTTAT
GTGGTGGGACATCTAATGGCGTTCCCTTACACAGGAGCCAGCTTCAATCCAGCAAGATCA
TTTGGTCCAGCAATCGTAACAAAT---------ACGTGGAGATTACATTGGATATATTGG
GTTGCTCCCGTGTTGGGTGGTATTATTGGTGGAGTTACGTACGACTACACCCAGGAGTAT
---CACCCG---CAGCAAACC---------CTGAGGAGATCCTTT---CGCAAGAAA---
------------------------GGGGGCATCACTGCAAGGGACCAGATTCAACGTGAT
CTGAGC---GCC---CTCTCCAATAACGAAACG---GACATCAGTCAGGACCACTGC---
------------CGTCTA--

>GIY41786_Caerostris_darwini_Blip
ATGGCCAGTTCG--AACGAT
CTCTACTCCATCCCCGAATCCAGA---------------------------AAGCTCTCT
GCT---------CCCAGCAACGTCTATTCTCCGGTCGAGACCATGCGTCGGTATATGGAC

---AGC
CTACCCCAGGATTTA---TCTCTCAAGGAAGAACTGACAGATCAA---CATTTCTGGAAG
GCCGTACGGACAGAATTCCTGGCCACTTTGTTGCTGACAGTGCTGGGTTGTGGGTCGTGC
CTCAGATGGAAGGCACCCCCCACG------------------------------------
------------------------------GACCATGACCAGATTACCATGGCAATTGTG
GAACTGAGGGTAGCCCTGGCTTTTGGACTTGCAGTGGCTACTCTTGTCCAGTGCGTGGGA
CAGGTCAGTGGTGGACACATGAATCCAGCTGTGACAGTGGCCCTTTTGGTGACAAGACAC
ATCAGTGTCGCCAGGAGTGCAGCATATGTGCTGGCCCAATGTCTTGGGGGTCTTGCTGGA
GCAGGAATATTGTATGGCCTGACCCCCCAGGGCCAGAGGTTGAGT---------------
---------------------------GCTGGACTTGGAGCAACCGAGCCAGCGGTTGGA
---------GTGGCAGCTTCAGAGGCTTTTGGAATGGAGTTCCTGGCTTCAGTAGTGGTG
GTGATTACAGTGCTATCT---AATATGGATCCTCAGAGG---------------------
---ACTGAGATG--------------GGATGCAAATCATTGTCCATTGGGTTTTCGTAT
ATGGTGGGACATCTAATGGCGTTCCCTTACACAGGGGCCAGCTTCAATCCAGCAAGATCA
TTTGGTCCAGCAATCGTAACAAAT---------ACATGGAGATTACACTGGATATATTGG
GTTGCCCCCGTGTTGGGCGGCATCATCGGCGGAGTCACGTACGACTACACCCAGGAGTAC
---CACCCC---CAGCAGACC---------CTGAGGAGATCCTTC---CGCAAGAAA---
------------------------GGGGGCATCACTGCAAGGGACCAGATCCAACGGGAC
CTGAGC---GCC---CTCTCCAATAACGAAACG---GACATCAGTCAGGATCACTGC---
------------CGTCTG--

>GGRD01220096_Pardosa_pseudoannulata_Blip
ATGTCCGGACCG--
GCCTACACCATACCTGACTCCGGCGGC---------GGTCCAACGGTC---AAGCTGACT
---------CCGCCGGCTAACGGCTACTCATCCGTCGAAGCAATGCGACGGTACATGGAG

---TCC
CTGCCGCAAGACCTC---TCCTTCAGGGAGGAACTGACAGACCAG---CATTTCTGGAAA
GCCGTGAGGACCGAATTTCTAGCAACTCTCCTGCTGACCGTCCTGGGATGTGGATCCTGC

CTGCGGTGGAAAGCACCTCCGACT------------------------------------
------------------------------GAC------CAGATGACCGCTTCGGCGGTC
GAACTCCGCGTGGCTCTCGCTTTCGGACTGGCCGTAACAACTCTGGTCCAGTGCATGGGA
CAAATTAGCGGAGGACATATGAATCCGGCAGTGACTGTCGCTCTTCTGGTCACCAGGCAC
ATAACTTTGACAAGAGGGGCGGCTTATGTCATGGCTCAGTGCTTGGGGGGCCTTGCTGGA
GCGGGAATACTGTACGGCCTGACCCCTCACGGCCAAAGGACAAGT---------------
---------------------------GCGGGCCTTGGGGCCACAGAGCCTGCGGTCGGG
---------GTGGCCGCCTCGGAGGCCTTCGGGATGGAATTCATGGCTTCGGTGGTGGTA
GTAATCACGGTGCTATCA---AACATGGATCCTCACAGG---------------------
---ACTGAAATG---------------GGATTTAAGGCACTCTCCATCGGATTCTCGTAC
GTGGTGGGACATCTGCTGGCTTTTCCATACACTGGAGCCAGCTTTAACCCTGCCAGATCC
TTCGGCCCTGCTATTGTGACCAAT---------ACATGGAGGTTACATTGGGTGTACTGG
GTAGCTCCCGTCCTCGGAGGCATAATTGGCGGAGTGACATACGATTACACACAAGAAACG
---AGGTTCGATCAACAAACA---------CTCAGAAGATCTTTC---AGGAAGAAA---
------------------------AATGGC---------CGACCAACCATAAACAGGGAC
ATCAGC---GCT---CTTTCAAATAACGAGACA---GAGTTCTCGTTAGACAACTGC---
------------CGGCTT--

>GIZV01004705_Schizocosa_ocreata_Blip
ATGGCCGGACCG--
GCCTACACCATACCGGAGTCCGGCGGC---------GGTCCAACGGTC---AAGCTCACC
---------CCGCCGGTGAACGGCTACTCGTCCGTCGAAGCGATGCGACGGTACATGGAG

---TCC
CTCCCGCAAGACCTC---TCCTTCAGGGAGGAGCTGACAGACCAG---CACTTCTGGAAA
GCCGTGAGGACCGAATTCCTAGCGACTCTCCTGCTGACCGTCCTGGGATGTGGATCCTGC
CTGCGGTGGAAAGCGCCCCCGACT------------------------------------
------------------------------GAC------CAGATGACCGCCTCGGCGGTC
GAACTCCGCGTGGCCCTCGCTTTCGGACTGGCCGTCACCACTCTGGTACAGTGCATGGGA
CAGATCAGCGGAGGGCACATGAATCCGGCAGTGACCGTCGCCCTGCTGGTCACGAGGCAC
ATAACTTTGACGAGAGGGGCGGCTTACGTCATGGCTCAGTGCTTGGGGGGCCTTGCCGGG
GCCGGCATACTGTACGGCCTGACCCCGCATGGCCAAAGGACAAGT---------------
---------------------------GCGGGCCTTGGGGCCACAGAGCCTGCGGTCGGG
---------GTGGCCGCCTCGGAGGCCTTCGGGATGGAATTCATGGCTTCGGTGGTGGTA
GTAATCACGGTGCTATCA---AACATGGATCCTCACAGG---------------------
---ACTGAAATG---------------GGATTTAAGGCACTCTCTATTGGATTTTCGTAC
GTGGTGGGACATCTACTGGCTTTTCCATATACTGGAGCCAGCTTTAACCCCGCCAGATCC
TTTGGACCAGCTATCGTGACCAAT---------ACATGGAGGTTACATTGGGTGTACTGG
GTAGCTCCAGTTCTCGGAGGCATAATTGGCGGAGTTACGTACGATTACACTCAAGAAACA
---AGGTTCGACCAACAGACG---------CTCAGAAGATCTTTC---AGGAAGAAA---
------------------------AATGGC---------CGACCAACCCTAAACAGGGAC
ATCAGC---GCC---CTCTCCAATAACGAGACA---GAGTTCTCGTTAGACAACTGT---
------------CGGCTT--


```
------------------------------------------------------------
------------------------------------------------------------
------------------------------------------------------------
------------------------------------------------------------
------------------------------------------------------------
------------------------------------------------------------
------------------------------------------------------------
------------------------------------------------------------
------------------------------------------------------------
------------------------------------------------------------
------------------------------------------------------------
------------------------------------------------------------
------------------------------------------------------------
------------------------------------------------------------
------------------------------------------------------------
------------------------------------------------------------
------------------------------------------------------------
------------------------------------------------------------
------------------------------------------------------------
------------------------------------------------------------
------------------------------------------------------------
------------------------------------------------------------
------------------------------------------------------------
------------------------------------------------------------
------------------------------------------------------------
------------------------------------------------------
>IAAA01035198_Parasteatoda_tepidariorum_Blip
ATGGCCAGTCCG------------------------------------------AACGAG
CTCTATTCCATCGGGGAGTCGGGATCT---------GGTAATCCGGTCAATAAACTGAAT
GGCCCACCCGGACCCACCAACGGTTACTCTCCCGTAGAAAACATGAAGCGTTACATGGAA
------------------------------------------------------------
---------------------------------------------------------TCT
TTGCCACAAGATTTT---ACATTCAAAGAAGAATTATGCAACCAA---CATTTCTGGAAG
GCGGTCAGAACTGAGTTTTTGGCCACACTTCTCTTGACCGTCCTGGGCTGTGGTTCGTGT
CTGAGATGGAAAGCTCCTCCAACT------------------------------------
------------------------------GAC------CAAATAACAGCCGCCGCGGTG
GAGCTACGCGTAGCACTCGCTTTCGGACTAGCAGTGACTACTCTGGTGCAGTGTGTTGGC
CAAGTAAGTGGTGGACACATGAATCCGGCAGTAACCATAGCCTTGCTGGTCACCAGGCAC
ATCAGTCTAACACGAAGTGCCGCGTATGTTGCGGCACAGTGTCTCGGTGGGCTGGCGGGT
GCCGGAATATTGTACGGCCTGACTCCACAAGGACAAAGACCGAAT---------------
---------------------------GCCGGACTGGGTGCCACTGAACCAGCTGTCGGA
---------GTAGCAGCATCCGAAGCATTCGGAATCGAATTTCTGGCCTCCGTCGTGGTG
GTCATCACGGTGCTTTCT---AACATGGACCCCCATAGG---------------------
---ACTGACATG---------------GGATTCAGAGCTCTCTCTATTGGATTCTCTTAC
GTCGTCGGTCATCTTCTTGCATTTCCTTATACTGGGGCCAGTTTCAACCCAGCTAGGTCT
TTTGGCCCAGCAATAGTGACTCAC---------ACATGGCGGCTACTTTGGGTATATTGT
GTGGGACCAGTTCTTGGTGGTATCATAGGTGTCGTCACTTACGATTACACTCAAGAAACT
---CACGCT---CAACAGGTT---------TTAAGGCGATCTTTT---AGAAAGAAA---
```

------------------------AATGGC---------AACACAGTAATAAACAGAGAT
CTAAGC---GCC---CTCTCCAATAATGAAACA---GAACTGACTCTGGACCACTGT---
------------CGGCTC--

>KFM80053_Stegodyphus_mimosarum_Blip
ATGGCTAATCCT--AATGAT
CTTTATTCCATCCCAGAGGCCACG------------GGACCGGCTGTC---AAACTGACC
AATTCTGCGGGTGCCTCTAACGGCTACTCACCGGTGGAAGCCATGCGGAGATATATGGAC

---TCT
TTACCGCAGGACTTT---ACTGTAAAGGAAGAACTGACAGACCAG---CATTTTTGGAAA
GCCGTGAGAACAGAATTCTTGGCTACCCTACTGCTGACCGTCCTGGGATGCGGGTCGTGT
CTGAGATGGAAGGCCCCACCAGCT------------------------------------
------------------------------GAT------CAAATGACAGCTTCGGCCGTC
GAACTGCGTGTGGCTCTTGCTTTCGGTCTCGCCGTGGCCACTCTAGTTCAGTGTGTAGGC
CAAGTTAGCGGGGGCCATATGAATCCAGCAGTGACTGTCGCTTTGCTTGTTACACGTCAC
ATCAGTTTGACCAGGAGCGCAGCTTATGTGACAGCACAGTGCCTGGGAGGCCTTGCTGGA
GCCGGGATCTTATATGGACTTACCCCGCACAGCCAAAGGCCCAGT---------------
---------------------------GCGGGACTTGGTGCCACAGAACCCGCAGTTGGG
---------GTTGCCGCGTCCGAAGCTTTTGGAATTGAGTTCCTTGCTTCAGTGGTAGTA
GTTATTACGGTGCTCGCT---AACATGGATCCACATAGA---------------------
---ACTGAGATG---------------GGTTTCAAAGCGTTGTCCATTGGTTTCTCTTAT
GTGGTGGGACATCTGCTGGCGTTTCCGTACACAGGAGCGAGCTTCAATCCAGCAAGATCA
TTTGGACCAGCTATCATTACGAAT---------ACGTGGAGACTGCATTGGGTGTACTGG
GTCGCTCCTGTATTAGGAGGTATCATTGGGGGTGTGACTTACGATTACACTCAAGAAACA
---CGATCA---CAGCAAAGC---------CTGAGACGTTCATTT---CGCAAGAAG---
------------------------AATGGC---------AGAATGAGCCTCAACAGAGAC
ATGAGC---GCC---CTCTCCAGTAATGAAACG---GAGCTAACCTTAGATCAGTGT---
------------AGACTT--

>KAG8191540_Oedothorax_gibbosus_Blip
ATG---TCCTCC--GACCCC
---TACTCCATACCGGAC---CCC---------------------------AAAGTC---
GGATCAGCC---------------------GGACTCGAAGCGATGCGGCGGTACATGGAC

---TCC
CTCCCCATGGACTAC---ACCCTTCGAGAGGAACTGACAGACCAG---AGATTCTGGAAA
GCCGTCAGAACAGAATTCCTGGCAACTTTGCTTCTGACCGTGCTGGGATGCGGGTCTTGC
ATACGTTTTAAGGCTCCGCCAGCC------------------------------------
------------------------------GAT------GTTGCAACCGCTGGAGCCGTA
GAATTGCGGGTCGCGCTGGCTTTCGGACTGGCTGTCGCGACTTTGGTCCAGTGCGTTGGA
CAAGTCAGTGGAGGACATATGAATCCGGCTGTGACTATTGCTCTGCTGGTGACGCGTCAC
ATATCGGCTGCCAGAACGGCGTGTTATGTAGCGGCGCAGTGTCTAGGTGGAATTGCTGGT
GCCGGAGTGCTGTACGGACTGACGCCTCCAGGACAGAGGCCCAGC---------------
---------------------------GCTGGACTGGGTGCCACGGAGCCGGCAGAAGGC
---------GTGGCAGCTTCTGAAGCATTCGGGTTCGAGTTTTTGGCATCTGTTGTGGTG
GTGATCACGGTGCTGTCG---AATATGGACCCGAGGAGG---------------------
---ACTGAGATG---------------GGGTGTGGAGCCTTGTCCATCGGGTTCTCGTAC
GTCGTGGGACACCTTTTAGCGTTTCCTTATACTGGTGCTAGTTTTAATCCTGCAAGATCA
TTTGGCCCGGCTATCGTCACAAAT---------ACGTGGAGACTTCATTGGATTTATTGG
GTGGCCCCCGTGCTAGGCGGGATCATAGGGGGCGTGACCTACGACTACACTCAAGAGTCG
---AGGTCG---CAGCCGACC---------TTGAGAAGGTCATTC---CGGAAAAGA---
------------------------CCACCA---------TCCGGCATCAACAGTCGCGAC
ATGAGC---GCC---CTTTCTAACAACGAGACG---GAGATGACAGTGGACCACTGC---
------------AGGCTG--

>IAST01018274_Octonoba_varians_Blip
ATG---TCCGCC--GACGGC
CTCTTTTGCATTCCGGACTCCGGC---------------------------AAG---TCC
GGCCGCCACCGCCG----------------CCCGTCGAGGCGATGCGTCGCTACATGGAA

---TCG
CTGCCCCAGGATCCGGCCCCGCTCCGGCAGGAGCTGAGCGACCGG---CGCTTCTGGGGC
GCAGTGCGCGCCGAGTTCCTCGCCACGCTGCTGCTCACAGTGCTGGGCTGCGGTGCTAGC
CTGCGCACCAAGGCGCCGCCCCCG------------------------------------
---------------------------------------CCGCCCGACGACGCCGCCGCC
CAGCTCAGGGCGGCCCTCGCCTTTGGCCTGGCCGTGGCTACGCTCGTCCGATGCCTGGGC
GGGGTGAGCGGGGCGCATATGAACCCGGCGGTCACCGCCGCCCTGTTAGTCACGAGGCAC
ATCGGGGCGGCAAGGGGCCTCGCCTACGTAGGCGCCCAGTGCGCGGGGGCGCTGGCCGGG
GCGGCCATCCTGTACGGGCTGGCGCCCCCCGCCTTGAGGGCCGAC---------------
---------------------------GCCGGGCTTGGAGCGACGGAGCCCCCGCCCTGG
---------GTGGGCGGCGGGGAGGCCTTCGGGCTGGAGTTCCTGGCCTCGGCTGTGGTA
GCCATCACTGTCCTGGCC---AACATGGAGCCCGGAAGG---------------------
---GAGCCTCTG---------------GGCACCGGGGCCCTATCCATAGGAATGGCGTAC
GTCGCGGGACACCTGCTTGCGTTTCCATATACGGGTGCCAGTTTCAACCCAGCACGATCC

TTCGGGCCGGCAGTCGTCACCAAC---------ACTTGGAGGCTGCATTGGGTGTACTGG
GTAGGCCCGGTTTTAGGTGGAATTCTGGGCGGAGTCACCTACGACTACACTCAAGAATCC
---CGATCC------CAGACG---------ATCCGACGATCTTTT---CGCAAGAGG---
------------------CGGAAGCCGCCCCCA------TCTACTGCCCTAAGTCGCGAC
GCCAGC---GCC---TTTTCCGGAAACGACACC---GAGATGACCCTGGACCACTGC---
------------AGGCTC--

>IAQA01013688_Octonoba_yesoensis_Blip
ATG---TCCGCC--GACGGC

CTCTTTTGCATTCCGGACTCCGGC---------------------------AAG---TCC
GGGCCGCCACCGCCG---------------CCCGTCGAGGCGATGCGTCGCTACATGGAA

---TCG
CTGCCCCAGGATCCGGCACCGCTCCGGCAGGAGCTCAGCGACCGG---CGCTTCTGGGGC
GCAGTGCGCGCTGAGTTCCTCGCCACGCTGCTACTCACAGTGCTGGGCTGCGGTGCTAGC
CTGCGCACCAAAGCGCCGCCCCCG------------------------------------
---------------------------------------CCGCCCGACGACGCCGCCGCC
CAAATCAGGGCGGCCCTCGCCTTCGGCCTGGCAGTGGCCACCCTCGTCCGATGCCTGGGC
GGGGTGAGCGGGGCTCATATGAACCCGGCGGTCACAGCCGCCCTGTTAGTCACGAGGCAC
ATCGGGGCGGCGAGGGGCGCCGCCTACGTGGGCGCCCAGTGCGCGGGTGCGCTGGCCGGG
GCGGCCATTCTGTACGGGCTGGCACCCCCCGCCTTGAGGGCCGAC---------------
---------------------------GCCGGGCTGGGGGCCACGGAGCCCCCTCCATGG
---------GTAGGCGGCGGGGAGGCCTTCGGGCTGGAGTTCCTGGCCTCGGCCGTGGTA
GCCATCACTGTCCTGGCC---AACATGGAGCCCGGGAGG---------------------
---GAGCCCCTG---------------GGCACGGGGGCCCTATCCATAGGAATGGCATAC
GTCGCGGGACACCTGCTTGCGTTTCCATACACTGGTGCCAGTTTCAACCCAGCACGATCC
TTCGGGCCGGCAGTCGTCACCAAC---------ACTTGGAGGCTGCATTGGGTGTACTGG
GTAGGTCCGGTTTTAGGTGGAATTCTGGGTGGAGTCACTTACGACTACACTCAAGAATCC
---CGCTCC------CAGACG---------ATCCGACGATCTTTT---CGCAAGAGG---
------------------CGGAAGCCGCCCCCA------ACCACTGCCCTGAGTCGCGAC
GCCAGC---GCC---TTTTCCGGAAACGACACC---GAGATGACCCTGGACCACTGC---
------------AGGCTC--

>AYEL01066910_01075601_Mesobuthus_martensii_Blip
ATGGAACGACCG--GAAAAT
TTCCGCGCCGCGGTGGGCGGCGGGCGCCACCGGCGTGGAGAAGGGCGCGGCCCCATTCTC
GGGATGGTCAAGCCGCAGTCCGTCCGGTCTCCGGTGGAGGCCGCCAGGCGATACGCCCAG

---TCG
TTGCCGGAGGACGTC---AGTTGCCGAGAAGAAGTGGGCAGCGGG---GCCTTCTGGAAG
GCGGTGGGTGCCGAATTTCTGGCCACTTGGCTGCTGACGGTGGTGGGCTGCGGTTCCTGC
CTGGACTGGCTG---TTGCCACCC------------------------------------
------------------------------GAA------TCGGCCCCGGAAGGTTGGCTC
GACTTGAAAGTGGGTCTGGCCTTTGGTCTGTCGGTCGCCACTCTAGTTCGATGCCTGGGT
CACGTCAGCGGTGCTCACATGAACCCGGCCGTCTCTCTGGCCTTCTTGGCCGTGGGCCAT
CTAACCACGGTCCGAGCGGTCGCCTACGTGCTAGCCCAGTGTCTGGGGTCGGCGGCCGGC
GCTTCGGTGGTATACGGCCTGACTCCGGCCCGACGGCGAGCGGCA---------------
---------------------------GCAGGCCTGGGGGCCACCCGTCCGGCCCGGGAG
---------CTGCACGTCAGTCAGGCCTTCGGCATGGAGTTCCTGGCCACTTTTCTAGTG
GTGCTCAGCGTGCTGGCC---AACGTGGATCCTCGCCGC---------------------
---ACCGATCCC---------------GGATCGGCAGCCGCTTCCATCGGACTGTCCTAC
CTAGTCGGACATCTACTGGCGTTTAGGTATACTGGAGCTAGCATCAATCCGGCCAGATCG
CTGGGACCGGCCTTGCTGACCAAC---------TGCTGGACGCATCATTGGGTATACTGG
GTGGGACCGGTGCTAGGAGGCGTGCTGGCTGCCATCACTTACGACTACACCCAAGACTCT
---TCCGAT---------CGGCGA------CCCAAGAGATCGTTC---CGCAAGAAA---
------------------------GCCACC---------AGAGAGGTT---AATCGAGAG
ATCAGT---GAC---CTCAGCCAC---CAGACG---GAAATGACTGCCACCTCCAAC---
---GATTAC---CGAATT--

>WUQG01017200_01062794_Androctonus_mauritanicus_Blip
ATGAGCAGACCA--GAAAAT
TTCCGCGCCGCGGTGGTCGGCGGGCGCCACCGGCGTGGAGAAGGGCGCGGTCCCATTCGT
AGGATGGCCAAGCCGCAGTCCGTCCGGTCTCCGGTGGAGGCCGCCAGGCGATACGCCCAG

---TCG
TTGCCAGAGGAGGTC---AGTTGCCGAGAAGAGGTGGGCAGTGGT---GCCTTCTGGAAG
GCGGTGGGTGCCGAATTTCTGGCTACCTGGCTGCTGACGGTGGTGGGCTGCGGTTCCTGC
CTAGATTGGCTG---TTGCCGCCC------------------------------------
------------------------------GAA------TCGGCCCCGGAAGGTTGGCTG
GACTTGAAAGTAGGTCTGGCCTTTGGTCTGTCAGTCGCCACTCTGGTTCGATGCCTGGGT
CACGTCAGCGGTGCTCACATGAACCCGGCCGTCTCTCTGGCCTTCTTGGCCGTGGGCCAT
CTAACC------------------------------CAGTGCCTGGGGTCGGCGGCCGGC
GCTTCAGTGGTATACGGCCTGACTCCGGCCCGACGGCGAGCGGCG---------------
----------------------------GCCGGCCTGGGGCCACCCGTCCGGCCCGGGAG
---------CTGCACGTCAGTCAGGCCTTCGGCATGGAGTTCCTGGCCACTTTTCTAGTG

GTGCTCAGCGTGCTTGCC---AACGTGGATCCCCGCCGC---------------------
---ACCGATCCC---------------GGATCGGCAGCCGCTTCCATCGGACTGTCCTAC
CTAGTCGGACATCTGCTGGCGTTTAGGTATACTGGAGCTAGCATCAATCCGGCCAGATCA
CTGGGACCGGCATTGCTGACCAAC---------TGCTGGACGCATCATTGGGTATACTGG
GTGGGACCGGTGCTAGGAGGCGTGCTGGCAGCCATCACTTACGACTACACCCAGGATTCT
---TCCGAT---------CGACGA------CCCAAGAGATCTTTC---CGCAAGAAA---
------------------------GCCACT---------AGAGAGGTC---AATCGAGAG
ATCAGC---GAC---CTCAGCCAC---CAGACG---GAAATGACGGCCGCCTCCAAC---
---GATTAT---CGAATC--


```
------------------------------------------------------
>AXZI01136975_AYEL01075601_Centruroides_exilicauda_Blip
ATGCCCAGA---------------------------------------------------
CTACAATCCATACCTGAAGGT---------------------------------TTGGCC
ACCGTCACCTCTAGTCATCAAGTGAGGTCACCGGTGGAGATCATGAGAAGATACATGGAA
------------------------------------------------------------
---------------------------------------------------------TCG
TTCCCCGAAGAAATA---AGTTGTCGAGAAGAAATGTCCACCTTT---CAATTCTGGAAG
GCTGTCAGAACCGAATTTCTAGCCACGTTGCTATTAACCGTTATGGGTTGTGGTTCCTGT
CTTGGATCTAAG---GTGCCACCCTCTGGA------------------------------
------------------------------GAAGAA---ATGGCCTACAATAGATTAGTA
GACATCAAAGTCAGTTTGGCTTTCGGATTCTCGGTGGCCACTTTGGTCCAATGCGTGGGC
CAGATCAGCGGGGCACACATGAATCCCGCTGTTTCCGTAGCCTTTCTTGTAGTCAGGCAC
ATTACCGCTGCTAGAGCGGTGGCTTATGTTCTGGCACAATGTTTGGGTGCTACTAGTGGT
GCTATTATTCTATATGGATTGACTCCTTTGGATCAAAGGGAAGCC---------------
---------------------------GCTGGTTTGGGTGCCACCGAACTCGCTGATGGC
---------GTACACGTGAGTCAGGCCTTCGGTATGGAATTTATGGCTTCTTTCTTGGTG
GTGCTCACTGTATTAGCA---AATGTAGATCCTCACAGA---------------------
---ACGGATATG---------------GGTTTCAGAGCTTTATCTATAGGACTTTCTTAC
GTAGTCGGACATTTTTTAGCGGTAAGGTATACTGGAGCTAGCATCAATCCGGCCAGATCG
CTGGGACCGGCCTTGCTGACCAAC---------TGCTGGACGCATCATTGGGTATACTGG
GTGGGACCGGTGCTAGGAGGCGTGCTGGCTGCCATCACTTACGACTACACCCAAGACTCT
---TCCGAT---------CGGCGA------CCCAAGAGATCGTTC---CGCAAGAAA---
------------------------GCCACC---------AGAGAGGTT---AATCGAGAG
ATCAGT---GAC---CTCAGCCAC---CAGACG---GAAATGACTGCCACCTCCAAC---
---GATTAC---CGAATT------------------------------------------
------------------------------------------------------------
------------------------------------------------------------
------------------------------------------------------------
------------------------------------------------------------
------------------------------------------------------------
------------------------------------------------------------
------------------------------------------------------------
------------------------------------------------------------
------------------------------------------------------------
------------------------------------------------------------
------------------------------------------------------------
------------------------------------------------------------
------------------------------------------------------------
------------------------------------------------------------
------------------------------------------------------------
------------------------------------------------------------
------------------------------------------------------------
------------------------------------------------------------
------------------------------------------------------------
------------------------------------------------------------
------------------------------------------------------------
------------------------------------------------------------
```


>XM_023372586_Centruroides_sculpturatus_Blip
ATGCCCAGA---
CTACAATCCATACCTGAAGGT---------------------------------TTGGCC
ACCGTCACCTCTAGTCATCAAGTGAGGTCACCGGTGGAGATCATGAGAAGATACATGGAA

---TCG
TTCCCCGAAGAAATA---AGTTGTCGAGAAGAAATGTCCACCTTT---CAATTCTGGAAG
GCTGTCAGAACCGAATTTCTAGCCACGTTGCTATTAACCGTTATGGGTTGTGGTTCCTGT
CTTGGATCTAAG---GTGCCACCCTCTGGA------------------------------
------------------------------GAAGAA---ATGGCCTACAATAGATTAGTA
GACATCAAAGTCAGTTTGGCTTTCGGATTCTCGGTGGCCACTTTGGTCCAATGCGTGGGC
CAGATCAGCGGGGCACACATGAATCCCGCTGTTTCCGTAGCCTTTCTTGTAGTCAGGCAC
ATTACCGCTGCTAGAGCGGTGGCTTATGTTCTGGCACAATGTTTGGGTGCTACTAGTGGT
GCTATTATTCTATATGGATTGACTCCTTTGGATCAAAGGGAAGCC---------------
---------------------------GCTGGTTTGGGTGCCACCGAACTCGCTGATGGC
---------GTACACGTGAGTCAGGCCTTCGGTATGGAATTTATGGCTTCTTTCTTGGTG
GTGCTCACTGTATTAGCA---AATGTAGATCCTCACAGA---------------------
---ACGGATATG---------------GGTTTCAGAGCTTTATCTATAGGACTTTCTTAC
GTAGTCGGACATTTTTTAGCGTTTAGGTACACCGGTGCGAGTATTAATCCAGCCAGATCG
TTAGGACCAGCTGTCGTTACTAAT---------TCCTGGAGACATCACTGGGTGTATTGG
GTAGGTCCTGTTTTAGGGGGTGTATTAGGAGCCATCACCTATGATTACACGCAAGACACC
---TCACGAACC---AGTCAGCAC------TTCAAGAGGTCCTTC---CGTAAAAGG---
------------------------TCTACT---------AGAGATATA---ATTCGGGAT
CAAAGC---GCA---CTTAGCAAT---GAAACC---GAAATGACAGGTACTTCCAAC---
---GACTTC---CGAATT--

>GEYB01061678_Hermannia_gibba_Blip
ATGGAAGAG---CAAGAA
GACAAC---AAT
ACTAATAATGTGAACGACAAAAGTCGTTCACCAGTGGAAGCCATGAGAAGATATATTGAG

---TCT
TTGCCCGAAGAAATC---AGTTTCAAGGAAGAAATATCTAGTTAT---CAGTTTTGGAAA
GCAGTCAGAACTGAATTCTTGGCCACTCTGTTATTCATTGTGTTTAGTTGTGGTTCATGT
TTGGGCTTTGCATCACTTCCTCCTAAACTATTTATTAAGTCTTCAAACAATACATTC---
---AATACT---------AATCAAAGTAAAGATGATATCAGTTATGAAACTATAGTTTTG
GAAATGAAAGTATCGCTAGTGTTCGGTCTGACTTCAGCGACTCTAATCCAATGTATGGGT
CACGTAAGCGGCTGTCATCTGACGCCAGCAATTACTCTATCACTTCTTGTCACTCGACGT
CTGACAATATTGCGCTGTTTTATGTACATATTTGCTCAGTGTTTTGCATCAATACTTGCC

TGTGGTATACTCTATGGTCTGTTAGACACAGAAGAGCGACAGTTT---------------
---------------------------TCTGGTCTCGGCCTAACTCTGCCGAGCGATCGT
---------TTGAATGCATCCCAAGCTTTTGGTTATGAATTTTTAGCCACTTTTATAGTG
GTTTTGGCTTATTTAGTC---AATTCTGATACACAGAGA---------------------
---ACTGATGCT---------------GGATTCAAGTCTCTATCTATAGGTTTTGCATAT
TCAGTCGCACATCTATTTGCGTTTCGACATACAGGAGCCTCACTTTCACCCGCGAGGTCT
CTCGGACCTGCAGTTATCAAAGGC---------ATTTGGGATCATCATTGGATCTATTGG
ATCGCACCGTTAATAGCAGGCATGTTGAGTGGTTTCACTTATGAATATATTAGATCAACC
---AATTCTAATGAATGTCAGACA------TTGAAGAGATCATTC---AGACGCAAA---
------------------------CATATT---------AAAGGTGTC---AAACGTGAC
GCCAGCAGTAAT---CTAAGTAACTATGAAACC---GAATTTACCATCACATCCACT---
---GAGTTT---

>GEYJ01056692_Nothrus_palustris_Blip
ATGGAACAA---CAAGAA
GAAAAC---AAT
GTCAATAATGTGGCTGCGAAAAGTCGTTCGCCCGTCGAAACAATGCGAAGATATATTGAA

---TCT
TTACCTGAAGAGATC---TCAGTTCGAGAAGAAATAACTAGTTAT---CAATTTTGGAAA
GCAGTCCGGACTGAATTTCTGGCCACTTTGCTATTCATTGTATTCGGTTGTGGTTCATGC
CTTAGCTATGCATCCCTTCCACCAAAAAAGTCAATTTGGTCTTCAAATACATCATCCATT
GATAATTCATTGCATAAAAATCAAAGTATAGATGATTATAATAATGACATTGTTATCTTA
GAAATAAAAGTATCGCTTGTTTTTGGTCTGACATCAGCGACTTTAATTCAATGTATGGGT
CAAGTGAGTGGATGTCATTTGACACCAGCTATTACTCTATCACTTCTGGTTACTCGACGT
TTGACAATATTGCGGTGTTGTTTGTACATATTTGCTCAGTGTTTTGCATCAATAGTCGCG
TGCGGTATTTTATTTGGTTTATTAAACACTGAAGAGCGACTTCAT---------------
---------------------------TCAGGGCTCGGCCTAACCTTACCAAATCACCAT
---------TTAAATGCATCTCAAGCTTTTGGCTATGAATTTTTAGCAACTTTTATAGTA
GTATTGGCTTACTTAGTA---AATTCTGATCCTCAGAGA---------------------
---GCTGACGCT---------------GGTTTTAAATCTTTATCTATAGGATTCGCATAT
TCGATCGCACATCTATTTTCGTTTCGCCATACAGGAGCCTCTCTTTCTCCCGCCAGATCT
CTCGGACCGGCAGTAATAAAAGGC---------ATTTGGGATTACCAATGGGTCTATTGG
GTATCACCTATGATAGCAGGCATGATAAGTGGTTTTACATATGAATACATTAGATCGACG
---ACATCTTCTGAATGCCAAACA------TTAAAGCGATCCTTT---AGAAGAAGA---
------------------------CCGAGT---------CAGGCCGTC---AAACGTGAC
GCGAGCAGCAAC---TTAAGCAACTATGAAACT---GAATTTACCATCACTTCAACT---
---GAATGTCCTCGATAT--

>GEYP01034974_Hypochthonius_rufulus_Blip
ATGGAACAA---CAGGAA
AACAAT---ACT
AATGCCACTGCTGCAGCAAAGAATCACTCACCCATTGAAGCGATGCGACGTTACATTGAA

---TCA
TTACCCGAGGATATT---AGTTTTAAGGAAGAGATTACGGATTAT---CAATTTTGGAAA
GCGGTGAGAACTGAGTTTTTAGCTACGATATTGTTAATTGTATTTGGTTGTGGCTCATGC
CTTTCGAGTATATCATTACCACCGAAGTTAACCGAAGCT------AACTTTACC---ATT
ACTAATCATTTCCAAACAAATCAATTAGTAGAG------TCC---GATTTAATTATCTTA
GAAATTAAAGTATCACTTGTGTTTGGTCTGACTGTGGCTACACTCGTCCAGTGCATGGGA
CAGGTGAGCGGGTGTCACATGAATCCGGCCGTAACAGTGTCGCTTTTAGTTACTAAACGA
TTGACTTTATTGCGATGTTTTATGTATATATTTGCTCAATGTATGGGATCAATTGTAGCG
TGTGGAATAATATACGGTTTACTCAACAATGAACAAAGACAATCT---------------
---------------------------TCAAGAATGGGGCTCACCCTACCATTAGATACT
---------CTTAACTCCAGTCAAGCGTTTGGTTATGAATTTTTAGGAACTTTCATAATT
GTGCTGTCGTTTTTGGTC---AATACCGACACTCAAAGA---------------------
---TCCGACACT---------------GGATTTAAGGCTTTGTCTATCGGATTCACTTAT
ACCGTCGCTCATTTATTTGCTTTTCGTCATACCGGAGCGGGTCTTAATCCGGTGCGAACA
TTTGGTCCTGCATTATTTAATGGC---------ATTTGGGATTATCAATGGCTCTATTGG
GTCGGACCATTAATAGCGGGTATGTTAAGCGGTTTCACGTATGAATACATTCGTTCGATA
---TCATCAAATGAATGTCAATCA------TTGAAACGGTCATTC---CGGCGAAGG---
------------------------CCGAGT---------AAAGGCTTA---AAACGTGAT
GCCAGCAGTGGG---ATTAGCAATAATGAGACT---GAATTCACCATCACATCGACT---
---GAATTCCCACGGTAC--

>GIFZ01009794_Dermanyssus_gallinae_Blip
ATG---
TGCTCGATTATTCACCACAGTAAT------------CCGGCGACGGGG---GCGGGTGGC
GGGGGACCAACGCCAGCGGCGGTAAAATCGCCCGTCGAGGAGATGCTCCGCTATATCGAC

---TCG
CTTCCGGAGGACCCGCCGAAAGCGTGCGTGGAGGTTCGTACGCGA---AAGTTCTGGATG
GCCATTTGGGCCGAGTTCTTGGCCACCTTGCTGCTAACCGTCGTGACG------------

---------------------------------------AGCCGATCGCGAGAGGACTCG

GATATTAGACAGAGTCTGGCGGTTGGCGCCAGCGCTGCCCTGCTC---TCGTAC------
ACGCTGCACGGGGTGGCCTTCAATCCGGCGGTGACGCTCTCGCGATGTCTCGTCGGGCAC
GCGTCACTGTTGAAGCTGTTCGCCAGCGTGCTCGTTCAGACGCTCGGCGCGGTCGCGGGC
ACACTCGTGTCGCTGGCACTTCAC---ACCAGCAATCGCTACCAG---------------
-----------------------------------CCC---ACCGTGTACCCCGGC---
---------CTCAACCTTAGCCAGATTTTCGGCGTCGAGTTCTTGGGCTCGCTTGTGGTC
GTCCTCACGGTGCTTACG---TCGGACGACGACGTCGAAAGC------------------
---GGTCTACTT---------------ACGGTGCAGGCGTTTCCGGTCGGCGTCGCTTAT
GGGGCCGCCACACTGTTTTCGTTTTCTCACACGGGTGTAGGACTAAATCCGGCTCGTGCT
CTGGGCCCAGCACTTCTCGCCAAC---------GAGTGGACCTATCTTTGGGTGTACTGG
ATCGGCCCTCTTCTCGGCGGCCTGATAGGGGGCTTTACGTATGATTACGCACGTTCGTCG
CGGAAGGACGTGCAGGTTCGTGCC------GAACGTCGAAGCTTA---CGCCAGCGC---
------------GCGCAGGACAAGGATCGACAG------GACGGAATT---CGACGGGAT
CTCAGC---GCT---CTGTCGCGG---GACACC---GAGCTTACCAACTCGCTGAACACG
AACGACTGT---CGAGTG--

>GHIT01009555_Amblyseius_swirskii_Blip
ATG---
TGCTCAGTGGTC--
AACAACAACACGGCATCGGCCGTGAAGTCTCCGGTGGAGGAGATGCTTCGATATATCGAC

---TCG
CTACCTGAAGAACCACCGAAACCACGTGATGAAATAAAGTCGCGA---TATTTCTGGATG
TCGGTCTGGTCAGAGTTCGTGTCAACCCTCATCCTCACTGTGATTGCC------------

---------------------------------------AGCGCTTCGACACAAGAATCC
GATGTGCGACAAAGTCTAGCCGTAGGATGCACCACCGCTCTGTTG---ACGTAT------
ACCCTGCACGGAGTCGCTTTCAACCCGGCAGTGGTCCTTGCACGTTTTCTGGTGGCCAAG
GTGTCGCTGCTGAAATTCTTCTCGTCGATATTCGTTCAAGCCATCGGTGCTATCGCGGGC
AGCGTCCTGGTGTTTGCTCTGCGT---AACGAGTACTATCAA------------------
--CTGGTCGAGTCCAAA---
---------CTCAACATGAGCCAGATATTTGGGGTTGAGTTCTTAGGCTCACTCGTGATC
GTTCTGACGGTCTTGACA---TCGGATGACGATGTCGAAACG------------------
---GGACTCTTG---------------AAGTTCCAAGCCTTGCCGGTCGGAGTAGCATAT
GGAGCTGCGACATTGTTTTCGTTTTCGTACACGGGCATTGGCCTTAACCCCGCTCGCGCT
CTGGGACCAGCGATTCTGACACAC---------GAATGGGATAACCACTGGGTGTACTGG
GTTGGTCCCCTACTTGGAGGCCTCATCGGCGGCTTCACGTACGATTACGCCAGGTCGTCC
CGCAAGGATGTTCAGGAACGGGCT------GAGAAG---AACCAT---CGGAAA------
---------------------GCCATCGCGGAG------TCA---CTG---AAGCGCGAC
TTTAGC---GCC---TTCTCCGGC---GAAACC---GAACTCACCAACTCCATGAATACC
AACGATTGT---CGGGTG--

>XM_003747175_Metaseiulus_occidentalis_Blip
ATG---
TGTTCAGTGGTC--
AACAATAACACAGCGTCA---GTGAAATCTCCCGTAGAAGAAATGCTTCGCTACATAGAC

---TCT
CTGCCAGAGGAACCGCCGAAACCGCGCGATGAAATAAAATCGCGG---CGCTTCTGGATG
TCGGTGTGGTCCGAGTTCATCTCTACCTTGATTCTTACAGTGATCGCA------------

---------------------------------------AGTGCCTCATCGCAAGAGTCC
GATGTACGGCAGAGTCTAGCGGTCGGTTGCACCAGTGCCCTCTTG---ACGTAT------
ACCCTCCACGGAGTCGCCTTCAATCCGGCCGTTGTCCTCGCCCGCTTCCTCGTGGCCAAA
GTATCGTTTCTGAAGTTTTTCGCCTCTGTACTCGTACAGGCTGTGGGTGCTATCGCAGGC
AGCGTCTTGGTTTTTGCTCTGCGT---AACGAGTTCTTCCAA------------------
--TTAGTGGAGTCCAAG---
---------CTCAACCTGAGCCAAATCTTCGGAGTAGAGTTCTTGGGCTCACTCGTGATC
GTTCTGACAGTTCTCACG---TCAGACGACGACGTCGAGACC------------------
---GGCTTGTTG---------------AAATTCCAAGCGTTCCCCGTCGGAATTGCTTAT
GGTGCGGCTACGCTATTCTCGTTCTCGTACACGGGAATCGGCCTCAATCCCGCCCGAGCT
CTCGGACCCGCGATACTCACTCAC---------GAATGGGATCATCACTGGGTGTATTGG
GTCGGACCACTGCTCGGAGGTCTCATCGGAGGATTCACTTACGATTACGCGAGATCGTCC
CGAAAGGACGTTCAGGAGCGCGCC------GAGAAA---ATCCAC---CGCAAG------
---------------------GCGATGGCTGAG------TCC---CTG---AAACGAGAT
TTCAGC---GCC---CTCTCCGGA---GAAACC---GAACTGACCAACTCGATGAATACG

AATGATTGT---CGAGTG--

>MNPL01028127_Tropilaelaps_mercedesae_Blip
ATG---
TGCTCAATTGTC--
CACAATAACAATCCGGCTGCGGTGAAATCGCCGGTCGAGGAGATGCTTCGCTACATCGAC

---TCA
CTTCCGGAGGACCCGCCAAAAGCCAGAGTGGAAATTCGAACGCGA---AAATTTTGGACA

```
GCAATTTGGGCGGAATTTTTGGCCACGTTGCTACTCATCGTCGTAACG------------
------------------------------------------------------------
---------------------------------------AGCGCGTCCAAAACTGACTCG
GACGTCCGGCAAAGTCTGGCGGTTGGTGCAAGCACCGCCCTCCTT---ACCTAC------
ATCCTCCACGGGGTGGCATTCAATCCGGCGGTGACCCTATCGCGTTGTCTCGTGGGTCAT
GCATCGCTCCTTAAGCTATTCGCTAGCGTCTTGGTGCAGGCACTAAGCGCTGTTCTTGGC
ACCCTCCTTATCATCGCTTTACGA---AGTGAGCATCATCAGCCA---------------
------------------------------------------TCG---GAACCCAAG---
---------CTCAACCTCAGCCAAATATTCGGTGTCGAGTTTCTCGGATCTCTGGTTGTC
GTCCTCACCGTGCTCACC---TCTGATGACGATGTCGAAAGC------------------
---GGGTTACTT---------------AGGTTTCAAGCCTTTCCGGTGGGAATCGCGTAT
GGAGCTGCTACACTTTTTTCGTTTTCGCACACTGGCGTAGGATTGAACCCTGCAAGGGCT
TTGGGACCGGCCCTAATAGCTAGA---------GACTGGACGCATCACTGGGTGTACTGG
ATTGGACCCCTTCTTGGTGGTCTCATCGGGGGCTTTACATATGATTACGCTCGCTCGTCG
AGAAAGGACGTCCAGGAACGCGCT------GAGAGGGGTTTGCGT---CAACGTGAA---
---------------------CAGGATCGGGAA------CGGGCATTA---CGACGGGAT
CTTAGC---GCT---CTTTCGCAG---GACACA---GAGATGACGAATTCCCTAAATCAA
AACGACTGT---CGGGTT------------------------------------------
------------------------------------------------------------
------------------------------------------------------------
------------------------------------------------------------
------------------------------------------------------------
------------------------------------------------------------
------------------------------------------------------------
------------------------------------------------------------
------------------------------------------------------------
------------------------------------------------------------
------------------------------------------------------------
------------------------------------------------------------
------------------------------------------------------------
------------------------------------------------------------
------------------------------------------------------------
------------------------------------------------------------
------------------------------------------------------------
------------------------------------------------------------
------------------------------------------------------------
------------------------------------------------------------
------------------------------------------------------------
------------------------------------------------------------
------------------------------------------------------------
------------------------------------------------------------
------------------------------------------------------------
------------------------------------------------------------
------------------------------------------------------------
------------------------------------------------------------
------------------------------------------------------------
------------------------------------------------------------
------------------------------------------------------------
```


>XP_022646344_Varroa_destructor_Blip
ATG---
TGTTCGATTGTC--
CGTAATAACGGCCCAACCGCTGTGAAGTCGCCCGTGGAAGAGATGCTACGCTATATCGAT

---TCT
CTCCCGGATGATCCGCCAAAAGCGAAAGTAGAAATTCGCACACGA---AAGTTTTGGGCG
GCAGTTTGGTCGGAATTTCTAGCTACACTTCTATTAATTGTTGTGACC------------

---------------------------------------AGCCCATCAACAGGTGAAGTG
GATGTTCGACAAAGTTTGGCAGTCGGGGCAAGCGCTGCCCTGCTC---ACTTAC------
ACGCTTCAAGGAGCAGCTTTTAATCCAGCTGTCAGCATCCCGCGATGCCTGGTCGGTCGT
TCATCACTTCTTAAATTGTTTGCTAGCGTCCTGGCACAGGCGTTTGGCGCCGTATTAGGT
AGCTTGTTAGTCTTCGCTCTTCAA---AGCGAAGGAAATCAGCCC---------------
--TTAATAGAAACGAAA---
---------CTACATCTTAGCCAGGTATTTGCTGTTGAGTTCCTTGGATCATTTGTAGTG
GTCCTCACCGTTCTCACC---TCTGATGACGATGTTGAGAGT------------------
---GGTTTAATG---------------AAGTTCCAAGCGTTTCCGGTCGGACTTGCGTAC
GGGGCCGCTACACTTTTTTCGTTTTCGCACACTGGCGTGGGATTGAACCCTGTAAGAGCT
TTAGGGCAGGTTTTAGTAGCGAGA---------AACTGGACCAATCATTGGGTGTACTGG
ATTGGGCCTTTGCTTGGTGGACTCATCGGAGGTTTTACGTATGATTACGCCCGGTCTTCT
AGAAAGGACGTACAGGAACAAGCC------GAAAGGACGTCGCGC---CAGCGAGAG---
---------------------TTGGACCGCGAG------AGAGGACTT---CGACGAGGT
CTGAGT---GCC---CTCTCGCAG---GATACA---GAAATGACGAACTCCTTAAATACC
AATGATTGC---CGAGTT--

>XM_022838439_Varroa_jacobsoni_Blip
ATG---
TGTTCGATTGTC--
CGTAATAACGGCCCAACCGCTGTGAAGTCGCCCGTGGAAGAGATGCTACGCTATATCGAT

---TCT
CTCCCGGATGATCCGCCAAAAGCGAAAGTAGAAATTCGCACACGA---AAGTTTTGGGCG
GCAGTTTGGTCGGAATTTCTAGCTACACTTCTACTAATTGTTGTGACC------------

---------------------------------------AGCCCATCAACAGGTGAAGTG
GATGTTCGACAAAGTTTGGCAGTCGGGGCAAGCGCTGCCCTGCTC---ACTTAC------
ACGCTTCAAGGAGCAGCTTTTAATCCAGCTGTCAGCATCCCGCGATGCCTGGTCGGTCGT
TCATCACTTCTTAAATTGTTTGCTAGCGTCCTGGCACAGGCGTTTGGCGCCGTATTAGGT
AGCTTGTTAGTCTTCGCTCTTCAA---AGCGAAGGAAATCAGCCC---------------
--TTAATAGAAACGAAA---
---------CTACATCTTAGCCAGGTATTTGCTGTTGAGTTCCTTGGATCATTTGTAGTG
GTCCTCACCGTTCTCACC---TCTGATGACGATGTTGAGAGT------------------
---GGTTTAATG---------------AAGTTCCAAGCGTTTCCGGTCGGACTTGCGTAC
GGGGCTGCTACACTTTTTTCGTTTTCGCACACTGGCGTGGGATTGAACCCTGTAAGAGCT
TTAGGGCAGGTTTTAGTAGCGAGA---------AACTGGACCAATCATTGGGTGTACTGG
ATTGGGCCTTTGCTTGGTGGACTCATCGGAGGTTTTACGTATGATTACGCCCGGTCTTCT

AGAAAGGACGTACAGGAACAAGCC------GAAAGGACGTCGCGC---CAGCGAGAG---
---------------------TTGGACCGCGAG------AGAGGACTT---CGACGAGGT
CTGAGT---GCC---CTCTCGCAG---GATACA---GAAATGACGAACTCCTTAAATACC
AATGATTGC---CGAGTT--

>JAEQBA010002393_BA010002712_Phalangium_opilio_Blip
ATGCCGGAG---

---------TCACCATTGACAGCTACGTCACCCGTGGCCGTCATGAGACGATACATTGAC

```
------------------------------------------------------------
---------------------------------------------------------TCC
TTACCTCCCGAAATG---ACCTTTCGCGAAGAGGTGACCTCTACA---AAGTTCTGGAAA
GCGGTCCGAACTGAATTGTTGGCGACATTGTTACTAACCATCTTTGGTTGCGGTTCGTGT
TTGACACTGAAA---CTACCACCTACCGGT------------------------------
------------------------------GACGAG---TCGGTG---ATGTTAGCCATA
GAAATTAAAGTTGCTTTAGCGTTCGGGGTCACCGTGTCCACTTTGGTCCAGTGTTTCGGA
CCAGTTAGCGGTGCCCATATGAACCCAGCTATCTCCGTTGCCTGTTTAGTCACCAGGCAT
ATTAGCGCTATTAGAGCGGCTTCGTATATTTTAGCCCAATATTTGGGTGGATTGTCTGGT
GCCGCTATTCTGTACGGGTTGACCCCGGAAAATTTGAGGTCAACC---------------
---------------------------GCGGGACTGGGTGCAACCGAACCCAGTTTGGAC
---------TTGCATCTTAGTCAAGCTTTTGGTTTGGAGTTCATGGGGACAGTCGTGATT
GTTTTGACGGTTTTGGCC---AATATGGACCCTAGAAGG---------------------
---ACTGATTTG---------------GGTTTCAGGGCTTTGTCTATAGGATTGTCCTAT
ACGGCGGCTCATTTACTAACTTTTCGATACACGGGTGCTGGTTTGAATCCGGCTCGTTCA
TTAGGACCGGCTGTGGTCACTAAT---------TCCTGGCGATGTCATTGGGTGTACTGG
ATCGGTCCTCTCTTGGGAGGTTTCATCGGGGGCTTCACTTACGAGTACAGCAGCGACTCA
---TCTTCGGTG---GCGACGGGT------CTTCGGCGCTCCTTC---CGCCGGAAG---
---------------AACCCATCTCATCGAGGTCCCGGAGGCGAAATG---ATCCGTGAT
CACTCTGGGGCC---CTGAGTAGCAACGAGACT---GAGATGACTGCCGCTTCGAACGCC
AATGACTAC---GGCGTT------------------------------------------
------------------------------------------------------------
------------------------------------------------------------
------------------------------------------------------------
------------------------------------------------------------
------------------------------------------------------------
------------------------------------------------------------
------------------------------------------------------------
------------------------------------------------------------
------------------------------------------------------------
------------------------------------------------------------
------------------------------------------------------------
------------------------------------------------------------
------------------------------------------------------------
------------------------------------------------------------
------------------------------------------------------------
------------------------------------------------------------
------------------------------------------------------------
------------------------------------------------------------
------------------------------------------------------------
------------------------------------------------------------
------------------------------------------------------------
------------------------------------------------------------
------------------------------------------------------------
------------------------------------------------------------
------------------------------------------------------------
------------------------------------------------------------
------------------------------------------------------------
------------------------------------------------------------
```


>JAALXS010000006_Tachypleus_gigas_Blip1
ATGGCATATCCA--TCGGAT
ACTAGCCTAGACTACGTGACGCAAACTGTAAAC------CCAACA---CGGTCATTAATG
ATGGGTCCTGTGGCTAACCCTAGTCAGTCGGACGTGGAAGTTATGCGGCAGTATATTGAG

---TCT
CTGCCACAGGACCTG---AGTTATCGTGAGGAACTGATGAGTGCT---CAGCTATGGAAG
TCAGTGAAAGGTGAGCTCCTGGGAACATTATTGTTGACTGTGGTGGGCTGTGGGGCTACT
CTGTCTTTTCAG------CCAGGC------------------------------------
---------------------------------------TCGGCTGCTACACATGCTGTC
ACGCTTCAAGTATCTCTGGCATTCGGACTGGTCATAGCTTCACTAGTCCAGTGGACTGGG
CCAGTCAGTGGCGCCCATATGAACCCATCGGTATCTTTAGCATTACTAGTGACCCACCAC
ATTAGCATGCTTCGTGCAGGACTCTACATACTGGCCCAGTGTGTTGGAGCTCTGGTTGGA
GCTGCTGTATTGTACGGTCTCACACCGTCAGAGCAGCGTGCTTTG---------------
---------------------------GTAAGTCTTGGAGCGTGCGAGCCATCTGAACAT
---------CTCCACCCAAGTCAAGCCTTTGGGTTAGAGTTTCTTGGGACCCTAGTAGTG
ATATTGACCATCTTATCC---AACGCTGACCCGGCCAGA---------------------
---ACAAACTCC---------------GGATCAAAGGCATTGTCCGTAGGATTATCTTAC
ACCGCTGCCCATTTGTTGGCATTTCCTTACACTGGTGCAGGACTTAATCCAGCTCGTGCT
TTCGGACCTGCTGTTGTGGCCGGT---------AGATGGCGTCTTCACTGGGTATACTGG
GTAGGCCCGATATTGGGGGGCATCATCGGTGGCTTCACGTACGAGTATAGTCAAGTTACA
---TCACAATCCACTCAGCAA---------CTGAAGCGTTCTTTC---CGCCGGAAG---
------------------------TCAGCC---------AGGGAAATG---ATGCGTGAT
CACAGC---GCC---CTCAGTAACAATGAGACA---GTGTTCACGGCTTTGTCCAAC---
---GACTGT---CGAGAA--

>RXZF01138922_Tachypleus_tridentatus_Blip1
ATGGCATATCCA--TCGGAT
ACTAGCCTAGACTACGTGACGCAAACCGTGAAC------CCAACA---CGGTCATTAATG
ATGGGTCCTGTGGCTAACCCTAGTCAGTCGGACGTGGAAGTTATGCGGCAGTATATTGAG

---TCT
CTGCCACAGGACCTG---AGTTATCGTGAGGAACTGATGAGTGCT---CAGCTATGGAAG
TCAGTGAAAGGTGAGCTCCTGGGAACATTACTATTGACTGTGGTGGGCTGCGGGGCTACT
CTGTCTTTTCAG------CCAGGC------------------------------------
---------------------------------------TCGGCTGATACACATGCTGTC
ACGCTTCAAGTATCTCTGGCATTTGGATTGGTCATAGCTTCACTAGTCCAGTGGATTGGG
CCAGTCAGTGGCGCCCATATGAACCCATCGGTATCTTTAGCATTACTAGTGACCCACCAC
ATTAGCATGCTTCGTGCAGGTCTTTACATACTGGCCCAGTGTGTTGGAGCTCTGGTTGGA
GCCGCTGTATTGTACGGTCTCACACCGTCAGAGCAGCGTGCTTTG---------------
---------------------------GTAAGTCTTGGAGCGTGCGAGCCATCTGAACAT
---------CTCCATCCAAGTCAAGCCTTTGGGTTAGAGTTTCTTGGGACCCTAGTAGTG
ATATTGACCATCTTGTCC---AACGCTGACCCGGCCAGA---------------------
---ACAAACTCC---------------GGATCAAAGGCATTGTCCGTAGGATTATCTTAT

ACCGCTGCCCATTTGTTGGCATTTCCTTACACTGGTGCAGGACTTAATCCAGCTCGTGCT
TTCGGACCTGCTGTTGTGGCCGGT---------AGATGGCGTCTTCACTGGGTATACTGG
GTAGGCCCGATATTGGGGGGTATCATCGGTGGCTTCACGTACGAGTATAGTCAAGTTACA
---TCACAATCCACTCAGCAA---------CTGAAGCGTTCTTTC---CGCCGGAAG---
------------------------TCAGCC---------AGGGAAATG---ATGCGTGAT
CACAGC---GCC---CTCAGTAACAATGAGACA---GTGTTCACGGCTTTATCCAAC---
---GACTGT---CGAGAA--

>WCHO01004207_Carcinoscorpius_rotundicauda_Blip1

```
ATGGCATATCCA------------------------------------------TCGGAT
ACTAGCCTAGATTACGTGACGCAAACCGTGAAC------CCAACA---CGGTCATTAATG
ATGGGTCCTGTGGCTAACCCTAGTCAGTCGGACGTGGAAGTTATGCGGCAGTATATTGAG
------------------------------------------------------------
---------------------------------------------------------TCT
CTGCCACAGAACCTG---AGTTATCGTGAGGAACTGATGAGTGCT---CAGCTGTGGAAG
TCAGTGAAAGGTGAGCTCCTGGGAACATTACTATTGACTGTGGTGGCTGCGGGGCTACT
CTGTCTTTTCAG------CCAGGC------------------------------------
---------------------------------------TCGGCTGCTACACATGCTGTC
ACGCTTCAAGTATCTCTGGCATTCGGATTGGTAATAGCTTCACTAGTCCAGTGGACTGGG
CCAGTCAGTGGCGCCCATATGAATCCATCGGTATCTTTAGCATTACTAGTGACCCACCAC
ATTAGCATGCTTCGTGCAGGCCTTTACATACTGGCCCAGTGTGTTGGAGCTCTGGTTGGG
GCCGCTGTATTGTACGGTCTCACTCCGTCAGAGCAGCGTGCTTTG---------------
---------------------------GTAAGTCTTGGAGCGTGCGAGCCATCTGAACAT
---------CTCCACCCAAGTCAAGCCTTTGGGTTAGAGTTTCTTGGGACCCTAGTAGTG
ATATTGACCATCTTGTCC---AACGCTGACCCGGCCAGA---------------------
---ACAAACTCC---------------GGATCAAAGGCATTGTCCGTAGGATTATCTTAC
ACCGCTGCCCATTTGTTGGCATTTCCTTACACTGGTGCAGGACTTAATCCAGCTCGTGCT
TTCGGACCTGCTGTTGTGGCCGGT---------AGATGGCGTCTTCACTGGGTATACTGG
GTGGGCCCGATATTGGGGGGCATCATCGGTGGCTTCACGTACGAGTATAGTCAAGTTACA
---TCACAATCCACTCAGCAA---------CTGAAGCGTTCTTTC---CGCCGGAAG---
------------------------TCAGCC---------AGGGAAATG---ATGCGTGAT
CACAGC---GCC---CTCAGTAACAATGAGACA---GTGTTCACGGCTTTATCCAAC---
---GACTGT---CGAGAA------------------------------------------
------------------------------------------------------------
------------------------------------------------------------
------------------------------------------------------------
------------------------------------------------------------
------------------------------------------------------------
------------------------------------------------------------
------------------------------------------------------------
------------------------------------------------------------
------------------------------------------------------------
------------------------------------------------------------
------------------------------------------------------------
------------------------------------------------------------
------------------------------------------------------------
------------------------------------------------------------
------------------------------------------------------------
------------------------------------------------------------
------------------------------------------------------------
------------------------------------------------------------
------------------------------------------------------------
------------------------------------------------------------
------------------------------------------------------------
------------------------------------------------------------
------------------------------------------------------------
------------------------------------------------------------
```

```
------------------------------------------------------------
------------------------------------------------------------
------------------------------------------------------------
------------------------------------------------------------
------------------------------------------------------------
------------------------------------------------------------
------------------------------------------------------------
------------------------------------------------------------
------------------------------------------------------------
------------------------------------------------------------
------------------------------------------------------------
------------------------------------------------------------
------------------------------------------------------------
------------------------------------------------------------
------------------------------------------------------------
------------------------------------------------------
>XP_013791159_Limulus_polyphemus_Blip1
ATGGCATATCCG------------------------------------------TCGGAT
ACTAGCCTAAACTACGTGACCCAAACCGTGAAC------CCAACA---CGGCCAGTAATA
ATGGGTCCGGCTGCTAACCCAAGTCAGTCGGACGTGGAAGTTATGCGGCAGTACATTGAG
------------------------------------------------------------
---------------------------------------------------------TCT
CTACCACAGGACTTG---AGTTATCGTGAGGAACTGATGAGTACT---CAGTTATGGAAG
TCAGTGAAAGGTGAGCTTCTGGGGACATTACTGTTGACTGTGGTGGGCTGTGGGGCTAGT
CTATCTTTCCAG------CCAGGC------------------------------------
---------------------------------------TCGGCTGCTTCGCATGCTGTC
ACGCTTCAAGTGTCTCTGGCATTCGGACTGGTCATCGCTTCACTTGTCCAGTGGACAGGG
CCTGTCAGCGGAGCTCATATGAACCCTTCGGTATCTTTAGCCTTACTAGTGACCCGCCAC
ATCAGCATGTTGCGTGCAGGTCTTTACATAGTGGCCCAGTGTGTTGGAGCTCTAGTTGGA
GCCGCTGTATTGTACGGTCTCACACCGTCAGAGCAACGGGGTTTA---------------
---------------------------GTAAGCCTTGGAGCGTGCGAGCCATCTGAACAT
---------CTCCACCCAAGTCAAGCCTTCGGGTTAGAGTTTCTTGGGACCCTAGTAGTC
ATATTGACCATCTTGTCT---AACGCTGACCCAGCCAGA---------------------
---ACAAACTCC---------------GGATCCAAGGCATTGTCCGTAGGATTATCTTAC
ACCGCTGCCCATTTGTTGGCATTTCCTTATACTGGTGCAGGACTTAATCCAGCTCGTGCT
TTTGGACCTGCTGTTGTGGCCGGT---------AGATGGCGTCTTCATTGGGTATACTGG
GTAGGCCCGATCCTGGGCGGTATCATTGGTGGCTTGACATACGAGTACAGTCAAGTAACA
---TCACAATCCACTCAACAA---------CTGAAGCGTTCTTTC---CGCCGGAAG---
------------------------TCAGCC---------AGGGAAATG---ATGCGTGAT
CACAGC---GCC---CTCAGTAACAATGAAACA---GTGTTCACGGCTTTATCCAAC---
---GACTGT---CGAGAA------------------------------------------
------------------------------------------------------------
------------------------------------------------------------
------------------------------------------------------------
------------------------------------------------------------
------------------------------------------------------------
------------------------------------------------------------
------------------------------------------------------------
```


>JAALXS010000006_Tachypleus_gigas_Blip2
ATGGCGCACCCG--ACGATT
CCCAGCTTGGACTATGATCCCCAGAGAGTAAGC------CCATCA---CGGGTAGCGATG
ATGGGATCTATCATTAGCCCCAGCCAATCTACTGTGGAGGTCATGCGACGATACATTGAA

---TCT
CTTCCACAAGACATG---ACCTTTCGGGAGGAGCTATCCAGTTCT---CAGCTTTGGAAG
TCCCTGAGGGGCGAGTTGTTGGGAACGTTATTGTTAACTGTGGTGGGATGTGGCGCTAGT
CTGTCTGTCCAG------CCAGAT------------------------------------
---------------------------------------TCTGCTGCTGTGCAAGCTGTC
ACACTTAAAGTGTCTTTAGCATTCGGGCTGGTTATTGCTTCTCTTGTGCAGTGGACGGGA
CCTGTCAGCGGTGCCCATATGAATCCTTCTGTTTCCATAGCTTTGCTCGTGACCCGTCAT
ATCAGCGTGCTGCGGGCCAGCCTCTACATTGTGGCTCAATGTGCTGGTGCTCTCGCTGGA
GCCGCCGTATTGTACGGACTCACACCACTGGAGCAGCGAGATTCG---------------
---------------------------GTAAGTCTTGGAGCGTGCGAGCCGTCGAAGCAC

---------CTCCACCCAAGTCAGGTTTTTGGGTTAGAGTTTTTGGGAACACTGCTGGTC
ATTCTGACCATCCTATCA---AACGCTGACCCAGCCAGG---------------------
---ACGGACTCT---------------GGTTCCAAGGCCTTGTCTGTAGGTTTTTCCTAC
ACTGCTGCACATCTGTTAGCGTTTCCATATACAGGAGCAGGACTCAACCCAGCTCGTGTT
CTTGGACCAGCGGTTGTTACCGGT---------AGATGGCGCCTTCACTGGGTGTATTGG
ATAGGACCAATGCTTGGTGGCATTATTGGAGGATTTACGTACGAATACAACCAAGATACA
---TCTCGAACCACTCAGCAA---------CTGAAGCGTTCATTC---CGCCGGAAG---
------------------------TCGGCT---------CGAGAAATG---ATGCGTGAT
CACAGC---GCC---CTCAGTAACAACGAAACG---GAATTCACGGCTATATCGAAC---
---GATTGT---CGAGCG--

>GGKL01007954_Tachypleus_tridentatus_Blip2
ATGGCGCACCCG--ACGATT
CCCAGCTTGGACTATGATCCCCAGACAGTAAGC------CCAACA---CGGGTAGCGATG
ATGGGATCTATCATTAGCCCCAGCCAATCTACTGTGGAGGTCATGCGTCGTTACATTGAA

---TCT
CTTCCACAAGACATG---ACCTTTCGGGAGGAGATAACCAGTTCT---CAGCTTTGGAAG
TCCCTGAAAGGCGAGTTGTTGGGAACGTTATTGTTAACTGTGGTGGGATGTGGCGCTAGT
CTGTCTGTCCAG------CCAGAT------------------------------------
---------------------------------------TCTGCAGCTGTGCAAGCTGTC
ACACTTAAAGTGTCTTTAGCATTCGGGCTGGTTATTGCTTCTCTTGTGCAGTGGACGGGA
CCTGTCAGCGGTGCCCATATGAATCCTTCTGTTTCCATAGCTTTGCTCGTGACCCGTCAT
ATCAGCGTGCTGCGGGCCAGCCTCTACATTGTGGCTCAATGTGCCGGTGCTCTCGCTGGA
GCCGCCGTATTGTACGGACTCACACCACTGGAGCAGCGAGATTCG---------------
---------------------------GTAAGTCTTGGAGCCTGCGAGCCGTCGAAGCAC
---------CTCCACCCAAGTCAGGTTTTTGGGTTAGAGTTTTTGGGAACACTGTTGGTC
GTTCTGACCATCCTATCA---AACGCTGACCCAGCCAGG---------------------
---ACGGACTCT---------------GGTTCCAAGGCCTTGTCTGTAGGTTTTTCCTAC
ACTGCTGCACATCTGTTAGCGTTTCCATATACAGGAGCAGGACTCAACCCAGCTCGTGTT
CTTGGACCAGCGGTTGTTACCGGT---------AGATGGCGCCTTCACTGGGTGTATTGG
ATAGGACCAATGCTTGGTGGTATTATTGGAGGATTTACGTACGAATACAACCAAGACACA
---TCTCGAACCACTCAGCAACTT------CTGAAGCGTTCCTTC---CGCCGGAAG---
------------------------TCGGCT---------CGAGAAATG---ATGCGTGAT
CACAGC---GCC---CTCAGTAACAATGAAACG---GAATTCACGGCTATATCGAAC---
---GATTGT---CGAGCG--

>VWRL01000910_Carcinoscorpius_rotundicauda_Blip2
ATGGCGCAGCCG--ACGATT
CCCAGCTTGGACTATGATCCCCAGACAGTAAGC------CCATCA---CGGGTAGCGATG
ATGGGATCTATTATTAGCCCCAGTCAATCTACTGTGGAGGTCATGCGACGTTACATTGAA

---TCT
CTTCCACAAGACATG---ACCTTTCGGGAGGAGCTAACCAGTTCT---CAGCTTTGGAAG
TCCCTAAGGGGCGAGTTGTTGGGAACGTTATTGTTAACTGTGGTGGGATGTGGCGCTAGT
CTGTCTGTCCAG------CCAGAT------------------------------------
---------------------------------------TCTGCTGCTGTGCAAGCTGTC
ACACTTAAAGTGTCTTTAGCATTCGGACTGGTTATTGCTTCTCTTGTGCAGTGGACGGGA
CCTGTCAGCGGTGCCCATATGAATCCTTCTGTTTCCATAGCTTTGCTCGTGACCCGCCAT
ATCAGCGTGCTGCGGGCCAGCCTCTACATTGTGGCTCAATGTGCCGGTGCTCTCGCTGGA
GCCGCCGTATTGTACGGACTCACACCACTGGAGCAGCGAGATTCG---------------
---------------------------GTCAGTCTTGGAGCGTGCGAGCCGTCGAAGCAC
---------CTCCACCCAAGTCAGGTTTTTGGGTTAGAGTTTTTGGGAACACTGCTGGTC
GTTTTGACCATCCTATCA---AACGCTGACCCAGCCAGG---------------------
---ACGGACTCT---------------GGTTCCAGGGCCTTGTCTGTAGGTTTTTCCTAC
ACTGCTGCACATCTGTTAGCGTTTCCATACACAGGAGCAGGACTCAACCCAGCTCGTGTT
CTTGGACCAGCAGTTGTTACCGGT---------AGATGGCGCCTTCATTGGGTGTATTGG
ATAGGACCAATGCTTGGTGGTATTATTGGAGGATTTACGTACGAATACAACCAAGATTCA
---TCTCGAACCACTCAGCAA---------CTGAAGCGTTCCTTC---CGCCGGAAG---
------------------------TCGGCT---------CGAGAAATG---ATGCGTGAT
CACAGC---GCC---CTCAGCAACAACGAAACG---GAATTCACGGCTATATCGAAC---
---GATTGT---CGAGCG--

>AZTN01049227_01194697_01049224_Limulus_polyphemus_Blip2
ATGGCGCACCCG--ACGATT
TCCAGCTTGGACTATGATCCCCAGTCAGTAAGC------CCAGCA---CGTGTAGCGATG
ATGGGATCTGCCATGAGCCCCAGCCAGTCTACTGTGGAGGTCATGCGACGTTACATCGAA

---TCT
CTTCCACAAGACATG---ACCTATCGGGAGGAGCTAACCAGTTCT---CAGCTCTGGAAG
TCCCTGAGGGGCGAGTTGTTGGGAACATTATTGTTGACTGTGGTGGGATGTGGGGCTAGT
TTGTCTGTCCAA------CCAGAT------------------------------------
---------------------------------------TCTGCTGCTGTGCAAGCTGTC
ACACTTAAAGTGTCTTTAGCTTTCGGACTGGTCATCGCTTCTCTTGTGCAGTGGACGGGA
CCTGTCAGCGGTGCCCATATGAATCCTTCAGTGTCGATAGCTTTGCTCGTGACCCGTCAT

ATCAGCGTGTTGCGGGCTGGTCTCTACATGGTGGCTCAGTGTGTCGGTGCTCTCGCTGGA
GCCGCCGTGTTGTATGGGCTCACACCATTGGAGCAGCGAGATTCG---------------
---------------------------GCAAGTCTTGGAGCGTGCGAGCCGTCGAAACAC
---------CTCCATCCAAGTCAAGCTTTCGGGTTAGAGTTTTTGGGAACGCTACTGATC
ATTCTGACTATACTATCA---AACGCTGATCCAGCCAGG---------------------
---ACAGACTCT---------------GGTTGCAAGGCCTTGTCCGTGGGTTTTTCTTAC
ACTGCTGCCCATCTATTGGCGTTTCCTTATACCGGAGCAGGGCTTAACCCAGCTCGCGCA
CTTGGACCAGCAGTTGTTACCGGT---------AGATGGCGTCTTCACTGGGTGTATTGG
GTAGGACCAATGCTTGGTGGCATTATTGGAGGGTTTACATACGAATACAACCAAGACACA
---TCTCGAACCACTCAGCAA---------CTGAAGCGTTCCTTC---CGCCGGAAG---
------------------------TCGGCT---------AGAGAGATG---ATGCACGAT
CACAGC---GCC---CTCAGTAACAACGAAACA---GAATTCACGGCAATATCGAAC---
---GATTGT---CGAGCA--

>XP_043221706_Amphibalanus_amphitrite_Blip
ATGGCA--
--GGCGGA
TCG---TCG---GACGACTCGGCACTCACCCCCGTCGAGCTGATGCGCCGCTATCTCGAC

---TCC
CTACCGCAAGACTTC---AGCGTGCGAGAGGAGCTGCGGTCGCCA---AAATTCTGGAAG
GCGTTGCGTGCCGAGCTGCTGGCGTCTCTCCTGGTGGCTGTATTCGTTACGGGCTCCACA
GCACGGCCACGAGCTCTCCACCAGCA----------------------------------
---------------CAGGGCCAACCACAGGCGACTGCGGGGTCCTCAGACGGCAGTGAC
GGCGCCCGGATGGCGCTGGCCTATGCGCTGACGACCGCCACACTTATCCAGTGCCTGGGC
AGCGTGTCGGGCGCGCACGCTAACCCTGCCGTGACGCTCGGCCTGCTCGTGAGGAGGCTG
GTCTCGCCCCTGAGGGCGGCCGCGTATGTGACGGCACAGCTAGCCGGCGGTCTGGCCGGC
GCGCTCATCCTGTACGGGCTGACACCTGCTGGGGTCGGTCACGGA---------------
---------------------------GATCAGGTAGCGGCGGTGCGTCCGGGC------
---------GTCTCGCTGCCCCAGGCGTTCGGCGCCGAGCTGCTGGCCACCTTCATACCC
GTGCTGACCACGCTGGCC---GCCCTGGAGCCGGTCAGG---------------------
---GAGGACGTC---------------GGATGTAAGGCGCTCTCCGTCGGACTGGCCTAC
GGCGTCGGCGCACTGTTCGCGTTTCAGCTGACCGGAGCAGGCCTCAACCCGGCCAGAACA
CTGGGACCAGCCGTTGTGTACAAC---------GTGTGGACCAACCATTGGGTGTACTGG
GTGGGGCCGCTGTTCGGCGGCCTCCTCGGCGGCTTTACGTACGAGTTTGTTCACGAGGCG
---TCACCGCGG---GGTCGTCTG------GTGCGCCGATCGTTC---CGGAGGTCACCC
GGCGGCGGGGTCAGAGGTCACCTGTCTGCCCCG---ATCAGAGGTCAGCCGGGCCGGGAG
GTCAGC---GGC---TTTAGCAGCGTGGCCACT---GACTGTCTCCAGCTGTCCGCCACT
GAGGAGGGTGCCGCCGTGACC---------------------------CGGCAC------

>ARA90644_Amphibalanus_improvisus_Blip
ATGGCA--
--GGCGGG
TCGGACGGCCGGGAGGAGCTGGCGATGAGTCCCGTGGAGATGATGCGGCGGTATCTCGAC

---TCG
CTTCCTCACGACTTC---AGTGTGCGAGAGGAGCTCGGATCGCCC---AAGTTCTGGAAG
GCGGTGCGAGCTGAGCTGCTCGGCTCGCTGCTGGTCGCTGTGTTCGTGACCGGCGCCGCT
GCGGGAAGGGCC---AGCGGTGAC------------------------------------
------------------GGCGCGGGTGGTGACGGAGGCGGTGACGGAGGCAGTGACGGA
GGTGCACGCGCTGCTCTCGCATATGCCCTAACCGCGGCTACTCTCGTACAGAGTCTCGGC
AGCGTATCGGGCGCGCATGCCAACCCGGCGGTCACGCTGTGCCTGCTCGTGCGCCGGCTG
GTGTCCCCTCTGCGTGCGGCTGTGTACGTGACGGCCCAGCTGGCCGGCGGACTGGCCGGG
GCGCTCATACTGTACGGGCTGACTCCACCCGGCGCCGCGCTCACA---------------
---------------------------GACCAGGTGGCCTCTGTGCGGCCGGGC------
---------GTCTCGCTACCACAGGCGTTCGGCGCTGAGCTTCTGGCCACGTTTCTGGTG
GCGCTGGCTACACTGGCG---GTGCTCGAGCCCGTCAGA---------------------
---GAGGATGTT---------------GGCACCAAGGCGCTCTCCGTCGGCCTCGCTTAC
GGGGCCGGTGCACTGTTTGCGTTTCAGCTGACGGGTGCTGGCCTGAACCCGGCGAGGACG
CTGGGGCCGGCCGTCATGTACAAC---------GTGTGGACAAACCACTGGGTGTACTGG
GTAGGGCCGCTGTTTGGCGGCCTCCTCGGCGGGTTCACATACGAGTTCGTACACGAGGCA
---TCTGCTCAG---GGCCGGCTG------GTGCGCCGCTCGTTC---CGCCGGCGAGGC
GGCAGCGGGGTTAGAGGGCAGAGAGACGGCCTG---GTCCCCGGTCAGCTGGGCCGGGAG
GTGAGC---GGT---CTCAGCACTACGGCTACT---GACTGTCTTCAGCTGTCCACCACC
ACCGATTGTCTACAGCTCTCCACCACTGAAGACGCCTCGGCAGCCGGCCGTCAC------

>GHBL01045886_Semibalanus_balanoides_Blip
ATGGCA--
--GGCAGG
TCGGAGGGCCGCGACGACGCGGCGCTCAGCCCCGTCGAGCTGATGCGGCGCTACCTCGAC

---TCG
CTCCCGCAGGACTTC---AGCGTGCGCGAGGAGATCCGGTCGCCC---AAGTTCTGGAAG
GCGCTGCGAGCCGAGCTGCTCTCCTCACTGCTGATGACCGTGTTTGTGAGCGGCGCTGCT
GAGCGGCGGACAGACGGAGCGGACACACCAGCC------------GGACACGGTCCTGCA

GCGGCAGCCGGACACGGCGCTGCAGCGCCAGCCGGAGCGGCAGCCGTGGACGGCGGAGAC
GGTACGCGGATGGCGCTGGCCTACGCGCTGACGGCGGCCACGCTGGTGCAGTGCCTGGGG
AACGTGAGCGGTACGCACGCCAACCCGGCGGTGACGCTCGGTTTCCTGGTGTGCCGACTG
GTGTCGCCGCTGCGGGCGGCCGCATACGTGGCCGTGCAGTTGGCGGGCGGCCTGGCCGGC
GCGCTCATCCTGTACGGGCTGACGCCGCTAGGTGGCGCGTACGTG---------------
---------------------------GACCAGGTAGCAGCAGTGCGGCCGGGA------
---------GTCTCCCTGACCCAGGCGTTTGGTGCCGAGTTTCTGGCCACGTTCCTGGTG
GTTTTCACCACGCTGGCC---ACGCTGGATCCGATCAGA---------------------
---CAAGACGTT---------------GGCTGTAAGGCGCTCTCCATAGGGCTGGCGTAC
GGCGTGGGATCGCTGTTCGCGTTTCCGTTGACAGGGGCTGGGCTGAATCCGACGAGAACC
CTGGGACCGGCTGTGGTGTATAAT---------ACATGGACAAATCATTGGGTGTACTGG
GTGGGGCCCGTGTTTGGCGGCCTTCTCGGAGGCTTCACGTACGAGTTCGTTCACGACTCG
---TCCTCTCAC---GGCCGGCTC------GTGCGCCGCTCGTTC---CGGCGGCGTCCC
GGCGGCGCGGCGCGGGCTCCTCTGGCCGGCGCG------CGGCCTCCGCTGGGCCGTGAC
GTCAGT---GGT---TTCAGCAGCGCCGCCACC---GACTGCCTCCAGCTGTCCGCCGCC
GATGACGTGTCGGGAGTCAGT---------------------------CGACAC------

>XP_037078235_Pollicipes_pollicipes_Blip
ATGGCA--
--GGCGGG
ACGGACAGTCACGAGAGCCCAGCGCTCAGTCCAGTGGAGCTGATGATGCGCTACCTGGAC

---TCG
CTGCCGCAAGACTTC---AGCGTGCGCGAAGAAGTACGCTCGCCC---AAGTTCTGGAAG
GCACTGCGGGCGGAGTTGCTCGCCTCGCTGCTGCTGGCGGTGTTCGTGGCGGGCGCGGCG
GAGAGC--
------------------GTCGGCGAGACGGGCGGGAAGGCGACGGCCAGCGACGCCGGC
GCGGCCCGACTGGTGCTCGCCTACACGCTGACGGCAGCGTCGCTGGTGCAGTGCGTCGGC
AACGTTAGCGGCGCGCACGCCAACCCGGCGGTGACGGTCGGCCTGCTGGTGTGCCGACTG
GTGTCGCCGCTGCGCGCCGCCGCCTACGTCACCGTCCAGTTGCTGGGCTCGCTAGCCGGC
GCGCTGATTCTGTACGGCCTGACACCGCTCGACGGACCGCAGCTG---------------
---------------------------GCGCAGGTGGCGGCCGTGCGGCCGGGC------
---------GTCTCCCTGACGCAGGCGTTCGGCGCCGAGTTCCTGGCCACGTTCCTGGTG
GTGTTGACCACGCTGGCC---TCGCTGGATCCGGTGCGT---------------------
---CAGGATGCC---------------GGCTGCAAGGCTCTCTCCATCGGACTGGCCTAC
GGCGCCGGAGCCCTCTTCGCGTTTCAGTTGACTGGAGCTGGACTCTGCCCTTCTAGGATC
TTCGGCTTGGCCGTGGTGTACAAC---------TCCTGGACAAATCACTGGGTGTACTGG
GTCGGTCCCACCTTCGGCGGAATTTTGGCCGGCTTCACGTACGAGTTCGTTCACGAGTCG
---TCGGCGCAG---GGGCGCTGG------GTGCGCCGCTCGTTC---CGACGGCGGCCC
GGCGCACCGCTCAGAGGTCACCTGGGTCAGCGA---GGTCACGGCCAGCTGGGGCGCGAG
GCCAGC---GGC---ATGAGCAGCATCACCACC---GACTGCTTGCAGCCGTCAGCCGCC
GAAGACGTGTCCAGCATCAGT---------------------------CGACAT------

>GHFC01061963_Neolepas_marisindica_Blip
ATGGCA--
--GGCGGG
CTGGAGCGGGGAGGAGACGCGGCACTGAGCCTCGTCGAGCTGATGATGCGCTACCTCGAC

---TCG
CTGCCGCAAGAGTTC---AGCTTCCGCGAGGAGGTGCGCTCGTCG---AAGTTCTGGAAG
GCGCTGCGGGCCGAGTTCCTGGCGACACTCCTGCTCGTCATCTTCGTCAGCGGCTGCAGC
TCCCTGTCGTCGACGGCGTCGCCGTCG---------------------------------
------------TCGTCGGGTGGCCACGTCAACGGCACCATCGCTGACAACGGCAGCAGC
ACATTCAGACTGGCGCTCGCCTATTGTCTGACCACCGCCACGCTGGTGCAGTGCTTCGGC
AATGTGAGCGGCGCGCAGACGAACCCGGCCGTGACGGTGGGTTTGCTGGTGACGCGCCGC
ATCTCGCCTCTGCGCGCCGCCAGCTACGTCACCGTCCAGACGGTCGGTGCGCTGGGCGGC
GCGCTCATCGTGTTTGGACTAACGCCACGCGACTGGCCGCCAGAA---------------
---------------------------CAGCAGGTGTTGACTGTGGGAGACGGC------
---------GTGGCGCTGACTCAGGCGTTTGGCATGGAGTTCCTGGCCACGTTCCTGGCC
GTGGTGACCACACTGGCT---GCGCTGGATCCGGCCCGC---------------------
---GAGGACGTC---------------GGCTGCAAGGCGCTCTCCATCGGCCTGGCCTAC
GGCGTCGGCACCCTCTTCTCGTTTCAGATGACCGGAGCGTGTCTCAACCCAGTGAGAGCC
CTGGGGCCCGCTGTGGTCAACAAC---------GTCTGGACCAACCACTGGGTGTACTGG
GTGGGGCCGGTCTTTGGCGGCATCCTGGGCGGATTCACGTACGAGTTCGTGCACGACTCG
---TCGCCCCAC---GGCCGCTAC------CTCCACCGGTCGTTC---CGGCGGCGGCCG
CCTGACCTGACCCGACCT------GACCCGGCG---GCGCGCGGCCGTCTGAGGCGGGAG

GTGAGC---GGC---ATCAGCACGGCCACCACC---GACGTCCTGCAGCCGGCCGGAGCC
---GAGATCTCCGGCGTGAGC---------------------------CGTCAC------

>JAFIDM010009631_Thereuonema_tuberculata_Blip
ATGTTC--
------------------------AACTATTCG------CCTTTGTTCATG---CAGAGC
TCGGCGAGCACTGGACCGGCTGCGGTGTCGCCGGTGGACGTAGTTCGTCGATACCTGGAG

---TCT

CTTCCTCAGGAGATT---CCTTTCAAGGAAGAGATTAGGAGCTTC---CAATTCTGGAAA
GCCGTGAGGACAGAATTTCTAGTGACTCTACTATTGGTTCTTTTCGGTTGCGGGGCGTCC
GTGCGATGGACGACTGCCCGCCCGCTAACCCTCGCC------------------------
---------GCGAACGAAACGGACACCGCCGACACGAGCGAGGTGACTCTCGTC---GAG
GAGGTGAAGATCTCCCTTACGTTCGGTCTCACCGTTGCCACACTGATCCAGTGTGTGGGC
AACATCAGCGGGGCCCATGCCAATCCGGCCGTGTCCATCGCCATGTTGGTGACACGGAAT
ATATCTCCTCTGCGGACCTTTACTTACGTGCTCGCTCAGTGCGGTGGAGCAATTACCGGT
GCCGGGATCTTGTACGGACTCACTCCGGACGTGGTTCGGCAT------------------
------------------------GGCCTGGGCGTCACTACCGTCCACCCTCGG------
---------GTTCATCCAACTCAAGCGTTCGGGGTTGAATTCATGACTACACTGGTAGTA
GTGTTCACTGTAATGGCC---AATGTGGACCCCCATCGA---------------------
---GCAGATATG---------------GGCTTCAAAGCACTTTCTATTGGATTAGCCTAC
ACGGCCGGACACTTCTTTGGGTTCCGTTATACTGGAGCCAGTATGAATCCCGCTAGATCT
CTTGGACCGGCTGTTGTAATGAGA---------AGCTGGAAACAACATTGGATCTATTGG
GTCGGTCCCATTCTCGGAGGCATTATAGGGGGTTTTACTTACGAGTATACACACGATTCT
---TCTCGGACT---CCCCAATAT------CTGAAGAGATCCTTC---CGCCGAAAG---
------------------------CGAGAAGCT------CGTGAACTG---AAGCGTGAA
CCCAGC---GGT---CTGAGCTCCAATGAGACG---GAACTGACTCCGACGTCCAAC---
---GATTGT---CGCGTG--

>JAAFCE010007744_Trigoniulus_corallinus_Blip
ATGGCCACAGGA--
------------------------CGCCAACACCGTCGTCCATCTGAAGCT------ACA
GCTAGTGGACATTATTCGGAAGGACCAACGGCCATTGAGGCTGTTCGGCGTTACATCGAA

---TCT
TTACCCCAACAGATG---CCTTTCAGGAAAGAAGTGAGGTGTGGA---CGGTTTTGGCGT
GCGGTATGGACAGAATTTGTGGTCACACTTCTCTTTCTTGTCATCGGTTGTGGTGCAGGA
GTCCGATGGAATTCAACAACAGACTCT---------------------------------
---------------AGTGGGTTGCCCACTGATATTTCGGTCGCCACCGCCAATGACGAT
GATCTTAGAGCACCACTTGCAATTGGTCTGACAGTGGCTGCCCTGATGCAATGTGCTTCA
CATATCAGTGGTTGTCACCTCAACCCGGCTGTTAGCATCGCTTTGCTAGTAACGAGAGCC
ATCACACCACTAAGGACGGCCCTATTTGTACTAGCTCAGTGTCTTGGTGCAATCGTCGGA
GCGTCCATTCTGTATGGATTGACGCCCAACGACATCAGGATTCAT---------------
------------------------GGCCTTCCGGCAACCGTGTTAAACCGAAAA------
---------GTTCATCTCAGTCAAGCCTTTGGAGTGGAATTTTTGACTAGCCTTCTTATT
GTGATCACTCTGATGGCC---ACTTACGACTCCATTCGACAA------------------
---GGACACTTC---------------TGTTTTAAAGGACTACCAGTTGGTGTGGCATAT
ATTGTGGGACATCTTTTTGGGTACCGATACACGGGTGCCAGTATGAATCCAGCTCGATCT
TTAGGTCTGGCGCTTATCACGAAT---------TCATGGACCTATCACTGGGTATACTGG
GTAGGTCCCATCCTGGGAGGCATCATTGGCGGTATCACATACGAATACATACGGGATAAT
AACTGCCAGAAAAGTGAACATCTT------TACCATCAGCAACAA---CAGCACCAG---
---CAATCATCCTGGAGAGAATCTCCTCATCACAAGAATCAAGAAATG---AAACTGAAT
GTTAGC---GCT---ATGACCTCCCAAGAGACT---GCACTTACAATCACATCCAAT---
---GATTAC---CAAGTT--

>GCIX01020944_Ommatoiulus_sabulosus_Blip

------AAGGCGTCTCTGGCGCTGGGCCTGACGGTGGCGGCGCTGGTGCAATGTGCGGCG
CCGGTCAGCGGAGGACACATGAACCCTGCGGTCAGCGTGGCCTTGTTGGTCACTCGTGCG
GTTACTCCCCTGCGCGCGGTCAGCTACGTGCTTGCTCAGTGCCTGGGCGCCATGGTTGGT
GCTGCCATCTTGAGCGCCCTCACACCCGCAGAAGTGCGTCTGGAA---------------
------------------------GGACTTACGGCTACCGTCTTGCACCCCGCA------
---------GTCCATCCCAGCCAGGCCTTCGGGGTGGAGTTCCTCATCACACTGTTGCTG
ATCGTCACCCTCGTGGCC---ACCCAAGGGGGCACTCGGCAT------------------
---GACCACGCC---------------TGCTTCCAGGGGCTTCCGGTGGGCGTGGCGTAC
ACGGTCGGACATCTTTTTGGGTACCGTTACACAGGTGCCGGAATGAACCCGGCCCGGTCC
TTGGGTCTTGCAGTCGTCACTAAC---------TCTTGGACCCATCACTGGGTCTACTGG

GTGGGACCCATTCTGGGCGGAATCATCGGTGGAATCACGTACGATTACATCCAGGACGGC
---GACAGCAGCGCCCTCATCGCC------TACCGGGCCAACTCC---GCTAGCAGA---
---------------CCGGATGCGCCATTGGTC------GGGGGAGTGAAGCAGTCGGAC
ACCAAC---ACA---TCGGTCGTTCATGAGACG---GAAATGACTTTCACTTCCGGC---
---GACTAC---CGCGTC--

>GCIH01033833_Symphylella_sp_Blip

------------GTG---AGTCTCAAAGAGGAAGTGCGCAGCTAC---CAGCTGTGGAAA
GCGGTACGGACAGAGTTTCTGGTCACGCTCTTCCTCGTTTTGATGGGCTGCGGCGCTTCG
GTTAATGTTACGTCATCGACGGAAACAACGGCGAAT------------------------
------CGAACTGCGTCCAGCAATTGGTCAGAGTCGCTAAATTTCTCGTGGTTT---CAC
GAACTTAAAGTGGCGTTTGCGTTCGGTTTCACGGTGACGACGCTAGTACAGTGTGTGGGG
AATATCAGCGGAGCGCATCTGAATCCGGCTGTAAGCGTGGCTCTGCTTGTAACTCGCCAC
ATATCGCCGCTGCGAACGGCCATGTACGTTGCGGCGCAATGCGGCGGCGCCGTCATGGGC
GCCGTTATTCTGTACGGACTGACGCCCTCCGTGTTTCTCGAGTCGGGG------------
------------------------CGGTTGGGTCAGACGTGCGTTCACTCGGAC------
---------GTCTATCCGACTCAGGCGTTCGGCGTCGAGTTTATGATCACGTTCCTTGTC
GTACTGACCGTCCTAGCA---AACATGGATCCCTACAGG---------------------
---GTGGACGCC---------------GGATTCAAGGCACTCTCGATAGGGCTGGCCTAT
ACGGTCGGACACCTGTTTGGGTATCGTTACACTGGTGCAAGCATGAACCCGGCCCGTTCT
CTGGGACCGGCTGTCGTTATGAAC---------ACCTGGTCTCTACACTGGGTCTACTGG
ATCGGACCCTTGTTGGGCGGAATAATCGCCGGTTTTACGTACGAGTACACGCAGGACTCT
---ACGCGGAGC---ACGCACTAC------CTGAAGCGCTCGTTT---CGGCGA------
------------------------CCTCGC---------GGCGAAATG---AAGAGAGAC
CAAAGT---GCT---CTCAGTTCCGCCGAAACC---GAACTCACCTCTCCC---------


```
------------------------------------------------------------
------------------------------------------------------------
------------------------------------------------------------
------------------------------------------------------------
------------------------------------------------------------
------------------------------------------------------------
------------------------------------------------------------
------------------------------------------------------------
------------------------------------------------------------
------------------------------------------------------------
------------------------------------------------------------
------------------------------------------------------------
------------------------------------------------------------
------------------------------------------------------
>GCAP01021432_Scolopendra_cingulata_Blip
------------------------------------------------------------
---------------------------------------------------------ATG
ATGAACGGCGGTGCCACG---ACTCAGTCGCCGACAGATGCCATTCGTCGCTACCTCGAA
------------------------------------------------------------
---------------------------------------------------------CGT
CTTCCTCGAGATCTC---CCTGTGAGGGAAGAATTGTGCAGCTAC---CGTTTCTGGAAA
GCCGTGAGGGCTGAGTTCCTGGCGGCGCTGATTGTCGTCTTATTCGGATGCGGAGCTGCG
TGGCCAGATAGAGAAACTCCAAATGGT---------------------------------
------------------CAAGATGGCAAAGATTCGGAAGGAGCATTTCTTCTC---CGA
GAGATGAAATCAGCACTGACCACTGGCTTTGCTATTAGTACCGTCATTCAATGTTTCGGA
AACATCAGTGGTGCCCACAGTAACCCAGCTGTGTCCGTTGCCATGCTTGTCACTAGAAAT
ATTTCTCCCATACGTACCTTTTTTTATGTAGTGGCCCAGTGTCTGGGAGCCATTGCAGCA
GCAGCTGTCTTTTATGGTCTGACTCCGGTTTCGTTAAGAACTAAT---------------
------------------------AATTTGGGAATAACCACTGTGCACGAACAG------
---------GTGCATGTAAGCCAAGCGTTTGGAGTCGAATTCTTAACAACGTTCGTGATG
GTGATGACAGTTTTGGCCATAAACATCGATGCGAACAGA---------------------
---GATGACGCC---------------GGTTATAAAGCGATTTCTGTAGGATTGACGTAC
GTCATTGGGCATTTTTTTGCATATAGGTGGACAGGAGCCAGCATGAATCCTGTTCGATCT
CTAGGTCCAGCTATTGTCATGAAC---------AAGTGGCAGAATCACTGGGTTTATTGG
GTCGGACCTTTGCTGGGTGGCATCATAGGAGCATTCACCCACGAATACACAAGAGATTCG
---TCGCAAACG---AACCAGTAC------TCCAAACGTTGCTCG---CGTCGCAAA---
------------------------TTTGAGGGT-------CGCGAAATG---AAGCGGAT
GAAAGC---GCC---CTCTGCTCTCAAGAAACG---GAACTGACAGCA------------
------------------------------------------------------------
------------------------------------------------------------
------------------------------------------------------------
------------------------------------------------------------
------------------------------------------------------------
------------------------------------------------------------
------------------------------------------------------------
------------------------------------------------------------
------------------------------------------------------------
------------------------------------------------------------
```


>JAHWFO010005382_Rhysida_immarginata_Blip

---ATG
ATGAACGGTAGTGTCCCG---GTTCAATCGCCAGCTGAAGCCATCCGTCGCTACTTGGAC

---CAA
CTTCCTCGTGAAATA---CCTGTACGTGAGGAGCTCTCCAGCCAC---CGTTTCTGGAAA
GCTGTAAGAGTTGAATTTCTAGCAGCTTTGCTGGTCGTGCTCTTTGGGTGTGGGGCTTCC
TGGCTCGAAGACGACGAGAAAACT------------------------------------
------------------------------GATGACGAAATGGCGCTTCTTTTA---CGG
GAGATGAAGGCCGCCCTGGTCGTGGGCTTTGCAGTCGTCACTATAGTTCATTGTTTTGGC
AGTGTAAATTGCGCCCATGGCAATCCGGCTGTCTCCGTCGCCATGCTCATCACACGAAAT
ATCTCGCCAATTCGTACTTTCTTTTATTTCATATCCCAGTGTTTAGGGGCCATCGTCGGA
GCTGCCGTCTTTTATGGTCTCACTCCTATGACTTTAAGAACTACC---------------
------------------------AGTCTAGGGATAACATCTGTTCATGAAGAA------
---------ATGCACTTGAGTCAGGCGTTCGGAGTGGAATTCTTGACGACACTGGTTGTT
GTGCTGGCAGTCTTGACC---CGGCTTGATTCCAATACA---------------------

---TCCCCAACA---------------CATTGCAGAGCACTCTGCGTTGGATTAGCCTAT
GCCATTGGCCAGTTGTTTGCA---------------------------------------
---GTATATTGG
GTAGGACCAGTACTGGGTGGCATAATAGGAGCCTTTACCCACGAGTACACACGTGAATCG
---TGCCCAACGAACCAACAGCAT------TCCAAGCGCTCGTTT---CGTCGCAAA---
------------------------TTCGAAGGAGGAGGACGTGAAATG---AAGCGCGAC
CAGAGC---GCT---CTCTGCTCCCAAGAAACC---GAATTGACAACAACCTCGCTG---
---GACGGG---CGCATT--

>JAAFCF010006680_Helicorthomorpha_holstii_Blip
ATGAGTGATCAC--CAGAGC
ATTTTAATGTCTCCCACCAGTTCATCATTGAATCATAATGCTTCAAATGCCACTCAGTAC
AACTATAACAACCGAAAAAACGGTGTAACGGCCGTGGAACTAGTTCGCAGGTACATTGAA

---TCG
CTTCCTCAGCATTTG---CCATTCAAAAAAGAAGTTCGAAGTGGA---CATTTCTGGAAA
GCAGTTTGGACAGAATTTATAGGTACTATGTTATTTGTTGTGATTGGTTGTGGTAGTGGA
ATTAGG---TGGCATCCATCTGACAGT---------------------------------
---------------------ATTCCA---GGAGTATCG------------------GAG
GATCTTAAGGCTTCTTTAGCCATCGGCTGTACAATGGCGGCTTTAGTTCACAGTGCCGCT
CATATTAGTGGGTGCCATCTGAATCCAGCGGTCAGTATAACACTTTTAGTAACGCGAAAT
ATTACACCTTTACGAACTGCCACCTACGCCTTGGCCCAGTGTCTAGGGGCTGTTGTAGGA
GCCGCAGTACTTTATGCATTGTCGCCTAGTGACATACGACAAACG---------------
------------------------GGATTGCCTGTCACCGTCCTCAGACACAAC------
---------ATTCATCCTAGTCAAGCTTTTGGAGTGGAATTTCTCATTACACTTTTGGTC
ATCATTACTCTTATTGCT---ACTTATGAAATGCAATCT---------------------
---AGTGAGACA---------------GGACTGAAAGGAATGAGCGTTGGAGTTGCTTAT
ACTGTCGGACACCTGTTTGGGTATCGATACACCGGTGCTAGTATGAATCCAGCTCGTTCC
TTTGGACTTGCTTTAATCAGCAAC---------ACCTGGGCCAATCAATGGGTTTACTGG
GTGGGGCCCATTTTAGGTGGAATTATTGGTGGAATAACTTACGAATATATACGTAGCAGT
---AAAAATGTCAACGGT---TAC------ATTCGTGCGGAACAGCCGTCAAATCAGCCA
CCGCAGTCGACGGCAATGACGGCTGACGTTAACGGTGGCGGTGAGCTT---AGACGAGAC
CAGAGC---GGT---TTCAGTTGCTGTTCAACC---GATTGTACT---------AAT---
---GACTAC---CGAATC--

>GEAZ01014274_Craterostigmus_tasmanianus_Blip

---------------------------TCGGATGCAGCTGTGCAATCGCTGCTG---GCA
GAACTTAAAATATCTTTGGCCTTTGGCTTGACGGTGGCTACTTTAGTGCAGTGCGTCGGA
AACATTAGCGGCGGGCACGGGAACCCTGCGGTTTCGATAGCGATGCTGGTGATTCGAAAC
ATTTCGCCGCTGCGTGCGGTGTGCTACGTGACGGCACAGTGCGTCGGTGGGATCACCGGG
GCGTGGATACTGTACGGACTGACCCCGTCGTCCGCCCGTTACCTGGGCAGT---------
------------------------GGATTAGGAGTTACGGCCGTTCACCCGCAA------
---------GTGAAAACCAGCCAAGCGTTTGGAATTGAATTCATGGCCACTCTGGTCGTG
ATCATCACGGTCATGGCC---AACGTAGATCCACATCGA---------------------
---GCAGACATG---------------GGATTCAAGTCACTGAGCATTGGTTTTGCGTAC
ACCGTGGGCCATTTCTTCGGGTATCAGTCCACAGGTGCTAGCATGAACCCAGCAAGATCT
CTGGGGCCAGCGTTAATTATGAAC---------AGTTGGAAAAATCATTGGGTGTATTGG
GTCGGTCCAATTCTCGGTGGGATCATCGGCGGAGTAACGTACGATTACACGCGCGACTCT
---TCCAGGACC---GTTCAGTAT------CTACGGCGAAGCTTC---CGGCGCAAA---
------------------------CACGAAACC---CACCACGTAATG---AAACGTGAC
CACAGT---GCC---CTTAGTTCTCAGGAAACG---GAGCTGACCACCGCATCCAAT---
---GATTGC---AGAATA--

>GCIL01020555_Himantarium_gabrielis_Blip

---GGATGCGGAGCGTGC
GTTCGA---TGGACACCGTTG---------------------------------------
---------------------GACGACATCGACGGA---ACGATGTCGATGGTC---GAA
GAAGTGAAAGTCGCTTTGGCATTCGGCGTCGTAAAGGGAACCGTCGTCCAATGCGTCGGG
CACATCAGCGGCGCCCACTCTAATCCAGCAGTATCCGTCGCAATGCTGGTAACGAGAAAT
ATTTCTTTACTGAGAGCAATGCTCTACGTCGTCGCTCAATGTCTCGGCGCAATGGCTGGA
TCTGCAATTCTCTACGGCCTCACCCCAGCTCCTCTCCGCCAT------------------

```
------------------------GGACTGGGCGTCACTCAACTTCATTCCCGC------
---------GTGCATCCGAGTCAAGCTTTCGGCGTCGAGTTCATGGGCACTCTCATAATC
GTTTTGACTGTTATGGCC---AATGTTGATCCTCAAAGG---------------------
---GCTGATATG---------------GGCTTCAAGGCATTGTCTATTGGATTGGCTTAC
ACGGGAGCTCACTTATTCGGATTTCGTTCTACGGGAGCTGGAATGAATCCTGCGCGGTCC
TTAGGCCCTGCTGTGATCATGAAT---------TCCTGGAAAAATCATTGGGTATATTGG
GTCGGGCCCATTCTTGGCGGCATCATCGGCGGCTTCACTTACGAATACACCCAGGATTCA
---TCGCGAGGA---CCTCAATAT------TTGAAGAGATCCTTT---CGGCATCGA---
------------------------CGCGAACAGAGTGGTCAACAGATA---AAAAGAGAA
TCGCAAAGCGGG---ATAAGTTCGCACGATACT---GAACTT------GCGTCGAAT---
---GATTGT---CGAGTG------------------------------------------
------------------------------------------------------------
------------------------------------------------------------
------------------------------------------------------------
------------------------------------------------------------
------------------------------------------------------------
------------------------------------------------------------
------------------------------------------------------------
------------------------------------------------------------
------------------------------------------------------------
------------------------------------------------------------
------------------------------------------------------------
------------------------------------------------------------
------------------------------------------------------------
------------------------------------------------------------
------------------------------------------------------------
------------------------------------------------------------
------------------------------------------------------------
------------------------------------------------------------
------------------------------------------------------------
------------------------------------------------------------
------------------------------------------------------------
------------------------------------------------------------
------------------------------------------------------------
------------------------------------------------------------
------------------------------------------------------------
------------------------------------------------------------
------------------------------------------------------------
------------------------------------------------------------
------------------------------------------------------------
------------------------------------------------------------
------------------------------------------------------------
------------------------------------------------------------
------------------------------------------------------------
------------------------------------------------------------
------------------------------------------------------------
------------------------------------------------------------
------------------------------------------------------------
```


>AFFK01021957_Strigamia_maritima_PripLA7
ATGGGT--

------------------------------TCGGAAACG---AGAGCT------------

--GCTATT
GCCAAGTGTATTAGCTGCTCCGAAGTAACG---TCTACG---------AACATTTGGAAG
TCTTGTCTTGCCGAATTTATCGGCACATTAGTCCTTGTTTTGGTCGGATGCGGTTCGTGC
GTTAGT---TGGAATGCT--
---GCCGTACCTGCCGAC
TTAGTGCAAATATCCTTGGCTTTTGGAGTCACCGTTGCAACCATAGTCCAATGCATTTGC
CACGTCAGTGGTGGCCACATCAATCCAGCGGTTTCCATCGGCATGTTAGTTACGTTGAAC
ATCAGTGTGCTGCGATGTGTGTTGTATGTCTTATGTCAATGTGTTGGTGCCATTGTTGGA
AGTGTCTTTTTGAAGGTTTTTACACCTAATTTCTTCCATAAA------------------
------------------------------TCTATGGGTCCAACGGTAGTAAATGCAAGT
---------ACAAGTTCATTTGAGGCGGTTGGTGTGGAGTTTTTCATCACTTTTGTACTG
GTTTTAACTGTATTTGCT---GTTTGTGATCCTAATCGCAAC------------------
---GATGTTAAG---------------GGGTCTGCGCCACTTGCCATTGGTCTTGCTGTG
GCTACTTGTCATTTATTTGCGATTCCGTATACTGGCTCGAGTATGAATCCCGCACGGTCT
CTAGGACCTGCTATTATTTTGAAT---------ATTTGGAATAATCATTGGGTATACTGG
ATTGGACCTATTCTTGGCGGAGTTACCGCGGCGATTTTCTATAAATTCGTTTTAAGCGAT
---CGTAAACGAGAT---------------------------TTATCTAGTACT------
------------------------GAAAGT---------CAGTGTCTT------ATCGGT
CGGTAC---CTA------GGA------TCT------------GAT---------CTT---
--GATAAG------

>AFFK01022706_Strigamia_maritima_PripLA8
ATGGCT--

------------------------------TCCCGACTT---AGAGATGTTGTATTCAGG
TGT---
--GTCGAC
TGTTGTACCAATGGCTGCTCCGAAGTTTTT---GAATCG---------AATATTTGGAAG
TCTCTCGCCGCCGAGTTTGTTGGTACACTGTTCCTCGTTTTGGTCGGTTGCGGCTCGTGT
ATCGGC---TGGAAT---
---GCCACCGAC
CTAGTGCAAATATCTCTGGCTTTTGGAGTGACCGTTGCAATCATGGTCCAATGCATTTGT
CACATTAGCGGTGGTCATATTAACCCGGCGGTTTCCATCGGTTTGTTAGTTGCGAGAAAA
ATCAGCGTTTTGCGATGCGTGTTGTACATCGTGTGCCAATGTGGAGGTGCCATTATCGGG
GCTCTTTTATTGAAAGCCTTTACACCCGCCGCGTTACGAAAA------------------
------------------------------TCTTTGGGCGCAACGATGGTAAATGCGAAA
---------ATCAGTTCTGTCGAGGCTTTTGGCGTGGAGTTTCTCATCACTTTTGTACTG
GTTTTTACCGTATTTGCC---GTTTGTGATCCACTTCGCAAA------------------
---GATGTTAAG---------------GGTTCTGCGCCATTAGCTATTGGTCTTTCGGTG
GCGACTTGTCATTTATTTGCAATTCCTTATACCGGTGCAGGAATGAATCCTGCTCGATCG
TTTGGACCGGCTGTGATAATGAAT---------ATTTGGGCTGACCATTGGGTATATTGG
ACTGGACCGATTCTTGGAGGAATCGTAGCTGCGGGTTTCTATCATTACGTTTTAAGGGCT
---AGTAAATATAAG------------------------------TCAACT---------
---------------------------------------TTAAGTCTT------ATTGAT
GAAGAG---TTA------------------------------GAT---------GCTAAC
--GACCCA------

>AFFK01023535_01023534_Strigamia_maritima_PripLA5
ATGGGA--

---------------------------------AGATTT---CAA---------------

---GAT
GCT---TTTTTGAAT---AAT---GCAACG------------------GACGTTTGGCGA
GGCCTTCTCGCCGAATTCATCGGCACACTGTTCCTCGTTTTAATCGGTTGTGGCGCGTGT
ATTCAT---TGGAGC---
---AGAGCCGAC
ATTGTGCAAATTTCCCTCGCGTTTGGAGTGACCGTGGCGACCATAGTCCAATGCTTCGGT

CACGTGAGCGGA---CACATCAATCCGGCCGTTACCATCGGTTTGCTCGTAACGAGAAAA
ATCGGCGCGCTACGATGTGTTCTTTACGTCGTAAGTCAATGTGTCGGCACCGTCGTTGGC
GCCGCTTTGTTGCGGGGTTTTACACCCATTTTGCTGCACAAA------------------
------------------------------ACAATGGGCGCTACGATGTTCAATACGACG
---------ATAAGTTCGTACGAGGCGTTTGGTGTTGAGTGTTTCATTACTTTTGTTCTG
GTTTTGACCGTGTTCGCT---GTTAGCGATCCAAATCGCGAC------------------
---GATGTTAAG---------------GGCTCGCCATCGCTCGCCATTGGTCTTTCAGTG
ACTGCTTGTCATTTATTTGCGATTTCCTACACTGGATCGAGTATGAATCCTGCAAGATCA
TTTGGTCCTGCAGTTATAATGAAC---------ATTTGGACCGGTCAT---GTTTATTGG
GCTGGGCCTATTCTTGGAGGCATTTTAGCCGCACTTTTGTATCGATTTGTTTTTCGTGCT
---CCTAAACCAGCT-------------------------------TCATCGTGT------
------------------------GAA---------------AGTCTC------GTTGAT
AGGGAA---TTG---AAGCCTACA---TAC------------GAA---------GCCATT

>AFFK01015980_Strigamia_maritima_PripLA1
ATGGGA--

---------------------------------AGATTT---CAA---------------

---GAT
GCT---TTTTTGAAT---AAT---GCAACG------------------GACGTTTGGCGA
GGCCTTCTCGCCGAATTCATCGGCACACTGTTCCTCGTTTTAATCGGTTGTGGCGCGTGT
ATTAAT---TGGAGC---
---GAAGCCGAC
ATTGTGCAAATTTCCCTCGCGTTTGGAGTGACCGTGGCGACCATAGTCCAATGCTTCGGT
CACGTGAGCGGA---CACATCAATCCGGCCGTTACCATCGGTTTGCTCGTAACGAGAAAA
ATCGGCGCGCTACGATGTGTTCTTTACGTCGTAAGTCAATGTGTCGGCGCCGTCGTTGGC
GCCGCTTTGTTGCGGGGTTTTACACCCATTTTGCTGCACAAA------------------
------------------------------ACAATGGGCGCTACGATGTTCAATACGACG
---------ATAAGTTCGTACGAGGCGTTTGGTGTTGAGTGTTTCATTACTTTTGTTCTG
GCCTTGACCGTGTTCGCC---GTTAGCGATCCAAATCGCGAC------------------
---GATGTTAAG---------------GGCTCGCCATCGCTCGCCATTGGTCTTTCAGTG
ACTGCTTGTCATTTATTTGCGATTTCCTACACTGGATCGAGTATGAATCCTGCAAGATCA
TTTGGTCCTGCAGTTATAATGAAC---------ATTTGGACCGGTCATTGGGTATATTGG
GCTGGGCCTATTCTTGGAGGCATTTTAGCCGCACTTTTGTATCGATTTGTTTTTCGTGCT
---CCTAAACCAGCT------------------------------TCATCGTGT------
------------------------GAA---------------AGTCTC------GTTGAT
AGGGAA---TTG---AAGCCTACA---TAC------------GAA---------GCCATT

>AFFK01018906_Strigamia_maritima_PripLA2
ATGGGT--

------------------------------TCCCGGGTA---AAAACA------------

--GCCATA
ATCAAATTTCTTGGCTGCAACGAATTGAAA---AAAGCA---------AACATTTGGAAG
TCTCTCATTGCTGAATTCATCGGAACATTTTTCCTTGTTTTAGTCGGATGTGGCTCATGT
ATTGGC---TGGAGT---
--ATTACTGCCGAC
TTGGTGCAAATAGCACTGGCTTTTGGAGTGACTGTTGCCACTATGGTTCAATGCATTTGT
CATGTAAGTGGTGGCCACATTAATCCAGCGGTTTCTATCGGTATGTTAGTTACAGGAAGA
ATTAGTTTGCTGCGATATTTCTTCTATATTTTGTGCCAATGTGGTGGTGCCATTGTTGGA
GCAGCTTTATTGAGGGCCTTTACACCTGATTCGTTGCATAAA------------------
------------------------------ACTTTGGGTTCAACGTTGTTTAATAGAAAT
---------ATAAGCGAATTAGAGGCTTTTGGTGTTGAGTTTTTCATCACTTTTGTGTTG
GTTTTTACTGTTTTCGCT---GTTTGCGATCCGAATCGCACT------------------
---GATGTTAAG---------------GGGTCTGCGCCACTTGCTATTGGTTTATCCGTG
GCTACTTGTCATTTGTTTGCGCTTCCTTATACGGGATCTAGTATGAATCCTGCCAGGTCA
TTAGGTCCTGCGGTTATTATGAAT---------ATTTGGAATGGTCATTGGGTATACTGG
GTGGGGCCTAATCTTGGCGGTATTGCAGCAGCCGTTTTATATCATTTAGTTTTAAGTGCT
---CCAAAACCAGTT------------------------------TCAACAGTG------
------------------------GAG---------------CTTCTT------ACAAAA
GAAGAG---GTG------CAAACT---TACTTG------AAGGAGGTC---AGAGCATCT
--GGGATA------

>GESK01018671_Strigamia_acuminata_PripLA
ATGACA--

------------------------------TCCAAA------TCGTTT------------

---CTC
GAGAAATTTCTCGGCCACGACGAAGTGACC---AAATCC---------AACATCTGGAAA
TCCCTTCTAGCCGAATTCATCGGCACTTTCTTCCTCGTTTTAGTCGGTTGTGGCGCGTGT

```
ATCGCC---TGGAAC---------------------------------------------
---------------------------------------------------GACGCCGAT
TTGGTGCAAATAGCTCTCGCTTTCGGCGTGACGGTCGCGACGATGGTGCAGTGTATTTGT
CACGTAAGCGGCGGCCACATCAACCCCGCCGTGTCCATCGGGATGTTGGTGACGGGGAAG
ACGAGTCTCGTGCGGTGTGCGTTGTACATTTTGAGCCAATGTGTCGGTGCAATCGTCGGT
TCGGCGCTGTTGAGAGCTTTCACGCCGGAGTCGCTGCACAAA------------------
------------------------------GCGCTGGGCGCGACTTTGGTGGCGGATAAC
---------ATAAGCGACGTCGAGGCGTTTGGCGTCGAGTTTTTCATAACGTTTGTGCTG
GTTTTGACGGTTTTCGCC---GTTTGCGATGAGAATCGGGAA------------------
---GATGTCAAG---------------GGTTCTGCACCGCTCGCCATTGGTCTTGCAGTG
GCCACTTGTCATTTATTCGCTATTCCTTACACTGGGTCCAGCATGAATCCGGCTCGGTCG
TTTGGTCCAGCTGTTGTTATGAAT---------CTTTGGGATGACCATTGGGTTTATTGG
ATTGGACCTAGTCTTGGAGGTTCGGCGGCAGCTATTTTGTATCATTTTGTTCTAAGTGGT
---TCGAAA------------------------------------GAGGAAGCG------
---------------------------------------AACAGTTTG------CTAAAA
------------------------------------------------------------
------------------------------------------------------------
------------------------------------------------------------
------------------------------------------------------------
------------------------------------------------------------
------------------------------------------------------------
------------------------------------------------------------
------------------------------------------------------------
------------------------------------------------------------
------------------------------------------------------------
------------------------------------------------------------
------------------------------------------------------------
------------------------------------------------------------
------------------------------------------------------------
------------------------------------------------------------
------------------------------------------------------------
------------------------------------------------------------
------------------------------------------------------------
------------------------------------------------------------
------------------------------------------------------------
------------------------------------------------------------
------------------------------------------------------------
------------------------------------------------------------
------------------------------------------------------------
------------------------------------------------------------
------------------------------------------------------------
------------------------------------------------------------
------------------------------------------------------------
------------------------------------------------------------
------------------------------------------------------------
------------------------------------------------------------
------------------------------------------------------------
------------------------------------------------------------
```

```
------------------------------------------------------------
------------------------------------------------------------
------------------------------------------------------------
------------------------------------------------------------
------------------------------------------------------------
------------------------------------------------------------
------------------------------------------------------------
------------------------------------------------------------
------------------------------------------------------
>AFFK01016279_Strigamia_maritima_PripLA6
ATGGGT------------------------------------------------------
------------------------------------------------------------
------------------------------TCCAAA------TCC---------------
------------------------------------------------------------
---------------------------------------------------------ATC
------TTCATCGGCCACAAAGAAATGACC---AACACC---------AACATCTGGAAA
TCCCTTCTCGCCGAATTCATCGGAACTTTCTTCCTCGTATTAGTCGGCTGCGGTTCCTGC
ATCAGT---TGGAAT---------------------------------------------
---------------------------------------------------GCCGCCGAC
TTGGTGCAGATATCCCTCGCGTTCGGAGTGACTGTTGCGACCATCGTCCAATGCATCTGC
CATGTCAGCGGCGGCCACATCAACCCCGCAGTGACCATTGGGATGTTGATAACTGGGAAA
ATCAGTTTGGTGCGATGTGTATTGTATATTTTGAGTCAATGTGTCGGGGCCATCGTGGGA
TCGGCAGTGTTGAGGGCGTTTACACCCGAGTCGTTGGTCAAA------------------
------------------------------TCGTTGGGTGCGACGTTGATCAATGAAAGA
---------ATCAGTTCGTTGGAAGCGTTTGGTGTTGAGTTTTTCATCAGTTTTATGTTG
ATTTTTACTGTTTTTGCT---GTTTGCGATGAAAATCGGACC------------------
---GATGTTAAA---------------GGGTCTGCGCCATTGGCCATTGGTCTTGCGGTG
GCCACGTGTCATTTGTTTGCGATACCATATACTGGGTCGAGTATGAATCCTGCGCGGTCG
TTTGGTCCAGCTGTTGTGATGAAT---------TTGTGGGATGACCATTGGGTATATTGG
ATTGGGCCTAGTGTTGGAGGTGCAGTAGCAGCAGTTTTATATCATTTTCGTGTTAAGTGC
---TAC------------------------------------------------------
------------------------------------------------------------
------------------------------------------------------------
------------------------------------------------------------
------------------------------------------------------------
------------------------------------------------------------
------------------------------------------------------------
------------------------------------------------------------
------------------------------------------------------------
------------------------------------------------------------
------------------------------------------------------------
------------------------------------------------------------
------------------------------------------------------------
------------------------------------------------------------
------------------------------------------------------------
------------------------------------------------------------
------------------------------------------------------------
```


>AFFK01018906_Strigamia_maritima_PripLA3
ATGAGT--

------------------------------TCCAAA------TCTTTT------------

---CTT
GACAAATTTCTTAACCACAAAGAAATCACG---ACCGCC---------AACCTCTGGAAG
TCTTTGATTGCCGAATTAATCGGAACTTTCTTTCTTGTTTTAGTTGGATGTGGCTCGTGT
ATTAGC---TGGAAT---
--GAGCCCGACGAC
TTACTGCAAATAGCCCTGGCTTTTGGCGTGACTGTTGCAACGATGGTTCAATGCATTTGT
CACATAAGTGGTGGCCATATCAATCCAGCAGTTTCTATTGGTATGTTAGTAACGGAAAAA
ATTGGTTTGATAAGATGTCTGTTATACATTTTGGGCCAATGTATTGGTGCTGTTATTGGA
TCAGCTTTATTGAGGGCTTTTACACCCGAAGCATTGCATAAA------------------
------------------------------TCATTGGGTGCAACGATAGTAAATGAGGAT
---------GTAGGCGGTGCAGAAGCTTTTGGCATTGAGTTGTTCATCACTTTTGTGCTG
GTTTTTACTGTTTTTGCT---GTTTGCGATGAGAATCGCGAA------------------
---GATGTTAAG---------------GGTTCTGCACCACTGGCCATTGGCCTTTCTGTC
GCTACTTGTCATTTGTTTGCAATACCGTATACCGGATCCAGCATGAATCCTGCTAGGTCT
TTTGGTCCTGCAGTTGTTATGAAT---------ATTTGGGATGACCATTGGGTATACTGG
GTTGGGCCTAGTTTTGGAGGTGCTCTAGCAGCAATTTTGTATCATTTTGGTTTAAGTGGT
---TCTAAA------------------------------------CAAGAACAG------

------------------------CAG------------AATGGCTTA------CCAAAG

>AFFK01013573_Strigamia_maritima_PripLA4
ATG---

------------------------------TCCTTAAAA---------------------

---ACA
TCTAAGATACTGGGGTTGAAGGATCTTAAAAGAACTCCC---------GATCTATGGAAG
ATTCTCATTAGCGAGCTGCTGGGCACGATGCTTCTTGTCGTTTTTGGATGTGGCGCGTCT
ATTCCC---TGGACAAAATCT---------------------------------------
--ACGGAT
ATAGTTCAAATTGCGTTAGCTGCTGGTTTCACGGTTGCTACTATTGTTCAAATTACTTTT
CACATCAGTGGAGGTCATATAAATCCTGCAATGTCATTGGCAATGATGGTTACCGGTAAA
ATGTCCATAATTATGTTTGTTTTATACGCGATTTGTCAATGTATTGGCGCCATCGGTGGT
GCATTATTGCTCAAAGTATTGACACCGGTTCATTTACGAAAC------------------
------------------------------AACCTCGGGATAAGTACCTTAAATACGGAT
---------GTATCAGAAGTTAACGGCTGTTGTGTCGAGTTCTTAATTACATTTATAATG
GTCATCACAGGATTTTCT---GTGTGTGATTCAAATCGCAGT------------------
---GATGTAACG---------------GGATCGGTTCCTCTTGCGATTGGTTTATCTGTA
ACAATAAGTCATTTTTTTGCGGTAAATTTTACGGGATGTAGTATAAATCCTGCTCGTTCG
TTGGGTCCAGCCGTCATTTTCAAC---------AAATGGCAGCATCACTGGGTATATTGG
GCTGGACCTTGCAGTGGAGCAATTGTAGCTGGTGTTTTATATCAGGTGGTATTTAAAGCA
---AATCGAGGA------------------------------TCGGTCAGC---------
------------------------TTA------------ATAAGTGAA------AATAAA
------------------CTCAAT------------------------------TCACTG
--GATATA------

>GESB01005378_Henia_illyrica_PripLA
ATGGGC--

------------------------------TCCTTCGCG---GACCGT------------

---TTC
ATGAAGTTTCTCGGTTGCAATGAATTAACA---GCAAATTTA------AACATATGGAAA
TCGCTCATCGCCGAATTCATCGGTACGATGTTTTTGACTTTGATCGGTTGTGGTGCATGC
ATTTCA---TGGGCA---
--CCGACTGCGGAT
TTCGTGCAGATTGCGCTGGCATTCGGAGTTGCTGTCGCGACCATGGTTCAATGCATTTGT
CATGTAAGTGGAGGTCACATCAATCCAGCGGTTTCGGTCGGTATGATTGTTACCGGGAAA
ATTAGCTTGGTGCGATTTTTATTTTACGTGTTGTGTCAATGCGCCGGTGCCATAGTCGGT
GCAGCGTTGTTGAGAGCTTTTACACCGGAATCTCTGCAAAAC------------------
------------------------------AATTTGGGTTCAACTTTAGTAACTGACGGT
---------GTTGAAAAAGCGCAGGCATTTGGAATTGAGTTTTTTATTACTTTTGTGCTG
GTTTTTACCGTGTTTGCT---GTTTGCGATGAAAATCGGACC------------------
---GATGTTAAA---------------GGCTCTGCACCTTTGGCTATTGGACTTTCGGTT
GCTGTTTGCCATTTATTTGCGATTCCGTTCACAGGATCTAGCATGAATCCCGCTAGGTCA
TTTGGACCTGCTGTCATCATGAAT---------AAATGGGATGACCATTGGGTATACTGG
GTCGGTCCTTGTCTCGGTGGTGCAGCGGCTGCTATTTTGTATCATTTGGTTCTTCGCGCT
---TCA---------------------------------------AATTCAGAA------
------------------------GAT---------------------------TCTAAA
GAAGAG---AAT---GCGAGAGGAGAATTC---------AAGTTT---------AAA---
--GGA---------

>GCAH01012881_Clinopodes_flavidus_PripLA
ATGGGT--

------------------------------TCCCGCAAG---GATGCC------------

---GTA
GTTAAATTCCTGGGGTGCAACGAGTTGGTT---TCGTCGACG------AATATCTGGAAA
TCGCTTTTCGGCGAGTTCCTCGGACTTTATTCCTCGTTTTTGATCGGCTGCGGGTCCTGC
GCCGGG---TGGCGT---
--TCGCCCGCCGAT
TTGGTGCAAATCGCGCTCGCTTTCGGCATTACGGTAGCCACGATGGTACAGTGCATCTGT
CACGTGAGCGGCGGACACATCAACCCGGCCGTGTCGGTCGGCATGCTCGTGACGGGCAAG
ATGAGTTTGGTTCGATTTTTCTTCTACGTTCTCTGCCAGTGCGCGGGGGGCATCACGGGG
GCCGCGTTGCTCAGAGCGTTCACGCCCGACAGGCTGCTGACG------------------
------------------------------AATTTGGGGATCACTTTTGTGCATGATGAT
---------ATTAGTAATGCGCAAGGTTTCGGTATCGAGTTGTTTATCACGTTTGTTTTG
GTTTTCACTGTTTACGCT---GTTTGTGATGCGAATCGCGGG------------------
---GATGTGAAG---------------GGGTCGGCGCCGCTGGCGATTGGGTTGGCCGTC
GGTGTTTGTCATTTGTTTGCGATTCCGTACACTGGGTCGAGTATGAATCCCGCACGCTCG

TTTGGGCCCGCGGTTATTATGAAT---------GTCTGGAAAGGCCATTGGGTTTACTGG
ATCGGCCCATGTCTCGGTGGAGCAGCCGCAGGACTCATTTACCAATTCGTTTTCAGCGAA
---CCCGCC---------------------------------AACGATTCTGAC------
------------------------GAA------------AGCCATCAC------TCGAAA
GACGAA------------TAC------------------AAATTC---------AAAGCA
---GCTGACATT------

>GCAP01021165_Scolopendra_cingulata_PripLA
ATGGCC--

------------------GCGACGGGCGCCGGTCGGTTC---CAGGGG------------

---ATT
CTCCCCGAACTCGGCCTCCAGGAGCACCGA---ACCCTC---------GACCTGTGGAAG
GCGGCCGCGGCAGAGTTCGTGGGGACCCTCGTCCTCGTGCTCGTGGCCTGCGGCGCATGC
GCAGTC--
---AACCCCACC
CGGGTGGACATCGCCCTCGCCTTCGGTTTCACGATCGCCACCGTGGTCCAGTGCATTTGC
CACGTGAGCGGGGGTCACGTCAACCCTGCTGTCACCATCGGGCTTCTGGTCACGCGGAAA
GTCACTGTCATCCGGGCCCTCGTCTACGTCATCGTTCAGTGCATCGGAGCCATTGTTGGC
GCTGCCCTGATCGAAGCCCTC---------GTCACCCATACT------------------
---------------------------GATGATGTGGGTGTTGCCCAAATTCACAACCGT
---------CTCAACGTGGGCCAAGGCCTGGGGGTTGAGTTGTTCATCACGTTCGTGCTC
GTGCTCACGGTCTACGCT---GTGACGGACACCAACCGAACT------------------
---GATGTCAAG---------------GGTTCAGCTCCCCTCGCCATCGGGCTCGCCATC
GGCATGTGCCATTTATTCGCTGTTCCGCTAACAGGGGCTGGCATGAACCCAGCACGTGCA
CTGGGACCTGCTGTCATCTACAAC---------AGCTGGAGAGACCATTGGGCCTATTGG
GTCGGTCCTATCTTTGGGGGAATCGCGGCGGGGCTTCTATACCATCTGGTCTTCAGCGCC
---CCTCCAACCTCG---------------------ACCCCCACCATCGTGGAG------
------------------------GACCGA---------CCTTTGCTC------CTCATG
AGACGG---TCC------CCTGCCAAATTTGCT---------GAA------ACGAGAAGC
---GGTGACGCCACC---
---GTC--

>GHBY01000515_Hemiscolopendra_marginata_PripLA
ATGGCC--

------------------ACGAACACCGTT---CGGTTC---CAAGGG------------

---GTT
TTGCCAGAGCTCGGAGTCCAGGAGTATCAA---AACCTT---------GACTTATGGAAG
GCGTCAGTGGCCGAATTCGTCGGAACGCTCCTGTTGGTGCTCGTGGCTTGCGGCACGTGC
GCCAGT--
---GAACCCGAG
TTAGTGGACATCGCCCTCGCTTTCGGCTTCTCGATAGCGACAGTGGTCCAATGCATCTGT
CATGTGAGCGGGGGACACATCAATCCAGCGGTCACGATAGGACTCCTAGTCACGCGCAAG
GTGACAGTCATCCGGGCACTCGTTTACGTCATCGTTCAGTGCATCGGAGCCATATTGGGC
GCTGCGTTGATCGAAGCGTTC---------GTGACACATGCG------------------
---------------------------GATGATGTCGGTGTCACTAAAATTCACGGTGAC
---------CTGAATGTGGGCCAAGGTTTGGGCGTCGAACTCTTCATCACGTTTATCTTG
GTGCTGACCGTGTACGCA---GTGACCGACGCCAATCGCACC------------------
---GATATCAAG---------------GGTTCAGCTCCTCTGGCTATTGGCCTCGCCATC
GCCATGTGCCATTTGTTCGCCGTTCCGCTTACAGGCTCTGGCATGAATCCTGCACGAGCG
TTCGGGCCTGCTCTCATTTATAAC---------AGTTGGGACGATCACTGGGTCTACTGG
GTGGGGCCTATTCTGGGAGGCATCGCTGCGGGTCTACTCTACCACCTTCTGCTCAGTGCC
---CCACCACCACCT------------------CAATCG---TCCATTGTAGAC------
------------------------GATCGT---------CCGTTGCTT------CTAATG
AGACGG---TCC------CCTGCCAAATTCCCT---------GAA------TCCAGGAGC
---GGAGATGCAACC---
---GTG--

>GERX01041167_Eupolybothrus_tridentinus_PripLA
ATGAGT--

------------------------------GGGCGTTAT---GACCGC------------

---CTC
CGGATTATTCTGGGTGCGCGTGAAATATTG---GACAATCGC------GATTTGTGGAGG
GCGCTACTGGCCGAATTCGTCGGTACGTTGTTCCTCGTATTAGTAGCTTGTGGTGGTTGT
ATTCGC--
--AGCGGTGGAGAC
CTGGTGCATATCGCATTAGCATTTGGTGTGACTGTAGCTACAATCGCTCAATGTATCGGA
CACGTAAGCGGAGGTCACCTCAACCCAGCGGTCACCTGCGGCATGTTGGTTGCCGGTAAA
GTCAGCGTCGTCCGAGCCCTGTTTCACATCATCTCCCAATGCGTAGGCGGAATCGTCGGC
GCGGCCCTTCTCAAAGCCCTTACTCCTGAACCCAAACTACAG------------------
---------------------------GGGAATTTATGCATGACCACACTGGCTCCTAAC
---------GTAAACGTGGCCCAAGCGTTCGGTGTAGAATACCTCATCACATTTGTGCTC

GTTCTGACTGTGTTCGGC---GTATGCGACGAATTCCGGACT------------------
---GATGTGAAA---------------GGTTCCGCCCCACTCGCAATAGGACTTTCTATC
AGCACATGCCACCTGTTTGCGGTGCCATACACTGGGTCAAGCATGAATCCAGCTCGTACG
TTCGGCCCTGCTGTCATGACTGGC---------ATCTGGACTGATCATTGGGTTTACTGG
CTCGGTCCCTGTCTAGGAGGCATCAGTGCTGCCGTCATCTACCAACTAGCGTTCCGGGCA
---CGGGCGCCCAAA------------------------CCCGCGACCGAACCG------
------------------------GAA------------GTACCCCTT------CTGCAC
CACAGA---CGG------GCCCCTTCACCCCCG------AAAGAG------GAAGTCACT
--AACAGGATGACG
GCTATT--

>GCAY01036619_Lithobius_forficatus_PripLA
ATGAGT--

------------------------------GGACGATTC---GACCGA------------

---CTC
CGACTTGTGTTGGGGGCCCGAGAGGTGCTG---GACAATCGG------AACTTATGGCGC
ATGTTGTTGGCCGAGTTTGTCGGGACCTTGTTCCTCGTCTTAATCGCGTGCGGCGGATGC
ATTCGT--
--AGCGGCGGGGAC
ATCGTGCACATCGCCTTGGGCTTCGGCGTCACCGTCGCCACCATCGCCCAATGCATCGGA
CACGTTAGCGGCGGTCACCTCAACCCAGCTGTCACCTTCGGTATGTTGGTGGCCGGCAAA
GTGAGCCTGGTTCGAGCCCTCTTCTACATCATCTCTCAGTGCGTGGGAGGTATTGTGGGG
GCGGCTCTGCTCAAAGCCCTCACCCCCGAAGCCAAGCTGCAA------------------
---------------------------GCCAATCTGTGCATGACGACCCTGGCTCCGCAC
---------GTCAATGTCGCCCAGGCGTTCGGAGTCGAGTTTCTCATCACCTTCGTCCTC
GTCTTGACCGTCTTCGGC---GTCTGCGACGGATTGCGATCT------------------
---GACGTCAAA---------------GGCTCGGCCCCCCTCGCCATCGGGCTCTCCATC
GCCACGTGCCACCTGTTTGCGGTTCCATACACGGGATCAAGTATGAACCCGGCTCGTACT
TTTGGGCCAGCAGTTATGACAGGC---------ATTTGGGATAACCATTGGATCTATTGG
CTTGGTCCTTGTCTTGGAGGAATCAGTGCTGCAGTTATTTACCAATTAGCCTTTCGAGCA
---CCAAGACCAATA---------------------------GTTGAGGAGCCA------
------------------------GAA------------GTTCACACT------CTTCTC
CACCGG---AGG------GCGCCATCTCCTCCT------AAAGAA------GAAGTTATC
--AACCGGACGACT
GCCATT--

>GEAZ01023082_Craterostigmus_tasmanianus_PripLA
ATGGCT--

------------------------------GGA---GCT---TCTCGA------------

---ATT
CGCAATATTATAGGAATTAACGAAATTACT---ACCAATCGG------GACATATGGAAG
GCTCTGTTGGCCGAGTTCTTGGGCACGATGTTTCTGGTGTTTATCGGGTGCGGTTCCTGC
ATC---AACCTCATGCAAGTA---------------------------------------
---CAAGTTGAT
ATCGTGCAAATATCCCTGGCTTTTGGCATTGCGGTAGCCACCATAATCCAATGCATAGGG
CACATCAGCGGGGGTCACATCAACCCGGCCGTCACGTGCGGGATGTTCGTCGCCGGTCGT
GTGAGCCTTATCCGTGCTCTGTTCTACATAATATCGCAGCTAATCGGGGCGATCGTGGGT
GCCGCCTTGCTCAAGTCCATCACGCCGCCGAAGTACATGGGT------------------
------------------------------AACTTGGGGCTTTCGCTCGTGACCCCAGGG
---------ATAGAACCCGTTCAGGCATTCGGAGTGGAATACCTCATCACGTTCGTGCTG
GTCTTGACCGTATTCGCA---GTGACCGACGCAAATCGTGGG------------------
---GACCTTAAG---------------GGATCCGCTCCTCTGGCCATTGGGTTGTCCGTT
ACAGTTTGCCATCTGTTTGCCATTCAATACACCGGAGCAAGCATGAATACAGCTGGCACA
TTTGGACCAGCGGTCATTACTGGA---------ATTTGGGACGATCATTGGGTATATTGG
GTTGGGCCACTCGGAGGTGGTTTTTCGGCTGGAATCCTCTACCACTACGTGTTCAAGGCG
---CCAGAACCGGTG------------------------------GTACGCGAC------
------------------------GAA------------CCCTCCTTA------CTG---
------------------AGAGAT---GGC---------AAAGAA---------GTCCCC
---CTCGACAGGACGACA
TCCATA--

>GCAQ01036274_Scutigera_coleoptrata_PripLA
ATGAGT--

------------------------------------AAT---TCGCGA------------

---GTG
AAGGAGTTCATCGGAGTGTCGGAGGTGATG---TCGAACCGC------GACTTGTGGAAG
GCCCTGATCGCCGAATTCGTGGGTACACTTTTCCTCGTTCTGATCGGCTGCGGTTCTTGC
ATACGGAAC---
---CCCGGCGAT
TTGGTACGGATATCGTTGGCCTTTGGTATCACCGTTGCTACCATGGTACAGTGTATCTGT
CATGTCAGCGGTGGTCACATCAACCCTGCCGTCACATGCGCTATGCTTGTCACAGGGAAA
GTGTCCGTACTTCGGGCAATCTTTTACATCATCTCGCAATGTATTGGAGCCATCGTAGGA

TCAGCCCTGCTCAAGGCTTTACTTCTTGATGAGGACCAAGGC------------------
------------------------------AATTTGGGCATGACTGTGGTTACGGAAGGC
---------ATGTCTCCAGTCAACGGATTCGGAATCGAATATCTCATCACTTTCGTACTC
ATCTTCACCATTTTCGGT---GTATGTGATGGAAACCGTTTA------------------
---GACATCAAA---------------GGTTCAGCCCCTCTTGCCATTGGTCTGGCTGTC
GCAACTTGCCACCTGTTTGCTATTCCCTTCACTGGATCCAGCATGAACTCGGCGAGGACA
TTTGGACCTGCAGTTATTACCGGG---------ATCTGGGATCATCACTGGGTTTACTGG
TTGGGTCCATGCCTAGGAGGGATGTCGGCCGCCATTCTTTACCATTACGTGTTTACTGCG
---AAGCCCGCTGTA------------------------------GTGGATCCC------
------------------------GAA------------CCAAGTTTA------TTGTGC
AAAAGA---AAC---TACGACGACAAGGACATG------AAAGAG---------------
---GCC---------ACT
TGC---

>GESA01013889_Haploglomeris_multistriata_PripLA
ATGGGC--

---------------------------------AAGACC---AACAAG------------

---TTT
CGTTTGGCACTGGGCGTTGACGAAGTGACC---AGCAACACT------GAAATCTGGAAG
GCCCTAACAGCTGAATTCCTCGGTACCCTCATCTTGGTTCTTGTGGGATGTGGCTCTTGT
TTAAAGAACTGGGGCACTGAC---------------------------------------
--ACTCCTCCATCC
ATTGTTGAAATCTCGCTTGCATTTGGACTGACCGTTGCTGTGATGGTACAGTGCATAGGT
CACGTGAGTGGTGGACACATCAATCCTGCAGTTACCTGTGCCATGTTGGTGGCTGGTAAA
GTGAGCCTAATCAGGTCTATACTCTACATACTATCGCAATGCATTGGGGCCATTGCTGGA
GCTGCCATCCTAAAGGCCATCACACCACCAGAATTCCAGAGA------------------
------------------------------TCCCTTGGAACACCACAAGTTGACGGCAAA
---------ATTAGCCCTGTCCAGGGCTTTGGCGTTGAGTTCCTCATCACCTTTGTACTG
GTGCTTACAGTTTTTGCT---GTTTGTGACCAGTACCGTGGT------------------
---GACAACAAG---------------GGATCTGCTCCAATCGCCATTGGACTTTCTGTT
GCCACATGCCATCTGTTTGCGATAAAATACACTGGTTCCAGTATGAACTCTGCTCGAGCC
TTTGGACCAACTGTAATCTCCGGC---------CTGTGGAACGATCACTGGGTTTACTGG
CTTGGTCCATGTTTGGGTGGAATGGCAGCAGGAATCCTCTACCATTACGTCTACAAGGCT
---CCATTACCAGTA-------------------------CAAGAGGAAGACCAT------
------------------------GAA------------CCTGCTCTT------CTATCA
AAATAC---AGA------AATGACAAAGGTTCA------AAAGAA---------GTTGTT
---GGAGACCGTACAACA
TGCATA--

>GERY01037200_Glomeridella_minima_PripLA2
ATGGGC--

---------------------------------AAGACC---AACAGG------------

---TTT
CGTTTGGCACTGGGTGTAGATGAAATAACC---AGCAACACT------GAAATCTGGAAA
GCCCTTACAGCCGAATTCCTTGGTGTCCTCATCTTGGTTCTTGTGGGATGTGGCTCTTGT
ATAATGAACTGGGGCACTGAC---------------------------------------
--ACAGCACCATCC
ATGGTTGAAATCTCTCTTGCATTTGGACTGACCGTTGCTGTGATGGTGCAGTGCATAGGT
CATATAAGTGGTGGACACATCAACCCTGCAGTCACCTGTGCCATGCTGGTGGCTGGTAAA
GTGAGCATCATCAGGGCTATACTCTACATACTATCACAATGCATTGGGGCCATTGCTGGA
GCTGCCATCCTAAAGGCCATCACACCGGAAAATTTTCAAAGA------------------
------------------------------AGCCTGGGAACACCACAAGTTGACAAACTA
---------ATTAGCCCTGTCCAGGGCTTTGGGGTTGAGTTCCTGATTACCTTTGTACTG
GTGCTTACAGTGTTTGCT---GTTTGTGACCAGAACCGTGGT------------------
---GACAACAAG---------------GGGTCTGCTCCAGTTGCCATTGGACTTTCAGTT
GCCACATGTCACTTGTTTGCGATCAAATACACAGGTTCCAGTATGAATTCTGCTCGAGCC
TTTGGACCAACTGTAATCACCGGC---------AAATGGGACCATCACTGGGTTTACTGG
CTTGGGCCATGTTTGGGTGGTATGGCAGCAGGAATCCTCTACAACTACGTTTACAATGCT
---CCATCACCTCCA---------------------------CAAGAAGAATAT------
------------------------GAA------------CCTGCTCTG------CTTTCA
AAATAC---AGA------AATGACAAAGGTTCT------AAAGAA---------GTTGTT
---GCAGACCGTACCACA
TCCATA--

>GAKW01039298_Glomeris_pustulata_PripLA

GCCCTAACGGCTGAATTCGTCGGTGTCCTCATCTTGGTGCTTGTGGGATGCGGCTCTTGT
ATACTGAACTGGGGCAGTAAT---------------------------------------
--ACACCTCCATCC

ATTGTTGAAATCTCTCTTGCATTTGGACTCACCGTTGCTGTGATGGTACAGTGCATAGGG
CATGTGAGTGGTGGACACATCAACCCTGCAGTTACCTGCGCCATGTTGGTGGCAGGTAAA
GTGAGCCTAATCAGGGCTATACTCTACATTCTATCACAATGCATTGGGGCCATTGCTGGA
GCTGCCATCCTAAAGGCCATCACGCCACCAGAATACCAGAAA------------------
------------------------------ACCCTTGGAACACCACAAGTTAACGATGAA
---------ATCAGCCCTGTCCAGGGCTTTGGCGTTGAGTTCCTCATCACCTTTGTACTG
GTGCTTACAGTTTTTGCT---GTTTGTGATCAGTACCGTGGT------------------
---GACAACAAG---------------GGATCTGCGCCCATTGCCATTGGACTTTCTGTT
GCCACATGCCATCTGTTTGCGATCAAATACACTGGTTCCAGTATGAACTCTGCTCGAGCC
TTTGGACCAACTGTAATCTCCGGC---------AAATGGGACCATCACTGGGTTTACTGG
CTTGGGCCATGTTTGGGTGGAATGGCGGCAGGAATCCTCTACCATTACGTCTACAAGGCT
---CCATTACCACCA---------------------------CAGGAAGATCAT------
------------------------GAA------------CCTGCTCTT------CTATCA
AAATAC---CGC------AATGACAAAGGTTCA------AAGGAA---------GTTGTT
---GGAGACCGTACAACA
TCCATA--

>GERY01012811_Glomeridella_minima_PripLA1
ATGGGC--

---------------------------------AAGACA---AACAAA------------

---TTT
CGTTTGGCACTTGGTGTTGATGAACTGACC---AGCAATACT------GAAATCTGGAAG
TCCCTGACTGCAGAGTTTATCGGTGTCCTCATCCTGGTCCTTGTTGGCTGTGGCTCTTGC
ATCCTCAACTGGGGCAGTAAA---------------------------------------
--ACTCCACCATCC
ATTGCTGAAATCTCTCTCGCGTTTGGACTGACAGTTGCAGTGATGGTCCAGTGCATTGGT
CACGTGAGTGGTGGACACATAAACCCTGCGGTTACCTGTGCTATGCTGGTTGCTGGCAAG
GTGAGCATCCTTAGGGCCATCCTGTACATTGTGTCCCAGTGCATCGGTGCCATAGCTGGA
GCTGCCATCCTCAAGGCCATCACTCCTGAGCAGTACCAGGCT------------------
------------------------------ACCCTTGGCACTCCATCGGTCAATCCGGAC
---------ATCAGCCCTGTGCAGGGTTTCGGTGTGGAATTCCTCATCACATTTGTCCTG
GTCCTCACAGTCTTCTCA---GTTTGTGATGGCAACCGTGGC------------------
---GACAACAAG---------------GGATCTGCTGCAATTGCCATTGGACTGTCGGTT
GCCACATGCCATCTGTTTGCGATCAAGTACACTGGTTCCAGTATGAACTCTGCCCGTGCT
TTTGGCCCAGCTGTAATCTCAGGC---------GGTTGGAAGGATCATTGGGTTTACTGG
CTAGGACCATGCCTTGGTGGAATGGCTGCAGGAATCCTCTACCATTATGTGTACAAGGCC
---CCATTGCCTCCA---------------------------CAAGAAGAATCG------
------------------------GAA------------CCAGCTCTT------CTATCC
AAGTACCACCGC------AATGACAAGGGTTCG------AAAGAA---------GTTGTC
---GGAGACCGTACAACA
TCCATA--

>GESN01006855_Polyzonium_germanicum_PripLA
ATG---

------------------------------------GCT---CGCAAT------------

---CTT
CGCGTGGTCATCGGTGTCCAAGAGTTCAGCGCCGGTGGCAGA------GATATCTGGAAG
GCGCTTCTGGCCGAGTTCCTCGGAGTCCTCTTCCTCGTCTTCATAGCCTGTGGGTCGACG
CTGCAA---TGGGACAAAACA---------------------------------------
--CAGCCCCCTACT
CACGTCCTCATCTCGCTCGCTTTCGGGCTCACCGTCGCCACCGTGGTCGCCTGCATAGCG
CACGTCAGCGGTGGTCACGTCAATCCCGCTGTCACTCTCGCCATGCTCGTGGCCGGCAAA
GTGAGTGTTCTGCGAGCGCTGCTCTACATCCTGGCGCAATGCGTCGGCGGCGTGGTCGGC
GCCGCGCTGCTCAAAGCGGTCACCCCGCCGCAGGGTCACGGA------------------
------------------------------AATCTCGGACTGACCACGCTCGGACCGCTC
---------GTCGAACCGGTCCAGGCGTTCGGCATCGAGTTTCTCATCACGTTCGTGCTT
CTCTTCACCGTGTTCGCC---GTCTGTGATGGCAACCGAATC------------------
---GATCATAAG---------------GGTTCCGCTCCGCTCGCCATCGGCTTGGCCGTC
GCCACCTGTCACCTGTTTGCCGCGAAATACACCGGACCTAGCATGAATCCAGCTAGAACG
TTTGGTCCAGCAATTATGTCCGGC---------ATCTGGGATAACCATTGGGTTTATTGG
CTTGGCCCTATTTTGGGTGGCATCGCTGCCGGTGTTCTCTACCAGTACGTTTTCAAGGCA
---CCCAGAGTGATC------------------------------ATGGAACGA------
------------------------GAC------------GTCCCACTG------CTGACC
ATC------CGA---AACAACGACAAGGACGGC------AAAGAG---------GTTGTG

---GCCGACAGGACCACC
TCAATT--

>GERS01004845_Craspedosoma_sp_PripLA
ATGGGT--

---------------------------------CGCACC---AAGAGC------------

---TTC
CGATCGGTGGTTGGGATCGACGATCTTACG---GCCAACAAG------GACATCTGGAAA

```
GCTCTGATTGCCGAATTTCTCGGCGTCTTCTTTCTGGTGCTGATCGGCTGCGGATCCTGT
CTGCAG---TGGGAAAACACG---------------------------------------
------------------------------------------------GGTCCCCCCTCC
ATTGTCCAGATCTCTCTGGCCTTCGGAGTGACGGTGGCCACCCTGGCCCAGTGTATCGGC
CACGTCAGTGGCGGTCACATCAATCCGGCAGTCACCTGCGGCATGCTGGTGGCCGGTAAG
GTCAGCATCCTGCGAGCCCTGTTCTACATCATCTCCCAGTGTGTCGGGGGCATCACCGGA
GCGGCGGTGCTCAAGGCTGTCTCCCCTGTCGACGGTCAGGGC------------------
------------------------------AACCTGGGCATGACGCTGGTCAACAAGGAC
---------TTGAGCCCCGTGCAGGGTTTCGGAGTGGAGTTTCTCATCACCTTTGTCTTG
ATTTTCACCGTCTTTGGA---GCCTGCGACGGCAACAGGAAC------------------
---GACGTCAAG---------------GGTTCGGCACCTCTGGCCATTGGACTGGCTGTC
GCCACCTGCCATTTGTTTGCGATCAAATACACCGGATCAAGCATGAATTCCGCCAGAACT
TTTGGACCAGCAGTGATCAGCGGA---------ATCTGGGATCACCACTGGGTCTACTGG
CTCGGACCATGCCTGGGAGGAATCGCAGCGGCCATCTTGTATCAGTACGTGTTCCAAGCC
---CCGGAGCGACAA------------------------------ATCAGTAAC------
------------------------GAC------------GCACCCCTG------CTCACC
ATCAAT---CGC------AAGGAATACAACGAC------AAAGAA---------GTAGTC
---------------------------------------------GCCGACAGAACGACG
TCCATC------------------------------------------------------
------------------------------------------------------------
------------------------------------------------------------
------------------------------------------------------------
------------------------------------------------------------
------------------------------------------------------------
------------------------------------------------------------
------------------------------------------------------------
------------------------------------------------------------
------------------------------------------------------------
------------------------------------------------------------
------------------------------------------------------------
------------------------------------------------------------
------------------------------------------------------------
------------------------------------------------------------
------------------------------------------------------------
------------------------------------------------------------
------------------------------------------------------------
------------------------------------------------------------
------------------------------------------------------------
------------------------------------------------------------
------------------------------------------------------------
------------------------------------------------------------
------------------------------------------------------------
------------------------------------------------------------
------------------------------------------------------------
------------------------------------------------------------
------------------------------------------------------------
------------------------------------------------------------
------------------------------------------------------------
```

```
------------------------------------------------------------
------------------------------------------------------------
------------------------------------------------------------
------------------------------------------------------------
------------------------------------------------------------
------------------------------------------------------------
------------------------------------------------------------
------------------------------------------------------------
------------------------------------------------------------
------------------------------------------------------
>GESI01021388_Polydesmus_complanatus_PripLA
ATGAGT------------------------------------------------------
------------------------------------------------------------
---------------------------------CGCGGC---AAACGTTCA---------
------------------------------------------------------------
---------------------------------------------------------GCT
CGCCAGATAATTGGCTTAGATGATCTCTCT---CCAAAGGCT------ATGATATGGAAG
GCTTTGATAGCCGAATTTTTATCCGTCTTTTTTCTGGTCTTAATCGGCTGCGGATCGTGC
TTGAAA---TGGGACTCAACG---------------------------------------
------------------------------------------------CCGGAGGTATAC
ATCGTCCAAATCTCATTAGCCTTTGGTGTGACGGTGGCCACGTTGGCTCATTGCATAGGA
CACATAAGTGGAGGACACATTAATCCAGCCGTCACCTGCTCCATGCTAGTGTGTGGAGAG
GTTAGCATCTTACGAGCGTTTTTTACATCCTTTCGCAGTGCATCGGTGGAATCACCGGA
GCCGCCGTTTTAAAAGCGGTCACGCCGGCTCACGGTGCGGGA------------------
------------------------------AACCTCGGCATGACCACAATCAACGAACAC
---------CTTTCGCCAGTGCAAGGATTTGGGGTAGAATTTCTCATCACCTTCGTCTTA
GTTTTCACCGTTTTTGGG---GCTTGTGACTCGAATCGAAAT------------------
---GATGTCAGA---------------GGTTCTTTTCATCTGGCCATCGGTCTTTCGGTA
GCCGTATGTCATCTTATGGCCATTAAATACACTGGCTCTAGCATGAATCCAGCACGAACT
TTTGGCCCAGCGGTTATTACTGGA---------ATATGGGATCACCACTGGGTATATTGG
TTGGGACCAATTCTAGGTGGAATTGCAGCTGGTGCACTTTACAAGTACGTCTTTGAAGCA
---CCAGAAGCCAAA------------------------------CCTCGCAAC------
------------------------GAA------------GTTGGGGAG------GATCCC
GCAGAA---GAG---TTGCAACGCAAGAATGGA------AAAGAA---------TTAGTG
---------------------------------------------GAGGCTCGAACGACA
ACCATG------------------------------------------------------
------------------------------------------------------------
------------------------------------------------------------
------------------------------------------------------------
------------------------------------------------------------
------------------------------------------------------------
------------------------------------------------------------
------------------------------------------------------------
------------------------------------------------------------
------------------------------------------------------------
------------------------------------------------------------
------------------------------------------------------------
------------------------------------------------------------
```


>GCIX01017304_Ommatoiulus_sabulosus_PripLA
ATGGGT--

---------------------------------CGTGCA---AGTGGA------------

---TTT
CGTGCCGTTTTGGGAGTGCAAGAATTCACG---AACAATCGC------AACATCTGGAAG
TCGTTGGTGGCCGAGTTCCTGGGCGTGTTCTTCCTCGTCCTGATCGGCTGCGGATCGTGC
CTCCAG---TGGGAGCCCTCCTCC------------------------------------
--CAAACCCTGCCGGTGTCC
ATCGTCCAGATCGCCCTGGCCTTCGGGATCACCGTGGCCACCCTGGCCCAATGCATCGGC
CACGTGAGCGGAGGCCACATCAACCCGGCGGTCACCATGGCCATGCTGGTCACCAGCAAG
GTGTCCATCCTCAGGGCGCTGCTCTACATCACCTCGCAGTGCTTGGGAGGCATCACCGGG
GCGGCTGTGCTCAAGTCGGTGACCCCCTCCGAGGGTCAGGGG------------------
------------------------------TCGCTCGGACTCTCCACCGTCAACTCGACC
---------ATCAGCCCTGTGCAGGCCTTTGGCGTGGAGTTCCTCATCACCTTCGTGCTC
GTCTTCACCGTCTTTGGG---GCCACCGATGGCAACCGGATG------------------
---GACGTCAAG---------------GGGTCGGCGCCTCTGGCCATTGGACTGTCCGTT
GCCGTGTGCCACCTGTTCGCAATCAGATACACGGGATCCAGCATGAATCCAGCTCGCACC
TTCGGTCCAGCAGTCATCACTGGA---------CTTTGGGAGCATCATTGGGTGTATTGG
TTGGGCCCATGCCTGGGTGGAATTGCTGCCGGAGTTTTATATCATTTCGTCTTCCAAGCC

---GAGGCCCAGAAG------------------------------GTGAACACC------
------------------------GAT------------GTCCCACTG------CTGGCC
ATCAACAGGAAGGACGCCAACGACAAGGACCCT------AAGGAA---------GTTGTA
---GCCGACAGGACGACA
TCCATT--

>GESM01015020_Polyxenus_lagurus_PripLA
ATGGGC--

---------------------------------AAGGGC---GGTCGT------------

---GTG
CGCTACGCGCTGGGGGTCCACGAGTTGTCC---TCCAACAGG------GACATCTGGAAG
GCGCTGATGGCCGAGTTCCTGGGTGTGTTGTTCCTGGTGCTGGTGGCCGACGGCGCCTGC
CTCAAGAACTGGGGCAGCCCCCAC------------------------------------
--CAGGACCCGTCC
ATCGTCGAGATCGCCCTGGCGTTTGGGGTGACCGTGGCCACGATCGCCCAGTGCATCGGC
CATGTCTCCGGGGGCCACATCAACCCGGCCGTCACCCTGGGCATGCTCGTCACCGGCAAG
GTGTCCCTCATCCGCGCCCTGCTCTACATCATGTCCCAGTGCATCGGGGGCATCGCTGGT
GCGGCCCTTCTGAAGATGCTGACGCCGGCCAACTACGAGCAG------------------
------------------------------ACGCTGGGCATGACCCAGGTGAATCCCTTG
---------ATCTCGCCGGTGCAGGGCTTCGGCGTCGAGTTCTTCATCACGTTCGTGCTG
GTGCTGACCGTGTTCGCC---GTGTGCGACGAGAACCGCACT------------------
---GACGTCAAG---------------GGTTCGGCCCCGCTGGCCATCGGCCTGTCCATC
GCCACTTGCCACCTCTTCGCCATCAAGTACACCGGTTCGAGCATGAACTCGGCCAGGACA
TTCGGCCCCGCGGTCATCACGGGC---------ATCTGGGAGCACCACTGGGTGTACTGG
ATGGGTCCGTGTCTGGGCGGCATGGCTGCCGGCATCCTCTACGAATACGTGTTCAAGGCA
AAACCGGTGGAG---------------------------------ACCGAGACC------
------------------------GAG------------CAGACGTTG------CTGTCC
GGCTAC---AGG---GGC---GAGAAGGACCCC------AAGGAG---------GTGGTG
---GCCGACAGGACCACG
TCCATC--

>GCIU01044233_Eudigraphis_takakuwai_PripLA
ATGGGC--

---------------------------------AAAGGG---AGCAAA------------

---GTG
CGATACGCCCTGGGCGTCCAGGAGCTGGCC---TCCAACCGG------GACATTTGGAAG
GCTCTGATGGCCGAGTTCCTGGGTGTCCTTCTGCTGGTGCTGGTGGCCGATGGGGCCTGT
TTAAACACGACGGATGGCGTT---------------------------------------
---GGGGCGTCT
CACGTGGCCATTGCGCTGGCCTTTGGCGTGACAGTGGCCACGATTGCCCAGGGAATTGGC
CATGTCAGTGGGGGACACATAAACCCGGCCGTGACGCTGGGCATGCTGGTCACCGGCAAT
GTGTCCCTGATCAGAGCCGTGTTCTACATTATCTCGCAGTGCATCGGGGGCATCGCCGGA
GCGGCCCTGCTGAAGATGCTGGTGCCGACCGGCTACCACGGC------------------
------------------------------ACGCTGGGCATGACCGTGATCAACAAGGAG
---------CTGTCGCCGATCCAGGGCTTCGGCATCGAGTTCTTCATCACCTTCATCCTG
GTGTTCACCGTGTTCGCC---GTGTGCGACGACAACCGCACT------------------
---GACGTCAAG---------------GGGTCGGCGCCCCTCGCCATCGGACTCTCCATC
ACCACCTGCCACCTCTTCGCCATCAAATACACGGGTGCCAGCATGAACTCCGCCAGGACG
TTTGGCCCAGCCGTGATCACCGGC---------ATTTGGGAGCATCATTGGGTCTACTGG
ATGGGCCCCTGCCTGGGAGGTATCGCCGCCGGCATCCTGTACCAGTACGTGTTCAGAGCG
CAACCGCAGCCCGCC------------------------------CCCGACCGC------
------------------------GAA------------ACGGCCCTG------CTGACT
GGCTAC---AGG---GGCGCCGACAAGGACCCC------AAGGAA---------GTGGTC
---GCCGACAGGACCACC
TCCATC--

>GCHC01030352_Hanseniella_sp_PripLB1
ATGGGA--

---TTC------------

---TGC
GACAAAGTAATCGGGCTTGATGAAGCTTTAGCGAACAAAGAT------CACGTCTTTCGA
TGTGCAGCAGCCGAGTGTTTAGGTGTTTTTTTTATTATAGTCATCGGTTGCGGTTCTTGT
ATCCAAGGATGGGAAAATTCC---------------------------------------
--GAAGCTCCGACT
TTGACGCAAATTTCATTAGCTTTTGGTTTAACCGTAGCTACCGTAATACACTGCACAGGT
CACGTTAGTGGGGGTCATGTTAATCCAGCGGTTACGCTTAGCATGCTCGTCGTAGTTAAA
ATCACTTTCGTAAGAGCTGCATTGTATATCGGAGCGCAACTTTGCGGAGCCATTGTTGGA
GCAGCAGTTTTGCGGGCTCTTACACCGGATGATTTGAAGGGT------------------
------------------------------TCCCTAGGTCAGGTTTCGGTCGATCCTTTA
---------TTGACGACGCTGCAAGGATTCTTTGTCGAGTGTACGTTAACATTTTGTTTG
GTGATTACCGTTTGTGCT---GTTACGGATAAGAACAGGAAC------------------
---ACTGACGGA---------------ATCTCCCCATCTTTCGCCATCGGATTGACGGTA

ACGGCGATTCATTTATTCGGTATAAAATACACAGGAGCTAGCGTAAACCCGGCTCGGGCA
TTCGGGCCCTGCATTGTGACAGGG---------CATTGGCATAGTCATTGGGTCTACTGG
TTCGGGCCATGTCTTGGTGGTCCATTGGCCTCGATTCTTTACCACAAGCTGTTCGAAGGA
---GTAAGTCCATTA---------------------------------CCAAGA------
------------------------TCT------------AACTCAGAT------CTTTCT
GACCTG---------------GAG------------GAA---------------------

>GCHC01040201_Hanseniella_sp_PripLB2

ATGGGA--

---------------------------------CGA---------CGC------------

---ATT
GACCGAGTTCTCCGGCCTTCGGACGTAGTGGGACGTGGACGT------GAAGTTTGGGCT
GCCCTAGTTGCCGAATTTATCGGACCTTTCTTCATCGTTTTGGTAGGCTGCGGATCTTGC
GTCCAAGGATGGGAGGAAGCGGAC------------------------------------
---GCGGTCAAC
AAAGTCCAAATTGCCCTTGCCTTTGGTCTAACGGTGGGTACCTTAGTTCAATGTGTTGGA
CATGTCAGCGGAGGACATTTGAACCCGGCGGTGAGCTTGGCAATGCTGGTGACCGGACGA
ATTGGACTTCTCAAGGCAATCCTGTACATCATCGCCCAGTGTCTTGGTGCGATTGCCGGC
GCTGGAGTACTGAAGGCTTTGACTCCTGACTCCTTGCATCAT------------------
------------------------------GCCCTTGGACAAACGACGGTTAATCCTAAG
---------CTGAATCCTGTGCAAGGATTTGGTGTGGAATTCCTCATTACCTTCGTTCTT
GTGTTTACCATCTTCTCA---GTGATTGATGAAAACAGAGTA------------------
---GACATCACT---------------GGATCAGTACCTTTGGCTGTCGGCTTGGCCGTT
GCAACATGCCATCTTTTTGCCATTCAGTACACTGGTTCCAGTATGAACTCTGCCCGTGCT
CTGGGACCTTCTGTCATCACCGGA---------ACATGGGAGCACCACTGGGTTTACTGG
TTGGGACCCTGCGGAGGAGGTATTGCAGCCGCCATTCTTTACCAGACCACGTTCTCTGCC
---AAACGTCGCGTC---------------------------------ACCGAG------
------------------------ACT------------CATGATGAG------TACGCT
TGTCCT---CCA---GGC---GAGAAG---GAC---GAGAAGGAG---------GCCCAC
AAA--TTGGTGCCT---GCA
GACAGTGGTTGC--

>GERZ01016798_Hanseniella_nivea_PripLB2
ATGGGA--

---------------------------------AGA---------CGC------------

---GTG
GACCGAGTACTCAGGTCTTCGGAAATCGTCGGAAAAGGACGC------GAAGTGTGGGCT
GCCCTCGTCGCCGAGTTTATCGGCCATTCTTCATCGTCCTGGTCGGCTGTGGTTCCTGC
GTTCAGAATTGGGAGGAATCGGAT------------------------------------
---GTGTTCAGC
AGAGTTCAAATCGCCCTGGCGTTCGGTTTGACCGTTGCAACGCTAGTACAATGCGTTGGA
CACATCAGCGGAGGACACCTAAACCCTGCTGTGAGCCTGGCTATGCTTGTAACCGGCCGA
ATCGGACTTCTGAAGGCACTTCTCTACATCATCGCTCAGTGCTTGGGCGCCATCGCTGGT
GCCGGGGTCCTGAAGGCCCTGACCCCTGACGAGCTCCAAGGG------------------
------------------------------AGTCTCGGACAAACCACGGTGGCCAAGAGC
---------TTGAATCCGGTGCAAGGATTCGGAGTGGAATTCCTCATCACCTTCGTGCTG
GTCTTCACCATCTTTTCG---GCCATCGACGAGAACCGAACT------------------
---GATGTTACT---------------GGATCCGTGCCGCTTGCCATTGGACTGGCTGTG
GCTACCTGCCATCTCTTTGCTATCAAGTACACTGGCTCCAGCATGAACTCCGCTCGTGCT
CTCGGACCCGCGGTCATCACCGGA---------ACCTGGACTCACCACTGGGTGTACTGG
CTCGGACCCTGCGGAGGCGGTATCGCGGCGGCCATCTTGTACCAGACAACTTTCTCCGCC
---AGGCGTCGGGTG---------------------------------ACCGAG------
------------------------TCG------------CACGAGGAG------TACGTG
GCGCCC---CCT---GGA---GACAAG---GAG---GAGAAGGAA---------GCGCAC
AAG--CTGATGCCC---GCG
GACAGTGGGTGT--

>GCIH01021882_Symphylella_sp_PripLB
ATGGGA--

---------------------------------CGC---------CGT------------

---TTC
AATACCGTACTCCGACCGGCCGATGTCGTAGGAAGGGGCAAG------GAAGTCTGGTCT
GCCCTTGTTGCCGAATTTCTCGGGCCCTTTTTCATCGTGTTGGTCGGCTGTGGATCCTGC
GTCCAAAACTGGGAGAACAAGGATAAC---------------------------------
---GCCGATCCACTTGAT
TTGGTCCAAATCGCCCTCGCCTTTGGTCTTACCGTTGCAACGCTGGTCCAGGCGGTTGGC
CACATCAGTGGAGCTCACCTCAACCCGGCCGTCACCCTGGCAATGGTGATCACGGGCAAG
ATAGGGCTCCTCAAGGCCCTTCTGTACGTGATTGCCCAGTGTCTGGGCGGCATTGCAGGA
GCGGGGGTCCTGAAGGCCCTCACTCCGGAGGTTCTCCATGCT------------------
---------------------------CGCGGGCTGGGACAAACGGTAATCAACCCTTCC

```
---------CTGAACCCGGTCCAAGGATTCGGCGTCGAGTTTCTCATCACCTTTGTTCTG
GTCCTGGTCGTCTTCTCC---CTGACTGATGAGGGAAGGTCT------------------
---GATATCACC---------------GGTTCGGTTCCCTTGGCAGTTGGAATCTCCATT
GCTATGTGCCATCTGTTCGCAATCAGGTACACCGGCTCCTCGATGAACCCTGCCCGTTCT
CTGGGACCAGCAATCATCACCAAC---------GTCTGGGAACATCACTGGGTCTACTGG
CTTGGCCCCTGCACCGGTGGCGCACTGGCAGCTATCCTCTACCACACCACCTTCAACGCT
---AACCGACGGCAG------------------------------GCCATCCCA------
------------------------CGC------------GGTGACGAG------TACGCT
GTAACC---GCA---CAGGTCCCA------GAC---ACGAAGGAA------------GAG
AAG------------------------------------------CTGATGCCA---GCC
GACAGCGGGTGC------------------------------------------------
------------------------------------------------------------
------------------------------------------------------------
------------------------------------------------------------
------------------------------------------------------------
------------------------------------------------------------
------------------------------------------------------------
------------------------------------------------------------
------------------------------------------------------------
------------------------------------------------------------
------------------------------------------------------------
------------------------------------------------------------
------------------------------------------------------------
------------------------------------------------------------
------------------------------------------------------------
------------------------------------------------------------
------------------------------------------------------------
------------------------------------------------------------
------------------------------------------------------------
------------------------------------------------------------
------------------------------------------------------------
------------------------------------------------------------
------------------------------------------------------------
------------------------------------------------------------
------------------------------------------------------------
------------------------------------------------------------
------------------------------------------------------------
------------------------------------------------------------
------------------------------------------------------------
------------------------------------------------------------
------------------------------------------------------------
------------------------------------------------------------
------------------------------------------------------------
------------------------------------------------------------
------------------------------------------------------------
------------------------------------------------------------
------------------------------------------------------------
------------------------------------------------------------
```


>AFFK01013573_Strigamia_maritima_PripLB
ATGAAT--

---------------------------AGCAGCAGGTCG---AATTTA------------

---GCC
GACCGAATAATTGGTTATTCGGAAGCGAAATCACAACCACGC------GATCTCATCAAG
GCACTTTTTGCCGAATTTATTGGGACGCTCATTCTTGTACTTGTTGGTTGCGGTTCTTGT
GTATCA---TGGGCC---
---CCAGAAGAT
CTGGTGCAGATTGCTCTGGCATTTGGATTGACCGTTGCCGTAATGATTGTCTACATCGGG
CATGTCAGTGGAGGGCACATCAACCCTGCAGTTACCGTGGGAATGCTCGTGACCGGTAAA
ATCAGCGTGCTGCGTGGCCTTTTGTACATCATTGTCCAGAGTTTGGGCGCTATCGCCGGG
GCTGGGATCCTGAAAGGACTCACGCCCAGCGGATTGCACGAA------------------
---------------------------GCTGGACTTGGAGCTCCTGGATTAAACAAAAAT
---------GTGGAAATTGCACAGGGACTCGGCATCGAATTTTTTGTTACCTTCATTTTA
GTTTCGGTTGTTTTTGCC---GCTTGTGATCCTAATCGAACT------------------
---GATGTCAAA---------------GGCGTAGCACCGTTTGCCATTGGGTTGGCAGTA
ACGACATGTCACTTACTTGCGGTTAAATACACTGGCTCCGGAATGAATCCAGCTCGTTCT
TTTGGCCCAACTGTTTTTTCGGGA---------GAGTGGGAGAACCATTGGGTATATTGG
CTTGGACCATGCGCGGGCGGAATGACAGCTGGCATTTTATACCATGCGATATTTGCTGAA
---AAACCGTCCGAT------------------------------TTTGGAAAA------
------------------------GAA------------AATGTGCAA------TTTATG
CAGCTG---AAG---ACTCATGACGGA---------GACAAGGAG------GGTGCTTCT
AAAGGT---------------------------------------CTCATGCCTGCTGGC
GACGCGTGT---

>GESK01020588_Strigamia_acuminata_PripLB
ATGAAT--

---------------------------AGCAGCAGGTCG---AATTTA------------

---GCA
GAACGAATAATTGGTTATTCGGAAGTAAAATCCCAACCACGC------GATCTTCTCAAG
GCACTTTTTGCTGAATTTATTGGGACTCAAATTCTTGTATTCATTGGTTGCGGTTCTTGC
GTCGCA---TGGGCC---
---CCAACAGAC
CTGGTTCAGATTTCTTTGGCTTTTGGATTGACCGTCGCCGTGGTGATTTGTTACATCGGC
CATGTGAGCGGAGGGCACATCAACCCTGCAGTTACCGCGGGAATGTTAGTGACCGGTAAA
ATCAGCCTGCTCCGTGGCCTTTTGTACATCATTGTCCAGAGTTTAGGAGCGGTCGCCGGC
GCGGGAATCTTGAAGGGACTCACGCCCAGCGGACTGCACGAA------------------
---------------------------CTTGGTCTTGGAACCACGGGATTAGCCACAACT
CACGAA---TTGGGCGTTGCACAAGCGCTCGGCGTCGAGTATTTTATTGCCTTCATTTTA
GTTTCCACGGTTTTTGGA---GTTTGCGATCCGAATAAACCG------------------
---GAGCTCAAA---------------GGGGCAGCACCTTTTGCCATTGGGTTAGCAGTA
GCCGCATGTCACTTGATGGCGATCTCATACACTGGCTCCAGTATGAATCCAGCTCGTTCT
TTCGGCGCAGCTGTTTTTGCAGGC---------AAATGGGAAGACCATTGGGTTTATTGG
CTCGGACCATGCGTGGGCGGAATGACAGCTGGCATTTTATACCAAACGATATTTACAGCA
---AAACCAGTCGAT------------------------------TTTGGAAA-------
------------------------GAT------------AATGTTCAA------TTTATG
CAGCTG---AAG---ACTCATGACGGT---------GACAAGGAA------GGTGCCTCT
AAAGGT---------------------------------------CTCATGCCTGCTGGC
GACGCATGT---

>GESB01028044_Henia_illyrica_PripLB
ATGAGT--

---------------------------GGCAGCAGATCA---AATTTA------------

---GTG
GAGCGAATTGTCGGTTTCAATGAAGCTAAATCGCAACCAATT------AACCTACTCAAA
GCTCTCTTCGCTGAATTCGTTGGAACTCTTGTTCTCGTACTTATCGGTTGCGGTTCTTGC
GTTTCA---TGGGCC---
---AAGATGGAC
TTGGTACAGATCGCTTTAGCATTCGGAGTGACTGTCGCAACGATGGTTCAATGTATCGGA
CACGTCAGCGGAGGGCATATCAACCCGGCAGTTACAATGGGAATGTTGGTAACCGGCAAA

ATCTCGATTATACGTGGCCTTTCGTACATCATCGTCCAAGTTTTAGGAGCCTTAGCTGGC
GCTGGCATTCTAAATAGGCTTACCCCCGGTGCTTTACACGAC------------------
---------------------------GATGGTCTTGGCGAGACGGGATTAGGAAACGGT
---------ATTGAACCGGCGCAAGGACTTTGCGTCGAATTTTTCATTACTTTCGTCTTG
GTTTTGACTGTTTTTGCT---GTTTGTGATGAAAATCGAACT------------------
---GATGTCAAA---------------GGTTCGGCGCCACTGGCGATCGGTTTGGCTGTG
GCAACAAGTCATTTATTTGCCATTAAATATACTGGCGCCGGTATGAATCCAGCCCGTGCT
TTTGGTCCGGCTGCTATAGCGGCA---------AAATGGGATGATCATTGGCTTTACTGG
ATTGGACCAATAGTGGGCGGCATGACAGCTGGAGTATTATACCAGATGATATTTGCAGCT
---AAAAAAGACGAT------------------------------ACCAGGAAT------
------------------------CAT------------GATGATCAA------TTTATA
CAACTC---AAG---ACTCATGAT------------GATAAGGAA---GATGGCGTT---
AAAGGT---------------------------------------TTCACGAGT---GGC
GACGCATGC---

>GCAH01014304_Clinopodes_flavidus_PripLB
ATGAAC--

---------------------------AGAAGCAACTCC---AATTTA------------

---GCG
GAACGGATTCTCGGTTTTAACGAAGTGAAATCGCAACCGCGC------GATTTACTCAAG
GCACTTTTTGCCGAATTTGTTGGGACGCTCCTCTTGGTACTTGTCGGTTGCGGTTCTTGC
GTTACC---TGGACCAAAGCAGAC------------------------------------
---------------------------------GCGGAAAAGCCACCGCCACCGGCAGAC
TTGGTGCAAATCGCCATTGCTTTCGGCGTGATCATCGCAACAATAGTCCAATGCATCGGT
CACGTGAGTGGAGGACACATCAATCCTGCGATTACATTGGGGCTGCTAGTGGCCGGCAAA
GTCACCTTGATCCGTGCACTTCTGTACGTCGTCGTCCAATGTTTAGGTGCCATCGCCGGT
GCTGGGATTCTTAAGGGACTCACTCCGCCCGACTTTCATTAC------------------
---------------------------TACGGTCTTGGTGAAACCAAGTTAGGCCAGGAT
---------GTGGACATCGTGCAAGGATTGGGCGTCGAGTACTTTATTACGTTTATTTA
GTTTTGGTTGTTTTCGGT---GTTTGCGATGGGAACCGAACA------------------
---GATGTCAAA---------------GGGTCAGCTCCTCTTGCCATAGGCTTGGCTGTG
ACTGCGAATGCCATGTTTGCGATTAAGTACACGGGTTCGAGCATGAATCCAGCGCGTTCG
TTCGGACCAGCTATTTTCACAGGA---------GTATGGACGATCATTGGGTTTACTGG
CTCGGACCAATCGTGGGTGGTATGACAGCTGGCATTTTGTACCACTCGTTATTTGAAGCA
---AAACCAGTCGAT------------------------------TTTGAAAAG------
------------------------GAT------------AATGTTCAA------TTTATG
CAACTC---AAG---ACTCACGATGGT---------GACAAGGAG------GGTGCCTCT
AAAGGT---------------------------------------CTCATGCCT---GGC
GACGCATGC---

>GCIL01013487_Himantarium_gabrielis_PripLB
ATGAGT--

------------------------------AACGCGTCA---ACCAGA------------

---GCA
GAGCGAATCGTCGGATTCAAAGAAGTGAAGGAAAAGCCACGT------GACCTGATCAAG
GCATTGTTTGCCGAATTTATGGGGACCATGTTCCTCGTGCTCGTCGGCTGTGGCTCATCC
ATCGCA---TGGTCCAAA--
---CCGGTCGAT
TTGGTTCAAATCTCTTTGGCAGCCGGCATTATAGTGGCTACTATGATTCAATGCACCTGT
CACATAAGCGGGGGACACCTCAATCCCGCCGTTACGAGCGGAATGCTAGTTGCCGGCAAG
ATGTCGCTTTTGAAGTCTTTGCTGTACATCGTCGTTCAAATTTTGGGCGCTATCACCGGC
GCGGGAATCCTAAAGGGCGTCATTCCATCTTCTCTTCACGAC------------------
---------------------------GACGGCCTCGGGCAAACCGTTCTCGACAAGGAT
AGTGAT---GTGGACGTGGTTCAAGGATTGGGCATCGAATTTTTTGTCACTTTCGTCCTC
GTGTTCACCGTGTTCGCC---GTTTGCGACGAGAACCGCAAA------------------
---GACATTAAA---------------GGATCGGCTCCCTTCGCGATTGGTCTCGCCGTG
ACAACTTGCCACTTATTCGCGATTAAACCCACCGGTTCCAGTATGAATCCAGCTCGTTCC
ATTGGACCTGCAATTGTAACTGGT---------GTTTGGGATGATCACTGGGTTTATTGG
TTGGGTCCGCTCGTCGGTGGAATGACGGCTGGAATTATTTACCAAACCATTTTCGCAGCT
---GAGCCATCCGAA---------------------------------GCCAAA------
------------------------GAC------------AACTCGTAT------TTTATG
CATCTG---AAG---GGGCAAGATGGT---------GATAAAGAA------GGAGCGTCA
AAGGGT---------------------------------------CTCATGCCA---GGA
GATACGTGTTGT--

>GESL01011069_Schendyla_carniolensis_PripLB
ATGAGC--

---------------------------TCCAGCACCCAA---AACGCG------------

---GTG
GAGAAAATTCTGGGCTATGCAGAAGTGAAAGAAAAGCCCAAG------GACCTGGTGACC
GCCCTCTTTGCCGAATTCGTGGGCACCCTGTTCCTGGTTCTCGTGGGCTGCGGCTCGTGC
GTCAAC---TGGTACGTCAAAGACCAG---------------------------------

---GACAGCAGACCCGAC
ATAGTGCAGATCGCCCTGGCCTTCGGGGTGACCGTCGCGACCATGGTCCAATGCATAGGG
CACGTGAGCGGAGGGCACATCAACCCGGGGGTCACCTGCGGGATGCTGGTGGCCGGCAAG
ATCTCTCTTCTCAAGGCCGTCCTGTACGTGATTTTCCAGAGTCTGGGCGGAATGGCAGGC
GCGGGGGTGCTCAAGGGGCTGACCCCCGCCGGACTCCACGGT------------------
---------------------------GCCGGAATAGGGGTGACGAAGGTGAATTACGAT
GACGAC---GTCGACGTGGTTCAGGGACTCGGGGTGGAGTTCTTCATCACTTTCGTTCTG
GTGTTCGTCGTGTTCGGG---GTGTGCGATTCGAATCGGAAC------------------
---GACGTCAAG---------------GGGTCCGCGCCGTTGGCCATCGGATTGGCGGTT
GCGACCTGTCATCTGTTTGCGGTGAAATACACAGGTTCTAGTATGAATCCAGCTCGATCG
GTTGGTCCTGCCATTGCCTTTGGC---------ATTTGGACGGATCACTGGATTTATTGG
TTGGGTCCCTGTGTGGGTGGCATGACAGCTGGAATTCTCTATCAATTCATATTCTCTGCC
---AAACCACTTGAA------------------------------CCTGGAAAG------
------------------------GAT------------GGTGTTTAT------TTCATG
CATCTC---AAG---ACACATGATGGA---------GAAAAGGAA------GGAGCTTCA
AAAGGA---------------------------------------CTCATGCCAGGAGGA
GACACATGCTGT--

>GHBZ01000487_Scolopocryptops_sexspinosus_PripLB
ATGTCG--

---------------------------TCCACGACCGCC---AACCGG------------

---CTC
GACCGCATCCTCGGCTACCGCGACCTGGCCGAGACGGGCCGC------CACATCTGGAAG
GCCCTGCTCGCCGAGTTCGTCGGCACCCTCTTCCTCGTGCTCGTGGGCTGCGGCTCGTGC
GTCGAGGGCTGGAAAAGGGC--
--CACATCAGCCAG
ACCGTGGGCATCGCCCTGGCCTTCGGCATCGCCGTCGCCACCGTCGTCCAGTGCGTGGGC
CACGTGAGCGGCGGCCACGTCAACCCGGCCGTCACCATGGGCATGCTCGTCACGGGCAAG
ATCGCCCTGCTGCGCGCCCTGCTCTACGTGGCGGCGCAGGTGGTGGGCGCCATCGCCGGG
GCCGGCATCCTCAAGGCCCTGACCCCCGACCCGCTGCACGGC------------------
------------------------------ACCATCGGCGTCACCAAAGTCAACCCCTTG
---------CTCAACCCGGTGCAGGGGTTCGGCGTCGAGTTCCTCGTCACGTTCGTGCTC
GTGCTCACCGTCTTCGCC---GTGTGCGACCCCAACCGGATC------------------
---GACGTCAAG---------------GGATCGGCCCCGCTCGCCATCGGCCTCTCCGTC
GCCGGATGCCACCTCCTGGCCATCAAATACACCGGCGCAGCAATGAACTCGGCACGAGCC
ATTGGCCCCTCGGTCGTCACCGGA---------TATTGGGATCACCATTGGGTCTACTGG
CTGGGACCGTGCTTGGGCGGCATGACCGCGGGCATCCTCTACCAGACGTCGTTCGCGGCC
---CAGGCTCCGGAC------------------------------GAAGGCAAG------
------------------------GAA------------GGCGTCTAC------TTCATG
CAGCTC---AAA---TCTCGCGACGGCGTCGCCTCCGAAAAGGAA------GGCGCCGAG
AAGGGA---------------------------------------CTCATGCCC---GGC
GACACTTGTTGC--

>GCIY01028236_Scolopocryptops_rubiginosus_PripLB
ATGTCG--

---------------------------TCCACGACCGCC---AACCGG------------

---CTC
GACCGCATCCTCGGCTACCGCGACCTGGCCGAGACGGGCCGC------CACGTGTGGAAG
GCCCTGCTCGCCGAGTTCGTCGGCACGCTCTTCATCGTGCTCGTGGGCTGCGGCTCGTGC
GTCCAGGGCTGGGAGAAGGGC---------------------------------------
--CACACCAGCCAG
CTGGTGGGCATCGCCCTGGCCTTCGGCTTCGCCGTCGCCACCGTCGTCCAGTGCCTGGGC
CACGTGAGCGGCGGCCACGTCAACCCGGCCGTCACCATGGGCATGCTCGTGACGGGCAAG
ATCGCCCTGCTCCGGGCCCTGCTCTACGTGGCGGCGCAGGTGGTGGGCGCCATCGCGGGG
GCCGGCATCCTCAAGGCCCTGACCCCGGACCCGCTGCACGAC------------------
------------------------------ACCATCGGCGTCACCAAAGTCAACCCGCAG
---------CTCAACCCCGTCATGGGGTTCGGCGCCGAGTTCCTCATCACCTTCCTGCTC
GTGCTCACCGTCTTCGCC---GTGTGCGACCCCAACCGGACC------------------
---GACGTCAAG---------------GGATCGGCCCCGCTCGCCATCGGAATCGCCGTC
GCTGGATGCCATCTCCTGGCCATCAAATACACCGGCGCAGCAATGAACTCGGCACGAGCC
ATTGGCCCCTCGGTCGTCACCGGA---------TATTGGGATCACCATTGGGTGTACTGG
CTGGGACCGTGCCTGGGCGGCATGACCGCGGGCATCCTCTACCAGACGGCGTTCTCGGCC
---CTGGCTCCGGAC------------------------------GAGGGCAAG------
------------------------GAG------------GGCGTCTAC------TTCATG

CAGCTC---AAG---TCGCGCGACGGCGTCGCCTCCGAAAAGGAA------GGCGCCGAG
AAGGGA---------------------------------------CTCATGCCT---AGC
GACACCTGTTGC--

>GESL01012122_Schendyla_carniolensis_PripLA
ATGCCT--

------------------------------TCC---------AACAGG------------

---GTT

ATCAAATTCCTCGGTTGCAACGAACTGACC---ACCGAAAGG------GACATCTGGAAG
GCCCTTATTGCGGAGTTTGTCGGCACCTTGTTCCTGGTAGTGGTCGGTTGCGGTTCCTGC
ATCCAA---GCGGAAGACAAA---------------------------------------
--CCGGCTCCGAAC
GTGGTCCAGATTGCTCTGGCGTTTGGAGTGACTGTGGGCACGATGGTACAGTGCATATGT
CACGTGAGTGGGGGGCACATCAATCCGGCTGTCACGTGCGCAATGCTGGCCACGTGCAAA
GTGTCCTTGGTTCGTGCCCTGTTCTACATAATATGCCAGTGCGTCGGTGGCATTACGGGA
GCGGCTTTGCTGTTGGCTCTCGTGCCGGGTTCCGACAAGCAG------------------
------------------------------CATCTCGGCCAGTCCGAAGTGAACGTCAAG
AATAAT---GTAACCGAGATCCAGGCTTTCGGCATCGAGCTTCTCATCACTTTTGTGCTG
GTGTTGACGGTGTTTGCG---GTCTGCGATGGCAATCGTGCC------------------
---GACGTCAAG----------------GGCTCCGCACCCTTGGCCATAGGTTGGCCGTC
GCGACTTGCCACTTGTTTGCGGTTCCGTTAACGGGATCTAGCATGAATCCGGCTCGAACC
TTGGGTCCAGCAGTAATAGGAAAT---------TATTGGACGAGTCATTGGGTATATTGG
GCAGGACCGTGCTTGGGTGGAATTGCAGCTGGTCTACTCTACCAGTTAGCCTTCAGGGCT
---CCATCACCACTC---------------------------------CTC---------
---------------------------------------AGTGTCAGT------AGACTC
GAATCA---TTT---AACAAG---------------GAAAGGAA----------CATCTA
AAACCA---------------------------------------GCA------------

>GCAP01016001_Scolopendra_cingulata_PripLB
ATGAGT--

---CGC------------

---GTG
GATCGAATTCTCGGGTACAAAGAGTTGCAGGAGACAGGAAAA------GACATTTGGAAA
TGTCTCTTAGCGGAGTTTCTAGGGACCCTATTCCTCGTGCTCATCCCATGTGGATCGTCC
GTCGAT---ATAAATACAACC---------------------------------------
--GGCACCATCGAT
ATCGTCCAAATCGCTTTGGCTGCCGGGATTACGGTGGCCACCATAGTCTTATGCATAGCC
CATGTGAGCGGGGGTCACATCAACCCGTCTGTCTCACTTGCGATGCTCGTCACGGGCAAG
ATGGCCCTACTCAAGGCCCTTCTCTACATCATGGTCCAAGTGTTGGGAGGGATCGTCGGT
GCGGCCATACTCAAATCCGTGACCCCGAGTGATTTTCACGGT------------------
------------------------------CACCTCGGCCAGACACTTGTGAACCCCAAA
---------CTGGACCCCATCCAAGGCTTCGGCGTCGAATTCCTCATCACTTTCATACTC
GTCTTCACCGTATTCGCC---GTCGTGGATGAGAACCGAACA------------------
---GACCTCAAA---------------GGTTCTGCCCCCTTAGCCATCGGTCTGTGTGCC
GGCGCTTGCCATTTGTTTGCTATTACTTTTACGGGAGCCTCGATGAACTCGGCTCGTTCT
ATTGGTCCAGCTGTGGTGACAGGA---------GCTTGGGAACATCACTGGGTGTACTGG
CTGGGTCCCTGTATTGGGGGTATGACAGCTGGCATCCTCTACCAGTCGGCTTTTGCAGCC
---AAGATGCCTGAC------------------------------GAAGGCAAA------
------------------------GAC------------GGCGTTTAT------TTCATG
CAACTG---AAA---CAACGAGACGGAGTCGGGGCCGAGAAGGAA------GGGGCCGCC
AAAGGG---------------------------------------TTGATGCCG---GGC
GACACCTGTTGC--

>GGNE01000500_Scolopendra_viridis_PripLB
ATGAGC--

---CGT------------

---TTG
GACCGTATCCTTGGTTACAAGGAATTGCATGAATCAGGTCGC------GATATTTGGAAA
TGTCTCCTAGCAGAGTTTGTGGGCACGCTCTTCCTCGTGACCGTTGGCTGTGGTTCAGGT
GTCGAC---TGGAGCGAAAAGTCT------------------------------------
--AAGCCAAAAGAGCCCAAT
ATTGTTCAAATCGCTCTGTGCGCCGGGATCGCCGTCGCCACACTCATAAAGTGCATATGT
CACATAAGCGGGGGTCATCTCAACCCGGCAGTAACGTGCGGTATGCTAGTCACCGGAAAA
TTTTCCCTGCTCAAAACCCTTCTCTATATCATTTCCCAAGTGTTAGGGGCAATCGTCGGC
GCGGGGATCTTAAAAGCTCTTACTCCTGATGACATGCAAAAC------------------
------------------------------AATCTCGGCCAGACCCTCGTCAACCCCCGT
---------CTCAATCCTGTCCAAGGCTTCGGAGTCGAATTCCTCATCACTTTCGTGCTC
GTGTTCACTGTGTTTGCT---GTTTGCGATTCCAACCGGACC------------------
---GATCTCAAA---------------GGGTCCGCACCTTTAGCCATCGGCCTTGCCGTC
CTCACGTGCCATATGTTTGCTCTTCCTTATACTGGCTCCTCGATGAACTCTGCTCGTTCT
ATTGGCCCAGCTGTGATTACAGGA---------GATTGGGAACACCACTGGGTTTACTGG

TTAGGACCTTGCATAGGGGGAATGACAGCTGGAATTCTCTACCAATCAGCTTTCGCGGCC
---AAGATGCCTGAT------------------------------GAAGGAAAG------
------------------------GAC------------GGCGTCTAC------TTCATG
CAACTG---AAA---CAACGCGACGGCGTTGCAGCTGAAAAAGAA------GGAGCCACC
AAAGGG---------------------------------------TTAATGCCA---GGG
GACACTTGCTGC--

>GERX01043099_Eupolybothrus_tridentinus_PripLB
ATGGGC--

```
---------------------------------------------CGA------------
------------------------------------------------------------
---------------------------------------------------------TTA
GAGCGATTCCTTGGTTATTCCGAATTAGCCGATACGGGAAGA------CACGTCTGGAAA
GCCTTGCTTGCAGAATTCGTCGGAACCTTGTTTCTCGTTCTCGTAGGCTGCGGCTCCTGC
GTGAGCGGCTGGATGATGAT----------------------------------------
------------------------------------------------TACGCCCCAACG
ATTGTCCAAATATCTTTAGCTTTTGGTGTGACCGTTGCTACGATGGTACAATGCATCGGC
CACGTAAGCGGGGGTCACATCAACCCAGCGGTGACCTGTGCCATGTTAGTGACCGGTAAA
ATGGCCCTATTGCGAGCCCTTCTCTACATCATCGCTCAAGTTTTAGGGGCGATCGTAGGC
GCGGGCATTTTAAAGGCACTCACTCCTGAAGCGATGCAAGAA------------------
------------------------------GGATTGGGTCAAACTCTCGTTCAGTCCAAG
---------CTCAACCCCGTTCAAGGATTTGGAGTGGAATTCCTCATAACTTTCGTCTTG
GTTTTGACTGTTTTTGCT---GTTTGTGATGACCACCGTGCT------------------
---GACTTGAAA---------------GGATCGGCTCCATTGGCTATTGGACTGGCTGTA
GTCACCTGTCACTTGTTCGCCATCAAATACACCGGAGCCAGCATGAACTCTGCGCGCGCT
ATTGGACCAGCAGTTGTGACCGGT---------GACTGGATGCACCATTGGGTCTACTGG
CTGGGTCCTTGCGTTGGTGGCATGACAGCAGGCATCCTGTATCAAGCAGCATTCGTCGCC
---AAACCCCCGGAA------------------------------CACGATAAA------
------------------------GAG------------GATGTCTAC------TTCATG
CAGCTC---AAG---ACTCGTGATGGCGTCGGT---GAGAAAGAA------GGTGCTTCC
AAGGGG---------------------------------------CTCGTC---GCTGGA
GACGCTGGTTGC------------------------------------------------
------------------------------------------------------------
------------------------------------------------------------
------------------------------------------------------------
------------------------------------------------------------
------------------------------------------------------------
------------------------------------------------------------
------------------------------------------------------------
------------------------------------------------------------
------------------------------------------------------------
------------------------------------------------------------
------------------------------------------------------------
------------------------------------------------------------
------------------------------------------------------------
------------------------------------------------------------
------------------------------------------------------------
------------------------------------------------------------
------------------------------------------------------------
------------------------------------------------------------
------------------------------------------------------------
------------------------------------------------------------
------------------------------------------------------------
------------------------------------------------------------
------------------------------------------------------------
------------------------------------------------------------
------------------------------------------------------------
------------------------------------------------------------
```


>GCAY01043079_Lithobius_forficatus_PripLB
ATGGGT--

---CGT------------

---TTG
GAACGCTTCTTGGGTTATTCGGAGTTGGCGGACACGGGTCGT------CATGTGTGGAAG
GCCCTGCTGGCCGAATTTGTGGGTACCTTGTTCCTCGTGCTCGTCGGCTGCGGCTCGTGC
GTTAGCGGCTGGGACGAGAAC---------------------------------------
--TACGGGCCGAGT
ATGGTCCAGATCGCGCTGGCTTTCGGCGTCACCGTCGGCACCATGGTCCAGTGCATCGGC
CACGTGAGTGGGGGCCACATCAACCCGGCCGTCACGTGCGCTATGCTGGTCACCGGTAAG
ATGGCCCTTCTCCGGGCCCTCCTCTACATCATTGCCCAGGTGCTAGGGGCGATCGTAGGG
GCGGGTATCCTCAAAGCCCTCACCCCAGACGATCAGCAGCAA------------------
---------------------------TTTGGCCTGGGTCAGACTTTAGTATCTCCGAAA
---------CTCAACCCCGTCCAAGGATTCGGCGTCGAATTCCTCATCACTTTCGTCCTC
GTCTTGACCGTCTTTGCT---GTTTGTGATGACCACCGAGGG------------------
---GACCTCAAG---------------GGATCCGCCCCCCTCGCCATTGGACTCTCCGTC
GCCACTTGCCATCTCTTTGCCATCAAATACACCGGATCCAGCATGAACTCGGCTCGCGCT
ATTGGACCGGCCGTCGTGACGGGA---------GACTGGGATCACCACTGGGTCTATTGG
CTGGGTCCCTGCGTGGGTGGAATGACAGCAGGCATCCTGTACCAAGCGGCCTTCGTGGCC
---AAACCTCCGGAA------------------------------CACGATAAA------
------------------------GAG------------GATGTCTAC------TTCATG
CAACTC---AAG---ACCAGAGACGGCGTTGGG---GAGAAGGAG------GGGGCGTCC
AAGGGG---------------------------------------CTCGTG---GCCGGA
GACGCTGGCTGC--

>GCAQ01015391_Scutigera_coleoptrata_PripLB
ATGAAT--

---CGT------------

---TTG
GACCGTTTCCTCGGGTACACGGAGCTCGTTGAGTCTCGCCGG------GTCATTTGGAAA
ATGCTTTTAGCGGAGTTCGTGGGGACGTTGTTCCTTGTCCTGGTGGGTTGCGGCTCCTGC
ACGAATGTCATCGGGGAAGAT---------------------------------------
--TATACGCCTACC
GTCACTCAGATAGCGTTTGCGTTCGGTCTCGCCGTGGCTACGATGGCCAGGTGCATCGGA
CATGTGAGCGGCGGACACATCAACCCAGCCGTCACGTGCGGAATGCTTGTCACCGGTAAA
TTGGGTCTGTTGCGAGCGCTGCTCTACATTGCGTCTCAGTGTCTTGGAGCCATCGCCGGA
GCTGCCATCTTACGGGGCCTCATAGTGGACGCTTCCGAAATA------------------
------------------------------GCTCTGGGTCAGACCATGATACAGAAGAAT
---------CTCACCTCGGTTCAGGGCTTCGGTGTGGAATTCCTCATTACCTTCTTATTG
GTTTTCACGGTGTTTGCT---GTGTGTGACGAACAACGCGCA------------------

```
---GCCGAAGGT---------------GGTTCAGGTGCACTGGCCATTGGTTTGGCGGTG
GTTGCCGGACATCTTATTGGGGTGAGTCTGACTGGAGCCAGCATGAACTCAGCTCGAGCG
CTTGGACCGGCTGTACTGACTGGC---------ACCTGGCAACATCATTGGGTGTACTGG
TTGGGTCCATGTTTGGGTGGAATGGCAGCTGGAATTCTCTATGAAACAGCTTTTAAAGCG
---AAATCCCCCGAA------------------------------GAAGATAAG------
------------------------AAC------------GGC---TAT------TTCATG
CAATTG---GCC---ACTCGAGAACCCACCGCC---GAGAAAGAA------AGCGCT---
------------------------------------------------------------
------------------------------------------------------------
------------------------------------------------------------
------------------------------------------------------------
------------------------------------------------------------
------------------------------------------------------------
------------------------------------------------------------
------------------------------------------------------------
------------------------------------------------------------
------------------------------------------------------------
------------------------------------------------------------
------------------------------------------------------------
------------------------------------------------------------
------------------------------------------------------------
------------------------------------------------------------
------------------------------------------------------------
------------------------------------------------------------
------------------------------------------------------------
------------------------------------------------------------
------------------------------------------------------------
------------------------------------------------------------
------------------------------------------------------------
------------------------------------------------------------
------------------------------------------------------------
------------------------------------------------------------
------------------------------------------------------------
------------------------------------------------------------
------------------------------------------------------------
------------------------------------------------------------
------------------------------------------------------------
------------------------------------------------------------
------------------------------------------------------------
------------------------------------------------------------
------------------------------------------------------------
------------------------------------------------------------
------------------------------------------------------------
------------------------------------------------------------
------------------------------------------------------------
------------------------------------------------------------
------------------------------------------------------------
------------------------------------------------------
```

>GERZ01014197_Hanseniella_nivea_PripLA
ATGGGG--

--CAGAAA------------

---ATG
AAGAATCTACTGGGAATTAACGAGCTGAAG---ACGAACAAG------GACGTATGGAAG
GCCCTACTAGCCGAGTTCCTCGGAGTACTGTTCCTAGTACTGATAGGCTGCGGAGCCTGT
ATTATCGGCTGGGAGAAAGAG---------------------------------------
--AGTTCTCCCAGT
AACAACGACATCGCCTTCACCTTCGGTCTGATGGTCGCCACCATGGTCACCTGCATCGCT
CACGTTAGCGGGGGTCACATCAACCCTGCCGTAACGACGGGTATGCTGGTCGCGGGTAAG
ATATCTCTCCTCAAGGGAATCTTCTACGTGTTGTCTCAGTGTATCGGGGCTGTAACTGGG
GCCGCGATCCTCAAGGCACTCACCCCTGCCGAGGTACAGGCT------------------
------------------------------AACCTGGGCCTGTCCACCGTGTCCCCTCGA
---------ATCAGCCCCGTGCAAGGGTTCGGAGTCGAATTCCTCATCACCTTCATTCTC
GTGTTCGTCGTGTTCGCG---ATCTGCGACGAGAACCGAGCC------------------
---GATCTCAAG---------------GGATCCGGACCTCTCGCTATCGGTCTCGCCATC
ATCGCCTGTCATCTCATGGCGATTAAGTACACCGGTTCGAGTATGAATCCTGCCAGGACA
TTTGGACCAGCCCTCGTATCCGGA---------GTCTGGCAAGACCACTGGGTCTACTGG
CTCGGTCCCTGCCTCGGAGGAACCGCCGCCGGAATTCTCTACGAACACGTGTTCCAGGCC
---AAGCCACTCGGA------------------------------CCGGAACCA------
------------------------GAA------------CCGGCCCTC------CTCCAG
CAGTACCACAAGAAACCGGAGAATTCCAGCAACAGCGACAAGGACATGAAGGAAGTGATC
---GCCGACAGGACGACG
TCCATA--

>GCIL01019162_Himantarium_gabrielis_PripLA
ATGTGC--

------------------------------TCC---AAA------CGT------------

---ATT
CTCAATTTTCTCGGTTGCAATGAGTTATTG---ACTGACAAA------AAATTATGGCAA
GCTCTCATCGGTGAGTTTTTGGGTACCTTACTTCTCGTTCTGATCGGATGCGGTACGTGT
ATA------ACGGAAACT--
---CAA
GTGGCAGGAATAGCGCTAGCATTCGGGTTGTCGGTGGCAACAATGGTACAGTGTATTTGT
CACGTTAGCGGAGGGCATATCAATCCGGCCGTAACTTTGGGAATGTTGGCGACGTGTAAA
ATAACTTTAATTCGTGCGTTGTTCTACATCATCTCTCAATGCGTCGGTGCAATCGTGGGA
GCTGCGTTACTTAAAGCTGTAACTCCGGAAAAATTTGCAACGGAA---------------
------------------CGGGCAGTGAGAACCCTCGGTCTCACGATGTTGGGGACAAAC
---------GTGACAGTGGCTGAAGCTTTTGGTGTCGAGTTTCTCATCACTTTTATACTC
GTCTTCACCGTGTCTGCT---GTGTGCGATTCGAATCGAACT------------------
---GACTTCAAA---------------GGATCGGCTCCGCTGGCGATTGGACTCGCTGTA
GTTTTATGCCATTTATTTGCGGTAAAATATACGGGATCCAGCATGAATCCAGCGAGGACA
TTTGGTCCAGCATTGATCACTAAT---------ATTTGGACGGACCACTGGGTGTATTGG
GTTGGACCGTGTTTGGGCGGTATCGGGGCTGCAGTTGTGTATCACTTCGCCCTTCGAGCA
---CCGGCGCCACCA---------------------------ACAATC------------
------------------------AAC------------------------------ATT
AGAAAGGATACA---ATATATAACAAAGAC------GAGAAATTC---------TTA---
---CAAGGA------GCT
GATCTT--

>GGNE01000217_Scolopendra_viridis_PripLA
ATGGCC--

------------------ACGACTACCGCCGGTCGATTC---CAGGGG------------

---ATT
TTGCCCGAACTCGGCGTACAGGAGTATCAG---ACCCTC---------GACTTATGGAAG
GCGGCGGTGGCTGAATTCGTCGGAACGCTCCTGCTGGTGCTCATCGCCTGTGGCGCATGT
GCGGTT--
---ACCCCCGAG
CGAGTGGACATCGCCTTGGCTTTTGGCTTCTCTGTGGCGACAATGGTCCAGTGCATCTGT
CATGTGAGTGGGGGACACATCAACCCAGCCGTTACGATAGGACTCCTTGTCACGCGCAAG
GTGACAGTCATCCGGGCTCTCGTGTACGTCATCGTTCAGTGTATTGGAGCCATAATCGGC
GCTGCGTTGATCGAAGCATTTTTACCAGATTAC---------------------------

```
---------------------------GATGATGTTGGTGTCGCTGACACTAAATCC---
---------CTCAATGCGGGCCAAGTATTAGGGGTTGAACTCTTCATCACTTTTATCCTG
GTGCTCACAGTCTACGCA---GTGACAGATGCCAATCGTACG------------------
---GATATCAAA---------------GGTTCAGCTCCCCTCGCCATTGGCCTTGCCATC
GGCATGTGTCATCTGTTCGCTGTACCACTCACAGGCGCTGGAATGAATCCGGCGCGTGCA
TTTGGGCCTGCTCTCATCTATAAC---------AGCTGGAACAATCACTGGGCTTACTGG
ATTGGTCCCTTTGTGGGAGCCATCGCAGCAGGGCTCCTTTACCATCTTGTCTTCAGTGCC
---CCACCGATCACC------------------CAATCGCCCTCTATTGTCGAA------
------------------------GATCGC---------CCTCTGCTT------CTGATG
CGAAGG---TCG------CCGGCCAAATTCCCT---GAA------------ACCAGAAGC
---------------------------------------------ACAGAT------GCC
GCCGTC------------------------------------------------------
------------------------------------------------------------
------------------------------------------------------------
------------------------------------------------------------
------------------------------------------------------------
------------------------------------------------------------
------------------------------------------------------------
------------------------------------------------------------
------------------------------------------------------------
------------------------------------------------------------
------------------------------------------------------------
------------------------------------------------------------
------------------------------------------------------------
------------------------------------------------------------
------------------------------------------------------------
------------------------------------------------------------
------------------------------------------------------------
------------------------------------------------------------
------------------------------------------------------------
------------------------------------------------------------
------------------------------------------------------------
------------------------------------------------------------
------------------------------------------------------------
------------------------------------------------------------
------------------------------------------------------------
------------------------------------------------------------
------------------------------------------------------------
------------------------------------------------------------
------------------------------------------------------------
------------------------------------------------------------
------------------------------------------------------------
------------------------------------------------------------
------------------------------------------------------------
------------------------------------------------------------
------------------------------------------------------------
------------------------------------------------------------
------------------------------------------------------------
```


>JAFIDM010009631_Thereuonema_tuberculata_PripLB
ATGAAC--

---CGC------------

---CTG
GACCGTTTCCTCGGTTATACGGAGATCGTCGAATCTCGCCGG------GTCATGTGGAAG
ATGCTTCTGGCCGAGTTCGTGGGCACCCTGTTCCTGGTGATCGTGGGCTGCGGCTCGTGC
ACCAACGTGGTCGGCGAAGGT---------------------------------------
--TACATACCGACC
ACCGTCCAGATAGCGTTCGCCTTCGGCCTGGCCGTGGCCACGATGGCTCGGTGCATCGGA
CACGTGAGCGGTGGACATATCAATCCGGCCGTCACGTGCGGCATGCTCGTCACCGGTAAG
CTGGGTCTGCTCCGCGCGCTGCTCTACATCGCCGCCCAGTGTCTGGGAGCCATCGCCGGC
GCCGCCGTACTTCGAGGGCTGGTGGTCGACGGCCCCGAGGTC------------------
------------------------------GCGCTCGGACAGACCACGGTACAGCAGAAT
---------CTGACGTCCGTCCAGGGTTTTGGAGTCGAATTCCTCATCACCTTCATGCTC
GTCTTCACCGTGTTTGCT---GTGTGCGACGAAAAGCGC---------------------
---GCTCCCGAG---------------GGATCAGGAGCCCTGTCTATCGGACTGTCCGTG
GTTGCCGGTCATCTGGTTGGGGTTAATCTTACTGGAGCTAGCATGAATTCTGCTCGTGCC
CTTGGACCTGCTGTGCTGACTGGA----------GTCTGGCAGATCATTGGGTCTACTGG
CTGGGTCCCTGCTTGGGCGGAATGGCAGCTGGAATCTTGTATGAAACAGCATTTAAGGCA
---AAATCACCCGAA------------------------------GAAGATAAA------
------------------------AAT------------GGA---TAT------TTCATG
CAACTC---GCT---ACCAGAGAACCAGCTCCT---GAGAAGGAA------ACTGCC---

>JAEQBA010003990_Phalangium_opilio_PripL2
ATGGGT--

---------------------------------CGAGTT---AGGAAA------------

---TTT
AGAGCCGTTGTCGGAATTGACGAATTAACC---AGTAATAGT------ACCTTATGGAAA
GCTATGTTAGCCGAATACATCGGTACCGCTTTACTGGTCCTCGTGGCGTGTGGATCATGT
ACCGAT---TGGAAAGACGGG---------------------------------------
--ACCCTACCAGAA
ATGTCCCAAATAGCCCTGGCTTTTGGCTTAATAGTCGGGACCATTGTCCAAGCGATTTGT
CACATTAGCGGCGGACATATCAATCCGGCCGTCACCGCCGGCATGTTGGTGACCGGAAAC
GTTTCGGTCATCAGAGCCTTGTTCTACATCATCAGTCAATGCATGGGTGGAATTACCGGA
GCCGCTCTGTTGAAGGCTTTGACACCGGCGGCCTTTCAAGGT------------------
------------------------------GGTCTCGGTGGAACTGGACTCGGAAAGGAG
---------GTATCGGCTATTCAAGGTTGCGGGGTCGAAGTGCTCATTACCTTCATTTTG
GTCTTCACCGTATTCTCA---GTTTGTGACGCTAACAGGCTG------------------
---GACGTCAGA---------------GGATCAGCCCCATTGGCTATCGGATTATCAGTA
GGGGCTTGTCACATGTTCGCGATCAAATATACCGGCGCCAGTATGAACACGGCCCGATCT
TTTGGACCAGCCGTCATGGGAAAT---------AACTGGGAAAATCATTGGATTTACTGG
GTAGGACCAATTTTGGGTGGCGTCATCGCTGGTGTGTTGTACCAGAACGTTTTCACGGCG
---CCCAAGATCGAC----------------------------------GCTGAA------
------------------------GCC------------TTGGACCGC------GTT---
CGATTG---GCC---GAATTACACCGGCTAAGC---GAAAAGGAG---AAGGAAGTTGTC
---GCTGATAGGACAACG
TCCATC--

>XM_037647046_Rhipicephalus_sanguineus_PripL
ATGGGC--

---------------------------------CGAGTC---CGCCAG------------

---TTT
CGTTCCTTCTGCGGCATCGACGAGGTGTCC---GCCACGAGC------AGCCTGTGGAAG
TCCATGGTGGCCGAATACATCGGGACGGCCGTGATCGTGCTCATCGGCTGCGGCTCGTGC
ATCAAC---TGGGGCTCGCCCGAGAAGCCA------------------------------
---CAGGGCGAGGCCACC
ATGGTGCAGATCGCCATCGGGTTCGGGATCACCGTAGCCAGCATGGTGCAGGCACTTGGC

CACGTGAGCGGCGGGCACCTGAATCCGGCCGTCACCATCGGGATGCTGTTCGTGGGACGC
GTGTCGCTGTTGCGCAGCTTCTTCTACCTGTGCAGCCAGCTCATCGGAGGCATCACGGGC
GCTGCCATCCTGCGCGCAGTCACTCCGGAGGCGCGACACGGG------------------
------------------------------GCTCTGGGCGGCACGTCGCTCGCCGAGGGC
---------GTCACGCCGCTCATGGGTCTCGGCGTCGAGGCCTGCATCACCTTCATCCTC
GTGCTGACGGTGTTCGGC---GTCTGCGACGCCAACCGGCTC------------------
---GACGTCCAG---------------GGCTCGGCGCCGCTGGCCATTGGTCTCTCGGTC
ACCACATGCCACGTCTTTGCGGTCCGATACACGGGAGCAGGCATGAACGTGGCGCGTTCT
TTCGGTCCGGCGGTGATGTCTGGA---------ATGTTTGACGACCACTGGGTCTTCTGG
CTGGGACCCATCCTCGGAGGCGTGATCGCCGCCGTGGTGTACGAGAACGTGTTCCAGGCG
---CCGCCCATCACC---------------------------------ACCGAG------
------------------------GAT------------CTGGACAAG------GCC---
CAGATC---ACG---GTCATGGCCAAGCTGGCC---AACAAGGAG---------GTGGTC
---GCCGATCGCACCACG
TCCATC--

>XM_037427671_Rhipicephalus_microplus_PripL
ATGGGC--

---------------------------------CGAGTT---CGCCAG------------

---TTC
CGTTCCTTTTGCGGCATCGATGAGGTGTCC---GCCACGAGC------AGCCTGTGGAAG
TCCATGGTGGCCGAATACATCGGAACAGCCGTGATCGTCCTCATCGGCTGCGGCTCGTGC
ATCAAC---TGGGGCTCGCCCGAGAAGCCG------------------------------
---CAGGGCGAGGCCACC
ATGGTGCAGATCGCCATTGGTTTCGGTATCACCGTGGCCAGCATGGTGCAGGCATTGGGC
CACGTGAGCGGGGGCCACCTGAATCCAGCCGTCACCATCGGGATGCTGTTCGTAGGACGC
GTGTCGCTGCTGCGCAGCTTCTTCTACCTGTGCAGCCAGCTCATCGGAGGCATCACGGGC
GCTGCCATCCTGCGCGCAGTCACTCCGGAAGCACGACACGGA------------------
------------------------------GCTCTCGGCGGCACGTCACTAGCCGAGGGC
---------GTCACACCGCTCATGGGTCTCGGCGTCGAGGCTTGCATCACCTTCATCCTC
GTGCTCACGGTGTTCGGC---GTCTGCGACGCCAACCGGCTC------------------
---GACGTCCAA---------------GGCTCAGCGCCACTGGCCATCGGTCTCTCCGTC
ACCACATGCCACGTCTTTGCGGTGCGATACACGGGAGCAGGCATGAACGTGGCTCGTTCT
TTCGGCCCGGCAGTCATGTCTGGA---------ATGTTCGACGACCACTGGGTCTTCTGG
CTGGGACCGATCCTCGGAGGTGTGATCGCTGCCGTGGTGTACGAGAACGTGTTCCAGGCT
---CCGCCTATCACC---------------------------------AGCGAG------
------------------------GAT------------CTCGACAAA------GCC---
CAGATC---ATG---GCCATGGCCAAGCTTACC---AACAAGGAG---------GTGGTT
---GCCGATCGCACCACG
TCCATC--

>XM_037726177_Dermacentor_silvarum_PripL
ATGGGT--

---------------------------------CGGGTG---CGCCAG------------

---TTC
CGTTCCTTCTGCGGCATCGACGAGGTGTCC---GCCACGAGC------AGCCTATGGAAG
TCCATGGTAGCCGAGTACATCGGGACGGCCGTGATCGTGCTCATCGGCTGCGGATCGTGC
ATCAAC---TGGGGCTCGCCAGAGAAGCCG------------------------------
---CAGGGCGAGGCCACC
ATGGTGCAGATCGCCATCGGGTTCGGCGTCACCGTGGCCACCATGGTGCAGGCGTTGGGC
CATGTGAGCGGCGGCCACCTGAATCCGGCCGTCACCATCGGCATGCTGTTCGTGGGGCGC
GTGTCGCTGCTGCGCAGCTTCTTCTACCTCTGCAGTCAGCTCATCGGAGGCATCACTGGC
GCCGCCATCCTGCGAGCCGTCACTCCGGAGGCGCGCCACGGG------------------
------------------------------GCCCTGGGCGGCACGTCGCTCGCCGAGGGC
---------GTCACGCCTCTCATGGGCCTCGGCGTCGAAGCCTGCATCACCTTCATCCTG
GTGCTCACGGTGTTCGGC---GTCTGCGACACCAACCGGCTC------------------
---GATGTCCAG---------------GGCTCGGCGCCGCTGGCCATCGGTCTCTCGGTC
ACCACCTGCCATGTCTTTGCGGTCCGGTACACGGGAGCAGGCATGAACGTGGCGCGCTCT
TTCGGTCCGGCGGTGATGTCTGGA---------ATGTTTGACGACCACTGGGTCTTCTGG
CTGGGACCCATCCTCGGAGGCGTGACCGCCGCCGTGGTGTACGAGAACGTGTTCCAGGCG
---CCGCCCATCACC---------------------------------AGCGAG------
------------------------GAC------------CTGGACAAG------GCC---
CAGATC---ACG---GTTATGGCCAAGCTGGCC---AACAAGGAG---------GTAGTC
---GCCGATCGCACCACG

```
TCCATC------------------------------------------------------
------------------------------------------------------------
------------------------------------------------------------
------------------------------------------------------------
------------------------------------------------------------
------------------------------------------------------------
------------------------------------------------------------
------------------------------------------------------------
------------------------------------------------------------
------------------------------------------------------------
------------------------------------------------------------
------------------------------------------------------------
------------------------------------------------------------
------------------------------------------------------------
------------------------------------------------------------
------------------------------------------------------------
------------------------------------------------------------
------------------------------------------------------------
------------------------------------------------------------
------------------------------------------------------------
------------------------------------------------------------
------------------------------------------------------------
------------------------------------------------------------
------------------------------------------------------------
------------------------------------------------------------
------------------------------------------------------------
------------------------------------------------------------
------------------------------------------------------------
------------------------------------------------------------
------------------------------------------------------------
------------------------------------------------------------
------------------------------------------------------------
------------------------------------------------------------
------------------------------------------------------------
------------------------------------------------------------
------------------------------------------------------------
------------------------------------------------------------
------------------------------------------------------------
------------------------------------------------------------
------------------------------------------------------
>XM_029977726_Ixodes_scapularis_PripL
ATGGGC------------------------------------------------------
------------------------------------------------------------
---------------------------------CGCGTT---CGCCAA------------
------------------------------------------------------------
---------------------------------------------------------TTC
CGTAACTTATGCGGCATCGACGAGGTGTCC---GCCTCCAGC------ACCCTATGGAAA
TCGATGGTCGCTGAATACATCGGCACCGCAGTCATCGTCTTGATCGGCTGCGGCTCGTGC
```

```
ATCAAC---TGGGGCTCACCCACGAAGCCG------------------------------
----------------------------------------------CTGGGGAAGCCACC
ATGGTCCAGATAGCCATAGGCTTTGGCATCACCGTGGCCACCATGGCCCAAGCCGTGGGT
CACGTCAGCGGAGCCCACTTGAACCCGGCCGTCACGATGGGCATGCTGTTCGTCGGCAAG
GTGTCCATCTTGAGGTCCTTCTTCTACATCTGCAGCCAGCTGATCGGCGGTATCACGGGA
GCCGCGATCTTGAGAGCGGTGACTCCCGATGCCCGTCACGGT------------------
------------------------------GCGCTCGGAGGAACTGGCCTTGCCGAGGGA
---------GTCACTCCGCTCATGGGCCTGGCGGTCGAGGTATGCATCACCTTCATCCTG
GTGCTCACCGTGTTCTCC---GTCTGCGACACCAACCGACTG------------------
---GACGTCCAA---------------GGGTCTGCACCGCTGGCCATCGGACTCTCGGTG
ACCACGTGCCACGTCTTTGCGGTGCGCTACACCGGAGCCGGTATGAACGTGGCAAGGTCG
TTCGGGCCTGCCGTGATGTCTGGC---------ATCTTTGATGACCACTGGGTGTTCTGG
GTGGGCCCGATCCTGGGCGGCATCATCGCGGCCGTCATCTACGAGAACGTGTTCCAGGCG
---CCGCCCATCACG----------------------------------ACCGAA------
------------------------GAG------------CTGGACAAG------GCC---
CAGATC---ACC---GCCATGGCCAAGCTGGCC---AACAAGGAG---------GTGGTG
---------------------------------------------GCCGACCGGACCACT
TCCATC------------------------------------------------------
------------------------------------------------------------
------------------------------------------------------------
------------------------------------------------------------
------------------------------------------------------------
------------------------------------------------------------
------------------------------------------------------------
------------------------------------------------------------
------------------------------------------------------------
------------------------------------------------------------
------------------------------------------------------------
------------------------------------------------------------
------------------------------------------------------------
------------------------------------------------------------
------------------------------------------------------------
------------------------------------------------------------
------------------------------------------------------------
------------------------------------------------------------
------------------------------------------------------------
------------------------------------------------------------
------------------------------------------------------------
------------------------------------------------------------
------------------------------------------------------------
------------------------------------------------------------
------------------------------------------------------------
------------------------------------------------------------
------------------------------------------------------------
------------------------------------------------------------
------------------------------------------------------------
------------------------------------------------------------
------------------------------------------------------------
```


>GIXX02017729_Ornithodoros_erraticus_PripL
ATGGGC--

---------------------------------CGTGTT---CGTCAA------------

---TTC
CGTTCAGTTTTTGGCATAGATGAGCTGTCA---GCTACGAGC------ACGCTATGGAAA
GCAATGATCGCAGAGTATATCGGCACCGCAGTTCTGGTCTTCATAGCATGTGGCACGTGC
ACCAAC---TGGGGTAGACCT---------------------------------------
--GGAGAACCGAGC
ATCACTCAGATCGCGCTCAGTTTTGCATTCATCGTGGCCACCATGGCTCAAGCCCTGGGT
CACGTCAGCGGAGCCCACATCAATCCTGCGGTAACGATGGGTATGCTGTGCGTCGGACAT
GTTTCCATTCTGCGAGCCTTCTTCTACATCTGCAGTCAGTTGATAGGAGGCATCACAGGA
GCAGCCATCTTGAGGGCCGTGACACCGGAAGATCGGCAGGGA------------------
------------------------------GCGCTGGGAGGCACGAAGCTGTCGGATGGC
---------GTTACCCCGTTCCAGGGCGTTGTCGTTGAGCTCATGATCACCTTCATACTG
GTACTGACTGTGTTTGCG---GTTTGCGACCAGAACAGGCTG------------------
---GACATTCTC---------------GGGTCTGCTCCACTGGCTATCGGTCTGTCAGTC
GGTGCCGGTCATCTTATGGCGATTGGCTACACTGGTGCTAGCATGAACACGGCGCGTACC
TTTGGCCCAGCTGTGATCTCTGGA---------GCATTTGATGACCACTGGGTGTACTGG
CTTGGCCCCATTGGTGGCGGTATCCTTGCCGGATTCGTCTACCAATACGTCTTCTCAGCG
---CCAGCGATAACG---------------------------------AGTGAA------
------------------------GCC------------CTGGACAAG------GCT---
CAGTTG---GCG---TCTCTGGCAAAGTTCAAC---AACAAGGAA---------GTCGTC
---GAAGATCGCACTACC
TCCATC--

>GCJJ01005421_Ornithodoros_rostratus_PripL
ATGGGC--

---------------------------------CGCGTA---CGCCAA------------

---TTC
CGATCCGTGTTCGGTATCGACGAACTGTCA---GCGACGAGC------ACGCTCTGGAAA
TCCATGATTGCGGAGTATATCGGCACGGCCATTCTGGTCTTCATAGCGTGCGGTACATGC
ACCAAC---TGGGGCAAACCA---------------------------------------
--GGAGAACCGAGC
GTCGCTCAGATCGCGCTTAGTTTCGCTTTCATCGTGGCCACCATGGCTCAAGCTCTGGGC
CACGTCAGCGGGGCGCACATCAACCCGGCGGTGACCATGGGAATGCTATGCGTCGGCCAC
ATCTCCATTCTCCGGGCCTTCTTCTATATCTGCAGCCAGCTGATAGGAGGCATCACTGGA
GCCGCCATCTTAAGGGCTGTAACTCCAGAAGCCCGCCAAGGA------------------
------------------------------CTGCTGGGAGGCACGACGCTCACGGATGGA
---------GTGACACCGTTCCAGGGCGTCGTCGTCGAGCTCATGATCACTTTCATTCTG
GTCCTGACTGTCTTCGCC---GTTTGCGACCAGAACAGGCTG------------------
---GACATCCTG---------------GGATCTCCCCCGCTGGCCATCGGTCTCGCGGTT
GGTGCCTGCCATCTCATGGCGATCGGCTACACAGGAGCCAGCATGAACACAGCCCGTACA
TTTGGACCTGCTGTAATCTCTGGT---------GTCTTTGATGACCACTGGGTGTACTGG
CTCGGCCCCATCGGTGGAGGCGTGCTTGCCGGCTTTGTGTACCAGTACGTCCTCTCGGCG
---CCCGCGATAACC---------------------------------AGTGAG------

------------------------GCC------------ATGGACAAG------GCT---
CAACTG---GCG---TGCCTGGCAAAGTTCCAC---AACAAGGAA---------GTCTCC
---GAGGATCACACGACA
GCTATC--

>GDIC01001077_Ornithodoros_turicata_PripL
ATGGGC--

---------------------------------CGTGTA---CGTCAA------------

---TTC
CGCTCAGTATTTGGCATCGACGAGCTGTCG---GCTACAAGC------ACGCTGTGGAAA
TCAATGATCGCCGAGTATATCGGCACTGCCATTCTGGTTTTCATAGCGTGCGGAACGTGC
ACAAAC---TGGGGTAAGCCT---------------------------------------
--GGAGAACCCAGC
ATCACTCAGATCGCGCTCAGTTTCGCTTTCATCGTGGCCACAATGGCTCAAGCTTTGGGT
CACGTCAGCGGAGCTCACATCAACCCGGCGGTAACGATGGGAATGCTATGCGTTGGACAC
GTCTCCATTCTCCGAGCCTTCTTCTATATCTGCAGCCAGTTGATAGGAGGCATCACAGGG
GCAGCCATCTTGAGGGCCGTAACACCGGAAGGCCAGCAAGGA------------------
------------------------------ATACTGGGAGGCACGAAGCTGGCGGATGGA
---------GTGACTCCGTTCCAGGGCGTCGTTGTCGAGCTCATGATCACTTTCATCTTG
GTACTGACTGTCTTCGCT---GTTTGCGACCAGAACAGGCTG------------------
---GACATCCTG---------------GGATCTCCTCCCCTTGCCATTGGTCTCTCAGTT
GGTGCCTGCCATCTGATGGCGATTGGCTACACAGGTGCCAGCATGAACACAGCGCGCACC
TTCGGCCCAGCGGTGATCTCGGGA---------GCGTTTGACGACCACTGGGTGTACTGG
CTTGGACCCATCGGTGGTGGTGTACTCGCCGGCTTCGTCTACCAATACGTCCTCTCGGCG
---CCAGCGATAACT---------------------------------AGCGAG------
------------------------GCC------------CTGGACAAG------GCC---
CAGCTG---GCG---TCTCTGGCAAAGTTCCAC---AACAAGGAA---------GTTGTC
---GAAGACCGCACTACT
TCCATC--

>GFJQ02006139_Ornithodoros_moubata_PripL
ATGGGC--

---------------------------------CGTGTT---CGCCAA------------

---TTC
CGCTCAATATTTGGCATCGACGAGCTGTCG---GCTACGAGC------ATGCTCTGGAAA
TCAATGATCGCCGAATACATCGGCACGGCCATGCTAGTTTTCATAGCATGCGGAACATGC
ACCAAC---TGGGGTCGAGGG---------------------------------------
--GGAGAACCCAGC
ATCGCTCAGATTGCGCTCAGTTTCGCTTTCATCGTGGCAACCATGGCTCAAGCCCTGGGT
CATGTCAGCGGAGCGCACATCAACCCAGCGGTGACGATGGGAATGCTGTGCGTCGGCCAT
ATCTCCATTCTCCGAGCCTTCTTCTATATCTGCAGTCAGCTCATTGGAGGTATCACTGGA
GCAGCCGTACTGAGGGCCGTGACACCAGAAGAGCGCCAGGGA------------------
------------------------------CTCCTGGGAGGCACGGCACTTTCGGAGGGA
---------GTGACACCGTTTCAAGGCGTCGTAGTCGAGCTCGTGATCACTTTCATTCTC
GTCTTGACCGTCTTTGCC---GTTTGCGACCAGAACAGGCTG------------------
---GACGTCCTG---------------GGATCTCCTCCGCTGGCCATCGGCCTCGCGGTT
GGTGCCGGTCATCTTATGGCGATTGGCTACACAGGAGCCAGCATGAACACAGCCCGTACA
TTCGGCCCAGCTGTAATCTCAGGA---------GCCTTCGATGACCATTGGGTGTACTGG
CTTGGCCCCATTGGTGGTGGTATTCTGGCTGGCTTTGTCTACCAGTACGTCCTCTCTGCT
---CCACCAATATCC---------------------------------TTCGAA------
------------------------GCT------------CTGGATAAG------GCC---
CAGCTG---GCG---TGTCTGGCAAGGTTTCAC---AACAAGGAA---------GCCGTC
---GAGGACCGCACCACG
GCCATC--

>XM_018638410_Metaseiulus_occidentalis_PripL
ATGGGG--

---------------------------------AAA------CGAACA------------

---CTG
CAGACTCTATGCGGCGCTTCGGAGGTGTCT---TCCGATCAG------ACGCTCTGGAAG
TGTCTTCTGGCGGAATTCATCGGTACAGCTACATTGGTCCTCATCGCGTGCGGAAGCTGC
TATGGA---GTGACGAGTCCAACA------------------------------------
--TCGGAAGCC---
GTGGTCCGCATCGCCTTCGGCTTCGGCTTGGCGGTGGCTTCAGTAGCGCAAGCCGTTGGT
CACATATCGGGCGGACATCTGAACCCTTGTGTCACAGCAGGCATGCTTGTGGCTGGTAAT
ATCTCATTCGTGAAAGGCTTTTTCTACGTTTGTTCTCAATGCATCGGAGCGATTACGGGT
GCGGCTATCCTGCAAGCTCTAACACCCGCTGGGTTC---GGT------------------
---------------------------------CTCGGGGCAACCTCGTTGGCCAAAGAC
---------GTCACTCCCCTCATGGGGTTCTTCATCGAAGCCACGATTTCATTCGTCCTC
GTCCTGACCGTCTTCGGA---GCGACTGATGGTAACCGACTC------------------
---GATGTTCTT---------------GGCTCAGTCCCTCTGGCCATCGGGCTGTCGATC
ACCGTTTGCCACCTATTCGCAATCCCGCTGACAGGGGCTAGCATGAATCCCGCGAGAACC

TTCGGGCCCGCTCTGATCCTCCAG---------AATTTCGAAAATCATCACATTTATTGG
CTCGGCCCTCTCCTCGGTGGCATCTCTGCTGGGCTCGTGTACCGCTATGCTTTCAGCGCC
---CCCAAACCAACC---------------------------------GTGGAC------
------------------------GAG------------TTGGAACGA------GCCGCT
AGAATG---TGC---TGTCTGGCCAAGATGAGC---GGCAAAGAA---------GTA---
---GAG---GCGTCGTCC
GGAATT--

>XP_018493926_Galendromus_occidentalis_PripL
ATGGGG--

---------------------------------AAA------CGAACA------------

---CTG
CAGACTCTATGCGGCGCTTCGGAGGTGTCT---TCCGATCAG------ACGCTCTGGAAG
TGTCTTCTGGCGGAATTCATCGGTACAGCTACATTGGTCCTCATCGCGTGCGGAAGCTGC
TATGGA---GTGACGAGTCCAACA------------------------------------
--TCGGAAGCC---
GTGGTCCGCATCGCCTTCGGCTTCGGCTTGGCGGTGGCTTCAGTAGCGCAAGCCGTTGGT
CACATATCGGGCGGACATCTGAACCCTTGTGTCACAGCAGGCATGCTTGTGGCTGGTAAT
ATCTCATTCGTGAAAGGCTTTTTCTACGTTTGTTCTCAATGCATCGGAGCGATTACGGGT
GCGGCTATCCTGCAAGCTCTAACACCCGCTGGGTTC---GGT------------------
---------------------------------CTCGGGGCAACCTCGTTGGCCAAAGAC
---------GTCACTCCCCTCATGGGGTTCTTCATCGAAGCCACGATTTCATTCGTCCTC
GTCCTGACCGTCTTCGGA---GCGACTGATGGTAACCGACTC------------------
---GATGTTCTT---------------GGCTCAGTCCCTCTGGCCATCGGGCTGTCGATC
ACCGTTTGCCACCTATTCGCAATCCCGCTGACAGGGGCTAGCATGAATCCCGCGAGAACC
TTCGGGCCCGCTCTGATCCTCCAG---------AATTTCGAAAATCATCACATTTATTGG
CTCGGCCCTCTCCTCGGTGGCATCTCTGCTGGGCTCGTGTACCGCTATGCTTTCAGCGCC
---CCCAAACCAACC---------------------------------GTGGAC------
------------------------GAG------------TTGGAACGA------GCCGCT
AGAATG---TGC---TGTCTGGCCAAGATGAGC---GGCAAAGAA---------GTA---
---GAG---GCGTCGTCC
GGAATT--


```
------------------------------------------------------------
------------------------------------------------------------
------------------------------------------------------------
------------------------------------------------------------
------------------------------------------------------------
------------------------------------------------------------
------------------------------------------------------------
------------------------------------------------------------
------------------------------------------------------------
------------------------------------------------------------
------------------------------------------------------------
------------------------------------------------------------
------------------------------------------------------------
------------------------------------------------------------
------------------------------------------------------
>GHIT01016147_Amblyseius_swirskii_PripL
ATGGGA------------------------------------------------------
------------------------------------------------------------
---------------------------------AAA------CGAACT------------
------------------------------------------------------------
---------------------------------------------------------CTG
CAGACGTTATGTGGCGCTTCAGAGGTGTCT---TCTGATCAG------ACTCTCTGGAAG
TGTCTCCTCGCTGAATTCATCGGCACAGCTATCCTAGTCCTCGTCGCCTGTGGAAGCTGT
TATGGA---GTCGCCGCTCCCACA------------------------------------
------------------------------------------------TCAGAGGCC---
ATAGTTCGCATCGCGTTCGGTTTCGGATTGACGGTCGCGTCAGTGGCGCAGGCAGTGGGC
CATGTTTCTGGCGGTCATCTGAATCCTTGTGTGACCGCGGGCATGCTCGTCGCCGGAAAT
ATTTCATTCATGAAAGGCTTCTTCTACGTCTGTTCACAGTGTATCGGAGCGATCACAGGT
GCGGCCATCCTGCAAGCTCTGACGCCCGCCGGTTTC---GGT------------------
---------------------------------CTCGGGGCGACCTCCTTGGCTAAAGAT
---------GTTACCCCGCTGATGGGATTCTTCATTGAAGCCTCGATCTCGTTCATTCTT
GTTCTGACTGTGTTCGGA---GCCACCGACGGAAATCGTCTC------------------
---GATGTTCTT---------------GGATCGGTCCCGTTGGCCATTGGATTGTCAATT
ACCGTGTGTCATCTTTTCGCTATTCCCTTGACTGGAGCTAGCATGAATCCTGCGAGAACA
TTCGGCCCTGCTCTGATAATGCAG---------AATTTCGAGAATCATCACATTTACTGG
CTCGGTCCACTCCTGGGTGGAATTTCTGCCGGGCTGGTCTACCGTTACGCGTTCAGCGCC
---CCGAAACCCACC---------------------------------GTGGAC------
------------------------GAG------------TTGGAACGT------GCCGCC
AGAATG---TGC---TGTCTGGCCAAGATGAGT---GGCAAAGAA---------GGT---
---------------------------------------------GATAACGGAGTGACA
TCCTTC------------------------------------------------------
------------------------------------------------------------
------------------------------------------------------------
------------------------------------------------------------
------------------------------------------------------------
------------------------------------------------------------
------------------------------------------------------------
------------------------------------------------------------
```


>MNPL01009468_01004526_Tropilaelaps_mercedesae_PripL
ATGGGT--

---------------------------------AAG------CGGACT------------

---CTA
CAAACATTATGTGGTGCCTCGGAGGTGTCG---TCCGATCAG------ACCCTATGGAAA
TGTCTCTTGGCCGAGTTCTTGGGGACAGCCATTTTAGTTTTGATCGCGTGCGGTTCGTGC
TACAGCAAAACTGACGTCGAT---------------------------------------
--TCTCAAGCAACT
GTTGTACGGATAGCCTTCGGCTTCGGCTTGACTGTCGCATCAGTCGCACAGGCTGTCGGC
CACGTGTCTGGTGGACATTTAAATCCGTGCGTAACGGCGGGCATGCTGGTCGCAGGAAAC
ATCTCTTTCGTAAAAGGTTTCTTCTACGTTGCTTCACAATGCATCGGAGCCATCACTGGT
GCGGCCATCTTACAAGCGTTGACACCAGCTGGCAAT------------------------
------------------------------ATGCTGGGTAACACCACGTTGGCTAACAAT
---------GTTTCAGCTCTCATGGGCTTCTTTATTGAAGCTGTCATTTCATTCGTTCTT

```
GTCATGACCGTATTCGGA---GCAACCGATGGAAATCGGCTG------------------
---GACGTTCTC---------------GGATCAGTGCCCCTAGCTATTGGACTCTCTATC
ACCGTCTGCCATCTGTTTGCAGTGCCTCTTACAGGTGCTAGCATGAATCCGGCACGAACA
TTTGGACCCGCTTTGATTTTACAG---------AACTTCGAAAACCATCATATCTATTGG
TTAGGCCCAATGCTCGGAGGCATCGCGGCCGGTCTTATCTACCGTTATGCGTTCTCCGCA
---CCCAAACCTACG---------------------------------GTTGAC------
------------------------GAG------------CTCGAGAGA------GCCGCT
CGCCTC---TGC---TGCTTGGCCAAAATGAGC---GGAAAAGAG---------CCT---
---------------------------------------------GATGGGGGGGTCACG
TCTTTT------------------------------------------------------
------------------------------------------------------------
------------------------------------------------------------
------------------------------------------------------------
------------------------------------------------------------
------------------------------------------------------------
------------------------------------------------------------
------------------------------------------------------------
------------------------------------------------------------
------------------------------------------------------------
------------------------------------------------------------
------------------------------------------------------------
------------------------------------------------------------
------------------------------------------------------------
------------------------------------------------------------
------------------------------------------------------------
------------------------------------------------------------
------------------------------------------------------------
------------------------------------------------------------
------------------------------------------------------------
------------------------------------------------------------
------------------------------------------------------------
------------------------------------------------------------
------------------------------------------------------------
------------------------------------------------------------
------------------------------------------------------------
------------------------------------------------------------
------------------------------------------------------------
------------------------------------------------------------
------------------------------------------------------------
------------------------------------------------------------
------------------------------------------------------------
------------------------------------------------------------
------------------------------------------------------------
------------------------------------------------------------
------------------------------------------------------------
------------------------------------------------------------
------------------------------------------------------------
------------------------------------------------------------
```

>XM_022796360_Varroa_destructor_PripL
ATGGGC--

---------------------------------AAG------CGAACC------------

---CTC
CAGTCCTTGTGCGGCGCCTCTGAAGTGTCT---TCTGATCAG------ACGCTTTGGAAG
TGTCTTCTCGCTGAGTTTATAGGAACGGCCATTCTAGTTCTCATCGCGTGTGGGTCGTGT
TTTAACCGAGTTGACGCTTCG---------------------------------------
--GGTCCTGATAAT
GTCATAAAAATAGCATTGGGTTTCGGCTTGACAGTTGCTTCGGTTGCACAAGCCGTTGGT
CATGTTTCGGGAGGACACTTAAACCCTTGTGTGACTGCGGGCATGCTCGTAACAGGAAAC
ATTTCATTCGTAAAGGGCTTTTTTTACGTCGCTTCGCAATGCATCGGAGCCATTACTGGT
GCAGCAATACTCCAAGCGTTAACGCCTGCCGGCTTCCAG---------------------
---------------------------------CTTGGTAACACTACATTAGGTAAGAAT
---------ATTACACCCCTGATGGGCTTCTTCATCGAGGCTGCCATTTCATTCGTACTC
GTCCTGACCGTGTTCGGG---GCAACCGATACCAATCGGCTG------------------
---GACGTTCTC---------------GGCTCCGTTCCCCTCGCGATTGGACTTTCTATC
ACCGTTTGCCATCTGTTTGCTGTGCCATTAACTGGTTCCAGCATGAATCCGGCTCGGACA
TTCGGACCCGCTCTGATACTGCAG---------GACTTCACGAATCACCACATCTACTGG
CTGGGCCCACTACTTGGGGGCGTCGCTGCAGGTCTGATTTATCGCTACGCCTTCCGCGCC
---CCCAAGCCTAAC---------------------------------GTGGAC------
------------------------GAT------------CTGGAACGT------GCCGTC
CGTCTG---TGC---TCTTTGGCCAAGATGAGC---GGAAAAGAG---------CCC---
---GACGGTGGGGTCACT
TCTTTC--

>XM_022835670_Varroa_jacobsoni_PripL
ATGGGC--

---------------------------------AAG------CGAACC------------

---CTC
CAGTCCTTGTGCGGCGCCTCTGAAGTGTCT---TCTGATCAG------ACGCTTTGGAAG
TGTCTTCTCGCTGAGTTTATAGGAACGGCCATTCTAGTTCTCATCGCGTGTGGGTCGTGT
TTTAACCGAGTTGACGCTTCG---------------------------------------
--GGTCCTGATAAT
GTCATAAAAATAGCATTGGGTTTCGGCTTGACAGTTGCTTCGGTTGCACAAGCCGTTGGT
CATGTTTCGGGAGGACACTTAAACCCTTGTGTGACTGCGGGCATGCTCGTAACAGGAAAC
ATTTCATTCGTAAAGGGCTTTTTTTATGTCGCTTCGCAATGCATCGGAGCCATTACTGGT
GCAGCAATACTCCAAGCGTTAACGCCTGCCGGCTTCCAG---------------------
---------------------------------CTTGGTAACACTACATTAGGTAAGAAT
---------ATTACACCCCTGATGGGCTTCTTCATCGAGGCTGCCATTTCATTCGTACTC
GTCCTGACCGTGTTCGGG---GCAACCGATACCAATCGGCTG------------------
---GACGTTCTC---------------GGCTCCGTTCCCCTCGCGATTGGACTTTCTATC
ACCGTTTGCCATCTGTTTGCTGTGCCATTAACTGGTTCCAGCATGAATCCGGCTCGGACA
TTCGGACCCGCTCTGATACTGCAG---------GACTTCACGAATCACCACATCTACTGG
CTGGGCCCACTACTTGGGGGCGTCGCTGCAGGTCTGATTTATCGCTACGCCTTCCGCGCC
---CCCAAGCCTAAC---------------------------------GTGGAC------
------------------------GAT------------CTGGAACGT------GCCGTC
CGTCTG---TGC---TCTTTGGCCAAGATGAGC---GGAAAAGAG---------CCC---
---GACGGTGGGGTCACT
TCTTTC--

>GCAR01007431_Euscorpius_sicanus_PripL2
ATGGGG--

---------------------------------CGCGTC---CGTCGG------------

---TTC
AGAACCATTTGCGGAATTGACGAGTTGACT---GGAACCAGC------ACATTATGGAGA
GCGCTCATAGCAGAGTATATAGGAACTACCATGTTGGTGCTGGTAGCCTGCGGATCTTGT
ACGATG---TGGAAAGGA--
--GGGGAGCCCACG
ATTACGCAGATAGCTCTGGCCTTTGGTTTCATAGTGGCATCTATGGTGCAATGTATAGCT
CACGTAAGCGGAGGACACATTAACCCGGCTGTTACCTGTGGCATGCTAGTTGCCGGTAGA
CTTTCGATTCTCCGTGCTCTCTTCTATGTCATTTCGCAGTTGCTTGGAGGTATTTCCGGG

```
GCTGCCATTATTAAAGCCGTAACTCCCTACGACTTGCAGGGA------------------
------------------------------AATTTGGGAGCTTCTCTTCCGGCCAAGGAT
---------GTAACGGCGTTCCAAGCATTCGGAGTCGAATTCTGTATCACGTTTATCTTG
GTGTTCACCGTTTTCTCT---GTCTGCGATAACAATCGTTTG------------------
---GACATCACC---------------GGGTCTGGACCTCTGGCAATTGGCTTAGCCATA
GCGGCCTGTCATCTGTTTGCCATCCGATATACTGGAGCCGGGATGAACAGCGCCAGATCC
TTCGGTCCAGCGCTCATTGCCGGA---------AAGTGGAAAGACCATTGGGTGTACTGG
GTTGGACCTATTCTCGGCGGTATAGTGGCAGGGCTTCTCTATCAGCATGTCTTCGTCGCT
---CCACCTCCAAAC---------------------------------CAGGAA------
------------------------GAC------------CTCGACAAG------GAA---
AGATGC---CGG---CAATTCATGCGTTTAAAG---GAGAAAGAA---------GTTGTT
---------------------------------------------TCCGACAGAACAACG
TCAATT------------------------------------------------------
------------------------------------------------------------
------------------------------------------------------------
------------------------------------------------------------
------------------------------------------------------------
------------------------------------------------------------
------------------------------------------------------------
------------------------------------------------------------
------------------------------------------------------------
------------------------------------------------------------
------------------------------------------------------------
------------------------------------------------------------
------------------------------------------------------------
------------------------------------------------------------
------------------------------------------------------------
------------------------------------------------------------
------------------------------------------------------------
------------------------------------------------------------
------------------------------------------------------------
------------------------------------------------------------
------------------------------------------------------------
------------------------------------------------------------
------------------------------------------------------------
------------------------------------------------------------
------------------------------------------------------------
------------------------------------------------------------
------------------------------------------------------------
------------------------------------------------------------
------------------------------------------------------------
------------------------------------------------------------
------------------------------------------------------------
------------------------------------------------------------
------------------------------------------------------------
------------------------------------------------------------
------------------------------------------------------------
------------------------------------------------------------
```


>XM_023371483_Centruroides_sculpturatus_PripL2
ATGGGT--

---------------------------------AGAGTT---AGCCAG------------

---TTT
AGGACCATCTGTGGCATCGACGAGCTGACC---AGCACCAGC------ACCTTATGGAGG
GCTTTGATTGCCGAATTCGTGGGCACAGCCATACTGGTCCTCATAGGTTGTGGGTCTTGT
ATCGGAGGATGGAGCGCAGAT---------------------------------------
--TACAAAGCCAAT
ATAGTGCAGATAGCGCTAGCCTTTGGTTTGACGGTTGGAACCGTGGTGCAGGGTATAGGA
CATGTCAGCGGTGGTCACATCAATCCTGCGGTAACTTGCGGTATGTTAGTGACTGGCAAA
TGCTCCATTTTTAGGTCTTTCTTCTATATTTGCTGCCAGTGCTTGGGTGGAATCGCGGGT
GCTGCCATACTTAAAGCCGTGACTCCCGAAAGTCAGCAAAGT------------------
------------------------------ATATTAGGTGCCACTAAAGTCAACGAAAGT
---------CTTAGCCCCTTTCAAGGGTTCGGAGTAGAGTTCTGCATAACTTTTATCCTA
GTTTTCACGGTTTTTGCA---GTCTGTGATAATAACCGTCTC------------------
---GACGTTACT---------------GGATCTGCACCACTCGCAATCGGCTTATCAGTT
GCAACCTGCCATTTGTTTGCGATAATGTATACAGGATCCAGTATGAACACAGCCAGATCT
TTTGGACCAGCAGTTGTTGCTGGA---------GTATGGGAGCATCACTGGGTTTATTGG
CTAGGACCAATTCTAGGTGGGATAGTGGCTGGATTATTATACCAACACGTATTTGTAGCA
---CCTGTCCCAGAA---------------------------------CATGAA------
------------------------GTG------------GTAGAAAGA------GAA---
CGTCTT---CGA---CATTTCCAACGTCTTAGA---GAGAAAGAA---------GTTGTT
---TCAGACAGGACAACT
TCCATT--

>AXZI01127083_Centruroides_exilicauda_PripL2
ATGGGT--

---------------------------------AGAGTT---AGCCAG------------

---TTT
AGGACCATCTGTGGCATCGACGAGCTGACC---AGCACCAGC------ACCTTATGGAGG
GCTTTGATTGCCGAATTCGTGGGCACAGCCATACTGGTCCTCATAGGTTGTGGGTCTTGT
ATCGGAGGATGGAGCGCAGAT---------------------------------------
--TACAAAGCCAAT
ATAGTGCAGATAGCGCTAGCCTTTGGTTTGACGGTTGGAACCGTGGTGCAGGGTATAGGA
CATGTCAGCGGTGGTCACATCAATCCTGCGGTAACTTGCGGTATGTTAGTGACTGGCAAA
TGCTCCATTTTTAGGTCTTTCTTCTATATTTGCTGCCAGTGCTTGGGTGGAATCGCGGGT
GCTGCCATACTTAAAGCCGTGACTCCCGAAAGTCAGCAAAGT------------------
------------------------------ATATTAGGTGCCACTAAAGTCAACGAAAGT
---------CTTAGCCCCTTTCAAGGGTTCGGAGTAGAGTTCTGCATAACTTTTATCCTA
GTTTTCACGGTTTTTGCA---GTCTGTGATAATAACCGTCTC------------------
---GACGTTACT---------------GGATCTGCACCACTCGCAATCGGCTTATCAGTT
GCAACCTGCCATTTGTTTGCGATAATGTATACAGGATCCAGTATGAACACAGCCAGATCT
TTTGGACCAGCAGTTGTTGCTGGA---------GTATGGGAGCATCACTGGGTATATTGG
CTAGGACCAATTCTAGGTGGGATAGTGGCTGGATTATTATACCAACACGTATTTGTAGCA
---CCTGTCCCAGAA---------------------------------CATGAA------
------------------------GTG------------GTAGAAAGA------GAA---
CGTCTT---CGA---CATTTCCAACGTCTTAGA---GAGAAAGAA---------GTTGTT
---TCAGACAGGACAACT
TCCATT--

>GIPT01004256_Centruroides_vittatus_PripL2
ATGGGT--

---------------------------------CGAGTT---AACCAG------------

---TTT
AGGACCATCTGTGGCATCGACGAGCTGACC---AGCACCAGC------ACCTTATGGAGG
GCTTTGATTGCCGAATTCGTGGGCACAACCATACTGGTTCTCATCGGTTGTGGGTCTTGT
ATCGGAGGATGGAGCACAGAT---------------------------------------
--TACAAAGCCAAT

ATAGTGCAGATAGCGCTAGCCTTTGGTTTGACGGTTGGTACCGTGGTGCAGGGTATAGGA
CATGTCAGCGGTGGTCATATCAATCCTGCGGTAACTTGCGGTATGTTAGTGACTGGTAAA
TGCTCCATTTTTAGATCTTTCTTCTATATTTGCTGCCAGTGCTTGGGTGGAATCGCGGGT
GCTGCCATACTTAAAGCCGTGACTCCCGAAAGTCAGCAAAGT------------------
------------------------------ATATTAGGTGCCACTAAAGTCAATGAGAGT
---------CTTAGCCCCTTTCAAGGGTTCGGAGTAGAGTTCTGCATAACTTTTATTCTG
GTTTTCACGGTTTTTGCA---GTCTGTGATAATAACCGTCTC------------------
---GATGTTACT---------------GGATCTGCACCACTCGCAATCGGCTTATCAGTT
GCAACCTGCCATTTGTTTGCGATAATGTATACAGGATCCAGTATGAACACAGCCAGATCT
TTTGGACCAGCAGTTGTTGCTGGA---------GTGTGGGAGCATCACTGGGTTTATTGG
CTAGGACCAATTCTAGGTGGGATAGTGGCTGGATTATTATACCAACACGTATTTGTAGCA
---CCCGTCCCAGAA---------------------------------CATGAA------
------------------------GTG------------GTAGAAAGA------GAA---
CGCCTT---CGA---CATTTC---------------------------------------

>GBZU01015267_Tityus_serrulatus_PripL2
ATGGGT--

---------------------------------AGAGTC---AACCAG------------

---TTT
CGGGCAATCTGCGGTATCGACGAGTTAACC---AGCACCAGC------ACCATATGGAGG
GCCATGATTGCAGAATTCCTTGGAACCGCCGTGCTGGTGCTCATCGGTTGCGGATCGTGC
ATCGGAGGATGGAGCGAAGAT---------------------------------------
--TACAAAGCCAGC
ATGGTGCAGATAGCGCTGGCATTCGGTTTGACGGTTGGAACCGTGGTTCAGGGTATCGGG
CACGTCAGCGGAGGTCACATCAATCCTGCAGTCACTTTCGGTATGCTAGTGACCGGTAAA
TGCTCCATTTTTAGATCTTTCTTCTACATTTGCTGCCAGTGCTTGGGTGGTATCGCTGGT
GCTGCCATACTTAAAGCCGTGACACCGGAGAACCAGCAGATT------------------
------------------------------ATATTGGGTGCCACTAAAGTCAACGGAAAT
---------CTTAGCCCGTTTCAGGGCTTCGGTGTAGAGTTCTGCATCACTTTCATCCTG
GTTTTCACCGTTTTTGCA---GTCTGTGATAATAATCGCCTC------------------
---GATGTCACC---------------GGTTCTGCACCACTCGCTATCGGGTTGTCAGTT
GCCACCTGCCATTTATTTGCGATCCAGTATACAGGATCTAGCATGAACACAGCAAGATCT
TTTGGTCCTGCGGTTGTTGCTGGA---------GTCTGGGAACATCATTGGGTCTACTGG
CTAGGACCAATTCTCGGCGGAATAGTGGCTGGACTTTTATATCAACACGTGTTTGTAGCA
---CCTGTGCCAGAG---------------------------------CACGAA------
------------------------GTC------------GTAGAAAGA------GAA---
CGCCTT---CGA---CATTTCCAGCGTCTGAGA---GAGAAAGAA---------GTCGTT
---TCAGATAGAACAACA
TCCATT--

>AYEL01088164_Mesobuthus_martensii_PripL2
ATGGGA--

---------------------------------AGAGAA---AGTAAA------------

---TTA
CGAAGCATCATCGGCGTAGACGAGTTAACA---AGCACCAGC------ACTCTTTGGAGG
AGTCTGTTGGCAGAATTCATAGGCACTGCCGTACTAGTTCTTATCGGTTGTGGTTCTTGC
ACGGAGGGATGGAGCCAAACG---------------------------------------
--TACAGTGCCACC
GTAGTTCAGATCGCTCTGGCCTTTGGAGTGACCGTAGCCACGATGGTTCAATGTATAGGA
CACGTCAGCGGAGGGCACATTAACCCAGCGGTGACATGCGGCATGTTAGTAACTGGTAAA
TGTTCCATCATCAGATCTCTATTCTATATCATCTGCCAATGTCTGGGTGGAATAGCTGGA
GCTGCCCTTCTTAAAGCCCTTACGCCCGAAGATCAGCAGGGT------------------
------------------------------TTCCTCGGTGGTACGTTCGTCAACAAA---
---------CTCAGCCCTTTTCAAGGATTCGGAGTCGAGTTCAGCATCACCTTCATCTTG
GTCTTCACCGTTTTTGCA---GTTTGTGACGGCAATCGTCTA------------------
---GACGTTACA---------------GGTTCTGCACCACTCGCAATTGGGTTATCAGTA
GCAACTTGCCATTTATTTGCAGTTAAATATACAGGTGCTAGCATGAATACAGCAAGATCA
TTTGGACCTGCAGTTATCACTGGT---------GAATGGGAACATCACTGGGTATATTGG
CTGGGACCAATTCTTGGTGGTATTGTAGCTGGACTTCTATACCAAAACGTTTTTGAAGCT
---CCTATTCCAGAA---------------------------------CATGAA------
------------------------GTA------------GTAGAGAGA------GAA---
CGACTT---CGA---CATTTCCAACGTCTGAGA---GAGAAGGAA---------ATTGTT

---GTAGATAGGACGACA
TCAATC--

>WUQG01006273_Androctonus_mauritanicus_PripL2
ATGGGA--

---------------------------------AGAGAA---AGTAAA------------

---TTA
CGAAGCATCATCGGCGTAGACGAGTTAACA---AGCACAAGC------ACTCTGTGGAGA

```
AGTCTGTTGGCAGAATTCATAGGCACTGCTGTACTAGTTCTTATCGGTTGTGGTTCTTGC
ACGGAGGGATGGAGCGAAACG---------------------------------------
------------------------------------------------TACAGTGCCACC
GTAGTTCAGATCGCTCTGGCCTTTGGAGTGACCGTAGCCACGATGGTTCAATGTATAGGG
CACGTCAGCGGTGGGCACATTAACCCAGCAGTGACATGCGGCATGTTAGTAACTGGTAAA
TGTTCCATCATCAGATCTCTCTTCTATATTATCTGCCAATGTTTAGGTGGAATAGCTGGA
GCTGCCCTTCTTAAAGCCCTTACGCCTGAAGATCAGCAGGGT------------------
------------------------------TTCCTCGGTGGTACGTTCGTCAACAAA---
---------CTCAGCCCTTTTCAAGGATTCGGAGTCGAGTTCAGCATCACCTTCATCTTG
GTCTTCACCGTTTTTGCA---GTTTGTGATGGCAATCGTCTT------------------
---GACGTTACA---------------GGTTCTGCACCACTCGCAATTGGGTTATCAGTA
GCAACTTGCCATTTATTTGCAATTAAATACACAGGTGCTAGCATGAACACAGCAAGATCA
TTTGGACCTGCAGTTATCACTGGT---------GAATGGGAACATCATTGGGTCTATTGG
TTGGGACCAATTCTTGGTGGTATCGTAGCTGGACTTCTATACCAAAATGTTTTTGAGGCT
---CCTATTCCAGAA----------------------------------CATGAA------
------------------------GTA------------GTAGAGAGA------GAA---
CGACTT---CGA---CATTTCCAACGTCTGAGA---GAGAAAGAA---------ATTGTT
---------------------------------------------GTAGATAGGACGACA
TCAATC------------------------------------------------------
------------------------------------------------------------
------------------------------------------------------------
------------------------------------------------------------
------------------------------------------------------------
------------------------------------------------------------
------------------------------------------------------------
------------------------------------------------------------
------------------------------------------------------------
------------------------------------------------------------
------------------------------------------------------------
------------------------------------------------------------
------------------------------------------------------------
------------------------------------------------------------
------------------------------------------------------------
------------------------------------------------------------
------------------------------------------------------------
------------------------------------------------------------
------------------------------------------------------------
------------------------------------------------------------
------------------------------------------------------------
------------------------------------------------------------
------------------------------------------------------------
------------------------------------------------------------
------------------------------------------------------------
------------------------------------------------------------
------------------------------------------------------------
------------------------------------------------------------
------------------------------------------------------------
```


>GILM01001462_Tachypleus_gigas_PripL3
ATGGGA--

---------------------------------CGCGTT---CGAAAA------------

---TTC
CGTGCGGTGTGTGGAATCGACGAGCTTTCC---AACGGGAAC------ATGCTATGGAAA
GCTTTGTTGGCCGAGTTCATCGGAACCGGGCTTTTGGTGCTTATAGGCTGTGGGTCCTGC
ATCTCTGGCTGGGACGAAGAT---------------------------------------
--TACAAACCATCT
ATAGTTCAAATAGCTCTAGCTTTCGGAGTGACTGTAGGTACCGTAGTTCAGGCCATATGT
CATATTAGCGGAGGACATATTAATCCAGCTGTTACTTTGGCCTTTCTAGCCACCGGTAAA
TGCTCTATTATTCGCGCCTTTTTCTATGTGTGTGCTCAGTGTGTGGGCGCAATTACAGGG
GCAGCAGTCTTGAAAGCACTAACACCTCTAGAACAGCAGAAG------------------
------------------------------TTACTAGGGGCCACCACTGTCCACTCACAT
---------TTAAACCCGATACAAGGATGCATTGTGGAGATCTGTATCACATTTGTCCTG
GTTCTGACAGTGTTTTCT---GTGTGTGATGACAACAGACTA------------------
---GATGTTAGA---------------GGATCACCACCCCTAGCCATCGGTCTTTCTGTC
GCCACTTGTCATATGTTTGCTGTCAAGTACACTGGGTCAAGTATGAACACAGCAAGGTCA
TTTGGACCTGCTGTAATATCTGGA---------GTTTGGGAATATCATTGGGTTTACTGG
CTAGGACCTATTCTCGGCGGAATAATTGGAGGAATTTTTTACCAACATACTTTCTCAGCC
---TTACCACCAGAG---------------------------------CCAGAA------
------------------------GAG------------CTGGAACGA------GTA---
CGACTT---AAC---AGCATCCGCCGTTACGAA---GAAAAAGAA---------GTCATT
---GAAGATAGGACTTCA
TCTGTT--

>GGKL01030661_Tachypleus_tridentatus_PripL3
ATGGGG--

---------------------------------CGCGTT---CGAAAA------------

---TTC
CGTGCGGTGTGTGGAATCGACGAGCTTTCC---AACGGGAAC------ATACTGTGGAAA
GCTTTGTTGGCCGAGTTCATCGGAACCGGGCTCTTGGTACTTATAGGCTGTGGGTCCTGC
ATCTCTGGCTGGGACGAAGAT---------------------------------------
--TACAAACCATCT
ATAGTTCAAATAGCTCTAGCTTTCGGAGTGACTGTAGGTACCGTAGTTCAGGCCATATGT
CATATTAGCGGAGGACATATTAATCCAGCTGTTACTTTGGCCTTTCTAGCCACCGGTAAA
TGCTCTATTATTCGCGCCTTTTTCTATGTGTGTGCTCAGTGTGTGGGCGCAATTATAGGG
GCAGCAGTCTTGAAAGCACTAACACCTCTAGAACAGCAGAAG------------------
------------------------------TTACTAGGGGCCACCACTGTCCACTCACAT
---------TTAAACCCAATACAAGGATGCATTGTGGAGATCTGTATCACATTTGTCCTG
GTTTTGACAGTGTTTTCT---GTGTGTGATGACAACAGACTA------------------
---GATGTTAGA---------------GGATCACCACCCCTAGCCATCGGTCTTTCTGTC
GCCACTTGTCATATGTTTGCTGTCAAGTACACTGGTTCAAGTATGAACACAGCAAGGTCA
TTTGGACCCGCTGTAATAGCTGGA---------GTTTGGGAATATCATTGGGTTTACTGG
CTAGGACCTATTCTCGGCGGAATAATTGGAGGGATTTTTTACCAACATACTTTCTCAGCC

---TTACCACCAGAG---------------------------------CCAGAA------
------------------------GAG------------CTGGAACGA------GTA---
CGACTT---AAC---AGCATCCGCCGTTACGAA---GAAAAAGAA---------ATCATT
---GAAGATAGGACTTCA
TCTGTT--

>GILQ01008878_Carcinoscorpius_rotundicauda_PripL3
ATGGGG--

---------------------------------CGCGTT------AAA------------

---TTC
CGTGCGGTGTGTGGAATCGACGAGCTTTCC---AACGGGAAC------ATGCTATGGAAA
GCTTTGTTGGCCGAGTTCATCGGAACCGGGCTTTTGGTGCTTATAGGCTGTGGGTCCTGC
ATCTCTGGCTGGGACGAAGAT---------------------------------------
--TACAAGCCATCT
ATAGTTCAAATAGCTCTAGCTTTCGGAGTGACTGTGGGTACTGTAGTTCAGGCCATATGT
CATATTAGCGGAGGACATATTAATCCAGCTGTTACTTTGGCCTTTCTAGCCACCGGTAAA
TGCTCTATAATTCGCGCCTTTTTCTATGTGTGTGCTCAGTGTGTGGGAGCAATTATAGGG
GCAGCAGTCTTGAAAGCACTAACACCTCTAGAACAGCAGAAG------------------
------------------------------TTTCTAGGGGCCACCACCGTCCACTCGCAT
---------TTAAACCCCATACAAGGATGCATTGTGGAGATCTGTATCACATTTGTCCTT
GTTCTGACGGTGTTTTCT---GTGTGTGATGAAAACAGACTA------------------
---GATGTTAGA---------------GGATCACCACCCCTAGCCATCGGTCTTTCTGTC
GCCACTTGTCATATGTTTGCTGTCAAGTACACTGGTTCAAGTATGAACACAGCAAGGTCA
TTTGGACCCGCTGTAATAGCTGGA---------GTTTGGGAATATCATTGGGTTTACTGG
CTAGGACCTATTCTCGGCGGAATAATTGGAGGGATTTTTTACCAACATACTTTCTCAGCC
---TCACCACCAGAA---------------------------------CCAGAA------
------------------------GAA------------CTGGAACGA------GTA---
CGACTT---AAC---AGCATCCGCCGTTACGAA---GAAAAAGAA---------GTCATT
---GAAGATAGGACTTCA
TCTGTT--

>XP_013773302_Limulus_polyphemus_PripL2
ATGGGG--

---------------------------------CGCGTT---CGAAAA------------

---TTT
CGTACGATTTGTGGAATCGATGAACTTTCT---AACGGAAAC------AAGTTATGGAAA
GCTATGTTGGCCGAATTCATTGGTACGGCGCTACTAGTTTTTATCGGCTGCGGATCCTGC
ATCTCCGGCTGGGACAAGGAC---------------------------------------
--TACAGCCCGTCT
ATGGTCCAGATTGCTTTAGCTTTCGGAGTAACCGTAGGAACCGTAGTTCAGGCCATAAGT
CATGTTAGCGGAGGGCATATAAACCCGGCGGTCACACTGGCATTTTTAGCTACCGGAAAA
TGTTCTTTTATTCGTGCCTTTTTCTACGTGTGTGCCCAGTGTGTGGGCGCGGTTGTAGGA
GCAGCAGTTTTGAAGGCACTAACTCCCGTAGAGCAGCAAGGG------------------
------------------------------TTACTAGGTGCCACCACTGTCCACTCACAT
---------TTAAACCCTATTCAAGGATGTATTGTGGAGATCTGTATCACGTTCGTCCTG
GTCTTGACTGTATTTTCT---GTGTGTGACAGCAATAGGCTT------------------
---GACGTGAGA---------------GGATCGGCACCTTTAGCTATTGGGTTGTCAGTT
GCTACCTGCCATTTATTTGCTATTAAGTACACCGGTTCTAGCATGAACACAGCGAGGTCC
TTTGGTCCTGCAGTGGCGGCTGGC---------GTCTGGAAATATCATTGGGTTTACTGG
CTAGGACCTATCTTAGGTGGCGTTATTGCCGGAATGGTCTATCAACATATTTCTCTGCT
---TCACCACCAAAA---------------------------------CTAGAA------
------------------------GAG------------CTAGAGAAG------ATA---
CGACTT---AAG---AGCCTCCGCCGTTACGAG---GAGAAAGAA---------GTTGTT
---GAAGACAGGACTTCA
TCTGTT--

>GILM01008493_Tachypleus_gigas_PripL2
ATGGGG--

---------------------------------CGCGTT---CGAAAA------------

---TTC
CGTGCGATTTGTGGAATCAACGAACTTTCT---AACGGAAAC------AAATTATGGAAA
GCCGTGTTGGCCGAATTCATTGGCACGGCGCTTCTTGTTTTCATCGGCTGCGGATCCTGC
ATCTCGGGCTGGGACAACGAC---------------------------------------
--TACAGCCCGTCT
ATGGTCCAGATTGCTTTAGCTTTCGGAGTGACCGTAGGAACCGTAGTTCAGGCCATATGT
CATGTTAGCGGAGGGCATATAAACCCGGCTGTCACGCTGGCATTTTTAGCTACCGGAAAA
TGTTCTTTTATTCGCGCCCTTTTCTACGTGTGCTCTCAGTGTGTGGGCGCGATTGCAGGA
GCAGCAATTTTGAAGGCTCTAACTCCTGTAGAGCAGCAAGGG------------------
------------------------------CTACTAGGTGCAACCACTGTCCACGCACAT
---------TTAAACCCTATTCAAGGTTGTATCGTGGAGATCTGTATCACGTTCGTCCTG
GTTTTGACTGTATTTTCT---GTGTGTGACAGCAATAGGCTT------------------
---GACGTGAGG---------------GGCTCGGCACCTTTAGCCATTGGGTTGTCAGTT

GCTACTTGCCATTTATTTGCTATTAAATACACCGGTTCCAGCATGAACACAGCGAGGTCC
TTCGGTCCTGCAGTGGCGGCTGGC---------GTTTGGGAATATCATTGGGTTTACTGG
TTAGGACCTATCCTAGGTGGCGTTATTGCCGGGATGGTCTATCAACATATTTTTTCTGCT
---TTACCACCAGAA---------------------------------CCAAAA------
------------------------GAG------------CTAGAGAAG------ATA---
CGCCTT---GAG---AACCTCCGCCGTTACGAG---GAGAAAGAA---------GATGAT
---GAAGACAGAACTTCA
TCAGTT--

>GGKL01030660_Tachypleus_tridentatus_PripL2

ATGGGG--

---------------------------------CGCGTT---CGAAAA------------

---TTC
CGTGCGATTTGTGGAATCAACGAACTTTCT---AACGGAAAC------AAACTATGTAAA
GCCATGTTGGCCGAATTCATTGGCACGGCGCTTCTTGTTTTCATCGGCTGCGGATCCTGC
ATCTCGGGCTGGGACAACGAC---------------------------------------
--TACAGCCCGTCT
GTGGTCCAGATTGCTTTAGCTTTCGGAGTGACCGTAGGAACCGTAGTTCAGGCCATATGT
CATGTTAGCGGAGGGCATATAAACCCGGCTGTCACGCTGGCATTTTTAGCTACCGGAAAA
TGTTCTTTTATTCGTGCCCTTTTCTACGTGTGCGCTCAGTGTGTGGGCGCGATTGCAGGA
GCAGCAATTTTGAAGGCTCTAACTCCTGTAGAGCAGCAAGGG------------------
------------------------------CTACTAGGTGCAACCACTGTCCACGCACAT
---------TTAAACCCTATTCAAGGATGTATCGTGGAGATCTGTATCACGTTCGTCCTG
GTTTTGACTGTATTTTCT---GTGTGTGACAGCAATAGGCTT------------------
---GACGTGAGG---------------GGCTCGGCACCTTTAGCCATTGGGTTGTCAGTT
GCTACTTGCCATTTATTTGCTATTAAGTACACCGGTTCCAGCATGAACACGGCGAGGTCC
TTCGGTCCTGCAGTGGCGGCTGGT---------GTTTGGGAATATCATTGGGTTTACTGG
TTAGGACCTATCCTAGGTGGCGTTATTGCCGGGATCGTCTATCAACATATTTTTTCTGCT
---TTACCACCAGAA---------------------------------TCAGAA------
------------------------GAG------------CTAGAGAAG------ATA---
CGCCTT---GAG---AACCTCCGCCGTTACGAG---GAGAAAGAA---------GATGCT
---GAAGACAGAACTTCA
TCTGTT--


```
------------------------------------------------------------
------------------------------------------------------------
------------------------------------------------------------
------------------------------------------------------------
------------------------------------------------------------
------------------------------------------------------------
------------------------------------------------------------
------------------------------------------------------------
------------------------------------------------------------
------------------------------------------------------------
------------------------------------------------------------
------------------------------------------------------------
------------------------------------------------------------
------------------------------------------------------------
------------------------------------------------------------
------------------------------------------------------
>GILQ01002235_Carcinoscorpius_rotundicauda_PripL2
ATGGGG------------------------------------------------------
------------------------------------------------------------
---------------------------------CGCGTT---CGAAAA------------
------------------------------------------------------------
---------------------------------------------------------TTC
CGTGCGATTTGTGGAATCAACGAACTTTCT---AACGGAAAC------AAACTATGGAAA
GCCATGTTGGCCGAATTCATTGGCACGGCGCTCCTTGTTTTCATCGGCTGCGGATCCTGC
ATTTCGGGCTGGGACAACGAT---------------------------------------
------------------------------------------------TACAGCCCGTCT
ATGGTCCAGATTGCTTTAGCTTTCGGAGTGACCGTAGGAACCGTAGTTCAGGCCATATGT
CATGTTAGCGGAGGGCATATAAACCCGGCTGTCACGCTGGCATTTTTAGCTACCGGAAAA
TGTTCTTTTATTCGCGCCCTTTTCTATGTGTGCGCTCAGTGTGTGGGCGCGATTGCAGGA
GCAGCAATTTTGAAGGCTCTAACTCCTGTAGAACAGCAAGGG------------------
------------------------------CTACTAGGTGCAACCACTGTCCACGCACAT
---------TTAAACCCTATTCAGGGTTGTATCGTGGAGATCTGTATCACGTTCGTCCTG
GTTTTAACTGTATTTTCT---GTGTGTGACAGCAACAGGCTT------------------
---GACGTGAGG---------------GGCTCGGCACCTTTAGCCATTGGGTTGTCAGTT
GTTACTTGCCATTTATTTGCTATTAAGTACACCGGTTCCAGCATGAACACAGCGAGGTCC
TTCGGTCCTGCAGTGGCGGCTGGC---------GTTTGGGAATATCATTGGGTTTACTGG
TTAGGACCTATTCTAGGTGGCGTTATTGCCGGGATGGTCTATCAACATATTTTTTCTGCT
---TTACCACCAGAA---------------------------------CCAGAA------
------------------------GAG------------CTAGAGAAG------ATA---
CGCCTT---GAG---AACCTCCGCCGTTACGAG---GAGAAAGAA---------GATGCT
---------------------------------------------GAAGACAGAACTTCA
TCTGTT------------------------------------------------------
------------------------------------------------------------
------------------------------------------------------------
------------------------------------------------------------
------------------------------------------------------------
------------------------------------------------------------
------------------------------------------------------------
```


>XP_013778287_Limulus_polyphemus_PripL3
ATGGGG--

---------------------------------CGCGTT---CGAAAA------------

---TTC
CGTGCGGTGTGTGGAATCGATGAGCTTTCC---AACGAGAAC------ATGCTATGGAAA
GCTTTGTTGGCCGAGTTCATCGGAACCGGGATTTTGGTGCTAATTGGATGCGGTTCTTGC
ATCTCTGGCTGGGACGAAGAT---------------------------------------
--TACAAGCCGTCT
GTAGTTCAAATAGCTCTAGCTTTCGGAGTGACTGTAGGTACCATAGTTCAGGCCATGTGT
CATATTAGCGGAGGGCACATTAATCCAGCTGTTACTTTGGCCTTTCTAGCCACCGGTAAA
TGCTCTATTCTTCGTGCCTTTTTCTACGTGTGTGCTCAGTGTGTGGGCGCAATCACAGGG
GCAGCAGTCTTGAAAGCGCTAACACCTCTAGCGCAGCAGGAG------------------
------------------------------TTACTAGGGGCCACCACTGTCCACCCGCAT

```
---------TTAAACCCCATACAAGGGTGCATTGTGGAAATCTGTATCACATTTGTCCTG
GTTCTTACAGTGTTTTCT---GTGTGTGATGGCAACAGACTA------------------
---GATGTAAGA---------------GGATCAGCCCCTCTGGCCATCGGTCTTTCTGTC
GCCACTTGTCATATGTTTGCTGTCAAGTATACTGGCTCGAGCATGAACACAGCAAGATCA
TTCGGACCAGCCGTAATAGCTGGA---------GTTTGGGAATATCATTGGATTTACTGG
CTCGGGCCTATTCTTGGTGGAATAATAGCAGGAATCTTTTACCAACATACTTTCTCTGCC
---TTACCACCAGAG---------------------------------CCAGAA------
------------------------GAG------------CTGGAACGA------GTA---
CGACTT---AAC---AGCATTCGCCGTTACGAA---GAAAAAGAA---------GTCATT
---------------------------------------------GAAGACAGAACTTCA
TCTGTT------------------------------------------------------
------------------------------------------------------------
------------------------------------------------------------
------------------------------------------------------------
------------------------------------------------------------
------------------------------------------------------------
------------------------------------------------------------
------------------------------------------------------------
------------------------------------------------------------
------------------------------------------------------------
------------------------------------------------------------
------------------------------------------------------------
------------------------------------------------------------
------------------------------------------------------------
------------------------------------------------------------
------------------------------------------------------------
------------------------------------------------------------
------------------------------------------------------------
------------------------------------------------------------
------------------------------------------------------------
------------------------------------------------------------
------------------------------------------------------------
------------------------------------------------------------
------------------------------------------------------------
------------------------------------------------------------
------------------------------------------------------------
------------------------------------------------------------
------------------------------------------------------------
------------------------------------------------------------
------------------------------------------------------------
------------------------------------------------------------
------------------------------------------------------------
------------------------------------------------------------
------------------------------------------------------------
------------------------------------------------------------
------------------------------------------------------------
------------------------------------------------------------
------------------------------------------------------------
```


>GILM01002510_Tachypleus_gigas_PripL1
ATGGGG--

---------------------------------CACGTT---CGAAAG------------

---TTT
CGAGCGGTTTGTGGAATTGACGAACTTACC---GTCAGAAGC------ACGTTATGGAAA
TCATTCTTAGCTGAGTTCATCGGAACAGCGCTTTTGGTGCTTGTGGGCTGCGGTTCCTGT
ATTTCCGGCTGGGACGAAAAC---------------------------------------
--TATAAGCCGACT
ATAGTCCAGATAGCTTTAGCTTTCGGATTAACTGTAGGTACTGTGGTTCAGGCCATATGT
CACGTCAGTGGTGGACACATCAATCCTGCTGTCACGTTGGCTTTTTTAGTTACCAAAAAA
TGTTCTTTTATTCGAGCTGTTTTCTACGTTAGTGCTCAGTGCTTGGGCGCCATTACAGGA
GCAGCGGTCTTAAAGGCACTGACCCCTACGAAGCAGCAGGGG------------------
------------------------------TCATTAGGGGCCACCACAATTCACGCGCAT
---------TTAAATCCTATCCAAGGATGTATTGTAGAGATCTCTATAACTTTCATCCTG
GTTTTTACAGTGTTCTCT---GTTTGTGACGACAACAGATTA------------------
---GACGTCATC---------------GGATCTGCTCCTTTGGCCATTGGGCTTTCCGTG
ACAACTTGCCATTTGTTTGCTATCAAGTATACGGGTGCCAGTATGAATACGGCAAGGTCG
TTCGGACCCGCTGTTATTTCAGGG---------GTTTGGGAATATCACTGGATTTACTGG
CTAGGTCCAATCCTTGGAGGGATAGTTGCGGGTATAATATATCAGCATATATTCTCGAGC
---TCTCCACCAGAT---------------------------------CCAGAA------
------------------------AAG------------CTGGAACGT------TTA---
CGATTA---AAT---AGAATTCAACGTTTAGAA---GAAAAGGAA---------GCCGTT
---GAGGACAGATCTTCG
TTTGTA--

>GGKL01023667_Tachypleus_tridentatus_PripL1
ATGGGG--

---------------------------------CACGTT---CGAAAG------------

---TTT
CGAGCGGTTTGTGGAATTGACGAACTTACC---GTCAGAAGC------ACGTTATGGAAA
TCATTCTTAGCTGAGTTCATCGGAACAGCGCTTTTGGTGCTTGTGGGCTGCGGTTCCTGT
ATCTCCGGCTGGGACGAAAAC---------------------------------------
--TATAAGCCAACT
ATAGTCCAGATAGCTTTAGCTTTCGGATTAACTGTAGGTACTGTGGTTCAGGCCATATGC
CACGTCAGTGGTGGACACATCAATCCTGCTGTCACGTTGGCTTTTTTAGTTACCAAAAAA
TGTTCTTTTATTCGAGCTGTTTTTTACGTTAGTGCTCAGTGCTTGGGCGCCATTACAGGA
GCAGCGGTCTTAAAGGCACTGACCCCTACGAAGCAGCAGGGG------------------
------------------------------TCATTAGGGGCCACCACAATTCACGCGCAT
---------TTAAATCCTATCCAAGGATGTATTGTAGAGATCTCTATAACTTTCATCCTG
GTTTTTACAGTGTTCTCT---GTTTGTGACGACAACAGATTA------------------
---GACGTCACC---------------GGATCTGCTCCTTTGGCTATTGGGCTTTCCGTG
ACAACTTGCCATTTGTTTGCTATCAAGTATACGGGTGCCAGTATGAACACGGCAAGGTCG
TTCGGACCCGCTGTTATTTCAGGG---------GTTTGGGAATATCACTGGATTTACTGG
CTAGGTCCAATCCTTGGAGGGATAGTTGCGGGTATAATATATCAGCATATGTTCTCGAGC
---TCTCCACCAGAT---------------------------------CCAGAA------
------------------------AAA------------CTGGAACGT------TTG---
CGATTA---AAT---AGAATTCAACGTTTAGAA---GAGAAGGAA---------GCCGTT
---GAGGACAGATCTTCA
TTTGTA--


```
------------------------------------------------------------
------------------------------------------------------------
------------------------------------------------------------
------------------------------------------------------------
------------------------------------------------------------
------------------------------------------------------------
------------------------------------------------------------
------------------------------------------------------------
------------------------------------------------------------
------------------------------------------------------------
------------------------------------------------------------
------------------------------------------------------------
------------------------------------------------------------
------------------------------------------------------------
------------------------------------------------------------
------------------------------------------------------------
------------------------------------------------------------
------------------------------------------------------------
------------------------------------------------------------
------------------------------------------------------------
------------------------------------------------------------
------------------------------------------------------------
------------------------------------------------------------
------------------------------------------------------------
------------------------------------------------------------
------------------------------------------------------------
------------------------------------------------------------
------------------------------------------------------------
------------------------------------------------------------
------------------------------------------------------------
------------------------------------------------------------
------------------------------------------------------------
------------------------------------------------------------
------------------------------------------------------------
------------------------------------------------------------
------------------------------------------------------
>GILQ01005617_Carcinoscorpius_rotundicauda_PripL1
ATGGGG------------------------------------------------------
------------------------------------------------------------
---------------------------------CACGTT---CGAAAG------------
------------------------------------------------------------
---------------------------------------------------------TTT
CGAGCGGTTTGTGGAATTGACGAACTTACC---GTCAGAAGC------ACGTTGTGGAAA
TCATTCCTAGCTGAGTTCATCGGAACAGGGCTTTTGGTGCTTGTGGGCTGCGGTTCCTGT
ATCTCCGGCTGGGACGAAAAC---------------------------------------
------------------------------------------------TATAAGCCGACT
ATAGTCCAGATAGCTTTAGCTTTTGGATTAACTGTAGGTACTGTGGTTCAGGCCATATGT
CACGTCAGTGGTGGACACATCAATCCTGCTGTCACGTTGGCTTTTTTAGTTACCAAAAAA
```

TGTTCTTTTATTCGAGCTGTTTTCTACGTTAGTGCTCAGTGCTTGGGCGCCATTACAGGA
GCAGCAGTCTTAAAGGCACTGACCCCTACGAAGCAGCAGGGG------------------
------------------------------TCATTAGGGGCCACCACAATTCACGCGCAT
---------TTAAATCCTATTCAAGGATGTATTGTAGAGATCTCTATAACTTTCATCCTG
GTTTTTACAGTATTCTCT---GTTTGTGACGACAACAGATTA------------------
---GACGTCACC---------------GGATCTGCTCCTTTGGCCATTGGGCTTTCCGTG
ACAACTTGCCATTTATTTGCTATCAAGTATACGGGTGCCAGTATGAACACGGCAAGGTCG
TTCGGACCCGCTGTTATTTCAGGG---------ATTTGGAATATCACTGGATTTACTGG
CTAGGTCCAATCCTTGGAGGAATAGTTGCAGGTATAATATATCAGCATATATTCTCGAGT
---TCTCCACCAGAT---------------------------------CCAGAA------
------------------------AAA------------CTGGAACGT------TTG---
CAATTA---AAT---AGAATTCAACGTTTAGAA---GAGAAGGAG---------GCCGTT
---GAGGACAGATCTTCA
TTTGTT--

>XP_013780027_Limulus_polyphemus_PripL1
ATGGGG--

---------------------------------CGCGTT---CGAAAG------------

---TTT
CGAGCGGTTTGCGGAATTGACGAACTTACC---GTCAGAAGC------ACGTTATGGAAG
GCATTCTTAGCCGAGTTCATCGGAACAGCGCTTCTGGTGCTTGTCGGCTGCGGTTCCTGT
ATCTCCGGTTGGGACGAAGAC---------------------------------------
--TATAAACCGACT
ATGGTCCAGATAGCTCTAGCTTTCGGAGTTACTGTAGGTACCGTAGTTCAGGCCATATGT
CACATTAGTGGTGGGCACATCAACCCAGCTGTCACGTTGGCTTTTTTAGTTACAAAAAAA
TGTTCTTTTATTCGAGCTCTTTTCTACGTCAGTGCTCAGTGCTTGGGCGCTATTACAGGA
GCTGCAGTCTTAAAGGCACTGACCCCTATAAAGCAGCAGGGG------------------
------------------------------TCACTAGGGGCCACCACAGTTCACGCGCAT
---------CTAAATCCTATCCAAGGATGTATTGTAGAGATCTCCATAACTTTCATCCTG
GTCCTGACGGTGTTTTCT---GTTTGTGACAACAACAGGTTA------------------
---GACGTCACA---------------GGATCTGCTCCTTTGGCCATTGGGCTTTCCGTG
ACAACTTGCCACCTGTTTGCTATCAAATATACCGGTGCCAGTATGAACACGGCAAGGTCG
TTCGGACCTGCTGTGATTGCTGGG---------GTTTGGGAATATCACTGGATTTACTGG
CTAGGTCCAATCCTTGGAGGGATAATTGCTGGAATGACCTATCAGCATATCTTCTCAGTT
---TCTCCACCAGAT---------------------------------CCAGAA------
------------------------GTA------------CTGGAACGT------TTA---
CGATTA---AAT---AGCATTCGACGTTACGAA---GAAAAGGAA---------GCCGTT
---GAAGATAGATCTTCA
TCTGTA--

>XM_023364955_Centruroides_sculpturatus_PripL1
ATGGGT--

---------------------------------CGAGTC---AGCCGC------------

---TTC
AGAAATATTTTCGGTATAAACGAGTTGTCA---GTTTTTAGT------CCTCTGTGGAAG
GCTCTCTTGGCGGAATACATAGGAACCGCTCTTCTAGTTTTAATCGGTTGTGGATCTTGT
ATCGGAGATTGGACTCGGAT--
--TACAAACCTACA
GTGGTACAAATCGCTCTGGCATTCGGATTAGCGGTGGGATGCATCGTACATGCTATAGGT
CACGTGAGTGGGGGTCACATCAATCCAGCGGTGACGATGGCTATGATGCTGACCGGAAAA
TGCTCCGTCATCAGATCCGTCTTCTATATAATCTGTCAATGCCTGGGAGCCGTCACCGGA
GCCGGCCTGTTGAAGGCATTGACACCCACCGCACAGAACGAA------------------
------------------------------GTTTTAGGTACGACTCTAGTCAATTCCAAC
---------TTGACACCAGTTCAAGCGTGGGGCGTGGAATTCCTCATCACCTTCGCTCTA
GTTTTAACCGTGTTTTCC---GTCTGCGATGCAAACAGGAGA------------------
---GATGCCACC---------------GGATCTCCCGCTCTGACCATCGGCCTGATTGTA
GTGACGTCACACCTCTTTGCCGTTAACTATACGGGATCCAGTATGAATTGTGCGAGATCT
CTGGGACCCGCTGTATTTTCGGGA---------ATGTGGAAAGATCACTGGGTGTACTGG
ATAGGTCCACTTTCGGGTGGAATCGTAGGAGGTCTGTTGTACGATGCTGTATTTTCTACT
---TTCCCTCCCTCT------------------------CTTCCAAAAAGTGAA------
------------------------GGT------------GTCGAAGCT------TGT---
TCCAGT---AAA---ATATGTACGGATGAAGAC---GAAAAAGAA---------GTTATC
---GCCGATAGAACAACA
ACAATT--

>AXZI01008908_Centruroides_exilicauda_PripL1
ATGGGT--

---------------------------------CGAGTC---AGCCGC------------

---TTC
AGAAATATTTTCGGTATAAACGAGTTGTCA---GTTTTTAGT------CCTCTGTGGAAG
GCTCTCTTGGCGGAATACATAGGAACCGCTCTTCTAGTTTTAATCGGTTGTGGATCTTGT
ATCGGAGATTGGACTCGGGAT---------------------------------------

```
------------------------------------------------TACAAACCTACA
GTGGTACAAATCGCTCTGGCATTCGGATTAGCGGTGGGATGCATCGTACATGCTATAGGT
CACGTGAGTGGGGGTCACATCAATCCAGCGGTGACGATGGCTATGATGCTGACCGGAAAA
TGCTCCGTCATCAGATCCGTCTTCTATATAATCTGTCAATGCCTGGGAGCCGTCACCGGA
GCCGGCCTGTTGAAGGCATTGACACCCACCGCACAGAACGAA------------------
------------------------------GTTTTAGGTACGACTCTAGTCAATTCCAAC
---------TTGACACCAGTTCAAGCGTGGGGCGTGGAATTCCTCATCACCTTCGCTCTA
GTTTTAACCGTGTTTTCC---GTCTGCGATGCAAACAGGAGA------------------
---GATGCCACC---------------GGATCTCCCGCTCTGACCATCGGCCTGATTGTA
GTGACGTCACACCTCTTTGCCGTTAACTATACGGGATCCAGTATGAATTGTGCGAGATCT
CTGGGACCCGCTGTATTTTCGGGA---------ATGTGGAAAGATCACTGGGTATACTGG
ATAGGTCCACTTTCGGGTGGAATCGTAGGAGGTCTGTTGTACGATGCTGTATTTTCTACT
---TTCCCTCCCTCT------------------------CTTCCAAAAAGTGAA------
------------------------GGT------------GTCGAAGCT------TGT---
TCCAGT---AAA---ATATGTACGGATGAAGAC---GAAAAAGAA---------GTTATC
---------------------------------------------GCCGATAGAACAACA
ACAATT------------------------------------------------------
------------------------------------------------------------
------------------------------------------------------------
------------------------------------------------------------
------------------------------------------------------------
------------------------------------------------------------
------------------------------------------------------------
------------------------------------------------------------
------------------------------------------------------------
------------------------------------------------------------
------------------------------------------------------------
------------------------------------------------------------
------------------------------------------------------------
------------------------------------------------------------
------------------------------------------------------------
------------------------------------------------------------
------------------------------------------------------------
------------------------------------------------------------
------------------------------------------------------------
------------------------------------------------------------
------------------------------------------------------------
------------------------------------------------------------
------------------------------------------------------------
------------------------------------------------------------
------------------------------------------------------------
------------------------------------------------------------
------------------------------------------------------------
------------------------------------------------------------
------------------------------------------------------------
------------------------------------------------------------
------------------------------------------------------------
------------------------------------------------------------
```


>GIPT01003695_Centruroides_vittatus_PripL1
ATGGGT--

---------------------------------CGAGTC---AGCCGC------------

---TTC
AGAAATATTTTCGGCATAAACGAGTTGTCA---GTTTTTAGT------CCTCTGTGGAAG
GCCCTCCTGGCGGAATACATAGGAACCGCTCTTCTAGTTTTAATCGGTTGTGGCTCTTGT
ATCGGAGGTTGGACTCGAGAT---------------------------------------
--TACGAACCTACA
GTGGTACAAATCGCTTTGGCATTCGGATTAGCGGTGGGATGCATCGTACACGCTATAGGT
CACGTGAGCGGGGGTCACATCAATCCGGCGGTGACGGTGGCTATGATGCTGACCGGAAAA
TGCTCCGTCATCAGATCCGTCTTCTATATAATCTGTCAATGCCTGGGAGCCGTCACCGGA
GCCGGCCTGTTGAAGGCATTGACACCCACCGCACAGAACGAA------------------
------------------------------GTTTTAGGTACGACTCTAGTCAATTCCAAC
---------TTGACACTCCCTCAAGCGTGGGGCGTGGAATTCCTCATCACCTTCGCTTTA
GTTTTAACCGTGTTTTCC---GTCTGCGATGCAAACAGGAAA------------------
---GATGCCACC---------------GGATCTCCCGCTCTGACCATCGGCCTGATTGTA
GTCACGTCACACCTCTTTGCCGTTAACTATACGGGATCGAGTATGAATTGTGCGAGATCT
CTGGGACCCGCCGTATTTTCGGGA---------ATGTGGGAACATCACTGGGTCTACTGG
ATAGGTCCACTTTCGGGTGGAATCGTAGGTGGTCTGTTGTACGATGCTGTATTTTCTACT
---CTCCCTCCCTCT------------------------CCTCCAAAAAGTGAA------
------------------------GGT------------GTCGAAGGT------TGT---
TCCAGT---AAA---ATATGTACGGATGAAGAC---GAGAAAGAA---------GTTATC
---GCCGATAGAACAACC
ACAATT--

>WUQG01001720_Androctonus_mauritanicus_PripL1
ATGGGG--

---------------------------------CGCGTT---AGCCGC------------

---TTC
AGAAATGTTTTCGGCATCAACGAAATACAC---GTTTTCAGC------CCTCTGTGGAAA
GCACTACTGGCAGAATTCATAGGAACCGCTATGTTAGTTCTAATCGGTTGTGGGTCGTGC
ATCGAAGGTTGGAAAGGCAGT---------------------------------------
--TATAAGTCTTCT
GTTGTGCAGATAGCTCTAGCATTCGGATTTGCTGTGGGATGCATCGTGCAGGCTATAGGA
CACGTGAGCGGAGGACACATCAACCCGGCTGTCACACTAGCTATGATGCTAACAGGCAAG
TGTTCCGTCATCAGATGCATTTTTTATATCATCTCTCAGTGTTTAGGAGCCGTGACCGGA
GCGGGCCTATTGAAAGCGTTGACACCCGAAAAACACGCCGAA------------------
------------------------------AATTTAGGCGCCACTCAAGTCAATTCAGAT
---------TTGACATTAGTTCAAGCATGGGCCGTGGAATTCCTAATTACCTTCGCTCTC
GTTCTAACAGTGTTTTCA---GTATGCGATGCCAACAGGAAA------------------
---GATGTCACT---------------GGATCACCAGCTTTGACCATAGGGTTGATTGTT
GTCACATCGCATCTGTTTGCCATAAATTACACGGGATCTAGCATGAATTGTGCCAGAGCA
CTGGGACCTGCAGTATTTTCGGGA---------AAATGGGAACATCATTGGGTCTACTGG
GTTGGTCCATTAAGCGGAGGAGCCATCGGTGGTCTGTTATACGACAGCGTGTTTTCAGTT
---TTCCCTTCGTCT------------------------TCGGAGAAAAATGAA------
------------------------TGC------------GTCGAAGCA------TCT---

```
TCCAGT---AAA---GTTTGCACGGATGTAGAC---GAGAAAGAA---------GTTATC
---------------------------------------------ACCGACAGAACTACC
ACTATT------------------------------------------------------
------------------------------------------------------------
------------------------------------------------------------
------------------------------------------------------------
------------------------------------------------------------
------------------------------------------------------------
------------------------------------------------------------
------------------------------------------------------------
------------------------------------------------------------
------------------------------------------------------------
------------------------------------------------------------
------------------------------------------------------------
------------------------------------------------------------
------------------------------------------------------------
------------------------------------------------------------
------------------------------------------------------------
------------------------------------------------------------
------------------------------------------------------------
------------------------------------------------------------
------------------------------------------------------------
------------------------------------------------------------
------------------------------------------------------------
------------------------------------------------------------
------------------------------------------------------------
------------------------------------------------------------
------------------------------------------------------------
------------------------------------------------------------
------------------------------------------------------------
------------------------------------------------------------
------------------------------------------------------------
------------------------------------------------------------
------------------------------------------------------------
------------------------------------------------------------
------------------------------------------------------------
------------------------------------------------------------
------------------------------------------------------------
------------------------------------------------------------
------------------------------------------------------------
------------------------------------------------------------
------------------------------------------------------
>AYEL01052592_Mesobuthus_martensii_PripL1
ATGGGG------------------------------------------------------
------------------------------------------------------------
---------------------------------CGCGTC---AGCCGC------------
------------------------------------------------------------
---------------------------------------------------------TTC
```

AGAAATGTTTTCGGCATCATCGAAATACAC---GTTTTCAGC------CCTCTGTGGAAA
GCACTACTGGCAGAATTCATAGGAACTGCTCTGTTAGTTCTAATCGGTTGTGGGTCGTGC
GTCGAAGGTTGGAAAGGCAGT---------------------------------------
--TATAAGTCTTCT
GTTGTGCAGATAGCTCTAGCATTCGGATTTGCCGTGGGATGCATCGTGCAGGTTATAGGA
CACGTGAGTGGAGGACACATCAACCCGGCTGTCACATTAGCCATGATGCTAACGGGAAAG
TGTTCCGTCATCAGATGCATTTTTTACATCATTTCTCAGTGTCTAGGAGCCGTAACTGGA
GCTGGCCTGTTGAAAGCGTTGACACCCGAAAAACACGCCGAA------------------
------------------------------AGCTTAGGCACCACTCAAGTCAGTTCAGAT
---------TTGACATTAGTTCAAGCATGGGCCGTGGAATTCCTAATTACCTTCGCTCTC
GTTCTAACAGTGTTTTCC---GTATGCGATGCCAACAGGAAA------------------
---GATGTCACT---------------GGATCACCAGCTTTGACCATAGGGTTGATTGTT
GTCACATCGCATCTGTTTGCCATTAATTACACGGGATCTAGCATGAATTGCGCCAGAGCA
TTGGGACCTGCAGTATTTTCGGGA---------AAGTGGGAACATCACTGGGTATACTGG
GTTGGTCCATTAAGCGGAGGAGCCATCGGTGGTCTGTTATACGACAGCGTATTTTCAGTT
---TTCCCTTCGTCT-------------------------TCGGAGAAAAATGAA------
------------------------TGC------------GTAGAAGCA------TCT---
TCCAGT---AAA---GTTTGCACGGATGTAGAC---GAGAAAGAA---------GTTATC
---ACCGACAGAACTACC
ACTATT--

>GBCS01013355_Latrodectus_hesperus_PripL2
ATGGGA--

---------------------------------CGCGTC---CGCAGA------------

---TTC
CGAGCCTTCGTCGGCATTGACGAGCTCTCC---TTCAACAGC------AGCCTGTGGCGG
GCCCTACCTGCAGAGTTCCTGGGCACCGGCTTCCTGGTCCTGGTGGCCTGCGGGTCCTGC
ACGGGTGGATGGGAGGAGAGC---------------------------------------
--TATTCTCCCACC
ATGGTGCAGATTGGGCTGTGCTTCGGCCTCGCGGTGGCCACCATGGTGCAGTGCATCTGT
CACGTGAGCGGGGGTCACATCAACCCCGCGGTGACGGCGGGCATGCTGGTGACAGGCAAG
TGTTCAGTCCTTAGAAGTCTGCTCTACGTGGTCATGCAGTGCGCGGGAGGCATAGCGGGG
GCGGCCGTGCTCAAGGCGGTCACTCCCGATGATCTGAGCTCA------------------
------------------------------CAGCTGGGTCAGACAGCACTCCATCCCAAA
---------CTGACCCCAATTCAAGGCGTGGGAGTAGAATTCCTCATCACCTTCGTTCTG
GTGTTAACCGTCTTCGGA---GTCTGTGATCCAAACAGAGCG------------------
---GACGTTAAG---------------GGGTCAGCCCCACTGGCCATCGGATTTTCTGTG
ACGGCCTGTCATCTGTGGGCAATCAAATACACTGGAGCAAGTATGAATACTGCCAGATCC
TTTGGTCCAGCAGTGATTTTAAAT---------AACTGGGAAAACCATTGGGTGTACTGG
GTAGGGCCGATTTTGGGAGGAGTTGTTGCTGGATTGATTTACGAGCATCTTTTTGCTGCT
---CCCGACAAGGAA---------------------------------CAACTG------
------------------------GAA------------CAAGCCAGA------ATG---
AATTCG---CAC------------GCTTACAAC---GATAAAGAT---------------
---TCGGATAGAACAACA
TCCATA--

>GBJM01135819_Latrodectus_geometricus_PripL2
ATGGGA--

---------------------------------CGCGTT---CGCAGA------------

---TTC
CGGGCCTTCGTCGGCATCGATGAACTCTCT---TTCAACAGC------AGCCTATGGCGG
GCCCTACCTGCCGAGTTCCTGGGCACGGGCTTTCTGGTCCTGGTGGCCTGTGGATCCTGC
ACGGGTGGATGGGAGGAGAGC---------------------------------------
--TACTCCCCCACT
ATGGTGCAGATAGGGCTGTGCTTCGGTCTCGCGGTGGCCACCATGGTGCAGTGCATCTGC
CACGTGAGCGGGGGTCACATCAACCCCGCTGTGACGGCGGGCATGCTGGTGACCGGCAAA
TGCTCAGTCCTTAGGAGTCTCCTCTACGTGGTCATGCAGTGCGCGGGCGGAATTGCGGGG
GCGGCCGTGCTCAAAGCGGTCACCCCCGATGATCTGAGCTCA------------------
------------------------------CAGCTGGGGCAGACAGCCCTCCATCCCAAG
---------CTGACCCCCATTCAGGGCGTGGGTGTAGAGTTCCTTATCACCTTCGTGCTG
GTGCTGACCGTCTTCGGC---GTCTGTGATCCAAACAGAGTG------------------
---GACGTCAAG---------------GGATCAGCCCCACTAGCCATCGGATTTTCTGTG
ACAGCCTGCCATCTATGGGCAATAAAATACACGGGAGCAAGTATGAATACTGCCAGGTCC
TTTGGTCCAGCAGTGATTTTAAAT---------AACTGGGAAAACCACTGGGTGTACTGG

GTAGGGCCAATTCTAGGAGGGCTGGTTGCTGGATTGATTTATGAGCATCTTTTTGCTGCA
---CCCGACAAGGAA---------------------------------CAACTG------
------------------------GAA------------CAGGGCAGA------ATG---
AATTCG---CAT------------GCTTACAAC---GATAAAGAT---------------
---TCGGACAGAACAACA
TCTATA--

>GBJQ01093518_Steatoda_grossa_PripL2
ATGGGA--

---------------------------------CGCGTA---CGCAGA------------

---TTC
CGAGCCTTCGTTGGCATTGATGAGCTCTCT---TTCAACAGC------AGCCTCTGGCGG
GCCCTACCCGCCGAGTTCCTAGGGACGGGCTTCCTGGTCCTGGTGGCATGCGGGTCCTGT
ACAGGGGGATGGAGGACTCC--
--TATTCTCCCTCC
ATGGTGCAGATCGGGCTGTGCTTCGGTCTCGCTGTGGCCACCATGGTGCAGGCCATCTGT
CACGTGAGCGGGGGACACATCAACCCCGCGGTGACTGCGGGGATGCTGGTGACTGGCAAG
TGTTCTGTCCTCAGGAGCCTGCTCTACGTTGTCATGCAATGCGCGGGAGGCATCGCCGGG
GCGGCCGTTCTCAAGGCGGTCACCCCAGACGACTTGGGCTCA------------------
------------------------------CAGCTGGGGCAGACGTCCCTCCATCCGAAG
---------CTGACTCCCATTCAGGGAGTGGGAGTGGAATTCCTCATCACCTTCGTGCTG
GTTTTGACTGTCTTTGGC---GTTTGTGACCCTAATAGAGTG------------------
---GACGTAAAA---------------GGGTCAGCCCCACTCGCTATCGGATTTTCCGTA
ACGGCTTGTCATCTATGGGCAATCAAATACACGGGTGCGAGTATGAACACTGCCAGATCC
TTTGGACCAGCAGTCATTCTAAAT---------AACTGGGACAATCATTGGGTATACTGG
GTAGGACCTATCTTGGGAGGGATTGTTGCTGGACTGGTTTACGAACACCTCTTCGCCGCA
---CCAGACAAAGAA---------------------------------CAGCTA------
------------------------GAA------------CAGGCCAGA------ATA---
AATTCC---CAT------------TCTTACAAC---GATAAGGAT---------------
---TCAGACAGAACAACT
TCCATA--

>XM_043051609_Parasteatoda_tepidariorum_PripL2
ATGGGA--

---------------------------------CGCGTT---CGAGAA------------

---TTT
CGTGCCGTTGTCGGCATCGACGAACTCTCG---TTCAACAGC------ACTCTATGGAGG
GCTCTCCCGGCAGAATTTCTGGGAACAGGATTCCTGGTACTCGTCGCCTGCGGATCCTGC
ACCGGAGGATGGGAGGAGACC---------------------------------------
--TACTCCCCAACC
ATGGTGCAAATTGGTCTGGCTTTCGGTCTTTCCGTGGCCACCATGGTACAGGCCATCTGT
CACGTGAGTGGAGGTCACATCAACCCGGCGGTGACGGCGGGGATGCTCATCGCCGGGAAA
TGCTCCATACTCCGCAGCATCCTGTATGTGGTGATGCAGTGTGCTGGAGGCATTGCGGGG
GCGGCAGTGCTGAAGTCCGTCACCCCCGAGGATCTGAACTCA------------------
------------------------------CAGTTGGGAATGACCTCCATCCATCCGAAG
---------ATGTCCCCCATCCAAGGTGTGGGAGTGGAATTTCTGATTACTTTTGTGTTG
GTTTTCACGGTGTTCGGT---GTGTGTGATCCCCACAGAAGG------------------
---GATGTAAAG---------------GGATCAGCCCCTCTCGCCATCGGCTTATCCGTC
ACGATGTGCCATTTGTGGGCTATAAAGTACACTGGTGCGAGTATGAACACGGCGAGATCT
TTTGGTCCTGCTGTTATACTCAAC---------AATTGGGAGAATCACTGGATATACTGG
GTAGGACCTATTCTCGGAGGGGTGGTAGCCGGACTTATATACGAACACCTCTTCGCTGCT
---TCACCTCATGAT---------------------------------AAAGAA------
------------------------GAA---------------------------------
CATTCT---CGA------------AACTACAAC---GAT---------------------
---TGTGACAGGACGACA
TCCATA--

>GIWR01263448_Argyroneta_aquatica_PripL2
ATGGGT--

---------------------------------CGGGTT---CGCAAG------------

---TTC
CGTGCGGTGGTCGGCATCGACGAACTGGCC---CTCAGCAGC------ACTCTCTGGAGG
GCCCTGCCCGCCGAGTTTCTCGGGACCGCATTCCTGGTGCTGATCGCGTGCGGGTCTTGT
ACAGGTGGCTGGGACGAGACC---------------------------------------
--TATGAACCGTCG
ATGGTGCAGATAGGACTCACCTTTGGCCTGATCGTGGCCACCATGGTCCAAGCCATCTGC
CACATCAGCGGGGGTCACATAAACCCGGCCGTGACCGCAGGGATGATGGTGACCGGCAAG
TGTTCCATCCTGCGGTCCCTGCTGTACATCATCATGCAGTGCGCGGGCGGCATCGCCGGG
GCTGCCGTGCTCAAGGCGGTCACCCCGGATGAACTGAATTCT------------------
------------------------------GGACTCGGCAACACAGCCCTCCACGCGAAG
---------ATGACCCCCCTCCAAGGAGTTGGCGTGGAGTTCCTCATCACTTTCGTCCTC
GTGTTCACGGTCTTCGGT---GTCTGCGACCCGAACAGGATG------------------

---GACGTCAAG---------------GGATCTGCACCCCTGGCGATAGGGTTCTCCGTT
GCAGCCTGCCACTTGTGGGCGATCAAGTACACAGGGGCCAGCATGAACACAGCCAGATCC
TTTGGCCCTTCTGTTATTCTGAAC---------GACTGGACTAATCACTGGGTATACTGG
GTCGGTCCTATCTTGGGAGGCATCGTCGCTGCTCTTGTGTATGAGCACCTGTTTGCAGCA
---CCATCTCCTGAC---------------------------------AAAGAG------
------------------------GAA------------ATCGAACGG------GCA---
AGAATG---CAA---GCCCTGCAGTCTATCAAC---GACAAAGAC---------------
---TCAGATAGAACGACA
TCCATA--

>XM_035351514_Stegodyphus_dumicola_PripL2
ATGGGT--

---------------------------------CGCGTA---CGTAAG------------

---TTT
CGTGCTATAGTCGGCATCGATGAACTAGCC---TGGAGTAGC------ACGCTCTGGCGG
TCCCTCCCGGCCGAATTTCTAGGCACTGGATTCCTGGTCCTAATAGCCTGTGGTTCTTGC
ACAGGGGGATGGAAAGCGAC--
--TACTCGCCTACA
ATCGTTCAGATAGGTCTCACTTTTGGTCTCATCGTGGGTACTATGGTGCAAGCCATTTGC
CACGTGAGTGGAGGCCACATTAATCCGGCTGTCACGGCAGGAATGTTGGTTACAGGTAAA
TGCTCAGTTCTGAGGGGCCTTCTCTATATTCTGATGCAATGTGCCGGTGGCATCGCCGGT
GCCGCTGTGCTCAAGGCGGTTACTCCAGAGGATCTAAATTCA------------------
------------------------------GCACTCGGGGAGACTGCTCTCCACCCCAAG
---------ATGAGCCCCATCCAGGGGGTAGGGGTCGAGTTCCTGATCACTTTTGTGCTC
GTCTTCACCGTTTTCGGA---GTGTGTGATCCCAACAGGGTG------------------
---GATGTTCGG---------------GGATCCGCTCCTCTAGCCATTGGGTTTTCTGTC
ACTGCTTGTCACCTGTGGGCGATTAAATACACTGGATCCAGTATGAATACTGCCAGATCT
TTTGGTCCCGCTGTGATACTAAAT---------AGATGGGAAAATCATTGGGTATATTGG
CTGGGCCCCATACTAGGTGGTGTCGTCGCTGGGCTTGTGTATGAGCATCTTTTCGCTGCT
---TCTCCACCTGAT---------------------------------AAGGAA------
------------------------GAC------------TTGGAACGG------GCC---
ACGATG---CAC---GCTTTGCAAGCCATCAAT---GACAAAGAC---------------
---TCAGACAGAACGACT
GCCATA--

>GBM59210_Araneus_ventricosus_PripL2
ATGGGT--

---------------------------------CGTGTT---CGAAAG------------

---TTC
CGTGCTGTCGTCGGGATCGACGAGTTGTCC---TGGAGTAGC------ACCCTCTGGCGC
TCCCTCCCGGCAGAGTTCCTTGGTACCGGATTCCTAGTACTGGTCGCCTGCGGTTCCTGT
ACCGGC---TGGGGAACGGGA---------------------------------------
--TATGACATGACACCATCC
ATAGTACAGATAGCCCTATCCTTTGGTCTCATAGTGGGCACCATGGTCCAATGTATCTGC
CACATATCAGGTGGGCACATCAACCCAGCTGTCACTGCTGGAATGCTGGTCGCTGGCAAG
ATATCTGTACTGCGAGCTTTGCTATACATCGTGATGCAGTGTGCTGGCGGCATTGCTGGG
GCAGCTGTACTGAAATCTGTGACTCCAGACGGACTGAACTCG------------------
------------------------------ACGCTGGGACAGACGATGCTACACGAGAAG
---------GTGACGCCTGTTCAAGGGACTGGAGTCGAGTTTCTCATCACATTCGTCCTG
GTGTTCACGGTGTTCTCG---GTGTGCGATTCCAATAGGCTG------------------
---GACGTGAAG---------------GGATCTGCTCCCCTGGCGATTGGACTGTCCATA
ACTGCGTGCCATTTGTGGGCGATCCGATACACAGGAGCCAGCATGAACACGGCCAGATCC
TTTGGACCAGCTGTCATCATGAAT---------AACTTTCAAAATCACTGGGTTTACTGG
GTTGGTCCCATCCTCGGTGGTGTAGTAGCAGGATTGATATACGAATATTTGTTCTCTGCC
---AATCCTCCAGAC---------------------------------AAAGAA------
------------------------GAA------------GTGGAGAGA------GCC---
AAACTA---CAT---GCTCTTCAGGCTTACAAC---GACAAGGAT---------------
---TCTGATAGAACGACT
TCCATC--

>GFT16935_Nephila_pilipes_PripL2
ATGGGT--

---------------------------------CGCGTA---CGAAAA------------

---TTC
CGTGCCGTGGTTGGCATCGACGAATTGTCC---TGGAGTAGC------ACCCTATGGCGC
AGCCTTCCAGCAGAATTCCTGGGCACAGGGTTCCTGGTCCTGGTCGCATGCGGGTCTTGC
ACGGGC---TGGGGTACGGAG---------------------------------------
--TACGATCAAACTCCGTCC
ATGGTCCAAATAGCCCTGTGTTTTGGTCTCATAGTGGCCACTATGGTCCAGTCAATCTGT
CATATCTCGGGGGGCCACATCAACCCAGCAGTGACTGCTGGTATGCTGGTGGCTGGACGC
TGCTCCGTACTCCGGGCGATGCTGTACGTTGTGATGCAGTGCGCTGGGGGTATTGCTGGT
GCCGCTGTGCTCAAGTCCGTAACTCCAGATGAGTTGAACACT------------------

------------------------------TCTTTGGGGATGACAGCCCTTCATCCCAAA
---------GTAACTCCAGTGCAAGGGACTGCGGTGGAATTCTTGATCACTTTCGTTCTG
GTATTCACTGTATTCGGG---GTTTGCGACGGGAATCGTCTG------------------
---GATGTGAAG---------------GGATCAGCACCTCTTGCCATTGGGTTCTCCATC
ACAGCATGCCATTTGTGGGCGATTCGATACACAGGAGCCAGTATGAACACAGCAAGATCT
TTTGGACCAGCCGTCATCATGAAC---------AACTTTGAAAACCACTGGGTGTACTGG
GTTGGTCCTATCCTTGGTGGCGTTGTAGCAGGATTGATATACGAATACCTCTTTTCCGCC
---AACCCTCCAGAT---------------------------------AAGGAA------
------------------------GAA------------TTGGAACGA------GCC---
AAAATG---CAC---GCTCTCCAGGCCTACAAC---GACAAAGAT---------------
---TCAGATAGAACCACT
TCCATC--

>GAZS01036184_Acanthoscurria_geniculata_PripL2
ATGGGC--

---------------------------------CGCGTT---CGAAGA------------

---TTT
CGGCGATTGTTGGTATTGACGAACTGGCC---TGGAACAAC------ACACTGTGGAGA
TCCCTTCCTGCTGAGTTTTTGGGCACGGCATTCTTAGTTTTGGTGGCTTGTGGCTCATGC
ACTGCGGATGGGAGAACGAC--
--TACTCACCTTCT
ATGGTTCAAATTGCATTAAGTTTTGGTCTTATAGTTGCTACCATGGTCCAGGCTATATGC
CATGTTAGTGGGGGCCATATTAATCCAGCAGTTACGGCAGGAATGTTAGTCACCGGAAAA
TGCTCAGTTATCCGAGCTCTGCTGTACATTGTGATGCAGTGTATTGGAGGTATAGCTGGA
GCTGCTGTACTGAAGGCAGTTACTCCCCCAAATCTGCAAGGT------------------
------------------------------GCTCTCGGCGGAACCGCGATAAATCCTAAT
---------CTGGGTGTCGAACAAGGACTGGCGGTTGAATTCCTCATCACCTTCGTTCTG
GTCTTCACCGTTTTTGCA---GTCTGCGATTGCAATAGGCTG------------------
---GATGTTCGA---------------GGATCAGCTCCTTTGGCAATTGGACTTTCCGTT
ACAGCATGCCACCTCTGGGCAATAAAATTAACTGGCGCAAGCATGAACACTGCAAGGTCA
TTTGGTCCAGCCGTTGTCGGAAAT---------AAATGGGAGAACCACTGGGTATATTGG
GTTGGACCAATACTAGGTGGCGTGGTCGCTGGCCTCATCTATGAACATCTCTTCGCAGCT
---CCTCCCCCAGAC---------------------------------AAAGAA------
------------------------GCC------------GTGGAACGG------GCT---
CGAATG---CAT---AGTTTACAAGGCATCAAC---GACAAGGAA---------GTGGTT
---GCCGACAGAACGACA
TCTATT--

>GIIZ01116426_Acanthoscurria_juruenicola_PripL2
ATGGGC--

---------------------------------CGCGTT---CGAAGA------------

---TTT
CGGGCGATTGTTGGTATTGACGAACTGGCC---TGGAACAAC------ACACTGTGGAGA
TCCCTTCCTGCTGAGTTTTTGGGCACGACATTCTTAGTTTTGGTGGCTTGTGGCTCATGC
ACTGCGGGATGGGAGAACGAC---------------------------------------
--TACTCACCTTCT
ATGGTTCAAATTGCATTAAGTTTTGGTCTTATAGTTGCTACCATGGTCCAGGCTATATGC
CATGTTAGTGGGGGCCATATTAATCCAGCAGTTACGGCAGGAATGTTAGTCACCGGAAAA
TGCTCAGTTATCCGAGCTCTGCTGTACATTGTGATGCAGTGTATTGGAGGTATAGCTGGA
GCTGCTGTACTGAAGGCAGTTACTCCCCCAAATCTGCAAGGT------------------
------------------------------GCTCTCGGCGGAACCGCGATAAACCCTAAT
---------CTGGGTGTCGAACAAGGACTGGCGGTTGAATTCCTCATCACCTTCGTTCTG
GTCTTCACCGTTTTTGCA---GTCTGCGATTGCAATAGGCTG------------------
---GATGTTCGA---------------GGATCAGCTCCTTTGGCAATTGGACTTTCCGTT
ACAGCATGCCATCTCTGGGCAATAAAATTAACTGGCGCAAGCATGAATACAGCAAGGTCA
TTTGGTCCAGCCGTTGTCGGAAAT---------AAATGGGAGAACCACTGGGTATATTGG
GTTGGACCAATACTAGGTGGCGTGGTCGCTGGCCTCATCTATGAACATCTCTTCGCAGCT
---CCTCCCCCAGAC---------------------------------AAAGAA------
------------------------GCC------------GTGGAACGG------GCT---
CGAATG---CAT---AGTTTACAAGGCATCAAC---GACAAGGAA---------GTGGTT
---GCCGACAGAACGACA
TCTATT--

>GBCS01013785_Latrodectus_hesperus_PripL1
ATGGGA--

---------------------------------CGCGTT---CGGGAA------------

---TTT
CGCTCTATATGCGGGATGGAGGAGTTGAGC---GCACTTAAC------CAGCTCGCGAAA
GCCATTCTGGCCGAATTAATAGGCACTGCGGTGCTAGTTCTAGTGGGATGCGGCTCTTGT
ATCGGAGGCTGGGGCGAAGGC---------------------------------------
--ACTCAGCCTACC
ATGGTTCAGATAGCCCTAGCCTTCGGGGTCACTGTCGCTACCGTAGCTCAATGCATAGGG

GGTATTAGCGGTGGTCACATCAACCCAGCGGTCACCTGTGGAATGATGGTCACGGGGAGA
TGCTCCTTCCTTCGCTCTGGCCTTTACATTGTTGCCCAATGCAGTGGAGCTATAGCAGGG
GCAGCAATTCTGAAAGCTGTAACTCCTGAAGCCCAGCATGGA------------------
------------------------------GCTTTGGGAGCGACACTACTCCACCCGAAT
---------CTGACGCTCATGCAAGGGCTGGCTGTTGAGTTTTGTATCACTTTTGTTCTT
GTCTTCACTGTTTTTGCT---GCATGTGATGAGCATCGGTTA------------------
---GATGTACAA---------------GGTTCGATTCCCCTGGCTATTGGTCTGTCTATC
ACAGCTTGTCATTGTTTCGCTATACGTTTTACGGGCTCAAGCATGAACACGGCACGTTCA
TTTGGTCCTGCGGTGATGACTGGA---------CTTTGGCAAAACCACTGGATATACTGG
GTCGGTCCCATTTTAGGTGGGGTAGTTGCTGGACTCCTTTATCAACATGTATTTGCTGCA
---CCTCCACCAGAT---------------------------------CCAGAA------
------------------------AGT------------ATTGAACGC------TTG---
AAATTA---CAA---AGTCTTCTGAAAATAAGA---GAAAAGGAA---------ATAATT
---ACCGACCGCACAACA
TGCATT--

>GFCZ01013118_Latrodectus_geometricus_PripL1
ATGGGA--

---------------------------------CGCGTT---CGAGAA------------

---TTT
CGCTCTATATGCGGAATGGAGGAGTTGAGC---GCACTAAAC------CAGCTCGCGAAA
GCCATTTTGGCCGAATTAATAGGCACTGCGGTGCTAGTTCTCGTAGGTTGCGGCTCTTGT
ATCGGAGGCTGGGGCGAAGAC---------------------------------------
--ACTCAGCCTACC
ATGGTCCAGATAGCCCTAGCTTTCGGGGTCACTGTCGCTACCGTAGCTCAATGCATAGGG
GGTATTAGCGGTGGTCACATCAACCCAGCGGTCACCTGTGGAATGATGGTGACTGGGAGA
TGCTCTTTCCTTCGCTCTGGCCTTTACATTGTTGCCCAGTGTAGTGGAGCTATAGCAGGG
GCAGCAATTTTGAAAGCTGTTACTCCTGAAGCCCAACACGGA------------------
------------------------------GCTTTAGGAGCAACGCTTCTCAACCCAAAT
---------CTGACAGTCATGCAAGGACTGGCTGTTGAGTTTTGCATCACTTTTGTTCTT
GTCTTCACTGTTTTCGCT---GCTTGTGATGAACATCGGTTA------------------
---GATGTACAG---------------GGTTCCATTCCTCTGGCTATTGGTCTGTCTATC
ACAGCTTGTCATTGTTTCGCTATACGTTTTACGGGCTCAAGCATGAACACAGCACGTTCA
TTTGGACCTGCGGTGATGACTGGG---------CTTTGGCAAAATCACTGGATATACTGG
GTCGGTCCCGTTTTAGGTGGGGTAGTTGCTGGACTTTTTTATCAACATGTATTTGCTGCA
---CCTCCTCCAGAT---------------------------------CCAGAA------
------------------------AGC------------ATTGAGCGT------TTG---
AAATTA---CAA---AGTCTTCTGAAGATAAGA---GAAAAGGAA---------ATAATT
---ACTGACCGCACAACA
TGCATT--

>AOMJ01121993_Parasteatoda_tepidariorum_PripL1
ATGGGA--

---------------------------------CGAGTG---AAAGAA------------

---TTT
AGATCTATTTGCGGTATGGAAGAGTTAGGA---GCAATTCGC------CAACTCGCAAAA
GCTGTTGTAGCTGAATTTTTAGGTACCGCAATTTTGGTACTTGTAGGTTGTGGTTCGTGT
ATTGGAGGCTGGGGAGAA--
---AGTCCCACT
ATTATCCAAATAGCTATAGCCTTTGGAATTACAGTAGCCACAGTGGCCCAATGTATTGGA
GGAATTAGTGGCTGTCACATTAATCCAGCTGTCACTTGTGGCATGATGGTCACTGGAAAA
TGCTCATTTTTGCGAGCCGGACTTTATATTGTTGCTCAATGTTGCGGAGCAATAGCAGGA
GCTTCTATTTTGAAAGCTGTAACCCCCCAAGCGCAGGTTGGG------------------
------------------------------GCTCTTGGATCTACTTTAATTAATCCAGAG
---------ATAACAGCGTTTCAAGGACTTGCTGTAGAATTCTGTATCACTTTTGTTCTC
GTATTTACTGTCTTCGCT---GCCTGTGATGATCATCGATTA------------------
---GACGTACAG---------------GGTTCGATTCCCCTCGCTATTGGGCTATCCATC
ACGGCTTGTCACTGCTTTGCTATTCGTTATACTGGCTCCAGCATGAATACAGCCCGCTCA
TTTGGCCCAGCCATTGTTACGGGA---------CTTTGGGAAAACCATTGGGTATACTGG
GTTGGACCAATTCTTGGAGGTGTTGTTGCTGGCCTTCTGTACCAACATTTATTCGCTTGC
---CCGCCACCTGAA---------------------------------CAGGAT------
------------------------AGC------------ATCGATCGT------TTA---
AAGCTG---CAA---TCATTATTGAAGTTGAGA---GAAAAAGAA---------ATAATA
---ACCGATCGCACAACT

TGCATT--

>GEWZ01007417_Nephilengys_cruentata_PripL1
ATGGGC--

---------------------------------CGTGTG---AGAGAA------------

---TTT
CGTTCAGTGTGTGGCATGGAAGAATTGGGT---GCTCTCAAC------CAGCTCTGTAAA
GCCGTTTTGGCTGAGTTCTTAGGCACGGCCATTTTGGTCCTAGTAGGATGCGGCTCTTGC

```
ATCGGCGGATGGGGCGAAAAC---------------------------------------
------------------------------------------------ACTTCACCGACC
ATCGTCCAGATCGCTCTCGCTTTTGGTGTCACTGTGGCCACTATTGCTCAGTGTATTGGA
GGGGTGAGTGGAGGTCATATTAACCCGGCGGTCACTTGTGCTATGATGATCACTGGGAGA
TGCTCATTTATTAGATCTGGACTTTACATTGTCGGTCAATGCTGCGGGGCTATTGCCGGA
GCGGCACTTTTGAAAGCTCTTACTCCGGAAGCTCAACGATTG------------------
------------------------------GCTTTGGGGTCGACTCTTGTGAACGAGGGG
---------TTAACTTCCGTGCAAGGGCTGGGAGTGGAATTCTGTATCACTTTTGTTCTC
GTGTTAACCGTATTTGCT---GCTTGCGATGAACATCGTTTG------------------
---GACGTACAA---------------GGATCTGTCCCATTGGCCATAGGATTGTCCATT
ACTGCATGTCATTGTTTCGCGATTCGCTACACAGGTTCCAGCATGAATACAGCCCGTTCA
TTTGGTCCAGCTGTTATGACAGGA---------TTATGGGAAAATCATTGGGTGTATTGG
GTAGGACCTATCTTAGGAGGAGTAACAGCTGGTTTGCTTTATCAACACGTATTTGCGTGT
---CCTCCACCTGAA---------------------------------CAAGAA------
------------------------AGT------------ATCGAACGC------CTC---
AAATTG---CAA---AGTCTTCTCAAAATAAGA---GAAAAAGAA---------ATCATT
---------------------------------------------ACAGATCGCACAACA
TGCATT------------------------------------------------------
------------------------------------------------------------
------------------------------------------------------------
------------------------------------------------------------
------------------------------------------------------------
------------------------------------------------------------
------------------------------------------------------------
------------------------------------------------------------
------------------------------------------------------------
------------------------------------------------------------
------------------------------------------------------------
------------------------------------------------------------
------------------------------------------------------------
------------------------------------------------------------
------------------------------------------------------------
------------------------------------------------------------
------------------------------------------------------------
------------------------------------------------------------
------------------------------------------------------------
------------------------------------------------------------
------------------------------------------------------------
------------------------------------------------------------
------------------------------------------------------------
------------------------------------------------------------
------------------------------------------------------------
------------------------------------------------------------
------------------------------------------------------------
------------------------------------------------------------
------------------------------------------------------------
------------------------------------------------------------
------------------------------------------------------------
```


>GFQ96285_Trichonephila_clavata_PripL1
ATGGGC--

---------------------------------CGTGTT---AGCGAA------------

---TTC
CGTTCAGTGTGTGGCATGGAAGAATTGGGT---GCTCTCAGC------CAGCTCTGCAAA
GCCGTTTTGGCTGAGTTCTTAGGCACAGCCATTTTAGTCCTGGTGGGATGCGGCTCTTGC
ATCGGAGGATGGGGCGAAAAC---------------------------------------
--ACTTCACCGACC
ATTGTCCAGATCGCTCTCGCTTTTGGGGTCACTGTGGCCACAATTGCTCAGTGTATTGGA
GGGGTGAGTGGAGGTCATATCAACCCGGCGGTCACCTGTGCTATGATGATCACTGGGAGA
TGCTCGTTTATTAGATCTGGACTTTACATTGTCGGTCAGTGCTGCGGAGCGATAGCCGGA
GCGGCGCTTTTGAAAGCTATTACTCCGGAAGATCAGCGAGGG------------------
------------------------------GCTTTGGGATCGACTCTTGTGAACGGGGAG
---------TTGTCTTCTGTACAAGGGCTAGGAGTGGAATTCTGTATCACTTTCGTTCTC
GTGCTCACCGTCTTTGCT---GCTTGCGATGAACATCGGTTG------------------
---GACGTGCAA---------------GGATCTGTCCCTTTGGCTATTGGATTGTCCATT
ACTGCCTGCCACTGTTTTGCGATTCGCTACACGGGTTCCAGCATGAATACAGCCCGCTCA
TTTGGCCCTGCAGTTATGACAGGA---------TTATGGGAAAATCATTGGGTATATTGG
GTAGGGCCTATCTTAGGAGGAATAACAGCTGGTTTGCTATATCAACATGTATTTGCCTGT
---CCTCCACCAGAG---------------------------------CCAGAA------
------------------------CAC------------ATAGAACGA------CTC---
AAGCTG---CAA---AGTCTTCTTAAAATAAGA---GAAAAGGAA---------ATAATT
---ACAGATCGCACAACA
TGCATT--

>GIY48399_Caerostris_extrusa_PripL1
ATGGGA--

---------------------------------CGAGTG---ACAGAT------------

---CTC
CGTTCGGTGTGCGGTATGGAAGAACTTGGG---GCTCTCAAC------CAACTCAGCAAG
GCTGTTTTAGCCGAGTTTCTTGGCACGGCAATTTTAGTTTTGGTCGGGTGCGGATCTTGC
ATTGGGGGTTGGGGAGAAAAT---------------------------------------
--AGTTCACCTACC
ATCGTCCAGATAGCTCTGGCTTTTGGAGTCACCGTGGCCACTGTGGCCCAATGCATTGGA
GGGGTCAGTGGAGGCCACATTAACCCTGCGGTCACATGTGCTATGATGATCACCGGAAGA
TGCTCTTTTATTAGATCTGGACTTTATATCGTGGGGCAGTGCTGTGGGGCTATCGCTGGT
GCAGCACTTTTGAAAGCCATCACCCCCGAAACCCAAAGAGGG------------------
------------------------------GCTCTGGGGTCAACTCTAGTGACTGAGGGG
---------GTGACTCCCATCCAGGGTTTGGGGGTGGAATTCTGTATCACTTTCGTTCTG
GTACTCACTGTCTTCGCT---GCTTGTGACGAGCATCGTCTG------------------
---GATGTCCAA---------------GGATCCGTCCCTCTTGCTATTGGATTGTCCATA
ACTGCTTGTCACTGTTTTGCGATCCGCTATACAGGCTCTAGTATGAATACAGCCCGATCA
TTTGGTCCAGCAGTTATGACAGGT---------TTATGGGAAAATCATTGGGTATATTGG
GTAGGACCCATCTTAGGAGGTGTAACAGCAGGTCTACTTTATCAACACGTCTTTGCTTGT
---CCACCACCAGAA---------------------------------CCAGAA------

------------------------AAT------------ATCGAACGT------CTG---
AAGTTG---CAA---ACCCTTCTTAAAATAAGA---GAAAAGGAA---------ATCATT
---ACAGATCGCACAACA
TGTATT--

>XM_035351860_Stegodyphus_dumicola_PripL1
ATGGGA--

---------------------------------CGAGTT---CGTGAA------------

---TTC
CGCTCTGTGTGCGGGATAGAGGAGTTGAGT---GCCTTGAGC------CAGCTTTCCAAA
GCAGTCCTAGCAGAATTCTTAGGTACTGCAATACTTGTCCTGGTGGGATGCGGCTCTTGT
ACTATTGGATGGAGTGAGGCT---------------------------------------
--TATTCTCCAACA
GTTGTCCAGATTGCATTGGCCTTTGGTGTCACTGTGGCCACTGTTGCTCAATGCATCGGA
GGGGTCAGTGGAGGTCACATCAATCCCGCCGTTACTTGCGATGATGGTTACTGGAAGA
TGTTCATTTCTACGCTCCGGGCTCTACATTGTTGGTCAGTGCGCCGGAGCAGTAGCAGGA
GCGGCACTTTTGAATGTGCTAACTCCAGATGCTCAGCGCGGA------------------
------------------------------GACCTGGGGATGACCAGGGTTCATCCAGGA
---------ATGACCCCAACACAAGGGCTGGGGGTTGAATTCTGCATCACATTCGTTCTT
GTTTTCACCGTTTTCGCT---GCTTGTGATGAAAGTCGCCTC------------------
---GATGTTCAG---------------GGCTCCATCCCGCTTGCTATAGGATTGTCCATA
ACAGCTTGCCATTGTTTCGCA---------------------------------------
---GTCTATTGG
GTAGGTCCTATATTAGGAGGTGTAACAGCTGGTCTCCTTTACCAACATATACTTGCTTGT
---CCACCACCAGAA---------------------------------CCTGAA------
------------------------AAC------------CTGGAGAGG------CTA---
AAACTC---CAA---AGTTTGCTCAAAATAAGA---GAAAAAGAA---------CTAATA
---ACTGATCGCACAACG
TGTATT--

>GIWR01118932_Argyroneta_aquatica_PripL1
ATGGGA--

---------------------------------CGTGTA---AAGGAA------------

---TTT
CGCTCTGCCTGTGGCATGGAGGAACTTGGG---GCCGTGAGA------CCCCTGCTCCGG
GCCGTCTTGGCGGAGTTCCTGGGGACGGCTATACTTGTCCTAGTTGGCTGCGGATCCTGT
GTCAAAGGATGGGACGAAACG---------------------------------------
--TATTCGCCGTCC
ATCGTCCAGATAGCACTTGCATTTGGAGTGACCGTGGCCACTGTTGCCCAGTGTATCGGG
GGCGTCAGCGGGGGACATATCAATCCCGCGGTCACCTGTGCTATGTTGGTCACCGGGAAG
TGTTCATTCATCAGATCAGGACTATATATCGTTGCCCAGTGTGTCGGAGCAGTTGCTGGT
GCTGCTGTTCTACAGGGTATTACTCCGGAAGCAGTCAGGGGA------------------
------------------------------AACCTAGGATCTACACTGATTCACTCCGGA
---------CTGACACCTGTCATGGGGCTGGGAGTAGAATTTTGCATCACTTTTATCTTG
GTGCTCACTGTCTTTGCA---GCTTGTGATGAGAACCGGCTG------------------
---GACGTTCAA---------------GGGTCAATCCCGCTTGCCATCGGTCTTTCCATC
ACTGCATGCCATTGCTTTGCGATTCGTTATACGGGATCCAGTATGAACACAGCACGCTCT
TTTGGACCGGCAATGATGGCAGGA---------CGTTGGGAACACCACTGGATATATTGG
GTAGGACCAATATTAGGAGGAGTTAGTGCTGGACTACTGTACCAAAATGTATTTGCATGT
---CCACCACCAGAG---------------------------------CCAGAA------
------------------------AGC------------TTGGAAAGA------ATG---
AAACTT---CAA---AGTTTGCTTAAAATTAGA---GAGAAAGAA---------ATTATT
---ACAGACAGAACCACT
TGCATT--

>GAZS01059885_Acanthoscurria_geniculata_PripL1
ATGGGG--

---------------------------------CGTGTA---AGCGAA------------

---TTC
AGACGAATGTTTGGCATGGACGAAATGCAG---AACGTGAAT------CAGCTTGCTAAA
GCTCTGCTTGCCGAATTTTTGGGCACGGGATTTCTCGTCTTCGTCGGATGCGGATCCTGC
ACGGGGGGATGGGATAATGAT---------------------------------------
--TATAAACCCACG
ATGGTTCAGATCGCCTTGGCTTTCGGTGTCGCGGTTGCAACCATCGCTCAGTGCATCGGG
CACATCAGTGGGGGACATATCAATCCTGCGGTCACCTGTGCCATGATGGTGACGGGAAAG
TGTTCTTTTCTGAGGGCATTTCTCTATGTGCTCAGTCAGTGTGTGGGAGCGATAGCAGGA
GCAGCCGTCCTCAAAGCCGTTACCCCAGAGATACTCAAAAGT------------------
------------------------------AAACTCGGAATAACTGCCCTAGATCCAAAA
---------GTTACTCCTGTTCAGGGTCTCGGGATTGAGTTCTTGATCACATTTGTTCTG
GTTTTAACAGTGTTCGCC---GCTTGCGATGAAAACAGACTG------------------
---GACGTTCTA---------------GGCTCTGTTCCACTTGCTATCGGACTTTCAATA
ACGGCATGCCATTGTTTCGCAATAAAATACACAGGATCTAGCATGAATACGGCAAGATCT

TTCGGTCCAGCAGTCATCACAGGA---------ATTTGGGATCATCACTGGGTCTACTGG
GTTGGGCCGATCATGGGCGGTCTGGTAGCTGGTTTGCTGTACCAGCATGTATTTGCCTGC
---CCACCTCCAGCA---------------------------------CCAGAA------
------------------------GAC------------ATTGAGCGC------GTA---
AAAGTC---CAC---AGCCTGCTGCGCTTAAAG---GAGAAAGAG---------ATTATC
---ACTGACAGAACCACA
TGCATT--

>GCER01014178_Archegozetes_longisetosus_PripL
ATGTCT--

```
------------------------------------------------------------
------------------------TCGAATCAAACCCAA---TCTTCG------------
------------------------------------------------------------
---------------------------------------------------------ACT
CAAAAGACTTTGTCTCGATTTCTATCATCT---GAACGCGCG------GACCTCATAAGA
ACTGCTTTGGCCGAGTTCCTGGGAACCGCTATTCTCGTGCTTTTCACATGCGGTACTTGT
GTCTCAACTCAAAAACGGGGC---------------------------------------
------------------------------------------------------GACGCT
TTGGTCTCGACCGCACTCTCCTTCGGTCTGACCGTCATGTGTTTGGTTGCAACTTTGGGC
CATGTGTCCGGCTGTCATATCAACCCCGGAGTCACAGTTGGACTGCTGGCAAACGGACAG
CTGAGTCTCGTTCGAGCTGGAATTTATATTTTGGCGCAACTTCTCGGAGGCATTGGGGGC
GCTTCGGTTTTGGCAGCAGTTCTGCCAACGGGTGCCCAG---------------------
------------------------------GACTTGTGCGCCACTAAATTGAATGGC---
---------TTGAATGCATTTCAGGGAATTTGCGTGGAAACAGTGATTGTATTCCTTTTG
GTTTTTGTCATTTGCAAT---GTTTGTGAGTCGAATTCCAGT------------------
------------------------------TTGGCCCCCTTGACTATTGGTCTGGCAGTG
GCCACAGGCCATCTATTCGCGATCCCATTGACCTCATCGTCGATGAATACGGCGCGTTCA
TTTGGACCGGCAGTCATACAGGGA---------TATTGGCAGCATCATTATGTCTATTGG
ATTGGACCACTTCTTGGCGGAGCTCTGGCTGGAGTTTTTCACCGCTATATATTTCAA---
---AATAAGACATTA---------------------------------CACCGG------
------------------------GAT------------CAAGACAGC------------
AAATCA---------AATGCAACTGCAATTAAT---GAACCGCCG---------AATGTG
------------------------------------------------------ACAACA
AGCATA------------------------------------------------------
------------------------------------------------------------
------------------------------------------------------------
------------------------------------------------------------
------------------------------------------------------------
------------------------------------------------------------
------------------------------------------------------------
------------------------------------------------------------
------------------------------------------------------------
------------------------------------------------------------
------------------------------------------------------------
------------------------------------------------------------
------------------------------------------------------------
------------------------------------------------------------
------------------------------------------------------------
------------------------------------------------------------
------------------------------------------------------------
------------------------------------------------------------
------------------------------------------------------------
------------------------------------------------------------
------------------------------------------------------------
------------------------------------------------------------
------------------------------------------------------------
------------------------------------------------------------
------------------------------------------------------------
```


>GEYZ01017649_Platynothrus_peltifer_PripL
ATGTCT--

---------------------------------CAATCA---TCATCG------------

---ACT
CGAAAGAGTATGTCATTATTTCTATTATCA---GAAAAGGGA------GACATAATAAGA
GCCGCTTTGGCCGAGTTTTTGGGAACTGCTATTCTTGTGCTGTTCACATGCGGAACATGT
GTCTCAACAGGCGGTAGAGGC---------------------------------------
--GATGCA
TTGGTTTCGACCGCACTATCATTTGGTCTGACAGTGACCTGTTTGGCGGCAACTATAGGT
CACGTTTCCGGATGTCATATCAACCCCGGGGTGACAATCGGACTCTTAGCCAACGGACAG
TTAAATCCAATTCGGGCCATAGTTTACATATTGGCACAACTTCTCGGAGGAATTGGTGGC
GCGTCTATTCTAGCGGCTGTCCTCCCGACTGGAACTAAG---------------------
------------------------------AACTTATGTGCGACTTCTTTGAATGGA---
---------GTGACACCATTGCAGGGAATTGCTGTCGAAGCTTTTATCGTCTTTCTGCTC
GTCTTTGTCGTGTGCAAT---GTTTGTGAGTCTAACTCTAAT------------------
------------------------------TTAGCGCCCGTTACCATAGGTCTGGCTGTG
GCCACCGGTCACTTGTTTGCGATCCCATTAACTTCATCATCGATGAATACGGCGCGTTCA
TTCGGTCCTGCAATCATACAAGGA---------TATTGGGATCACCACTATGTTTATTGG
ATTGGACCACTTCTTGGCGGTGCATTCGGTGGCATAGCTCACCGATTTATATTCCAG---
---AACAAAGCACTT---------------------------------TATGGT------
------------------------AAA------------CGTTCATCT------ATT---
CCAACA---AAA------GAA---------------GAGCCAATA---------AACGTG
--ACA---

>GEYB01097695_Hermannia_gibba_PripL
ATGTCT--

---------------------------------TCGCAT---TCATCC------------

---ATT
CGAAAAAGTATGTCTGGATTCATAACGAAA---GACAAATCA------GATATAATAAGA
TCTATTTTGGCCGAATTCGTGGGCACTGCTATTCTCGTTCTGTTCACTTGTGGTACTTGT
GTTCAGACTTCTGGAAAAGGC---------------------------------------
--GATGCA
TTGGTCTCAACTGCTCTGTCATTCGGACTGACCGTCACCTGTTTGGCCGCCACAATAGGT
CACGTGTCTGGATGTCATATCAATCCCGGAGTAACGGTCGGTCTCCTAACTAACGGCCAG
ATCAGCCCAATTAAAGCTATCGCTTATATATTGTCTCAACTTCTTGGAGGCATTGCTGGC
GCATCTGTTTTGGCCGCTGTATTGCCGTCCGGGGCCGAT---------------------
------------------------------AATTTGTGTGCGACCGCACTCAACAAAGAT
---------ATCAGTCCAGTTCAGGAATTATTATCGAAGGCTTTATTGTATTTCTATTG

GTATTTGTAGTTTGCAAT---GTTTGTGAATCCAACTCAAAC------------------
------------------------------TTGGCTCCGGTCACTATCGGTCTGGCGGTG
ACCACTGGTCATCTCTTTGCGATACCATTAACATCTTCATCAATGAACACAGCGCGTTCA
TTGGGTCCGGCAGTCATACAAGGC---------TATTGGGATGATCATTATGTCTATTGG
GTTGGCCCACTCTTGGGAGGTGCTGTCGGAGGACTGTTTCATCGGTATATTTTTCAG---
---AATAAGACGTTT---------------------------------TATGGT------
------------------------AAA------------TGTTTATCA------GTT---
TCAACA---------TCTGGC---------------GAACCAACA---------AACGTA
--ACCACA
ACTATA--

>GEYP01056151_Hypochthonius_rufulus_PripL
ATGTCC--

---------------------------------AATACT---GCTAAC------------

---GGT
AGAGTTAATTTTTCAAAATTTATACTCTCT---GATCGCGGA------GATTTGATACGC
GCCGCATTAGCCGAGTTTATCGGTACGGCAATTCTCGTACTATTCACTTGTGGTACGTGC
GTTGCTTCGGGCCCACGCGCT---------------------------------------
--GATACT
CTTGTGGCCACTGCCCTATCATTTGGCTTAACCATTGTATGTCTCGCGGCGACATTGGGT
CATGTTTCCGGATGTCACATAAATCCGGGTGTAACCATTGGTCTCTTCCTGAACGGTCAA
ATCAATATAATGCGATCGGCAATTTATATAATCGCCCAACTTTTGGGTGGCATCGCAGGT
GCTGCCATTTTGGCGGGTGTACTCTCTACGGGCGCGGAA---------------------
------------------------------AATGTATGTGCGACACGATTAAATGGT---
---------ATTACACCATTACAGGGAATTATCGTTGAAACTGCAATCGTATTTCTATTG
GTTTTTGTCATTTGTAAC---GTATGCGAATCCAATTCAAAT------------------
------------------------------CTCGCGCCAATAACTATTGGTTTTGCTATA
ACCGCGGGACATTTATTTGCGATTCCACTGACATCATCCTCAATGAATACAGCGCGTTCA
TTTGGTCCTGCAATAGTACAGGGA---------TATTGGGATGATCATTACGTCTATTGG
ATCGGTCCACTATTAGGGGGCGCAATCGGGGGTTTGGCTCATCGATTCCTCTTCCCG---
---AATAAGACACTT----------------------------------TACACA------
------------------------AAA------------TGTACATCT------GTC---
CCA------------TCGGGA---------------GACCCGACA---------AATGTT
--ACCACA
ACCATT--

>GEYQ01017884_Steganacarus_magnus_PripL
ATGTCT--

------------------------TCGGGTCACAATTCA---TCGTCA------------

---ACT
CGAAAGGGTATTCCGAAAATGATCGCT------GATAGAAGA------GACATAATCAAA
GCGATTTTAGCCGAACTCATCGGTACCGCAATTTTGGTCATCTTTACGTGTGGCACATGT
GCTGCAACTGGAGAACATTTT---------------------------------------
--AAAAATGAGACA
CTAGTTTCCACATCGCTGTCGTTTGGTTTAACCGTAATGTGTTTGTGCGCAACACTCGGT
CACGTCTCAGGGTGTCACGTAAATCCTGGCGTTACAATCGGTCTGTTAGCGAATGGTCAA
GTGTCGATTATTCGCGCAGTTTTGTTCATTATCTCGCAAGTTATTGGTGGTGTTTGTGGC
GCTTCTATTCTTGCCGGTATTCTGAAGAGTGGTTCGACT---------------------
------------------------------AATTTATGCGTAACTAAACTGCATGGT---
---------TTGTCACCACTGCAAGGCATTATAGTAGAGTTTTTTATTGTATTTCTGCTG
GTATTTGTTGCTTGCAAT---GTGTGCGAATCAAACTCTAAT------------------
------------------------------TTGGCTCCAATTAGTATTGGATTAGCAGTC
GCTTGTGGTCATCTTTTTGCTATACCGTTAACCTCGTCATCAATGAATCCCGCGCGTTCG
TTCGGTCCCGCGCTTATTCAAAAC---------AAATGGGATAATCATTACGTGTATTGG
ATCGGACCTTTAATTGGTGGTGCATTTGCAGGTCTCACACATCGCTATTTATTTCCA---
---AATAAGACATTT---------------------------------TACAAT------
------------------------ACT------------TGTAAGATT------CAA---
AAACAT---------TCTGGC---------------GATCCAAGT---------GATGTC
--ACAACA
ACCATA--


```
------------------------------------------------------------
------------------------------------------------------------
------------------------------------------------------------
------------------------------------------------------------
------------------------------------------------------------
------------------------------------------------------------
------------------------------------------------------------
------------------------------------------------------------
------------------------------------------------------------
------------------------------------------------------------
------------------------------------------------------------
------------------------------------------------------------
------------------------------------------------------------
------------------------------------------------------------
------------------------------------------------------------
------------------------------------------------------------
------------------------------------------------------------
------------------------------------------------------------
------------------------------------------------------------
------------------------------------------------------------
------------------------------------------------------------
------------------------------------------------------------
------------------------------------------------------------
------------------------------------------------------------
------------------------------------------------------------
------------------------------------------------------------
------------------------------------------------------------
------------------------------------------------------------
------------------------------------------------------------
------------------------------------------------------------
------------------------------------------------------------
------------------------------------------------------------
------------------------------------------------------------
------------------------------------------------------------
------------------------------------------------------
>GCAC01003772_Panonychus_ulmi_PripL
ATGTCC------------------------------------------------------
------------------------------------------------------------
---------------------------------AAAGACGATTCAACT------------
------------------------------------------------------------
---------------------------------------------------------GTT
TCTTCACCTTTT---AAATGGTTAATTTCA---TCTAAAGGT------GAACTGTTGAGA
TCTTTTCTTGCTGAACTAATTGGAACTGCGATTCTGGTCATTTTTACTTGTGGCACTTGT
ACGGCAACAGGTTCAAAAGCA---------------------------------------
------------------------------------------------------GATACC
CTTGTGTCCACTGCCCTTTCATTTGGATTAACTGTCGCTTGTTTGGTTGCTACTTTGGGT
CATGTTTCCGGTTGTCATATTAACCCAGCGGTTACAATTGGTCTTCTAGTCAATGGTCAA
GTTTCACTTGTTCGGTCAATCGTCTACCTGATCGCCCAACTCATCGGTAGTGTCGCTGGT
```

GCAGGTCTACTTTATGGTGCTCTC---ACCAAGCCGGAG---------------------
------------------------------ACACTTTGTGCCACCGTCTTATCG------
---------GTTGGCCTGATCCAAGGGATAATCATCGAGTGCTTAATCTGTTTCCTTTTG
GTATTAACTGTTTGCAAT---GTTTGCCAATCAAATCCGTCC------------------
------------------------------AATGCTCCGCTCTTAATTGGCCTCGCAGTG
ACCACTGGTCACTTATTTGCGATTCCATTGACTTCTTCATCAATGAACACTGCACGATCT
TTTGGACCTGCAGTTATCGCCGGA---------GTTTGGGACAATCATTGGGTCTATTGG
GTTGGTCCTTTACTCGGTGGTGCTCTTGGTGGTTTTGTCCATCGATTCATTTTCCTC---
---AACAAATCAATT---------------------------------TACGAT------
------------------------AAA------------TGTGAAACC------TCA---
GCCAAA---GGA---AACGAT---------------GAAGTACCA---------AATGTG
--ACAACA
TCTATT--

>GIIF01001683_Panonychus_citri_PripL
ATGTCC--

---------------------------------AAAGACGATTCAACT------------

---GTT
TCTTCACCTTTT---AAATGGTTAATTTCA---TCTAAAGGT------GAACTGTTCAGA
TCTTTTCTTGCTGAATTAGTTGGAACTGCGATTCTGGTCATTTTTACCTGTGGTACTTGT
ACGGCGACAGGTACACGACCT---------------------------------------
--GATACC
CTTGTGTCCACTGCCCTTTCCTTTGGATTAACTGTCGCTTGTTTGGTCGCTACTTTGGGT
CATGTTTCCGGTTGTCATATTAATCCAGCGGTTACAATTGGTCTTCTGGTCAACGGTCAA
GTTTCACTTGTTCGGTCAATCGTCTACCTGATCGCCCAACTCATCGGCGGTGTCGCGGGT
GCCGGTCTACTTTATGGTGCTCTC---TCCAAACCGGAA---------------------
------------------------------ACACTTTGTGCCACCGTCTTATCG------
---------GTTGACCCGATCCAAGGGATAATCGTCGAGTGCTTAATCTGTTTCCTTTTG
GTATTAACTGTTTGCAAT---GTTTGCCAATCAAATCCGTCC------------------
------------------------------AATGCTCCGCTCTTAATTGGCCTCGCAGTG
ACCACTGGTCACTTATTTGCGATTCCATTGACTTCTTCATCAATGAACACTGCACGATCT
TTTGGACCTGCAGTTATCGCCGGA---------GTTTGGACAATCATTGGGTCTATTGG
GTTGGTCCCTTACTCGGCGGTGCTCTTGGTGGTTTTGTCCATCGATTCATTTTCCTC---
---AACAAATCAATT---------------------------------TACGAT------
------------------------AAA------------TGTGAAACC------TCA---
GCCAAA---GGA---AACGAT---------------GAAGTACCG---------AATGTG
--ACAACA
TCTATT--

>JR695181_Tetranychus_urticae_PripL
ATGTCT--

---------------------------------AAGGACGATTCAGGG------------

---GTA
ACCAATCCTTTA---ACTTGGCTGGTATCA---TCTAATGGT------GACTTGGTTCGA
TCTTTTCTGGCCGAGTTCATTGGAACAGCGATTCTGGTTATATTCACTTGTGGTACCTGT
ACAGCAACATCACAAAATCCC---------------------------------------
--GATACT
TTAGTATCTACTGCTTTATCTTTTGGGTTGACTGTTGCTTGCCTTGTCTTTACCCTTGGT
CATGTCTCTGGTTGCCACATTAATCCAGCAGTTACCATTGGTCTTCTAGTAAATGGTCAA
ATAAATCTTATCCGATCAATTGTTTACATGATTGCTCAATTAACTGGTGGTATCGCTGGT
GCTGGTTTACTTCATGTCATCCTAAAATCTAAAAGT------------------------
------------------------------AATCTTTGTGCCACTGCATTGAAA------
---------GTTGACCCTATCCAAGGAATCATCGTTGAGTGCCTTATATGCTTCCTCTTG
GTCTTAACTGTTTGCAAC---GTTTGCCAATCAAATCCA---------------------
---GAAATT------------------------GCTCCCATCATAATAGCCCTTGCAGTC
ACCACTGGTCATCTATTTGCGATCCCATTGACCAGTTCCTCGATGAACACTGCTCGATCT
TTCGGACCCGCTGTCATTGCCAAT---------TTTTGGGACCACCATTGGGTTTACTGG
GTTGGACCGTTGCTTGGTGGAGCTCTTGGTGGCTTCTTCCATCGATATATCTTCATC---
---AACAAATCCATC---------------------------------TATGAC------
------------------------CAA------------TGCGAAACT------TCG---
TCCAAA---GCC---AATGAT---------------GAAACTCCC----------AATGTG
--ACAACC
TCCATT--

>GILM01005123_Tachypleus_gigas_PripL4
ATGGCA--

---------------------------------GCTGGA---ACAAAT------------

---ATT
CGACAGATAGTAGGACTTGAGGATCTGGGG---AAAAAGAAG------GTATTGTCCGCT
GCTCTACTAGCAGAATTTCTGGGAACAGCTATATTAGTACTTATCGGCTGTGGTTCCTGT
ATCACCGGCTGGGACCTAGAC---------------------------------------
--TACTCCCCCACT

```
ATTGTCCAGATCGCCCTAGCCTTCGGTGTGACTGTAGGAACAGTAGTACAGTTTATTGGA
CACGTCAGCGGAGGACACATTAACCCTGCTGTCACCATAGGATTTCTGGTCGCCAAGAAG
ATTTCCCTACTTCGAGCAATACTTTATGTGATTGTCCAGTGTTTGGGGGCAATAGTAGGC
GCTGGTTTGCTAAAGGGGCTCACACCTTCTAACTTACAAGGA------------------
------------------------------AACTTAGGGGCTACAGTTGTGAACCAACAT
---------TTGAGCCCTGTCCAAGGCTTCGTCGTTGAGCTGTGCATCACTTTTGTCTTG
GTGTTCACAGTATTTGCC---TGTTGCGATGAAAACAGAGGT------------------
---GACATTAAA---------------GGATCTGCCCCTCTCGCAATCGGTTTGTCTGTG
GCAACCTGTCATATGTTTGCAATTAAATACACCGGGGCCAGTATGAATACAGCTCGTTCC
TTCGGACCTGCAGTTGTAGCTAAC---------ATTTGGGTGAACCATTGGGTGTATTGG
ATTGGGCCTATCCTTGGTGGTGTGATAGCAGCACTGCTGTATCAATTTATCTTTTCGGCA
---CCAATGTCTGCT---------------------------------AGTGAT------
------------------------GAA------------TATGAAGAT------ACG---
GAAAAA---------GTGGAATTAAAA---------GAAAAGCCA---------GAG---
------------------------------------------------------ACACCT
TCTGTT------------------------------------------------------
------------------------------------------------------------
------------------------------------------------------------
------------------------------------------------------------
------------------------------------------------------------
------------------------------------------------------------
------------------------------------------------------------
------------------------------------------------------------
------------------------------------------------------------
------------------------------------------------------------
------------------------------------------------------------
------------------------------------------------------------
------------------------------------------------------------
------------------------------------------------------------
------------------------------------------------------------
------------------------------------------------------------
------------------------------------------------------------
------------------------------------------------------------
------------------------------------------------------------
------------------------------------------------------------
------------------------------------------------------------
------------------------------------------------------------
------------------------------------------------------------
------------------------------------------------------------
------------------------------------------------------------
------------------------------------------------------------
------------------------------------------------------------
------------------------------------------------------------
------------------------------------------------------------
------------------------------------------------------------
------------------------------------------------------------
------------------------------------------------------------
------------------------------------------------------------
```


>RXZF01034588_Tachypleus_tridentatus_PripL4a
ATGGCA--

---------------------------------GCTGGA---ACAAAT------------

---CTT
CGACAGATACTAGGACTTGAGGACCTGGGG---AAAAAGAAG------GTATTGTCCGCT
GCTCTACTAGCAGAATTTCTGGGAACAGCTATATTAGTACTTATTGGCTGTGGTTCCTGT
ATCACCGGCTGGACCTAGAC--
--TACTCCCCCACT
ATTGTCCAGATCGCCCTAGCTTTCGGTGTGACTGTGGGAACAGTAGTACAATTTATCGGG
CACGTCAGCGGAGGACACATTAACCCTGCTGTCACCATAGGACTTCTGGTCGCTAAGAAG
ATTTCCCTACTTCGAGCAATACTTTATGTGATAGTCCAGTCTTTGGGGGCAATAGTAGGC
GCTGGTTTGCTAAAGGGGCTCACACCTTCTAACTTACAAGGA------------------
------------------------------AACTTAGGGGCTACAGTTGTGAACCAACAC
---------TTGAGCCCTGTCCAAGGCTTCGTCGTTGAGCTGTGCATCACTTTTGTCTTG
GTGTTCACAGTATTTGCC---TGTTGCGATGAAAATAGAGGT------------------
---GACATTAAA---------------GGATCTGCCCCTCTGGCAATCGGTTTGTCTGTA
GCAACCTGTCATATGTTTGCAATTAAATACACCGGGGCCAGTATGAATACAGCTCGTTCC
TTTGGACCTGCAGTTGTAGCCAAC---------ATTTGGGTGAACCATTGGGTGTATTGG
GTTGGTCCTATCCTTGGTGGCGTGATAGCAGCATTGGTGTATCAATTTATCTTTTCGGCA
---CCAACGCTAGCA---------------------------------AGTGAT------
------------------------GAA------------TATGAAGAT------ACG---
GAAAAA---------GTGGAATTAAAA---------GAAAAACCA---------GAG---
--ATACCT
TCTGTT--

>RQPD01000111_Tachypleus_tridentatus_PripL4b
ATGACA--

---------------------------------TCTGGA---ACAAAT------------

---ATT
CGGCAGATACTAGGACTTGAGGATCTGGGG---AAAAAGAAG------GTATTGTCCGCT
GCTCTACTAGCAGAATTTCTGGGAACAGCTATATTAGTACTTATTGGCTGTGGTTCCTGT
ATCACCGGCTGGGACCTAGAC---------------------------------------
--TACTCCCCCACT
ATTGTCCAGATCGCCCTAGCCTTCGGTGTGACTGTGGGAACAGTAGTACAATTTATCGGG
CACGTCAGCGGAGGACACATTAACCCTGCTGTCACCATAGGATTTCTGGTCGCCAAGAAG
ATTTCCCTACTTCGAGCAATACTTTATGTGATAGTCCAGTGTTTGGGGGCAATAGTAGGC
GCTGGTTTGCTAAAGGGGCTCACACCTTCTAACTTACAAGGA------------------
------------------------------AACTTAGGGGCTACAGTTGTGAACCAACAC
---------TTGAGCCCTGTCCAAGGCTTCGTCGTTGAGCTGTGCATCACTTTTGTCTTG
GTGTTCACAGTATTTGCC---TGTTGCGATGAAAACAGAGGT------------------
---GACATTAAA---------------GGATCTGCCCCTCTCGCAATCGGTTTGTCTGTA
GCAACCTGTCATATGTTTGCAATTAAATACACCGGAGCCAGTATGAATACAGCTCGTTCC
TTTGGACCTGCAGTTGTAGCCAAC---------ATTTGGGTGAACCATTGGGTGTATTGG
GTTGGTCCTATCCTTGGTGGCGTGATAGCAGCATTGGTGTATCAATTTATCTTTTCGGCA
---CCAACGCTAGCA---------------------------------AGTGAT------
------------------------GAA------------TATGAAGAT------ACG---
GAAAAA---------GTGGAATTAAAA---------GAAAAACCA---------GAG---

--ATACCT
TCTGTT--

>GILQ01008850_Carcinoscorpius_rotundicauda_PripL4
ATGGCA--

---------------------------------GCTGGA---ACAAAT------------

---ATT
AGACAGATACTAGGACTTGAAGATCTGGGG---AAAAAGAAG------GTATTGTCCGCT

```
GCTCTACTAGCAGAATTTCTGGGAACAGCTATATTAGTACTTATCGGCTGTGGTTCCTGT
ATCACCGGCTGGGACCTAGAC---------------------------------------
------------------------------------------------TACTCTCCCACT
ATTGTCCAGATCGCTCTAGCCTTCGGTGTGACTGTGGGAACAGTAGTACAATTTATCGGG
CACGTCAGCGGAGGACACATTAACCCTGCTGTCACCATAGGATTTCTGGTCGCCAAGAAG
ATTTCCCTACTTCGAGCAATACTTTATGTGATAGTCCAGTGTTTGGGGGCAATAGTAGGC
GCTGGTTTGCTAAAGGGGCTCACACCTTCTAACTTACAAGGA------------------
------------------------------AACTTAGGGGCTACAGTTGTGAACCAACAC
---------TTGAGCCCTGTCCAAGGCTTCGTCGTTGAGCTGTGCATCACTTTTGTCTTG
GTGTTCACAGTATTTGCC---TGTTGCGATGAAAACAGAGGT------------------
---GACATTAAA---------------GGATCTGCCCCTCTAGCAATCGGTTTGTCTGTA
GCAACCTGTCATATGTTTGCAATTAAATACACCGGAGCCAGTATGAATACAGCTCGTTCC
TTTGGACCTGCAGTTGTAGCCAAC---------ATTTGGGTGAACCATTGGGTGTATTGG
ATTGGTCCTGTCCTTGGTGGCGTGATAGCAGCACTGCTGTATCAATTTATCTTTTCGGCA
---CCAACGGTAACT---------------------------------AGTGAT------
------------------------GAA------------TATGAAGAT------ACG---
GAAAAA---------GTGGAGTTAAAA---------GAAAAACCA---------GAG---
------------------------------------------------------ACACCT
TCTGTT------------------------------------------------------
------------------------------------------------------------
------------------------------------------------------------
------------------------------------------------------------
------------------------------------------------------------
------------------------------------------------------------
------------------------------------------------------------
------------------------------------------------------------
------------------------------------------------------------
------------------------------------------------------------
------------------------------------------------------------
------------------------------------------------------------
------------------------------------------------------------
------------------------------------------------------------
------------------------------------------------------------
------------------------------------------------------------
------------------------------------------------------------
------------------------------------------------------------
------------------------------------------------------------
------------------------------------------------------------
------------------------------------------------------------
------------------------------------------------------------
------------------------------------------------------------
------------------------------------------------------------
------------------------------------------------------------
------------------------------------------------------------
------------------------------------------------------------
------------------------------------------------------------
------------------------------------------------------------
------------------------------------------------------------
```

```
------------------------------------------------------------
------------------------------------------------------------
------------------------------------------------------------
------------------------------------------------------------
------------------------------------------------------------
------------------------------------------------------------
------------------------------------------------------------
------------------------------------------------------------
------------------------------------------------------------
------------------------------------------------------
>XP_013785705_Limulus_polyphemus_PripL4
ATGGCA------------------------------------------------------
------------------------------------------------------------
---------------------------------GCTGGG---ACAAAT------------
------------------------------------------------------------
---------------------------------------------------------ATT
CGACAGATACTAGGACTTGAGGACCTGGCT---AAAATGAAG------GTACTGTCCGCG
GCTCTACTAGCAGAATTTCTGGGGACAGCTATATTAGTACTTATCGGCTGTGGCTCTTGT
ATCACTGGATGGGACCTAGAC---------------------------------------
------------------------------------------------TACTCTCCCACA
ATTGTCCAGATCGCCCTAGCCTTCGGTGTGACTGTGGGGACAGTAGTACAGTTTATCGGG
CACGTCAGTGGAGGACACATTAACCCTGCTGTCACCCTAGGATTTCTGGTCACCAAGAAG
ATTTCCCTTTTTCGTGCGATGCTTTATGTGATTGTCCAGTGTTTGGGGGCAATAGTAGGC
GCTGGTTTGCTAAAGGACTCACACCTTCCAACTTACAAGGA-------------------
------------------------------AACTTAGGGGCTACAGTGGTGAATCAGCAC
---------TTGAGCCCAGTCCAAGGCTTCGTAGTTGAGCTGTGCATCACTTTTGTCTTG
GTGTTCACAGTCTTTGCC---TGTTGCGATGAAAACAGGGGT------------------
---GACATTAAA---------------GGATCTGCCCCTCTCGCAATCGGTTTGTCTGTA
GCAACCTGTCATATGTTTGCAATTAAATACACCGGGGCCAGTATGAATACAGCTCGTTCC
TTCGGACCTGCGGTTGTAGCCAAC---------GTTTGGTCGAACCACTGGGTGTACTGG
ATTGGCCCTATCCTTGGTGGCGTGATAGCAGCACTGCTGTACCAGTTCATCTTCTCGGCA
---CCAACGCCACCT---------------------------------AGTGAA------
------------------------GAA------------TCTGAATAT------GCG---
GAAAAA---------GTGGAATTACGA---------GAGAAACAA---------GAG---
------------------------------------------------------ACACCT
TCGGTT------------------------------------------------------
------------------------------------------------------------
------------------------------------------------------------
------------------------------------------------------------
------------------------------------------------------------
------------------------------------------------------------
------------------------------------------------------------
------------------------------------------------------------
------------------------------------------------------------
------------------------------------------------------------
------------------------------------------------------------
------------------------------------------------------------
------------------------------------------------------------
```


>JAALXS010000003_Tachypleus_gigas_PripL5
ATGGCA--

---------------------------------GCTAGA---AGCAAG------------

---CTA
CGTCAAATGCTAGGGTCTGGAGACATTGCC---AACAAGAGG------GCACTGTCCGTA
GCTCTGTTGGCAGAGTTTTTGGGGACAGCTATATTAGTCCTAATCGGTTGTGGCTCTTGC
ATTACCGGCTGGAATTCGCAT---------------------------------------
--TACTCTCCCACT
ATTGTCCAGATAGCTCTGGCCTTCGGCGTGACCGTAGGGACAGTGGTACAATTCATCGGG
CATGTTAGTGGAGGACACATCAACCCTGCCGTTACTTTCGCTTTTCTGGTCACCGCGAAA
ATTTCCTTACTCCGCGCAGTCCTGTACGTTATCGTTCAATGCTTAGGAGGAATAGTAGGG
GCTGGTTTGCTGAAGGCACTCACACCGTCCAACCTGCAAGGA------------------
------------------------------AACTTAGGAGCCACTGTTCTTAGCGAGCAC
---------CTGAGCCCAGTCCAAGGCTTCGTTGTCGAGCTCTGCATTACCTTGGTTTTG
GTGTTTACAGTGTTTGCC---TGTTGTGACGAGAACAGACAA------------------
---GACATCAAA---------------GGATCCGCCCCCCTGGCCATCGGATTGTCCGTG
GCAGCCTGCCACATGTTTGCTATTAAATACACCGGATCCAGTATGAATACGGCTCGATCC
ATTGGACCAGCCGTCGTAGCCAAC---------ATCTGGACTGATTACTGGGTATACTGT
CTTGGTCCTATTCTCGGTGGAGTTATAGCAGGACTGTTGTACGATCATATTTTCTCAGCG

---CCAGCACCAAAG---------------CCAGCACCAACGCCATCTAGTGAT------
------------------------GTA------------ACCCAACAT------TTA---
GAAAAA---------GTGGAAGTGTAT---------------------------------

>QXHF01001748_Tachypleus_tridentatus_PripL5
ATGGCA--

---------------------------------GCTAGA---AGCAAC------------

---CTA
CGTCAGATGCTAGGGTCTGGAGACATCGCT---AACAAGAGG------GCACTGTCCGTA
GCTCTGTTGGCAGAGTTTTTGGGGACAGCTATATTAGTCCTAATCGGTTGTGGCTCTTGC
ATTACCGGCTGGGATTCCCAT---------------------------------------
--TACTCTCCCACT
ATTGTCCAGATAGCCCTGGCCTTCGGCGTGACCGTGGGGACGGTGGTACAGTTCATCGGG
CATGTTAGTGGAGGACACATCAACCCTGCCGTTACATTCGCTTTTCTGGTCACCGCGAAA
ATTTCCTTACTCCGCGCAGTCCTGTACGTTATCGTTCAATGCTTAGGAGGAATAGTAGGG
GCTGGCTTGCTGAAGGCACTCACACCGTCCAACCTGCAAGGA------------------
------------------------------AACTTAGGGGCCACTGTTCTTAGCGAGCAC
---------CTGAGCCCAGTCCAAGGCTTCGTTGTCGAGCTCTGCATTACCTTGGTTTTG
GTGTTTACAGTGTTTGCC---TGTTGTGACGAGAACAGACAA------------------
---GACATCAAA---------------GGATCCGCCCCCCTGGCCATCGGCTTGTCCGTG
GCAACCTGCCACATGTTTGCTATTAAATACACCGGATCCAGTATGAATACGGCTCGATCT
ATTGGGCCAGCCGTCGTAGCCAAC---------ATCTGGACTGATTATTGGGTATACTGT
CTTGGCCCTATTCTTGGTGGAGTTATAGCAGGACTGTTGTATGATCATATTTTCTCTGCG
---CCAGCACCAACG---------------------------CCACCTAGTGAA------
------------------------GTA------------ACCGAACAT------TTG---
GAAAAA---------GTGGAAGTGCAT---------------------------------

>VWRL01000813_Carcinoscorpius_rotundicauda_PripL5
ATGGCA--

---------------------------------GCTAAG---AGCAAC------------

---CTA
CGTAAGATGCTAGGGTCTGGAGACATTGCC---AACAAGAGG------GCACTGTCCGTA
GCTCTGTTGGCAGAGTTTCTGGGGACAGCTATATTAGTCCTAATCGGTTGTGGTTCTTGC
ATTACCGGCTGGAATTCGCAT---------------------------------------
--TACTCTCCCACT
ATTGTCCAGATAGCCCTGGCCTTCGGCGTGACCGTGGGGACAGTGGTACAGTTTATTGGG
CATGTTAGTGGAGGACACATCAACCCTGCCGTTACATTCGCTTTTCTGGTCACCGCGAAA
ATTTCCTTACTCCGTGCAGTCCTGTACATTATCGTTCAATGCTTAGGAGGAATAGTAGGG
GCTGGCATGCTGAAGGCACTCACACCGTCCAACCTGCAAGGA------------------
------------------------------AACTTAGGGGCCACTGTTCTCAGCGAGCAC
---------TTGAGCCCAGTCCAAGGCTTCGTTGTCGAGCTCTGCATTACCTTGGTTTTG
GTGTTCACAGTGTTTGCC---TGTTGTGACGAGAACAGACAA------------------
---GACATCAAA---------------GGATCCGCTCCCCTGGCCATCGGCTTGTCCGTG
GCAGCCTGCCACATGTTCGCTATTAAATACACAGGATCCAGCATGAATACGGCTCGATCT
ATTGGACCAGCCGTTGTAGCCAAC---------ATCTGGACTGATTACTGGGTATACTGT
CTCGGTCCTATTCTTGGTGGAGTGATAGCAGGACTGTTGTATGATCATATTTTCTCTGCC
---CCAGCATCAACG---------------------------CCATCTAGTGAA------
------------------------GTA------------ACCGAACAT------TTG---
GAAAAA---------GTGGAAGTGCAT---------------------------------

>AZTN01286763_Limulus_polyphemus_PripL5
ATGGCA--

---------------------------------GCTAGA---AGCAAC------------

---GTA
CGTCAGATGCTAGGGTTTGGAGACCTGGCC---AACAAGAGG------GCACTGTCCGTA
GCTCTGTTCGCGGAGTTTATAGGGACAGCTATACTGGTCCTGATCGGTTGCGGCTCTTGC
ATTACCGGCTGGGATTCGGAT---------------------------------------
--TACTCTCCTACT
ATTGTCCAGATAGCCCTGGCTTTCGGCGTGACCGTGGGGACGGTGGTACAGTTTATCGGG
CATGTCAGTGGAGGACACATAAACCCTGCCGTTACATTCGCTTTTCTGGTCACCGCGAAG
ATCTCCTTACTCCGTGCAGTCCTGTACATTATCGTCCAGTGCTTAGGGGGAATAGTGGGT
GCCGGCTTGCTGAAGGCACTCACACCCTCCCACCTGCAAGGA------------------
------------------------------AACTTGGGAGCCACTGTTCTAAGCCAGCAC
---------CTGAGCCCCGTCCAAGGCTTCATCGTCGAGCTCTGCATTACCTTGGTTTTG
GTGTTCACAGTGTTCGCC---TGTTGTGACGAGAACAGAGGA------------------
---GACATCAAA---------------GGATCCGCACCCCTAGCCATCGGCTTGTCCGTG

GCAGCCTGCCACATGTTTGCTATAAAATACACCGGATCCAGTATGAATACTGCTCGATCT
ATTGGACCAGCCGTCGTAGCCAAC---------ATCTGGACTGATCACTGGGTGTACTGC
CTTGGTCCTATTCTTGGTGGAGTTATAGCAGGACTGTTGTATGATCATATTTTTTCTGCA
---CCAGCATCAGCA---------------------------CCACCTAGTGAA------
------------------------GTA------------ACCGAATAT------GTG---
GAAAAA---------GTGGAAGTAAGT---------------------------------

>GILM01008056_Tachypleus_gigas_PripL6

```
ATGTCA------------------------------------------------------
------------------------------------------------------------
---------------------------------GAGAGA---AACAAG------------
------------------------------------------------------------
---------------------------------------------------------TTC
CAAGACATGATAGGATACGGGGAGTTTAAG---AACAAGAAG------TCATTTGGAAGA
GCCTTATTGGCAGAATTTCTTGGTACGGCTGTACTAGTCTTAATCGGATGTGGCTCTTGC
ATTAAGGGTTGGGACAAGGAC---------------------------------------
------------------------------------------------TATTCTCCGACC
ATTGTCCAGATTGCTTTAGCCTTCGGTGTGACCGTAGGAACAGTAGTTCAATTCATTGGT
CACGTCAGTGGAGGACACATTAACCCAGCGGTTACTGTGGCGTTTCTGATAACCAGAAAA
ATATCTATCTTCCGTGCAATCCTCTATATCGTTGTCCAGTGCTTAGGGGCAACTGTTGGT
GCATCTGTGCTGAAAGGAGTTACCCCTTTTAAACAGCAAGGA------------------
------------------------------GAGTTAGGAGCAACGCTTATTCATGATGAC
---------ATGAGTGCCGTCCAAGGCTTTGCTGTTGAACTTTTAATCACTTTTGTTTTG
GTTTTCACTGTATTTTCC---TGTTGTGACACAAACAGACAT------------------
---GACGTCAAA---------------GGGTCCACTCCTTTAGCTATCGGCCTCGCAGTT
GCCACTTGCCACATGTTTGCTATCAAATACACTGGATCCAGTATGAACTCGGCTCGATCG
TTTGGTCCCGCAGTTATGGCCGAC---------GTCTGGCTACACCACTGGGTGTACTGG
ACAGGGCCTATTCTTGGAGGTATTATAGCAGGGCTCTTGTACGAGCACATTTTTGCTGCT
---TCAGCGCCACCT---------------------------------AATGAC------
------------------------GAA------------CTGGACAAA------GTA---
GAAACA---------GCAGAACTCAAA---------------------------------
------------------------------------------------------------
------------------------------------------------------------
------------------------------------------------------------
------------------------------------------------------------
------------------------------------------------------------
------------------------------------------------------------
------------------------------------------------------------
------------------------------------------------------------
------------------------------------------------------------
------------------------------------------------------------
------------------------------------------------------------
------------------------------------------------------------
------------------------------------------------------------
------------------------------------------------------------
------------------------------------------------------------
------------------------------------------------------------
------------------------------------------------------------
------------------------------------------------------------
------------------------------------------------------------
------------------------------------------------------------
------------------------------------------------------------
------------------------------------------------------------
------------------------------------------------------------
------------------------------------------------------------
------------------------------------------------------------
```


>GGKL01005703_Tachypleus_tridentatus_PripL6
ATGTCA--

---------------------------------GAGACA---AACAAG------------

---CTC
CAAGACATGATAGGGTACGGGGAGTTTAAG---AATAAGAAG------TCATTTGGAAGA
GCCTTATTGGCAGAATTTCTTGGTACGGCTGTACTAGTCTTAATCGGATGTGGCTCTTGC
ATTAAGGGTTGGGACAAGGAC---------------------------------------
--TATTCTCCGACC
ATTGTCCAGATTGCTTTAGCCTTCGGTGTGACCGTAGGAACAGTAGTTCAATTCATTGGT
CACGTCAGTGGAGGACACATTAACCCAGCGATTACTGTGGCGTTTCTGATAACCAGAAAA
ATATCTATCTTCCGTGCAATCCTCTATATCGTTGTCCAGTGCTTGGGGGCAACTGTTGGT
GCATCTGTGCTGAAAGGAGTCACCCCTTTTAAACAGCAAGGA------------------
------------------------------GAGTTAGGAGCAACGCTTATTCATGATGAC
---------CTGAGTGCCGTCCAAGGCTTTGCTGTTGAACTTTTAATCACTTTTGTTTTG
GTTTTCACTGTATTTTCC---TGTTGTGACACAAACAGACAA------------------
---AACGTCAAA---------------GGGTCCACTCCTTTAGCTATTGGCCTCGCAGTA
GCCACTTGCCACATGTTTGCTATCAAATACACCGGATCCAGTATGAACTCGGCTCGATCG
TTTGGTCCTGCTGTTATGACCAAC---------GTCTGGCTACACCACTGGGTGTATTGG
ACAGGGCCTATTCTTGGAGGTATTATAGCAGGGCTCTTGTACGAGCATATTTTTGCTGCT
---TCAGCGCCACCT---------------------------------AATGAC------
------------------------GAA------------CTGGACAAC------GTA---
GAAACA---------GCAGAACTCAAA---------------------------------

>VWRL01000063_Carcinoscorpius_rotundicauda_PripL6
ATGCCA--

---------------------------------CAGAGA---AACAAG------------

---CTC
CAAGACATGATTGGGTACGGAGAGTTTAAG---AACAAGAAA------TCATTTGGAAGA
GACTTATTGGCAGAATTTCTTGGTACGGCTGTACTAGTCTTAATCGGATGTGGCTCTTGC
ATTAAGGGTTGGGACAAGGAC---------------------------------------
--TATTCTCCGACC
ATTGTCCAGATTGCTTTAGCCTTCGGTGTGACCGTAGGAACAGTAATTCAATTCATTGGT
CACGTCAGTGGAGGACACATTAACCCAGCGGTTACTGTGGCGTTTCTGATAACCAGAAAA
ATATCTATCGTCCGTGCAATCCTCTATATCGTTGTCCAGTGCTTGGGGGCAACTGTTGGT
GCATCTGTGCTGAAAGGAGTCACCCCTTTTAAACAGCAAGGA------------------
------------------------------GAGTTAGGAGCAACGCTTATTCATGATGAC

```
---------CTGAGTGCCGTCCAAGGCTTTGCTGTTGAACTTTTAATCACTTCTGTTTTG
GTTTTCACTGTATTCTCC---TGTTGTGACACAAACAGACAA------------------
---GACGTCAAA---------------GGGTCCACTCCTTTAGCTATCGGCCTTGCAGTA
GCCGCTTGCCACATGTTTGCTATCAAATACACTGGATGCAGTATGAACTCGGCTCGATCG
TTTGGTCCTGCTGTTATGGCCGAC---------GTCTGGCTACACCACTGGGTGTATTGG
ACAGGGCCTATTCTTGGAGGTATTATAGCAGGGCTCTTGTACGAGCACATTTTTGCTGCT
---TCAGCGCCACCT---------------------------------AATGAC------
------------------------GAG------------CTGGACAAC------GTA---
GAAACA---------GCAGAACTCAAA---------------------------------
------------------------------------------------------------
------------------------------------------------------------
------------------------------------------------------------
------------------------------------------------------------
------------------------------------------------------------
------------------------------------------------------------
------------------------------------------------------------
------------------------------------------------------------
------------------------------------------------------------
------------------------------------------------------------
------------------------------------------------------------
------------------------------------------------------------
------------------------------------------------------------
------------------------------------------------------------
------------------------------------------------------------
------------------------------------------------------------
------------------------------------------------------------
------------------------------------------------------------
------------------------------------------------------------
------------------------------------------------------------
------------------------------------------------------------
------------------------------------------------------------
------------------------------------------------------------
------------------------------------------------------------
------------------------------------------------------------
------------------------------------------------------------
------------------------------------------------------------
------------------------------------------------------------
------------------------------------------------------------
------------------------------------------------------------
------------------------------------------------------------
------------------------------------------------------------
------------------------------------------------------------
------------------------------------------------------------
------------------------------------------------------------
------------------------------------------------------------
------------------------------------------------------------
------------------------------------------------------------
------------------------------------------------------------
```


>XP_013789474_Limulus_polyphemus_PripL6
ATGCCA--

---------------------------------GAAAGA---AACAAA------------

---CTC
CAAGACATTCTAGGGCTTCGAGAGTTTTCC---AACAAAAAG------TTCCTGGGAAAG
GCGTTATTGGCAGAATTCATCGGCACAGCCGTACTGGTCTTAATCGGTTGTGGCTCCTGC
ACTAAGGGTTGGGACACGGGC---------------------------------------
--TATTCTCCGACC
ATTGTCCAAATAGCTCTAGCCTTCGGTGTGACCGTAGGAACAGCAGTTCAATTTATTGGT
CACATCAGTGGAGGTCATATTAACCCGGCGGTTACCGTGGCGTTTTTGATAACCAGAAAA
ATATCCATCTTTCGTGCAGTTCTTTACATTGTTGTCCAGTGCTTGGGGGCGATTGTCGGT
GCTTCTGTACTAAAAGGAGTCACCCCTATTAAACAGCAAGGA------------------
------------------------------GACTTAGGAGCGACGCTTGTTCACAATAAC
---------CTGAGCGCCGTCCAAGGTTTCATCGTTGAACTTTTCATCACTTTTGTATTG
GTTTTCACTGTATTCTCC---TGTTGTGATACAAACAGACAA------------------
---GACATCAAG---------------GGATCTACTCCTTTAGCCATCGGCCTCTCGGTG
GCAACTTGCCACATGTTTGCTATCAAATACACTGGATCAAGTATGAATTCAGCTCGATCA
TTTGGACCTGCTGTCATAGCCGAC---------GTCTGGCTACATCACTGGGTGTACTGG
ATAGGGCCTGTTCTTGGAGGCGTTGTGGCAGGGCTCTTGTACGAGCACGTTTTTGCTGCT
---TCAGCGCCACCT---------------------------------AATGAC------
------------------------GAA------------CTTGACAAC------TTG---
GAAATA---------GCAGAACTCAGA---------------------------------

>DWQY01110261_Augiades_crinisus_Leip
ATGCCG--

------------------------------------TTT---ATTAAC------------

--GATATA
AAAACAAATCTCGGAATAGACGAGATGAAA---AACTATAAA------ATGTTGATCAGG
CAACTATTGGCGGAATTTATTGGCACATTTCTATATTTGTCAATCGTTTTGTTTGCGGGT
ACG------------ACC--
--CTTGCAGCTTCT
AATATCACAATCGCCTTCGCTAATGGATTGCTTATTGCCTCAGTCATCCAAATCATCCAA
CACATATCTGGTGGACATATCAACCCTGCCGTCACCATTGGCGTTCTGGTCTGTGGCAAA
ATCAAGATTTTGAAAGCAATTCTCTACATAATAGCCCAAATCTTGGGCTCGATTATAGGT
TCTATTGTGGCTTATGCAATTTCTGAAGAAGGTAGCCGTGGA------------------
------------------------------AACCTTGGAGCTACAGTTCCATTTGAAAGA
---------TCAAGAGTGGATCAAGTGTTTGCTTTTGAGTTTTTGATGACTTTTGTACTA
GTTGCTGTAGTTTTAAGT---GTTGTGGATATGAACCGCAGT------------------
---AACGGTTTG---------------GGGTCAGCCCCTTTAGCTATAGGCCTTAGTGTG
ACAGCTTGTCAATGTTCAGCTTTGAGTTACACAGGG---TCGTTGAACCCCGTTAGATCT
CTGGGACCTGCAGTAGTGATGAAC---------ATTTGGACGCATCATTGGGTCTACTGG
GTAGGACCTCTTCTGGGTGGTCTTGTGGCGGGACTCGTTTACAAATTTATATTTACT---

>GCDG01030046_Amphipyra_pyramidea_Leip
ATGGCG--

---------------------------------CAC------------------------

---AAA
AAAGACTGGCTCGGCATCAAAGAAATGAAA---GATCACAAA------ACAGTTTTCAAG
CAATTGTTCTCAGAATTTTTGGGCACATTTGTGTATTTGGCTTTAGTGTTGTCGGCGGGC
ATTGCC------CACGGT--
--ACCAACAACTCA
ACTGTTGTCGTCGCCTTGGCCAATGGACTGGTCGTTGCTAGTATTATCCAGATATTCGGT
CATGTATCCGGGAGTCATATAAATCCGGCTATAACGGTTGGAGCTTTGGTCTGTGGACAT

GTTAAGCCTTTGAAGGCCATGTGTTATGTCGTTATGCATGTTTTGGGCAGTATTGCTGGT
GCAGGAGTGGCCTACGCAGTATCAGCAGACACAATCCGAGGC------------------
------------------------------AACCTTGGAGCTGTGATACCCTACAAATAT
---------ACCACTCATGACCAGATCTTCATCTTAGAGTTCCTGATGACCTTCCTTGTA
GTAGCTCTTTATTTAAGC---GTAGTCGACACTCAGAGGGG-------------------
---CGAGGACTT---------------GGGTCCTCTCCTTTAGCTATAGGCGTATGCATC
ACAGGTTGTTTATGCTCCTGTATACCTTATACCGGG---TCTTTGAATCCTGTAAGGTCC
CTAGGACCAGCAGTTGTGATGGAT---------ATCTGGAATTTCATTGGGATACTGG
ATGGCACCCATTTTAGGTGGCCTTGCGGCTGGGGTGTTGTATAGATTTGTGTTACGAGTC
---TACCATAGG--

>ALA27396_Lepeophtheirus_salmonis_PripL
ATGCCT--

------------------------------TCGCGATTT---ATTCTT------------

---GCT
CTCCCCAAAATTGGACTGGATGAAATCAAA---AATCCATTA------TGGCTAATCAAA
TGTTTCATGGCAGAGTTTATTGGTACATTCATACTTGTTCTAATTGGGTGTGGATCTTGC
ATTGGA---GGGCAT---
---TCTGCTGAA
ATTGTTCGAATCTCATTTGCCTTTGGAATTGGTGTTGCAACAGCAGCTCAGAGCTTCGGT
CATATATCAGGGTGTCATGTGAATCCTGCTGTAACGGCTGGGTTATTTTGCGCTCGGAAA
ATTGGACTGATCAAAGGATTGTTCTACATTATTTTCAACTTCTTGGAGCTCTTGCAGCG
GCTTTTATTCTCTATCTAATTTCCAACAAATCCGTTAGAGGGGCTGAT------------
------------------------------CGCATTGGAATGACAAGTATTGATGAAAAC
---------TTAAATCAATTGCAAGGATTTGCAGTCGAGTTCTTTATTGTCTTTATTCTC
GTACTTGTCGTATTTGGGGCAGCTGCAGATGAAAATAAC---------------------
---GAAGTAAAA---------------GGATCGGCTCCATTGGCCATTGGATTAGCAATT
ACCTGTTGCCATCTTCTGGCAATTCCGTTTACTGGGTCAAGTATGAATCCTGCGAGAACA
TTTGGACCGGCTGTTATTCTAAAT---------GACTGGAAGGATCATTGGGTGTATTGG
GTTGGACCTATAATGGGTGGAGTTGCAGCTTCATATACCTATCAATTGTTTTTCCAAGCT
---CCTTCAAAACAGCGT---------------------------CCTTCTCAC------
------------------------TCCCTC---------GCTGAAGAA------TACGGA
ATTGTT---AGG------ACT---------------------------------------

>ACO10737_Caligus_rogercresseyi_PripL
ATGACG--

------------------------------TCCCGTTTC---CTTCTC------------

---TCG
TGTCCTAAAATCGGTCTGGATGAGCTTAAG---AACCCAGGC------TGGGTCATTAAA
TGCCTATTTGCGGAGTTTATTGGGACGTTTATACTAGTTTTGATTGGATGTGGATCTTGT
CTGGGA---GGGGAG---
---AAGGCGGAA
ATCGTACGAATCTCAATTGCCTTTGGCATCAGTGTCGCCTCGGCTGCACAGGCCTTTGGC
CACATTTCAGGCTGTCATATTAACCCTGCTGTCACGATTGGTCTCTTCTTTGGTCGGAAA
ATCGGTCTACTCAAGGGACTTCTTTACATTGCCGCCCAACTCTTGGGAGGACTCAGCGGA
GCCTTTATTCTCTATCTCATTTCTAACAAATCCGTTCGAGGAGCAAGC------------
------------------------------CGCATTGGAATGACGGCCATTGACGCTAAT
---------TTGTCACCGTTCCAAGGTTTTGCTGTGGAATTCTTTATAACTTTCATCCTG
GTATTGGTCGTCTTTGGAGCTGCGGGCGATGAAAACAAT---------------------
---GATGTCAAA---------------GGATCTCCTCCGTTAGCAATTGGGCTATCCATT
ACGGCGTGTCATCTTTTGGCGATTCCACTAACAGGTTCCAGCATGAATCCAGCTCGAACA
TTTGGACCCGCAGTTATTTTGAAT---------GACTGGACAAATCATTGGGTTTACTGG
GCTGGACCCTGTCTGGGCGGGATTTTAGCTTCTTATACCTATATGCTCCTCTTCACAGCA
---CCAACCAAG---CGA---------------------------CCCTTA---------
---------------------------------------GGAGATCAA------TACGGG
ATCGTA---CGG------ACTCAA------------------------------------

>GO416492_Lernaeocera_branchialis_PripL
ATGGCA--

--TCTTCC------------

---ATT
CTACCAGAGATCGGCCTCAGCGATTGCAAG---GACCCTTTC------GAGATGGTCAGG
TGCTTTCTCGCAGAGCTCATCGGGACCTTTTTTCTGGTGTTTGTTGGTTGTGGGTCTTGC
ACAGGC---GGTGAA---

---GGAGGCGAT
TTAGTGCGAATATCTTTGGCGTTCGGGCTAACGGTGGCGGCTTTAGCCCAAGCGATCGGT
CACATCTCCGGCTGTCATGTCAATCCAGCTGTAACGGCCGGGCTTTTTGTTGCAAGGAAA
ATTGGTCTGGTAAAGGGACTCATGTACATAGCCGCCCAATGCATAGGAGCCGTCATTGGA
GCCTTAACATTAAAGGTCTACAGTAATGAAAAAATAAGAGGATCCCAG------------
------------------------------TCTCTAGGAATGACTGGTATAAACGAGCAT
---------ATAGATTCTTATCAAGGATTTGGAATGGAGTTTATCATCACCTTTGCTTTG
GTGATCGTTGTGTTTGGAGCCGCGGCGGATGAGAATAAT---------------------
---TATGTAAAA---------------GGATCGCCCCCTCTGGCCATCGGCCTTACCATC
ACCGCCTGTCACCTTTTCGCTATACCATATACTGGTTCTAGTATGAATCCAGCTCGCTCA
TTCGGACCGGCTTTTGTGAATGGA---------CTGTGGACAAATCATTGGGTTTACTGG
GCAGGACCTATTCTTGGTGGAATAAGCGCTTCTTACATGTATCAACTTTTTTTCAAAGCA
---CCGTTAAAG---------------------------------CCCTTAATT------
---------------------------------------GTCAGAAAT------TTTGAA
GTT---

>JV192099_Tigriopus_californicus_PripL
ATGGCT--

--GGAAAC------------

---TTC
TTACCGTACCTAGGGATTGAGGAGATCAAG---CAAGTGGTT------CCCCTTCTCAAG
AGTCTCACCGCTGAGCTGATTGGAACTCTGCTGCTTGTGCTGATTGGTTGTGGCTCGTGT
TTGGGT---GGCCCT---
---TCGGCGGAC
TTTGTGCGAATCGCTCTTGCTTTTGGCGTGACGGTGGCTACCATAGCCCAAAGCATCGGA
CATATCTCAGGCTGCCACATCAATCCTGCTGTTACTGCCGGCCTCTTTGTGGGGCGCAAA
ATCGGCCTGATCAAAGCTTTGTTATACATTGTCGTTCAATGTTTGGGCGCCACCTTGGGT
TCGGGAATTCTCATGGCTTTGAGCAATGAGACGATCAGAGGCTACGGA------------
------------------------------GGCCTGGGGATGACAAGTGTGAATCCCAAA
---------ATTTCTGCTGGCCAAGCTTTCGGTATTGAATTCCTGATAACGTTTGTTCTA
ATATTGGTTATATTCGGAGCCGCTGCGGATGACAATAATACT------------------
---AATGTCAAA---------------GGATCGGCTCCTCTGGCCATTGGTCTCTCAATC
ACAACTTGCCATCTATTTGCCATACCCTTTACTGGATCCAGCATGAACCCGGCCAGAACC
TTTGGGCCTGCCCTTGTGTTAGGA---------GATTGGAACCATCACTGGGTCTATTGG
CTTGGCCCTATTTTGGGAGGGGTCTGTTCATCTCTGATCTACCAATTGATACTGCGTGCT
---CCCTCCTAC---GCC---------------------------CCAGTGAGC------
------------------------AAA------------GTGGAGACC------CATGGA
CCA---

>GCUK01047365_Vargula_hilgendorfii_PripL

---AGTATTGGG
CACGTGTCTGGAGGTCACATCAACCCGGCGGTCACAGTTGGCATGCTGGTAGCCGGTAAG
ATCAGTGTCATTATGGCCATCTGCTACATCATTGTCCAGTGCTGTGGAGCTATTGTTGGA
TCAGCGTTGTTGCAGGCTGTAACTCCTGAATTTCAAGAAGGG------------------
------------------------------TCCTTGGGCATGACTGCATTAGATTCTAAC
---------GTGACTCCCGCACAAGGTTTCGGTGTTGAAGTGATCATCACTTTTCTTCTG
GTCCTGGTCGTCTTTGCA---GCCACTGACGACCGTCGCAAT------------------
---GATGTCACC---------------GGGTCCGCTCCCCTGGCCATCGGCCTGTCAATC
ACAGCCGCTCATCTTGGAGCTATTCAGTACACAGGATCAAGTATGAATCCAGCTAGAACT
TTTGGCCCTGCTGTGATTGGAGGA---------CAATGGGAAAATCACTGGGTTTACTGG
GTCGGTCCCATCGCGGGAGGGTTGATAGCCTCCTCTCTGTACACCCTGGTCTTCAGACCA
---CCGAGA---------------------------------------CCTACA------
------------------------TCC------------GTCGACAGCGAC---AAGAAT

---------GAA---ATGACC---AAGTGC------------------------------

>KAF2355401_Trinorchestia_longiramus_PripL1
ATGGGT--

---------------------------------CGACTG---AAATCC------------

---TCT

GCTGACATTCTTGGAACACAAGAAATCAAG---ACTGTA---------GGTCTGTGGAAG
GCTTTAGCTGCAGAGTTTCTGGGGACCCTCATTCTTGTGCTATTTGGGTGCGCCTCTACC
CTCGGA------GAG---
---AAGACAGAC
ATCGTCAAAGTTTCCCTCACATTCGGCTTTACTCTCGCCTGCCTCATCCAGGCACTGGAC
CACATATCAGGATGCCACGTGAACCCCTCAGTGACTCTGGGCATGTTGGTTGCCCGCTAC
ATGTCTGTGACTCGCAGCATCCTGTATGTCGTCGCCCAGACACTCGGAGGAATTGCGGGA
GCTGGCATTCTTTACGGAATAACACCGACAGAAATTCAATAC------------------
------------------------------GGCATGGGTGTCAGCCGAGTGAACCCTGAG
---------CTGTCGGAGGGCCAGGCGTTCGGGGTTGAGGTTTTGTGCACCTTTTTGCTG
GTGTTCACCATCTTCGGT---ACTACAGACTCCCGCAGAACT------------------
---GACGTCAAG---------------GGCTCTATTTCCCTGGCCATTGGTCTCGCTGCG
GTCTGCACCCACTTGTTTGGCATTCCATACACCGGAGCATCCCTCAACCCTGCTCGCACC
TTCGGTCCCGCTCTTGTTGTGGGTGGCGAC---GCCTGGGCGCATCATTGGGTGTACTGG
GCGGGTCCGATCTGCGGGGGTATTTTGGGGGCACTTGTGTACCAGACCTTCTTTAGGGGG
---TCCCACAAGCCCGCCCCT------------------------CCCCCCTCC------
------------------------GAC------------CTCCCCTCATCC---AGAGCC
CTCATT---CCC---GTCACCACTAAGGAGCTC------AAAAACATG------GAAATG
--GAAGCC------

>GDUJ01079851_Talitrus_saltator_PripL1
ATGGGT--

---------------------------------CGACTA---AAATCC------------

---TCA
GCTGACATTCTTGGGACCCAAGAAATCAAG---ACTGTA---------GGCTTGTGGAAG
GCCTTAGCTGCTGAGTTTCTCGGGACCCTCATCCTGGTGCTGTTTGGGTGCGCCTCAACC
CTCGGA------GAA---
---AAGACAGAC
GTCGTCAAAGTGTCCCTCACTTTTGGTTTCACTCTGGCCTGCCTCATCCAGGCGCTGGAC
CACATCTCAGGATGTCACGTCAACCCGTCCGTCACGCTGGGCATGCTGGTGGCGCGCTAC
ATGTCGGTGACCCGCAGCCTCCTCTACGTCGTCGCTCAGACCCTCGGAGGAATCGCTGGA
GCTGGCATCCTCTACGGAATTACTCCTTCAAGTATTCAGTAC------------------
------------------------------GGCATGGGCGTCAGCCGCGTCAATCCAGAA
---------CTTTCGGACGGACAGGCGTTCGGAGTGGAGGTCTTGTGTACGTTTTTGCTG
GTGCTTACAATCTTCGGC---ACCACTGACACGCGCAGAACT------------------
---GACGTCAAA---------------GGCTCTGTCTCCATGGCCATTGGCCTAGCTGCG
GTCTGCACCCACTTGTTCGGCATCCCGTACACCGGCGCGTCCCTCAACCCTGCCCGCACC
TTCGGGCCCGCCCTCGTGGTGGGCGGTGAC---GCATGGGCCAGGCACTGGGTGTACTGG
GCAGGCCCCATCAGCGGGGGCATCCTGGCGGCCGTGGTGTACCAAGCCTTCTTCCGAGGC
---TCCCATAAGCCCGCTCCT------------------------CCCCCATCA------
------------------------GAT------------CTTCCCTCATCC---AGAGCC
CTCATC---TCG---GTCACCTCCAAGGAGCTC------AAGAACATG------GAGATG
--GAAGCC------

>XP_018023579_Hyalella_azteca_PripL1
ATGGGC--

---------------------------------CGCCTT---AAATCT------------

---TCG
GCCGATATCCTAGGCACTCAGGAGATCAAA---ACTGTA---------GGCCTATGGAAG
GCTTTGGCGGCCGAATTTTTGGGCACTCTGATCCTGGTGCTGTTCGGCTGCGCCTCGACC
CTCGAC------GAG---
---AAAACAGAC
ATCGTTAGAGTCTCACTCACCTTCGGCTTCACACTTGCATGTCTTATTCAGGCCCTGGGT
CACATCTCTGGATGCCACGTGAACCCCTCAGTGACGCTGGGCATGCTAGTCGCGCGCTAC
ATCTCCGTGACGCGCAGCATCCTGTACGTCGTCGCCCAGACGCTCGGTGGCATTGCAGGA
GCTGGCATCCTTTACGGCATCACACCTGAAAGCCTTCGATAT------------------
------------------------------GGCATGGGATGTACCCGCGTGAACCCTGAG
---------CTGACGGAAGGACAGGCGTTCGGCGTGGAGGTTTTGTGCACGTTCTTGCTG
GTCTTCACGATCTTCGGC---TCTACGGACTCCCGCAGGACG------------------
---GACGTTAAA---------------GGATCAATCTCCCTCGCTATCGGTCTCGCCGCC
ATCTGCACTCACATGTTTGGT---------------------------------------
---GTATACTGG

GCTGGTCCGGTATGCGGAGGTATACTTGGAGCCCTCGTGTACCAAGCCCTCTTCAGAGGA
---GCCCACAAGCCCACTCCC------------------------CCCCCCTCA------
------------------------GAC------------CTCCACACCGCC---CGCCCT
CTCATC---CCC---ATCACACACAAGGAGCTC------AAGAACCTG------GAGATG
--GAGGCC------

>GEPX01063587_Eulimnogammarus_cruentus_PripL1
ATGGGT--

---------------------------------CGCCTA---AAATCC------------

---TCC
TCCGATATTATTGGAACCCATGAGCTCAAA---ACCGTT---------GGCCTGTACAAA
GCTCTTGCCGCTGAGTTTATTGGCACTTTGATCCTCGTGTTGTTCGGCTGCGCGTCTACT
TTAGGC------GAG---
---AAAACCGAC
GTCGTCAAAGTGTCTCTTACCTTCGGCTTCACACTAGCATGTCTTATCCAGGCGTTTGAC
CACATTTCCGGGTGCCACGTGAACCCGTCCGTCACCCTGGGCATGCTGGTGGCCCGCTAC
ATCTCAGTCACCAGAACGCTGACTTACATCGTGGCACAGACCCTCGGAGGCATCGCTGGA
GCCGGCATCCTCTACGCCATCACCCCGGCCGCCAATCGCTTT------------------
------------------------------GGAATGGGTGTGAGCCACATCAACTCTGAG
---------ATGTCCGAGAGCCAGGCTTTCAGCGTGGAGCTCATCTGCACCTTCGTGCTG
GTCATCACCGTCTTTGGC---ACCACCGATAACAGGCGCACC------------------
---GACCTCAAG---------------GGAGCCGTCTCGTTGTCCATCGGCCTCGCTGCC
GTCTGCACACATCTTTTCGGGATCCCCTACACCGGTGCCTCCCTCAACCCCGCCCGTACC
TTCGGTCCCGCCCTCGTTGTTGGAGGCGAC---GCCTGGGCCCACCACTGGGTGTACTGG
GCTGGCCCAATCTGCGGCGGTGTGCTGGGGGCCCTAGTGTACCTGCACTTCTTCCGAGGA
---ACCCACGTGCCCCAAAAG------------------------CAGCCCAGC------
------------------------AAC------------CTGCCCGTGTCC---CGCCCC
CTCATC---CCC---ATCAACCAGAAGGAACTC------AAGAATCTC------GAGATG
--GAAGCG------

>GEQL01028723_Macropereiopus_wagneri_PripL1
ATGGGC--

---------------------------------CGCCTA---AAATCC------------

---TCC
TCCGATATTATTGGCACCCATGAGCTCAAA---ACAATT---------GGCCTATACAAA
GCTCTGGCCGCCGAGTTTATTGGCACTATGATCCTCGTGTTGTTCGGCTGCGCGGCTACT
TTAGGC------GAG---
---AAAACCGAC
GTCGTCAAAGTGTCGCTTACCTTCGGCTTCACACTAGCATGTCTTATCCAGGCGTTTGAC
CACATTTCCGGGTGCCACGTGAACCCGTCCGTCACCCTGGGCATGCTGGTGGCCCGCTAC
ATCTCAGTCACCAGAACGCTGACTTACATCGTGGCACAGACCCTCGGAGGCATCGCTGGA
GCCGGCATCCTCTACGCCATCACCCCAGCCGCCAATCGCTTC------------------
------------------------------GGAATGGGCGTGAGCCGCATCAACTCTGAG
---------ATGACCGAAAGCCAGGCTTTCAGCGTGGAGCTCATCTGCACCTTTGTGCTG
GTGATCACAGTCTTCGGC---ACCACCGATAACAGGCGCACC------------------
---GACCTCAAG---------------GGAGCCGTCTCGTTGTCCATCGGCCTCGCTGCC
GTCTGCACACATCTTTTCGGGATCCCCTACACCGGTGCCTCCCTCAACCCCGCCCGTACC
TTCGGTCCCGCCCTCGTTGTTGGAGGCGAC---GCCTGGGCCCACCACTGGGTGTACTGG
GCTGGCCCAATCTGCGGCGGTGTGCTGGGGGCCCTAGTGTACCTGCACTTCTTCCGAGGA
---AGCCACGTGCCCCAAAAG------------------------CAGCCCAGC------
------------------------AAC------------CTGCCCGTGTCC---CGCCCC
CTCATC---CCC---ATCAACCAGAAGGAACTC------AAGAATCTC------GAGATG
--GAAGCG------

>GEPB01055884_Oxyacanthus_flavus_PripL1
ATGGGC--

---------------------------------CGCCTA---AAATCC------------

---TCT
TCCGATATTATTGGCACCCATGAGCTCAAA---ACCGTT---------GGCCTGTACAAA
GCTCTTGCCGCCGAGTTTATTGGCACTTTGATCCTCGTGTTGTTCGGCTGCGCGTCAACT
TTAGGC------GAG---
---AAAACCGAC
GTCGTCAAAGTGTCTCTTACCTTCGGCTTCACACTAGCATGTCTTATCCAGGCGTTTGAC
CACATTTCCGGGTGCCACGTGAACCCGTCCGTCACCCTGGGCATGCTGGTGGCCCGCTAC
ATCTCAGTCACCAGGACGCTTACTTACATCGTGGCACAGACCCTCGGAGGCATCGCTGGA
GCCGGCATCCTCTACGCCATCACCCCGGCCGCCAATCGATTC------------------
------------------------------GGAATGGGCGTGAGCCACATCAACTCTGAG
---------ATGACCGAGAGCCAGGCTTTCAGCGTGGAGCTCATCTGCACCTTCGTGCTG
GTGATCACCGTCTTTGGC---ACCACCGATACCAGGCGCACC------------------

```
---GACCTCAAA---------------GGAGCCATCTCGTTGTCCATCGGCCTCGCTGCC
GTCTGCACACATCTTTTCGGGATCCCCTACACCGGTGCCTCCCTCAACCCCGCCCGTACC
TTCGGTCCCGCCCTTATTGTTGGAGGCGAC---GCCTGGGCCCACCACTGGGTGTACTGG
GCTGGCCCAATCTGTGGCGGTGTGCTGGGGGCCCTAGTGTACCTGCACTTCTTCCGAGGA
---ACCCACGTGCCCAAAAAA------------------------CAGCCTAGC------
------------------------AAC------------CTGCCCGTGTCC---CGCCCC
CTCATC---CCC---ATCAACCAGAAGGAACTC------AAGAATCTC------GAGATG
------------------------------------------------GAAGCG------
------------------------------------------------------------
------------------------------------------------------------
------------------------------------------------------------
------------------------------------------------------------
------------------------------------------------------------
------------------------------------------------------------
------------------------------------------------------------
------------------------------------------------------------
------------------------------------------------------------
------------------------------------------------------------
------------------------------------------------------------
------------------------------------------------------------
------------------------------------------------------------
------------------------------------------------------------
------------------------------------------------------------
------------------------------------------------------------
------------------------------------------------------------
------------------------------------------------------------
------------------------------------------------------------
------------------------------------------------------------
------------------------------------------------------------
------------------------------------------------------------
------------------------------------------------------------
------------------------------------------------------------
------------------------------------------------------------
------------------------------------------------------------
------------------------------------------------------------
------------------------------------------------------------
------------------------------------------------------------
------------------------------------------------------------
------------------------------------------------------------
------------------------------------------------------------
------------------------------------------------------------
------------------------------------------------------------
------------------------------------------------------------
------------------------------------------------------------
------------------------------------------------------------
------------------------------------------------------------
------------------------------------------------------
```

>GAKD01017822_Melita_plumulosa_PripL1
ATGGGT--

---------------------------------CGACTA---AAATCT------------

---TCC
GCTGACATCCTTGGCACCCAAGAGCTGAAG---ACCGTG---------GGTCTGTACAAG
GCTCTGGCTGCAGAGTTCATCGGCACTCTTATTCTGGTATTGTTCGGCTGTGCCTCTACG
CTCGGC------GAG---
---AAGACTGAT
GTCGTCAAGGTGTCGCTAACCTTTGGCCTTACTCTACAGTGTCTCATTCAGGCACTCGAC
CACATCTCGGGATGTCACGTGAACCCGTCCGTCACGCTGGGCATGTTGGTGGCCCGTTAC
ATGTCCGTCACCAGAACTTTGCTTTACATCGTCGCCCAGACGCTCGGAGGCATCGCAGGA
GCAGGCATTCTCTATGGCATCACTCCAGAGCCCCTGAGGTTC------------------
------------------------------GGTATGGGGGTCAGCAAGATCAACGCCGAG
---------CTGAGCGAAGGACAGGCCTTCGGGGTCGAAGTCCTCTGCACCTTCGTACTC
GTGCTCACCATCTTCGGC---TCCACTGACGAGAGGAGGACC------------------
---GATGTGCGC---------------GGCTCCATCGCCCTCGCTATCGGCATCGCAGCG
GTCGCCACACATTTATTCGGAATCCCCTACACCGGCGCCTCCCTGAACCCCGCGCGTACC
TTCGGGCCCGCCCTCGTGGTCGGGGGCGAG---GCCTGGGCCCACCACTGGGTTTACTGG
GTCGCCCCACCCGTTGGAGGAATGATGGCCGGACTCCTATACTCTCACTTCTTCCGCCAA
---GAGAAGCAG---------------------------------CAGCCCCAG------
------------------------CAG------------ACGCCATCTAAT---GACATC
GAAATC---GGC--


```
------------------------------------------------------------
------------------------------------------------------------
------------------------------------------------------------
------------------------------------------------------------
------------------------------------------------------------
------------------------------------------------------------
------------------------------------------------------------
------------------------------------------------------------
------------------------------------------------------------
------------------------------------------------------------
------------------------------------------------------------
------------------------------------------------------------
------------------------------------------------------------
------------------------------------------------------------
------------------------------------------------------------
------------------------------------------------------------
------------------------------------------------------
>GARO01009541_Echinogammarus_veneris_PripL1
ATGGGC------------------------------------------------------
------------------------------------------------------------
---------------------------------CGCCTG---AACACT------------
------------------------------------------------------------
---------------------------------------------------------CTC
CGAGAGATGGTGGGAGTGGAGGAGCTGAAG---AAAGAG---------GCTCTCTACAGG
GCACTGGCTGCTGAGTTCCTTGGAACGATGGTGCTGGTGATGATGGCTTGTGGGGTGGGC
TTCTAT------------------------------------------------------
---------------------------------------------------GGGACAGAC
---GTGGGAGTGGCACTCACCGTTGGCATAACACTCGGCACACTCATCCTGGCTCTTGGC
CACGTGTCGGGAGGTCACTTCAACCCCGCAGTGACCCTGGGCCTGACCACGTCCCGGTGC
ATCTCCCTAGTACGCGCCATGCTGTACGTGGCAGCACAGGTGGTGGGGGGCATCGCTGGA
GCAGCCATACTCTATGGCATAATGCCAGAGGACTTCCGTGAT------------------
------------------------------AATATGGGACTACTGACGATCAACGATAAG
---------ATGACAGAATGGCAGGCGTTTGGCATGGAGTTGATGCTCACCTTCCTCCTC
GTACTGACGATCGTATCA---GCAACCGATGAGCGGCGAACA------------------
---GATGTGAAA---------------GGAATGCTTCCTGTCGCTATCGGTCTCGTGGTG
GTCGCTGACATCTCCTTTGGAATGCACCTGAGCGGGGCGTGCATGAACCCTGCCCGCGCC
ATAGCTCCTGCCATCATCGTGGGGGGGAAGTCAGCCTGGACAGCACAGTGGGTGTACTGG
CTGGGTCCCCTCTCTGGTGGTGTTGTGGCCAGTGTGGTTCACACCCTCCTTTTCAGGAAC
---AAAAAGGAAGTAGTTATGGTC---------------TCTGTTAACGGAAAC------
------------------------GGCCAC---------CTCACCAGCAGC---CAGCAA
CACATA---ATC---ACA---GATCGCGACAGA------TTC------------------
------------------------------------------------------------
------------------------------------------------------------
------------------------------------------------------------
------------------------------------------------------------
------------------------------------------------------------
------------------------------------------------------------
------------------------------------------------------------
```


>GHCW01075749_Marinogammarus_marinus_PripL1
ATGGGT--

---------------------------------CGCTTG---AACTCT------------

---CTC
CGAGAGATGGTGGGGGTCGAGGAGCTCAAG---ACGCAG---------GCGCTGTACAGA
GCATTGGCCGCAGAGTGTCTCGGCACACTGGTGTTGGTGATGATGGCTTGTGGAGTGGGC
TATCAG--
---GCATCAGAT
---GTTGGAGTGGCGCTCACCTGCGGCATCACACTGGCCACTCTCATCCTGTCTCTGGGC
CACGTGTCGGGAGGCCACTTCAACCCCGCGGTGACCCTGGGCCTCACGATGTCGAGATGC
ACTTCCATAGTGCGGGCCATGCTCTACGTGGCATCACAGGTGGTGGGAGGCATCGCTGGG
GCCGGCATCCTCTATAGCATAATGCCAGACGAGTTCCGCAGT------------------

------------------------------GACATGGGAGTGCTGAAGATCAACGCCGCC
---------ATGACGGAGGAGCAAGCGTTTGGCATGGAGTTCATGTTCACCTTCATCTTC
GTCTTCACGATCGTGTCA---GCCACCGATGAGCGTCGAACA------------------
---GACGTTAAA---------------GGAGCGCTGCCTTTAGCCATAGGGCTGGTGCTA
ACCTCCAACATCTCATTCGGAATGCACCTGAGCGGAGCCTGCATGAACCCTGCCCGCGCA
ATAGCGCCCGCCATCATCGTTGGGGGACATCGGCTTGGGCTGGACATTGGGTATACTGG
CTGGGCCCCCTCGCCGGTGGGGCTGTGGCCAGTGTGGTGCACACGATCCTCTTCAGGAAG
---AAGAAGGACATTGTTAGGATG---------------GCTGTTAACGGGCAC------
------------------------AACCCC---------CTCACCAGCAGC---CAGCAA
CACATC---ATC---GTA---CAACCGGACAGA------TTC------------------

>GEQH01057555_Hyalellopsis_grisea_PripL1
ATGGGT--

---------------------------------CGCCTG---AAAACC------------

---CTT
CGAGAGATGGTGGGAGTAGAGGAGCTCAAG---ACAGGG---------GCGCTCTACAAG
GCCTTGGCCGCTGAATTCCTCGGCACGCTGGTGCTGGTCATGATGGCCTGTGGTGTGGGC
TACTCA--
---ACTGCCGAC
---ATCAGGGTACCCATCGCCGTCGGCCTCACCGTGGCCTCTCTCATCCTGGCTTTGGGC
CATGTGTCGGGGTGCCACGTGAACCCCGCGGTGACGATAGGTTTCCTGACGTCGCGGTAC
ACGTCCCTAGTCCGGAGCCTGCTCTACGTGGCGGCGCAGGTGGTGGGCGGCATCGCGGGA
GCCGCTATCCTCTACGGAATAATGCCGGAGGAATTCAGGAGC------------------
------------------------------AGCATGGGAGCGCTGAAGATCAACACTAGC
---------ATGACGGAGAGCCAGGCCTTTGGCATGGAGCTCATCCTCACCTTCACCTTA
GTGTTCACGATCCTGTCG---GCGACGGACGAGCGACGAACG------------------
---GACGTCAAG---------------GGATCGGTGCCGCTATCCATCGGGCTGGTGCTG
GTCGCCAACATCTCGTTCGGGATGAACCTGAGCGGGGCTTGCATGAACCCTGCCCGGGCG
TTGGCCCCTGCCATCCTCGTGGGAGAGCCCTGGGCATGGAATGGACATTGGGTGTACTGG
ATGGGCCCCCTCGCTGGCGGGGTGGTGGCTAGCCTCATCCACACTGTCTTCCGGGAGAAA
---AAGAAGGAGATTGTCATGTTA---------------TCCATTAACGGGCAA------
------------------------AACCAC---------ATGAGCTCCAGT---CAACAG
CATATC---ATA---ACA---CGGCCGGACAGC------TTC------------------

>GEPB01044867_Oxyacanthus_flavus_PripL1
ATGGGT--

---------------------------------CGCCTG---AAAACC------------

---CTT
CGAGAGATGGTGGGAGTAGAGGAGCTCAAG---ACAGGG---------GCGCTCTACAAG
GCCTTGGCCGCTGAATTCCTCGGCACGCTGGTGGTGGTCATGATGGCCTGTGGTGCGGGC
TACCCA--
---ACTGCCGAC
---ATCAGGGCACCCATCACCGTCGGCATCACCTTGGCCGCTCTCATCCTGGCTTTGGGC
CATGTGTCGGGGTGCCACGTGAACCCCGCGGTGACGATAGCTTTCCTGACGTCGCGGTAC
ACGTCCCTAGTCCGGAGCCTGCTCTACGTGGCGGCGCAGGTGGTGGGCGGCATCGCGGGA
GCCGCTATCCTCTACGGAATAATGCCGGAGACCTTCAGGAGC------------------
------------------------------AACATGGGAGCGCTGAAGATCAACCCTGAG
---------ATGACGGAGGTTCAGGCCTTTGGCATGGAGCTCATCCTCACCTTCACCTTC
GTGTTCACGATCCTGTCG---GCGACGGACGAGCGACGAACG------------------
---GACGTCAAG---------------GGATCAGTGCCGCTATCCATCGGACTGGTGCTG
GTCGCCAACATCTCGTTCGGGATGAACCTGAGCGGGGCTTGCATGAACCCTGCCCGGGCG
TTGGCCCCTGCCATCATCGTGGCAGAGCCCTGGGCGTGGAATGGACATTGGGTGTATTGG
ATGGGCCCCCTCGTTGGCGGGGTGGTGGCTAGCCTCATCCACACTGTCTTCCGGGAGAAG
---AAGAAGGAGATTGTCATGTTA---------------TCCATTAACGGGCAA------
------------------------AACCAC---------ATGAGCTCCAGC---CAACAA
CATATT---ATA---ACG---CGGCCGGACAGC------TTC------------------

>GHCU01006402_Echinogammarus_berilloni_PripL1
ATGGGT--

---------------------------------CGCTTG---AACACC------------

---CTC
CGGGAGATGGTGGGAGTCGAGGAGCTCAAG---ACAGCG---------CAACTGTACAGA
GCACTAGCCGCTGAGTTCCTCGGGTCGCTGGTGTTGGTGATGATGGCCTGTGGAGTGGGT
TACGGT--
--GCCGAA
GGCATCGGTGTAGCCGTCACCGTCGGCATCACTTTGGCCACTCTCATTCTGGGTATGGGC

```
CACGTGTCTGGAGGCCACTTCAACCCTGCGGTGACCATCGGCGTACTAACGTCGCGGTGC
ATCCCTGCGGTGAGGGCCGTGCTCTACGTGGCGTCGCAGGCGGTGGGGGGCATCGCAGGG
GCAGCCATACTCTACGGCATAATGCCAGAAGACTTCCAGACC------------------
-------------------------------AACATGGACTGCTGACGATCAACCCCGGC
---------ATGACGGAGGCTCAAGCGTTTGGTATGGAGCTCATGCTCACCTTCATTCTC
GTCTTTACGATAGTGTCA---GCCACAGACGAGAGGCGAACG------------------
---GACGTCAAG---------------GGAGCAGTGCCATTGTCAATTGGGCTGGTGGTT
ACCGCCAACATTTCCTTCGGGATGACACTGAGCGGGGCCTGCATGAACCCTGCCCGGGCG
CTGGCCCCCGCGGTCATCATTGGCACCGCGTCTGCGTGGACCTCTCAATGGGTGTACTGG
CTGGGCCCCATCGCTGGCGGGGTCGTGGCCAGCATTGTCCATACAAGCCTCTTCAGAGAG
---AAGAAGGATATCGTGATGGTG---------------GCCATCAATGGTCAC------
------------------------ACTAAC---------CTAGCCAACAGT---CAACAG
CACATC---ATC---GGC------CATGATAGG------TTC------------------
------------------------------------------------------------
------------------------------------------------------------
------------------------------------------------------------
------------------------------------------------------------
------------------------------------------------------------
------------------------------------------------------------
------------------------------------------------------------
------------------------------------------------------------
------------------------------------------------------------
------------------------------------------------------------
------------------------------------------------------------
------------------------------------------------------------
------------------------------------------------------------
------------------------------------------------------------
------------------------------------------------------------
------------------------------------------------------------
------------------------------------------------------------
------------------------------------------------------------
------------------------------------------------------------
------------------------------------------------------------
------------------------------------------------------------
------------------------------------------------------------
------------------------------------------------------------
------------------------------------------------------------
------------------------------------------------------------
------------------------------------------------------------
------------------------------------------------------------
------------------------------------------------------------
------------------------------------------------------------
------------------------------------------------------------
------------------------------------------------------------
------------------------------------------------------------
------------------------------------------------------------
------------------------------------------------------------
------------------------------------------------------------
```


>GFCV01088542_Gammarus_chevreuxi_PripL1
ATGGGG--

---------------------------------CGCCAC---AACACC------------

---CTC
AGGGAGATGGTGGGGGTCGCGGAGCTCAAG---ACGGGG---------GCGCTCTACAGG
GCCCTAGCCGCTGAATTCCTCGGCACGCTGGTGCTGGTCATGATGGCTTGCGGCGTGGGC
TACATG--
---GCCGACTCC
GCCCTCGGCGTGGCCTTCACATGCGGCATCACATTGGGGACCCTCATACTGGGTCTGGGC
CACGTGTCGGGGTGCCATGTCAACCCAGCGGTCACCCTGGGCACCCTAATATCCCGGTGC
ACGTCCCTAGTCAGGGCCTTGCTCTATGTGGCGGCGCAGGTGGTGGGAGGCATCGCGGGA
GCGGGCATCCTCTACGGCATAATGCCGAAGGACTTCCGTAAC------------------
------------------------------GACATGGGAGCGCTGACCATCAACCCAGGC
---------ATGACGGAGGGGCAGGCCTTCGGCATGGAACTCATGCTCACCTTCATCCTC
GCCTTCACGATCCTCTCG---GCCACGGACGAGCGGCGGACA------------------
---GATGTCAAG---------------GGATCGGTTCCGATATCCGTGGGGCTGGTGGTT
GTGGCCAACATCTCTTTCGGGATGCGGCTGAGCGGGGCGTGCATGAACCCTGCCCGAGCG
CTGGCACCCGCCATCATCGTGGGAAAGTCGTCGGTGTGGGCTGTCCAATGGGTGTACTGG
ATGGGCCCCCTGGCGGGCGGGGCAGTGGCTAGCGTGGTCCACACGGCCCTCTTCAGGGAG
---AAGAAGGAGGTGGTCATGGTG---------------TCCCTCAACGACCGC------
------------------------AACCAC---------GTCACCACCAGC---CAACAA
CATTTC---ATC---AGC------CCGGACCGC------TTC------------------


```
------------------------------------------------------------
------------------------------------------------------------
------------------------------------------------------------
------------------------------------------------------------
------------------------------------------------------------
------------------------------------------------------------
------------------------------------------------------------
------------------------------------------------------------
------------------------------------------------------------
------------------------------------------------------------
------------------------------------------------------------
------------------------------------------------------------
------------------------------------------------------------
------------------------------------------------------------
------------------------------------------------------------
------------------------------------------------------------
------------------------------------------------------------
------------------------------------------------------------
------------------------------------------------------------
------------------------------------------------------------
------------------------------------------------------------
------------------------------------------------------------
------------------------------------------------------
>GFVL01022146_Parhyale_hawaiensis_PripL2
ATGGGG------------------------------------------------------
------------------------------------------------------------
---------------------------------AAGTTA---AACACG------------
------------------------------------------------------------
---------------------------------------------------------TGC
ACCGATTTTCTTGGGGTGGGCGAAGTGACT---TCGTTC---------GACCTGTACAAA
GCCTTGATAGCCGAGTTCTGCGGGACGCTCCTGCTGGTGCTGGTGGGCTGCGGGTCCTGC
CTGGGG------------------------------------------------------
---------------------------------------------------GTCACCAAC
GTCACCAGGATCGCTCTTACTTTCGGACTCACCGTCGCTACCATTGCGCAGGCGGTGGGA
CACGTGTCAGGCTGCCACATCAACCCTGCCGTGACGCTGGGTATGTTGCTGGCCAGGTGC
ACGTCCCTCATCCGTGCGGTGCTCTACATCATCTTCCAGTGCATTGGTGCCATCACCGGG
GCCGCCATCTTGTATGGTCTGGTTCCCTCGGCGTTCGTTGGA------------------
------------------------------GGCCTGGGCAACACAGGAGTGGGTGCTGCT
---------GTGTCCGGCGGCCAGGCGTTCGGTATTGAGTTCTTCATCACATTCGTGCTT
GTGCTCACCGTGTTCGGC---GCCTGTGATGACCGGAGGGAG------------------
---GACGTCAAG---------------GGCTCTGTTCCCCTGGCCATCGGACTCTCCATC
ACAGCCTGCCATCTGTTCGCTGTACCCCTGACTGGATCTTCGATGAACCCCGCCCGCAGC
TTTGGCCCGGCGGTGGTGATGGGCGGCGAC---TACTGGAACTACCACTGGGTATATTGG
TTCGGACCCCTGCTCGGAGGTGCCTGTGCTGGCTTGCTGTACTCGCTGCTCTTCAGATCT
---CGAGGAGAG---------------------------------AATCAGAGC------
------------------------TAC------------TCACCTTCTGAG---ACGAGC
AAGTTC---TTG---AAG------CCCACCCGA------TCTTCG---------GAACCC
------------------------------------------------------------
```


>XM_047882375_Hyalella_azteca_PripL2
ATGGGG--

---------------------------------AAGCTG---AACTCG------------

---TGC
AGCGACATCATCGGTGCCCAGGAGCTGACC---AGCTTG---------GGATTATACAAG
GCCCTGGCTGCGGAGTTCATAGGCACCCTCCTGCTGGTGCTGGTGGGGTGTGGGTCGTGC

TTGGGT--
---GTCACCAAC
ACCACCAGGATCGCGCTCACCTTCGGCCTCACAGTCGCCACCATCGCACAGGCGATCGGC
CATGTTTCGGGGTGTCACATCAATCCCGCGGTGACGTTGGGGATGTTGATGGCACGGTGC
ACATCAGTGGTCAGAGCTGTGCTGTACATCATCTTCCAGTGCATCGGAGCCATCACTGGA
GCTGCCTTGCTTTACGGTCTGGTGCCATCATCGTTCGTTGGT------------------
------------------------------TCGCTCGGCAACACGGGCCTGAACTCTGCG
---------GTGTCGGGCGGCCAAGGCTTCGGCATCGAGTTCTTCATCACATTTGTGTTG
GTGCTGACGGTGTTCGGT---GCGTGTGATGACCGCAGGAGT------------------
---GATGTTAAG---------------GGCTCCGTGCCTCTGGCCATTGGGCTGTCCATC
ACTGCATGCCATCTCTTCGCGGTCCCTTTGACTGGCTCCTCCATGAACCCCGCACGCAGC
TTCGGCCCCGCCGTCATGATGGGTGATGAC---TACTGGAACGACCACAGGGTATACTGG
GTGGGCCCATTGGGCGGTGGTGCCTTGGCAGGGATCTTGTACTCGTACCTCTTCAGGCAG
---CCTAAGGCGCTCTC-----------------------------ACCACCAAC------
------------------------GAC------------ATCGAAATT-------------
---GGG---------AAG------CAGGATCGC------TTC------------------

>GDUJ01056168_Talitrus_saltator_PripL2
ATGGGA--

---------------------------------AAACTG---AACACG------------

---TGC
AGTGACATAGTAGGGGTGCAGGAGCTGATG---AGCTTC---------GGCCTGTACAAG
GCTGTAGTCGCCGAGTTCATAGGCACCCTGCTGCTGGTGCTGGTGGGCTGCGGATCCTGC
CTCGGT--
---GTCACCAAC
ACCACCAGGATTGCCCTCACTTTTGGCCTCACCGTCGCCACCATCGCCCAGGCCATCGGG
CACGTGTCGGGATGCCACATCAACCCGGCGGTGACACTGGGCATGGTGCTGGCACGCTGC
ACGTCCATCATACGCGCTGTGCTCTACATCATCTTCCAGTGCATTGGCGCCATCACTGGC
GCTGCTCTGCTCTATGGCCTCGTTCCAACTTCGTTCGTCGGG------------------
------------------------------TCCCTGGGCAACACGGGGCTGAACACGGCT
---------GTGTCGGGGGGCCAGGCCTTCGGAATTGAGTTTTTCATCACTTTTGTGCTG
GTGCTGACCGTCTTCGGG---GCGTGTGACGACAGGCGGGAC------------------
---GACGTCAAA---------------GGTTCTGTCCCTCTGGCCATCGGACTGTCCATC
ACCGCCTGCCATCTCTTTGCCGTCCCCCTGACTGGGTCATCGATGAACCCTGCACGGAGC
TTTGGTCCCGCCGTGGTGATGGGGGGTGAC---TACTGGAACTACCACTGGGTGTATTGG
GTGGGGCCCCTCGGCGGAGGTGCCCTGGCCGGGGTCTTGTATTCCTTCATCTTCCGTCAG
---CCCAAGAACATCCTC---------------------------TCCACCAAC------
------------------------GAC------------ATCGAAATC------------
---GGG---------AAA------CAGGATCGC------TTC------------------

>KAF2355402_Trinorchestia_longiramus_PripL2
ATGGGT--

---------------------------------AAACTA---AACACA------------

---TGC
AGTGATATAATAGGAGCACAGGAGCTAACA---AGCTTC---------GGTTTGTACAAG
GCTGTAGTGGCGGAGTTCATCGGCACGCTGTTGCTGGTGCTGGTGGGCTGTGGGTCGTGC
CTCGGC--
---GTCACCAAC
ACCACCAGGATCGCACTCACTTTTGGACTCACTGTCGCCACCATCGCACAGGCGATCGGG
CACGTTTCGGGATGCCACATCAACCCAGCCGTGACGTTGGGGATGGTGGTGGCCCGATGC
ACATCCCTGGTACGGGCAGTGCTCTACATCATCTTCCAGTGCATCGGTGCCATCACGGGG
GCCGCTTTGCTTTACGGGCTCGTTCCTACTTCATTCGTGGGA------------------
------------------------------TCCCTCGGCAACACGGGCCTGAACACGGCG
---------GTGTCTGGGGGCCAGGCTTTCGGTATTGAATTGTTCATCACTTTTGTGCTG
GTCCTGACGGTCTTTGGA---GCGTGTGACGACAGGAGAGAC------------------
---GACGTTAAA---------------GGATCGGTGCCTCTAGCCATCGGGCTGTCCATC
ACCGCCTGCCATCTCTTCGCGGTTCCCCTGACTGGATCATCGATGAACCCAGCCCGCAGC
TTTGGTCCTGCTGTTGTTATGGGGGGTGAC---TATTGGAACTATCACTGGGTGTATTGG
GTGGGTCCTCTGGGTGGTGGAGTTCTGGCCGGTGTCTTGTATTCCTTCATCTTCCGTCAT
---CCGAAGAACATCCTC---------------------------TCCACCAAC------

------------------------GAC------------ATCGAAATC------------
---GGG---------AAA------CAGGATCGC------TTC------------------

>GEPS01058213_Eulimnogammarus_cyaneus_PripL2
ATGGGC--

---------------------------------CGCCTC---AAGGGT------------

```
---------------------------------------------------------ATC
GGAGACGCCATTGGAGTAAAGGAGCTAACC---AAGCTG---------TGCCTGTACAAG
GCTCTAGGAGCGGAGTTCGTGGGCTCGCTGTTTCTGGTCCTGGTCGGCTGCGGCTCGTGC
CTGGGT------------------------------------------------------
----------------------------------------------------AACACCAT
GTCACCAGGATCGCCCTCACCTTCGGCATCACCGTGGCCACACTCGCTCAGGCTCTGGGC
CATGTTTCCGGGTGCCACATCAACCCCGCGGTGACTGCGGGCATGCTGGTGACCCGCTTC
GTGTCCGTGACGCGGGCCCTCCTCTACGTCCTAGCACAGGCGCTGGGAGCCATGACTGGA
GCAGGCATCCTTGTCGGTCTGGTCCCTGAGCAGCTAAAGGGT------------------
------------------------------TCGCTGGGCAACACGGGTCTGGGCCCAGGC
---------GTGACGCCGGAGCAGGGCTTCGGCATCGAGATGATGATCACGTTCGTGCTC
GTGCTGACGGTGTTCGGC---GTGTGCGACGAGCGCAGGATC------------------
---GACGTCAAG---------------GGCTCGGCACCTCTCGCCATCGGCCTCTCCATC
ACCGCATGTCATCTCTTCGCGGTCCCCCTGACCGGGTCCTCCATGAACCCCACACGCAGC
TTCGGCCCCGCCGTCGTCATGGGGGGAGAC---TACTGGATGAACCACTGGGTGTACTGG
GTAGGCCCCATTACAGGAGGCGTGCTGGCGGGCCTCGTCTACTCGTACCTGTTCAGGGAG
---TGCAAGACCGAGGGT---------------------------GGCCGCAAC------
------------------------GAC------------ATCGAGCTC------------
---GGG---------AAA------AACGATCGC------TAC------------------
------------------------------------------------------------
------------------------------------------------------------
------------------------------------------------------------
------------------------------------------------------------
------------------------------------------------------------
------------------------------------------------------------
------------------------------------------------------------
------------------------------------------------------------
------------------------------------------------------------
------------------------------------------------------------
------------------------------------------------------------
------------------------------------------------------------
------------------------------------------------------------
------------------------------------------------------------
------------------------------------------------------------
------------------------------------------------------------
------------------------------------------------------------
------------------------------------------------------------
------------------------------------------------------------
------------------------------------------------------------
------------------------------------------------------------
------------------------------------------------------------
------------------------------------------------------------
------------------------------------------------------------
------------------------------------------------------------
------------------------------------------------------------
------------------------------------------------------------
------------------------------------------------------------
------------------------------------------------------------
```

>GEQG01052742_Hyalellopsis_costata_PripL2

ATGGGC--

---------------------------------CGCCTC---AAAGGT------------

---ATC

GGAGATGCCATTGGAGTACAGGAGCTAACC---AAGGTG---------TGCCTGTACAAG

GCTCTGGGAGCGGAGTTCGTGGGCTCGCTGTTTCTGGTCCTGGTCGGCTGCGGCTCGTGC

CTGGGT--

---AACACCAAT

GTCACCAGGATCGCTCTCACCTTCGGCATCACCGTGGCCACACTCGCTCAGGCTCTGGGC

CATGTTTCCGGGTGTCACATCAACCCCGCGGTGACTGCGGGCATGCTGGTGACCCGCTTC

GTGTCCGTGACGCGAGCCCTCCTCTACGTCCTAGCACAGGCGCTGGGAGCCATGACTGGA

GCCGGCATCCTTGTCGGTCTGGTCCCTGAGCAGCTGAAGGGC------------------

------------------------------TCGCTGGGCAACACGGGTCTGGGCCCAGGC

---------GTGACGCCTGAGCAGGGCTTCGGCATCGAGATGATGATCACGTTCGTGCTC

GTGCTGACGGTGTTCGGC---GTGTGCGACGAGCGCAGGATC------------------

---GACGTCAAG---------------GGCTCGGCACCTCTCGCCATCGGCCTCTCCATC

ACCGCATGTCATCTCTTCGCGGTCCCCCTGACCGGCTCCTCCATGAACCCCACCCGCAGC

TTCGGCCCCGCCCTCGTCATGGGGGGAGAC---TACTGGATGAACCACTGGGTGTACTGG

GTAGGCCCGATTACAGGAGGCGTGCTGGCAGGCCTCGTCTACTCGTACCTGTTCAGGGAG

---TGCAAGACCGAGGGT---------------------------GGCCGCAAC------

------------------------GAC------------ATCGAGCTC------------

---GGG---------AAA------ACTGATCGC------TAC------------------

>GEPI01026331_Baikalogammarus_pullus_PripL2
ATGGGA--

---------------------------------CGCCTA---AAAGCT------------

---ATC
GGAGACGCCATTGGAGTGAAGGAGCTGACC---AAGGTG---------TGCCTGTACAAG
GCTCTGGGAGCTGAGTTCGTGGGCTCGCTGTTTCTGGTCCTGGTGGGCTGCGGCTCGTGT
CTCGGG--
---AACACCAAC
GTCACCAGGATCGCCCTCACCTTCGGCATCACGGTGGCCACTCTTGCTCAGGCTCTGGGC
CATGTCTCGGGGTGCCACATCAACCCCGCGGTGACTGCGGGCATGCTGGTCACCCGCTTC
GTGTCGGTGACGCGGGCGCTGCTGTATATCGTCGCGCAGGCGCTGGGGGCCATGACTGGG
GCAGGCATCCTTGTCGGTCTGGTGCCTGAGCAGCTGAAGGGA------------------
------------------------------TCGCTGGGCAACACCGGTCTGGGCCCAGGC
---------GTGACCCCGGAGCAGGGCTTCGGCATCGAGATGATGATCACCTTCGTGCTC
GTGCTGACGGTGTTCGGG---GTGTGCGACGAGCGCAGGGTT------------------
---GACGTCAAG---------------GGCTCGGCACCTCTCGCAATCGGCCTCTCCATC
ACCGCATGCCATCTCTTTGCGGTCCCCCTGACCGGCTCCTCCATGAACCCCACCCGCAGC

TTCGGCCCCGCAGTCGTCATGGGGGGAGAC---TACTGGATGAACCACTGGGTGTACTGG
GTGGGCCCCATTACAGGAGGCGTGCTGGCGGGCCTGGTCTACTCGTACCTGTTCAGGGAG
---AGCAAGACCCCGGGT---------------------------GGCCCCAAC------
------------------------GAC------------ATCGAGCTC------------
---GGC---------AAA---------GAACGC------TAC------------------

>GEID01080605_Callinectes_sapidus_PripL
ATGGGC--

```
------------------------------------------------------------
---------------------------------AAGATT---AAGGAT------------
------------------------------------------------------------
---------------------------------------------------------ACC
CGCGAGTACATGGGGGCCAGCGAAATCACG---CAGCTG---------GCTACCTGGAAG
GCGGTGGCGGCTGAGGTGGTGGGCACGATGATGCTGACCCTCGTGGTGTGTGGCTCCTGC
ATCGGA---TTCAAT---------------------------------------------
------------------------------------------------ACTCCACCCACC
AACGAACAGATCGCCTTCTCTGTCGGCATTATTGTCTTCTGCATGGTTCAGGCGCTGGGT
CACGTGTCGGGAGGTCACATCAACCCCGCAGTGACCTTAGCAATGCTGGTCGCCCGCTAC
GTGTCTGTGGTTCGCGCCCTGCTCTACATCATGGCCCAGTGTGTCGGTGCCCTGGCCGCC
TCTGCCATCTTGAAGGGCCTCACGCCACAGGAAAAGCAAGGC------------------
------------------------------AGCCTGGGAATGACGCAGCTGGGTGAGGGC
---------GTGAACAGTGGCCAAGGGTTCGGGGTGGAGCTCCTCATTACCTTCATTCTC
GTGCTGACGGTGTTCGGG---GTCTGTGACGAGCGACGCAAC------------------
---GATGTCAAA---------------GGGACAGGACCTCTCGCTATTGGCCTCTCCATC
ACTGCCAGCCATCTCGGAGCACTTCCCTATACTGGTGCAGCCATGAACCCCGCCCGCTCC
TTCGGCCCTGCTGTCATCACGGGC---------ATCTGGGCCAACCACTGGGTGTACTGG
GCCGGCCCCATCTCTGGCGGCATCCTCGCGGCGCTCCTCTACACCTACGTCTTCCGAGCA
---CCCAAGGCG---------------------------------GCCAGTTAC------
------------------------GAT------------CTCGAGATG------GACAAC
TACGGC---------AAG------AGGAACACC------CAACCC---------------
------------------------------------------------------------
------------------------------------------------------------
------------------------------------------------------------
------------------------------------------------------------
------------------------------------------------------------
------------------------------------------------------------
------------------------------------------------------------
------------------------------------------------------------
------------------------------------------------------------
------------------------------------------------------------
------------------------------------------------------------
------------------------------------------------------------
------------------------------------------------------------
------------------------------------------------------------
------------------------------------------------------------
------------------------------------------------------------
------------------------------------------------------------
------------------------------------------------------------
------------------------------------------------------------
------------------------------------------------------------
------------------------------------------------------------
------------------------------------------------------------
------------------------------------------------------------
------------------------------------------------------------
------------------------------------------------------------
------------------------------------------------------------
```


>GIXE01030638_Scylla_paramamosain_PripL
ATGGGC--

---------------------------------AAGATT---AAGGAT------------

---ACC
CGTGAGTACATGGGGGCCAGCGAGCTCACG---CAGCTG---------GCCACGTGGAAG
GCGGTGGCGGCGGAGGTGGTGGGCACGATGATGCTGACCCTTGTGGTGTGCGGCTCCTGC
ATCGGA---TTCACT---
--GCCCCACCAACC
AACGAACAGATCGCCTTCGCTGTCGGCATTATTGTCTTCTGTATGGTTCAGGCGCTGGGT
CACGTGTCGGGAGGTCACATCAACCCTGCGGTGACCTGCGCGATGCTGGTTGCCCGCTAC
GTGTCAGTGGTTCGCGCCCTGCTCTACATCATGGCGCAGTGTGTTGGTGCCCTGGCCGCC
TCTGCCATCCTGAAGGGTCTCACGCCGACGGACAAGCAAGGC------------------
------------------------------AGCCTGGGAATGACGCAGCTGGGTGAGGGC
---------GTGAACAGTGGCCAGGGCTTCGGGGTGGAGCTCCTCATTACCTTCATCCTC
GTGCTGACGGTGTTCGGG---GTGTGTGACGAGCGACGCAAC------------------
---GATGTCAAG---------------GGGGCAGGACCTCTCGCTATCGGCCTCTCCATC
ACCGCCAGCCATCTTGGGGCACTCCCCTACACCGGCGCTGCCATGAACCCCGCCCGCTCC
TTCGGTCCTGCTGTCATCACGGGG---------ATCTGGGCCAACCACTGGGTGTACTGG
GCTGGTCCCATCGCTGGAGGAATCCTCGCCGCGCTCCTCTACACCTACGTCTTCCGAGCA
---CCCAAGGCG---------------------------------GCCAGCTAC------
------------------------GAC------------CTCGAGATG------GACAAC
TACGGC---------AAG------AGGAACACC------CAACCA---------------

>XP_045129483_Portunus_trituberculatus_PripL1
ATGGGC--

---------------------------------AAGATT---AAGGAT------------

---ACC
CGTGAGTACATGGGGACCAGCGAGCTCACG---CAACTG---------GCCACCTGGAAG
GCAGTGGCGGCGGAGGTGGTGGGCACGATGTTGCTGACCCTCGTGGTGTGTGGCTCCTGC
ATCGGA---TTCAAC---
--ACTCCACCCACC
AATGAACAGATCGCCTTCGCTGTCGGCATTATTGTCTTCTGTATGGTTCAGGCGCTGGGT
CACGTGTCGGGAGGTCACATCAACCCTGCAGTGACCTTAGGAATGCTGGTTGCACGCTAC
GTGTCAGTGGTTCGCGCTCTGCTCTACATCATGGCCCAGTGTGTTGGTGCCCTGGCTGCC
TCTGCTATCCTGAAGGGCCTCACGCCGACGGACAAACAAGGC------------------
------------------------------AACCTGGGAATGACGCAGCTGGGTGAGGGT
---------GTGAACAGTGGCCAGGCCTTCGGGGTGGAGCTCCTCATTACCTTCATCCTC

GTGCTGACGGTGTTCGGG---GTGTGTGACGAGCGACGCAAC------------------
---GATGTCAAA---------------GGGACAGGACCTCTCGCCATCGGTCTCTCCGTC
ACCGCCAGCCATCTCGGGGCACTTCCCTACACCGGAGCTGCCATGAACCCCGCTCGCTCC
TTCGGCCCTGCAGTCATCACTGGC---------ATCTGGGCCAACCACTGGGTGTACTGG
GCTGGTCCCATCTCTGGCGGCATCCTCGCGGCACTCCTCTACACCTACGTCTTCCGAGCA
---CCCAAGGCG---------------------------------GCCAGCTAC------
------------------------GAC------------CTCGAGATG------GATAAC
TACGGC---------AAG------AGGAACACC------CAACCC---------------

>XM_045273547_Portunus_trituberculatus_PripL2
ATGGAG--

---------------------------------AAAAAG---AAGAAC------------

---GTA
CAGGAGATCTTTGGGCTGAGTGAGATGAAG---ACGTGG---------GCGTTGTGGCGG
GCAGTGGTGGCCGAGCTGATAGGCACGATGATGCTAGTGCTGGTGGTGACTGCTTCTGTG
CTGTCC---TGGACCCCTGTG---------------------------------------
---GCCGCTCCCCCC---
GTGGTGCAGATTTCCCTGGCTGTGGGGCTCGTGGTGGCTTCCATGGCTCAGGCGCTGGGT
CACGTGTCGGGAGGTCACATCAACCCTGCAGTGACCTTAGGAATGCTGGTTGCACGCTAC
GTGTCAGTGGTTCGCGCTCTGCTCTACATCATGGCCCAGTGTGTTGGTGCCCTGGCTGCC
TCTGCTATCCTGAAGGGCCTCACGCCGACGGACAAACAAGGC------------------
------------------------------AACCTGGGAATGACGCAGCTGGGTGAGGGT
---------GTGAACAGTGGCCAGGCCTTCGGGGTGGAGCTCCTCATTACCTTCATCCTC
GTGCTGACGGTGTTCGGG---GTGTGTGACGAGCGACGCAAC------------------
---GATGTCAAA---------------GGGACAGGACCTCTCGCCATCGGTCTCTCCGTC
ACCGCCAGCCATCTCGGGGCACTTCCCTACACCGGAGCTGCCATGAACCCCGCTCGCTCC
TTCGGCCCTGCAGTCATCACTGGC---------ATCTGGCCAACCACTGGGTGTACTGG
GCTGGTCCCATCTCTGGCGGCATCCTCGCGGCACTCCTCTACACCTACGTCTTCCGAGCA
---CCCAAGGCG---------------------------------GCCAGCTAC------
------------------------GAC------------CTCGAGATG------GATAAC
TACGGC---------AAG------AGGAACACC------CAACCC---------------

>XM_042365857_Homarus_americanus_PripL
ATGGGA--

---------------------------------AAGATC---AAGGAC------------

---ATG
CGCGATTACGTGGGCAAGGCCGAGCTGCGG---GAACTG---------GACCTGTGGAAG
GCGGTGGCGGCGGAGTTCTTGGGGACTATGGTGTTGGTCTTCGTCGGTACCAGCTCCTGC
CTCAACGGGTGGACTGAA--
---GGATACGCTCCCTCC
GTCGTCCAGATCTCCTTGTGCTTCGGCATCACCCTCGCTACTCTGGCACAGGCATTGGGT
CACATCTCAGGTTGCCACGTGAACCCGGCCGTGACCTGTGGGATGCTGGTAGCTCGTCAC
ATCTCGGTGGTGAGGTCCCTGTTCTACATCATCGCCCAGTGTGCTGGAGGACTGGTTGGA
TCGGCTATACTGTTGGGAGTGACGCCAGCTGAGCGACAGGGC------------------
------------------------------AGCCTGGGCATGACTTCCATCAACCCGGCC
---------GTGAACGTAGGCCAGGCGGTGGGTTTCGAGCTGCTCTTCACTTTCGTACTG
GTGCTGACGGTTTTCAGC---GTCTGTGACGAGCGAAGGAAC------------------
---GATGTCAAG---------------GGCTCGGCACCTCTAGCCATTGGCCTCTCCGTC
GCTGCCTGCCATCTCGCCGGCGTGCCCATCACAGGTGCCTCCATGAACCCGGCTCGCACC
TTTGGCCCAGCTGTCGTCGCCGGA---------CTTTGGGAGAACCATTGGGTGTACTGG
ACTGGTCCCATCCTCGGCGGGATGATGGCTGCCCTCATCTACACCTACCTGTTCAGGGCC
---CCCAAGATTACC------------------------------CCCAGCTAC------
------------------------GAC------------ATCAACCTG------GAGAAC
TGCAAT---------AAG------GGCGGCAAC------AAAGCC---------------

>AEI25531_Penaeus_monodon_PripL
ATGGGC--

---------------------------------AAAATC---AAGGAC------------

---ATG
AAGGAGTACATCGGCACGGGCGAACTCCTGAACGACCGCCGC---------GTGTGGAAA
GCCTTCCTGGCGGAGTTCCTGGGCACCATGTTCCTCGTGTTCATCGGCTGCGGCTCCTGC
ATCGGCTCCTGGAGCGAA--
---GGCTACGCCCCCAGC
GTCGTCCAGATCTCGCTCGCCTTCGGGGTCACCGTGGCGTCCATCGCGCAGGCCGTCGGT
CATGTCTCTGGCTGTCATATCAACCCAGCTGTGACATGCGCCATGCTTGTTGCTCGTCAT
GTCTCTGTCATCCGGGCACTCATCTACATCGTGTGCCAGTGCCTTGGTGCCATCGTAGGG

GCTGCCATTCTGAAGGGAGTGACCCCCGCAGACATCCAGGGG------------------
------------------------------TCCCTCGGCATGACCCTGAGAAATGAGAAG
---------ATTGACACGGCCCAGGCATTGGGCATCGAGCTCCTCATCACTTTCGTCCTA
GTCATCACCGTCTTCGGG---GCGTGTGATGAGCGAAGGAAT------------------
---GACGTGAAG---------------GGGTCAGCTCCTCTCGCCATTGGCCTGTCGATC
ACCACTTGTCATCTCTTCGCCGTCCCCATCACCGGATCCTCGATGAACCCGGCAAGGAGC
TTCGGTCCAGCGGTCATTTCCGGT---------CTCTGGCAGGACCACTGGGTGTACTGG
GCTGGTCCTATTCTTGGTGGTCTCGCAGCCGCTCTAATCTACTCCTACGTTTTCCGAGCT
---CCCAAGGACGCC------------------------------GCTGCTTAT------
------------------------GAC------------GTGGAGATG------GACAAC
TATAAC---------AAG------AGAACCAAC------AATGCT---------------


```
------------------------------------------------------------
------------------------------------------------------------
------------------------------------------------------------
------------------------------------------------------
>XM_047637140_Penaeus_chinensis_PripL
ATGGGC------------------------------------------------------
------------------------------------------------------------
---------------------------------AAGATC---AAGGAC------------
------------------------------------------------------------
---------------------------------------------------------ATG
AAGGAGTACATCGGCACGGGCGAACTCCTGAACGACCGCCGC---------GTGTGGAAG
GCCTTCCTGGCGGAGTTCTTGGGCACCATGTTCCTCGTGTTCATCGGCTGTGGCTCCTGC
ATCGGCTCCTGGAGCGAA------------------------------------------
---------------------------------------------GGCTACGCCCCCAGC
GTCGTCCAGATCTCGCTCGCCTTCGGGGTCACCGTGGCGTCTATCGCGCAGGCTGTCGGT
CATGTCTCTGGCTGTCATATCAACCCAGCTGTGACATGCGCCATGCTTGTTGCTCGTCAC
GTCTCTGTCGTCCGGGCACTGATCTACATCGTCTGCCAGTGCCTCGGTGCCATCGTAGGA
GCTGCCATTCTGAAGGGCGTGACCCCCTCAGATATCCAGGGC------------------
------------------------------TCCCTCGGCATGACCCTGAGGAATGAAAAG
---------ATTGACACGGCCCAGGCATTGGGCATCGAGCTCCTCATCACCTTCGTCTTG
GTCATCACCGTCTTCGGG---GCGTGTGATGAACGAAGGAAT------------------
---GACGTGAAG---------------GGGTCAGCTCCTCTCGCTATTGGCCTGTCGATC
ACCACTTGTCATCTCTTCGCCGTCCCCATCACTGGATCTTCGATGAACCCGGCAAGGAGC
TTCGGTCCAGCGGTCATTTCCGGT---------CTCTGGCAGGACCACTGGGTGTACTGG
GCTGGTCCTATTCTCGGTGGTCTTGCGGCCGCTCTTATCTACTCCTACGTTTTCCGAGCT
---CCCAAGGACACC------------------------------GCTGCTTTT------
------------------------GAC------------GTGGAGATG------GACAAC
TATAAC---------AAG------AGAACCAAC------AATGCT---------------
------------------------------------------------------------
------------------------------------------------------------
------------------------------------------------------------
------------------------------------------------------------
------------------------------------------------------------
------------------------------------------------------------
------------------------------------------------------------
------------------------------------------------------------
------------------------------------------------------------
------------------------------------------------------------
------------------------------------------------------------
------------------------------------------------------------
------------------------------------------------------------
------------------------------------------------------------
------------------------------------------------------------
------------------------------------------------------------
------------------------------------------------------------
------------------------------------------------------------
------------------------------------------------------------
------------------------------------------------------------
```


>FE136953_JP420962_FE081160_Penaeus_vannamei_PripL
ATGGGC--

---------------------------------AAGATC---AAGGAC------------

---ATG
AAGGAGTACATCGGCACGGGCGAGCCCATGAATGACCGCCGC---------GTGTGGAAG
GCTTTCCTGGCGGAGTTCCTGGGCACCATGTTCCTCGTGTTCATCGGCTGCGGCTCCTGC
ATCGGCTCCTGGAAAGAG--
---GGATACGCCCCCAGT
GTCGTCCAGATCTCACTCGCCTTCGGGGTCACCGTGGCCTCCATCGCGCAGGCTGTGGGT
CATGTCTCCGGCTGTCATATCAACCCAGCCGTGACATGCGCCATGCTGGTTGCCCGTCAC
GTCTCCGTCATCCGGGCACTGATCTACATCGTGTGCCAGTGCCTCGGTGCCATCGTCGGG
GCGGCCATCTTGAAGGGAGTGACCCCCGCAGACATCCAGGGG------------------
------------------------------TCCCTTGGCATGACCCTGAGGAACGAGAAG
---------ATCGACACCGCTCAGGCATTGGGCATCGAACTCCTCATCACCTTCGTCCTC
GTCATCACTGTCTTCGGG---GCCTGTGATGAGCGTAGGAAC------------------
---GACGTGAAG---------------GGATCAGCTCCTCTCGCCATTGGCCTGTCCATC
ACCACTTGCCATCTCTTCGCCGTGCCCATCACAGGGTCTTCGATGAACCCCGCAAGGAGT
TTCGGTCCAGCTGTCATCTCCGGT---------CTCTGGCAGGACCACTGGGTATACTGG
GCTGGGCCTATTCTCGGTGGTCTGGCAGCAGCTCTTATCTACTCCTACGTATTCCGAGCC
---CCAAAGGACCCC-------------------------------GCTGCTTAT------
------------------------GAC------------GTGGAGATG------GACAAC
TATAAC---------AAG------AGAACCAAC------AATGCT---------------

>AET34919_Macrobrachium_rosenbergii_PripL
ATGGGC--

---------------------------------AAGATA---AAGGAC------------

---ATG
CGCGATTACATCGGCACCAGCGAACTGAAG---GAGTACGGC------TCCCTCGGGAAG
GCCCTCCTGGCAGAATTCCTGGGCACCATGATCCTCGTGTTCGTCGGCTGCGGAAGCTGC
ATCGGCGGATGGACAGAC--
---GGTTACGCCCCCTCC

```
AAAGTGCAGATCGCCTTCGCCTTCGGAGTCACCGTGGCCACTGTGGCTCAGGCAATTGGA
CACATCTCTGGATGTCACATCAACCCAGCTGTCACTTGCGGCCTGCTGGTGGCTCGCTAT
GTGTCGGTGCTGCGCTCTCTCCTGTACATCATCGCTCAGTGCTTGGGAGCCTTGGTCGGG
GCCGCTATTCTTAAGGGCGTGACAGCAGAAAAAATCCAAGGC------------------
------------------------------AATCTCGGTATGACGACGGTTAATCCACAG
---------ATCAGCGCCGGGCAAGCCCTTGGAGTAGAACTCATCATCACCTTCGTGTTG
GTTTTAACTGTCTTCGGC---GTCTGCGACGAGAGGCGAAAT------------------
---GATGTTAAG---------------GGATCAGCTCCTCTCGCTATTGGACTCTCCATC
ACAACCTGCCATCTCATGGCTATTCCCATCACTGGTTCTTCAATGAATCCTGCTCGTACT
TTTGGTCCCGCTGTGATTTCAGGC---------GTCTGGCTGACCACTGGGTGTACTGG
GCTGGACCTATTCTGGGAGGAATCCTGGCAGCCATCATCTACTCCTACATCTTCAGAGCT
---CCCAAGACTCAG------------------------------CCTTATGAC------
------------------------GAC------------GTTCAAATG------GACAAT
TACAAC---------AAG------AGAGGAAAC------AACCCT---------------
------------------------------------------------------------
------------------------------------------------------------
------------------------------------------------------------
------------------------------------------------------------
------------------------------------------------------------
------------------------------------------------------------
------------------------------------------------------------
------------------------------------------------------------
------------------------------------------------------------
------------------------------------------------------------
------------------------------------------------------------
------------------------------------------------------------
------------------------------------------------------------
------------------------------------------------------------
------------------------------------------------------------
------------------------------------------------------------
------------------------------------------------------------
------------------------------------------------------------
------------------------------------------------------------
------------------------------------------------------------
------------------------------------------------------------
------------------------------------------------------------
------------------------------------------------------------
------------------------------------------------------------
------------------------------------------------------------
------------------------------------------------------------
------------------------------------------------------------
------------------------------------------------------------
------------------------------------------------------------
------------------------------------------------------------
------------------------------------------------------------
------------------------------------------------------------
------------------------------------------------------------
------------------------------------------------------------
```


>KY508287_Amphibalanus_improvisus_PripL1
ATGGCC--

------------------------------------TGG---TCCACT------------

---CTG
GAGGAGGCTCTGGGCCTGAAGGAGATGGTG---CGGAACAGG------GACCTGTGGAAG
GCGCTGCTGGCAGAGTTCCTCGGCACCATGCTGCTCACGCTCATCGGCTGCTTCAGCACC
ATCGGC---TGGGCGGCCAACGACGACAAG------------------------------
--GACCCCTACACGCCGGAC
ATGACGCAGATCGCACTCGCGTTCGGCATCACCGTCGCCACGCTCGCTCAGAGTATCGGC
CACATCAGCGGGTGCCACATCAACCCGGCGGTGACGGCGGGCATGCTGGTGGCGCGCCAG
GTCAGCCTCATGAGGGCCTTCTTCTACATCATCGTCCAGTGCATCGGAGCCATCGTCGGC
TCGGCGCTGCTCAAGGCGCTGACTCCGGAGGACGTGCAGGGC------------------
------------------------------TCGCTCGGCATGACCACCGTCAACCCGAAG
---------CTGACGGCGTTCCAGGGCTTCGGCGTGGAGCTCTTCATCACGTTTGTGCTC
GTGTTCGTCGTGTTCGGC---GTCTGTGACGAGGGTCGTGAT------------------
---GACGTCAAG---------------GGCTCGGCGCCGCTCGCCATCGGTCTCTCCATC
ACCGGCTGCCACCTGGGCGCGATCAAGTACACCGGCTCGTCGATGAACCCGGCTCGTACG
TTCGGCCCGGCCGTCGTCACTGGC---------ATCTGGGACAACCACTGGGTGTACTGG
GCGGGTCCTCTGGCGGGCGGCATCATCGCTGGCCTGGTCTATACGCATGCGTTCCGCGCC
---CGCCACATG---------------------------------GCCGTCGCC------
------------------------GCC------------GTGGAACTGACC---GGACTG
ACCGAA---GTC---AAGCCCCAGCGCGGCAGC---------AAG---------------

>XM_043382025_Amphibalanus_amphitrite_PripL1
ATGGCC--

------------------------------------TGG---TCGACA------------

---GTC
GAGGAGGCCCTCGGCCTGAAGGAGATGGTC---CGTAACCGG------GACCTGTGGAAG
GCGCTGCTGGCTGAGTTCCTCGGTACGATGCTGCTGACGCTCATCGGCTGCTTCAGCACC
ATCGGC---TGGGCGGAGGGAGATGCCAAG------------------------------
--GATCCCTACATGCCGTCT
ATGGTGCAGATCGCGCTGGCGTTCGGTATCACCGTGGCTACACTGGCCCAGAGTATCGGT
CACATCAGCGGATGCCACATCAACCCGGCGGTGACGATGGGCATGCTGGTGGCGCGTCAG
GTCAGCCTCATGAGGGCCTTCTTCTACATTGTCGTCCAGTGCATCGGAGCCATCGTCGGC
TCGGCGCTGCTCAAGGCTCTGACCCCGGAGGACGTACAGGGC------------------
------------------------------TCTCTGGGAATGACCACCGTCAACCCCAAG
---------CTGACGGCGTTCCAAGGCTTCGGCGTCGAGCTGTTCATCACGTTCGTGCTC
GTGTTCGTCGTGTTCGGC---GTGTGTGACGAGGGCCGTGAT------------------
---GACGTCAAG---------------GGCTCGGCACCGCTGGCCATCGGTCTCTCCATC
ACCGGCTGCCACCTGGGAGCGATCAAGTACACCGGCTCGTCCATGAACCCGGCCCGCACA
TTCGGGCCGGCTGTGGTGACCGGC----------ATCTGGGCTAACCACTGGGTGTACTGG
GCGGGCCCTCTGGCCGGTGGCATCATCGCCGGCCTCGTCTATACGCATGCCTTCCGCGCC
---CGCCACATG---------------------------------GCTGTCGAG------
------------------------GCG------------GTCGAGTTG------------
ACCGAG---GTG---AAGCCACAGCGCGCCAGC---------AAA---------------

>GHBL01005519_Semibalanus_balanoides_PripL1
ATGGCC--

------------------------------------TGG---TCCACC------------

---ATC
GAGGAGGCCCTCGGCCTCAAGGAGATGGTC---CGCAACCGG------GACCTGTGGAAG

GCACTGCTGGCCGAGTTCCTGGGCACCATGCTGCTGACGCTGATCGGCTGCTTCAGCACC
ATCGGC---TGGGCGGCTGATGACGTCAGC------------------------------
--GACAGCTACTCGCCGTCT
ATGGTGCAGATCGCCCTGTCGTTCGGCATCACGGTGGCCACTCTGGCGCAGAGTATCGGT
CACATCAGTGGCTGCCACATCAACCCGGCGGTGACGATCGGTATGCTGGTGGCCCGTCAG
GTCAGCCTGATGAGAGCCTTCTTCTACATCCTCGTCCAGTGCATCGGAGCTATCGTCGGC
TCGGCGCTCCTAAAGGCGCTGACTCCGGAGGACGTGCAGGGA------------------
------------------------------TCGCTCGGCATGACCACCATCAACCCCAAG
---------CTGACGGCGTTCCAAGGCTTCGGCGTCGAGCTGTTCATCACGTTCGTGCTG
GTGTTCGTCGTGTTCGGC---GTGTGTGACGAAGGCCGTGAT------------------
---GACGTCAAG---------------GGCTCGGCGCCGCTCGCCATCGGTCTCTCCATC
ACGGGCTGCCATCTGGGCGCGATCAAGTACACCGGCTCGTCGATGAACCCGGCGCGCACG
TTCGGCCCGGCCGTCGTCACCGGT---------ATCTGGGCGAACCACTGGGTGTATTGG
ATGGGGCCGCTGGCGGGCGGTGTGATCGCCGGACTCGTCTATACGCATGCCTTCCGGGCT
---CACCATATG---------------------------------GCCGTGGAG------
------------------------GCC------------GTGGAACTG-------------
ACGGAG---GTC---AAGCCGCAGCGATCGAGC---------AAA---------------


```
------------------------------------------------------------
------------------------------------------------------------
------------------------------------------------------------
------------------------------------------------------------
------------------------------------------------------------
------------------------------------------------------------
------------------------------------------------------------
------------------------------------------------------------
------------------------------------------------------------
------------------------------------------------------
>XM_037229222_Pollicipes_pollicipes_PripL1
ATGGCC------------------------------------------------------
------------------------------------------------------------
---------------------------------CCGTGG---ACGAAC------------
------------------------------------------------------------
---------------------------------------------------------GTC
GAGGAGGCGCTTGGTCTCAAGGAGATGGTG---CGCAACCGC------GACCTGTGGAAG
GCGCTGCTGGCCGAGTTCATCGGCACGCTGCTGCTGACGCTCATCGGCTGCCTGAGCACG
ATCGGC---TGGGCCGAC------------------------------------------
------------------------------------------GACGGCTACAAGCCGTCC
ATGGTGCAGATCGCGCTGGCGTTCGGTGTCACCGTCGCCACACTGGCGCAGAGTATCGGC
CACGTGAGCGGGTGCCACATCAACCCGGCCGTCACCGCCGGCATGCTGGTGGCCCGTCAG
GTCAGCCTGATGCGGGCCGTCTTCTACGTCCTGGTCCAGTGCATCGGAGCCATCGTCGGC
TCAGCGCTCTTGAAGGCTCTCACCCCGGAGGAAGTGCAGGGA------------------
------------------------------TCGCTGGGCATGACGACCATCAACCCCAAG
---------CTGTCCGCCTTCCAAGGCTTCGGTGTCGAGCTCTTCATCACCTTCGTTCTG
GTCTTCGTCGTGTTCGGC---GTCTGCGACGAGAGCCGCGAT------------------
---GACGTCAAG---------------GGTTCGGCGCCGCTGGCCATCGGACTTTCCATC
ACCGCTTGCCACCTGGGCGCCATTAAATACACGGGCTCCTCGATGAACCCGGCTCGGACC
TTCGGCCCCGCCGTCGTCACCGGC---------ACCTGGGCCAACCACTGGGTCTACTGG
GTGGGACCGATTGTTGGTGGAATCCTGGCCGGGCTCGTCTATACGCATGCCTTCAGGGCA
---CGCCACATG---------------------------------AGCGTCGAG------
------------------------TCG------------GTCGAGCTG------------
ACCGAA---GTG---AAACCCCAGATGTCGAGC------AATAAG---------------
------------------------------------------------------------
------------------------------------------------------------
------------------------------------------------------------
------------------------------------------------------------
------------------------------------------------------------
------------------------------------------------------------
------------------------------------------------------------
------------------------------------------------------------
------------------------------------------------------------
------------------------------------------------------------
------------------------------------------------------------
------------------------------------------------------------
------------------------------------------------------------
------------------------------------------------------------
```

```
------------------------------------------------------------
------------------------------------------------------------
------------------------------------------------------------
------------------------------------------------------------
------------------------------------------------------------
------------------------------------------------------------
------------------------------------------------------------
------------------------------------------------------------
------------------------------------------------------------
------------------------------------------------------------
------------------------------------------------------------
------------------------------------------------------------
------------------------------------------------------------
------------------------------------------------------------
------------------------------------------------------------
------------------------------------------------------------
------------------------------------------------------------
------------------------------------------------------------
------------------------------------------------------------
------------------------------------------------------------
------------------------------------------------------------
------------------------------------------------------------
------------------------------------------------------------
------------------------------------------------------------
------------------------------------------------------------
------------------------------------------------------------
------------------------------------------------------
>GHFC01026169_Neolepas_marisindica_PripL1
ATGCCC------------------------------------------------------
------------------------------------------------------------
---------------------------------GGCTGG---ACCACC------------
------------------------------------------------------------
---------------------------------------------------------GTG
CAGGAGGGGCTCGGCTTCAAGGAGATGGTG---CGCAACCGC------GACCTGTGGAAG
GCGCTGCTGGCCGAGTTCCTGGGCACGCTGCTGCTGACGCTCATCGGCTGCTTCAGCACC
ATCGGCGGCTGGGACGAC------------------------------------------
------------------------------------------GAC---TACAAGCCGTCC
GTGGTGCAGATCGCCCTGGCGTTCGGCATCACTGTGGCCACCCTGGCTCAGAGCATTGGC
CACATCAGCGGGTGCCACATCAACCCGGCTATCACCGTCGGCATGCTGATCGCCCGCCAA
GTCAGCCTGATGAGGGCCGTCTTCTACATCCTCGTCCAGTGCATCGGCGCCATCGTCGGC
TCGGCACTGCTCAAGGTGCTGACCCCGGAGGACGTGCAGGGC------------------
------------------------------TCGCTGGGCATGACCACCGTCAGCGGCAAG
---------CTGTCGCCGTTCCAGGGCTTCGGTGTCGAGCTGTTCATCACGTTCGTGCTG
GTGTTCGTCGTGTTCGGC---GTGTGCGACGAGGCGCGCACC------------------
---GACGTCACC---------------GGCTCGGCTCCGCTCGCCATCGGCCTCTCCATC
ACCGCCTGCCACCTGGGCGCCATCAAGTACACCGGCTCCTCGATGAACCCGGCGCGCACG
TTCGGACCTGCTGTGGTGACCGGC---------AACTGGGCGAACCACTGGGTGTACTGG
GCGGGCCCGCTTCTGGGCGGCACCATCGCCAGTCTGGTCTACACACACGCCTTCAGGGCA
```

```
---CGCAACATG---------------------------------GCCGTCGAC------
------------------------GCA------------GTTGAGCTG------------
ACCGAG---GTC---AAGCCGCACAGGTCGAAC------AATAAG---------------
------------------------------------------------------------
------------------------------------------------------------
------------------------------------------------------------
------------------------------------------------------------
------------------------------------------------------------
------------------------------------------------------------
------------------------------------------------------------
------------------------------------------------------------
------------------------------------------------------------
------------------------------------------------------------
------------------------------------------------------------
------------------------------------------------------------
------------------------------------------------------------
------------------------------------------------------------
------------------------------------------------------------
------------------------------------------------------------
------------------------------------------------------------
------------------------------------------------------------
------------------------------------------------------------
------------------------------------------------------------
------------------------------------------------------------
------------------------------------------------------------
------------------------------------------------------------
------------------------------------------------------------
------------------------------------------------------------
------------------------------------------------------------
------------------------------------------------------------
------------------------------------------------------------
------------------------------------------------------------
------------------------------------------------------------
------------------------------------------------------------
------------------------------------------------------------
------------------------------------------------------------
------------------------------------------------------------
------------------------------------------------------------
------------------------------------------------------------
------------------------------------------------------------
------------------------------------------------------------
------------------------------------------------------------
------------------------------------------------------
>KY508284_Amphibalanus_improvisus_PripL2
ATGCCT------------------------------------------------------
------------------------------------------------------------
---------------------------------GCTTGG---AGTACC------------
```

```
------------------------------------------------------------
---------------------------------------------------------GCC
CGTGAAGGACTTGGCCTGCAGGAGGTCGTC---AACAATAAG------GACCTGTGGAAG
ACTCTGCTGGCCGAGTTCGTGGGGACGCTGTTTCTGGTGTTCATCGGATGTCTGACGTGC
ATCGGA---TGGACGGAT------------------------------------------
------------------------------------------GAGGGATACGCTCCGTCC
GTAGTTCAGATCGCCCTCGGATTCGGCATCACCGTGGCCACGATGGCACAGAGTATCGGT
CACATCAGCGGGTGCCACATCAACCCCGCGGTGACGGTGGCGATGATTGTGACGCGCAAA
ATCCCGCTGTTCCGTGCGCTGTGCTACATCGTCATGCAGTGTATGGGAGCTGCTGCCGGC
TCGGCGCTACTCAAGGCGCTGACACCGGAGGACATCCACGGC------------------
------------------------------ACCCTGGGGATGACTCAGCTGAACCCGAAA
---------CTGACCGCAGCTCAGGGGTTCGGCGTGGAGGCCCTCATCACGTTCGTCCTC
GTGCTGGTTGTGTTTGGA---GTGTGTGACGAGAGGAGGGAG------------------
---GACGTGCTC---------------GGATCGGGACCGCTCGCCATTGGACTCTCCGTC
ACCACCTGCCACGTGGGCGCGATCAAGTACACAGGAGCCTCCATGAACCCGGCTCGGTCA
TTCGGACCCGCCCTCGTCACCGGG---------CTCTGGGACAACCACTGGGTGTACTGG
GCGGGTCCGGTGTTTGGTGGCCTCCTCGCCGGTGGTCTGTACGCCACGGCCTTCCGCGCC
---GGCCGGCTG---------------------------------CACTCGGCC------
------------------------TCAGAC---------TCGGAC------------AGA
GAGGAC---ACC---AAGATGGAGAAATACGAG------CCC------------------
------------------------------------------------------------
------------------------------------------------------------
------------------------------------------------------------
------------------------------------------------------------
------------------------------------------------------------
------------------------------------------------------------
------------------------------------------------------------
------------------------------------------------------------
------------------------------------------------------------
------------------------------------------------------------
------------------------------------------------------------
------------------------------------------------------------
------------------------------------------------------------
------------------------------------------------------------
------------------------------------------------------------
------------------------------------------------------------
------------------------------------------------------------
------------------------------------------------------------
------------------------------------------------------------
------------------------------------------------------------
------------------------------------------------------------
------------------------------------------------------------
------------------------------------------------------------
------------------------------------------------------------
------------------------------------------------------------
------------------------------------------------------------
------------------------------------------------------------
------------------------------------------------------------
```


>XP_043197127_Amphibalanus_amphitrite_PripL2
ATGCCC--

---------------------------------GCCTGG---AGTACC------------

---GCG
CGTGAGGCTCTGGGCCTACAGGAGGTCATC---GCCAACAAG------GACCTGTGGAAG
GCGCTGCTGGCAGAGTTCGTGGGCACGCTGTTCCTGGTGTTCCTTGGCTGTCTGACTACC
ATCGGC---TGGCAGGAC--
--GAGGGCTACGCGCCGTCC
ATGGTGCAGATCGCCATCGGCTTCGGCATCACAGTGGCCACCATGGCACAGAGTATCGGT
CACATCAGCGGGTGTCACATCAACCCTGCCGTGACGGTGGCCATGTTTGTGACGCGTAAC
ATCCCGCTGTTCCGTGCCGTCTGCTACATCGTGATGCAGTGCCTGGGAGGCACCGCCGGA
TCGGCGCTGCTGAAGGCGCTGACTCCGGAGGGCATCCAGGGC------------------
------------------------------TCTCTGGGCATGACCCAGGTGCACCCGGAG
---------CTGACGGCCGCGCAGGGGTTCGGCATCGAGGTGCTCATCACCTTCGTCCTG
GTGTTCGTCGTGTTCGGT---GTCTGTGACGAGAAGAGGACA------------------
---GACGTGCTC---------------GGCGCCGGGCCGCTGGCCATCGGACTGGCCGTC
ACCACGTGCCACGTGGGAGCGCTGAAGTGCACCGGTGCCTCGATGAACCCGGCGAGGTCG
TTTGGACCTGCCGTTGTCACCGGA---------ATCTGGGCTAACCACTGGGTGTTCTGG
GCGGGCCCGCTGCTGGGGGGCCTCCTGGCCGCCGTTCTGTACTCGGTGGCGTTCCGCGCC
---GACCGTCTG---------------------------------GGCTCTCCT------
------------------------CGC------------TCGACA------------GGG
GACGAG---AGC---AAGCCGGAGAAATACGAG------CCC------------------

>GHBL01005893_01003293_Semibalanus_balanoides_PripL2
ATGCCC--

---------------------------------GCGTGG---ACCACG------------

---GCT
CGCGAGGGCCTCGGCCTGCAGGAGGTCATC---ACCGACAAG------GACCTGTGGAAG
GCGCTGGTGGCCGAGTTCATCGGTACGCTGTTCCTGGTGTTCCTCGGCTGCCTTACGACC
ATCGGC---TGGCCGGAC--
--GAGGGCTACCTGCCCTCC
ATGGTGCAGATCTCCATCGGCTTCGGCATCACCGTCGCGACGATGGCTCAGACTATTGGC
CACATCAGCGGCTGCCACATCAACCCTGCCATCACGATCGCGATGCTGGTCACGCGTAAC
ATCCCGCTGTTCAGAGCCGTCGCCTACATCATCATGCAGTGCCTGGGCGGCACGGCAGGC
TCGGCGCTCCTCAAGGCTCTGACGCCGGAGGGCATCCAGGGC------------------
------------------------------TCGCTGGGCATGACACAGGTGAACCCGGCG
---------CTGACGCCGGCGCAGGGCTTC---------------------------CTG
GTGTTCGTCGTGTTCGGC---GTCTGTGACGAGCAGCGCACC------------------
---GACGTTCTC---------------GGCTCGGCGCCGCTGGCTATCGGACTCTCCATC

ACCACCTGTCACGTCGGAGCGCTCAAGTGCACCGGAGCCTCGATGAACCCGGCCCGCTCC
TTCGGCCCCGCCGTCGTCACCGGC---------ATCTGGGCCAACCACTGGGTGTTCTGG
GCGGGACCTGTGCTGGGGGGCCTGCTGGCCGGACTGATGTACACACTGGCGTTCCGCGCC
---GAACGGCTC---------------------------------GGGGGAGCA------
------------------------GGC------------GTTGTCCAG---------GGC
GAGGAG---GTC---AGACCGGACAAATACGAGGCCAGACCGGAG---------AAATAC
--GAGCCC------

>XP_037068075_Pollicipes_pollicipes_PripL2

ATGCCC--

---------------------------------GTCTGG---AACACC------------

---GCT
CGCGAGGGCCTCGGCCTGCAGGAGGTCACC---CGCGGCTGC------GACCTGTGGAAG
GCGCTGCTGGCCGAGTTCATTGGCACGCTGTTCCTTGTGTTCCTGGGCTGCCTGAGCACC
ATCCCG---TGGCCCGAC--
--GAAGGCTACCAGGCGTCC
ATGGTGCAGATCGCCATCGCGTTCGGCATCACCGTCGCCACCATGGCGCAGTCGATCGGC
CATATCAGCGGCTGCCACATCAACCCGGCCATCACGCTGGCCATGCTGGTGACGCGTCAC
ATCACCCTGATCAGGGCCGTCTGCTACATCATTGTCCAGTGCCTGGGCGGAGTGGTCGGC
TCCGCGCTACTCAAGGCGGTGATTCCGGTTATCTACCAGGGT------------------
------------------------------AGCCTGGGCATGACCATGGTGAACCCAGCC
---------ATGACGCCGGCGCAGGGCTTCGGCGTCGAGGTGCTCATCACCTTCGTCCTG
GTGTTCGTCGTGTTCGGC---GTCTGCGACGAGCAGCGCACT------------------
---GACGTCATG---------------GGCTCGGCGCCGCTGGCCATCGGCCTCTCCATC
ACCACGTGCCACGTGGGAGCTCTGAAATGTACGGGCGCTTCGATGAACCCGGCACGCTCC
TTCGGACCCGCTGTCGTCACTGGT---------ATCTGGGCCAACCACTGGGTGTTCTGG
GTGGGCCCGGTGCTTGGAGGCCTGCTGGCTGCCCTGATATACACAGGTGCCTTCCGAGCC
---GGCCATTTGACC-------------------------------CGGGCACCG------
------------------------GGT------------GACGAGGTC------------
---GAG---GTC---AAACCGGCCTCCCGCGGC------CCC------------------


```
------------------------------------------------------------
------------------------------------------------------------
------------------------------------------------------------
------------------------------------------------------------
------------------------------------------------------------
------------------------------------------------------------
------------------------------------------------------------
------------------------------------------------------------
------------------------------------------------------------
------------------------------------------------------------
------------------------------------------------------------
------------------------------------------------------------
------------------------------------------------------------
------------------------------------------------------------
------------------------------------------------------------
------------------------------------------------------
>GHFC01030481_Neolepas_marisindica_PripL2
ATG---------------------------------------------------------
------------------------------------------------------------
---------------------------------GTGTGG---TCGTCG------------
------------------------------------------------------------
---------------------------------------------------------GCT
CGTGAGGGGCTCGGCCTGCAGGAGGTCATC---GTCAGCAAG------GACCTGTGGAAG
GCGCTGGTGGCCGAGTTCATCGGCACGCTGTTCCTGGTGTTCCTCGGCTGCCTGAGCACC
ATCGCC---TGGCCGGAG------------------------------------------
------------------------------------------GAGGGCTACATGCCATCC
ATGGTGCAGGAGGCCATCGCCTTCGGCATCACCGTGGCCACCATGGCGCAGGCTATCGGC
CACATCAGCGGGTGTCACATCAACCCGGCTATCACGATCGCAATGCTGGTGACGCGTCAC
GTGACACTGACGCGCGCATTCTTCTACGTCATCGTCCAGTGTCTGGGCGGTATCGTGGGC
TCTGCTCTGCTCAAGGCCGTGATCCCAGTCGCCCACCAGGGC------------------
------------------------------ACCCTCGGCATGACGATGGTGCACCCGGAC
---------ATGACTCCCGCCCAGGGGTTCGGCGTCGAGGTGCTCATCACATTCGTGCTG
GTCTTCGTCGTGTTCGGC---GTTTGCGACGAGAAGCGCACG------------------
---GACATCATG---------------GGCTCGGCACCGCTGGCCATCGGCTTCTCCATC
ACCACCTGTCACGTCGGAGCGCTGAAGTGCACCGGAGCTTCGATGAACCCGGCGCGCTCC
TTCGGACCTGCAGTGGTCACCGGC---------ATATGGGCTAACCACTGGGTGTTCTGG
GGTGGTCCGGTCCTGGGAGGCCTGTTGGCAGCTCTGATTTACACCTTCGCCTTCCGCGCC
---AGTCACCTGTCG------------------------------GGCGTCGCC------
---------------------AGCGGA---------------AATGCC------------
---GAG---GTC---AAACCCGACAAAAGCGACCGCAGCCCC------------------
------------------------------------------------------------
------------------------------------------------------------
------------------------------------------------------------
------------------------------------------------------------
------------------------------------------------------------
------------------------------------------------------------
------------------------------------------------------------
------------------------------------------------------------
```


>Q25074_Haematobia_irritans_exigua_Drip
ATGGTG--

---------------------------------GAGAAA---CTAGAT------------

---ATG
TCAGCTGTAGTAGGTGTCAAGGATATCACA---GATAATAAG------AAAATATGGCGA
CAGTTAATGGCCGAACTCATCGGTACCTTCTTCTTGGTTGTCATTGGCGTTGGTAGCTGC
ACTGGT---GGTTCGGAA--
--TGGAGTCCCTCT
ATACCTCAAATAGCCTTCACATTTGGTTTAACAGTAGCCACATTGGCCCAGGCTATTGGA
CACATTAGCGGTTGTCACATTAATCCAGCCGTGACCGTTGGCTTCCTGATTGTGGGTGAG
ATGAGTATAATTAAAAGTGTCCTGTATATTGCTGTACAATGTGTTGGAGCTATTGCTGGG
GCCGCAGTCATTAAGGTTGGAGTTTCTGAAGCCGTTTCAGGT------------------
---------------------------CTAGATTTGGGTGTTTCCAGCTTTTCCAGCACT

```
---------TTAACAGTGGGTCAAGCTGTATTGATAGAAGCTTTGATTACCTTCATTTTG
GTAGTTGTTGTTAAAGGA---GTCTCCGATCCTGGTCGCACT------------------
---GACATTAAA---------------GGATCTGCACCTTTAGCCGTGGGTCTTTCTATT
GCTGCTGGACACTTGTGTGCTATTAAATTAACTGGTGCTAGTATGAATCCAGCCCGTTCA
TTCGGTCCTGCTGTGGTACAAAAT---------ATGTGGATCGATCACTGGGTTTATTGG
GTAGGACCAATTGTTGGTGCCATTGTCGCAGCACTGTTGTACAAATTCGTCTTCAAAGTA
---CGCAAGGGA------------------------GATGATGAG---------------
------------------------GCC------------AACTCTTAT------GACTTC
------------------------------------------------------------
------------------------------------------------------------
------------------------------------------------------------
------------------------------------------------------------
------------------------------------------------------------
------------------------------------------------------------
------------------------------------------------------------
------------------------------------------------------------
------------------------------------------------------------
------------------------------------------------------------
------------------------------------------------------------
------------------------------------------------------------
------------------------------------------------------------
------------------------------------------------------------
------------------------------------------------------------
------------------------------------------------------------
------------------------------------------------------------
------------------------------------------------------------
------------------------------------------------------------
------------------------------------------------------------
------------------------------------------------------------
------------------------------------------------------------
------------------------------------------------------------
------------------------------------------------------------
------------------------------------------------------------
------------------------------------------------------------
------------------------------------------------------------
------------------------------------------------------------
------------------------------------------------------------
------------------------------------------------------------
------------------------------------------------------------
------------------------------------------------------------
------------------------------------------------------------
------------------------------------------------------------
------------------------------------------------------------
------------------------------------------------------------
------------------------------------------------------------
------------------------------------------------------------
------------------------------------------------------------
------------------------------------------------------------
```


>BAM26200_Phormia_regina_Drip
ATGGTG--

---------------------------------GAAAAA---GTCGAT------------

---ATG
TCAGCTATGGTTGGTGTTAAGGACATCACA---GATAATAAA------AAGATCTGGCGT
CAATTGTTGGCTGAATTGATTGGTACATTCTTTTTGGTTGTAATTGGTGTTGGCAGTTGT
ACAGGT---GGTGCAGAT--
--TGGACACCATCA
ATACCACAAATAGCCTTTACTTTTGGTTTAACTGTGGCCACATTGGCTCAGGCTATTGGA
CATATTTCTGGTTGTCATATTAATCCAGCTGTTACCCTTGGCTTCCTTATTGTTGGCGAA
ATGAGCATAATTAAAAGTGCTCTCTATATAGCTGTACAATGTGTGGGTGCTATTGCTGGA
GCCGCTGTTATTAAGGTTGGTGTCTCTGAAGCAGTGGGTGGT------------------
---------------------------AATGAATTGGGTGTTTCAGCCTATGCAGCTTCT
---------CTTACAGTGGGTCAAGCTGTACTCATCGAAGCTTTAATTACTTTCATTTTG
GTATTTGTTGTTAAAGGT---GTGTCCGATCCTGGTCGCACA------------------
---GATATCAAG---------------GGCTCAGCTCCTTTAGCTGTAGGTTTGTCCATA
GTCGCTGGTCATTTATGTGCTATCAAATTAACTGGTGCTAGCATGAATCCAGCTCGTTCT
TTTGGTCCCGCTGTTGTACAAAAT---------ATGTGGGAAGATCATTGGGTCTATTGG
GTGGGTCCTTTTGCCGGTGCTGCTGTAGCTGCTTTACTTTATAAATTTGTTTTTAAAGTA
---CGCAAAGGT------------------------GATGATGAG---------------
------------------------GCT------------AATTCTTAT------GATTTT

>ADD19102_Glossina_morsitans_morsitans_Drip1
ATGGTA--

---------------------------------GAGAAA---TTAGAT------------

---ATG
GGCGACATAGTCGGTGTAAAGGATTTGAAA---GAAAATAAA------CATCTCTGGAAG
GAGTTATTGGCAGAATTTGTCGGCACATTTGTTCTACTTTTTCTGGGCTTATTCAGTTGC
CTTGGA---GGA--
---TCG
GTGGAACGAATAGCATTCGCCTTCGGTCTAGCCGTGGCTTCAATGGCTCAAGCCGTAGGA
CATATCAGTGGTTGTCATATTAATCCGGCAATTACTTTGGGATTTCTAGTCGTTGGAGAA
ATAAGCATTTTAAAGGGTTTATTCTTTATAATAATGCAATGTCTCGGTGCGGTGGCCGGT
GCCGGTGTAGTTTATTTATCATTAATAGATACTCTAATGGGC------------------
---------------------------GCTAATTTAGGTATAACATCACCGGTGGCAAAC
---------CTACATGTTGGTCAAGCCATACTTATTGAAGCCTTGATAACGTTTGTATTG
GTACTTGTCGTGAAAGCT---GTTTCGGACGTAGAACGTATC------------------
---GATATTAAA---------------GGTTCCGCTCCATTGGCTGTTGGACTTTCGATA
ACAGCTGGACATATGTGCGCCGTTCCTCTAACCGGTGCCAGTATGAATCCCGCTCGCAGT
TTCGGACCGGCCGTAGTGCAAAAT---------TCATGGGATAGTCATTGGATTTACTGG
GTTGGCCCCAATATAGGAGGTATCTTGGCTGGACTTCTCTATCGTTTATGCTTTAAACAA
---AGCAGTGCA------------------------------GAA---------------
------------------------AGCATT------------------------------


```
------------------------------------------------------------
------------------------------------------------------------
------------------------------------------------------------
------------------------------------------------------------
------------------------------------------------------------
------------------------------------------------------------
------------------------------------------------------------
------------------------------------------------------------
------------------------------------------------------------
------------------------------------------------------------
------------------------------------------------------------
------------------------------------------------------------
------------------------------------------------------------
------------------------------------------------------------
------------------------------------------------------------
------------------------------------------------------------
------------------------------------------------------------
------------------------------------------------------------
------------------------------------------------------------
------------------------------------------------------------
------------------------------------------------------------
------------------------------------------------------------
------------------------------------------------------------
------------------------------------------------------------
------------------------------------------------------------
------------------------------------------------------------
------------------------------------------------------------
------------------------------------------------------------
------------------------------------------------------------
------------------------------------------------------------
------------------------------------------------------------
------------------------------------------------------------
------------------------------------------------------------
------------------------------------------------------------
------------------------------------------------------------
------------------------------------------------------
>ADD20051_Glossina_morsitans_morsitans_Drip2
ATG---------------------------------------------------------
------------------------------------------------------------
------------------------------------AAA---GCAGAC------------
------------------------------------------------------------
---------------------------------------------------------ATG
TCAAAATTTATAGGAATTTCCGAATTTACG---CAGAATAGG------AAAATATGGCGA
ATGCTATTTGCTGAGCTTGCGGGAACATTCTTACTGGTTATCATTGGTATAGGCAGTTGC
ACGAGT---GGTGCGGAC------------------------------------------
------------------------------------------------TGGTCCCCCAGC
GTGCCTCAAATAGCATTCACATTTGGTTTAACTGTGGCAACGCTTGCACAGACTATTGGT
CATATAAGTGGATGTCACATAAATCCTGCTGTTACAGTCGGTTTCTTGATAGTAGGCGAG
```

ATGACTTTACTAAAAGCTATTTTTTACATAATAGTACAGTGCGTCGGAGCAATGGCTGGA
TCGGCGGTTTTGAGTCTAGCTATCCCTGACACACTTGGCGGT------------------
---------------------------AATGGCTTGGGCGTGTCTAACTTTTCC---TCG
---------CTCAGTGCCGGACAAGCGGTGTCAATTGAAGCCTTCATTACAGCTATCTTA
GTGCTGGTAGTGAAAGCC---GTGTCAGATTCAAAACGACAA------------------
---GATATAACG---------------GGATCAGCCCCATTGGCCGTAGGCCTGGCTATC
GCTACGGGTCATCTTTGTGCGATTAAATTAACTGGAGCCAGCATGAACCCTGCGCGCTCA
TTCGGACCGGCCGTAGTTCACGAT---------GTTTGGCAAAATCATTGGGTTTATTGG
ATTGGTCCCCTCGTTGGCAGCGTGGTAGCTGCCGTTATCTATAAATTAATCTTCAAGCAA
---TCTAAAGAA-------------------------GATGATGAC---------------
------------------------ACG------------AACTCCTAC------GATTTT

>FBpp0087240_Drosophila_melanogaster_Drip
ATGGTC--

---------------------------------GAGAAA---ACAGAA------------

---ATG
TCGAAATTCGTTGGCGTTGCCGACATAACC---GAGAACAAG------AAAATCTGGCGC
ATGCTGCTCGGCGAACTGGTGGGCACATTTTTCTTGATCTTCGTCGGCGTTGGCAGCACG
ACGAGC---GGA--
---AGT
GTACCACAAATCGCATTCACCTTTGGCTTGACGGTGGCCACCATCGCCCAGGGCTTGGGC
CACCTCAGTGGCTGTCACATTAATCCGGCGGTCACCCTTGGATTCCTGATCGTTGGTGAG
ATCAGCATTCTGAAGGCTGCCTTCTACATCATCGTCCAATGCGTGGGCGCCATTGCTGGA
GCGGCTGTGATAAAGGTGGCACTCGATGGAGTGGCTGGTGGC------------------
------------------------------GACCTGGGAGTATCCTCCTTTGATCCCTCC
----------CTGAACTGTGCACAGGCGGTGCTGATCGAAGCGCTGATCACCTTTATTTG
GTTTTCGTGGTCAAGGCT---GTTTCGGATCCTGGACGCCAG------------------
---GATATCAAG---------------GGATCAGCGCCACTGGCTGTTGGTTTGGCCATC
GCCGCTGGCCATTTGTGTGCAATCAAACTGAGCGGTGCCAGCATGAATCCCGCCCGGTCC
TTTGGTCCCGCCGTAGTGCAGGGC---------GTCTGGACCTATCACTGGGTTTACTGG
GTGGGTCCCATTGCCGGCGGCCTGTTGGCCGGAATCATCTACAGATTAATCTTCAAGGTA
---CGCAAGGGC------------------------GATGATGAG---------------
------------------------ACC------------GACTCGTAC------GACTTC

>FBpp0154409_Drosophila_grimshawi_Drip
ATGGTT--

---------------------------------GAGAAA---ACAGCC------------

---ATG
ACGAATTTCGTTGGCGTCGCCGACATCACG---GAGAACAAG------AAAATCTGGCGC
ATGCTGCTCGGCGAGTTGGTGGGCACATTTTTCTTGGTGTTCATTGGCGTTGGCAGCACT
GCCACC---GGAAGT---
--GCCACA
GTGCCACAGATAGCGTTCACCTTTGGCTTGACGGTGGCAACGCTGGCCCAGGGACTGGGT
CACATCAGTGGCTGTCACATCAATCCGGCTGTGACGATCGGTTTCCTTATCGTTGGCGAG
ATGAGCATCCTGAAGGCGGCCTTCTACATAATCGTGCAATGTGTGGGCGCCATTGCTGGG
GCAGCTGTCATTCGTGCTGCGCTCAGTGGCATGACTATCACC------------------
------------------------------TCATTGGGCGTCTCATCATATGATGCCTCG
---------CTCCAAGTGGGTCAGGTGGTGCTGATCGAGGCACTGATCACCTTTATTCTG
GTGTTTGTCGTGAAGGCC---GTGTCGGATGCAGGTCGTATG------------------
---GATATCAAG---------------GGATCGGCTCCGTTGGCCGTGGGCCTCTCAATT
GCAGCTGGACATTTGTGTGCGATTCACCTCACTGGTGCCAGCATGAATCCGGCTCGCTCC
TTTGGCCCCGCCGTTGTCCAGGGC---------ATATGGACAGATCATTGGGTCTATTGG
GTGGGTCCCATTGCTGGCGGTATTGTGGCCGCCCTCATCTACAGATTCATCTTTAAGGTG
---CGCAAGGGC------------------------GATGATGAG---------------
------------------------ACC------------AGCTCGTAT------GATTTC

>AGAP008842_Anopheles_gambiae_Drip
ATGACT--

---------------------------------GAAAGC---GCAGGA------------

---GTG
AAACAGATCGTCGGCGTCTCCGATATCACC---GAGAACCGC------AACATATGGCGC
ATGCTGGTGGCCGAGTTTCTCGGCACCTTCTTTCTGGTGGCGATCGGCATCGGTAGCACG
ACCGGC---TGGACCGAC--

```
------------------------------------------------TACAGTCCCACG
CTCACGCAGATTGCGTTCACCTTCGGCCTGGTGGTCGCCACGCTTGCACAGGCATTTGGG
CACGTGAGCGGATGCCACATCAATCCGGCCGTCACGATCGGGCTGATAGTAACGGCCGAC
GTCAGCATACTGAAGGGTGCTTTCTACATCGTTTCCCAGTGCATCGGCGCAATTGCGGGT
GCAGCCGTCATTAAGGCCGCCACACCGTCGGAAGTGGTCGGC------------------
------------------------------GGGCTCGGCGTGACCGGCATCGCCCCGGGA
---------CTGTCCACCGGGCAGGGTGTCCTGATCGAAGCGCTCATCACCTTCATGCTG
GTGTTCGTCGTCCATGGC---GTGTGCGATAATCGGCGCACG------------------
---GACGTCAAG---------------GGTTCGGCTCCGCTCGCTATCGGACTCTCGATC
ACGGCCGGCCATTTGGCTGCGATCAAGTACACCGGCGCCAGCATGAACCCGGCCCGCTCG
TTCGGACCGGCCGTTGTGATGGGT---------AATTACACCGATCTGTGGGTATATTGG
GTTGGACCGATTGTCGGTGGCATTGTTGCCGGTGCCGTCTATCGTCTGTTCTTCAAAGTA
---AGAAAAGGT------------------------GACGAAGA----------------
------------------------TCA------------AATTCATAC------GATTTT
------------------------------------------------------------
------------------------------------------------------------
------------------------------------------------------------
------------------------------------------------------------
------------------------------------------------------------
------------------------------------------------------------
------------------------------------------------------------
------------------------------------------------------------
------------------------------------------------------------
------------------------------------------------------------
------------------------------------------------------------
------------------------------------------------------------
------------------------------------------------------------
------------------------------------------------------------
------------------------------------------------------------
------------------------------------------------------------
------------------------------------------------------------
------------------------------------------------------------
------------------------------------------------------------
------------------------------------------------------------
------------------------------------------------------------
------------------------------------------------------------
------------------------------------------------------------
------------------------------------------------------------
------------------------------------------------------------
------------------------------------------------------------
------------------------------------------------------------
------------------------------------------------------------
------------------------------------------------------------
------------------------------------------------------------
------------------------------------------------------------
------------------------------------------------------------
------------------------------------------------------------
------------------------------------------------------------
```


>EZ976133_Anopheles_funestus_Drip
ATGACT--

---------------------------------GAAAGT---GCAGGA------------

---GTG
AAACAGATCGTCGGCGTCTCCGATATCACT---GAGAATCGC------AACATATGGCGC
ATGCTGATTGCCGAGTTTCTCGGTACCTTCTTTTTGGTCGCGATTGGCATCGGTAGCACG
ACGGGC---TGGACCGAC--
--TACAATCCCACG
CTTACGCAGATTGCATTCACCTTCGGCCTGGTGGTCGCGACGCTAGCACAGGCTTTTGGA
CACGTGAGCGGATGCCACATCAATCCCGCCGTTACAATTGGGTTGATGGTAACTGCCGAC
GTCAGCATACTGAAAGGTGCTTTCTACATTGTTTCACAGTGTATCGGTGCAATTGCAGGA
GCAGCCGTTATTAAGGCCGCCACACCGTCGGAAGTTGTTGGA------------------
------------------------------GGACTCGGTGTGACCGGTATAGCCCCAGGA
---------CTGTCCACTGGGCAGGGTGTCCTGATAGAAGCGCTCATCACCTTCATGCTG
GTGTTCGTCGTCCATGGC---GTGTGCGATAATCGGCGCACG------------------
---GACATTAAG---------------GGTTCGGCGCCGCTTGCTATCGGGCTCTCAATC
ACGGCCGGCCATCTGGCAGCGATTAAGTACACTGGCGCCAGCATGAACCCAGCACGTTCA
TTTGGACCGGCCGTTGTAATGGGT---------AATTACACGGATCTGTGGGTGTATTGG
GTCGGTCCGATTGTCGGTGGCATCGTCGCTGGTGCCGTCTACCGTCTGTTCTTCAAAGTA
---AGAAAAGGT------------------------GACGAGGAA---------------
------------------------TCA------------AATTCATAC------GATTTT


```
------------------------------------------------------------
------------------------------------------------------------
------------------------------------------------------------
------------------------------------------------------------
------------------------------------------------------------
------------------------------------------------------------
------------------------------------------------------------
------------------------------------------------------------
------------------------------------------------------------
------------------------------------------------------------
------------------------------------------------------------
------------------------------------------------------------
------------------------------------------------------------
------------------------------------------------------------
------------------------------------------------------------
------------------------------------------------------------
------------------------------------------------------------
------------------------------------------------------------
------------------------------------------------------------
------------------------------------------------------------
------------------------------------------------------------
------------------------------------------------------------
------------------------------------------------------------
------------------------------------------------------------
------------------------------------------------------
>GAAF01000345_Macrosiphum_euphorbiae_Drip
ATGCCT------------------------------------------------------
------------------------------------------------------------
---------------------------------AGATCA---AAAGTT------------
------------------------------------------------------------
---------------------------------------------------------GTA
CATGGTATGATAGCAATGGACGACGTATCT---GACAGTAAC------AAAATATGGAGA
ATGCTGTTTGCCGAATTTTTGGGCACAGCTATTCTATTATTTCTTGGTTGTGGCAGCATA
ATGTGG---CTTAACGGTTCAACT------------------------------------
------------------------------------------------AATTCATCAGAT
ATCCTAGCAATCTCTCTGACTTTTGGTTTCACAATAGCAACACTAGTACAGATATTCGGT
CAGACCAGTGGATGTCATATCAACCCAGCAGTCACGGTTAGCTTTTTGGTGAGCGGCCAA
TGCTCATTTTTGAAATCTGCTCTGTACATAGTTGCACAGTGTTTGGGGGCTATAGCCGGT
ATCTACCTTCTAGAATTTGTTACTCCAGACGCAGTAACTAAA------------------
------------------------------GGTTTAGGAAAGACAGACATAAACACACTG
---------CTTCAACCGGGACAAGGATTCGTCGTAGAAGCATTCATTACCTTTATTCTA
GTGTTGGTTATTCACTCG---GTCTGTGATGAAGCCAACCGA------------------
---AGCAACATA---------------GTTACGCCGTCCATTTCCATAGGTCTGACCATT
GCTGCCGCCCACCTAGCAGCGATTAAATACACAGGAGCTAGCATGAATCCAGCAAGATCT
TTGGGCCCAGCAGTTGCTCTAGGT---------GCATGGTCAAACCATTGGGTTTACTGG
GTCGGTCCTATTGTCGGTGGCATACTTGGAGGAACCGTCCACACTTTTGTGTTGAAA---
---CGTCATACT---------------------------GAAGAG---------------
------------------------ACA------------TCAAAC---------------
```


>AJL33751_Rhopalosiphum_padi_Drip
ATGCCT--

---------------------------------AGATCA---AAAGTT------------

---GTA

CATGGTATGATAGCAATGGACGACGTATCT---GACAATAAC------AAAATATGGAGA
ATGCTGTTTGCCGAATTTTTGGGCACGGCTATTCTATTGTTTCTTGGCTGTGGCAGCATT
ATGTGG---CTTGACGGATTA---------------------------------------
--GGTTCATCGGCT
ATCATACAAATCTCTCTGACTTTTGGTTTCACAATAGCAACACTAGTACAGATATTCGGT
CAGACCAGTGGATGTCATATCAACCCAGCAGTCACGGTTAGCTTTTTGGTGAGCGGCCAA
TGCTCATTTTTGAAATCAGCTCTGTACATAGTTGCACAGTGTTTGGGGGCTATAGCTGGT
ATATACCTTCTAGAATTCGTTACTCCAGACGCAGTAACTAAA------------------
------------------------------GGTTTAGGTAAAACAGCCGTAAACACTTTG
---------CTTCAACCCGGACAAGGATTTGTTGTAGAAGCATTCATTACCCTTATTCTA
GTCTTGGTTATTCACTCG---GTTTGTGATGAAGCCAACCGA------------------
---AGCAACATA---------------GTTACGCCATCCATTTCTATAGGTCTGACCATT
GCTGCCGCCCATTTAGCAGCGATTAAATATACAGGAGCTAGCATGAACCCAGCAAGATCT
TTAGGCCCAGCCGTTGCTCTTGGC---------TTTTGGACAAACCATTGGGTATATTGG
ATCGGACCTATTGTTGGTGGTATACTTGGAGGAACCATCCACACTTTTGTGTTGAAA---
---CGTCATACT---------------------------GAAGAG---------------
------------------------GCA------------AGCTCTTAT------GATCTT

>GAWE01111259_Ramulus_artemis_Drip
ATGGGC--

---------------------------------AAGGCT---GTAGAG------------

---GCG
AAGGCCATCCTGGGTCTGCAGGACATCACC---GACAACAGG------AACATCTGGCGC
ATGCTGGTGGCAGAGTTCCTGGGAACCTTCCTGCTGGTGCTGGTGGGCTGTGGTAGCATC
ACCTCGGAGTGGGACGACGGC---------------------------------------
--TACGCGCCCAGC
ATCGTGCAGATCGCGCTTACGTTCGGCCTCGCCGTCGCCACCATCGTACAGGCGGTGGGA
CATGTGAGCGGAGGTCATGTGAACCCTGCGGTGACCTGCGGCCTGCTAGTGACGGGACAC
ATCAGCCTGCTCAAGGCTATCTTCTACGTGGCTGTGCAGTGCATCGGTGCTGTGGCCGGC
TCCGCCCTTCTCAAGGCGATCACGCCCGAGCACACGGTCGGC------------------
------------------------------AACCTAGGCATGACAACACTGAGCCCGTAC
---------CTGTCTCCACTACAAGGCCTAGTGGTGGAGGCGGCCATCACCTTCCTATTG
GTGCTCGTGGTACATGGA---GTGTGTGACGACAAGAGGGGC------------------
---GACCTCAAG---------------GGATCTGCGCCACTAGCCATCGGGCTCTCCATC
TCCTGCTGCCATCTTGCCGCTATCAAGTACACGGGCTCCAGCATGAACCCTGCCAGAACA
TTCGGGCCGGCCGTCATGACGGGC---------ATCTGGGCCAACCACTGGGTGTACTGG
GCCGGGCCTATCGCGGGAGGCGTGATCGCGGGGCTCGCCTATAGGCTGCTCTTCCAGGCC
---AATAAGGGC------------------------GACGACGAG---------------
------------------------GCC------------AGCTCATAC------GACTTC

>GAWD01031379_Medauroidea_extradentata_Drip
ATGGGC--

---------------------------------AAGGCT---GTAGAG------------

---GCG
AAGGCCATCCTGGGTCTGCAAGACATCACC---GACAACAGG------AACATCTGGCGC
ATGTTGGTGGCGGAGTTCCTAGGCACCTTCTTGCTGGTGCTTGTTGGCTGTGGCAGTATC
ACCGCTAACTGGGAAGTCGAT---------------------------------------
--TATTCTCCCAGC
ATTGTGCAGATAGCGCTCGCCTTCGGTCTCGCCGTCGCCACCATCGTCCAGGCGGTGGGC
CATGTCAGCGGAGGCCACGTGAACCCAGCTGTGACCTGTGGCTTGCTGGTGACGGGACAC
ATCAGTCTGCTCAAGGCAATCTTCTACGTGGCGGTGCAGTGCATCGGGGCTGTGGCCGGC
GCCGCCCTACTCAAGGTAATCACGCCAGAGCAAACAGTGGGC------------------
------------------------------AACCTGGGTATGACGAAGCTGAGCCCGTAC
---------TTGTCGCCACTGCAAGGACTTGTAGTGGAGGCTGCCATCACCTTCGTACTG
GTGCTGGTTGTACACGGG---GTGTGTGACGACAAGAGGGGA------------------
---GACATCAAG---------------GGATCTGCGCCGCTCGCCATCGGCCTGTCCATC
TCCTGCTGCCATCTTGCCGCCATCAAGTACACTGGCTCCAGCATGAACCCTGCCAGGACC
TTCGGACCGGCCGTCATGACCGGC---------ATCTGGGCCAATCACTGGGTGTACTGG

GCAGGACCAATCGCTGGCGGCGTCATCGCGGGGCTCACCTACAGGTTTCTCTTCCAGGCC
---AACAAGGGC------------------------GACGACGAG---------------
------------------------GCC------------AGCTCCTAC------GACTTC

>GAWG01027137_Extatosoma_tiaratum_Drip
ATGGGC--

---------------------------------AAGGCT---GTAGAG------------

---GCG
AAGGCGATCCTGGGGCTGCAGGACATCACG---GACAACAGG------AACATCTGGCGG
ATGCTGGTGGCGGAGTTCCTCGGCACCTTCCTGCTGGTGCTCGTCGGCTGCGGCAGCGTC
ACCTCCAAGTGGACGGACAAC---------------------------------------
--TACTCGCCCAGC
GTCGTGCAGATAGCGCTGGCGTTCGGCCTCTCCGTCGCCACCATCGTCCAGGCGGTGGGC
CACGTCAGCGGAGGCCACGTGAACCCGGCGGTGACCTGCGGCCTGCTGGTCACGGGACGC
ATCAGCCTGCTCAAGGCCGTCTTCTACGTCGCCGTGCAGTGCATCGGGGCCGTCGCCGGC
GCCGCTCTGCTCAAGGCGATCACGCCCGAGGAGACGGTGGGC------------------
------------------------------ACGCTCGGCATGACGACCCTCGGCCCCTAC
---------ATGACGCCGCTGCAGGACTCGTGGTGGAGGCGGCCATCACCTTCGTGCTG
GTGCTCGTCGTGCACGGC---GTCTGCGACGACAAGAGGGGC------------------
---GACCTCAAG---------------GGCTCGGCGCCGCTGGCCATCGGCCTCTCCATC
TCCGCGTGCCACCTCGCTGCCATCCAGTACACGGGCTCCAGTATGAACCCCGCCAGGACA
TTCGGCCCGGCTGTGATGACCGGC---------ATCTGGGCCAACCACTGGGTGTACTGG
GCCGGGCCTATCGCCGGCGGCGTGGTGGCAGGGCTCGCCTACAGGGTCCTCTTCCAGGCC
---AACAAGGGC------------------------GACGACGAG---------------
------------------------GCC------------AGCTCCTAC------GACTTC

>GAWC01082932_Aretaon_asperrimus_Drip
ATGGGT--

---------------------------------AAAGCT---GTAGGTGTTGCAGAG---

---GCG
AAGGCCATCCTGGGACTGCAGGACATCACG---GACAACAGG------AACATCTGGCGC
ATGCTGGTGGCTGAGTTTTTGGGCACCTTCTTGCTGGTGCTGGTCGGCTGCGGCAGCGCC
ACCTCCAACTGGACAGAGAAC---------------------------------------
--TACTCCCCCAGC
GTCGTCCAAATTGCTCTCGCATTCGGTCTAGCCGTCGCCACCATTGTCCAGGCGGTGGGT
CATGTCAGCGGCGGCCATGTGAACCCGGCAGTGACATGCGGCCTTCTGGTGACCGGACAC
ATCAGCCTGCTGAAGGCTGTCTTCTACGTGGCGGTGCAGTGCATCGGGGCTGTGGCTGGC
GCTGCTCTGCTCAAGGCGATCACTCCCCAGGAGACTGTGGGC------------------
------------------------------ACGCTGGGCATGACGACCATCAGCCCGTAC
---------ATGACTCCGCTGCAAGGACTGGTGGTGGAGGCGGCCATCACCTTCGTGCTA
GTGCTGGTGGTACATGGC---GTGTGTGACGACAAGCGAGGC------------------
---GACCTCAAG---------------GGCTCGGCGCCGCTCGCCATCGGCCTGTCCATC
TCCACCTGTCACCTCGCTGCCATCCAGTACACTGGCTCCAGCATGAACCCCGCAAGGACG
TTCGGACCGGCTGTGATGACGGGC---------ATGTGGACAAACCACTGGGTATACTGG
GCTGGACCCATTGCTGGAGGTGTGGTGGCAGGGGTCGTCTACAGGTGCTTGTTCCAGGCC
---AATAAGGGC------------------------GATGATGAG---------------
------------------------GCC------------AGCTCCTAC------GACTTC

>BAG72254_Coptotermes_formosanus_Drip
ATGGTA--

---------------------------------AAACCA---ACAGGT------------

---GCG
AGGGCTCTGCTCGGTCTGCACGACATCACG---GACAACCGA------AACATATGGCGC
ATGCTGTCCGCTGAATTCTTGGGCACATTCTTCCTTGTCTTGGTTGGCTGCAGCAGCATC
ACGCCTTTGGCAGGC---
---CGTTCCAGT
GTCATCCAGATCGCCTTCACTTTCGGCCTGGCTGTTGCCACGATAGCGCAGGCTGTGGGA
CATGTCAGTGGTTGCCACATCAACCCGGCCGTAACGTGTGGGCTGATCGTCTCGGGGCAC
GTCAGCATCCTCAAGGCTGTCTTTTACATCGCCGTGCAGTGCATCGGTGCAGTTGCTGGG
GCTGCAGTGTTGCAGGCTCTGACCCCTGCAGAAGCGGAGGAC------------------
------------------------------TCGCTGGGTATGACGACAATAAACGCGCTC
---------GTGACGCCGGTACAGGCCTTCTTTATGGAGGCACTTATCACCTTCGTGCTG
GTTCTCACTGTGGAGGCC---GTGTGTGACGAGAGAAGAACC------------------

```
---GACGTGAAG---------------GGCTCCGCTCCTCTGGCCATTGGCTTGTCCATT
GCTACCTGTCATCTTGCAGCGATAAACTACACAGGAGCTAGCATGAATCCCGCCCGGTCA
TTTGGACCCGCCGTAATGACAGGA---------ATGTGGAATCATCACTGGGTGTACTGG
GCGGGCCCCATCGTGGGCGGTGTGGTTGCTGGAGCAGTGTACAGTTTCATCTTCCGAGCC
---AGGAAAGGG------------------------GAAGACGAG---------------
------------------------ACC------------AGCTCGTAT------GACTTC
------------------------------------------------------------
------------------------------------------------------------
------------------------------------------------------------
------------------------------------------------------------
------------------------------------------------------------
------------------------------------------------------------
------------------------------------------------------------
------------------------------------------------------------
------------------------------------------------------------
------------------------------------------------------------
------------------------------------------------------------
------------------------------------------------------------
------------------------------------------------------------
------------------------------------------------------------
------------------------------------------------------------
------------------------------------------------------------
------------------------------------------------------------
------------------------------------------------------------
------------------------------------------------------------
------------------------------------------------------------
------------------------------------------------------------
------------------------------------------------------------
------------------------------------------------------------
------------------------------------------------------------
------------------------------------------------------------
------------------------------------------------------------
------------------------------------------------------------
------------------------------------------------------------
------------------------------------------------------------
------------------------------------------------------------
------------------------------------------------------------
------------------------------------------------------------
------------------------------------------------------------
------------------------------------------------------------
------------------------------------------------------------
------------------------------------------------------------
------------------------------------------------------------
------------------------------------------------------------
------------------------------------------------------------
------------------------------------------------------------
------------------------------------------------------------
------------------------------------------------------
```

```
>FL637621_GO899611_Reticulitermes_flavipes_Drip
ATGGTA------------------------------------------------------
------------------------------------------------------------
---------------------------------AAGCCA---ACAGGT------------
------------------------------------------------------------
---------------------------------------------------------GCG
AGGGCTGTGATCGGGCTGCACGACATCACG---GACAACCGC------AACATATGGCGC
ATGCTGTCCGCTGAATTCTTGGGCACGTTCTTCCTCGTCTTGGTTGGCTGCAGCAGCATC
ACTTCTTTGGCCGGC---------------------------------------------
---------------------------------------------------TCCTCCGGT
GACATCCAGATCGCCTTCACTTTCGGCCTGGCTGTTGCCACGATAGCGCAGGCTGTGGGA
CATGTCAGTGGTTGCCACATCAACCCGGCCGTAACGTGTGGGCTGATCATCTCCGGGCAC
ATCAGCATCCTCAAGGCTGTCTTTTACATCGTCGTGCAGTGCATCGGTGCAGTTGCTGGG
GCCGCAGTGCTGCAGGCTCTGGTCCCTAAAGAAATGGAGGGC------------------
------------------------------TCGCTGGGTATAACGAACATAAATTTGCTC
---------GTGACGCCCGTGCAGGCCTTCTTCATGGAGGCGCTGATCGCCTTCGTTCTG
GTCCTCACTGTGGAGGCA---GTATGTGATGACAGAAGAACC------------------
---GACGTGAAG---------------GGCTCCGCTCCTCTGGCCATCGGTTTGTCAATT
GCTACCTGTCACCTTGCAGCGATAAAATACACAGGAGCTAGCATGAACCCCGCCCGGACA
TTTGGACCCGCCGTAATAACAGGA---------ATGTGGGATAATCACTGGGTGTACTGG
GCGGGCCCCATCGTGGGCGGTGTGGTGGCTGGAGCAGCGTACAGTTTCATCTTCCGGGCC
---AGGAAAGGG------------------------GAAGACGAG---------------
------------------------ACC------------AGCTCGTAT------GACTTC
------------------------------------------------------------
------------------------------------------------------------
------------------------------------------------------------
------------------------------------------------------------
------------------------------------------------------------
------------------------------------------------------------
------------------------------------------------------------
------------------------------------------------------------
------------------------------------------------------------
------------------------------------------------------------
------------------------------------------------------------
------------------------------------------------------------
------------------------------------------------------------
------------------------------------------------------------
------------------------------------------------------------
------------------------------------------------------------
------------------------------------------------------------
------------------------------------------------------------
------------------------------------------------------------
------------------------------------------------------------
------------------------------------------------------------
------------------------------------------------------------
------------------------------------------------------------
------------------------------------------------------------
------------------------------------------------------------
```


>BAM19007_Papilio_polytes_Drip
ATGAAA--

------------ACGGATTACGCCATGGATGAGGTGCAG---AAGAGA------------

---ACG
ACGTCGATCATCGGCGTGTCGGACATTACA---CAGAACAAG------ATGATCTGGCGA
CAGGTGGTAGCCGAGCTGGTGGGCACGTTCCTCCTCACCTCCATCGGCGTCGCCTCCTGC
ATCGCTATCTCAGAATCCAAT---------------------------------------
---GCCCCAAAG
GTCACCAGCATTGCGCTCTGCTTCGGTTTGCTAGTCGGCTCCATCGTACAGGCGATCGGG
CACGTGTCTGGCGGGCACATCAACCCAGCAGTGACGTGCGGTCTGTTCGTATCTGGTGAC
ATCAAGCTGCTAAAAGCATTGCTGTACGTGGTCGCACAAGCGGTAGGCGCGGTCGCGGGA
GCTGCGTTCATCAGGCTCGCTGTGCCCGAGAATGTTGTCGGA------------------
------------------------------GGCTTCGGTATGACTTTGCCTGGACCAGGA
---------GTTACTGACGGACAGGCTTTGCTGGTGGAGGCTCTGATCACCTTCGTTCTG
GTGCTGGTGGTGCAGGGC---GTGTGTGACGGCGGCCGCACG------------------
---GACCTCAGG---------------GGCTCCGCGCCTCTCGCTATCGGACTCAGCATC
ACCGCCTGCCATGCCGCATGCATCCCGTTCTCTGGCTCGAGCATGAACCCTGCGCGTTCC
TTCGGCCCTGCCCTCGTAATGGGC---------GACTGGACTTCGCACTGGGTGTACTGG
GTGGGACCGATCGTGGGCGGTGTGGTTGCCGGCCTGGTGTACAGGTTCATCTTCTGGATC
---GGCAAG---------------------------GGCGGCGAG---------------
------------------------AGC------------GGCTCCTAC------GACTTC

>BAM17858_Papilio_xuthus_Drip
ATGAAA--

------------ACGGATTACGCCATGGATGAGGTGCAG---AAGAGA------------

---ACG
ACCTCTATCATCGGAGTATCGGACATCACA---GAGAACAAG------CAGATCTGGAGG
CAGGTGGTAGCCGAGCTGGTAGGCACGTTCCTGCTTACCTCAATCGGCGTCGCATCATGC
ATCGCCATCTCTGAATCCAAC---------------------------------------
---GCTCCTAAG
GTCACCAGCATTGCGCTCTGCTTCGGTCTCCTAGTCGGCTCTATCGTACAGGCGATCGGG
CACGTGTCGGGCGGGCACATTAACCCGGCGGTGACATGCGGACTGTTCGTGTCGGGAGAC
ATCAAATTGCTCAAGGCTCTGCTGTACGTGGTCGCGCAGGCAGTCGGCGCAGTCGCTGGT
GCCGCATTCATCAGGCTCGCCGTGCCCGAGAATGTTGTGGGA------------------

```
------------------------------GGCTTCGGTATGACCCTGCCCGGACCTGGA
---------GTTAATGATGGACAGGCTCTGCTGGTGGAAGCATTGATCACGTTTGTACTC
GTGTTGGTGGTGCAAGGT---GTATGCGACGGCGGCCGTACC------------------
---GACCTGAAG---------------GGCTCCGCACCCCTTGCTATCGGCCTCAGCATT
ACCGCCTGCCATGCCGCATGTATCCCATTCTCTGGCTCGAGCATGAACCCGGCGCGTTCC
TTCGGTCCTGCCCTCGTTATGGGC---------GACTGGACTTCCCACTGGGTGTACTGG
GTAGGTCCGATCGTGGGTGGTGTGGTCGCCGGCCTGGTGTACAAGTTTATCTTCTGGATC
---GGCAAG---------------------------AGCGGCGAG---------------
------------------------AGT------------GGCTCCTAC------GACTTC
------------------------------------------------------------
------------------------------------------------------------
------------------------------------------------------------
------------------------------------------------------------
------------------------------------------------------------
------------------------------------------------------------
------------------------------------------------------------
------------------------------------------------------------
------------------------------------------------------------
------------------------------------------------------------
------------------------------------------------------------
------------------------------------------------------------
------------------------------------------------------------
------------------------------------------------------------
------------------------------------------------------------
------------------------------------------------------------
------------------------------------------------------------
------------------------------------------------------------
------------------------------------------------------------
------------------------------------------------------------
------------------------------------------------------------
------------------------------------------------------------
------------------------------------------------------------
------------------------------------------------------------
------------------------------------------------------------
------------------------------------------------------------
------------------------------------------------------------
------------------------------------------------------------
------------------------------------------------------------
------------------------------------------------------------
------------------------------------------------------------
------------------------------------------------------------
------------------------------------------------------------
------------------------------------------------------------
------------------------------------------------------------
------------------------------------------------------------
------------------------------------------------------------
------------------------------------------------------------
------------------------------------------------------------
```


>AFC34081_Chilo_suppressalis_Drip
ATGAAA--

------------ACGGATTACGCTGTGGATGAGATGCAG---AAAAGG------------

---ACG
AGCTCGATCATCGGCCTGTCCGATGTGACG---GACAACAAG------CTGATTTGGCGG
CAGCTAGTGGCCGAGCTGATGGGCACCTTTGTGCTGACATCGCTTGGTGTCGCCGCCTGT
ATCGCCATCAACCAATCGCAG---------------------------------------
---GCGCCGCAG
ACCACAAGCATCGCACTCGCCTTCGGGCTGCTCGTCGGATCCATCGTACAGGCAATCGGT
CATGTGTCTGGTGGGCACATCAACCCTGCAGTCACCTGCGGACTGTTCGTCTCTGGCGAC
ATCAAACTGCTCAAGGCCATCTTCTACATCGTGGTGCAGTCTCTCGGTGCCGTAGCAGGC
GCAGCCTTCGTCAGGTTGGCTGTTCCGGAAGCAGCACTAGAACAC---------------
------------------------------GGATTCGGTTTGACTTTGCCAGGCACAGGA
---------GTTAACGAAGCTCAGGCGTTGCTCATCGAAGCCTTGATCACATTCCTGTTA
GTGCTGGTGGTGCAGGGC---GTGTGCGACGGGCGCCGCAGC------------------
---GACATCAAG---------------GGCTCCGCGCCCCTCGCTATAGGGCTCACCATC
ACCGCCTGCCACGCCGCCTGCATTCCTTTCTCAGGATCGAGTATGAACCCGGCTAGATCT
TTCGGGCCCGCTCTAGTTATTGGT---------GACTGGACATCACACTGGGTGTACTGG
GTAGGCCCCATAGTGGGAGGTGTGATTGCCGGCCTACTCTACAGGTTTGTGTTCCGTATC
---GGCAAG---------------------------AGTGGTGAC---------------
------------------------AGC------------GGCTCCTAT------GACTTC

>CAEZ01007265_Heliconius_melpomene_melpomene_Drip
ATGAAG--

------------ACGGATTATCCAATGGATGAGATGCAT---AAGAGG------------

---ACG
AGCTCCATAATCGGTTTGGCCGATGTAACA---GACAACAAA------CTGATCTGGAGG
CAGCTGACGGCGGAGCTGATCGGAACATTCCTGCTCACCTCCATCGGTGTTGCTTCGTGT
ATTGCCATATCTGATGCAGCA---------------------------------------
---GTACCACAC
GTCGCTAGTATCGCGTTATGCTTCGGTCTTCTTGTTGCTTCTATCGTGCAGGCTATAGGC
CACGTGTCCGGCGGACATATTAATCCGGCTGTTACATGCGGACTGTTCGCGGCTGGCGAC
GTGAAGCTGCTGAAGGCGATATTCTACATCGTGGTACAATGCTTGGGAGCTATAGCTGGG
GCAGCGTTCATCAGGTTGGCTGTACCTGAAGATAGAGTTATG------------------
------------------------------TCGTTTGGAATGACTCTACCTGCATCTAAT
---------GTCACCGAACCACAGGCACTGCTAATAGAGGCACTCATCACGTTCGTGCTA
GTGATAGTGGTTCAGGGT---GTTTGCGACGCGCGCCGCAAC------------------
---GACATCAAA---------------GGGTCCGCACCGTTGGCCATCGGCCTCAGCATC
ACCGCCTGTCATGCTGCTTGCATACCGTTCTCGGGTTCCAGCATGAACCCCGCGCGTTCT
TTCGGCCCCGCCCTCGTCATGGGC---------GTCTGGACCGCGCACTGGGTATACTGG
GTGGGTCCTATCGTGGGCGGCGTGGTCGCCGGCCTCATCTACAAATATGTACTTCGCATT
---GGCAAG---------------------------AGTGGAGAC---------------
------------------------AGT------------GGCTCATAC------GATTTA

>XM_032654866_Danaus_plexippus_Drip
ATGAAG--

------------ACGGATTATCCGATGGATGAAATGCAG---AAGAGG------------

---ACT
GGTTCTATCATTGGTCTGTCGGATGTGACT---GAAAACAAA------CTGATATGGCGC
CAGTTGGTCGCGGAGCTGGTGGGGACCTTTCTGCTGGTGTCTGTGGGTGTAGCTTCCACC
ATCGCTATCACGGAAATGAAT---------------------------------------
---GCTCCTATA
ACCACCAGCATCGCGCTGTGCTTCGGATTACTCGTCGGATCTATCGTGCAGTGCATCGGT

CACGTGTCTGGAGGTCACATAAACCCGGCCGTAACCCTCGGACTGCTGGCATCTGGTGAC
GTCAAAGTGTTGAAAGCCATCTTCTACATAGTGGTCCAATGTCTCGGAGCTACAGCTGGA
GCTGCTTTCATAAGGTTGGCTGTACCTGAGAACAGAGTGTTT------------------
------------------------------TCATTCGGTGTAACACAACCAGGTCCAGGG
---------GTCACTGACGCTCAGGCATTGCTGGTGGAGGCACTGATAACGTTTATGCTG
GTGTCAGTGGTGCAGGGA---GTGTGCGACGCCGGTCGTAAT------------------
---GACATCAAA---------------GGTTCAGCACCCCTGGCTATTGGGCTAAGCATC
ACCGCCTGCCACGCTGCTTGTATTCCATTTTCTGGTTCAAGCATGAACCCCGCGAGATCT
TTCGGGCCGGCTCTCGTTATGGGC---------AACTGGAACGCACACTGGGTATACTGG
GTGGGTCCGGTCCTGGGAGGAGTCCTCGCCGGCCTGGTGTACAGGTTCGTGCTCCGCATC
---AACAAA---------------------------GCCGGCGAC---------------
------------------------AGC------------GGCTCCTAC------GACCTG

>AB178640_Bombyx_mori_Drip
ATGGCA--

------------ACG---------------AAAACTACT---GAGAAG------------

---ACA
AGCTCCATCATCGGGCTGTCAGATGTAACG---GACAACAAG------CTGATCTGGAGG
CAGCTGGTCGCGGAGCTGGTCGGCACCTTCCTCCTTACTTCTATTGGCGTGGCCGCCTGC
ATCACCATCAACGCCAGCACG---------------------------------------
---GCGCCCCAC
ACCACCAGCATTGCGTTGTGCTTTGGGTTGCTCGTTGGATCTATAGTGCAGGGCATCGGT
CACGTGTCTGGAGGACACATCAACCCCGCGGTGACAGCCGGTCTCTTCGCGGCGGGAGAC
ATTAAGCTGCTAAAAGCAATCTTCTACATCGTAGTGCAGAGCCTCGGAGCAGTAGCTGGA
GCCGCCTTTATCAGGTTGGCGATTCCTGCCGACAGCATCGGT------------------
------------------------------GGTTTTGGTATGACCCTACCCGGACCCGGA
---------GTTACAGAAGCGCAGGCCGTGCTGGTGGAGGCTCTGATCACGTTCGTGTTG
GTCATGGTGGTGATGGGT---GTGTGCGACCCGCAACGTAAT------------------
---GACCTCAAG---------------GGTTCCGCTCCCCTGGCTATCGGACTCAGCATC
ACCGCCTGCCACGCTGCTGTCATACCTTTCACGGGATCCAGCATGAACCCGGCCCGAACA
TTCGGCCCAGCCTTGGTGATCGGC---------AATTGGACATCTCAATGGGTTTACTGG
GTGGGTCCCGTCGTGGGCAGCGTGGTCGCTGGACTACTCTACAAATTCGCTCTGCGCATC
---AAAAAA---------------------------GCCGGAGAC---------------
------------------------ACC------------GGCTCTTAT------GACTTC

>Q23808_Cicadella_viridis_Drip
ATGGCC--

------------------------GCCGACAAGTCAGTG---GACAAC------------

---ACC
AAGAAAATCATCGGCATCGATGACATCACC---GACACCAAG------ACCATTTGGCGG
TGCTTGGCTGCCGAGCTTATCGGAACCCTGCTGCTGGTACTGATAGGCACAGGGTCCTGC
ACCGGGGTCCAGATCTCAGAG---------------------------------------
---GGTGATGTT
GTGGTCAGGATCGCCCTCACCTTCGGCTTCATCATCGCCACCATGGTCCAGTGTATTGGC
CACGTGTCAGGGTGCCACATCAACCCCGCCGTCACCTGCGGCCTCCTCGTCACCGGCCAC
ATCAGTATCCTGAAGGCGATCTTCTACATCATCGTCCAGTGTGTGGGTGCCATCGCCGGC
TCTGCCATCCTCAAGGTAATAACCCCTGCAGAGTTCAGGGGC------------------
------------------------------ACTCTTTGTATGACGAGTCTCGCCCCCGGT
---------GTGACCCCGCCCATGGGCTTCCTGGTGGAGGCTTGCATCACCTTCGTGCTG
ATCCTGCTGGTGCAGAGC---GTCTGCGACGACCGCCGCAAG------------------
---AACCTGGGC---------------AACGCCGCTCCAGTTGCCGTAGGCCTAGCCATC
ACATGCTGCCATCTCGCTGCCATCAAGTACACCGGATCCAGCATGAACCCCGCCAGGTCA
TTCGGGCCCGCAGTCAATGGAGACGAC------AACTGGGCCAACCACTGGGTGTACTGG
GCGGGCCCCATCGTGGGGGGAGTGGTTGCCGGCATCACCTACCGAGCCCTGTTCAGGGCC
---AGGAAGCCC------------------------GAGGAAGAG---------------
------------------------GCC------------AGCTCCTAC------GACTTC

>JP783671_JP783672_Nylanderia_pubens_Drip
ATGAGT--

------------------------------AAACAGTGG---AAAGGT------------

---GTC
AAGGAGTTCGTCGGTCTGGAGGAAGTCACC---AAGCTCGAC---------TTCCTGGTG
ATGCTGTTTGCCGAAATTCTGGGCACGTTTCTGTTGGTCCTCCTAGGCTGCGCCTCTTGC

ATCACATGGTCTTTGGATACA---------------------------------------
---CCACCGACT
GTTTTGCACATTGCGTTCACCTTTGGCCTCGCCGTGGCAAGTTTGGCACAAGTTTTAGGT
CCGATTTCCGGTTGTCACGTAAACCCAGCTGTATCACTCGGTCTGATGGTCAGCGGAAAT
TGCAGTCTTCTTAAAACAATTAGTTATGTGGTCTGTCAATGTTGCGGTGCTATTGCGGGA
GCTGCTGTTCTTAAGCTGATAATACCCGGGACGCAGCCCGCGAAC---------------
------------------------------GGCCTCGGTGCCACCATGCTGGGTCACGGC
---------GTATCGGTGTCTCAGGGTGTCGTGATGGAGGCCATCATCACGTTCCTCCTA
GTGCTGGTGGTGCACGCA---GTGACAGATGCCAAGAGGTCC------------------
---GACACGAAG---------------GGCTGGGCCCCGTTGGCGATCGGTCTGACGATC
GCGACGGCTCACATGGCAGCTGTACCCATCACTGGCAGCAGCATGAATCCCACCAGGACT
CTGGGTCCAGCTGTGGTGGAGGAT---------GCGTATCAGAATATATGGGTCTACTGG
GTGGGTCCGCTGCTCGGTGCCCTCTTGGCCGGTGGCGTGTACAGGATGGGTCTGAGGGCC
---CACAGG---------------------------GATGAGGAT---------------
------------------------GAG------------GGTTCGTAC------GACTTT

>XM_011270912_Camponotus_floridanus_Drip
ATGATT--

------------------------------TTTTTTATT---TCAGGT------------

---ATT
AAGGAGTTTGTCGGCTTTGAAGAGGTCACC---AAGCTCGAC---------TTCCTGGTG
ATGCTGTTCGCCGAGATTCTGGGCACGTTCCTGTTGGTCCTCATAGGCTGTGCCTCTTGC
GTTACGTGGATAGAAAATACG---------------------------------------
---CCGCCGACT
GTTGTGCATATTGCATTCACCTTTGGCCTCGCTGTGGCAAGTTTGGCTCAGGTTTTAGGT
CCAATTTCTGGATGTCACGTGAATCCAGCTGTGTCACTTGGTCTAATGGTCAGCGGAAAT
TGCAGTTTTCTTAAAACTGTTAGTTATATAATCTGTCAATGTTGCGGCGCTATCGCGGGT
TCTGCCGTTCTCAAGCTGATAATACCCAAGACACCAGCTGCTGGC---------------
------------------------------GGTCTCGGCGCTACCATGCTGGGTCACGGC
---------GTGTCGGTAAGCCAGGGTGTCCTAATGGAGATCATCATCACGTTCCTGCTG
GTGCTGGTGGTACACGCG---GTGACGGATGCTAAGAGGTCC------------------
---GACACGAGG---------------GGTTGGGCCCCGGTGGCGATCGGTCTGGCGATC
GTGGCATCTCATATGGCGGCGGTCCCCATCACCGGCAGCAGCATGAATCCTGCCAGGACT
CTGGGCCCAGCCGTGGTGGAGGAT---------ATGTATCAGGACGTATGGATTTACTGG
GTGGGTCCGCTCATCGGAGGTTGCTTGGCCGGGGGCATCTACAGGATGGGCTTAAGGGCT
---AATAGG---------------------------GAGGACGAC---------------
------------------------GAG------------GGTTCATAC------GACTTT

>ADOQ01012968_Linepithema_humile_Drip
ATGAGC--

------------------------------AAACCGTGG---AAAGGT------------

---GTG
AAGGAGTTTGTCGGCCTGGAGGAGGTCAGT---AGGCTTGAA---------TTTCTGGTG
ATGCTATTCGCTGAGGTCCTGGGCACGTTTCTGCTGGTCCTCATTGGCTGTGCCTCCTGC
ATCAAATGG---GCAACCACA---------------------------------------
--CCGAAC
GTATTGCACATCGCGTTCACCTTTGGTCTCGCAGTGGCAAGTTTGGCACAGGTGTTAGGT
CCGATTTCTGGTTGTCACGTGAACCCGGCTGTGTCGTTCGGTCTGATGGTCAGCGGAAAT
TGCACTTTTCTCAAAACCGTCAGTTATGTGGTTTGTCAGTGTTGCGGCGCTATTGCGGGA
TCCGCCGTTCTCAAGGTAATGATACCCAAGACGCAGATTGAGAAC---------------
------------------------------GGTCTGGGCGCCACTATGCTGGGCCCCGGC
---------GTGTCGGTGGCCCAGGGTGTCGTGATGGAGGCGATTGTCACGTTTCTCCTG
GTGCTGGTGGTGCACGCG---GTCACAGACCCCAAGAGAACC------------------
---GACACGAAG---------------GGCTGGGCGCCGCTGGCGATCGGCCTGACGATC
GCGGTGGCTCACATGGCGGCGGTGCCCATCACCGGCAGCAGCATGAACCCGGCCAGGACT
CTGGGCCCGGCCGTGGTGGAGGAT---------AATTATCAGGACATGTGGGTCTACTGG
GTGGGTCCGCTCATCGGCGGCTGCGTAGCCGGCGGTGTGTACAGAATGGCGCTGAGGGCC
---AACAAG---------------------------GAAGATGAC---------------

------------------------GAG------------GGTTCGTAC------GATTTC

>XM_026139579_Solenopsis_invicta_Drip

------------------------------TTTTCTTTC---GCAGGC------------

---ATG
AAGGAGTTCGTTGGCCTGGAGGAGGTCACC---AAGCTCGAC---------TTCCTGGTG
ATGCTGATCGCTGAGATCCTGGGCACGTTTCTGCTGGTCCTCATAGGCTGCGCTTCCTGC
ATCTCGTGGATAGTGACGATG---------------------------------------
---CCACCGAAC
GTCTTGCACATCGCGTTCACCTTCGGCCTCGCGGTAGCGAGCTTAGCGCAGGCTCTAGGT
CCGATTTCCGGCTGTCACGTAAACCCGGCCGTGTCGTTCGGCCTATTGATCAGCGGAAAT
TGCAGTCTCCTCAAAACCATCTGTTACATAGTCTGCCAGTGTTGCGGCGCTATTGCCGGA
TCCGCTATTCTCAAGCTGATCATACCCAAGACACAAGCCTCCCAC---------------
------------------------------GGCCTAGGCGTCACCGCGTTGCATCCCGAC
---------GTGACCGCGGGTCAGGGTTTAGGAATGGAAATAATCATCACGTTCCTGCTG
GTGCTGGTCGTGCACGCC---GTCACGGATCCCAAGAGGACC------------------
---GACGCGAAG---------------GGTTGGGCCCCGCTCGCGATTGGCCTGACGATC
TCGGTGGCCCATATGGCGGCGGTGCCCATCACCGGCAGCAGCATGAACCCCGCTAGGAGT
CTAGGCCCGGCCGTGGTGGACGAC---------AAGTACGTGAATCTATGGGTTTACTGG
ATAGGCCCGCTCGTCGGCGGTGGCATAGCCGGTTTGGTGTACAGGATGGGCCTGAAGGCC
---AACAAG---------------------------GAGGACGAC---------------
------------------------GAG------------GGCTCGTAC------GACTTC

>XP_003394164_Bombus_terrestris_Drip
ATGGGC--

------------------------------AAGGGTTGG---AAAGGC------------

---ATG
AAAGATTTCGTCGGCCTCGAAGAGGTGATG---AAGTTCGAG---------TTTCTGTTG
AAGCTGTTCGCCGAAACCTTGGGAACGTTTCTTCTGGTGCTGATCGGCTGTGCATCCTGT
ATCACGTGGACCGCGGACAAT---------------------------------------
---CCTCCGACG
GTGGTACACATCGCGTTCACCTTTGGTCTGGCTGTCGCCAGCTTAGCTCACGTATTAGGT
CCGATTTCAGGATGTCACGTGAATCCAGCTGTATCGATGGGACTTTTAGTAAGCGGGAAT
TGCAGCTTTCTAAAGACAATTTGTTACATAGTTTGCCAGTGTTGCGGAGCAATCGCGGGT
TCAGGAATTTTAAAGCTATTGATCCCTGTTGAAGCGGCAAATAAC---------------
------------------------------GGCTTAGGAGCGACAAATTTGGGCTTACTG
---------GTCAACCAAATGCAAGGCATCTTCATGGAAGCGATCGTTACATTCCTGCTA
CTTCTGGTTATCCATGCA---GTAACAGATCCCAAGAGAACC------------------
---GACACAAAA---------------GGATGGGCGCCTTTGGCGATCGGGTTGACCATC
ACCGTGGCTCACATGGCTGCTGTACCTGTAACAGGAAGTAGCATGAACCCGGCTAGGTCG
TTAGGTCCTGCGATCGTCCTTGGT---------AAATGGACGATCTGTGGATCTATTGG
GTCGGACCGATCCTCGGAGCCTGTATCGCTGGTGCCTTGTACAAATTGGCGTTCAGACAT
---AAAACG---------------------------ATGGACGAT---------------
------------------------GAT------------GCTTCTTAC------GACTTC

>XP_003487533_Bombus_impatiens_Drip
ATGGGC--

------------------------------AAGGGTTGG---AAAGGC------------

---ATG
AAAGATTTCGTCGGCCTCGAAGAGGTGATG---AAGTTCGAG---------TTTCTGTTG
AAGCTGTTCGCCGAAACCTTGGGAACGTTTCTTCTGGTGCTGATCGGCTGTGCATCCTGT
ATCACGTGGACCGCGGACAAT---------------------------------------
---CCTCCGACG
GTGGTACACATCGCGTTCACCTTTGGTCTGGCTGTCGCCAGCTTAGCTCACGTATTAGGT
CCGATTTCAGGATGTCACGTGAATCCAGCGGTATCGATGGGACTTTTAGTGAGCGGGAAT
TGCAGCTTTCTAAAGACAATTTGTTACATAGTTTGTCAGTGTTGCGGAGCAATCGCGGGT
TCAGGAATTTTAAAGCTATTGATCCCTGTTGAAGCGGCAGATAAG---------------
------------------------------GGCTTAGGAGCCACAAATTTGGGCTTACTG
---------GTCAACCAAATGCAAGGCATCTTCATGGAAGCGATTGTTACATTCCTGCTA
CTTCTGGTTATCCATGCA---GTAACAGATCCCAAGAGAACC------------------
---GACACGAAA---------------GGATGGGCGCCTTTGGCGATCGGATTGACCATC
ACCGTGGCTCACATGGCTGCTGTACCTGTAACAGGAAGTAGCATGAACCCGGCTAGGTCG

TTAGGTCCTGCGATCGTCCTTGGT---------GAATGGGACGATCTGTGGATCTATTGG
GTGGGACCGATCCTCGGAGCCTGTATCGCCGGTGCCTTGTACAAATTGGCGTTCAGACAT
---AAAACG---------------------------ATGGACGAT---------------
------------------------GAA------------GCTTCTTAC------GACTTC

>XM_016986305_Nasonia_vitripennis_Drip
ATGAGC--

------------------------------AAATCGTGG---CAGGGC------------

---GCT
AAGGACTACGTGGGCATCGACGAGGTGACG---AAGGTGGGC---------TTCCTCGTG
CCCCTGTTCGCCGAGGCTCTAGGCACCTGTCTCCTCGTCATAATCGGCTGCGCGTCCTGC
ATCAACTGGGGC------ACC---------------------------------------
---GCGCCCACC
GTCTTGCACATCGCCCTCACCTTTGGTCTCGCCGTTGCCAGTTTAGCGCACGTGCTGGGA
CCGGTGTCCGGCTGCCACGTGAATCCTGCGGTGACTCTGGGCCTCCTCGTCAGCGGCAAC
TGCACGATACTGAAGGCGATATGCTACGCCGTCTGCCAGTGCTGCGGTGCCATCGCCGGC
GCTGCAATTTTGAGAGCCGCGCTACCAGCGAAGGCGACGTCCCAA---------------
------------------------------GGTCTGGGCATGACGCTCCTGGGCAACGAG
---------GTGAACAACGGCCAGGGTATCCTGATCGAGGCGATCATCACCTTCCTCCTG
GTCCTCGTGGTCCACGGA---GTAACCGACCCAAAGCGCGAA------------------
---GACTGCAAA---------------GGCTGGGCTCCGATGGCCATAGGCCTGAGCATC
AGCGTCTCGCACTTGGCGGCCGTGCCGCTTACCGGCAGCAGCATGAATCCCGCCAGATCA
CTCGGACCCGCCGTCATCGTTGGC---------CTCTGGACCGACCAGTGGATCTACTGG
GTCGGACCGCTTCTCGGAGCTCTGGTCGCCGGTGCGCTTTACAAGATCAGTCTCAGGGCC
---AGAAGCAAG------------------------GAGGAGGAG---------------
------------------------CAG------------GCGTCGTAC------GACTTC

>GAKG01000818_Cotesia_vestalis_Drip
ATGAGC--

------------------------------AAGACGTGG---AAAGGT------------

---GCG
AAAGATTTTCTAGGCTTGGAAGAAGTCTCT---AAAATCGAG---------TTCCTGGTG
CCGCTGTTCAGCGAAGCTCTAGGAACGTTTTTATTGGTCCTTATTGGGTGCGCATCCTGC
CTCACATGGGATGAATCCCAT---------------------------------------
---CCGCCGACT
GTGATCCACATCGCATTTACTTTTGGTCTCGCTGTTGCTGCGTTAGCTCAGGTACTTGGA
CCAGTATCTGGATGTCACGTGAACCCTGCGGTGACGGCAGGACTTGTCGTCAGTGGAAAC
TGTTCATTATTAAGAGCAATTTGCTACATTGTTTGTCAATGTTGTGGTGCAATAGCTGGA
GCAGCTGTTCTGAAAGTCGTAATACCTGGACCCACGACATCTCGC---------------
------------------------------GGAATAGGAGCGACGACCCTGAGCGACGGA
---------GTGGACAGTGGTCAGGGAATTCTGATGGAAGCGATAGTAACGTTTCTCCTG
GTGCTGGTGGTCCACGCA---ACGACTGACTCGAAGCGTCGG------------------
---GACACCGTT---------------GGCTGGGCTCCGCTGGCGATCGGGTTGACAATA
ACAGTGTCCCACCTGGCAGCTGTTCCACTCACTGGAAGCAGCATGAACCCCGCGAGGTCT
TTCGGCCCAGCGGTGATCCTCGGC---------TCCTGGACTGACCAGTGGATTTACTGG
GTTGGACCCGTCGTAGGTGGGATTGTCGCCGGTGGACTCTACAAGTTGGGACTCAGGGCC
---AAGAATAAA------------------------GATGATGAT---------------
------------------------GAA------------GCTTCTTAT------GACTTT

>JR487579_Rhynchophorus_ferrugineus_Drip
ATGGTT--

---------------GAATTG---------AAGGGT------TCTGGA------------

---GCT
GAGAGTATCATCGGTGTCTCGGATATAACC---GAGAATCGA------AGCATCTGGACA
GCTTTGATAGCTGAGTTTGTGGGTACCTTCCTGTTGGTTGCTATAGGATGTGGTTCTTGT
ATAGCTATCAGCGCATCCAGA---------------------------------------
---GTCTCCAGT
GAGGTTCAAATCGCTCTTACCTTTGGTTTAATTGTGGCTGCTATGGCTCAGTCTATTGGT
CACGTCAGCGGATGCCACATCAATCCTGCCGTAACAGCGAGCCTCTTCGTAACAGGAGAC
ATTAAACTACTGAAATCATTGCTGTACATTCTTGTTCAATGTATTGGAGCCGCAGCTGGG
GCGGCATTCTTGAAATTCATGTTGCCTGAAAGTGCTGTAGGG------------------
------------------------------AATTTGGGAATGACCAGTGTTAATGCTGCC
---------CTGACTCCAGCTCAAGGATTTTTATTCGAACTCACACTGACGTTCGTTCTT

TTGTTCGTTGTGCAAGCG---GTTTGCGATGGAAGGAGGAAG------------------
---GATATTAAG---------------GGATCTGTGCCTCTGGCCATTGGTCTGGCTGTT
GCTGTAGTCCATTTATGTGGCATTCAGTTTACTGGTTCCAGCGTAAATCCAGCCAGGACT
CTAGGACCTGCGATTGTGATGAAC---------CTTTGGGAAAACCACTGGGTATATTGG
GCGGGTCCCATTTCTGGTGGAGTTTTAGCTGGACTTCTCTACAAACTCGTCTTCAAAGTA
---CAGAAG------------------------------GGAGAC---------------
------------------------AAC------------GATTCTTAC------GACTTT


```
------------------------------------------------------
>AEE63193_Dendroctonus_ponderosae_Drip
ATGGCT------------------------------------------------------
------------------------------------------------------------
---------------GATGTC---------AAAGGTACCTCGAGCTTG------------
------------------------------------------------------------
---------------------------------------------------------AGC
GATAGTATCCTAGGCCTGAACGAGATCACA---GAAAACAAA------TCGCTATGGAAA
ATGCTGGCTGCTGAGTTTATTGGCACACTGATTTTGGTCACAGTGGGTTGTGGATCCTGC
CTGGGA------------------------------------------------------
---------------------------------------------------AATCCCACA
ATTGTCCAGATCGCCCTGGCTTTTGGCCTAACAGTTGCCGCTCTAGTCCAAGCAATCGGC
CATGTGAGCGGCGGCCACATTAATCCTGCAGTAACAGTCAGTTTCTTCATCACTGGAGAG
ATCAATTTGGTGCGAACCATTCTCTTCATTCTAGTGCAATGCATTGGAGCTATTGCGGGG
GCTGGAATATTAAAGGCTTTGCTGCCCGCTTCCTTCATCAACGGC---------------
------------------------------AACCTGGGCTTCACTGAAGTGGCCCCAGAA
---------ATCCAGCCAATCAATGGACTGTTCTTCGAGCTTGTTTTCACTTTTATTCTG
GTGTTTGTTATCCATGGC---GTTTGCGATGGAAGAAGGAAC------------------
---GACATCAAA---------------GGCAGCATTCCGCTGGCCATTGGACTTACCGTT
ACTGCAGCCCATTTAAGTGGGATTCACTTCACTGGGTCCAGCATCAATCCAGCCCGCACT
TTCGGCCCTTCCGTGGTCATGAAC---------TTCTGGAATGACCACTGGGTCTATTGG
GTGGGCCCAATTGCAGGCGGGGTGCTCGCAGGGTTGCTTTACAAGTACATCTTTAAGGTG
---CAGAAG------------------------------GGCGAC---------------
------------------------AAC------------GATTCCTAC------GATTTT
------------------------------------------------------------
------------------------------------------------------------
------------------------------------------------------------
------------------------------------------------------------
------------------------------------------------------------
------------------------------------------------------------
------------------------------------------------------------
------------------------------------------------------------
------------------------------------------------------------
------------------------------------------------------------
------------------------------------------------------------
------------------------------------------------------------
------------------------------------------------------------
------------------------------------------------------------
------------------------------------------------------------
------------------------------------------------------------
------------------------------------------------------------
------------------------------------------------------------
------------------------------------------------------------
------------------------------------------------------------
------------------------------------------------------------
------------------------------------------------------------
------------------------------------------------------------
------------------------------------------------------------
```


>XM_967769_Tribolium_castaneum_Drip
ATGGCC--

---------AACAAAGAAATC---------AAGGGT------------------------

---GCG
GACAGCGTTTTAGGCCTTTCAGAAATCACC---GAAAACAAA------GCAATATGGCGA
ATGTTAGTGGCAGAATTCATCGGCACATTTTTACTGGTCGTCATTGGTTGTGGCTCAATA
GTT---------TTATCAGAA---------------------------------------
---GAGGTAGTA
ATTGTTCGCATTGGTGCAACATTTGGAATAACAGTTCCTACATTAGCACAGAGTATTGGT
CACGTCAGCGGCTGCCACATCAACCCCGCAGTGACGCTGAGCCTTTTCGCAACAGGCAAC
ATAAAACTTCTCCGAGCTTTGCTCTTCATCGCCGTTCAACTCATCGGTGCTGTTGGAGGA
TCAGCAGTTTTAAGGTTGATGGTTCCTGAAGAAAAAGAACAC------------------
------------------------------TCCCTGGGCATCACAAACATAGGGCCAGGC
---------ATCACTGACGTCCAGGGTTTCCTGATGGAAATAATCTTAACATTTTTATTA
CTCTTTATCATCCACGCT---GTGTGCGATCCTCGCCGTAAA------------------
---GATATCAAA---------------GGATCGGCTCCTCTAGCTATAGGATTAGCCGTC
ACAGCTTGCCACCTCTGTGGTATCCCTTATTCCGGCTCAAGCGTAAACCCAGCTCGTAGT
TTCGGACCGGCTGTAATCATGGAT---------TCGTGGGAAAATCATTGGGTCTACTGG
GCTGGGCCGCTACTTGGCGGTGTGTTGGCTGGTCTCATCTACAAATATCTCTTCAAAACG
---AGAAAA------------------------------TCTGAC---------------
------------------------GTC------------GATTCGTAC------GATTTT

>BAM83568_Anomala_cuprea_Drip
ATGTCC--

------------AAGGATATG---------AAGGCT------------------------

---GCA
GAGAGTTACGTTGGCCTATCAGAATTAACA---GCAAGCACA------AATTTATGGAGG
GCACTATGTGCGGAATTCATAGGAACGTTTCTACTCGTCCTAATAGGTTGCGGAACTATT
CTACAGTTCAAACCAACGGCA---------------------------------------
---GCACCATCC
ATCGTTGCAATCTCGTTAGCATTTGGTTTAATAGTCGCCACATTAGCACAGGCTATCGGT
CACGTGAGTGGATGCCATGTAAATCCCGCTGTAACGCTAAGTCTCTTCGTAACTGGTGAT
TGCAAATTGATAAGGTCGTGCCTGTATATTGTGGTACAATGTTTGGGGGCAATGGCTGGA

TCTGCAATGTTAATGGTTGCAACACCGAGTGACTTCCAAGGT------------------
------------------------------AATCTCGGTGCAACTGCTCCACATGCAGAT
---------TTAGCACCTGCCCAAGCATTTTTCTACGAAGCTGTCCTCACATTTTTACTT
TGCTTCGTTATTCATGGA---GTATGCGATGCCAAACGAAAA------------------
---GACATTAAA---------------GGATCTGCTCCACTTGCAATTGGTTTAGCTATT
ACCGCCTGCCATTTAAGTGGAATAAAATACACTGGCTCCAGCATAAATCCCGCTAGGAGT
TTTGGCCCAGCTGTCATAAAGAAT---------AATTGGCACAATCATTGGATTTATTGG
GCTGGACCGATTATTGGTGGTCTCGTCGCTGGTCTTATCTACAAATTTATTTTTAAAGTT
---CCAAAG------------------------------GATGAA---------------
------------------------GAA------------GATTCAATG------GATTTT

>EHJ68353_Danaus_plexippus_Leip
ATGTCG--

------TTTCTCGGATTAGAGGAACTCAAG---GATTATGTA------ATGGTGTTAAAG
CAATTATTTGCGGAATTCTTAGGAACGTTTTTGTATTTGTCAATCACACTTCTAGCTGGA
TTAACTTCTAACAACAGCGTA---------------------------------------
---TCTTTCCATAGCCTG
GTGGTGGTAACGGCATTGTCTAATGGATTTTTAGTGGCATCCATCATCCAAGTAACAGGT
CATATATCTGGAGGACATATAAATCCAGCCGTCACTGTCGGGGTTCTGGCAAGTGGACGC
ATGAAGATTGCTAAGGGAATTCTGTATATAGGAGCGCAAATTCTGGGCAGCTTACTCGGA
GCGTTCGTAGCGTACGGTATTTCTGAGATTACTACAAGAGGC------------------
------------------------------AATCTCGGAGCCACTATTCCGTACAATGGA
---------TTAAGAGTAGACCAAGTCTTCGGTTTGGAGTTTCTCATGACCTTTATTTTA
GTTTCCGTAGTTTTGAGC---GTTAGTGATACTAACAAACCA------------------
---GCTGGCTTG---------------GGTTCTGGAGCTCTCGCTATAGGGATAAGCATT
GTAGCTTGTCAAAGTTCTGCGTTATTTTATAGTGCA---TCACTGAACCCAGTGAGATCT
TTGGGACCGGCTGTAATGATGAAT---------ATTTGGACTCATCACTGGGTTTTCTGG
ATAGGACCATTGTTAGGTGGTCTATGTGCTGGACTTATATATAGATTCATGTTGGTTTAT
---AACTCCGAT---ATGTTA---------------------------------------

>DVQQ01005194_Danaus_erippus_Leip
ATGTCG--

------TTTCTCGGATTAGAGGAACTCAAG---GATTATGTA------ATGGTGTTAAAG
CAATTATTTGCGGAATTCTTAGGAACGTTTTTGTACTTGTCAATCACACTTCTAGCTGGA
TTAACTTCTAACAACAGCGCA---------------------------------------
---TCTTTCCATAGCCTG
GTGGTGGTAACGGCATTGTCTAATGGATTTTTAGTGGCATCCATCATCCAAGTAACAGGT
CATATATCTGGAGGACATATAAATCCAGCCGTCACTGTCGGGGTTCTGGCAAGTGGACGC
ATGAAGATTGCTAAGGGAATTCTGTATATAGGAGCGCAAATTGTGGGCAGCTTATTCGGA
GCGTTCGTAGCGTACGGTATTTCTGAGATTACTACAAGAGGC------------------
------------------------------AATCTCGGAGCAACTATTCCGTACAATGGA
---------TTAAGAGTAGACCAAGTCTTCGGTTTGGAGTTTCTCATGACCTTTATTTTA
GTTTCCGTAGTTTTGAGC---GTTAGTGATACTAACAAACCA------------------
---GCTGGATTG---------------GGTTCTGGAGCTCTCGCTATAGGGATAAGCATT
GTAGCTTGTCAAAGTTCTGCGTTATTTTATAGTGCA---TCACTGAACCCAGTGAGATCT
TTGGGACCGGCTGTAATGATGAAT---------ATTTGGACTCATCACTGGGTTTTCTGG
ATAGGACCATTGTTAGGTGGTCTATGTGCTGGACTTGTATATAGATTCATGTTGGTTTAT
---CACTCCGAT---ATGTTA---------------------------------------

>DVQP01034203_Danaus_eresimus_Leip
ATGTCG--

------TTTCTCGGATTGGAGGAACTAAAA---GACTACGTA------AAGGTGTTAAAG
CAATTATTTGCAGAATTCATAGGAACGTTCTTGTACTTGTCGATCACGCTTCTGGCTGGA
TTAACTTCTAACAACAATGTA---------------------------------------
---TCTTGGCATAGCCTG

GTGGTGGTAACGGCCTTGTCTAATGGATTTTTAGTGGCATCCATCATCCAAGTAACAGGT
CATGTATCTGGAGGGCATATAAATCCAGCAGTCACTGTCGGGGTTCTGGCATGTGGTCGC
ATGAAAATTGCTAAAGGAATTCTGTATATACTAGCCCAAATTCTGGGAAGCTTATTTGGA
GCGTTCGTAGCGTACGGTATTTCTGAGATTTCTACAAGAGGC------------------
------------------------------AATCTTGGAGCAACAATTCCGTACAATGGA
---------TTAAGAGTAGACCAAGTCTTCGGTTTGGAGTTTCTTATGACTTTTATTTA
GTTTCCGTAGTTTTGAGC---GTTATTGACACTAACAAACCC------------------
---ACTGGCTTG---------------GGCTCTGGAGCTCTCGCTATAGGAATAAGCATT
GTAGCTTGCCAAAGTTCTGCGTTATTTTACAGTGCA---TCACTGAATCCAGTGAGATCT
TTGGCACCAGCTGTTATGATGAAT---------ATTTGGACGTATCACTGGGTTTTTGG
ATAGGACCATTGCTAGGCGGTCTTTGTGCCGGACTAGTATATAGATTTATGTTAACTTAT
---AATTCTGAT---ATGTTA---------------------------------------

>CAKASE010000081_Danaus_chrysippus_Leip
ATGTCG--

------CTTCTCGGACTAGAGGAACTAAAA---GATTACGTA------AAGGTGTTGAAG
CAATTATTTGCAGAATTCTTAGGAACATTTTTGTACTTATCAATCACACTTCTAGCTGGA
TTGACTTCTAACAACAGTGTA---------------------------------------
---TCTTGGCATAGCCTG
GTAGTGGTTACAGCGTTGTCTAATGGATTTTTAGTGGCATCTATTATCCAAGTAACAGGT
CATATATCTGGAGGACATATAAACCCAGCAGTTACTGTCGGAGTTCTGGCATGTGGACGC
ATGAAGATAGCCAAGGGAATTCTGTATATATCAGCGCAAATTCTGGGCAGTCTATTTGGC
GCATTTGTGGCGTATGGTATTTCCGAGATTTCTACAAGAGGC------------------
------------------------------AATCTTGGAGCAACTATTCCGTACAATGGA
---------TTAAGAGTAGACCAAGTCTTCGGTTTGGAGTTTCTTATGACTTTTATTTTA
GTTTCCGTAGTTTTGAGC---GTGATTGATACTAATAAACCA------------------
---ACCGGCTTG---------------GGCTCTGGAGCTCTCGCTATAGGAATAAGTATT
GTAGCCTGTCAAAGTTCTGCATTATTTTATAGTGCA---TCACTGAACCCAGTGAGATCT
TTGGGACCAGCTGTGATGATGAAT---------ATTTGGTCGCATCACTGGGTTTTCTGG
ATAGGACCACTGTTAGGTGGTCTATGTGCTGGACTAGTATATAGATTTATGTTGGTTTAT
---AACTCTGAT---ATG--

>DWQU01003080_Danaus_melanippus_Leip
ATGTCG--

------TTTCACGGTTTAGAGGAACTAAAG---GATTACGTA------AAGGTGTTGAAG
CAATTATTTGCAGAATTCTTAGGAACGTTTTTGTACTTGTCAATCACGCTTTTAGCTGGA
TTAACTTCTAACAACAGCGAA---------------------------------------
---TCTCTGCATAGCACG
GTACTGGTAAAAGCATTGTCTAGTGGATTTTTAGTGGCATCCATCATCCAAGTAATAGGT
CATATATCTGGAGGACATATAAATCCAGCCGTCACTCTCGGGGTTTTGGCACATGGACGC
ATGAAGATTGCTAAGGGAATTCTGTATATATTAGCGCAAATTCTGGGGAGTTTATTTGGA
GCGTTCGTAGCGTATTTTTTTTCTGAGACTTCTACAAGAGGC------------------
------------------------------AATCTTGGAGCAACTATCCCATACAATGGG
---------TTAAGAGTCGGCCAAGTTCTCGGTTTGGAGTTTATAACGACTTTCATTTTA
GTTTCTGTAGTTCTGAGC---GTCATTGATACTAAAAAACCA------------------
---ACTGGCTTG---------------GGCTCTGGATCTCTCGCTATAGGAATATGCATT
GTAGCTTGTCAATGTTCTGCATTATTGTATACTGCA---TCAATGAACCCAGTGAGATCT
TCTGGCCCAGCTGTAATGATGAAT---------ATCTGGACGCATCACTGGGTTTTTTGG
GTAGGACCATTATTAGGTGGTCTATGTGCTGGACTAGTATATAGATTTTTGCTGGTTTAT
---CACTCTGAT---ATG--

>DVSA01041710_Junonia_nigrosuffusa_Leip
ATGTCT--

------ATTCTCGGTTTAGAAGATATAAAG---AACTACAAA------ACCCTTTTAAAA

```
CAACTGTTTGCTGAATTCCTCGGGACCTTCCTCTATCTATCCATTTCCCTGTTGGCTGAA
---ACG---AAT------------------------------------------------
------------------------------------------------CTTGGAAGAAGC
GTTGTGACCACGGCATTGGCTAGTGGACTGCTAATTGCATCTATCGTCCAAATGTTTGGC
CATATATCTGGAGGCCACGTCAACCCAGCTGTTACGATTGGCGTTTTAATTTGTGGACAT
ATCACGGTGCTGAAAGCAATCTTCTATATAATTGTCCAAATTCTTGGAAGTGTGCTTGGA
TCGGCTGTCGCTTATGGGTTAACAGATGATGCTTCGAGAGAT------------------
------------------------------GGTCTTGGAGCTACCGTTCCTTACAATAAT
---------ATGAGAGCTGACCAAGTATTTGGATTGGAATTTCTTATGACCTTTATTTTA
GTTGGTGTTGTTTTAAGT---GTTAGCGACGATAGGAAGAAA------------------
---AGTGGCGTA---------------GCTTCTGCGTCATTGGCAATAGGGTTAAGTATA
ACTGCTTGTCAAAGTGCTGCAGTTTTGTATAGTGGT---TCATTGAACCCTGTCAGGTCT
CTTGGACCGGCTGTTGTAATGAAT---------GTCTGGATCCATCATTGGGTGTATTGG
GTTGGACCAATTTTGGGTGGGTTAGCTTCAGGTATTATTTACAGATTTGTTTTT------
------------------------------------------------------------
------------------------------------------------------------
------------------------------------------------------------
------------------------------------------------------------
------------------------------------------------------------
------------------------------------------------------------
------------------------------------------------------------
------------------------------------------------------------
------------------------------------------------------------
------------------------------------------------------------
------------------------------------------------------------
------------------------------------------------------------
------------------------------------------------------------
------------------------------------------------------------
------------------------------------------------------------
------------------------------------------------------------
------------------------------------------------------------
------------------------------------------------------------
------------------------------------------------------------
------------------------------------------------------------
------------------------------------------------------------
------------------------------------------------------------
------------------------------------------------------------
------------------------------------------------------------
------------------------------------------------------------
------------------------------------------------------------
------------------------------------------------------------
------------------------------------------------------------
------------------------------------------------------------
------------------------------------------------------------
------------------------------------------------------------
------------------------------------------------------------
------------------------------------------------------------
------------------------------------------------------------
```


>DVUL01069533_Junonia_evarete_oscura_Leip
ATGTCT--

------ATTCTCGGTTTAGAAGATATAAAG---AACTACAAA------ACCCTTTTAAAA
CAACTGTTTGCTGAATTCCTCGGAACCTTCCTCTATCTATCCATTTCCCTATTGGCTGAA
---ACG---AAT--
--CTTGGAAGAAGC
GTTGTGACCACGGCATTGGCTAATGGACTGCTAATTGCATCTATCGTCCAAATGATTGGC
CACATATCTGGAGGCCACGTCAACCCAGCTGTTACGATTGGCGTTTTAATTTGTGGACAT
ATCACGGTGCTGAAAGCAATCTTCTATATAATTGTCCAAATTCTTGGAAGTCTGCTTGGA
TCGGCTGTCGCTTATGGGTTAACAAATGATGCTTCGAGAGAT------------------
------------------------------GGTCTTGGAGCTACCGTTCCTTACAATAAT
---------ATGAGACCTGACCAAGTATTTGGATTGGAATTTCTCATGACCTTTATTTA
GTTGGTGTTGTTTTAAGT---GTTAACGACGATAGAAAA---------------------
---AGTGGCGTA---------------GGTTCTGCGTCATTGGCAATAGGATTAAGTATA
ACTGCCTGTCAATGTTCTGCAGTTTTGTATAGTGGT---TCATTGAACCCTGTCAGGTCT
CTTGGACCTGCAGTTATTATGAAT---------GTCTGGATCCATCATTGGGTATACTGG
GTTGGACCAATTTTGGGTGGGTTAGCTGCGGGTATTATTTACAGATTTGTTTTT------

>DWQF01062434_Charaxes_varanes_Leip
ATGTCT--

------ATTCTAGGAACAAAAGAAATTAAG---TTTTACAGA------ACAACAGGCCAG
CAGCTTTTTGCTGAATTTATTGGAACTTTTTTGTATTTGTCCATAACGTTGATAGCAGGA
---ACA---GAT--
--CTTAATAAGAGT
GTAGTTGCAACAGCGTTATCGAATGGACTTCTAGTTGCCTCGGTGATCCAAATTCTCGGA
CATATATCTGGAGGCCATATTAATCCAGCCGTAACCGTCGGTGTTTTAGCATGCGGGAAA
ATAAAATTGATTAAAGCAGGTCTATATATTATTGCACAAATTCTTGGCTGTCTGCTTGGA
GCATTGGTGTCATATGGACTAACTGATATCACTACAAGAAGA------------------
------------------------------TACCTTGGAGCCACCATTCCTCACCCCAAT
---------TTAAATTGTGGTCAAGTATTTGGATTGGAATTTCTTATGACCTTTATTTTG
GTTGCAGTAGTGTTAAGT---GTTTTGGATACAACCAGGCCT------------------
---AGAGGCCTT---------------GGTTCAGGTCCTTTAGCGATTGGAATAAGTATA
ACAGCATGTCAGTGTTCTGCTGTATTATATACTGGA---TCTCTGAATCCTGTACGGTCT
TTGGGCCCAGCTGTGGTTATGAAT---------ATTTGGACTGATCATTGGGTATACTGG
GTTGCCCCACTTCTAGGAGGGCTTACAGCAGGCTTTATTTACCGATATATCTTCACACTT

---CGCTATGAAGAAAATGTAGTA---------------AAT------------------

>DVOX01146676_Maniola_jurtina_Leip
ATGGCA--

------ATATTTGGTCAGGAAGAAATAAAA---GACTACAAA------ACAACTCTGCAA
CAAGTCTTTGCCGAATTCCTGGGCACATTCTTGTATTTGTCAATATGTTTGCTAGCTGGG
---ACC---AAC--
--GTTGAACCAAAC
ATTGAAGCGATAGCGTTGGCAAATGGTCTCCTTGTTGCTTCAGTTGTCCAAATCATTGGA
CAAATATCCGGAGGGCATATCAACCCAGCTGTTACTATTGGTGTTTTTGCACGTGGACAT
ATTAAATTGCTCAAAGCAATTCTCTATATAATTGCCCAAATCCTTGGCAGCCTGCTTGGA
GCATTATTGGCATATGCAGTCACTCATACCAACTTAAGACAA------------------
------------------------------GATCTTGGAGCAACTGTACCATATAAAGAT
---------TTGAGAGCAGACCAAGTCGTTGGACTTGAATTTATTATGACGTTTATATTA
GTTTCTGTTGTTTTAAGT---GTTTTAGATACGAGAAGATCT------------------
---AGAGGACTC---------------GGATCTGGCCCACTGGCTATTGGTTTGAGTATA
ACAGCTTGCCAAGTGTCTGCATTTTTGTACACTGGA---TCATTAAATCCAGTACGATCT
TTGGGACCAGCAGTGGTGATGAAT---------ATTTGGACCAAACATTGGGTGTATTGG
CTTGGGCCAATTCTTGGAGGTGCGGCTGCTGGCTTAATTTACAGATTTGTTTTGGCCGAT
---CGTTACCGGAGAAGTGATGAA---------------AAC------------------

>SGBE01000536_Hypolimnas_misippus_Leip
ATGAAT--

------GTTCTTGGTTTAGAAGATATCAAA---AACTATAAA------ACTCTCTTAAGG
CAACTTTTCGCTGAATTCCTCGGAACATTCCTCTATTTATCCATAACCCTATTGGCTGAA
---ACA---AAT--
--CTTGGAAAGAGT
GTTGTAACCACGGCATTGGCTAATGGACTGCTAATTGCGTCTATCGTCCAAGTGATTGGC
CATATATCTGGAGGGCACATCAATCCAGCCGTGACAATTGGTGTTTTAGCTTGTGGACAC
ATCACGATACTGAAAGCGATCTTTTATATAATCGTCCAAATTATTGGGAGCCTGCTTGGA
TCAGTTGTCGCTTATGGTTTGACTGATGATGTCTCAAGGAAT------------------
------------------------------GACCTTGGAGCTACCGTCCCGTACCATAAC
---------ATGAGAGCAGACCAAGTATTTGGATTGGAGTTTATTATGACCTTCATTTTA
GTTGCTGTCGTTTTAAGC---GTTAGTGACAATAGAAAG---------------------
---AATGGCTTA---------------GGTTCAGCCTCATTAGCGATAGGGTTAAGTGTG
ACCGCTTGCCAGAGTGCTGCAGTTTTATACAGCGGT---TCGTTAAACCCTGTGAGGTCT
CTGGGACCGGCTGTTGTGATGAAT---------ATCTGGATCCATCATTGGGTGTACTGG
ATTGGACCGATTTTGGGAGGGGTAACTGCGGGAATTATATATAAGTTTGTTTTTAACATT
---ACACCTGATAGAAACATAAAT---------------GTT------------------

>DWNB01092036_Pararge_aegeria_Leip
ATGTCA--

------ATACTCGGTCAAGAAGAAATCAAA---GACTACAAA------ACGATACTAAAG
CAACTGTTTGCAGAATTCCTGGGAACATTTCTGTATATTTCCATATGTCTATTAGCTGGA
---ACA---AAC--
--ATTGAACCAAAT
GTTGTAGCGGTAGCACTGGCCAACGGACTCCTAATCGCGTCAGTTGTCCAAATAATTGGA
CAAATATCTGGAGGACATATCAACCCTGCTGTAACAGTAGGTGTTTTTGCTTGCGGACGT
ATTAAATTGATGAAAGCATTTCTCTACATCATTGCCCAAATCCTCGGCAGTTTGATTGGA
TCAACAGTGGCGTATGCTATCACAGACAGCGACTCCAGACAC------------------
------------------------------GAGCTGGGAGCAACTGTTCCCCGTAAAGAA
---------CTAAGGGCCGACCAAGTTTTCGGACTTGAATTTATAATGACGTTCGTATTA
GTCGCAGTTGTTTTAAGT---GTGCTGGATACGAAACGATCT------------------
---AGAGGACTT---------------GGTTCCGCACCCTTGGCGATTGGTTTGAGCGTA

ACTGCCTGCCAAGTCTCCGCATTTTTGTATACAGGA---TCGCTTAATCCGGTGAGATCC
TTGGGGCCGGCGGTGGTTATGAAT---------ATTTGGACCAAACATTGGGTTTACTGG
ATTGGACCAATTCTAGGAGGTTTGACTGCTGGTTTAAATTACAGATTTATTTTTGGAAAG
---CATTTTTACAACGGCGAGAAC---GATTTTACAGAGGGAGAA---------------

>XM_004926772_Bombyx_mori_Leip

```
ATGAGT------------------------------------------------------
------------------------------------------------------------
------------------------------------------ACAAAAGCA---------
------------------------------------------------------------
------------------------------------------------------AGAATC
GGCACAATTATAGGCATAAACGAGTTAAAA---AACTATCAT------GTGCTGGCGCGA
CAAATGCTCTCAGAATTTCTGGGAACTTTTTTTGTATTTATCCTTAATTTTGCTCACGGT
---ACT---AGT------------------------------------------------
------------------------------------------------TTGAATCAAGGC
CCGACTGGCAGTGCCTTGGCGAACGGCCTTCTCATTTCCAGCATTATCCAGTTCACTGGC
CATATCTCTGGTGGGCATTTGAACCCGGCTATCACCTTTGGGGTGATGGCCAACGGTCAA
ATTACGATGGTCAGAGGTGCTTGTTATATCGCAGCGCAGATACTTGGCTCCATAGTAGGT
TCATCAGTGGCTTATGCCGTCACAGTATCTTCATTAAGGAGT------------------
------------------------------ACGCTGGGTACGACTGTTCCGAATCCAAAT
---------TTAAGAGCAGAACAAATCTTCGCCATAGAATTTTTGATCACATTCATTTTA
GTGGCGGTCGTGTTAAGC---GTTATCGACCCAAACCGAGGA------------------
---GCTGGCATC---------------GGCTCTGGTGCTTTCGCTATTGGCCTCAGTATT
ACCGGCTGCCAATGTTCTTGTCTACCTTACACTGGA---ACTCTGAATCCCATCAGGTCT
TTAGGACCCGCTGTATTAATGAAT---------TCGTGGGATTCCCATTGGGTGTACTGG
GTTGGGCCAATTTCGGGTGGCGTAGCCGCTGGTTTGGTCTACAGGTTTTTGTTAAGACCC
---AATTGGAAGAACGTTGGCAAT------TAT---GAAGAA------------------
------------------------------------------------------------
------------------------------------------------------------
------------------------------------------------------------
------------------------------------------------------------
------------------------------------------------------------
------------------------------------------------------------
------------------------------------------------------------
------------------------------------------------------------
------------------------------------------------------------
------------------------------------------------------------
------------------------------------------------------------
------------------------------------------------------------
------------------------------------------------------------
------------------------------------------------------------
------------------------------------------------------------
------------------------------------------------------------
------------------------------------------------------------
------------------------------------------------------------
------------------------------------------------------------
------------------------------------------------------------
------------------------------------------------------------
------------------------------------------------------------
------------------------------------------------------------
------------------------------------------------------------
------------------------------------------------------------
------------------------------------------------------------
------------------------------------------------------------
```


>XM_028173193_Bombyx_mandarina_Leip
ATGAGT--

--ACAAAAGCA---------

--AGAATC
GGCACAATTATAGGCATAAACGAGTTAAAA---AACTATCAT------GTGCTGGCGCGA
CAAATGCTCTCAGAATTTCTGGGAACTTTTTTGTATTTATCCTTAATTTTGCTCACGGGT
---ACT---AGT--
--TTGAATCAAGGC
CCGACTGGCAGTGCCTTGGCAAACGGCCTTCTCATTTCCAGCATTATCCAGTTCACTGGC
CATATCTCTGGTGGGCATTTGAACCCGGCTATCACCTTTGGGGTGATGGCCAACGGTCAA
ATTACGATGGTCAGAGGTGCTTGTTATATCGCAGCGCAGATACTTGGCTCCATAGTAGGT
TCATCAGTGGCTTATGCCGTCACAGTATCTTCATTAAGGAGT------------------
------------------------------ACGCTGGGTACGACTGTTCCGAATCCAAAT
---------TTAAGAGCAGAACAAATCTTCGCTATAGAATTTTTGATCACATTCATTTTA
GTGGCGGTCGTGTTAAGC---GTTATCGACCCAAACCGAGGA------------------
---GCTGGCATC---------------GGCTCTGGTGCTTTCGCTATTGGCCTCAGTATT
ACCGGCTGCCAATGTTCTTGTCTACCTTACACTGGA---ATTCTGAATCCCATCAGGTCT
TTAGGACCCGCTGTATTAATGAAT---------TCGTGGGATTCCCATTGGGTGTACTGG
GTTGGGCCAATTTCGGGTGGCGTAGCCGCTGGTTTGGTCTACAGGTTTTTGTTAAGACCC
---AATTGGAAGAACGTTGGCAAT------TAT---GAAGAA------------------

>FBpp0087236_Drosophila_melanogaster_Prip
ATGGGA--

---AAA------------

---TTC
GAATACTCACTGGGTCTCAATGAGCTCAAA---TCCAAGGAG---CTGCGATTGTGGCAG
GCCTTGATTGGAGAATTCCTTGGCAATTTGATCCTCAATTTCTTTGCCTGCGGCGCATGC
ACCCAAATTGAG--
---GAT
GGCACCTTCAAGGCCTTGGCTTTTGGCCTGGCTATCTTCATGGCCATCACGATTGTGGGC
CACTTGAGTGGTGGTCATGTGAATCCTGCCGTTACCGCTGGAATGTTGGTCGCAGGACGA
ATTAGCTTGATCAGAGCCTTTTTCTATGTGGTTTTCCAGTGCTTGGGTGCAATTGCCGGA
ACTGCAGCGGTTAAGATTCTCATCGATCAGGACTATTACAAT------------------
------------------------------GGCCTGGGTCACACCTCTCTGGCGCCCAAT

---------ATCACCGAGCTGCAGGGCCTGGGAATCGAGTTCTTCCTGGGACTCCTGCTG
GTGCTCGTCGTCTTTGGG---GCCTGTGATCCCCACAAGCCC------------------
---GATTCCCGC----------------TACACGGCGCCCTTGGCCATCGGAATGGCGGTG
ACCCTGGGTCACTTGGGCACCATCCGCTACACCGGGGCCAGCATGAATCCCGCTCGCACC
GTGGGCACCGCCTTTGCCACCGAC---------ATCTGGGCGTCGCATTGGGTATACTGG
GTGGGACCTGTGCTGGGGGGCGTGGCTGCCGCCCTGCTCTACACCCAGGTCCTGGAGGCG
---AAGCCCGTGCCA-------------------AAGGTTAACGAGGCATCCGAG------
------------------------AAG------------TACCGAACG------CACGCC
---GAT---GAA---CGCGAGATGCGCAAACTG---------GAG---------GGA---
---GCCCGTGAC------
TACGCC--

>FBpp0155293_Drosophila_grimshawi_Prip
ATGGGC--

---AAA------------

---TAC
GAGTACTCGCTGGGCCTCAATGAATTCAAA---TCGAAGGAT---TTGCGTCTGTGGCAG
GCGCTCATCGCCGAATTCATTGGCAATTTGATATTGAACTTTTTCGCCTGTGGCGCCTGC
ACCCAGCAGGAG--
---GAT
GGCACCTTTAAGGCTCTCGCCTTTGGACTGGGCGTCTTCATGGCCATTACGATCATCGGC
CATTTGAGTGGAGGTCATGTTAATCCTGCTGTTACCGTTGCCATGTTGGTAGCTGGCAGG
ATTAGCGTCCTTCGCGCTGTCTTCTACATTGTGTTCCAGTGTCTGGGCGCCATTGCCGGC
ACAGCATGTGTCAAGATCCTGCTGGACTCGAACTATCACAAC------------------
------------------------------GGATTGGGCCACACTAATCTGGCCCCAAAT
---------ATCAGTGAGCTCCAGGGACTGGGCATTGAGTTCTTCCTCGGCTTGGTGCTG
GTGTTGACCGTGTTCGGT---GCCTGCGATGGCCACAAACCG------------------
---GACTCGCGA---------------TACACTGCTCCGCTGGCCATTGGCATGTCGGTC
ACATTGGGTCATCTGGGCACAATTCACTATACGGGCGCCAGCATGAATCCGGCTCGTACA
CTGGGCACCGCCTTTGCCACCGAC---------AACTGGAACGCCCATTGGGTGTACTGG
GTGGGACCGGTGCTGGGCGGTGTGGCAGCTGCTCTGCTCTACACCCAGGTGCTGGAGGCT
---AAGGCGCCAGCG------------------AAGAGCAACGAAACCGCTGAG------
------------------------AAA------------TATCGCACA------CATGCC
---GAC---GAA---CGTGAGCTGCGCAAACTG---------GAA---------GGA---
---TCTCGCGAC------
TTTGCC--

>ACT34032_Eurosta_solidaginis_Prip
ATGCCG--

--CAAAAA------------

---TTT
GATTATTCACTTGGCCTACACGAGCTAAAG---TCAAAAGAA---CATCGCCTATGGCAA
GCGCTCGTCGCTGAATTTTTAGGAAACTTTTTATTGAATTTCTTTGCTTGCGGTGCGTGT
ACCCAACCCGAG--
---GGC
GGTACATTTAAGGCGTTGGCATTTGGGTTGGCTGTTTTCATAGCGATCACGGTTATTGGA
AATATAAGCGGCGGTCACGTAAATCCTGCCGTAACAATAGGCTTATTAGTGGCGGGGCGT
GTAACCGTTTTGCGTGCCGTTTGTTATATAATATTCCAATGTCTGGGTTCCATCGCCGGT
ACAGCGGCTATTCGTACACTCATAGATGAGGAGTATTACGGT------------------
------------------------------GGCTTAGGTCATACACATCTGGCGCCTAAT
---------ATTACCGAACTACAAGGCCTCGGTATTGAATTCTTTTTGGGCTTGGTCTTG
GTGCTCACAGTTTTTGGT---GCTCTTGATGCCAATAAACCA------------------
---GATTCTCGT---------------TTCACTGCGCCACTCGCCATCGGTCTCTCTGTC
ACCCTCGGTCATTTGGGCACAATTCGTTATACCGGTGCAAGCATGAATCCAGCACGTACA
CTCGGCACAGCCTTTGCTGTACAC---------AATTGGGATGCACACTGGGTCTACTGG
ATAGGTCCAATTATGGGCGGTATCGCAGCAGCGCTCATCTACACACAAATCATAGAGAAG
---CCACTCGTACACACGGCCGTC---------AAGGTGATTGAAGTATCCGAG------
------------------------AAA------------TATCGAACA------CACGCT
---GAT---GAT---CGTGAGATGAGAAAGCTG---------GAT---------AGT---
---ACACGCGAT------
TACGCT--

>ADD19396_Glossina_morsitans_morsitans_Prip
ATGGCT--

--GGAAAA------------

---TTT
GAATACTCGCTTGGTATAAATGAACTGAAA---TCAAAAGAT---TACCGTTTATGGCAA
TCTTTGGTTGGCGAATTTCTTGGAAATTTAATATTGAATTTTTTCGCTTGTGGAGCATGC
ACGCAACCTGAG--
---GAT
GGTACATTTAAGGCTTTGGCATTTGGCTTGGGAGTCTTTATGGCAATCACAGTTGTCGGG
CACTTAAGTGGAGGGCACGTAAACCCTGCTGTCACACTGGGTATGCTGGTAGCTGGTCGA

GTCAGTTTATTGCGAGCAATCTTGTACGTAATATTTCAGTGTCTGGGGTCAATAGCGGGT
ACTGCCGCCATTAGGACACTCATCGATGAGGTCTACTACAAT------------------
------------------------------GGTTTGGGTCATACAAGTCTGGCCCCGAAT
---------ATAACAGAATTGCAAGGTGTCGGAATTGAATTTTTCCTTGGTTTGGTTTTA
GTTTTAACAGTTTTTGGA---GCTTGTGATGCCAATAAACCA------------------
---GATTCGCGC----------------TACATCGCCCCGCTGGCTATCGGTATGGCCGTT
ACTTTGGGTCATTTGGGCACAATACGTTATACTGGAGCTAGCATGAATCCTGCCCGCACT
GTCGGTACTGCTTTTGCGACCAAC---------AACTGGGATTCGCATTGGGTCTACTGG
GTTGGTCCAGTTTTGGGTGGCATCGCAGCTGCTTTGATTTACACTCAAATTCTTGAAAAA
---CCAGCCGCTAAG---

---------------------ATGAGAAAATTG---------GAA---------AGC---
---GGACGTGAC------
TATGCT--

>AAEL003550_Aedes_aegypti_Prip
ATGGGG--

---ACT
TCGTATAAGCTTGGAATGGACGAACTTTCC---GGTCGGTCTTCCAACAGCATATGGAAG
GCGCTGGTCGCTGAATTCATTGGTATCTTCATCCTGAACTTTTTTGGCTGCGCTGCATGT
ACTCATGCAGCT---GGA--
---GAT
AAAACACTCATTTCACTTGCTTTTGGTCTGAGTGTATTCATGGTTGTGATGTCTATCGGG
CACATTAGCGGTGGACACATCAATCCTGCAGTAACTGCTGGCTTACTTGCGGCTGGCAAA
GTCAGCATTGTTCGAGCACTTTTGTACGTCGTAGCTCAATGTGCCGGAGCAGTGGCTGGA
ACTGCTTCACTAAAGGCACTTTTACCGGAAGCATACCAGAAT------------------
------------------------------GGTCTTGGCAACACAGGCCTTAAAGAAAAC
---------GTCCAAGATATGCAAGGATTGGGAATTGAGTTCTTCCTAGGTTTCATCCTG
GTACTGTGCGTTTTCGGA---GTTTGCGATGAGAACAAACCA------------------
---GATTCACGC---------------TTTGTTGCCCCATTGGCTATCGGCATGACGGTT
ACTTTGGGTCATCTCGGAGTTGTAGAGTATACCGGTTCCAGCATGAATCCCGCTCGCTCG
TTTGGAACGGCATTTATCGGTGAT---------AATTGGGCTAATCATTGGATCTATTGG
GCTGGACCTATTTTGGGCGGTATTTGTGCTTCTCTTCTGTATTGCCAAGTGTTCAAAGCA
---CCTCAACCAGAAGGAGAA---------------------TCCGCCTCAGAG------
------------------------CGC------------TACCGTGTA------GCTGCT
---GAC---GAG---AAAGAGCTAAAACGTTTG---------GAT---------GGA---
---AAACGTGAT------
ATGGCC--

>AB523397_Anopheles_gambiae_Prip
ATGGGA--

---TACAGCCTAGGAACGGAGGAGCTTTCC---AGCAAGTCGAGC---GGACTGTGGCGT
GCGCTGCTGGCCGAGTTTGTAGGCATTTTCATACTGAATTTCTTCGCTTGCGCCGCCTGT
ACGCATGCGAAT---GGA--
---GAT
AAAACGCTGATCTCGCTGGCATTCGGGCTGAGCGTGTTCATGGTGGTGATGTCGATCGGA
CACATCAGCGGTGGTCACATCAATCCCGCCGTGACGGCTGGAATGTTGGCCGCGGGCAAG
GTTAGCCTGATCCGTGCGCTGATGTACGTTGCGGCCCAGTGTGCCGGTGCCGTTGCGGCC
ACCACCGCCCTCGATGTGCTGATTCCGAAAAGCTTCCAGAAC------------------
------------------------------GGGCTCGGCAACACCGGACTGAAGGAGGGC
---------GTCACCGACATGCAGGGGCTCGGGTTCGAGTTCTTCCTCGGCTTCGTGCTG
GTGCTGTGCGTGTTTGGC---GTCTGTGACGAGAACAAACCG------------------
---GACTCGCGC---------------TTTGTGGCACCGCTAGCCATCGGGCTAACGGTT
ACGCTCGGCCATCTGGGCGTGGTGGAGTACACTGGGTCAAGCATGAACCCGGCCCGTTCC
TTCGGCACCGCATTCGTTACGGAC---------AGCTGGGCCCATCACTGGATCTACTGG
GCTGGACCGATCCTGGGCGGTGTGACGGCGGCTCTGCTCTACTGTCAACTGTTCAAGGCC
---CCGACCGCCAGC---------------------------GATGCTTCCGAG------
------------------------CGC------------TACCGTACC------AGCGCG
---GAC---GAT---AAGGAGATGCGTCGACTG---------GAC---------GGC---
---AAGCATGAT------
ATGGCG--

>BAF62090_Polypedilum_vanderplanki_Prip
ATGGCG--

---TTT
AAGTATTCATTAGGTGCAGACGAGCTTAAA---GGAAAAACAGGCACCAGTTTGTACAAG
GCAATTTTCGCAGAATTCTTTGGAATATTTATACTCAACTTTTTTGGTTGTGCAGCTTGC
ACTCATGCCAAA---GGC--

---GAT
GAAGTTCTGATTGCACTTGCTTTTGGTCTCAGTGTTTTTATGGCAGCAATGACCATTGGC
CACGTGTCTGGATGCCACATTAATCCTGCTGTAACTTTCGGCCTCTTAGCAGCCGGAAAA
ATTTCACTTATTCGTGCCATTTTCTATGTTTTGGCTCAATGTGTAGGTAGTGTTGCTGGT
ACAGCTTCATTAGCCGTTCTTACAAATACAGAAATTGCAATC------------------
------------------------------GGTATCGGACATACTCAATTAAATCCAACT
---------GTTTCCGTATATCAAGGATTAGGATTCGAATTTTTCTTGGGTTTCATTTTA
ATTCTCTGCGTGGTTGGT---GTTTGTGATGAAAACAAACCG------------------
---GATTCACGT---------------TTTATTGCACCATTGGCTATCGGTCTCACTGTA
ACTCTTGGACATCTTGGAGTTGTTACTTATACAGGTTCATCAATGAATCCAGCAAGAAGT
TTTGGAACAGCTTTCATTACAGGC---------GACTGGGAGAATCACTGGGTGTATTGG
CTTGGACCAATTGCTGGTGGTATTGCTGCCAGTCTTTTATACTCAATTTTCTTCTCTGCA
---CCCGATATTGAAGTA------------------------CATCGTTCTGAC------
------------------------AAA------------TACAGACAA---GTTACACAG
AATGAC---GAT---AAAGAATTACGCACATTG---------AGC---------GCT---

>GAFU01006086_Spodoptera_exigua_Prip
ATGGGT--

---GAA------------

---CTA
GGAGCTAAGCTTGGTCTCGATGAGCTGACT---GGAGGAGCT---GCCGGCATCAGCAAG
GCACTGCTGGCAGAGTTCATCGGTAATCTGTTACTGAACCTGTTCGGATGCGGCGCCTGT
ATCAACATCATTCAAGGCTCC---------------------------------------
---AGTGCTCCTCCCAAC
ATAGTGCTTATCGCCCTTGCCTTCGGTCTGGCGGTGTTTGCTAGTGTCTCGGCGATAGGA
CACATCTCAGGTGGCCACATCAACCCCGCCGTGACTCTAGGTCTGGCGTCAACGGGTCGT
GTTAAGATTGCGCGTGCGATTCTGTACATCATAGCGCAGTGCGCAGGCGCGGCCGCTGGA
TCTGGCCTGCTGAAAGCCCTTACTCCCGATCGAGCGGCTGGT------------------
------------------------------ACCCTCGGCACCACCGGTCTCGGCGCTGAC
---------GTCACTCCCTTGCAAGGCTTCGGAATCGAATTCTTCCTAGGATTCGTGCTT
ATCTTTGTCGTGTGTGGG---GTATGTGACCCTAATAAACCT------------------
---GACAGCAAA---------------GCCACCGCGCCTCTGGCTATTGGCCTAACAGTG
ACGCTAGGCCACTTGCTTGCTATCGACTACACCGGCTCGGCCATGAACCCTGCTCGTTCC
TTCGGTTCAGCTCTCGTCGCCAAC---------GTGTGGACTGACCATTGGGTATACTGG
GCGGGTCCTATCGCCGGTGGCATCGCAGCTGCACTACTGTACGTTCACGGATTCTCAGCA
---CCCGCTGTA---AGC------------------------ACGCCC---CCG------
------------------------CGC------------TACCGCTCC------GTCGCC
GGAGAC---GAG---AAGGAGCTGAAGAGGCTC---------GAC---------GGT---
---AAAGGCGAAGAC---
ATGGCT--

>EZ981212_Spodoptera_littoralis_Prip
ATGGGT--

---GAA------------

---CTA
GGAGCGAAACTTGGTCTCGATGAGCTGACT---GGAGGAGCT---GCCGGCATCAGCAAG
GCATTGCTGGCGGAGTTTATCGGTAATCTGCTGCTGAACCTGTTCGGTTGCGGCGCTTGT
ATCAACATCATACAAGGCTCC---------------------------------------
---AGTGCTCCTCCCAAC
ATCGTGCTCATCGCCCTTGCCTTTGGTCTGGCGGTGTTCGCCAGTGTATCAGCTATAGGA
CACATCTCAGGTGGCCACATCAACCCAGCCGTGACCCTAGGTCTGGCTTCAACGGGTCGT
GTGAAGATTGCGCGTGCGATTCTGTACATCATAGCGCAGTGCGCAGGCGCGGCTGCTGGC
TCTGGTCTGCTAAAGGCTCTCACTCCTGACCGAGCGGCTGGA------------------
------------------------------ACCCTCGGGACCACCGGCCTCGGCGCTGAC
---------GTCACTGCACTGCAAGGCTTTGGAATTGAATTCTTCCTCGGATTCGTTCTC
ATCTTCGTCGTGTGTGGG---GTATGTGACCCTAACAAGCCT------------------
---GACAGCAAG---------------GCCACAGCTCCCCTGGCCATTGGCCTGACGGTG
ACGCTCGGCCACTTGCTCGCTATCGACTACACCGGCTCTGCCATGAACCCTGCCCGCTCC
TTCGGCTCAGCGCTCGTCGCCAAC---------GTGTGGACTGACCATTGGGTATACTGG
GCGGGTCCTATTGCCGGTGGCATAGCGGCTGCGCTATTGTACGTGCATGGATTTTCAGCC
---CCCGCTGTA---AGC------------------------ACGCCC---CCG------
------------------------CGC------------TACCGCTCC------GTCGCA

GGAGAC---GAG---AAAGAGCTGAAGAGGCTG---------GAC---------GGT---
---AAAGGCGAAGAC---
ATGGCT--

>GAJS01000346_Chilo_suppressalis_Prip
ATGGGT--

---GAA------------

---CTT

GGATCAAAATTGGGTCTGAGCGAGCTATCT---GGTGGTGCT---GCGGGTATCAGCAAA
GCCCTACTCGCTGAATTTATTGGCAATCTCCTCCTGAACCTGTTCGGCTGCGGCGCGTGT
ATAAACATCAGCCAGGGTAAC---------------------------------------
---ACGGCCGCCCCCGAC
ATCGTACTCATCGCCTTCGCCTTCGGTCTCACCATATTCGCCGCTATCTCGGCGATAGGT
CATGTATCTGGTGGACACATCAACCCGGCCGTGACGATAGGCTTGGCAGCGGCCGGGCGC
GTGAAGGTAGTGCGCGCCGTGTTGTACATCATAGCACAGTGCGCAGGCGCCGCAGCCGGC
TCCGGCCTGCTGAAGGCATTCACACCGGACCGCGTGGCTGGC------------------
------------------------------TCCCTGTGCGCCACCAGTCTCGGCACTGAC
---------GTCACAGCGCTTCAAGGATTCGGCATCGAGTTCTTCCTCGGATTCGTCCTC
GTATTCGTCGTCTGTGGG---GTATGTGATCCTAACAAGCCG------------------
---GACAGCAAG---------------GCGACCGCCCCCCTGGCTATCGGGCTGACGGTA
ACCCTGGGTCATCTGCTAGCCGTGGACTACACCGGCTCCGCTATGAACCCGGCGCGCTCT
TTCGGTTCCGCGCTGGTCGTCAAC---------ATGTGGACTTCTCATTGGGTATACTGG
GTGGGTCCCATAGCTGGTGGGGTGGCCGCTGCGCTCCTCTACGTGCACGGGTTCTCGGCG
---CCGGCACAGGAGACG---------------------------------CCG------
------------------------CGC------------TACAAAGCT------GTGGCC
AGCGAT---GAG---AAAGAGCTGAAGCGCCTG---------GAC---------GGCAGT
---AAAGAGGAAGAT---
CTAGCC--

>JP612966_Erynnis_propertius_Prip
ATGGGA--

---GAG------------

---CTC
GGCGCGAAACTGGGTCTCGACGAGTTGGCC---GGCGGAGCG---GCCGGCATCAGCAGG
GCGCTGCTCGCTGAATTTATTGGTAATCTCCTCCTAAACCTGTTCGGTTGTGGCGCCTGC
ATCAACATCGCTCAGGGCACG---------------------------------------
---ACCGCAGCTCCCGAT
ATCGTTCTCATTGCTCTCGCCTTCGGTCTAGCCGTGTTCGCTGCTGTTTCGGCGATCGGT
CACGTTTCTGGTGGGCACATAAACCCAGCAGTGACGCTAGGCTTGGCCTCAGCCGGTAAG
GTGAAGCTCGTACGAGCCGTCCTGTACGTGATCGCCCAGTGCGCTGGTGCCGCTGCTGGC
TCCGGGCTCCTCAAGGCCTTCACTCCTGACAAGGTAGCGGGC------------------
------------------------------TCGCTCGGAGTGACCGGCCTGGGCTCTGAC
---------GTCACACAGCTCCAAGGCTTTGGCATCGAGTTCTTCCTTGGCTTCGTTCTC
GTGTTCGTTGTTTGCGGG---GTTTGCGACCCCAACAAGCCG------------------
---GACGGCAAA---------------TCCACCGCGCCACTGACCATCGGACTGACGGTG
ACCCTCGGCCACCTCCTCGCCGTGGACTACACCGGCTCCGCCATGAACCCCGCTCGTTCC
TTCGGCTCTGCTCTTGTCGCCAAC---------GTATGGGCTGATCACTGGGTATACTGG
GCTGGTCCCATCGCCGGCGGAGTGGCGGCTGCCCTCCTATACGTGCATGGATTCTCCGCG
---CCCCCGGTTGAGGCC------------------------AGCTCC---CCT------
------------------------CGG------------TACAAGCCC------GTGGCT
GGTGAT---GAG---AAGGAGCTGAAGCGCCTG---------GAC---------GGC---
---AAAGCTGACGAC---
CTTGCC--

>JP717913_Papilio_zelicaon_Prip
ATGGGT--

---GAA------------

---CTT
GGAAGGAAACTTGGACTGGACGAGCTGTCT---GGAGGAACC---TCGGGCATCATCAAA
GCAATTTTAGCCGAGTTTATTGGTAACTTGCTACTGAACTTGTTCGGTTGCGGTGCGTGC
GTGAACATAGCGCAAGGTTCA---------------------------------------
---GGCGCAGCGCCTGAT
ATTGTGCTCATTGCACTCGCGTTTGGGTTATCTGTGTTTGCTATTGTTTCTGCAATCGGC
CACGTATCAGGGGGTCATGTAAACCCGGCTGTGACAGCGGGACTCGCATCTGCCGGTCGC
GTAAAGCCCGTGCGCGCGGTACTGTACGTGATCGCGCAGTGCGCGGGCGCGGCAGCCGGC
TCCGGCCTACTGAAGGCTTTCACGCCGGACCGGGCGGCGGGC------------------
------------------------------TCACTTGGCGTCACCGGACTGGGTACTGAG
---------GTTACGGCGTTACAAGGATTCGGGATTGAATTCTTCCTCGGATTCGTGCTC
GTCTTCGTCGTGTGTGGC---GTATGTGATCCCAACAAACCG------------------
---GACAGCAAA---------------GCGACTGCGCCCCTGGCGATAGGGCTAACAGTC
ACCCTTGGACATCTGCTGGCTGTGGACTACACAGGCTCCGCTATGAACCCCGCCCGCTCC
TTCGGATCCGCTATCGTCGCCAAT---------GTTTGGAATGACCACTGGGTGTACTGG

GCGGGCCCGGTGGCCGGCGGTGTGGCAGCGGCACTACTCTACGTGCACGGCTTCTCCGCC
---CCGGCCCCCGACAAC---------------------ATCACCCCA---CCC------
------------------------CGC------------TACAGACCC------GTCGCC
GGAGAC---GAA---AAAGAGCTGAAGAGGTTG---------GAC---------GGT---
---AAAGGCGAGGAC---
ATGGCC--

>NP_001153661_Bombyx_mori_Prip
ATGGGT--

```
---------------------------------------------GAG------------
------------------------------------------------------------
---------------------------------------------------------CTT
GGTACTAAACTGGGATTGGATGAGCTGACC---GGAGGAGCC---GCTACGATCAGCAGA
GCACTACTCGCTGAGTTTATCGGTAACTTGCTCCTAAATCTATTCGGCTGTGGTGCCTGT
GTGAAGATTTCGTTGGAGTCC---------------------------------------
---------------------------------------------AACAGTGAAACGGAC
ATCTTGCTCATCGCGTTGGCGTTTGGGCTAGCGGTATTTGCTGCTGTCTCGGCAATAGGT
CATATTTCTGGCGGACACCTAAACCCGGCGGTGACAGCCGGCATGCTGTGCACGGGCCGC
ATCAAGCTCATCCGTGCGGTGCTGTACGTCATCGTGCAATGCGCCGGGGCCGCGGCCGGT
TCGGGACTGCTCAAGGCGCTGACGCCGGACCGCATGGCCGGC------------------
------------------------------AGTCTCGGCTGCACCGGTCTCGGCGTTGAC
---------GTTACAGAGCTCCAGGGTTTCGGTATTGAGTTCTTCCTTGGATTCTTACTT
GTCTTCATCGTTTGTGGG---GTGTGCGATGCCAATAAGCCG------------------
---GACAGTAAA---------------GCAACGGCCCCGCTCGCCATCGGCCTCACCGTG
ACCCTGGGTCACTTGCTCGCCGTCGACTACACCGGCTCTGCCATGAACCCAGCCCGCTCC
TTTGGCTCGGCCCTCGTCGCTAGC---------AACTGGAGTCACCATTGGATATACTGG
GCTGGACCTATTGCTGGCGGTATTGCTGGTGCTTTGCTGTACGTCCACGGATTTACCGCG
---CCACCCCAGGAG-------------------------------CCGCCGACC------
------------------------AGA------------TACAGATCT------GTTGCC
GGTGAC---GAA---AAGGAGCTGAAGCGACTT---------GAT---------GGTGGT
---------------------------------------------AAACTGGACGAC---
ATGGCC------------------------------------------------------
------------------------------------------------------------
------------------------------------------------------------
------------------------------------------------------------
------------------------------------------------------------
------------------------------------------------------------
------------------------------------------------------------
------------------------------------------------------------
------------------------------------------------------------
------------------------------------------------------------
------------------------------------------------------------
------------------------------------------------------------
------------------------------------------------------------
------------------------------------------------------------
------------------------------------------------------------
------------------------------------------------------------
------------------------------------------------------------
------------------------------------------------------------
------------------------------------------------------------
------------------------------------------------------------
------------------------------------------------------------
------------------------------------------------------------
------------------------------------------------------------
------------------------------------------------------------
------------------------------------------------------------
------------------------------------------------------------
```


>XM_032661754_Danaus_plexippus_Prip
ATGGGT--

---GAG------------

---CTG
GGTAACAAACTAGGTCTGGATGAGCTGTCC---GGGGGTGCA---GCGGGAATCAGCAAG
GCTCTACTCGCTGAGTTCATCGGTAATCTATTGCTGAACTTATTCGGATGCGGCGCTTGT
ATCAACATAGCGCAGGGGTCG---------------------------------------
---GAAGGAGTCCCTGAC
ATCGTACTCATAGCCCTTGCATTCGGATTAGCGGTCTTCGCCATAGTTTCTGCGATAGGT
CATGTGTCGGGGGGCCACGTGAACCCCGCGGTGACAGTGGGCATGGCTGCCACGGGACGT
GTTAAACCTGTACGAGCTGTACTATATATAATAGCTCAATGTGCTGGCGCTGCCGCGGGG
TCGGGGTTGCTGAAGGCCTTCACCCCAGAAGCAGTGGCCGGC------------------
------------------------------AAGTTGGGGGTCACCGCCTTAGGGAAGAAC
---------GTTACCCCCCTACAGGGATTCGGCATTGAATTCTTCCTAGGTTTCGTCCTT
GTCTTCGTTGTATTTGGG---GTATGCGATCCTAACAAATCC------------------
---GACAGTAAG---------------TCGACGGCACCACTTGCCATCGGTCTGACAGTA
ACCCTGGGTCACTTACTGGCCGTGGACTACACCGGCTCAGCTATGAACCCAGCACGTTCC
TTCGGATCAGCGCTGGTGGCCAGC---------GACTGGGCCGATCACTGGGTATACTGG
GCGGGTCCTATATGCGGAGGTATCGCTGCCTCCTTGTTGTACTCGCACGGACTTGCCGCC
---CCTCCC---GAGGTC---------------------------GCTCCCCCG------
------------------------CGA------------TACAGATCC------GTGGCC
GGAGAC---GAG---AAAGAGCTTAAACGCCTG---------GAT---------GGC---
---AAGGCGGAAGAC---
ATGGCC--

>CAEZ01007983_Heliconius_melpomene_melpomene_Prip
ATGGGA--

---GAA------------

---CTC
GGTAAAAAACTTGGCTTAGATGAACTGTCG---GGAGGTGCA---GCTGGTATCAGCAAA
GCATTGCTCGCGGAATTTGTCGGTAATCTACTGCTCAACCTGTTTGGTTGTGGAGCAGTC
GTCAATATTGCACAAGGTTCT---------------------------------------
---GAAGGTGCCCAGGAT
ATTGTACTCATCGCCTTGGCTTTTGGTCTCGCTGTCTTCGCCATTGTTTCTGCAATAGGT
CACGTATCAGGTGGTCATGTGAACCCTGCTGTAACAGCTGGTATGGCCGCCACCGGCAAA
ATCAAACCGATTCGAGGTATCCTCTACGTGATAGCACAATGTGCTGGTGCAGCTGCAGGA
TCTGGACTACTTAAGGCGTTTACACCAGAACAGGTGGCCGGT------------------
------------------------------ACCCTCGGTACTACGGGACTTGGCAAGAAT
---------GTCACGCAATTGCAAGGTTTCGGAATTGAATTCTTCCTGGGATTCGTCCTC
GTGTTCGTTGTTTGCGGG---GTATGCGACGCAAACAAACCT------------------

---GACAGCAAA---------------TCAACGGCGCCACTCGCCATCGGTCTCACTGTC
ACACTGGGACACTTGCTGGCGGTGGACTACACGGGCTCCGCCATGAACCCCGCGAGATCC
TTCGGCTCTGCCCTCGTCGCCAGC---------ACCTGGGATGACCACTGGGTATACTGG
GCGGGACCGATAGCTGGCGGTGTGGCCGCGGCACTGCTCTATGTTCACGGCTTCGCGGCG
---CCTCCACCGGACTCGAAC---------------------TCGCTCTCTCCT------
------------------------CGG------------TACCGCCCG------GTCGCT
GCTGAT---GAA---AAAGAGGTA------------------------------------

>EL599805_EL596094_Heliconius_erato_Prip
ATGGGA--

---GAA------------

---CTC
GGTAAAAAGCTTGGTTTGGACGAATTGTCG---GGAGGTGCA---GCTGGTATCAGCAAA
GCATTGCTCGCGGAATTTGTCGGAAATCTACTGCTGAACTTGTTTGGTTGTGGAGCTGTG
ATCAATATTGCGCAAGGTTCT---------------------------------------
---GAAGGTGCCCAAGAC
ATTGTACTCATTGCCCTGGCTTTTGGTCTCGCTGTCTTCGCCATTGTTTCTGCCATAGGT
CACGTATCAGGTGGTCACGTGAACCCTGCTGTAACAGCTGGTATGGCCGCCACCGGCAAA
ATCAAACCGATTCGAGGTATCCTCTATGTGATAGCGCAATGTGCTGGTGCAGCTGCAGGA
TCTGGACTGCTTAAGGCGTTCACACCAGAACAAGTGGCCGGC------------------
------------------------------ACCCTCGGTACTACGGGACTTGGCAAAAAT
---------GTCACGCAATTACAAGGTTTCGGGATTGAGTTCTTCCTGGGATTCGTCCTC
GTGTTCGTTGTTTGCGGG---GTTTGCGACGCTAACAAACCT------------------
---GACAGCAAA---------------TCAACGGCGCCCCTCGCCATCGGCCTGAACGTG
ACACTGGGGCACTTACTGGCGGTGGACTACACCGGCTCCGCCATGAACCCCGCGAGGTCC
TTTGGCTCTGCCCTCGTCGCCAGC---------ACCTGGGATGACCACTGGGTATACTGG
GCGGGACCAATAGCTGGCGGTGTGGCCGCGGCACTACTCTATGTTCACGGTTTCGCAGCA
---CCTCCACCGGACACCAAC---------------------TCGCTCTCACCT------
------------------------CGG------------TACCGCCCC------GTCGCC
TCCGAT---GAA---AAAGAGTTGAAGCGCCTG---------GAC---------GGT---
---AAAGCCGACGAC---
ATGGCC--

>XM_008192181_Tribolium_castaneum_Prip
ATGGGA--

---AAC------------

---TTC
CACGAAACCCTCGGAATCAATGAATTGAAA---AACAAA------AATGCAATATGGAAA
GCACTTTTAGCTGAATTTCTGGCTAATGTCCTTCTCAATTTCTTCGGTTGTGCTTCCTGT
ACCCACATTTCA---GGG--
--AAACCCCCGAGT
TTAGTATTGATTGCGCTCACTTTCGGACTCGTCATCTTTATTCTAGCCCAAACTATTGAG
CATGTGAGTGGTGGGCACATAAACCCAGCTGTCACAGCTGGAATGATGGCCACCGGCAAT
ATTGGAATAGTGAAAGGAATTCTTTACATTATTGTGCAATGTCTGGGGGCTTTAGCCGGA
TCAGCGGTCTTAAAAGCCCTTACCCCCGAAGATTTGCACAAATCG---------------
------------------------------GGGCTTGGTAACACACAATTAGCCCCCGGT
---------TTTAGCCCAATTCAAGGCTTTGGGGTTGAATTTTTTCTCGGATTTGTTCTA
ATTCTTGTTATTTGTGGC---GTCTGCGATCCTAATAAACCT------------------
---CAAGCCAAA---------------CCTGCAGCAACTCTAGCTATTGGAATGGCTGTG
GCAGTGGGTCATTTAGCCACTGTTGACTTCACTGGTTCTAGTATGAACCCAGCAAGGAGT
TTCGGGTCGGCTTTAATCGCCAAT---------AATTGGACTGATCATTGGGTTTATTGG
GCTGGGCCTATTTTGGGTGGAGTTGCTGCAGCTCTGCTTTATAAACATGCGTTGGCTGCA
---CCGAATCTTGGACCA------------------------ATGAGGATTGTA------
------------------------GAACGA---------TATACG---------ATT---
GCAGAT---GAG---AAAGAATTGAAGAGGTTG---------GAC---------GTCAAC
---AGAGATGAACCG---

>AEE61850_Dendroctonus_ponderosae_Prip
ATGGGC--

---AAT------------

---TTC
CGAGAAGTTTTAGGCATCAATGAGGTGTCTTCATCCAAAGTGATCAACGCCGTATGGAAG
GCTCTTTTGGCCGAATTCCTCGGCAACTTCCTGCTGAATTTCTTCGGATGCGGATCGGTG
ATTGTGTTCAGTGCGGAA--
--CACAAGCCCAAC
TTCGTGTTGGTGTCGCTCACATTCGGACTGGTTGTTTTCGTCTGTGTTCAGGCTCTTGGT
CATGTGAGCGGCGCTCACCTCAATCCAGCCGTAACAGCGGGAATGTTGTCTGTAGGAAAC
ATCAAAGTAGTGAAAGCGATACTGTATGTTATAGTGCAGTGCATTGGAGCTTTGGCTGGA
TCCGCCACCCTTAAGGCCCTGTTGCCCGAAGAGTGGCACAATTCC---------------

```
------------------------------GGCCTGGGCTTGACAAACCTCAACCCGAAA
---------ATCACTCCTCTGCAAGGTTTCGGCTTTGAGTTCTTCCTCGGCTTCGTCTTG
GTGTTGGTGGTGAGTGGA---GTATGCGACCCGAACAGGCCG------------------
---GAAGCCAAA---------------GCTGCTGGGCCTCTAGCCATTGGTTTGGCTGTC
GCTTTGGGACACATGGGAGCCATCAACTTGACCGGCGCCTCCATGAATCCTGCCAGGAGT
TTTGGGAGTGCGGTGATTGCAGGC---------ATTTGGGAAAACCACTGGGTTTATTGG
GTGGGGCCAATAGCAGGTGGAGTGGTTGCTGCCCTGATCTACCACCACGCTCTTCGAGCG
---CCCACCCTAACAACT------------------------GTGAAGATCATC------
------------------------GAA---------------AGT---------------
------------------------------------------------------------
------------------------------------------------------------
------------------------------------------------------------
------------------------------------------------------------
------------------------------------------------------------
------------------------------------------------------------
------------------------------------------------------------
------------------------------------------------------------
------------------------------------------------------------
------------------------------------------------------------
------------------------------------------------------------
------------------------------------------------------------
------------------------------------------------------------
------------------------------------------------------------
------------------------------------------------------------
------------------------------------------------------------
------------------------------------------------------------
------------------------------------------------------------
------------------------------------------------------------
------------------------------------------------------------
------------------------------------------------------------
------------------------------------------------------------
------------------------------------------------------------
------------------------------------------------------------
------------------------------------------------------------
------------------------------------------------------------
------------------------------------------------------------
------------------------------------------------------------
------------------------------------------------------------
------------------------------------------------------------
------------------------------------------------------------
------------------------------------------------------------
------------------------------------------------------------
------------------------------------------------------------
------------------------------------------------------------
------------------------------------------------------------
------------------------------------------------------------
------------------------------------------------------------
------------------------------------------------------------
```


>GAWD01074919_Medauroidea_extradentata_Prip
ATGCCG--

--AGCGCG------------

---CTG
GAGGAACGTCTGGGACTGCGGGAGGTGAAG---------------GCTGGCATATGGAAG
CCTCTGCTCGCAGAGTTCCTGGGCAACCTGCTTCTCAATCTACTCGGCTGTGGCAGTGTC
ATCAACCTTGGCATTGACTCC---------------------------------------
--TTGCAGCCAAAC
ATCGTGCTCATCAGCCTCGCCTTCGGTCTCGCCATCATGATGATCGTCCAGTCGGTTGGA
CACGTGAGTGGAGCACACATCAACCCGGCCGTTACCTTCGGCATGCTCGTCACAGGCAAC
ATCACCATCATCAAGGGACTGCTGTACGTCATAGTGCAGTGTGTCGGCGCCATTGCCGGC
ACCGCTGTACTTAAGGCTCTGACGCCGCTGCAGTACCAAGGC------------------
------------------------------AACCTAGGCACGACGATGCTCCACCACTCA
---------GTGACGATGCTGCAAGGCTTCGGTATCGAGTTCTTCCTCGGCTTCATCCTG
GTGTTCGTCGTATCCGGC---GTGTGTGACAGCAACCGCGGA------------------
---GAGACCAAG---------------GGCTTTGCGCCGCTCGCCATCGGCCTCACTATC
ACGCTGGGACACCTCTTCGCGATTGACTACACTGGATCTAGCATGAACCCCGCCAGGAGT
CTTGGCTCTTCAGCTATCTCTGGA---------ATTTGGGACAATCATTGGGTGTACTGG
CTGGGACCTATCCTGGGCGGTATAGCGGCAGCTCTTATCTACAAACACGCCTTCTCTGCC
---CCAACAGTGGAGGTG---------------------------------GCC------
------------------------GAG------------TACAGTCTT------GTGCAG
---------------------CTTAAACAATTT---------GAT---------GGGAAG
---AAGGACGAAGATGGT
AACGCT--

>GAWE01078541_Ramulus_artemis_Prip
ATGCCG--

--AGCGCG------------

---CTG
CAGGAACGTCTGGGTCTGAGGGAGGTGAAG---------------GCGGGCATCTGGAAG
CCCCTGCTGGCAGAGTTCCTCGGCAACCTGCTGCTCAACCTGCTGGGCTGTGGCAGTGTC
ATCAACCTGGGCATCGACTCC---------------------------------------
--CTGCAGCCCAAC
ATCGTGCTCATCGCCCTTGCTTTCGGCCTCTCCATCATGATGATCATACAGACGGTGGGA
CATGTGAGTGGGGCGCACATCAACCCAGCAGTGACCTGCGGCATGCTCGTCACTGGCAAC
ATCACCATCATCAAGGGTCTGCTGTACGTCGTGGTGCAGTGCATCGGGGCCATCGCTGGC
ACCGCGGTGCTCAAGGCGCTGACGCCGACGAACTACCAAGGC------------------
------------------------------AACCTCGGCACGACTCTGCTGCATCCCTCC
---------ATGACCATGCTGCAAGGCTTCGGTATCGAGTTCTTCCTCGGCTTCATCCTG
GTGTACGTGGTGATGGGG---GTCTGCGACGGCAACCGTAGC------------------
---GAGACCAAG---------------GGGTTCGCGCCGCTCGCCATCGGCCTCACCATC
ACGCTGGGCCACCTCTTCGCGATTGACTACACTGGATCAAGCATGAACCCTGCCAGGAGT
CTTGGCAGCGCAACTATCTCAGGA---------ATTTGGGACAACCACTGGATCTACTGG
CTGGGACCAATCCTGGGCGGTGTGGCGGCAGCTCTCCTCTACAAACACGCCTTCTCTGCC
---CCTACGGTGGAGGTT---------------------------------GCC------
------------------------GAG------------TACAGTCTG------GTGCAG
---------------------CTAAAGCAGTTT---------GAT---------GGGAAG
---AAGGACGAAGACGGA
AACGCT--

>GAWG01042575_Extatosoma_tiaratum_Prip
ATGCCG--

--AGCGCG------------

---CTG
AAGGAACGCCTGGGGCTGCGCGAGGTGACG---------------GCGGGCATCTGGAAG
CCTCTGCTCGCGGAGTTCCTCGGCAACCTGCTGCTCAACCTCTTCGGCTGCGGCAGCGTC
GTCAACCTGGGAGTCGACTCG---------------------------------------
--CTGCAGCCCAAC
ATCGTGCTCATCGCCCTCGCCTTCGGCCTCGCCATCATGATGATCGTGCAGACGGTGGGT

CACGTGAGCGGCGCGCACGTCAACCCGGCCGTCACGTGCGGCATGCTCGTCACTGGCAAC
ATCACCGTGCTCAAGGGCCTGCTGTACATCGTGGTGCAGTGCGTCGGCGCCATCACCGGC
ACAGCCGTGCTCAAGGCTCTGACGCCGGAGTTGTACCAGGGT------------------
------------------------------AACCTCGGCACGACGCTCGTCAACCCCGCC
---------CTGACGGCGCTCCAGGGTTTCGGCATCGAGTTCTTCCTCGGCTTCATCCTG
CTGATCGTGGTGTTCGGC---GTGTGCGACGCCAACCGCAGC------------------
---GAGACCAAG-----------------GGCTTCGCCGCTCGCCATCGGCCTCACCATC
ACGCTGGGACACCTCGTCGCGATCGACTTCACCGGGTCCAGCATGAACCCCGCCAGGAGT
CTTGGTTCCGCAGTTCTGTCCGGA----------GTGTGGACAACCACTGGGTGTACTGG
CTGGGACCCATCCTGGGCGGCCTGGCGGCAGCTCTCATCTACAAACACGCCTTCTCCGCC
---CCAACGGTGGAAGTG---------------------------------GCT------
------------------------GAG------------TACAGTCTG------GTGCAG
---------------------CTAAAACAATT----------GAT---------GGGAAG
---AAGGATGAAGATGGC
AACGCT--

>GAWC01082955_Aretaon_asperrimus_Prip
ATGCCT--

--AGCGCG------------

---CTG
GTAGAACGCCTGGGGCTGCAGGAGGTGAAA---------------GCGGGTATCTGGAGG
GCTCTACTTGCAGAGTTCCTCGGCAACCTGGTGCTCAACTTGTTCGGCTGCGGCAGTTGC
ATCAACCTGGGTGTCGACCAG---------------------------------------
--TTGACTCCCAAC
ATCGTGCTCATCGCACTCGCCTTCGGCCTCGCCATCATGATGATTGTGCAGACGGTGGGT
CACGTGAGTGGGGCGCATGTGAACCCAGCTGTGACGTGTGGCATGCTGGTTACCGGCAAC
ATCACCGTGCTCAAGGGCCTGCTGTACATCATCGTGCAGTGCGTGGGCGCCATCACTGGC
ACAGCCGTGCTCAAGGCGCTGACACCGGACACGGCCTATGGT------------------
------------------------------AACTTGGGCACGACGATGATCCACGGGGCC
---------CTGAAGCCGATCCAGGGCTTCGGCATCGAGTTCTTCCTCGGCTTCATCCTC
GTCATGGTGGTGTTCGGC---GTGTGCGACGCCAACCGCAGT------------------
---GAGACCAAG---------------GGCTTCGCTCCTCTTGCCATTGGACTGACTGTC
AGCCTGGGTCACCTCGTCGCGATTGAATACACTGGTTCCAGCATGAACCCCGCCAGGAGC
CTTGGCTCAGCAGTTCTGTCTGGT---------ATCTGGGAGAATCACTGGATTTACTGG
CTGGGTCCAATCCTGGGCGGCATCTCGGCAGCTCTCATCTACAAGCACGCCTTCTCTGCT
---CCGGTGGTGGAGGTA---------------------------------GCA------
------------------------GAG------------TACAGTCTG------GTGCAG
---------------------CTAAAACAATTT---------GAT---------GGGAAG
---AAGGACGAAGATGGT
AACGCT--

>GAWF01050038_01050037_Sipyloidea_sipylus_Prip
ATGCCG--

--AACGCA------------

---GTG
GTTGAACGACTTGGTCTGAGTGAGGTGAAG---------------GCAGGTATATGGAAG
GCACTACTCGCAGAGTTCCTAGGAAACCTACTACTAAACCTGTTTGGTTGTGGTAGTTGT
ATCAACCTAGGAGTGGATCCT---------------------------------------
--ATCAGTCCTAAC
CTTGTACTAATCAGTCTGACATTCGGTCTGGCCATCATGATGATTGTACAGACGTTTGGT
CACGTGAGTGGAGCACATGTGAACCCGGCTGTAACGTGTGGTATGCTAGTTACAGGCAAC
ATTACAATACTTAAAGGTCTATTGTATATTATAGTACAATGTGTGGGCGCCATTGCTGGT
ACTGCTATTCTCAAGGCACTAACACCAGATGATTACCAAGCT------------------
------------------------------ACTCTAGGTACAACAGTTGTCAATGGTAAA
---------ATGACATCACTACAAGGCTTTGGTATAGAGTTCTTCTTAGGATTCATATTA
CTTCTGGTCGTGTTTGGA---GTATGTGATTCAAACAGAAGT------------------
---GATACTAAA---------------GGCTTCGCTCCTCTTGCCATCGGACTGACCATC
TCACTGGGACATCTTGTTGCTGTAGACTACACTGGCTCGAGTATGAATCCTGCAAGAAGT
CTAGGATCATCAGTTCTGTCTGGA---------ATATGGGACGATCACTGGGTGTACTGG
CTGGGACCAATCCTGGGTGGAATAGCAGCAGCTCTCTTGTACAAACATGCATTCTCTGCC
---CCAGTAGTAGAGGTT---------------------------------GCT------
------------------------GAG------------TACAGTCTT------GTGCAG
---------------------CTGAAGCAGTTT---------GAT---------GGGAAG
---AAAGACGAAGATGGC

GTTGCT--

>FL638365_GO907955_Reticulitermes_flavipes_Prip
ATGGCT--

---------------------------------------GGCTCTGGC------------

---ATC
AAGGAGAAGCTGGGCGTGGATGAGATTACT---------------CGGGGGATTTGGAAG
GTGCTACTCGCAGAATTCCTGGGCAACCTGCTGCTGAATTTCTACGGGTGCGCGAGCTGC

```
GTCGGCACTGGAAACGAA------------------------------------------
------------------------------------------------------AAGAGT
GCAGTGCTAGTTGCCCTCACTTTTGGTCTTGTCATCATGGCCATTGTTCAGTCTGTGGGT
CATGTGAGTGGTGCACATGTGAACCCGGCAGTTACGTGTGGCTTGCTAGCTGCTGGAAAC
ATCTCCATCCTCAAAGCACTGCTCTACATCACTGTACAATGCCTTGGTGCAGTTGCTGGC
ACTGGAGTACTTAAGGCCCTAACTCCACTAGAGGCACAAAAG------------------
------------------------------AAATTGGGTATTACTCTACTCTCACCAGGT
---------GTCACACCTGTGCAAGGGTTTGGTATTGAGTTCTTCCTTGGATTTGTGCTT
GTACTAGTGGTGTTTGGA---GTATGTGACGTGAACCGCCCT------------------
---GAAGTGAAG---------------GGGCTGGCTCCTGTTGCCATTGGACTTACCATT
GCTATGGGACACCTCGCTGTGCTGGATTATACAGGTTCCAGCATGGATCCTGCACGCACT
CTTGGTTCTGCTATAATAACAGGG----------ATATGGAAAATCACTGGGTGTATTGG
TTGGGTCCCATACTTGGAGGAATCTCAGCTGCCTTGATCTACAAGCACGCTTTCTCTGCA
---GTTCCTGTTGAGATA------------------------------AGGACA------
------------------------GAC------------TACACTCCA------GTGCAG
---------------------CTGAAGCGACTC---------GAC---------AACAAG
---------------------------------------------AAAGATGAGGATGGA
ATGGCT------------------------------------------------------
------------------------------------------------------------
------------------------------------------------------------
------------------------------------------------------------
------------------------------------------------------------
------------------------------------------------------------
------------------------------------------------------------
------------------------------------------------------------
------------------------------------------------------------
------------------------------------------------------------
------------------------------------------------------------
------------------------------------------------------------
------------------------------------------------------------
------------------------------------------------------------
------------------------------------------------------------
------------------------------------------------------------
------------------------------------------------------------
------------------------------------------------------------
------------------------------------------------------------
------------------------------------------------------------
------------------------------------------------------------
------------------------------------------------------------
------------------------------------------------------------
------------------------------------------------------------
------------------------------------------------------------
------------------------------------------------------------
------------------------------------------------------------
------------------------------------------------------------
------------------------------------------------------------
------------------------------------------------------------
------------------------------------------------------------
```


>JK445261_JK445262_Coptotermes_formosanus_Prip
ATGGCT--

---------------------------------------GGCTCTGGC------------

---ATC
AAGGAGAAGCTGGGCGTGGAGGAGATTACT---------------CGGGGGATTTGGAAA
GCGCTACTCGCAGAATTCCTCGGCAACCTGCTGCTAAATTTCTACGGGTGCGCAAGCTGC
GTCGGCACCGGACAGGGA--
--AAGAGT
GCAGTGCTAATTGCCCTCACTTTCGGCCTTGTTATCATGGCCATTGTTCAGTCTGTGGGT
CATGTGAGTGGCGCACATGTGAACCCAGCAGTTACGTGTGGCATGCTAGCTACTGGAAAC
ATCTGCATCCTCAAAGCACTACTCTACATCGCTGTACAATGCCTAGGTGCAGTTGCTGGG
ACTGGAGTGCTTAAGGCCCTAACTCCACTACATGCACAAAAG------------------
------------------------------CAATTGGGTATTACTCTACTCTCAGAAGGT
---------GTGACACCTGTCCAAGGTTTCGGTATTGAGTTCTTCCTTGGATTTGTGCTT
GTACTAGTGGTATTTGGA---GTATGTGACGTGAACCGCTCT------------------
---GAAGTGAAG---------------GGGCTGGCTCCTGTTGCCATTGGACTTACCATT
GCTATGGGACACCTGGCTGTCCTGGATTATACAGGTTCCAGCATGAATCCTGCACGCACT
CTTGGTTCTGCTATAATCACAGGA---------ATATGGGAAGACCACTGGGTGTACTGG
TTGGGTCCCATACTTGGAGGAATTTCAGCTGCCTTGATCTACAAGCACGCTTTTTCAGCA
---GTTCCTGTTGAGATG------------------------------ACGACA------
------------------------GAC------------TACACTCCA------GTGCAG
---------------------CTGAAGCGACTC---------GAC---------AACAAG
---AAAGATGAGGATGGA
ATGGCT--

>AUST01002325_01002324_Zootermopsis_nevadensis_Prip
ATGGCT--

---------------------------------------GGCTCCAGC------------

---ATC
AAGGAGAAGCTGGGCGTGGAGGAGATTACG---------------CGGGGAATCTGGAGG
GCGCTACTCGCCGAATTCCTTGGCAACTTGCTCCTTAACTTCTACGGGTGTGCGAGTTGC
ATAAGCATTGGAGACAGC--
---GCGAAGGGT
CCAGTGCTGGTCTCCCTCACTTTTGGGCTTGTCATCATGGCCATTGTCCAGTCCATAGGG
CACGTAAGCGGGGCCCATGTGAACCCGGCGGTGACATGTGGCATGCTGGTTACTGGAAAT
ATCTCCATCCTCAAAGCCCTTCTCTACATTACTGTGCAGTGCCTTGGTGCAGTTGCAGGC
ACAGGCGTGCTAAAGGCTCTAACACCACATTACGCACAGAGT------------------
------------------------------AAGTTGGGCATTACTGTACTCACCCATGGT
---------GTGACACCCGTACAAGGCTTTGGCATCGAGTACTTCCTCGGATTTGTGCTT
GTACTAGTGGTATTTGGA---GTGTGTGATGTGAATCGCTCT------------------
---GAAGTGAAG---------------GGACTGGCTCCTGTTGCCATTGGGCTTACCATT
GCCATGGGCCACCTCGCTGTACTGGATTACACAGGTTCCAGCATGAATCCTGCACGCACT
CTTGGTTCTGCTATACTCACAGGA---------ATATGGGATAACCATTGGGTATACTGG
CTGGGGCCCATACTTGGAGGAATCTCAGCTGCCTTGATCTATAAGCATGCTTTCTCTGCA
---GTCCCCATTGAGAGA------------------------------ACCACA------

------------------------GAA------------TACACTCCAGTACAGGTACAG
GTTCAG---GAC---AAAGAATTGAAACGGCTG---------GAC---------AACAAG
---AAAGACGAGGATGGA
ATGGCT--

>EEB16742_Pediculus_humanus_corporis_Prip
ATGCCG--

---------------------------------------AAAAGAAGC------------

---ATA
AAAGAACAATTGGGTATAAACGAATTACTC---GACAAAAAA---GGTGGAATATGGAAA
GCTTTACTCGCTGAATTTTTAGGTACATTTCTTCTTAACTTTTTCGGTTGCGCAAGTTGT
TTGCACATAACAAAT---CCGGAT------------------------------------
--GATGTAACGGAT
TTAGTTAGAATATCATTAACGTTTGGTCTCGTCATATTTGGAGCAGTTCAGGCTTTTTGT
CACGTGAGTGGTGCTCATCTTAATCCGGCCGTTACGATTGCCGTACTCGTAACGGGTAAA
ATTCCTATTATAAAATCATTATTTTATGTAATAGTTCAATGCCTTGGATCCGTTGCAGGT
TCAGCTGTATTAAAGGCTCTTACACCGGATGATCCTACAGTACAG---------------
---------------------------GGAACGTTAGGATTAACGGCTGTTAATCCAAAA
---------TTAAGTCCTGTTCAAGGTTTCGGAGTTGAATTTTTTCTAGGTTTTGTTTTG
GTCATGGTTGTTTTCGGA---GTTTGCGATTCTAATAGAAAA------------------
---TCTGTCGAT---------------ATACCTGCACCCCTTGCAATCGGTTTAACGGTC
GGAATGGGTCATTTGGCAACGATCGAACTCACTGGATCCAGCATGAATCCTGCCAGAACA
TTCGGATCGGCTGTTATTGCAAAC---------ATTTGGACTAATCATTGGGTTTACTGG
TTGGGACCCATATTAGGTGGTATAGCAGCTGCATTGATATACCATCACACATTTTCAGCT
---CCGCCATTAGATGAA------------------------------GTAACA------
------------------------GAA------------TATAGTCCG------GTACGA
GCTGAA---GAA---AAAGAAGTG---AGTTAT---------------------------

>CO821055_CO821056_Locusta_migratoria_Prip
ATGGCT--

---------------------------------------GGA---GGA------------

---TTC
AAGGAAAGGCTTGGAATTTCTGACATTACT---GATAAA------AATGGAATCTGGAGA
TGTCTACTGGCAGAATTTATTGGTACCTTACTGCTGAATTTCTTTGGTTGTGGAAGTTGT
GTCTATGCTGGTAATGATCCAGGT------------------------------------
--GCCAAATCCAGC
CAGGTTCTCATTGCACTCACCTTTGGTCTTGTCATCATGGCAATAGTTCAGATTGTGGGG
CATGTGAGTGGAGCACATGTGAACCCAGCAGTTACTGCTGGAATGCTTTTTACAGGAAAT
ATTACAGTACTCAAGGGGCTGCTGTATATAATAGCTCAGTGTCTTGGATCCATCACAGGA
AGTGCAATCCTGAAGGCTCTTACTCCTGATGCTAAAGCTGGA------------------
------------------------------ACCCTTGGACTCACAAAAGTGACAGAAGGT
---------GTATCATCAGTTCAAGGATTTGGTATTGAATTTTTCCTTGGTTTTGTCCTG
GTACTGGTTGTATTTGGT---GTTTGTGATGTCAACAAACCA------------------
---GAAAGTAAG---------------GGATTTGCTCCTCTGGTTATAGGACTGACAATT
GCAATGGGACATCTAGCTGCAGTTGATTACACTGGAGCAAGTATGAACCCTGCAAGGACA
CTTGGATCAGCTGTCATTGCCAAT---------ATATGGACAGACCACTGGGTATACTGG
CTAGGACCAATCCTAGGTGGTATTGCTGCTGCTCTGCTGTACAAAAATGCTTTTGCTGCA
---CCCTCACCTGATGTC------------------------------AACATG------
------------------------GAA------------TACAGTCCA------GTTCAG
---------------------CTTAAGCGACTA---------GAT---------AATCGG
---AAAGATGAAGATGGA
ATGGCT--

>EH632028_Laupala_kohalensis_Prip
ATGGCG--

---------------------------------------GGT---CAA------------

---TTT
ATGGAACGCGTGGGAGCTACAGAGCTCCGA---GACAAGAGG---ACAAATATCTGGAAG
GCCCTGATTGCTGAATTTACAGGCGTCCTACTATTGAATCTCTTTGGATGCGGCTCGTGC
CTTAATGTTGCTAACAATCCACTC------------------------------------
--GACGAGGGGAAC
TACGTCACAATCAGTCTCACCTTCGGCCTCATCATCATGGCTGTTGTTCAGGCTTTTGGT
CATGTGTCAGGAGCACATGTCAATCCTGCCGTAACAGCTGGAATGCTTATCACTGGTAAT
ACTTTACCTCTCAGGGGTATGCTCTATATTATTACACAATGTCTGGGAGGGATCACAGGA
ACAGCTGTGCTGAAGGCTCTAACTCCTGAAGGTGTACAAGGA------------------
------------------------------GGACTTGGAATTACAAAGCTTGGTCCAGGT
---------GTTACTCCCATTCAAGGTTTTGGAATAGAATTCTTCCTTGGTTTTGTTCTT
GTGCTTGTGGTATTCGGA---GTGTGTGATCCAAACAAGCCA------------------
---ATAACTCAA---------------GGAATTGCACCCCTTGTTATTGGTCTCACTGTC
ACAGTGGGACACTTGATGGCTATTGACTACACTGGATCAAGCATGAACCCTGCACGCACA

CTGGGATCAGCTGTTCTATCCAAC---------ATTTGGGCGAATCACTGGGTATATTGG
CTTGGTCCGATTTTGGGAGGAGTAGCAGCTGGTCTCCTTTACCACCATGTTTTCAGAGCA
---CCAGCTGAAGAAGCC------------------------------CCCACT------
------------------------GAG------------TACTCTCCC------GTTGAG

>GAIZ01018549_Gryllus_firmus_Prip
ATGTCG--

```
------------------------------------------------------------
---------------------------------------GCG---AAC------------
------------------------------------------------------------
---------------------------------------------------------TTG
ATGGAACGAGTGGGCGTGAACGAGCTAAAG---GATCGACGC---GGGGGAATATGGAAA
GCGCTCCTCGCAGAGTTCCTTGGCACTATGATACTGAACCTCTTCGGATGCGGATCCTGC
ATCAATGTCAATGAAACC------------------------------------------
------------------------------------------------CAAACTGGAGGT
TATATTCTCATCAGCCTTTCCTTTGGTCTTGCAGTCATGGCGGCTATTCAGGCTGTTGGT
CACGTGTCTGGGGCACATGTCAATCCTGCTGTAACTGCCGGAATGCTCGTGACCGGTCGT
GTATATCCACTAAAGGGACTTCTTTATATAATAGCACAATGCATCGGTGCAATTACTGGC
ACTGCTATTCTGAAGGCCTTGACGCCCGACAAAGTGCAGAAT------------------
------------------------------AATCTAGGCATGACGACACTTGGCCCGAAT
---------GTCACCGCAATTCAAGGATTCGGAATTGAATTTTTCCTTGGATTCATTCTT
GTGCTGGTAGTTTTTGGT---GTGTGCGATCCTAACAAACCT------------------
---ACTACAGCA---------------GGAATTGCCCCGCTGGTAATTGGACTTACCGTT
ACTGTGGGTCACTTGATGGCGATTGATTACACTGGATCCAGCATGAATCCTGCCCGCACT
CTGGGCTCTGCAGTGGTGAGCGCT---------CACTGGGATGACCACTGGGTGTATTGG
TTGGGACCAATTCTTGGTGGAATCTCTGCAGCCCTTCTTTATTATCATGCCTTCAGTGCA
---CCTGCTGTTGAAGCA------------------------------CCTACA------
------------------------GAA------------TACAGCCCT------GTGCAG
---------------------TTGAAGCGCCTT---------GAC---------AAGAAC
---------------------------------------------AAGGACGAGGATGGA
ATGGCT------------------------------------------------------
------------------------------------------------------------
------------------------------------------------------------
------------------------------------------------------------
------------------------------------------------------------
------------------------------------------------------------
------------------------------------------------------------
------------------------------------------------------------
------------------------------------------------------------
------------------------------------------------------------
------------------------------------------------------------
------------------------------------------------------------
------------------------------------------------------------
------------------------------------------------------------
------------------------------------------------------------
------------------------------------------------------------
------------------------------------------------------------
------------------------------------------------------------
------------------------------------------------------------
------------------------------------------------------------
------------------------------------------------------------
------------------------------------------------------------
------------------------------------------------------------
------------------------------------------------------------
------------------------------------------------------------
```

```
------------------------------------------------------------
------------------------------------------------------------
------------------------------------------------------------
------------------------------------------------------------
------------------------------------------------------------
------------------------------------------------------------
------------------------------------------------------------
------------------------------------------------------------
------------------------------------------------------------
------------------------------------------------------------
------------------------------------------------------------
------------------------------------------------------------
------------------------------------------------------------
------------------------------------------------------------
------------------------------------------------------
>XP_003394168_Bombus_terrestris_Prip1
ATGGCA------------------------------------------------------
------------------------------------------------------------
---------------------------------------------AAT------------
------------------------------------------------------------
---------------------------------------------------------TTA
AAGGAGGCTTTAGGAACAGACGAGCTGACT---AATAAAAAA---GCTGGCCTTTACCGA
GCACTGATCGCAGAATTTCTTGGTACGCTGCTGTTGAACTTCTTTGGATGTGGATCTGTC
GTCACG------------------------------------------------------
------------------------------------------------------GGCAAC
GTCGTGGCCATAAGCTTGGCTTTTGGTTTGACTGTGGCCGCAGCAATTCAAGGAATAGGA
CATATTTCTGGTGGTCATGTTAATCCCGCTGTCACGTTCGGATTAATGGTGATTGGCAAG
GTGCCAGTTATCAGAGGCTTGCTGTACGTAATACTTCAATGTATAGGCGCAGTAGCAGGA
TCTGGTATACTAAAAGCGTTATCTCCCGAGAGGATGGAACAT------------------
------------------------------GTTTTAGGCGTAGTATCTCTTTCGCCAGGC
---------GTTACTCCTGTTCAAGGTTTCGGTATAGAATTTTTCCTCGCCTTCGCATTG
GTTCTGGTCGTATGCGGT---GCATGCGATGCAGCAAAACCA------------------
---GACAGCAAG---------------GGGATTGCTCCTCTTATAATCGGATTTACGGTT
TCTCTCTGTCATATCGTCGGTATACCAAGAACTGGCGCAGGTATGAATCCAGCTCGGTCA
CTCGGTAGTGCCATTGTAATGGAT---------ATATATGAGGATCACTGGCTGTATTGG
GTTGGTCCAATCCTTGGTGGTATGGCAGCTGGATTGATCTACGCGTTCGTAATCGGTCCT
---GCGAAAGAGTCGAAAAAT------------------------TCCAACCGA------
------------------------GTG------------TACACGGTC------GCCGCG
ACGGAC---GAG---AAGGAGAAGTTCGAGTAC------ATAGAC---------AGCGGA
---------------------------------------------CACGGT------GGA
---CTT------------------------------------------------------
------------------------------------------------------------
------------------------------------------------------------
------------------------------------------------------------
------------------------------------------------------------
------------------------------------------------------------
------------------------------------------------------------
------------------------------------------------------------
```


>XM_012382617_Bombus_impatiens_Prip1
ATGGCA--

---AAT------------

---TTA
AAGGAGGCTTTAGGTACAAACGAGCTGACC---AATAAAAAA---GCTGGCCTTTACCGA
GCACTGATCGCAGAATTTCTTGGTACGTTGCTGTTGAACTTCTTTGGCTGTGGATCTGTC
GTCACG--
--GGCAAC
GTCGTGGCCATAAGCTTGGCTTTTGGTTTGACTGTAGCCGCAGCGATTCAAGGAATAGGA
CATATTTCTGGTGGTCATGTTAATCCCGCTGTCACGTTCGGATTAATGGTGATTGGCAAG
GTGCCAGTTATCAGAGGCTTGCTGTACGTAATACTTCAATGTATAGGCGCAGTAGCAGGA
TCTGGTATACTAAAAGCGTTATCTCCCGAGAGGATGGAACAT------------------
------------------------------GTTTTAGGCGTAGTATCTCTTTCACCAGGC
---------GTTACTCCTGTCCAAGGTTTCGGTATAGAATTTTTCCTTGCCTTCGCATTG

```
GTTCTGGTCGTATGTGGT---GCATGCGATGCAGCAAAACCA------------------
---GACAGCAAG---------------GGGATTGCTCCTCTTATAATCGGATTTACAGTT
TCTGTCTGTCATATCGTCGGTATACCAAGAACTGGCGCAGGTATGAATCCAGCTCGATCA
CTCGGTAGCGCCATTGTAATGGAT---------ATATATGAGGATCACTGGCTGTATTGG
GTTGGCCCAATCCTTGGTGGTATGGCAGCTGGATTGATCTATACGTTCGTAATCGGTCCT
---GCGAAAGAGTCGAAAAAT------------------------TCCAACCGA------
------------------------GTG------------TACACGGTT------GCCGCG
ACGGAC---GAG---AAGGAGAAGTTCGAGTAC------ATAGAC---------AGCGGA
---------------------------------------------CACGGT------GGA
---CTT------------------------------------------------------
------------------------------------------------------------
------------------------------------------------------------
------------------------------------------------------------
------------------------------------------------------------
------------------------------------------------------------
------------------------------------------------------------
------------------------------------------------------------
------------------------------------------------------------
------------------------------------------------------------
------------------------------------------------------------
------------------------------------------------------------
------------------------------------------------------------
------------------------------------------------------------
------------------------------------------------------------
------------------------------------------------------------
------------------------------------------------------------
------------------------------------------------------------
------------------------------------------------------------
------------------------------------------------------------
------------------------------------------------------------
------------------------------------------------------------
------------------------------------------------------------
------------------------------------------------------------
------------------------------------------------------------
------------------------------------------------------------
------------------------------------------------------------
------------------------------------------------------------
------------------------------------------------------------
------------------------------------------------------------
------------------------------------------------------------
------------------------------------------------------------
------------------------------------------------------------
------------------------------------------------------------
------------------------------------------------------------
------------------------------------------------------------
------------------------------------------------------------
------------------------------------------------------------
------------------------------------------------------------
```

>XM_048404060_Bombus_terrestris_Prip2
ATGGCA--

---AAT------------

---TTA
AAGGAGGCTTTAGGAACAGACGAGCTGACT---AATAAAAAA---GCTGGCCTTTACCGA
GCACTGATCGCAGAATTTCTTGGTACGCTGCTGTTGAACTTCTTTGGATGTGGATCTGTC
GTCACG--
--GGCAAC
GTCGTGGCCATAAGCTTGGCTTTTGGTTTGACTGTGGCCGCAGCAATTCAAGGAATAGGA
CATATTTCTGGTGGTCATGTTAATCCCGCTGTCACGTTCGGATTAATGGTGATTGGCAAG
GTGCCAGTTATCAGAGGCTTGCTGTACGTAATACTTCAATGTATAGGCGCAGTAGCAGGA
TCTGGTATACTAAAAGCGTTATCTCCCGAGAGGATGGAACAT------------------
------------------------------GTTTTAGGCGTAGTATCTCTTTCGCCAGGC
---------GTTACTCCTGTTCAAGGTTTCGGTATAGAATTTTTCCTCGCCTTCGCATTG
GTTCTGGTCGTATGCGGT---GCATGCGATGCAGCAAAACCA------------------
---GACAGCAAG---------------GGGATTGCTCCTCTTATAATCGGATTTACGGTT
TCTCTCTGTCATATCGTCGGTATACCAAGAACTGGCGCAGGTATGAATCCAGCTCGGTCA
CTCGGTAGTGCCATTGTAATGGAT---------ATATATGAGGATCACTGGCTGTATTGG
GTTGGTCCAATCCTTGGTGGTATGGCAGCTGGATTGATCTACGCGTTCGTAATCGGTCCT
---GCGAAAGAGTCGAAAAT-------------------------TCCAACCGA------
------------------------GTG------------TACACGGTC------GCCGCG
ACGGAC---GAG---AAGGAGCTACAGAATCTT------ACCAGC---------AAGAGG
---AAAGATAAC---CCC
---GCT--

>XM_012382616_Bombus_impatiens_Prip2
ATGGCA--

---AAT------------

---TTA
AAGGAGGCTTTAGGTACAAACGAGCTGACC---AATAAAAAA---GCTGGCCTTTACCGA
GCACTGATCGCAGAATTTCTTGGTACGTTGCTGTTGAACTTCTTTGGCTGTGGATCTGTC
GTCACG--
--GGCAAC
GTCGTGGCCATAAGCTTGGCTTTTGGTTTGACTGTAGCCGCAGCGATTCAAGGAATAGGA
CATATTTCTGGTGGTCATGTTAATCCCGCTGTCACGTTCGGATTAATGGTGATTGGCAAG
GTGCCAGTTATCAGAGGCTTGCTGTACGTAATACTTCAATGTATAGGCGCAGTAGCAGGA
TCTGGTATACTAAAAGCGTTATCTCCCGAGAGGATGGAACAT------------------
------------------------------GTTTTAGGCGTAGTATCTCTTTCACCAGGC
---------GTTACTCCTGTCCAAGGTTTCGGTATAGAATTTTTCCTTGCCTTCGCATTG
GTTCTGGTCGTATGTGGT---GCATGCGATGCAGCAAAACCA------------------
---GACAGCAAG---------------GGGATTGCTCCTCTTATAATCGGATTTACAGTT
TCTGTCTGTCATATCGTCGGTATACCAAGAACTGGCGCAGGTATGAATCCAGCTCGATCA
CTCGGTAGCGCCATTGTAATGGAT---------ATATATGAGGATCACTGGCTGTATTGG
GTTGGCCCAATCCTTGGTGGTATGGCAGCTGGATTGATCTATACGTTCGTAATCGGTCCT
---GCGAAAGAGTCGAAAAAT------------------------TCCAACCGA------
------------------------GTG------------TACACGGTT------GCCGCG
ACGGAC---GAG---AAGGAGCTACAGAATCTT------ACCAGC---------AAGAGG
---AAAGATAAC---CCC
---GCT--

>XP_394391_Apis_mellifera_Prip
ATGGCA--

---AAT------------

---TTG
AGACAGGCACTGGGCGCGAGTGAACTTACC---GACAAAAAG---GCGGGCCTTTACCGC
GCGCTAGTTGCAGAATTTCTTGGCACGTTGTTGCTGAATTTCTTCGGTTGCGGATCTGTC
ATCACG--
--GGGAAC
GTCGTGGCTATAAGCTTGGCTTTCGGCCTGGCAGTGGCAACAGCGATTCAAGGAGTAGGA
CACGTTTCCGGTGGTCATGTTAATCCTGCTGTCACATTCGGTTTGATGGTTATTGGCAAG
GTGCCAGTTATTAGAGGCTTATTGTATGTAATACTTCAATGCATAGGTGCAATAGCGGGA

```
TCTGGTATATTAAGAGCATTATCTCCTGAAAGGATGGAGCAT------------------
------------------------------GCTCTAGGCGTAGTATCTCTCTCACCAGGC
---------GTTACCCCTGTCCAAGGTTTCGGTATAGAATTTTTCCTTGCCTTTGTGTTG
GTACTGGTCGTGTGCGGT---GCATGCGACGCAGCGAAGCCG------------------
---GACAGCAAG---------------GGAATAGCTCCTCTTATAATCGGACTTTCAGTT
TCCGTTGGCCATATTATCGGCGTGCCAAGAACTGGTGCAGGTATGAATCCAGCCAGATCA
TTCGGTAGCGCTGTTGTAATGGGT---------TCGTTCGCCGATCACTGGTTATATTGG
ATCGGACCAATACTTGGTGGAATGGCAGCTGGATTGATCTATGCGTTTGTAATTGGTCCT
---GCGAAAGAACCTGAAAAT------------------------TCCAATACA------
------------------------GCG------------TATACCGTT------GCCGCA
ACAGAT---GAG---AAGGAGCTACAGAGCCTT------ACCGGT---------AAGAAG
---------------------------------------------AAAGACAAT---CCT
---GCT------------------------------------------------------
------------------------------------------------------------
------------------------------------------------------------
------------------------------------------------------------
------------------------------------------------------------
------------------------------------------------------------
------------------------------------------------------------
------------------------------------------------------------
------------------------------------------------------------
------------------------------------------------------------
------------------------------------------------------------
------------------------------------------------------------
------------------------------------------------------------
------------------------------------------------------------
------------------------------------------------------------
------------------------------------------------------------
------------------------------------------------------------
------------------------------------------------------------
------------------------------------------------------------
------------------------------------------------------------
------------------------------------------------------------
------------------------------------------------------------
------------------------------------------------------------
------------------------------------------------------------
------------------------------------------------------------
------------------------------------------------------------
------------------------------------------------------------
------------------------------------------------------------
------------------------------------------------------------
------------------------------------------------------------
------------------------------------------------------------
------------------------------------------------------------
------------------------------------------------------------
------------------------------------------------------------
------------------------------------------------------------
------------------------------------------------------------
```


>XP_003701667_Megachile_rotundata_Prip
ATGTCG--

---AAC------------

---TTG
AAAGAGGCTCTGGGGCTCAGAGAACTTACC---GATAAGGAA---TCCGGCATTTATCGA
GCTCTGGTAGCGGAATTCTTTGGTACGATGCTGTTGAACTTCTTTGGCTGCGGATCGGTC
GTCACA--
--GAGAAC
GTTCTGACCATAAGCTTGGCCTTTGGTTTGACGGTTGCCGCCGCTGTTCAAGGAATAGGA
CATCTCTCCGGCGGCCATATTAATCCCGCTGTTACCTTCGGTTTAATGGTAATTGGCAAG
GTGCCAATAGTGAGGGGGTTGTTGTACGTGGTAGCTCAATGCATCGGAGCAATAGCAGGC
TCGGGTGTGTTAAGAGCATTATCCCCCGAAAGAATGGAAAGT------------------
------------------------------TTACTAGGGGTTGTGTCTCTTTCCATCGAT
---------GTTACACCTGTTCAAGGATTTGGTATAGAATTTTTCTTAGCCCTAATACTG
GTCTTCGTCGTTTGCGGA---GCCTGCGACTCGGCTAAAAAT------------------
---TACAGCAAA---------------GGAATTGCTCCGCTTGTAATCGGACTTACTGTT
TCGGTTGGACATATCGTTGGTGTGCCAAGAACAGGAGCAGGAATGAATCCAGCTCGATCA
TTGGGCAGCGCAGCGGTGATGAAC---------ATGTTCGATGACCACTGGTTGTACTGG
GTGGGTCCAATTCTCGGCGGAATGGCAGGAGCTCTGATTTACGTGTTCGTAGTCGGTCCT
---GCGAAGGAAGAGGAACTT------------------------CCTCGAAGA------
------------------------GTT------------TACGCTACT------GTTGCG
ACAGCC---GAA---AAA------------------------GGG---------GAACGG

>EFN88447_Harpegnathos_saltator_Prip
ATGGTG--

---AAT------------

---CTA
AAGAGCGCCTTGGGCGCTGACGAACTCACC---GACAAGAAG---GCCGGCCTTCCTCGC
GCCCTCCTGGCAGAATTCTTCGGCACTCTGCTGCTGAACTTTTTCGGCTGCGGCGCTGTA
GTGACT--
--GGAAAC
GTCGTCGCCATCAGTCTGGCCTTTGGTTTGACGGTGATGGCAGCGATTCAAGGAATAGGT
CACGTATCTGGGGGTCATGTGAATCCAGCGGTCACATTCGGCCTCCTCGTCGTCGGGAAG
GTGCCGGTGATCAGAGGCATCCTCTACGTCTTGGCGCAATGTGCGGGTGCCATAGCGGGA
TCAGCAATTCTGAGGGCACTCTCATCGGACGGGATGGAGGAC------------------
------------------------------GCCTTAGGGGTTGTGAGCCTCTCCCCCGGG
---------GTCAGCGCTGTCCAAGGATTCGGCGTGGAGTTCTTCCTCGCCCTGATCCTG
GTGCTGGTTGTTTGCGGC---GCGTGCGACGGCGCTAAGCCG------------------
---GACAGCAAG---------------GGCGTCGCGCCTCTTATAATCGGCCTCGCGGTC
ACCGTCGGCCACATTGTCGGCGTGCCGCGAACGGGCGCCGGAATGAACCCGGCGCGTTCA
TTGGGCAGCGCCGTGGTGATGGGC---------GCTTTCAAAGACCACTGGCTCTACTGG
GTAGGCCCGATCTTGGGCGGCATCGCTGGCGCGCTGCTCTACGTGCACGCGATCGGACCC
---GCCAAGGAGCCGGAAGTG------------------------CCCGCCAGG------
------------------------ACC------------TACGCCTCC------GTCGCC
ACCGAC---GAG---AAGGAGGTATATTACAGC------ACAAGA---------ATG---

>XM_012200640_Atta_cephalotes_Prip
ATGGTG--

---AAT------------

---CTG
AAGAACGCCTTGGGTGCTGATGAGCTTACC---GATAAGAAG---GTCGGTCTTCCTCGT
GCTCTTCTGGCAGAATTTTTAGGCACCTTATTACTGAACTTCTTCGGCTGCGGTGCCGCG
TTGACC--
--GGAGAT

GTTACCGCCATCAGCTTGGCCTTTGGTTTGACGGTAGCGTGCGCAATTCAAGGGATAGGA
CACGTCTCTGGAGGTCATGTCAATCCGGCGGTCACACTCGGTCTTGTCGTCATTGGGAAG
GTTCCAATTATTAGAGGCATTTTGTATGTTGTGGCGCAATGCGTGGGCGCTATCGCAGGT
TCAGCGATTCTAAGGGCTCTTTCGAATGACAAACTGGAGGAC------------------
------------------------------TTTCTAGGTGTGGTGAAGCTTGCTGAAGGG
---------ATTACTCCAGTGCAAGGATTCGGCGTGGAATTCTTCCTTGCGTTGATCTTG
GTGTTGGTAGTTTGCGGG---GTATGCGATGCCGCTAAACCG------------------
---GACAACAAA---------------GCCATCACGCCTCTCATAATCGGTGTGGCGGTC
ACTGTTAGTCATCTCGTCGGAGTACCGCGAACGGGTGCGGGGATGAATCCAGCACGTTCG
CTGGGCAGCGCCGTGGTGATGGGC---------GCTTTCAAAGATCATTGGGTGTATTGG
GTGGGTCCGATTATGGGCGGTATGGCCGGTGCACTCCTCTATGTGCATGCTATCGGACCC
---GCCAAGCAACCAGAAGCG------------------------CCCACCAGG------
------------------------ACC------------TATGCTTCC------GTCTCC
ACCGAG---GAA---AAGGAGGTATATTATACT------ACAAGA---------ATA---

>XR_004227896_Nasonia_vitripennis_Prip
ATGGCA--

---AGC------------

---CTT
CGGAATATCCTGGGAGCGCAGGAGCTCACG---GACAAGAAG---TCCAATCTGTACCGC
GCCCTCGTAGCCGAATTCCTGGGCACGATGTTGCTGAACTTCTTCGGCTGCGGTTCGGCC
ATCACC--
--GGTGAC
ACTCTCGCCATCAGCTTCTCCTTCGGCCTGACCGTCGCCGCGGTCATCCAAGCCATCGGA
CACGTTTCCGGCGGACACGTCAACCCGGCCGTCACTTTC---------------------
ATTCCGGTGATCCGTGGACTGCTGTACGTGATATCCCAGTGTTGCGGGGCGATCGCGGGC
TCAGCGGTGTTGCGAGCGCTGACCAGTCCGGAGAAGGAAATG------------------
------------------------------GTGCTCGGCGCTGTGGCGCTGAAGAACGGC
---------ACCGATGGCGCCAAGGGTATGGGCGTCGAGTTCTTCCTCGCGTTCATACTC
GTCCTCGTAGTCTGCGGT---GCCTGTGATCCTGGCAAACCG------------------
---GAAGCTAAG---------------CCACTGGCGCCGCTGATTATTGGCCTCGCCGTC
ACCGTTGGACACATCGTTGGTATCGAGCGAACGAGCACCGGCATGAACCCAGCACGAGCA
CTGGGCAGTGCGGTCGTCGTTGGC---------GAGTTCCCCGACCACTGGCTCTACTGG
GTAGGCCCAATCCTGGGAGGCGCCGCTGGTGCCCTTATCTACACGCATGTGATTGGCGCT
---GCCAAAGAGCCCGAATTG------------------------CCC---AGA------
------------------------TCG------------TACACGACC------GTCGCT
ACCGAG---GAG---AAAGAGAGACAT---------------------------------


```
------------------------------------------------------------
------------------------------------------------------------
------------------------------------------------------------
------------------------------------------------------------
------------------------------------------------------------
------------------------------------------------------------
------------------------------------------------------------
------------------------------------------------------------
------------------------------------------------------------
------------------------------------------------------------
------------------------------------------------------------
------------------------------------------------------------
------------------------------------------------------------
------------------------------------------------------------
------------------------------------------------------------
------------------------------------------------------------
------------------------------------------------------------
------------------------------------------------------------
------------------------------------------------------------
------------------------------------------------------------
------------------------------------------------------------
------------------------------------------------------------
------------------------------------------------------------
------------------------------------------------------
>HBDT01130127_Argulus_foliaceus_PripL
ATGACT------------------------------------------------------
---------------------------TCAAAACACTCTCGCTCTCCGAGTGGATCGCAT
CTCTCTTCCATCAGTGTGACCAACGAG---AATAACAAACTGGAGGGT------------
------------------------------------------------------------
---------------------------------------------------------GTC
AAGGGATTCCTGGGCCTGCAGGAGTGCAGCCAGAAGAATAGG------GACTTGTGGAGG
GCCGTTCTGGCCGAGTTTCTGGGTACTTTCCTATTGGTCTTTCTCGCCACACCATCATGC
ATAGGAAACTGGGATGAGGGG---------------------------------------
------------------------------------------------TACTCACCGTCT
ATTGTCCATATCACCCTGGCCTTTGGCCTGACTGTGGCAGCTATTGCTCAGGCTATTGGA
CACGTCAGTGGTTGCCACATAAATCCAGCCGTGACCATAGCGATGTTGGTAACAGCCAAA
GTCAGTCTTCTTAAAGCCTTTTGCTACATTGCAGCCCAGGTAGTTGGGGGAATCGCGGGC
TCAGCTATTGTGATGGCAATCACTCCAGAAAGCATTCAAGGA------------------
------------------------------AACTTGGGACTGACCACAATAGATCCAAAG
---------CTGACCAGCCTTCAAGGTTTCGGAGTTGAATTTTTCATTACTTTCATTCTC
GTTTTGGTGGTATTTGGT---TGCTGTGATGACAATAGAACT------------------
---GATTTGAAA---------------GGCTCAGCGCCTCTTGCCATTGGCCTAGCTGTG
TCAGTGGGACAGTTAACAGCTATCAAGTACACCGGTGCCAGCATGAACCCTGCCAGGACA
TTTGGGCCTGCAGTTATGTCCGGG---------ATCTGGGACAACCATTGGGTATACTGG
GTGGGTCCAATACTGGGTGGAGTCGTTGCCGCTCTCATCTATCACTTTCTTTTTGCTCAA
---AAACGGCTGACCAGCACT---------------GAAGTTTACGAGCTTCAG------
------------------------AAC------------CATGGGGTC------AAACCT
ATCGAT---TCAATC------TATTCGCCATCT------TCG------------GCCTCA
```

--CAGCCTGGCGCT
ACCTCA--

>JW950393_Argulus_siamensis_PripL
ATGGCG--
---------------------AGATCTTCAAACACATCTCGCGCTCCGAGTACATCGCAC
CTCTCCTCCGACAGTGTGGCTGCCGACAAAACCAACAAGCTGGAGGGT------------

---CTG
AAAGGATTTCTTGGACTGCAAGAATGCAGTCAGAAAAATAGG------GATTTGTGGAGA

GCTGTCCTGGCCGAGTTCCTTGGGACGTTCCTTCTTGTCTTCCTCGCCACCCCTTCTTGC
CTAGCCAACTGGGAAGATGGG---------------------------------------
--TACGCCCCCACC
GTCGTGCATATAACATTAGCCTTCGGTCTAACCGTTGCAGGAATCGCTCAGGCCATTGGA
CATGTCAGTGGATGCCACATAAACCCAGCTGTCACTATTGCAATGTTGGTGACTGCAAAG
GTTAGCTTGATCAAAGCATTTTGCTATATTGTTGCCCAAATTGTTGGGGGCATCGCAGGG
TCAGCAGTTGTTAAGGCAGTTACACCTGACGAAATCCAGGGA------------------
------------------------------AACCTTGGTCTAACGATGATTAGCGATAAA
---------CTCACCAGTTTGCAGGGATTCGGGGTGGAGTTCTTCATCACTTTCATCTTG
GTGCTGGTCGTCTTTGGA---GTTTGTGATGACAATAGAAAT------------------
---GACCTCAAA---------------GGGTCAGGTCCTTTGGCAATCGGCTTGACTATC
TCAGTTGGACAACTCACTGCCGTGAAATATACTGGAGCGAGTATGAATCCCGCCAGAACG
TTTGGACCAGCCGTTATGACAGGA---------ATATGGGATCATCACTGGGTATACTGG
GTCGGTCCCATCTTGGGTGGGGTCATAGCTGCTTTACTTTATCATTTTCTGTTTGCTAAA
---AAGCGACTAACCAACGCC---------------GATGAGTACGAACTTCGA------
------------------------GAA------------CAGAGCGTT------CGACCT
ATTGAC---ACTGTT------TATTCACAAACA------TCT------------TTGCCT
--CACCCAGGCGCC

>XM_046589641_Daphnia_pulex_EglpL
ATGGAA--

------------------------------------TTTTGGGCGAGA------------

---ATG
CGCAACGTATTCGGCTGCGATGAGCTCAAC---AAGAACCGC------GACATCTGGCGC
ATGCTCATGGCCGAATTCGTCGGCCCGCTCTTTCTCGTCCTCATCGGCTGCGCTTCCTGC
GTCGAGGGATGGAACGACCAG---------------------------------------
--TACTCGCCGCAC
ATTGTCCAGGTCGCCCTCTCCTTCGGCGTCACCATCGCAACAATGGCCCAGGCCTTGGGA
CATGTGAGCGGAGGCCATTTCAACCCGGCCGTGACGGTGGCCTGTCTCGTGACCGGCAAA
ATCTCCATCGTCAAGTCCATCTTCTACATCGTTGCCCAGTGCCTGGGCGCCATATGTGGA
GCTGCTCTTCTCCAGGCTCTGACGCCGAGCGATTTCCACAAT------------------
------------------------------ACGTTGGGCGTGACGGAGATCCACAAAGCC
---------CTGACCCCGACGCAGGGCTTTGGCGTCGAGTTCTTCTCGACGTTCACGCTG
GTCCTGGTCGTCTTTGGC---GTCTGCGACGAGAACCGCAAA------------------
---GACGTCAAA---------------GGATCGGCCCCGTTGGCCATCGGCCTCTGCATC
GCCACCGCCATTTTGGCCACCGGTAATTACACTGGTGGTAGCTTGAATCCAGCCCGCTCC
CTAGGACCGGCAGTCATCAGCAAC---------AAGTGGGCTTATCACTGGGTCTACTGG
GCGGGACCGATCGTCGGTGGCGTAGTGGCAGCACTCACCTACCAGAAGGCCTTCAAGGCC
---CGTTCGCCC---------------------GAAGAGGAAGTC---------------
------------------------GAGCTG---------GAATCCTAC------CAGTAC
CGAGTC---GCC---AACTCGAAAGAGAGTGAG------ATT------------ATCGCC
--GACCGAACGACC
ACCATC--

>XM_046802164_Daphnia_pulicaria_EglpL
ATGGAA--

------------------------------------TTTTGGGCGAGA------------

---ATG
CGCAACGTATTCGGCTGCGATGAGCTCAAC---AAGAACCGC------GACATCTGGCGC
ATGCTCATGGCCGAATTCGTCGGCCCGCTCTTTCTCGTCCTCATCGGCTGCGCTTCCTGC
GTCGAGGGATGGAACGACCAG---------------------------------------
--TACTCGCCGCAC
ATTGTCCAGGTCGCCCTCTCCTTCGGCGTCACCATCGCAACAATGGCCCAGGCCTTGGGA
CATGTGAGCGGAGGCCATTTCAACCCGGCCGTGACGGTGGCCTGTCTCGTGACCGGCAAA
ATCTCCATCGTCAAGTCCATCTTCTACATCGTTGCCCAGTGCCTGGGCGCCATATGTGGA
GCTGCTCTTCTCCAGGCTCTGACGCCGAGCGATTTCCACAAT------------------
------------------------------ACGTTGGGCGTGACGGAGATCCACAAAGCC
---------CTGACCCCGACGCAGGGCTTTGGCGTCGAGTTCTTCTCGACGTTCACGCTG
GTCCTGGTCGTCTTTGGC---GTCTGCGACGAGAACCGCAAA------------------
---GACGTCAAA---------------GGATCGGCCCCGTTGGCCATCGGCCTCTGCATC
GCCACCGCCATTTTAGCCACTGGTAATTACACTGGCGGCAGCTTGAATCCGGCCCGCTCC
CTAGGACCGGCAGTCATCAGCAAC---------AAGTGGGCTTATCACTGGGTCTACTGG
GCGGGACCGATCGTCGGTGGCGTAGTGGCAGCACTCACCTACCAGAAGGCCTTCAAGGCC

---CGTTCGCCC---------------------GAAGAGGAAGTC---------------
------------------------GAGCTG---------GAATCCTAC------CAGTAC
CGAGTC---GCC---AACTCGAAAGAGAGTGAG------ATT------------ATCGCC
--GACCGAACGACC
ACCATC--

>JAACYE010000007_Daphnia_obtusa_EglpL
ATGGAA--

------------------------------------TTTTGGGCGAGA------------

---ATG
CGCAACGTATTCGGCTGCGATGAGCTCAAC---AAGAACCGC------GACATCTGGCGC
ATGCTCATGGCCGAATTCGTCGGCCCGCTCTTTCTCGTCCTCATCGGCTGCGCTTCCTGC
GTCGAGGGATGGAACGACCAG---------------------------------------
--TACTCGCCGCAC
ATCGTCCAGGTCGCCCTCTCCTTCGGCGTCACCATCGCAACAATGGCCCAGGCCTTGGGG
CATGTGAGCGGAGGCCATTTCAACCCGGCCGTGACGGTGGCCTGTCTCGTGACCGGCAAA
ATCTCCATCGTCAAGTCCATCTTCTACATCATTGCCCAGTGCCTGGGCGCCATATGTGGA
GCTGCTCTTCTGCAGGCTCTGACGCCGACCGATTTCCACAAT------------------
------------------------------ACGTTGGGCGTGACGGAGATACACAAAGCC
---------CTGACCCCGACCCAGGGCTTCGGCGTCGAGTTCTTCTCGACGTTCACACTG
GTCCTAGTCGTCTTCGGC---GTCTGCGATGATAACCGCAAA------------------
---GACGTCAAA---------------GGATCGGCCCCGTTGGCCATCGGTCTCTGCATC
GCCACCGCCATTTTGGCCACCGGTAATTACACTGGCGGCAGCTTGAATCCGGCCCGTTCC
CTCGGACCGGCAGTCATCAGCAAC---------AAGTGGACTTATCACTGGGTGTACTGG
GCGGGACCGATCGTCGGTGGCGTAGTGGCGGCACTCACCTACCAGAAGGCATTCAAGGCC
---CGCTCGCCC---------------------GAAGAGGAAGTC---------------
------------------------GAGCTG---------GAATCCTAC------CAGTAC
CGAGTC---GCC---AACTCGAAAGAGAGCGAG------ATT------------ATCGCC
--GACCGAACGACC
ACCATC--


```
------------------------------------------------------------
------------------------------------------------------------
------------------------------------------------------------
------------------------------------------------------------
------------------------------------------------------------
------------------------------------------------------------
------------------------------------------------------------
------------------------------------------------------------
------------------------------------------------------------
------------------------------------------------------------
------------------------------------------------------------
------------------------------------------------------------
------------------------------------------------------
>HAFN01000200_Daphnia_galeata_EglpL
ATGGCA------------------------------------------------------
------------------------------------------------------------
------------------------------------TTTTGGGCGAGA------------
------------------------------------------------------------
---------------------------------------------------------ATG
CGCAACGTATTCGGCTGCGATGAGCTCAGC---AAGAACCGC------GACATCTGGCGT
ATGCTCATGGCCGAGTTCGTCGGGCCGCTCTTTCTCGTCCTCGTCGGCTGCGCTTCCTGT
GTCGAAGGATGGAACGATCAG---------------------------------------
------------------------------------------------TACTCACCGCAC
ATCGTGCAGGTCGCCCTCTCCTTCGGCATCACCATCGCAACAATGGCCCAGGCCTTGGGA
CATGTGAGCGGAGGGCATTTCAACCCGGCCGTGACGGTGGCCTGTCTCGTGACCGGCAAA
ATTTCCATCGTCAAGTCCATCTTCTACATCATTGCCCAGTGTTTGGGCGCCATATGTGGA
GCTGCTCTTCTACAGGCTCTGACGCCGACCGAGTTCCACAAC------------------
------------------------------ACGTTGGGCGTGACGGAGGTACACAAAGCC
---------CTGACGCCGACACAGGGTTTCGGTGTTGAGTTTTTCTCTACATTCACACTT
GTCCTAGTCGTCTTTGGT---GTATGCGACGATAACCGCAAA------------------
---GATGTCAAA---------------GGATCGGGCCCACTGGCCATCGGCCTCTGTATT
GCCACCGCCATTTTGGCCACTGGTAATTACACTGGCGGCAGCTTGAATCCGGCCCGCTCT
CTCGGACCGGCAGTCATCAGCAAC---------AAGTGGAATTATCACTGGGTGTACTGG
GCAGGACCAATCGTCGGTGGCGTAGTTGCAGCTCTTACCTACCAGAAGGCATTCAAGGCC
---CGTTCTCCC---------------------GAAGAAGAAGTG---------------
------------------------GAATTG---------GAATCCTACCAC---CAATAC
CGCATA---GCC---AATTCGAAAGAGAGTGAG------ATC------------ATCGCC
------------------------------------------------GACCGAACGACT
ACCATC------------------------------------------------------
------------------------------------------------------------
------------------------------------------------------------
------------------------------------------------------------
------------------------------------------------------------
------------------------------------------------------------
------------------------------------------------------------
------------------------------------------------------------
------------------------------------------------------------
------------------------------------------------------------
```


>JAAVJA010000733_010012108_Daphnia_dubia_EglpL
ATGGCA--

------------------------------------TTTTGGGCGAGA------------

---ATG
CGCAACGTATTCGGCTGCGATGAGCTCAGC---AAGAACCGC------GACATCTGGCGT
ATGCTCATGGCCGAGTTCGTTGGGCCGCTCTTTCTCGTCCTCGTCGGCTGCGCTTCCTGT
GTTGAAGGATGGAACGATCAG---------------------------------------
--TACTCACCGCAC
ATCGTCCAGGTCGCCCTCTCCTTCGGCGTCACCATCGCAACAATGGCCCAGGCCTTGGGG
CATGTGAGCGGAGGCCATTTCAACCCGGCCGTGACGGTGGCTTGTCTCGTGACCGGCAAA
ATCTCCATCGTCAAGTCCATCTTCTACATCATTGCCCAGTGCTTGGGCGCCATATGTGGA
GCTGCTCTTCTG---GCTCTGACGCCGACCGAGTTTCACAAT------------------
------------------------------ACCTTGGGCGTGACGGAGGTACACAAAGCC
---------CTGACTCCGACCCAGGGTTTCGGCGTCGAGTTCTTCTCGACATTCACACTC
GTCCTGGTCGTCTTTGGT---GTCTGCGATGAGAACCGAAAA------------------
---GACGTCAAA---------------GGATCGGCTCCATTGGCCATCGGCCTGTGTATT

GCCACCGCCATTTTGGCCACTGGCAATTACACTGGTGGCAGCTTGAATCCGGCCCGTTCT
CTCGGCCCGGCAGTCATCAGCAAC---------AAGTGGAATTACCACTGGGTATACTGG
GCAGGGCCGATCGTCGGTGGAGTAGTGGCAGCTTTGACCTACCAAAAGGCTTTCAAGGCT
---CGTTCACCC---------------------GAGGAGGAAGTC---------------
------------------------GAGTTG---------GAATCCTACCAC---CAGTAC
CGAGTC---GCC---AATTCGAAAGAGAGCGAG------ATC------------ATCGCC
--GACCGAACGACT
ACCATC--

>CM023197_Daphnia_carinata_EglpL

ATGGCA--

------------------------------------ATCTGGCAGAGA------------

---ATG
CGCAACGTATTCGGCTGCGATGAGCTCAGC---AAGAACCGC------GACATCTGGCGG
ATGCTCATGGCCGAGTTCGTCGGCCCACTCTTTCTCGTCCTCATCGGCTGTGCTTCCTGC
GTCGAAGGATGGAACGACCAG---------------------------------------
--TACTCACCGCAC
ATCGTCCAGGTCGCTCTCTCCTTCGGCGTCACCATCGCCACAATGGCGCAGGCCTTGGGT
CATGTGAGTGGAGGCCATTTCAACCCGGCCGTGACGGTCGCTTGTCTTGTGACGGGCAAG
ATTTCCATCGTCAAGTCCATCTTCTACATCGTGGCCCAGTGTCTGGGCGCCATATGTGGA
GCTGCTCTTTTGCAGGCATTGACTCCGACTGATTTCCACAAC------------------
------------------------------ACGTTGGGCGTGACAGAGATCCACAAGGCC
---------CTGACTCCGACGCAGGGTTTCGGCGTGGAATTCTTCTCCACGTTCACGCTC
GTCCTCGTCGTCTTCGGT---GTCTGCGATAGCAATCGCAAA------------------
---GACGTCAAA---------------GGATCGGCACCATTGGCTATCGGCCTCTGCATC
GCCACCGCCATCTTGGCCACCGGAAACTACACGGGTGGCAGTTTGAATCCGGCTCGATCC
CTCGGCCCAGCAGTCATCAGCAAC---------AAGTGGAGTTATCACTGGGTTTACTGG
GCAGGACCAATCATCGGTGGTGTGGTGGCCGCCCTTCTCTATCAGAAGGTTTTCAAAGCG
---CGTTCGGCC---------------------GAAGAAGAAAGC---------------
------------------------GAACTG---------GAATCTTAC------CAGTAC
CAGGCG---GCC---AATTCAAAGGAAAGCGAA------ATT------------ATCGCT
--GATCGAACCACG
ACCATC--

>XM_032941243_Daphnia_magna_EglpL
ATGGCA--

------------------------------------ATCTGGCAGAGA------------

---ATG
CGCAACGTATTCGGCTGCGATGAGCTTAGC---AAGAACCGC------GACATCTGGCGG
ATGCTCATGGCCGAGTTCGTCGGCCCACTCTTTCTCGTCCTCATCGGATGTGCTTCCTGC
GTCGAAGGATGGAACGACCAG---------------------------------------
--TACTCACCGCAC
ATCGTCCAGGTCGCTCTCTCCTTTGGCGTCACCATCGCCACAATGGCGCAGGCCTTGGGT
CATGTGAGTGGAGGCCATTTCAACCCAGCCGTGACGGTGGCCTGTCTTGTGACGGGAAAG
ATTTCTATCGTCAAGTCCATCTTCTACATCTTGGCACAGTGCTTAGGCGCCATATGTGGA
GCTGCTCTTTTGCAGGCATTGACTCCGACCGAGTTCCACAAC------------------
------------------------------ACACTGGGCGTGACGGAGATTCACAAAGCC
---------CTGACTCCGACGCAGGGTTTCGGTGTGGAATTCTTCTCTACGTTCACGCTC
GTCCTCGTCGTCTTCGGT---GTCTGCGATAGCAATCGCAAA------------------
---GACGTCAAA---------------GGATCGGCACCATTGGCCATCGGCCTCTGCATC
GCCACCGCCATTTTGGCCACCGGAAACTACACGGGTGGTAGTTTGAATCCGGCTCGATCC
CTCGGTCCGGCAGTCATCAGCAAC---------AAGTGGAGTTATCATTGGGTTTACTGG
GCAGGACCAATCATCGGTGGTGTGGTGGCCGCCCTTCTCTACCAGAAGGTTTTTAAAGCC
---CGTTCGGTC---------------------GAAGAAGAAATC---------------
------------------------GAATTG---------GAATCTTAC------CAGTAC
CATGTG---GCC---AATTCAAAGGAGAGCGAA------ATT------------ATCGCT
--GATCGAACCACG
ACCATC--

>JAKUCG010000213_Ceriodaphnia_dubia_EglpL
ATGGCA--

------------------------------------TTTTGGGCGAGA------------

---ATG
AAGAGCGTATTCGGTTGCGATGAGCTCAGC---AAGAACCGC------GACATCTGGCGA
ATGCTCATGGCCGAATTCGTCGGCCCACTCTTCCTCGTCCTCATCGGCTGCGCTTCTTGC
GTCGAGGGATGGAACGAGCAA---------------------------------------
--TATTCACCACAC
ATCGTTCAGGTCGCCCTATCCTTCGGCGTCACCATAGCCACAATGGCACAGGCCTTGGGA
CATGTTAGCGGTGGCCACTTCAACCCTGCCGTGACGGTGGCCTGCCTCGTGACCGGCAAA
ATCACCATCGTCAAGTCCATCTTCTACATAATAGCCCAATGTTTGGGCGCCATATGTGGA
GCTGCTCTTCTCCAGGCTTTGACGCCGAGCGAGTTCCACAAC------------------
------------------------------ACACTGGGCGTGACGGAAGTGCACAAAGCC

---------CTGACGCCGACGCAGGGATTCGGCGTCGAGTTCTTCTCCACATTCACCCTG
GTGCTGGTCGTTTTCGGC---GTTTGCGACGAGAACCGCAAA------------------
---GATGTCAAA---------------GGATCGGCCCCATTAGCCATCGGCCTCTGCATC
GCTACCGCCATTTTGGCCACCGGTAACTACACAGGCGGCAGCTTGAATCCGGCCCGTTCC
CTGGGACCAGCAGTCATCAGCAAC---------AAATGGGCTTACCACTGGGTGTATTGG
TCTGGACCGATCGTCGGTGGTATTGTTGCTGCTTTGCTCTACCAAAAAGCCTTCAAGGCT
---CGTTCGGCA---------------------GATGAAGAACAG---------------
------------------------GAGCTT---------GAATCCTAT------CAGTAT
CGCGCT---GCC---AACTCGAAAGAGAGTGAA------ATT------------ATCGCC
--GACCGAACCACT
ACTATT--

>GGQP01006117_Diaphanosoma_celebensis_EglpL1
ATGGCC--

------------------------------------ATTTGGGCGAAG------------

---ATG
CGCGCAATCTTCGGCTGCGATGAACTGTCC---AAGAACCGC------GACATCTGGCGA
ATGCTCATCGCCGAATTCGTCGGCACCATGATGCTCGTCCTCATCGGATGCGCCTCCTGC
GTCGAAGGATGGAACGAGCAA---------------------------------------
--TATTCCGTCTCG
CTGGTCGCCGTCGCCCTCACGTTCGGCATCACCATCGCCACCTTGGCTCAGGTGATCGGA
CATGTTAGCGGTTGTCACATCAATCCGGCCGTCTCCATCAGCTTGGCCACGACGGGCAAA
ATGCCCATCGTCCGGGCCATTTTCTACATCGCTTCGCAGTGCGTCGGCGCCATTTGCGGC
TCTGCTCTCCTGCAGTCTCTGACTCCGGAGAAATTCCACAAC------------------
------------------------------ACCATGGGCGTCACGTCGGTTCACGAGTCC
---------GTGACACCGACGCAGGCCTTCGGAGTCGAGTTCTTCGGCACGTTCATCCTG
GTGCTGATCGTCTTCGCG---CTCACCGACGAGAACCGTAAA------------------
---GATGTTCAT---------------GGAACGGCGCCGATGGCCATCGGCTTGACCGTC
ACTGCCGTAGTCTGCGCCATCGGAATGTACAGTGGCGCCAGTTTGAACCCGGCGCGTTCA
TTAGGACCATCCGTCGTCACCGGC---------AAATGGGATCTTCACTGGGTCTACTGG
GCAGGGCCGATCGTCGGCGCAATCGTGGCGGCACTGCTCTACCAGAAGGTCTTCCGGAGC
---CGCTCGCCC---------------------GAAGAGGAGCAGCAGTTC---------
------------------------GAA------------GAC---TAC------AAATAC
CGCGCC---GCC---GACGTCAAGGAGAACGAA------GTG------------ATCGCC
--GATCGAACCACC
ACCATC--


```
------------------------------------------------------------
------------------------------------------------------------
------------------------------------------------------------
------------------------------------------------------------
------------------------------------------------------------
------------------------------------------------------------
------------------------------------------------------------
------------------------------------------------------------
------------------------------------------------------------
------------------------------------------------------------
------------------------------------------------------------
------------------------------------------------------------
------------------------------------------------------------
------------------------------------------------------------
------------------------------------------------------------
------------------------------------------------------------
------------------------------------------------------------
------------------------------------------------------------
------------------------------------------------------
>GGQP01085796_Diaphanosoma_celebensis_EglpL2
ATGGCC------------------------------------------------------
------------------------------------------------------------
------------------------------------ATTTGGGAAAAA------------
------------------------------------------------------------
---------------------------------------------------------ATG
CGATCGGTCCTCGGCTGCAAGGAATTCTCC---AAGAACCGC------GACATTTGGCAA
ATGCTGATCGCCGAATTCGTTGGTACCATGTTCCTTGTGCTCATTGGCTGCGCTGCTTGT
GTCGAAGGCTGGAGCGAGCAA---------------------------------------
------------------------------------------------TATTCGTCCAGC
ATCGTCCAAGTCGCCTTATCTTTCGGTGTCACCATCGCCACAATGGCTCAGACCATCGGC
CATGTCAGCGGCTGTCACATCAACCCTGCGGTGACAACGGCGATGATGATCACAGGCAAA
ATCAGCGTCATGAGGGCCATTGCGTACGTCGTTTCGCAATGCATCGGCGCCATTTGTGGA
GCAGCTCTTCTCCAGTCCCTGACACCGGAGAAGTTCCACAAC------------------
------------------------------ACGCTGGGTGTCACCACGGTCCACGAATTC
---------CTCACCCCGACCCAGGGCTTCGGCGTGGAGTTTTTCACCACTTTCACCTTG
GTGCTGATCGTTTTCGGC---GTCAGCGATGAAAACCGCACC------------------
---GATGTCAAG---------------GGCTCGGCCCCTTTGGCCATTGGGCTGACTGTC
ACCACGGCCATTTTGGCCACGGGAATGTATACCGGCGGCAGCTTGAATCCGGCGCGCTCC
TTGGGTCCGGCAGTCGTCACTGGC---------AAATTTGCCCTCCACTGGGTCTACTGG
GCGGGTCCGATCGTCGGTGGCATGGTGGCCGCCCTGCTCTACGAGAAAGTCTTCCGTGCC
---CGTTCCGCC---------------------GAGGAGGAGCAAGAATGGCGC------
------------------------GAAATG---------GAGGACTAT------CAGTAC
CGCATT---GCC---AACGCCAAGGAGACCGAA------ATC------------ACCGGC
------------------------------------------------GAACGAGTCGTC
ACCGTCGATGTTCAGAAGCAC---------------------------------------
------------------------------------------------------------
------------------------------------------------------------
------------------------------------------------------------
```


>RJJA01000114_Lepidurus_arcticus_EglpL1

---ATGATTTGCTTGTTCAGGGAAACGACAACGCATGCCTGGGCTGGT------------

---TTC
AAAAGTTCACTGCAATGTTGCGACTTCAAC------AGCCAT------GAGCTATGGAAG
TGCTTGCTAGCGGAATTCATTGGCACTTTGTTTCTCGTGTTTTCTGGCTGTGCTGCATGT
TCTGAAGGTTGGAACGAATCC---------------------------------------
--TACTCGCCAACA
ATGGTTCAAGTGGCTCTTTGTTTTGGTATCACCGTCGCTACCATGGTGCAGGCGATCGGT
CATATAAGTAAATGCCATATCAACCCGGCGGTAACTACGTCGATGATGCTGGTAGGCAAG

ATTACGGTGCTGAAAGGACTAGCTTATGTCATTGCACAATGCACAGGCGGTATCGCAGGA
GTTGCTGTTTTGCAGATTTTGACACCTCCGGAATTTCGCAGC------------------
------------------------------ACTTTAGGCGTTACGGAACTGCACAACAAA
---------GTGACACCATCGCAAGGGTTTGGTATCGAATTTTTCGCTACTTTTGTGCTT
GTGCTTATCATCTGCAGT---GTATGTGACGAAAATCGCACT------------------
---GATGTTGGA---------------GTATTAGCACCCTTAGCCATCGGCCTTACTGTG
TCGTCACAAGTTTTAGCTATTGGAATGTATACTGGAGGTAGTATGAATCCAGCGCGATCG
TTAGGTCCTGCAGTGCTTCTTAAC---------AAATGGAGTTGCCATTGGGTGTATTGG
GTTGGTCCCGTCATTGGCGGCGGAGTAGCTGCATTAGTTTACCAACGGGCTTTTGCAGCT
---CCTAGC---AAA---

>JAGJRP010000017_Lepidurus_couesii_EglpL1
ATGATC--
---CAGAGGTCT
AACATGATTTGCTTGTTCAGGGAAACGACAACGTATGCCTGGGCTGGT------------

---TTC
AAAAGTTCACTGCAATGTTGCGACTTCAAC------AGCCAT------GAGCTATGGAAG
TGCTTGCTAGCGGAATTCATTGGCACTTTGTTTCTCGTGTTTTCTGGCTGTGCTGCATGT
TCTGAAGGTTGGAACGAATCC---------------------------------------
--TACTCGCCAACA
ATGGTTCAAGTGGCTCTTTGTTTTGGTATCACCGTCGCTACCATGGTGCAGGCGATCGGG
CATATAAGTAAATGCCACATCAACCCGGCGGTAACTACGTCGATGATGCTGGTAGGCAAG
ATTACGGTGGTGAAAGGACTAGCTTATGTCATTGCACAATGCACAGGCGGTATCGCAGGA
GTTGCTGTTTTGCAGATTCTGACACCTCCGGAATTTCGCAGC------------------
------------------------------ACTTTAGGCGTTACGGAACTGCACAACAAA
---------GTGACACCATCGCAAGGGTTTGGTATCGAATTTTTCGCTACTTTTGTGCTT
GTGCTTATCATCTGCAGT---GTATGTGACGAAAATCGCACT------------------
---GATGTTGGA---------------GTATTAGCACCCTTAGCCATCGGCCTTACTGTG
TCGTCACAAATTTTGGCTATTGGAATGTATACTGGAGGTAGCATGAATCCAGCGCGGTCG
TTAGGTCCTGCAGTGATTCTTAAC---------GAATGGAGTTGCCATTGGGTGTATTGG
GTTGGTCCCATCATTGGCGGCGGAGTAGCTGCATTAGTTTACCAATGGGCTTTTGCAGCT
---CCTAGC---AAA---

>JAKZAN010000001_Lepidurus_packardi_EglpL1
ATGATC--
---CAA---TCT
AACATGATTTGCTTGTTCAGAGAAACGACAACGTATTCCTGGGCTGGT------------

---TTC
AAAAATTCACTACGATGTTGCGACTTCAAC------AGCCAT------GAGCTATGGAAG
TGCTTGCTAGCAGAATTCATTGGCACTTTGTTTCTCGTGTTTTCTGGCTGTGCTGCATGT
TCTGAAGGTTGGAACGAATCC---------------------------------------
--TACTCGCCAACA
ATGGTTCAAGTGGCTCTTTGTTTTGGTATCACCGTCGCTACCATGGCGCAGGCGATTGGT
CATATAAGTGGATGCCATATCAACCCGGCGGTAACTACGTCGATGATGCTGGTAGGCAAG
ATTACGGTGCTGAAAGGACTAGCTTATGTTATTGCACAATGCACAGGTGGTATCGCAGGA
GTTGCTGTTTTGCAGATTTTGACACCTCCGGAATTTCGCAGC------------------
------------------------------ACTTTAGGCTTTACGGAGCTGCACAACAAA
---------GTGACACCATCGCAGGGGTTTGGTATCGAATTTTTCGCTACGTTTATGCTT
GTGCTAATCATCTGCAGT---GTATGTGACGAAAATCGCACG------------------
---GATATTGGA---------------GGATTAGCCCCCTTAGCCATAGGCTTTACTGTG
TCGTCACAGGTTTTGGCTATTGGAATGTATACTGGAGGTAGCATGAATCCAGCGCGATCG
TTAGGTCCTGCAGTGATTCTTAAC---------AAGTGGAGTTGCCATTGGGTGTATTGG
GTTGGTCCCGTCATTGGCGGTGGTGCATCTGCATTAGTTTACCAACGGGCTTTTGCAGCT
---CCTAGC---AAA---

>RJJB01000085_Lepidurus_apus_EglpL1
ATGCTC--
---CAG---TCT
ACCATGATTTGCTTGTTCAGAGAAACGACAACGTACTCCTGGGCTGGT------------

---TTC
AAAAGTTCACTGCGATGTTGCGACTTCAAC------AGCCAT------GAGCTATGGAAG
TGCCTGCTAGCAGAATTCATCGGCACTTTGTTTCTCGTGTTTTGTGGCTGTGCCGCATGT
TCTGAAGGTTGGAACGAATCC---------------------------------------

--TATTCGCCAACA
ATGGTTCAAGTGGCTCTTTGTTTTGGTATCACCATCGCTACCATGGCGCAGGCGATTGGT
CATATTAGTGGATGCCATATCAACCCGGCGGTAACTACGTCGATGATGGTGGCAGGCAAG
ATTGCGGTACTGAAAGGACTAGCTTATATCATTGCCCAATGCACAGGTGCTATCGCAGGA
GCTGCTGTTTTGCAGATTTTGACACCTCCGGAATTCCGCAGC------------------
------------------------------ACTTTAGGCGTTACGGAGCTGCACAGCAAA
---------GTGACACCATCGCAAGGGTTTGGTATCGAATTTTTCGCTACTTTTGTGCTT
GTGCTTATCATCTGCAGT---GTATGTGACGAAAATCGCACT------------------
---GATATTGGA---------------GGATTAGCACCCTTAGCAATCGGACTTACTGTG
TCGTCACAGGTTCTGGCTATTGGAATGTATACTGGAGGAAGCATGAATCCAGCGCGATCG
TTAGGTCCTGCAGTGATTCTTAAC---------AATTGGAGTTGCCATTGGGTGTACTGG
GTTGGTCCCATCATTGGTGGTGGCGTAGCTGCCCTAGTGTACCAACGGGCTTTTACTGCC
---CCTAGT---AAA---

>JAGQDJ010000046_Triops_longicaudatus_EglpL
ATGGGA--

------------------------------------GGCTGGGACAGC------------

---ATG
AAGAACGCGTTCGGTTGCGGCGAATTCAAC------AACGGT------GAATTGTGGAAG
TCCCTTTTGGCGGAGTTTATCGGCACCTTATTTCTCGTGCTCGTCGGCTGTGCATCTTGT
TCACAAGGTTGGAATGAAAGC---------------------------------------
--TACTCTCCAACT
ATGGTTCAAGTCGCCCTTTCCTTCGGTGTCACCATCGCTACTATGGCGCAGGCGATTGGC
CATGTCAGTGGTTGTCATATTAATCCCGCAGTCACTACGTCTTTGATGATAGTGGGCAAG
ATTTCTCTGCTGAAAGCAATTTTCTATATCGCAGCCCAGTGTGTTGGAGCCATTTCAGGG
GCTGCAATCTTACAGGCTCTGACACCTGCGGAGTTCCACAGT------------------
------------------------------ACATTAGGTGTTACTGAACTACACGCCAAG
---------TTGTCAGCCACTCAAGGCTTTGGAGTCGAGTTTTTCGCCACTTTTACTCTT
GTTCTGATCGTCTGTGGC---GTTTGTGACGAAAATCGCAAT------------------
---GATATTAAA---------------GGTTCTGCTCCCTTGGCAATTGGCCTCACCGTT
ACAACGCAGATTTTGGCTATTGGAATGTATACAGGAGGAAGCTTGAATCCAGCAAGATCT
TTAGGTCCGGCCGTGATCCTGAAC---------AAGTGGGACAGCCATTGGGTGTACTGG
GTGGGTCCCATGGTGGGAGGTGCTGTAGCTTCCCTGTTATATCAGAGAGCCTTCACAGCG
---CCGAGTAACAAGCGGGAGCCT---------GACGAGGAGGAA---CCG---------
------------------------GAC------------TACCCTTAT------CGGTAC
CGGGCG---ACG---AACGATAAAGAGAAGGAA------ATA------------ATCGCA
--GATAGGACAACA
TCAATC--

>GEHY01012481_Triops_newberryi_EglpL
ATGGGA--

------------------------------------GGCTGGGACAGC------------

---ATG
AAGAACGCGTTCGGTTGCGGCGAATTCAAC------AACGGT------GAATTGTGGAAG
TCCCTTTTGGCGGAGTTTATCGGCACCTTATTTCTCGTGCTCGTCGGCTGTGCATCTTGT
TCACAAGGTTGGAATGAAAGC---------------------------------------
--TATTCTCCAACT
ATGGTTCAAGTCGCCCTTTCCTTCGGTGTCACCATCGCTACTATGGCGCAGGCGATTGGC
CATGTCAGTGGTTGTCATATTAATCCCGCAGTCACTACGTCCATGATGATAGTGGGCAAG
ATTTCTCTGCTGAAAGCAATTTTCTATATTGCAGCCCAATGTGTTGGAGCCATTTCAGGG
GCTGCAATCTTACAGGCTCTGACACCTGCAGAGTTCCACAGT------------------
------------------------------ACATTAGGTGTTACTGAACTACACCCCAAG
---------TTGTCAGCCACTCAAGGCTTTGGAGTCGAGTTTTTCGCCACTTTTACTCTT
GTTCTGATCGTCTGTGGC---GTTTGTGACGAAAATCGCAAT------------------
---GATATTAAA---------------GGTTCTGCTCCCTTGGCAATTGGCCTCACCGTT
ACAACGCAGATTTTGGCTATTGGAATGTATACAGGAGGAAGTTTGAACCCAGCAAGATCT
TTAGGTCCGGCCGTAATCTTAAAC---------AAGTGGGACAGCCATTGGGTGTACTGG
GTGGGTCCCATGGTGGGAGGTGCTGTAGCTTCCCTGTTATACCAGAGAGCCTTCACAGCG
---CCGAGTAACAAGCGGGAGCCT---------GACGAGGAGGAA---CCG---------
------------------------GAC------------TACCCTTAT------CGGTAC

CGGGCG---ACG---AACGATAAAGAGAAGGAA------ATA------------ATTGCA
--GATAGGACAACA
TCAATC--

>GCAT01014384_Triops_cancriformis_EglpL
ATGGGA--

------------------------------------GGCTGGGACAGC------------

---ATG

AAGAACGCGTTCGGTTGCGGCGAATTCAAC------AACGGG------GAATTATGGAAG
TCCCTTTTGGCGGAGTTTATCGGCACTTTGTTTCTCGTGCTAGTCGGCTGTGCGTCGTGT
TCACAAGGTTGGAATGAAAGC---------------------------------------
--TATTCCCCAACA
ATGGTTCAAGTCGCCCTTTCTTTCGGTGTCACTATTGCTACTATGGCGCAGGCGATTGGC
CATGTCAGTGGTTGCCATATTAATCCCGCCGTCACTACATCCTTGATGATAGTGGGCAAG
ATTTCTCTACTGAAAGCACTTTTTTATATCGCAGCTCAATGTGTGGGGGCCATTTCGGGG
GCTGCAATCTTACAGGCTCTGACACCTGCGGAGTTTCACAGT------------------
------------------------------ACATTAGGTGTTACTGAACTACACCCCAAG
---------TTGTCAGCCACTCAAGGCTTCGGAGTCGAGTTTTTTGCCACGTTTACTCTT
GTTTTGATCGTCTGTGGC---GTTTGTGACGGAAATCGCAAT------------------
---GATATCAAA---------------GGTTCTGCTCCTTTGGCAATTGGCCTCACCGTT
ACAACGCAAATTTTGGCCATTGGAATGTATACAGGAGGAAGTCTAAATCCAGCAAGGTCT
CTAGGTCCAGCCGTAATCCTGAAC---------AAGTGGGACAGCCATTGGGTGTACTGG
GTGGGCCCAATGGTGGGAGGTGCTGTGGCTTCCCTGTTGTACCAGAGAGCTTTCACAGCG
---CCGAGCAACAAGCGGGTGCCC---------GACGAGGAGGAA---CCA---------
------------------------GAC------------TACCCTTAT------CGGTAC
CGGGCG---GCG---AACGATAAAGAGAAGGAA------ATA------------ATTGCA
--GATAGGACTACA
TCAATC--

>RJJB01000085_Lepidurus_apus_EglpL2
ATGGGA--

------------------------------------GGCTGGGATAGT------------

---ATG
AAGAACGTGTTTGGCTGCGGCGAGTTCAAC------AACGGT------GAAGTGTGGAAA
TCCCTATTGGCGGAATTCATTGGCACTTTGTTTCTCGTACTCGTTGGTTGTGCGTCGTGT
TCACAAGGCTGGAATGAATCT---------------------------------------
--TATTCGCCAACA
ATGGTTCAAGTGGCCCTTTCCTTCGGTGTCACCATTGCCACAATGGCACAGTCGATTGGT
CACGTCAGCGGATGTCATATCAATCCCGCAGTCACAACATCGATGATGATGGTTGGCAAG
ATTTCTGTGCTGAAGGCTCTTTTCTACATCGCAGCCCAATGCGTTGGAGCTATTTCTGGA
GCTGCTATTTTGCAGGCCCTGACACCTGTGGAGTTCCACAGT------------------
------------------------------ACATTAGGCGTTACAGAGTTACATCCCAAG
---------TTATCAGCCACCCAGGGTTTTGGTGTCGAGTTTTTTGCCACTTTCACGCTG
GTCCTCATCGTCAGTGGC---GTTTGTGATGAAAATCGCAAT------------------
---GATATCAAA---------------GGATCCGCTCCCTTGGCCATCGGTCTCACCGTT
ACCACGCAAATTCTAGCTATTGGAATGTACACAGGAGGAAGCCTGAATCCCGCACGGTCC
TTGGGTCCCGCCGTAATACTCAAC---------AAATGGGACAACCATTGGGTGTACTGG
GTGGGTCCCATCGTGGGCGGAGCTGTAGCTGCCTTGTTATACCAACGGGCTTTCACCGCA
---CCGAGCCATAAGCGAGAGCCC---------GACGAGGAAGAA---CCA---------
------------------------GAT------------TATCCTTAT------CGTTAT
CGGGCT---GCA---AACGAAAAAGAGAAGGAA------GTA------------ATTGCG
--GACAGGACCACG
TCAATC--

>JAKZAN010000001_Lepidurus_packardi_EglpL2
ATGGGT--

------------------------------------GGCTGGGATAGT------------

---ATG
AAGAACGTGTTTGGCTGCGGCGAGTTCAAT------AATGGT------GAAGTGTGGAAA
TCCCTATTGGCGGAATTCATTGGCACGTTGTTTCTCGTACTCATTGGTTGTGCGTCATGT
TCACAAGGTTGGAATGAATCT---------------------------------------
--TATTCGCCAACA
ATGGTTCAAGTGGCCCTTTCCTTCGGTGTCACCATTGCCACAATGGCACAGTCGATTGGC
CACGTCAGCGGATGTCATATCAATCCCGCAGTTACAACATCGATGATGATGGTTGGCAAG
ATCTCTGTGTTGAAGGCTCTTTTCTACATCGCAGCCCAATGTGTTGGAGCTATTGCCGGA
GCTGCTATTTTGCAGGCCCTGACACCTGTGGAGTTCCACAGT------------------
------------------------------ACATTAGGCGTTACAGAATTACATCCCAAG
---------TTATCAGCCACCCAAGGTTTTGGTGTCGAGTTTTTTGCCACTTTCACGCTG
GTCCTCATCGTCAATGGC---GTTTGCGATGAAAATCGCAAT------------------
---GATATCAAA---------------GGTTCCGCTCCCTTGGCCATCGGTCTCACCGTT
ACCACGCAAATTCTGGCTATTGGAATGTACACAGGAGGAAGCCTGAATCCTGCACGGTCC
TTGGGTCCAGCCGTAATACTCAAC---------AAATGGGATAATCATTGGGTGTACTGG

GTGGGTCCCATCGTGGGCGGTGCTGTAGCTGCCCTGTTATACCAACGGGCTTTCACCGCG
---CCGAGCCATAAGCGAGAGCCT---------GACGAGGAAGAA---CCA---------
------------------------GAT------------TATCCTTAT------CGTTAT
CGGGCT---GCA---AACGAAAAAGAGAAGGAA------GTA------------ATAGCG
--GACAGGACCACG
TCAATC--

>RJJA01000114_Lepidurus_arcticus_EglpL2
ATGGGA--

------------------------------------GGCTGGGATAGT------------

---ATG
AAGAACGTGTTTGGCTGCGGCGAGTTCAAC------AACGGT------GAAGTGTGGAAA
TCCCTATTGGCGGAATTCATTGGCACTTTGTTCCTCGTACTCGTTGGTTGTGCGTCGTGT
TCACAAGGTTGGAATGAATCT---------------------------------------
--TATTCGCCAACA
ATGGTTCAAGTGGCCCTTTCCTTCGGTGTCACCATTGCCACAATGGCGCAGTCGATTGGC
CACGTCAGCGGATGTCATATCAATCCCGCGGTCACAACGTCGATGATGATGGTTGGCAAG
ATCTCTGTGCTGAAGGCTCTTTTCTACATCGCAGCTCAATGTGTTGGAGCTATTGCCGGA
GCTGCTATTTTGCAGGCCCTGACACCTGTGGAGTTCCACAGT------------------
------------------------------ACATTAGGCGTTACAGAATTACATCCCAAG
---------TTATCAGCCACCCAAGGTTTTGGTGTCGAGTTTTTTGCCACTTTCACGCTG
GTCCTCATCGTCAGTGGC---GTTTGTGATGAAAATCGCAAT------------------
---GATATCAAA---------------GGCTCCGCTCCCTTGGCCATCGGTCTCACCGTT
ACCACGCAAATTCTGGCTATTGGAATGTACACAGGAGGAAGCCTGAATCCTGCACGGTCC
TTGGGTCCCGCCGTAATACTCAAC---------AAATGGACAACCATTGGGTGTACTGG
GTGGGTCCCATCGTGGGCGGTGCTGTAGCTGCCCTGTTATATCAGCGGGCTTTCACGGCG
---CCGAGCCATAAGAGAGAGCCT---------AACGAGGAAGAA---CCA---------
------------------------GAT------------TATCCTTAT------CGTTAT
CGGGCC---GCA---AACGAAAAGGAGAAGGAA------GTA------------ATTGCG
--GACAGGACCACG
TCAATC--

>JAGJRP010000017_Lepidurus_couesii_EglpL2
ATGGGA--

------------------------------------GGCTGGGATAGT------------

---ATG
AAGAACGTGTTTGGCTGCGGCGAGTTCAAC------AACGGT------GAAGTGTGGAAA
TCCCTATTGGCGGAATTCATTGGCACTTTGTTTCTCGTACTCGTTGGCTGTGCGTCGTGT
TCACAAGGTTGGAATGAATCT---------------------------------------
--TATTCGCCAACA
ATGGTTCAAGTGGCCCTTTCTTTCGGTGTCACCATTGCCACAATGGCGCAGTCGATTGGC
CACGTCAGCGGATGTCATATCAATCCCGCAGTCACAACGTCGATGATGATGGTTGGCAAG
ATCTCTGTGCTGAAGGCTCTTTTCTACATCGCAGCTCAATGTGTTGGAGCTATTGCCGGA
GCTGCTATTTTGCAGGCCCTGACACCTGTGGAGTTCCACAGT------------------
------------------------------ACATTAGGCGTTACAGAATTACATCCCAAG
---------TTATCAGCCACCCAAGGTTTTGGTGTCGAGTTTTTTGCCACTTTCACGCTG
GTCCTCATCGTCAGTGGC---GTTTGCGATGAAAATCGCAAT------------------
---GATATCAAA---------------GGCTCCGCTCCCTTGGCCATCGGTCTCACCGTT
ACCACGCAAATTCTGGCTATTGGAATGTACACAGGAGGAAGCCTAAATCCTGCACGGTCC
TTGGGTCCGGCCGTAATACTCAAC---------AAATGGGACAACCATTGGGTGTACTGG
GTGGGTCCCATCGTGGGCGGTGCTGTAGCTGCCCTGTTATACCAACGGGCTTTCACGGCG
---CCGAGCCATAAGCGAGAACCT---------GACGAGGAAGAA---CCA---------
------------------------GAT------------TATCCTTAT------CGTTAT
CGGGCC---GCA---AACGAAAAAGAGAAGGAA------GTA------------ATTGCG
--GACAGGACCACG
TCAATC--

>JAIQCU010000075_Leptestheria_dahalacensis_EglpL2
ATGGCC--

------------------------------------TTTTGGAGCAAG------------

---ATG
CGAGAGATTTTTGGCTGTGCGGAATTCTCG---GCCAATCGC------GATGTCTGGCGC
GCCATTTTGGCGGAATTCGTCGGAACCTTGTTCCTGGTGCTGATCGGATGCGCCTCGTGC
GTCTCCGGATGGAATGAGGAT---------------------------------------
--CACTCGCCGCAT
GTGGTTCAAGTGGCACTGGCTTTCGGTGTCACCATTGCTACTATTGCACAGGCTATCGGG
CACGTCTCTGGTTGCCATCTGAACCCGGCCGTGTCCGTCGCCATGTTGATCACCGGTAAA
ATCAGTATCCTCCGGACCATCACCTACAGTATCGCCCAGTGCTTGGGAGCCATTTCTGGC
GCTGCGATCCTTCAGGCCCTGACTCCACCAGAATTCCACGCA------------------
------------------------------ACTCTGGGTGTGACCTCCCTCCATCCGAAA
---------GTGACAGTAGCGCAGGGCTTCGGCATTGAATTCTTCGCCACGTTCATGTTG
CTGCTCATCATCTTCGGC---GTGTGCGACTCCAAACGCGAT------------------

```
---GATATCAGA---------------GGATCGGCGCCCTTGGCCATCGGTTTGACCGTA
TCGGCAGCTGCACTGTCCATCGGTAGCTACACAGGCGCTAGTTTGAACCCAGCCCGCTCT
CTCGGCCCTGCAGTCATCGGCAAC---------TCCTGGGAACACCATTGGGTCTACTGG
GTGGGTCCGATCGTGGGAGGCTCAGTGGCCGGCATGGTGTACGAGAAGGCGTTCCGGGCA
---CGCAAACCG---------------------GAGGAAGAGGAT---AAC---------
------------------------CAC------------AGCAACTAC------CGTTAT
GAATTA---ACC---GATGTCAAGGATGTGAAG------GAA---GTC------ATCGCT
------------------------------------------------GACCGGACCACT
TCAATC------------------------------------------------------
------------------------------------------------------------
------------------------------------------------------------
------------------------------------------------------------
------------------------------------------------------------
------------------------------------------------------------
------------------------------------------------------------
------------------------------------------------------------
------------------------------------------------------------
------------------------------------------------------------
------------------------------------------------------------
------------------------------------------------------------
------------------------------------------------------------
------------------------------------------------------------
------------------------------------------------------------
------------------------------------------------------------
------------------------------------------------------------
------------------------------------------------------------
------------------------------------------------------------
------------------------------------------------------------
------------------------------------------------------------
------------------------------------------------------------
------------------------------------------------------------
------------------------------------------------------------
------------------------------------------------------------
------------------------------------------------------------
------------------------------------------------------------
------------------------------------------------------------
------------------------------------------------------------
------------------------------------------------------------
------------------------------------------------------------
------------------------------------------------------------
------------------------------------------------------------
------------------------------------------------------------
------------------------------------------------------------
------------------------------------------------------------
------------------------------------------------------------
------------------------------------------------------------
------------------------------------------------------
```

>NKDA01000003_Eulimnadia_texana_EglpL2
ATGGCG--

---------------------------------AAGTGCGGCACCAGA------------

---ATG
CGCAACATCATTGGCTGCGGCGAATTCTCATCGGCCAATCGG------GACATTTGGCGC
GCCATTTTGGCTGAATTTATCGGCACCTTCTTCTTGGTGCTGATCGGTTGCGCCTCTTGC
GTCAGTGGCTGGAACGAAGAT---------------------------------------
--CACTCGCCCCAC
ATCGTACAAGTGGCCCTGGCCTTCGGTGTCACTATTGCCACTATGGCACAGGCAATTGGT
CACGTGTCTGGCTGTCACATCAACCCTGCGGTTTCCATTGCGCTGCTGATCACGGGAAAG
ATCTCCATTCTTCGCACCATCTTCTACATCATCGCCCAATGCGTTGGATCCATCGCCGGA
ACCGCCGTCCTTCAGGCTCTGACTCCGGCCGACTTCCACTCC------------------
------------------------------AACTTAGGTATCACTTCACTTCACCCCAAA
---------GTGACTCCCAGCCAGGGTTTCGGCGTCGAGTTCTTCGCTACTTTCGTCCTC
GTCCTCGTCGTCTTCGCC---GTTTGCGACGACAGGCGCGAC------------------
---GACATCAAG---------------GGATCAGCTCCTCTGGCCATCGGTTTGACAGTC
TCAGCTGCTGTCCTGTCTATCGCAAGCTACACTGGAGCTAGTTTGAACCCGGCCCGTTCC
TTCGGACCTGCTGTCATTAGCAAC---------TCTTGGGAAAACCACTGGGTATACTGG
ATCGGACCTATTATTGGTGGATCAGTAGCGGCCATGATGTACCAGAAGGCCTTCCGCGCT
---CGCAAGTCCCCG------------------GAAGCGGAGGAT---AAC---------
------------------------CGC------------AGCGATTAC------CGCTAT
GAGCTA---GCT---GACGGCAAAAACAGCAAA---------------------------
--CAAGCCACCAGT
TCCGCT--

>NKDA01000001_Eulimnadia_texana_EglpL1
ATGATT--

--ACTAGA------------

---TTT
GGGAATATTATCGGATTGGGAGAGCTGTCGAGC---AGTACT------AATCTGTGGCGT
GCTGTTATCGCCGAATTCATCAGTACGTTGCTGCTGGTCTTCTTCGGTTGCGCTTCCTGC
GTATCGGAATGGAGAGAGAAC---------------------------------------
--TACTCGCCCAAC
ATTATCCAGATTGCGCTTACCTTTGGCATCGTTATTGCTACCATGGTTCAGGTAATTGGT
CATATCTCGGGAGGACACATCAACCCAGCAATAAGTGTAGCGATGTTACTGGCCGGTAAA
ATGAGCGTTCTGCGGACGTTACTCTACATAGTCGCCCAAACGGTGGGAGCTATTGCGGGA
GCTGGAATTCTGCAGGGACTGATTCCATCAGACATGCAGTCC------------------
------------------------------AACATCGGACTTACAAACATTCACCACAGA
---------CTGACTCCAGCACAGGGTTTCGGAGCAGAATTTTTCGCGACTTTTTTGCTG
GCGATTGTTGTTTTCGGG---GTGACGGACGACAGCCGCAGG------------------
---GACATCCAA---------------GGATCTCGACCGCTCGCTATCGGCCTTGCTGTA
ACGACTGCAATTCTGGCTATTGGTGACTTTACAGGGGGCGGTTTGAATCCAGCTCGTTCT
TTCGGCCCGGCTGTGGTCAAGAAC---------TTCTGGAACAATCACTGGGTATATTGG
GTGGGACCAATGGCAGGGGCAGTGGCTGCAGCGTTTATCTATCAGGGTATTTTTCGCTCG
---GGAACATCGGTGAGCGTGGTG---------GAACCAGCAGGC---GTA---------
------------------------ACTCTT---------CAGCAAGCA------GTGTTA
GCCAAC---ATG---GACGCGGAT---AAAGAA------CCAACAACA------GTGGAT
--CCTGCTACATCC
ATGATA--

>NKDA01000001_Eulimnadia_texana_EglpL3
ATGGCC--

---------------------------------AGGTGC---AATAGA------------

---ATG
CGCAACATCATTGGCTGTCACGAGTTCACC---GAGAACCGG------GATGTCTGGAGA
GCGATCCTGGCGGAATTTGTAGGAACTATGTTCCTGGTACTCATCGGATGCGCTTCCTGC
GTTAACGGCTGGAACGATTCC---------------------------------------
--TACAATCCGCAT
ATTGTCCAAGTCGCACTGTCCTTCGGTATCACTATTGCCACTATGGCACAGGTAATTGGT
CACGTGAGCGGTTGTCACATCAACCCTGCAGTGACGGTGGCCATGTTGCTGAGCGGCAAG
ATGAGCATCCTTCGCGCCATTTTCTATGTCCTTGCCCAATGCATAGGATCCATTTCCGGT
GCTGCAATCCTGCAGGCACTGACACCTCCCGAATTGCACTCC------------------

```
------------------------------AGTCTGGGCGTCACAGTTTTACACCCAAAA
----------CTGAGTCCAACCCAGGGTTTCGGCGTCGAATTTTCGCCACGTTCACGCTG
ATCATCATCGTCTTCGGA---GTCTGCGACGAGAACCGCACC------------------
---GACGTCAAG---------------GGATCCGCGCCCTTGGCCATCGGACTCACCGTC
TCCACGGCCATCTTGGCTATTGGCCAGTACACGGGAGGTAGTCTGAATCCGGCTCGTTCG
TTGGGACCCGCAGTTATCTGCAAT---------ACGTGGACGAATCATTGGGTATACTGG
GCTGGACCTATTGTTGGTGGAATCGTGGCTTCGATGTTGTATGACAGAGCATTCAGTGCA
---CGAAAAGCTAATCAG---------------GACGAATCCGAT---AAA---------
------------------------TAT------------ATCGATTAC------CGATAC
CAGCCG---GCC---GACGGCAAAGGCAAGGAA---CTGAAGGAAATC------ATCGCC
------------------------------------------------GACCGAACTACG
ACCATT------------------------------------------------------
------------------------------------------------------------
------------------------------------------------------------
------------------------------------------------------------
------------------------------------------------------------
------------------------------------------------------------
------------------------------------------------------------
------------------------------------------------------------
------------------------------------------------------------
------------------------------------------------------------
------------------------------------------------------------
------------------------------------------------------------
------------------------------------------------------------
------------------------------------------------------------
------------------------------------------------------------
------------------------------------------------------------
------------------------------------------------------------
------------------------------------------------------------
------------------------------------------------------------
------------------------------------------------------------
------------------------------------------------------------
------------------------------------------------------------
------------------------------------------------------------
------------------------------------------------------------
------------------------------------------------------------
------------------------------------------------------------
------------------------------------------------------------
------------------------------------------------------------
------------------------------------------------------------
------------------------------------------------------------
------------------------------------------------------------
------------------------------------------------------------
------------------------------------------------------------
------------------------------------------------------------
------------------------------------------------------------
------------------------------------------------------------
------------------------------------------------------------
```


>JAIQCU010000047_Leptestheria_dahalacensis_EglpL3
ATGGCC--

---------------------------------AGGTGT---CAGAGA------------

---ATG
CGCAATATCGTCGGCTGCCACGAATTCACC---GAGAATCGC------GATGTGTGGCGG
GCGATTCTGGCCGAATTCGTCGGTACCATGTTTCTGGTGCTGATCGGATGCGCGTCGTGC
GTCAACGGATGGAACGACTCG---------------------------------------
--TACAGTCCGCAC
ATTGTCCAGGTGGCCATCTCCTTTGGAGTCACCATTGCTACCATGGCGCAGGCAATCGGA
CACGTGAGCGGTTGCCACATCAATCCCGCAGTTACAGTAGCCATGCTGCTCAGCGGGAAG
ATCAGCATTCTGCGGGGAATCTTCTACGTTGTTGCTCAATGTGTAGGCTCCATTTCCGGT
GCTGCAATCCTACAGGCCTTGACACCTCCCGAGTTCCACTCG------------------
------------------------------ACACTGGGCGTGACGTCTTTGCATCCCAAG
---------CTGAGCCCGACTCAAGGTTTCGGAGTCGAATTTTTCGCCACCTTCACCCTG
GTGCTCATCGTCTTCGGC---GTCTCTGATGAAAACCGCAAT------------------
---GACGTCAAA---------------GGATCAGCGCCTCTGGCCATCGGTCTTACCGTC
ACAACATCCATTCTGGCCATTGGAATGTACACAGGCGGAAGTTTGAATCCGGCGCGATCC
TTGGGGCCTGCCATCATCAGTAAC---------TCCTGGGAGCATCACTGGGTGTACTGG
GCCGGTCCTATCATCGGAGGTATCGTCGCTTCCATGATGTACGACAAGGCGTTCCGGGCG
---CGGAAGGCCAACAAC---------------GACGACGACGAC---GTC---------
------------------------TAC------------ATCGATTAC------CGCTAC
AGACCC---GCT---GACGTCAAAGAGAAGGAA---CTGAAGGAAATA------ATCGCC
--GATAGAACGACC
ACAATT--

>JAKZAO010000012_Branchinecta_lindahli_EglpL1_v1
ATGGGT--

------TGTACTTGTTGTAGTGCTTGGTGGGGAAGACAGTGTGACAGA------------

---ATG
AAGGATACACTTGGTTGTTCCGAGATTGCTGGGGAAAATAGG------CACATTTGGAGG
TGTGCCCTAGCGGAGGCGATTGGAACTTTGTTTCTTGTTCTTGTCGGCTGCGGGTCGTGT
TTGTCGATTCATGGTGAA--
--GCCAAC
TCCGTTGCTGTTGCGGTGTCCTTTGGACTTGCACTGGCTACAATGGCTCAGTCAATTGGC
CACGTCAGCGGGTGTCACATCAACCCAGCCGTAACGGCCGGATTTTTGATCACCGGAAAA
ATCACGATTGTCCGGGCAATTCTTTATGTTGCCGCGCAGTGCATCGGAGCTACTGCCGGA
TCAGCAGCACTTGAGTGCCTCACGCCTCCAAAGTTCCACGGA------------------
------------------------------TCACTGGGAGTCACCGCAATCAACGCTGAT
---------GTCTCTCCATCGCAAGCGTTTGGAGTGGAATTTTTTGCGACCTTCGTCCTC
ATCTTCGTTATTTTCGCC---GTCTGCGATGAAAACAGAACT------------------
---GACGTCAAG---------------GGATCGGCCCCGATGGCCATTGGCCTTACCGTC
ACCATGATGGCTCTTGCTTTTGGCTCCTACACTGGCTGCAGTATGAACCCCGCCAGAAGT
CTTGGACCTGCCATCATCACAGGA---------AGATTTGAACATCACTGGGTTTACTGG
GTTGGACCCATCCTAGGTGGAATCGTTGCAGCCCTTATCTACCAATCCACTTTCAGAGTC
---CGGCCAGAACCC---CTGCCA---------CTCGAAGACGAA---GAA---------
------------------------ACC------------GGTGGCAAG------ATTTAC
CACTTTAGGGTA---TTAAACAAGAAAAAGTTT------CCG------------TTCCGT
--AAAAAGTCGTTT
TTTTCTAAAACTATCGATTTGATGAGCCAGTTGGCAAATACTCCCCCCCCCCCCCCCCAA
AAAAAAAAAAAAAAACAGTTTGGC------------------------------------

>JAKZAP010000012_Branchinecta_lynchi_EglpL1_v1
ATGGGT--

------TGTACTTGCTGTAGTGGTTGGTGGGGAAGACAGTGTGCAAGA------------

---ATG
AAGGATACACTTGGTTGTTCCGAGATTGCTGGGGAAAATAGG------CACATTTGGAGA
TGTGCTCTTGCGGAGGCGATTGGAACTTTGTTCCTTGTGCTTGTGGGAAGCGGGTCGTGT
TCGTCCATTCATGGTGAA--
--CCCAAC
TCCCTTGCTGTTGCGCTGTCCTTCGGACTTGCGCTGGCTGCAATGGTTCAGTCAATTGGA

CACGTCAGCGGGTGTCACATCAACCCAGCCGTAACGGGCGCATTTTTGATCACCGGAAAA
ATCACGATTGTCCGGGCAGTTCTTTACGTCGCCGCACAGTGCATCGGAGCTACTGCCGGA
TCAGCAGCCCTTCAGTGTCTGACGCCCCCGAAGTTTCACGGG------------------
------------------------------TCACTGGGAGTCACGGCTATCCACGCTGAT
---------GTCTCTCCGTCCCAAGCGTTTGGGGTGGAATTTTTCGCCACCTTCGTCCTT
ATCCTCGTTATTTTTGCT---GTCTGCGATGAAAACAGAACT------------------
---GACGTCAAA---------------GGATCGGCACCGATGGCCATTGGCCTCACCGTC
ACCATGATGGCTCTTGCTTTTGGCTCCTACACTGGCTGCAGTATGAACCCCGCCAGAAGT
CTTGGACCTGCCATCATCACAGGA---------AGATTCGAACATCACTGGGTTTACTGG
GTTGGACCCATCCTAGGGGGAGTCGTTGCAGCCCTTATCTACCAGTCCACTTTCAGAGTC
---CGGCCAGAACCC---ATGCCA---------CATGAAGACGAA---GAA---------
------------------------ACC------------GGTGGCAAT------ATATAC
CACTTTAAGGTA---TCTAACGAGAAAAAAGCT------TCC------------GTGTCG

>JAKZAO010000012_Branchinecta_lindahli_EglpL1_v2
ATGGGT--

------GTTCCTTGC------------TTTGGCAACCTTTTTGAGAGA------------

---ATG
AAGGACGCCATGAGTTGTTCGGAAATGTCTGGA---AAT---------CAGATTTGGAAG
ATGTTGCTGTCCGAGGCCCTGGGTACTATGCTGCTTGTCTTTATAGGCTGTGCTTCGTGT
TCCCCAATCCATGGAGAG--
--CCAAAT
GCAGTGGGGATTTCCATTACGTTTGGATTGGCAGTGGCTGCACTTGCTCAGTCAATTGGC
CACGTCAGCGGGTGTCACATCAACCCAGCCGTAACGGCCGGATTTTTGATCACCGGAAAA
ATCACGATTGTCCGGGCAATTCTTTATGTTGCCGCGCAGTGCATCGGAGCTACTGCCGGA
TCAGCAGCACTTGAGTGCCTCACGCCTCCAAAGTTCCACGGA------------------
------------------------------TCACTGGGAGTCACCGCAATCAACGCTGAT
---------GTCTCTCCATCGCAAGCGTTTGGAGTGGAATTTTTTGCGACCTTCGTCCTC
ATCTTCGTTATTTTCGCC---GTCTGCGATGAAAACAGAACT------------------
---GACGTCAAG---------------GGATCGGCCCCGATGGCCATTGGCCTTACCGTC
ACCATGATGGCTCTTGCTTTTGGCTCCTACACTGGCTGCAGTATGAACCCCGCCAGAAGT
CTTGGACCTGCCATCATCACAGGA---------AGATTTGAACATCACTGGGTTTACTGG
GTTGGACCCATCCTAGGTGGAATCGTTGCAGCCCTTATCTACCAATCCACTTTCAGAGTC
---CGGCCAGAACCC---CTGCCA---------CTCGAAGACGAA---GAA---------
------------------------ACC------------GGTGGCAAG------ATTTAC
CACTTTAGGGTA---TTAAACAAGAAAAAGTTT------CCG------------TTCCGT
--AAAAAGTCGTTT
TTTTCTAAAACTATCGATTTGATGAGCCAGTTGGCAAATACTCCCCCCCCCCCCCCCCAA
AAAAAAAAAAAAAAACAGTTTGGC------------------------------------

>JAKZAP010000012_Branchinecta_lynchi_EglpL1_v2
ATGGGT--

------GTTCTCTGC------------TTTGGCAACCTTGTCGAGAGA------------

---ATG
AAGGGCGCCATGAGTTGTTCGGAAATGTCTGGA---AAT---------CAGATTTGGAAG
ATGTTGCTATCTGAGGCTCTGGGTACAATGCTGCTTGTATTTGTAGGCTGTGCTTCTTGC
TCACCCATCCATGGTGAA--
--CCAAAT
GCGGTGGGAATTTCTTTGACGTTTGGATTGGCAGTGGCCGCACTTGCTCAGTCAATTGGA
CACGTCAGCGGGTGTCACATCAACCCAGCCGTAACGGGCGCATTTTTGATCACCGGAAAA
ATCACGATTGTCCGGGCAGTTCTTTACGTCGCCGCACAGTGCATCGGAGCTACTGCCGGA
TCAGCAGCCCTTCAGTGTCTGACGCCCCCGAAGTTTCACGGG------------------
------------------------------TCACTGGGAGTCACGGCTATCCACGCTGAT
---------GTCTCTCCGTCCCAAGCGTTTGGGGTGGAATTTTTCGCCACCTTCGTCCTT
ATCCTCGTTATTTTTGCT---GTCTGCGATGAAAACAGAACT------------------
---GACGTCAAA---------------GGATCGGCACCGATGGCCATTGGCCTCACCGTC
ACCATGATGGCTCTTGCTTTTGGCTCCTACACTGGCTGCAGTATGAACCCCGCCAGAAGT
CTTGGACCTGCCATCATCACAGGA---------AGATTCGAACATCACTGGGTTTACTGG
GTTGGACCCATCCTAGGGGGAGTCGTTGCAGCCCTTATCTACCAGTCCACTTTCAGAGTC
---CGGCCAGAACCC---ATGCCA---------CATGAAGACGAA---GAA---------
------------------------ACC------------GGTGGCAAT------ATATAC
CACTTTAAGGTA---TCTAACGAGAAAAAAGCT------TCC------------GTGTCG

>GCBP01028494_Eubranchipus_grubii_EglpL1
ATGGGT--

------TGCTGTAGTCAA------TCTTGTGCTCGGTTGATGAATAAG------------

---ATG
AAGAATATATTGGGATGTTCTGAAATTGCTGGTGAAAATAGG------CAAATATGGAAA
GCACTCCTTGCGGAGGCACTGGGAACTATGTTCCTTGTGCTAGTGGGTTGTGCATCGTGT

GTGGAATGGAACGGAGTA--
---GCGCCAAGC
CCGGTGGCAATTTCGTTGGCTTTTGGTGTAACGTTGGCGGCAATGGCACAGGCACTTGGT
CACGTCAGCGGTTGCCATATTAATCCAGCAGTGACAGCGGGATTTCTAATTACCGGCAAG
ATCACGATTGTTCGCGCCATTCTTTATATTGCGGCACAATGTATCGGCGCCACGGCAGGA
GCGGCATGCCTTGAGTGTCTGACCCCAGAAGAGTTCCACGGG------------------
------------------------------GCCCTTGGCGTTACCTCTATTGCGGCGAAA
---------GTGTCGCCATCTCAAGGATTCGGCGTCGAATTTTTCGCTACGTTCACACTC
GTTCTCGTAATTTTTGCC---GTATGCGATGAGAACCGAACT------------------
---GATGTTAAG---------------GGATCCGCCCCTCTAGCCATCGGACTTGTGGTC
ACCATGCAGGCTCTTGCTTTCGGCTCATACACGGGTTGCAGTATGAATCCAGCAAGGAGT
CTTGGACCGGCAATTATAAAAGGA---------AAATTTGAACATCACTGGGTGTATTGG
GTTGGACCGATCCTTGGAGGGATCGTCGCTGCACTACTTTACGAAAACGCATTTAAAGCA
---CAATCCGAAGTT---GAATGT---------GAAGATGATGAA---GAG---------
------------------------CAAATT---------AAGCCACGA------GTTTAT
CATTTCAAGGAA---AACAATCGAAAAGGAAGT------TTT------------ATTGCC
--GAGAAGGAAAAA
TTGGAATTAATTGTAGATCGTCAATCGTCACTC---------------------------

>GCBP01028516_Eubranchipus_grubii_EglpL2
ATGGGC--

------TGCTGTGAC------------------CATTTGTTCAGAAAA------------

---GTG
AGGAATATGGCCAACTGCGAAGAAATGTCCGGG---AAC---------AAGATCTGGCTA
TCTCTTCTGTCAGAGGCTCTGGGAACCATGCTCCTGGTTTTCATTGGCTGTGCGTCATGC
TTGCCTATCCACGGAGAG--
--CCAAAT
TCAGTGGGAATTTCATTGGCTTTCGGCCTATTAGTCGCATCTTTAGCTCAGGCACTTGGT
CACGTCAGCGGTTGCCATATTAATCCAGCAGTGACAGCGGGATTTCTAATTACCGGCAAG
ATCACGATTGTTCGCGCCATTCTTTATATTGCGGCACAATGTATCGGCGCCACGGCAGGA
GCGGCATGCCTTGAGTGTCTGACCCCAGAAGAGTTCCACGGG------------------
------------------------------GCCCTTGGCGTTACCTCTATTGCGGCGAAA
---------GTGTCGCCATCTCAAGGATTCGGCGTCGAATTTTTCGCTACGTTCACACTC
GTTCTCGTAATTTTTGCC---GTATGCGATGAGAACCGAACT------------------
---GATGTTAAG---------------GGATCCGCCCCTCTAGCCATCGGACTTGTGGTC
ACCATGCAGGCTCTTGCTTTCGGCTCATACACGGGTTGCAGTATGAATCCAGCAAGGAGT
CTTGGACCGGCAATTATAAAAGGA---------AAATTTGAACATCACTGGGTGTATTGG
GTTGGACCGATCCTTGGAGGGATCGTCGCTGCACTACTTTACGAAAACGCATTTAAAGCA
---CAATCCGAAGTT---GAATGT---------GAAGATGATGAA---GAG---------
------------------------CAAATT---------AAGCCACGA------GTTTAT
CATTTCAAGGAA---AACAATCGAAAAGGAAGT------TTT------------ATTGCC
--GAGAAGGAAAAA
TTGGAATTAATTGTAGATCGTCAATCGTCACTC---------------------------

>PQ469250_Artemia_franciscana_EglpL
ATGGGC--

------TGCTTCAGT------------------GGTTTGTGGGAGCGG------------

---GTG
AAAAAAATGGCCAAGTTCTCAAAAATGATGGGA---AGT---------AACATTTGGCAA
GCTCTTTTCGCTGAAGCAATGGGTACCATGCTTCTAGTTTATGTAGGATGTGGCTCTGCA
TTGCCAATACATGGACCA--
--CCTAAC
CCAGTGGCAATCTCTTTAGTTTTTGGGTTGACTGTGGCAACACTAGTTCAGGGTATTGGA
CATGTGAGTGGTTGTCACATAAATCCTGCTGTAACTGCAGCCATGGCAGTAACTGGAAAA
ATGCCATTTCTCCGTGCAGCTCTCTATGTGGTAGCACAGTGCATCGGTGCAATTGCTGGA
TCAGCTTGCCTTGAGTGGCTAACACCAGAGCAGTTCCGTGGA------------------
------------------------------ACTTTGGGCGCAACTACTGTCAGTGAGCAT
---------GTAACGCCATCTCAAGCACTTGGCATTGAGTTTTTTTCAACTTTTATTTTA
TTGTTTTTCATTTTCGCA---GTCACGGATGAACGAAAAACG------------------
---GATATCCTC---------------GGATCCCCTGCATTGGCTATTGGTTTTGTAGTC
ACAGCTCAAGCCCTGGCTTTTGGTTCTTATGCTGGTTGTAGTATGAATCCTGCGAGAAGT
CTTGGTCCTGCAGTTCTGATTGGA---------GACTTCAGGCTGCACTGGGTATATTGG
GTTGGGCCGATTTTTGGAGCTATAGTAGCAGGTTTCGTTTATGAGAAGTTCTTCAGAGCA
---GAATTATATGCT---GAACGA------------GCAAATGAC---GAG---------

------------------------ATATTT---------GAAACTAAG------AAGTAT
CATTTT------------CAGAAATCTCCAAGC------AGT------------CTCGTT
---AATGATATAGAAAAA
---GAACTAATTGTGGAAACAGATAAA---------------------------------

>CM051267_Artemia_sinica_EglpL
ATGGGC--

------TGCTTCAGT------------------GGTTTGTGGGAGCGG------------

```
---------------------------------------------------------GTG
AAAAAAATGGCCAAGTTCTCAAAAATGATGGGA---AGT---------AACATTTGGCAA
GCTCTTTTTGCTGAAGCAATGGGTACCATGCTTTTAGTTTATGTAGGATGTGGCTCTGCA
TTGCCAATACATGGACCA------------------------------------------
------------------------------------------------------CCGAAC
CCAGTGGCAATCTCTTTAGTTTTTGGGCTGACTGTGGCAACACTTGTTCAGGGTATTGGA
CATGTGAGCGGTTGTCACATAAATCCTGCTGTAACTGCAGCTATGGCAGTTACTGGAAAA
ATACCATTTCTCCGTGCAGCTCTCTATGCTGTAGCGCAATGCATCGGTGCAATTGCTGGA
TCAGCTTGCCTTGAGTGGCTAACACCAGAGCAGTTCCGTGGC------------------
------------------------------ACTTTGGGTGCAACTACCGTCAGTGAACAT
---------GTAACACCATCTCAAGCACTTGGCATTGAGTTCTTTTCAACTTTTATTTTA
TTGTTTTTCATTTTCGCA---GTCACTGATGAACGAAAAACG------------------
---GATATCCTC---------------GGATCTCCTGCATTGGCTATTGGTTTTGTAGTC
ACAGCTCAAGCCCTGGCTTTTGGTTCCTATGCTGGATGTAGTATGAATCCTGCAAGAAGT
CTTGGTCCTGCAGTTCTGATTGGA---------GACTTCAGGCTGCACTGGGTATATTGG
GTTGGACCGATTTTTGGAGCTATAGTAGCAGGTCTTGTCTATGAGAAGTTCTTCAGAGCA
---GAATTATATGCT---GAACCA------------GCAAATGAC---GAA---------
------------------------ATATTT---------GAAACTAAG------AAGTAT
CATTTT------------CAG---TCACCAAGC------AGT------------CTTGTT
---------------------------------------------AATGATATAGAAAAA
---GAATTAATTGTAGAAACAGATAAA---------------------------------
------------------------------------------------------------
------------------------------------------------------------
------------------------------------------------------------
------------------------------------------------------------
------------------------------------------------------------
------------------------------------------------------------
------------------------------------------------------------
------------------------------------------------------------
------------------------------------------------------------
------------------------------------------------------------
------------------------------------------------------------
------------------------------------------------------------
------------------------------------------------------------
------------------------------------------------------------
------------------------------------------------------------
------------------------------------------------------------
------------------------------------------------------------
------------------------------------------------------------
------------------------------------------------------------
------------------------------------------------------------
------------------------------------------------------------
------------------------------------------------------------
------------------------------------------------------------
------------------------------------------------------------
------------------------------------------------------------
------------------------------------------------------------
------------------------------------------------------------
```


>ICLE01166527_Heterocypris_incongruens_EglpL1
ATGGGT--

---------------------GACTTCGCGCAGTGGCTGGGCTTGAAA------------

AAA---------GCTCCAGGA---------ATCGATGCGGTG------ATTTCATGGCGT
TCTTTGATCGCCGAGTACTTGGGAACACTGTTACTCGTGTTCATGGGGACCATGTTGGCT
GTGGCA---------CCA--
--GGAAGCATCGAC
CCTATAGCTGGACCATTTGCTGGTGGTCTCGCCTTGGCTTCAATCATCCAGGCCATTGGA
CATGTGAGTGGGGCTCACATTAACCCTGCGATGAGTCTGGGGTTCTTCGTCGCCAGAAGG
ATGTCTTTGCTGAAAGCTCTTGCTTACATGTTGGCACAATGTGTTGGAGCTATTTCTGGA
ACTGCTATTTTGAAGGCCATCACCCCGAAGGCAAACCACGGA------------------
------------------------------ACCATGGGAGCCACTGCCCTCAATCCGGAT
---------TTGGCCCCAGCTCAAGGCTTTGCGATTGAAATCGTCATAACCTTCATCCTT
GTCTTCATGACTGTTTCA---TCAACTGACCCTCTGAGGAAA------------------
---GATGTCATT---------------GGATCACTCCCATTGGCTATTGGAGTGTATGTC
ACAGGGGCAGCCCTGGCAGCGGGCTCTTTCACCGGTGCCAGCATGAACCCAGCGAGAAGC
CTTGGACCAGCTGTTATTGGCGGA---------TTTTGGGAACATCATTGGATTTACTGG
GCTGGCCCAATGTTCGGAGGTGCCCTGGCTGGAATCATCCACTCAACCCTTTTCGAAGCT
---CGGTTCTTGCAACCAGCAGCC------CAGGAAGTCAAGGAT---TCA---------
------------------------------------CCCCAA---GAA---GATGAGAGT
GACGCC---GAA------AGTGGGCGTCGTGCG---ACATCGTCGTCGTACGAGGAGAAA
--CCCAAG---TAT

>CAJPEX010000337_Notodromas_monacha_EglpL1
ATGGGT--

---------------AAGCTCGACTGTCTCGTGTGGCTTGGCCTGAAG------------

CAA------GGCCCCCCAGGGAGTACA---ATCGACGGTGTT------GTCACGTGGCGA
ATGCTGATAGCCGAGTATCTCGGCACGCTCTTGCTCGTTTTCTTTGGCACCATGCTCGCC
GTGGCT---------CCA--
--GGAAACGTGGAC
CCGATCTCTGGACCTTTCGCTGGGGGACTAGCGCTTGCCTGCATCGTTCAGGCATTGGCG
CACGTGAGTGGTGCGATGGTTAATCCGGCAATGTCACTGGGCTTCTTCACCTGTAGGCGG
ATTTCCTTCATCAAGTTGTGCTTGTTTTCGCTGGCCCAATGCGCAGGAGCCATCTCCGGA
TCCGCGCTTCTGAAGGCAGTAACCCCGAGCACGAACCACGCG------------------
------------------------TTGCATGCAATGGGATCGACCACTCTGGCCCCCGGA
---------GTGACGATTTTCCATGGGATCTGCATTGAGATCATGATCACATTCCTGCTC
GTCTTCCTCACTGTGTCT---GCCACTGATGGTCGACGAGTG------------------
---GACGTTACC---------------GGCTCCATCCCGCTCATCATCGGCCTGGCCATC
GTTGCCTGCTCATTCGCTGCCGGACCCTTCACCGGAGCCAGCATGAATCCCGCAAGAAGC

CTCGGACCTGCTGTCATCAGCGGA---------TACTGGGAGCATCAATACGTGTATTGG
ATCGGTCCGTTGATTGGCGGCGCTTTGGGCGGTCTAATCTACGCCGTGCTGTTCGACGCC
---AAGTACTTCGAGAAAAGCGAG---------GACCTGACCCAG---------------
------------------------GAGACA------CCTAGACCCTTTGACGACGAGACT
CACTTT---GGC---TCTCAAGGC---CACTAC---GCTGAGGGAAAGGGC---AGCAAG
---TAC

>ICLE01031650_Heterocypris_incongruens_EglpL2
ATGCGC--

```
------------------------------------------------------------
---ACAAGGAAAGCAGGATTCGATTGCATGGTATGGATGGGAGTGAAG------------
------------------------------------------------------------
------------------------------------------------------------
AAA---------CCAGCTGAAAACAGCCCAATGGACGCCAAG------CTGTCCTGGAGA
GCATTGATTGCTGAATACCTTGGCACCTTGCTGTTGGTTTTCATGGGAACGATCGGCTCA
GTTCAC---------AAA------------------------------------------
------------------------------------------------GACCCAAATTTT
GCGAACTTTGGGCCATTTGCTGGTGGTTTTGCTGTTGGTACAATCGTTAAGGCCATCGCT
CACGTCAGTGGAGGACACATCAACCCAGCTGTCAGCGTCGGGTTCTTCGCGTGCCATCGG
ATGACTCTCGTCAAAACAATCCTCTACATCCTGGCTCAATGCGCTGGCGCAATATCCGGT
TCGGCCATACTGAAGGCCATTCTGCCAGATGATGTCCTGGAT------------------
------------------------TTGTCCTCATTGGGGGTTACCGATATTCTTTTC---
---------GTATCTCCTGGACAAGCCATGGTTGTGGAGACTATCATCACCTTCATCCTG
GTCTTCATGATTGTTTCA---ACTACCGATGGCGGTCGAAAT------------------
---GACGTTTCT---------------GGATCCGTGCCTTTGGCTATTGGGATCTATATT
GTCGGCGCAGCTTTCGCAGCGGGACCATTGACTGGAGCAAGCATGAACCCAGCCAGAAGC
CTCGGCCCAGCTGTCATTTCTGGC---------CACTGGGACAACCACTGGGTTTATTGG
GCGGGTCCGATCTGCGGCGGTGCTCTAGCTGCCCTCATTTACCACAACCTGTTCGACGCT
---AGATTCGTCCTGAAGTTGGAC---------GAGGAAGACGAA---------------
------------------------GAGGCT------CCTCAGCCAGTG------ACCTAT
CACCAC---AGC---CACAACCCAAGGGGATAC---ATCGAGAACAGATTC---TCCAGC
------------------------------------------------CCTCGCCGACAT
TCGCCACCA---TCTTCTGCTCAACCCGTAGTTGGCCGAGTGTACGACGAAAAGGCACGG
AAATAT------------------------------------------------------
------------------------------------------------------------
------------------------------------------------------------
------------------------------------------------------------
------------------------------------------------------------
------------------------------------------------------------
------------------------------------------------------------
------------------------------------------------------------
------------------------------------------------------------
------------------------------------------------------------
------------------------------------------------------------
------------------------------------------------------------
------------------------------------------------------------
------------------------------------------------------------
------------------------------------------------------------
------------------------------------------------------------
------------------------------------------------------------
------------------------------------------------------------
------------------------------------------------------------
------------------------------------------------------------
------------------------------------------------------------
------------------------------------------------------------
------------------------------------------------------------
------------------------------------------------------------
```


>CAJPEX010000340_Notodromas_monacha_EglpL2
ATGAAC--

---------GTGATCCACTAT---TGCTTAGTATGGTTGGGACTGAAG------------

AAGCCCGAGGATGAAGTTGACAGCACG---ATGGATGCAAGG------CTCACGTGGCGA
GCGCTGATTGCCGAGTATTTGGGCACATTGTTTCTGGTACTTCTCGGCACGATGTCGACG
ATCCAC---CCGCCGGGA--
---AGCAACCCTTTCGGA
GCCGTGACAGGACCATTTGCTGGCGGGTTTGCCGTCGGTACGATTGTTAAGGCCATTGCT
CACGTCAGCGGCGGACACATCAACCCTGCCGTCAGCATCGGATTTTTCACTTGCCGAAGG
ATGACTTTGGTGAAAACTTTGCTGTACATTTTCGCGCAGTCCCTGGGCGCAATAACTGGA
TCAGCGATTCTGAAGGCGTTAAGCGTGGAGTATTCAGACGGG------------------
------------------------------ATGCTCGGAGTGACCGTGCTGGCCACGCAG
---------ATTACGTCGGTGCAAGGACTCGCGATCGAGATCATCATCACTTTCATGCTC
GTTTTCTTGATTGTGTCC---ACCACTGATGGAGGACGACAG------------------
---GACGTTTCT---------------GGTTCAGTGCCTCTCGCTATTGGGATCTACATC
GTCGGCGCGGCTTTCGCAGCTGGCCCATTAACCGGTGGGAGTATGAATCCGGCGAGAAGT
CTCGGACCTGCTGTCATCACAGGG---------CACTGGAAAGCTCACTGGGTATACTGG
GCGGGTCCGCTCGCGGGTGGAGCGCTAGCCGGACTCCTGTACAGCAACTTGTTCGATGCC
---AAATACGTCGTCGAACCGGAG---------AACGAGGACGCG---------------
------------------------GACACC------CCGGCGCCGCTA------GCTAGC
CCACCA---GTA---AGAACACAGCAGGCTCCT---TAC------CAC------CTCCAA
--GGAATTCATTAC
CAGCCGGAGGAATCCTACAATTCAAGGCGTATCATCAACAGCTACGATGCGAAGGGAGCC
AAATTC--

>FBpp0072014_Drosophila_melanogaster_Eglp4
ATGAGC--

------------------------------------CACGAGATTCGTTGC---------

---TGT
AAG---------GTCTTCAAAATG---AAGGATCGACGCTG-------GACAAAATCTCC
GCCTTCCTCGGCGAGCTCATCGGCACCGGCATCCTGGTCTTCCTGGGCTGCATGGGTTGC
GTGAAG------ACGGAC--
---CTCTTTCCCAACAAC
CATCTGCAGATTGTGCTGAACTTCGGCTTCGCCGTCCTCATCGCCATCCAGTGCTTTGGC
TGCGTCTCCGGTGCCCATCTGAATCCCGCCGTGACCGTGGCCGCCTACATCTACGAGATG
GTCACCCTGCGCATGGCATTTGCCTACTTCGCCGCCCAGATGCTGGGCGCCTTCATCGGC
TACGGCCTGCTCATGGTCCTGCTTCCCTCGCCCACACTTACGGTG---------------
------------------------GGCGCTGGGCTCTGCGTGACCTTGCCCCACACCAGC
---------GTAACCACGGGCCAGGCCCTCGGCATCGAGTTCGTAATCACCTCCATCCTG

GTCATCGTCTGCTGCGGA---GTGTGGGATCCGCGCAACTCC------------------
---AAGTTCCAT---------------GACTCCGTGGGCATTCGATTTGGCCTGGCCATC
GCCTGTCTCGCCTGCGCTGCCGGTCCCTTCACCGGCGGCAGCATGAACCCGGCCAGGTCG
TTCGCCCCCGCCCTGTGGAACAAG---------CACTTCGAGAGCAACTGGATCTACTGG
CTGGCCCCGCTGAGCTCCTCCGCCATCACCGCCTACGCCTACAAGGTCGTCTTCCGCCGC
---GAGGTGGTTGAGGCGGAGATC------------ACCTCCAAT---GAGAAG------
------------------------CTGCGG---------CAACTGGAG---GACGTCCAG
CTGTCG--

>FBpp0155125_Dros_Drosophila_grimshawi_Eglp4

---------------------ATG---AAGGGTTCGATGTTG------GATAAAATCAGC
TGCTTCCTGGCCGAGCTGATGGGCACAGGAATTCTCGTCTTTCTGGGCTGCATTGGTTGC
GTAAAA------ACGGAC--
---ATATTCCCCAACAAT
CATCTACAGATTATTCTCAACTTTGGCTTTGCTGTTCTGATCGCCATCCAGTGTTTTGGC
TGTGTATCCGGAGCTCATATCAATCCCGCCGTCACAGTGGCTGCCTATATCTATGATTTG
GTCACGTTGCCAATGGCACTTGTATATTTTGCGGGCCAAATGCTTGGCGCTTTCATTGGT
TATGGCCTGCTGAAGGCATTGTTGCCAGACCCAACGTTAGAT------------------
------------------------------GGCTTGTGCGTTACGCTGCCACATGCAACT
---------GTGACGACTCCTCAGGCCTTTGGCATTGAGTTTGTGGCCACCTCAATTCTG
ATAATTGTCTGCTGTGGC---GTCTGGGATCCCCGCAATGCC------------------
---AAACACCAC---------------GACTCAGTGGCCATTCGCTTTGGCCTGGCCATT
GCCGGTTTGGCATGTGCTGCCGGTCCATTCACTGGCGCCAGCATGAATCCGGCCAGGTCC
TTTGCGCCTGCCCTGTGGAACACT---------AACTTTACGGCACATTGGATCTATTGG
CTGGCACCACTGAGCAGTGCTGCCATTACTGCCATTACCTATAAGGCTGTCTTCCGGCGC
---GAGGTTGTCGAGGCGCAGGTC------------ATTGCAGAA---GAGAAA------
------------------------CTGCGT---------CAGCTTGAG---GATGTGCAG
CTGTCA--

>AFP49896_Glossina_morsitans_morsitans_Eglp2a
ATGAAG--

---AAT
TTCCTGTTATTAACCAGACCATTTGAAAATGGGATTATTGTGCGC---GAACACATCTGC
TGTTTCCTGAGCGAATTTTTTGGTACCGGTATGTTATTATTTTTCGGTTGCATGGGCTGT
GTATAT------TATGAA--
---GTTAACAAATATAAC
CAATGGCTATCAGCTTTCAATTTTGGTTTAGTTGTGCTAATCGTAGTCCAATGCTTTGGC
TGTTGTTCTGGTGCTCATCTAAATCCTGTTATTACTATTGCCGCTTGGATATATAATATG
TTATCGGCTTTTGCTGCTTCGCTATATATATTGGCACAATTTACAGGCGCTTATTGTGGT
TATGCTCTGTTGCATTTGCTCTTACCCAGCTCCCTGTGTACG------------------
------------------------CCG---AATTTGTGCATGACAACGTTACGTGTAGAT
---------ATAACAATTTGGCAAGCTATAGGTATTGAATTTTGTACAACCGTAGCCTTG
GTTTGGGTAACATGCGGC---GTTTGGGATCCACGCAATGTC------------------
---CGTATGCAA---------------GATTCTGCCGCTATACGAATTGGTTTAACAATA
TCAGCTTTGGCTTTGGCAGCGGGGCCTCTTACCGGTGCTAGTATGAATCCGGCACGTTCT
TTTGCCCCGGCTGTATGGCATAAT---------TCTTATGATCACCATTGGATTTATTGG
CTATCCCCAATTATGGCCGGTATATTTGCTGCCAGCTTCTATAGAGTTGTGTTTCGACGT
---GAAGTGGACTCAGCTGATATT------------AATAATCCG---AAATTA------
------------------------GAAGCC------------ATACAA------ATTCCA

>ADD18960_Glossina_morsitans_morsitans_Eglp4

---------------------ATG---CATTCTTCCGTACGT------CATGCCGTCAAG
TGTTTTTTCGCCGAAATTCTTGGTACCGGTCTACTGGTATTTCTCGGCTGCATGGGTTGT
AGCTCG------ACGGTA--
---AACTCAATCACCTCG
CCTTTGCAGGCCAGCATCAACGTTAGCTTAGTGGTAATGATGATGGTTCAGTGCTTCGGT
TGTATTTCGGGCGCTCATATTAATCCAATGATAACTGTGGCCGCATGTGTGTACGATTTG
GTGTCGATACCTTTGGGTTTAGTTTATGTTTGTGCGCAAATCTTAGGCGCTATGTTTGGC

TATGGCTTACTAAAAGTTTTCTTACCAGCCTATATAATGGACGTGGCATACAAT------
------------------------GATCATGGCTTTTGTGTAACTGTGCCAGCCAAGGAT
---------ATAACGGTTGCTCAAGCTTTCGGTATTGAATTCATAATCACATCGGTATTT
GTGATGGTTTGTTGCGGC---ATTTGGACCCACGTAGTGCT-------------------
---ACTCTGATT---------------GATTCGGTTGCCTTACGTTTAGGTCTTACGGTT
GGTGGCTTAGTGGGCGTAGCTGGCTCATTTTCTGGTGCCAGCATGAACCCAGCTAGATCA
TTAGGACCCGCTTTATGGCATATG---------GATTTTGAAAATCATTGGATTTATTGG
GTGGCTCCTCTCACTTCTTCACTTCTCACTTCTTACCTTTATAAGTATTTTTTTAGAAGT
---ACATTACGTCCTGGTTCAGTT---------------TCGGAC---ATT---------

>FBpp0072016_Drosophila_melanogaster_Eglp2
ATGGCT--

---ACAACCGCA------

---AGC
CAATCCAACTGCTGG---CTGCTG---CAGCGCCGCCAGCTG------GACAGCATCACA
ACAGTTCTTGCCGAGATGATAGCCACCGCTATGCTGATGTTCCTCGGATGCATGGGCAGT
GTGGAG------AACTCC--
---GTGTTCACCAACTCG
GACTTCCAGAGCGCCCTGAACTTCGGATTCGTGGTGCTAATCTGCATCCAGTGCTTCGGT
TGCGTGTGTGGCGCCCACCTGAATCCCGCCGTCACCCTGGCCACCTACGTCTACAACATG
ATCTCCCTGCCGATGGCTTTGGCCTATTTTGTGGCCCAGATGGTGGGCGCCTTCATTGGC
TACGGCCTGCTGAAGGCGGTGCTCCCGGAGAGCGCCATCTACAGCGCTGAGAAC------
------------------------CCCAATGGCGTCTGCCTCACCTCGCTCAACAGCACC
---------TTGACCCCATGGCAGGGACTGGCCGTGGAGTTCCTGATCACCTGCGTCCTC
ATCTCCGTGTGCTGCGGC---GTCTGGGATCCCCGCAATGCC------------------
---ACCAAGCAG---------------GACTCGCTGCCAGTGCGATTTGGTCTGGCCATC
GCATGCCTGTCGCTCACAGCGGGACAACTGACTGGGGCTAGTATGAATCCAGTCCGATCC
TTTGCTCCGGCCATTTGGAACGGA---------TTCTGGGATGACCACTGGATCTACTGG
GTGGGTCCCATGGCGGCTGCCCTGATCACCTCGGTGATCTACAAGCACGCCTTCCGGCGG
---GAGCTGGAGGAATCGGAGGTG------------GACGAGACGACGATGTCA------
------------------------ACCAAG---------AGGACCTCG---GAGGCGGAG
CTCGCC--

>FBpp0155127_Drosophila_grimshawi_Eglp2
ATGACT--

--CCGTCAAAATTC------

---TCA
ATAAAGTATATAGAT---CGATTG---TCAGGTGTCATTGTG------GACAAGCTATGC
ATCATCTTTGGCGAACTGACAGCAACGGCATTTCTCTTGTTCCTGGGCTGCATGGGTTGT
GTCCAG------ACATCG--
---GAGTATATTAACTCC
CATTTGCAGTGCTCCTTAAACTTTGGGTTCGTTGTGATGGTATGCATTCAGTGCTTCGGT
TGTGTCTCTGGTGCCCATCTCAATCCCGCCGTAACGCTTGCCAGCTACATTTATGATATG
ATATCGCTGCCGATGGCACTGGCATACTTTGTGGCCCAAATTGCTGGCGCATTCATCGGC
TATGGACTGCTGAAGGCATCGTTGCCCCATAATGCCATATGCAGCTTGGATACC------
------------------------CCGCTCGGCGCCTGCGTAACAACTGTCGACAGCGGC
---------ATTAGCGTTTGGCAGGCGGCCCTCATTGAGTTCCTGATCACATGTGTGCTC
ATTACCATCTGTTGCGGC---GTTTGGATCCCCGTAATACG-------------------
---AAATTCCAG---------------GATTCGACTGCCATACGTTTCGGGCTAGCCATT
GCATGCTTATCCCTAACTGCGGGTCAGTATACGGGCGCCAGTATGAATCCGGTGCGATCC
TTCGCGCCTGCCTTGTGGAACAAT---------GTCTGGGAGAGCCACTGGATCTACTGG
GTGTGTCCCATTGCTGGGTCATTGGTCACGTCCCTCATATACAAGTATGTCTTCAGGCGC
---GAGTCA---AAC---GATGCC------------GATAAGTGC---ATCCAA------
------------------------CCTAAT---------GAGTCACAG---------CCG

>FBpp0072015_Drosophila_melanogaster_Eglp3
ATGTCG--

------------------------CAACAG---AAGCAAAAATCGCAGGAGTCC------

---AAT
CCGAACGCCAGGTGG---CGCCTG---GAAGGACACCAAAGG------AGTGCGATAGCC
TGCTTCTTCGGTGAACTGGCGGCGACTGCCGTCTTCGTTTTCATAGCCTGCATGGGCTGT
GTGGAA------ACGCCG--
---CTGTTCCAGAATTCG

CACTTTCGCAGTGGCCTCACCTTCGGCCTGGCCATTCTCATCGCCATCCAGTGCTTCGGC
TCCGTTTCCGGTGCCCACTTAAATCCGGCCATCACCCTGGCTGCCTGGCTCTACGGCGCA
ATCGGTTGGATCCGGGCCATTGCTTACTTTGTGGCCCAGGCTGCCGGAGCCCTGATAGGC
TATGGTCTCCTGGTGGCTGTTCTGCCTGGGAACTCCATCAAGGGTGTGGACAAC------
------------------------CCGTCGGGTGTTTGTGTGACAATTCTGGCCCCCGGA
---------ATCAGCGTACTTCAGGGGGTCTTCATCGAGTTCCTCATTACCTGCTGCCTG
GTCATGGTGGCCTGTTCG---GTGTGGATCCACGGAACGCC-------------------
---AAGCTGCAG---------------GATTCGGTGCCCGTGAGATTCGGTCTCACCGTC
TCCTGTCTGATCCTCACAGCGGGTCTCTTCACGGGAGCCAGCATGAATCCGACCAGATCA
CTGGGTCCAGCTGTTTGGAACGAC---------TCCTGGGCACACCACTGGATCTACTGG
GTGGGTCCTTTGGTGGCAGGAGCTGTGACCTCGCTGATCTATCGCATGGCCTTTAAGGGC
---GAC------GAG---GAGATC------------GATCTGAGG---ACTTCC------
------------------------GATGCC---------AAGATACGA---ATGATCGGA
GAGGTT---ATA---GTGCAA---------------------------------------

>FBpp0155126_Drosophila_grimshawi_Eglp3
ATGTCG--

------------------------CAGCAGACATCATGGGTGGACAGCGACTCC------

---AGT
TGGAGATAT---CTG---AAGATG---CGATCGGAGCACAGG------GCTCACGTGGCT
GCCTTTTTTGGTGAGCTTGCTGGCACGGCGACATTCATATTTGTGGCCTGCATGGGCTGT
GTGACT------TCACCC--
---GTGTTCTACAACACT
CACTTTGAGCTGAGCCTGAACTTTGGCCTCGGCATTATGATTGCCATACAGAGCTTTGGC
TCCATTTCCGGCGCCCATCTCAATCCGGCCGTTACTCTAGCTGCTTGGATCTTTGGTGCA
TTGAGTTGGCCCATGGCTATTGCGTATTTGGTTGCACAGGTGGCTGGAGGTCTCATTGGC
TACGGTCTGCTGAAGGCAGTGCTGCCTTTGAATGCCATCGTCAGTGTGGACAAT------
------------------------CCGGCGGGTGTCTGTGTCACAGTTCTCAATAGTGAT
---------ATATCTGTGCTGCAGGGTGTCTTCATTGAATTCTTAATTACCAGCTGTTTG
GTGATGGTCGCCTGCTCG---ATCTGGGATCCTCGCAATGCC------------------
---AGACTTAAG---------------GATTCAGTGCCACTGCGCTTTGGTCTGACAGTC
TCCAGTCTTAACTTGGCCGCGGGCCTTTTCACCGGATCCAGCATGAATCCGACACGTTCC
CTGGGACCGGCTGTGTGGAACGAT---------TCGTGGCAAGATCATTGGATCTACTGG
GTGGGTCCACTTGCGGGTGCAGCAGTCACTGCAATCATCTATCGACTCTTCTTCAAGGGG
---CGCCCTGCAGAA---GTGGTT------------GAGCTACAA---ACCTCA------
------------------------AATGCC---------AAAATACAG---TTCGTCGAA

>ADD19418_Glossina_morsitans_morsitans_Eglp2b

---------------------ATG---AAGCCTCATGCTTGG------AAAATGATAACC
TGTTTCGTTTCTGAATTTATAGGCGCCTTTATGCTGGTTTTCCTTGGGTGCATGGGTTGC
ATTTCG------ACGCCG--
---TTGATATCTAATAAT
CATTTCCAAATGTGTTTCAATTTTGGTTTAGTCGTTATGATAGTAGTTCAGTGCTGCGCT
TGTGTCTCCGGTGCGCATGCGAATCCAGCTATATCGATCGCTGCTCTAATCTATGGAACA
TTGACTTTGCCAATGACTTTCATTTACTGCGTCGCTCAGATGTTAGGTGCTTTCATGGGT
TACGGCCTATTAAGGGGTATACTGCCCGAAGTCGTAAGGGAAGCGCCACATGCA------
------------------------AATCATGCCTTATGTGTCACAACAGTCCATCCAGAA
---------CTCTATCCATTACAAGGAGTATTTGTCGAATTTATTGCTACCGGTATCCTG
TTATTGATTTGCTGTGCT---GTGTGGGATGCGCGTAACGCC------------------
---AACTATCAC---------------GATTCAGTGTCCATTCGCTTCGGCCTGGCTGTT
GCATGCTTGGCGATCACAGCAGGGCCTTACACGGATGCAGCATGAACCCGGCTCGCTCG
TTTGCACCCGCTCTATGGAATGTT---------GATTTCACGGATCATTGGGTTTATTGG
TTGGGGCCAATATCGGGCGCCATCACAGCTACCTGTTTATGGAAATACATTTTAAGAGCT
---AAAGAGGAAAAGAAGATAATC------------GAAAGAGAA---GAACTT------
------------------------CCTCTT---------AAT------------------

>AFP49898_Glossina_morsitans_morsitans_Eglp2c
ATGCAA--

---TTTCGTTGG------

---------------------CTTTCCCGAGAGGAAGGGTGG------AACATTTTTGCG

```
CGCTTTGTGTCTGAAGTTATTGCCACTTTCCTTTTGATCTTTTTTGGTTGCATGGGTTGC
ATTAAA------TCTGAA------------------------------------------
---------------------------------------------TATATAACTAACGAT
CGCTTCGCATTAAGTTTTCATTTTGGTGGTGCGGTTTTATTACTTGTTCAGTGTTGCGCT
TGTGTTTCCGGTGCACATGCAAATCCAGCAATTTCGGTAGCGGCTCTCTTCTATGGAACA
TTAAGCTGGCCCATGATGTTCCTCTACATTCTTGCTCAAGTCATAGGCGCTACTATTGGT
TATGGCAGTTTGCTCGGAATTGTACCGCGTGTTTTAAGGATTGATCCAAACGCG------
------------------------AAACACGGTCTCTGCGTTGCCACAATACATCCAGAA
---------GTACATATTTGGCAAGCAATACTTTTAGAGTTTTTTGCAACATCTACGCTG
GTACTAATGTGCTGCGCA---GTATGGGATAAACGTAACTCT------------------
---AATCGTTTT---------------GATTCAGTTTCAATTCGTTTCGGTTTGGCGGTC
AGCAGCTTAGTATATTACGTGGGACCTTACACTGGGTGTAGTATGAATCCGGCTCGATCG
TTGGGCCCCGCGATATGGAATCTT---------GATTTTACGAATCATTGGTTATATTGG
TTCTCCCCTACCTTAGGTGGGATAGTTACTGCTGTGTTATGGAGATATGTTTTG------
---AAGGAGAAATCTTTTGAA---------------ACTAAGGAG---CAGCAG------
------------------------GAGGTC---------AAT---GAG------------
------------------------------------------------------------
------------------------------------------------------------
------------------------------------------------------------
------------------------------------------------------------
------------------------------------------------------------
------------------------------------------------------------
------------------------------------------------------------
------------------------------------------------------------
------------------------------------------------------------
------------------------------------------------------------
------------------------------------------------------------
------------------------------------------------------------
------------------------------------------------------------
------------------------------------------------------------
------------------------------------------------------------
------------------------------------------------------------
------------------------------------------------------------
------------------------------------------------------------
------------------------------------------------------------
------------------------------------------------------------
------------------------------------------------------------
------------------------------------------------------------
------------------------------------------------------------
------------------------------------------------------------
------------------------------------------------------------
------------------------------------------------------------
------------------------------------------------------------
------------------------------------------------------------
------------------------------------------------------------
------------------------------------------------------------
------------------------------------------------------------
------------------------------------------------------------
```


>FBpp0071980_Drosophila_melanogaster_Eglp1

---------------------ATG---ATCTGGGAC---TTC------TTTACCTTTATA
CGCGGAGCCACCGAGTTCAGTGCCACTGCGCTACTGATTTTGCTCGGTTGCATGGGAGAC
TCAACG------AACCAA--
---GGTGGTGAGAGCAAA
TTCTTGGTGGCCAGCGTTCACTATGGCCTGACGGTGATGGTGGTGATGCACGTGTTTGGC
TTCGTATCCGGAGCCCATTCGAATCCATGCATCTCGATCTCATGCTACTTGATGGGCTAC
ATCGCCCTGGAAGTGATGATGATGTACGTGGTGTGTCAGATGGCCGGTGCTTTCCTTGGT
TACTTCCTGCTAATGCAACTGCTGCCCAAGGAGCTGGTGGACAAAAGC------------
------------------------AAGCCAGGCATTTGCCTGGTACAACCGATGGACACT
---------CTGTCCACATACCAGGTCGTCATCATCGAGTGCCTGCTGACCGCGGTCCTC
GTGCTCGGATGGTGCTCC---TTGTGGGACGTGCGAAACGGA------------------
---AGGTTCCTC---------------GACTCGGTCGCCATTCGCATGGGTCTCCTCGTG
ATCGCTTGCAGTTTCGCAGGGATTCAACTAACTGGAGCCAGTATGAACCCCGCCAAGACA
TTGGTGCCTGCCATATTCTACGGC---------AGTCCGAATTCGGTTCTAATGCAGCTG
ACTGGCCAGATCCTGGCCGCCATCATGGTGCCCTTCGTGTGGAATCATGCGTATACGCCA
---CCTTATAAGCCCTTGGAAATT------------CCAGTGTGC---AAC---------

>FBpp0154569_Drosophila_grimshawi_Eglp1a

---------------------ATG---ATATTCAATAGT---------TTTACCTGTATG
CGTGGATTGAGCGAGTTTTGTGCAACAGCTTTGTTCATGTTAATCAGTTGCTTGGGCGGC
ACCGTGACCAGCGACCAG--
---AATGGAGCCAATGGG
AATCTTATGTCAAGTGTACACTACGGATTGGCCGTCGTTGTGATAATGCACATCTTTGGA
TTTATATCTGGCGCCCATGCGAATCCTTGCGTATCGATCGCTTGCTGTTTACTTGGCTAT
ATTGGCTACAAGATGATGTTCCTCTATGTGATCTGCCAGCTGGCGGGAGCAGCTTTTGGT
TACTTTCTACTGCTGCAAATGCTCACACAAGGCATGATTGATGGCTGC------------
------------------------AAGCAGGGCATTTGCATTGCAGAGCCCATGAAGAGT
---------CTGTCCTACGTACAGATATTGAGCATTGAAGGCTTCCTTACAGCAGTCCTA
GTTCTGGCCTGGTGCGCC---CTTTGGGACGTACGCAGCGGT------------------
---CGATTTCTT---------------GACTCGGTCACCCTGCGAATGGGCTGCTTAGTA
ATTGCATGCAATTTGGCCGGCGGTCAGCTAACGGGCGCAACGATGAATCCTGGCAAACTT
CTTATTCCCACGCTGTTCAAGGGG---------AATCCGCAAACGGTGCTCCTGCAACTG
GGCGGACAACTGTTGGCATCCATCATTGTGCCACCCATTTGGCAATATGCTTGCACACCG

---CGCTACAAACCCCTACAGGCT------------GACAACACT---GAGAGG------
------------------------CCCACT---------CAA---GAA---CTGTATTTA
ACGCCA--

[truncated: 304,390 more chars]
